# Filling knowledge gaps in insect conservation by leveraging genetic data from public archives

**DOI:** 10.1093/database/baae002

**Published:** 2024-01-29

**Authors:** Serena Baini, Alessio De Biase

**Affiliations:** Department of Biology and Biotechnologies ‘Charles Darwin’, Sapienza University of Rome, Viale dell’Università 32, Rome I-00185, Italy; Department of Biology and Biotechnologies ‘Charles Darwin’, Sapienza University of Rome, Viale dell’Università 32, Rome I-00185, Italy

## Abstract

Insect decline has become a growing concern in recent years, with studies showing alarming declines in populations of several taxa. Our knowledge about genetic spatial patterns and evolutionary history of insects still exhibits significant gaps hindering our ability to effectively conserve and manage insect populations and species. Genetic data may provide valuable insights into the diversity and the evolutionary relationships of insects’ species and populations. Public repositories, such as GenBank and BOLD, containing vast archives of genetic data with associated metadata, offer an irreplaceable resource for researchers contributing to our understanding of species diversity, population structure and evolutionary relationships. However, there are some issues in using these data, as they are often scattered and may lack accuracy due to inconsistent sampling protocols and incomplete information. In this paper we describe a curated georeferenced database of genetic data collected in GenBank and BOLD, for insects listed in the International Union for Conservation of Nature (IUCN) Italian Red Lists (dragonflies, bees, saproxylic beetles and butterflies). After querying these repositories, we performed quality control and data standardization steps. We created a dataset containing approximately 33 000 mitochondrial sequences and associated metadata about taxonomy, collection localities, geographic coordinates and IUCN Red List status for 1466 species across the four insect lists. We describe the current state of geographical metadata in queried repositories for species listed under different conservation status in the Italian Red Lists to quantify data gaps posing barriers to prioritization of conservation actions. Our curated dataset is available for data repurposing and analysis, enabling researchers to conduct comparative studies. We emphasize the importance of filling knowledge gaps in insect diversity and distribution and highlight the potential of this dataset for promoting other research fields like phylogeography, macrogenetics and conservation strategies. Our database can be downloaded through the Zenodo repository in SQL format.

**Database URL:**  https://zenodo.org/records/8375181

## Introduction

Invertebrates make up nearly 95% of all animal species, totaling over 1.25 million documented species. Among them, the phylum Arthropoda is the most diverse with around 1.11 million described species. Insects comprise approximately 85% of all arthropods representing one of the most diverse taxonomic groups and playing a critical role to many ecosystem functions (1, 2). With their abundance and taxonomic diversity, insects hold pivotal roles in several aspects of ecosystem health and human well-being. Their influence spans a spectrum of ecological functions, contributing to essential services and occupying crucial niches within natural communities (3). By enhancing soil health, aiding in agricultural pest control through predators and parasitoids, establishing plant–herbivore associations or participating in the decomposition of organic matter and litter, insects are indispensable for maintaining the balance of terrestrial and aquatic ecosystems (4). They also play key roles in regulating seed dispersal and nutrient cycling, providing irreplaceable benefits from their role in pollination and decomposition to serving as indicators of habitat quality (5–8). Any decline or alteration in the diversity and abundance of insects can have significant repercussions on numerous ecosystem functions and services and therefore the protection of these organisms is urgent and necessary for the well-being of both the planet and human beings (9). However, assessments of their global patterns lag behind many of their vertebrate counterparts. Moreover, despite the vast functional and taxonomic diversification of insects, the International Union for Conservation of Nature (IUCN) Red Lists currently focus on only four taxonomic groups—dragonflies, bees, saproxylic beetles and butterflies—although they are charismatic in a conservation context and play crucial roles in ecosystems. In fact, butterflies, aside from their ecological significance, stand out as a model in biology and serve as a flagship group for invertebrate conservation (10). Bees, as crucial pollinators, play a vital role in maintaining ecosystem health and promoting biodiversity (8). Saproxylic organisms, on the other hand, contribute significantly to decomposition in forest ecosystems (11). Lastly, among freshwater bioindicators, Odonata is a group gaining growing social and scientific interest (12, 13). Generally, our understanding of biodiversity is biased by several knowledge gaps, known as the Linnean, Wallacean, Prestonian and Darwinian shortfalls, which are particularly evident in the case of invertebrates (14, 15). The Linnean shortfall refers to the discrepancy between formally described species and the number of species that actually exist, while the Wallacean shortfall refers to lack of knowledge about the geographical distribution of species and stems from geographic biases in the information on species distributions. Even for those species that have been identified, we often have little or no information about where they live, making it difficult to know which ones are endangered and where to concentrate efforts to preserve them (16). The Prestonian shortfall can be defined as lack of knowledge about the abundance of species and their population dynamics in space and time (16). In fact, despite the fundamental importance of abundance data for addressing many ecological questions, such information is scarce for most species (16), and consequently there is a lack of information about population size, fluctuations and changes over time. Finally, the Darwinian shortfall refers to our limited knowledge of the evolutionary relationships and traits of species (17). In spite of advancements in molecular data and computational methods, there are still challenges to overcome for more comprehensive ecological comparative analyses in order to understand phylogenetic relationships among all species. Invertebrates are known to suffer from these shortfalls, leading to significant gaps in our knowledge and contributing to their rapid and alarming decline. Recent studies suggest substantial changes in invertebrate diversity and community composition that have occurred nearly unnoticed and indicate that species may be going extinct before we even know they existed (18, 19). Studies conducted in North America and Europe have identified agriculture intensification, climate change, habitat loss and fragmentation, pollution, invasive species and insecticide use as drivers of the drastic decrease in insect diversity and abundance (20). The European Union (EU) is aware of this phenomenon, and through the EU Biodiversity Strategy for 2030 highlights the alarming decline of insects, particularly pollinators, and their role as key indicators of the health of agroecosystems. Moreover, it commits to achieving legal protection of at least 30% of the EU’s land and sea area.

Important evidence of insect decline comes from the number of threatened species reported by the IUCN European Red Lists, which ranges from 9% to 26% of the total number of species assessed at the European level for selected taxonomic groups (21). Comprehensive regional Red List assessments for insect groups suggest that the outlook is concerning. For example, 11% of European saproxylic beetles are listed in one of the three threatened categories, 13% are Near Threatened and 28% are Data Deficient (22). The situation is even more critical for endemic saproxylic beetles in the Mediterranean, where 53% are classified as threatened (23). The Red List of bees in Europe reveals also alarming trends, with 57% listed as Data Deficient (24). Indeed, another alarming issue is the high number of Data Deficient records, i.e. species for which knowledge about life cycles and ecological traits is insufficient and which lack assessments according to the IUCN criteria.

In investigating patterns of biodiversity, assessments of species richness, abundance and biomass may not fully capture the nuanced changes that can be revealed through assessments of genetic and phylogenetic diversity in community composition. In fact, knowledge of patterns of genetic diversity is of critical importance to fully understand the potential of species to adapt to global change, and ultimately to succeed in halting biodiversity loss (25). It has been studied how natural populations facing a new stress, such as habitat destruction, pollution or climate change, can survive through adaptive evolutionary changes, a concept referred to, in conservation biology, as ‘evolutionary rescue’. The success of evolutionary rescue relies on the presence of genetic variability within the population and natural selection acting on heritable variation (26). Furthermore, assessing both species diversity and intraspecific variability is now recognized as crucial in conservation efforts (25, 27). By considering genetic diversity, conservation initiatives can effectively address the unique evolutionary history and adaptive potential of populations, thereby enhancing their adaptive capacity, viability and overall conservation outcomes (25). In addition, the incorporation of genetic criteria is essential for identifying Key Biodiversity Areas (KBAs), which are crucial for the conservation of global biodiversity. For instance, by integrating estimates of phylogenetic diversity into the identification process of KBAs, conservation policies can prioritize areas that not only exhibit high species richness but also preserve evolutionary distinctiveness. This approach ensures the conservation areas that harbor unique evolutionary lineages and represent significant branches of the tree of life.

Genetic data are being generated at unprecedented rates and the existence of georeferenced data in public repositories such as GenBank and the Barcode of Life Database (BOLD), where hundreds of samples are collected and archived every day, allows new comparative analyses. The GenBank database hosted at the National Center for Biotechnology Information (NCBI) stores over two hundred million DNA sequences, a number that grows monthly, and collects genetic data along with two other databases, DNA DataBank of Japan (DDBJ) and European Nucleotide Archive (ENA), also adhering to the International Nucleotide Sequence Database (INSDC). On the other hand, BOLD contains the barcoding data for about 600 000 species with well-represented and curated sets of mitochondrial Cytochrome c oxidase subunit I (COI) sequences (28). However, most of the sequences deposited in public databases often lack metadata associated with specimens sampling sites (29). For example, since 2005, the submission of DNA sequences to the NCBI has been encouraged but not required to include geographic information (30). This limits the potential use of these data by preventing recycling in any analysis that requires geospatial information. Another common issue related to geographic metadata deposited in genetic public databases is that they are often inaccurate (31). Records affected by these issues can introduce severe bias depending on the research question and the geographical scale of analyses. Reliable production, storage and public access to genetic data are essential to enable evaluation of the reproducibility of research, enabling data reuse and addressing diverse issues beyond the scope of original research. These include, for example, macrogenetic studies delineating genetic ‘hot spots’, the impact of climate change or the ongoing biodiversity crisis (20).

With increasing human-mediated disturbances and ongoing global environmental changes, there is a pressing need for large-scale biodiversity assessments and ecological studies and a deeper understanding of biodiversity connections at larger scales. The use of COI sequences has emerged as a powerful tool for species identification and classification, becoming the standard mitochondrial marker for barcoding animal DNA (32) as a first step in biodiversity assessments. In fact, this approach offers significant advantages over traditional morphology-based identification methods by being rapid, accurate and standardized. In addition, molecular analyses can provide a unique advantage by revealing patterns of regional genetic divergence, allowing for biodiversity comparisons at broader geographic and taxonomic scales (33–36). In this context, nuclear DNA is generally preferred when the goal is to provide a comprehensive representation of Intraspecific Genetic Variation (IGV), especially considering neutral markers. In fact, unlike mtDNA, nDNA undergoes recombination, providing independent assortment of genetic material during gamete production and allowing for more comprehensive assessments of population history and genetic diversity. Moreover, nDNA is inherited from both parents, providing a broader representation of a population’s genetic composition although generally evolving more slowly than mtDNA and making it less suitable for reconstructing very recent evolutionary events. On the other hand, mtDNA’s maternal inheritance and haploid nature simplify the interpretation of evolutionary patterns, making it particularly useful for unraveling phylogeographic patterns, while also for investigating climate adaptation, by tracing historical changes in distribution patterns and identifying multiple glacial refugia for species (37). The rapid growth of mtDNA data, especially in barcode sequences coverage, facilitates more feasible comparative macrogenetic analyses (38). Nevertheless, although COI haplotypes provide valuable information in a phylogeographic context, their utility may be limited in elucidating finer-scale intraspecific diversity. The accelerated lineage sorting rate of COI can result in fact in oversimplified evolutionary relationships, potentially overlooking crucial details in population histories (38). However, the gap between maximum intraspecific and minimum interspecific distances has been employed for species delimitation in various animal groups, including insects (33–35). This method has proven effective in resolving cryptic species complexes, uncovering genetic divergence patterns and contributing to the exploration of biodiversity in phylogeography studies (39). Whenever feasible, it is advantageous to combine both nDNA and mtDNA data, to leverage their respective strengths and complement each other in addressing different research objectives. Unfortunately, nuclear genetic data accessible from centralized databases are currently more limited, which may hinder widespread use and pose a significant challenge in building databases, especially for little-studied species like insects, making large-scale studies more challenging. Despite not being ideal for capturing large-scale IGV patterns, available COI sequences are more abundant and can still provide useful insights in molecular studies of biodiversity.

In the present study, we collected for the selected insects’ taxa all COI mitochondrial DNA sequences from GenBank and BOLD databases through a time-saving bioinformatics approach. The objective of this data paper is to describe a georeferenced and standardized dataset of mitochondrial genetic data (COI) that we assembled for four groups of selected insects listed in the Italian IUCN Red Lists: Odonata, Hymenoptera, saproxylic Coleoptera and Lepidoptera. We decided to focus our efforts on red-listed species due to their significance in conservation policies and management frameworks. Furthermore, given the forthcoming goals outlined in the European Biodiversity Strategy, there is the need for collecting reliable data on these insect species to meet the growing demands for biodiversity conservation and management. Although the IUCN Red List serves as the prevailing tool for monitoring and enforcing conservation policies, the attention and research dedicated to these species significantly lag behind that of vertebrates. This study aims to provide a comprehensive overview of the current state of knowledge, highlighting both accomplishments and existing gaps. Specifically, our focus is on assessing the taxonomic and geographic coverage of genetic data for the red-listed species, with implications for conservation purposes. This choice underscores the importance of these insect groups, even though they represent only a fraction of the broader insect diversity and functional groups. Despite this, the available knowledge about these four groups remains limited, and it is crucial to communicate the current state of genetic data for these important taxa within the scientific community.

## Methods

### Data compilation and validation workflow

**Figure 1.**
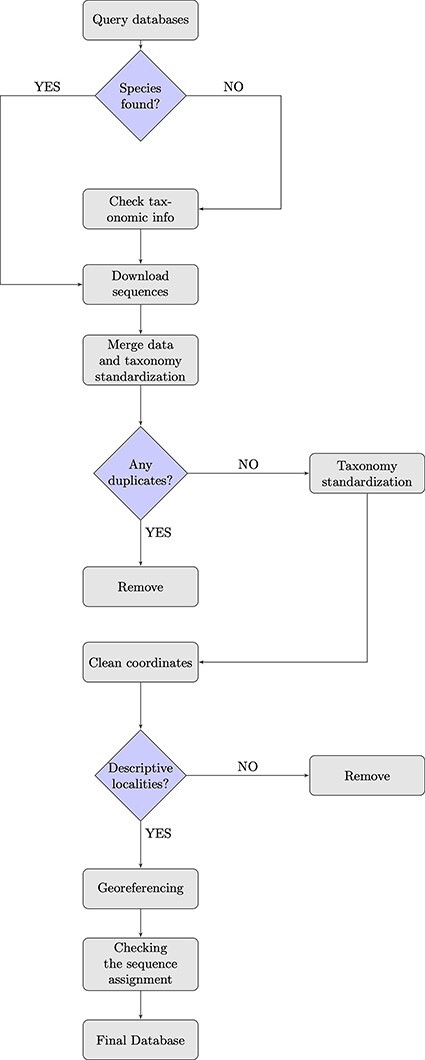
Flowchart showing the database building process.

To achieve our purpose, we (i) queried GenBank and BOLD databases and combined the information from both sources (Figure 1). While merging the downloaded sequences, (ii) a pre-filtering and data quality control step was conducted, in which we removed duplicates and implemented a taxonomy standardization phase wherein we harmonized scientific names according to the taxonomy used in the IUCN Italian Red Lists. We then (iii) excluded records with invalid or suspicious information on geographical coordinates. For data entries that lacked precise coordinates but had detailed collection site information, we conducted georeferencing to assign accurate spatial references and enhance the accuracy of the data. Additionally, (iv) we verified the taxonomic assignments of DNA sequences at the species level using tools that matched the sequences against a reference database with reliable taxonomic information, ensuring accurate species identification and taxonomic assignment. When not otherwise specified, all steps here described were performed using R (version 4.2.1, R Core Team, 2022) and Python (version 3.9.16, Python Software Foundation, 2022). In particular, R was employed for data visualization, while both R and Python were used for data handling and processing. The entire procedure is detailed in the following paragraphs.

### Data download

We collected mitochondrial DNA sequences, and all associated metadata from NCBI GenBank (www.ncbi.nlm.nih.gov/genbank) and BOLD (www.boldsystems.org) online databases in January 2023, querying all names of species included in the IUCN Italian Red Lists for the biogeographic regions in Europe according to the country boundaries as listed by the European Environment Agency (EEA) (https://www.eea.europa.eu/data-and-maps/figures/biogeographical-regions-in-europe-2). We defined the geographic extent from west to east with longitudes ranging from −25 to 66 and from north to south with latitudes ranging from 36 to 71. For Coleoptera Red List, which also contains several subspecies taxa, we excluded and elevated them to species level. This means that when we retrieved data from databases, we only referred to species taxonomic rank. The decision to focus on species-level information was made because of the scant availability of sequences attributed at the subspecies level and the often-heterogeneous assignment approaches, whether based on geography, phylogeography or different reference taxonomies. Maintaining a focus at the rank of species, we therefore tried to avoid duplication or confusion in the dataset. For each selected taxon, we obtained the species lists from the Italian IUCN documents available on the official IUCN website (http://www.iucn.it/liste-rosse-italiane.php). To identify the corresponding taxonomic identifiers (taxonIDs) for Linnean binomials, we followed Gratton *et al.*’s, 2017 procedure, submitting the species lists to the NCBI Taxonomy name/id Status Report Page (http://www.ncbi.nlm.nih.gov/Taxonomy/TaxIdentifier/tax_identifier.cgi). Genus taxonIDs were obtained using the R/CHNOSZ package (40). To search for the species in the NCBI GenBank database, we used a custom Python script available from the same paper (30). Furthermore, we queried BOLD from the webpage using the available application-platform interface (API) (https://v4.boldsystems.org/index.php/api_home).

### Taxonomy and standardization

For those species listed in the IUCN Italian Red Lists for which we did not find any data in GenBank and BOLD databases, we checked for undetected taxonomy variations, synonyms or misspellings to retrieve possible data deposited under different names. Therefore, we manually queried several taxonomic databases, such as Pan-European Species directories Infrastructure (PESI; http://www.eu-nomen.eu/portal/search.php?search=adv), FaunaEuropea (https://fauna-eu.org), European Nature Information System (EUNIS; https://eunis.eea.europa.eu/species.jsp), the Catalogue of Life (https://www.catalogueoflife.org) and Integrated Taxonomic Information System (ITIS; https://www.itis.gov/index.html), and also used the R/taxize package, which interacts with many taxonomic data sources (41) to obtain taxonomic information for the species of interest. We then compiled a list of all alternative names associated with the target species and re-ran the databases queries. Following this process, we tried to capture all available genetic data for the target species, avoiding overlooking relevant information due to naming discrepancies.

In BOLD, in addition to the Process IDs column of automatically generated unique codes for each record, there is also a column listing the GenBank accession numbers. We relied on this column to remove duplicates, that is, records that occurred in both databases with the same GenBank accession number. In cases where duplicate entries were identified, we further checked that the species name, the geographical description and the geographical coordinates were the same. When taxonomic names varied due to synonyms, misspellings or different reference taxonomies, we decided which records to keep in order to standardize our dataset according to the nomenclature adopted in the IUCN Red Lists.

We performed a data quality control and standardization step while merging the data downloaded from the two repositories. Since GenBank and BOLD use different column names for the same fields, we standardized the column names we want to retain, ensuring uniformity and consistency in the merged dataset. In addition, we standardized all available information for each observation. For example, we assigned unique gene symbol for Cytochrome oxidase I (COI) that is often depicted by different names. A column was added providing information about the conservation status in the IUCN Red Lists (IUCN, 2020) for each collected species.

### Geographic coordinates

We cleaned up the distribution data retrieved by removing all records incorrectly georeferenced or with a low level of accuracy and records with missing coordinates. We used R/CoordinateCleaner package (42) to compare the coordinates of our records to reference databases and identify data entry errors. Such errors fall into different categories revealing frequent errors in databases that collect species occurrence points. These are represented by the ‘capitals’ category, referring to records falling under capitals and the ‘institutions’ category that include records that fall within biodiversity institutions. The ‘outliers’ and ‘seas’ categories include, respectively, records identified as outliers based on their spatial distribution and records falling into the sea. For outlier identification, we utilized the ‘quantile’ method, relying on a boxplot approach that employs the interquartile range (IQR) to establish a range within which most of the data are expected to fall. In the case of the ‘quantile’ method, records are flagged as outliers if their mean distance to all other records of the same species exceeds *mltpl* times the interquartile range of the mean distance for all records of this species, where the default value for *mltpl* is set to 5. The identified outliers are then flagged for further investigation or removal. The ‘centroids’ category refers to occurrence records assigned to centroids of countries or provinces due to automated georeferencing from vague location descriptions.

In GenBank, the geographic description field varies in terms of accuracy. For example, some entries have only the country information, while other entries are more detailed. In contrast, BOLD has different fields for each precision degree of the geographical data. When geographic information featured as text only, we ranked its accuracy level and removed geographic information provided just as ‘country’. Data that had more detailed information were georeferenced using the package Nominatim included in the GeoPy Python library which searches within OpenStreetMap (OSM) geographic database. We removed inaccurate georeferenced data by performing the same procedure described above with R/CoordinateCleaner package (42). To measure the accuracy of our geocoding procedure from location data, we applied the same georeferencing GeoPy method on 20% of database sequences retrieved from GenBank and BOLD that were already annotated with both location names and geographic coordinates. Using the descriptive location entries, we obtained new geographic coordinates for each record, and to compare their accuracy, we calculated the distance between the original geographic coordinates and those assigned with GeoPy. We transformed the distance into kilometers using the haversine formula (30, 43).

### Sequence taxonomic assignment

In animal barcoding and metabarcoding studies, a fragment of the mitochondrial Cytochrome oxidase subunit I gene is sequenced and subsequently assigned to a known taxon. To reach confident taxonomic assignment, we adopted a widely common procedure employing the QIIME2 platform (44). We created a taxonomic classifier using a recently curated database (45) as a reference to train the Naive Bayes classifier with q2-feature-classifier, using both the reference sequences and the corresponding taxonomic classifications. We then used this trained classifier for taxonomic classification of all sequences to be included in our curated database, setting a confidence threshold for taxonomic depth limitation at 0.7. This threshold controls the degree of taxonomic resolution applied to the assignments, allowing for a balance between accuracy and inclusivity. Additionally, the classification was executed with an automatic determination of read orientation based on confidence estimates for the initial 100 reads, ensuring optimal alignment to reference sequences. Since combining different methods is an encouraged approach for maximizing reliability of taxonomic results, we employed the same curated database to conduct our assignment analysis also using the Statistical Assignment Package (SAP) software (46). Given that this command-line tool requires huge computation time when using the Bayesian default option (*Barcoder*), we choose for the assignment parameter (—assignment) the alternative option *ConstrainedNJ*. Unlike the Bayesian approach, the *ConstrainedNJ* method incorporates both the neighbor joining and bootstrapping algorithm to assign taxonomy based on distances between sequences and evaluate the statistical support. For the alignment (—alignment) parameter we used the default option ClustalW2 alignment method. Occasionally, sequences downloaded from public databases contained gaps (-) due to errors during the submission process. To ensure accurate taxonomic assignments and avoid false negatives, we removed gaps from all retrieved validated COI sequences and used only those longer than 200 bp before performing both taxonomic classification methods. Following taxonomic assignment analysis, before dropping sequences without species-level assignments, we ensured that they were represented in the reference database, checking at least at the genus level, and exploring potential synonyms. We removed sequences lacking species-level assignments even though reaching the genus rank with a confidence level of 0.9 or higher, considering that the prevalent use of our database is in the conservation field, where precise species-level identification is crucial.

## Results

### Data download

**Figure 2.**
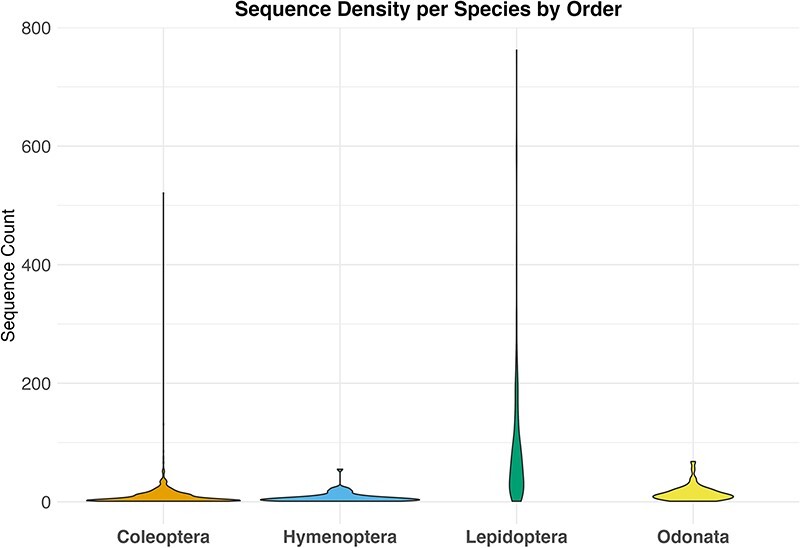
Violin plot showing the distribution of sequence counts per species across different orders, Where the y-axis indicates the number of sequences for each species in the dataset and the x-axis the examined taxa.

By downloading all available sequence records and all associated annotations for the four selected invertebrate taxa, we obtained a total of 79 000 unique sequences, 38 000 from GenBank and 41 000 from BOLD. After the data filtering process (Table 1), we populated our mtDNA dataset with a total of about 33 000 validated sequences distributed across various biogeographic regions in Europe, representing 1466 species. These included 473 sequences for 62 species of Hymenoptera, 1293 sequences for 87 species of Odonata, 23 451 sequences for 262 species of Lepidoptera and 10 474 sequences for 1055 species of Coleoptera (Figure 2; Table 2). The violin plot in Figure 2 depicts the distribution pattern of sequence counts across different species within each order.

**Table 1.** Summary of data cleaning steps

	Records removed	Total
Initial GenBank and BOLD download data		108 551
Identical rows between databases	33 670	74 881
Records with genetic markers other than mtDNA (COI)	18 877	56 004
Records without sequences	1775	54 229
Records with sequences shorter than 200 bp were excluded	114	54 115
Records falling outside the borders of biogeographic Europe	9489	44 626
Records removed that had a poor level of accuracy (CoordinateCleaner)	2797	41 829
Records with missing lat/lon field after georeferencing	1455	40 374
Records removed after taxonomic assignment check (QIIME2)	7480	**32 894**

**Table 2.** Table showing the number of species included in the IUCN Italian Red Lists that we searched, those found in the queried databases and the species in the final dataset. For the order Coleoptera, subspecies were elevated to species level

	IUCN list	GenBank	Bold	Total species after cleaning
Odonata	88	87	84	**87**
Hymenoptera	151	71	68	**62**
Coleoptera	1971	1141	1198	**1055**
Lepidoptera	285	267	256	**262**
**Total species**				**1466**

**Figure 3.**
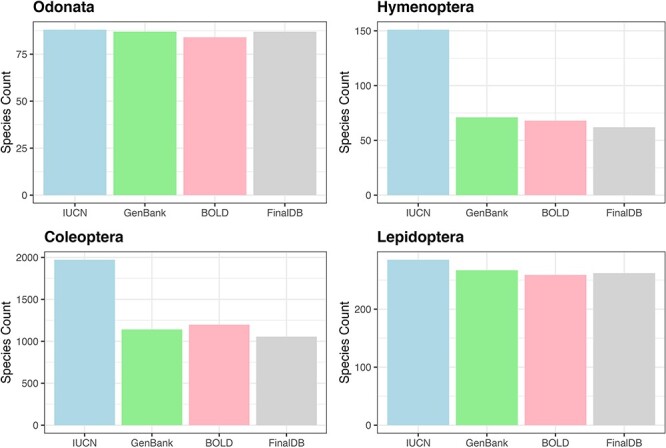
Bar plot showing retrieved species numbers from online databases compared with IUCN Red Lists for the examined taxa.

For Hymenoptera, we retrieved nearly 41% of the 151 species included in the Red List, 99% of 88 species in the Odonata, 91% out of 289 Lepidoptera in the Red List and 52% for 2008 Coleoptera (Figure 3). All conservation status of the IUCN Red List were proportionally represented in our database; however, there was a notable scarcity of data for the most threatened Coleoptera and Hymenoptera (see Supplementary material, Figure S1).

**Figure 4.**
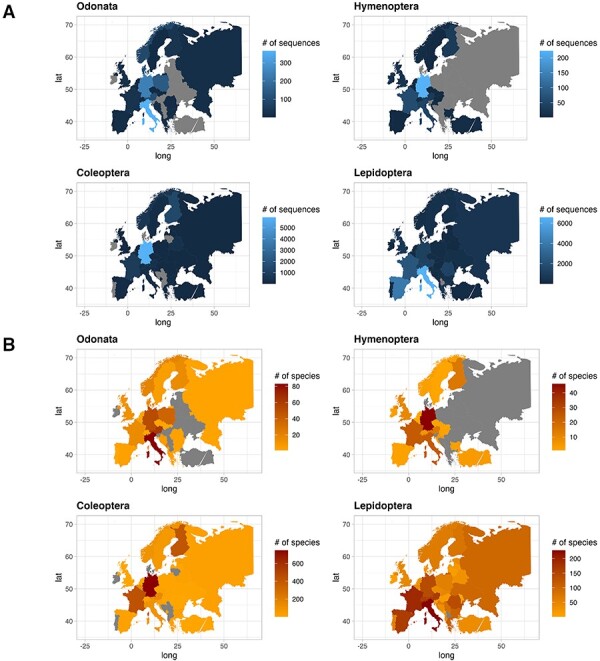
Plot showing the number of sequences for each country for the four IUCN Red Listed insect orders in the final database (A), and plot showing the number of species for each country for the four IUCN Red Listed insect orders in the final database (B), where grey color refers to the absence of information.

The contribution of COI sequences per species, deposited in the two gene archives, varied between countries. In this text, we only report few countries characterized by the highest number of sequences and species. For a quick overview, see Figure 4 for sequences and species distribution and Table 3 for a summary. Among the Odonata sequences, Italy recorded the most abundant number of sequences (365), followed by Germany (241) and Poland (138). Similarly, considering the number of unique Odonata species, Italy had a large number (83), followed by Austria and Germany with 62 and 51 unique species, respectively. As for Hymenoptera, Germany recorded the most abundant sequences collection (223), followed by France (61), Italy (46) and Austria (30). The number of species per country was higher in Germany with 46 unique species, followed by France and Italy with 24 unique species each. In the order of Coleoptera, Germany recorded the highest number of sequences (5914), followed by Finland (1360) and France (863). Looking at the species count, Germany had 746 unique species, followed by France and Finland with 429 and 408 unique species, respectively. Finally, regarding Lepidoptera, Italy recorded more sequences (6569), followed by Spain (3993) and France (2304). Focusing on Lepidoptera species, Italy recorded the most abundant number of species (244), followed by France and Spain with 203 and 179 unique species, respectively.

**Table 3.** The ten countries with the most abundant numbers of sequences and species for the orders examined

	Country	Number of sequences	Number of species
**Coleoptera**	Germany	5914	746
	Finland	1360	408
	France	863	429
	Norway	582	95
	Austria	323	146
	Belarus	304	22
	Italy	184	89
	Belgium	178	125
	Sweden	138	63
	Bulgaria	115	32
**Hymenoptera**	Germany	223	46
	France	61	24
	Italy	46	24
	Austria	31	15
	Finland	30	15
	United Kingdom	19	10
	Ireland	12	3
	Switzerland	9	6
	Turkey	7	4
	Bulgaria	6	3
	Croatia	6	5
**Lepidoptera**	Italy	6569	244
	Spain	3993	179
	France	2304	203
	Romania	1319	150
	Austria	1215	172
	Germany	1196	152
	Switzerland	1185	174
	United Kingdom	788	50
	Greece	650	122
	Russia	527	105
**Odonata**	Italy	365	83
	Germany	241	51
	Poland	138	38
	Denmark	113	16
	Austria	105	62
	Norway	87	20
	Malta	48	9
	Finland	29	16
	Montenegro	28	25
	France	27	15

### Taxonomy and standardization

**Figure 5.**
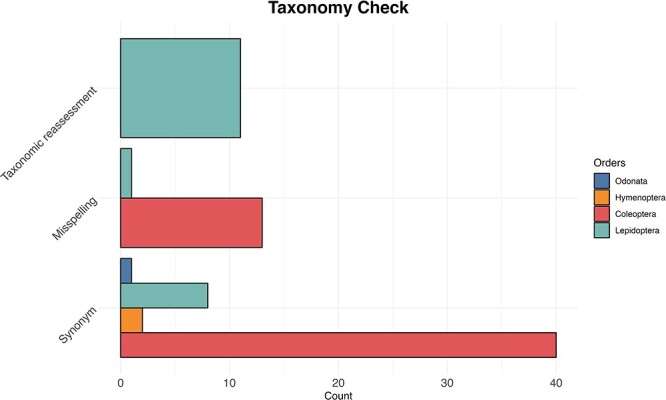
The bar plot shows for each examined taxon the count of the three most common reasons (synonyms, misspelling and taxonomic reassessment) for taxonomic discrepancy between the IUCN Red Lists and the reference taxonomy of GenBank and BOLD databases.

We searched for the names listed in the IUCN Red Lists and when we did not find a match, we performed a manual search for each species. We found that 754 (41%) of Coleoptera species had no data available in GenBank and BOLD; after a manual search, we found that 53 of those were either deposited with their synonyms (38) or misspelled (12). For Hymenoptera species, 64 (42%) were initially not found in the databases, and we were able to retrieve data for only 4 species deposited under synonyms. In the case of Lepidoptera, of the 22 (8%) species not found, we recovered 8 species archived as synonyms, one species was found to be misspelled and 11 underwent taxonomic re-evaluation (Figure 5). Finally, we found data for all Odonata species except one, which was identified after manual search as a synonym.

Out of the selected taxa, the order Coleoptera exhibited the highest diversity in terms of numbers of families, with a total of 46 families represented. Within this order, Cerambycidae was the most abundant family, comprising 1196 sequences (15%). In the order of Lepidoptera, we recovered data from 6 different families of which Nymphalidae being the most numerous with 9793 (89%) sequences. Hymenoptera and Odonata had respectively 5 and 9 families, with Halictidae and Libellulidae being the most abundant families, representing 217 (50%) and 380 (30%) sequences records (see Supplementary material, Figure S2).

### Geographic coordinates

**Figure 6.**
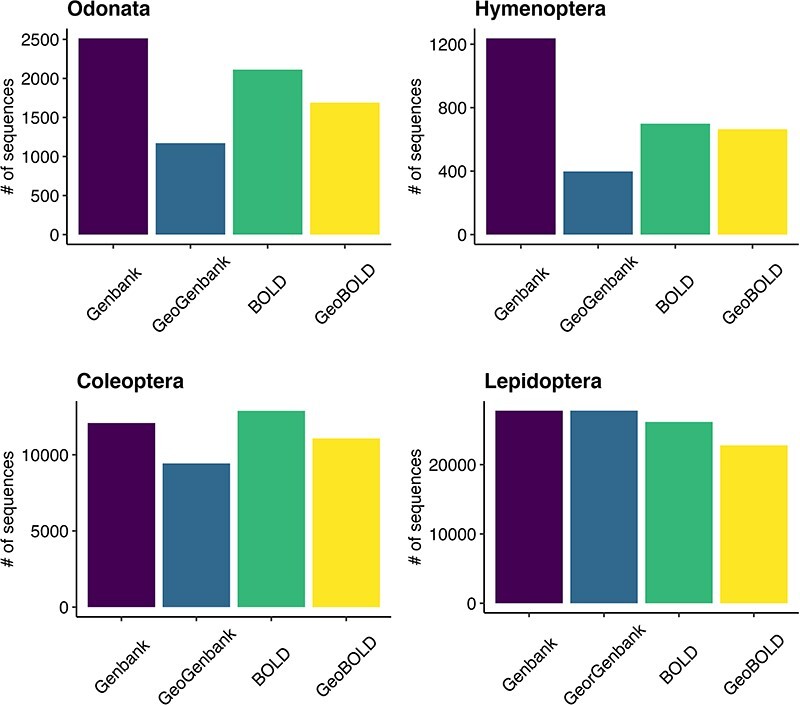
Grouped bar plot showing differences between BOLD and GenBank retrieved data on the left and georeferenced records on the right for the examined taxa.

Of the approximately 38 000 total sequences downloaded from GenBank, 89% had the coordinates, while out of the about 41 000 sequences retrieved from BOLD, 86% included the coordinates (Figure 6). Of all the sequences initially downloaded, 82% had descriptive location indications and of these, 15% (7244) were without coordinates (see Supplementary material, Appendix 2). After using Geopy we were able to recover and validate coordinates for 67% (4831) of the descriptive locations retrieved. Overall, our quality control procedures indicated that the method used to assign geographic coordinates to unannotated sequences is powerful and almost accurate. The results show that 75% of the locations assigned using Geopy were within 27 km of the locations originally uploaded as annotations, while the mode of the entire dataset was found to be 4.6 km. After the data quality control procedures were performed with CoordinateCleaner, 2797 coordinates records were removed (see Supplementary material, Appendix 4) that had a poor level of accuracy (see Supplementary material, Figure S3). The findings reveal a prevalent occurrence of coordinate errors in the analyzed datasets, with the ‘outliers’ (29%) and ‘seas’ (97%) categories being the most observed types of errors, particularly abundant in the Hymenoptera and Coleoptera data. The ‘centroids’ category showed a moderate frequency (7%), especially in Odonata and Hymenoptera. The ‘capitals’ category exhibited a relatively high frequency (24%), particularly in Coleoptera and Hymenoptera. On the other hand, ‘institutions’ (2%) category had low frequencies across all orders data.

### Sequence taxonomic assignment

For the taxonomic assignment, we employed the SAP software and the Naive Bayes classifier trained with the q2-feature-classifier method of the QIIME2 tool, using a recently curated taxonomic database (44–46). The results showed that out of 1923 occurrences of Odonata, 17% (337) were classified without species-level identification. Similarly, 12% (3525) out of 28 138 occurrences of Lepidoptera, 6% (31) out of 528 occurrences of Hymenoptera and 12% (1643) out of 14 094 occurrences of Coleoptera were not identified at the species level. Consequently, these unidentifiable occurrences were removed from the analysis to ensure the accuracy of the dataset (see Supplementary material, Appendix 5). We removed sequences that were not assigned to any species because not included in the reference database and sequences assigned only at genus level with an accuracy of 0.9 or higher: two species with a total of six observations for Hymenoptera and four species with a total of six observations for saproxylic Coleoptera. We also get species assigned to different species names compared to GenBank and BOLD downloaded records. We investigated whether this discrepancy was due to synonymous species, and when we did not find any congruency, we proceeded to remove the corresponding records. Specifically, we removed 4.9% records in Hymenoptera, 6.3% records for Odonata, 2.7% for Lepidoptera and 4.8% records in Coleoptera (see Supplementary material, Appendix 5). In 1% of the cases over the entire dataset, we observed inconsistencies between the results obtained from two different assignment methods. In such cases, we relied on the assignment confidence values, with QIIME averaging 0.98 and SAP averaging 0.25 (see Supplementary material, Appendix 6). Furthermore, in 25% of the cases, the SAP assignment method failed to assign species names to sequences, whereas the QIIME method was shown to assign species with a confidence level greater than or equal to 0.9%. This discrepancy can be attributed to the difference in assignment metrics: while the Bayesian method option provides a posterior probability of assignment, *ConstrainedNJ* offers an approximation of the bootstrap proportion. In particular, the Bayesian method is considered more reliable, but the advantage of *ConstrainedNJ* lies in its significantly faster execution, which makes it a viable solution, particularly in scenarios where the computational requirements of the MCMC approach become prohibitive. Finally, the overall mean number of sequences per species in the database was calculated to be 24.3. The order with the highest mean number of observations was Lepidoptera, with a mean of 89.5 observations per species. This was followed by Odonata with a mean of 14.9 observations per species, Coleoptera with a mean of 9.9 observations per species and Hymenoptera with a mean of 7.6 observations per species.

Our database can be downloaded at the Zenodo open data repository (https://zenodo.org/records/8375181) in SQL format and can be queried using any software that supports SQL tables. For a detailed description of the record structure, please refer to Table S5 in the Supplementary Material.

## Discussion

Our study aimed to create a curated and standardized dataset of georeferenced genetic data for selected insect groups in Italy and Europe, which we achieved by querying public repositories like GenBank and BOLD. We assembled a dataset comprising approximately 33 000 mitochondrial sequences associated with related annotations for 1466 species distributed across various biogeographic regions in Europe. Insect populations globally are facing alarming declines, emphasizing the need to integrate genetic data into conservation initiatives. Genetic information plays a vital role in unraveling the phylogenetic relationships among and within species, providing insights into their evolutionary history and overall biodiversity. The utilization of publicly accessible genetic data offers significant benefits in understanding biodiversity changes and advancing conservation efforts. These vast repositories of genetic information enable assessments of biodiversity responses to large-scale environmental impacts. Nevertheless, insufficient or undisclosed metadata poses a significant challenge, leading to the exclusion of a substantial portion of genetic datasets. Previous studies have reported that 40–73% of potentially suitable data are ultimately excluded due to inadequate metadata (31, 33, 34, 47). The Genomic Standards Consortium has established the Minimum Information about any Sequence (MIxS) standards, which outline the essential metadata requirements for genetic sequence data (48). These standards include information such as the sampling date, geographic location (preferably specified as decimal latitude and longitude) and environmental context (e.g. biome, feature or material) based on the nature of the study (25, 49). To address these issues and enhance data deposition and accessibility, various policies have been implemented. Initiatives like the Joint Data Archiving Policy and the Findable, Accessible, Interoperable and Reusable (FAIR) guiding principles have played a crucial role (50). These policies emphasize the importance of making genetic datasets interoperable and reproducible, promoting the curation of associated spatial and ecological metadata. Efforts to adhere to established metadata standards and promote data accessibility contribute to the advancement of scientific research and facilitate the reproducibility and interoperability of genetic studies.

Without taking into account duplicate records that were present in both databases, genetic markers other than mitochondrial DNA (COI) and records outside the geographical boundaries of biogeographic Europe, we had to exclude an additional 32% of the sequences due to various reasons such as missing or inaccurate coordinates, sequences of poor quality or taxonomic assignments that were incompatible. The percentage of species that we were able to retrieve from genetic databases compared to those listed on the IUCN Red Lists highlight the urgent need for increased efforts to study and collect genetic information for these important groups. In particular, only 41% of the Hymenoptera and 53% for Coleoptera species included in the IUCN Red List were found in public genetic repositories, suggesting that there is a need for more extensive genetic data collection to fully understand the genetic diversity and conservation status of these taxa. Moreover, for these two orders, we also found a scarcity of data belonging to species ranked at the threated conservation status of IUCN, which are particularly in need for conservation action. After a taxonomic check, we found that synonyms were the most common reason for missing data. This underlines the importance of maintaining updated taxonomic information and the need to use harmonized and standardized taxonomy, including the adoption of permanent and global taxon_IDs to represent taxonomic classifications and improve the completeness and consistency of such databases (51).

The availability of georeferenced genetic data is also essential for understanding the distribution and ecology of species, as well as for informing conservation efforts (52, 53). In this study, we found that most of the sequences downloaded from both GenBank and BOLD included coordinates (89% and 86%, respectively), indicating that there is an important amount of georeferenced genetic data available for the four selected invertebrate taxa. Additionally, it is also necessary to take into account the accuracy and reliability of georeferenced data. While the inclusion of coordinates is a crucial step, errors in georeferencing can occur, leading to inaccurate species distribution records. Our analysis of the genetic data downloaded from GenBank and BOLD using CoordinateCleaner revealed a meaning number of coordinate errors, with 2797 (8%) total records removed due to poor accuracy. The prevalence of coordinate errors in the analyzed datasets were the ‘outliers’ and ‘seas’ categories, being the most observed types of errors. This highlights the importance of validating the accuracy of georeferenced data before using it for conservation or ecological studies and the significance of implementing standardized protocols when entering data. Indeed, although geocoding offers a partial solution to the dearth of georeferenced data, the general amount of data potentially useful for an automated spatial analysis approach is still limited (30). These problems become greater when invertebrates are considered. We found that, although spatially and temporally detailed data exist for some of the insect orders studied, such as butterflies in the European Union and dragonflies in Italy, there is still a lack of information on red-listed bees and saproxylic beetles. These outcomes are in line with those of the Red List report on bees and saproxylic beetles, which indicate that many species are categorized as Data Deficient based on the criteria established by the IUCN. In fact, there is limited information available about these species, making it difficult to assess their conservation status.

In conclusion, our findings emphasize the importance of curated genetic data with metadata and the potential of publicly available genetic databases for advancing our understanding of insect species distributions, taxonomy and conservation. Our database provides a valuable resource for researchers and underscores the need for more focused attention on insect groups within global biodiversity research and planning. The database that we have created will enable researchers to be aware of gaps to be filled in the distribution, taxonomy and patterns of genetic diversity of insect species. Even though we only included species listed in the IUCN Red Lists not encompassing all taxonomic groups with diverse functional roles from an ecological perspective, our focus on species of community interest aligns with conservation policies in Europe. Indeed, our database represents a comprehensive collection dedicated to species conservation, with a specific focus on insects listed on the IUCN Italian Red Lists. In this field, data regarding these insects are patchy, limited and often inaccurate. Global environmental change and expanding human-mediated disturbance underscores the demand for a deeper understanding of biodiversity connections on a global scale. Although the genetic data provided by our database have inherent limitations due to the nature of the used marker (COI), we believe our collection of DNA data can help contributing to studies aimed at unraveling biodiversity patterns and enabling spatial genetic assessments. Leveraging methods like the geographical projection of genetic patterns through genetic divergence landscapes, our database facilitates the identification of diversity and evolutionary hotspots, potential barriers to gene flow and spatial genetic patterns across different lineages (54). The utility extends to dealing with multiple species, where the calculation of an average ‘multispecies landscape’ aids in detecting shared spatial components with similar evolutionary effects. This approach is invaluable for investigating the distribution of spatial genetic diversity and for comparing and classifying patterns through multivariate clustering of genetic landscapes. Our database stands out as a reliable resource as it contains a carefully curated collection of mitochondrial genetic data that have been subjected to thorough taxonomic and geographical verification processes, ensuring its accuracy. One of the significant results of our database is the assignment of geographic coordinates to almost 70% of the collected data through a meticulous georeferencing process recovering a considerable amount of otherwise unusable data. This valuable addition of geographical information greatly enhances the usefulness and accessibility of the database, making it a valuable tool for researchers and conservationists in the field of insect conservation. By repurposing these data for comparative studies, we can facilitate spatial analyses on a large scale. We also intend to enhance the database by incorporating future data and expanding it through both automated workflows, as outlined in the paper, and manual retrieval of additional data from existing scientific publications.

## Supplementary Material

baae002_Supp

## Data Availability

The data underlying this article are available in Zenodo open data repository at https://zenodo.org/records/8375181 and are currently subject to an embargo of 18 months from the publication date of the article or are available upon reasonable request.
