# Supplementary material for: Filling knowledge gaps in insect conservation by leveraging genetic data from public archives
Source: Database (Oxford). 2024 Jan 29;2024:baae002. doi: 10.1093/database/baae002 (PMC10878047; doi:10.1093/database/baae002)
Supplement: baae002_Supp [file baae002_supp.zip › suppl_data/appendices-csv.pdf]

Appendix_1_Data_short_Sequences;;;;;;;
order_name;family_name;genus_name;species_name;country;sourceDatabase;processid;genbank_accession
Hymenoptera;Apidae;Andrena;Andrena ovatula;Germany;Bolder;HYMAA315-22;-
Hymenoptera;Apidae;Andrena;Andrena ovatula;United Kingdom;Bolder;HYMAA317-22;-
Hymenoptera;Colletidae;Colletes;Colletes floralis;Ireland;GenBank;-;EF137744
Hymenoptera;Colletidae;Colletes;Colletes floralis;Ireland;GenBank;-;EF137746
Hymenoptera;Colletidae;Colletes;Colletes floralis;Ireland;GenBank;-;EF137748
Hymenoptera;Colletidae;Colletes;Colletes floralis;Ireland;GenBank;-;FJ041149
Hymenoptera;Colletidae;Colletes;Colletes floralis;Ireland;GenBank;-;FJ041150
Hymenoptera;Halictidae;Lasioglossum;Lasioglossum intermedium;Germany;Bolder;FBAPD659-11;-
Lepidoptera;Nymphalidae;Boloria;Boloria graeca;France;GenBank;-;MT916670
Lepidoptera;Nymphalidae;Boloria;Boloria napaea;Sweden;GenBank;-;MT916665
Lepidoptera;Nymphalidae;Boloria;Boloria napaea;Sweden;GenBank;-;MT916664
Lepidoptera;Nymphalidae;Boloria;Boloria pales;Austria;GenBank;-;MT916668
Lepidoptera;Nymphalidae;Boloria;Boloria pales;Austria;GenBank;-;MT916667
Lepidoptera;Nymphalidae;Boloria;Boloria pales;Slovenia;GenBank;-;MT916666
Lepidoptera;Lycaenidae;Callophrys;Callophrys rubi;Croatia;GenBank;-;MZ124262
Lepidoptera;Lycaenidae;Callophrys;Callophrys rubi;Croatia;GenBank;-;MZ104897
Lepidoptera;Lycaenidae;Callophrys;Callophrys rubi;Croatia;GenBank;-;MZ119987
Lepidoptera;Lycaenidae;Callophrys;Callophrys rubi;Croatia;GenBank;-;MZ106550
Lepidoptera;Lycaenidae;Callophrys;Callophrys rubi;Croatia;GenBank;-;MZ106685
Lepidoptera;Lycaenidae;Callophrys;Callophrys rubi;Croatia;GenBank;-;MZ106753
Lepidoptera;Lycaenidae;Callophrys;Callophrys rubi;Croatia;GenBank;-;MZ124191
Lepidoptera;Lycaenidae;Callophrys;Callophrys rubi;Croatia;GenBank;-;MZ105417
Lepidoptera;Lycaenidae;Callophrys;Callophrys rubi;Croatia;GenBank;-;MZ102574
Lepidoptera;Lycaenidae;Callophrys;Callophrys rubi;Croatia;GenBank;-;MZ102849
Lepidoptera;Lycaenidae;Callophrys;Callophrys rubi;Croatia;GenBank;-;MZ102923
Lepidoptera;Lycaenidae;Callophrys;Callophrys rubi;Croatia;GenBank;-;MZ125347
Lepidoptera;Lycaenidae;Callophrys;Callophrys rubi;Croatia;GenBank;-;MZ106887
Lepidoptera;Lycaenidae;Callophrys;Callophrys rubi;Croatia;GenBank;-;MZ125784
Lepidoptera;Lycaenidae;Callophrys;Callophrys rubi;Croatia;GenBank;-;MZ104519
Lepidoptera;Lycaenidae;Callophrys;Callophrys rubi;Croatia;GenBank;-;MZ104760
Lepidoptera;Lycaenidae;Callophrys;Callophrys rubi;Croatia;GenBank;-;MZ124932
Lepidoptera;Lycaenidae;Callophrys;Callophrys rubi;Croatia;GenBank;-;MZ125924
Lepidoptera;Lycaenidae;Callophrys;Callophrys rubi;Croatia;GenBank;-;MZ125989
Lepidoptera;Lycaenidae;Callophrys;Callophrys rubi;Croatia;GenBank;-;MZ122750
Lepidoptera;Lycaenidae;Callophrys;Callophrys rubi;Croatia;GenBank;-;MZ107025
Lepidoptera;Lycaenidae;Callophrys;Callophrys rubi;Croatia;GenBank;-;MZ118754
Lepidoptera;Lycaenidae;Callophrys;Callophrys rubi;Croatia;GenBank;-;MZ117954
Lepidoptera;Lycaenidae;Callophrys;Callophrys rubi;Croatia;GenBank;-;MZ121326
Lepidoptera;Lycaenidae;Callophrys;Callophrys rubi;Croatia;GenBank;-;MZ121465
Lepidoptera;Lycaenidae;Callophrys;Callophrys rubi;Croatia;GenBank;-;MZ121737
Lepidoptera;Lycaenidae;Callophrys;Callophrys rubi;Croatia;GenBank;-;MZ119045
Lepidoptera;Lycaenidae;Callophrys;Callophrys rubi;Croatia;GenBank;-;MZ119339
Lepidoptera;Lycaenidae;Callophrys;Callophrys rubi;Croatia;GenBank;-;MZ113765
Lepidoptera;Lycaenidae;Callophrys;Callophrys rubi;Croatia;GenBank;-;MZ113403
Lepidoptera;Lycaenidae;Callophrys;Callophrys rubi;Croatia;GenBank;-;MZ113187
Lepidoptera;Lycaenidae;Callophrys;Callophrys rubi;Croatia;GenBank;-;MZ113037
Lepidoptera;Lycaenidae;Callophrys;Callophrys rubi;Croatia;GenBank;-;MZ112668
Lepidoptera;Lycaenidae;Callophrys;Callophrys rubi;Croatia;GenBank;-;MZ114797
Lepidoptera;Lycaenidae;Callophrys;Callophrys rubi;Croatia;GenBank;-;MZ114728
Lepidoptera;Lycaenidae;Callophrys;Callophrys rubi;Croatia;GenBank;-;MZ114437
Lepidoptera;Lycaenidae;Callophrys;Callophrys rubi;Croatia;GenBank;-;MZ114223
Lepidoptera;Lycaenidae;Callophrys;Callophrys rubi;Croatia;GenBank;-;MZ115690
Lepidoptera;Lycaenidae;Callophrys;Callophrys rubi;Croatia;GenBank;-;MZ110398
Lepidoptera;Lycaenidae;Callophrys;Callophrys rubi;Croatia;GenBank;-;MZ109964
Lepidoptera;Lycaenidae;Callophrys;Callophrys rubi;Croatia;GenBank;-;MZ111047
Lepidoptera;Lycaenidae;Callophrys;Callophrys rubi;Croatia;GenBank;-;MZ117312
Lepidoptera;Lycaenidae;Callophrys;Callophrys rubi;Croatia;GenBank;-;MZ116391
Lepidoptera;Lycaenidae;Callophrys;Callophrys rubi;Croatia;GenBank;-;MZ116051
Lepidoptera;Lycaenidae;Callophrys;Callophrys rubi;Croatia;GenBank;-;MZ116465
Lepidoptera;Pieridae;Colias;Colias alfacariensis;Italy;Bolder;LEATG525-14;-
Lepidoptera;Nymphalidae;Euphydryas;Euphydryas aurinia;France;GenBank;-;AY491813
Lepidoptera;Nymphalidae;Euphydryas;Euphydryas aurinia;France;GenBank;-;AY491809
Lepidoptera;Nymphalidae;Maniola;Maniola jurtina;France;GenBank;-;KT264272
Lepidoptera;Nymphalidae;Melanargia;Melanargia galathea;Italy;Bolder;VNMB808-09;-
Lepidoptera;Nymphalidae;Melanargia;Melanargia galathea;Italy;Bolder;VNMB807-09;-
Lepidoptera;Nymphalidae;Melanargia;Melanargia galathea;Italy;Bolder;VNMB814-09;-
Lepidoptera;Nymphalidae;Melanargia;Melanargia galathea;Italy;Bolder;VNMB810-09;-
Lepidoptera;Nymphalidae;Melanargia;Melanargia galathea;Italy;Bolder;VNMB809-09;-
Lepidoptera;Nymphalidae;Melitaea;Melitaea cinxia;Finland;GenBank;-;AM041061
Lepidoptera;Nymphalidae;Melitaea;Melitaea cinxia;Finland;GenBank;-;AM041063
Lepidoptera;Nymphalidae;Melitaea;Melitaea cinxia;Finland;GenBank;-;JX878561
Lepidoptera;Nymphalidae;Nymphalis;Nymphalis io;Czech Republic;GenBank;-;MZ440841
Lepidoptera;Papilionidae;Parnassius;Parnassius apollo;France;GenBank;-;AY491909
Lepidoptera;Papilionidae;Parnassius;Parnassius apollo;France;GenBank;-;AY491932
Lepidoptera;Papilionidae;Parnassius;Parnassius apollo;Spain;GenBank;-;HG798923

Lepidoptera;Papilionidae;Parnassius;Parnassius apollo;Spain;GenBank;-;HG798922
Lepidoptera;Papilionidae;Parnassius;Parnassius apollo;Spain;GenBank;-;HG779448
Lepidoptera;Papilionidae;Parnassius;Parnassius apollo;Spain;GenBank;-;HG779452
Lepidoptera;Papilionidae;Parnassius;Parnassius apollo;Spain;GenBank;-;HG779449
Odonata;Libellulidae;Sympetrum;Sympetrum striolatum;Montenegro;Bolder;AODON787-20;-
Coleoptera;Cerambycidae;Aegomorphus;Aegomorphus clavipes;Finland;Bolder;COLFG013-13;-
Coleoptera;Cerambycidae;Aegomorphus;Aegomorphus clavipes;Finland;Bolder;COLFG011-13;-
Coleoptera;Elateridae;Ampedus;Ampedus pomorum;France;Bolder;PSFOR531-13;-
Coleoptera;Apocy-ceae;Anisotoma;Anisotoma castanea;Germany;Bolder;FBCOH511-12;-
Coleoptera;Apocy-ceae;Anisotoma;Anisotoma castanea;Germany;Bolder;FBCOE929-12;-
Coleoptera;Apocy-ceae;Anisotoma;Anisotoma humeralis;Germany;Bolder;FBCOG334-12;-
Coleoptera;Curculionidae;Cryptorhynchus;Cryptorhynchus lapathi;Norway;Bolder;COLHH1562-18;-
Coleoptera;Nitidulidae;Cychramus;Cychramus luteus;Austria;Bolder;ANIT015-20;-
Coleoptera;Nitidulidae;Cychramus;Cychramus luteus;Austria;Bolder;ANIT002-20;-
Coleoptera;Nitidulidae;Cychramus;Cychramus luteus;Austria;Bolder;ANIT008-20;-
Coleoptera;Nitidulidae;Cychramus;Cychramus luteus;Austria;Bolder;ANIT024-20;-
Coleoptera;Nitidulidae;Cychramus;Cychramus luteus;Austria;Bolder;ANIT011-20;-
Coleoptera;Nitidulidae;Cychramus;Cychramus luteus;Austria;Bolder;ANIT009-20;-
Coleoptera;Nitidulidae;Cychramus;Cychramus luteus;Austria;Bolder;ANIT010-20;-
Coleoptera;Nitidulidae;Cychramus;Cychramus luteus;Austria;Bolder;ANIT021-20;-
Coleoptera;Nitidulidae;Cychramus;Cychramus luteus;Austria;Bolder;ANIT025-20;-
Coleoptera;Nitidulidae;Cychramus;Cychramus luteus;Austria;Bolder;ANIT023-20;-
Coleoptera;Nitidulidae;Cychramus;Cychramus variegatus;Austria;Bolder;ANIT019-20;-
Coleoptera;Nitidulidae;Cychramus;Cychramus variegatus;Austria;Bolder;ANIT006-20;-
Coleoptera;Nitidulidae;Cychramus;Cychramus variegatus;Austria;Bolder;ANIT007-20;-
Coleoptera;Nitidulidae;Cychramus;Cychramus variegatus;Austria;Bolder;ANIT020-20;-
Coleoptera;Nitidulidae;Cychramus;Cychramus variegatus;Austria;Bolder;ANIT018-20;-
Coleoptera;Lymexylidae;Lymexylon;Lymexylon navale;Germany;Bolder;GBCOD790-13;-
Coleoptera;Cerambycidae;Oberea;Oberea oculata;Finland;Bolder;COLFG012-13;-
Coleoptera;Peltidae;Peltis;Peltis grossa;Finland;Bolder;COLFG092-13;-
Coleoptera;Histeridae;Plegaderus;Plegaderus caesus;Germany;Bolder;FBCOF497-12;-
Coleoptera;Scarabaeidae;Protaetia;Protaetia cuprea;France;Bolder;PSFOR248-13;-
Coleoptera;Cerambycidae;Rhamnusium;Rhamnusium bicolor;Finland;Bolder;COLFG008-13;-
Coleoptera;Salpingidae;Salpingus;Salpingus ruficollis;France;GenBank;-;KM286067
Coleoptera;Curculionidae;Scolytus;Scolytus intricatus;Finland;Bolder;COLFG084-13;-
Coleoptera;Curculionidae;Scolytus;Scolytus intricatus;Finland;Bolder;COLFG083-13;-
Coleoptera;Elateridae;Ste-gostus;Ste-gostus rhombeus;Germany;Bolder;FBCOE298-12;-
Coleoptera;Cerambycidae;Tetropium;Tetropium fuscum;Finland;Bolder;COLFG072-13;-
Coleoptera;Cerambycidae;Tetropium;Tetropium fuscum;Finland;Bolder;COLFG071-13;-
;;;;;;;

Appendix_2_Data_without_Coordinates;;;;;;;
order_name;family_name;genus_name;species_name;country;sourceDatabase;processid;genbank_accessio
n
Hymenoptera;Halictidae;Lasioglossum;Lasioglossum majus;France;GenBank;NA;AF104653
Hymenoptera;Colletidae;Colletes;Colletes albomaculatus;Greece;GenBank;NA;EF028492
Hymenoptera;Colletidae;Colletes;Colletes collaris;Austria;GenBank;NA;EF028495
Hymenoptera;Colletidae;Colletes;Colletes fodiens;Switzerland;GenBank;NA;EF028504
Hymenoptera;Colletidae;Colletes;Colletes foveolaris;Spain;GenBank;NA;EF028506
Hymenoptera;Colletidae;Colletes;Colletes graeffei;Austria;GenBank;NA;EF028508
Hymenoptera;Colletidae;Colletes;Colletes succinctus;Germany;GenBank;NA;EF028525
Hymenoptera;Colletidae;Colletes;Colletes wolfi;Italy;GenBank;NA;EF028527
Hymenoptera;Colletidae;Colletes;Colletes graeffei;Greece;GenBank;NA;EF218722
Hymenoptera;Halictidae;Sphecodes;Sphecodes hyalinatus;Ireland;GenBank;NA;JQ909877
Hymenoptera;Halictidae;Sphecodes;Sphecodes schenckii;Turkey;GenBank;NA;JX256660
Hymenoptera;Halictidae;Sphecodes;Sphecodes croaticus;Czech Republic;GenBank;NA;JX256662
Hymenoptera;Halictidae;Sphecodes;Sphecodes cristatus;Slovakia;GenBank;NA;JX256667
Hymenoptera;Halictidae;Sphecodes;Sphecodes hyalinatus;Czech Republic;GenBank;NA;JX256691
Hymenoptera;Halictidae;Sphecodes;Sphecodes hyalinatus;Bulgaria;GenBank;NA;JX256692
Hymenoptera;Halictidae;Sphecodes;Sphecodes majalis;Czech Republic;GenBank;NA;JX256703
Hymenoptera;Halictidae;Sphecodes;Sphecodes spinulosus;Czech Republic;GenBank;NA;JX256721
Hymenoptera;Halictidae;Sphecodes;Sphecodes spinulosus;Hungary;GenBank;NA;JX256722
Hymenoptera;Halictidae;Sphecodes;Sphecodes rubicundus;Czech Republic;GenBank;NA;JX256725
Hymenoptera;Apidae;Bombus;Bombus monticola;Norway;GenBank;NA;KF434334
Hymenoptera;Apidae;Bombus;Bombus monticola;Norway;GenBank;NA;KF434335
Hymenoptera;Apidae;Bombus;Bombus monticola;Norway;GenBank;NA;KF434336
Hymenoptera;Apidae;Bombus;Bombus monticola;Norway;GenBank;NA;KF434337
Hymenoptera;Apidae;Bombus;Bombus monticola;Norway;GenBank;NA;KF434338
Hymenoptera;Apidae;Bombus;Bombus monticola;Norway;GenBank;NA;KF434339
Hymenoptera;Apidae;Bombus;Bombus monticola;Norway;GenBank;NA;KF434340
Hymenoptera;Apidae;Bombus;Bombus monticola;Norway;GenBank;NA;KF434341
Hymenoptera;Halictidae;Lasioglossum;Lasioglossum sexnotatum;Germany;GenBank;NA;KX374788
Hymenoptera;Halictidae;Lasioglossum;Lasioglossum brevicorne;Germany;GenBank;NA;KX374792
Hymenoptera;Apidae;Bombus;Bombus konradini;Italy;GenBank;NA;KY317973
Hymenoptera;Apidae;Bombus;Bombus monticola;Switzerland;Bold;GBAH17005-19;KY317975
Hymenoptera;Apidae;Bombus;Bombus monticola;United Kingdom;Bold;GBAH17011-19;KY317976
Hymenoptera;Apidae;Bombus;Bombus monticola;France;Bold;GBAH17007-19;KY317977
Hymenoptera;Apidae;Bombus;Bombus monticola;Sweden;Bold;GBAH17003-19;KY317978
Hymenoptera;Apidae;Bombus;Bombus monticola;Italy;Bold;GBAH17004-19;KY317979
Hymenoptera;Apidae;Bombus;Bombus monticola;Greece;Bold;GBAH17006-19;KY317980
Hymenoptera;Apidae;Bombus;Bombus mucidus;Switzerland;GenBank;NA;MG309777
Hymenoptera;Apidae;Bombus;Bombus haematurus;Austria;GenBank;NA;MH319126
Hymenoptera;Halictidae;Lasioglossum;Lasioglossum laevigatum;Austria;GenBank;NA;MH319135
Hymenoptera;Andrenidae;Nomada;Nomada armata;Austria;GenBank;NA;MH319146
Hymenoptera;Apidae;Andrena;Andrena ovatula;Austria;GenBank;NA;MH319152
Hymenoptera;Halictidae;Halictus;Halictus quadricinctus;Austria;GenBank;NA;MH319165
Hymenoptera;Halictidae;Lasioglossum;Lasioglossum majus;Austria;GenBank;NA;MH319246
Hymenoptera;Apidae;Bombus;Bombus alpinus;Switzerland;Bold;BBBO067-10;NA
Hymenoptera;Apidae;Bombus;Bombus alpinus;Sweden;Bold;WASPS382-14;NA
Hymenoptera;Halictidae;Sphecodes;Sphecodes rubicundus;United Kingdom;Bold;BOWGF169-08;NA
Lepidoptera;Nymphalidae;Euphydryas;Euphydryas aurinia;France;GenBank;NA;AF153920
Lepidoptera;Nymphalidae;Euphydryas;Euphydryas maturna;Finland;GenBank;NA;AF153933
Lepidoptera;Hesperiidae;Carterocephalus;Carterocephalus palaemon;Russia;GenBank;NA;AY585892
Lepidoptera;Hesperiidae;Carterocephalus;Carterocephalus palaemon;Belgium;GenBank;NA;AY585893
Lepidoptera;Hesperiidae;Carterocephalus;Carterocephalus palaemon;Belgium;GenBank;NA;AY585894
Lepidoptera;Pieridae;Pieris;Pieris rapae;Sweden;GenBank;NA;DQ148916
Lepidoptera;Papilionidae;Parnassius;Parnassius apollo;Russia;GenBank;NA;EF514429
Lepidoptera;Papilionidae;Papilio;Papilio hospiton;Italy;GenBank;NA;EF514438
Lepidoptera;Papilionidae;Papilio;Papilio hospiton;Italy;GenBank;NA;EF514443
Lepidoptera;Papilionidae;Parnassius;Parnassius apollo;Russia;GenBank;NA;EF514457
Lepidoptera;Nymphalidae;Erebia;Erebia euryale;Slovakia;GenBank;NA;FJ628423
Lepidoptera;Nymphalidae;Erebia;Erebia euryale;Slovakia;GenBank;NA;FJ628424
Lepidoptera;Nymphalidae;Erebia;Erebia medusa;Slovakia;GenBank;NA;FJ628425
Lepidoptera;Nymphalidae;Erebia;Erebia manto;Slovakia;GenBank;NA;FJ628427
Lepidoptera;Nymphalidae;Erebia;Erebia euryale;Slovakia;GenBank;NA;FJ628428
Lepidoptera;Nymphalidae;Erebia;Erebia manto;Slovakia;GenBank;NA;FJ628429
Lepidoptera;Nymphalidae;Erebia;Erebia gorge;Slovakia;GenBank;NA;FJ628430
Lepidoptera;Nymphalidae;Erebia;Erebia ligea;Slovakia;GenBank;NA;FJ628431
Lepidoptera;Nymphalidae;Erebia;Erebia epiphron;Slovakia;GenBank;NA;FJ628432
Lepidoptera;Nymphalidae;Erebia;Erebia epiphron;Slovakia;GenBank;NA;FJ628433
Lepidoptera;Nymphalidae;Erebia;Erebia aethiops;Slovakia;GenBank;NA;FJ628434
Lepidoptera;Nymphalidae;Erebia;Erebia pronoe;Slovakia;GenBank;NA;FJ628435
Lepidoptera;Nymphalidae;Erebia;Erebia euryale;Slovakia;GenBank;NA;FJ628437
Lepidoptera;Nymphalidae;Erebia;Erebia euryale;Slovakia;GenBank;NA;FJ628438
Lepidoptera;Nymphalidae;Erebia;Erebia pronoe;Slovakia;GenBank;NA;FJ628439
Lepidoptera;Nymphalidae;Erebia;Erebia euryale;Slovakia;GenBank;NA;FJ628440
Lepidoptera;Nymphalidae;Erebia;Erebia euryale;Slovakia;GenBank;NA;FJ628441
Lepidoptera;Nymphalidae;Erebia;Erebia euryale;Slovakia;GenBank;NA;FJ628442
Lepidoptera;Nymphalidae;Erebia;Erebia euryale;Slovakia;GenBank;NA;FJ628443

Lepidoptera;Nymphalidae;Erebia;Erebia medusa;Slovakia;GenBank;NA;FJ628444
Lepidoptera;Nymphalidae;Erebia;Erebia pharte;Slovakia;GenBank;NA;FJ628447
Lepidoptera;Pieridae;Euchloe;Euchloe crameri;Spain;Bold;GBGL6385-09;FM196449
Lepidoptera;Pieridae;Euchloe;Euchloe ausonia;Russia;Bold;GBGL6380-09;FM196454
Lepidoptera;Pieridae;Euchloe;Euchloe simplonia;France;Bold;GBGL6377-09;FM196457
Lepidoptera;Pieridae;Euchloe;Euchloe insularis;Italy;Bold;GBGL6374-09;FM196460
Lepidoptera;Pieridae;Euchloe;Euchloe ausonia;Italy;Bold;GBGL6357-09;FM196477
Lepidoptera;Pieridae;Euchloe;Euchloe ausonia taurica;Cyprus;GenBank;NA;FM196478
Lepidoptera;Pieridae;Euchloe;Euchloe simplonia;Switzerland;Bold;GBGL6353-09;FM196481
Lepidoptera;Pieridae;Euchloe;Euchloe crameri;Italy;Bold;GBGL6352-09;FM196482
Lepidoptera;Pieridae;Euchloe;Euchloe insularis;France;Bold;GBGL6351-09;FM196483
Lepidoptera;Pieridae;Euchloe;Euchloe simplonia;Italy;Bold;GBGL6337-09;FM196497
Lepidoptera;Pieridae;Euchloe;Euchloe simplonia;Italy;Bold;GBGL6336-09;FM196498
Lepidoptera;Pieridae;Euchloe;Euchloe crameri;Italy;Bold;GBGL6335-09;FM196499
Lepidoptera;Pieridae;Euchloe;Euchloe ausonia;Italy;Bold;GBGL6332-09;FM196502
Lepidoptera;Pieridae;Euchloe;Euchloe tagis bellezina;France;GenBank;NA;FM196520
Lepidoptera;Pieridae;Euchloe;Euchloe ausonia;Russia;Bold;GBGL6310-09;FM196524
Lepidoptera;Nymphalidae;Melanargia;Melanargia arge;Italy;GenBank;NA;GQ200901
Lepidoptera;Nymphalidae;Melanargia;Melanargia arge;Italy;GenBank;NA;GQ200902
Lepidoptera;Nymphalidae;Melanargia;Melanargia arge;Italy;GenBank;NA;GQ200903
Lepidoptera;Nymphalidae;Melanargia;Melanargia arge;Italy;GenBank;NA;GQ200904
Lepidoptera;Nymphalidae;Melanargia;Melanargia galathea;Germany;GenBank;NA;GQ200925
Lepidoptera;Nymphalidae;Melanargia;Melanargia galathea;Macedonia;GenBank;NA;GQ200926
Lepidoptera;Nymphalidae;Melanargia;Melanargia galathea;Greece;GenBank;NA;GQ200927
Lepidoptera;Nymphalidae;Melanargia;Melanargia galathea;Turkey;GenBank;NA;GQ200928
Lepidoptera;Nymphalidae;Melanargia;Melanargia galathea;Turkey;GenBank;NA;GQ200929
Lepidoptera;Nymphalidae;Melanargia;Melanargia galathea;Greece;GenBank;NA;GQ200931
Lepidoptera;Nymphalidae;Melanargia;Melanargia galathea;Greece;GenBank;NA;GQ200932
Lepidoptera;Nymphalidae;Melanargia;Melanargia galathea;Greece;GenBank;NA;GQ200933
Lepidoptera;Nymphalidae;Melanargia;Melanargia galathea;Macedonia;GenBank;NA;GQ200934
Lepidoptera;Nymphalidae;Melanargia;Melanargia galathea;Macedonia;GenBank;NA;GQ200935
Lepidoptera;Nymphalidae;Melanargia;Melanargia galathea;Macedonia;GenBank;NA;GQ200937
Lepidoptera;Nymphalidae;Melanargia;Melanargia galathea;Macedonia;GenBank;NA;GQ200938
Lepidoptera;Nymphalidae;Melanargia;Melanargia galathea;Greece;GenBank;NA;GQ200939
Lepidoptera;Nymphalidae;Melanargia;Melanargia galathea;Greece;GenBank;NA;GQ200940
Lepidoptera;Nymphalidae;Melanargia;Melanargia galathea;Russia;GenBank;NA;GQ200942
Lepidoptera;Nymphalidae;Melanargia;Melanargia galathea;Russia;GenBank;NA;GQ200943
Lepidoptera;Nymphalidae;Melanargia;Melanargia galathea;Italy;GenBank;NA;GQ200944
Lepidoptera;Nymphalidae;Melanargia;Melanargia galathea;Italy;GenBank;NA;GQ200945
Lepidoptera;Nymphalidae;Melanargia;Melanargia occitanica;France;GenBank;NA;GQ201102
Lepidoptera;Nymphalidae;Melanargia;Melanargia occitanica;France;GenBank;NA;GQ201103
Lepidoptera;Nymphalidae;Melanargia;Melanargia occitanica;France;GenBank;NA;GQ201106
Lepidoptera;Nymphalidae;Melanargia;Melanargia occitanica;France;GenBank;NA;GQ201107
Lepidoptera;Nymphalidae;Melanargia;Melanargia occitanica;Italy;GenBank;NA;GQ201115
Lepidoptera;Nymphalidae;Melanargia;Melanargia occitanica;Italy;GenBank;NA;GQ201116
Lepidoptera;Nymphalidae;Melanargia;Melanargia occitanica;Spain;GenBank;NA;GQ201117
Lepidoptera;Nymphalidae;Melanargia;Melanargia occitanica;Spain;GenBank;NA;GQ201118
Lepidoptera;Nymphalidae;Melanargia;Melanargia occitanica;Italy;GenBank;NA;GQ201119
Lepidoptera;Nymphalidae;Melanargia;Melanargia russiae;France;GenBank;NA;GQ201132
Lepidoptera;Nymphalidae;Melanargia;Melanargia russiae;Italy;GenBank;NA;GQ201133
Lepidoptera;Nymphalidae;Melanargia;Melanargia russiae;France;GenBank;NA;GQ201141
Lepidoptera;Nymphalidae;Melanargia;Melanargia russiae;Turkey;GenBank;NA;GQ201146
Lepidoptera;Nymphalidae;Melanargia;Melanargia russiae;Italy;GenBank;NA;GQ201147
Lepidoptera;Nymphalidae;Melanargia;Melanargia russiae;Italy;GenBank;NA;GQ201148
Lepidoptera;Nymphalidae;Melanargia;Melanargia russiae;Russia;GenBank;NA;GQ201157
Lepidoptera;Nymphalidae;Melanargia;Melanargia russiae;Russia;GenBank;NA;GQ201158
Lepidoptera;Nymphalidae;Melanargia;Melanargia russiae;Russia;GenBank;NA;GQ201159
Lepidoptera;Papilionidae;Papilio;Papilio machaon;Spain;GenBank;NA;GU675943
Lepidoptera;Hesperiidae;Pyrgus;Pyrgus armoricanus;Bulgaria;GenBank;NA;GU676051
Lepidoptera;Hesperiidae;Pyrgus;Pyrgus alveus;Germany;GenBank;NA;GU687041
Lepidoptera;Lycaenidae;Cyaniris;Cyaniris semiargus;Austria;GenBank;NA;HM393232
Lepidoptera;Lycaenidae;Agriades;Agriades glandon;Spain;GenBank;NA;HM401000
Lepidoptera;Lycaenidae;Agriades;Agriades glandon;Spain;GenBank;NA;HM401001
Lepidoptera;Lycaenidae;Agriades;Agriades glandon;Andorra;GenBank;NA;HM401002
Lepidoptera;Lycaenidae;Aricia;Aricia nicias;Spain;GenBank;NA;HM401003
Lepidoptera;Lycaenidae;Aricia;Aricia nicias;Spain;GenBank;NA;HM401004
Lepidoptera;Lycaenidae;Aricia;Aricia nicias;Spain;GenBank;NA;HM401005
Lepidoptera;Lycaenidae;Aricia;Aricia nicias;Spain;GenBank;NA;HM401006
Lepidoptera;Lycaenidae;Aricia;Aricia nicias;Spain;GenBank;NA;HM401007
Lepidoptera;Lycaenidae;Aricia;Aricia nicias;Spain;GenBank;NA;HM401008
Lepidoptera;Lycaenidae;Satyrium;Satyrium w-album;Finland;GenBank;NA;HM872811
Lepidoptera;Papilionidae;Parnassius;Parnassius mnemosyne;Estonia;GenBank;NA;HM872929
Lepidoptera;Lycaenidae;Lycaena;Lycaena tityrus;Estonia;GenBank;NA;HM872932
Lepidoptera;Hesperiidae;Pyrgus;Pyrgus serratulae;Estonia;GenBank;NA;HM872933
Lepidoptera;Nymphalidae;Boloria;Boloria dia;Estonia;GenBank;NA;HM872934
Lepidoptera;Pieridae;Leptidea;Leptidea juvernica;Estonia;GenBank;NA;HM872935
Lepidoptera;Nymphalidae;Euphydryas;Euphydryas aurinia;Estonia;GenBank;NA;HM872936
Lepidoptera;Nymphalidae;Nymphalis;Nymphalis polychloros;Estonia;GenBank;NA;HM873021

Lepidoptera;Nymphalidae;Hipparchia;Hipparchia statilinus;Spain;GenBank;NA;HM901231
Lepidoptera;Nymphalidae;Melanargia;Melanargia russiae;Portugal;GenBank;NA;HM901237
Lepidoptera;Nymphalidae;Coenonympha;Coenonympha dorus;Portugal;GenBank;NA;HM901238
Lepidoptera;Nymphalidae;Erebia;Erebia pronoe;Spain;GenBank;NA;HM901244
Lepidoptera;Nymphalidae;Erebia;Erebia pronoe;Spain;GenBank;NA;HM901254
Lepidoptera;Nymphalidae;Erebia;Erebia pronoe;Spain;GenBank;NA;HM901258
Lepidoptera;Nymphalidae;Erebia;Erebia pronoe;Spain;GenBank;NA;HM901260
Lepidoptera;Nymphalidae;Erebia;Erebia pronoe;Spain;GenBank;NA;HM901267
Lepidoptera;Hesperiidae;Pyrgus;Pyrgus onopordi;Spain;GenBank;NA;HM901359
Lepidoptera;Hesperiidae;Pyrgus;Pyrgus armoricanus;Spain;GenBank;NA;HM901360
Lepidoptera;Hesperiidae;Pyrgus;Pyrgus armoricanus;Spain;GenBank;NA;HM901361
Lepidoptera;Nymphalidae;Melitaea;Melitaea diamina;Spain;GenBank;NA;HM901373
Lepidoptera;Nymphalidae;Melitaea;Melitaea diamina;Spain;GenBank;NA;HM901374
Lepidoptera;Riodinidae;Hamearis;Hamearis lucina;Spain;GenBank;NA;HM901376
Lepidoptera;Nymphalidae;Limenitis;Limenitis reducta;Spain;GenBank;NA;HM901393
Lepidoptera;Nymphalidae;Limenitis;Limenitis reducta;Spain;GenBank;NA;HM901394
Lepidoptera;Nymphalidae;Erebia;Erebia euryale;Spain;GenBank;NA;HM901449
Lepidoptera;Nymphalidae;Erebia;Erebia euryale;Spain;GenBank;NA;HM901450
Lepidoptera;Nymphalidae;Melitaea;Melitaea cinxia;Spain;GenBank;NA;HM901487
Lepidoptera;Lycaenidae;Lycaena;Lycaena hippothoe;Spain;GenBank;NA;HM901488
Lepidoptera;Lycaenidae;Lycaena;Lycaena hippothoe;Spain;GenBank;NA;HM901489
Lepidoptera;Lycaenidae;Satyrium;Satyrium acaciae;Spain;GenBank;NA;HM901490
Lepidoptera;Nymphalidae;Erebia;Erebia euryale;Spain;GenBank;NA;HM901497
Lepidoptera;Nymphalidae;Erebia;Erebia epiphron;Spain;GenBank;NA;HM901499
Lepidoptera;Nymphalidae;Hipparchia;Hipparchia statilinus;Spain;GenBank;NA;HM901500
Lepidoptera;Nymphalidae;Hipparchia;Hipparchia statilinus;Spain;GenBank;NA;HM901501
Lepidoptera;Nymphalidae;Chazara;Chazara briseis;Spain;GenBank;NA;HM901502
Lepidoptera;Nymphalidae;Erebia;Erebia neoridas;Spain;GenBank;NA;HM901503
Lepidoptera;Nymphalidae;Erebia;Erebia neoridas;Spain;GenBank;NA;HM901504
Lepidoptera;Nymphalidae;Erebia;Erebia oeme;Spain;GenBank;NA;HM901505
Lepidoptera;Nymphalidae;Araschnia;Araschnia levana;Spain;GenBank;NA;HM901511
Lepidoptera;Nymphalidae;Arethusana;Arethusana arethusa;Spain;GenBank;NA;HM901530
Lepidoptera;Nymphalidae;Arethusana;Arethusana arethusa;Spain;GenBank;NA;HM901531
Lepidoptera;Lycaenidae;Cupido;Cupido minimus;Spain;GenBank;NA;HM901532
Lepidoptera;Lycaenidae;Cupido;Cupido osiris;Spain;GenBank;NA;HM901533
Lepidoptera;Lycaenidae;Cupido;Cupido osiris;Spain;GenBank;NA;HM901534
Lepidoptera;Lycaenidae;Cupido;Cupido osiris;Spain;GenBank;NA;HM901535
Lepidoptera;Lycaenidae;Cupido;Cupido osiris;Spain;GenBank;NA;HM901536
Lepidoptera;Nymphalidae;Melitaea;Melitaea nevadensis;Spain;GenBank;NA;HM901538
Lepidoptera;Nymphalidae;Melitaea;Melitaea nevadensis;Spain;GenBank;NA;HM901539
Lepidoptera;Nymphalidae;Melitaea;Melitaea nevadensis;Spain;GenBank;NA;HM901540
Lepidoptera;Nymphalidae;Arethusana;Arethusana arethusa;Spain;GenBank;NA;HM901543
Lepidoptera;Nymphalidae;Arethusana;Arethusana arethusa;Spain;GenBank;NA;HM901544
Lepidoptera;Nymphalidae;Limenitis;Limenitis camilla;Spain;GenBank;NA;HM901620
Lepidoptera;Nymphalidae;Erebia;Erebia gorge;Spain;GenBank;NA;HM901656
Lepidoptera;Nymphalidae;Erebia;Erebia gorge;Spain;GenBank;NA;HM901657
Lepidoptera;Nymphalidae;Erebia;Erebia gorge;Spain;GenBank;NA;HM901658
Lepidoptera;Nymphalidae;Erebia;Erebia gorge;Spain;GenBank;NA;HM901659
Lepidoptera;Nymphalidae;Erebia;Erebia pronoe;Spain;GenBank;NA;HM901710
Lepidoptera;Nymphalidae;Erebia;Erebia pronoe;Spain;GenBank;NA;HM901711
Lepidoptera;Nymphalidae;Apatura;Apatura ilia;Spain;GenBank;NA;HM901712
Lepidoptera;Nymphalidae;Apatura;Apatura iris;Spain;GenBank;NA;HM901713
Lepidoptera;Nymphalidae;Apatura;Apatura ilia;Spain;GenBank;NA;HM901714
Lepidoptera;Pieridae;Colias;Colias phicomone;Spain;GenBank;NA;HM901717
Lepidoptera;Pieridae;Colias;Colias phicomone;Spain;GenBank;NA;HM901718
Lepidoptera;Nymphalidae;Boloria;Boloria euphrosyne;Spain;GenBank;NA;HM901719
Lepidoptera;Nymphalidae;Boloria;Boloria euphrosyne;Spain;GenBank;NA;HM901720
Lepidoptera;Pieridae;Euchloe;Euchloe simplonia;Spain;GenBank;NA;HM901723
Lepidoptera;Nymphalidae;Brenthis;Brenthis ino;Spain;GenBank;NA;HM901756
Lepidoptera;Papilionidae;Parnassius;Parnassius mnemosyne;Spain;GenBank;NA;HM901765
Lepidoptera;Nymphalidae;Coenonympha;Coenonympha glycerion;Spain;GenBank;NA;HM901767
Lepidoptera;Nymphalidae;Brenthis;Brenthis ino;Spain;GenBank;NA;HM901768
Lepidoptera;Nymphalidae;Coenonympha;Coenonympha glycerion;Spain;GenBank;NA;HM901769
Lepidoptera;Hesperiidae;Thymelicus;Thymelicus sylvestris;Spain;GenBank;NA;HM901771
Lepidoptera;Nymphalidae;Erebia;Erebia gorge;Spain;GenBank;NA;HM901774
Lepidoptera;Nymphalidae;Boloria;Boloria pales;Spain;GenBank;NA;HM901775
Lepidoptera;Nymphalidae;Arethusana;Arethusana arethusa;Spain;GenBank;NA;HM901779
Lepidoptera;Nymphalidae;Boloria;Boloria euphrosyne;Spain;GenBank;NA;HM901781
Lepidoptera;Nymphalidae;Boloria;Boloria pales;Italy;GenBank;NA;HM901782
Lepidoptera;Nymphalidae;Erebia;Erebia pronoe;Spain;GenBank;NA;HM901783
Lepidoptera;Nymphalidae;Erebia;Erebia pronoe;Spain;GenBank;NA;HM901784
Lepidoptera;Nymphalidae;Erebia;Erebia pronoe;Spain;GenBank;NA;HM901785
Lepidoptera;Nymphalidae;Erebia;Erebia pronoe;Spain;GenBank;NA;HM901786
Lepidoptera;Nymphalidae;Erebia;Erebia pronoe;Spain;GenBank;NA;HM901787
Lepidoptera;Hesperiidae;Muschampia;Muschampia proto;Spain;GenBank;NA;HM901823
Lepidoptera;Nymphalidae;Melitaea;Melitaea cinxia;Italy;GenBank;NA;HM910512
Lepidoptera;Lycaenidae;Cupido;Cupido minimus;Italy;GenBank;NA;HM913961
Lepidoptera;Lycaenidae;Glaucopsyche;Glaucopsyche alexis;Italy;GenBank;NA;HM913963

Lepidoptera;Riodinidae;Hamearis;Hamearis lucina;Italy;GenBank;NA;HM913970
Lepidoptera;Nymphalidae;Melitaea;Melitaea trivia;Ukraine;GenBank;NA;HM998304
Lepidoptera;Nymphalidae;Melitaea;Melitaea trivia;Ukraine;GenBank;NA;HM998305
Lepidoptera;Nymphalidae;Melitaea;Melitaea trivia;Ukraine;GenBank;NA;HM998306
Lepidoptera;Hesperiidae;Pyrgus;Pyrgus cacaliae;Germany;GenBank;NA;HQ957213
Lepidoptera;Nymphalidae;Boloria;Boloria thore;Germany;GenBank;NA;HQ957214
Lepidoptera;Hesperiidae;Pyrgus;Pyrgus sidae;Romania;GenBank;NA;HQ996661
Lepidoptera;Hesperiidae;Pyrgus;Pyrgus sidae;Romania;GenBank;NA;HQ996662
Lepidoptera;Hesperiidae;Pyrgus;Pyrgus sidae;Romania;GenBank;NA;HQ996663
Lepidoptera;Hesperiidae;Pyrgus;Pyrgus sidae;Romania;GenBank;NA;HQ996664
Lepidoptera;Pieridae;Leptidea;Leptidea juvernica;Romania;GenBank;NA;JF512573
Lepidoptera;Pieridae;Leptidea;Leptidea juvernica;Russia;GenBank;NA;JF512574
Lepidoptera;Pieridae;Leptidea;Leptidea juvernica;Russia;GenBank;NA;JF512575
Lepidoptera;Pieridae;Leptidea;Leptidea juvernica;Russia;GenBank;NA;JF512576
Lepidoptera;Pieridae;Leptidea;Leptidea juvernica;Russia;GenBank;NA;JF512577
Lepidoptera;Pieridae;Leptidea;Leptidea juvernica;Russia;GenBank;NA;JF512578
Lepidoptera;Pieridae;Leptidea;Leptidea sinapis;Romania;GenBank;NA;JF512580
Lepidoptera;Pieridae;Leptidea;Leptidea sinapis;Romania;GenBank;NA;JF512581
Lepidoptera;Pieridae;Leptidea;Leptidea sinapis;Romania;GenBank;NA;JF512582
Lepidoptera;Pieridae;Leptidea;Leptidea sinapis;Romania;GenBank;NA;JF512584
Lepidoptera;Pieridae;Leptidea;Leptidea sinapis;France;GenBank;NA;JF512585
Lepidoptera;Pieridae;Leptidea;Leptidea sinapis;France;GenBank;NA;JF512586
Lepidoptera;Pieridae;Leptidea;Leptidea sinapis;France;GenBank;NA;JF512587
Lepidoptera;Pieridae;Leptidea;Leptidea sinapis;Spain;GenBank;NA;JF512589
Lepidoptera;Pieridae;Leptidea;Leptidea sinapis;Spain;GenBank;NA;JF512590
Lepidoptera;Pieridae;Leptidea;Leptidea sinapis;Italy;GenBank;NA;JF512591
Lepidoptera;Pieridae;Leptidea;Leptidea sinapis;Romania;GenBank;NA;JF512592
Lepidoptera;Pieridae;Leptidea;Leptidea sinapis;Italy;GenBank;NA;JF512593
Lepidoptera;Pieridae;Leptidea;Leptidea sinapis;Spain;GenBank;NA;JF512594
Lepidoptera;Pieridae;Leptidea;Leptidea sinapis;Italy;GenBank;NA;JF512595
Lepidoptera;Pieridae;Leptidea;Leptidea sinapis;Italy;GenBank;NA;JF512596
Lepidoptera;Pieridae;Leptidea;Leptidea sinapis;Italy;GenBank;NA;JF512597
Lepidoptera;Pieridae;Leptidea;Leptidea sinapis;France;GenBank;NA;JF512598
Lepidoptera;Pieridae;Leptidea;Leptidea sinapis;France;GenBank;NA;JF512599
Lepidoptera;Pieridae;Leptidea;Leptidea sinapis;France;GenBank;NA;JF512600
Lepidoptera;Pieridae;Leptidea;Leptidea reali;Spain;GenBank;NA;JF512603
Lepidoptera;Pieridae;Leptidea;Leptidea reali;France;GenBank;NA;JF512604
Lepidoptera;Pieridae;Leptidea;Leptidea reali;France;GenBank;NA;JF512605
Lepidoptera;Pieridae;Leptidea;Leptidea reali;France;GenBank;NA;JF512606
Lepidoptera;Pieridae;Leptidea;Leptidea reali;France;GenBank;NA;JF512607
Lepidoptera;Pieridae;Leptidea;Leptidea reali;France;GenBank;NA;JF512608
Lepidoptera;Pieridae;Leptidea;Leptidea reali;Italy;GenBank;NA;JF512609
Lepidoptera;Pieridae;Leptidea;Leptidea reali;Italy;GenBank;NA;JF512610
Lepidoptera;Pieridae;Leptidea;Leptidea reali;Italy;GenBank;NA;JF512611
Lepidoptera;Pieridae;Leptidea;Leptidea reali;Italy;GenBank;NA;JF512612
Lepidoptera;Pieridae;Leptidea;Leptidea reali;Italy;GenBank;NA;JF512613
Lepidoptera;Pieridae;Leptidea;Leptidea reali;Italy;GenBank;NA;JF512614
Lepidoptera;Pieridae;Leptidea;Leptidea reali;Italy;GenBank;NA;JF512615
Lepidoptera;Pieridae;Leptidea;Leptidea reali;Italy;GenBank;NA;JF512616
Lepidoptera;Pieridae;Leptidea;Leptidea reali;Spain;GenBank;NA;JF512617
Lepidoptera;Pieridae;Leptidea;Leptidea sinapis;Spain;GenBank;NA;JF512623
Lepidoptera;Pieridae;Leptidea;Leptidea juvernica;Romania;GenBank;NA;JF512628
Lepidoptera;Pieridae;Leptidea;Leptidea juvernica;Russia;GenBank;NA;JF512629
Lepidoptera;Pieridae;Leptidea;Leptidea juvernica;Russia;GenBank;NA;JF512630
Lepidoptera;Pieridae;Leptidea;Leptidea juvernica;Russia;GenBank;NA;JF512631
Lepidoptera;Pieridae;Leptidea;Leptidea juvernica;Russia;GenBank;NA;JF512632
Lepidoptera;Pieridae;Leptidea;Leptidea juvernica;Russia;GenBank;NA;JF512633
Lepidoptera;Pieridae;Leptidea;Leptidea juvernica;Russia;GenBank;NA;JF512634
Lepidoptera;Pieridae;Leptidea;Leptidea juvernica;Romania;GenBank;NA;JF512635
Lepidoptera;Pieridae;Leptidea;Leptidea juvernica;Romania;GenBank;NA;JF512636
Lepidoptera;Pieridae;Leptidea;Leptidea juvernica;Romania;GenBank;NA;JF512637
Lepidoptera;Pieridae;Leptidea;Leptidea juvernica;Romania;GenBank;NA;JF512638
Lepidoptera;Pieridae;Leptidea;Leptidea juvernica;Romania;GenBank;NA;JF512639
Lepidoptera;Pieridae;Leptidea;Leptidea juvernica;Romania;GenBank;NA;JF512640
Lepidoptera;Pieridae;Leptidea;Leptidea juvernica;Romania;GenBank;NA;JF512641
Lepidoptera;Pieridae;Leptidea;Leptidea juvernica;Romania;GenBank;NA;JF512642
Lepidoptera;Pieridae;Leptidea;Leptidea juvernica;Romania;GenBank;NA;JF512643
Lepidoptera;Pieridae;Leptidea;Leptidea juvernica;Romania;GenBank;NA;JF512644
Lepidoptera;Pieridae;Leptidea;Leptidea juvernica;Czech Republic;GenBank;NA;JF512645
Lepidoptera;Pieridae;Leptidea;Leptidea juvernica;Slovenia;GenBank;NA;JF512646
Lepidoptera;Pieridae;Leptidea;Leptidea juvernica;Russia;GenBank;NA;JF512649
Lepidoptera;Pieridae;Leptidea;Leptidea juvernica;France;GenBank;NA;JF512650
Lepidoptera;Pieridae;Leptidea;Leptidea juvernica;France;GenBank;NA;JF512651
Lepidoptera;Pieridae;Leptidea;Leptidea juvernica;France;GenBank;NA;JF512652
Lepidoptera;Pieridae;Leptidea;Leptidea juvernica;France;GenBank;NA;JF512653
Lepidoptera;Pieridae;Leptidea;Leptidea juvernica;France;GenBank;NA;JF512654
Lepidoptera;Pieridae;Leptidea;Leptidea juvernica;France;GenBank;NA;JF512655
Lepidoptera;Pieridae;Leptidea;Leptidea reali;Spain;GenBank;NA;JF512656

Lepidoptera;Pieridae;Leptidea;Leptidea reali;Spain;GenBank;NA;JF512657
Lepidoptera;Pieridae;Leptidea;Leptidea reali;Italy;GenBank;NA;JF512658
Lepidoptera;Pieridae;Leptidea;Leptidea reali;Italy;GenBank;NA;JF512659
Lepidoptera;Pieridae;Leptidea;Leptidea reali;Italy;GenBank;NA;JF512660
Lepidoptera;Pieridae;Leptidea;Leptidea reali;Spain;GenBank;NA;JF512661
Lepidoptera;Pieridae;Leptidea;Leptidea sinapis;Romania;GenBank;NA;JF512662
Lepidoptera;Pieridae;Leptidea;Leptidea sinapis;Spain;GenBank;NA;JF512663
Lepidoptera;Pieridae;Leptidea;Leptidea sinapis;Italy;GenBank;NA;JF512665
Lepidoptera;Pieridae;Leptidea;Leptidea sinapis;Italy;GenBank;NA;JF512666
Lepidoptera;Pieridae;Leptidea;Leptidea sinapis;Spain;GenBank;NA;JF512667
Lepidoptera;Pieridae;Leptidea;Leptidea sinapis;Spain;GenBank;NA;JF512668
Lepidoptera;Pieridae;Leptidea;Leptidea reali;Spain;GenBank;NA;JF512669
Lepidoptera;Pieridae;Leptidea;Leptidea reali;Spain;GenBank;NA;JF512670
Lepidoptera;Pieridae;Leptidea;Leptidea reali;France;GenBank;NA;JF512671
Lepidoptera;Pieridae;Leptidea;Leptidea reali;France;GenBank;NA;JF512672
Lepidoptera;Pieridae;Leptidea;Leptidea reali;France;GenBank;NA;JF512673
Lepidoptera;Pieridae;Leptidea;Leptidea reali;France;GenBank;NA;JF512674
Lepidoptera;Pieridae;Leptidea;Leptidea reali;France;GenBank;NA;JF512675
Lepidoptera;Pieridae;Leptidea;Leptidea reali;Spain;GenBank;NA;JF512676
Lepidoptera;Pieridae;Leptidea;Leptidea reali;Spain;GenBank;NA;JF512677
Lepidoptera;Pieridae;Leptidea;Leptidea reali;Spain;GenBank;NA;JF512678
Lepidoptera;Pieridae;Leptidea;Leptidea reali;Spain;GenBank;NA;JF512679
Lepidoptera;Pieridae;Leptidea;Leptidea reali;Spain;GenBank;NA;JF512680
Lepidoptera;Pieridae;Leptidea;Leptidea reali;Spain;GenBank;NA;JF512681
Lepidoptera;Pieridae;Leptidea;Leptidea reali;Spain;GenBank;NA;JF512682
Lepidoptera;Pieridae;Leptidea;Leptidea reali;Spain;GenBank;NA;JF512683
Lepidoptera;Pieridae;Leptidea;Leptidea reali;Spain;GenBank;NA;JF512684
Lepidoptera;Pieridae;Leptidea;Leptidea reali;Spain;GenBank;NA;JF512685
Lepidoptera;Pieridae;Leptidea;Leptidea reali;Spain;GenBank;NA;JF512686
Lepidoptera;Pieridae;Leptidea;Leptidea sinapis;Ireland;GenBank;NA;JF512687
Lepidoptera;Pieridae;Leptidea;Leptidea sinapis;Ireland;GenBank;NA;JF512688
Lepidoptera;Pieridae;Leptidea;Leptidea sinapis;Ireland;GenBank;NA;JF512689
Lepidoptera;Pieridae;Leptidea;Leptidea sinapis;Ireland;GenBank;NA;JF512690
Lepidoptera;Pieridae;Leptidea;Leptidea sinapis;Ireland;GenBank;NA;JF512691
Lepidoptera;Pieridae;Leptidea;Leptidea sinapis;Estonia;GenBank;NA;JF512692
Lepidoptera;Pieridae;Leptidea;Leptidea sinapis;Bulgaria;GenBank;NA;JF512693
Lepidoptera;Pieridae;Leptidea;Leptidea sinapis;Croatia;GenBank;NA;JF512694
Lepidoptera;Pieridae;Leptidea;Leptidea sinapis;Croatia;GenBank;NA;JF512695
Lepidoptera;Pieridae;Leptidea;Leptidea sinapis;Ukraine;GenBank;NA;JF512696
Lepidoptera;Pieridae;Leptidea;Leptidea sinapis;Czech Republic;GenBank;NA;JF512697
Lepidoptera;Pieridae;Leptidea;Leptidea sinapis;Czech Republic;GenBank;NA;JF512698
Lepidoptera;Pieridae;Leptidea;Leptidea sinapis;Italy;GenBank;NA;JF512699
Lepidoptera;Pieridae;Leptidea;Leptidea sinapis;Italy;GenBank;NA;JF512700
Lepidoptera;Pieridae;Leptidea;Leptidea sinapis;Italy;GenBank;NA;JF512701
Lepidoptera;Pieridae;Leptidea;Leptidea sinapis;Italy;GenBank;NA;JF512702
Lepidoptera;Pieridae;Leptidea;Leptidea sinapis;Italy;GenBank;NA;JF512703
Lepidoptera;Pieridae;Leptidea;Leptidea reali;Italy;GenBank;NA;JF512704
Lepidoptera;Pieridae;Leptidea;Leptidea sinapis;Italy;GenBank;NA;JF512705
Lepidoptera;Pieridae;Leptidea;Leptidea sinapis;Italy;GenBank;NA;JF512706
Lepidoptera;Pieridae;Leptidea;Leptidea sinapis;Switzerland;GenBank;NA;JF512707
Lepidoptera;Pieridae;Leptidea;Leptidea sinapis;Greece;GenBank;NA;JF512708
Lepidoptera;Pieridae;Leptidea;Leptidea sinapis;Greece;GenBank;NA;JF512709
Lepidoptera;Pieridae;Leptidea;Leptidea sinapis;Slovenia;GenBank;NA;JF512710
Lepidoptera;Pieridae;Leptidea;Leptidea sinapis;Slovenia;GenBank;NA;JF512711
Lepidoptera;Pieridae;Leptidea;Leptidea reali;Italy;GenBank;NA;JF512712
Lepidoptera;Pieridae;Leptidea;Leptidea juvernica;Ireland;GenBank;NA;JF512713
Lepidoptera;Pieridae;Leptidea;Leptidea juvernica;Ireland;GenBank;NA;JF512714
Lepidoptera;Pieridae;Leptidea;Leptidea juvernica;Ireland;GenBank;NA;JF512715
Lepidoptera;Pieridae;Leptidea;Leptidea juvernica;Ireland;GenBank;NA;JF512716
Lepidoptera;Pieridae;Leptidea;Leptidea reali;Spain;GenBank;NA;JF512720
Lepidoptera;Pieridae;Leptidea;Leptidea sinapis;Bulgaria;GenBank;NA;JF512721
Lepidoptera;Pieridae;Leptidea;Leptidea sinapis;Italy;GenBank;NA;JF513008
Lepidoptera;Pieridae;Leptidea;Leptidea sinapis;Italy;GenBank;NA;JF513009
Lepidoptera;Pieridae;Leptidea;Leptidea sinapis;Romania;GenBank;NA;JF513010
Lepidoptera;Pieridae;Leptidea;Leptidea sinapis;Italy;GenBank;NA;JF513011
Lepidoptera;Pieridae;Leptidea;Leptidea sinapis;Spain;GenBank;NA;JF513013
Lepidoptera;Pieridae;Leptidea;Leptidea sinapis;Spain;GenBank;NA;JF513014
Lepidoptera;Pieridae;Leptidea;Leptidea sinapis;Spain;GenBank;NA;JF513015
Lepidoptera;Pieridae;Leptidea;Leptidea sinapis;Spain;GenBank;NA;JF513016
Lepidoptera;Pieridae;Leptidea;Leptidea sinapis;Romania;GenBank;NA;JF513017
Lepidoptera;Pieridae;Leptidea;Leptidea sinapis;Romania;GenBank;NA;JF513018
Lepidoptera;Pieridae;Leptidea;Leptidea sinapis;Romania;GenBank;NA;JF513019
Lepidoptera;Pieridae;Leptidea;Leptidea sinapis;Romania;GenBank;NA;JF513020
Lepidoptera;Pieridae;Leptidea;Leptidea sinapis;Romania;GenBank;NA;JF513021
Lepidoptera;Pieridae;Leptidea;Leptidea sinapis;Romania;GenBank;NA;JF513022
Lepidoptera;Pieridae;Leptidea;Leptidea sinapis;Romania;GenBank;NA;JF513023
Lepidoptera;Pieridae;Leptidea;Leptidea sinapis;Romania;GenBank;NA;JF513026
Lepidoptera;Pieridae;Leptidea;Leptidea sinapis;Romania;GenBank;NA;JF513029

Lepidoptera;Pieridae;Leptidea;Leptidea sinapis;Romania;GenBank;NA;JF513031
Lepidoptera;Pieridae;Leptidea;Leptidea sinapis;Italy;GenBank;NA;JF513033
Lepidoptera;Pieridae;Leptidea;Leptidea sinapis;France;GenBank;NA;JF513034
Lepidoptera;Pieridae;Leptidea;Leptidea sinapis;Romania;GenBank;NA;JF513036
Lepidoptera;Pieridae;Leptidea;Leptidea sinapis;Romania;GenBank;NA;JF513037
Lepidoptera;Pieridae;Leptidea;Leptidea sinapis;Italy;GenBank;NA;JF513038
Lepidoptera;Pieridae;Leptidea;Leptidea sinapis;Romania;GenBank;NA;JF513039
Lepidoptera;Pieridae;Leptidea;Leptidea sinapis;Spain;GenBank;NA;JF513040
Lepidoptera;Pieridae;Leptidea;Leptidea sinapis;Spain;GenBank;NA;JF513041
Lepidoptera;Pieridae;Leptidea;Leptidea sinapis;Spain;GenBank;NA;JF513042
Lepidoptera;Pieridae;Leptidea;Leptidea sinapis;Spain;GenBank;NA;JF513043
Lepidoptera;Pieridae;Leptidea;Leptidea sinapis;Spain;GenBank;NA;JF513044
Lepidoptera;Pieridae;Leptidea;Leptidea sinapis;Spain;GenBank;NA;JF513045
Lepidoptera;Lycaenidae;Satyrium;Satyrium pruni;Finland;GenBank;NA;JF853830
Lepidoptera;Lycaenidae;Lycaena;Lycaena dispar;Russia;GenBank;NA;JF854415
Lepidoptera;Lycaenidae;Lycaena;Lycaena dispar;Russia;GenBank;NA;JF854416
Lepidoptera;Lycaenidae;Scolitantides;Scolitantides orion;Russia;GenBank;NA;JF854417
Lepidoptera;Nymphalidae;Erebia;Erebia euryale;Russia;GenBank;NA;JF854419
Lepidoptera;Nymphalidae;Erebia;Erebia euryale;Russia;GenBank;NA;JF854420
Lepidoptera;Hesperiidae;Heteropterus;Heteropterus morpheus;Russia;GenBank;NA;JF854423
Lepidoptera;Hesperiidae;Heteropterus;Heteropterus morpheus;Russia;GenBank;NA;JF854424
Lepidoptera;Nymphalidae;Lasiommata;Lasiommata megera;Greece;GenBank;NA;JF854491
Lepidoptera;Nymphalidae;Lasiommata;Lasiommata megera;Hungary;GenBank;NA;JF854492
Lepidoptera;Papilionidae;Iphiclides;Iphiclides podalirius;Russia;GenBank;NA;JF854496
Lepidoptera;Nymphalidae;Boloria;Boloria titania;Switzerland;GenBank;NA;JF860243
Lepidoptera;Lycaenidae;Phengaris;Phengaris alcon alcon;Poland;GenBank;NA;JN160200
Lepidoptera;Lycaenidae;Phengaris;Phengaris alcon rebeli;Poland;GenBank;NA;JN160201
Lepidoptera;Lycaenidae;Phengaris;Phengaris alcon rebeli;Lithuania;GenBank;NA;JN160202
Lepidoptera;Nymphalidae;Charaxes;Charaxes jasius;Spain;GenBank;NA;JN264880
Lepidoptera;Nymphalidae;Melitaea;Melitaea cinxia;Spain;GenBank;NA;JN274546
Lepidoptera;Nymphalidae;Melitaea;Melitaea diamina;Finland;GenBank;NA;JN274637
Lepidoptera;Nymphalidae;Melitaea;Melitaea diamina;Finland;GenBank;NA;JN274638
Lepidoptera;Lycaenidae;Cupido;Cupido osiris;Spain;GenBank;NA;JN276892
Lepidoptera;Nymphalidae;Minois;Minois dryas;Germany;GenBank;NA;JN278909
Lepidoptera;Lycaenidae;Plebejus;Plebejus argus;Poland;GenBank;NA;JN581045
Lepidoptera;Lycaenidae;Plebejus;Plebejus argus;Poland;GenBank;NA;JN581046
Lepidoptera;Lycaenidae;Plebejus;Plebejus argus;Poland;GenBank;NA;JN581047
Lepidoptera;Lycaenidae;Plebejus;Plebejus argus;Poland;GenBank;NA;JN581048
Lepidoptera;Lycaenidae;Plebejus;Plebejus argus;Poland;GenBank;NA;JN581049
Lepidoptera;Lycaenidae;Plebejus;Plebejus argus;Poland;GenBank;NA;JN581050
Lepidoptera;Lycaenidae;Plebejus;Plebejus argus;Poland;GenBank;NA;JN581051
Lepidoptera;Lycaenidae;Plebejus;Plebejus argus;Poland;GenBank;NA;JN581052
Lepidoptera;Lycaenidae;Plebejus;Plebejus argus;Poland;GenBank;NA;JN581053
Lepidoptera;Lycaenidae;Plebejus;Plebejus argus;Poland;GenBank;NA;JN581054
Lepidoptera;Lycaenidae;Plebejus;Plebejus argus;Poland;GenBank;NA;JN581055
Lepidoptera;Lycaenidae;Plebejus;Plebejus argus;Poland;GenBank;NA;JN581056
Lepidoptera;Lycaenidae;Plebejus;Plebejus argus;Poland;GenBank;NA;JN581057
Lepidoptera;Lycaenidae;Plebejus;Plebejus argus;Poland;GenBank;NA;JN581058
Lepidoptera;Lycaenidae;Plebejus;Plebejus argus;Poland;GenBank;NA;JN581059
Lepidoptera;Lycaenidae;Plebejus;Plebejus argus;Poland;GenBank;NA;JN581060
Lepidoptera;Lycaenidae;Aricia;Aricia artaxerxes;Russia;Bold;GBMIN16198-13;JX678097
Lepidoptera;Lycaenidae;Aricia;Aricia artaxerxes;Russia;Bold;GBMIN16336-13;JX678098
Lepidoptera;Lycaenidae;Lycaena;Lycaena helle;Germany;Bold;GBGLL181-13;KC208818
Lepidoptera;Lycaenidae;Lycaena;Lycaena helle;Germany;Bold;GBGLL180-13;KC208819
Lepidoptera;Lycaenidae;Lycaena;Lycaena helle;Germany;Bold;GBGLL179-13;KC208820
Lepidoptera;Lycaenidae;Lycaena;Lycaena helle;Germany;Bold;GBGLL178-13;KC208821
Lepidoptera;Lycaenidae;Lycaena;Lycaena helle;France;Bold;GBGLL177-13;KC208822
Lepidoptera;Lycaenidae;Lycaena;Lycaena helle;France;Bold;GBGLL176-13;KC208823
Lepidoptera;Lycaenidae;Lycaena;Lycaena helle;France;Bold;GBGLL175-13;KC208824
Lepidoptera;Lycaenidae;Lycaena;Lycaena helle;France;Bold;GBGLL174-13;KC208825
Lepidoptera;Lycaenidae;Lycaena;Lycaena helle;Germany;Bold;GBGLL173-13;KC208826
Lepidoptera;Lycaenidae;Lycaena;Lycaena helle;Germany;Bold;GBGLL172-13;KC208827
Lepidoptera;Lycaenidae;Lycaena;Lycaena helle;Germany;Bold;GBGLL171-13;KC208828
Lepidoptera;Lycaenidae;Lycaena;Lycaena helle;Germany;Bold;GBGLL170-13;KC208829
Lepidoptera;Lycaenidae;Lycaena;Lycaena helle;Germany;Bold;GBGLL169-13;KC208830
Lepidoptera;Lycaenidae;Lycaena;Lycaena helle;Poland;Bold;GBGLL168-13;KC208831
Lepidoptera;Lycaenidae;Lycaena;Lycaena helle;Poland;Bold;GBGLL167-13;KC208832
Lepidoptera;Lycaenidae;Lycaena;Lycaena helle;Poland;Bold;GBGLL166-13;KC208833
Lepidoptera;Lycaenidae;Lycaena;Lycaena helle;Poland;Bold;GBGLL165-13;KC208834
Lepidoptera;Lycaenidae;Lycaena;Lycaena helle;Germany;Bold;GBGLL164-13;KC208835
Lepidoptera;Lycaenidae;Lycaena;Lycaena helle;Germany;Bold;GBGLL163-13;KC208836
Lepidoptera;Lycaenidae;Lycaena;Lycaena helle;Germany;Bold;GBGLL162-13;KC208837
Lepidoptera;Lycaenidae;Lycaena;Lycaena helle;France;Bold;GBGLL161-13;KC208838
Lepidoptera;Lycaenidae;Lycaena;Lycaena helle;France;Bold;GBGLL160-13;KC208839
Lepidoptera;Lycaenidae;Lycaena;Lycaena helle;France;Bold;GBGLL159-13;KC208840
Lepidoptera;Lycaenidae;Lycaena;Lycaena helle;France;Bold;GBGLL158-13;KC208841
Lepidoptera;Lycaenidae;Lycaena;Lycaena helle;Sweden;Bold;GBGLL157-13;KC208842
Lepidoptera;Lycaenidae;Lycaena;Lycaena helle;Sweden;Bold;GBGLL156-13;KC208843

Lepidoptera;Lycaenidae;Lycaena;Lycaena helle;Sweden;Bold;GBGLL155-13;KC208844
Lepidoptera;Lycaenidae;Lycaena;Lycaena helle;Sweden;Bold;GBGLL154-13;KC208845
Lepidoptera;Lycaenidae;Lycaena;Lycaena helle;Sweden;Bold;GBGLL153-13;KC208846
Lepidoptera;Lycaenidae;Lycaena;Lycaena helle;Poland;Bold;GBGLL152-13;KC208847
Lepidoptera;Lycaenidae;Lycaena;Lycaena helle;Poland;Bold;GBGLL151-13;KC208848
Lepidoptera;Lycaenidae;Lycaena;Lycaena helle;Poland;Bold;GBGLL150-13;KC208849
Lepidoptera;Nymphalidae;Melitaea;Melitaea cinxia;Finland;GenBank;NA;KC465909
Lepidoptera;Nymphalidae;Melitaea;Melitaea cinxia;Sweden;GenBank;NA;KC465910
Lepidoptera;Nymphalidae;Melitaea;Melitaea cinxia;Estonia;GenBank;NA;KC465911
Lepidoptera;Nymphalidae;Melitaea;Melitaea cinxia;Sweden;GenBank;NA;KC465912
Lepidoptera;Nymphalidae;Melitaea;Melitaea cinxia;Finland;GenBank;NA;KC465913
Lepidoptera;Nymphalidae;Melitaea;Melitaea cinxia;Estonia;GenBank;NA;KC465914
Lepidoptera;Nymphalidae;Melitaea;Melitaea cinxia;Sweden;GenBank;NA;KC465915
Lepidoptera;Nymphalidae;Melitaea;Melitaea cinxia;Finland;GenBank;NA;KC465916
Lepidoptera;Nymphalidae;Melitaea;Melitaea cinxia;Sweden;GenBank;NA;KC465917
Lepidoptera;Pieridae;Leptidea;Leptidea juvernica;Germany;Bold;GBGLP260-13;KC865973
Lepidoptera;Pieridae;Leptidea;Leptidea sinapis;Luxembourg;Bold;GBGLP211-13;KC866022
Lepidoptera;Pieridae;Leptidea;Leptidea sinapis;Croatia;Bold;GBGLP208-13;KC866025
Lepidoptera;Pieridae;Leptidea;Leptidea sinapis;Croatia;Bold;GBGLP207-13;KC866026
Lepidoptera;Pieridae;Leptidea;Leptidea sinapis;Finland;Bold;GBGLP105-13;KC866128
Lepidoptera;Pieridae;Leptidea;Leptidea juvernica;Germany;Bold;GBGLP103-13;KC866130
Lepidoptera;Nymphalidae;Vanessa;Vanessa atalanta;Poland;GenBank;NA;KJ649004
Lepidoptera;Nymphalidae;Maniola;Maniola jurtina;Greece;Bold;GBLN5038-14;KM033871
Lepidoptera;Nymphalidae;Erebia;Erebia euryale;Slovakia;GenBank;NA;KM196507
Lepidoptera;Nymphalidae;Euphydryas;Euphydryas aurinia;Finland;GenBank;NA;KM572689
Lepidoptera;Nymphalidae;Maniola;Maniola jurtina;France;GenBank;NA;KP032275
Lepidoptera;Nymphalidae;Maniola;Maniola jurtina;France;GenBank;NA;KP032276
Lepidoptera;Nymphalidae;Maniola;Maniola jurtina;France;GenBank;NA;KP032277
Lepidoptera;Nymphalidae;Maniola;Maniola jurtina;France;GenBank;NA;KP032278
Lepidoptera;Nymphalidae;Maniola;Maniola jurtina;France;GenBank;NA;KP032279
Lepidoptera;Nymphalidae;Maniola;Maniola jurtina;Albania;GenBank;NA;KP032289
Lepidoptera;Nymphalidae;Maniola;Maniola jurtina;Austria;GenBank;NA;KP032295
Lepidoptera;Nymphalidae;Maniola;Maniola jurtina;Portugal;GenBank;NA;KP032297
Lepidoptera;Nymphalidae;Maniola;Maniola jurtina;Spain;GenBank;NA;KP032317
Lepidoptera;Nymphalidae;Maniola;Maniola jurtina;France;GenBank;NA;KP032318
Lepidoptera;Nymphalidae;Pyronia;Pyronia cecilia;Italy;Bold;GBLN5432-15;KP032346
Lepidoptera;Nymphalidae;Pyronia;Pyronia cecilia;Italy;Bold;GBLN5431-15;KP032347
Lepidoptera;Nymphalidae;Maniola;Maniola jurtina;Spain;GenBank;NA;KP032364
Lepidoptera;Lycaenidae;Lysandra;Lysandra coridon;Germany;GenBank;NA;KR007013
Lepidoptera;Lycaenidae;Lysandra;Lysandra coridon;Germany;GenBank;NA;KR007014
Lepidoptera;Lycaenidae;Lysandra;Lysandra coridon;Germany;GenBank;NA;KR007015
Lepidoptera;Lycaenidae;Lysandra;Lysandra coridon;Germany;GenBank;NA;KR007016
Lepidoptera;Lycaenidae;Lysandra;Lysandra coridon;Germany;GenBank;NA;KR007017
Lepidoptera;Lycaenidae;Lysandra;Lysandra coridon;Germany;GenBank;NA;KR007018
Lepidoptera;Lycaenidae;Lysandra;Lysandra coridon;Germany;GenBank;NA;KR007019
Lepidoptera;Lycaenidae;Lysandra;Lysandra coridon;Germany;GenBank;NA;KR007020
Lepidoptera;Lycaenidae;Lysandra;Lysandra coridon;Germany;GenBank;NA;KR007021
Lepidoptera;Lycaenidae;Lysandra;Lysandra coridon;Germany;GenBank;NA;KR007022
Lepidoptera;Lycaenidae;Lysandra;Lysandra coridon;Germany;GenBank;NA;KR007023
Lepidoptera;Lycaenidae;Lysandra;Lysandra coridon;Germany;GenBank;NA;KR007024
Lepidoptera;Lycaenidae;Lysandra;Lysandra coridon;Germany;GenBank;NA;KR007025
Lepidoptera;Lycaenidae;Lysandra;Lysandra coridon;Germany;GenBank;NA;KR007026
Lepidoptera;Lycaenidae;Lysandra;Lysandra coridon;Germany;GenBank;NA;KR007027
Lepidoptera;Lycaenidae;Lysandra;Lysandra coridon;Germany;GenBank;NA;KR007038
Lepidoptera;Lycaenidae;Lysandra;Lysandra coridon;Germany;GenBank;NA;KR007039
Lepidoptera;Lycaenidae;Lysandra;Lysandra coridon;Germany;GenBank;NA;KR007040
Lepidoptera;Lycaenidae;Lysandra;Lysandra coridon;Germany;GenBank;NA;KR007041
Lepidoptera;Lycaenidae;Lysandra;Lysandra coridon;France;GenBank;NA;KR007056
Lepidoptera;Lycaenidae;Lysandra;Lysandra coridon;France;GenBank;NA;KR007057
Lepidoptera;Lycaenidae;Lysandra;Lysandra coridon;France;GenBank;NA;KR007058
Lepidoptera;Lycaenidae;Lysandra;Lysandra coridon;France;GenBank;NA;KR007059
Lepidoptera;Lycaenidae;Lysandra;Lysandra coridon;France;GenBank;NA;KR007060
Lepidoptera;Lycaenidae;Lysandra;Lysandra coridon;France;GenBank;NA;KR007066
Lepidoptera;Lycaenidae;Lysandra;Lysandra coridon;France;GenBank;NA;KR007067
Lepidoptera;Lycaenidae;Lysandra;Lysandra coridon;France;GenBank;NA;KR007068
Lepidoptera;Lycaenidae;Lysandra;Lysandra coridon;France;GenBank;NA;KR007070
Lepidoptera;Lycaenidae;Lysandra;Lysandra coridon;Italy;GenBank;NA;KR007072
Lepidoptera;Lycaenidae;Lysandra;Lysandra coridon;Italy;GenBank;NA;KR007073
Lepidoptera;Lycaenidae;Lysandra;Lysandra coridon;Italy;GenBank;NA;KR007074
Lepidoptera;Lycaenidae;Lysandra;Lysandra coridon;Italy;GenBank;NA;KR007075
Lepidoptera;Lycaenidae;Lysandra;Lysandra coridon;Switzerland;GenBank;NA;KR007076
Lepidoptera;Lycaenidae;Lysandra;Lysandra coridon;Switzerland;GenBank;NA;KR007077
Lepidoptera;Lycaenidae;Lysandra;Lysandra coridon;Switzerland;GenBank;NA;KR007078
Lepidoptera;Lycaenidae;Lysandra;Lysandra coridon;Switzerland;GenBank;NA;KR007079
Lepidoptera;Lycaenidae;Lysandra;Lysandra coridon;Switzerland;GenBank;NA;KR007080
Lepidoptera;Nymphalidae;Hyponephele;Hyponephele lycaon;Spain;Bold;GBGL37335-19;KT448686
Lepidoptera;Lycaenidae;Scolitantides;Scolitantides orion;Finland;GenBank;NA;KT782365
Lepidoptera;Nymphalidae;Hipparchia;Hipparchia hermione;Latvia;GenBank;NA;KT782440

Lepidoptera;Lycaenidae;Polyommatus;Polyommatus damon;Latvia;GenBank;NA;KT782578
Lepidoptera;Nymphalidae;Euphydryas;Euphydryas intermedia;Russia;GenBank;NA;KT989872
Lepidoptera;Nymphalidae;Erebia;Erebia pronoe;Slovakia;GenBank;NA;KX277936
Lepidoptera;Nymphalidae;Erebia;Erebia pronoe;Slovakia;GenBank;NA;KX277937
Lepidoptera;Nymphalidae;Erebia;Erebia pronoe;Slovakia;GenBank;NA;KX277938
Lepidoptera;Nymphalidae;Erebia;Erebia pronoe;Slovakia;GenBank;NA;KX277939
Lepidoptera;Nymphalidae;Erebia;Erebia pronoe;Slovakia;GenBank;NA;KX277940
Lepidoptera;Nymphalidae;Erebia;Erebia pronoe;Slovakia;GenBank;NA;KX277941
Lepidoptera;Nymphalidae;Erebia;Erebia pronoe;Slovakia;GenBank;NA;KX277942
Lepidoptera;Nymphalidae;Erebia;Erebia pronoe;Slovakia;GenBank;NA;KX277943
Lepidoptera;Nymphalidae;Erebia;Erebia pronoe;Slovakia;GenBank;NA;KX277944
Lepidoptera;Nymphalidae;Erebia;Erebia pronoe;Slovakia;GenBank;NA;KX277945
Lepidoptera;Nymphalidae;Erebia;Erebia pronoe;Slovakia;GenBank;NA;KX277946
Lepidoptera;Nymphalidae;Erebia;Erebia pronoe;Slovakia;GenBank;NA;KX277947
Lepidoptera;Nymphalidae;Erebia;Erebia pronoe;Slovakia;GenBank;NA;KX277948
Lepidoptera;Nymphalidae;Erebia;Erebia pronoe;Italy;GenBank;NA;KX277949
Lepidoptera;Nymphalidae;Erebia;Erebia pronoe;Italy;GenBank;NA;KX277950
Lepidoptera;Nymphalidae;Erebia;Erebia pronoe;Italy;GenBank;NA;KX277951
Lepidoptera;Nymphalidae;Erebia;Erebia pronoe;Italy;GenBank;NA;KX277952
Lepidoptera;Nymphalidae;Erebia;Erebia pronoe;Italy;GenBank;NA;KX277953
Lepidoptera;Nymphalidae;Erebia;Erebia pronoe;Italy;GenBank;NA;KX277954
Lepidoptera;Nymphalidae;Erebia;Erebia pronoe;Italy;GenBank;NA;KX277955
Lepidoptera;Nymphalidae;Erebia;Erebia pronoe;Spain;GenBank;NA;KX277956
Lepidoptera;Nymphalidae;Erebia;Erebia pronoe;Spain;GenBank;NA;KX277957
Lepidoptera;Nymphalidae;Erebia;Erebia pronoe;Spain;GenBank;NA;KX277958
Lepidoptera;Nymphalidae;Erebia;Erebia pronoe;Spain;GenBank;NA;KX277959
Lepidoptera;Nymphalidae;Erebia;Erebia pronoe;Spain;GenBank;NA;KX277960
Lepidoptera;Nymphalidae;Erebia;Erebia pronoe;Spain;GenBank;NA;KX277961
Lepidoptera;Nymphalidae;Erebia;Erebia pronoe;Spain;GenBank;NA;KX277962
Lepidoptera;Nymphalidae;Aglais;Aglais urticae;Poland;Bold;GBGL31771-19;KY128341
Lepidoptera;Nymphalidae;Erebia;Erebia aethiopellus;Italy;Bold;GBGL36886-19;LC340553
Lepidoptera;Nymphalidae;Erebia;Erebia aethiopellus;Italy;Bold;GBGL36887-19;LC340562
Lepidoptera;Nymphalidae;Erebia;Erebia aethiopellus;Italy;Bold;GBGL36888-19;LC340683
Lepidoptera;Nymphalidae;Erebia;Erebia aethiopellus;Italy;Bold;GBGL36889-19;LC340692
Lepidoptera;Nymphalidae;Aglais;Aglais urticae;Sweden;Bold;GBMNC57277-20;LC471701
Lepidoptera;Lycaenidae;Aricia;Aricia agestis;United Kingdom;Bold;SPMIS061-22;LR990279.1
Lepidoptera;Lycaenidae;Plebejus;Plebejus argus;Serbia;GenBank;NA;LT219847
Lepidoptera;Lycaenidae;Plebejus;Plebejus argus;Russia;GenBank;NA;LT219904
Lepidoptera;Lycaenidae;Plebejus;Plebejus argus;Russia;GenBank;NA;LT219905
Lepidoptera;Lycaenidae;Polyommatus;Polyommatus amandus;Russia;GenBank;NA;LT628553
Lepidoptera;Lycaenidae;Polyommatus;Polyommatus amandus;Russia;GenBank;NA;LT628556
Lepidoptera;Lycaenidae;Aricia;Aricia agestis;Greece;GenBank;NA;LT628804
Lepidoptera;Lycaenidae;Aricia;Aricia agestis;Greece;GenBank;NA;LT628805
Lepidoptera;Lycaenidae;Polyommatus;Polyommatus dorylas;Romania;GenBank;NA;LT628881
Lepidoptera;Lycaenidae;Phengaris;Phengaris arion;Russia;GenBank;NA;LT628891
Lepidoptera;Lycaenidae;Phengaris;Phengaris arion;Russia;GenBank;NA;LT628892
Lepidoptera;Lycaenidae;Phengaris;Phengaris arion;Russia;GenBank;NA;LT628893
Lepidoptera;Lycaenidae;Phengaris;Phengaris arion;Russia;GenBank;NA;LT628894
Lepidoptera;Lycaenidae;Phengaris;Phengaris arion;Russia;GenBank;NA;LT628895
Lepidoptera;Lycaenidae;Phengaris;Phengaris arion;Russia;GenBank;NA;LT628896
Lepidoptera;Lycaenidae;Aricia;Aricia agestis;United Kingdom;GenBank;NA;LT628915
Lepidoptera;Lycaenidae;Aricia;Aricia agestis;United Kingdom;GenBank;NA;LT628916
Lepidoptera;Lycaenidae;Polyommatus;Polyommatus amandus;Sweden;GenBank;NA;LT628930
Lepidoptera;Lycaenidae;Phengaris;Phengaris alcon;Denmark;GenBank;NA;LT628931
Lepidoptera;Lycaenidae;Phengaris;Phengaris alcon;Denmark;GenBank;NA;LT628932
Lepidoptera;Lycaenidae;Phengaris;Phengaris alcon;Denmark;GenBank;NA;LT628933
Lepidoptera;Lycaenidae;Phengaris;Phengaris alcon;Denmark;GenBank;NA;LT628934
Lepidoptera;Lycaenidae;Phengaris;Phengaris alcon;Denmark;GenBank;NA;LT628935
Lepidoptera;Lycaenidae;Phengaris;Phengaris alcon;Denmark;GenBank;NA;LT628936
Lepidoptera;Lycaenidae;Phengaris;Phengaris alcon;Denmark;GenBank;NA;LT628937
Lepidoptera;Lycaenidae;Phengaris;Phengaris alcon;Denmark;GenBank;NA;LT628938
Lepidoptera;Lycaenidae;Phengaris;Phengaris alcon;Denmark;GenBank;NA;LT628939
Lepidoptera;Lycaenidae;Phengaris;Phengaris alcon;Denmark;GenBank;NA;LT628940
Lepidoptera;Lycaenidae;Cyaniris;Cyaniris semiargus;Sweden;GenBank;NA;LT628941
Lepidoptera;Lycaenidae;Phengaris;Phengaris alcon;Denmark;GenBank;NA;LT628942
Lepidoptera;Lycaenidae;Phengaris;Phengaris alcon;Denmark;GenBank;NA;LT628943
Lepidoptera;Lycaenidae;Phengaris;Phengaris alcon;Denmark;GenBank;NA;LT628944
Lepidoptera;Lycaenidae;Phengaris;Phengaris alcon;Denmark;GenBank;NA;LT628945
Lepidoptera;Lycaenidae;Phengaris;Phengaris alcon;Denmark;GenBank;NA;LT628946
Lepidoptera;Lycaenidae;Phengaris;Phengaris alcon;Denmark;GenBank;NA;LT628947
Lepidoptera;Lycaenidae;Phengaris;Phengaris alcon;Denmark;GenBank;NA;LT628948
Lepidoptera;Lycaenidae;Phengaris;Phengaris alcon;Denmark;GenBank;NA;LT628949
Lepidoptera;Lycaenidae;Phengaris;Phengaris alcon;Denmark;GenBank;NA;LT628950
Lepidoptera;Lycaenidae;Phengaris;Phengaris alcon;Denmark;GenBank;NA;LT628951
Lepidoptera;Lycaenidae;Phengaris;Phengaris alcon;Denmark;GenBank;NA;LT628952
Lepidoptera;Lycaenidae;Phengaris;Phengaris alcon;Denmark;GenBank;NA;LT628953
Lepidoptera;Lycaenidae;Phengaris;Phengaris alcon;Denmark;GenBank;NA;LT628954
Lepidoptera;Lycaenidae;Polyommatus;Polyommatus icarus;Denmark;GenBank;NA;LT628955

Lepidoptera;Lycaenidae;Polyommatus;Polyommatus amandus;Sweden;GenBank;NA;LT628956
Lepidoptera;Lycaenidae;Phengaris;Phengaris alcon;Denmark;GenBank;NA;LT628957
Lepidoptera;Lycaenidae;Phengaris;Phengaris alcon;Denmark;GenBank;NA;LT628958
Lepidoptera;Lycaenidae;Phengaris;Phengaris alcon;Denmark;GenBank;NA;LT628959
Lepidoptera;Lycaenidae;Phengaris;Phengaris alcon;Denmark;GenBank;NA;LT628960
Lepidoptera;Lycaenidae;Phengaris;Phengaris alcon;Denmark;GenBank;NA;LT628961
Lepidoptera;Lycaenidae;Polyommatus;Polyommatus icarus;Denmark;GenBank;NA;LT628963
Lepidoptera;Lycaenidae;Phengaris;Phengaris alcon;Denmark;GenBank;NA;LT628964
Lepidoptera;Lycaenidae;Phengaris;Phengaris alcon;Denmark;GenBank;NA;LT628965
Lepidoptera;Lycaenidae;Phengaris;Phengaris alcon;Denmark;GenBank;NA;LT628966
Lepidoptera;Lycaenidae;Phengaris;Phengaris alcon;Denmark;GenBank;NA;LT628967
Lepidoptera;Lycaenidae;Phengaris;Phengaris alcon;Denmark;GenBank;NA;LT628968
Lepidoptera;Lycaenidae;Phengaris;Phengaris alcon;Denmark;GenBank;NA;LT628969
Lepidoptera;Lycaenidae;Phengaris;Phengaris alcon;Denmark;GenBank;NA;LT628970
Lepidoptera;Lycaenidae;Phengaris;Phengaris alcon;Denmark;GenBank;NA;LT628971
Lepidoptera;Lycaenidae;Phengaris;Phengaris alcon;Denmark;GenBank;NA;LT628972
Lepidoptera;Lycaenidae;Phengaris;Phengaris arion;Sweden;GenBank;NA;LT628973
Lepidoptera;Lycaenidae;Phengaris;Phengaris alcon;Denmark;GenBank;NA;LT628974
Lepidoptera;Lycaenidae;Polyommatus;Polyommatus amandus;Bosnia and Herzegovina;GenBank;NA;LT628980
Lepidoptera;Lycaenidae;Phengaris;Phengaris alcon;Serbia;GenBank;NA;LT628981
Lepidoptera;Lycaenidae;Cyaniris;Cyaniris semiargus;Serbia;GenBank;NA;LT628982
Lepidoptera;Lycaenidae;Phengaris;Phengaris arion;Serbia;GenBank;NA;LT628983
Lepidoptera;Lycaenidae;Phengaris;Phengaris arion;Serbia;GenBank;NA;LT628984
Lepidoptera;Lycaenidae;Plebejus;Plebejus argyrognomon;Serbia;GenBank;NA;LT628985
Lepidoptera;Lycaenidae;Phengaris;Phengaris arion;Serbia;GenBank;NA;LT628988
Lepidoptera;Lycaenidae;Phengaris;Phengaris arion;Serbia;GenBank;NA;LT628989
Lepidoptera;Lycaenidae;Phengaris;Phengaris arion;Serbia;GenBank;NA;LT628990
Lepidoptera;Lycaenidae;Lycaena;Lycaena alciphron;Montenegro;GenBank;NA;LT628991
Lepidoptera;Lycaenidae;Phengaris;Phengaris arion;Serbia;GenBank;NA;LT628992
Lepidoptera;Lycaenidae;Polyommatus;Polyommatus daphnis;Montenegro;GenBank;NA;LT628993
Lepidoptera;Lycaenidae;Phengaris;Phengaris alcon;Serbia;GenBank;NA;LT628994
Lepidoptera;Lycaenidae;Phengaris;Phengaris alcon;Serbia;GenBank;NA;LT628998
Lepidoptera;Lycaenidae;Plebejus;Plebejus argus;Serbia;GenBank;NA;LT628999
Lepidoptera;Lycaenidae;Polyommatus;Polyommatus icarus;France;GenBank;NA;LT629001
Lepidoptera;Lycaenidae;Polyommatus;Polyommatus icarus;Turkey;GenBank;NA;LT629002
Lepidoptera;Lycaenidae;Aricia;Aricia agestis;Turkey;GenBank;NA;LT629003
Lepidoptera;Lycaenidae;Polyommatus;Polyommatus icarus;Turkey;GenBank;NA;LT629004
Lepidoptera;Lycaenidae;Aricia;Aricia agestis;Belgium;GenBank;NA;LT629005
Lepidoptera;Lycaenidae;Polyommatus;Polyommatus icarus;Finland;GenBank;NA;LT629006
Lepidoptera;Lycaenidae;Polyommatus;Polyommatus icarus;Romania;GenBank;NA;LT629007
Lepidoptera;Lycaenidae;Polyommatus;Polyommatus icarus;Spain;GenBank;NA;LT629008
Lepidoptera;Lycaenidae;Polyommatus;Polyommatus icarus;Spain;GenBank;NA;LT629009
Lepidoptera;Lycaenidae;Polyommatus;Polyommatus icarus;Spain;GenBank;NA;LT629010
Lepidoptera;Lycaenidae;Polyommatus;Polyommatus icarus;Turkey;GenBank;NA;LT629011
Lepidoptera;Lycaenidae;Polyommatus;Polyommatus icarus;Turkey;GenBank;NA;LT629012
Lepidoptera;Lycaenidae;Polyommatus;Polyommatus icarus;Turkey;GenBank;NA;LT629013
Lepidoptera;Lycaenidae;Aricia;Aricia agestis;Serbia;GenBank;NA;LT629016
Lepidoptera;Lycaenidae;Phengaris;Phengaris alcon;Spain;GenBank;NA;LT629017
Lepidoptera;Lycaenidae;Phengaris;Phengaris alcon;Spain;GenBank;NA;LT629018
Lepidoptera;Lycaenidae;Phengaris;Phengaris alcon;Spain;GenBank;NA;LT629019
Lepidoptera;Lycaenidae;Phengaris;Phengaris alcon;Spain;GenBank;NA;LT629020
Lepidoptera;Lycaenidae;Phengaris;Phengaris alcon;Spain;GenBank;NA;LT629021
Lepidoptera;Lycaenidae;Polyommatus;Polyommatus icarus;Spain;GenBank;NA;LT629022
Lepidoptera;Lycaenidae;Polyommatus;Polyommatus icarus;Denmark;GenBank;NA;LT629023
Lepidoptera;Lycaenidae;Polyommatus;Polyommatus icarus;Romania;GenBank;NA;LT629024
Lepidoptera;Lycaenidae;Aricia;Aricia agestis;Romania;GenBank;NA;LT629025
Lepidoptera;Lycaenidae;Polyommatus;Polyommatus icarus;Romania;GenBank;NA;LT629026
Lepidoptera;Lycaenidae;Polyommatus;Polyommatus icarus;Romania;GenBank;NA;LT629027
Lepidoptera;Lycaenidae;Aricia;Aricia agestis;Romania;GenBank;NA;LT629028
Lepidoptera;Lycaenidae;Polyommatus;Polyommatus icarus;Romania;GenBank;NA;LT629029
Lepidoptera;Lycaenidae;Polyommatus;Polyommatus icarus;Bulgaria;GenBank;NA;LT629030
Lepidoptera;Lycaenidae;Phengaris;Phengaris arion;Romania;GenBank;NA;LT629031
Lepidoptera;Lycaenidae;Phengaris;Phengaris arion;Romania;GenBank;NA;LT629032
Lepidoptera;Lycaenidae;Phengaris;Phengaris alcon;Spain;GenBank;NA;LT629033
Lepidoptera;Lycaenidae;Phengaris;Phengaris alcon;Spain;GenBank;NA;LT629034
Lepidoptera;Lycaenidae;Polyommatus;Polyommatus icarus;Spain;GenBank;NA;LT629035
Lepidoptera;Lycaenidae;Polyommatus;Polyommatus icarus;Spain;GenBank;NA;LT629036
Lepidoptera;Lycaenidae;Polyommatus;Polyommatus icarus;Spain;GenBank;NA;LT629037
Lepidoptera;Lycaenidae;Polyommatus;Polyommatus icarus;Spain;GenBank;NA;LT629038
Lepidoptera;Lycaenidae;Polyommatus;Polyommatus icarus;Spain;GenBank;NA;LT629039
Lepidoptera;Lycaenidae;Plebejus;Plebejus argus;Spain;GenBank;NA;LT629040
Lepidoptera;Lycaenidae;Polyommatus;Polyommatus icarus;Spain;GenBank;NA;LT629041
Lepidoptera;Lycaenidae;Polyommatus;Polyommatus icarus;Greece;GenBank;NA;LT629042
Lepidoptera;Lycaenidae;Polyommatus;Polyommatus icarus;Greece;GenBank;NA;LT629043
Lepidoptera;Nymphalidae;Boloria;Boloria selene;Russia;GenBank;NA;LT795285
Lepidoptera;Nymphalidae;Boloria;Boloria selene;Russia;GenBank;NA;LT795291
Lepidoptera;Nymphalidae;Boloria;Boloria selene;Belarus;GenBank;NA;LT795292

Lepidoptera;Nymphalidae;Boloria;Boloria selene;Belarus;GenBank;NA;LT795293
Lepidoptera;Nymphalidae;Boloria;Boloria selene;Russia;GenBank;NA;LT795294
Lepidoptera;Nymphalidae;Boloria;Boloria selene;Sweden;GenBank;NA;LT795296
Lepidoptera;Nymphalidae;Boloria;Boloria selene;Norway;GenBank;NA;LT795297
Lepidoptera;Nymphalidae;Boloria;Boloria selene;Poland;GenBank;NA;LT795298
Lepidoptera;Nymphalidae;Boloria;Boloria selene;Czech Republic;GenBank;NA;LT795299
Lepidoptera;Nymphalidae;Boloria;Boloria selene;Czech Republic;GenBank;NA;LT795300
Lepidoptera;Nymphalidae;Boloria;Boloria selene;Czech Republic;GenBank;NA;LT795301
Lepidoptera;Nymphalidae;Boloria;Boloria selene;Czech Republic;GenBank;NA;LT795302
Lepidoptera;Nymphalidae;Boloria;Boloria selene;Czech Republic;GenBank;NA;LT795303
Lepidoptera;Nymphalidae;Boloria;Boloria selene;Czech Republic;GenBank;NA;LT795304
Lepidoptera;Nymphalidae;Boloria;Boloria selene;Czech Republic;GenBank;NA;LT795305
Lepidoptera;Nymphalidae;Boloria;Boloria selene;Russia;GenBank;NA;LT795306
Lepidoptera;Nymphalidae;Boloria;Boloria selene;Russia;GenBank;NA;LT795307
Lepidoptera;Nymphalidae;Boloria;Boloria selene;Russia;GenBank;NA;LT795308
Lepidoptera;Nymphalidae;Boloria;Boloria selene;Czech Republic;GenBank;NA;LT795309
Lepidoptera;Nymphalidae;Boloria;Boloria selene;Czech Republic;GenBank;NA;LT795310
Lepidoptera;Nymphalidae;Boloria;Boloria selene;Czech Republic;GenBank;NA;LT795311
Lepidoptera;Nymphalidae;Boloria;Boloria selene;Czech Republic;GenBank;NA;LT795312
Lepidoptera;Nymphalidae;Boloria;Boloria selene;Czech Republic;GenBank;NA;LT795313
Lepidoptera;Nymphalidae;Boloria;Boloria selene;Romania;GenBank;NA;LT795314
Lepidoptera;Nymphalidae;Boloria;Boloria selene;Romania;GenBank;NA;LT795315
Lepidoptera;Nymphalidae;Boloria;Boloria selene;Denmark;GenBank;NA;LT795316
Lepidoptera;Nymphalidae;Boloria;Boloria selene;Denmark;GenBank;NA;LT795317
Lepidoptera;Nymphalidae;Boloria;Boloria selene;Denmark;GenBank;NA;LT795318
Lepidoptera;Nymphalidae;Boloria;Boloria selene;Denmark;GenBank;NA;LT795319
Lepidoptera;Nymphalidae;Boloria;Boloria selene;Denmark;GenBank;NA;LT795320
Lepidoptera;Nymphalidae;Boloria;Boloria selene;Denmark;GenBank;NA;LT795321
Lepidoptera;Nymphalidae;Boloria;Boloria selene;Denmark;GenBank;NA;LT795322
Lepidoptera;Nymphalidae;Boloria;Boloria selene;Russia;GenBank;NA;LT795323
Lepidoptera;Nymphalidae;Boloria;Boloria selene;Russia;GenBank;NA;LT795324
Lepidoptera;Nymphalidae;Boloria;Boloria selene;Russia;GenBank;NA;LT795325
Lepidoptera;Nymphalidae;Boloria;Boloria selene;Russia;GenBank;NA;LT795326
Lepidoptera;Nymphalidae;Boloria;Boloria selene;Spain;GenBank;NA;LT795327
Lepidoptera;Nymphalidae;Boloria;Boloria selene;Spain;GenBank;NA;LT795328
Lepidoptera;Nymphalidae;Boloria;Boloria selene;Czech Republic;GenBank;NA;LT795329
Lepidoptera;Nymphalidae;Boloria;Boloria selene;Czech Republic;GenBank;NA;LT795330
Lepidoptera;Nymphalidae;Boloria;Boloria selene;Czech Republic;GenBank;NA;LT795331
Lepidoptera;Nymphalidae;Boloria;Boloria selene;Czech Republic;GenBank;NA;LT795332
Lepidoptera;Nymphalidae;Boloria;Boloria selene;Czech Republic;GenBank;NA;LT795333
Lepidoptera;Nymphalidae;Boloria;Boloria selene;Czech Republic;GenBank;NA;LT795334
Lepidoptera;Nymphalidae;Boloria;Boloria selene;Czech Republic;GenBank;NA;LT795335
Lepidoptera;Nymphalidae;Boloria;Boloria selene;Czech Republic;GenBank;NA;LT795336
Lepidoptera;Nymphalidae;Boloria;Boloria selene;Czech Republic;GenBank;NA;LT795337
Lepidoptera;Nymphalidae;Boloria;Boloria selene;Czech Republic;GenBank;NA;LT795338
Lepidoptera;Nymphalidae;Euphydryas;Euphydryas aurinia;Portugal;GenBank;NA;MF458706
Lepidoptera;Lycaenidae;Scolitantides;Scolitantides orion;Albania;GenBank;NA;MH407225
Lepidoptera;Pieridae;Pieris;Pieris rapae;Italy;GenBank;NA;MH418624
Lepidoptera;Pieridae;Pieris;Pieris rapae;Italy;GenBank;NA;MH418752
Lepidoptera;Lycaenidae;Celastrina;Celastrina argiolus;Italy;GenBank;NA;MH419164
Lepidoptera;Nymphalidae;Hipparchia;Hipparchia fagi;Italy;GenBank;NA;MN140412
Lepidoptera;Lycaenidae;Polyommatus;Polyommatus celina;Spain;GenBank;NA;MN196663
Lepidoptera;Lycaenidae;Polyommatus;Polyommatus icarus;Spain;GenBank;NA;MN196664
Lepidoptera;Nymphalidae;Neptis;Neptis rivularis;Poland;GenBank;NA;MT283600
Lepidoptera;Nymphalidae;Hipparchia;Hipparchia hermione;Spain;GenBank;NA;MT542035
Lepidoptera;Lycaenidae;Scolitantides;Scolitantides orion;Poland;GenBank;NA;MT820115
Lepidoptera;Lycaenidae;Scolitantides;Scolitantides orion;Poland;GenBank;NA;MT820116
Lepidoptera;Lycaenidae;Aricia;Aricia artaxerxes;Montenegro;GenBank;NA;MT883946
Lepidoptera;Lycaenidae;Aricia;Aricia artaxerxes;Sweden;GenBank;NA;MT883959
Lepidoptera;Nymphalidae;Coenonympha;Coenonympha oedippus;Slovenia;GenBank;NA;MW303898
Lepidoptera;Nymphalidae;Coenonympha;Coenonympha oedippus;Slovenia;GenBank;NA;MW303899
Lepidoptera;Nymphalidae;Coenonympha;Coenonympha oedippus;Slovenia;GenBank;NA;MW303900
Lepidoptera;Nymphalidae;Coenonympha;Coenonympha oedippus;Slovenia;GenBank;NA;MW303901
Lepidoptera;Nymphalidae;Coenonympha;Coenonympha oedippus;Slovenia;GenBank;NA;MW303902
Lepidoptera;Nymphalidae;Coenonympha;Coenonympha oedippus;Slovenia;GenBank;NA;MW303903
Lepidoptera;Lycaenidae;Lycaena;Lycaena phlaeas;Norway;Bold;GBMND21139-21;MW431042
Lepidoptera;Pieridae;Anthocharis;Anthocharis cardamines;Turkey;Bold;GBMND23406-21;MW431044
Lepidoptera;Nymphalidae;Erebia;Erebia medusa;Norway;Bold;GBMND22110-21;MW431052
Lepidoptera;Lycaenidae;Hypaurotis;Hypaurotis quercus;Hungary;GenBank;NA;MW441622
Lepidoptera;Lycaenidae;Hypaurotis;Hypaurotis quercus;Hungary;GenBank;NA;MW441624
Lepidoptera;Nymphalidae;Hyponephele;Hyponephele lycaon;Poland;Bold;EULEP4866-16;MW501381
Lepidoptera;Nymphalidae;Aglais;Aglais urticae;Latvia;Bold;EULEP313-14;MW501843
Lepidoptera;Nymphalidae;Hyponephele;Hyponephele lycaon;France;Bold;EULEP4091-16;MW502368
Lepidoptera;Pieridae;Leptidea;Leptidea reali;Spain;GenBank;NA;MW750512
Lepidoptera;Pieridae;Leptidea;Leptidea sinapis;Russia;GenBank;NA;MW750513
Lepidoptera;Pieridae;Leptidea;Leptidea sinapis;Russia;GenBank;NA;MW750693
Lepidoptera;Pieridae;Leptidea;Leptidea sinapis;Russia;GenBank;NA;MW750694
Lepidoptera;Pieridae;Pieris;Pieris napi;Sweden;Bold;GBMND76647-21;MW768130

Lepidoptera;Lycaenidae;Aricia;Aricia eumedon;Italy;GenBank;NA;MW861639
Lepidoptera;Lycaenidae;Aricia;Aricia eumedon;Serbia;GenBank;NA;MW861640
Lepidoptera;Lycaenidae;Aricia;Aricia eumedon;Greece;GenBank;NA;MW861641
Lepidoptera;Lycaenidae;Aricia;Aricia eumedon;France;GenBank;NA;MW861642
Lepidoptera;Lycaenidae;Aricia;Aricia eumedon;Ukraine;GenBank;NA;MW861643
Lepidoptera;Lycaenidae;Aricia;Aricia eumedon;Spain;GenBank;NA;MW861645
Lepidoptera;Lycaenidae;Aricia;Aricia eumedon;Italy;GenBank;NA;MW861647
Lepidoptera;Lycaenidae;Aricia;Aricia eumedon;Italy;GenBank;NA;MW861648
Lepidoptera;Lycaenidae;Aricia;Aricia eumedon;Switzerland;GenBank;NA;MW861649
Lepidoptera;Lycaenidae;Aricia;Aricia eumedon;Austria;GenBank;NA;MW861650
Lepidoptera;Lycaenidae;Aricia;Aricia eumedon;Italy;GenBank;NA;MW861651
Lepidoptera;Lycaenidae;Aricia;Aricia eumedon;Italy;GenBank;NA;MW861652
Lepidoptera;Lycaenidae;Aricia;Aricia eumedon;Sweden;GenBank;NA;MW861653
Lepidoptera;Lycaenidae;Aricia;Aricia eumedon;Serbia;GenBank;NA;MW861654
Lepidoptera;Lycaenidae;Aricia;Aricia eumedon;Albania;GenBank;NA;MW861655
Lepidoptera;Lycaenidae;Aricia;Aricia eumedon;Estonia;GenBank;NA;MW861656
Lepidoptera;Lycaenidae;Aricia;Aricia eumedon;Spain;GenBank;NA;MW861658
Lepidoptera;Lycaenidae;Aricia;Aricia eumedon;Spain;GenBank;NA;MW861659
Lepidoptera;Lycaenidae;Aricia;Aricia eumedon;Spain;GenBank;NA;MW861662
Lepidoptera;Lycaenidae;Aricia;Aricia eumedon;Spain;GenBank;NA;MW861663
Lepidoptera;Lycaenidae;Aricia;Aricia eumedon;Spain;GenBank;NA;MW861664
Lepidoptera;Lycaenidae;Aricia;Aricia eumedon;Spain;GenBank;NA;MW861668
Lepidoptera;Lycaenidae;Aricia;Aricia eumedon;Spain;GenBank;NA;MW861669
Lepidoptera;Lycaenidae;Aricia;Aricia eumedon;Sweden;GenBank;NA;MW861674
Lepidoptera;Lycaenidae;Aricia;Aricia eumedon;Sweden;GenBank;NA;MW861675
Lepidoptera;Lycaenidae;Aricia;Aricia eumedon;Spain;GenBank;NA;MW861676
Lepidoptera;Lycaenidae;Aricia;Aricia eumedon;Spain;GenBank;NA;MW861677
Lepidoptera;Lycaenidae;Aricia;Aricia eumedon;Spain;GenBank;NA;MW861678
Lepidoptera;Lycaenidae;Aricia;Aricia eumedon;Spain;GenBank;NA;MW861679
Lepidoptera;Lycaenidae;Aricia;Aricia eumedon;Sweden;GenBank;NA;MW861680
Lepidoptera;Nymphalidae;Maniola;Maniola jurtina;Spain;Bold;GBMND76330-21;MW868959
Lepidoptera;Nymphalidae;Euphydryas;Euphydryas intermedia;Russia;GenBank;NA;MZ128129
Lepidoptera;Nymphalidae;Euphydryas;Euphydryas intermedia;Russia;GenBank;NA;MZ128130
Lepidoptera;Nymphalidae;Euphydryas;Euphydryas intermedia;Finland;GenBank;NA;MZ128131
Lepidoptera;Nymphalidae;Euphydryas;Euphydryas intermedia;Finland;GenBank;NA;MZ128132
Lepidoptera;Nymphalidae;Euphydryas;Euphydryas maturna;Finland;GenBank;NA;MZ128133
Lepidoptera;Nymphalidae;Euphydryas;Euphydryas maturna;Finland;GenBank;NA;MZ128134
Lepidoptera;Nymphalidae;Euphydryas;Euphydryas intermedia;Russia;GenBank;NA;MZ128135
Lepidoptera;Lycaenidae;Callophrys;Callophrys rubi;Russia;GenBank;NA;OL457027
Lepidoptera;Lycaenidae;Callophrys;Callophrys rubi;Russia;GenBank;NA;OL457028
Lepidoptera;Lycaenidae;Callophrys;Callophrys rubi;Russia;GenBank;NA;OL457029
Lepidoptera;Lycaenidae;Callophrys;Callophrys rubi;Russia;GenBank;NA;OL457030
Lepidoptera;Lycaenidae;Callophrys;Callophrys rubi;Russia;GenBank;NA;OL457031
Lepidoptera;Lycaenidae;Callophrys;Callophrys rubi;Russia;GenBank;NA;OL457032
Lepidoptera;Nymphalidae;Melitaea;Melitaea phoebe;Spain;GenBank;NA;OL873481
Lepidoptera;Nymphalidae;Melitaea;Melitaea phoebe;Spain;GenBank;NA;OL873483
Lepidoptera;Nymphalidae;Melitaea;Melitaea phoebe;Spain;GenBank;NA;OL873484
Lepidoptera;Nymphalidae;Melitaea;Melitaea phoebe;Spain;GenBank;NA;OL873485
Lepidoptera;Nymphalidae;Melitaea;Melitaea phoebe;Spain;GenBank;NA;OL873487
Lepidoptera;Nymphalidae;Melitaea;Melitaea phoebe;Portugal;GenBank;NA;OL873488
Lepidoptera;Nymphalidae;Melitaea;Melitaea phoebe;Spain;GenBank;NA;OL873489
Lepidoptera;Nymphalidae;Melitaea;Melitaea phoebe;Spain;GenBank;NA;OL873490
Lepidoptera;Nymphalidae;Melitaea;Melitaea phoebe;Spain;GenBank;NA;OL873491
Lepidoptera;Nymphalidae;Melitaea;Melitaea phoebe;Romania;GenBank;NA;OL873496
Lepidoptera;Nymphalidae;Melitaea;Melitaea phoebe;Spain;GenBank;NA;OL873500
Lepidoptera;Nymphalidae;Melitaea;Melitaea phoebe;Spain;GenBank;NA;OL873501
Lepidoptera;Nymphalidae;Melitaea;Melitaea phoebe;Spain;GenBank;NA;OL873504
Lepidoptera;Nymphalidae;Melitaea;Melitaea phoebe;Spain;GenBank;NA;OL873505
Lepidoptera;Nymphalidae;Melitaea;Melitaea phoebe;Spain;GenBank;NA;OL873506
Lepidoptera;Nymphalidae;Melitaea;Melitaea phoebe;Spain;GenBank;NA;OL873521
Lepidoptera;Nymphalidae;Melitaea;Melitaea phoebe;Spain;GenBank;NA;OL873522
Lepidoptera;Nymphalidae;Melitaea;Melitaea phoebe;Spain;GenBank;NA;OL873524
Lepidoptera;Nymphalidae;Melitaea;Melitaea phoebe;Spain;GenBank;NA;OL873526
Lepidoptera;Nymphalidae;Melitaea;Melitaea phoebe;Portugal;GenBank;NA;OL873528
Lepidoptera;Nymphalidae;Melitaea;Melitaea phoebe;Portugal;GenBank;NA;OL873529
Lepidoptera;Nymphalidae;Melitaea;Melitaea phoebe;Spain;GenBank;NA;OL873530
Lepidoptera;Nymphalidae;Melitaea;Melitaea phoebe;Spain;GenBank;NA;OL873534
Lepidoptera;Nymphalidae;Melitaea;Melitaea phoebe;Spain;GenBank;NA;OL873535
Lepidoptera;Nymphalidae;Melitaea;Melitaea phoebe;Spain;GenBank;NA;OL873536
Lepidoptera;Nymphalidae;Melitaea;Melitaea phoebe;Spain;GenBank;NA;OL873538
Lepidoptera;Nymphalidae;Melitaea;Melitaea phoebe;Spain;GenBank;NA;OL873540
Lepidoptera;Nymphalidae;Melitaea;Melitaea phoebe;Spain;GenBank;NA;OL873542
Lepidoptera;Pieridae;Colias;Colias hyale;Austria;GenBank;NA;ON436476
Lepidoptera;Nymphalidae;Aglais;Aglais urticae;Russia;Bold;RDBBC321-05;NA
Lepidoptera;Nymphalidae;Aglais;Aglais urticae;Russia;Bold;RDBBC320-05;NA
Lepidoptera;Nymphalidae;Aglais;Aglais urticae;Russia;Bold;RDBBC322-05;NA
Lepidoptera;Nymphalidae;Aglais;Aglais urticae;Russia;Bold;RDBBC323-05;NA
Lepidoptera;Nymphalidae;Apatura;Apatura ilia;Poland;Bold;EULEP6091-20;NA

Lepidoptera;Nymphalidae;Aphantopus;Aphantopus hyperantus;Poland;Bold;EULEP6088-20;NA
Lepidoptera;Pieridae;Aporia;Aporia crataegi;Spain;Bold;SHIBU015-13;NA
Lepidoptera;Nymphalidae;Araschnia;Araschnia levana;Poland;Bold;EULEP6092-20;NA
Lepidoptera;Nymphalidae;Argynnis;Argynnis aglaja;Sweden;Bold;EULEP6021-20;NA
Lepidoptera;Nymphalidae;Boloria;Boloria selene;Russia;Bold;RDBBC319-05;NA
Lepidoptera;Nymphalidae;Boloria;Boloria selene;Russia;Bold;HBNK056-07;NA
Lepidoptera;Nymphalidae;Boloria;Boloria selene;Denmark;Bold;SNA088-07;NA
Lepidoptera;Nymphalidae;Boloria;Boloria selene;Russia;Bold;HBNK054-07;NA
Lepidoptera;Nymphalidae;Boloria;Boloria selene;Russia;Bold;HBNK055-07;NA
Lepidoptera;Nymphalidae;Boloria;Boloria selene;Russia;Bold;HBNK057-07;NA
Lepidoptera;Nymphalidae;Boloria;Boloria selene;Russia;Bold;RDBBC316-05;NA
Lepidoptera;Nymphalidae;Boloria;Boloria selene;Russia;Bold;RDBBC318-05;NA
Lepidoptera;Nymphalidae;Boloria;Boloria selene;Russia;Bold;HBNK053-07;NA
Lepidoptera;Nymphalidae;Boloria;Boloria selene;Russia;Bold;HBNK052-07;NA
Lepidoptera;Nymphalidae;Boloria;Boloria thore;Russia;Bold;HBNK078-07;NA
Lepidoptera;Nymphalidae;Boloria;Boloria thore;Russia;Bold;HBNK076-07;NA
Lepidoptera;Nymphalidae;Boloria;Boloria thore;Russia;Bold;HBNK075-07;NA
Lepidoptera;Nymphalidae;Boloria;Boloria thore;Russia;Bold;HBNK077-07;NA
Lepidoptera;Nymphalidae;Boloria;Boloria thore;Russia;Bold;HBNK074-07;NA
Lepidoptera;Nymphalidae;Boloria;Boloria titania;Albania;Bold;EULEP6232-20;NA
Lepidoptera;Nymphalidae;Brenthis;Brenthis ino;Albania;Bold;EULEP6217-20;NA
Lepidoptera;Hesperiidae;Carterocephalus;Carterocephalus palaemon;Albania;Bold;EULEP6228-20;NA
Lepidoptera;Nymphalidae;Coenonympha;Coenonympha arcania;Poland;Bold;EULEP6096-20;NA
Lepidoptera;Nymphalidae;Coenonympha;Coenonympha arcania;Sweden;Bold;EULEP6020-20;NA
Lepidoptera;Lycaenidae;Cupido;Cupido osiris;Albania;Bold;EULEP6229-20;NA
Lepidoptera;Nymphalidae;Erebia;Erebia aethiops;Albania;Bold;EULEP6219-20;NA
Lepidoptera;Nymphalidae;Erebia;Erebia epiphron;Albania;Bold;EULEP6211-20;NA
Lepidoptera;Hesperiidae;Hesperia;Hesperia comma;Albania;Bold;EULEP6213-20;NA
Lepidoptera;Lycaenidae;Eumedonia;Eumedonia eumedon;Greece;Bold;EULEP6266-20;NA
Lepidoptera;Riodinidae;Hamearis;Hamearis lucina;Albania;Bold;EULEP6230-20;NA
Lepidoptera;Nymphalidae;Hyponephele;Hyponephele lycaon;Poland;Bold;EULEP6093-20;NA
Lepidoptera;Nymphalidae;Nymphalis;Nymphalis antiopa;Russia;Bold;HBNKB288-07;NA
Lepidoptera;Lycaenidae;Lycaena;Lycaena phlaeas;Romania;Bold;EULEP6013-20;NA
Lepidoptera;Lycaenidae;Lycaena;Lycaena thersamon;Albania;Bold;EULEP6233-20;NA
Lepidoptera;Lycaenidae;Lycaena;Lycaena tityrus;Albania;Bold;EULEP6218-20;NA
Lepidoptera;Lycaenidae;Lycaena;Lycaena virgaureae;Poland;Bold;EULEP6090-20;NA
Lepidoptera;Nymphalidae;Melanargia;Melanargia galathea;Poland;Bold;EULEP6097-20;NA
Lepidoptera;Nymphalidae;Melitaea;Melitaea diamina;Albania;Bold;EULEP6231-20;NA
Lepidoptera;Nymphalidae;Nymphalis;Nymphalis antiopa;Russia;Bold;HBNKB222-07;NA
Lepidoptera;Nymphalidae;Nymphalis;Nymphalis antiopa;Russia;Bold;HBNKB297-07;NA
Lepidoptera;Nymphalidae;Nymphalis;Nymphalis antiopa;Russia;Bold;HBNKB298-07;NA
Lepidoptera;Nymphalidae;Nymphalis;Nymphalis antiopa;Finland;Bold;RDHP380-06;NA
Lepidoptera;Nymphalidae;Nymphalis;Nymphalis antiopa;Russia;Bold;HBNKB287-07;NA
Lepidoptera;Hesperiidae;Ochlodes;Ochlodes sylvanus;Poland;Bold;EULEP6094-20;NA
Lepidoptera;Papilionidae;Papilio;Papilio machaon;Albania;Bold;EULEP6216-20;NA
Lepidoptera;Lycaenidae;Polyommatus;Polyommatus amandus;Albania;Bold;EULEP6220-20;NA
Lepidoptera;Lycaenidae;Polyommatus;Polyommatus icarus;Russia;Bold;WMB6631-18;NA
Lepidoptera;Lycaenidae;Polyommatus;Polyommatus icarus;Russia;Bold;WMB6630-18;NA
Lepidoptera;Pieridae;Pontia;Pontia callidice;Switzerland;Bold;WMB6886-19;NA
Lepidoptera;Pieridae;Pontia;Pontia edusa;Poland;Bold;EULEP6089-20;NA
Lepidoptera;Nymphalidae;Satyrus;Satyrus ferula;Albania;Bold;EULEP6214-20;NA
Lepidoptera;Hesperiidae;Thymelicus;Thymelicus lineola;Sweden;Bold;EULEP6019-20;NA
Lepidoptera;Nymphalidae;Vanessa;Vanessa cardui;Albania;Bold;EULEP6237-20;NA
Odonata;Aeshnidae;Aeshna;Aeshna juncea;Russia;GenBank;NA;AB708582
Odonata;Aeshnidae;Aeshna;Aeshna juncea;Russia;GenBank;NA;AB708583
Odonata;Aeshnidae;Aeshna;Aeshna juncea;Russia;GenBank;NA;AB708591
Odonata;Corduliidae;Somatochlora;Somatochlora alpestris;Russia;GenBank;NA;AB708911
Odonata;Corduliidae;Somatochlora;Somatochlora alpestris;Russia;GenBank;NA;AB708912
Odonata;Corduliidae;Somatochlora;Somatochlora metallica;Sweden;GenBank;NA;AB708931
Odonata;Corduliidae;Somatochlora;Somatochlora metallica;Sweden;GenBank;NA;AB708932
Odonata;Libellulidae;Sympetrum;Sympetrum depressiusculum;Russia;GenBank;NA;AB709125
Odonata;Libellulidae;Sympetrum;Sympetrum depressiusculum;Russia;GenBank;NA;AB709128
Odonata;Libellulidae;Sympetrum;Sympetrum depressiusculum;Russia;GenBank;NA;AB709129
Odonata;Libellulidae;Sympetrum;Sympetrum depressiusculum;Russia;GenBank;NA;AB709131
Odonata;Libellulidae;Sympetrum;Sympetrum depressiusculum;Russia;GenBank;NA;AB709132
Odonata;Libellulidae;Sympetrum;Sympetrum depressiusculum;Russia;GenBank;NA;AB709134
Odonata;Libellulidae;Sympetrum;Sympetrum depressiusculum;Russia;GenBank;NA;AB709135
Odonata;Libellulidae;Sympetrum;Sympetrum depressiusculum;Russia;GenBank;NA;AB709137
Odonata;Libellulidae;Sympetrum;Sympetrum depressiusculum;Russia;GenBank;NA;AB709139
Odonata;Libellulidae;Sympetrum;Sympetrum depressiusculum;Russia;GenBank;NA;AB709140
Odonata;Libellulidae;Sympetrum;Sympetrum depressiusculum;Russia;GenBank;NA;AB709141
Odonata;Libellulidae;Sympetrum;Sympetrum depressiusculum;Russia;GenBank;NA;AB709143
Odonata;Aeshnidae;Aeshna;Aeshna juncea;Finland;GenBank;NA;AB711458
Odonata;Aeshnidae;Aeshna;Aeshna subarctica;Finland;GenBank;NA;AB711462
Odonata;Aeshnidae;Aeshna;Aeshna juncea;Russia;GenBank;NA;AB711463
Odonata;Coenagrionidae;Ischnura;Ischnura elegans;Germany;GenBank;NA;HM376192
Odonata;Libellulidae;Leucorrhinia;Leucorrhinia pectoralis;Sweden;GenBank;NA;JN991193
Odonata;Libellulidae;Leucorrhinia;Leucorrhinia dubia;Sweden;GenBank;NA;JN991194

Odonata;Aeshnidae;Aeshna;Aeshna cyanea;Germany;GenBank;NA;KC912199
Odonata;Aeshnidae;Aeshna;Aeshna cyanea;Germany;GenBank;NA;KC912200
Odonata;Aeshnidae;Aeshna;Aeshna cyanea;Germany;GenBank;NA;KC912201
Odonata;Aeshnidae;Aeshna;Aeshna cyanea;Germany;GenBank;NA;KC912202
Odonata;Aeshnidae;Aeshna;Aeshna grandis;Germany;GenBank;NA;KC912203
Odonata;Aeshnidae;Aeshna;Aeshna mixta;Germany;GenBank;NA;KC912204
Odonata;Aeshnidae;Aeshna;Aeshna mixta;Germany;GenBank;NA;KC912205
Odonata;Aeshnidae;Brachytron;Brachytron pratense;France;GenBank;NA;KC912235
Odonata;Aeshnidae;Brachytron;Brachytron pratense;France;GenBank;NA;KC912236
Odonata;Libellulidae;Orthetrum;Orthetrum coerulescens;Italy;GenBank;NA;KC912263
Odonata;Libellulidae;Orthetrum;Orthetrum coerulescens;Italy;GenBank;NA;KC912264
Odonata;Libellulidae;Orthetrum;Orthetrum coerulescens;Italy;GenBank;NA;KC912265
Odonata;Libellulidae;Orthetrum;Orthetrum coerulescens;Germany;GenBank;NA;KC912266
Odonata;Libellulidae;Orthetrum;Orthetrum coerulescens;Germany;GenBank;NA;KC912267
Odonata;Libellulidae;Orthetrum;Orthetrum coerulescens;Germany;GenBank;NA;KC912268
Odonata;Libellulidae;Orthetrum;Orthetrum coerulescens;Germany;GenBank;NA;KC912269
Odonata;Libellulidae;Orthetrum;Orthetrum coerulescens;Germany;GenBank;NA;KC912270
Odonata;Libellulidae;Orthetrum;Orthetrum coerulescens;Germany;GenBank;NA;KC912271
Odonata;Coenagrionidae;Ceriagrion;Ceriagrion tenellum;Spain;GenBank;NA;KC912305
Odonata;Coenagrionidae;Ceriagrion;Ceriagrion tenellum;Spain;GenBank;NA;KC912306
Odonata;Coenagrionidae;Ceriagrion;Ceriagrion tenellum;Spain;GenBank;NA;KC912307
Odonata;Coenagrionidae;Ceriagrion;Ceriagrion tenellum;Spain;GenBank;NA;KC912308
Odonata;Coenagrionidae;Ceriagrion;Ceriagrion tenellum;Spain;GenBank;NA;KC912309
Odonata;Coenagrionidae;Enallagma;Enallagma cyathigerum;Spain;GenBank;NA;KC912310
Odonata;Coenagrionidae;Enallagma;Enallagma cyathigerum;Spain;GenBank;NA;KC912311
Odonata;Coenagrionidae;Enallagma;Enallagma cyathigerum;Spain;GenBank;NA;KC912312
Odonata;Coenagrionidae;Enallagma;Enallagma cyathigerum;Spain;GenBank;NA;KC912313
Odonata;Coenagrionidae;Enallagma;Enallagma cyathigerum;Spain;GenBank;NA;KC912314
Odonata;Aeshnidae;Aeshna;Aeshna juncea;Netherlands;GenBank;NA;KF369278
Odonata;Coenagrionidae;Ischnura;Ischnura elegans;Netherlands;GenBank;NA;KF369415
Odonata;Cordulegastridae;Cordulegaster;Cordulegaster bidentata;Montenegro;GenBank;NA;KF584922
Odonata;Cordulegastridae;Cordulegaster;Cordulegaster bidentata;Albania;GenBank;NA;KF584923
Odonata;Cordulegastridae;Cordulegaster;Cordulegaster bidentata;Albania;GenBank;NA;KF584924
Odonata;Cordulegastridae;Cordulegaster;Cordulegaster bidentata;Greece;GenBank;NA;KF584925
Odonata;Cordulegastridae;Cordulegaster;Cordulegaster bidentata;Greece;GenBank;NA;KF584926
Odonata;Cordulegastridae;Cordulegaster;Cordulegaster bidentata;Greece;GenBank;NA;KF584927
Odonata;Cordulegastridae;Cordulegaster;Cordulegaster bidentata;Greece;GenBank;NA;KF584928
Odonata;Cordulegastridae;Cordulegaster;Cordulegaster bidentata;Italy;Bold;GBMHO342-14;KF584929
Odonata;Cordulegastridae;Cordulegaster;Cordulegaster bidentata;Italy;Bold;GBMHO343-14;KF584930
Odonata;Cordulegastridae;Cordulegaster;Cordulegaster bidentata;Greece;GenBank;NA;KF584931
Odonata;Cordulegastridae;Cordulegaster;Cordulegaster boltonii
iberica;Austria;GenBank;NA;KF584932
Odonata;Cordulegastridae;Cordulegaster;Cordulegaster boltonii;Italy;GenBank;NA;KF584933
Odonata;Cordulegastridae;Cordulegaster;Cordulegaster boltonii;Italy;GenBank;NA;KF584934
Odonata;Cordulegastridae;Cordulegaster;Cordulegaster heros;Greece;Bold;GBMHO338-14;KF584940
Odonata;Cordulegastridae;Cordulegaster;Cordulegaster trinacriae;Italy;GenBank;NA;KF584946
Odonata;Cordulegastridae;Cordulegaster;Cordulegaster boltonii iberica;Spain;GenBank;NA;KF584956
Odonata;Cordulegastridae;Cordulegaster;Cordulegaster boltonii
immaculifrons;France;GenBank;NA;KF584957
Odonata;Cordulegastridae;Cordulegaster;Cordulegaster bidentata;Germany;GenBank;NA;KF584958
Odonata;Cordulegastridae;Cordulegaster;Cordulegaster boltonii
boltonii;Portugal;GenBank;NA;KF584959
Odonata;Cordulegastridae;Cordulegaster;Cordulegaster boltonii
boltonii;France;GenBank;NA;KF584960
Odonata;Cordulegastridae;Cordulegaster;Cordulegaster boltonii iberica;Spain;GenBank;NA;KF584961
Odonata;Cordulegastridae;Cordulegaster;Cordulegaster boltonii iberica;Spain;GenBank;NA;KF584962
Odonata;Cordulegastridae;Cordulegaster;Cordulegaster bidentata;France;Bold;GBMHO324-14;KF584963
Odonata;Cordulegastridae;Cordulegaster;Cordulegaster boltonii algirica;Spain;GenBank;NA;KF584964
Odonata;Cordulegastridae;Cordulegaster;Cordulegaster boltonii
immaculifrons;Portugal;GenBank;NA;KF584965
Odonata;Cordulegastridae;Cordulegaster;Cordulegaster boltonii iberica;Spain;GenBank;NA;KF584967
Odonata;Cordulegastridae;Cordulegaster;Cordulegaster boltonii iberica;Spain;GenBank;NA;KF584968
Odonata;Aeshnidae;Anax;Anax imperator;Portugal;GenBank;NA;KF584974
Odonata;Aeshnidae;Aeshna;Aeshna juncea;Germany;GenBank;NA;KU180297
Odonata;Aeshnidae;Aeshna;Aeshna subarctica;Germany;GenBank;NA;KU180298
Odonata;Aeshnidae;Aeshna;Aeshna grandis;Germany;GenBank;NA;KU180299
Odonata;Aeshnidae;Aeshna;Aeshna cyanea;Belgium;GenBank;NA;KU180304
Odonata;Aeshnidae;Aeshna;Aeshna cyanea;Italy;GenBank;NA;KU180305
Odonata;Aeshnidae;Aeshna;Aeshna cyanea;Poland;GenBank;NA;KU180306
Odonata;Aeshnidae;Aeshna;Aeshna cyanea;Germany;GenBank;NA;KU180307
Odonata;Aeshnidae;Aeshna;Aeshna cyanea;Poland;GenBank;NA;KU180308
Odonata;Aeshnidae;Aeshna;Aeshna cyanea;Spain;GenBank;NA;KU180309
Odonata;Aeshnidae;Aeshna;Aeshna cyanea;Russia;GenBank;NA;KU180312
Odonata;Aeshnidae;Aeshna;Aeshna cyanea;Russia;GenBank;NA;KU180313
Odonata;Aeshnidae;Aeshna;Aeshna cyanea;Russia;GenBank;NA;KU180317
Odonata;Aeshnidae;Aeshna;Aeshna cyanea;Russia;GenBank;NA;KU180318
Odonata;Coenagrionidae;Pyrrhosoma;Pyrrhosoma nymphula;Belgium;GenBank;NA;KU220874
Odonata;Coenagrionidae;Pyrrhosoma;Pyrrhosoma nymphula;Turkey;GenBank;NA;KU220878

Odonata;Coenagrionidae;Pyrrhosoma;Pyrrhosoma nymphula;Portugal;GenBank;NA;KU220879
Odonata;Coenagrionidae;Pyrrhosoma;Pyrrhosoma nymphula;Portugal;GenBank;NA;KU220880
Odonata;Coenagrionidae;Pyrrhosoma;Pyrrhosoma nymphula;Portugal;GenBank;NA;KU220881
Odonata;Coenagrionidae;Pyrrhosoma;Pyrrhosoma nymphula;Portugal;GenBank;NA;KU220884
Odonata;Coenagrionidae;Pyrrhosoma;Pyrrhosoma nymphula;Macedonia;GenBank;NA;KU220885
Odonata;Coenagrionidae;Coenagrion;Coenagrion puella;Netherlands;GenBank;NA;KU695839
Odonata;Libellulidae;Leucorrhinia;Leucorrhinia dubia;Germany;GenBank;NA;LC366710
Odonata;Libellulidae;Leucorrhinia;Leucorrhinia dubia;Germany;GenBank;NA;LC366712
Odonata;Libellulidae;Libellula;Libellula quadrimaculata;Germany;GenBank;NA;LC366713
Odonata;Aeshnidae;Brachytron;Brachytron pratense;Germany;GenBank;NA;LC366714
Odonata;Libellulidae;Sympetrum;Sympetrum striolatum;Croatia;GenBank;NA;LC366852
Odonata;Libellulidae;Crocothemis;Crocothemis erythraea;Croatia;GenBank;NA;LC366854
Odonata;Libellulidae;Crocothemis;Crocothemis erythraea;Croatia;GenBank;NA;LC366855
Odonata;Libellulidae;Sympetrum;Sympetrum vulgatum;France;GenBank;NA;LT634113
Odonata;Libellulidae;Sympetrum;Sympetrum vulgatum;France;GenBank;NA;LT634114
Odonata;Libellulidae;Sympetrum;Sympetrum vulgatum;Finland;GenBank;NA;LT634115
Odonata;Libellulidae;Sympetrum;Sympetrum vulgatum;Finland;GenBank;NA;LT634116
Odonata;Libellulidae;Sympetrum;Sympetrum vulgatum;Spain;GenBank;NA;LT634117
Odonata;Libellulidae;Sympetrum;Sympetrum vulgatum;Spain;GenBank;NA;LT634118
Odonata;Libellulidae;Sympetrum;Sympetrum vulgatum;Spain;GenBank;NA;LT634119
Odonata;Libellulidae;Sympetrum;Sympetrum vulgatum;Spain;GenBank;NA;LT634120
Odonata;Libellulidae;Sympetrum;Sympetrum vulgatum;Spain;GenBank;NA;LT634121
Odonata;Libellulidae;Sympetrum;Sympetrum vulgatum;Spain;GenBank;NA;LT634122
Odonata;Libellulidae;Sympetrum;Sympetrum vulgatum;Spain;GenBank;NA;LT634123
Odonata;Libellulidae;Sympetrum;Sympetrum vulgatum;Spain;GenBank;NA;LT634124
Odonata;Cordulegastridae;Cordulegaster;Cordulegaster heros;Bulgaria;GenBank;NA;MK779811
Odonata;Cordulegastridae;Cordulegaster;Cordulegaster bidentata;Czech Republic;GenBank;NA;MK779814
Odonata;Aeshnidae;Aeshna;Aeshna juncea;France;GenBank;NA;MN735555
Odonata;Aeshnidae;Aeshna;Aeshna mixta;Russia;Bold;GBMND49018-21;MT001428
Odonata;Cordulegastridae;Cordulegaster;Cordulegaster bidentata;Bulgaria;GenBank;NA;MT811768
Odonata;Cordulegastridae;Cordulegaster;Cordulegaster bidentata;Bulgaria;GenBank;NA;MT811769
Odonata;Cordulegastridae;Cordulegaster;Cordulegaster bidentata;Bulgaria;GenBank;NA;MT811770
Odonata;Gomphidae;Gomphus;Gomphus flavipes;Romania;GenBank;NA;MW139673
Odonata;Coenagrionidae;Erythromma;Erythromma viridulum;Romania;GenBank;NA;MW139674
Odonata;Lestidae;Sympecma;Sympecma fusca;Albania;GenBank;NA;MW208367
Odonata;Lestidae;Sympecma;Sympecma fusca;Albania;GenBank;NA;MW208368
Odonata;Lestidae;Chalcolestes;Chalcolestes viridis;Bosnia and Herzegovina;GenBank;NA;MW208373
Odonata;Lestidae;Chalcolestes;Chalcolestes viridis;Montenegro;GenBank;NA;MW208374
Odonata;Lestidae;Chalcolestes;Chalcolestes parvidens;Bosnia and Herzegovina;GenBank;NA;MW208375
Odonata;Lestidae;Chalcolestes;Chalcolestes parvidens;Bosnia and Herzegovina;GenBank;NA;MW208376
Odonata;Lestidae;Chalcolestes;Chalcolestes parvidens;Austria;GenBank;NA;MW208377
Odonata;Libellulidae;Orthetrum;Orthetrum cancellatum;Albania;GenBank;NA;MW208378
Odonata;Libellulidae;Orthetrum;Orthetrum cancellatum;Albania;GenBank;NA;MW208379
Odonata;Coenagrionidae;Ischnura;Ischnura elegans;Croatia;GenBank;NA;MW208391
Odonata;Coenagrionidae;Ischnura;Ischnura elegans;Albania;GenBank;NA;MW208393
Odonata;Coenagrionidae;Ischnura;Ischnura elegans;Albania;GenBank;NA;MW208394
Odonata;Coenagrionidae;Ischnura;Ischnura elegans;Albania;GenBank;NA;MW208395
Odonata;Coenagrionidae;Ischnura;Ischnura elegans;Albania;GenBank;NA;MW208396
Odonata;Coenagrionidae;Ischnura;Ischnura elegans;Albania;GenBank;NA;MW208397
Odonata;Coenagrionidae;Ischnura;Ischnura elegans;Austria;GenBank;NA;MW208398
Odonata;Coenagrionidae;Ischnura;Ischnura elegans;Austria;GenBank;NA;MW208399
Odonata;Coenagrionidae;Erythromma;Erythromma lindenii;Austria;GenBank;NA;MW208400
Odonata;Coenagrionidae;Enallagma;Enallagma cyathigerum;Croatia;GenBank;NA;MW208402
Odonata;Coenagrionidae;Enallagma;Enallagma cyathigerum;Bosnia and Herzegovina;GenBank;NA;MW208403
Odonata;Coenagrionidae;Enallagma;Enallagma cyathigerum;Austria;GenBank;NA;MW208404
Odonata;Coenagrionidae;Enallagma;Enallagma cyathigerum;Austria;GenBank;NA;MW208405
Odonata;Gomphidae;Gomphus;Gomphus vulgatissimus;Albania;GenBank;NA;MW208406
Odonata;Gomphidae;Gomphus;Gomphus vulgatissimus;Albania;GenBank;NA;MW208407
Odonata;Gomphidae;Gomphus;Gomphus vulgatissimus;Austria;GenBank;NA;MW208408
Odonata;Gomphidae;Gomphus;Gomphus vulgatissimus;Austria;GenBank;NA;MW208409
Odonata;Calopterygidae;Calopteryx;Calopteryx splendens;Croatia;GenBank;NA;MW208413
Odonata;Calopterygidae;Calopteryx;Calopteryx splendens;Austria;GenBank;NA;MW208414
Odonata;Calopterygidae;Calopteryx;Calopteryx splendens;Austria;GenBank;NA;MW208415
Odonata;Coenagrionidae;Ischnura;Ischnura elegans;Sweden;GenBank;NA;MW509062
Odonata;Coenagrionidae;Ischnura;Ischnura elegans;Finland;GenBank;NA;MW509063
Odonata;Coenagrionidae;Ischnura;Ischnura elegans;Finland;GenBank;NA;MW509064
Odonata;Coenagrionidae;Ischnura;Ischnura elegans;Finland;GenBank;NA;MW509065
Odonata;Coenagrionidae;Ischnura;Ischnura elegans;Finland;GenBank;NA;MW509066
Odonata;Gomphidae;Gomphus;Gomphus flavipes;Germany;GenBank;NA;MW570923
Odonata;Gomphidae;Gomphus;Gomphus flavipes;Germany;GenBank;NA;MW570924
Odonata;Gomphidae;Gomphus;Gomphus flavipes;Germany;GenBank;NA;MW570925
Odonata;Gomphidae;Gomphus;Gomphus vulgatissimus;Germany;GenBank;NA;MW570933
Odonata;Gomphidae;Gomphus;Gomphus vulgatissimus;France;GenBank;NA;MW570935
Odonata;Gomphidae;Ophiogomphus;Ophiogomphus cecilia;Germany;GenBank;NA;MW570947
Odonata;Gomphidae;Ophiogomphus;Ophiogomphus cecilia;Germany;GenBank;NA;MW570948
Odonata;Calopterygidae;Paragomphus;Paragomphus genei;Portugal;GenBank;NA;MW570956

Odonata;Calopterygidae;Paragomphus;Paragomphus genei;Spain;GenBank;NA;MW570957
Odonata;Calopterygidae;Paragomphus;Paragomphus genei;Portugal;GenBank;NA;MW570958
Odonata;Gomphidae;Lindenia;Lindenia tetraphylla;Turkey;GenBank;NA;MW570961
Odonata;Coenagrionidae;Ischnura;Ischnura elegans;Germany;Bold;GBMHO3835-19;NC_031824
Coleoptera;Ciidae;Cis;Cis pygmaeus;Slovakia;Bold;GBCL32888-19;EF490164
Coleoptera;Elateridae;Ampedus;Ampedus nigroflavus;Finland;GenBank;NA;EF589381
Coleoptera;Curculionidae;Crypturgus;Crypturgus hispidulus;Norway;GenBank;NA;EU011807
Coleoptera;Curculionidae;Crypturgus;Crypturgus pusillus;Sweden;GenBank;NA;EU011812
Coleoptera;Curculionidae;Crypturgus;Crypturgus pusillus;Norway;GenBank;NA;EU011813
Coleoptera;Curculionidae;Kyklioacalles;Kyklioacalles navieresi;France;GenBank;NA;EU286450
Coleoptera;Curculionidae;Echinodera;Echinodera hypocrita;Germany;GenBank;NA;EU286452
Coleoptera;Curculionidae;Acallorneuma;Acallorneuma doderoi;Italy;GenBank;NA;EU286457
Coleoptera;Curculionidae;Elliptacalles;Elliptacalles longus;Italy;GenBank;NA;EU286458
Coleoptera;Curculionidae;Coloracalles;Coloracalles humerosus;France;GenBank;NA;EU286460
Coleoptera;Curculionidae;Dichromacalles;Dichromacalles rolletii;Italy;GenBank;NA;EU286470
Coleoptera;Curculionidae;Echinodera;Echinodera siciliensis;Italy;GenBank;NA;EU286480
Coleoptera;Curculionidae;Kyklioacalles;Kyklioacalles teter;Italy;GenBank;NA;EU286484
Coleoptera;Curculionidae;Kyklioacalles;Kyklioacalles characivorus;Italy;GenBank;NA;EU286485
Coleoptera;Curculionidae;Kyklioacalles;Kyklioacalles punctaticollis punctaticollis;Spain;GenBank;NA;EU286487
Coleoptera;Curculionidae;Kyklioacalles;Kyklioacalles punctaticollis meteoricus;Spain;GenBank;NA;EU286489
Coleoptera;Curculionidae;Kyklioacalles;Kyklioacalles roboris;France;GenBank;NA;EU286494
Coleoptera;Curculionidae;Elliptacalles;Elliptacalles longus;Spain;GenBank;NA;EU286499
Coleoptera;Curculionidae;Elliptacalles;Elliptacalles longus;Spain;GenBank;NA;EU286501
Coleoptera;Curculionidae;Kyklioacalles;Kyklioacalles aubei;Slovenia;GenBank;NA;EU286502
Coleoptera;Curculionidae;Kyklioacalles;Kyklioacalles aubei;France;GenBank;NA;EU286503
Coleoptera;Curculionidae;Echinodera;Echinodera bellieri bellieri;Italy;GenBank;NA;EU286505
Coleoptera;Curculionidae;Acallocrates;Acallocrates denticollis;Croatia;Bold;GBCL4192-09;EU286509
Coleoptera;Curculionidae;Echinodera;Echinodera hypocrita;Croatia;GenBank;NA;EU286520
Coleoptera;Curculionidae;Cryptorhynchus;Cryptorhynchus lapathi;Germany;GenBank;NA;EU286523
Coleoptera;Cerambycidae;Prinobius;Prinobius myardi myardi;France;GenBank;NA;EU752053
Coleoptera;Cerambycidae;Prinobius;Prinobius myardi myardi;France;GenBank;NA;EU752054
Coleoptera;Ciidae;Cis;Cis festivus;Slovakia;Bold;GBCL32887-19;FJ904060
Coleoptera;Staphylinidae;Dinaraea;Dinaraea aequata;Czech Republic;GenBank;NA;GQ980935
Coleoptera;Cerambycidae;Tetropium;Tetropium fuscum;Germany;GenBank;NA;GU003936
Coleoptera;Scraptiidae;Anaspis;Anaspis frontalis;Germany;GenBank;NA;HQ954526
Coleoptera;Lucanidae;Platycerus;Platycerus caraboides;Germany;GenBank;NA;HQ954543
Coleoptera;Scarabaeidae;Calicnemis;Calicnemis latreillii;France;GenBank;NA;JF888650
Coleoptera;Scarabaeidae;Calicnemis;Calicnemis latreillii;France;GenBank;NA;JF888651
Coleoptera;Cerambycidae;Stenostola;Stenostola ferrea;Czech Republic;GenBank;NA;JQ907477
Coleoptera;Curculionidae;Trypophloeus;Trypophloeus alni;Russia;GenBank;NA;JX263805
Coleoptera;Curculionidae;Dryocoetes;Dryocoetes autographus;Russia;GenBank;NA;JX263816
Coleoptera;Curculionidae;Taphrorychus;Taphrorychus villifrons;Ukraine;GenBank;NA;JX263818
Coleoptera;Curculionidae;Hylastinus;Hylastinus fankhauseri;Austria;GenBank;NA;JX263824
Coleoptera;Curculionidae;Hylesinus;Hylesinus toranio;United Kingdom;GenBank;NA;JX263828
Coleoptera;Curculionidae;Hylastes;Hylastes brunneus;Norway;GenBank;NA;JX263831
Coleoptera;Curculionidae;Orthotomicus;Orthotomicus proximus;Sweden;GenBank;NA;JX263835
Coleoptera;Curculionidae;Polygraphus;Polygraphus poligraphus;Sweden;GenBank;NA;JX263851
Coleoptera;Curculionidae;Trypodendron;Trypodendron domesticum;Norway;GenBank;NA;JX263874
Coleoptera;Cerambycidae;Acanthocinus;Acanthocinus aedilis;Poland;GenBank;NA;KC593301
Coleoptera;Cerambycidae;Acanthocinus;Acanthocinus aedilis;Poland;GenBank;NA;KC593302
Coleoptera;Cerambycidae;Acanthocinus;Acanthocinus aedilis;Poland;GenBank;NA;KC593303
Coleoptera;Cerambycidae;Acanthocinus;Acanthocinus aedilis;Poland;GenBank;NA;KC593304
Coleoptera;Cerambycidae;Acanthocinus;Acanthocinus aedilis;Poland;GenBank;NA;KC593305
Coleoptera;Cerambycidae;Arhopalus;Arhopalus rusticus;Poland;GenBank;NA;KC593309
Coleoptera;Cerambycidae;Arhopalus;Arhopalus rusticus;Poland;GenBank;NA;KC593310
Coleoptera;Cerambycidae;Arhopalus;Arhopalus rusticus;Poland;GenBank;NA;KC593311
Coleoptera;Cerambycidae;Callidium;Callidium violaceum;Poland;GenBank;NA;KC593313
Coleoptera;Cerambycidae;Callidium;Callidium violaceum;Poland;GenBank;NA;KC593314
Coleoptera;Cerambycidae;Callidium;Callidium violaceum;Poland;GenBank;NA;KC593315
Coleoptera;Cerambycidae;Callidium;Callidium violaceum;Poland;GenBank;NA;KC593316
Coleoptera;Cerambycidae;Callidium;Callidium violaceum;Poland;GenBank;NA;KC593317
Coleoptera;Cerambycidae;Molorchus;Molorchus minor;Poland;GenBank;NA;KC593326
Coleoptera;Cerambycidae;Molorchus;Molorchus minor;Poland;GenBank;NA;KC593327
Coleoptera;Cerambycidae;Molorchus;Molorchus minor;Poland;GenBank;NA;KC593328
Coleoptera;Cerambycidae;Molorchus;Molorchus minor;Poland;GenBank;NA;KC593329
Coleoptera;Cerambycidae;Molorchus;Molorchus minor;Poland;GenBank;NA;KC593330
Coleoptera;Cerambycidae;Pyrrhidium;Pyrrhidium sanguineum;Poland;GenBank;NA;KC593336
Coleoptera;Cerambycidae;Pyrrhidium;Pyrrhidium sanguineum;Poland;GenBank;NA;KC593337
Coleoptera;Cerambycidae;Pyrrhidium;Pyrrhidium sanguineum;Poland;GenBank;NA;KC593338
Coleoptera;Curculionidae;Crypturgus;Crypturgus hispidulus;Russia;GenBank;NA;KC845431
Coleoptera;Curculionidae;Pityophthorus;Pityophthorus glabratus;Sweden;GenBank;NA;KC845435
Coleoptera;Curculionidae;Pityophthorus;Pityophthorus lichtensteinii;Norway;GenBank;NA;KC845436
Coleoptera;Curculionidae;Cryphalus;Cryphalus saltuarius;Sweden;GenBank;NA;KC845437
Coleoptera;Curculionidae;Cryphalus;Cryphalus saltuarius;Norway;GenBank;NA;KC845438
Coleoptera;Curculionidae;Ernoporus;Ernoporus tiliae;Denmark;GenBank;NA;KC845439
Coleoptera;Curculionidae;Dryocoetes;Dryocoetes hectographus;Norway;GenBank;NA;KC845446

Coleoptera;Curculionidae;Dryocoetes;Dryocoetes autographus;Hungary;GenBank;NA;KC845447
Coleoptera;Curculionidae;Dryocoetes;Dryocoetes autographus;Estonia;GenBank;NA;KC845450
Coleoptera;Curculionidae;Dryocoetes;Dryocoetes autographus;Norway;GenBank;NA;KC845451
Coleoptera;Curculionidae;Dryocoetes;Dryocoetes villosus;Norway;GenBank;NA;KC845460
Coleoptera;Curculionidae;Hylesinus;Hylesinus varius;Ukraine;GenBank;NA;KC845461
Coleoptera;Curculionidae;Hylesinus;Hylesinus varius;Hungary;GenBank;NA;KC845462
Coleoptera;Curculionidae;Hylurgops;Hylurgops palliatus;Norway;GenBank;NA;KC845463
Coleoptera;Curculionidae;Hylurgops;Hylurgops palliatus;Norway;GenBank;NA;KC845464
Coleoptera;Curculionidae;Hylastes;Hylastes cunicularius;Norway;GenBank;NA;KC845465
Coleoptera;Curculionidae;Hylastes;Hylastes cunicularius;Russia;GenBank;NA;KC845466
Coleoptera;Curculionidae;Hylastes;Hylastes opacus;Sweden;GenBank;NA;KC845468
Coleoptera;Curculionidae;Hylastes;Hylastes opacus;Russia;GenBank;NA;KC845471
Coleoptera;Curculionidae;Hylastes;Hylastes attenuatus;Sweden;GenBank;NA;KC845472
Coleoptera;Curculionidae;Ips;Ips typographus;Norway;GenBank;NA;KC845475
Coleoptera;Curculionidae;Ips;Ips typographus;Estonia;GenBank;NA;KC845476
Coleoptera;Curculionidae;Ips;Ips sexdentatus;Spain;GenBank;NA;KC845477
Coleoptera;Curculionidae;Orthotomicus;Orthotomicus proximus;Estonia;GenBank;NA;KC845478
Coleoptera;Curculionidae;Orthotomicus;Orthotomicus suturalis;Estonia;GenBank;NA;KC845479
Coleoptera;Curculionidae;Orthotomicus;Orthotomicus erosus;Spain;GenBank;NA;KC845480
Coleoptera;Curculionidae;Pityogenes;Pityogenes trepanatus;Sweden;GenBank;NA;KC845481
Coleoptera;Curculionidae;Pityogenes;Pityogenes bidentatus;Sweden;GenBank;NA;KC845482
Coleoptera;Curculionidae;Pityogenes;Pityogenes chalcographus;Norway;GenBank;NA;KC845483
Coleoptera;Curculionidae;Pityogenes;Pityogenes chalcographus;Estonia;GenBank;NA;KC845484
Coleoptera;Curculionidae;Polygraphus;Polygraphus grandiclava;Czech Republic;GenBank;NA;KC845493
Coleoptera;Curculionidae;Polygraphus;Polygraphus grandiclava;Italy;GenBank;NA;KC845494
Coleoptera;Curculionidae;Polygraphus;Polygraphus grandiclava;Italy;GenBank;NA;KC845495
Coleoptera;Curculionidae;Polygraphus;Polygraphus poligraphus;Sweden;GenBank;NA;KC845496
Coleoptera;Curculionidae;Polygraphus;Polygraphus poligraphus;Norway;GenBank;NA;KC845497
Coleoptera;Curculionidae;Polygraphus;Polygraphus poligraphus;Estonia;GenBank;NA;KC845498
Coleoptera;Curculionidae;Polygraphus;Polygraphus poligraphus;Hungary;GenBank;NA;KC845499
Coleoptera;Curculionidae;Scolytus;Scolytus rugulosus;Hungary;GenBank;NA;KC845508
Coleoptera;Curculionidae;Scolytus;Scolytus pygmaeus;Denmark;GenBank;NA;KC845509
Coleoptera;Curculionidae;Scolytus;Scolytus pygmaeus;Czech Republic;GenBank;NA;KC845510
Coleoptera;Curculionidae;Scolytus;Scolytus multistriatus;Denmark;GenBank;NA;KC845512
Coleoptera;Curculionidae;Scolytus;Scolytus carpini;Czech Republic;GenBank;NA;KC845513
Coleoptera;Curculionidae;Scolytus;Scolytus ensifer;Czech Republic;GenBank;NA;KC845514
Coleoptera;Curculionidae;Tomicus;Tomicus piniperda;Estonia;GenBank;NA;KC845515
Coleoptera;Curculionidae;Trypodendron;Trypodendron lineatum;Norway;GenBank;NA;KC845516
Coleoptera;Curculionidae;Trypodendron;Trypodendron domesticum;Germany;GenBank;NA;KC845517
Coleoptera;Curculionidae;Trypodendron;Trypodendron signatum;Germany;GenBank;NA;KC845518
Coleoptera;Curculionidae;Xyleborinus;Xyleborinus saxesenii;Czech Republic;GenBank;NA;KC845519
Coleoptera;Curculionidae;Xyleborus;Xyleborus monographus;Germany;GenBank;NA;KC845522
Coleoptera;Curculionidae;Xyleborus;Xyleborus monographus;Czech Republic;GenBank;NA;KC845523
Coleoptera;Laemophloeidae;Cryptolestes;Cryptolestes ferrugineus;Czech Republic;GenBank;NA;KC977914
Coleoptera;Curculionidae;Platypus;Platypus cylindrus;Portugal;GenBank;NA;KP297972
Coleoptera;Curculionidae;Platypus;Platypus cylindrus;Portugal;GenBank;NA;KP297973
Coleoptera;Curculionidae;Platypus;Platypus cylindrus;Portugal;GenBank;NA;KP297974
Coleoptera;Cerambycidae;Trichoferus;Trichoferus holosericeus;France;GenBank;NA;KU531396
Coleoptera;Cerambycidae;Rosalia;Rosalia alpina;Italy;GenBank;NA;KX241486
Coleoptera;Lucanidae;Lucanus;Lucanus cervus;Italy;GenBank;NA;KX241489
Coleoptera;Elateridae;Melanotus;Melanotus castanipes;Poland;GenBank;NA;KY040259
Coleoptera;Elateridae;Melanotus;Melanotus castanipes;Poland;GenBank;NA;KY040260
Coleoptera;Elateridae;Melanotus;Melanotus villosus;Poland;GenBank;NA;KY040261
Coleoptera;Elateridae;Melanotus;Melanotus villosus;Poland;GenBank;NA;KY040262
Coleoptera;Elateridae;Melanotus;Melanotus villosus;Poland;GenBank;NA;KY040263
Coleoptera;Elateridae;Melanotus;Melanotus villosus;Poland;GenBank;NA;KY040264
Coleoptera;Buprestidae;Meliboeus;Meliboeus fulgidicollis;Slovakia;Bold;GBCL28309-19;MF286145
Coleoptera;Buprestidae;Agrilus;Agrilus roscidus;Slovakia;GenBank;NA;MF286229
Coleoptera;Buprestidae;Agrilus;Agrilus salicis;Slovakia;GenBank;NA;MF286232
Coleoptera;Buprestidae;Agrilus;Agrilus viridis viridis;Slovakia;GenBank;NA;MF286233
Coleoptera;Buprestidae;Agrilus;Agrilus viridis viridis;Slovakia;GenBank;NA;MF286234
Coleoptera;Buprestidae;Agrilus;Agrilus viridis viridis;Slovakia;GenBank;NA;MF286235
Coleoptera;Buprestidae;Agrilus;Agrilus viridis viridis;Slovakia;GenBank;NA;MF286236
Coleoptera;Buprestidae;Agrilus;Agrilus viridis viridis;Slovakia;GenBank;NA;MF286237
Coleoptera;Buprestidae;Agrilus;Agrilus viridis viridis;Slovakia;GenBank;NA;MF286238
Coleoptera;Buprestidae;Agrilus;Agrilus auricollis auricollis;Hungary;GenBank;NA;MF286242
Coleoptera;Buprestidae;Agrilus;Agrilus auricollis auricollis;Hungary;GenBank;NA;MF286243
Coleoptera;Buprestidae;Agrilus;Agrilus auricollis auricollis;Hungary;GenBank;NA;MF286244
Coleoptera;Buprestidae;Agrilus;Agrilus salicis;Slovakia;GenBank;NA;MF286253
Coleoptera;Buprestidae;Agrilus;Agrilus viridis viridis;Hungary;GenBank;NA;MF286256
Coleoptera;Buprestidae;Agrilus;Agrilus viridis viridis;Slovakia;GenBank;NA;MF286257
Coleoptera;Buprestidae;Agrilus;Agrilus viridis viridis;Slovakia;GenBank;NA;MF286258
Coleoptera;Buprestidae;Agrilus;Agrilus viridis viridis;Slovakia;GenBank;NA;MF286259
Coleoptera;Buprestidae;Agrilus;Agrilus viridis viridis;Slovakia;GenBank;NA;MF286260
Coleoptera;Buprestidae;Agrilus;Agrilus viridis viridis;Slovakia;GenBank;NA;MF286261
Coleoptera;Buprestidae;Agrilus;Agrilus viridis viridis;Slovakia;GenBank;NA;MF286262
Coleoptera;Buprestidae;Agrilus;Agrilus antiquus croaticus;Slovakia;GenBank;NA;MF286267

Coleoptera;Buprestidae;Agrilus;Agrilus antiquus croaticus;Slovakia;GenBank;NA;MF286268
Coleoptera;Buprestidae;Agrilus;Agrilus angustulus angustulus;Slovakia;GenBank;NA;MF286273
Coleoptera;Buprestidae;Agrilus;Agrilus angustulus angustulus;Slovakia;GenBank;NA;MF286274
Coleoptera;Buprestidae;Agrilus;Agrilus angustulus angustulus;Slovakia;GenBank;NA;MF286275
Coleoptera;Buprestidae;Agrilus;Agrilus angustulus angustulus;Slovakia;GenBank;NA;MF286276
Coleoptera;Buprestidae;Agrilus;Agrilus derasofasciatus;Slovakia;GenBank;NA;MF286277
Coleoptera;Buprestidae;Agrilus;Agrilus derasofasciatus;Slovakia;GenBank;NA;MF286278
Coleoptera;Buprestidae;Agrilus;Agrilus graminis graminis;Slovakia;GenBank;NA;MF286279
Coleoptera;Buprestidae;Agrilus;Agrilus graminis graminis;Slovakia;GenBank;NA;MF286280
Coleoptera;Buprestidae;Agrilus;Agrilus hastulifer hastulifer;Slovakia;GenBank;NA;MF286281
Coleoptera;Buprestidae;Agrilus;Agrilus hastulifer hastulifer;Slovakia;GenBank;NA;MF286282
Coleoptera;Buprestidae;Agrilus;Agrilus laticornis;Slovakia;GenBank;NA;MF286283
Coleoptera;Buprestidae;Agrilus;Agrilus obscuricollis;Slovakia;GenBank;NA;MF286284
Coleoptera;Buprestidae;Agrilus;Agrilus obscuricollis;Slovakia;GenBank;NA;MF286285
Coleoptera;Buprestidae;Agrilus;Agrilus obscuricollis;Slovakia;GenBank;NA;MF286286
Coleoptera;Buprestidae;Agrilus;Agrilus olivicolor;Slovakia;GenBank;NA;MF286287
Coleoptera;Buprestidae;Agrilus;Agrilus sulcicollis sulcicollis;Slovakia;GenBank;NA;MF286288
Coleoptera;Buprestidae;Agrilus;Agrilus biguttatus;Slovakia;GenBank;NA;MF286289
Coleoptera;Buprestidae;Agrilus;Agrilus biguttatus;Slovakia;GenBank;NA;MF286290
Coleoptera;Buprestidae;Agrilus;Agrilus guerini;Slovakia;GenBank;NA;MF286291
Coleoptera;Buprestidae;Agrilus;Agrilus pratensis pratensis;Slovakia;GenBank;NA;MF286294
Coleoptera;Buprestidae;Agrilus;Agrilus pratensis pratensis;Slovakia;GenBank;NA;MF286295
Coleoptera;Buprestidae;Agrilus;Agrilus convexicollis;Slovakia;GenBank;NA;MF286296
Coleoptera;Buprestidae;Agrilus;Agrilus convexicollis;Slovakia;GenBank;NA;MF286297
Coleoptera;Buprestidae;Agrilus;Agrilus convexicollis;Slovakia;GenBank;NA;MF286298
Coleoptera;Buprestidae;Agrilus;Agrilus convexicollis;Slovakia;GenBank;NA;MF286299
Coleoptera;Buprestidae;Agrilus;Agrilus cyanescens;Slovakia;GenBank;NA;MF286300
Coleoptera;Buprestidae;Agrilus;Agrilus cyanescens;Slovakia;GenBank;NA;MF286301
Coleoptera;Buprestidae;Agrilus;Agrilus cyanescens;Slovakia;GenBank;NA;MF286302
Coleoptera;Buprestidae;Meliboeus;Meliboeus fulgidicollis;Slovakia;Bold;GBCL28310-19;MF286310
Coleoptera;Buprestidae;Agrilus;Agrilus elegans;Spain;GenBank;NA;MF286312
Coleoptera;Buprestidae;Agrilus;Agrilus viridis viridis;Slovakia;GenBank;NA;MF286316
Coleoptera;Buprestidae;Agrilus;Agrilus suvorovi;Slovakia;GenBank;NA;MF286318
Coleoptera;Buprestidae;Agrilus;Agrilus suvorovi;Slovakia;GenBank;NA;MF286319
Coleoptera;Buprestidae;Agrilus;Agrilus viridis viridis;Slovakia;GenBank;NA;MF286320
Coleoptera;Buprestidae;Agrilus;Agrilus viridis viridis;Slovakia;GenBank;NA;MF286321
Coleoptera;Buprestidae;Agrilus;Agrilus sulcicollis sulcicollis;Slovakia;GenBank;NA;MF286324
Coleoptera;Buprestidae;Agrilus;Agrilus sulcicollis sulcicollis;Slovakia;GenBank;NA;MF286325
Coleoptera;Buprestidae;Agrilus;Agrilus viridis viridis;Slovakia;GenBank;NA;MF286326
Coleoptera;Buprestidae;Agrilus;Agrilus biguttatus;Slovakia;GenBank;NA;MF286328
Coleoptera;Buprestidae;Agrilus;Agrilus biguttatus;Slovakia;GenBank;NA;MF286329
Coleoptera;Buprestidae;Agrilus;Agrilus viridis viridis;Czech Republic;GenBank;NA;MF286330
Coleoptera;Buprestidae;Agrilus;Agrilus viridis viridis;Czech Republic;GenBank;NA;MF286331
Coleoptera;Curculionidae;Trypodendron;Trypodendron lineatum;Slovenia;GenBank;NA;MF373743
Coleoptera;Buprestidae;Agrilus;Agrilus viridis;Italy;GenBank;NA;MF543033
Coleoptera;Buprestidae;Agrilus;Agrilus viridis;Italy;GenBank;NA;MF543034
Coleoptera;Buprestidae;Agrilus;Agrilus viridis;Italy;GenBank;NA;MF543035
Coleoptera;Buprestidae;Agrilus;Agrilus viridis;Italy;GenBank;NA;MF543036
Coleoptera;Buprestidae;Agrilus;Agrilus viridis;Italy;GenBank;NA;MF543037
Coleoptera;Curculionidae;Xyleborus;Xyleborus monographus;Portugal;GenBank;NA;MF611985
Coleoptera;Curculionidae;Xyleborus;Xyleborus monographus;Portugal;GenBank;NA;MF611986
Coleoptera;Curculionidae;Xyleborus;Xyleborus monographus;Portugal;GenBank;NA;MF611987
Coleoptera;Curculionidae;Xyleborus;Xyleborus monographus;Portugal;GenBank;NA;MF611988
Coleoptera;Curculionidae;Xyleborus;Xyleborus monographus;Portugal;GenBank;NA;MF611989
Coleoptera;Curculionidae;Xyleborus;Xyleborus monographus;Portugal;GenBank;NA;MF611990
Coleoptera;Curculionidae;Xyleborus;Xyleborus monographus;Portugal;GenBank;NA;MF611991
Coleoptera;Curculionidae;Xyleborus;Xyleborus monographus;Portugal;GenBank;NA;MF611992
Coleoptera;Curculionidae;Xyleborus;Xyleborus monographus;Portugal;GenBank;NA;MF611993
Coleoptera;Curculionidae;Xyleborinus;Xyleborinus saxesenii;Portugal;GenBank;NA;MF611994
Coleoptera;Curculionidae;Xyleborinus;Xyleborinus saxesenii;Portugal;GenBank;NA;MF611995
Coleoptera;Curculionidae;Xyleborinus;Xyleborinus saxesenii;Portugal;GenBank;NA;MF611996
Coleoptera;Curculionidae;Xyleborinus;Xyleborinus saxesenii;Portugal;GenBank;NA;MF611997
Coleoptera;Curculionidae;Xyleborinus;Xyleborinus saxesenii;Portugal;GenBank;NA;MF611998
Coleoptera;Curculionidae;Xyleborus;Xyleborus dryographus;Portugal;GenBank;NA;MF611999
Coleoptera;Curculionidae;Xyleborus;Xyleborus dryographus;Portugal;GenBank;NA;MF612000
Coleoptera;Curculionidae;Xyleborus;Xyleborus dryographus;Portugal;GenBank;NA;MF612001
Coleoptera;Curculionidae;Xyleborus;Xyleborus dryographus;Portugal;GenBank;NA;MF612002
Coleoptera;Scarabaeidae;Cetonia;Cetonia aurata;Russia;GenBank;NA;MF706407
Coleoptera;Scarabaeidae;Cetonia;Cetonia aurata;Russia;GenBank;NA;MF706408
Coleoptera;Lucanidae;Dorcus;Dorcus parallelipipedus;Russia;GenBank;NA;MF706411
Coleoptera;Buprestidae;Chalcophora;Chalcophora mariana;Russia;GenBank;NA;MF706412
Coleoptera;Scarabaeidae;Oryctes;Oryctes nasicornis;Russia;GenBank;NA;MF706415
Coleoptera;Cerambycidae;Spondylis;Spondylis buprestoides;Russia;GenBank;NA;MF706420
Coleoptera;Lycidae;Lygistopterus;Lygistopterus sanguineus;Russia;GenBank;NA;MF706421
Coleoptera;Cerambycidae;Prionus;Prionus coriarius;Russia;GenBank;NA;MF706433
Coleoptera;Cerambycidae;Stenurella;Stenurella bifasciata;Russia;GenBank;NA;MF706444
Coleoptera;Scarabaeidae;Trichius;Trichius fasciatus;Russia;GenBank;NA;MF706446
Coleoptera;Lucanidae;Lucanus;Lucanus cervus;Russia;GenBank;NA;MF706447

Coleoptera;Scraptiidae;Anaspis;Anaspis frontalis;Poland;GenBank;NA;MH020275
Coleoptera;Scraptiidae;Anaspis;Anaspis frontalis;Poland;GenBank;NA;MH020276
Coleoptera;Scraptiidae;Anaspis;Anaspis frontalis;Poland;GenBank;NA;MH020277
Coleoptera;Melandryidae;Serropalpus;Serropalpus barbatus;Poland;GenBank;NA;MH020281
Coleoptera;Cerambycidae;Prionus;Prionus coriarius;Poland;GenBank;NA;MH020282
Coleoptera;Cerambycidae;Prionus;Prionus coriarius;Poland;GenBank;NA;MH020283
Coleoptera;Cerambycidae;Pachyta;Pachyta quadrimaculata;Poland;GenBank;NA;MH020284
Coleoptera;Cerambycidae;Pachyta;Pachyta quadrimaculata;Poland;GenBank;NA;MH020285
Coleoptera;Cleridae;Clerus;Clerus mutillarius;Poland;GenBank;NA;MH020286
Coleoptera;Cleridae;Clerus;Clerus mutillarius;Poland;GenBank;NA;MH020287
Coleoptera;Cleridae;Thanasimus;Thanasimus formicarius;Austria;GenBank;NA;MH020288
Coleoptera;Cleridae;Thanasimus;Thanasimus formicarius;Poland;GenBank;NA;MH020289
Coleoptera;Cleridae;Thanasimus;Thanasimus formicarius;Poland;GenBank;NA;MH020290
Coleoptera;Cerambycidae;Pidonia;Pidonia lurida;Poland;GenBank;NA;MH020291
Coleoptera;Cerambycidae;Pidonia;Pidonia lurida;Poland;GenBank;NA;MH020292
Coleoptera;Cerambycidae;Pidonia;Pidonia lurida;Poland;GenBank;NA;MH020293
Coleoptera;Mycetophagidae;Mycetophagus;Mycetophagus multipunctatus;Poland;GenBank;NA;MH020296
Coleoptera;Mycetophagidae;Mycetophagus;Mycetophagus quadripustulatus;Poland;GenBank;NA;MH020298
Coleoptera;Mycetophagidae;Mycetophagus;Mycetophagus quadripustulatus;Poland;GenBank;NA;MH020299
Coleoptera;Mycetophagidae;Mycetophagus;Mycetophagus quadripustulatus;Poland;GenBank;NA;MH020300
Coleoptera;Mycetophagidae;Mycetophagus;Mycetophagus piceus;Poland;GenBank;NA;MH020301
Coleoptera;Zopheridae;Bitoma;Bitoma crenata;Poland;GenBank;NA;MH020302
Coleoptera;Zopheridae;Bitoma;Bitoma crenata;Poland;GenBank;NA;MH020303
Coleoptera;Cerambycidae;Rhagium;Rhagium sycophanta;Austria;GenBank;NA;MH020317
Coleoptera;Cerambycidae;Rhagium;Rhagium sycophanta;Poland;GenBank;NA;MH020318
Coleoptera;Cerambycidae;Rhagium;Rhagium inquisitor;Poland;GenBank;NA;MH020319
Coleoptera;Cerambycidae;Rhagium;Rhagium inquisitor;Poland;GenBank;NA;MH020320
Coleoptera;Cerambycidae;Rhagium;Rhagium mordax;Poland;GenBank;NA;MH020321
Coleoptera;Melyridae;Dasytes;Dasytes plumbeus;Poland;GenBank;NA;MH020322
Coleoptera;Melyridae;Dasytes;Dasytes plumbeus;Poland;GenBank;NA;MH020323
Coleoptera;Cerambycidae;Strangalia;Strangalia attenuata;Poland;GenBank;NA;MH020328
Coleoptera;Cerambycidae;Strangalia;Strangalia attenuata;Poland;GenBank;NA;MH020329
Coleoptera;Cerambycidae;Oxymirus;Oxymirus cursor;Poland;GenBank;NA;MH020332
Coleoptera;Oedemeridae;Chrysanthia;Chrysanthia geniculata;Poland;GenBank;NA;MH020339
Coleoptera;Oedemeridae;Chrysanthia;Chrysanthia geniculata;Poland;GenBank;NA;MH020340
Coleoptera;Oedemeridae;Chrysanthia;Chrysanthia geniculata;Poland;GenBank;NA;MH020341
Coleoptera;Cerambycidae;Rutpela;Rutpela maculata;Poland;GenBank;NA;MH020342
Coleoptera;Cerambycidae;Rutpela;Rutpela maculata;Poland;GenBank;NA;MH020343
Coleoptera;Cerambycidae;Stenurella;Stenurella nigra;Poland;GenBank;NA;MH020344
Coleoptera;Cerambycidae;Anoplodera;Anoplodera sexguttata;Poland;GenBank;NA;MH020345
Coleoptera;Cerambycidae;Anoplodera;Anoplodera sexguttata;Poland;GenBank;NA;MH020346
Coleoptera;Cerambycidae;Anastrangalia;Anastrangalia reyi;Poland;GenBank;NA;MH020347
Coleoptera;Cerambycidae;Anastrangalia;Anastrangalia reyi;Poland;GenBank;NA;MH020348
Coleoptera;Cerambycidae;Anastrangalia;Anastrangalia sanguinolenta;Poland;GenBank;NA;MH020349
Coleoptera;Cerambycidae;Anastrangalia;Anastrangalia sanguinolenta;Poland;GenBank;NA;MH020350
Coleoptera;Cerambycidae;Alosterna;Alosterna tabacicolor;Poland;GenBank;NA;MH020351
Coleoptera;Cerambycidae;Alosterna;Alosterna tabacicolor;Poland;GenBank;NA;MH020352
Coleoptera;Pyrochroidae;Pyrochroa;Pyrochroa coccinea;Poland;GenBank;NA;MH020353
Coleoptera;Pyrochroidae;Pyrochroa;Pyrochroa coccinea;Poland;GenBank;NA;MH020354
Coleoptera;Pyrochroidae;Pyrochroa;Pyrochroa coccinea;Poland;GenBank;NA;MH020355
Coleoptera;Pyrochroidae;Pyrochroa;Pyrochroa coccinea;Poland;GenBank;NA;MH020356
Coleoptera;Pyrochroidae;Schizotus;Schizotus pectinicornis;Poland;GenBank;NA;MH020357
Coleoptera;Pyrochroidae;Schizotus;Schizotus pectinicornis;Poland;GenBank;NA;MH020358
Coleoptera;Pyrochroidae;Schizotus;Schizotus pectinicornis;Poland;GenBank;NA;MH020359
Coleoptera;Pyrochroidae;Schizotus;Schizotus pectinicornis;Poland;GenBank;NA;MH020360
Coleoptera;Cerambycidae;Arhopalus;Arhopalus rusticus;Poland;GenBank;NA;MH020361
Coleoptera;Cerambycidae;Arhopalus;Arhopalus rusticus;Poland;GenBank;NA;MH020362
Coleoptera;Mycetophagidae;Litargus;Litargus connexus;Poland;GenBank;NA;MH020363
Coleoptera;Scarabaeidae;Valgus;Valgus hemipterus;Bulgaria;GenBank;NA;MH020440
Coleoptera;Scarabaeidae;Valgus;Valgus hemipterus;Bulgaria;GenBank;NA;MH020441
Coleoptera;Scarabaeidae;Cetonia;Cetonia aurata;Poland;GenBank;NA;MH020442
Coleoptera;Pythidae;Pytho;Pytho depressus;Poland;GenBank;NA;MH020446
Coleoptera;Pythidae;Pytho;Pytho depressus;Poland;GenBank;NA;MH020447
Coleoptera;Cerambycidae;Pseudovadonia;Pseudovadonia livida;Poland;GenBank;NA;MH020448
Coleoptera;Tenebrionidae;Diaperis;Diaperis boleti;Poland;GenBank;NA;MH020449
Coleoptera;Sphindidae;Sphindus;Sphindus dubius;Poland;GenBank;NA;MH020450
Coleoptera;Sphindidae;Sphindus;Sphindus dubius;Poland;GenBank;NA;MH020451
Coleoptera;Cerambycidae;Rusticoclytus;Rusticoclytus rusticus;Poland;GenBank;NA;MH020452
Coleoptera;Cerambycidae;Rusticoclytus;Rusticoclytus rusticus;Poland;GenBank;NA;MH020454
Coleoptera;Cerambycidae;Hylotrupes;Hylotrupes bajulus;Poland;GenBank;NA;MH020455
Coleoptera;Cerambycidae;Hylotrupes;Hylotrupes bajulus;Poland;GenBank;NA;MH020456
Coleoptera;Cerambycidae;Stenurella;Stenurella melanura;Poland;GenBank;NA;MH020457
Coleoptera;Cerambycidae;Stenurella;Stenurella melanura;Poland;GenBank;NA;MH020458
Coleoptera;Cerambycidae;Stenurella;Stenurella melanura;Poland;GenBank;NA;MH020459
Coleoptera;Salpingidae;Salpingus;Salpingus ruficollis;Poland;GenBank;NA;MH020475
Coleoptera;Cerambycidae;Aromia;Aromia moschata;Poland;GenBank;NA;MH020476
Coleoptera;Cerambycidae;Aromia;Aromia moschata;Poland;GenBank;NA;MH020477
Coleoptera;Cerambycidae;Aromia;Aromia moschata;Poland;GenBank;NA;MH020478

Coleoptera;Cerambycidae;Phymatodes;Phymatodes testaceus;Poland;GenBank;NA;MH020479
Coleoptera;Cerambycidae;Phymatodes;Phymatodes testaceus;Poland;GenBank;NA;MH020480
Coleoptera;Erotylidae;Dacne;Dacne bipustulata;Poland;GenBank;NA;MH020481
Coleoptera;Erotylidae;Dacne;Dacne rufifrons;Bulgaria;GenBank;NA;MH020482
Coleoptera;Erotylidae;Dacne;Dacne rufifrons;Bulgaria;GenBank;NA;MH020483
Coleoptera;Erotylidae;Dacne;Dacne rufifrons;Bulgaria;GenBank;NA;MH020484
Coleoptera;Cerambycidae;Paracorymbia;Paracorymbia maculicornis;Poland;GenBank;NA;MH020520
Coleoptera;Cerambycidae;Paracorymbia;Paracorymbia maculicornis;Poland;GenBank;NA;MH020521
Coleoptera;Curculionidae;Hylobius;Hylobius abietis;Bulgaria;GenBank;NA;MH115471
Coleoptera;Curculionidae;Ips;Ips typographus;Poland;GenBank;NA;MH115481
Coleoptera;Cerambycidae;Dinoptera;Dinoptera collaris;Poland;GenBank;NA;MH115482
Coleoptera;Curculionidae;Dryocoetes;Dryocoetes autographus;Poland;GenBank;NA;MH115483
Coleoptera;Curculionidae;Dryocoetes;Dryocoetes autographus;Poland;GenBank;NA;MH115484
Coleoptera;Curculionidae;Pityogenes;Pityogenes chalcographus;Slovakia;GenBank;NA;MH115485
Coleoptera;Erotylidae;Triplax;Triplax russica;Poland;GenBank;NA;MH115489
Coleoptera;Erotylidae;Triplax;Triplax aenea;Slovakia;GenBank;NA;MH115490
Coleoptera;Erotylidae;Combocerus;Combocerus glaber;Bulgaria;GenBank;NA;MH115491
Coleoptera;Erotylidae;Tritoma;Tritoma bipustulata;Poland;GenBank;NA;MH115492
Coleoptera;Erotylidae;Tritoma;Tritoma bipustulata;Poland;GenBank;NA;MH115493
Coleoptera;Buprestidae;Agrilus;Agrilus derasofasciatus;Poland;GenBank;NA;MH115496
Coleoptera;Cerambycidae;Spondylis;Spondylis buprestoides;Poland;GenBank;NA;MH115501
Coleoptera;Lycidae;Lygistopterus;Lygistopterus sanguineus;Poland;GenBank;NA;MH115527
Coleoptera;Lycidae;Lygistopterus;Lygistopterus sanguineus;Poland;GenBank;NA;MH115528
Coleoptera;Lucanidae;Dorcus;Dorcus parallelipipedus;Poland;GenBank;NA;MH115537
Coleoptera;Lucanidae;Dorcus;Dorcus parallelipipedus;Poland;GenBank;NA;MH115538
Coleoptera;Cerambycidae;Plagionotus;Plagionotus detritus;Poland;GenBank;NA;MH115547
Coleoptera;Buprestidae;Agrilus;Agrilus suvorovi;Poland;GenBank;NA;MH115551
Coleoptera;Salpingidae;Salpingus;Salpingus ruficollis;Poland;GenBank;NA;MH115559
Coleoptera;Buprestidae;Anthaxia;Anthaxia nitidula;Poland;GenBank;NA;MH115561
Coleoptera;Cerambycidae;Tetropium;Tetropium fuscum;Poland;GenBank;NA;MH115562
Coleoptera;Cerambycidae;Tetropium;Tetropium fuscum;Poland;GenBank;NA;MH115563
Coleoptera;Buprestidae;Bostrichus;Bostrichus capucinus;Austria;GenBank;NA;MH115570
Coleoptera;Cerambycidae;Dinoptera;Dinoptera collaris;Poland;GenBank;NA;MH115573
Coleoptera;Curculionidae;Trypodendron;Trypodendron lineatum;Poland;GenBank;NA;MH115574
Coleoptera;Cerambycidae;Obrium;Obrium brunneum;Poland;GenBank;NA;MH115575
Coleoptera;Curculionidae;Platypus;Platypus cylindrus;Czech Republic;GenBank;NA;MW000661
Coleoptera;Ptinidae;Dryophilus;Dryophilus pusillus;Slovakia;GenBank;NA;MW441347
Coleoptera;Ptinidae;Ernobius;Ernobius pini;Switzerland;GenBank;NA;MW441351
Coleoptera;Ripiphoridae;Pelecotoma;Pelecotoma fennica;Finland;GenBank;NA;MZ632802
Coleoptera;Curculionidae;Hylastes;Hylastes brunneus;United Kingdom;Bold;GBCL53656-19;NC_036262
Coleoptera;Monotomidae;Monotoma;Monotoma quadricollis;United Kingdom;Bold;GBCL43113-19;NC_036266
Coleoptera;Silvanidae;Silvanus;Silvanus bidentatus;United Kingdom;Bold;GBCL44994-19;NC_036273
Coleoptera;Salpingidae;Vincenzellus;Vincenzellus ruficollis;United Kingdom;Bold;GBCL44391-19;NC_036274
Coleoptera;Tetratomidae;Tetratoma;Tetratoma fungorum;United Kingdom;Bold;GBCL45028-19;NC_036276
Coleoptera;Ripiphoridae;Pelecotoma;Pelecotoma fennica;Czech Republic;Bold;GBCL44346-19;NC_036277
Coleoptera;Curculionidae;Ips;Ips sexdentatus;United Kingdom;Bold;GBCL53707-19;NC_036281
Coleoptera;Curculionidae;Dryocoetes;Dryocoetes villosus;United Kingdom;Bold;GBCL53527-19;NC_036282
Coleoptera;Curculionidae;Trypodendron;Trypodendron domesticum;United Kingdom;Bold;GBCL54041-19;NC_036286
Coleoptera;Curculionidae;Dryocoetes;Dryocoetes autographus;United Kingdom;Bold;GBCL53526-19;NC_036287
Coleoptera;Curculionidae;Pityophthorus;Pityophthorus pubescens;United Kingdom;Bold;GBCL53888-19;NC_036288
Coleoptera;Curculionidae;Pityogenes;Pityogenes bidentatus;United Kingdom;Bold;GBCL53883-19;NC_036289
Coleoptera;Curculionidae;Hylastes;Hylastes attenuatus;United Kingdom;Bold;GBCL53655-19;NC_036290
Coleoptera;Curculionidae;Orthotomicus;Orthotomicus laricis;United Kingdom;Bold;GBCL53823-19;NC_036291
Coleoptera;Curculionidae;Trypodendron;Trypodendron signatum;United Kingdom;Bold;GBCL54048-19;NC_036292
Coleoptera;Curculionidae;Anisandrus;Anisandrus dispar;United Kingdom;Bold;GBCL53332-19;NC_036293
Coleoptera;Curculionidae;Gnathotrichus;Gnathotrichus materiarius;United Kingdom;Bold;GBCL53586-19;NC_036294
Coleoptera;Elateridae;Ampedus;Ampedus nemoralis;France;Bold;PSFOR527-13;NA
Coleoptera;Elateridae;Ampedus;Ampedus scrofa;France;Bold;PSFOR523-13;NA
Coleoptera;Buprestidae;Anthaxia;Anthaxia hungarica;Greece;Bold;JSBIC178-18;NA
Coleoptera;Curculionidae;Dryocoetes;Dryocoetes villosus;Norway;Bold;NOCLP1785-19;NA
Coleoptera;Cerambycidae;Ergates;Ergates faber;France;Bold;SICOC487-18;NA
Coleoptera;Cerambycidae;Prinobius;Prinobius myardi;Greece;Bold;CERAF133-08;NA
Coleoptera;Cerambycidae;Prinobius;Prinobius myardi;Macedonia;Bold;CERAF088-07;NA
Coleoptera;Cerambycidae;Prinobius;Prinobius myardi;Greece;Bold;CERAF132-08;NA
Coleoptera;Cerambycidae;Prinobius;Prinobius myardi;Macedonia;Bold;CERAF087-07;NA
;;;;;;;

Appendix_3_Data_without_Sequences;;;;;;;
order_name;family_name;genus_name;species_name;country;sourceDatabase;processid;genbank_accession
Hymenoptera;Andrenidae;Andrena;Andrena argentata;Germany;Bold;GBACU1079-12;-
Hymenoptera;Andrenidae;Andrena;Andrena argentata;Germany;Bold;GBACU3075-13;-
Hymenoptera;Andrenidae;Andrena;Andrena argentata;Germany;Bold;FBHAP872-09;-
Hymenoptera;Andrenidae;Andrena;Andrena argentata;Germany;Bold;GBACU343-12;-
Hymenoptera;Andrenidae;Andrena;Andrena argentata;Germany;Bold;GBACU1078-12;-
Hymenoptera;Andrenidae;Andrena;Andrena argentata;Germany;Bold;FBAPC679-11;-
Hymenoptera;Andrenidae;Andrena;Andrena argentata;Germany;Bold;GBACU1076-12;-
Hymenoptera;Andrenidae;Andrena;Andrena argentata;Czech Republic;Bold;GBACU1349-12;-
Hymenoptera;Andrenidae;Andrena;Andrena argentata;Norway;Bold;NOBEE013-09;-
Hymenoptera;Andrenidae;Andrena;Andrena argentata;Germany;Bold;GBACU1075-12;-
Hymenoptera;Andrenidae;Andrena;Andrena argentata;Germany;Bold;GBACU1077-12;-
Hymenoptera;Andrenidae;Andrena;Andrena argentata;Norway;Bold;NOBEE012-09;-
Hymenoptera;Andrenidae;Andrena;Andrena curvana;Germany;Bold;BCHYM2470-14;-
Hymenoptera;Andrenidae;Andrena;Andrena hattorfiana;Germany;Bold;FBAPC039-10;-
Hymenoptera;Andrenidae;Andrena;Andrena hattorfiana;Germany;Bold;FBHAP958-09;-
Hymenoptera;Andrenidae;Andrena;Andrena hattorfiana;Germany;Bold;FBAPC037-10;-
Hymenoptera;Andrenidae;Andrena;Andrena hattorfiana;Italy;Bold;GBLGC043-12;-
Hymenoptera;Andrenidae;Andrena;Andrena hattorfiana;Germany;Bold;FBHAP956-09;-
Hymenoptera;Andrenidae;Andrena;Andrena ovatula;Spain;Bold;HYMAA313-22;-
Hymenoptera;Andrenidae;Andrena;Andrena ovatula;Germany;Bold;GBACU2347-13;-
Hymenoptera;Andrenidae;Andrena;Andrena ovatula;Germany;Bold;GBACU2388-13;-
Hymenoptera;Andrenidae;Andrena;Andrena ovatula;Belgium;Bold;HYMAA316-22;-
Hymenoptera;Andrenidae;Andrena;Andrena ovatula;Germany;Bold;FBHAP546-09;-
Hymenoptera;Andrenidae;Andrena;Andrena ovatula;Germany;Bold;GBACU2386-13;-
Hymenoptera;Andrenidae;Andrena;Andrena ovatula;Portugal;Bold;HYMAA314-22;-
Hymenoptera;Andrenidae;Andrena;Andrena ovatula;Germany;Bold;FBAPD257-11;-
Hymenoptera;Andrenidae;Andrena;Andrena ovatula;Germany;Bold;FBAPD255-11;-
Hymenoptera;Andrenidae;Andrena;Andrena ovatula;Germany;Bold;FBAPD233-11;-
Hymenoptera;Andrenidae;Andrena;Andrena ovatula;Austria;Bold;TDAAT343-19;-
Hymenoptera;Andrenidae;Andrena;Andrena ovatula;Germany;Bold;FBAPD254-11;-
Hymenoptera;Andrenidae;Andrena;Andrena ovatula;Germany;Bold;GBACU2298-13;-
Hymenoptera;Andrenidae;Andrena;Andrena ovatula;Germany;Bold;GBACU2341-13;-
Hymenoptera;Andrenidae;Andrena;Andrena ovatula;Germany;Bold;FBAPD256-11;-
Hymenoptera;Andrenidae;Andrena;Andrena ovatula;Germany;Bold;FBAPC074-10;-
Hymenoptera;Andrenidae;Andrena;Andrena ovatula;Germany;Bold;FBAPD259-11;-
Hymenoptera;Andrenidae;Andrena;Andrena ovatula;Germany;Bold;GBACU2387-13;-
Hymenoptera;Andrenidae;Andrena;Andrena ovatula;Germany;Bold;FBHAP545-09;-
Hymenoptera;Andrenidae;Andrena;Andrena ovatula;Germany;Bold;FBAPC073-10;-
Hymenoptera;Andrenidae;Andrena;Andrena ovatula;Germany;Bold;BCHYM4595-14;-
Hymenoptera;Andrenidae;Andrena;Andrena ovatula;Germany;Bold;GBACU2390-13;-
Hymenoptera;Andrenidae;Andrena;Andrena ovatula;Germany;Bold;FBAPD260-11;-
Hymenoptera;Andrenidae;Andrena;Andrena ovatula;Germany;Bold;FBAPD258-11;-
Hymenoptera;Andrenidae;Andrena;Andrena ovatula;Germany;Bold;FBHAP547-09;-
Hymenoptera;Andrenidae;Andrena;Andrena ovatula;Germany;Bold;GBACU2389-13;-
Hymenoptera;Andrenidae;Andrena;Andrena suerinensis;Germany;Bold;FBAPC091-10;-
Hymenoptera;Andrenidae;Andrena;Andrena suerinensis;Germany;Bold;FBAPC090-10;-
Hymenoptera;Megachilidae;Anthidium;Anthidium montanum;Germany;Bold;FBAPB453-09;-
Hymenoptera;Megachilidae;Anthidium;Anthidium montanum;Germany;Bold;FBAPB450-09;-
Hymenoptera;Megachilidae;Anthidium;Anthidium montanum;Switzerland;Bold;FBAPB451-09;-
Hymenoptera;Apidae;Bombus;Bombus alpinus;Italy;Bold;FBAPB991-09;-
Hymenoptera;Apidae;Bombus;Bombus confusus;Germany;Bold;FBHAP773-09;-
Hymenoptera;Apidae;Bombus;Bombus gerstaeckeri;Germany;Bold;FBHAP778-09;-
Hymenoptera;Apidae;Bombus;Bombus monticola;Germany;Bold;FBAPC810-11;-
Hymenoptera;Apidae;Bombus;Bombus monticola;Germany;Bold;FBHAP810-09;-
Hymenoptera;Apidae;Bombus;Bombus mucidus;Germany;Bold;FBHAP812-09;-
Hymenoptera;Apidae;Bombus;Bombus mucidus;Germany;Bold;FBHAP813-09;-
Hymenoptera;Apidae;Bombus;Bombus muscorum;Norway;Bold;ARTNO110-17;-
Hymenoptera;Colletidae;Colletes;Colletes floralis;Italy;Bold;FBAPB981-09;-
Hymenoptera;Colletidae;Colletes;Colletes succinctus;Germany;Bold;GBACU2319-13;-
Hymenoptera;Colletidae;Colletes;Colletes succinctus;Germany;Bold;FBHAP212-09;-
Hymenoptera;Colletidae;Colletes;Colletes succinctus;Germany;Bold;GBACU2320-13;-
Hymenoptera;Colletidae;Colletes;Colletes succinctus;Germany;Bold;GBACU2340-13;-
Hymenoptera;Colletidae;Colletes;Colletes succinctus;Germany;Bold;FBHAP213-09;-
Hymenoptera;Colletidae;Colletes;Colletes succinctus;Germany;Bold;GBACU2318-13;-
Hymenoptera;Colletidae;Colletes;Colletes succinctus;Germany;Bold;GBACU2317-13;-
Hymenoptera;Halictidae;Dufourea;Dufourea dentiventris;Italy;Bold;GBACU3257-13;-
Hymenoptera;Halictidae;Dufourea;Dufourea inermis;Germany;Bold;FBAPB516-09;-
Hymenoptera;Halictidae;Dufourea;Dufourea inermis;Germany;Bold;FBAPB515-09;-
Hymenoptera;Halictidae;Dufourea;Dufourea minuta;Italy;Bold;GBACU3259-13;-
Hymenoptera;Halictidae;Dufourea;Dufourea minuta;Italy;Bold;FBAPB518-09;-
Hymenoptera;Halictidae;Dufourea;Dufourea minuta;Italy;Bold;GBACU2971-13;-
Hymenoptera;Apidae;Epeolus;Epeolus cruciger;Germany;Bold;GBACU2178-13;-
Hymenoptera;Apidae;Epeolus;Epeolus cruciger;Germany;Bold;FBAPC465-10;-
Hymenoptera;Halictidae;Halictus;Halictus leucaheneus;Germany;Bold;FBAPB320-09;-
Hymenoptera;Halictidae;Halictus;Halictus leucaheneus;Germany;Bold;FBAPB318-09;-

Hymenoptera;Halictidae;Halictus;Halictus quadricinctus;Germany;Bold;FBAPD445-11;-
Hymenoptera;Halictidae;Lasioglossum;Lasioglossum brevicorne;Slovakia;Bold;GBACU1386-12;-
Hymenoptera;Halictidae;Lasioglossum;Lasioglossum brevicorne;Germany;Bold;FBHAP671-09;-
Hymenoptera;Halictidae;Lasioglossum;Lasioglossum brevicorne;Germany;Bold;FBAPB370-09;-
Hymenoptera;Halictidae;Lasioglossum;Lasioglossum clypeare;Germany;Bold;FBAPC1007-11;-
Hymenoptera;Halictidae;Lasioglossum;Lasioglossum convexiusculum;Germany;Bold;FBHAP676-09;-
Hymenoptera;Halictidae;Lasioglossum;Lasioglossum convexiusculum;Italy;Bold;GBCHA1138-13;-
Hymenoptera;Halictidae;Lasioglossum;Lasioglossum convexiusculum;Germany;Bold;FBAPC996-11;-
Hymenoptera;Halictidae;Lasioglossum;Lasioglossum costulatum;Germany;Bold;FBAPB375-09;-
Hymenoptera;Halictidae;Lasioglossum;Lasioglossum costulatum;Germany;Bold;FBAPB376-09;-
Hymenoptera;Halictidae;Lasioglossum;Lasioglossum intermedium;Germany;Bold;FBAPD660-11;-
Hymenoptera;Halictidae;Lasioglossum;Lasioglossum intermedium;Germany;Bold;FBAPB870-09;-
Hymenoptera;Halictidae;Lasioglossum;Lasioglossum intermedium;Germany;Bold;GBCHA077-13;-
Hymenoptera;Halictidae;Lasioglossum;Lasioglossum intermedium;Germany;Bold;FBAPB871-09;-
Hymenoptera;Halictidae;Lasioglossum;Lasioglossum intermedium;Germany;Bold;FBAPB869-09;-
Hymenoptera;Halictidae;Lasioglossum;Lasioglossum intermedium;Germany;Bold;GBCHA078-13;-
Hymenoptera;Halictidae;Lasioglossum;Lasioglossum laevigatum;Austria;Bold;TDAAT374-19;-
Hymenoptera;Halictidae;Lasioglossum;Lasioglossum laevigatum;Italy;Bold;GBACU2958-13;-
Hymenoptera;Halictidae;Lasioglossum;Lasioglossum laevigatum;Germany;Bold;FBHAP690-09;-
Hymenoptera;Halictidae;Lasioglossum;Lasioglossum laevigatum;Italy;Bold;GBACU2959-13;-
Hymenoptera;Halictidae;Lasioglossum;Lasioglossum majus;Germany;Bold;FBAPB893-09;-
Hymenoptera;Halictidae;Lasioglossum;Lasioglossum majus;Germany;Bold;FBAPB892-09;-
Hymenoptera;Halictidae;Lasioglossum;Lasioglossum marginellum;Germany;Bold;FBAPD650-11;-
Hymenoptera;Halictidae;Lasioglossum;Lasioglossum minutulum;Germany;Bold;FBAPB898-09;-
Hymenoptera;Halictidae;Lasioglossum;Lasioglossum minutulum;Germany;Bold;FBAPB897-09;-
Hymenoptera;Halictidae;Lasioglossum;Lasioglossum minutulum;Germany;Bold;FBAPB899-09;-
Hymenoptera;Halictidae;Lasioglossum;Lasioglossum minutulum;Germany;Bold;FBHAP716-09;-
Hymenoptera;Halictidae;Lasioglossum;Lasioglossum minutulum;Germany;Bold;GBCHA073-13;-
Hymenoptera;Halictidae;Lasioglossum;Lasioglossum prasinum;Germany;Bold;FBAPB920-09;-
Hymenoptera;Halictidae;Lasioglossum;Lasioglossum prasinum;Germany;Bold;FBAPB919-09;-
Hymenoptera;Halictidae;Lasioglossum;Lasioglossum prasinum;Italy;Bold;FBAPB918-09;-
Hymenoptera;Halictidae;Lasioglossum;Lasioglossum prasinum;Germany;Bold;FBAPB921-09;-
Hymenoptera;Halictidae;Lasioglossum;Lasioglossum pygmaeum;Germany;Bold;FBAPD483-11;-
Hymenoptera;Halictidae;Lasioglossum;Lasioglossum pygmaeum;Germany;Bold;FBAPD482-11;-
Hymenoptera;Halictidae;Lasioglossum;Lasioglossum pygmaeum;Germany;Bold;FBAPD481-11;-
Hymenoptera;Halictidae;Lasioglossum;Lasioglossum pygmaeum;Germany;Bold;FBAPB927-09;-
Hymenoptera;Halictidae;Lasioglossum;Lasioglossum quadrinotatulum;Germany;Bold;FBAPC424-10;-
Hymenoptera;Halictidae;Lasioglossum;Lasioglossum quadrinotatulum;Germany;Bold;FBAPC425-10;-
Hymenoptera;Halictidae;Lasioglossum;Lasioglossum quadrinotatulum;Germany;Bold;FBAPC423-10;-
Hymenoptera;Halictidae;Lasioglossum;Lasioglossum quadrinotatulum;Italy;Bold;FBAPB937-09;-
Hymenoptera;Halictidae;Lasioglossum;Lasioglossum quadrinotatulum;Germany;Bold;FBAPC422-10;-
Hymenoptera;Halictidae;Lasioglossum;Lasioglossum sexnotatum;Germany;Bold;FBAPC1022-11;-
Hymenoptera;Halictidae;Lasioglossum;Lasioglossum sexnotatum;Germany;Bold;FBAPB951-09;-
Hymenoptera;Halictidae;Lasioglossum;Lasioglossum sexnotatum;Germany;Bold;FBACB916-11;-
Hymenoptera;Halictidae;Lasioglossum;Lasioglossum sexnotatum;Germany;Bold;FBAPB952-09;-
Hymenoptera;Halictidae;Lasioglossum;Lasioglossum sexnotatum;Germany;Bold;FBAPC387-10;-
Hymenoptera;Halictidae;Lasioglossum;Lasioglossum sexnotatum;Germany;Bold;FBAPC386-10;-
Hymenoptera;Halictidae;Lasioglossum;Lasioglossum sexnotatum;Germany;Bold;FBAPC1021-11;-
Hymenoptera;Halictidae;Lasioglossum;Lasioglossum subfasciatum;France;Bold;GBACU3066-13;-
Hymenoptera;Halictidae;Lasioglossum;Lasioglossum subfasciatum;Switzerland;Bold;FBAPB958-09;-
Hymenoptera;Halictidae;Lasioglossum;Lasioglossum subfasciatum;Italy;Bold;FBAPB956-09;-
Hymenoptera;Halictidae;Lasioglossum;Lasioglossum subfasciatum;Italy;Bold;FBAPB957-09;-
Hymenoptera;Halictidae;Lasioglossum;Lasioglossum subfasciatum;Germany;Bold;FBAPC1013-11;-
Hymenoptera;Halictidae;Lasioglossum;Lasioglossum subfasciatum;Germany;Bold;FBAPC1014-11;-
Hymenoptera;Melittidae;Melitta;Melitta tricincta;Germany;Bold;BCHYM2159-14;-
Hymenoptera;Melittidae;Melitta;Melitta tricincta;Germany;Bold;FBAPC110-10;-
Hymenoptera;Apidae;Nomada;Nomada argentata;Germany;Bold;FBAPB133-09;-
Hymenoptera;Apidae;Nomada;Nomada armata;Germany;Bold;FBAPB135-09;-
Hymenoptera;Apidae;Nomada;Nomada armata;Germany;Bold;FBHAP640-09;-
Hymenoptera;Apidae;Nomada;Nomada armata;Germany;Bold;FBAPC161-10;-
Hymenoptera;Apidae;Nomada;Nomada mutica;Germany;Bold;FBAPB250-09;-
Hymenoptera;Apidae;Nomada;Nomada mutica;Germany;Bold;FBAPB251-09;-
Hymenoptera;Apidae;Nomada;Nomada mutica;Germany;Bold;FBAPB252-09;-
Hymenoptera;Apidae;Nomada;Nomada rhenana;France;Bold;FBAPB268-09;-
Hymenoptera;Apidae;Nomada;Nomada rhenana;France;Bold;FBAPB267-09;-
Hymenoptera;Apidae;Nomada;Nomada roberjeotiana;Germany;Bold;FBAPB270-09;-
Hymenoptera;Halictidae;Rophites;Rophites quinquespinosus;Germany;Bold;GBACU2373-13;-
Hymenoptera;Halictidae;Rophites;Rophites quinquespinosus;Germany;Bold;GBACU2374-13;-
Hymenoptera;Halictidae;Rophites;Rophites quinquespinosus;Germany;Bold;GBACU2370-13;-
Hymenoptera;Halictidae;Rophites;Rophites quinquespinosus;Czech Republic;Bold;GBACU1377-12;-
Hymenoptera;Halictidae;Rophites;Rophites quinquespinosus;Germany;Bold;FBAPB773-09;-
Hymenoptera;Halictidae;Rophites;Rophites quinquespinosus;Germany;Bold;BCHYM2157-14;-
Hymenoptera;Halictidae;Rophites;Rophites quinquespinosus;Germany;Bold;BCHYM2158-14;-
Hymenoptera;Halictidae;Rophites;Rophites quinquespinosus;Germany;Bold;FBAPD509-11;-
Hymenoptera;Halictidae;Sphecodes;Sphecodes cristatus;Germany;Bold;FBAPB070-09;-
Hymenoptera;Halictidae;Sphecodes;Sphecodes cristatus;Germany;Bold;FBAPB069-09;-
Hymenoptera;Halictidae;Sphecodes;Sphecodes cristatus;Germany;Bold;FBAPB068-09;-
Hymenoptera;Halictidae;Sphecodes;Sphecodes croaticus;Italy;Bold;GBCHA969-13;-

Hymenoptera;Halictidae;Sphecodes;Sphecodes croaticus;Germany;Bold;BCHYM1615-14;-
Hymenoptera;Halictidae;Sphecodes;Sphecodes hyalinatus;Germany;Bold;FBHAP617-09;-
Hymenoptera;Halictidae;Sphecodes;Sphecodes majalis;Germany;Bold;FBAPB080-09;-
Hymenoptera;Halictidae;Sphecodes;Sphecodes majalis;Germany;Bold;FBAPB078-09;-
Hymenoptera;Halictidae;Sphecodes;Sphecodes majalis;Germany;Bold;GBACU806-12;-
Hymenoptera;Halictidae;Sphecodes;Sphecodes rubicundus;France;Bold;FBAPB088-09;-
Hymenoptera;Halictidae;Sphecodes;Sphecodes rubicundus;France;Bold;FBAPB089-09;-
Hymenoptera;Halictidae;Sphecodes;Sphecodes schenckii;Italy;Bold;FBAPB104-09;-
Hymenoptera;Halictidae;Sphecodes;Sphecodes spinulosus;Germany;Bold;FBAPB109-09;-
Hymenoptera;Halictidae;Sphecodes;Sphecodes spinulosus;Germany;Bold;FBAPD472-11;-
Hymenoptera;Halictidae;Sphecodes;Sphecodes spinulosus;Germany;Bold;BCHYM4575-14;-
Hymenoptera;Halictidae;Sphecodes;Sphecodes spinulosus;Germany;Bold;FBAPB771-09;-
Hymenoptera;Halictidae;Sphecodes;Sphecodes spinulosus;Germany;Bold;GBACU851-12;-
Hymenoptera;Halictidae;Sphecodes;Sphecodes spinulosus;Germany;Bold;FBAPD698-11;-
Hymenoptera;Halictidae;Sphecodes;Sphecodes spinulosus;Germany;Bold;GBACU2962-13;-
Hymenoptera;Halictidae;Sphecodes;Sphecodes spinulosus;Germany;Bold;FBAPB772-09;-
Hymenoptera;Halictidae;Sphecodes;Sphecodes spinulosus;Germany;Bold;FBAPD700-11;-
Hymenoptera;Halictidae;Sphecodes;Sphecodes spinulosus;Germany;Bold;FBAPB107-09;-
Hymenoptera;Halictidae;Sphecodes;Sphecodes spinulosus;Germany;Bold;FBAPB108-09;-
Hymenoptera;Halictidae;Sphecodes;Sphecodes spinulosus;Germany;Bold;FBAPD699-11;-
Hymenoptera;Halictidae;Sphecodes;Sphecodes spinulosus;Germany;Bold;FBAPB110-09;-
Hymenoptera;Halictidae;Systropha;Systropha curvicornis;Germany;Bold;GBACU1165-12;-
Hymenoptera;Halictidae;Systropha;Systropha curvicornis;Germany;Bold;GBACU1166-12;-
Hymenoptera;Halictidae;Systropha;Systropha curvicornis;Germany;Bold;GBACU1164-12;-
Lepidoptera;Nymphalidae;Aglais;Aglais urticae;Switzerland;Bold;LEPAA612-16;-
Lepidoptera;Nymphalidae;Apatura;Apatura ilia;Austria;Bold;LEASS998-17;-
Lepidoptera;Nymphalidae;Apatura;Apatura iris;Austria;Bold;LEASS841-17;-
Lepidoptera;Pieridae;Aporia;Aporia crataegi;Norway;Bold;LON6180-17;-
Lepidoptera;Pieridae;Aporia;Aporia crataegi;Norway;Bold;LON6179-17;-
Lepidoptera;Nymphalidae;Argynnis;Argynnis adippe;Austria;Bold;PHLAI484-13;-
Lepidoptera;Nymphalidae;Argynnis;Argynnis adippe;Austria;Bold;LEASS1000-17;-
Lepidoptera;Nymphalidae;Argynnis;Argynnis aglaja;United Kingdom;Bold;ANBIO099-19;-
Lepidoptera;Nymphalidae;Argynnis;Argynnis niobe;Norway;Bold;LON5660-17;-
Lepidoptera;Nymphalidae;Argynnis;Argynnis paphia;Germany;Bold;AMTPC536-15;-
Lepidoptera;Nymphalidae;Argynnis;Argynnis paphia;Switzerland;Bold;LEPAA680-16;-
Lepidoptera;Lycaenidae;Aricia;Aricia agestis;Italy;Bold;LEATH724-14;-
Lepidoptera;Lycaenidae;Aricia;Aricia agestis;Netherlands;Bold;NLLEA1493-14;-
Lepidoptera;Lycaenidae;Aricia;Aricia agestis;Switzerland;Bold;LEPPA1224-17;-
Lepidoptera;Lycaenidae;Aricia;Aricia agestis;Austria;Bold;LEASS834-17;-
Lepidoptera;Lycaenidae;Aricia;Aricia agestis;Austria;Bold;LEASS833-17;-
Lepidoptera;Lycaenidae;Aricia;Aricia agestis;Switzerland;Bold;LEPPA1220-17;-
Lepidoptera;Lycaenidae;Aricia;Aricia agestis;Switzerland;Bold;LEPAA636-16;-
Lepidoptera;Lycaenidae;Aricia;Aricia artaxerxes;Austria;Bold;PHLAI509-13;-
Lepidoptera;Lycaenidae;Aricia;Aricia artaxerxes;Switzerland;Bold;LEPAA454-16;-
Lepidoptera;Lycaenidae;Aricia;Aricia artaxerxes;Switzerland;Bold;LEPPA1227-17;-
Lepidoptera;Lycaenidae;Aricia;Aricia artaxerxes;Austria;Bold;PHLAH468-12;-
Lepidoptera;Lycaenidae;Aricia;Aricia artaxerxes;Austria;Bold;PHLAH469-12;-
Lepidoptera;Lycaenidae;Aricia;Aricia nicias;Switzerland;Bold;LEPAA764-16;-
Lepidoptera;Lycaenidae;Aricia;Aricia nicias;Switzerland;Bold;LEPAA845-16;-
Lepidoptera;Lycaenidae;Aricia;Aricia nicias;Switzerland;Bold;LEPAA606-16;-
Lepidoptera;Nymphalidae;Boloria;Boloria euphrosyne;Austria;Bold;PHLAI485-13;-
Lepidoptera;Nymphalidae;Boloria;Boloria euphrosyne;Norway;Bold;LON4631-16;-
Lepidoptera;Nymphalidae;Boloria;Boloria napaea;Italy;Bold;LEATG438-14;-
Lepidoptera;Nymphalidae;Boloria;Boloria napaea;Norway;Bold;LON5658-17;-
Lepidoptera;Nymphalidae;Boloria;Boloria napaea;Austria;Bold;LEASS1003-17;-
Lepidoptera;Nymphalidae;Boloria;Boloria pales;Switzerland;Bold;LEPAA082-16;-
Lepidoptera;Nymphalidae;Boloria;Boloria pales;Austria;Bold;LEATJ1300-16;-
Lepidoptera;Nymphalidae;Boloria;Boloria pales;Austria;Bold;LEATJ1304-16;-
Lepidoptera;Nymphalidae;Boloria;Boloria selene;Switzerland;Bold;LEPAA484-16;-
Lepidoptera;Nymphalidae;Boloria;Boloria selene;Switzerland;Bold;LEPAA110-16;-
Lepidoptera;Nymphalidae;Boloria;Boloria selene;Switzerland;Bold;LEPAA716-16;-
Lepidoptera;Nymphalidae;Boloria;Boloria thore;Norway;Bold;LON5666-17;-
Lepidoptera;Nymphalidae;Boloria;Boloria titania;Switzerland;Bold;LEPAA682-16;-
Lepidoptera;Nymphalidae;Brenthis;Brenthis daphne;Switzerland;Bold;LEPAA016-16;-
Lepidoptera;Nymphalidae;Brenthis;Brenthis daphne;Austria;Bold;LEASS1002-17;-
Lepidoptera;Nymphalidae;Brenthis;Brenthis hecate;Austria;Bold;ABOLD484-16;-
Lepidoptera;Nymphalidae;Brenthis;Brenthis ino;Austria;Bold;PHLAI482-13;-
Lepidoptera;Lycaenidae;Callophrys;Callophrys rubi;Norway;Bold;LON4577-16;-
Lepidoptera;Hesperiidae;Carcharodus;Carcharodus alceae;Austria;Bold;LEASS670-17;-
Lepidoptera;Hesperiidae;Carcharodus;Carcharodus alceae;Croatia;Bold;LON7250-18;-
Lepidoptera;Hesperiidae;Carcharodus;Carcharodus alceae;Greece;Bold;LON4617-16;-
Lepidoptera;Hesperiidae;Carcharodus;Carcharodus alceae;Switzerland;Bold;LEPAA065-16;-
Lepidoptera;Hesperiidae;Carcharodus;Carcharodus alceae;Italy;Bold;LEATG398-14;-
Lepidoptera;Hesperiidae;Carcharodus;Carcharodus lavatherae;Austria;Bold;ABOLD487-16;-
Lepidoptera;Hesperiidae;Carcharodus;Carcharodus lavatherae;Austria;Bold;ABOLD486-16;-
Lepidoptera;Hesperiidae;Carcharodus;Carcharodus lavatherae;Switzerland;Bold;LEPAA753-16;-
Lepidoptera;Hesperiidae;Carterocephalus;Carterocephalus palaemon;Austria;Bold;LEASS501-17;-
Lepidoptera;Hesperiidae;Carterocephalus;Carterocephalus palaemon;Switzerland;Bold;LEPAA148-16;-

Lepidoptera;Hesperiidae;Carterocephalus;Carterocephalus palaemon;Austria;Bold;PHLAI511-13;-
Lepidoptera;Lycaenidae;Celastrina;Celastrina argiolus;Norway;Bold;LON5648-17;-
Lepidoptera;Lycaenidae;Celastrina;Celastrina argiolus;Switzerland;Bold;LEPAA119-16;-
Lepidoptera;Lycaenidae;Celastrina;Celastrina argiolus;Switzerland;Bold;LEPAA328-16;-
Lepidoptera;Nymphalidae;Chazara;Chazara briseis;Austria;Bold;LEASS989-17;-
Lepidoptera;Nymphalidae;Coenonympha;Coenonympha darwiniana;Austria;Bold;LEASS1061-17;-
Lepidoptera;Nymphalidae;Coenonympha;Coenonympha darwiniana;Switzerland;Bold;LEPPA1183-17;-
Lepidoptera;Nymphalidae;Coenonympha;Coenonympha darwiniana;Switzerland;Bold;LEPAA174-16;-
Lepidoptera;Nymphalidae;Coenonympha;Coenonympha darwiniana;Austria;Bold;LEASS1060-17;-
Lepidoptera;Nymphalidae;Coenonympha;Coenonympha darwiniana;Austria;Bold;PHLAA708-09;-
Lepidoptera;Nymphalidae;Coenonympha;Coenonympha gardetta;Switzerland;Bold;LEPAA529-16;-
Lepidoptera;Nymphalidae;Coenonympha;Coenonympha gardetta;Switzerland;Bold;LEPPA1189-17;-
Lepidoptera;Nymphalidae;Coenonympha;Coenonympha gardetta;Switzerland;Bold;LEPAA677-16;-
Lepidoptera;Nymphalidae;Coenonympha;Coenonympha gardetta;Switzerland;Bold;LEPPA1265-17;-
Lepidoptera;Nymphalidae;Coenonympha;Coenonympha gardetta;Switzerland;Bold;LEPPA1194-17;-
Lepidoptera;Nymphalidae;Coenonympha;Coenonympha gardetta;Switzerland;Bold;LEPPA1191-17;-
Lepidoptera;Nymphalidae;Coenonympha;Coenonympha gardetta;Switzerland;Bold;LEPAA507-16;-
Lepidoptera;Nymphalidae;Coenonympha;Coenonympha glycerion;Switzerland;Bold;LEPAA728-16;-
Lepidoptera;Nymphalidae;Coenonympha;Coenonympha glycerion;Switzerland;Bold;LEPPA1243-17;-
Lepidoptera;Nymphalidae;Coenonympha;Coenonympha glycerion;Austria;Bold;LEASS981-17;-
Lepidoptera;Nymphalidae;Coenonympha;Coenonympha oedippus;Italy;Bold;OEDIP017-18;-
Lepidoptera;Nymphalidae;Coenonympha;Coenonympha oedippus;Poland;Bold;OEDIP002-18;-
Lepidoptera;Nymphalidae;Coenonympha;Coenonympha oedippus;France;Bold;OEDIP016-18;-
Lepidoptera;Nymphalidae;Coenonympha;Coenonympha oedippus;Poland;Bold;OEDIP035-18;-
Lepidoptera;Nymphalidae;Coenonympha;Coenonympha oedippus;Germany;Bold;OEDIP025-18;-
Lepidoptera;Nymphalidae;Coenonympha;Coenonympha oedippus;Poland;Bold;OEDIP038-18;-
Lepidoptera;Nymphalidae;Coenonympha;Coenonympha oedippus;France;Bold;OEDIP015-18;-
Lepidoptera;Nymphalidae;Coenonympha;Coenonympha oedippus;France;Bold;OEDIP032-18;-
Lepidoptera;Nymphalidae;Coenonympha;Coenonympha oedippus;France;Bold;OEDIP030-18;-
Lepidoptera;Nymphalidae;Coenonympha;Coenonympha oedippus;France;Bold;OEDIP008-18;-
Lepidoptera;Nymphalidae;Coenonympha;Coenonympha oedippus;France;Bold;OEDIP024-18;-
Lepidoptera;Nymphalidae;Coenonympha;Coenonympha oedippus;Poland;Bold;OEDIP001-18;-
Lepidoptera;Nymphalidae;Coenonympha;Coenonympha oedippus;France;Bold;OEDIP022-18;-
Lepidoptera;Nymphalidae;Coenonympha;Coenonympha oedippus;Italy;Bold;OEDIP011-18;-
Lepidoptera;Nymphalidae;Coenonympha;Coenonympha oedippus;France;Bold;OEDIP029-18;-
Lepidoptera;Nymphalidae;Coenonympha;Coenonympha oedippus;France;Bold;OEDIP003-18;-
Lepidoptera;Nymphalidae;Coenonympha;Coenonympha oedippus;France;Bold;OEDIP033-18;-
Lepidoptera;Nymphalidae;Coenonympha;Coenonympha oedippus;Liechtenstein;Bold;OEDIP034-18;-
Lepidoptera;Nymphalidae;Coenonympha;Coenonympha oedippus;France;Bold;OEDIP028-18;-
Lepidoptera;Nymphalidae;Coenonympha;Coenonympha oedippus;Slovenia;Bold;OEDIP006-18;-
Lepidoptera;Nymphalidae;Coenonympha;Coenonympha oedippus;France;Bold;OEDIP007-18;-
Lepidoptera;Nymphalidae;Coenonympha;Coenonympha oedippus;Italy;Bold;OEDIP018-18;-
Lepidoptera;Nymphalidae;Coenonympha;Coenonympha oedippus;Slovenia;Bold;OEDIP010-18;-
Lepidoptera;Nymphalidae;Coenonympha;Coenonympha oedippus;Slovenia;Bold;OEDIP005-18;-
Lepidoptera;Nymphalidae;Coenonympha;Coenonympha oedippus;France;Bold;OEDIP023-18;-
Lepidoptera;Nymphalidae;Coenonympha;Coenonympha oedippus;Slovenia;Bold;OEDIP020-18;-
Lepidoptera;Nymphalidae;Coenonympha;Coenonympha oedippus;Slovenia;Bold;OEDIP009-18;-
Lepidoptera;Nymphalidae;Coenonympha;Coenonympha oedippus;Liechtenstein;Bold;OEDIP026-18;-
Lepidoptera;Nymphalidae;Coenonympha;Coenonympha oedippus;Slovenia;Bold;OEDIP019-18;-
Lepidoptera;Nymphalidae;Coenonympha;Coenonympha oedippus;Italy;Bold;OEDIP004-18;-
Lepidoptera;Nymphalidae;Coenonympha;Coenonympha oedippus;Italy;Bold;OEDIP014-18;-
Lepidoptera;Nymphalidae;Coenonympha;Coenonympha oedippus;Italy;Bold;OEDIP037-18;-
Lepidoptera;Nymphalidae;Coenonympha;Coenonympha oedippus;Germany;Bold;OEDIP027-18;-
Lepidoptera;Nymphalidae;Coenonympha;Coenonympha oedippus;Italy;Bold;OEDIP013-18;-
Lepidoptera;Nymphalidae;Coenonympha;Coenonympha oedippus;Italy;Bold;OEDIP036-18;-
Lepidoptera;Nymphalidae;Coenonympha;Coenonympha oedippus;Italy;Bold;OEDIP012-18;-
Lepidoptera;Nymphalidae;Coenonympha;Coenonympha oedippus;Slovenia;Bold;OEDIP021-18;-
Lepidoptera;Nymphalidae;Coenonympha;Coenonympha oedippus;France;Bold;OEDIP031-18;-
Lepidoptera;Nymphalidae;Coenonympha;Coenonympha pamphilus;Switzerland;Bold;LEPAA370-16;-
Lepidoptera;Pieridae;Colias;Colias alfacariensis;Italy;Bold;SACOL239-18;-
Lepidoptera;Pieridae;Colias;Colias alfacariensis;France;Bold;SACOL235-18;-
Lepidoptera;Pieridae;Colias;Colias alfacariensis;Switzerland;Bold;LEPAA430-16;-
Lepidoptera;Pieridae;Colias;Colias alfacariensis;Italy;Bold;ABOLB052-15;-
Lepidoptera;Pieridae;Colias;Colias alfacariensis;Russia;Bold;SACOL346-19;-
Lepidoptera;Pieridae;Colias;Colias hyale;Sweden;Bold;LON5677-17;-
Lepidoptera;Pieridae;Colias;Colias hyale;Turkey;Bold;SACOL242-18;-
Lepidoptera;Pieridae;Colias;Colias hyale;Russia;Bold;SACOL246-18;-
Lepidoptera;Pieridae;Colias;Colias hyale;Russia;Bold;SACOL249-18;-
Lepidoptera;Pieridae;Colias;Colias palaeno;Switzerland;Bold;LEPAA679-16;-
Lepidoptera;Pieridae;Colias;Colias palaeno;Italy;Bold;LEATG527-14;-
Lepidoptera;Pieridae;Colias;Colias palaeno;Switzerland;Bold;LEPAA212-16;-
Lepidoptera;Pieridae;Colias;Colias palaeno;Switzerland;Bold;LEPAA748-16;-
Lepidoptera;Pieridae;Colias;Colias palaeno;Norway;Bold;LON4623-16;-
Lepidoptera;Lycaenidae;Cupido;Cupido alcetas;Switzerland;Bold;LEPAA172-16;-
Lepidoptera;Lycaenidae;Cupido;Cupido alcetas;Switzerland;Bold;LEPAA181-16;-
Lepidoptera;Lycaenidae;Cupido;Cupido argiades;Switzerland;Bold;LEPAA813-16;-
Lepidoptera;Lycaenidae;Cupido;Cupido argiades;Switzerland;Bold;LEPAA676-16;-
Lepidoptera;Lycaenidae;Cupido;Cupido argiades;Switzerland;Bold;LEPAA664-16;-

Lepidoptera;Lycaenidae;Cupido;Cupido minimus;Switzerland;Bold;LEPAA115-16;-
Lepidoptera;Lycaenidae;Cupido;Cupido minimus;Austria;Bold;LEASS543-17;-
Lepidoptera;Lycaenidae;Cupido;Cupido minimus;Austria;Bold;PHLAI500-13;-
Lepidoptera;Lycaenidae;Cupido;Cupido osiris;Austria;Bold;ABOLD493-16;-
Lepidoptera;Lycaenidae;Cupido;Cupido osiris;Switzerland;Bold;LEPAA790-16;-
Lepidoptera;Lycaenidae;Cupido;Cupido osiris;Switzerland;Bold;LEPAA772-16;-
Lepidoptera;Lycaenidae;Cyaniris;Cyaniris semiargus;Switzerland;Bold;LEPAA147-16;-
Lepidoptera;Lycaenidae;Cyaniris;Cyaniris semiargus;Austria;Bold;PHLAI497-13;-
Lepidoptera;Nymphalidae;Erebia;Erebia arvernensis;Switzerland;Bold;LEPPA1196-17;-
Lepidoptera;Nymphalidae;Erebia;Erebia epiphron;Austria;Bold;HBOK108-08;-
Lepidoptera;Nymphalidae;Erebia;Erebia epiphron;Italy;Bold;LEASW1772-20;-
Lepidoptera;Nymphalidae;Erebia;Erebia eriphyle;Austria;Bold;HBOK102-08;-
Lepidoptera;Nymphalidae;Erebia;Erebia eriphyle;Austria;Bold;ABOLD039-16;-
Lepidoptera;Nymphalidae;Erebia;Erebia euryale;Switzerland;Bold;LEPAA112-16;-
Lepidoptera;Nymphalidae;Erebia;Erebia euryale;Austria;Bold;CUPED080-16;-
Lepidoptera;Nymphalidae;Erebia;Erebia euryale;Austria;Bold;CUPED079-16;-
Lepidoptera;Nymphalidae;Erebia;Erebia euryale;Austria;Bold;CUPED075-16;-
Lepidoptera;Nymphalidae;Erebia;Erebia euryale;Austria;Bold;CUPED094-16;-
Lepidoptera;Nymphalidae;Erebia;Erebia euryale;Austria;Bold;CUPED090-16;-
Lepidoptera;Nymphalidae;Erebia;Erebia euryale;Austria;Bold;CUPED077-16;-
Lepidoptera;Nymphalidae;Erebia;Erebia euryale;Austria;Bold;CUPED081-16;-
Lepidoptera;Nymphalidae;Erebia;Erebia euryale;Austria;Bold;CUPED089-16;-
Lepidoptera;Nymphalidae;Erebia;Erebia euryale;Austria;Bold;CUPED091-16;-
Lepidoptera;Nymphalidae;Erebia;Erebia euryale;Austria;Bold;CUPED084-16;-
Lepidoptera;Nymphalidae;Erebia;Erebia euryale;Austria;Bold;PHLAI491-13;-
Lepidoptera;Nymphalidae;Erebia;Erebia euryale;Austria;Bold;CUPED087-16;-
Lepidoptera;Nymphalidae;Erebia;Erebia euryale;Austria;Bold;CUPED088-16;-
Lepidoptera;Nymphalidae;Erebia;Erebia euryale;Austria;Bold;CUPED093-16;-
Lepidoptera;Nymphalidae;Erebia;Erebia euryale;Austria;Bold;CUPED078-16;-
Lepidoptera;Nymphalidae;Erebia;Erebia euryale;Austria;Bold;CUPED086-16;-
Lepidoptera;Nymphalidae;Erebia;Erebia euryale;Austria;Bold;CUPED076-16;-
Lepidoptera;Nymphalidae;Erebia;Erebia euryale;Austria;Bold;CUPED095-16;-
Lepidoptera;Nymphalidae;Erebia;Erebia euryale;Austria;Bold;CUPED092-16;-
Lepidoptera;Nymphalidae;Erebia;Erebia euryale;Austria;Bold;CUPED082-16;-
Lepidoptera;Nymphalidae;Erebia;Erebia euryale;Austria;Bold;CUPED083-16;-
Lepidoptera;Nymphalidae;Erebia;Erebia euryale;Switzerland;Bold;LEPPA1185-17;-
Lepidoptera;Nymphalidae;Erebia;Erebia euryale;Switzerland;Bold;LEPAA861-16;-
Lepidoptera;Nymphalidae;Erebia;Erebia euryale;Italy;Bold;CUPED065-16;-
Lepidoptera;Nymphalidae;Erebia;Erebia euryale;Italy;Bold;LEASW1769-20;-
Lepidoptera;Nymphalidae;Erebia;Erebia euryale;Austria;Bold;CUPED085-16;-
Lepidoptera;Nymphalidae;Erebia;Erebia flavofasciata;Austria;Bold;HBOK105-08;-
Lepidoptera;Nymphalidae;Erebia;Erebia flavofasciata;Switzerland;Bold;LEPAA773-16;-
Lepidoptera;Nymphalidae;Erebia;Erebia gorge;Austria;Bold;HBOK125-08;-
Lepidoptera;Nymphalidae;Erebia;Erebia ligea;Austria;Bold;HBOK096-08;-
Lepidoptera;Nymphalidae;Erebia;Erebia ligea;Switzerland;Bold;LEPAA102-16;-
Lepidoptera;Nymphalidae;Erebia;Erebia manto;Austria;Bold;CUPED015-16;-
Lepidoptera;Nymphalidae;Erebia;Erebia manto;Austria;Bold;CUPED016-16;-
Lepidoptera;Nymphalidae;Erebia;Erebia manto;Austria;Bold;CUPED021-16;-
Lepidoptera;Nymphalidae;Erebia;Erebia manto;Austria;Bold;CUPED028-16;-
Lepidoptera;Nymphalidae;Erebia;Erebia manto;Austria;Bold;CUPED024-16;-
Lepidoptera;Nymphalidae;Erebia;Erebia manto;Austria;Bold;CUPED034-16;-
Lepidoptera;Nymphalidae;Erebia;Erebia manto;Austria;Bold;CUPED023-16;-
Lepidoptera;Nymphalidae;Erebia;Erebia manto;Austria;Bold;CUPED031-16;-
Lepidoptera;Nymphalidae;Erebia;Erebia manto;Austria;Bold;CUPED033-16;-
Lepidoptera;Nymphalidae;Erebia;Erebia manto;Switzerland;Bold;LEPPA1186-17;-
Lepidoptera;Nymphalidae;Erebia;Erebia manto;Switzerland;Bold;LEPPA1188-17;-
Lepidoptera;Nymphalidae;Erebia;Erebia manto;Switzerland;Bold;LEPAA502-16;-
Lepidoptera;Nymphalidae;Erebia;Erebia manto;Switzerland;Bold;LEPPA1187-17;-
Lepidoptera;Nymphalidae;Erebia;Erebia manto;Switzerland;Bold;LEPAA958-18;-
Lepidoptera;Nymphalidae;Erebia;Erebia manto;Austria;Bold;CUPED022-16;-
Lepidoptera;Nymphalidae;Erebia;Erebia manto;Austria;Bold;CUPED014-16;-
Lepidoptera;Nymphalidae;Erebia;Erebia manto;Austria;Bold;CUPED036-16;-
Lepidoptera;Nymphalidae;Erebia;Erebia manto;Austria;Bold;CUPED027-16;-
Lepidoptera;Nymphalidae;Erebia;Erebia manto;Austria;Bold;CUPED032-16;-
Lepidoptera;Nymphalidae;Erebia;Erebia manto;Austria;Bold;CUPED018-16;-
Lepidoptera;Nymphalidae;Erebia;Erebia manto;Austria;Bold;CUPED029-16;-
Lepidoptera;Nymphalidae;Erebia;Erebia manto;Austria;Bold;CUPED019-16;-
Lepidoptera;Nymphalidae;Erebia;Erebia manto;Austria;Bold;CUPED025-16;-
Lepidoptera;Nymphalidae;Erebia;Erebia manto;Austria;Bold;CUPED026-16;-
Lepidoptera;Nymphalidae;Erebia;Erebia manto;Austria;Bold;CUPED035-16;-
Lepidoptera;Nymphalidae;Erebia;Erebia manto;Austria;Bold;CUPED017-16;-
Lepidoptera;Nymphalidae;Erebia;Erebia manto;Austria;Bold;CUPED020-16;-
Lepidoptera;Nymphalidae;Erebia;Erebia manto;Austria;Bold;CUPED030-16;-
Lepidoptera;Nymphalidae;Erebia;Erebia medusa;Austria;Bold;HBOK120-08;-
Lepidoptera;Nymphalidae;Erebia;Erebia melampus;Austria;Bold;HBOK111-08;-
Lepidoptera;Nymphalidae;Erebia;Erebia melampus;Switzerland;Bold;LEPAA164-16;-
Lepidoptera;Nymphalidae;Erebia;Erebia melampus;Austria;Bold;HBOK112-08;-
Lepidoptera;Nymphalidae;Erebia;Erebia meolans;Austria;Bold;HBOK159-08;-

Lepidoptera;Nymphalidae;Erebia;Erebia meolans;Austria;Bold;HBOK160-08;-
Lepidoptera;Nymphalidae;Erebia;Erebia montana;Switzerland;Bold;LEPAA311-16;-
Lepidoptera;Nymphalidae;Erebia;Erebia nivalis;Austria;Bold;LEASS843-17;-
Lepidoptera;Nymphalidae;Erebia;Erebia nivalis;Switzerland;Bold;LEPAA774-16;-
Lepidoptera;Nymphalidae;Erebia;Erebia oeme;Italy;Bold;LEATD015-13;-
Lepidoptera;Nymphalidae;Erebia;Erebia oeme;Austria;Bold;HBOK147-08;-
Lepidoptera;Nymphalidae;Erebia;Erebia oeme;Austria;Bold;HBOK148-08;-
Lepidoptera;Nymphalidae;Erebia;Erebia pandrose;Austria;Bold;HBOK164-08;-
Lepidoptera;Nymphalidae;Erebia;Erebia pharte;Austria;Bold;HBOK110-08;-
Lepidoptera;Nymphalidae;Erebia;Erebia pharte;Austria;Bold;HBOK109-08;-
Lepidoptera;Nymphalidae;Erebia;Erebia pluto;Switzerland;Bold;LEPAA494-16;-
Lepidoptera;Nymphalidae;Erebia;Erebia pluto;Austria;Bold;HBOK123-08;-
Lepidoptera;Nymphalidae;Erebia;Erebia pluto;Austria;Bold;HBOK124-08;-
Lepidoptera;Nymphalidae;Erebia;Erebia pronoe;Switzerland;Bold;LEPAA243-16;-
Lepidoptera;Nymphalidae;Erebia;Erebia pronoe;Switzerland;Bold;LEPAA776-16;-
Lepidoptera;Nymphalidae;Erebia;Erebia styx;Switzerland;Bold;LEPAA778-16;-
Lepidoptera;Nymphalidae;Erebia;Erebia styx;Austria;Bold;HBOK154-08;-
Lepidoptera;Nymphalidae;Erebia;Erebia styx;Switzerland;Bold;LEPAA608-16;-
Lepidoptera;Nymphalidae;Erebia;Erebia styx;Austria;Bold;HBOK153-08;-
Lepidoptera;Nymphalidae;Erebia;Erebia triaria;Italy;Bold;LEATD014-13;-
Lepidoptera;Nymphalidae;Erebia;Erebia triaria;Italy;Bold;LEATD013-13;-
Lepidoptera;Nymphalidae;Erebia;Erebia tyndarus;Switzerland;Bold;LEPPA1197-17;-
Lepidoptera;Nymphalidae;Erebia;Erebia tyndarus;Switzerland;Bold;LEPAA113-16;-
Lepidoptera;Nymphalidae;Erebia;Erebia tyndarus;Switzerland;Bold;LEPPA1195-17;-
Lepidoptera;Hesperiidae;Erynnis;Erynnis tages;Switzerland;Bold;LEPAA523-16;-
Lepidoptera;Hesperiidae;Erynnis;Erynnis tages;Austria;Bold;PHLAI515-13;-
Lepidoptera;Hesperiidae;Erynnis;Erynnis tages;Switzerland;Bold;LEPAA418-16;-
Lepidoptera;Hesperiidae;Erynnis;Erynnis tages;Switzerland;Bold;LEPAA546-16;-
Lepidoptera;Hesperiidae;Erynnis;Erynnis tages;Switzerland;Bold;LEPAA292-16;-
Lepidoptera;Pieridae;Euchloe;Euchloe simplonia;Switzerland;Bold;LEPAA678-16;-
Lepidoptera;Lycaenidae;Eumedonia;Eumedonia eumedon;Switzerland;Bold;LEPAA117-16;-
Lepidoptera;Lycaenidae;Favonius;Favonius quercus;Austria;Bold;LEATI058-15;-
Lepidoptera;Lycaenidae;Favonius;Favonius quercus;Austria;Bold;LEATI059-15;-
Lepidoptera;Lycaenidae;Glaucopsyche;Glaucopsyche alexis;Austria;Bold;LEATI055-15;-
Lepidoptera;Lycaenidae;Glaucopsyche;Glaucopsyche alexis;Austria;Bold;LEATI056-15;-
Lepidoptera;Pieridae;Gonepteryx;Gonepteryx cleopatra;Greece;Bold;LON4614-16;-
Lepidoptera;Hesperiidae;Heteropterus;Heteropterus morpheus;Switzerland;Bold;LEPAA609-16;-
Lepidoptera;Hesperiidae;Heteropterus;Heteropterus morpheus;Austria;Bold;LEASS967-17;-
Lepidoptera;Nymphalidae;Hipparchia;Hipparchia fagi;Switzerland;Bold;LEPAA955-18;-
Lepidoptera;Nymphalidae;Hipparchia;Hipparchia fagi;Austria;Bold;LEATJ1320-16;-
Lepidoptera;Nymphalidae;Hipparchia;Hipparchia fagi;Italy;Bold;LEASX714-21;-
Lepidoptera;Nymphalidae;Hipparchia;Hipparchia fagi;Switzerland;Bold;LEPAA959-18;-
Lepidoptera;Nymphalidae;Hipparchia;Hipparchia fagi;Austria;Bold;LEASS985-17;-
Lepidoptera;Nymphalidae;Hipparchia;Hipparchia hermione;Norway;Bold;LON6144-17;-
Lepidoptera;Nymphalidae;Hipparchia;Hipparchia semele;Norway;Bold;LON6145-17;-
Lepidoptera;Nymphalidae;Hipparchia;Hipparchia semele;Denmark;Bold;LON913-12;-
Lepidoptera;Nymphalidae;Hipparchia;Hipparchia semele;Austria;Bold;LEASS987-17;-
Lepidoptera;Nymphalidae;Hipparchia;Hipparchia semele;Norway;Bold;LON6147-17;-
Lepidoptera;Nymphalidae;Hipparchia;Hipparchia semele;Norway;Bold;LON6146-17;-
Lepidoptera;Nymphalidae;Hipparchia;Hipparchia semele;Norway;Bold;LON6148-17;-
Lepidoptera;Nymphalidae;Hipparchia;Hipparchia semele;Norway;Bold;LON4636-16;-
Lepidoptera;Nymphalidae;Hipparchia;Hipparchia semele;Austria;Bold;LEASS902-17;-
Lepidoptera;Nymphalidae;Hipparchia;Hipparchia semele;Denmark;Bold;LON912-12;-
Lepidoptera;Nymphalidae;Hipparchia;Hipparchia semele;Austria;Bold;LEASS901-17;-
Lepidoptera;Nymphalidae;Hipparchia;Hipparchia statilinus;Austria;Bold;ABOLB166-15;-
Lepidoptera;Nymphalidae;Hyponephele;Hyponephele lycaon;Austria;Bold;LEASS899-17;-
Lepidoptera;Nymphalidae;Hyponephele;Hyponephele lycaon;Austria;Bold;LEASS900-17;-
Lepidoptera;Nymphalidae;Hyponephele;Hyponephele lycaon;Austria;Bold;LEASS976-17;-
Lepidoptera;Lycaenidae;Iolana;Iolana iolas;Switzerland;Bold;LEPAA775-16;-
Lepidoptera;Nymphalidae;Issoria;Issoria lathonia;Norway;Bold;LON5661-17;-
Lepidoptera;Nymphalidae;Issoria;Issoria lathonia;Norway;Bold;LON5662-17;-
Lepidoptera;Lycaenidae;Lampides;Lampides boeticus;Switzerland;Bold;LEPAA669-16;-
Lepidoptera;Lycaenidae;Lampides;Lampides boeticus;Greece;Bold;LON4616-16;-
Lepidoptera;Nymphalidae;Lasiommata;Lasiommata maera;Norway;Bold;LON4632-16;-
Lepidoptera;Nymphalidae;Lasiommata;Lasiommata megera;Italy;Bold;LEATD012-13;-
Lepidoptera;Pieridae;Leptidea;Leptidea juvernica;Switzerland;Bold;LEPPA1245-17;-
Lepidoptera;Pieridae;Leptidea;Leptidea juvernica;Switzerland;Bold;LEPAA558-16;-
Lepidoptera;Pieridae;Leptidea;Leptidea sinapis;Bosnia and Herzegovina;Bold;HBOK094-08;-
Lepidoptera;Pieridae;Leptidea;Leptidea sinapis;Switzerland;Bold;LEPPA1244-17;-
Lepidoptera;Pieridae;Leptidea;Leptidea sinapis;Switzerland;Bold;LEPAA827-16;-
Lepidoptera;Pieridae;Leptidea;Leptidea sinapis;Switzerland;Bold;LEPAA135-16;-
Lepidoptera;Pieridae;Leptidea;Leptidea sinapis;Switzerland;Bold;LEPAA383-16;-
Lepidoptera;Lycaenidae;Leptotes;Leptotes pirithous;Austria;Bold;LEASS918-17;-
Lepidoptera;Nymphalidae;Libythea;Libythea celtis;Italy;Bold;LEATG435-14;-
Lepidoptera;Nymphalidae;Libythea;Libythea celtis;Italy;Bold;LEATG436-14;-
Lepidoptera;Nymphalidae;Limenitis;Limenitis camilla;Austria;Bold;LEASS994-17;-
Lepidoptera;Nymphalidae;Limenitis;Limenitis populi;Italy;Bold;LEATH721-14;-
Lepidoptera;Nymphalidae;Limenitis;Limenitis populi;Austria;Bold;LEASS846-17;-

Lepidoptera;Nymphalidae;Limenitis;Limenitis populi;Austria;Bold;LEASS996-17;-
Lepidoptera;Nymphalidae;Limenitis;Limenitis populi;Austria;Bold;LEATI001-15;-
Lepidoptera;Nymphalidae;Limenitis;Limenitis populi;Austria;Bold;LEASS995-17;-
Lepidoptera;Nymphalidae;Lopinga;Lopinga achine;Austria;Bold;LEASS775-17;-
Lepidoptera;Nymphalidae;Lopinga;Lopinga achine;Austria;Bold;PHLAW024-13;-
Lepidoptera;Nymphalidae;Lopinga;Lopinga achine;Austria;Bold;LEASS505-17;-
Lepidoptera;Lycaenidae;Lycaena;Lycaena alciphron;Austria;Bold;LEASS546-17;-
Lepidoptera;Lycaenidae;Lycaena;Lycaena helle;Austria;Bold;LASTS097-14;-
Lepidoptera;Lycaenidae;Lycaena;Lycaena helle;Switzerland;Bold;LEPPA1241-17;-
Lepidoptera;Lycaenidae;Lycaena;Lycaena helle;Austria;Bold;LEASS509-17;-
Lepidoptera;Lycaenidae;Lycaena;Lycaena helle;Switzerland;Bold;LEPAA704-16;-
Lepidoptera;Lycaenidae;Lycaena;Lycaena hippothoe;Switzerland;Bold;LEPPA1205-17;-
Lepidoptera;Lycaenidae;Lycaena;Lycaena hippothoe;Switzerland;Bold;LEPAA683-16;-
Lepidoptera;Lycaenidae;Lycaena;Lycaena hippothoe;Sweden;Bold;LON5672-17;-
Lepidoptera;Lycaenidae;Lycaena;Lycaena phlaeas;Switzerland;Bold;LEPAA629-16;-
Lepidoptera;Lycaenidae;Lycaena;Lycaena phlaeas;Switzerland;Bold;LEPAA132-16;-
Lepidoptera;Lycaenidae;Lycaena;Lycaena phlaeas;Switzerland;Bold;LEPAA307-16;-
Lepidoptera;Lycaenidae;Lycaena;Lycaena tityrus;Switzerland;Bold;LEPAA836-16;-
Lepidoptera;Lycaenidae;Lycaena;Lycaena tityrus;Switzerland;Bold;LEPPA1228-17;-
Lepidoptera;Lycaenidae;Lycaena;Lycaena virgaureae;Switzerland;Bold;LEPAA515-16;-
Lepidoptera;Lycaenidae;Lycaena;Lycaena virgaureae;Switzerland;Bold;LEPAA675-16;-
Lepidoptera;Lycaenidae;Lycaena;Lycaena virgaureae;Switzerland;Bold;LEPAA889-16;-
Lepidoptera;Nymphalidae;Maniola;Maniola jurtina;Norway;Bold;LON6140-17;-
Lepidoptera;Nymphalidae;Maniola;Maniola jurtina;Norway;Bold;LON6826-18;-
Lepidoptera;Nymphalidae;Maniola;Maniola jurtina;Norway;Bold;LON6141-17;-
Lepidoptera;Nymphalidae;Maniola;Maniola jurtina;Switzerland;Bold;LEPAA142-16;-
Lepidoptera;Nymphalidae;Melanargia;Melanargia arge;Italy;Bold;VNMB553-08;-
Lepidoptera;Nymphalidae;Melitaea;Melitaea asteria;Switzerland;Bold;LEPAA808-16;-
Lepidoptera;Nymphalidae;Melitaea;Melitaea asteria;Switzerland;Bold;LEPPA1207-17;-
Lepidoptera;Nymphalidae;Melitaea;Melitaea asteria;Switzerland;Bold;LEPPA1208-17;-
Lepidoptera;Nymphalidae;Melitaea;Melitaea asteria;Switzerland;Bold;LEPAA648-16;-
Lepidoptera;Nymphalidae;Melitaea;Melitaea aurelia;Austria;Bold;LEASS881-17;-
Lepidoptera;Nymphalidae;Melitaea;Melitaea britomartis;Switzerland;Bold;LEPAA610-16;-
Lepidoptera;Nymphalidae;Melitaea;Melitaea britomartis;Austria;Bold;LEASS956-17;-
Lepidoptera;Nymphalidae;Melitaea;Melitaea britomartis;Austria;Bold;LEASS1015-17;-
Lepidoptera;Nymphalidae;Melitaea;Melitaea didyma;Austria;Bold;LEASS685-17;-
Lepidoptera;Nymphalidae;Melitaea;Melitaea didyma;Switzerland;Bold;LEPPA1200-17;-
Lepidoptera;Nymphalidae;Melitaea;Melitaea didyma;Switzerland;Bold;LEPAA108-16;-
Lepidoptera;Nymphalidae;Melitaea;Melitaea nevadensis;Switzerland;Bold;LEPPA1210-17;-
Lepidoptera;Nymphalidae;Melitaea;Melitaea nevadensis;Switzerland;Bold;LEPPA1214-17;-
Lepidoptera;Nymphalidae;Melitaea;Melitaea nevadensis;Switzerland;Bold;LEPPA1218-17;-
Lepidoptera;Nymphalidae;Melitaea;Melitaea nevadensis;Switzerland;Bold;LEPPA1219-17;-
Lepidoptera;Nymphalidae;Melitaea;Melitaea parthenoides;Austria;Bold;LEASS954-17;-
Lepidoptera;Nymphalidae;Melitaea;Melitaea trivia;Austria;Bold;LEATJ1324-16;-
Lepidoptera;Nymphalidae;Minois;Minois dryas;Austria;Bold;LEASS992-17;-
Lepidoptera;Nymphalidae;Neptis;Neptis rivularis;Austria;Bold;LEASS889-17;-
Lepidoptera;Nymphalidae;Neptis;Neptis sappho;Austria;Bold;LEASS988-17;-
Lepidoptera;Nymphalidae;Nymphalis;Nymphalis antiopa;Switzerland;Bold;LEPAA161-16;-
Lepidoptera;Nymphalidae;Nymphalis;Nymphalis antiopa;Italy;Bold;PHLAI552-13;-
Lepidoptera;Nymphalidae;Nymphalis;Nymphalis antiopa;Austria;Bold;PHLAI553-13;-
Lepidoptera;Nymphalidae;Nymphalis;Nymphalis polychloros;Italy;Bold;PHLAI495-13;-
Lepidoptera;Nymphalidae;Nymphalis;Nymphalis polychloros;Italy;Bold;PHLAI554-13;-
Lepidoptera;Nymphalidae;Pararge;Pararge aegeria;Switzerland;Bold;LEPAA160-16;-
Lepidoptera;Nymphalidae;Pararge;Pararge aegeria;Switzerland;Bold;LEPAA321-16;-
Lepidoptera;Papilionidae;Parnassius;Parnassius apollo;Switzerland;Bold;LEPAA283-16;-
Lepidoptera;Pieridae;Pieris;Pieris bryoniae;Switzerland;Bold;LEPAA829-16;-
Lepidoptera;Pieridae;Pieris;Pieris bryoniae;Switzerland;Bold;LEPAA865-16;-
Lepidoptera;Pieridae;Pieris;Pieris mannii;Italy;Bold;LEATG535-14;-
Lepidoptera;Pieridae;Pieris;Pieris mannii;Austria;Bold;ABOLD482-16;-
Lepidoptera;Pieridae;Pieris;Pieris mannii;Italy;Bold;LEATG534-14;-
Lepidoptera;Pieridae;Pieris;Pieris napi;Switzerland;Bold;LEPAA355-16;-
Lepidoptera;Pieridae;Pieris;Pieris napi;Denmark;Bold;LON915-12;-
Lepidoptera;Pieridae;Pieris;Pieris napi;Switzerland;Bold;LEPAA151-16;-
Lepidoptera;Pieridae;Pieris;Pieris napi;Switzerland;Bold;LEPAA320-16;-
Lepidoptera;Pieridae;Pieris;Pieris napi;Turkey;Bold;LON916-12;-
Lepidoptera;Pieridae;Pieris;Pieris rapae;Germany;Bold;AMTPC461-15;-
Lepidoptera;Lycaenidae;Plebejus;Plebejus argus;Italy;Bold;LEATG414-14;-
Lepidoptera;Lycaenidae;Plebejus;Plebejus argus;Switzerland;Bold;LEPAA574-16;-
Lepidoptera;Lycaenidae;Plebejus;Plebejus argus;Serbia;Bold;LYCAE463-15;-
Lepidoptera;Lycaenidae;Plebejus;Plebejus argus;Switzerland;Bold;LEPAA463-16;-
Lepidoptera;Lycaenidae;Plebejus;Plebejus idas;France;Bold;PHLAA429-09;-
Lepidoptera;Lycaenidae;Plebejus;Plebejus idas;Austria;Bold;PHLAI498-13;-
Lepidoptera;Nymphalidae;Polygonia;Polygonia c-album;Italy;Bold;LEATG475-14;-
Lepidoptera;Lycaenidae;Polyommatus;Polyommatus amandus;Norway;Bold;LON6191-17;-
Lepidoptera;Lycaenidae;Polyommatus;Polyommatus amandus;Norway;Bold;LON6192-17;-
Lepidoptera;Lycaenidae;Polyommatus;Polyommatus amandus;Switzerland;Bold;LEPAA646-16;-
Lepidoptera;Lycaenidae;Polyommatus;Polyommatus coridon;Romania;Bold;EZRMN400-09;-
Lepidoptera;Lycaenidae;Polyommatus;Polyommatus daphnis;Austria;Bold;LEASS678-17;-

Lepidoptera;Lycaenidae;Polyommatus;Polyommatus dorylas;Italy;Bold;LEATH740-14;-
Lepidoptera;Lycaenidae;Polyommatus;Polyommatus dorylas;Switzerland;Bold;LEPAA627-16;-
Lepidoptera;Lycaenidae;Polyommatus;Polyommatus dorylas;Austria;Bold;LEASS1039-17;-
Lepidoptera;Lycaenidae;Polyommatus;Polyommatus icarus;Austria;Bold;PHLAI496-13;-
Lepidoptera;Lycaenidae;Polyommatus;Polyommatus icarus;Switzerland;Bold;LEPAA811-16;-
Lepidoptera;Lycaenidae;Polyommatus;Polyommatus icarus;Switzerland;Bold;LEPAA412-16;-
Lepidoptera;Lycaenidae;Polyommatus;Polyommatus icarus;Netherlands;Bold;NLLEA1448-14;-
Lepidoptera;Pieridae;Pontia;Pontia daplidice;Austria;Bold;LEASS958-17;-
Lepidoptera;Pieridae;Pontia;Pontia edusa;Austria;Bold;LEASS866-17;-
Lepidoptera;Pieridae;Pontia;Pontia edusa;Austria;Bold;LEASS482-17;-
Lepidoptera;Pieridae;Pontia;Pontia edusa;Austria;Bold;LEASS867-17;-
Lepidoptera;Lycaenidae;Pseudophilotes;Pseudophilotes baton;Switzerland;Bold;LEPAA792-16;-
Lepidoptera;Lycaenidae;Pseudophilotes;Pseudophilotes vicrama;Austria;Bold;ABOLD479-16;-
Lepidoptera;Lycaenidae;Pseudophilotes;Pseudophilotes vicrama;Austria;Bold;LEASS968-17;-
Lepidoptera;Hesperiidae;Pyrgus;Pyrgus alveus;Norway;Bold;LON5689-17;-
Lepidoptera;Hesperiidae;Pyrgus;Pyrgus alveus;Switzerland;Bold;LEPAA754-16;-
Lepidoptera;Hesperiidae;Pyrgus;Pyrgus armoricanus;Austria;Bold;LEASS832-17;-
Lepidoptera;Hesperiidae;Pyrgus;Pyrgus armoricanus;Switzerland;Bold;LEPAA565-16;-
Lepidoptera;Hesperiidae;Pyrgus;Pyrgus armoricanus;Switzerland;Bold;LEPAA655-16;-
Lepidoptera;Hesperiidae;Pyrgus;Pyrgus armoricanus;Austria;Bold;LEASS922-17;-
Lepidoptera;Hesperiidae;Pyrgus;Pyrgus carlinae;Switzerland;Bold;LEPAA801-16;-
Lepidoptera;Hesperiidae;Pyrgus;Pyrgus carthami;Austria;Bold;LEASS751-17;-
Lepidoptera;Hesperiidae;Pyrgus;Pyrgus warrenensis;Switzerland;Bold;LEPAA805-16;-
Lepidoptera;Nymphalidae;Pyronia;Pyronia tithonus;Switzerland;Bold;LEPPA1180-17;-
Lepidoptera;Nymphalidae;Pyronia;Pyronia tithonus;Switzerland;Bold;LEPPA1179-17;-
Lepidoptera;Nymphalidae;Pyronia;Pyronia tithonus;Switzerland;Bold;LEPAA652-16;-
Lepidoptera;Lycaenidae;Satyrium;Satyrium acaciae;Austria;Bold;LEASS1029-17;-
Lepidoptera;Lycaenidae;Satyrium;Satyrium ilicis;Italy;Bold;LEATG394-14;-
Lepidoptera;Lycaenidae;Satyrium;Satyrium ilicis;Austria;Bold;LEASS1030-17;-
Lepidoptera;Lycaenidae;Satyrium;Satyrium ilicis;Switzerland;Bold;LEPAA794-16;-
Lepidoptera;Lycaenidae;Satyrium;Satyrium pruni;Austria;Bold;ABOLD644-17;-
Lepidoptera;Lycaenidae;Satyrium;Satyrium spini;Austria;Bold;LEASS1032-17;-
Lepidoptera;Lycaenidae;Satyrium;Satyrium spini;Switzerland;Bold;LEPAA795-16;-
Lepidoptera;Lycaenidae;Satyrium;Satyrium w-album;Switzerland;Bold;LEPAA706-16;-
Lepidoptera;Lycaenidae;Satyrium;Satyrium w-album;Switzerland;Bold;LEPAA862-16;-
Lepidoptera;Lycaenidae;Scolitantides;Scolitantides orion;Switzerland;Bold;LEPAA611-16;-
Lepidoptera;Lycaenidae;Scolitantides;Scolitantides orion;Switzerland;Bold;LEPAA166-16;-
Lepidoptera;Lycaenidae;Scolitantides;Scolitantides orion;Switzerland;Bold;LEPAA789-16;-
Lepidoptera;Hesperiidae;Spialia;Spialia sertorius;Austria;Bold;ABOLB167-15;-
Lepidoptera;Hesperiidae;Spialia;Spialia sertorius;Switzerland;Bold;LEPAA851-16;-
Lepidoptera;Hesperiidae;Spialia;Spialia sertorius;Italy;Bold;LEATG408-14;-
Lepidoptera;Lycaenidae;Thecla;Thecla betulae;Austria;Bold;LEASS550-17;-
Lepidoptera;Hesperiidae;Thymelicus;Thymelicus lineola;Austria;Bold;LEATJ1232-16;-
Lepidoptera;Hesperiidae;Thymelicus;Thymelicus lineola;Switzerland;Bold;LEPPA1236-17;-
Lepidoptera;Hesperiidae;Thymelicus;Thymelicus lineola;Switzerland;Bold;LEPAA545-16;-
Lepidoptera;Hesperiidae;Thymelicus;Thymelicus lineola;Netherlands;Bold;NLLEA1477-14;-
Lepidoptera;Hesperiidae;Thymelicus;Thymelicus sylvestris;Switzerland;Bold;LEPAA414-16;-
Lepidoptera;Hesperiidae;Thymelicus;Thymelicus sylvestris;Switzerland;Bold;LEPPA1240-17;-
Lepidoptera;Hesperiidae;Thymelicus;Thymelicus sylvestris;Switzerland;Bold;LEPPA1238-17;-
Lepidoptera;Hesperiidae;Thymelicus;Thymelicus sylvestris;Switzerland;Bold;LEPPA1239-17;-
Lepidoptera;Nymphalidae;Vanessa;Vanessa cardui;Italy;Bold;PHLAI557-13;-
Lepidoptera;Papilionidae;Zerynthia;Zerynthia polyxena;Austria;Bold;LEASS540-17;-
Odonata;Aeshnidae;Aeshna;Aeshna affinis;Italy;Bold;ZPLOD002-20;-
Odonata;Aeshnidae;Aeshna;Aeshna affinis;Italy;Bold;ZPLOD006-20;-
Odonata;Aeshnidae;Aeshna;Aeshna affinis;Italy;Bold;ZPLOD008-20;-
Odonata;Aeshnidae;Aeshna;Aeshna affinis;Italy;Bold;ZPLOD005-20;-
Odonata;Aeshnidae;Aeshna;Aeshna affinis;Italy;Bold;ZPLOD003-20;-
Odonata;Aeshnidae;Aeshna;Aeshna affinis;Italy;Bold;ZPLOD009-20;-
Odonata;Aeshnidae;Aeshna;Aeshna cyanea;Germany;Bold;FBAQU476-10;-
Odonata;Aeshnidae;Aeshna;Aeshna cyanea;Italy;Bold;ZPLOD011-20;-
Odonata;Aeshnidae;Aeshna;Aeshna cyanea;Italy;Bold;ZPLOD015-20;-
Odonata;Aeshnidae;Aeshna;Aeshna cyanea;Germany;Bold;FBAQU1602-13;-
Odonata;Aeshnidae;Aeshna;Aeshna cyanea;Germany;Bold;FBAQU1535-13;-
Odonata;Aeshnidae;Aeshna;Aeshna cyanea;Italy;Bold;ZPLOD014-20;-
Odonata;Aeshnidae;Aeshna;Aeshna cyanea;Germany;Bold;FBAQU1589-13;-
Odonata;Aeshnidae;Aeshna;Aeshna cyanea;Italy;Bold;ZPLOD013-20;-
Odonata;Aeshnidae;Aeshna;Aeshna cyanea;Germany;Bold;FBAQU477-10;-
Odonata;Aeshnidae;Aeshna;Aeshna cyanea;Italy;Bold;ZPLOD012-20;-
Odonata;Aeshnidae;Aeshna;Aeshna grandis;Germany;Bold;FBAQU1536-13;-
Odonata;Aeshnidae;Aeshna;Aeshna grandis;United Kingdom;Bold;ANBIO201-19;-
Odonata;Aeshnidae;Aeshna;Aeshna grandis;United Kingdom;Bold;DTNHM3378-21;-
Odonata;Aeshnidae;Aeshna;Aeshna juncea;Germany;Bold;FBAQU1538-13;-
Odonata;Aeshnidae;Aeshna;Aeshna juncea;Italy;Bold;ZPLOD027-20;-
Odonata;Aeshnidae;Aeshna;Aeshna juncea;Italy;Bold;ZPLOD029-20;-
Odonata;Aeshnidae;Aeshna;Aeshna juncea;Italy;Bold;ZPLOD031-20;-
Odonata;Aeshnidae;Aeshna;Aeshna juncea;Italy;Bold;ZPLOD032-20;-
Odonata;Aeshnidae;Aeshna;Aeshna mixta;Germany;Bold;FBAQU1539-13;-
Odonata;Aeshnidae;Aeshna;Aeshna mixta;Italy;Bold;ZPLOD035-20;-

Odonata;Aeshnidae;Aeshna;Aeshna mixta;Italy;Bold;ZPLOD037-20;-
Odonata;Aeshnidae;Aeshna;Aeshna mixta;Austria;Bold;FBAQU1564-13;-
Odonata;Aeshnidae;Aeshna;Aeshna mixta;Italy;Bold;ZPLOD036-20;-
Odonata;Aeshnidae;Aeshna;Aeshna subarctica;Italy;Bold;ZPLOD039-20;-
Odonata;Aeshnidae;Anax;Anax ephippiger;Italy;Bold;ZPLOD043-20;-
Odonata;Aeshnidae;Anax;Anax imperator;Italy;Bold;ZPLOD047-20;-
Odonata;Aeshnidae;Anax;Anax imperator;Italy;Bold;ZPLOD054-20;-
Odonata;Aeshnidae;Anax;Anax imperator;Italy;Bold;ZPLOD055-20;-
Odonata;Aeshnidae;Anax;Anax imperator;Italy;Bold;ZPLOD052-20;-
Odonata;Aeshnidae;Anax;Anax imperator;Germany;Bold;FBAQU1540-13;-
Odonata;Aeshnidae;Anax;Anax imperator;United Kingdom;Bold;DTNHM3008-21;-
Odonata;Aeshnidae;Anax;Anax imperator;Italy;Bold;ZPLOD050-20;-
Odonata;Aeshnidae;Anax;Anax parthenope;Italy;Bold;ZPLOD059-20;-
Odonata;Aeshnidae;Anax;Anax parthenope;Italy;Bold;ZPLOD061-20;-
Odonata;Aeshnidae;Boyeria;Boyeria irene;Italy;Bold;ZPLOD067-20;-
Odonata;Aeshnidae;Boyeria;Boyeria irene;Italy;Bold;ZPLOD064-20;-
Odonata;Aeshnidae;Boyeria;Boyeria irene;Italy;Bold;ZPLOD069-20;-
Odonata;Aeshnidae;Boyeria;Boyeria irene;Italy;Bold;ZPLOD065-20;-
Odonata;Aeshnidae;Boyeria;Boyeria irene;Italy;Bold;ZPLOD071-20;-
Odonata;Libellulidae;Brachythemis;Brachythemis impartita;Italy;Bold;ZPLOD077-20;-
Odonata;Libellulidae;Brachythemis;Brachythemis impartita;Italy;Bold;ZPLOD074-20;-
Odonata;Libellulidae;Brachythemis;Brachythemis impartita;Italy;Bold;ZPLOD076-20;-
Odonata;Aeshnidae;Brachytron;Brachytron pratense;United Kingdom;Bold;DTNHM663-21;-
Odonata;Aeshnidae;Brachytron;Brachytron pratense;Germany;Bold;FBAQU1541-13;-
Odonata;Aeshnidae;Brachytron;Brachytron pratense;Italy;Bold;ZPLOD080-20;-
Odonata;Calopterygidae;Calopteryx;Calopteryx haemorrhoidalis;Italy;Bold;ZPLOD092-20;-
Odonata;Calopterygidae;Calopteryx;Calopteryx haemorrhoidalis;Italy;Bold;ZPLOD094-20;-
Odonata;Calopterygidae;Calopteryx;Calopteryx haemorrhoidalis;Italy;Bold;ZPLOD089-20;-
Odonata;Calopterygidae;Calopteryx;Calopteryx haemorrhoidalis;Italy;Bold;ZPLOD097-20;-
Odonata;Calopterygidae;Calopteryx;Calopteryx haemorrhoidalis;Italy;Bold;ZPLOD093-20;-
Odonata;Calopterygidae;Calopteryx;Calopteryx haemorrhoidalis;Italy;Bold;ZPLOD088-20;-
Odonata;Calopterygidae;Calopteryx;Calopteryx haemorrhoidalis;Italy;Bold;ZPLOD087-20;-
Odonata;Calopterygidae;Calopteryx;Calopteryx haemorrhoidalis;Italy;Bold;ZPLOD099-20;-
Odonata;Calopterygidae;Calopteryx;Calopteryx haemorrhoidalis;Italy;Bold;ZPLOD100-20;-
Odonata;Calopterygidae;Calopteryx;Calopteryx haemorrhoidalis;Italy;Bold;ZPLOD090-20;-
Odonata;Calopterygidae;Calopteryx;Calopteryx splendens;Italy;Bold;ZPLOD111-20;-
Odonata;Calopterygidae;Calopteryx;Calopteryx splendens;Germany;Bold;FBAQU1582-13;-
Odonata;Calopterygidae;Calopteryx;Calopteryx splendens;Germany;Bold;FBAQU1600-13;-
Odonata;Calopterygidae;Calopteryx;Calopteryx splendens;Germany;Bold;FBAQU309-09;-
Odonata;Calopterygidae;Calopteryx;Calopteryx splendens;Germany;Bold;FBAQU524-10;-
Odonata;Calopterygidae;Calopteryx;Calopteryx splendens;Germany;Bold;FBAQU523-10;-
Odonata;Calopterygidae;Calopteryx;Calopteryx splendens;United Kingdom;Bold;DTNHM2510-21;-
Odonata;Calopterygidae;Calopteryx;Calopteryx splendens;Germany;Bold;FBAQU482-10;-
Odonata;Calopterygidae;Calopteryx;Calopteryx splendens;Italy;Bold;ZPLOD112-20;-
Odonata;Calopterygidae;Calopteryx;Calopteryx splendens;Italy;Bold;ZPLOD109-20;-
Odonata;Calopterygidae;Calopteryx;Calopteryx splendens;Italy;Bold;ZPLOD108-20;-
Odonata;Calopterygidae;Calopteryx;Calopteryx splendens;Germany;Bold;FBAQU1521-13;-
Odonata;Calopterygidae;Calopteryx;Calopteryx splendens;Italy;Bold;ZPLOD106-20;-
Odonata;Calopterygidae;Calopteryx;Calopteryx splendens;Italy;Bold;ZPLOD104-20;-
Odonata;Calopterygidae;Calopteryx;Calopteryx splendens;Germany;Bold;FBAQU122-09;-
Odonata;Calopterygidae;Calopteryx;Calopteryx splendens;Italy;Bold;ZPLOD107-20;-
Odonata;Calopterygidae;Calopteryx;Calopteryx splendens;Italy;Bold;ZPLOD105-20;-
Odonata;Calopterygidae;Calopteryx;Calopteryx splendens;Italy;Bold;ZPLOD101-20;-
Odonata;Calopterygidae;Calopteryx;Calopteryx virgo;Italy;Bold;ZPLOD121-20;-
Odonata;Calopterygidae;Calopteryx;Calopteryx virgo;Germany;Bold;FBAQU483-10;-
Odonata;Calopterygidae;Calopteryx;Calopteryx virgo;United Kingdom;Bold;DTNHM3773-21;-
Odonata;Calopterygidae;Calopteryx;Calopteryx virgo;Italy;Bold;ZPLOD120-20;-
Odonata;Calopterygidae;Calopteryx;Calopteryx virgo;Italy;Bold;ZPLOD122-20;-
Odonata;Calopterygidae;Calopteryx;Calopteryx virgo;Italy;Bold;ZPLOD116-20;-
Odonata;Calopterygidae;Calopteryx;Calopteryx virgo;Germany;Bold;FBAQU525-10;-
Odonata;Calopterygidae;Calopteryx;Calopteryx virgo;Germany;Bold;FBAQU1522-13;-
Odonata;Calopterygidae;Calopteryx;Calopteryx virgo;Italy;Bold;ZPLOD118-20;-
Odonata;Calopterygidae;Calopteryx;Calopteryx xanthostoma;Italy;Bold;ZPLOD126-20;-
Odonata;Coenagrionidae;Ceriagrion;Ceriagrion tenellum;Italy;Bold;ZPLOD132-20;-
Odonata;Coenagrionidae;Ceriagrion;Ceriagrion tenellum;Italy;Bold;ZPLOD133-20;-
Odonata;Coenagrionidae;Ceriagrion;Ceriagrion tenellum;Italy;Bold;ZPLOD139-20;-
Odonata;Coenagrionidae;Ceriagrion;Ceriagrion tenellum;Italy;Bold;ZPLOD137-20;-
Odonata;Coenagrionidae;Ceriagrion;Ceriagrion tenellum;Italy;Bold;ZPLOD131-20;-
Odonata;Coenagrionidae;Ceriagrion;Ceriagrion tenellum;Italy;Bold;ZPLOD128-20;-
Odonata;Coenagrionidae;Ceriagrion;Ceriagrion tenellum;Italy;Bold;ZPLOD136-20;-
Odonata;Lestidae;Chalcolestes;Chalcolestes parvidens;Italy;Bold;ZPLOD144-20;-
Odonata;Lestidae;Chalcolestes;Chalcolestes parvidens;Italy;Bold;ZPLOD145-20;-
Odonata;Lestidae;Chalcolestes;Chalcolestes parvidens;Italy;Bold;ZPLOD146-20;-
Odonata;Lestidae;Chalcolestes;Chalcolestes viridis;Germany;Bold;FBAQU498-10;-
Odonata;Lestidae;Chalcolestes;Chalcolestes viridis;Italy;Bold;ZPLOD176-20;-
Odonata;Lestidae;Chalcolestes;Chalcolestes viridis;Italy;Bold;ZPLOD161-20;-
Odonata;Lestidae;Chalcolestes;Chalcolestes viridis;Italy;Bold;ZPLOD184-20;-
Odonata;Lestidae;Chalcolestes;Chalcolestes viridis;Italy;Bold;ZPLOD170-20;-

Odonata;Lestidae;Chalcolestes;Chalcolestes viridis;Italy;Bold;ZPLOD175-20;-
Odonata;Lestidae;Chalcolestes;Chalcolestes viridis;Italy;Bold;ZPLOD169-20;-
Odonata;Lestidae;Chalcolestes;Chalcolestes viridis;Italy;Bold;ZPLOD185-20;-
Odonata;Lestidae;Chalcolestes;Chalcolestes viridis;Germany;Bold;FBAQU1524-13;-
Odonata;Lestidae;Chalcolestes;Chalcolestes viridis;Italy;Bold;ZPLOD171-20;-
Odonata;Lestidae;Chalcolestes;Chalcolestes viridis;Italy;Bold;ZPLOD188-20;-
Odonata;Lestidae;Chalcolestes;Chalcolestes viridis;Italy;Bold;ZPLOD178-20;-
Odonata;Lestidae;Chalcolestes;Chalcolestes viridis;Italy;Bold;ZPLOD172-20;-
Odonata;Lestidae;Chalcolestes;Chalcolestes viridis;Italy;Bold;ZPLOD182-20;-
Odonata;Lestidae;Chalcolestes;Chalcolestes viridis;Austria;Bold;FBAQU1562-13;-
Odonata;Lestidae;Chalcolestes;Chalcolestes viridis;Italy;Bold;ZPLOD183-20;-
Odonata;Lestidae;Chalcolestes;Chalcolestes viridis;Italy;Bold;ZPLOD162-20;-
Odonata;Lestidae;Chalcolestes;Chalcolestes viridis;Italy;Bold;ZPLOD187-20;-
Odonata;Lestidae;Chalcolestes;Chalcolestes viridis;Italy;Bold;ZPLOD180-20;-
Odonata;Lestidae;Chalcolestes;Chalcolestes viridis;Germany;Bold;FBAQU499-10;-
Odonata;Lestidae;Chalcolestes;Chalcolestes viridis;Italy;Bold;ZPLOD163-20;-
Odonata;Lestidae;Chalcolestes;Chalcolestes viridis;Italy;Bold;ZPLOD166-20;-
Odonata;Coenagrionidae;Coenagrion;Coenagrion caerulescens;Italy;Bold;ZPLOD199-20;-
Odonata;Coenagrionidae;Coenagrion;Coenagrion caerulescens;Italy;Bold;ZPLOD191-20;-
Odonata;Coenagrionidae;Coenagrion;Coenagrion caerulescens;Italy;Bold;ZPLOD193-20;-
Odonata;Coenagrionidae;Coenagrion;Coenagrion hastulatum;Italy;Bold;ZPLOD200-20;-
Odonata;Coenagrionidae;Coenagrion;Coenagrion hastulatum;Germany;Bold;FBAQU485-10;-
Odonata;Coenagrionidae;Coenagrion;Coenagrion hastulatum;Germany;Bold;FBAQU1531-13;-
Odonata;Coenagrionidae;Coenagrion;Coenagrion mercuriale;Germany;Bold;FBAQU486-10;-
Odonata;Coenagrionidae;Coenagrion;Coenagrion mercuriale;Germany;Bold;FBAQU557-10;-
Odonata;Coenagrionidae;Coenagrion;Coenagrion mercuriale;Germany;Bold;FBAQU558-10;-
Odonata;Coenagrionidae;Coenagrion;Coenagrion mercuriale;Italy;Bold;ZPLOD205-20;-
Odonata;Coenagrionidae;Coenagrion;Coenagrion mercuriale;Italy;Bold;ZPLOD206-20;-
Odonata;Coenagrionidae;Coenagrion;Coenagrion ornatum;Italy;Bold;ZPLOD215-20;-
Odonata;Coenagrionidae;Coenagrion;Coenagrion puella;Germany;Bold;FBAQU1533-13;-
Odonata;Coenagrionidae;Coenagrion;Coenagrion puella;Italy;Bold;ZPLOD226-20;-
Odonata;Coenagrionidae;Coenagrion;Coenagrion puella;Germany;Bold;FBAQU1556-13;-
Odonata;Coenagrionidae;Coenagrion;Coenagrion puella;Germany;Bold;FBAQU487-10;-
Odonata;Coenagrionidae;Coenagrion;Coenagrion puella;Germany;Bold;FBAQU1555-13;-
Odonata;Coenagrionidae;Coenagrion;Coenagrion puella;Italy;Bold;ZPLOD225-20;-
Odonata;Coenagrionidae;Coenagrion;Coenagrion puella;Italy;Bold;ZPLOD244-20;-
Odonata;Coenagrionidae;Coenagrion;Coenagrion puella;Italy;Bold;ZPLOD248-20;-
Odonata;Coenagrionidae;Coenagrion;Coenagrion puella;Italy;Bold;ZPLOD247-20;-
Odonata;Coenagrionidae;Coenagrion;Coenagrion puella;United Kingdom;Bold;ANBIO134-19;-
Odonata;Coenagrionidae;Coenagrion;Coenagrion puella;Italy;Bold;ZPLOD229-20;-
Odonata;Coenagrionidae;Coenagrion;Coenagrion puella;Italy;Bold;ZPLOD224-20;-
Odonata;Coenagrionidae;Coenagrion;Coenagrion puella;Italy;Bold;ZPLOD217-20;-
Odonata;Coenagrionidae;Coenagrion;Coenagrion puella;Italy;Bold;ZPLOD236-20;-
Odonata;Coenagrionidae;Coenagrion;Coenagrion puella;Italy;Bold;ZPLOD235-20;-
Odonata;Coenagrionidae;Coenagrion;Coenagrion puella;Italy;Bold;ZPLOD250-20;-
Odonata;Coenagrionidae;Coenagrion;Coenagrion puella;Italy;Bold;ZPLOD216-20;-
Odonata;Coenagrionidae;Coenagrion;Coenagrion puella;Italy;Bold;ZPLOD242-20;-
Odonata;Coenagrionidae;Coenagrion;Coenagrion puella;Italy;Bold;ZPLOD219-20;-
Odonata;Coenagrionidae;Coenagrion;Coenagrion pulchellum;United Kingdom;Bold;DTNHM2159-21;-
Odonata;Coenagrionidae;Coenagrion;Coenagrion pulchellum;Germany;Bold;FBAQU1534-13;-
Odonata;Coenagrionidae;Coenagrion;Coenagrion pulchellum;Germany;Bold;FBAQU1430-13;-
Odonata;Coenagrionidae;Coenagrion;Coenagrion scitulum;Italy;Bold;ZPLOD286-20;-
Odonata;Coenagrionidae;Coenagrion;Coenagrion scitulum;Italy;Bold;ZPLOD283-20;-
Odonata;Coenagrionidae;Coenagrion;Coenagrion scitulum;Italy;Bold;ZPLOD280-20;-
Odonata;Coenagrionidae;Coenagrion;Coenagrion scitulum;Italy;Bold;ZPLOD268-20;-
Odonata;Coenagrionidae;Coenagrion;Coenagrion scitulum;Italy;Bold;ZPLOD265-20;-
Odonata;Coenagrionidae;Coenagrion;Coenagrion scitulum;Italy;Bold;ZPLOD282-20;-
Odonata;Coenagrionidae;Coenagrion;Coenagrion scitulum;Italy;Bold;ZPLOD284-20;-
Odonata;Coenagrionidae;Coenagrion;Coenagrion scitulum;Italy;Bold;ZPLOD271-20;-
Odonata;Coenagrionidae;Coenagrion;Coenagrion scitulum;Italy;Bold;ZPLOD277-20;-
Odonata;Coenagrionidae;Coenagrion;Coenagrion scitulum;Italy;Bold;ZPLOD279-20;-
Odonata;Coenagrionidae;Coenagrion;Coenagrion scitulum;Italy;Bold;ZPLOD270-20;-
Odonata;Coenagrionidae;Coenagrion;Coenagrion scitulum;Italy;Bold;ZPLOD276-20;-
Odonata;Coenagrionidae;Coenagrion;Coenagrion scitulum;Italy;Bold;ZPLOD267-20;-
Odonata;Coenagrionidae;Coenagrion;Coenagrion scitulum;Italy;Bold;ZPLOD274-20;-
Odonata;Coenagrionidae;Coenagrion;Coenagrion scitulum;Italy;Bold;ZPLOD269-20;-
Odonata;Coenagrionidae;Coenagrion;Coenagrion scitulum;Italy;Bold;ZPLOD281-20;-
Odonata;Coenagrionidae;Coenagrion;Coenagrion scitulum;Italy;Bold;ZPLOD285-20;-
Odonata;Cordulegastridae;Cordulegaster;Cordulegaster bidentata;Germany;Bold;FBAQU560-10;-
Odonata;Cordulegastridae;Cordulegaster;Cordulegaster bidentata;Italy;Bold;ZPLOD291-20;-
Odonata;Cordulegastridae;Cordulegaster;Cordulegaster bidentata;Italy;Bold;ZPLOD290-20;-
Odonata;Cordulegastridae;Cordulegaster;Cordulegaster bidentata;Italy;Bold;ZPLOD292-20;-
Odonata;Cordulegastridae;Cordulegaster;Cordulegaster heros;Italy;Bold;ZPLOD305-20;-
Odonata;Cordulegastridae;Cordulegaster;Cordulegaster trinacriae;Italy;Bold;ZPLOD313-20;-
Odonata;Corduliidae;Cordulia;Cordulia aenea;Germany;Bold;FBAQU1433-13;-
Odonata;Corduliidae;Cordulia;Cordulia aenea;Germany;Bold;FBAQU1544-13;-
Odonata;Corduliidae;Cordulia;Cordulia aenea;Germany;Bold;FBAQU1432-13;-
Odonata;Corduliidae;Cordulia;Cordulia aenea;Italy;Bold;ZPLOD318-20;-

Odonata;Libellulidae;Crocothemis;Crocothemis erythraea;Italy;Bold;ZPLOD324-20;-
Odonata;Libellulidae;Crocothemis;Crocothemis erythraea;Italy;Bold;ZPLOD326-20;-
Odonata;Libellulidae;Crocothemis;Crocothemis erythraea;Italy;Bold;ZPLOD325-20;-
Odonata;Libellulidae;Crocothemis;Crocothemis erythraea;Italy;Bold;ZPLOD322-20;-
Odonata;Libellulidae;Crocothemis;Crocothemis erythraea;Italy;Bold;ZPLOD323-20;-
Odonata;Libellulidae;Crocothemis;Crocothemis erythraea;Italy;Bold;ZPLOD321-20;-
Odonata;Libellulidae;Crocothemis;Crocothemis erythraea;Italy;Bold;ZPLOD330-20;-
Odonata;Libellulidae;Crocothemis;Crocothemis erythraea;Italy;Bold;ZPLOD327-20;-
Odonata;Libellulidae;Crocothemis;Crocothemis erythraea;Italy;Bold;ZPLOD329-20;-
Odonata;Coenagrionidae;Enallagma;Enallagma cyathigerum;Italy;Bold;ZPLOD347-20;-
Odonata;Coenagrionidae;Enallagma;Enallagma cyathigerum;Italy;Bold;ZPLOD338-20;-
Odonata;Coenagrionidae;Enallagma;Enallagma cyathigerum;Italy;Bold;ZPLOD340-20;-
Odonata;Coenagrionidae;Enallagma;Enallagma cyathigerum;Italy;Bold;ZPLOD341-20;-
Odonata;Coenagrionidae;Enallagma;Enallagma cyathigerum;Italy;Bold;ZPLOD337-20;-
Odonata;Coenagrionidae;Enallagma;Enallagma cyathigerum;Italy;Bold;ZPLOD339-20;-
Odonata;Coenagrionidae;Enallagma;Enallagma cyathigerum;Italy;Bold;ZPLOD342-20;-
Odonata;Coenagrionidae;Enallagma;Enallagma cyathigerum;United Kingdom;Bold;UKAN249-22;-
Odonata;Coenagrionidae;Enallagma;Enallagma cyathigerum;Germany;Bold;FBAQU1530-13;-
Odonata;Coenagrionidae;Enallagma;Enallagma cyathigerum;Italy;Bold;ZPLOD350-20;-
Odonata;Coenagrionidae;Enallagma;Enallagma cyathigerum;Italy;Bold;ZPLOD344-20;-
Odonata;Coenagrionidae;Enallagma;Enallagma cyathigerum;Italy;Bold;ZPLOD349-20;-
Odonata;Corduliidae;Epitheca;Epitheca bimaculata;Germany;Bold;FBAQU1437-13;-
Odonata;Corduliidae;Epitheca;Epitheca bimaculata;Germany;Bold;FBAQU1436-13;-
Odonata;Coenagrionidae;Erythromma;Erythromma lindenii;Italy;Bold;ZPLOD363-20;-
Odonata;Coenagrionidae;Erythromma;Erythromma lindenii;Italy;Bold;ZPLOD356-20;-
Odonata;Coenagrionidae;Erythromma;Erythromma lindenii;Italy;Bold;ZPLOD402-20;-
Odonata;Coenagrionidae;Erythromma;Erythromma lindenii;Italy;Bold;ZPLOD403-20;-
Odonata;Coenagrionidae;Erythromma;Erythromma lindenii;Italy;Bold;ZPLOD361-20;-
Odonata;Coenagrionidae;Erythromma;Erythromma lindenii;Italy;Bold;ZPLOD368-20;-
Odonata;Coenagrionidae;Erythromma;Erythromma lindenii;Italy;Bold;ZPLOD404-20;-
Odonata;Coenagrionidae;Erythromma;Erythromma lindenii;Italy;Bold;ZPLOD400-20;-
Odonata;Coenagrionidae;Erythromma;Erythromma lindenii;Italy;Bold;ZPLOD382-20;-
Odonata;Coenagrionidae;Erythromma;Erythromma lindenii;Italy;Bold;ZPLOD357-20;-
Odonata;Coenagrionidae;Erythromma;Erythromma lindenii;Italy;Bold;ZPLOD372-20;-
Odonata;Coenagrionidae;Erythromma;Erythromma lindenii;Italy;Bold;ZPLOD359-20;-
Odonata;Coenagrionidae;Erythromma;Erythromma lindenii;Italy;Bold;ZPLOD371-20;-
Odonata;Coenagrionidae;Erythromma;Erythromma lindenii;Italy;Bold;ZPLOD353-20;-
Odonata;Coenagrionidae;Erythromma;Erythromma lindenii;Italy;Bold;ZPLOD362-20;-
Odonata;Coenagrionidae;Erythromma;Erythromma lindenii;Italy;Bold;ZPLOD360-20;-
Odonata;Coenagrionidae;Erythromma;Erythromma lindenii;Italy;Bold;ZPLOD398-20;-
Odonata;Coenagrionidae;Erythromma;Erythromma lindenii;Italy;Bold;ZPLOD369-20;-
Odonata;Coenagrionidae;Erythromma;Erythromma lindenii;Italy;Bold;ZPLOD370-20;-
Odonata;Coenagrionidae;Erythromma;Erythromma lindenii;Italy;Bold;ZPLOD405-20;-
Odonata;Coenagrionidae;Erythromma;Erythromma najas;Italy;Bold;ZPLOD408-20;-
Odonata;Coenagrionidae;Erythromma;Erythromma najas;Germany;Bold;FBAQU1529-13;-
Odonata;Coenagrionidae;Erythromma;Erythromma najas;Germany;Bold;FBAQU564-10;-
Odonata;Coenagrionidae;Erythromma;Erythromma viridulum;Italy;Bold;ZPLOD415-20;-
Odonata;Coenagrionidae;Erythromma;Erythromma viridulum;Italy;Bold;ZPLOD413-20;-
Odonata;Coenagrionidae;Erythromma;Erythromma viridulum;Italy;Bold;ZPLOD416-20;-
Odonata;Coenagrionidae;Erythromma;Erythromma viridulum;Italy;Bold;ZPLOD423-20;-
Odonata;Coenagrionidae;Erythromma;Erythromma viridulum;Italy;Bold;ZPLOD410-20;-
Odonata;Coenagrionidae;Erythromma;Erythromma viridulum;Germany;Bold;FBAQU493-10;-
Odonata;Coenagrionidae;Erythromma;Erythromma viridulum;Italy;Bold;ZPLOD420-20;-
Odonata;Coenagrionidae;Erythromma;Erythromma viridulum;Italy;Bold;ZPLOD411-20;-
Odonata;Coenagrionidae;Erythromma;Erythromma viridulum;Italy;Bold;ZPLOD418-20;-
Odonata;Gomphidae;Gomphus;Gomphus vulgatissimus;Germany;Bold;FBAQU566-10;-
Odonata;Gomphidae;Gomphus;Gomphus vulgatissimus;Italy;Bold;ZPLOD427-20;-
Odonata;Gomphidae;Gomphus;Gomphus vulgatissimus;Germany;Bold;FBAQU533-10;-
Odonata;Gomphidae;Gomphus;Gomphus vulgatissimus;Germany;Bold;FBAQU126-09;-
Odonata;Gomphidae;Gomphus;Gomphus vulgatissimus;Germany;Bold;FBAQU1595-13;-
Odonata;Gomphidae;Gomphus;Gomphus vulgatissimus;Germany;Bold;FBAQU1553-13;-
Odonata;Coenagrionidae;Ischnura;Ischnura elegans;Italy;Bold;ZPLOD429-20;-
Odonata;Coenagrionidae;Ischnura;Ischnura elegans;Italy;Bold;ZPLOD443-20;-
Odonata;Coenagrionidae;Ischnura;Ischnura elegans;United Kingdom;Bold;DTNHM2763-21;-
Odonata;Coenagrionidae;Ischnura;Ischnura elegans;Italy;Bold;ZPLOD428-20;-
Odonata;Coenagrionidae;Ischnura;Ischnura elegans;Italy;Bold;ZPLOD432-20;-
Odonata;Coenagrionidae;Ischnura;Ischnura elegans;Italy;Bold;ZPLOD440-20;-
Odonata;Coenagrionidae;Ischnura;Ischnura elegans;Italy;Bold;ZPLOD442-20;-
Odonata;Coenagrionidae;Ischnura;Ischnura elegans;Italy;Bold;ZPLOD436-20;-
Odonata;Coenagrionidae;Ischnura;Ischnura elegans;Italy;Bold;ZPLOD433-20;-
Odonata;Coenagrionidae;Ischnura;Ischnura elegans;Italy;Bold;ZPLOD446-20;-
Odonata;Coenagrionidae;Ischnura;Ischnura elegans;Italy;Bold;ZPLOD445-20;-
Odonata;Coenagrionidae;Ischnura;Ischnura elegans;Italy;Bold;ZPLOD437-20;-
Odonata;Coenagrionidae;Ischnura;Ischnura elegans;Italy;Bold;ZPLOD435-20;-
Odonata;Coenagrionidae;Ischnura;Ischnura elegans;Italy;Bold;ZPLOD434-20;-
Odonata;Coenagrionidae;Ischnura;Ischnura elegans;Italy;Bold;ZPLOD449-20;-
Odonata;Coenagrionidae;Ischnura;Ischnura elegans;Austria;Bold;FBAQU1563-13;-
Odonata;Coenagrionidae;Ischnura;Ischnura elegans;Germany;Bold;FBAQU1610-13;-

Odonata;Coenagrionidae;Ischnura;Ischnura elegans;Italy;Bold;ZPLOD441-20;-
Odonata;Coenagrionidae;Ischnura;Ischnura elegans;Italy;Bold;ZPLOD448-20;-
Odonata;Coenagrionidae;Ischnura;Ischnura elegans;Italy;Bold;ZPLOD439-20;-
Odonata;Coenagrionidae;Ischnura;Ischnura genei;Italy;Bold;ZPLOD456-20;-
Odonata;Coenagrionidae;Ischnura;Ischnura genei;Italy;Bold;ZPLOD450-20;-
Odonata;Coenagrionidae;Ischnura;Ischnura genei;Italy;Bold;ZPLOD451-20;-
Odonata;Coenagrionidae;Ischnura;Ischnura genei;Italy;Bold;ZPLOD458-20;-
Odonata;Coenagrionidae;Ischnura;Ischnura genei;Italy;Bold;ZPLOD453-20;-
Odonata;Coenagrionidae;Ischnura;Ischnura genei;Italy;Bold;ZPLOD455-20;-
Odonata;Coenagrionidae;Ischnura;Ischnura pumilio;Italy;Bold;ZPLOD465-20;-
Odonata;Coenagrionidae;Ischnura;Ischnura pumilio;Italy;Bold;ZPLOD464-20;-
Odonata;Coenagrionidae;Ischnura;Ischnura pumilio;Italy;Bold;ZPLOD463-20;-
Odonata;Coenagrionidae;Ischnura;Ischnura pumilio;Italy;Bold;ZPLOD459-20;-
Odonata;Lestidae;Lestes;Lestes barbarus;Italy;Bold;ZPLOD473-20;-
Odonata;Lestidae;Lestes;Lestes barbarus;Germany;Bold;FBAQU316-09;-
Odonata;Lestidae;Lestes;Lestes barbarus;Italy;Bold;ZPLOD471-20;-
Odonata;Lestidae;Lestes;Lestes barbarus;Italy;Bold;ZPLOD469-20;-
Odonata;Lestidae;Lestes;Lestes barbarus;Germany;Bold;FBAQU173-09;-
Odonata;Lestidae;Lestes;Lestes barbarus;Italy;Bold;ZPLOD470-20;-
Odonata;Lestidae;Lestes;Lestes barbarus;Italy;Bold;ZPLOD477-20;-
Odonata;Lestidae;Lestes;Lestes barbarus;Italy;Bold;ZPLOD467-20;-
Odonata;Lestidae;Lestes;Lestes barbarus;Italy;Bold;ZPLOD472-20;-
Odonata;Lestidae;Lestes;Lestes barbarus;Romania;Bold;RODI024-20;-
Odonata;Lestidae;Lestes;Lestes dryas;Italy;Bold;ZPLOD479-20;-
Odonata;Lestidae;Lestes;Lestes dryas;Italy;Bold;ZPLOD483-20;-
Odonata;Lestidae;Lestes;Lestes dryas;Italy;Bold;ZPLOD480-20;-
Odonata;Lestidae;Lestes;Lestes dryas;Italy;Bold;ZPLOD487-20;-
Odonata;Lestidae;Lestes;Lestes macrostigma;Italy;Bold;ZPLOD490-20;-
Odonata;Lestidae;Lestes;Lestes macrostigma;Italy;Bold;ZPLOD489-20;-
Odonata;Lestidae;Lestes;Lestes sponsa;Italy;Bold;ZPLOD493-20;-
Odonata;Lestidae;Lestes;Lestes sponsa;Germany;Bold;FBAQU317-09;-
Odonata;Lestidae;Lestes;Lestes sponsa;Italy;Bold;ZPLOD495-20;-
Odonata;Lestidae;Lestes;Lestes sponsa;Germany;Bold;FBAQU539-10;-
Odonata;Lestidae;Lestes;Lestes sponsa;Italy;Bold;ZPLOD494-20;-
Odonata;Lestidae;Lestes;Lestes sponsa;Germany;Bold;FBAQU102-09;-
Odonata;Lestidae;Lestes;Lestes sponsa;Italy;Bold;ZPLOD497-20;-
Odonata;Lestidae;Lestes;Lestes sponsa;Italy;Bold;ZPLOD498-20;-
Odonata;Lestidae;Lestes;Lestes sponsa;Germany;Bold;FBAQU1525-13;-
Odonata;Lestidae;Lestes;Lestes sponsa;Germany;Bold;FBAQU497-10;-
Odonata;Lestidae;Lestes;Lestes virens;Germany;Bold;FBAQU174-09;-
Odonata;Lestidae;Lestes;Lestes virens;Italy;Bold;ZPLOD511-20;-
Odonata;Lestidae;Lestes;Lestes virens;Germany;Bold;FBAQU1526-13;-
Odonata;Lestidae;Lestes;Lestes virens;Italy;Bold;ZPLOD510-20;-
Odonata;Lestidae;Lestes;Lestes virens;Italy;Bold;ZPLOD507-20;-
Odonata;Lestidae;Lestes;Lestes virens;Germany;Bold;FBAQU540-10;-
Odonata;Lestidae;Lestes;Lestes virens;Italy;Bold;ZPLOD502-20;-
Odonata;Lestidae;Lestes;Lestes virens;Italy;Bold;ZPLOD508-20;-
Odonata;Lestidae;Lestes;Lestes virens;Italy;Bold;ZPLOD506-20;-
Odonata;Libellulidae;Leucorrhinia;Leucorrhinia dubia;Italy;Bold;ZPLOD514-20;-
Odonata;Libellulidae;Leucorrhinia;Leucorrhinia dubia;Italy;Bold;ZPLOD513-20;-
Odonata;Libellulidae;Leucorrhinia;Leucorrhinia dubia;Italy;Bold;ZPLOD516-20;-
Odonata;Libellulidae;Leucorrhinia;Leucorrhinia dubia;Germany;Bold;FBAQU1549-13;-
Odonata;Libellulidae;Leucorrhinia;Leucorrhinia dubia;Germany;Bold;FBAQU567-10;-
Odonata;Libellulidae;Leucorrhinia;Leucorrhinia dubia;Italy;Bold;ZPLOD521-20;-
Odonata;Libellulidae;Leucorrhinia;Leucorrhinia dubia;Italy;Bold;ZPLOD517-20;-
Odonata;Libellulidae;Leucorrhinia;Leucorrhinia dubia;Italy;Bold;ZPLOD518-20;-
Odonata;Libellulidae;Leucorrhinia;Leucorrhinia dubia;Italy;Bold;ZPLOD519-20;-
Odonata;Libellulidae;Libellula;Libellula depressa;Italy;Bold;ZPLOD525-20;-
Odonata;Libellulidae;Libellula;Libellula depressa;Italy;Bold;ZPLOD532-20;-
Odonata;Libellulidae;Libellula;Libellula depressa;Italy;Bold;ZPLOD529-20;-
Odonata;Libellulidae;Libellula;Libellula depressa;Italy;Bold;ZPLOD530-20;-
Odonata;Libellulidae;Libellula;Libellula depressa;Italy;Bold;ZPLOD533-20;-
Odonata;Libellulidae;Libellula;Libellula fulva;Italy;Bold;ZPLOD542-20;-
Odonata;Libellulidae;Libellula;Libellula fulva;Italy;Bold;ZPLOD539-20;-
Odonata;Libellulidae;Libellula;Libellula fulva;Italy;Bold;ZPLOD537-20;-
Odonata;Libellulidae;Libellula;Libellula fulva;Italy;Bold;ZPLOD540-20;-
Odonata;Libellulidae;Libellula;Libellula fulva;Italy;Bold;ZPLOD538-20;-
Odonata;Libellulidae;Libellula;Libellula quadrimaculata;United Kingdom;Bold;ANBIO194-19;-
Odonata;Libellulidae;Libellula;Libellula quadrimaculata;Germany;Bold;FBAQU318-09;-
Odonata;Libellulidae;Libellula;Libellula quadrimaculata;Germany;Bold;FBAQU1547-13;-
Odonata;Libellulidae;Libellula;Libellula quadrimaculata;Italy;Bold;ZPLOD548-20;-
Odonata;Libellulidae;Libellula;Libellula quadrimaculata;Italy;Bold;ZPLOD546-20;-
Odonata;Libellulidae;Libellula;Libellula quadrimaculata;Germany;Bold;FBAQU1511-13;-
Odonata;Gomphidae;Lindenia;Lindenia tetraphylla;Italy;Bold;ZPLOD551-20;-
Odonata;Gomphidae;Lindenia;Lindenia tetraphylla;Italy;Bold;ZPLOD554-20;-
Odonata;Coenagrionidae;Nehalennia;Nehalennia speciosa;Italy;Bold;ZPLOD559-20;-
Odonata;Coenagrionidae;Nehalennia;Nehalennia speciosa;Italy;Bold;ZPLOD558-20;-
Odonata;Gomphidae;Onychogomphus;Onychogomphus uncatus;Italy;Bold;ZPLOD584-20;-

Odonata;Gomphidae;Onychogomphus;Onychogomphus uncatus;Italy;Bold;ZPLOD583-20;-
Odonata;Gomphidae;Onychogomphus;Onychogomphus uncatus;Italy;Bold;ZPLOD586-20;-
Odonata;Gomphidae;Ophiogomphus;Ophiogomphus cecilia;Germany;Bold;FBAQU1599-13;-
Odonata;Gomphidae;Ophiogomphus;Ophiogomphus cecilia;Germany;Bold;FBAQU568-10;-
Odonata;Gomphidae;Ophiogomphus;Ophiogomphus cecilia;Germany;Bold;FBAQU1543-13;-
Odonata;Libellulidae;Orthetrum;Orthetrum albistylum;Italy;Bold;ZPLOD596-20;-
Odonata;Libellulidae;Orthetrum;Orthetrum albistylum;Hungary;Bold;FBAQU1438-13;-
Odonata;Libellulidae;Orthetrum;Orthetrum albistylum;Italy;Bold;ZPLOD593-20;-
Odonata;Libellulidae;Orthetrum;Orthetrum albistylum;Italy;Bold;ZPLOD595-20;-
Odonata;Libellulidae;Orthetrum;Orthetrum albistylum;Italy;Bold;ZPLOD591-20;-
Odonata;Libellulidae;Orthetrum;Orthetrum albistylum;Italy;Bold;ZPLOD590-20;-
Odonata;Libellulidae;Orthetrum;Orthetrum albistylum;Italy;Bold;ZPLOD592-20;-
Odonata;Libellulidae;Orthetrum;Orthetrum brunneum;Italy;Bold;ZPLOD601-20;-
Odonata;Libellulidae;Orthetrum;Orthetrum brunneum;Italy;Bold;ZPLOD608-20;-
Odonata;Libellulidae;Orthetrum;Orthetrum brunneum;Italy;Bold;ZPLOD606-20;-
Odonata;Libellulidae;Orthetrum;Orthetrum brunneum;Italy;Bold;ZPLOD607-20;-
Odonata;Libellulidae;Orthetrum;Orthetrum brunneum;Italy;Bold;ZPLOD599-20;-
Odonata;Libellulidae;Orthetrum;Orthetrum brunneum;Italy;Bold;ZPLOD597-20;-
Odonata;Libellulidae;Orthetrum;Orthetrum brunneum;Italy;Bold;ZPLOD602-20;-
Odonata;Libellulidae;Orthetrum;Orthetrum brunneum;Italy;Bold;ZPLOD604-20;-
Odonata;Libellulidae;Orthetrum;Orthetrum cancellatum;Italy;Bold;ZPLOD615-20;-
Odonata;Libellulidae;Orthetrum;Orthetrum cancellatum;Italy;Bold;ZPLOD621-20;-
Odonata;Libellulidae;Orthetrum;Orthetrum cancellatum;Italy;Bold;ZPLOD624-20;-
Odonata;Libellulidae;Orthetrum;Orthetrum cancellatum;Germany;Bold;FBAQU1548-13;-
Odonata;Libellulidae;Orthetrum;Orthetrum cancellatum;Italy;Bold;ZPLOD617-20;-
Odonata;Libellulidae;Orthetrum;Orthetrum cancellatum;Italy;Bold;ZPLOD622-20;-
Odonata;Libellulidae;Orthetrum;Orthetrum coerulescens;Italy;Bold;ZPLOD660-20;-
Odonata;Libellulidae;Orthetrum;Orthetrum coerulescens;Italy;Bold;ZPLOD655-20;-
Odonata;Libellulidae;Orthetrum;Orthetrum coerulescens;Italy;Bold;ZPLOD653-20;-
Odonata;Libellulidae;Orthetrum;Orthetrum coerulescens;Italy;Bold;ZPLOD657-20;-
Odonata;Libellulidae;Orthetrum;Orthetrum coerulescens;Italy;Bold;ZPLOD641-20;-
Odonata;Libellulidae;Orthetrum;Orthetrum coerulescens;Germany;Bold;FBAQU569-10;-
Odonata;Libellulidae;Orthetrum;Orthetrum coerulescens;Italy;Bold;ZPLOD629-20;-
Odonata;Libellulidae;Orthetrum;Orthetrum coerulescens;Italy;Bold;ZPLOD650-20;-
Odonata;Libellulidae;Orthetrum;Orthetrum coerulescens;Germany;Bold;FBAQU570-10;-
Odonata;Libellulidae;Orthetrum;Orthetrum coerulescens;Italy;Bold;ZPLOD634-20;-
Odonata;Libellulidae;Orthetrum;Orthetrum coerulescens;Italy;Bold;ZPLOD646-20;-
Odonata;Libellulidae;Orthetrum;Orthetrum coerulescens;Italy;Bold;ZPLOD654-20;-
Odonata;Libellulidae;Orthetrum;Orthetrum coerulescens;Italy;Bold;ZPLOD636-20;-
Odonata;Libellulidae;Orthetrum;Orthetrum coerulescens;Italy;Bold;ZPLOD633-20;-
Odonata;Libellulidae;Orthetrum;Orthetrum coerulescens;Italy;Bold;ZPLOD635-20;-
Odonata;Libellulidae;Orthetrum;Orthetrum coerulescens;Italy;Bold;ZPLOD652-20;-
Odonata;Libellulidae;Orthetrum;Orthetrum coerulescens;Italy;Bold;ZPLOD630-20;-
Odonata;Libellulidae;Orthetrum;Orthetrum coerulescens;Italy;Bold;ZPLOD631-20;-
Odonata;Libellulidae;Orthetrum;Orthetrum coerulescens;Italy;Bold;ZPLOD637-20;-
Odonata;Libellulidae;Orthetrum;Orthetrum coerulescens;Italy;Bold;ZPLOD638-20;-
Odonata;Libellulidae;Orthetrum;Orthetrum coerulescens;Germany;Bold;FBAQU1585-13;-
Odonata;Libellulidae;Orthetrum;Orthetrum coerulescens;Germany;Bold;FBAQU1441-13;-
Odonata;Libellulidae;Orthetrum;Orthetrum coerulescens;Italy;Bold;ZPLOD647-20;-
Odonata;Libellulidae;Orthetrum;Orthetrum coerulescens;Italy;Bold;ZPLOD645-20;-
Odonata;Libellulidae;Orthetrum;Orthetrum coerulescens;Italy;Bold;ZPLOD643-20;-
Odonata;Libellulidae;Orthetrum;Orthetrum coerulescens;Italy;Bold;ZPLOD656-20;-
Odonata;Libellulidae;Orthetrum;Orthetrum nitidinerve;Italy;Bold;ZPLOD662-20;-
Odonata;Libellulidae;Orthetrum;Orthetrum trinacria;Italy;Bold;ZPLOD670-20;-
Odonata;Libellulidae;Orthetrum;Orthetrum trinacria;Italy;Bold;ZPLOD669-20;-
Odonata;Libellulidae;Orthetrum;Orthetrum trinacria;Italy;Bold;ZPLOD666-20;-
Odonata;Synthemistidae;Oxygastra;Oxygastra curtisii;Italy;Bold;ZPLOD673-20;-
Odonata;Gomphidae;Paragomphus;Paragomphus genei;Italy;Bold;ZPLOD677-20;-
Odonata;Platycnemididae;Platycnemis;Platycnemis pennipes;Italy;Bold;ZPLOD686-20;-
Odonata;Platycnemididae;Platycnemis;Platycnemis pennipes;Germany;Bold;FBAQU508-10;-
Odonata;Platycnemididae;Platycnemis;Platycnemis pennipes;Germany;Bold;FBAQU507-10;-
Odonata;Platycnemididae;Platycnemis;Platycnemis pennipes;Germany;Bold;FBAQU1609-13;-
Odonata;Platycnemididae;Platycnemis;Platycnemis pennipes;Italy;Bold;ZPLOD692-20;-
Odonata;Platycnemididae;Platycnemis;Platycnemis pennipes;Germany;Bold;FBAQU546-10;-
Odonata;Platycnemididae;Platycnemis;Platycnemis pennipes;Germany;Bold;FBAQU545-10;-
Odonata;Platycnemididae;Platycnemis;Platycnemis pennipes;Germany;Bold;FBAQU1527-13;-
Odonata;Platycnemididae;Platycnemis;Platycnemis pennipes;Italy;Bold;ZPLOD682-20;-
Odonata;Platycnemididae;Platycnemis;Platycnemis pennipes;Germany;Bold;FBAQU121-09;-
Odonata;Platycnemididae;Platycnemis;Platycnemis pennipes;Italy;Bold;ZPLOD678-20;-
Odonata;Platycnemididae;Platycnemis;Platycnemis pennipes;Italy;Bold;ZPLOD689-20;-
Odonata;Platycnemididae;Platycnemis;Platycnemis pennipes;Italy;Bold;ZPLOD684-20;-
Odonata;Platycnemididae;Platycnemis;Platycnemis pennipes;Italy;Bold;ZPLOD688-20;-
Odonata;Coenagrionidae;Pyrrhosoma;Pyrrhosoma nymphula;Italy;Bold;ZPLOD697-20;-
Odonata;Coenagrionidae;Pyrrhosoma;Pyrrhosoma nymphula;Germany;Bold;FBAQU1583-13;-
Odonata;Coenagrionidae;Pyrrhosoma;Pyrrhosoma nymphula;United Kingdom;Bold;DTNHM3459-21;-
Odonata;Coenagrionidae;Pyrrhosoma;Pyrrhosoma nymphula;Italy;Bold;ZPLOD694-20;-
Odonata;Coenagrionidae;Pyrrhosoma;Pyrrhosoma nymphula;Italy;Bold;ZPLOD693-20;-
Odonata;Coenagrionidae;Pyrrhosoma;Pyrrhosoma nymphula;Germany;Bold;FBAQU1528-13;-

Odonata;Coenagrionidae;Pyrrhosoma;Pyrrhosoma nymphula;Germany;Bold;FBAQU1554-13;-
Odonata;Libellulidae;Selysiothemis;Selysiothemis nigra;Italy;Bold;ZPLOD702-20;-
Odonata;Libellulidae;Selysiothemis;Selysiothemis nigra;Italy;Bold;ZPLOD704-20;-
Odonata;Libellulidae;Selysiothemis;Selysiothemis nigra;Italy;Bold;ZPLOD699-20;-
Odonata;Corduliidae;Somatochlora;Somatochlora alpestris;Italy;Bold;ZPLOD705-20;-
Odonata;Corduliidae;Somatochlora;Somatochlora alpestris;Italy;Bold;ZPLOD708-20;-
Odonata;Corduliidae;Somatochlora;Somatochlora arctica;Italy;Bold;ZPLOD711-20;-
Odonata;Corduliidae;Somatochlora;Somatochlora flavomaculata;Italy;Bold;ZPLOD716-20;-
Odonata;Corduliidae;Somatochlora;Somatochlora flavomaculata;Germany;Bold;FBAQU1434-13;-
Odonata;Corduliidae;Somatochlora;Somatochlora flavomaculata;Germany;Bold;FBAQU1545-13;-
Odonata;Corduliidae;Somatochlora;Somatochlora flavomaculata;Romania;Bold;RODI023-20;-
Odonata;Corduliidae;Somatochlora;Somatochlora flavomaculata;Italy;Bold;ZPLOD714-20;-
Odonata;Corduliidae;Somatochlora;Somatochlora flavomaculata;Italy;Bold;ZPLOD719-20;-
Odonata;Corduliidae;Somatochlora;Somatochlora flavomaculata;Germany;Bold;FBAQU512-10;-
Odonata;Corduliidae;Somatochlora;Somatochlora metallica;Germany;Bold;FBAQU1435-13;-
Odonata;Corduliidae;Somatochlora;Somatochlora metallica;Germany;Bold;FBAQU549-10;-
Odonata;Corduliidae;Somatochlora;Somatochlora metallica;Germany;Bold;FBAQU514-10;-
Odonata;Corduliidae;Somatochlora;Somatochlora metallica;Italy;Bold;ZPLOD732-20;-
Odonata;Corduliidae;Somatochlora;Somatochlora metallica;Germany;Bold;FBAQU1546-13;-
Odonata;Corduliidae;Somatochlora;Somatochlora metallica;Germany;Bold;FBAQU513-10;-
Odonata;Corduliidae;Somatochlora;Somatochlora metallica;Germany;Bold;FBAQU1604-13;-
Odonata;Corduliidae;Somatochlora;Somatochlora metallica;Germany;Bold;FBAQU1594-13;-
Odonata;Corduliidae;Somatochlora;Somatochlora metallica;Germany;Bold;FBAQU1587-13;-
Odonata;Lestidae;Sympecma;Sympecma fusca;Czech Republic;Bold;FBAQU1565-13;-
Odonata;Lestidae;Sympecma;Sympecma fusca;Germany;Bold;FBAQU1566-13;-
Odonata;Lestidae;Sympecma;Sympecma fusca;Austria;Bold;FBAQU1561-13;-
Odonata;Lestidae;Sympecma;Sympecma fusca;Germany;Bold;FBAQU1598-13;-
Odonata;Lestidae;Sympecma;Sympecma fusca;Italy;Bold;ZPLOD743-20;-
Odonata;Lestidae;Sympecma;Sympecma fusca;Italy;Bold;ZPLOD736-20;-
Odonata;Lestidae;Sympecma;Sympecma fusca;Italy;Bold;ZPLOD738-20;-
Odonata;Lestidae;Sympecma;Sympecma paedisca;Germany;Bold;FBAQU1567-13;-
Odonata;Lestidae;Sympecma;Sympecma paedisca;Germany;Bold;FBAQU1523-13;-
Odonata;Lestidae;Sympecma;Sympecma paedisca;Czech Republic;Bold;FBAQU1570-13;-
Odonata;Lestidae;Sympecma;Sympecma paedisca;Czech Republic;Bold;FBAQU1571-13;-
Odonata;Lestidae;Sympecma;Sympecma paedisca;Lithuania;Bold;FBAQU1574-13;-
Odonata;Lestidae;Sympecma;Sympecma paedisca;Czech Republic;Bold;FBAQU1568-13;-
Odonata;Lestidae;Sympecma;Sympecma paedisca;Lithuania;Bold;FBAQU1572-13;-
Odonata;Lestidae;Sympecma;Sympecma paedisca;Lithuania;Bold;FBAQU1573-13;-
Odonata;Libellulidae;Sympetrum;Sympetrum danae;Italy;Bold;ZPLOD747-20;-
Odonata;Libellulidae;Sympetrum;Sympetrum danae;Germany;Bold;FBAQU550-10;-
Odonata;Libellulidae;Sympetrum;Sympetrum danae;Germany;Bold;FBAQU1550-13;-
Odonata;Libellulidae;Sympetrum;Sympetrum depressiusculum;Italy;Bold;ZPLOD750-20;-
Odonata;Libellulidae;Sympetrum;Sympetrum depressiusculum;Italy;Bold;ZPLOD751-20;-
Odonata;Libellulidae;Sympetrum;Sympetrum depressiusculum;Germany;Bold;FBAQU551-10;-
Odonata;Libellulidae;Sympetrum;Sympetrum flaveolum;Italy;Bold;ZPLOD756-20;-
Odonata;Libellulidae;Sympetrum;Sympetrum flaveolum;Italy;Bold;ZPLOD754-20;-
Odonata;Libellulidae;Sympetrum;Sympetrum flaveolum;Italy;Bold;ZPLOD759-20;-
Odonata;Libellulidae;Sympetrum;Sympetrum flaveolum;Italy;Bold;ZPLOD755-20;-
Odonata;Libellulidae;Sympetrum;Sympetrum fonscolombii;Italy;Bold;ZPLOD765-20;-
Odonata;Libellulidae;Sympetrum;Sympetrum fonscolombii;Italy;Bold;ZPLOD769-20;-
Odonata;Libellulidae;Sympetrum;Sympetrum fonscolombii;Italy;Bold;ZPLOD767-20;-
Odonata;Libellulidae;Sympetrum;Sympetrum fonscolombii;Italy;Bold;ZPLOD771-20;-
Odonata;Libellulidae;Sympetrum;Sympetrum fonscolombii;Italy;Bold;ZPLOD761-20;-
Odonata;Libellulidae;Sympetrum;Sympetrum fonscolombii;Italy;Bold;ZPLOD770-20;-
Odonata;Libellulidae;Sympetrum;Sympetrum fonscolombii;Italy;Bold;ZPLOD766-20;-
Odonata;Libellulidae;Sympetrum;Sympetrum fonscolombii;Italy;Bold;ZPLOD764-20;-
Odonata;Libellulidae;Sympetrum;Sympetrum meridionale;Austria;Bold;FBAQU1560-13;-
Odonata;Libellulidae;Sympetrum;Sympetrum meridionale;Italy;Bold;ZPLOD774-20;-
Odonata;Libellulidae;Sympetrum;Sympetrum meridionale;Italy;Bold;ZPLOD773-20;-
Odonata;Libellulidae;Sympetrum;Sympetrum meridionale;Italy;Bold;ZPLOD775-20;-
Odonata;Libellulidae;Sympetrum;Sympetrum pedemontanum;Italy;Bold;ZPLOD782-20;-
Odonata;Libellulidae;Sympetrum;Sympetrum pedemontanum;Italy;Bold;ZPLOD778-20;-
Odonata;Libellulidae;Sympetrum;Sympetrum pedemontanum;Italy;Bold;ZPLOD780-20;-
Odonata;Libellulidae;Sympetrum;Sympetrum sanguineum;Austria;Bold;FBAQU1559-13;-
Odonata;Libellulidae;Sympetrum;Sympetrum sanguineum;Austria;Bold;FBAQU1557-13;-
Odonata;Libellulidae;Sympetrum;Sympetrum sanguineum;Italy;Bold;ZPLOD788-20;-
Odonata;Libellulidae;Sympetrum;Sympetrum sanguineum;Italy;Bold;ZPLOD789-20;-
Odonata;Libellulidae;Sympetrum;Sympetrum sanguineum;Italy;Bold;ZPLOD784-20;-
Odonata;Libellulidae;Sympetrum;Sympetrum sanguineum;Italy;Bold;ZPLOD787-20;-
Odonata;Libellulidae;Sympetrum;Sympetrum sanguineum;Italy;Bold;ZPLOD792-20;-
Odonata;Libellulidae;Sympetrum;Sympetrum sanguineum;Italy;Bold;ZPLOD785-20;-
Odonata;Libellulidae;Sympetrum;Sympetrum sanguineum;Italy;Bold;ZPLOD791-20;-
Odonata;Libellulidae;Sympetrum;Sympetrum striolatum;Italy;Bold;ZPLOD801-20;-
Odonata;Libellulidae;Sympetrum;Sympetrum striolatum;Italy;Bold;ZPLOD798-20;-
Odonata;Libellulidae;Sympetrum;Sympetrum striolatum;Italy;Bold;ZPLOD796-20;-
Odonata;Libellulidae;Sympetrum;Sympetrum vulgatum;Austria;Bold;FBAQU1558-13;-
Odonata;Libellulidae;Sympetrum;Sympetrum vulgatum;Germany;Bold;FBAQU1551-13;-
Odonata;Libellulidae;Trithemis;Trithemis annulata;Italy;Bold;ZPLOD810-20;-

Odonata;Libellulidae;Trithemis;Trithemis annulata;Italy;Bold;ZPLOD807-20;-
Odonata;Libellulidae;Trithemis;Trithemis annulata;Italy;Bold;ZPLOD808-20;-
Odonata;Libellulidae;Trithemis;Trithemis annulata;Italy;Bold;ZPLOD806-20;-
Coleoptera;Melandryidae;Abdera;Abdera bifasciata;France;Bold;PSFOR262-13;-
Coleoptera;Melandryidae;Abdera;Abdera biflexuosa;United Kingdom;Bold;UKAN462-22;-
Coleoptera;Melandryidae;Abdera;Abdera flexuosa;France;Bold;PSFOR263-13;-
Coleoptera;Melandryidae;Abdera;Abdera quadrifasciata;France;Bold;PSFOR1016-14;-
Coleoptera;Melandryidae;Abdera;Abdera quadrifasciata;France;Bold;PSFOR362-13;-
Coleoptera;Buprestidae;Acmaeodera;Acmaeodera cylindrica;France;Bold;PSFOR1107-17;-
Coleoptera;Buprestidae;Acmaeodera;Acmaeodera degener;France;Bold;PSFOR572-13;-
Coleoptera;Buprestidae;Acmaeoderella;Acmaeoderella adspersula;France;Bold;PSFOR574-13;-
Coleoptera;Buprestidae;Acmaeoderella;Acmaeoderella flavofasciata;France;Bold;PSFOR575-13;-
Coleoptera;Cerambycidae;Acmaeops;Acmaeops septentrionis;Poland;Bold;PSFOR100-13;-
Coleoptera;Cerambycidae;Aegomorphus;Aegomorphus clavipes;Finland;Bold;COLFE165-12;-
Coleoptera;Cerambycidae;Aegomorphus;Aegomorphus clavipes;Russia;Bold;VVGPL2857-15;-
Coleoptera;Cerambycidae;Aegomorphus;Aegomorphus clavipes;Finland;Bold;COLFF143-13;-
Coleoptera;Cerambycidae;Aegomorphus;Aegomorphus clavipes;Finland;Bold;COLFD840-12;-
Coleoptera;Leiodidae;Agathidium;Agathidium seminulum;Finland;Bold;COLFE1031-13;-
Coleoptera;Leiodidae;Agathidium;Agathidium seminulum;Finland;Bold;COLFE1030-13;-
Coleoptera;Buprestidae;Agrilus;Agrilus laticornis;Austria;Bold;TDAAT984-20;-
Coleoptera;Buprestidae;Agrilus;Agrilus sulcicollis;Finland;Bold;COLFA221-10;-
Coleoptera;Buprestidae;Agrilus;Agrilus viridis;Finland;Bold;COLFA196-10;-
Coleoptera;Buprestidae;Agrilus;Agrilus viridis;Finland;Bold;COLFA229-10;-
Coleoptera;Buprestidae;Agrilus;Agrilus viridis;Finland;Bold;COLFA193-10;-
Coleoptera;Buprestidae;Agrilus;Agrilus viridis;Finland;Bold;COLFA239-10;-
Coleoptera;Buprestidae;Agrilus;Agrilus viridis;Finland;Bold;COLFA231-10;-
Coleoptera;Buprestidae;Agrilus;Agrilus viridis;Finland;Bold;COLFA234-10;-
Coleoptera;Buprestidae;Agrilus;Agrilus viridis;Finland;Bold;COLFA232-10;-
Coleoptera;Tenebrionidae;Allecula;Allecula rhenana;France;Bold;PSFOR1511-17;-
Coleoptera;Tenebrionidae;Allecula;Allecula suberina;France;Bold;PSFOR1512-17;-
Coleoptera;Cleridae;Allonyx;Allonyx quadrimaculatus;France;Bold;PSFOR622-13;-
Coleoptera;Elateridae;Ampedus;Ampedus auripes;France;Bold;PSFOR958-14;-
Coleoptera;Elateridae;Ampedus;Ampedus brunnicornis;France;Bold;PSFOR836-13;-
Coleoptera;Elateridae;Ampedus;Ampedus cardinalis;France;Bold;PSFOR943-14;-
Coleoptera;Elateridae;Ampedus;Ampedus cardinalis;France;Bold;PSFOR724-13;-
Coleoptera;Elateridae;Ampedus;Ampedus elegantulus;France;Bold;PSFOR945-14;-
Coleoptera;Elateridae;Ampedus;Ampedus erythrogonus;France;Bold;PSFOR917-14;-
Coleoptera;Elateridae;Ampedus;Ampedus erythrogonus;France;Bold;PSFOR825-13;-
Coleoptera;Elateridae;Ampedus;Ampedus nemoralis;France;Bold;PSFOR931-14;-
Coleoptera;Elateridae;Ampedus;Ampedus nemoralis;France;Bold;PSFOR830-13;-
Coleoptera;Elateridae;Ampedus;Ampedus nigerrimus;France;Bold;PSFOR944-14;-
Coleoptera;Elateridae;Ampedus;Ampedus nigerrimus;France;Bold;PSFOR336-13;-
Coleoptera;Elateridae;Ampedus;Ampedus nigrinus;France;Bold;PSFOR1283-17;-
Coleoptera;Elateridae;Ampedus;Ampedus nigrinus;France;Bold;PSFOR961-14;-
Coleoptera;Elateridae;Ampedus;Ampedus nigroflavus;France;Bold;PSFOR729-13;-
Coleoptera;Elateridae;Ampedus;Ampedus pomorum;France;Bold;PSFOR545-13;-
Coleoptera;Elateridae;Ampedus;Ampedus praeustus;France;Bold;PSFOR846-13;-
Coleoptera;Elateridae;Ampedus;Ampedus quercicola;France;Bold;PSFOR337-13;-
Coleoptera;Elateridae;Ampedus;Ampedus quercicola;Switzerland;Bold;ELAAA076-16;-
Coleoptera;Elateridae;Ampedus;Ampedus quercicola;Switzerland;Bold;ELAAA073-16;-
Coleoptera;Elateridae;Ampedus;Ampedus rufipennis;France;Bold;PSFOR934-14;-
Coleoptera;Elateridae;Ampedus;Ampedus rufipennis;France;Bold;PSFOR935-14;-
Coleoptera;Elateridae;Ampedus;Ampedus sanguineus;France;Bold;PSFOR826-13;-
Coleoptera;Elateridae;Ampedus;Ampedus sanguineus;Norway;Bold;NOCOL190-12;-
Coleoptera;Elateridae;Ampedus;Ampedus sanguineus;France;Bold;PSFOR831-13;-
Coleoptera;Elateridae;Ampedus;Ampedus sanguinolentus;Switzerland;Bold;ELAAA092-16;-
Coleoptera;Elateridae;Ampedus;Ampedus sanguinolentus;Switzerland;Bold;ELAAA091-16;-
Coleoptera;Elateridae;Ampedus;Ampedus sanguinolentus;Switzerland;Bold;ELAAA095-16;-
Coleoptera;Elateridae;Ampedus;Ampedus scrofa;France;Bold;PSFOR817-13;-
Coleoptera;Elateridae;Ampedus;Ampedus scrofa;France;Bold;PSFOR874-14;-
Coleoptera;Elateridae;Ampedus;Ampedus scrofa;France;Bold;PSFOR499-13;-
Coleoptera;Elateridae;Ampedus;Ampedus scrofa;France;Bold;PSFOR498-13;-
Coleoptera;Elateridae;Ampedus;Ampedus scrofa;France;Bold;PSFOR506-13;-
Coleoptera;Elateridae;Ampedus;Ampedus scrofa;France;Bold;PSFOR515-13;-
Coleoptera;Elateridae;Ampedus;Ampedus scrofa;France;Bold;PSFOR514-13;-
Coleoptera;Elateridae;Ampedus;Ampedus scrofa;France;Bold;PSFOR509-13;-
Coleoptera;Elateridae;Ampedus;Ampedus scrofa;France;Bold;PSFOR500-13;-
Coleoptera;Elateridae;Ampedus;Ampedus scrofa;France;Bold;PSFOR501-13;-
Coleoptera;Elateridae;Ampedus;Ampedus scrofa;France;Bold;PSFOR513-13;-
Coleoptera;Elateridae;Ampedus;Ampedus scrofa;France;Bold;PSFOR505-13;-
Coleoptera;Elateridae;Ampedus;Ampedus scrofa;France;Bold;PSFOR496-13;-
Coleoptera;Elateridae;Ampedus;Ampedus scrofa;France;Bold;PSFOR816-13;-
Coleoptera;Elateridae;Ampedus;Ampedus scrofa;France;Bold;PSFOR497-13;-
Coleoptera;Elateridae;Ampedus;Ampedus scrofa;France;Bold;PSFOR525-13;-
Coleoptera;Bostrichidae;Amphicerus;Amphicerus bimaculatus;France;Bold;PSFOR1090-17;-
Coleoptera;Bostrichidae;Amphicerus;Amphicerus bimaculatus;France;Bold;PSFOR1092-17;-
Coleoptera;Bostrichidae;Amphicerus;Amphicerus bimaculatus;France;Bold;PSFOR1091-17;-
Coleoptera;Nitidulidae;Amphotis;Amphotis marginata;France;Bold;PSFOR643-13;-

Coleoptera;Cerambycidae;Anaglyptus;Anaglyptus gibbosus;France;Bold;PSFOR033-13;-
Coleoptera;Cerambycidae;Anaglyptus;Anaglyptus mysticus;France;Bold;PSFOR866-14;-
Coleoptera;Scraptiidae;Anaspis;Anaspis frontalis;Austria;Bold;TDAOE581-21;-
Coleoptera;Scraptiidae;Anaspis;Anaspis frontalis;Austria;Bold;TDAOE319-21;-
Coleoptera;Aderidae;Anidorus;Anidorus sanguinolentus;France;Bold;PSFOR1077-17;-
Coleoptera;Aderidae;Anidorus;Anidorus sanguinolentus;France;Bold;PSFOR1076-17;-
Coleoptera;Curculionidae;Anisandrus;Anisandrus dispar;Norway;Bold;NOCLP1792-19;-
Coleoptera;Curculionidae;Anisandrus;Anisandrus dispar;Russia;Bold;SCOLY173-11;-
Coleoptera;Leiodidae;Anisotoma;Anisotoma castanea;Austria;Bold;TDAAT1774-20;-
Coleoptera;Leiodidae;Anisotoma;Anisotoma humeralis;France;Bold;PSFOR298-13;-
Coleoptera;Ptinidae;Anobium;Anobium hederae;France;Bold;PSFOR1049-14;-
Coleoptera;Ptinidae;Anobium;Anobium punctatum;Austria;Bold;TDAAT1245-20;-
Coleoptera;Ptinidae;Anobium;Anobium punctatum;France;Bold;PSFOR696-13;-
Coleoptera;Ptinidae;Anobium;Anobium punctatum;Austria;Bold;TDAAT1574-20;-
Coleoptera;Oedemeridae;Anogcodes;Anogcodes seladonius;France;Bold;PSFOR1417-17;-
Coleoptera;Cerambycidae;Anoplodera;Anoplodera sexguttata;France;Bold;PSFOR037-13;-
Coleoptera;Buprestidae;Anthaxia;Anthaxia hungarica;France;Bold;PSFOR577-13;-
Coleoptera;Buprestidae;Anthaxia;Anthaxia salicis;France;Bold;PSFOR578-13;-
Coleoptera;Buprestidae;Anthaxia;Anthaxia sepulchralis;France;Bold;PSFOR579-13;-
Coleoptera;Anthribidae;Anthribus;Anthribus nebulosus;France;Bold;PSFOR178-13;-
Coleoptera;Anthribidae;Anthribus;Anthribus nebulosus;Norway;Bold;NOCLP3347-22;-
Coleoptera;Anthribidae;Anthribus;Anthribus nebulosus;Norway;Bold;NOCLP3313-22;-
Coleoptera;Anthribidae;Anthribus;Anthribus nebulosus;Norway;Bold;NOCLP3314-22;-
Coleoptera;Anthribidae;Anthribus;Anthribus scapularis;Finland;Bold;COLFC122-12;-
Coleoptera;Curculionidae;Aphanommata;Aphanommata filum;France;Bold;PSFOR1164-17;-
Coleoptera;Cerambycidae;Aromia;Aromia moschata;France;Bold;PSFOR038-13;-
Coleoptera;Cerambycidae;Aromia;Aromia moschata;France;Bold;PSFOR039-13;-
Coleoptera;Sphindidae;Aspidiphorus;Aspidiphorus orbiculatus;United Kingdom;Bold;UKAN393-22;-
Coleoptera;Sphindidae;Aspidiphorus;Aspidiphorus orbiculatus;France;Bold;PSFOR666-13;-
Coleoptera;Zopheridae;Aulonium;Aulonium ruficorne;France;Bold;PSFOR788-13;-
Coleoptera;Staphylinidae;Bibloporus;Bibloporus minutus;Finland;Bold;COLFD129-12;-
Coleoptera;Biphyllidae;Biphyllus;Biphyllus frater;France;Bold;PSFOR608-13;-
Coleoptera;Zopheridae;Bitoma;Bitoma crenata;France;Bold;PSFOR003-13;-
Coleoptera;Staphylinidae;Bolitochara;Bolitochara obliqua;United Kingdom;Bold;UKAN527-22;-
Coleoptera;Tenebrionidae;Bolitophagus;Bolitophagus interruptus;France;Bold;PSFOR1549-17;-
Coleoptera;Tenebrionidae;Bolitophagus;Bolitophagus reticulatus;France;Bold;PSFOR482-13;-
Coleoptera;Tenebrionidae;Bolitophagus;Bolitophagus reticulatus;Finland;Bold;COLFC555-12;-
Coleoptera;Bostrichidae;Bostrichus;Bostrichus capucinus;France;Bold;PSFOR609-13;-
Coleoptera;Bostrichidae;Bostrichus;Bostrichus capucinus;Finland;Bold;COLFA168-10;-
Coleoptera;Bothrideridae;Bothrideres;Bothrideres bipunctatus;France;Bold;PSFOR618-13;-
Coleoptera;Bothrideridae;Bothrideres;Bothrideres bipunctatus;France;Bold;PSFOR1098-17;-
Coleoptera;Elateridae;Brachygonus;Brachygonus megerlei;Switzerland;Bold;ELAAA105-16;-
Coleoptera;Elateridae;Brachygonus;Brachygonus megerlei;Switzerland;Bold;ELAAA001-16;-
Coleoptera;Elateridae;Brachygonus;Brachygonus ruficeps;France;Bold;PSFOR742-13;-
Coleoptera;Buprestidae;Buprestis;Buprestis haemorrhoidalis;Italy;Bold;PSFOR581-13;-
Coleoptera;Ptinidae;Cacotemnus;Cacotemnus rufipes;France;Bold;PSFOR672-13;-
Coleoptera;Cryptophagidae;Caenoscelis;Caenoscelis ferruginea;Finland;Bold;COLFD084-12;-
Coleoptera;Elateridae;Calambus;Calambus bipustulatus;France;Bold;PSFOR743-13;-
Coleoptera;Elateridae;Calambus;Calambus bipustulatus;France;Bold;PSFOR334-13;-
Coleoptera;Cerambycidae;Callidium;Callidium coriaceum;Finland;Bold;COLFA501-12;-
Coleoptera;Cerambycidae;Callidium;Callidium violaceum;France;Bold;PSFOR043-13;-
Coleoptera;Cerambycidae;Callimus;Callimus angulatus;France;Bold;PSFOR110-13;-
Coleoptera;Oedemeridae;Calopus;Calopus serraticornis;France;Bold;PSFOR650-13;-
Coleoptera;Curculionidae;Camptorhinus;Camptorhinus simplex;France;Bold;PSFOR1173-17;-
Coleoptera;Elateridae;Cardiophorus;Cardiophorus anticus;France;Bold;PSFOR946-14;-
Coleoptera;Elateridae;Cardiophorus;Cardiophorus gramineus;France;Bold;PSFOR745-13;-
Coleoptera;Curculionidae;Carphoborus;Carphoborus perrisi;France;Bold;PSFOR1175-17;-
Coleoptera;Curculionidae;Carphoborus;Carphoborus perrisi;France;Bold;PSFOR1176-17;-
Coleoptera;Latridiidae;Cartodere;Cartodere nodifer;United Kingdom;Bold;UKAN361-22;-
Coleoptera;Cerambycidae;Cerambyx;Cerambyx cerdo;Spain;Bold;OAKCE002-18;-
Coleoptera;Cerambycidae;Cerambyx;Cerambyx cerdo;Spain;Bold;OAKCE001-18;-
Coleoptera;Cerophytidae;Cerophytum;Cerophytum elateroides;France;Bold;PSFOR237-13;-
Coleoptera;Cerylonidae;Cerylon;Cerylon deplanatum;France;Bold;PSFOR804-13;-
Coleoptera;Cerylonidae;Cerylon;Cerylon fagi;Austria;Bold;TDAOE341-21;-
Coleoptera;Cerylonidae;Cerylon;Cerylon fagi;France;Bold;PSFOR805-13;-
Coleoptera;Cerylonidae;Cerylon;Cerylon ferrugineum;France;Bold;PSFOR806-13;-
Coleoptera;Cerylonidae;Cerylon;Cerylon ferrugineum;Finland;Bold;COLFD131-12;-
Coleoptera;Cerylonidae;Cerylon;Cerylon histeroides;France;Bold;PSFOR807-13;-
Coleoptera;Cerylonidae;Cerylon;Cerylon histeroides;Finland;Bold;COLFA059-10;-
Coleoptera;Cerylonidae;Cerylon;Cerylon histeroides;France;Bold;PSFOR1043-14;-
Coleoptera;Scarabaeidae;Cetonia;Cetonia aurata;France;Bold;PSFOR300-13;-
Coleoptera;Scarabaeidae;Cetonia;Cetonia aurata;Finland;Bold;COLFA132-10;-
Coleoptera;Cerambycidae;Chlorophorus;Chlorophorus figuratus;France;Bold;PSFOR047-13;-
Coleoptera;Oedemeridae;Chrysanthia;Chrysanthia geniculata;France;Bold;PSFOR651-13;-
Coleoptera;Ciidae;Cis;Cis bidentatus;Finland;Bold;COLFD112-12;-
Coleoptera;Ciidae;Cis;Cis boleti;United Kingdom;Bold;UKAN468-22;-
Coleoptera;Ciidae;Cis;Cis laminatus;France;Bold;PSFOR1144-17;-
Coleoptera;Ciidae;Cis;Cis rugulosus;France;Bold;PSFOR620-13;-

Coleoptera;Ciidae;Cis;Cis striatulus;France;Bold;PSFOR1145-17;-
Coleoptera;Cleridae;Clerus;Clerus mutillarius;Austria;Bold;SICOA155-18;-
Coleoptera;Cleridae;Clerus;Clerus mutillarius;France;Bold;PSFOR623-13;-
Coleoptera;Zopheridae;Colobicus;Colobicus hirtus;France;Bold;PSFOR790-13;-
Coleoptera;Zopheridae;Colobicus;Colobicus hirtus;France;Bold;PSFOR328-13;-
Coleoptera;Zopheridae;Colobicus;Colobicus hirtus;France;Bold;PSFOR477-13;-
Coleoptera;Zopheridae;Colydium;Colydium elongatum;France;Bold;PSFOR327-13;-
Coleoptera;Zopheridae;Colydium;Colydium elongatum;France;Bold;PSFOR485-13;-
Coleoptera;Melandryidae;Conopalpus;Conopalpus brevicollis;France;Bold;PSFOR364-13;-
Coleoptera;Melandryidae;Conopalpus;Conopalpus testaceus;France;Bold;PSFOR265-13;-
Coleoptera;Buprestidae;Coraebus;Coraebus undatus;France;Bold;PSFOR585-13;-
Coleoptera;Tenebrionidae;Corticeus;Corticeus bicoloroides;France;Bold;PSFOR1523-17;-
Coleoptera;Tenebrionidae;Corticeus;Corticeus fasciatus;France;Bold;PSFOR193-13;-
Coleoptera;Tenebrionidae;Corticeus;Corticeus suberis;France;Bold;PSFOR1524-17;-
Coleoptera;Tenebrionidae;Corticeus;Corticeus unicolor;France;Bold;PSFOR476-13;-
Coleoptera;Tenebrionidae;Corticeus;Corticeus unicolor;Austria;Bold;TDAOE364-21;-
Coleoptera;Curculionidae;Cossonus;Cossonus parallelepipedus;Norway;Bold;NOCLP1403-19;-
Coleoptera;Curculionidae;Cossonus;Cossonus parallelepipedus;Norway;Bold;NOCLP1402-19;-
Coleoptera;Zopheridae;Coxelus;Coxelus pictus;Italy;Bold;PSFOR792-13;-
Coleoptera;Curculionidae;Cryphalus;Cryphalus intermedius;France;Bold;PSFOR1181-17;-
Coleoptera;Curculionidae;Cryphalus;Cryphalus intermedius;France;Bold;PSFOR1180-17;-
Coleoptera;Nitidulidae;Cryptarcha;Cryptarcha undata;France;Bold;PSFOR1027-14;-
Coleoptera;Laemophloeidae;Cryptolestes;Cryptolestes ferrugineus;Germany;Bold;CSP050-09;-
Coleoptera;Cryptophagidae;Cryptophagus;Cryptophagus dentatus;Norway;Bold;NOCLP3198-22;-
Coleoptera;Cryptophagidae;Cryptophagus;Cryptophagus dorsalis;Norway;Bold;NOCLP3346-22;-
Coleoptera;Cryptophagidae;Cryptophagus;Cryptophagus intermedius;Norway;Bold;NOCLP3199-22;-
Coleoptera;Cryptophagidae;Cryptophagus;Cryptophagus lapponicus;Finland;Bold;COLFC520-12;-
Coleoptera;Cryptophagidae;Cryptophagus;Cryptophagus pilosus;Norway;Bold;NOCLP3200-22;-
Coleoptera;Cryptophagidae;Cryptophagus;Cryptophagus scanicus;Norway;Bold;NOCLP3197-22;-
Coleoptera;Cryptophagidae;Cryptophagus;Cryptophagus scutellatus;Norway;Bold;NOCLP3196-22;-
Coleoptera;Erotylidae;Cryptophilus;Cryptophilus integer;United Kingdom;Bold;UKAN395-22;-
Coleoptera;Curculionidae;Cryptorhynchus;Cryptorhynchus lapathi;Norway;Bold;NOCLP1426-19;-
Coleoptera;Curculionidae;Crypturgus;Crypturgus hispidulus;Norway;Bold;NOCLP1765-19;-
Coleoptera;Curculionidae;Crypturgus;Crypturgus hispidulus;Norway;Bold;NOCLP1766-19;-
Coleoptera;Curculionidae;Crypturgus;Crypturgus mediterraneus;France;Bold;PSFOR1186-17;-
Coleoptera;Curculionidae;Crypturgus;Crypturgus numidicus;France;Bold;PSFOR1185-17;-
Coleoptera;Curculionidae;Crypturgus;Crypturgus numidicus;France;Bold;PSFOR1183-17;-
Coleoptera;Curculionidae;Crypturgus;Crypturgus numidicus;France;Bold;PSFOR1184-17;-
Coleoptera;Dermestidae;Ctesias;Ctesias serra;United Kingdom;Bold;UKAN379-22;-
Coleoptera;Nitidulidae;Cychramus;Cychramus luteus;Finland;Bold;COLFA525-12;-
Coleoptera;Nitidulidae;Cychramus;Cychramus luteus;Norway;Bold;NOCLP3141-22;-
Coleoptera;Nitidulidae;Cychramus;Cychramus luteus;France;Bold;PSFOR288-13;-
Coleoptera;Nitidulidae;Cychramus;Cychramus variegatus;Norway;Bold;NOCLP3139-22;-
Coleoptera;Nitidulidae;Cychramus;Cychramus variegatus;Norway;Bold;NOCLP3140-22;-
Coleoptera;Nitidulidae;Cyllodes;Cyllodes ater;France;Bold;PSFOR645-13;-
Coleoptera;Erotylidae;Dacne;Dacne bipustulata;France;Bold;PSFOR325-13;-
Coleoptera;Erotylidae;Dacne;Dacne bipustulata;France;Bold;PSFOR324-13;-
Coleoptera;Erotylidae;Dacne;Dacne bipustulata;France;Bold;PSFOR487-13;-
Coleoptera;Elateridae;Danosoma;Danosoma fasciatum;Switzerland;Bold;ELAAA108-16;-
Coleoptera;Melyridae;Dasytes;Dasytes caeruleus;France;Bold;PSFOR1373-17;-
Coleoptera;Melyridae;Dasytes;Dasytes croceipes;France;Bold;PSFOR1375-17;-
Coleoptera;Melyridae;Dasytes;Dasytes pauperculus;France;Bold;PSFOR1380-17;-
Coleoptera;Curculionidae;Dendroctonus;Dendroctonus micans;France;Bold;PSFOR704-13;-
Coleoptera;Cleridae;Denops;Denops albofasciatus;France;Bold;PSFOR624-13;-
Coleoptera;Elateridae;Denticollis;Denticollis linearis;Norway;Bold;NOCOL017-12;-
Coleoptera;Elateridae;Denticollis;Denticollis linearis;France;Bold;PSFOR333-13;-
Coleoptera;Tenebrionidae;Diaperis;Diaperis boleti;Austria;Bold;TDAAT1941-20;-
Coleoptera;Tenebrionidae;Diaperis;Diaperis boleti;Finland;Bold;COLFA150-10;-
Coleoptera;Lycidae;Dictyoptera;Dictyoptera aurora;France;Bold;PSFOR639-13;-
Coleoptera;Latridiidae;Dienerella;Dienerella vincenti;Finland;Bold;COLFD230-12;-
Coleoptera;Biphyllidae;Diplocoelus;Diplocoelus fagi;France;Bold;PSFOR915-14;-
Coleoptera;Biphyllidae;Diplocoelus;Diplocoelus fagi;France;Bold;PSFOR299-13;-
Coleoptera;Biphyllidae;Diplocoelus;Diplocoelus fagi;France;Bold;PSFOR007-13;-
Coleoptera;Melandryidae;Dircaea;Dircaea australis;France;Bold;PSFOR260-13;-
Coleoptera;Anthribidae;Dissoleucas;Dissoleucas niveirostris;Norway;Bold;NOCLP3318-22;-
Coleoptera;Anthribidae;Dissoleucas;Dissoleucas niveirostris;Norway;Bold;NOCLP3317-22;-
Coleoptera;Anthribidae;Dissoleucas;Dissoleucas niveirostris;France;Bold;PSFOR308-13;-
Coleoptera;Ptinidae;Dorcatoma;Dorcatoma flavicornis;France;Bold;PSFOR1444-17;-
Coleoptera;Ptinidae;Dorcatoma;Dorcatoma flavicornis;France;Bold;PSFOR1445-17;-
Coleoptera;Eucnemidae;Dromaeolus;Dromaeolus barnabita;France;Bold;PSFOR995-14;-
Coleoptera;Eucnemidae;Dromaeolus;Dromaeolus barnabita;France;Bold;PSFOR590-13;-
Coleoptera;Staphylinidae;Dropephylla;Dropephylla koltzei;Norway;Bold;NOCLP1804-19;-
Coleoptera;Curculionidae;Dryocoetes;Dryocoetes alni;Norway;Bold;NOCLP1786-19;-
Coleoptera;Curculionidae;Dryocoetes;Dryocoetes alni;Norway;Bold;NOCLP1787-19;-
Coleoptera;Elateridae;Elater;Elater ferrugineus;France;Bold;PSFOR752-13;-
Coleoptera;Elateridae;Elater;Elater ferrugineus;France;Bold;PSFOR948-14;-
Coleoptera;Elateridae;Elater;Elater ferrugineus;Switzerland;Bold;ELAAA107-16;-
Coleoptera;Lymexylidae;Elateroides;Elateroides dermestoides;France;Bold;PSFOR887-14;-

Coleoptera;Tenebrionidae;Eledonoprius;Eledonoprius serrifrons;France;Bold;PSFOR1553-17;-
Coleoptera;Endomychidae;Endomychus;Endomychus coccineus;France;Bold;PSFOR631-13;-
Coleoptera;Anthribidae;Enedreytes;Enedreytes hilaris;France;Bold;PSFOR1080-17;-
Coleoptera;Anthribidae;Enedreytes;Enedreytes hilaris;France;Bold;PSFOR1081-17;-
Coleoptera;Anthribidae;Enedreytes;Enedreytes sepicola;France;Bold;PSFOR172-13;-
Coleoptera;Anthribidae;Enedreytes;Enedreytes sepicola;France;Bold;PSFOR895-14;-
Coleoptera;Latridiidae;Enicmus;Enicmus fungicola;Finland;Bold;COLFD123-12;-
Coleoptera;Latridiidae;Enicmus;Enicmus testaceus;United Kingdom;Bold;UKAN371-22;-
Coleoptera;Latridiidae;Enicmus;Enicmus testaceus;Austria;Bold;TDAOE609-21;-
Coleoptera;Eucnemidae;Epiphanis;Epiphanis cornutus;France;Bold;PSFOR591-13;-
Coleoptera;Ptinidae;Episernus;Episernus angulicollis;France;Bold;PSFOR1480-17;-
Coleoptera;Ptinidae;Episernus;Episernus gentilis;France;Bold;PSFOR1479-17;-
Coleoptera;Ptinidae;Episernus;Episernus striatellus;France;Bold;PSFOR1481-17;-
Coleoptera;Nitidulidae;Epuraea;Epuraea angustula;Norway;Bold;NOCLP3176-22;-
Coleoptera;Nitidulidae;Epuraea;Epuraea angustula;Norway;Bold;NOCLP3175-22;-
Coleoptera;Nitidulidae;Epuraea;Epuraea biguttata;Norway;Bold;NOCLP3143-22;-
Coleoptera;Nitidulidae;Epuraea;Epuraea binotata;Norway;Bold;NOCLP3182-22;-
Coleoptera;Nitidulidae;Epuraea;Epuraea boreella;Norway;Bold;NOCLP3183-22;-
Coleoptera;Nitidulidae;Epuraea;Epuraea boreella;Norway;Bold;NOCLP3184-22;-
Coleoptera;Nitidulidae;Epuraea;Epuraea limbata;Norway;Bold;NOCLP3154-22;-
Coleoptera;Nitidulidae;Epuraea;Epuraea longiclavis;Norway;Bold;NOCLP3185-22;-
Coleoptera;Nitidulidae;Epuraea;Epuraea marseuli;Norway;Bold;NOCLP3179-22;-
Coleoptera;Nitidulidae;Epuraea;Epuraea marseuli;Norway;Bold;NOCLP3181-22;-
Coleoptera;Nitidulidae;Epuraea;Epuraea marseuli;Norway;Bold;NOCLP3180-22;-
Coleoptera;Nitidulidae;Epuraea;Epuraea melanocephala;Norway;Bold;NOCLP3159-22;-
Coleoptera;Nitidulidae;Epuraea;Epuraea melanocephala;Norway;Bold;NOCLP3160-22;-
Coleoptera;Nitidulidae;Epuraea;Epuraea neglecta;Norway;Bold;NOCLP3161-22;-
Coleoptera;Nitidulidae;Epuraea;Epuraea neglecta;Norway;Bold;NOCLP3162-22;-
Coleoptera;Nitidulidae;Epuraea;Epuraea placida;Norway;Bold;NOCLP3157-22;-
Coleoptera;Nitidulidae;Epuraea;Epuraea placida;Norway;Bold;NOCLP3156-22;-
Coleoptera;Nitidulidae;Epuraea;Epuraea placida;Norway;Bold;NOCLP3155-22;-
Coleoptera;Nitidulidae;Epuraea;Epuraea pygmaea;Norway;Bold;NOCLP3178-22;-
Coleoptera;Nitidulidae;Epuraea;Epuraea pygmaea;Norway;Bold;NOCLP3177-22;-
Coleoptera;Nitidulidae;Epuraea;Epuraea rufomarginata;Norway;Bold;NOCLP3153-22;-
Coleoptera;Nitidulidae;Epuraea;Epuraea silacea;Norway;Bold;NOCLP3142-22;-
Coleoptera;Nitidulidae;Epuraea;Epuraea terminalis;Austria;Bold;TDAOE261-21;-
Coleoptera;Nitidulidae;Epuraea;Epuraea unicolor;Norway;Bold;NOCLP3147-22;-
Coleoptera;Nitidulidae;Epuraea;Epuraea unicolor;Norway;Bold;NOCLP3146-22;-
Coleoptera;Nitidulidae;Epuraea;Epuraea unicolor;Norway;Bold;NOCLP3145-22;-
Coleoptera;Nitidulidae;Epuraea;Epuraea unicolor;Norway;Bold;NOCLP3144-22;-
Coleoptera;Nitidulidae;Epuraea;Epuraea variegata;Norway;Bold;NOCLP3148-22;-
Coleoptera;Nitidulidae;Epuraea;Epuraea variegata;Norway;Bold;NOCLP3149-22;-
Coleoptera;Cerambycidae;Ergates;Ergates faber;France;Bold;PSFOR1139-17;-
Coleoptera;Ptinidae;Ernobius;Ernobius gigas;France;Bold;PSFOR1475-17;-
Coleoptera;Ptinidae;Ernobius;Ernobius kiesenwetteri;France;Bold;PSFOR1474-17;-
Coleoptera;Ptinidae;Ernobius;Ernobius laticollis;France;Bold;PSFOR1471-17;-
Coleoptera;Ptinidae;Ernobius;Ernobius longicornis;France;Bold;PSFOR1472-17;-
Coleoptera;Ptinidae;Ernobius;Ernobius mollis;France;Bold;PSFOR693-13;-
Coleoptera;Ptinidae;Ernobius;Ernobius parens;France;Bold;PSFOR1473-17;-
Coleoptera;Ptinidae;Ernobius;Ernobius pruinosus;France;Bold;PSFOR1470-17;-
Coleoptera;Curculionidae;Ernoporus;Ernoporus tiliae;Norway;Bold;NOCLP1777-19;-
Coleoptera;Mycetophagidae;Esarcus;Esarcus abeillei;France;Bold;PSFOR1407-17;-
Coleoptera;Eucnemidae;Eucnemis;Eucnemis capucina;France;Bold;PSFOR322-13;-
Coleoptera;Eucnemidae;Eucnemis;Eucnemis capucina;France;Bold;PSFOR867-14;-
Coleoptera;Anthribidae;Eusphyrus;Eusphyrus vasconicus;France;Bold;PSFOR1082-17;-
Coleoptera;Cerambycidae;Exocentrus;Exocentrus adspersus;France;Bold;PSFOR983-14;-
Coleoptera;Staphylinidae;Gabrius;Gabrius splendidulus;Norway;Bold;NOCLP3244-22;-
Coleoptera;Staphylinidae;Gabrius;Gabrius splendidulus;Norway;Bold;NOCLP3245-22;-
Coleoptera;Ptinidae;Gastrallus;Gastrallus corsicus;France;Bold;PSFOR1436-17;-
Coleoptera;Cerambycidae;Glaphyra;Glaphyra marmottani;France;Bold;PSFOR1111-17;-
Coleoptera;Nitidulidae;Glischrochilus;Glischrochilus quadripunctatus;Finland;Bold;COLFA415-12;-
Coleoptera;Dermestidae;Globicornis;Globicornis bifasciata;France;Bold;PSFOR1263-17;-
Coleoptera;Dermestidae;Globicornis;Globicornis fasciata;France;Bold;PSFOR1266-17;-
Coleoptera;Dermestidae;Globicornis;Globicornis fasciata;France;Bold;PSFOR1265-17;-
Coleoptera;Dermestidae;Globicornis;Globicornis variegata;France;Bold;PSFOR1267-17;-
Coleoptera;Histeridae;Gnathoncus;Gnathoncus rotundatus;United Kingdom;Bold;UKAN455-22;-
Coleoptera;Cerambycidae;Gracilia;Gracilia minuta;Austria;Bold;SICOE926-19;-
Coleoptera;Cerambycidae;Gracilia;Gracilia minuta;France;Bold;PSFOR053-13;-
Coleoptera;Cerambycidae;Grammoptera;Grammoptera abdominalis;France;Bold;PSFOR126-13;-
Coleoptera;Cerambycidae;Grammoptera;Grammoptera ruficornis;France;Bold;PSFOR127-13;-
Coleoptera;Cerambycidae;Grammoptera;Grammoptera ustulata;France;Bold;PSFOR128-13;-
Coleoptera;Ptinidae;Grynobius;Grynobius planus;France;Bold;PSFOR691-13;-
Coleoptera;Ptinidae;Hadrobregmus;Hadrobregmus pertinax;Finland;Bold;COLFD125-12;-
Coleoptera;Tetratomidae;Hallomenus;Hallomenus binotatus;United Kingdom;Bold;UKAN342-22;-
Coleoptera;Tetratomidae;Hallomenus;Hallomenus binotatus;France;Bold;PSFOR293-13;-
Coleoptera;Ptinidae;Hemicoelus;Hemicoelus canaliculatus;France;Bold;PSFOR1434-17;-
Coleoptera;Ptinidae;Hemicoelus;Hemicoelus costatus;France;Bold;PSFOR701-13;-
Coleoptera;Ptinidae;Hemicoelus;Hemicoelus fulvicornis;France;Bold;PSFOR1050-14;-

Coleoptera;Histeridae;Hololepta;Hololepta plana;France;Bold;PSFOR285-13;-
Coleoptera;Curculionidae;Hylastes;Hylastes angustatus;France;Bold;PSFOR705-13;-
Coleoptera;Curculionidae;Hylastes;Hylastes angustatus;Norway;Bold;NOCLP1736-19;-
Coleoptera;Curculionidae;Hylastes;Hylastes attenuatus;Norway;Bold;NOCLP1738-19;-
Coleoptera;Curculionidae;Hylastes;Hylastes attenuatus;Norway;Bold;NOCLP1737-19;-
Coleoptera;Curculionidae;Hylastes;Hylastes cunicularius;Norway;Bold;NOCLP1730-19;-
Coleoptera;Curculionidae;Hylastes;Hylastes cunicularius;Norway;Bold;NOCLP1731-19;-
Coleoptera;Eucnemidae;Hylis;Hylis cariniceps;Austria;Bold;TDAAT1951-20;-
Coleoptera;Eucnemidae;Hylis;Hylis cariniceps;France;Bold;PSFOR316-13;-
Coleoptera;Eucnemidae;Hylis;Hylis foveicollis;France;Bold;PSFOR997-14;-
Coleoptera;Eucnemidae;Hylis;Hylis foveicollis;France;Bold;PSFOR896-14;-
Coleoptera;Eucnemidae;Hylis;Hylis foveicollis;France;Bold;PSFOR592-13;-
Coleoptera;Eucnemidae;Hylis;Hylis olexai;United Kingdom;Bold;UKAN396-22;-
Coleoptera;Eucnemidae;Hylis;Hylis olexai;France;Bold;PSFOR902-14;-
Coleoptera;Eucnemidae;Hylis;Hylis olexai;France;Bold;PSFOR898-14;-
Coleoptera;Eucnemidae;Hylis;Hylis olexai;France;Bold;PSFOR593-13;-
Coleoptera;Eucnemidae;Hylis;Hylis olexai;France;Bold;PSFOR1330-17;-
Coleoptera;Eucnemidae;Hylis;Hylis olexai;France;Bold;PSFOR1009-14;-
Coleoptera;Eucnemidae;Hylis;Hylis simonae;France;Bold;PSFOR594-13;-
Coleoptera;Eucnemidae;Hylis;Hylis simonae;France;Bold;PSFOR1045-14;-
Coleoptera;Curculionidae;Hylobius;Hylobius pinastri;Estonia;Bold;COLFC732-12;-
Coleoptera;Curculionidae;Hylobius;Hylobius pinastri;Norway;Bold;NOCLP1413-19;-
Coleoptera;Curculionidae;Hylobius;Hylobius pinastri;Norway;Bold;NOCLP1412-19;-
Coleoptera;Cerambycidae;Hylotrupes;Hylotrupes bajulus;France;Bold;PSFOR054-13;-
Coleoptera;Cerambycidae;Hylotrupes;Hylotrupes bajulus;France;Bold;PSFOR055-13;-
Coleoptera;Curculionidae;Hylurgops;Hylurgops glabratus;Norway;Bold;NOCLP1724-19;-
Coleoptera;Curculionidae;Hylurgops;Hylurgops palliatus;France;Bold;PSFOR707-13;-
Coleoptera;Tenebrionidae;Hymenalia;Hymenalia rufipes;France;Bold;PSFOR207-13;-
Coleoptera;Elateridae;Hypoganus;Hypoganus inunctus;France;Bold;PSFOR970-14;-
Coleoptera;Cerambycidae;Icosium;Icosium tomentosum;France;Bold;PSFOR1113-17;-
Coleoptera;Nitidulidae;Ipidia;Ipidia binotata;France;Bold;PSFOR646-13;-
Coleoptera;Curculionidae;Ips;Ips sexdentatus;Norway;Bold;NOCLP1769-19;-
Coleoptera;Curculionidae;Ips;Ips typographus;Norway;Bold;NOCLP1771-19;-
Coleoptera;Elateridae;Ischnodes;Ischnodes sanguinicollis;France;Bold;PSFOR964-14;-
Coleoptera;Elateridae;Ischnodes;Ischnodes sanguinicollis;France;Bold;PSFOR966-14;-
Coleoptera;Oedemeridae;Ischnomera;Ischnomera caerulea;France;Bold;PSFOR653-13;-
Coleoptera;Oedemeridae;Ischnomera;Ischnomera cinerascens;France;Bold;PSFOR654-13;-
Coleoptera;Oedemeridae;Ischnomera;Ischnomera sanguinicollis;France;Bold;PSFOR656-13;-
Coleoptera;Eucnemidae;Isorhipis;Isorhipis melasoides;France;Bold;PSFOR314-13;-
Coleoptera;Elateridae;Lacon;Lacon punctatus;France;Bold;PSFOR942-14;-
Coleoptera;Elateridae;Lacon;Lacon querceus;France;Bold;PSFOR968-14;-
Coleoptera;Laemophloeidae;Laemophloeus;Laemophloeus kraussi;France;Bold;PSFOR910-14;-
Coleoptera;Laemophloeidae;Laemophloeus;Laemophloeus monilis;France;Bold;PSFOR1014-14;-
Coleoptera;Laemophloeidae;Laemophloeus;Laemophloeus nigricollis;France;Bold;PSFOR636-13;-
Coleoptera;Buprestidae;Lamprodila;Lamprodila festiva;France;Bold;PSFOR587-13;-
Coleoptera;Buprestidae;Lamprodila;Lamprodila festiva;Austria;Bold;TDAAT961-20;-
Coleoptera;Laemophloeidae;Lathropus;Lathropus sepicola;France;Bold;PSFOR637-13;-
Coleoptera;Cerambycidae;Leiopus;Leiopus nebulosus;Austria;Bold;TDAOE622-21;-
Coleoptera;Cerambycidae;Leiopus;Leiopus nebulosus;United Kingdom;Bold;UKAN375-22;-
Coleoptera;Cerambycidae;Leptura;Leptura aethiops;France;Bold;PSFOR129-13;-
Coleoptera;Cerambycidae;Leptura;Leptura aurulenta;France;Bold;PSFOR058-13;-
Coleoptera;Staphylinidae;Leptusa;Leptusa fumida;United Kingdom;Bold;UKAN503-22;-
Coleoptera;Staphylinidae;Leptusa;Leptusa pulchella;United Kingdom;Bold;UKAN461-22;-
Coleoptera;Bostrichidae;Lichenophanes;Lichenophanes varius;France;Bold;PSFOR610-13;-
Coleoptera;Salpingidae;Lissodema;Lissodema denticolle;United Kingdom;Bold;UKAN542-22;-
Coleoptera;Salpingidae;Lissodema;Lissodema denticolle;France;Bold;PSFOR779-13;-
Coleoptera;Mycetophagidae;Litargus;Litargus connexus;France;Bold;PSFOR358-13;-
Coleoptera;Mycetophagidae;Litargus;Litargus connexus;France;Bold;PSFOR353-13;-
Coleoptera;Lucanidae;Lucanus;Lucanus cervus;Austria;Bold;TDAOE286-21;-
Coleoptera;Endomychidae;Lycoperdina;Lycoperdina bovistae;France;Bold;PSFOR1314-17;-
Coleoptera;Endomychidae;Lycoperdina;Lycoperdina bovistae;France;Bold;PSFOR1315-17;-
Coleoptera;Elmidae;Macronychus;Macronychus quadrituberculatus;United Kingdom;Bold;DTNHM1432-21;-
Coleoptera;Histeridae;Margarinotus;Margarinotus merdarius;France;Bold;PSFOR1352-17;-
Coleoptera;Histeridae;Margarinotus;Margarinotus merdarius;United Kingdom;Bold;UKAN529-22;-
Coleoptera;Histeridae;Margarinotus;Margarinotus striola;Finland;Bold;COLFC450-12;-
Coleoptera;Histeridae;Margarinotus;Margarinotus striola;France;Bold;PSFOR1353-17;-
Coleoptera;Histeridae;Margarinotus;Margarinotus striola;Finland;Bold;COLFE957-13;-
Coleoptera;Histeridae;Margarinotus;Margarinotus striola;Finland;Bold;COLFE956-13;-
Coleoptera;Elateridae;Megapenthes;Megapenthes lugens;France;Bold;PSFOR757-13;-
Coleoptera;Dermestidae;Megatoma;Megatoma undata;France;Bold;PSFOR1268-17;-
Coleoptera;Melandryidae;Melandrya;Melandrya barbata;France;Bold;PSFOR267-13;-
Coleoptera;Elateridae;Melanotus;Melanotus castanipes;France;Bold;PSFOR820-13;-
Coleoptera;Elateridae;Melanotus;Melanotus castanipes;France;Bold;PSFOR821-13;-
Coleoptera;Elateridae;Melanotus;Melanotus villosus;France;Bold;PSFOR822-13;-
Coleoptera;Eucnemidae;Melasis;Melasis buprestoides;France;Bold;PSFOR318-13;-
Coleoptera;Buprestidae;Meliboeus;Meliboeus fulgidicollis;France;Bold;PSFOR1001-14;-
Coleoptera;Tenebrionidae;Menephilus;Menephilus cylindricus;France;Bold;PSFOR1535-17;-
Coleoptera;Cerambycidae;Menesia;Menesia bipunctata;France;Bold;PSFOR061-13;-

Coleoptera;Curculionidae;Mesites;Mesites aquitanus;France;Bold;PSFOR1165-17;-
Coleoptera;Curculionidae;Mesites;Mesites cunipes;France;Bold;PSFOR1166-17;-
Coleoptera;Ptinidae;Mesocoelopus;Mesocoelopus collaris;France;Bold;PSFOR674-13;-
Coleoptera;Ptinidae;Mesocoelopus;Mesocoelopus niger;France;Bold;PSFOR675-13;-
Coleoptera;Cerambycidae;Mesosa;Mesosa nebulosa;France;Bold;PSFOR307-13;-
Coleoptera;Ptinidae;Mesothes;Mesothes ferrugineus;France;Bold;PSFOR1486-17;-
Coleoptera;Eucnemidae;Microrhagus;Microrhagus emyi;France;Bold;PSFOR595-13;-
Coleoptera;Eucnemidae;Microrhagus;Microrhagus emyi;France;Bold;PSFOR1334-17;-
Coleoptera;Eucnemidae;Microrhagus;Microrhagus lepidus;France;Bold;PSFOR320-13;-
Coleoptera;Eucnemidae;Microrhagus;Microrhagus lepidus;Norway;Bold;NOCLP1708-19;-
Coleoptera;Eucnemidae;Microrhagus;Microrhagus pygmaeus;France;Bold;PSFOR321-13;-
Coleoptera;Eucnemidae;Microrhagus;Microrhagus pygmaeus;Norway;Bold;NOCLP1713-19;-
Coleoptera;Eucnemidae;Microrhagus;Microrhagus pygmaeus;Norway;Bold;NOCLP1711-19;-
Coleoptera;Ptinidae;Mizodorcatoma;Mizodorcatoma dommeri;France;Bold;PSFOR1463-17;-
Coleoptera;Mordellidae;Mordellistena;Mordellistena humeralis;Norway;Bold;NOCLP3576-22;-
Coleoptera;Mordellidae;Mordellistena;Mordellistena humeralis;Austria;Bold;TDAOE577-21;-
Coleoptera;Mordellidae;Mordellistena;Mordellistena humeralis;Norway;Bold;NOCLP3577-22;-
Coleoptera;Endomychidae;Mycetina;Mycetina cruciata;France;Bold;PSFOR879-14;-
Coleoptera;Endomychidae;Mycetina;Mycetina cruciata;France;Bold;PSFOR235-13;-
Coleoptera;Endomychidae;Mycetina;Mycetina cruciata;France;Bold;PSFOR903-14;-
Coleoptera;Tenebrionidae;Mycetochara;Mycetochara humeralis;France;Bold;PSFOR210-13;-
Coleoptera;Tenebrionidae;Mycetochara;Mycetochara thoracica;France;Bold;PSFOR213-13;-
Coleoptera;Mycetophagidae;Mycetophagus;Mycetophagus atomarius;France;Bold;PSFOR185-13;-
Coleoptera;Mycetophagidae;Mycetophagus;Mycetophagus decempunctatus;France;Bold;PSFOR186-13;-
Coleoptera;Mycetophagidae;Mycetophagus;Mycetophagus fulvicollis;France;Bold;PSFOR466-13;-
Coleoptera;Mycetophagidae;Mycetophagus;Mycetophagus multipunctatus;France;Bold;PSFOR354-13;-
Coleoptera;Mycetophagidae;Mycetophagus;Mycetophagus piceus;Finland;Bold;COLFD273-12;-
Coleoptera;Mycetophagidae;Mycetophagus;Mycetophagus piceus;France;Bold;PSFOR355-13;-
Coleoptera;Mycetophagidae;Mycetophagus;Mycetophagus quadripustulatus;France;Bold;PSFOR904-14;-
Coleoptera;Mycetophagidae;Mycetophagus;Mycetophagus quadripustulatus;France;Bold;PSFOR352-13;-
Coleoptera;Oedemeridae;Nacerdes;Nacerdes carniolica;France;Bold;PSFOR657-13;-
Coleoptera;Tenebrionidae;Nalassus;Nalassus alpigradus;France;Bold;PSFOR1555-17;-
Coleoptera;Eucnemidae;Nematodes;Nematodes filum;France;Bold;PSFOR1329-17;-
Coleoptera;Eucnemidae;Nematodes;Nematodes filum;France;Bold;PSFOR596-13;-
Coleoptera;Tenebrionidae;Neomida;Neomida haemorrhoidalis;France;Bold;PSFOR230-13;-
Coleoptera;Laemophloeidae;Notolaemus;Notolaemus castaneus;Czech Republic;Bold;PSFOR1361-17;-
Coleoptera;Staphylinidae;Nudobius;Nudobius lentus;Norway;Bold;NOCLP3530-22;-
Coleoptera;Staphylinidae;Nudobius;Nudobius lentus;Norway;Bold;NOCLP3531-22;-
Coleoptera;Cerambycidae;Obrium;Obrium brunneum;France;Bold;PSFOR068-13;-
Coleoptera;Cerambycidae;Obrium;Obrium cantharinum;Finland;Bold;COLFA548-12;-
Coleoptera;Cerambycidae;Obrium;Obrium cantharinum;France;Bold;PSFOR069-13;-
Coleoptera;Cerambycidae;Obrium;Obrium cantharinum;Finland;Bold;COLFA547-12;-
Coleoptera;Bothrideridae;Ogmoderes;Ogmoderes angusticollis;France;Bold;PSFOR1099-17;-
Coleoptera;Cleridae;Opilo;Opilo mollis;France;Bold;PSFOR625-13;-
Coleoptera;Cleridae;Opilo;Opilo mollis;France;Bold;PSFOR374-13;-
Coleoptera;Melandryidae;Orchesia;Orchesia fasciata;France;Bold;PSFOR269-13;-
Coleoptera;Melandryidae;Orchesia;Orchesia micans;France;Bold;PSFOR271-13;-
Coleoptera;Melandryidae;Orchesia;Orchesia minor;France;Bold;PSFOR909-14;-
Coleoptera;Melandryidae;Orchesia;Orchesia undulata;France;Bold;PSFOR365-13;-
Coleoptera;Dermestidae;Orphilus;Orphilus niger;France;Bold;PSFOR1274-17;-
Coleoptera;Dermestidae;Orphilus;Orphilus niger;France;Bold;PSFOR1273-17;-
Coleoptera;Curculionidae;Orthotomicus;Orthotomicus suturalis;Finland;Bold;COLFB225-12;-
Coleoptera;Curculionidae;Orthotomicus;Orthotomicus suturalis;Finland;Bold;COLFD139-12;-
Coleoptera;Curculionidae;Orthotomicus;Orthotomicus suturalis;Finland;Bold;COLFD140-12;-
Coleoptera;Melandryidae;Osphya;Osphya bipunctata;France;Bold;PSFOR1401-17;-
Coleoptera;Melandryidae;Osphya;Osphya bipunctata;France;Bold;PSFOR275-13;-
Coleoptera;Melandryidae;Osphya;Osphya bipunctata;France;Bold;PSFOR1402-17;-
Coleoptera;Melandryidae;Osphya;Osphya bipunctata;France;Bold;PSFOR1398-17;-
Coleoptera;Bothrideridae;Oxylaemus;Oxylaemus variolosus;France;Bold;PSFOR1100-17;-
Coleoptera;Cerambycidae;Oxymirus;Oxymirus cursor;France;Bold;PSFOR889-14;-
Coleoptera;Cerambycidae;Oxymirus;Oxymirus cursor;Finland;Bold;COLFB864-12;-
Coleoptera;Cerambycidae;Oxymirus;Oxymirus cursor;Italy;Bold;PSFOR310-13;-
Coleoptera;Cerambycidae;Pachyta;Pachyta lamed;Finland;Bold;COLFA495-12;-
Coleoptera;Cerambycidae;Pachytodes;Pachytodes cerambyciformis;France;Bold;PSFOR134-13;-
Coleoptera;Cerambycidae;Pachytodes;Pachytodes erraticus;France;Bold;PSFOR135-13;-
Coleoptera;Tenebrionidae;Palorus;Palorus depressus;France;Bold;PSFOR1540-17;-
Coleoptera;Tenebrionidae;Palorus;Palorus depressus;France;Bold;PSFOR1539-17;-
Coleoptera;Cerambycidae;Parmena;Parmena unifasciata;France;Bold;PSFOR1132-17;-
Coleoptera;Cerambycidae;Parmena;Parmena unifasciata;France;Bold;PSFOR1133-17;-
Coleoptera;Histeridae;Paromalus;Paromalus flavicornis;France;Bold;PSFOR479-13;-
Coleoptera;Histeridae;Paromalus;Paromalus flavicornis;France;Bold;PSFOR1012-14;-
Coleoptera;Cucujidae;Pediacus;Pediacus depressus;France;Bold;PSFOR630-13;-
Coleoptera;Cucujidae;Pediacus;Pediacus dermestoides;France;Bold;PSFOR301-13;-
Coleoptera;Cucujidae;Pediacus;Pediacus fuscus;Finland;Bold;COLFD282-12;-
Coleoptera;Cerambycidae;Penichroa;Penichroa fasciata;France;Bold;PSFOR073-13;-
Coleoptera;Tenebrionidae;Pentaphyllus;Pentaphyllus chrysomeloides;France;Bold;PSFOR1533-17;-
Coleoptera;Tenebrionidae;Pentaphyllus;Pentaphyllus chrysomeloides;France;Bold;PSFOR231-13;-
Coleoptera;Buprestidae;Phaenops;Phaenops cyanea;France;Bold;PSFOR588-13;-

Coleoptera;Cerylonidae;Philothermus;Philothermus semistriatus;France;Bold;PSFOR1140-17;-
Coleoptera;Curculionidae;Phloeosinus;Phloeosinus thujae;United Kingdom;Bold;UKAN389-22;-
Coleoptera;Phloeostichidae;Phloeostichus;Phloeostichus denticollis;France;Bold;PSFOR905-14;-
Coleoptera;Phloeostichidae;Phloeostichus;Phloeostichus denticollis;France;Bold;PSFOR634-13;-
Coleoptera;Curculionidae;Phloeotribus;Phloeotribus pubifrons;France;Bold;PSFOR1260-17;-
Coleoptera;Curculionidae;Phloeotribus;Phloeotribus spinulosus;Norway;Bold;NOCLP1742-19;-
Coleoptera;Staphylinidae;Phyllodrepa;Phyllodrepa melanocephala;Norway;Bold;NOCLP1795-19;-
Coleoptera;Staphylinidae;Phyllodrepa;Phyllodrepa nigra;Norway;Bold;NOCLP1794-19;-
Coleoptera;Aderidae;Phytobaenus;Phytobaenus amabilis;France;Bold;PSFOR1078-17;-
Coleoptera;Curculionidae;Pissodes;Pissodes castaneus;Norway;Bold;NOCLP1416-19;-
Coleoptera;Curculionidae;Pissodes;Pissodes piceae;Austria;Bold;TDAAT1093-20;-
Coleoptera;Curculionidae;Pissodes;Pissodes pini;Norway;Bold;NOCLP1424-19;-
Coleoptera;Curculionidae;Pissodes;Pissodes pini;Norway;Bold;NOCLP1423-19;-
Coleoptera;Curculionidae;Pissodes;Pissodes piniphilus;Norway;Bold;NOCLP1417-19;-
Coleoptera;Curculionidae;Pissodes;Pissodes piniphilus;Norway;Bold;NOCLP1418-19;-
Coleoptera;Curculionidae;Pissodes;Pissodes validirostris;Norway;Bold;NOCLP1420-19;-
Coleoptera;Curculionidae;Pissodes;Pissodes validirostris;Norway;Bold;NOCLP1422-19;-
Coleoptera;Curculionidae;Pissodes;Pissodes validirostris;Norway;Bold;NOCLP1421-19;-
Coleoptera;Curculionidae;Pityogenes;Pityogenes bidentatus;Norway;Bold;NOCLP1762-19;-
Coleoptera;Curculionidae;Pityogenes;Pityogenes chalcographus;Norway;Bold;NOCLP1761-19;-
Coleoptera;Curculionidae;Pityogenes;Pityogenes chalcographus;Norway;Bold;NOCLP1760-19;-
Coleoptera;Nitidulidae;Pityophagus;Pityophagus ferrugineus;Norway;Bold;NOCLP3138-22;-
Coleoptera;Nitidulidae;Pityophagus;Pityophagus ferrugineus;France;Bold;PSFOR490-13;-
Coleoptera;Nitidulidae;Pityophagus;Pityophagus laevior;France;Bold;PSFOR803-13;-
Coleoptera;Curculionidae;Pityophthorus;Pityophthorus buyssoni;France;Bold;PSFOR1223-17;-
Coleoptera;Curculionidae;Pityophthorus;Pityophthorus buyssoni;France;Bold;PSFOR1235-17;-
Coleoptera;Curculionidae;Pityophthorus;Pityophthorus buyssoni;France;Bold;PSFOR1236-17;-
Coleoptera;Curculionidae;Pityophthorus;Pityophthorus lichtensteinii;Norway;Bold;NOCLP1753-19;-
Coleoptera;Curculionidae;Pityophthorus;Pityophthorus pubescens;Norway;Bold;NOCLP1757-19;-
Coleoptera;Curculionidae;Pityophthorus;Pityophthorus pubescens;United Kingdom;Bold;UKAN463-22;-
Coleoptera;Curculionidae;Pityophthorus;Pityophthorus pubescens;Norway;Bold;NOCLP1755-19;-
Coleoptera;Laemophloeidae;Placonotus;Placonotus testaceus;France;Bold;PSFOR312-13;-
Coleoptera;Staphylinidae;Placusa;Placusa tachyporoides;Finland;Bold;COLFD128-12;-
Coleoptera;Staphylinidae;Placusa;Placusa tachyporoides;Finland;Bold;COLFD127-12;-
Coleoptera;Cerambycidae;Plagionotus;Plagionotus detritus;France;Bold;PSFOR075-13;-
Coleoptera;Cerambycidae;Plagionotus;Plagionotus detritus;Austria;Bold;TDAAT1085-20;-
Coleoptera;Lycidae;Platycis;Platycis minutus;France;Bold;PSFOR641-13;-
Coleoptera;Histeridae;Platysoma;Platysoma angustatum;Finland;Bold;COLFD585-12;-
Coleoptera;Histeridae;Platysoma;Platysoma angustatum;Finland;Bold;COLFD584-12;-
Coleoptera;Histeridae;Platysoma;Platysoma elongatum;France;Bold;PSFOR1354-17;-
Coleoptera;Histeridae;Platysoma;Platysoma elongatum;Austria;Bold;TDAAT1937-20;-
Coleoptera;Histeridae;Platysoma;Platysoma elongatum;France;Bold;PSFOR495-13;-
Coleoptera;Histeridae;Platysoma;Platysoma elongatum;France;Bold;PSFOR1355-17;-
Coleoptera;Histeridae;Platysoma;Platysoma filiforme;France;Bold;PSFOR1356-17;-
Coleoptera;Histeridae;Platysoma;Platysoma lineare;Finland;Bold;COLFE946-13;-
Coleoptera;Anthribidae;Platystomos;Platystomos albinus;France;Bold;PSFOR014-13;-
Coleoptera;Anthribidae;Platystomos;Platystomos albinus;Norway;Bold;NOCLP3316-22;-
Coleoptera;Anthribidae;Platystomos;Platystomos albinus;France;Bold;PSFOR378-13;-
Coleoptera;Anthribidae;Platystomos;Platystomos albinus;Norway;Bold;NOCLP3315-22;-
Coleoptera;Histeridae;Plegaderus;Plegaderus vulneratus;Finland;Bold;COLFD132-12;-
Coleoptera;Cerambycidae;Pogonocherus;Pogonocherus fasciculatus;Finland;Bold;COLFB883-12;-
Coleoptera;Cerambycidae;Pogonocherus;Pogonocherus fasciculatus;France;Bold;PSFOR150-13;-
Coleoptera;Cerambycidae;Pogonocherus;Pogonocherus fasciculatus;Finland;Bold;COLFA561-12;-
Coleoptera;Cerambycidae;Pogonocherus;Pogonocherus hispidus;Finland;Bold;COLFB895-12;-
Coleoptera;Cerambycidae;Pogonocherus;Pogonocherus hispidus;France;Bold;PSFOR152-13;-
Coleoptera;Cerambycidae;Pogonocherus;Pogonocherus hispidus;France;Bold;PSFOR309-13;-
Coleoptera;Cerambycidae;Pogonocherus;Pogonocherus ovatus;France;Bold;PSFOR908-14;-
Coleoptera;Curculionidae;Polygraphus;Polygraphus poligraphus;France;Bold;PSFOR713-13;-
Coleoptera;Tenebrionidae;Prionychus;Prionychus fairmairii;France;Bold;PSFOR1522-17;-
Coleoptera;Elateridae;Procraerus;Procraerus tibialis;France;Bold;PSFOR341-13;-
Coleoptera;Elateridae;Procraerus;Procraerus tibialis;France;Bold;PSFOR977-14;-
Coleoptera;Scarabaeidae;Protaetia;Protaetia cuprea;France;Bold;PSFOR907-14;-
Coleoptera;Curculionidae;Pselactus;Pselactus spadix;France;Bold;PSFOR1172-17;-
Coleoptera;Cerambycidae;Pseudosphegesthes;Pseudosphegesthes cinerea;France;Bold;PSFOR080-13;-
Coleoptera;Cerambycidae;Pseudosphegesthes;Pseudosphegesthes cinerea;France;Bold;PSFOR1120-17;-
Coleoptera;Bostrichidae;Psoa;Psoa dubia;France;Bold;PSFOR1097-17;-
Coleoptera;Curculionidae;Pteleobius;Pteleobius vittatus;France;Bold;PSFOR1224-17;-
Coleoptera;Ptiliidae;Ptenidium;Ptenidium nitidum;United Kingdom;Bold;UKAN535-22;-
Coleoptera;Cryptophagidae;Pteryngium;Pteryngium crenatum;Finland;Bold;COLFC512-12;-
Coleoptera;Ptinidae;Ptilinus;Ptilinus pectinicornis;Austria;Bold;TDAOE412-21;-
Coleoptera;Ptinidae;Ptilinus;Ptilinus pectinicornis;Austria;Bold;TDAOE624-21;-
Coleoptera;Ptinidae;Ptinomorphus;Ptinomorphus imperialis;France;Bold;PSFOR893-14;-
Coleoptera;Ptinidae;Ptinomorphus;Ptinomorphus imperialis;France;Bold;PSFOR380-13;-
Coleoptera;Ptinidae;Ptinomorphus;Ptinomorphus imperialis;United Kingdom;Bold;UKAN367-22;-
Coleoptera;Ptinidae;Ptinus;Ptinus lichenum;France;Bold;PSFOR684-13;-
Coleoptera;Cerambycidae;Purpuricenus;Purpuricenus budensis;France;Bold;PSFOR1121-17;-
Coleoptera;Cerambycidae;Purpuricenus;Purpuricenus kaehleri;France;Bold;PSFOR081-13;-
Coleoptera;Lycidae;Pyropterus;Pyropterus nigroruber;France;Bold;PSFOR642-13;-

Coleoptera;Staphylinidae;Quedius;Quedius cruentus;Norway;Bold;NOCLP3205-22;-
Coleoptera;Staphylinidae;Quedius;Quedius cruentus;Norway;Bold;NOCLP3206-22;-
Coleoptera;Staphylinidae;Quedius;Quedius cruentus;United Kingdom;Bold;UKAN431-22;-
Coleoptera;Staphylinidae;Quedius;Quedius microps;Finland;Bold;COLFG224-13;-
Coleoptera;Staphylinidae;Quedius;Quedius xanthopus;Norway;Bold;NOCLP3209-22;-
Coleoptera;Anthribidae;Rhaphitropis;Rhaphitropis marchicus;France;Bold;PSFOR1086-17;-
Coleoptera;Monotomidae;Rhizophagus;Rhizophagus bipustulatus;France;Bold;PSFOR858-14;-
Coleoptera;Monotomidae;Rhizophagus;Rhizophagus bipustulatus;France;Bold;PSFOR857-14;-
Coleoptera;Monotomidae;Rhizophagus;Rhizophagus bipustulatus;France;Bold;PSFOR1046-14;-
Coleoptera;Monotomidae;Rhizophagus;Rhizophagus bipustulatus;United Kingdom;Bold;UKAN528-22;-
Coleoptera;Monotomidae;Rhizophagus;Rhizophagus bipustulatus;France;Bold;PSFOR567-13;-
Coleoptera;Monotomidae;Rhizophagus;Rhizophagus dispar;France;Bold;PSFOR563-13;-
Coleoptera;Monotomidae;Rhizophagus;Rhizophagus dispar;United Kingdom;Bold;UKAN504-22;-
Coleoptera;Monotomidae;Rhizophagus;Rhizophagus ferrugineus;France;Bold;PSFOR562-13;-
Coleoptera;Monotomidae;Rhizophagus;Rhizophagus nitidulus;France;Bold;PSFOR1390-17;-
Coleoptera;Monotomidae;Rhizophagus;Rhizophagus parvulus;France;Bold;PSFOR570-13;-
Coleoptera;Monotomidae;Rhizophagus;Rhizophagus perforatus;France;Bold;PSFOR856-14;-
Coleoptera;Monotomidae;Rhizophagus;Rhizophagus unicolor;France;Bold;PSFOR860-14;-
Coleoptera;Monotomidae;Rhizophagus;Rhizophagus unicolor;France;Bold;PSFOR1392-17;-
Coleoptera;Curculionidae;Rhyncolus;Rhyncolus ater;Norway;Bold;NOCLP1406-19;-
Coleoptera;Cerambycidae;Ropalopus;Ropalopus ungaricus;Slovakia;Bold;PSFOR1122-17;-
Coleoptera;Cerambycidae;Rutpela;Rutpela maculata;France;Bold;PSFOR006-13;-
Coleoptera;Salpingidae;Salpingus;Salpingus planirostris;France;Bold;PSFOR347-13;-
Coleoptera;Salpingidae;Salpingus;Salpingus planirostris;United Kingdom;Bold;UKAN355-22;-
Coleoptera;Salpingidae;Salpingus;Salpingus planirostris;United Kingdom;Bold;UKAN460-22;-
Coleoptera;Cerambycidae;Saperda;Saperda carcharias;Finland;Bold;COLFA377-12;-
Coleoptera;Cerambycidae;Saperda;Saperda perforata;Finland;Bold;LEFIJ1888-13;-
Coleoptera;Cerambycidae;Saperda;Saperda scalaris;Finland;Bold;LEFIJ1986-13;-
Coleoptera;Bostrichidae;Scobicia;Scobicia chevrieri;France;Bold;PSFOR612-13;-
Coleoptera;Bostrichidae;Scobicia;Scobicia pustulata;France;Bold;PSFOR613-13;-
Coleoptera;Curculionidae;Scolytus;Scolytus carpini;France;Bold;PSFOR1225-17;-
Coleoptera;Curculionidae;Scolytus;Scolytus intricatus;France;Bold;PSFOR1073-14;-
Coleoptera;Curculionidae;Scolytus;Scolytus intricatus;Norway;Bold;NOCLP1747-19;-
Coleoptera;Curculionidae;Scolytus;Scolytus koenigi;France;Bold;PSFOR1226-17;-
Coleoptera;Curculionidae;Scolytus;Scolytus rugulosus;Norway;Bold;NOCLP1748-19;-
Coleoptera;Curculionidae;Scolytus;Scolytus rugulosus;United Kingdom;Bold;UKAN381-22;-
Coleoptera;Melandryidae;Serropalpus;Serropalpus barbatus;Italy;Bold;PSFOR279-13;-
Coleoptera;Staphylinidae;Siagonium;Siagonium quadricorne;United Kingdom;Bold;UKAN526-22;-
Coleoptera;Silvanidae;Silvanoprus;Silvanoprus fagi;Finland;Bold;COLFB250-12;-
Coleoptera;Silvanidae;Silvanoprus;Silvanoprus fagi;France;Bold;PSFOR302-13;-
Coleoptera;Silvanidae;Silvanus;Silvanus bidentatus;France;Bold;PSFOR1031-14;-
Coleoptera;Lucanidae;Sinodendron;Sinodendron cylindricum;Finland;Bold;COLFG079-13;-
Coleoptera;Lucanidae;Sinodendron;Sinodendron cylindricum;Finland;Bold;COLFA129-10;-
Coleoptera;Nitidulidae;Soronia;Soronia grisea;France;Bold;PSFOR1074-14;-
Coleoptera;Nitidulidae;Soronia;Soronia grisea;France;Bold;PSFOR290-13;-
Coleoptera;Nitidulidae;Soronia;Soronia grisea;Norway;Bold;NOCLP3134-22;-
Coleoptera;Nitidulidae;Soronia;Soronia grisea;Norway;Bold;NOCLP3133-22;-
Coleoptera;Nitidulidae;Soronia;Soronia oblonga;France;Bold;PSFOR648-13;-
Coleoptera;Nitidulidae;Soronia;Soronia oblonga;France;Bold;PSFOR1413-17;-
Coleoptera;Nitidulidae;Soronia;Soronia punctatissima;Finland;Bold;COLFE930-13;-
Coleoptera;Nitidulidae;Soronia;Soronia punctatissima;France;Bold;PSFOR916-14;-
Coleoptera;Nitidulidae;Soronia;Soronia punctatissima;France;Bold;PSFOR649-13;-
Coleoptera;Sphindidae;Sphindus;Sphindus dubius;France;Bold;PSFOR667-13;-
Coleoptera;Ptinidae;Stagetus;Stagetus byrrhoides;France;Bold;PSFOR1462-17;-
Coleoptera;Ptinidae;Stagetus;Stagetus elongatus;France;Bold;PSFOR1467-17;-
Coleoptera;Elateridae;Stenagostus;Stenagostus rhombeus;France;Bold;PSFOR947-14;-
Coleoptera;Elateridae;Stenagostus;Stenagostus rhombeus;France;Bold;PSFOR331-13;-
Coleoptera;Elateridae;Stenagostus;Stenagostus rhombeus;France;Bold;PSFOR760-13;-
Coleoptera;Cerambycidae;Stenocorus;Stenocorus meridianus;France;Bold;PSFOR092-13;-
Coleoptera;Tenebrionidae;Stenomax;Stenomax aeneus;France;Bold;PSFOR224-13;-
Coleoptera;Tenebrionidae;Stenomax;Stenomax aeneus;France;Bold;PSFOR1035-14;-
Coleoptera;Tenebrionidae;Stenomax;Stenomax aeneus;Austria;Bold;TDAAT1091-20;-
Coleoptera;Tenebrionidae;Stenomax;Stenomax aeneus;Austria;Bold;TDAOE325-21;-
Coleoptera;Cerambycidae;Stenostola;Stenostola dubia;France;Bold;PSFOR157-13;-
Coleoptera;Cerambycidae;Stenurella;Stenurella sennii;France;Bold;PSFOR1136-17;-
Coleoptera;Cerambycidae;Stenurella;Stenurella sennii;France;Bold;PSFOR1137-17;-
Coleoptera;Bostrichidae;Stephanopachys;Stephanopachys linearis;Finland;Bold;COLFD280-12;-
Coleoptera;Cerambycidae;Stictoleptura;Stictoleptura cordigera;France;Bold;PSFOR094-13;-
Coleoptera;Anamorphidae;Symbiotes;Symbiotes gibberosus;France;Bold;PSFOR632-13;-
Coleoptera;Anamorphidae;Symbiotes;Symbiotes latus;France;Bold;PSFOR633-13;-
Coleoptera;Curculionidae;Taphrorychus;Taphrorychus bicolor;Austria;Bold;TDAOE340-21;-
Coleoptera;Curculionidae;Taphrorychus;Taphrorychus villifrons;France;Bold;PSFOR419-13;-
Coleoptera;Curculionidae;Taphrorychus;Taphrorychus villifrons;France;Bold;PSFOR418-13;-
Coleoptera;Tenebrionidae;Tenebrio;Tenebrio obscurus;France;Bold;PSFOR1537-17;-
Coleoptera;Tenebrionidae;Tenebrio;Tenebrio punctipennis;Spain;Bold;PSFOR1541-17;-
Coleoptera;Tenebrionidae;Tenebrio;Tenebrio punctipennis;Spain;Bold;PSFOR1542-17;-
Coleoptera;Trogossitidae;Tenebroides;Tenebroides fuscus;France;Bold;PSFOR1037-14;-
Coleoptera;Histeridae;Teretrius;Teretrius parasita;France;Bold;PSFOR1351-17;-

Coleoptera;Tetratomidae;Tetratoma;Tetratoma ancora;France;Bold;PSFOR912-14;-
Coleoptera;Tetratomidae;Tetratoma;Tetratoma ancora;France;Bold;PSFOR292-13;-
Coleoptera;Tetratomidae;Tetratoma;Tetratoma ancora;France;Bold;PSFOR291-13;-
Coleoptera;Tetratomidae;Tetratoma;Tetratoma ancora;France;Bold;PSFOR486-13;-
Coleoptera;Tetratomidae;Tetratoma;Tetratoma fungorum;Finland;Bold;COLFB496-12;-
Coleoptera;Cerambycidae;Tetropium;Tetropium castaneum;Russia;Bold;VVGPL2876-15;-
Coleoptera;Cerambycidae;Tetropium;Tetropium castaneum;Russia;Bold;VVGPL2878-15;-
Coleoptera;Cerambycidae;Tetropium;Tetropium castaneum;Russia;Bold;VVGPL2877-15;-
Coleoptera;Cleridae;Thanasimus;Thanasimus formicarius;France;Bold;PSFOR372-13;-
Coleoptera;Cleridae;Thanasimus;Thanasimus formicarius;France;Bold;PSFOR1064-14;-
Coleoptera;Curculionidae;Tomicus;Tomicus minor;Norway;Bold;NOCLP3342-22;-
Coleoptera;Curculionidae;Tomicus;Tomicus minor;Norway;Bold;NOCLP3343-22;-
Coleoptera;Curculionidae;Tomicus;Tomicus piniperda;France;Bold;PSFOR718-13;-
Coleoptera;Mordellidae;Tomoxia;Tomoxia bucephala;Finland;Bold;COLFG081-13;-
Coleoptera;Rhadalidae;Trichoceble;Trichoceble floralis;France;Bold;PSFOR1389-17;-
Coleoptera;Rhadalidae;Trichoceble;Trichoceble floralis;France;Bold;PSFOR1388-17;-
Coleoptera;Cerambycidae;Trichoferus;Trichoferus fasciculatus;France;Bold;PSFOR1123-17;-
Coleoptera;Cerambycidae;Trichoferus;Trichoferus pallidus;France;Bold;PSFOR016-13;-
Coleoptera;Erotylidae;Triplax;Triplax aenea;France;Bold;PSFOR598-13;-
Coleoptera;Erotylidae;Triplax;Triplax collaris;France;Bold;PSFOR599-13;-
Coleoptera;Erotylidae;Triplax;Triplax lepida;France;Bold;PSFOR601-13;-
Coleoptera;Erotylidae;Triplax;Triplax lepida;France;Bold;PSFOR998-14;-
Coleoptera;Erotylidae;Triplax;Triplax melanocephala;France;Bold;PSFOR1326-17;-
Coleoptera;Erotylidae;Triplax;Triplax melanocephala;France;Bold;PSFOR602-13;-
Coleoptera;Erotylidae;Triplax;Triplax rufipes;France;Bold;PSFOR603-13;-
Coleoptera;Erotylidae;Triplax;Triplax russica;France;Bold;PSFOR604-13;-
Coleoptera;Erotylidae;Triplax;Triplax russica;Finland;Bold;COLFC696-12;-
Coleoptera;Erotylidae;Tritoma;Tritoma bipustulata;France;Bold;PSFOR323-13;-
Coleoptera;Throscidae;Trixagus;Trixagus atticus;Austria;Bold;TDAAT876-20;-
Coleoptera;Anthribidae;Tropideres;Tropideres albirostris;France;Bold;PSFOR011-13;-
Coleoptera;Curculionidae;Trypodendron;Trypodendron domesticum;Norway;Bold;NOCLP1784-19;-
Coleoptera;Curculionidae;Trypodendron;Trypodendron lineatum;Norway;Bold;NOCLP1782-19;-
Coleoptera;Mycetophagidae;Typhaea;Typhaea stercorea;Finland;Bold;COLFB406-12;-
Coleoptera;Mycetophagidae;Typhaea;Typhaea stercorea;Finland;Bold;COLFB408-12;-
Coleoptera;Mycetophagidae;Typhaea;Typhaea stercorea;Finland;Bold;COLFB407-12;-
Coleoptera;Mycetophagidae;Typhaea;Typhaea stercorea;France;Bold;PSFOR190-13;-
Coleoptera;Tenebrionidae;Uloma;Uloma rufa;France;Bold;PSFOR228-13;-
Coleoptera;Scarabaeidae;Valgus;Valgus hemipterus;Austria;Bold;TDAAT465-19;-
Coleoptera;Scarabaeidae;Valgus;Valgus hemipterus;Austria;Bold;TDAAT1989-20;-
Coleoptera;Scarabaeidae;Valgus;Valgus hemipterus;France;Bold;PSFOR247-13;-
Coleoptera;Salpingidae;Vincenzellus;Vincenzellus ruficollis;France;Bold;PSFOR461-13;-
Coleoptera;Salpingidae;Vincenzellus;Vincenzellus ruficollis;France;Bold;PSFOR013-13;-
Coleoptera;Curculionidae;Xyleborinus;Xyleborinus saxesenii;Norway;Bold;NOCLP1776-19;-
Coleoptera;Curculionidae;Xyleborus;Xyleborus cryptographus;France;Bold;PSFOR720-13;-
Coleoptera;Curculionidae;Xyleborus;Xyleborus cryptographus;Germany;Bold;SCOLY263-11;-
Coleoptera;Curculionidae;Xyleborus;Xyleborus monographus;Norway;Bold;NOCLP1790-19;-
Coleoptera;Curculionidae;Xylechinus;Xylechinus pilosus;Finland;Bold;COLFC351-12;-
Coleoptera;Curculionidae;Xylechinus;Xylechinus pilosus;Finland;Bold;COLFC352-12;-
Coleoptera;Melandryidae;Xylita;Xylita laevigata;France;Bold;PSFOR281-13;-
Coleoptera;Bostrichidae;Xylopertha;Xylopertha praeusta;France;Bold;PSFOR616-13;-
Coleoptera;Bostrichidae;Xylopertha;Xylopertha praeusta;France;Bold;PSFOR812-13;-
Coleoptera;Bostrichidae;Xylopertha;Xylopertha praeusta;France;Bold;PSFOR1094-17;-
Coleoptera;Bostrichidae;Xylopertha;Xylopertha retusa;France;Bold;PSFOR617-13;-
Coleoptera;Bostrichidae;Xylopertha;Xylopertha retusa;France;Bold;PSFOR992-14;-
Coleoptera;Eucnemidae;Xylophilus;Xylophilus corticalis;France;Bold;PSFOR869-14;-
Coleoptera;Eucnemidae;Xylophilus;Xylophilus corticalis;France;Bold;PSFOR597-13;-
Coleoptera;Eucnemidae;Xylophilus;Xylophilus corticalis;France;Bold;PSFOR1338-17;-
Coleoptera;Eucnemidae;Xylophilus;Xylophilus testaceus;Czech Republic;Bold;PSFOR1337-17;-
Coleoptera;Cerambycidae;Xylotrechus;Xylotrechus antilope;France;Bold;PSFOR024-13;-
Coleoptera;Cerambycidae;Xylotrechus;Xylotrechus antilope;France;Bold;PSFOR169-13;-
Coleoptera;Cerambycidae;Xylotrechus;Xylotrechus arvicola;France;Bold;PSFOR170-13;-
Coleoptera;Cerambycidae;Xylotrechus;Xylotrechus arvicola;France;Bold;PSFOR019-13;-
;;;;;;
;;;;;;

Appendix_4_DataRemoved_after_CoordinateCleanerCheck;;;;;;;
order_name;family_name;genus_name;species_name;country;sourceDatabase;processid;genbank_accessio
n
Hymenoptera;Apidae;Andrena;Andrena hattorfiana;Croatia;GenBank;-;KJ838690
Hymenoptera;Apidae;Andrena;Andrena hattorfiana;Turkey;GenBank;-;KJ839559
Hymenoptera;Apidae;Andrena;Andrena hattorfiana;Finland;GenBank;-;MZ623535
Hymenoptera;Apidae;Andrena;Andrena hattorfiana;Austria;Bold;ABEE162-17;-
Hymenoptera;Apidae;Andrena;Andrena ovatula;Austria;Bold;ABEE140-17;-
Hymenoptera;Apidae;Andrena;Andrena ovatula;United Kingdom;Bold;BEEEE144-15;-
Hymenoptera;Colletidae;Colletes;Colletes floralis;Norway;Bold;NOBEE044-09;-
Hymenoptera;Colletidae;Colletes;Colletes fodiens;United Kingdom;GenBank;-;KT074041
Hymenoptera;Colletidae;Colletes;Colletes fodiens;France;GenBank;-;MT869551
Hymenoptera;Colletidae;Colletes;Colletes fodiens;United Kingdom;Bold;BEEEE216-15;-
Hymenoptera;Colletidae;Colletes;Colletes succinctus;United Kingdom;Bold;BEEEE139-15;-
Hymenoptera;Apidae;Epeolus;Epeolus cruciger;Finland;GenBank;-;MZ625426
Hymenoptera;Apidae;Epeolus;Epeolus cruciger;Norway;Bold;NOBEE071-09;-
Hymenoptera;Apidae;Epeolus;Epeolus cruciger;Norway;Bold;NOBEE070-09;-
Hymenoptera;Halictidae;Halictus;Halictus quadricinctus;Spain;GenBank;-;KJ839292
Hymenoptera;Halictidae;Lasioglossum;Lasioglossum clypeare;Austria;Bold;ABEE225-17;-
Hymenoptera;Halictidae;Lasioglossum;Lasioglossum costulatum;Croatia;GenBank;-;KJ839220
Hymenoptera;Halictidae;Lasioglossum;Lasioglossum costulatum;Croatia;GenBank;-;KJ839709
Hymenoptera;Halictidae;Lasioglossum;Lasioglossum xanthopus;Turkey;GenBank;-;KJ836625
Hymenoptera;Halictidae;Sphecodes;Sphecodes cristatus;Austria;Bold;ABEE080-17;-
Hymenoptera;Apidae;Triepeolus;Triepeolus tristis;Macedonia;GenBank;-;MN342316
Lepidoptera;Nymphalidae;Erebia;Erebia aethiops;Russia;Bold;GBLN1217-08;AB324833
Lepidoptera;Pieridae;Anthocharis;Anthocharis cardamines;Spain;Bold;GBGL4379-07;AM262796
Lepidoptera;Papilionidae;Parnassius;Parnassius apollo;Finland;GenBank;-;AY346254
Lepidoptera;Papilionidae;Iphiclides;Iphiclides podalirius;Greece;GenBank;-;AY556868
Lepidoptera ;Lycaenidae;Lampides;Lampides boeticus;Turkey;GenBank;-;AY557016
Lepidoptera;Lycaenidae;Aricia;Aricia agestis;Turkey;GenBank;-;AY557031
Lepidoptera;Lycaenidae;Lysandra;Polyommatus bellargus;Turkey;GenBank;-;AY557102
Lepidoptera;Nymphalidae;Maniola;Maniola jurtina;Netherlands;GenBank;-;DQ008088
Lepidoptera;Lycaenidae;Cupido;Cupido osiris;Ukraine;GenBank;-;FJ663445
Lepidoptera;Nymphalidae;Maniola;Maniola jurtina;Ukraine;GenBank;-;FJ663756
Lepidoptera;Nymphalidae;Melanargia;Melanargia galathea;Ukraine;GenBank;-;FJ663761
Lepidoptera;Nymphalidae;Melanargia;Melanargia galathea;Ukraine;GenBank;-;FJ663762
Lepidoptera;Lycaenidae;Polyommatus;Polyommatus daphnis;Ukraine;GenBank;-;FJ663769
Lepidoptera;Lycaenidae;Polyommatus;Polyommatus daphnis;Ukraine;GenBank;-;FJ663770
Lepidoptera;Lycaenidae;Polyommatus;Polyommatus daphnis;Ukraine;GenBank;-;FJ663771
Lepidoptera;Lycaenidae;Polyommatus;Polyommatus daphnis;Ukraine;GenBank;-;FJ663772
Lepidoptera;Lycaenidae;Polyommatus;Polyommatus daphnis;Ukraine;GenBank;-;FJ663773
Lepidoptera;Lycaenidae;Plebejus;Plebejus argus;Ukraine;GenBank;-;FJ663951
Lepidoptera;Lycaenidae;Plebejus;Plebejus argus;Ukraine;GenBank;-;FJ663952
Lepidoptera;Lycaenidae;Polyommatus;Polyommatus icarus;Germany;GenBank;-;GU655005
Lepidoptera;Nymphalidae;Coenonympha;Coenonympha pamphilus;Germany;GenBank;-;GU655020
Lepidoptera;Hesperiidae;Muschampia;Sloperia proto;Spain;GenBank;-;GU662046
Lepidoptera;Hesperiidae;Muschampia;Sloperia proto;Spain;GenBank;-;GU662047
Lepidoptera;Nymphalidae;Coenonympha;Coenonympha pamphilus;Spain;GenBank;-;GU668039
Lepidoptera;Nymphalidae;Erebia;Erebia manto;Romania;Bold;EZROM1060-09;GU669649
Lepidoptera;Nymphalidae;Erebia;Erebia manto;Romania;Bold;EZROM1061-09;GU669650
Lepidoptera;Pieridae;Pieris;Pieris mannii;Spain;GenBank;-;GU669674
Lepidoptera;Hesperiidae;Ochlodes;Ochlodes sylvanus;Spain;GenBank;-;GU669692
Lepidoptera;Nymphalidae;Erebia;Erebia manto;Spain;GenBank;-;GU669838
Lepidoptera;Nymphalidae;Erebia;Erebia manto;Spain;GenBank;-;GU669840
Lepidoptera;Nymphalidae;Erebia;Erebia manto;Spain;GenBank;-;GU669841
Lepidoptera;Nymphalidae;Erebia;Erebia manto;Spain;GenBank;-;GU669842
Lepidoptera;Nymphalidae;Erebia;Erebia manto;Spain;GenBank;-;GU669843
Lepidoptera;Nymphalidae;Vanessa;Vanessa cardui;Spain;GenBank;-;GU669857
Lepidoptera ;Lycaenidae;Leptotes;Leptotes pirithous;Spain;GenBank;-;GU675655
Lepidoptera;Nymphalidae;Danaus;Danaus chrysippus;Spain;GenBank;-;GU675765
Lepidoptera;Nymphalidae;Danaus;Danaus chrysippus;Spain;GenBank;-;GU675948
Lepidoptera;Nymphalidae;Pyronia;Pyronia cecilia;Spain;Bold;EZSPM069-09;GU676027
Lepidoptera;Nymphalidae;Coenonympha;Coenonympha pamphilus;Spain;GenBank;-;GU676033
Lepidoptera;Lycaenidae;Polyommatus;Polyommatus icarus;Spain;Bold;EZSPN446-09;GU676593
Lepidoptera;Nymphalidae;Vanessa;Vanessa cardui;Spain;GenBank;-;GU676594
Lepidoptera;Nymphalidae;Vanessa;Vanessa atalanta;Spain;GenBank;-;GU676595
Lepidoptera;Pieridae;Colias;Colias crocea;Spain;Bold;EZSPN449-09;GU676596
Lepidoptera;Lycaenidae;Celastrina;Celastrina argiolus;Spain;GenBank;-;GU676712
Lepidoptera;Lycaenidae;Polyommatus;Polyommatus icarus;Spain;Bold;EZSPN314-09;GU676726
Lepidoptera;Lycaenidae;Lycaena;Lycaena phlaeas;Spain;GenBank;-;GU676727
Lepidoptera;Pieridae;Pontia;Pieris daplidice;Spain;GenBank;-;GU676728
Lepidoptera;Lycaenidae;Celastrina;Celastrina argiolus;Spain;GenBank;-;GU676729
Lepidoptera ;Pieridae;Gonepteryx;Gonepteryx cleopatra;Spain;GenBank;-;GU676730
Lepidoptera;Nymphalidae;Coenonympha;Coenonympha pamphilus;Spain;GenBank;-;GU676731
Lepidoptera;Nymphalidae;Coenonympha;Coenonympha pamphilus;Spain;GenBank;-;GU676732
Lepidoptera;Nymphalidae;Vanessa;Vanessa cardui;Spain;GenBank;-;GU676733
Lepidoptera;Nymphalidae;Vanessa;Vanessa atalanta;Spain;GenBank;-;GU676735
Lepidoptera;Pieridae;Pieris;Pieris rapae;Spain;GenBank;-;GU676736

Lepidoptera;Pieridae;Pieris;Pieris brassicae;Spain;GenBank;-;GU676737
Lepidoptera ;Nymphalidae;Pararge;Pararge aegeria;Spain;GenBank;-;GU676738
Lepidoptera;Nymphalidae;Vanessa;Vanessa atalanta;Spain;GenBank;-;GU676742
Lepidoptera;Pieridae;Euchloe;Euchloe crameri;Spain;GenBank;-;GU676783
Lepidoptera;Lycaenidae;Phengaris;Maculinea alcon;Spain;GenBank;-;GU676918
Lepidoptera;Lycaenidae;Phengaris;Maculinea alcon;Spain;GenBank;-;GU676921
Lepidoptera;Lycaenidae;Lysandra;Polyommatus coridon;Spain;GenBank;-;GU677024
Lepidoptera;Lycaenidae;Lysandra;Polyommatus coridon;Spain;GenBank;-;GU677027
Lepidoptera;Hesperiidae;Pyrgus;Pyrgus malvae;Finland;Bold;LEFIA1335-10;GU828583
Lepidoptera;Lycaenidae;Polyommatus;Polyommatus icarus;Turkey;GenBank;-;HM116940
Lepidoptera;Nymphalidae;Boloria;Boloria thore;Finland;GenBank;-;HM386883
Lepidoptera;Nymphalidae;Boloria;Boloria thore;Finland;GenBank;-;HM386884
Lepidoptera;Nymphalidae;Boloria;Boloria napaea;Finland;GenBank;-;HM386890
Lepidoptera;Lycaenidae;Favonius;Favonius quercus;Finland;Bold;LEFIB001-10;HM870914
Lepidoptera;Papilionidae;Papilio;Papilio machaon;Finland;GenBank;-;HM871158
Lepidoptera;Nymphalidae;Melitaea;Melitaea cinxia;Finland;GenBank;-;HM871218
Lepidoptera;Hesperiidae;Pyrgus;Pyrgus malvae;Finland;GenBank;-;HM871219
Lepidoptera;Lycaenidae;Favonius;Favonius quercus;Finland;Bold;LEFIB330-10;HM871230
Lepidoptera;Lycaenidae;Lycaena;Lycaena phlaeas;Finland;GenBank;-;HM872111
Lepidoptera;Lycaenidae;Glaucopsyche;Glaucopsyche alexis;Finland;GenBank;-;HM872188
Lepidoptera;Nymphalidae;Apatura;Apatura iris;Finland;GenBank;-;HM872958
Lepidoptera;Nymphalidae;Apatura;Apatura ilia;Finland;GenBank;-;HM872960
Lepidoptera;Hesperiidae;Pyrgus;Pyrgus alveus;Finland;GenBank;-;HM873490
Lepidoptera;Hesperiidae;Hesperia;Hesperia comma;Finland;GenBank;-;HM873567
Lepidoptera;Nymphalidae;Hipparchia;Hipparchia semele;Finland;GenBank;-;HM873568
Lepidoptera;Nymphalidae;Maniola;Maniola jurtina;Finland;GenBank;-;HM873571
Lepidoptera;Nymphalidae;Coenonympha;Coenonympha pamphilus;Finland;GenBank;-;HM874610
Lepidoptera;Lycaenidae;Lycaena;Lycaena phlaeas;Finland;GenBank;-;HM874632
Lepidoptera;Pieridae;Pontia;Pieris edusa;Finland;GenBank;-;HM874820
Lepidoptera;Pieridae;Pontia;Pieris edusa;Finland;GenBank;-;HM874821
Lepidoptera ;Nymphalidae;Issoria;Issoria lathonia;Finland;GenBank;-;HM876030
Lepidoptera;Nymphalidae;Coenonympha;Coenonympha pamphilus;Finland;GenBank;-;HM876033
Lepidoptera;Nymphalidae;Melitaea;Melitaea didyma;Spain;GenBank;-;HM901283
Lepidoptera;Nymphalidae;Fabriciana;Argynnis niobe;Spain;GenBank;-;HM901284
Lepidoptera;Lycaenidae;Lycaena;Lycaena alciphron;Spain;GenBank;-;HM901285
Lepidoptera;Nymphalidae;Melanargia;Melanargia russiae;Spain;GenBank;-;HM901286
Lepidoptera;Nymphalidae;Brenthis;Brenthis hecate;Spain;GenBank;-;HM901287
Lepidoptera;Nymphalidae;Brenthis;Brenthis hecate;Spain;GenBank;-;HM901288
Lepidoptera ;Hesperiidae;Gegenes;Gegenes nostrodamus;Spain;GenBank;-;HM901311
Lepidoptera;Nymphalidae;Erebia;Erebia manto;Spain;GenBank;-;HM901318
Lepidoptera;Nymphalidae;Melanargia;Melanargia russiae;Spain;GenBank;-;HM901383
Lepidoptera;Nymphalidae;Coenonympha;Coenonympha pamphilus;Spain;GenBank;-;HM901569
Lepidoptera;Hesperiidae;Thymelicus;Thymelicus sylvestris;Spain;GenBank;-;HM901574
Lepidoptera;Nymphalidae;Argynnis;Argynnis pandora;Spain;GenBank;-;HM901577
Lepidoptera;Nymphalidae;Aglais;Aglais urticae;Spain;Bold;EZSPC769-10;HM901624
Lepidoptera ;Nymphalidae;Pararge;Pararge aegeria;Spain;GenBank;-;HM901648
Lepidoptera;Lycaenidae;Polyommatus;Polyommatus icarus;Spain;GenBank;-;HM901649
Lepidoptera;Lycaenidae;Lysandra;Polyommatus coridon;Spain;GenBank;-;HM901660
Lepidoptera;Lycaenidae;Lysandra;Polyommatus coridon;Spain;GenBank;-;HM901661
Lepidoptera;Nymphalidae;Boloria;Boloria titania;Romania;GenBank;-;HQ004108
Lepidoptera;Nymphalidae;Boloria;Boloria titania;Romania;GenBank;-;HQ004109
Lepidoptera;Nymphalidae;Boloria;Boloria titania;Romania;GenBank;-;HQ004110
Lepidoptera;Nymphalidae;Boloria;Boloria titania;Romania;GenBank;-;HQ004111
Lepidoptera;Nymphalidae;Erebia;Erebia manto;Romania;GenBank;-;HQ004405
Lepidoptera;Nymphalidae;Erebia;Erebia manto;Romania;GenBank;-;HQ004406
Lepidoptera;Nymphalidae;Erebia;Erebia manto;Romania;GenBank;-;HQ004407
Lepidoptera;Nymphalidae;Erebia;Erebia manto;Romania;GenBank;-;HQ004408
Lepidoptera;Nymphalidae;Erebia;Erebia manto;Romania;GenBank;-;HQ004409
Lepidoptera;Nymphalidae;Erebia;Erebia manto;Romania;GenBank;-;HQ004410
Lepidoptera;Nymphalidae;Erebia;Erebia manto;Romania;GenBank;-;HQ004411
Lepidoptera;Nymphalidae;Erebia;Erebia manto;Romania;GenBank;-;HQ004412
Lepidoptera;Nymphalidae;Erebia;Erebia manto;Romania;GenBank;-;HQ004413
Lepidoptera;Nymphalidae;Erebia;Erebia pharte;Romania;GenBank;-;HQ004435
Lepidoptera;Nymphalidae;Erebia;Erebia pharte;Romania;GenBank;-;HQ004436
Lepidoptera;Nymphalidae;Nymphalis;Nymphalis polychloros;Romania;GenBank;-;HQ004866
Lepidoptera;Nymphalidae;Boloria;Boloria napaea;Finland;GenBank;-;HQ570285
Lepidoptera;Nymphalidae;Boloria;Boloria thore;Finland;GenBank;-;HQ570286
Lepidoptera;Nymphalidae;Erebia;Erebia medusa;Finland;Bold;LEFIF127-10;HQ570388
Lepidoptera;Nymphalidae;Erebia;Erebia medusa;Finland;Bold;LEFIF128-10;HQ570389
Lepidoptera;Lycaenidae;Phengaris;Maculinea alcon;Denmark;GenBank;-;HQ918119
Lepidoptera;Lycaenidae;Polyommatus;Polyommatus icarus;Turkey;GenBank;-;HQ989581
Lepidoptera;Nymphalidae;Fabriciana;Argynnis niobe;Finland;GenBank;-;JF853516
Lepidoptera;Nymphalidae;Fabriciana;Argynnis niobe;Finland;GenBank;-;JF853517
Lepidoptera;Lycaenidae;Lycaena;Lycaena dispar;Finland;GenBank;-;JF853545
Lepidoptera;Hesperiidae;Hesperia;Hesperia comma;Finland;GenBank;-;JF853614
Lepidoptera;Hesperiidae;Hesperia;Hesperia comma;Finland;GenBank;-;JF853615
Lepidoptera;Pieridae;Pieris;Pieris brassicae;Finland;GenBank;-;JF853625
Lepidoptera;Pieridae;Pontia;Pieris edusa;Finland;GenBank;-;JF853630

Lepidoptera;Lycaenidae;Celastrina;Celastrina argiolus;Finland;GenBank;-;JF853633
Lepidoptera;Lycaenidae;Plebejus;Plebejus argus;Finland;GenBank;-;JF853638
Lepidoptera;Lycaenidae;Cyaniris;Cyaniris semiargus;Finland;GenBank;-;JF853640
Lepidoptera ;Lycaenidae;Thecla;Thecla betulae;Finland;GenBank;-;JF853643
Lepidoptera;Nymphalidae;Nymphalis;Nymphalis io;Finland;GenBank;-;JF853646
Lepidoptera;Nymphalidae;Apatura;Apatura ilia;Finland;GenBank;-;JF853652
Lepidoptera;Nymphalidae;Fabriciana;Argynnis adippe;Finland;GenBank;-;JF853656
Lepidoptera;Nymphalidae;Fabriciana;Argynnis niobe;Finland;GenBank;-;JF853657
Lepidoptera ;Nymphalidae;Issoria;Issoria lathonia;Finland;GenBank;-;JF853658
Lepidoptera;Nymphalidae;Boloria;Boloria selene;Finland;GenBank;-;JF853661
Lepidoptera;Nymphalidae;Hipparchia;Hipparchia semele;Finland;GenBank;-;JF853667
Lepidoptera;Nymphalidae;Erebia;Erebia medusa;Finland;Bold;LEFIJ553-10;JF853668
Lepidoptera;Nymphalidae;Coenonympha;Coenonympha pamphilus;Finland;GenBank;-;JF853673
Lepidoptera ;Nymphalidae;Pararge;Pararge aegeria;Finland;GenBank;-;JF853675
Lepidoptera ;Nymphalidae;Pararge;Pararge aegeria;Finland;GenBank;-;JF853676
Lepidoptera;Papilionidae;Parnassius;Parnassius mnemosyne;Finland;GenBank;-;JF853828
Lepidoptera;Papilionidae;Parnassius;Parnassius mnemosyne;Finland;GenBank;-;JF853829
Lepidoptera;Nymphalidae;Hyponephele;Hyponephele lycaon;Russia;Bold;LEFIL234-10;JF854545
Lepidoptera;Lycaenidae;Phengaris;Maculinea arion;Estonia;GenBank;-;JF854554
Lepidoptera;Lycaenidae;Polyommatus;Polyommatus icarus;France;Bold;GBMIN32543-13;JN084701
Lepidoptera;Lycaenidae;Polyommatus;Polyommatus icarus;Italy;Bold;GBMIN32564-13;JN084706
Lepidoptera;Nymphalidae;Boloria;Boloria napaea;Finland;GenBank;-;JN272537
Lepidoptera;Hesperiidae;Pyrgus;Pyrgus alveus;Finland;GenBank;-;JN277847
Lepidoptera;Nymphalidae;Hipparchia;Hipparchia statilinus;Spain;GenBank;-;JN278890
Lepidoptera;Nymphalidae;Hipparchia;Hipparchia statilinus;Spain;GenBank;-;JN278891
Lepidoptera;Lycaenidae;Lycaena;Lycaena dispar;Austria;Bold;GWOSK784-11;JN280795
Lepidoptera;Lycaenidae;Lycaena;Lycaena dispar;Finland;GenBank;-;JN280797
Lepidoptera;Lycaenidae;Satyrium;Satyrium pruni;Russia;GenBank;-;JX112880
Lepidoptera;Lycaenidae;Satyrium;Satyrium spini;Russia;GenBank;-;JX112881
Lepidoptera;Lycaenidae;Satyrium;Satyrium w-album;Russia;GenBank;-;JX112882
Lepidoptera;Lycaenidae;Satyrium;Satyrium acaciae;Russia;GenBank;-;JX112886
Lepidoptera;Lycaenidae;Aricia;Aricia agestis;Spain;Bold;GBMIN16215-13;JX678063
Lepidoptera;Lycaenidae;Aricia;Aricia agestis;Greece;Bold;GBMIN16183-13;JX678127
Lepidoptera;Lycaenidae;Aricia;Aricia agestis;Slovenia;Bold;GBMIN16319-13;JX678132
Lepidoptera;Pieridae;Pieris;Pieris brassicae;Austria;GenBank;-;KC462795
Lepidoptera;Pieridae;Pontia;Pieris edusa;Austria;GenBank;-;KC462865
Lepidoptera;Pieridae;Pontia;Pieris edusa;Austria;GenBank;-;KC462866
Lepidoptera;Pieridae;Leptidea;Leptidea juvernica;Ireland;Bold;GBGLP241-13;KC865992
Lepidoptera;Pieridae;Leptidea;Leptidea sinapis;Hungary;Bold;GBGLP202-13;KC866031
Lepidoptera;Pieridae;Leptidea;Leptidea sinapis;Croatia;Bold;GBGLP187-13;KC866046
Lepidoptera;Pieridae;Leptidea;Leptidea sinapis;Italy;Bold;GBGLP182-13;KC866051
Lepidoptera;Pieridae;Leptidea;Leptidea sinapis;Croatia;Bold;GBGLP181-13;KC866052
Lepidoptera;Pieridae;Leptidea;Leptidea sinapis;Hungary;Bold;GBGLP134-13;KC866099
Lepidoptera;Pieridae;Leptidea;Leptidea sinapis;Ireland;Bold;GBGLP096-13;KC866137
Lepidoptera;Nymphalidae;Minois;Minois dryas;Russia;GenBank;-;KF723544
Lepidoptera;Nymphalidae;Fabriciana;Argynnis niobe;Russia;GenBank;-;KF723548
Lepidoptera;Nymphalidae;Brenthis;Brenthis ino;Russia;GenBank;-;KF723550
Lepidoptera ;Lycaenidae;Lampides;Lampides boeticus;Russia;GenBank;-;KF860853
Lepidoptera;Nymphalidae;Melitaea;Melitaea trivia;Russia;GenBank;-;KJ638698
Lepidoptera;Nymphalidae;Maniola;Maniola jurtina;France;GenBank;-;KM020807
Lepidoptera;Nymphalidae;Maniola;Maniola jurtina;Italy;GenBank;-;KM020809
Lepidoptera;Nymphalidae;Maniola;Maniola jurtina;Italy;GenBank;-;KM020818
Lepidoptera;Nymphalidae;Maniola;Maniola jurtina;Italy;GenBank;-;KM020820
Lepidoptera;Nymphalidae;Maniola;Maniola jurtina;Italy;GenBank;-;KM020822
Lepidoptera;Nymphalidae;Maniola;Maniola jurtina;France;GenBank;-;KM020823
Lepidoptera;Nymphalidae;Maniola;Maniola jurtina;Italy;GenBank;-;KM020826
Lepidoptera;Nymphalidae;Maniola;Maniola jurtina;Malta;GenBank;-;KM020828
Lepidoptera;Nymphalidae;Maniola;Maniola jurtina;Malta;GenBank;-;KM020835
Lepidoptera;Nymphalidae;Maniola;Maniola jurtina;Italy;GenBank;-;KM020838
Lepidoptera;Nymphalidae;Maniola;Maniola jurtina;France;GenBank;-;KM020842
Lepidoptera;Nymphalidae;Maniola;Maniola jurtina;Italy;GenBank;-;KM020843
Lepidoptera;Nymphalidae;Maniola;Maniola jurtina;Italy;GenBank;-;KM020844
Lepidoptera;Nymphalidae;Maniola;Maniola jurtina;Italy;GenBank;-;KM020846
Lepidoptera;Nymphalidae;Maniola;Maniola jurtina;Italy;GenBank;-;KM020851
Lepidoptera;Nymphalidae;Maniola;Maniola jurtina;Italy;GenBank;-;KM020852
Lepidoptera;Nymphalidae;Maniola;Maniola jurtina;Italy;GenBank;-;KM020861
Lepidoptera;Nymphalidae;Maniola;Maniola jurtina;Italy;GenBank;-;KM020868
Lepidoptera;Nymphalidae;Maniola;Maniola jurtina;Italy;GenBank;-;KM020870
Lepidoptera;Nymphalidae;Maniola;Maniola jurtina;Italy;GenBank;-;KM020875
Lepidoptera;Nymphalidae;Maniola;Maniola jurtina;Malta;GenBank;-;KM020877
Lepidoptera;Nymphalidae;Maniola;Maniola jurtina;Malta;GenBank;-;KM020879
Lepidoptera;Nymphalidae;Maniola;Maniola jurtina;Italy;GenBank;-;KM020880
Lepidoptera;Nymphalidae;Maniola;Maniola jurtina;Italy;GenBank;-;KM020882
Lepidoptera;Nymphalidae;Maniola;Maniola jurtina;France;Bold;GBLN5018-14;KM033851
Lepidoptera;Nymphalidae;Maniola;Maniola jurtina;Italy;Bold;GBLN5065-14;KM033898
Lepidoptera;Nymphalidae;Maniola;Maniola jurtina;Italy;Bold;GBLN5066-14;KM033899
Lepidoptera;Nymphalidae;Maniola;Maniola jurtina;Italy;Bold;GBLN5072-14;KM033905
Lepidoptera;Nymphalidae;Maniola;Maniola jurtina;Italy;Bold;GBLN5073-14;KM033906

Lepidoptera;Nymphalidae;Maniola;Maniola jurtina;Italy;Bold;GBLN5076-14;KM033909
Lepidoptera;Nymphalidae;Maniola;Maniola jurtina;Italy;Bold;GBLN5077-14;KM033910
Lepidoptera;Nymphalidae;Maniola;Maniola jurtina;Italy;Bold;GBLN5078-14;KM033911
Lepidoptera;Nymphalidae;Maniola;Maniola jurtina;France;Bold;GBLN5087-14;KM033920
Lepidoptera;Nymphalidae;Maniola;Maniola jurtina;France;Bold;GBLN5088-14;KM033921
Lepidoptera;Nymphalidae;Maniola;Maniola jurtina;Italy;Bold;GBLN5103-14;KM033936
Lepidoptera;Nymphalidae;Maniola;Maniola jurtina;Italy;Bold;GBLN5104-14;KM033937
Lepidoptera;Nymphalidae;Maniola;Maniola jurtina;Italy;Bold;GBLN5105-14;KM033938
Lepidoptera;Nymphalidae;Maniola;Maniola jurtina;Italy;Bold;GBLN5106-14;KM033939
Lepidoptera;Lycaenidae;Aricia;Aricia agestis;Spain;Bold;GBGL18167-15;KM459029
Lepidoptera;Lycaenidae;Aricia;Aricia agestis;France;Bold;GBGL18190-15;KM459052
Lepidoptera;Lycaenidae;Aricia;Aricia agestis;France;Bold;GBGL18191-15;KM459053
Lepidoptera;Lycaenidae;Aricia;Aricia agestis;Italy;Bold;GBGL18199-15;KM459061
Lepidoptera;Lycaenidae;Aricia;Aricia agestis;Italy;Bold;GBGL18200-15;KM459062
Lepidoptera;Lycaenidae;Aricia;Aricia agestis;Italy;Bold;GBGL18201-15;KM459063
Lepidoptera;Lycaenidae;Aricia;Aricia agestis;Italy;Bold;GBGL18202-15;KM459064
Lepidoptera;Lycaenidae;Aricia;Aricia agestis;Italy;Bold;GBGL18203-15;KM459065
Lepidoptera;Lycaenidae;Aricia;Aricia agestis;Italy;Bold;GBGL18207-15;KM459069
Lepidoptera;Lycaenidae;Aricia;Aricia agestis;Italy;Bold;GBGL18208-15;KM459070
Lepidoptera;Lycaenidae;Aricia;Aricia agestis;Italy;Bold;GBGL18209-15;KM459071
Lepidoptera;Lycaenidae;Aricia;Aricia agestis;Italy;Bold;GBGL18210-15;KM459072
Lepidoptera;Lycaenidae;Aricia;Aricia agestis;Italy;Bold;GBGL18211-15;KM459073
Lepidoptera;Lycaenidae;Aricia;Aricia agestis;France;Bold;GBGL18222-15;KM459084
Lepidoptera;Lycaenidae;Aricia;Aricia agestis;Italy;Bold;GBGL18238-15;KM459100
Lepidoptera;Lycaenidae;Aricia;Aricia cramera;Italy;Bold;GBGL18265-15;KM459127
Lepidoptera;Lycaenidae;Aricia;Aricia cramera;Italy;Bold;GBGL18266-15;KM459128
Lepidoptera;Lycaenidae;Aricia;Aricia cramera;Italy;Bold;GBGL18273-15;KM459135
Lepidoptera;Lycaenidae;Polyommatus;Polyommatus icarus;Spain;Bold;GBGL19935-15;KM459184
Lepidoptera;Lycaenidae;Polyommatus;Polyommatus icarus;Spain;Bold;GBGL19937-15;KM459186
Lepidoptera;Lycaenidae;Polyommatus;Polyommatus icarus;Italy;Bold;GBGL19963-15;KM459214
Lepidoptera;Lycaenidae;Polyommatus;Polyommatus icarus;Italy;Bold;GBGL19964-15;KM459215
Lepidoptera;Lycaenidae;Polyommatus;Polyommatus icarus;Italy;Bold;GBGL19965-15;KM459216
Lepidoptera;Lycaenidae;Polyommatus;Polyommatus icarus;Malta;Bold;GBGL19976-15;KM459234
Lepidoptera;Lycaenidae;Polyommatus;Polyommatus icarus;Malta;Bold;GBGL19977-15;KM459235
Lepidoptera;Lycaenidae;Polyommatus;Polyommatus icarus;Malta;Bold;GBGL19978-15;KM459236
Lepidoptera;Lycaenidae;Polyommatus;Polyommatus icarus;Malta;Bold;GBGL19979-15;KM459237
Lepidoptera;Lycaenidae;Polyommatus;Polyommatus icarus;Malta;Bold;GBGL19980-15;KM459238
Lepidoptera;Lycaenidae;Polyommatus;Polyommatus icarus;Malta;Bold;GBGL19981-15;KM459239
Lepidoptera;Lycaenidae;Polyommatus;Polyommatus icarus;Italy;Bold;GBGL19987-15;KM459245
Lepidoptera;Lycaenidae;Polyommatus;Polyommatus icarus;Italy;Bold;GBGL20010-15;KM459268
Lepidoptera;Lycaenidae;Polyommatus;Polyommatus icarus;Italy;Bold;GBGL20011-15;KM459269
Lepidoptera;Lycaenidae;Polyommatus;Polyommatus icarus;Italy;Bold;GBGL20012-15;KM459270
Lepidoptera;Lycaenidae;Polyommatus;Polyommatus icarus;Italy;Bold;GBGL20013-15;KM459271
Lepidoptera;Lycaenidae;Polyommatus;Polyommatus icarus;Italy;Bold;GBGL20014-15;KM459272
Lepidoptera;Lycaenidae;Polyommatus;Polyommatus icarus;Italy;Bold;GBGL20015-15;KM459273
Lepidoptera;Lycaenidae;Polyommatus;Polyommatus icarus;Italy;Bold;GBGL20016-15;KM459274
Lepidoptera;Lycaenidae;Polyommatus;Polyommatus icarus;Italy;Bold;GBGL20017-15;KM459275
Lepidoptera;Lycaenidae;Polyommatus;Polyommatus icarus;Italy;Bold;GBGL20018-15;KM459276
Lepidoptera;Lycaenidae;Polyommatus;Polyommatus icarus;Italy;Bold;GBGL20019-15;KM459277
Lepidoptera;Lycaenidae;Polyommatus;Polyommatus icarus;Italy;Bold;GBGL20020-15;KM459278
Lepidoptera;Lycaenidae;Polyommatus;Polyommatus icarus;Italy;Bold;GBGL20021-15;KM459279
Lepidoptera;Lycaenidae;Polyommatus;Polyommatus icarus;Italy;Bold;GBGL20022-15;KM459280
Lepidoptera;Lycaenidae;Polyommatus;Polyommatus icarus;Italy;Bold;GBGL20023-15;KM459281
Lepidoptera;Lycaenidae;Polyommatus;Polyommatus icarus;Italy;Bold;GBGL20040-15;KM459303
Lepidoptera;Lycaenidae;Polyommatus;Polyommatus icarus;Italy;Bold;GBGL20041-15;KM459304
Lepidoptera;Lycaenidae;Polyommatus;Polyommatus icarus;Italy;Bold;GBGL20052-15;KM459315
Lepidoptera;Lycaenidae;Polyommatus;Polyommatus icarus;Italy;Bold;GBGL20053-15;KM459316
Lepidoptera;Lycaenidae;Polyommatus;Polyommatus icarus;Italy;Bold;GBGL20058-15;KM459321
Lepidoptera;Lycaenidae;Polyommatus;Polyommatus icarus;Italy;Bold;GBGL20059-15;KM459322
Lepidoptera;Lycaenidae;Polyommatus;Polyommatus icarus;Italy;Bold;EULEP095-14;KM459357
Lepidoptera;Lycaenidae;Polyommatus;Polyommatus icarus;Italy;Bold;GBGL20092-15;KM459375
Lepidoptera;Lycaenidae;Polyommatus;Polyommatus icarus;France;Bold;GBGL20104-15;KM459388
Lepidoptera;Lycaenidae;Polyommatus;Polyommatus icarus;France;Bold;GBGL20105-15;KM459389
Lepidoptera;Lycaenidae;Polyommatus;Polyommatus icarus;Italy;Bold;GBGL20117-15;KM459401
Lepidoptera;Lycaenidae;Polyommatus;Polyommatus icarus;Italy;Bold;GBGL20118-15;KM459402
Lepidoptera;Lycaenidae;Polyommatus;Polyommatus icarus;Italy;Bold;GBGL20119-15;KM459403
Lepidoptera;Lycaenidae;Polyommatus;Polyommatus icarus;Italy;Bold;GBGL20120-15;KM459404
Lepidoptera;Lycaenidae;Polyommatus;Polyommatus icarus;Italy;Bold;GBGL20121-15;KM459405
Lepidoptera;Lycaenidae;Polyommatus;Polyommatus icarus;Italy;Bold;GBGL20129-15;KM459415
Lepidoptera;Lycaenidae;Polyommatus;Polyommatus icarus;Italy;Bold;GBGL20130-15;KM459416
Lepidoptera;Lycaenidae;Polyommatus;Polyommatus icarus;Italy;Bold;GBGL20137-15;KM459423
Lepidoptera;Lycaenidae;Polyommatus;Polyommatus icarus;France;Bold;GBGL20140-15;KM459426
Lepidoptera;Lycaenidae;Polyommatus;Polyommatus icarus;France;Bold;GBGL20142-15;KM459428
Lepidoptera;Lycaenidae;Polyommatus;Polyommatus icarus;Italy;Bold;GBGL20152-15;KM459439
Lepidoptera;Lycaenidae;Aricia;Aricia agestis;Italy;GenBank;-;KM517842
Lepidoptera;Lycaenidae;Polyommatus;Polyommatus icarus;Finland;GenBank;-;KM572032
Lepidoptera;Lycaenidae;Cyaniris;Cyaniris semiargus;Finland;GenBank;-;KM573316
Lepidoptera;Nymphalidae;Boloria;Boloria selene;Finland;GenBank;-;KM573567

Lepidoptera;Lycaenidae;Lysandra;Polyommatus coridon;Austria;GenBank;-;KM972617
Lepidoptera;Lycaenidae;Aricia;Aricia agestis;Italy;Bold;GBGL18243-15;KP052710
Lepidoptera;Pieridae;Pieris;Pieris rapae;Spain;GenBank;-;KP870276
Lepidoptera;Lycaenidae;Phengaris;Maculinea alcon;Spain;GenBank;-;KP870289
Lepidoptera;Nymphalidae;Vanessa;Vanessa atalanta;Spain;GenBank;-;KP870311
Lepidoptera;Nymphalidae;Minois;Minois dryas;Spain;GenBank;-;KP870317
Lepidoptera;Nymphalidae;Minois;Minois dryas;Spain;GenBank;-;KP870368
Lepidoptera;Pieridae;Pieris;Pieris brassicae;Portugal;GenBank;-;KP870372
Lepidoptera;Nymphalidae;Arethusana;Arethusana arethusa;Spain;GenBank;-;KP870380
Lepidoptera;Nymphalidae;Nymphalis;Nymphalis antiopa;Spain;GenBank;-;KP870403
Lepidoptera;Nymphalidae;Erebia;Erebia manto;Spain;GenBank;-;KP870407
Lepidoptera;Nymphalidae;Minois;Minois dryas;Spain;GenBank;-;KP870420
Lepidoptera ;Lycaenidae;Thecla;Thecla betulae;Spain;GenBank;-;KP870422
Lepidoptera;Lycaenidae;Phengaris;Maculinea alcon;Spain;GenBank;-;KP870427
Lepidoptera;Nymphalidae;Vanessa;Vanessa cardui;Spain;GenBank;-;KP870469
Lepidoptera;Lycaenidae;Phengaris;Maculinea alcon;Spain;GenBank;-;KP870487
Lepidoptera;Nymphalidae;Lasiommata;Lasiommata megera;Spain;GenBank;-;KP870492
Lepidoptera;Lycaenidae;Phengaris;Maculinea alcon;Spain;GenBank;-;KP870501
Lepidoptera ;Lycaenidae;Thecla;Thecla betulae;Spain;GenBank;-;KP870561
Lepidoptera;Pieridae;Pieris;Pieris brassicae;Portugal;GenBank;-;KP870572
Lepidoptera;Nymphalidae;Hipparchia;Hipparchia semele;Sweden;GenBank;-;KP870578
Lepidoptera;Nymphalidae;Erebia;Erebia manto;Spain;GenBank;-;KP870586
Lepidoptera;Nymphalidae;Hipparchia;Hipparchia semele;Sweden;GenBank;-;KP870629
Lepidoptera;Lycaenidae;Phengaris;Maculinea alcon;Spain;GenBank;-;KP870639
Lepidoptera;Nymphalidae;Nymphalis;Nymphalis antiopa;Spain;GenBank;-;KP870651
Lepidoptera;Nymphalidae;Minois;Minois dryas;Spain;GenBank;-;KP870743
Lepidoptera;Nymphalidae;Nymphalis;Nymphalis antiopa;Spain;GenBank;-;KP870835
Lepidoptera;Nymphalidae;Nymphalis;Nymphalis io;Spain;GenBank;-;KP870896
Lepidoptera;Lycaenidae;Phengaris;Maculinea alcon;Spain;GenBank;-;KP870946
Lepidoptera;Nymphalidae;Vanessa;Vanessa cardui;Spain;GenBank;-;KP870949
Lepidoptera;Lycaenidae;Phengaris;Maculinea alcon;Spain;GenBank;-;KP870963
Lepidoptera;Nymphalidae;Minois;Minois dryas;Spain;GenBank;-;KP870992
Lepidoptera;Nymphalidae;Minois;Minois dryas;Spain;GenBank;-;KP870995
Lepidoptera;Nymphalidae;Minois;Minois dryas;Spain;GenBank;-;KP871008
Lepidoptera;Nymphalidae;Vanessa;Vanessa cardui;Spain;GenBank;-;KP871009
Lepidoptera;Nymphalidae;Vanessa;Vanessa cardui;Spain;GenBank;-;KP871040
Lepidoptera ;Hesperiidae;Carcharodus;Carcharodus alceae;Spain;GenBank;-;KP871152
Lepidoptera;Lycaenidae;Phengaris;Maculinea arion;Latvia;GenBank;-;KT782549
Lepidoptera;Nymphalidae;Melitaea;Melitaea phoebe;Austria;GenBank;-;KT907186
Lepidoptera ;Hesperiidae;Spialia;Spialia sertorius;Italy;GenBank;-;KU905343
Lepidoptera ;Hesperiidae;Spialia;Spialia sertorius;France;GenBank;-;KU905484
Lepidoptera;Nymphalidae;Nymphalis;Nymphalis polychloros;Finland;GenBank;-;KX041438
Lepidoptera;Lycaenidae;Lycaena;Lycaena hippothoe;Croatia;GenBank;-;KX044480
Lepidoptera;Nymphalidae;Lasiommata;Lasiommata megera;Norway;GenBank;-;KX047683
Lepidoptera ;Pieridae;Gonepteryx;Gonepteryx rhamni;Norway;GenBank;-;KX047756
Lepidoptera;Lycaenidae;Lycaena;Lycaena phlaeas;Norway;GenBank;-;KX047828
Lepidoptera;Hesperiidae;Hesperia;Hesperia comma;Norway;GenBank;-;KX047906
Lepidoptera;Lycaenidae;Scolitantides;Scolitantides orion;Norway;GenBank;-;KX048470
Lepidoptera;Lycaenidae;Cupido;Cupido minimus;Norway;GenBank;-;KX048709
Lepidoptera;Lycaenidae;Scolitantides;Scolitantides orion;Norway;GenBank;-;KX048958
Lepidoptera;Lycaenidae;Polyommatus;Polyommatus icarus;Norway;GenBank;-;KX049307
Lepidoptera;Nymphalidae;Erebia;Erebia medusa;Norway;Bold;LON171-08;KX049579
Lepidoptera;Lycaenidae;Lycaena;Lycaena phlaeas;Norway;GenBank;-;KX049723
Lepidoptera;Nymphalidae;Coenonympha;Coenonympha pamphilus;Norway;GenBank;-;KX049735
Lepidoptera;Nymphalidae;Hyponephele;Hyponephele lycaon;Russia;Bold;LEFIL235-10;KX049854
Lepidoptera;Nymphalidae;Vanessa;Vanessa atalanta;Norway;GenBank;-;KX049889
Lepidoptera ;Lycaenidae;Thecla;Thecla betulae;Norway;GenBank;-;KX049957
Lepidoptera;Lycaenidae;Aricia;Aricia allous;Norway;GenBank;-;KX050013
Lepidoptera;Nymphalidae;Satyrus;Satyrus ferula;Russia;GenBank;-;KY000505
Lepidoptera;Nymphalidae;Satyrus;Satyrus ferula;Russia;GenBank;-;KY000510
Lepidoptera;Nymphalidae;Erebia;Erebia ligea;Russia;GenBank;-;LC229018
Lepidoptera;Nymphalidae;Erebia;Erebia euryale;Russia;GenBank;-;LC340478
Lepidoptera;Nymphalidae;Erebia;Erebia scipio;Italy;GenBank;-;LC340559
Lepidoptera;Nymphalidae;Erebia;Erebia medusa;Norway;GenBank;-;LC340564
Lepidoptera;Lycaenidae;Agriades;Albulina optilete;Denmark;GenBank;-;LT219473
Lepidoptera;Lycaenidae;Agriades;Albulina optilete;Denmark;GenBank;-;LT219474
Lepidoptera;Lycaenidae;Agriades;Albulina optilete;Denmark;GenBank;-;LT219475
Lepidoptera;Lycaenidae;Agriades;Albulina optilete;Denmark;GenBank;-;LT219477
Lepidoptera;Lycaenidae;Agriades;Albulina optilete;Denmark;GenBank;-;LT219478
Lepidoptera;Lycaenidae;Agriades;Albulina optilete;Denmark;GenBank;-;LT219479
Lepidoptera;Lycaenidae;Agriades;Albulina optilete;Denmark;GenBank;-;LT219480
Lepidoptera;Lycaenidae;Agriades;Albulina optilete;Denmark;GenBank;-;LT219481
Lepidoptera;Lycaenidae;Plebejus;Plebejus argus;Denmark;GenBank;-;LT219906
Lepidoptera;Lycaenidae;Plebejus;Plebejus argus;Denmark;GenBank;-;LT219907
Lepidoptera;Lycaenidae;Plebejus;Plebejus argus;Denmark;GenBank;-;LT219908
Lepidoptera;Lycaenidae;Plebejus;Plebejus argus;Denmark;GenBank;-;LT219909
Lepidoptera;Lycaenidae;Plebejus;Plebejus argus;Denmark;GenBank;-;LT219910
Lepidoptera;Lycaenidae;Plebejus;Plebejus argus;Denmark;GenBank;-;LT219911

Lepidoptera;Lycaenidae;Plebejus;Plebejus argus;Denmark;GenBank;-;LT219912
Lepidoptera;Nymphalidae;Melanargia;Melanargia russiae;Russia;GenBank;-;MG817334
Lepidoptera ;Nymphalidae;Pararge;Pararge aegeria;Italy;GenBank;-;MH089591
Lepidoptera ;Nymphalidae;Pararge;Pararge aegeria;Italy;GenBank;-;MH089592
Lepidoptera ;Nymphalidae;Pararge;Pararge aegeria;France;GenBank;-;MH089593
Lepidoptera ;Nymphalidae;Pararge;Pararge aegeria;France;GenBank;-;MH089599
Lepidoptera ;Nymphalidae;Pararge;Pararge aegeria;Italy;GenBank;-;MH089604
Lepidoptera ;Nymphalidae;Pararge;Pararge aegeria;Italy;GenBank;-;MH089605
Lepidoptera ;Nymphalidae;Pararge;Pararge aegeria;Malta;GenBank;-;MH089610
Lepidoptera ;Nymphalidae;Pararge;Pararge aegeria;Italy;GenBank;-;MH089612
Lepidoptera ;Nymphalidae;Pararge;Pararge aegeria;Italy;GenBank;-;MH089627
Lepidoptera ;Nymphalidae;Pararge;Pararge aegeria;Italy;GenBank;-;MH089630
Lepidoptera ;Nymphalidae;Pararge;Pararge aegeria;Italy;GenBank;-;MH089640
Lepidoptera ;Nymphalidae;Pararge;Pararge aegeria;Italy;GenBank;-;MH089643
Lepidoptera ;Nymphalidae;Pararge;Pararge aegeria;Italy;GenBank;-;MH089654
Lepidoptera ;Nymphalidae;Pararge;Pararge aegeria;Italy;GenBank;-;MH089657
Lepidoptera ;Nymphalidae;Pararge;Pararge aegeria;Italy;GenBank;-;MH089659
Lepidoptera ;Nymphalidae;Pararge;Pararge aegeria;Italy;GenBank;-;MH089664
Lepidoptera ;Nymphalidae;Pararge;Pararge aegeria;Italy;GenBank;-;MH089668
Lepidoptera ;Nymphalidae;Pararge;Pararge aegeria;Italy;GenBank;-;MH089672
Lepidoptera ;Nymphalidae;Pararge;Pararge aegeria;Malta;GenBank;-;MH089674
Lepidoptera ;Nymphalidae;Pararge;Pararge aegeria;Italy;GenBank;-;MH089675
Lepidoptera ;Nymphalidae;Pararge;Pararge aegeria;Italy;GenBank;-;MH089678
Lepidoptera ;Nymphalidae;Pararge;Pararge aegeria;Italy;GenBank;-;MH089689
Lepidoptera ;Nymphalidae;Pararge;Pararge aegeria;Italy;GenBank;-;MH089691
Lepidoptera ;Nymphalidae;Pararge;Pararge aegeria;Italy;GenBank;-;MH089693
Lepidoptera ;Nymphalidae;Pararge;Pararge aegeria;Italy;GenBank;-;MH089696
Lepidoptera ;Nymphalidae;Pararge;Pararge aegeria;Italy;GenBank;-;MH089697
Lepidoptera ;Nymphalidae;Pararge;Pararge aegeria;Italy;GenBank;-;MH089702
Lepidoptera ;Nymphalidae;Pararge;Pararge aegeria;Italy;GenBank;-;MH089706
Lepidoptera ;Nymphalidae;Pararge;Pararge aegeria;France;GenBank;-;MH089710
Lepidoptera ;Nymphalidae;Pararge;Pararge aegeria;Italy;GenBank;-;MH089716
Lepidoptera ;Nymphalidae;Pararge;Pararge aegeria;Italy;GenBank;-;MH089723
Lepidoptera ;Nymphalidae;Pararge;Pararge aegeria;United Kingdom;GenBank;-;MH089732
Lepidoptera ;Nymphalidae;Pararge;Pararge aegeria;Italy;GenBank;-;MH089738
Lepidoptera ;Nymphalidae;Pararge;Pararge aegeria;France;GenBank;-;MH089751
Lepidoptera ;Nymphalidae;Pararge;Pararge aegeria;Spain;GenBank;-;MH089753
Lepidoptera ;Nymphalidae;Pararge;Pararge aegeria;Spain;GenBank;-;MH089760
Lepidoptera ;Nymphalidae;Pararge;Pararge aegeria;Italy;GenBank;-;MH089761
Lepidoptera ;Nymphalidae;Pararge;Pararge aegeria;Italy;GenBank;-;MH089771
Lepidoptera ;Nymphalidae;Pararge;Pararge aegeria;Italy;GenBank;-;MH089775
Lepidoptera ;Nymphalidae;Pararge;Pararge aegeria;Italy;GenBank;-;MH089778
Lepidoptera ;Nymphalidae;Pararge;Pararge aegeria;Italy;GenBank;-;MH089779
Lepidoptera ;Nymphalidae;Pararge;Pararge aegeria;France;GenBank;-;MH089781
Lepidoptera ;Nymphalidae;Pararge;Pararge aegeria;Malta;GenBank;-;MH089784
Lepidoptera ;Nymphalidae;Pararge;Pararge aegeria;Italy;GenBank;-;MH089793
Lepidoptera ;Nymphalidae;Pararge;Pararge aegeria;Italy;GenBank;-;MH089798
Lepidoptera ;Nymphalidae;Pararge;Pararge aegeria;Malta;GenBank;-;MH089799
Lepidoptera ;Nymphalidae;Pararge;Pararge aegeria;Italy;GenBank;-;MH089802
Lepidoptera ;Nymphalidae;Pararge;Pararge aegeria;Italy;GenBank;-;MH089810
Lepidoptera ;Nymphalidae;Pararge;Pararge aegeria;Italy;GenBank;-;MH089813
Lepidoptera ;Nymphalidae;Pararge;Pararge aegeria;Italy;GenBank;-;MH089814
Lepidoptera ;Nymphalidae;Pararge;Pararge aegeria;Italy;GenBank;-;MH089815
Lepidoptera ;Nymphalidae;Pararge;Pararge aegeria;Italy;GenBank;-;MH089817
Lepidoptera ;Nymphalidae;Pararge;Pararge aegeria;Italy;GenBank;-;MH089831
Lepidoptera ;Nymphalidae;Pararge;Pararge aegeria;Italy;GenBank;-;MH089835
Lepidoptera ;Nymphalidae;Pararge;Pararge aegeria;Italy;GenBank;-;MH089836
Lepidoptera ;Nymphalidae;Pararge;Pararge aegeria;Italy;GenBank;-;MH089837
Lepidoptera;Nymphalidae;Melanargia;Melanargia galathea;France;GenBank;-;MH308277
Lepidoptera;Nymphalidae;Melanargia;Melanargia galathea;Italy;GenBank;-;MH308286
Lepidoptera;Nymphalidae;Melanargia;Melanargia galathea;Italy;GenBank;-;MH308327
Lepidoptera ;Lycaenidae;Leptotes;Leptotes pirithous;Italy;GenBank;-;MH418493
Lepidoptera;Nymphalidae;Lasiommata;Lasiommata paramegaera;Italy;GenBank;-;MH418496
Lepidoptera;Pieridae;Pontia;Pieris edusa;Italy;GenBank;-;MH418498
Lepidoptera;Lycaenidae;Callophrys;Callophrys rubi;Italy;GenBank;-;MH418507
Lepidoptera;Pieridae;Pieris;Pieris rapae;Italy;GenBank;-;MH418515
Lepidoptera ;Hesperiidae;Gegenes;Gegenes pumilio;Italy;GenBank;-;MH418516
Lepidoptera;Pieridae;Pontia;Pieris edusa;Italy;GenBank;-;MH418519
Lepidoptera;Nymphalidae;Lasiommata;Lasiommata megera;Italy;GenBank;-;MH418524
Lepidoptera;Nymphalidae;Charaxes;Charaxes jasius;Italy;GenBank;-;MH418528
Lepidoptera;Papilionidae;Papilio;Papilio machaon;Italy;GenBank;-;MH418538
Lepidoptera ;Lycaenidae;Lampides;Lampides boeticus;Italy;GenBank;-;MH418541
Lepidoptera ;Pieridae;Gonepteryx;Gonepteryx cleopatra;Italy;GenBank;-;MH418547
Lepidoptera;Nymphalidae;Vanessa;Vanessa atalanta;Italy;GenBank;-;MH418554
Lepidoptera;Nymphalidae;Pyronia;Pyronia cecilia;Italy;Bold;WMB1948-13;MH418557
Lepidoptera ;Hesperiidae;Carcharodus;Carcharodus alceae;France;GenBank;-;MH418558
Lepidoptera ;Lycaenidae;Lampides;Lampides boeticus;France;GenBank;-;MH418561
Lepidoptera;Nymphalidae;Hipparchia;Hipparchia aristaeus;Italy;GenBank;-;MH418562

Lepidoptera;Nymphalidae;Lasiommata;Lasiommata megera;Italy;GenBank;-;MH418563
Lepidoptera ;Lycaenidae;Leptotes;Leptotes pirithous;Italy;GenBank;-;MH418565
Lepidoptera;Nymphalidae;Melitaea;Melitaea cinxia;Italy;GenBank;-;MH418573
Lepidoptera;Pieridae;Pieris;Pieris rapae;Italy;GenBank;-;MH418576
Lepidoptera;Lycaenidae;Lycaena;Lycaena phlaeas;Italy;GenBank;-;MH418583
Lepidoptera;Nymphalidae;Vanessa;Vanessa atalanta;Italy;GenBank;-;MH418592
Lepidoptera;Nymphalidae;Lasiommata;Lasiommata megera;Italy;GenBank;-;MH418594
Lepidoptera;Papilionidae;Papilio;Papilio machaon;Italy;GenBank;-;MH418596
Lepidoptera;Nymphalidae;Lasiommata;Lasiommata megera;Malta;GenBank;-;MH418597
Lepidoptera;Nymphalidae;Lasiommata;Lasiommata paramegaera;Italy;GenBank;-;MH418604
Lepidoptera;Nymphalidae;Lasiommata;Lasiommata paramegaera;Italy;GenBank;-;MH418606
Lepidoptera;Lycaenidae;Celastrina;Celastrina argiolus;Italy;GenBank;-;MH418621
Lepidoptera;Pieridae;Pontia;Pieris edusa;Italy;GenBank;-;MH418623
Lepidoptera;Papilionidae;Papilio;Papilio machaon;Italy;GenBank;-;MH418625
Lepidoptera;Papilionidae;Papilio;Papilio machaon;Italy;GenBank;-;MH418642
Lepidoptera;Pieridae;Pontia;Pieris edusa;Italy;GenBank;-;MH418645
Lepidoptera;Pieridae;Pieris;Pieris brassicae;Italy;GenBank;-;MH418653
Lepidoptera;Papilionidae;Papilio;Papilio machaon;Italy;GenBank;-;MH418659
Lepidoptera ;Pieridae;Gonepteryx;Gonepteryx cleopatra;Italy;GenBank;-;MH418662
Lepidoptera;Nymphalidae;Pyronia;Pyronia cecilia;France;Bold;WMB1526-13;MH418665
Lepidoptera;Lycaenidae;Celastrina;Celastrina argiolus;Italy;GenBank;-;MH418670
Lepidoptera;Nymphalidae;Pyronia;Pyronia tithonus;Italy;Bold;WMB063-11;MH418671
Lepidoptera;Nymphalidae;Charaxes;Charaxes jasius;Italy;GenBank;-;MH418675
Lepidoptera;Pieridae;Pieris;Pieris rapae;Italy;GenBank;-;MH418677
Lepidoptera;Nymphalidae;Vanessa;Vanessa cardui;Italy;GenBank;-;MH418681
Lepidoptera;Lycaenidae;Celastrina;Celastrina argiolus;Malta;GenBank;-;MH418684
Lepidoptera ;Lycaenidae;Lampides;Lampides boeticus;Malta;GenBank;-;MH418688
Lepidoptera;Nymphalidae;Vanessa;Vanessa atalanta;Malta;GenBank;-;MH418690
Lepidoptera;Lycaenidae;Lycaena;Lycaena phlaeas;Italy;GenBank;-;MH418696
Lepidoptera;Nymphalidae;Lasiommata;Lasiommata megera;Italy;GenBank;-;MH418697
Lepidoptera;Nymphalidae;Limenitis;Limenitis reducta;Italy;GenBank;-;MH418707
Lepidoptera ;Lycaenidae;Leptotes;Leptotes pirithous;Italy;GenBank;-;MH418711
Lepidoptera;Pieridae;Pieris;Pieris mannii;Italy;GenBank;-;MH418720
Lepidoptera;Papilionidae;Papilio;Papilio machaon;Italy;GenBank;-;MH418722
Lepidoptera;Pieridae;Pieris;Pieris brassicae;Italy;GenBank;-;MH418728
Lepidoptera;Pieridae;Pieris;Pieris rapae;Italy;GenBank;-;MH418731
Lepidoptera;Pieridae;Euchloe;Euchloe ausonia;Italy;GenBank;-;MH418741
Lepidoptera;Nymphalidae;Limenitis;Limenitis reducta;France;GenBank;-;MH418744
Lepidoptera;Pieridae;Pieris;Pieris rapae;Italy;GenBank;-;MH418751
Lepidoptera ;Lycaenidae;Lampides;Lampides boeticus;Italy;GenBank;-;MH418753
Lepidoptera;Nymphalidae;Pyronia;Pyronia cecilia;Italy;Bold;WMB1984-13;MH418758
Lepidoptera;Nymphalidae;Lasiommata;Lasiommata megera;Malta;GenBank;-;MH418759
Lepidoptera;Lycaenidae;Celastrina;Celastrina argiolus;Italy;GenBank;-;MH418762
Lepidoptera ;Lycaenidae;Leptotes;Leptotes pirithous;Italy;GenBank;-;MH418765
Lepidoptera;Lycaenidae;Lycaena;Lycaena phlaeas;Italy;GenBank;-;MH418768
Lepidoptera;Nymphalidae;Pyronia;Pyronia cecilia;Italy;Bold;WMB1808-13;MH418771
Lepidoptera;Nymphalidae;Coenonympha;Coenonympha corinna;France;GenBank;-;MH418782
Lepidoptera;Pieridae;Pieris;Pieris brassicae;Italy;GenBank;-;MH418791
Lepidoptera;Nymphalidae;Lasiommata;Lasiommata megera;Italy;GenBank;-;MH418792
Lepidoptera;Nymphalidae;Lasiommata;Lasiommata megera;Italy;GenBank;-;MH418797
Lepidoptera ;Lycaenidae;Lampides;Lampides boeticus;Malta;GenBank;-;MH418805
Lepidoptera;Lycaenidae;Glaucopsyche;Glaucopsyche alexis;France;GenBank;-;MH418811
Lepidoptera;Nymphalidae;Lasiommata;Lasiommata megera;Italy;GenBank;-;MH418813
Lepidoptera ;Hesperiidae;Gegenes;Gegenes pumilio;Italy;GenBank;-;MH418819
Lepidoptera;Pieridae;Pieris;Pieris rapae;Italy;GenBank;-;MH418828
Lepidoptera;Pieridae;Pontia;Pieris edusa;Italy;GenBank;-;MH418838
Lepidoptera;Nymphalidae;Lasiommata;Lasiommata paramegaera;Italy;GenBank;-;MH418841
Lepidoptera;Pieridae;Pieris;Pieris rapae;Italy;GenBank;-;MH418842
Lepidoptera;Nymphalidae;Lasiommata;Lasiommata megera;Italy;GenBank;-;MH418844
Lepidoptera;Pieridae;Pontia;Pieris edusa;Italy;GenBank;-;MH418849
Lepidoptera ;Lycaenidae;Lampides;Lampides boeticus;Malta;GenBank;-;MH418850
Lepidoptera;Pieridae;Pieris;Pieris rapae;Italy;GenBank;-;MH418860
Lepidoptera;Nymphalidae;Vanessa;Vanessa atalanta;Italy;GenBank;-;MH418863
Lepidoptera;Pieridae;Pieris;Pieris rapae;France;GenBank;-;MH418874
Lepidoptera;Lycaenidae;Celastrina;Celastrina argiolus;Italy;GenBank;-;MH418875
Lepidoptera ;Pieridae;Gonepteryx;Gonepteryx cleopatra;Italy;GenBank;-;MH418879
Lepidoptera;Pieridae;Pieris;Pieris rapae;Italy;GenBank;-;MH418889
Lepidoptera;Lycaenidae;Lycaena;Lycaena phlaeas;Italy;GenBank;-;MH418898
Lepidoptera;Nymphalidae;Pyronia;Pyronia cecilia;France;Bold;WMB2638-13;MH418906
Lepidoptera ;Lycaenidae;Leptotes;Leptotes pirithous;Italy;GenBank;-;MH418910
Lepidoptera;Lycaenidae;Lycaena;Lycaena phlaeas;Italy;GenBank;-;MH418913
Lepidoptera;Nymphalidae;Charaxes;Charaxes jasius;Italy;GenBank;-;MH418915
Lepidoptera;Papilionidae;Papilio;Papilio machaon;Italy;GenBank;-;MH418929
Lepidoptera ;Hesperiidae;Carcharodus;Carcharodus alceae;Italy;GenBank;-;MH418938
Lepidoptera;Papilionidae;Papilio;Papilio machaon;Italy;GenBank;-;MH418943
Lepidoptera;Pieridae;Pieris;Pieris rapae;Italy;GenBank;-;MH418965
Lepidoptera;Lycaenidae;Callophrys;Callophrys rubi;Italy;GenBank;-;MH418972
Lepidoptera;Nymphalidae;Limenitis;Limenitis reducta;France;GenBank;-;MH418978

Lepidoptera;Lycaenidae;Lycaena;Lycaena phlaeas;Italy;GenBank;-;MH418987
Lepidoptera;Nymphalidae;Lasiommata;Lasiommata megera;Italy;GenBank;-;MH419002
Lepidoptera;Pieridae;Pieris;Pieris rapae;Italy;GenBank;-;MH419003
Lepidoptera;Nymphalidae;Pyronia;Pyronia cecilia;Italy;Bold;WMB064-11;MH419008
Lepidoptera;Pieridae;Pontia;Pieris edusa;Italy;GenBank;-;MH419010
Lepidoptera;Papilionidae;Papilio;Papilio machaon;Malta;GenBank;-;MH419011
Lepidoptera;Nymphalidae;Lasiommata;Lasiommata paramegaera;France;GenBank;-;MH419015
Lepidoptera;Pieridae;Pieris;Pieris rapae;Italy;GenBank;-;MH419016
Lepidoptera;Papilionidae;Papilio;Papilio machaon;Italy;GenBank;-;MH419019
Lepidoptera;Pieridae;Pieris;Pieris mannii;Italy;GenBank;-;MH419022
Lepidoptera;Nymphalidae;Melitaea;Melitaea didyma;Italy;GenBank;-;MH419023
Lepidoptera;Pieridae;Pieris;Pieris rapae;Italy;GenBank;-;MH419031
Lepidoptera;Pieridae;Pieris;Pieris rapae;Italy;GenBank;-;MH419040
Lepidoptera;Papilionidae;Papilio;Papilio machaon;Italy;GenBank;-;MH419041
Lepidoptera;Lycaenidae;Lycaena;Lycaena phlaeas;France;GenBank;-;MH419043
Lepidoptera;Nymphalidae;Pyronia;Pyronia cecilia;Italy;Bold;WMB4607-14;MH419047
Lepidoptera ;Lycaenidae;Lampides;Lampides boeticus;Italy;GenBank;-;MH419057
Lepidoptera;Nymphalidae;Pyronia;Pyronia tithonus;France;Bold;WMB1529-13;MH419066
Lepidoptera;Pieridae;Pieris;Pieris brassicae;Italy;GenBank;-;MH419067
Lepidoptera ;Hesperiidae;Spialia;Spialia sertorius;Italy;GenBank;-;MH419085
Lepidoptera;Nymphalidae;Lasiommata;Lasiommata megera;Italy;GenBank;-;MH419088
Lepidoptera;Pieridae;Pieris;Pieris brassicae;Italy;GenBank;-;MH419090
Lepidoptera;Pieridae;Pieris;Pieris rapae;Malta;GenBank;-;MH419099
Lepidoptera;Nymphalidae;Lasiommata;Lasiommata megera;Italy;GenBank;-;MH419103
Lepidoptera;Nymphalidae;Pyronia;Pyronia cecilia;Italy;Bold;WMB1947-13;MH419104
Lepidoptera;Pieridae;Pieris;Pieris mannii;Italy;GenBank;-;MH419112
Lepidoptera ;Lycaenidae;Lampides;Lampides boeticus;Italy;GenBank;-;MH419113
Lepidoptera;Nymphalidae;Lasiommata;Lasiommata paramegaera;Italy;GenBank;-;MH419115
Lepidoptera ;Lycaenidae;Lampides;Lampides boeticus;Italy;GenBank;-;MH419119
Lepidoptera;Lycaenidae;Celastrina;Celastrina argiolus;Malta;GenBank;-;MH419123
Lepidoptera;Nymphalidae;Lasiommata;Lasiommata megera;Italy;GenBank;-;MH419125
Lepidoptera;Lycaenidae;Celastrina;Celastrina argiolus;Italy;GenBank;-;MH419142
Lepidoptera;Nymphalidae;Lasiommata;Lasiommata paramegaera;Italy;GenBank;-;MH419152
Lepidoptera;Nymphalidae;Lasiommata;Lasiommata megera;Italy;GenBank;-;MH419162
Lepidoptera;Nymphalidae;Lasiommata;Lasiommata megera;Italy;GenBank;-;MH419168
Lepidoptera;Nymphalidae;Lasiommata;Lasiommata paramegaera;France;GenBank;-;MH419169
Lepidoptera;Papilionidae;Papilio;Papilio machaon;Malta;GenBank;-;MH419173
Lepidoptera;Nymphalidae;Lasiommata;Lasiommata megera;Italy;GenBank;-;MH419174
Lepidoptera;Nymphalidae;Coenonympha;Coenonympha corinna;Italy;GenBank;-;MH419179
Lepidoptera;Nymphalidae;Lasiommata;Lasiommata megera;Italy;GenBank;-;MH419181
Lepidoptera ;Hesperiidae;Gegenes;Gegenes pumilio;Italy;GenBank;-;MH419194
Lepidoptera ;Pieridae;Gonepteryx;Gonepteryx cleopatra;Italy;GenBank;-;MH419198
Lepidoptera;Pieridae;Pieris;Pieris rapae;Italy;GenBank;-;MH419199
Lepidoptera;Nymphalidae;Lasiommata;Lasiommata megera;Italy;GenBank;-;MH419201
Lepidoptera ;Lycaenidae;Leptotes;Leptotes pirithous;Italy;GenBank;-;MH419207
Lepidoptera;Lycaenidae;Celastrina;Celastrina argiolus;Italy;GenBank;-;MH419216
Lepidoptera ;Lycaenidae;Lampides;Lampides boeticus;Italy;GenBank;-;MH419228
Lepidoptera;Nymphalidae;Coenonympha;Coenonympha pamphilus;France;GenBank;-;MH419231
Lepidoptera;Papilionidae;Papilio;Papilio machaon;Italy;GenBank;-;MH419239
Lepidoptera;Papilionidae;Papilio;Papilio machaon;Italy;GenBank;-;MH419240
Lepidoptera ;Lycaenidae;Leptotes;Leptotes pirithous;Italy;GenBank;-;MH419257
Lepidoptera;Lycaenidae;Lycaena;Lycaena phlaeas;Italy;GenBank;-;MH419258
Lepidoptera;Nymphalidae;Argynnis;Argynnis pandora;Italy;GenBank;-;MH419261
Lepidoptera;Pieridae;Pontia;Pieris edusa;Italy;GenBank;-;MH419265
Lepidoptera;Nymphalidae;Lasiommata;Lasiommata megera;Italy;GenBank;-;MH419269
Lepidoptera;Lycaenidae;Glaucopsyche;Glaucopsyche alexis;Italy;GenBank;-;MH419276
Lepidoptera;Nymphalidae;Vanessa;Vanessa atalanta;Italy;GenBank;-;MH419278
Lepidoptera;Pieridae;Pieris;Pieris brassicae;Italy;GenBank;-;MH419297
Lepidoptera;Pieridae;Pieris;Pieris rapae;Italy;GenBank;-;MH419298
Lepidoptera;Nymphalidae;Pyronia;Pyronia cecilia;Italy;Bold;WMB065-11;MH419301
Lepidoptera;Nymphalidae;Vanessa;Vanessa cardui;Italy;GenBank;-;MH419312
Lepidoptera;Pieridae;Pontia;Pieris edusa;Italy;GenBank;-;MH419313
Lepidoptera;Papilionidae;Papilio;Papilio machaon;Italy;GenBank;-;MH419314
Lepidoptera;Nymphalidae;Lasiommata;Lasiommata paramegaera;Italy;GenBank;-;MH419317
Lepidoptera;Nymphalidae;Pyronia;Pyronia cecilia;Italy;Bold;WMB1935-13;MH419319
Lepidoptera;Lycaenidae;Lycaena;Lycaena phlaeas;Italy;GenBank;-;MH419323
Lepidoptera;Lycaenidae;Celastrina;Celastrina argiolus;Italy;GenBank;-;MH419324
Lepidoptera ;Lycaenidae;Leptotes;Leptotes pirithous;Italy;GenBank;-;MH419332
Lepidoptera;Pieridae;Pieris;Pieris brassicae;Italy;GenBank;-;MH419335
Lepidoptera;Nymphalidae;Pyronia;Pyronia cecilia;Italy;Bold;WMB2761-13;MH419336
Lepidoptera;Lycaenidae;Lycaena;Lycaena phlaeas;Italy;GenBank;-;MH419338
Lepidoptera;Nymphalidae;Vanessa;Vanessa atalanta;Italy;GenBank;-;MH419344
Lepidoptera;Nymphalidae;Pyronia;Pyronia cecilia;Italy;Bold;WMB5927-17;MH419348
Lepidoptera;Nymphalidae;Lasiommata;Lasiommata megera;Italy;GenBank;-;MH419354
Lepidoptera;Pieridae;Leptidea;Leptidea sinapis;France;GenBank;-;MH419356
Lepidoptera;Nymphalidae;Argynnis;Argynnis pandora;Italy;GenBank;-;MH419361
Lepidoptera;Nymphalidae;Vanessa;Vanessa cardui;France;GenBank;-;MH419362
Lepidoptera;Lycaenidae;Glaucopsyche;Glaucopsyche alexis;Italy;GenBank;-;MH419369

Lepidoptera;Nymphalidae;Lasiommata;Lasiommata megera;Italy;GenBank;-;MH419370
Lepidoptera;Nymphalidae;Vanessa;Vanessa cardui;Italy;GenBank;-;MH419375
Lepidoptera;Nymphalidae;Pyronia;Pyronia cecilia;Italy;Bold;WMB5929-17;MH419381
Lepidoptera;Pieridae;Pontia;Pieris edusa;Italy;GenBank;-;MH419393
Lepidoptera ;Hesperiidae;Gegenes;Gegenes pumilio;Italy;GenBank;-;MH419396
Lepidoptera ;Lycaenidae;Lampides;Lampides boeticus;Italy;GenBank;-;MH419397
Lepidoptera ;Hesperiidae;Carcharodus;Carcharodus alceae;Italy;GenBank;-;MH419401
Lepidoptera ;Pieridae;Gonepteryx;Gonepteryx cleopatra;France;GenBank;-;MH419402
Lepidoptera;Pieridae;Pieris;Pieris brassicae;Italy;GenBank;-;MH419403
Lepidoptera;Nymphalidae;Pyronia;Pyronia cecilia;Italy;Bold;WMB5928-17;MH419405
Lepidoptera;Nymphalidae;Coenonympha;Coenonympha pamphilus;Italy;GenBank;-;MH419419
Lepidoptera;Nymphalidae;Coenonympha;Coenonympha pamphilus;Italy;GenBank;-;MH419421
Lepidoptera ;Lycaenidae;Leptotes;Leptotes pirithous;Italy;GenBank;-;MH419431
Lepidoptera ;Lycaenidae;Leptotes;Leptotes pirithous;Italy;GenBank;-;MH419436
Lepidoptera ;Lycaenidae;Lampides;Lampides boeticus;France;GenBank;-;MH419439
Lepidoptera ;Hesperiidae;Carcharodus;Carcharodus alceae;Italy;GenBank;-;MH419441
Lepidoptera ;Nymphalidae;Issoria;Issoria lathonia;Italy;GenBank;-;MH419442
Lepidoptera;Pieridae;Pieris;Pieris brassicae;Italy;GenBank;-;MH419460
Lepidoptera;Nymphalidae;Charaxes;Charaxes jasius;Italy;GenBank;-;MH419467
Lepidoptera;Lycaenidae;Aricia;Aricia agestis;Italy;GenBank;-;MH419470
Lepidoptera;Nymphalidae;Coenonympha;Coenonympha pamphilus;Italy;GenBank;-;MH419476
Lepidoptera ;Lycaenidae;Leptotes;Leptotes pirithous;Italy;GenBank;-;MH419483
Lepidoptera ;Lycaenidae;Lampides;Lampides boeticus;Italy;GenBank;-;MH419484
Lepidoptera ;Pieridae;Gonepteryx;Gonepteryx cleopatra;Italy;GenBank;-;MH419490
Lepidoptera;Pieridae;Pieris;Pieris rapae;Italy;GenBank;-;MH419491
Lepidoptera ;Hesperiidae;Carcharodus;Carcharodus alceae;Italy;GenBank;-;MH419492
Lepidoptera;Pieridae;Pieris;Pieris rapae;Italy;GenBank;-;MH419497
Lepidoptera;Nymphalidae;Charaxes;Charaxes jasius;Italy;GenBank;-;MH419500
Lepidoptera;Pieridae;Pieris;Pieris rapae;Italy;GenBank;-;MH419509
Lepidoptera;Nymphalidae;Coenonympha;Coenonympha pamphilus;Italy;GenBank;-;MH419513
Lepidoptera;Nymphalidae;Melitaea;Melitaea nevadensis;Italy;GenBank;-;MH419515
Lepidoptera ;Pieridae;Gonepteryx;Gonepteryx cleopatra;Italy;GenBank;-;MH419516
Lepidoptera;Pieridae;Pontia;Pieris edusa;Italy;GenBank;-;MH419521
Lepidoptera;Nymphalidae;Coenonympha;Coenonympha pamphilus;France;GenBank;-;MH419522
Lepidoptera ;Pieridae;Gonepteryx;Gonepteryx cleopatra;Italy;GenBank;-;MH419524
Lepidoptera;Pieridae;Pieris;Pieris rapae;France;GenBank;-;MH419525
Lepidoptera;Lycaenidae;Celastrina;Celastrina argiolus;Italy;GenBank;-;MH419527
Lepidoptera;Pieridae;Pieris;Pieris rapae;Italy;GenBank;-;MH419529
Lepidoptera;Nymphalidae;Lasiommata;Lasiommata paramegaera;France;GenBank;-;MH419543
Lepidoptera ;Lycaenidae;Leptotes;Leptotes pirithous;Italy;GenBank;-;MH419544
Lepidoptera;Nymphalidae;Vanessa;Vanessa atalanta;Italy;GenBank;-;MH419549
Lepidoptera;Lycaenidae;Lycaena;Lycaena phlaeas;France;GenBank;-;MH419555
Lepidoptera;Pieridae;Pieris;Pieris rapae;Italy;GenBank;-;MH419559
Lepidoptera;Pieridae;Pontia;Pieris edusa;Italy;GenBank;-;MH419560
Lepidoptera;Pieridae;Pieris;Pieris brassicae;Italy;GenBank;-;MH419566
Lepidoptera ;Lycaenidae;Lampides;Lampides boeticus;Italy;GenBank;-;MH419569
Lepidoptera;Lycaenidae;Lycaena;Lycaena phlaeas;Italy;GenBank;-;MH419576
Lepidoptera;Nymphalidae;Charaxes;Charaxes jasius;Italy;GenBank;-;MH419585
Lepidoptera;Nymphalidae;Lasiommata;Lasiommata megera;Italy;GenBank;-;MH419587
Lepidoptera;Nymphalidae;Coenonympha;Coenonympha corinna;Italy;GenBank;-;MH419590
Lepidoptera;Nymphalidae;Pyronia;Pyronia tithonus;France;Bold;WMB1528-13;MH419591
Lepidoptera;Nymphalidae;Vanessa;Vanessa cardui;Italy;GenBank;-;MH419594
Lepidoptera;Nymphalidae;Lasiommata;Lasiommata megera;Italy;GenBank;-;MH419599
Lepidoptera;Lycaenidae;Celastrina;Celastrina argiolus;Italy;GenBank;-;MH419621
Lepidoptera;Pieridae;Pieris;Pieris rapae;Malta;GenBank;-;MH419630
Lepidoptera;Pieridae;Pieris;Pieris brassicae;Italy;GenBank;-;MH419640
Lepidoptera;Nymphalidae;Pyronia;Pyronia cecilia;Italy;Bold;WMB2775-13;MH419642
Lepidoptera;Pieridae;Euchloe;Euchloe ausonia;Italy;GenBank;-;MH419647
Lepidoptera;Lycaenidae;Lycaena;Lycaena phlaeas;Italy;GenBank;-;MH419653
Lepidoptera;Lycaenidae;Celastrina;Celastrina argiolus;Italy;GenBank;-;MH419657
Lepidoptera ;Lycaenidae;Lampides;Lampides boeticus;Italy;GenBank;-;MH419658
Lepidoptera;Nymphalidae;Lasiommata;Lasiommata paramegaera;Italy;GenBank;-;MH419660
Lepidoptera;Nymphalidae;Lasiommata;Lasiommata megera;Malta;GenBank;-;MH419663
Lepidoptera;Papilionidae;Papilio;Papilio machaon;Italy;GenBank;-;MH419665
Lepidoptera;Nymphalidae;Pyronia;Pyronia cecilia;Italy;Bold;WMB1934-13;MH419667
Lepidoptera ;Hesperiidae;Gegenes;Gegenes pumilio;Italy;GenBank;-;MH419668
Lepidoptera;Nymphalidae;Melitaea;Melitaea didyma;Italy;GenBank;-;MH419673
Lepidoptera;Nymphalidae;Pyronia;Pyronia cecilia;Italy;Bold;WMB656-11;MH419675
Lepidoptera;Nymphalidae;Lasiommata;Lasiommata megera;Italy;GenBank;-;MH419680
Lepidoptera;Nymphalidae;Charaxes;Charaxes jasius;Italy;GenBank;-;MH419687
Lepidoptera;Pieridae;Pieris;Pieris rapae;Malta;GenBank;-;MH419690
Lepidoptera;Hesperiidae;Thymelicus;Thymelicus acteon;Italy;GenBank;-;MH419692
Lepidoptera;Lycaenidae;Callophrys;Callophrys rubi;Italy;GenBank;-;MH419693
Lepidoptera;Nymphalidae;Vanessa;Vanessa cardui;Italy;GenBank;-;MH419702
Lepidoptera ;Hesperiidae;Carcharodus;Carcharodus alceae;Italy;GenBank;-;MH419708
Lepidoptera;Papilionidae;Papilio;Papilio machaon;Italy;GenBank;-;MH419718
Lepidoptera;Pieridae;Pieris;Pieris rapae;Italy;GenBank;-;MH419719
Lepidoptera;Nymphalidae;Lasiommata;Lasiommata paramegaera;Italy;GenBank;-;MH419720

Lepidoptera;Lycaenidae;Celastrina;Celastrina argiolus;France;GenBank;-;MH419723
Lepidoptera ;Hesperiidae;Gegenes;Gegenes pumilio;Italy;GenBank;-;MH419725
Lepidoptera;Pieridae;Pieris;Pieris mannii;Italy;GenBank;-;MH419729
Lepidoptera;Nymphalidae;Pyronia;Pyronia cecilia;Italy;Bold;WMB517-11;MH419733
Lepidoptera;Nymphalidae;Lasiommata;Lasiommata paramegaera;Italy;GenBank;-;MH419734
Lepidoptera ;Lycaenidae;Lampides;Lampides boeticus;Italy;GenBank;-;MH419738
Lepidoptera ;Pieridae;Gonepteryx;Gonepteryx cleopatra;Italy;GenBank;-;MH419740
Lepidoptera;Pieridae;Pieris;Pieris rapae;Italy;GenBank;-;MH419742
Lepidoptera;Nymphalidae;Coenonympha;Coenonympha corinna;Italy;GenBank;-;MH419750
Lepidoptera;Nymphalidae;Vanessa;Vanessa atalanta;Italy;GenBank;-;MH419755
Lepidoptera ;Pieridae;Gonepteryx;Gonepteryx cleopatra;Italy;GenBank;-;MH419759
Lepidoptera ;Hesperiidae;Gegenes;Gegenes pumilio;Italy;GenBank;-;MH419761
Lepidoptera;Pieridae;Pieris;Pieris rapae;Italy;GenBank;-;MH419770
Lepidoptera;Lycaenidae;Lycaena;Lycaena phlaeas;Italy;GenBank;-;MH419772
Lepidoptera ;Pieridae;Gonepteryx;Gonepteryx cleopatra;Italy;GenBank;-;MH419779
Lepidoptera;Pieridae;Pontia;Pieris edusa;Italy;GenBank;-;MH419781
Lepidoptera;Lycaenidae;Celastrina;Celastrina argiolus;Italy;GenBank;-;MH419785
Lepidoptera ;Hesperiidae;Carcharodus;Carcharodus alceae;Italy;GenBank;-;MH419796
Lepidoptera;Nymphalidae;Vanessa;Vanessa cardui;Italy;GenBank;-;MH419799
Lepidoptera ;Pieridae;Gonepteryx;Gonepteryx cleopatra;Malta;GenBank;-;MH419805
Lepidoptera;Nymphalidae;Vanessa;Vanessa atalanta;Italy;GenBank;-;MH419809
Lepidoptera;Pieridae;Pieris;Pieris rapae;Italy;GenBank;-;MH419815
Lepidoptera;Nymphalidae;Lasiommata;Lasiommata paramegaera;Italy;GenBank;-;MH419816
Lepidoptera ;Lycaenidae;Lampides;Lampides boeticus;Italy;GenBank;-;MH419824
Lepidoptera;Lycaenidae;Callophrys;Callophrys rubi;Italy;GenBank;-;MH419831
Lepidoptera;Nymphalidae;Vanessa;Vanessa atalanta;Italy;GenBank;-;MH419832
Lepidoptera ;Pieridae;Gonepteryx;Gonepteryx cleopatra;Italy;GenBank;-;MH419833
Lepidoptera;Pieridae;Pieris;Pieris rapae;Italy;GenBank;-;MH419839
Lepidoptera ;Lycaenidae;Lampides;Lampides boeticus;Italy;GenBank;-;MH419842
Lepidoptera ;Pieridae;Gonepteryx;Gonepteryx cleopatra;France;GenBank;-;MH419843
Lepidoptera;Nymphalidae;Lasiommata;Lasiommata megera;Italy;GenBank;-;MH419851
Lepidoptera ;Pieridae;Gonepteryx;Gonepteryx cleopatra;Italy;GenBank;-;MH419855
Lepidoptera;Nymphalidae;Vanessa;Vanessa atalanta;Italy;GenBank;-;MH419859
Lepidoptera;Lycaenidae;Lycaena;Lycaena phlaeas;Italy;GenBank;-;MH419879
Lepidoptera ;Hesperiidae;Carcharodus;Carcharodus alceae;Italy;GenBank;-;MH419880
Lepidoptera;Lycaenidae;Celastrina;Celastrina argiolus;Italy;GenBank;-;MH419896
Lepidoptera;Papilionidae;Papilio;Papilio machaon;Italy;GenBank;-;MH419910
Lepidoptera;Nymphalidae;Coenonympha;Coenonympha pamphilus;Italy;GenBank;-;MH419911
Lepidoptera;Nymphalidae;Lasiommata;Lasiommata megera;Italy;GenBank;-;MH419920
Lepidoptera;Nymphalidae;Coenonympha;Coenonympha corinna;Italy;GenBank;-;MH419925
Lepidoptera;Pieridae;Pieris;Pieris rapae;Italy;GenBank;-;MH419926
Lepidoptera;Nymphalidae;Lasiommata;Lasiommata paramegaera;France;GenBank;-;MH419927
Lepidoptera;Papilionidae;Papilio;Papilio machaon;Malta;GenBank;-;MH419932
Lepidoptera ;Lycaenidae;Leptotes;Leptotes pirithous;Italy;GenBank;-;MH419935
Lepidoptera;Nymphalidae;Lasiommata;Lasiommata megera;Italy;GenBank;-;MH419937
Lepidoptera;Pieridae;Pieris;Pieris rapae;Italy;GenBank;-;MH419942
Lepidoptera;Nymphalidae;Coenonympha;Coenonympha corinna;Italy;GenBank;-;MH419945
Lepidoptera;Nymphalidae;Maniola;Maniola jurtina;France;GenBank;-;MH419946
Lepidoptera ;Lycaenidae;Lampides;Lampides boeticus;Italy;GenBank;-;MH419950
Lepidoptera;Pieridae;Pieris;Pieris brassicae;Italy;GenBank;-;MH419952
Lepidoptera ;Lycaenidae;Lampides;Lampides boeticus;France;GenBank;-;MH419965
Lepidoptera;Pieridae;Pieris;Pieris mannii;Italy;GenBank;-;MH419969
Lepidoptera;Pieridae;Leptidea;Leptidea sinapis;Italy;GenBank;-;MH419970
Lepidoptera;Nymphalidae;Pyronia;Pyronia cecilia;Italy;Bold;WMB2788-13;MH419971
Lepidoptera;Nymphalidae;Lasiommata;Lasiommata paramegaera;Italy;GenBank;-;MH419974
Lepidoptera ;Lycaenidae;Leptotes;Leptotes pirithous;Italy;GenBank;-;MH419975
Lepidoptera ;Hesperiidae;Gegenes;Gegenes pumilio;Italy;GenBank;-;MH419977
Lepidoptera;Nymphalidae;Lasiommata;Lasiommata megera;Italy;GenBank;-;MH419982
Lepidoptera;Lycaenidae;Lycaena;Lycaena phlaeas;Italy;GenBank;-;MH419984
Lepidoptera ;Lycaenidae;Leptotes;Leptotes pirithous;Italy;GenBank;-;MH419988
Lepidoptera ;Lycaenidae;Leptotes;Leptotes pirithous;Italy;GenBank;-;MH419990
Lepidoptera;Nymphalidae;Lasiommata;Lasiommata megera;Italy;GenBank;-;MH419996
Lepidoptera ;Lycaenidae;Lampides;Lampides boeticus;Italy;GenBank;-;MH420003
Lepidoptera;Pieridae;Pieris;Pieris mannii;Italy;GenBank;-;MH420005
Lepidoptera;Nymphalidae;Lasiommata;Lasiommata megera;Italy;GenBank;-;MH420011
Lepidoptera;Nymphalidae;Hipparchia;Hipparchia aristaeus;Italy;GenBank;-;MH420016
Lepidoptera ;Lycaenidae;Lampides;Lampides boeticus;Malta;GenBank;-;MH420024
Lepidoptera;Nymphalidae;Lasiommata;Lasiommata megera;Italy;GenBank;-;MH420033
Lepidoptera;Lycaenidae;Celastrina;Celastrina argiolus;Italy;GenBank;-;MH420035
Lepidoptera ;Lycaenidae;Leptotes;Leptotes pirithous;Italy;GenBank;-;MH420040
Lepidoptera;Pieridae;Euchloe;Euchloe ausonia;Italy;GenBank;-;MH420042
Lepidoptera;Nymphalidae;Lasiommata;Lasiommata megera;Italy;GenBank;-;MH420051
Lepidoptera;Nymphalidae;Lasiommata;Lasiommata megera;Italy;GenBank;-;MH420055
Lepidoptera ;Hesperiidae;Gegenes;Gegenes pumilio;Italy;GenBank;-;MH420056
Lepidoptera;Nymphalidae;Lasiommata;Lasiommata megera;Malta;GenBank;-;MH420061
Lepidoptera;Papilionidae;Papilio;Papilio machaon;Malta;GenBank;-;MH420063
Lepidoptera;Lycaenidae;Lycaena;Lycaena phlaeas;Italy;GenBank;-;MH420069
Lepidoptera;Lycaenidae;Lycaena;Lycaena phlaeas;Italy;GenBank;-;MH420073

Lepidoptera;Nymphalidae;Lasiommata;Lasiommata megera;Italy;GenBank;-;MH420076
Lepidoptera ;Lycaenidae;Lampides;Lampides boeticus;Italy;GenBank;-;MH420085
Lepidoptera;Pieridae;Anthocharis;Anthocharis cardamines;Italy;GenBank;-;MH420092
Lepidoptera ;Lycaenidae;Leptotes;Leptotes pirithous;Italy;GenBank;-;MH420118
Lepidoptera;Nymphalidae;Vanessa;Vanessa cardui;Italy;GenBank;-;MH420133
Lepidoptera;Pieridae;Pieris;Pieris rapae;Malta;GenBank;-;MH420135
Lepidoptera;Nymphalidae;Pyronia;Pyronia cecilia;Italy;Bold;WMB1807-13;MH420138
Lepidoptera;Lycaenidae;Celastrina;Celastrina argiolus;Italy;GenBank;-;MH420143
Lepidoptera;Nymphalidae;Pyronia;Pyronia cecilia;Italy;Bold;WMB4601-14;MH420145
Lepidoptera;Nymphalidae;Vanessa;Vanessa atalanta;Italy;GenBank;-;MH420146
Lepidoptera;Nymphalidae;Vanessa;Vanessa atalanta;Italy;GenBank;-;MH420153
Lepidoptera;Lycaenidae;Lycaena;Lycaena phlaeas;Italy;GenBank;-;MH420158
Lepidoptera;Papilionidae;Papilio;Papilio machaon;Italy;GenBank;-;MH420159
Lepidoptera ;Hesperiidae;Carcharodus;Carcharodus alceae;Italy;GenBank;-;MH420164
Lepidoptera;Nymphalidae;Lasiommata;Lasiommata megera;Italy;GenBank;-;MH420168
Lepidoptera;Lycaenidae;Celastrina;Celastrina argiolus;Italy;GenBank;-;MH420170
Lepidoptera;Nymphalidae;Coenonympha;Coenonympha pamphilus;France;GenBank;-;MH420173
Lepidoptera;Pieridae;Pieris;Pieris rapae;Italy;GenBank;-;MH420174
Lepidoptera;Nymphalidae;Coenonympha;Coenonympha corinna;Italy;GenBank;-;MH420175
Lepidoptera ;Lycaenidae;Lampides;Lampides boeticus;Italy;GenBank;-;MH420176
Lepidoptera ;Lycaenidae;Leptotes;Leptotes pirithous;Italy;GenBank;-;MH420178
Lepidoptera ;Hesperiidae;Carcharodus;Carcharodus alceae;Italy;GenBank;-;MH420180
Lepidoptera;Nymphalidae;Lasiommata;Lasiommata megera;Italy;GenBank;-;MH420189
Lepidoptera;Nymphalidae;Lasiommata;Lasiommata paramegaera;Italy;GenBank;-;MH420194
Lepidoptera;Nymphalidae;Vanessa;Vanessa cardui;Italy;GenBank;-;MH420196
Lepidoptera ;Pieridae;Gonepteryx;Gonepteryx cleopatra;Italy;GenBank;-;MH420199
Lepidoptera;Pieridae;Pieris;Pieris mannii;Italy;GenBank;-;MH420202
Lepidoptera;Nymphalidae;Lasiommata;Lasiommata megera;Italy;GenBank;-;MH420204
Lepidoptera;Pieridae;Pieris;Pieris rapae;Italy;GenBank;-;MH420205
Lepidoptera;Lycaenidae;Celastrina;Celastrina argiolus;Italy;GenBank;-;MH420209
Lepidoptera;Pieridae;Pieris;Pieris rapae;Italy;GenBank;-;MH420210
Lepidoptera;Nymphalidae;Pyronia;Pyronia cecilia;Italy;Bold;WMB1922-13;MH420212
Lepidoptera;Nymphalidae;Lasiommata;Lasiommata paramegaera;France;GenBank;-;MH420214
Lepidoptera;Nymphalidae;Vanessa;Vanessa atalanta;Italy;GenBank;-;MH420217
Lepidoptera;Pieridae;Pieris;Pieris brassicae;Italy;GenBank;-;MH420218
Lepidoptera;Nymphalidae;Lasiommata;Lasiommata megera;Italy;GenBank;-;MH420224
Lepidoptera;Nymphalidae;Coenonympha;Coenonympha corinna;Italy;GenBank;-;MH420226
Lepidoptera;Lycaenidae;Lycaena;Lycaena phlaeas;Italy;GenBank;-;MH420232
Lepidoptera ;Lycaenidae;Lampides;Lampides boeticus;Italy;GenBank;-;MH420233
Lepidoptera;Pieridae;Euchloe;Euchloe ausonia;Italy;GenBank;-;MH420238
Lepidoptera;Nymphalidae;Vanessa;Vanessa cardui;Italy;GenBank;-;MH420240
Lepidoptera;Nymphalidae;Pyronia;Pyronia cecilia;Italy;Bold;WMB1061-13;MH420246
Lepidoptera;Nymphalidae;Coenonympha;Coenonympha corinna;Italy;GenBank;-;MH420251
Lepidoptera;Nymphalidae;Pyronia;Pyronia cecilia;Italy;Bold;WMB542-11;MH420253
Lepidoptera;Nymphalidae;Pyronia;Pyronia cecilia;Italy;Bold;WMB2766-13;MH420262
Lepidoptera;Pieridae;Pieris;Pieris rapae;Italy;GenBank;-;MH420266
Lepidoptera;Nymphalidae;Vanessa;Vanessa cardui;Italy;GenBank;-;MH420271
Lepidoptera;Pieridae;Pieris;Pieris rapae;Italy;GenBank;-;MH420274
Lepidoptera;Lycaenidae;Celastrina;Celastrina argiolus;Italy;GenBank;-;MH420275
Lepidoptera;Nymphalidae;Coenonympha;Coenonympha pamphilus;Italy;GenBank;-;MH420277
Lepidoptera ;Pieridae;Gonepteryx;Gonepteryx rhamni;Italy;GenBank;-;MH420280
Lepidoptera ;Lycaenidae;Leptotes;Leptotes pirithous;Italy;GenBank;-;MH420283
Lepidoptera ;Lycaenidae;Lampides;Lampides boeticus;Italy;GenBank;-;MH420284
Lepidoptera;Nymphalidae;Coenonympha;Coenonympha corinna;Italy;GenBank;-;MH420287
Lepidoptera;Nymphalidae;Hipparchia;Hipparchia aristaeus;Italy;GenBank;-;MH420289
Lepidoptera;Nymphalidae;Lasiommata;Lasiommata megera;Italy;GenBank;-;MH420294
Lepidoptera;Nymphalidae;Charaxes;Charaxes jasius;Italy;GenBank;-;MH420297
Lepidoptera;Lycaenidae;Celastrina;Celastrina argiolus;Italy;GenBank;-;MH420301
Lepidoptera;Nymphalidae;Argynnis;Argynnis pandora;Italy;GenBank;-;MH420304
Lepidoptera;Papilionidae;Papilio;Papilio machaon;France;GenBank;-;MH420311
Lepidoptera;Lycaenidae;Lycaena;Lycaena phlaeas;Italy;GenBank;-;MH420315
Lepidoptera;Nymphalidae;Lasiommata;Lasiommata megera;Italy;GenBank;-;MH420316
Lepidoptera;Lycaenidae;Celastrina;Celastrina argiolus;Italy;GenBank;-;MH420324
Lepidoptera;Lycaenidae;Lycaena;Lycaena phlaeas;Italy;GenBank;-;MH420326
Lepidoptera;Pieridae;Pieris;Pieris rapae;Italy;GenBank;-;MH420330
Lepidoptera;Lycaenidae;Callophrys;Callophrys rubi;Italy;GenBank;-;MH420333
Lepidoptera;Nymphalidae;Lasiommata;Lasiommata megera;Italy;GenBank;-;MH420335
Lepidoptera;Pieridae;Pieris;Pieris brassicae;Italy;GenBank;-;MH420364
Lepidoptera;Lycaenidae;Celastrina;Celastrina argiolus;Italy;GenBank;-;MH420369
Lepidoptera;Pieridae;Pieris;Pieris napi;Italy;GenBank;-;MH420372
Lepidoptera;Pieridae;Pieris;Pieris brassicae;Italy;GenBank;-;MH420374
Lepidoptera ;Hesperiidae;Carcharodus;Carcharodus alceae;Italy;GenBank;-;MH420379
Lepidoptera;Nymphalidae;Pyronia;Pyronia cecilia;France;Bold;WMB862-13;MH420382
Lepidoptera;Hesperiidae;Pyrgus;Pyrgus armoricanus;Italy;GenBank;-;MH420394
Lepidoptera;Pieridae;Pieris;Pieris rapae;Italy;GenBank;-;MH420397
Lepidoptera;Nymphalidae;Vanessa;Vanessa cardui;France;GenBank;-;MH420399
Lepidoptera;Nymphalidae;Charaxes;Charaxes jasius;Italy;GenBank;-;MH420413
Lepidoptera;Nymphalidae;Vanessa;Vanessa cardui;Italy;GenBank;-;MH420417

Lepidoptera;Nymphalidae;Erebia;Erebia manto;Romania;GenBank;-;MH670084
Lepidoptera;Nymphalidae;Erebia;Erebia manto;France;GenBank;-;MH670090
Lepidoptera;Nymphalidae;Erebia;Erebia manto;France;GenBank;-;MH670105
Lepidoptera;Nymphalidae;Erebia;Erebia manto;Bosnia and Herzegovina;GenBank;-;MH670108
Lepidoptera;Nymphalidae;Erebia;Erebia manto;Bosnia and Herzegovina;GenBank;-;MH670123
Lepidoptera;Nymphalidae;Erebia;Erebia manto;Romania;GenBank;-;MH670131
Lepidoptera;Nymphalidae;Erebia;Erebia manto;Romania;GenBank;-;MH670141
Lepidoptera;Nymphalidae;Erebia;Erebia manto;Romania;GenBank;-;MH670143
Lepidoptera;Nymphalidae;Erebia;Erebia manto;Bosnia and Herzegovina;GenBank;-;MH670153
Lepidoptera;Nymphalidae;Erebia;Erebia manto;Bosnia and Herzegovina;GenBank;-;MH670166
Lepidoptera;Nymphalidae;Erebia;Erebia manto;Romania;GenBank;-;MH670182
Lepidoptera;Nymphalidae;Erebia;Erebia manto;Bosnia and Herzegovina;GenBank;-;MH670201
Lepidoptera;Nymphalidae;Erebia;Erebia manto;France;GenBank;-;MH670202
Lepidoptera;Nymphalidae;Erebia;Erebia manto;France;GenBank;-;MH670215
Lepidoptera;Nymphalidae;Erebia;Erebia manto;Romania;GenBank;-;MH670217
Lepidoptera;Nymphalidae;Erebia;Erebia epiphron;Macedonia;GenBank;-;MK155193
Lepidoptera;Lycaenidae;Satyrium;Satyrium pruni;Russia;GenBank;-;MK343429
Lepidoptera;Lycaenidae;Pseudophilotes;Pseudophilotes baton;Italy;GenBank;-;MK567857
Lepidoptera;Papilionidae;Iphiclides;Iphiclides podalirius;Italy;GenBank;-;MK587201
Lepidoptera;Hesperiidae;Thymelicus;Thymelicus sylvestris;United Kingdom;GenBank;-;MK812967
Lepidoptera;Lycaenidae;Aricia;Aricia agestis;Czech Republic;GenBank;-;MN107394
Lepidoptera;Lycaenidae;Aricia;Aricia agestis;Czech Republic;GenBank;-;MN107395
Lepidoptera;Nymphalidae;Libythea;Libythea celtis;Italy;GenBank;-;MN138510
Lepidoptera;Nymphalidae;Argynnis;Argynnis paphia;Sweden;GenBank;-;MN138517
Lepidoptera;Nymphalidae;Coenonympha;Coenonympha pamphilus;United Kingdom;GenBank;-;MN138548
Lepidoptera;Pieridae;Pieris;Pieris brassicae;Italy;GenBank;-;MN138552
Lepidoptera;Pieridae;Pieris;Pieris rapae;France;GenBank;-;MN138553
Lepidoptera;Lycaenidae;Polyommatus;Polyommatus icarus;United Kingdom;GenBank;-;MN138555
Lepidoptera;Nymphalidae;Libythea;Libythea celtis;Austria;GenBank;-;MN138556
Lepidoptera ;Lycaenidae;Lampides;Lampides boeticus;Italy;GenBank;-;MN138557
Lepidoptera;Lycaenidae;Celastrina;Celastrina argiolus;Italy;GenBank;-;MN138587
Lepidoptera;Lycaenidae;Lycaena;Lycaena phlaeas;Italy;GenBank;-;MN138589
Lepidoptera ;Hesperiidae;Gegenes;Gegenes nostrodamus;Italy;GenBank;-;MN138601
Lepidoptera;Nymphalidae;Coenonympha;Coenonympha corinna;Italy;GenBank;-;MN138602
Lepidoptera;Nymphalidae;Maniola;Maniola jurtina;United Kingdom;GenBank;-;MN138630
Lepidoptera;Lycaenidae;Celastrina;Celastrina argiolus;United Kingdom;GenBank;-;MN138636
Lepidoptera;Hesperiidae;Thymelicus;Thymelicus acteon;Italy;GenBank;-;MN138653
Lepidoptera;Papilionidae;Papilio;Papilio machaon;Italy;GenBank;-;MN138654
Lepidoptera;Nymphalidae;Maniola;Maniola jurtina;United Kingdom;GenBank;-;MN138659
Lepidoptera;Nymphalidae;Hipparchia;Hipparchia fagi;Italy;GenBank;-;MN138679
Lepidoptera;Nymphalidae;Melanargia;Melanargia arge;Italy;GenBank;-;MN138692
Lepidoptera;Nymphalidae;Vanessa;Vanessa atalanta;Italy;GenBank;-;MN138694
Lepidoptera;Lycaenidae;Satyrium;Satyrium ilicis;Italy;GenBank;-;MN138699
Lepidoptera;Pieridae;Anthocharis;Anthocharis cardamines;United Kingdom;GenBank;-;MN138700
Lepidoptera;Nymphalidae;Hipparchia;Hipparchia leighebi;Italy;GenBank;-;MN138710
Lepidoptera;Nymphalidae;Maniola;Maniola jurtina;United Kingdom;GenBank;-;MN138714
Lepidoptera;Lycaenidae;Polyommatus;Polyommatus icarus;United Kingdom;GenBank;-;MN138725
Lepidoptera;Lycaenidae;Polyommatus;Polyommatus icarus;United Kingdom;GenBank;-;MN138731
Lepidoptera;Lycaenidae;Celastrina;Celastrina argiolus;Italy;GenBank;-;MN138745
Lepidoptera;Pieridae;Pieris;Pieris mannii;Italy;GenBank;-;MN138775
Lepidoptera;Nymphalidae;Melitaea;Melitaea nevadensis;Italy;GenBank;-;MN138802
Lepidoptera;Pieridae;Euchloe;Euchloe ausonia;Italy;GenBank;-;MN138808
Lepidoptera;Nymphalidae;Hipparchia;Hipparchia statilinus;Italy;GenBank;-;MN138838
Lepidoptera ;Pieridae;Gonepteryx;Gonepteryx cleopatra;Italy;GenBank;-;MN138859
Lepidoptera;Nymphalidae;Coenonympha;Coenonympha corinna;Italy;GenBank;-;MN138880
Lepidoptera;Nymphalidae;Hipparchia;Hipparchia semele;United Kingdom;GenBank;-;MN138897
Lepidoptera ;Lycaenidae;Lampides;Lampides boeticus;France;GenBank;-;MN138912
Lepidoptera;Pieridae;Pieris;Pieris rapae;France;GenBank;-;MN138915
Lepidoptera;Nymphalidae;Maniola;Maniola jurtina;United Kingdom;GenBank;-;MN138916
Lepidoptera;Nymphalidae;Maniola;Maniola jurtina;Italy;GenBank;-;MN138918
Lepidoptera;Nymphalidae;Polygonia;Polygonia c-album;Italy;Bold;OXB836-15;MN138959
Lepidoptera;Nymphalidae;Lasiommata;Lasiommata paramegaera;Italy;GenBank;-;MN139015
Lepidoptera;Nymphalidae;Hipparchia;Hipparchia leighebi;Italy;GenBank;-;MN139051
Lepidoptera;Lycaenidae;Polyommatus;Polyommatus icarus;United Kingdom;GenBank;-;MN139070
Lepidoptera;Lycaenidae;Satyrium;Satyrium ilicis;Italy;GenBank;-;MN139072
Lepidoptera;Nymphalidae;Maniola;Maniola jurtina;United Kingdom;GenBank;-;MN139094
Lepidoptera;Nymphalidae;Melanargia;Melanargia arge;Italy;GenBank;-;MN139098
Lepidoptera;Nymphalidae;Hipparchia;Hipparchia sbordonii;Italy;GenBank;-;MN139101
Lepidoptera;Nymphalidae;Maniola;Maniola jurtina;United Kingdom;GenBank;-;MN139107
Lepidoptera;Lycaenidae;Plebejus;Plebejus argus;United Kingdom;GenBank;-;MN139116
Lepidoptera;Nymphalidae;Libythea;Libythea celtis;Austria;GenBank;-;MN139125
Lepidoptera;Nymphalidae;Maniola;Maniola jurtina;United Kingdom;GenBank;-;MN139154
Lepidoptera;Lycaenidae;Polyommatus;Polyommatus icarus;United Kingdom;GenBank;-;MN139193
Lepidoptera;Hesperiidae;Muschampia;Carcharodus flocciferus;Italy;GenBank;-;MN139211
Lepidoptera;Nymphalidae;Maniola;Maniola jurtina;United Kingdom;GenBank;-;MN139220
Lepidoptera;Nymphalidae;Coenonympha;Coenonympha pamphilus;Italy;GenBank;-;MN139242
Lepidoptera;Lycaenidae;Polyommatus;Polyommatus icarus;United Kingdom;GenBank;-;MN139258
Lepidoptera;Nymphalidae;Maniola;Maniola jurtina;United Kingdom;GenBank;-;MN139299

Lepidoptera;Pieridae;Pieris;Pieris rapae;Italy;GenBank;-;MN139309
Lepidoptera;Nymphalidae;Charaxes;Charaxes jasius;France;GenBank;-;MN139316
Lepidoptera;Lycaenidae;Plebejus;Plebejus argus;Slovenia;GenBank;-;MN139324
Lepidoptera ;Lycaenidae;Lampides;Lampides boeticus;Italy;GenBank;-;MN139332
Lepidoptera;Nymphalidae;Melanargia;Melanargia arge;Italy;GenBank;-;MN139340
Lepidoptera;Pieridae;Pieris;Pieris napi;United Kingdom;GenBank;-;MN139350
Lepidoptera;Hesperiidae;Thymelicus;Thymelicus acteon;Italy;GenBank;-;MN139437
Lepidoptera;Lycaenidae;Polyommatus;Polyommatus icarus;United Kingdom;GenBank;-;MN139453
Lepidoptera ;Pieridae;Gonepteryx;Gonepteryx cleopatra;France;GenBank;-;MN139481
Lepidoptera;Lycaenidae;Cupido;Cupido minimus;United Kingdom;GenBank;-;MN139515
Lepidoptera;Nymphalidae;Pyronia;Pyronia tithonus;France;Bold;OXB1341-15;MN139529
Lepidoptera;Nymphalidae;Limenitis;Limenitis reducta;Italy;GenBank;-;MN139556
Lepidoptera;Nymphalidae;Lasiommata;Lasiommata megera;Italy;GenBank;-;MN139563
Lepidoptera;Pieridae;Pieris;Pieris rapae;Italy;GenBank;-;MN139603
Lepidoptera ;Nymphalidae;Brintesia;Kanetisa circe;France;GenBank;-;MN139613
Lepidoptera;Nymphalidae;Pyronia;Pyronia cecilia;Italy;Bold;WMB910-13;MN139622
Lepidoptera;Lycaenidae;Phengaris;Maculinea arion;France;GenBank;-;MN139638
Lepidoptera;Nymphalidae;Coenonympha;Coenonympha corinna;Italy;GenBank;-;MN139660
Lepidoptera;Nymphalidae;Hipparchia;Hipparchia neapolitana;Italy;GenBank;-;MN139670
Lepidoptera;Pieridae;Leptidea;Leptidea juvernica;Slovenia;GenBank;-;MN139694
Lepidoptera ;Nymphalidae;Brintesia;Kanetisa circe;Italy;GenBank;-;MN139721
Lepidoptera;Pieridae;Pieris;Pieris napi;United Kingdom;GenBank;-;MN139735
Lepidoptera;Pieridae;Pieris;Pieris rapae;Italy;GenBank;-;MN139737
Lepidoptera;Pieridae;Pieris;Pieris brassicae;United Kingdom;GenBank;-;MN139746
Lepidoptera;Hesperiidae;Muschampia;Carcharodus flocciferus;Italy;GenBank;-;MN139748
Lepidoptera;Nymphalidae;Lasiommata;Lasiommata megera;Italy;GenBank;-;MN139790
Lepidoptera;Pieridae;Pontia;Pieris daplidice;Italy;GenBank;-;MN139791
Lepidoptera;Nymphalidae;Hipparchia;Hipparchia leighebi;Italy;GenBank;-;MN139805
Lepidoptera;Nymphalidae;Nymphalis;Nymphalis io;France;GenBank;-;MN139816
Lepidoptera;Nymphalidae;Hipparchia;Hipparchia leighebi;Italy;GenBank;-;MN139839
Lepidoptera;Hesperiidae;Thymelicus;Thymelicus acteon;United Kingdom;GenBank;-;MN139848
Lepidoptera;Nymphalidae;Coenonympha;Coenonympha corinna;Italy;GenBank;-;MN139863
Lepidoptera;Pieridae;Pieris;Pieris napi;United Kingdom;GenBank;-;MN139864
Lepidoptera;Nymphalidae;Hipparchia;Hipparchia aristaeus;Italy;GenBank;-;MN139900
Lepidoptera;Nymphalidae;Hipparchia;Hipparchia semele;United Kingdom;GenBank;-;MN139919
Lepidoptera;Nymphalidae;Melitaea;Melitaea didyma;Italy;GenBank;-;MN139924
Lepidoptera;Nymphalidae;Maniola;Maniola jurtina;United Kingdom;GenBank;-;MN139972
Lepidoptera;Nymphalidae;Coenonympha;Coenonympha oedippus;Austria;GenBank;-;MN139986
Lepidoptera;Lycaenidae;Polyommatus;Polyommatus icarus;Italy;GenBank;-;MN139987
Lepidoptera;Nymphalidae;Lasiommata;Lasiommata megera;France;GenBank;-;MN140053
Lepidoptera;Hesperiidae;Thymelicus;Thymelicus lineola;Italy;GenBank;-;MN140070
Lepidoptera;Lycaenidae;Satyrium;Satyrium ilicis;Italy;GenBank;-;MN140082
Lepidoptera;Papilionidae;Papilio;Papilio machaon;Italy;GenBank;-;MN140087
Lepidoptera;Pieridae;Leptidea;Leptidea sinapis;Italy;GenBank;-;MN140088
Lepidoptera;Lycaenidae;Celastrina;Celastrina argiolus;Italy;GenBank;-;MN140125
Lepidoptera ;Hesperiidae;Carcharodus;Carcharodus alceae;Italy;GenBank;-;MN140145
Lepidoptera;Lycaenidae;Polyommatus;Polyommatus icarus;Ireland;GenBank;-;MN140154
Lepidoptera ;Pieridae;Gonepteryx;Gonepteryx cleopatra;Italy;GenBank;-;MN140156
Lepidoptera;Nymphalidae;Vanessa;Vanessa cardui;Italy;GenBank;-;MN140202
Lepidoptera;Pieridae;Pieris;Pieris mannii;Italy;GenBank;-;MN140223
Lepidoptera;Pieridae;Pieris;Pieris napi;United Kingdom;GenBank;-;MN140241
Lepidoptera;Nymphalidae;Lasiommata;Lasiommata paramegaera;Italy;GenBank;-;MN140247
Lepidoptera;Nymphalidae;Lasiommata;Lasiommata megera;Italy;GenBank;-;MN140255
Lepidoptera;Nymphalidae;Arethusana;Arethusana arethusa;Slovenia;GenBank;-;MN140311
Lepidoptera;Lycaenidae;Polyommatus;Polyommatus icarus;Italy;GenBank;-;MN140344
Lepidoptera;Lycaenidae;Celastrina;Celastrina argiolus;Italy;GenBank;-;MN140351
Lepidoptera;Lycaenidae;Aricia;Aricia cramera;Italy;GenBank;-;MN140353
Lepidoptera;Nymphalidae;Aphantopus;Aphantopus hyperantus;United Kingdom;Bold;OXB278-15;MN140418
Lepidoptera;Nymphalidae;Vanessa;Vanessa cardui;France;GenBank;-;MN140444
Lepidoptera;Nymphalidae;Euphydryas;Euphydryas aurinia;United Kingdom;GenBank;-;MN140450
Lepidoptera;Pieridae;Pieris;Pieris napi;United Kingdom;GenBank;-;MN140454
Lepidoptera;Nymphalidae;Melitaea;Melitaea aetherie;Italy;GenBank;-;MN140458
Lepidoptera ;Lycaenidae;Lampides;Lampides boeticus;Italy;GenBank;-;MN140469
Lepidoptera;Nymphalidae;Maniola;Maniola jurtina;United Kingdom;GenBank;-;MN140470
Lepidoptera;Nymphalidae;Hipparchia;Hipparchia aristaeus;Italy;GenBank;-;MN140494
Lepidoptera;Papilionidae;Papilio;Papilio machaon;Italy;GenBank;-;MN140511
Lepidoptera;Pieridae;Pieris;Pieris napi;United Kingdom;GenBank;-;MN140526
Lepidoptera ;Pieridae;Gonepteryx;Gonepteryx cleopatra;Italy;GenBank;-;MN140546
Lepidoptera;Nymphalidae;Maniola;Maniola jurtina;United Kingdom;GenBank;-;MN140554
Lepidoptera;Pieridae;Pieris;Pieris napi;United Kingdom;GenBank;-;MN140561
Lepidoptera;Pieridae;Pieris;Pieris napi;United Kingdom;GenBank;-;MN140592
Lepidoptera;Lycaenidae;Polyommatus;Polyommatus icarus;United Kingdom;GenBank;-;MN140599
Lepidoptera ;Hesperiidae;Gegenes;Gegenes pumilio;Italy;GenBank;-;MN140616
Lepidoptera;Lycaenidae;Polyommatus;Polyommatus icarus;Italy;GenBank;-;MN140633
Lepidoptera ;Pieridae;Gonepteryx;Gonepteryx cleopatra;France;GenBank;-;MN140645
Lepidoptera;Lycaenidae;Callophrys;Callophrys rubi;Italy;GenBank;-;MN140677
Lepidoptera;Nymphalidae;Vanessa;Vanessa cardui;Italy;GenBank;-;MN140696
Lepidoptera;Nymphalidae;Maniola;Maniola jurtina;United Kingdom;GenBank;-;MN140700

Lepidoptera ;Pieridae;Gonepteryx;Gonepteryx rhamni;Italy;GenBank;-;MN140755
Lepidoptera ;Hesperiidae;Gegenes;Gegenes pumilio;Italy;GenBank;-;MN140782
Lepidoptera;Nymphalidae;Erebia;Erebia montana;Italy;GenBank;-;MN140801
Lepidoptera;Nymphalidae;Maniola;Maniola jurtina;United Kingdom;GenBank;-;MN140891
Lepidoptera;Lycaenidae;Polyommatus;Polyommatus icarus;United Kingdom;GenBank;-;MN140897
Lepidoptera;Nymphalidae;Hipparchia;Hipparchia leighebi;Italy;GenBank;-;MN140898
Lepidoptera;Nymphalidae;Maniola;Maniola jurtina;United Kingdom;GenBank;-;MN140914
Lepidoptera;Lycaenidae;Plebejus;Plebejus argus;United Kingdom;GenBank;-;MN140942
Lepidoptera;Papilionidae;Papilio;Papilio machaon;Italy;GenBank;-;MN140953
Lepidoptera;Nymphalidae;Lasiommata;Lasiommata megera;Italy;GenBank;-;MN140977
Lepidoptera;Nymphalidae;Hipparchia;Hipparchia statilinus;Italy;GenBank;-;MN140993
Lepidoptera;Nymphalidae;Hipparchia;Hipparchia leighebi;Italy;GenBank;-;MN141016
Lepidoptera;Nymphalidae;Maniola;Maniola jurtina;United Kingdom;GenBank;-;MN141021
Lepidoptera;Nymphalidae;Aglais;Aglais urticae;France;Bold;OXB1343-15;MN141027
Lepidoptera ;Pieridae;Gonepteryx;Gonepteryx cleopatra;Italy;GenBank;-;MN141085
Lepidoptera;Nymphalidae;Maniola;Maniola jurtina;France;GenBank;-;MN141125
Lepidoptera;Pieridae;Euchloe;Euchloe ausonia;Italy;GenBank;-;MN141144
Lepidoptera;Pieridae;Leptidea;Leptidea reali;Italy;GenBank;-;MN141160
Lepidoptera ;Lycaenidae;Lampides;Lampides boeticus;Italy;GenBank;-;MN141170
Lepidoptera;Nymphalidae;Maniola;Maniola jurtina;United Kingdom;GenBank;-;MN141188
Lepidoptera;Pieridae;Euchloe;Euchloe insularis;Italy;GenBank;-;MN141190
Lepidoptera;Pieridae;Euchloe;Euchloe ausonia;Italy;GenBank;-;MN141201
Lepidoptera;Pieridae;Pieris;Pieris brassicae;France;GenBank;-;MN141206
Lepidoptera ;Hesperiidae;Carcharodus;Carcharodus alceae;France;GenBank;-;MN141216
Lepidoptera;Nymphalidae;Lasiommata;Lasiommata megera;Italy;GenBank;-;MN141272
Lepidoptera;Pieridae;Pontia;Pieris edusa;Italy;GenBank;-;MN141278
Lepidoptera;Nymphalidae;Maniola;Maniola jurtina;United Kingdom;GenBank;-;MN141284
Lepidoptera;Lycaenidae;Polyommatus;Polyommatus icarus;United Kingdom;GenBank;-;MN141288
Lepidoptera;Pieridae;Pieris;Pieris rapae;United Kingdom;GenBank;-;MN141416
Lepidoptera;Nymphalidae;Coenonympha;Coenonympha oedippus;Austria;GenBank;-;MN141430
Lepidoptera;Nymphalidae;Melitaea;Melitaea cinxia;France;GenBank;-;MN141462
Lepidoptera;Lycaenidae;Callophrys;Callophrys rubi;Italy;GenBank;-;MN141469
Lepidoptera;Papilionidae;Papilio;Papilio machaon;Italy;GenBank;-;MN141479
Lepidoptera;Nymphalidae;Maniola;Maniola jurtina;United Kingdom;GenBank;-;MN141496
Lepidoptera;Nymphalidae;Hipparchia;Hipparchia aristaeus;Italy;GenBank;-;MN141498
Lepidoptera;Nymphalidae;Maniola;Maniola jurtina;United Kingdom;GenBank;-;MN141511
Lepidoptera;Nymphalidae;Maniola;Maniola jurtina;United Kingdom;GenBank;-;MN141524
Lepidoptera ;Pieridae;Gonepteryx;Gonepteryx cleopatra;Italy;GenBank;-;MN141556
Lepidoptera;Nymphalidae;Euphydryas;Euphydryas aurinia;United Kingdom;GenBank;-;MN141558
Lepidoptera;Lycaenidae;Polyommatus;Polyommatus icarus;Italy;GenBank;-;MN141565
Lepidoptera;Lycaenidae;Agriades;Albulina optilete;Sweden;GenBank;-;MN141586
Lepidoptera;Nymphalidae;Lasiommata;Lasiommata paramegaera;Italy;GenBank;-;MN141597
Lepidoptera;Pieridae;Pontia;Pieris daplidice;Italy;GenBank;-;MN141621
Lepidoptera;Hesperiidae;Pyrgus;Pyrgus malvoides;Italy;GenBank;-;MN141639
Lepidoptera;Nymphalidae;Vanessa;Vanessa atalanta;France;GenBank;-;MN141664
Lepidoptera;Nymphalidae;Coenonympha;Coenonympha corinna;Italy;GenBank;-;MN141687
Lepidoptera;Pieridae;Pontia;Pieris daplidice;Italy;GenBank;-;MN141705
Lepidoptera;Lycaenidae;Lycaena;Lycaena tityrus;France;GenBank;-;MN141707
Lepidoptera;Nymphalidae;Maniola;Maniola jurtina;France;GenBank;-;MN141721
Lepidoptera;Pieridae;Pieris;Pieris mannii;Italy;GenBank;-;MN141754
Lepidoptera;Nymphalidae;Maniola;Maniola jurtina;United Kingdom;GenBank;-;MN141769
Lepidoptera;Pieridae;Pieris;Pieris brassicae;United Kingdom;GenBank;-;MN141782
Lepidoptera;Pieridae;Pieris;Pieris mannii;Italy;GenBank;-;MN141791
Lepidoptera;Lycaenidae;Lycaena;Lycaena phlaeas;Italy;GenBank;-;MN141811
Lepidoptera;Nymphalidae;Coenonympha;Coenonympha corinna;Italy;GenBank;-;MN141812
Lepidoptera;Nymphalidae;Maniola;Maniola jurtina;United Kingdom;GenBank;-;MN141815
Lepidoptera;Nymphalidae;Maniola;Maniola jurtina;United Kingdom;GenBank;-;MN141819
Lepidoptera;Nymphalidae;Maniola;Maniola jurtina;United Kingdom;GenBank;-;MN141838
Lepidoptera;Nymphalidae;Charaxes;Charaxes jasius;France;GenBank;-;MN141889
Lepidoptera;Pieridae;Pieris;Pieris mannii;Italy;GenBank;-;MN141900
Lepidoptera;Pieridae;Pieris;Pieris brassicae;Italy;GenBank;-;MN141944
Lepidoptera;Nymphalidae;Coenonympha;Coenonympha pamphilus;Italy;GenBank;-;MN141978
Lepidoptera;Nymphalidae;Vanessa;Vanessa atalanta;Italy;GenBank;-;MN141980
Lepidoptera;Nymphalidae;Vanessa;Vanessa atalanta;Italy;GenBank;-;MN142007
Lepidoptera;Nymphalidae;Vanessa;Vanessa cardui;Italy;GenBank;-;MN142014
Lepidoptera;Nymphalidae;Vanessa;Vanessa atalanta;Italy;GenBank;-;MN142042
Lepidoptera;Nymphalidae;Maniola;Maniola jurtina;United Kingdom;GenBank;-;MN142044
Lepidoptera;Nymphalidae;Maniola;Maniola jurtina;United Kingdom;GenBank;-;MN142046
Lepidoptera ;Lycaenidae;Lampides;Lampides boeticus;France;GenBank;-;MN142086
Lepidoptera;Nymphalidae;Boloria;Boloria selene;United Kingdom;GenBank;-;MN142093
Lepidoptera;Lycaenidae;Polyommatus;Polyommatus icarus;France;GenBank;-;MN142118
Lepidoptera;Lycaenidae;Polyommatus;Polyommatus icarus;Italy;GenBank;-;MN142148
Lepidoptera;Lycaenidae;Aricia;Aricia allous;United Kingdom;GenBank;-;MN142161
Lepidoptera;Nymphalidae;Maniola;Maniola jurtina;United Kingdom;GenBank;-;MN142168
Lepidoptera ;Lycaenidae;Lampides;Lampides boeticus;Italy;GenBank;-;MN142211
Lepidoptera;Nymphalidae;Maniola;Maniola jurtina;United Kingdom;GenBank;-;MN142213
Lepidoptera ;Hesperiidae;Gegenes;Gegenes pumilio;Italy;GenBank;-;MN142214
Lepidoptera;Nymphalidae;Vanessa;Vanessa cardui;Italy;GenBank;-;MN142221

Lepidoptera;Pieridae;Pieris;Pieris rapae;Italy;GenBank;-;MN142249
Lepidoptera;Pieridae;Pieris;Pieris rapae;Italy;GenBank;-;MN142252
Lepidoptera;Lycaenidae;Celastrina;Celastrina argiolus;Italy;GenBank;-;MN142255
Lepidoptera ;Hesperiidae;Carcharodus;Carcharodus alceae;France;GenBank;-;MN142277
Lepidoptera ;Pieridae;Gonepteryx;Gonepteryx cleopatra;Italy;GenBank;-;MN142295
Lepidoptera;Lycaenidae;Glaucopsyche;Glaucopsyche melanops;Italy;GenBank;-;MN142304
Lepidoptera;Nymphalidae;Maniola;Maniola jurtina;United Kingdom;GenBank;-;MN142312
Lepidoptera;Nymphalidae;Maniola;Maniola jurtina;United Kingdom;GenBank;-;MN142319
Lepidoptera ;Hesperiidae;Carcharodus;Carcharodus alceae;Italy;GenBank;-;MN142326
Lepidoptera;Pieridae;Pieris;Pieris napi;United Kingdom;GenBank;-;MN142332
Lepidoptera;Nymphalidae;Maniola;Maniola jurtina;United Kingdom;GenBank;-;MN142343
Lepidoptera;Lycaenidae;Polyommatus;Polyommatus icarus;United Kingdom;GenBank;-;MN142379
Lepidoptera;Nymphalidae;Maniola;Maniola jurtina;United Kingdom;GenBank;-;MN142391
Lepidoptera;Pieridae;Pieris;Pieris napi;United Kingdom;GenBank;-;MN142404
Lepidoptera;Lycaenidae;Polyommatus;Polyommatus icarus;Italy;GenBank;-;MN142455
Lepidoptera;Nymphalidae;Hipparchia;Hipparchia neapolitana;Italy;GenBank;-;MN142472
Lepidoptera ;Pieridae;Gonepteryx;Gonepteryx rhamni;United Kingdom;GenBank;-;MN142478
Lepidoptera;Nymphalidae;Maniola;Maniola jurtina;United Kingdom;GenBank;-;MN142491
Lepidoptera;Nymphalidae;Maniola;Maniola jurtina;United Kingdom;GenBank;-;MN142619
Lepidoptera;Nymphalidae;Lasiommata;Lasiommata megera;Italy;GenBank;-;MN142648
Lepidoptera;Lycaenidae;Polyommatus;Polyommatus icarus;United Kingdom;GenBank;-;MN142654
Lepidoptera;Nymphalidae;Maniola;Maniola jurtina;United Kingdom;GenBank;-;MN142656
Lepidoptera ;Lycaenidae;Lampides;Lampides boeticus;Italy;GenBank;-;MN142667
Lepidoptera;Pieridae;Pieris;Pieris napi;United Kingdom;GenBank;-;MN142675
Lepidoptera;Nymphalidae;Pyronia;Pyronia cecilia;Italy;Bold;WMB3034-14;MN142734
Lepidoptera;Nymphalidae;Lasiommata;Lasiommata megera;Italy;GenBank;-;MN142745
Lepidoptera;Nymphalidae;Melitaea;Melitaea nevadensis;Italy;GenBank;-;MN142752
Lepidoptera;Pieridae;Pieris;Pieris rapae;France;GenBank;-;MN142773
Lepidoptera;Nymphalidae;Lasiommata;Lasiommata megera;France;GenBank;-;MN142784
Lepidoptera;Nymphalidae;Lasiommata;Lasiommata megera;Italy;GenBank;-;MN142821
Lepidoptera;Lycaenidae;Polyommatus;Polyommatus icarus;United Kingdom;GenBank;-;MN142826
Lepidoptera;Nymphalidae;Vanessa;Vanessa cardui;Italy;GenBank;-;MN142853
Lepidoptera;Nymphalidae;Pyronia;Pyronia cecilia;Italy;Bold;WMB1551-13;MN142855
Lepidoptera;Papilionidae;Papilio;Papilio machaon;France;GenBank;-;MN142863
Lepidoptera ;Lycaenidae;Lampides;Lampides boeticus;Italy;GenBank;-;MN142889
Lepidoptera;Pieridae;Pieris;Pieris napi;United Kingdom;GenBank;-;MN142901
Lepidoptera;Lycaenidae;Polyommatus;Polyommatus icarus;United Kingdom;GenBank;-;MN142936
Lepidoptera;Hesperiidae;Thymelicus;Thymelicus acteon;Italy;GenBank;-;MN142948
Lepidoptera;Pieridae;Pieris;Pieris rapae;Italy;GenBank;-;MN142949
Lepidoptera;Nymphalidae;Maniola;Maniola jurtina;United Kingdom;GenBank;-;MN142962
Lepidoptera;Pieridae;Pieris;Pieris rapae;Italy;GenBank;-;MN142967
Lepidoptera;Nymphalidae;Maniola;Maniola jurtina;United Kingdom;GenBank;-;MN142974
Lepidoptera;Nymphalidae;Coenonympha;Coenonympha dorus;Italy;GenBank;-;MN143002
Lepidoptera;Nymphalidae;Hipparchia;Hipparchia leighebi;Italy;GenBank;-;MN143025
Lepidoptera;Nymphalidae;Maniola;Maniola jurtina;United Kingdom;GenBank;-;MN143032
Lepidoptera;Nymphalidae;Lasiommata;Lasiommata megera;Italy;GenBank;-;MN143033
Lepidoptera;Lycaenidae;Polyommatus;Polyommatus icarus;United Kingdom;GenBank;-;MN143042
Lepidoptera ;Hesperiidae;Carcharodus;Carcharodus alceae;Italy;GenBank;-;MN143051
Lepidoptera;Pieridae;Pieris;Pieris brassicae;Italy;GenBank;-;MN143059
Lepidoptera;Pieridae;Leptidea;Leptidea reali;Italy;GenBank;-;MN143076
Lepidoptera;Nymphalidae;Maniola;Maniola jurtina;United Kingdom;GenBank;-;MN143079
Lepidoptera;Hesperiidae;Thymelicus;Thymelicus acteon;United Kingdom;GenBank;-;MN143095
Lepidoptera;Lycaenidae;Polyommatus;Polyommatus icarus;United Kingdom;GenBank;-;MN143102
Lepidoptera;Lycaenidae;Polyommatus;Polyommatus icarus;Italy;GenBank;-;MN143107
Lepidoptera;Nymphalidae;Erebia;Erebia mnestra;Austria;GenBank;-;MN143144
Lepidoptera;Lycaenidae;Cupido;Cupido minimus;United Kingdom;GenBank;-;MN143152
Lepidoptera;Nymphalidae;Hipparchia;Hipparchia leighebi;Italy;GenBank;-;MN143170
Lepidoptera;Nymphalidae;Maniola;Maniola jurtina;United Kingdom;GenBank;-;MN143174
Lepidoptera;Nymphalidae;Lasiommata;Lasiommata paramegaera;Italy;GenBank;-;MN143193
Lepidoptera;Nymphalidae;Pyronia;Pyronia cecilia;Italy;Bold;WMB2823-13;MN143221
Lepidoptera;Hesperiidae;Thymelicus;Thymelicus acteon;Italy;GenBank;-;MN143251
Lepidoptera;Papilionidae;Papilio;Papilio machaon;Italy;GenBank;-;MN143257
Lepidoptera;Pieridae;Pieris;Pieris mannii;Italy;GenBank;-;MN143263
Lepidoptera;Nymphalidae;Pyronia;Pyronia cecilia;Italy;Bold;WMB911-13;MN143302
Lepidoptera;Nymphalidae;Hipparchia;Hipparchia semele;United Kingdom;GenBank;-;MN143307
Lepidoptera;Pieridae;Pieris;Pieris mannii;Italy;GenBank;-;MN143331
Lepidoptera;Pieridae;Pieris;Pieris rapae;Italy;GenBank;-;MN143343
Lepidoptera ;Lycaenidae;Leptotes;Leptotes pirithous;Italy;GenBank;-;MN143373
Lepidoptera;Pieridae;Euchloe;Euchloe ausonia;Italy;GenBank;-;MN143388
Lepidoptera ;Lycaenidae;Lampides;Lampides boeticus;France;GenBank;-;MN143409
Lepidoptera;Nymphalidae;Hipparchia;Hipparchia fagi;Italy;GenBank;-;MN143411
Lepidoptera;Pieridae;Leptidea;Leptidea sinapis;Slovenia;GenBank;-;MN143420
Lepidoptera;Nymphalidae;Hipparchia;Hipparchia fagi;Italy;GenBank;-;MN143432
Lepidoptera;Lycaenidae;Polyommatus;Polyommatus icarus;Italy;GenBank;-;MN143452
Lepidoptera;Nymphalidae;Maniola;Maniola jurtina;United Kingdom;GenBank;-;MN143494
Lepidoptera;Lycaenidae;Polyommatus;Polyommatus icarus;United Kingdom;GenBank;-;MN143538
Lepidoptera;Nymphalidae;Lasiommata;Lasiommata megera;France;GenBank;-;MN143547
Lepidoptera;Hesperiidae;Ochlodes;Ochlodes sylvanus;Italy;GenBank;-;MN143550

Lepidoptera;Nymphalidae;Lasiommata;Lasiommata megera;Italy;GenBank;-;MN143554
Lepidoptera;Nymphalidae;Coenonympha;Coenonympha corinna;Italy;GenBank;-;MN143566
Lepidoptera;Pieridae;Leptidea;Leptidea reali;Italy;GenBank;-;MN143589
Lepidoptera;Lycaenidae;Polyommatus;Polyommatus icarus;United Kingdom;GenBank;-;MN143601
Lepidoptera;Pieridae;Pontia;Pieris daplidice;Italy;GenBank;-;MN143616
Lepidoptera;Nymphalidae;Lasiommata;Lasiommata paramegaera;Italy;GenBank;-;MN143629
Lepidoptera;Nymphalidae;Maniola;Maniola jurtina;United Kingdom;GenBank;-;MN143666
Lepidoptera;Nymphalidae;Lasiommata;Lasiommata paramegaera;Italy;GenBank;-;MN143678
Lepidoptera ;Nymphalidae;Issoria;Issoria lathonia;Italy;GenBank;-;MN143704
Lepidoptera;Nymphalidae;Erebia;Erebia mnestra;Austria;GenBank;-;MN143707
Lepidoptera ;Hesperiidae;Carcharodus;Carcharodus alceae;Austria;GenBank;-;MN143736
Lepidoptera;Nymphalidae;Maniola;Maniola jurtina;United Kingdom;GenBank;-;MN143757
Lepidoptera;Pieridae;Pieris;Pieris rapae;Italy;GenBank;-;MN143758
Lepidoptera;Nymphalidae;Coenonympha;Coenonympha corinna;Italy;GenBank;-;MN143776
Lepidoptera;Nymphalidae;Maniola;Maniola jurtina;United Kingdom;GenBank;-;MN143796
Lepidoptera;Pieridae;Pieris;Pieris rapae;Italy;GenBank;-;MN143823
Lepidoptera;Nymphalidae;Erebia;Erebia aethiops;United Kingdom;GenBank;-;MN143838
Lepidoptera ;Lycaenidae;Leptotes;Leptotes pirithous;France;GenBank;-;MN143866
Lepidoptera ;Pieridae;Gonepteryx;Gonepteryx cleopatra;Italy;GenBank;-;MN143883
Lepidoptera ;Lycaenidae;Lampides;Lampides boeticus;Portugal;GenBank;-;MN143892
Lepidoptera;Nymphalidae;Hipparchia;Hipparchia statilinus;Italy;GenBank;-;MN143906
Lepidoptera;Pieridae;Pieris;Pieris napi;United Kingdom;GenBank;-;MN143928
Lepidoptera ;Lycaenidae;Lampides;Lampides boeticus;Italy;GenBank;-;MN143967
Lepidoptera;Lycaenidae;Polyommatus;Polyommatus icarus;Italy;GenBank;-;MN143970
Lepidoptera;Hesperiidae;Thymelicus;Thymelicus acteon;United Kingdom;GenBank;-;MN143979
Lepidoptera;Nymphalidae;Maniola;Maniola jurtina;United Kingdom;GenBank;-;MN143989
Lepidoptera;Pieridae;Leptidea;Leptidea reali;Italy;GenBank;-;MN143990
Lepidoptera;Pieridae;Pieris;Pieris rapae;Italy;GenBank;-;MN143992
Lepidoptera;Nymphalidae;Lasiommata;Lasiommata megera;Italy;GenBank;-;MN143997
Lepidoptera;Nymphalidae;Melanargia;Melanargia galathea;United Kingdom;GenBank;-;MN144012
Lepidoptera;Nymphalidae;Lasiommata;Lasiommata megera;Italy;GenBank;-;MN144013
Lepidoptera;Papilionidae;Papilio;Papilio machaon;Italy;GenBank;-;MN144041
Lepidoptera;Nymphalidae;Melitaea;Melitaea nevadensis;Italy;GenBank;-;MN144053
Lepidoptera;Pieridae;Euchloe;Euchloe insularis;Italy;GenBank;-;MN144070
Lepidoptera;Lycaenidae;Aricia;Aricia agestis;Italy;GenBank;-;MN144078
Lepidoptera;Lycaenidae;Polyommatus;Polyommatus icarus;United Kingdom;GenBank;-;MN144093
Lepidoptera;Lycaenidae;Polyommatus;Polyommatus icarus;United Kingdom;GenBank;-;MN144095
Lepidoptera;Pieridae;Leptidea;Leptidea sinapis;Italy;GenBank;-;MN144105
Lepidoptera;Pieridae;Euchloe;Euchloe insularis;Italy;GenBank;-;MN144108
Lepidoptera;Lycaenidae;Celastrina;Celastrina argiolus;Italy;GenBank;-;MN144129
Lepidoptera;Hesperiidae;Thymelicus;Thymelicus acteon;Italy;GenBank;-;MN144138
Lepidoptera;Lycaenidae;Polyommatus;Polyommatus icarus;United Kingdom;GenBank;-;MN144141
Lepidoptera;Nymphalidae;Maniola;Maniola jurtina;United Kingdom;GenBank;-;MN144164
Lepidoptera;Nymphalidae;Hipparchia;Hipparchia semele;United Kingdom;GenBank;-;MN144166
Lepidoptera;Nymphalidae;Hipparchia;Hipparchia leighebi;Italy;GenBank;-;MN144175
Lepidoptera;Nymphalidae;Maniola;Maniola jurtina;United Kingdom;GenBank;-;MN144188
Lepidoptera;Hesperiidae;Thymelicus;Thymelicus acteon;Italy;GenBank;-;MN144206
Lepidoptera;Nymphalidae;Melitaea;Melitaea didyma;Italy;GenBank;-;MN144220
Lepidoptera;Nymphalidae;Hipparchia;Hipparchia leighebi;Italy;GenBank;-;MN144251
Lepidoptera;Lycaenidae;Plebejus;Plebejus argus;United Kingdom;GenBank;-;MN144252
Lepidoptera;Nymphalidae;Lasiommata;Lasiommata megera;Italy;GenBank;-;MN144258
Lepidoptera;Nymphalidae;Lasiommata;Lasiommata megera;Sweden;GenBank;-;MN144260
Lepidoptera ;Pieridae;Gonepteryx;Gonepteryx cleopatra;Italy;GenBank;-;MN144285
Lepidoptera ;Hesperiidae;Gegenes;Gegenes nostrodamus;Italy;GenBank;-;MN144291
Lepidoptera;Nymphalidae;Hipparchia;Hipparchia semele;United Kingdom;GenBank;-;MN144294
Lepidoptera;Pieridae;Aporia;Aporia crataegi;Italy;GenBank;-;MN144305
Lepidoptera;Nymphalidae;Maniola;Maniola jurtina;United Kingdom;GenBank;-;MN144310
Lepidoptera;Lycaenidae;Polyommatus;Polyommatus icarus;United Kingdom;GenBank;-;MN144322
Lepidoptera ;Hesperiidae;Carcharodus;Carcharodus baeticus;Italy;Bold;EULEP5527-17;MN144356
Lepidoptera;Lycaenidae;Polyommatus;Polyommatus icarus;United Kingdom;GenBank;-;MN144398
Lepidoptera;Pieridae;Pieris;Pieris mannii;Italy;GenBank;-;MN144408
Lepidoptera ;Nymphalidae;Brintesia;Kanetisa circe;France;GenBank;-;MN144413
Lepidoptera;Nymphalidae;Vanessa;Vanessa cardui;Italy;GenBank;-;MN144429
Lepidoptera;Nymphalidae;Maniola;Maniola jurtina;United Kingdom;GenBank;-;MN144437
Lepidoptera;Nymphalidae;Coenonympha;Coenonympha corinna;Italy;GenBank;-;MN144470
Lepidoptera ;Hesperiidae;Gegenes;Gegenes pumilio;Italy;GenBank;-;MN144503
Lepidoptera;Nymphalidae;Maniola;Maniola jurtina;United Kingdom;GenBank;-;MN144628
Lepidoptera;Nymphalidae;Maniola;Maniola jurtina;United Kingdom;GenBank;-;MN144630
Lepidoptera;Pieridae;Pieris;Pieris brassicae;Italy;GenBank;-;MN144663
Lepidoptera;Hesperiidae;Ochlodes;Ochlodes sylvanus;France;GenBank;-;MN144667
Lepidoptera ;Hesperiidae;Gegenes;Gegenes pumilio;Italy;GenBank;-;MN144675
Lepidoptera;Lycaenidae;Lycaena;Lycaena tityrus;Italy;GenBank;-;MN144700
Lepidoptera;Nymphalidae;Lasiommata;Lasiommata megera;Italy;GenBank;-;MN144715
Lepidoptera;Lycaenidae;Polyommatus;Polyommatus icarus;United Kingdom;GenBank;-;MN144720
Lepidoptera;Papilionidae;Papilio;Papilio machaon;Italy;GenBank;-;MN144727
Lepidoptera;Hesperiidae;Thymelicus;Thymelicus sylvestris;Germany;GenBank;-;MN144747
Lepidoptera;Nymphalidae;Maniola;Maniola jurtina;United Kingdom;GenBank;-;MN144780
Lepidoptera;Nymphalidae;Maniola;Maniola jurtina;United Kingdom;GenBank;-;MN144797

Lepidoptera;Nymphalidae;Melanargia;Melanargia occitanica;Italy;GenBank;-;MN144812
Lepidoptera;Nymphalidae;Maniola;Maniola jurtina;United Kingdom;GenBank;-;MN144820
Lepidoptera ;Lycaenidae;Lampides;Lampides boeticus;Italy;GenBank;-;MN144854
Lepidoptera;Nymphalidae;Coenonympha;Coenonympha corinna;Italy;GenBank;-;MN144861
Lepidoptera;Lycaenidae;Polyommatus;Polyommatus icarus;United Kingdom;GenBank;-;MN144884
Lepidoptera;Nymphalidae;Pyronia;Pyronia cecilia;Italy;Bold;WMB2822-13;MN144885
Lepidoptera;Pieridae;Pieris;Pieris napi;United Kingdom;GenBank;-;MN144887
Lepidoptera;Nymphalidae;Lasiommata;Lasiommata megera;Italy;GenBank;-;MN144946
Lepidoptera;Nymphalidae;Lasiommata;Lasiommata megera;Italy;GenBank;-;MN144997
Lepidoptera;Nymphalidae;Vanessa;Vanessa atalanta;Italy;GenBank;-;MN144998
Lepidoptera;Nymphalidae;Pyronia;Pyronia cecilia;Italy;Bold;WMB1546-13;MN145003
Lepidoptera;Nymphalidae;Pyronia;Pyronia cecilia;Italy;Bold;WMB3033-14;MN145004
Lepidoptera;Nymphalidae;Coenonympha;Coenonympha corinna;Italy;GenBank;-;MN145022
Lepidoptera;Lycaenidae;Callophrys;Callophrys rubi;Spain;GenBank;-;MN145080
Lepidoptera ;Lycaenidae;Leptotes;Leptotes pirithous;France;GenBank;-;MN145084
Lepidoptera;Nymphalidae;Hipparchia;Hipparchia semele;United Kingdom;GenBank;-;MN145089
Lepidoptera;Nymphalidae;Pyronia;Pyronia tithonus;France;Bold;BIBSA1244-15;MN145103
Lepidoptera;Lycaenidae;Satyrium;Satyrium ilicis;France;GenBank;-;MN145111
Lepidoptera;Nymphalidae;Lasiommata;Lasiommata megera;France;GenBank;-;MN145125
Lepidoptera;Nymphalidae;Maniola;Maniola jurtina;United Kingdom;GenBank;-;MN145133
Lepidoptera;Nymphalidae;Pyronia;Pyronia cecilia;Italy;Bold;WMB4600-14;MN145135
Lepidoptera;Nymphalidae;Hipparchia;Hipparchia sbordonii;Italy;GenBank;-;MN145157
Lepidoptera;Lycaenidae;Polyommatus;Polyommatus icarus;United Kingdom;GenBank;-;MN145164
Lepidoptera;Hesperiidae;Thymelicus;Thymelicus acteon;Italy;GenBank;-;MN145177
Lepidoptera;Nymphalidae;Maniola;Maniola jurtina;United Kingdom;GenBank;-;MN145187
Lepidoptera;Nymphalidae;Vanessa;Vanessa cardui;Italy;GenBank;-;MN145198
Lepidoptera;Nymphalidae;Argynnis;Argynnis paphia;United Kingdom;GenBank;-;MN145209
Lepidoptera;Lycaenidae;Lycaena;Lycaena tityrus;Italy;GenBank;-;MN145214
Lepidoptera;Lycaenidae;Polyommatus;Polyommatus icarus;United Kingdom;GenBank;-;MN145225
Lepidoptera;Pieridae;Pieris;Pieris napi;United Kingdom;GenBank;-;MN145235
Lepidoptera;Pieridae;Pieris;Pieris napi;United Kingdom;GenBank;-;MN145237
Lepidoptera;Lycaenidae;Polyommatus;Polyommatus icarus;United Kingdom;GenBank;-;MN145255
Lepidoptera;Hesperiidae;Thymelicus;Thymelicus acteon;Italy;GenBank;-;MN145292
Lepidoptera;Lycaenidae;Polyommatus;Polyommatus icarus;United Kingdom;GenBank;-;MN145293
Lepidoptera;Pieridae;Pieris;Pieris brassicae;Italy;GenBank;-;MN145300
Lepidoptera;Lycaenidae;Aricia;Aricia agestis;France;GenBank;-;MN145302
Lepidoptera;Papilionidae;Papilio;Papilio machaon;Italy;GenBank;-;MN145307
Lepidoptera ;Pieridae;Gonepteryx;Gonepteryx cleopatra;Italy;GenBank;-;MN145321
Lepidoptera;Pieridae;Pontia;Pieris edusa;Italy;GenBank;-;MN145325
Lepidoptera;Papilionidae;Papilio;Papilio machaon;Italy;GenBank;-;MN145339
Lepidoptera;Lycaenidae;Celastrina;Celastrina argiolus;France;GenBank;-;MN145342
Lepidoptera;Nymphalidae;Maniola;Maniola jurtina;United Kingdom;GenBank;-;MN145349
Lepidoptera;Pieridae;Leptidea;Leptidea sinapis;France;GenBank;-;MN145352
Lepidoptera;Nymphalidae;Maniola;Maniola jurtina;United Kingdom;GenBank;-;MN145357
Lepidoptera;Pieridae;Pieris;Pieris rapae;Italy;GenBank;-;MN145418
Lepidoptera;Nymphalidae;Hipparchia;Hipparchia leighebi;Italy;GenBank;-;MN145441
Lepidoptera;Lycaenidae;Glaucopsyche;Glaucopsyche alexis;Italy;GenBank;-;MN145448
Lepidoptera;Nymphalidae;Vanessa;Vanessa cardui;Italy;GenBank;-;MN145450
Lepidoptera;Pieridae;Pieris;Pieris brassicae;United Kingdom;GenBank;-;MN181609
Lepidoptera;Pieridae;Pieris;Pieris brassicae;United Kingdom;GenBank;-;MN181610
Lepidoptera;Pieridae;Pieris;Pieris brassicae;United Kingdom;GenBank;-;MN181611
Lepidoptera;Pieridae;Pieris;Pieris rapae;Finland;GenBank;-;MN181894
Lepidoptera;Pieridae;Pieris;Pieris rapae;Finland;GenBank;-;MN181895
Lepidoptera;Pieridae;Pieris;Pieris rapae;Finland;GenBank;-;MN181896
Lepidoptera;Pieridae;Pieris;Pieris rapae;Finland;GenBank;-;MN181898
Lepidoptera;Pieridae;Pieris;Pieris rapae;Finland;GenBank;-;MN182192
Lepidoptera;Pieridae;Pieris;Pieris rapae;Russia;GenBank;-;MN182195
Lepidoptera;Pieridae;Pieris;Pieris rapae;Russia;GenBank;-;MN182196
Lepidoptera;Pieridae;Pieris;Pieris rapae;Russia;GenBank;-;MN182197
Lepidoptera;Pieridae;Pieris;Pieris rapae;Spain;GenBank;-;MN182207
Lepidoptera;Pieridae;Pieris;Pieris rapae;Italy;GenBank;-;MN182219
Lepidoptera;Pieridae;Pieris;Pieris rapae;United Kingdom;GenBank;-;MN182295
Lepidoptera;Lycaenidae;Polyommatus;Polyommatus icarus;United Kingdom;GenBank;-;MT151338
Lepidoptera;Lycaenidae;Polyommatus;Polyommatus icarus;United Kingdom;GenBank;-;MT151339
Lepidoptera;Lycaenidae;Polyommatus;Polyommatus icarus;United Kingdom;GenBank;-;MT151340
Lepidoptera;Lycaenidae;Polyommatus;Polyommatus icarus;United Kingdom;GenBank;-;MT151341
Lepidoptera;Lycaenidae;Polyommatus;Polyommatus icarus;United Kingdom;GenBank;-;MT151342
Lepidoptera;Lycaenidae;Polyommatus;Polyommatus icarus;United Kingdom;GenBank;-;MT151343
Lepidoptera;Lycaenidae;Polyommatus;Polyommatus icarus;United Kingdom;GenBank;-;MT151344
Lepidoptera;Lycaenidae;Polyommatus;Polyommatus icarus;United Kingdom;GenBank;-;MT151345
Lepidoptera;Lycaenidae;Polyommatus;Polyommatus icarus;United Kingdom;GenBank;-;MT151347
Lepidoptera;Lycaenidae;Polyommatus;Polyommatus icarus;United Kingdom;GenBank;-;MT151348
Lepidoptera;Lycaenidae;Polyommatus;Polyommatus icarus;United Kingdom;GenBank;-;MT151349
Lepidoptera;Lycaenidae;Polyommatus;Polyommatus icarus;United Kingdom;GenBank;-;MT151350
Lepidoptera;Lycaenidae;Polyommatus;Polyommatus icarus;United Kingdom;GenBank;-;MT151351
Lepidoptera;Lycaenidae;Polyommatus;Polyommatus icarus;United Kingdom;GenBank;-;MT151354
Lepidoptera;Lycaenidae;Polyommatus;Polyommatus icarus;United Kingdom;GenBank;-;MT151355
Lepidoptera;Lycaenidae;Polyommatus;Polyommatus icarus;United Kingdom;GenBank;-;MT151356

Lepidoptera;Lycaenidae;Polyommatus;Polyommatus icarus;United Kingdom;GenBank;-;MT151361
Lepidoptera;Lycaenidae;Polyommatus;Polyommatus icarus;United Kingdom;GenBank;-;MT151362
Lepidoptera;Lycaenidae;Polyommatus;Polyommatus icarus;United Kingdom;GenBank;-;MT151363
Lepidoptera;Lycaenidae;Polyommatus;Polyommatus icarus;United Kingdom;GenBank;-;MT151364
Lepidoptera;Lycaenidae;Polyommatus;Polyommatus icarus;United Kingdom;GenBank;-;MT151365
Lepidoptera;Nymphalidae;Hyponephele;Hyponephele lupina;Russia;Bold;EULEP4925-16;MT260571
Lepidoptera;Nymphalidae;Lasiommata;Lasiommata megera;Greece;GenBank;-;MT260729
Lepidoptera;Nymphalidae;Lasiommata;Lasiommata maera;Sweden;GenBank;-;MT260735
Lepidoptera;Nymphalidae;Lasiommata;Lasiommata megera;Russia;GenBank;-;MT260741
Lepidoptera;Nymphalidae;Lasiommata;Lasiommata maera;Russia;GenBank;-;MT260760
Lepidoptera;Nymphalidae;Lasiommata;Lasiommata megera;Russia;GenBank;-;MT260782
Lepidoptera;Nymphalidae;Lasiommata;Lasiommata megera;Croatia;GenBank;-;MT260786
Lepidoptera;Nymphalidae;Lasiommata;Lasiommata megera;Croatia;GenBank;-;MT260799
Lepidoptera;Pieridae;Pontia;Pieris edusa;Denmark;GenBank;-;MT755954
Lepidoptera;Lycaenidae;Aricia;Aricia agestis;Greece;GenBank;-;MT883881
Lepidoptera;Lycaenidae;Aricia;Aricia agestis;Greece;GenBank;-;MT883882
Lepidoptera;Lycaenidae;Aricia;Aricia allous;Russia;GenBank;-;MT883901
Lepidoptera;Lycaenidae;Aricia;Aricia allous;Russia;GenBank;-;MT883921
Lepidoptera;Lycaenidae;Aricia;Aricia allous;Russia;GenBank;-;MT883930
Lepidoptera;Nymphalidae;Boloria;Boloria napaea;Sweden;GenBank;-;MT916606
Lepidoptera;Nymphalidae;Boloria;Boloria napaea;Sweden;GenBank;-;MT916607
Lepidoptera;Nymphalidae;Boloria;Boloria napaea;Sweden;GenBank;-;MT916615
Lepidoptera;Hesperiidae;Carterocephalus;Carterocephalus palaemon;Russia;GenBank;-;MW115573
Lepidoptera;Nymphalidae;Satyrus;Satyrus ferula;Russia;GenBank;-;MW378057
Lepidoptera;Nymphalidae;Satyrus;Satyrus ferula;Russia;GenBank;-;MW378062
Lepidoptera;Nymphalidae;Satyrus;Satyrus ferula;Russia;GenBank;-;MW378122
Lepidoptera;Lycaenidae;Iolana;Iolana iolas;Macedonia;GenBank;-;MW378130
Lepidoptera;Lycaenidae;Polyommatus;Polyommatus icarus;United Kingdom;GenBank;-;MW394241
Lepidoptera;Lycaenidae;Polyommatus;Polyommatus icarus;United Kingdom;GenBank;-;MW394242
Lepidoptera;Lycaenidae;Polyommatus;Polyommatus icarus;United Kingdom;GenBank;-;MW394243
Lepidoptera;Lycaenidae;Polyommatus;Polyommatus icarus;United Kingdom;GenBank;-;MW394244
Lepidoptera;Lycaenidae;Polyommatus;Polyommatus icarus;United Kingdom;GenBank;-;MW394259
Lepidoptera;Lycaenidae;Polyommatus;Polyommatus icarus;United Kingdom;GenBank;-;MW394273
Lepidoptera;Lycaenidae;Polyommatus;Polyommatus icarus;United Kingdom;GenBank;-;MW394274
Lepidoptera;Lycaenidae;Polyommatus;Polyommatus icarus;United Kingdom;GenBank;-;MW394275
Lepidoptera;Lycaenidae;Polyommatus;Polyommatus icarus;United Kingdom;GenBank;-;MW394276
Lepidoptera;Lycaenidae;Polyommatus;Polyommatus icarus;United Kingdom;GenBank;-;MW394277
Lepidoptera;Lycaenidae;Polyommatus;Polyommatus icarus;United Kingdom;GenBank;-;MW394278
Lepidoptera;Lycaenidae;Polyommatus;Polyommatus icarus;United Kingdom;GenBank;-;MW394279
Lepidoptera;Lycaenidae;Polyommatus;Polyommatus icarus;United Kingdom;GenBank;-;MW394280
Lepidoptera;Lycaenidae;Polyommatus;Polyommatus icarus;United Kingdom;GenBank;-;MW394281
Lepidoptera;Lycaenidae;Polyommatus;Polyommatus icarus;United Kingdom;GenBank;-;MW394282
Lepidoptera;Lycaenidae;Polyommatus;Polyommatus icarus;United Kingdom;GenBank;-;MW394283
Lepidoptera;Lycaenidae;Polyommatus;Polyommatus icarus;United Kingdom;GenBank;-;MW394297
Lepidoptera;Lycaenidae;Polyommatus;Polyommatus icarus;United Kingdom;GenBank;-;MW394298
Lepidoptera;Lycaenidae;Polyommatus;Polyommatus icarus;United Kingdom;GenBank;-;MW394299
Lepidoptera;Lycaenidae;Polyommatus;Polyommatus icarus;United Kingdom;GenBank;-;MW394300
Lepidoptera;Lycaenidae;Polyommatus;Polyommatus icarus;United Kingdom;GenBank;-;MW394301
Lepidoptera;Lycaenidae;Polyommatus;Polyommatus icarus;United Kingdom;GenBank;-;MW394302
Lepidoptera;Lycaenidae;Polyommatus;Polyommatus icarus;United Kingdom;GenBank;-;MW394316
Lepidoptera;Lycaenidae;Polyommatus;Polyommatus icarus;United Kingdom;GenBank;-;MW394317
Lepidoptera;Lycaenidae;Polyommatus;Polyommatus icarus;United Kingdom;GenBank;-;MW394318
Lepidoptera;Lycaenidae;Polyommatus;Polyommatus icarus;United Kingdom;GenBank;-;MW394319
Lepidoptera;Lycaenidae;Polyommatus;Polyommatus icarus;United Kingdom;GenBank;-;MW394320
Lepidoptera;Lycaenidae;Polyommatus;Polyommatus icarus;United Kingdom;GenBank;-;MW394321
Lepidoptera;Lycaenidae;Polyommatus;Polyommatus icarus;United Kingdom;GenBank;-;MW394322
Lepidoptera;Lycaenidae;Polyommatus;Polyommatus icarus;United Kingdom;GenBank;-;MW394323
Lepidoptera;Lycaenidae;Polyommatus;Polyommatus icarus;United Kingdom;GenBank;-;MW394324
Lepidoptera;Lycaenidae;Polyommatus;Polyommatus icarus;United Kingdom;GenBank;-;MW394325
Lepidoptera;Lycaenidae;Polyommatus;Polyommatus icarus;United Kingdom;GenBank;-;MW394326
Lepidoptera;Lycaenidae;Polyommatus;Polyommatus icarus;United Kingdom;GenBank;-;MW394327
Lepidoptera;Lycaenidae;Polyommatus;Polyommatus icarus;United Kingdom;GenBank;-;MW394328
Lepidoptera;Lycaenidae;Polyommatus;Polyommatus icarus;United Kingdom;GenBank;-;MW394329
Lepidoptera;Lycaenidae;Polyommatus;Polyommatus icarus;United Kingdom;GenBank;-;MW394330
Lepidoptera;Pieridae;Pieris;Pieris brassicae;Belarus;GenBank;-;MW498992
Lepidoptera;Pieridae;Leptidea;Leptidea juvernica;Russia;GenBank;-;MW498996
Lepidoptera ;Pieridae;Gonepteryx;Gonepteryx rhamni;Belarus;GenBank;-;MW499009
Lepidoptera;Nymphalidae;Vanessa;Vanessa atalanta;Estonia;GenBank;-;MW499034
Lepidoptera;Lycaenidae;Lycaena;Lycaena hippothoe;Norway;GenBank;-;MW499072
Lepidoptera;Nymphalidae;Erebia;Erebia manto;Ukraine;GenBank;-;MW499121
Lepidoptera;Nymphalidae;Boloria;Boloria thore;Sweden;GenBank;-;MW499144
Lepidoptera;Nymphalidae;Maniola;Maniola jurtina;United Kingdom;GenBank;-;MW499308
Lepidoptera;Nymphalidae;Apatura;Apatura ilia;Belarus;GenBank;-;MW499340
Lepidoptera;Lycaenidae;Cyaniris;Cyaniris semiargus;Sweden;GenBank;-;MW499391
Lepidoptera;Hesperiidae;Ochlodes;Ochlodes sylvanus;Lithuania;GenBank;-;MW499424
Lepidoptera;Nymphalidae;Boloria;Boloria napaea;Sweden;GenBank;-;MW499545
Lepidoptera;Pieridae;Aporia;Aporia crataegi;Belarus;GenBank;-;MW499607
Lepidoptera;Pieridae;Pontia;Pieris edusa;Ukraine;GenBank;-;MW499628

Lepidoptera;Pieridae;Pieris;Pieris rapae;Italy;GenBank;-;MW499669
Lepidoptera;Nymphalidae;Coenonympha;Coenonympha pamphilus;Greece;GenBank;-;MW499715
Lepidoptera;Nymphalidae;Hipparchia;Hipparchia semele;United Kingdom;GenBank;-;MW499729
Lepidoptera;Pieridae;Leptidea;Leptidea juvernica;Russia;GenBank;-;MW499739
Lepidoptera;Lycaenidae;Phengaris;Maculinea teleius;Ukraine;GenBank;-;MW499743
Lepidoptera;Nymphalidae;Erebia;Erebia gorge;Macedonia;GenBank;-;MW499770
Lepidoptera;Nymphalidae;Argynnis;Argynnis paphia;Sweden;GenBank;-;MW499779
Lepidoptera;Nymphalidae;Hipparchia;Hipparchia semele;United Kingdom;GenBank;-;MW499799
Lepidoptera;Lycaenidae;Phengaris;Maculinea teleius;Switzerland;GenBank;-;MW499832
Lepidoptera;Nymphalidae;Erebia;Erebia mnestra;Switzerland;GenBank;-;MW499855
Lepidoptera;Nymphalidae;Neptis;Neptis rivularis;Ukraine;GenBank;-;MW499954
Lepidoptera;Nymphalidae;Coenonympha;Coenonympha glycerion;Finland;GenBank;-;MW500143
Lepidoptera;Nymphalidae;Hipparchia;Hipparchia aristaeus;Italy;GenBank;-;MW500177
Lepidoptera;Lycaenidae;Cupido;Cupido minimus;Norway;GenBank;-;MW500261
Lepidoptera;Nymphalidae;Boloria;Boloria pales;Macedonia;GenBank;-;MW500301
Lepidoptera;Pieridae;Pieris;Pieris napi;Belarus;GenBank;-;MW500303
Lepidoptera;Nymphalidae;Erebia;Erebia euryale;Russia;GenBank;-;MW500322
Lepidoptera;Nymphalidae;Hipparchia;Hipparchia semele;United Kingdom;GenBank;-;MW500339
Lepidoptera;Nymphalidae;Hipparchia;Hipparchia statilinus;Italy;GenBank;-;MW500377
Lepidoptera;Lycaenidae;Aricia;Aricia nicias;Sweden;GenBank;-;MW500445
Lepidoptera;Pieridae;Pieris;Pieris mannii;Liechtenstein;GenBank;-;MW500532
Lepidoptera;Nymphalidae;Erebia;Erebia gorge;Macedonia;GenBank;-;MW500558
Lepidoptera;Nymphalidae;Fabriciana;Argynnis adippe;Belarus;GenBank;-;MW500581
Lepidoptera;Pieridae;Leptidea;Leptidea juvernica;Russia;GenBank;-;MW500629
Lepidoptera;Pieridae;Colias;Colias alfacariensis;Serbia;Bold;EULEP537-15;MW500742
Lepidoptera;Nymphalidae;Charaxes;Charaxes jasius;Croatia;GenBank;-;MW500862
Lepidoptera;Nymphalidae;Boloria;Boloria pales;Macedonia;GenBank;-;MW500879
Lepidoptera;Nymphalidae;Boloria;Boloria thore;Norway;GenBank;-;MW500887
Lepidoptera;Nymphalidae;Maniola;Maniola jurtina;United Kingdom;GenBank;-;MW500900
Lepidoptera;Pieridae;Leptidea;Leptidea juvernica;Russia;GenBank;-;MW500931
Lepidoptera ;Nymphalidae;Pararge;Pararge aegeria;Lithuania;GenBank;-;MW500933
Lepidoptera;Nymphalidae;Vanessa;Vanessa cardui;Belarus;GenBank;-;MW500944
Lepidoptera;Nymphalidae;Coenonympha;Coenonympha rhodopensis;Italy;GenBank;-;MW501003
Lepidoptera;Nymphalidae;Vanessa;Vanessa cardui;Italy;GenBank;-;MW501060
Lepidoptera;Nymphalidae;Coenonympha;Coenonympha dorus;Italy;GenBank;-;MW501065
Lepidoptera;Hesperiidae;Pyrgus;Pyrgus malvoides;Italy;GenBank;-;MW501068
Lepidoptera;Nymphalidae;Coenonympha;Coenonympha pamphilus;Belarus;GenBank;-;MW501109
Lepidoptera;Lycaenidae;Polyommatus;Polyommatus icarus;Liechtenstein;GenBank;-;MW501110
Lepidoptera;Nymphalidae;Boloria;Boloria thore;Sweden;GenBank;-;MW501135
Lepidoptera;Papilionidae;Papilio;Papilio machaon;Greece;GenBank;-;MW501166
Lepidoptera;Nymphalidae;Maniola;Maniola jurtina;United Kingdom;GenBank;-;MW501221
Lepidoptera;Nymphalidae;Aphantopus;Aphantopus hyperantus;Belarus;Bold;EULEP3436-16;MW501239
Lepidoptera;Papilionidae;Papilio;Papilio machaon;Greece;GenBank;-;MW501266
Lepidoptera;Nymphalidae;Coenonympha;Coenonympha corinna;Italy;GenBank;-;MW501269
Lepidoptera;Nymphalidae;Hipparchia;Hipparchia leighebi;Italy;GenBank;-;MW501350
Lepidoptera;Nymphalidae;Erebia;Erebia euryale;Russia;GenBank;-;MW501387
Lepidoptera;Nymphalidae;Maniola;Maniola jurtina;United Kingdom;GenBank;-;MW501398
Lepidoptera;Nymphalidae;Vanessa;Vanessa atalanta;Latvia;GenBank;-;MW501460
Lepidoptera;Nymphalidae;Hipparchia;Hipparchia semele;United Kingdom;GenBank;-;MW501471
Lepidoptera;Pieridae;Colias;Colias hyale;Belarus;GenBank;-;MW501544
Lepidoptera;Lycaenidae;Lycaena;Lycaena alciphron;Belarus;GenBank;-;MW501565
Lepidoptera;Lycaenidae;Lycaena;Lycaena alciphron;Latvia;GenBank;-;MW501579
Lepidoptera;Lycaenidae;Lycaena;Lycaena dispar;Estonia;GenBank;-;MW501590
Lepidoptera;Nymphalidae;Coenonympha;Coenonympha oedippus;Liechtenstein;GenBank;-;MW501631
Lepidoptera;Papilionidae;Papilio;Papilio machaon;Belarus;GenBank;-;MW501635
Lepidoptera;Nymphalidae;Boloria;Boloria thore;Norway;GenBank;-;MW501656
Lepidoptera;Lycaenidae;Agriades;Albulina orbitulus;Sweden;GenBank;-;MW501709
Lepidoptera;Nymphalidae;Minois;Minois dryas;Switzerland;GenBank;-;MW501712
Lepidoptera;Lycaenidae;Polyommatus;Polyommatus ripartii;Russia;GenBank;-;MW501727
Lepidoptera;Pieridae;Pieris;Pieris mannii;Liechtenstein;GenBank;-;MW501828
Lepidoptera;Pieridae;Pieris;Pieris napi;Norway;GenBank;-;MW501853
Lepidoptera ;Nymphalidae;Pararge;Pararge aegeria;Belarus;GenBank;-;MW501885
Lepidoptera;Nymphalidae;Boloria;Boloria thore;Sweden;GenBank;-;MW501892
Lepidoptera;Nymphalidae;Hyponephele;Hyponephele lupina;Russia;Bold;EULEP4926-16;MW501979
Lepidoptera;Nymphalidae;Chazara;Chazara briseis;Ukraine;GenBank;-;MW501989
Lepidoptera;Nymphalidae;Hipparchia;Hipparchia semele;United Kingdom;GenBank;-;MW502021
Lepidoptera;Nymphalidae;Euphydryas;Euphydryas cynthia;Bulgaria;GenBank;-;MW502025
Lepidoptera;Nymphalidae;Erebia;Erebia melampus;Austria;GenBank;-;MW502090
Lepidoptera;Nymphalidae;Boloria;Boloria selene;Norway;GenBank;-;MW502104
Lepidoptera;Nymphalidae;Hipparchia;Hipparchia semele;United Kingdom;GenBank;-;MW502106
Lepidoptera ;Nymphalidae;Brintesia;Kanetisa circe;Greece;GenBank;-;MW502138
Lepidoptera ;Nymphalidae;Brintesia;Kanetisa circe;Greece;GenBank;-;MW502145
Lepidoptera;Lycaenidae;Aricia;Aricia cramera;Spain;GenBank;-;MW502151
Lepidoptera;Nymphalidae;Maniola;Maniola jurtina;United Kingdom;GenBank;-;MW502209
Lepidoptera;Nymphalidae;Boloria;Boloria thore;Finland;GenBank;-;MW502237
Lepidoptera;Pieridae;Leptidea;Leptidea juvernica;Russia;GenBank;-;MW502261
Lepidoptera;Pieridae;Leptidea;Leptidea juvernica;Russia;GenBank;-;MW502289
Lepidoptera;Nymphalidae;Erebia;Erebia eriphyle;Switzerland;GenBank;-;MW502296

Lepidoptera;Nymphalidae;Vanessa;Vanessa atalanta;Croatia;GenBank;-;MW502410
Lepidoptera;Nymphalidae;Hipparchia;Hipparchia hermione;Norway;GenBank;-;MW502422
Lepidoptera;Nymphalidae;Hipparchia;Hipparchia semele;United Kingdom;GenBank;-;MW502439
Lepidoptera;Nymphalidae;Erebia;Erebia medusa;Norway;GenBank;-;MW502512
Lepidoptera;Lycaenidae;Cupido;Cupido alcetas;Ukraine;GenBank;-;MW502543
Lepidoptera;Lycaenidae;Satyrium;Satyrium w-album;Sweden;GenBank;-;MW502549
Lepidoptera;Nymphalidae;Minois;Minois dryas;Liechtenstein;GenBank;-;MW502633
Lepidoptera;Lycaenidae;Phengaris;Maculinea teleius;Liechtenstein;GenBank;-;MW502690
Lepidoptera;Nymphalidae;Hipparchia;Hipparchia semele;United Kingdom;GenBank;-;MW502726
Lepidoptera;Nymphalidae;Euphydryas;Euphydryas cynthia;Bulgaria;GenBank;-;MW502804
Lepidoptera;Pieridae;Leptidea;Leptidea juvernica;Russia;GenBank;-;MW502816
Lepidoptera;Nymphalidae;Erebia;Erebia medusa;Norway;GenBank;-;MW502838
Lepidoptera;Nymphalidae;Erebia;Erebia medusa;Norway;GenBank;-;MW502855
Lepidoptera;Nymphalidae;Aphantopus;Aphantopus hyperantus;United Kingdom;Bold;OXB1517-16;MW502908
Lepidoptera;Nymphalidae;Aphantopus;Aphantopus hyperantus;United Kingdom;Bold;OXB1516-16;MW502977
Lepidoptera;Pieridae;Leptidea;Leptidea juvernica;Finland;GenBank;-;MW503006
Lepidoptera;Pieridae;Pieris;Pieris napi;Liechtenstein;GenBank;-;MW503047
Lepidoptera;Nymphalidae;Maniola;Maniola jurtina;United Kingdom;GenBank;-;MW503069
Lepidoptera;Nymphalidae;Argynnis;Argynnis aglaja;United Kingdom;GenBank;-;MW503079
Lepidoptera;Nymphalidae;Maniola;Maniola jurtina;United Kingdom;GenBank;-;MW503080
Lepidoptera;Nymphalidae;Hyponephele;Hyponephele lupina;Russia;Bold;EULEP4927-16;MW503095
Lepidoptera;Nymphalidae;Maniola;Maniola jurtina;United Kingdom;GenBank;-;MW503098
Lepidoptera;Papilionidae;Papilio;Papilio machaon;Liechtenstein;GenBank;-;MW503139
Lepidoptera;Lycaenidae;Agriades;Albulina orbitulus;Sweden;GenBank;-;MW503185
Lepidoptera;Nymphalidae;Hipparchia;Hipparchia leighebi;Italy;GenBank;-;MW503285
Lepidoptera;Nymphalidae;Erebia;Erebia gorge;Macedonia;GenBank;-;MW503331
Lepidoptera;Nymphalidae;Vanessa;Vanessa atalanta;United Kingdom;GenBank;-;MW503337
Lepidoptera;Nymphalidae;Hipparchia;Hipparchia sbordonii;Italy;GenBank;-;MW503389
Lepidoptera;Nymphalidae;Boloria;Boloria thore;Sweden;GenBank;-;MW503413
Lepidoptera;Nymphalidae;Hipparchia;Hipparchia aristaeus;Italy;GenBank;-;MW503436
Lepidoptera;Nymphalidae;Maniola;Maniola jurtina;United Kingdom;GenBank;-;MW503474
Lepidoptera;Hesperiidae;Thymelicus;Thymelicus sylvestris;Belarus;GenBank;-;MW503489
Lepidoptera ;Hesperiidae;Gegenes;Gegenes pumilio;Greece;GenBank;-;MW503500
Lepidoptera;Pieridae;Pieris;Pieris rapae;Belarus;GenBank;-;MW503501
Lepidoptera;Pieridae;Pieris;Pieris brassicae;Belarus;GenBank;-;MW503512
Lepidoptera;Nymphalidae;Nymphalis;Nymphalis io;Belarus;GenBank;-;MW503524
Lepidoptera;Nymphalidae;Erebia;Erebia gorge;Macedonia;GenBank;-;MW503531
Lepidoptera;Lycaenidae;Polyommatus;Polyommatus icarus;Estonia;GenBank;-;MW503533
Lepidoptera;Pieridae;Leptidea;Leptidea juvernica;Russia;GenBank;-;MW503551
Lepidoptera;Pieridae;Leptidea;Leptidea sinapis;Belarus;GenBank;-;MW503553
Lepidoptera;Lycaenidae;Polyommatus;Polyommatus icarus;Greece;GenBank;-;MW503555
Lepidoptera;Nymphalidae;Boloria;Boloria napaea;Sweden;GenBank;-;MW503589
Lepidoptera;Nymphalidae;Melitaea;Melitaea trivia;Greece;GenBank;-;MW503679
Lepidoptera;Nymphalidae;Chazara;Chazara briseis;Czech Republic;GenBank;-;MW653338
Lepidoptera;Lycaenidae;Agriades;Albulina orbitulus;Sweden;GenBank;-;MW857290
Lepidoptera;Nymphalidae;Fabriciana;Argynnis adippe;Sweden;GenBank;-;MZ026264
Lepidoptera;Nymphalidae;Fabriciana;Argynnis adippe;Sweden;GenBank;-;MZ026271
Lepidoptera;Nymphalidae;Fabriciana;Argynnis adippe;Finland;GenBank;-;MZ026282
Lepidoptera;Pieridae;Leptidea;Leptidea juvernica;Finland;GenBank;-;MZ622833
Lepidoptera;Pieridae;Leptidea;Leptidea sinapis;Finland;GenBank;-;MZ622839
Lepidoptera;Pieridae;Leptidea;Leptidea sinapis;Finland;GenBank;-;MZ623372
Lepidoptera;Pieridae;Leptidea;Leptidea sinapis;Finland;GenBank;-;MZ623386
Lepidoptera;Pieridae;Leptidea;Leptidea juvernica;Finland;GenBank;-;MZ625522
Lepidoptera;Pieridae;Leptidea;Leptidea sinapis;Finland;GenBank;-;MZ628594
Lepidoptera ;Hesperiidae;Gegenes;Gegenes pumilio;Italy;GenBank;-;OK345547
Lepidoptera ;Hesperiidae;Carcharodus;Carcharodus baeticus;Italy;Bold;WMB6801-19;OK345571
Lepidoptera ;Hesperiidae;Carcharodus;Carcharodus alceae;Spain;GenBank;-;OK345603
Lepidoptera;Papilionidae;Papilio;Papilio machaon;Denmark;GenBank;-;OM346749
Lepidoptera ;Hesperiidae;Carcharodus;Carcharodus alceae;Croatia;GenBank;-;ON435775
Lepidoptera;Hesperiidae;Erynnis;Erynnis tages;Croatia;GenBank;-;ON435820
Lepidoptera;Hesperiidae;Muschampia;Sloperia proto;Spain;GenBank;-;ON435882
Lepidoptera;Lycaenidae;Aricia;Aricia agestis;Croatia;GenBank;-;ON436027
Lepidoptera;Lycaenidae;Scolitantides;Scolitantides orion;Croatia;GenBank;-;ON436064
Lepidoptera;Pieridae;Anthocharis;Anthocharis cardamines;Croatia;GenBank;-;ON436231
Lepidoptera;Pieridae;Euchloe;Euchloe ausonia;Croatia;GenBank;-;ON436250
Lepidoptera;Papilionidae;Zerynthia;Zerynthia polyxena;Russia;GenBank;-;ON437050
Lepidoptera;Pieridae;Pieris;Pieris rapae;Croatia;GenBank;-;ON437129
Lepidoptera;Lycaenidae;Callophrys;Callophrys rubi;Russia;GenBank;-;OP430995
Lepidoptera;Nymphalidae;Aglais;Aglais ichnusa;Italy;Bold;BDE763-21;-
Lepidoptera;Nymphalidae;Aglais;Aglais ichnusa;Italy;Bold;BDE765-21;-
Lepidoptera;Nymphalidae;Aglais;Aglais urticae;Sweden;Bold;BDE753-21;-
Lepidoptera;Nymphalidae;Aglais;Aglais urticae;Greece;Bold;BDE755-21;-
Lepidoptera;Nymphalidae;Aglais;Aglais urticae;Italy;Bold;BDE764-21;-
Lepidoptera;Nymphalidae;Aglais;Aglais urticae;Denmark;Bold;BDE752-21;-
Lepidoptera;Nymphalidae;Aglais;Aglais urticae;Poland;Bold;BDE756-21;-
Lepidoptera;Nymphalidae;Aglais;Aglais urticae;Sweden;Bold;BDE754-21;-
Lepidoptera;Nymphalidae;Aphantopus;Aphantopus hyperantus;Norway;Bold;LON4634-16;-
Lepidoptera;Nymphalidae;Aphantopus;Aphantopus hyperantus;Finland;Bold;EULEP6379-20;-

Lepidoptera;Lycaenidae;Celastrina;Celastrina argiolus;Italy;Bold;BIBSA2289-20;-
Lepidoptera;Nymphalidae;Argynnis;Argynnis pandora;Italy;Bold;BIBSA2176-20;-
Lepidoptera;Nymphalidae;Argynnis;Argynnis paphia;France;Bold;BIBSA1981-19;-
Lepidoptera;Lycaenidae;Aricia;Aricia agestis;France;Bold;BIBSA1944-19;-
Lepidoptera;Lycaenidae;Aricia;Aricia agestis;France;Bold;BIBSA1987-19;-
Lepidoptera;Lycaenidae;Aricia;Aricia agestis;France;Bold;BIBSA1942-19;-
Lepidoptera;Lycaenidae;Aricia;Aricia agestis;France;Bold;BIBSA1943-19;-
Lepidoptera;Lycaenidae;Aricia;Aricia agestis;France;Bold;BIBSA1988-19;-
Lepidoptera;Pieridae;Colias;Colias crocea;France;Bold;BIBSA1965-19;-
Lepidoptera;Nymphalidae;Boloria;Boloria napaea;Norway;Bold;LON5659-17;-
Lepidoptera;Nymphalidae;Boloria;Boloria thore;Finland;Bold;EULEP6381-20;-
Lepidoptera;Nymphalidae;Boloria;Boloria titania;Estonia;Bold;EULEP2681-15;-
Lepidoptera;Nymphalidae;Hipparchia;Hipparchia semele;Sweden;Bold;EULEP6005-20;-
Lepidoptera;Lycaenidae;Callophrys;Callophrys rubi;Greece;Bold;EULEP6264-20;-
Lepidoptera;Lycaenidae;Callophrys;Callophrys rubi;Finland;Bold;EULEP6254-20;-
Lepidoptera;Lycaenidae;Callophrys;Callophrys rubi;France;Bold;BIBSA1940-19;-
Lepidoptera ;Hesperiidae;Carcharodus;Carcharodus alceae;Italy;Bold;BIBSA2177-20;-
Lepidoptera ;Hesperiidae;Carcharodus;Carcharodus alceae;Croatia;Bold;EULEP6170-20;-
Lepidoptera;Nymphalidae;Erebia;Erebia montana;Italy;Bold;BIBSA2609-20;-
Lepidoptera;Lycaenidae;Celastrina;Celastrina argiolus;Italy;Bold;BIBSA2175-20;-
Lepidoptera;Lycaenidae;Celastrina;Celastrina argiolus;Italy;Bold;BIBSA2275-20;-
Lepidoptera;Nymphalidae;Charaxes;Charaxes jasius;Italy;Bold;BIBSA2107-20;-
Lepidoptera;Nymphalidae;Charaxes;Charaxes jasius;Italy;Bold;BIBSA2391-20;-
Lepidoptera;Nymphalidae;Charaxes;Charaxes jasius;France;Bold;ATLAS071-22;-
Lepidoptera;Nymphalidae;Coenonympha;Coenonympha corinna;Italy;Bold;BIBSA2266-20;-
Lepidoptera;Nymphalidae;Coenonympha;Coenonympha corinna;Italy;Bold;WMB6554-18;-
Lepidoptera;Pieridae;Euchloe;Euchloe ausonia;Italy;Bold;BIBSA2292-20;-
Lepidoptera;Nymphalidae;Coenonympha;Coenonympha oedippus;Austria;Bold;ABOLD495-16;-
Lepidoptera;Nymphalidae;Coenonympha;Coenonympha pamphilus;France;Bold;BIBSA1978-19;-
Lepidoptera;Lycaenidae;Eumedonia;Eumedonia eumedon;Norway;Bold;LON6411-17;-
Lepidoptera;Nymphalidae;Coenonympha;Coenonympha pamphilus;France;Bold;BIBSA1953-19;-
Lepidoptera;Nymphalidae;Coenonympha;Coenonympha pamphilus;France;Bold;BIBSA1952-19;-
Lepidoptera;Nymphalidae;Coenonympha;Coenonympha pamphilus;France;Bold;BIBSA1977-19;-
Lepidoptera;Pieridae;Colias;Colias alfacariensis;Russia;Bold;SACOL345-19;-
Lepidoptera;Pieridae;Colias;Colias crocea;Italy;Bold;BIBSA2286-20;-
Lepidoptera;Pieridae;Colias;Colias crocea;Italy;Bold;BIBSA2385-20;-
Lepidoptera;Pieridae;Colias;Colias crocea;Italy;Bold;BIBSA2282-20;-
Lepidoptera;Pieridae;Colias;Colias crocea;Italy;Bold;BIBSA2428-20;-
Lepidoptera;Pieridae;Colias;Colias crocea;Italy;Bold;BIBSA2400-20;-
Lepidoptera;Pieridae;Colias;Colias crocea;Italy;Bold;BIBSA2386-20;-
Lepidoptera;Pieridae;Colias;Colias crocea;Italy;Bold;BIBSA2399-20;-
Lepidoptera;Pieridae;Colias;Colias crocea;Italy;Bold;BIBSA2285-20;-
Lepidoptera;Pieridae;Colias;Colias crocea;Italy;Bold;BIBSA2427-20;-
Lepidoptera;Pieridae;Colias;Colias hyale;Turkey;Bold;SACOL243-18;-
Lepidoptera;Pieridae;Colias;Colias hyale;Russia;Bold;SACOL182-17;-
Lepidoptera;Pieridae;Colias;Colias hyale;Russia;Bold;SACOL181-17;-
Lepidoptera;Pieridae;Colias;Colias palaeno;Norway;Bold;LON4576-16;-
Lepidoptera;Lycaenidae;Cupido;Cupido argiades;France;Bold;BIBSA1945-19;-
Lepidoptera;Lycaenidae;Cupido;Cupido argiades;France;Bold;BIBSA1946-19;-
Lepidoptera;Lycaenidae;Cupido;Cupido minimus;Sweden;Bold;EULEP6032-20;-
Lepidoptera;Lycaenidae;Cupido;Cupido minimus;Croatia;Bold;MIMAB011-21;-
Lepidoptera;Nymphalidae;Hipparchia;Hipparchia aristaeus;Italy;Bold;ATLAS257-22;-
Lepidoptera;Nymphalidae;Hipparchia;Hipparchia fagi;Italy;Bold;BIBSA2407-20;-
Lepidoptera;Nymphalidae;Hipparchia;Hipparchia neapolitana;Italy;Bold;BIBSA2395-20;-
Lepidoptera;Nymphalidae;Hipparchia;Hipparchia semele;France;Bold;BIBSA1986-19;-
Lepidoptera;Nymphalidae;Erebia;Erebia manto;Spain;Bold;BDE233-19;-
Lepidoptera;Nymphalidae;Erebia;Erebia montana;Italy;Bold;BIBSA2608-20;-
Lepidoptera ;Lycaenidae;Lampides;Lampides boeticus;Italy;Bold;BIBSA2178-20;-
Lepidoptera;Lycaenidae;Favonius;Favonius quercus;Norway;Bold;LON5668-17;-
Lepidoptera ;Hesperiidae;Gegenes;Gegenes nostrodamus;Spain;Bold;VNMB335-08;-
Lepidoptera ;Hesperiidae;Gegenes;Gegenes nostrodamus;Spain;Bold;VNMB336-08;-
Lepidoptera ;Hesperiidae;Gegenes;Gegenes pumilio;Greece;Bold;VNMB327-08;-
Lepidoptera ;Pieridae;Gonepteryx;Gonepteryx cleopatra;Italy;Bold;BIBSA2548-20;-
Lepidoptera ;Pieridae;Gonepteryx;Gonepteryx cleopatra;Italy;Bold;BIBSA2165-20;-
Lepidoptera ;Pieridae;Gonepteryx;Gonepteryx cleopatra;Italy;Bold;BIBSA2383-20;-
Lepidoptera ;Pieridae;Gonepteryx;Gonepteryx cleopatra;Italy;Bold;BIBSA2549-20;-
Lepidoptera ;Pieridae;Gonepteryx;Gonepteryx cleopatra;Italy;Bold;BIBSA2576-20;-
Lepidoptera ;Pieridae;Gonepteryx;Gonepteryx cleopatra;Italy;Bold;BIBSA2550-20;-
Lepidoptera ;Pieridae;Gonepteryx;Gonepteryx cleopatra;Italy;Bold;BIBSA2429-20;-
Lepidoptera;Hesperiidae;Heteropterus;Heteropterus morpheus;France;Bold;BIBSA1950-19;-
Lepidoptera;Hesperiidae;Heteropterus;Heteropterus morpheus;France;Bold;BIBSA1951-19;-
Lepidoptera;Nymphalidae;Hipparchia;Hipparchia neapolitana;Italy;Bold;BIBSA2396-20;-
Lepidoptera;Nymphalidae;Hipparchia;Hipparchia semele;France;Bold;BIBSA1985-19;-
Lepidoptera;Nymphalidae;Hipparchia;Hipparchia statilinus;Italy;Bold;BIBSA2422-20;-
Lepidoptera;Lycaenidae;Iolana;Iolana iolas;Turkey;Bold;ATLAS297-22;-
Lepidoptera;Lycaenidae;Iolana;Iolana iolas;Turkey;Bold;ATLAS296-22;-
Lepidoptera ;Nymphalidae;Issoria;Issoria lathonia;Italy;Bold;BIBSA2128-20;-
Lepidoptera ;Lycaenidae;Lampides;Lampides boeticus;Italy;Bold;BIBSA2430-20;-

Lepidoptera ;Lycaenidae;Lampides;Lampides boeticus;Italy;Bold;BIBSA2288-20;-
Lepidoptera ;Lycaenidae;Lampides;Lampides boeticus;Italy;Bold;BIBSA2287-20;-
Lepidoptera ;Lycaenidae;Lampides;Lampides boeticus;Italy;Bold;BIBSA2415-20;-
Lepidoptera ;Lycaenidae;Lampides;Lampides boeticus;Italy;Bold;BIBSA2431-20;-
Lepidoptera ;Lycaenidae;Lampides;Lampides boeticus;Italy;Bold;BIBSA2416-20;-
Lepidoptera ;Lycaenidae;Lampides;Lampides boeticus;Italy;Bold;BIBSA2359-20;-
Lepidoptera;Nymphalidae;Maniola;Maniola jurtina;France;Bold;BIBSA1967-19;-
Lepidoptera;Nymphalidae;Lasiommata;Lasiommata megera;France;Bold;BIBSA1962-19;-
Lepidoptera;Nymphalidae;Lasiommata;Lasiommata megera;France;Bold;BIBSA1972-19;-
Lepidoptera;Nymphalidae;Lasiommata;Lasiommata megera;Italy;Bold;BIBSA2361-20;-
Lepidoptera;Nymphalidae;Lasiommata;Lasiommata megera;Italy;Bold;BIBSA2360-20;-
Lepidoptera;Nymphalidae;Lasiommata;Lasiommata megera;Italy;Bold;BIBSA2362-20;-
Lepidoptera;Nymphalidae;Lasiommata;Lasiommata megera;Italy;Bold;BIBSA2284-20;-
Lepidoptera;Nymphalidae;Lasiommata;Lasiommata megera;Italy;Bold;BIBSA2291-20;-
Lepidoptera;Nymphalidae;Lasiommata;Lasiommata megera;France;Bold;BIBSA1971-19;-
Lepidoptera;Nymphalidae;Lasiommata;Lasiommata paramegaera;Italy;Bold;BIBSA2216-20;-
Lepidoptera;Nymphalidae;Lasiommata;Lasiommata paramegaera;Italy;Bold;BIBSA2217-20;-
Lepidoptera;Pieridae;Leptidea;Leptidea juvernica;Czech Republic;Bold;HBOK089-08;-
Lepidoptera;Pieridae;Leptidea;Leptidea sinapis;Norway;Bold;LON5682-17;-
Lepidoptera;Pieridae;Leptidea;Leptidea sinapis;Slovenia;Bold;EULEP6457-20;-
Lepidoptera;Pieridae;Leptidea;Leptidea sinapis;Slovenia;Bold;EULEP6456-20;-
Lepidoptera;Pieridae;Leptidea;Leptidea sinapis;Slovenia;Bold;EULEP6452-20;-
Lepidoptera;Pieridae;Leptidea;Leptidea sinapis;Slovenia;Bold;EULEP6453-20;-
Lepidoptera;Pieridae;Leptidea;Leptidea sinapis;Slovenia;Bold;EULEP6455-20;-
Lepidoptera;Pieridae;Leptidea;Leptidea sinapis;France;Bold;BIBSA1957-19;-
Lepidoptera;Pieridae;Leptidea;Leptidea sinapis;France;Bold;BIBSA1955-19;-
Lepidoptera;Pieridae;Leptidea;Leptidea sinapis;France;Bold;BIBSA1956-19;-
Lepidoptera;Pieridae;Leptidea;Leptidea sinapis;Slovenia;Bold;EULEP6454-20;-
Lepidoptera ;Lycaenidae;Leptotes;Leptotes pirithous;Italy;Bold;BIBSA2432-20;-
Lepidoptera ;Lycaenidae;Leptotes;Leptotes pirithous;Italy;Bold;BIBSA2392-20;-
Lepidoptera ;Lycaenidae;Leptotes;Leptotes pirithous;Italy;Bold;BIBSA2393-20;-
Lepidoptera ;Lycaenidae;Leptotes;Leptotes pirithous;Italy;Bold;BIBSA2390-20;-
Lepidoptera ;Lycaenidae;Leptotes;Leptotes pirithous;Italy;Bold;BIBSA2394-20;-
Lepidoptera ;Lycaenidae;Leptotes;Leptotes pirithous;Italy;Bold;BIBSA2433-20;-
Lepidoptera;Lycaenidae;Lycaena;Lycaena phlaeas;Sweden;Bold;EULEP6033-20;-
Lepidoptera;Lycaenidae;Lycaena;Lycaena phlaeas;Italy;Bold;BIBSA2408-20;-
Lepidoptera;Lycaenidae;Lycaena;Lycaena phlaeas;Italy;Bold;BIBSA2405-20;-
Lepidoptera;Lycaenidae;Lycaena;Lycaena phlaeas;Italy;Bold;BIBSA2404-20;-
Lepidoptera;Lycaenidae;Lycaena;Lycaena phlaeas;Netherlands;Bold;NLLEA1466-14;-
Lepidoptera;Hesperiidae;Ochlodes;Ochlodes sylvanus;France;Bold;BIBSA1963-19;-
Lepidoptera;Papilionidae;Papilio;Papilio machaon;Italy;Bold;BIBSA2417-20;-
Lepidoptera;Papilionidae;Papilio;Papilio machaon;Italy;Bold;BIBSA2434-20;-
Lepidoptera;Nymphalidae;Maniola;Maniola jurtina;France;Bold;BIBSA1937-19;-
Lepidoptera;Nymphalidae;Maniola;Maniola jurtina;Norway;Bold;LON6142-17;-
Lepidoptera;Nymphalidae;Maniola;Maniola jurtina;France;Bold;BIBSA1936-19;-
Lepidoptera;Nymphalidae;Maniola;Maniola jurtina;France;Bold;BIBSA1935-19;-
Lepidoptera;Nymphalidae;Maniola;Maniola jurtina;France;Bold;BIBSA1968-19;-
Lepidoptera;Nymphalidae;Maniola;Maniola jurtina;Norway;Bold;LON6829-18;-
Lepidoptera;Nymphalidae;Maniola;Maniola jurtina;Netherlands;Bold;NLLEA1465-14;-
Lepidoptera ;Nymphalidae;Pararge;Pararge aegeria;France;Bold;BIBSA1934-19;-
Lepidoptera ;Nymphalidae;Pararge;Pararge aegeria;France;Bold;BIBSA1933-19;-
Lepidoptera;Nymphalidae;Melanargia;Melanargia galathea;France;Bold;BIBSA1980-19;-
Lepidoptera;Pieridae;Pieris;Pieris mannii;Italy;Bold;BIBSA2409-20;-
Lepidoptera;Nymphalidae;Melitaea;Melitaea aurelia;Ukraine;Bold;MBMPA194-09;-
Lepidoptera;Nymphalidae;Melitaea;Melitaea aurelia;Ukraine;Bold;MBMPA191-09;-
Lepidoptera;Nymphalidae;Melitaea;Melitaea aurelia;Ukraine;Bold;MBMPA196-09;-
Lepidoptera;Nymphalidae;Melitaea;Melitaea aurelia;Ukraine;Bold;MBMPA198-09;-
Lepidoptera;Nymphalidae;Melitaea;Melitaea aurelia;Ukraine;Bold;MBMPA197-09;-
Lepidoptera;Nymphalidae;Melitaea;Melitaea aurelia;Ukraine;Bold;MBMPA189-09;-
Lepidoptera;Nymphalidae;Melitaea;Melitaea aurelia;Ukraine;Bold;MBMPA193-09;-
Lepidoptera;Nymphalidae;Melitaea;Melitaea aurelia;Ukraine;Bold;MBMPA192-09;-
Lepidoptera;Nymphalidae;Melitaea;Melitaea aurelia;Ukraine;Bold;MBMPA190-09;-
Lepidoptera;Nymphalidae;Melitaea;Melitaea aurelia;Ukraine;Bold;MBMPA195-09;-
Lepidoptera;Nymphalidae;Melitaea;Melitaea cinxia;Norway;Bold;LON1107-12;-
Lepidoptera;Nymphalidae;Melitaea;Melitaea didyma;Croatia;Bold;LON7271-18;-
Lepidoptera;Pieridae;Pieris;Pieris rapae;Slovakia;Bold;HMYZ021-19;-
Lepidoptera;Hesperiidae;Ochlodes;Ochlodes sylvanus;Norway;Bold;LON6412-17;-
Lepidoptera;Papilionidae;Papilio;Papilio machaon;Finland;Bold;EULEP6383-20;-
Lepidoptera;Papilionidae;Papilio;Papilio machaon;Italy;Bold;BIBSA2281-20;-
Lepidoptera;Papilionidae;Papilio;Papilio machaon;France;Bold;EULEP6152-20;-
Lepidoptera;Papilionidae;Papilio;Papilio machaon;Italy;Bold;BIBSA2542-20;-
Lepidoptera;Papilionidae;Papilio;Papilio machaon;Italy;Bold;BIBSA2418-20;-
Lepidoptera;Papilionidae;Papilio;Papilio machaon;Italy;Bold;BIBSA2556-20;-
Lepidoptera ;Nymphalidae;Pararge;Pararge aegeria;Italy;Bold;BIBSA2540-20;-
Lepidoptera ;Nymphalidae;Pararge;Pararge aegeria;France;Bold;BIBSA1976-19;-
Lepidoptera ;Nymphalidae;Pararge;Pararge aegeria;Norway;Bold;LON6413-17;-
Lepidoptera ;Nymphalidae;Pararge;Pararge aegeria;France;Bold;BIBSA1966-19;-
Lepidoptera;Lycaenidae;Polyommatus;Polyommatus icarus;France;Bold;BIBSA1970-19;-

Lepidoptera;Lycaenidae;Polyommatus;Polyommatus icarus;Turkey;Bold;LOWAB287-09;-
Lepidoptera ;Nymphalidae;Pararge;Pararge aegeria;Italy;Bold;BIBSA2301-20;-
Lepidoptera ;Nymphalidae;Pararge;Pararge aegeria;France;Bold;BIBSA1975-19;-
Lepidoptera;Nymphalidae;Vanessa;Vanessa atalanta;Italy;Bold;BIBSA2420-20;-
Lepidoptera;Pieridae;Pieris;Pieris brassicae;Italy;Bold;BIBSA2425-20;-
Lepidoptera;Pieridae;Pieris;Pieris brassicae;Italy;Bold;BIBSA2204-20;-
Lepidoptera;Pieridae;Pieris;Pieris brassicae;Italy;Bold;BIBSA2172-20;-
Lepidoptera;Pieridae;Pieris;Pieris brassicae;France;Bold;BIBSA2267-20;-
Lepidoptera;Pieridae;Pontia;Pieris edusa;Italy;Bold;BIBSA2283-20;-
Lepidoptera;Pieridae;Pieris;Pieris mannii;Italy;Bold;BIBSA2410-20;-
Lepidoptera;Pieridae;Pieris;Pieris mannii;Italy;Bold;BIBSA2426-20;-
Lepidoptera;Pieridae;Pieris;Pieris mannii;Croatia;Bold;EULEP6154-20;-
Lepidoptera;Pieridae;Pieris;Pieris mannii;Italy;Bold;BIBSA2363-20;-
Lepidoptera;Pieridae;Pieris;Pieris napi;Netherlands;Bold;NLLEA1467-14;-
Lepidoptera;Pieridae;Pieris;Pieris rapae;Netherlands;Bold;NLLEA1464-14;-
Lepidoptera;Pieridae;Pieris;Pieris rapae;Italy;Bold;BIBSA2414-20;-
Lepidoptera;Pieridae;Pieris;Pieris rapae;Italy;Bold;BIBSA2543-20;-
Lepidoptera;Pieridae;Pieris;Pieris rapae;Italy;Bold;BIBSA2233-20;-
Lepidoptera;Pieridae;Pieris;Pieris rapae;Italy;Bold;BIBSA2424-20;-
Lepidoptera;Pieridae;Pieris;Pieris rapae;Belarus;Bold;GMBMR047-17;-
Lepidoptera;Pieridae;Pieris;Pieris rapae;France;Bold;BIBSA1941-19;-
Lepidoptera;Pieridae;Pieris;Pieris rapae;France;Bold;BIBSA1979-19;-
Lepidoptera;Pieridae;Pieris;Pieris rapae;Italy;Bold;BIBSA2553-20;-
Lepidoptera;Pieridae;Pieris;Pieris rapae;Italy;Bold;BIBSA2300-20;-
Lepidoptera;Nymphalidae;Pyronia;Pyronia cecilia;Italy;Bold;BIBSA2234-20;-
Lepidoptera;Nymphalidae;Polygonia;Polygonia egea;Italy;Bold;BIBSA2459-20;-
Lepidoptera;Lycaenidae;Polyommatus;Polyommatus icarus;Italy;Bold;BIBSA2171-20;-
Lepidoptera;Lycaenidae;Polyommatus;Polyommatus icarus;Italy;Bold;WMB229-11;-
Lepidoptera;Hesperiidae;Thymelicus;Thymelicus acteon;France;Bold;BIBSA1948-19;-
Lepidoptera;Pieridae;Pontia;Pieris edusa;Italy;Bold;BIBSA2413-20;-
Lepidoptera;Hesperiidae;Thymelicus;Thymelicus acteon;France;Bold;BIBSA1947-19;-
Lepidoptera;Lycaenidae;Polyommatus;Polyommatus icarus;Norway;Bold;TSZJK790-16;-
Lepidoptera;Lycaenidae;Polyommatus;Polyommatus icarus;Turkey;Bold;LOWAB167-09;-
Lepidoptera;Lycaenidae;Polyommatus;Polyommatus icarus;France;Bold;BIBSA1969-19;-
Lepidoptera;Lycaenidae;Polyommatus;Polyommatus icarus;France;Bold;BIBSA1961-19;-
Lepidoptera;Lycaenidae;Polyommatus;Polyommatus icarus;France;Bold;BIBSA1960-19;-
Lepidoptera;Lycaenidae;Polyommatus;Polyommatus icarus;France;Bold;BIBSA1958-19;-
Lepidoptera;Lycaenidae;Polyommatus;Polyommatus icarus;France;Bold;BIBSA1959-19;-
Lepidoptera;Lycaenidae;Polyommatus;Polyommatus icarus;Italy;Bold;BIBSA2419-20;-
Lepidoptera;Lycaenidae;Polyommatus;Polyommatus icarus;Italy;Bold;ATLAS620-22;-
Lepidoptera;Nymphalidae;Vanessa;Vanessa atalanta;France;Bold;BIBSA1964-19;-
Lepidoptera;Nymphalidae;Vanessa;Vanessa cardui;Norway;Bold;LON5674-17;-
Lepidoptera;Nymphalidae;Vanessa;Vanessa cardui;Italy;Bold;BIBSA2387-20;-
Lepidoptera;Nymphalidae;Vanessa;Vanessa cardui;Italy;Bold;BIBSA2402-20;-
Lepidoptera;Nymphalidae;Vanessa;Vanessa cardui;Italy;Bold;BIBSA2544-20;-
Lepidoptera;Pieridae;Pontia;Pieris edusa;Italy;Bold;ATLAS663-22;-
Lepidoptera;Pieridae;Pontia;Pieris edusa;Italy;Bold;BIBSA2398-20;-
Lepidoptera;Pieridae;Pontia;Pieris edusa;Italy;Bold;BIBSA2397-20;-
Lepidoptera;Lycaenidae;Pseudophilotes;Pseudophilotes baton;Spain;Bold;BDE289-19;-
Lepidoptera;Nymphalidae;Pyronia;Pyronia tithonus;France;Bold;BIBSA1938-19;-
Lepidoptera;Nymphalidae;Pyronia;Pyronia tithonus;France;Bold;BIBSA1939-19;-
Lepidoptera;Hesperiidae;Pyrgus;Pyrgus armoricanus;Italy;Bold;ATLAS704-22;-
Lepidoptera;Hesperiidae;Thymelicus;Thymelicus sylvestris;United Kingdom;Bold;ANBIO330-19;-
Lepidoptera;Nymphalidae;Vanessa;Vanessa cardui;Italy;Bold;BIBSA2436-20;-
Lepidoptera;Nymphalidae;Pyronia;Pyronia cecilia;Italy;Bold;BIBSA2215-20;-
Lepidoptera;Hesperiidae;Thymelicus;Thymelicus acteon;France;Bold;BIBSA1984-19;-
Lepidoptera;Hesperiidae;Thymelicus;Thymelicus acteon;France;Bold;BIBSA1949-19;-
Lepidoptera;Nymphalidae;Pyronia;Pyronia cecilia;Italy;Bold;BIBSA2168-20;-
Lepidoptera;Nymphalidae;Pyronia;Pyronia tithonus;France;Bold;BIBSA1974-19;-
Lepidoptera;Nymphalidae;Vanessa;Vanessa atalanta;United Kingdom;Bold;ANBIO196-19;-
Lepidoptera;Nymphalidae;Vanessa;Vanessa atalanta;Italy;Bold;BIBSA2403-20;-
Lepidoptera;Hesperiidae;Thymelicus;Thymelicus acteon;France;Bold;BIBSA1983-19;-
Lepidoptera;Nymphalidae;Vanessa;Vanessa cardui;Italy;Bold;BIBSA2388-20;-
Lepidoptera;Nymphalidae;Vanessa;Vanessa cardui;Italy;Bold;BIBSA2435-20;-
Lepidoptera;Nymphalidae;Vanessa;Vanessa cardui;Italy;Bold;BIBSA2421-20;-
Lepidoptera;Nymphalidae;Vanessa;Vanessa cardui;Italy;Bold;BIBSA2389-20;-
Lepidoptera;Nymphalidae;Vanessa;Vanessa atalanta;Italy;Bold;BIBSA2412-20;-
Lepidoptera;Nymphalidae;Vanessa;Vanessa cardui;Italy;Bold;BIBSA2401-20;-
Lepidoptera;Nymphalidae;Vanessa;Vanessa atalanta;Italy;Bold;BIBSA2411-20;-
Lepidoptera;Nymphalidae;Vanessa;Vanessa cardui;Italy;Bold;BIBSA2557-20;-
Lepidoptera;Nymphalidae;Vanessa;Vanessa cardui;Italy;Bold;BIBSA2547-20;-
Lepidoptera;Nymphalidae;Vanessa;Vanessa atalanta;Italy;Bold;BIBSA2384-20;-
Odonata;Coenagrionidae;Nehalennia;Nehalennia speciosa;Russia;GenBank;-;AM696290
Odonata;Coenagrionidae;Nehalennia;Nehalennia speciosa;Russia;GenBank;-;FN252225
Odonata;Coenagrionidae;Nehalennia;Nehalennia speciosa;Russia;GenBank;-;FN252227
Odonata;Coenagrionidae;Nehalennia;Nehalennia speciosa;Russia;GenBank;-;FN252228
Odonata;Coenagrionidae;Nehalennia;Nehalennia speciosa;Russia;GenBank;-;FN252229
Odonata;Coenagrionidae;Nehalennia;Nehalennia speciosa;Russia;GenBank;-;FN252230

Odonata;Coenagrionidae;Coenagrion;Coenagrion mercuriale;Italy;GenBank;-;KX241514
Odonata;Cordulegastridae;Cordulegaster;Cordulegaster heros;Bulgaria;GenBank;-;MK779812
Odonata;Libellulidae;Sympetrum;Sympetrum fonscolombii;Malta;GenBank;-;MN701480
Odonata;Libellulidae;Sympetrum;Sympetrum fonscolombii;Malta;GenBank;-;MN701485
Odonata;Libellulidae;Sympetrum;Sympetrum fonscolombii;Malta;GenBank;-;MN701488
Odonata;Libellulidae;Crocothemis;Crocothemis erythraea;Malta;GenBank;-;MN701489
Odonata;Coenagrionidae;Ischnura;Ischnura genei;Malta;GenBank;-;MN701492
Odonata;Aeshnidae;Anax;Anax imperator;Malta;GenBank;-;MN701497
Odonata;Coenagrionidae;Ischnura;Ischnura genei;Malta;GenBank;-;MN701499
Odonata;Libellulidae;Sympetrum;Sympetrum fonscolombii;Malta;GenBank;-;MN701503
Odonata;Coenagrionidae;Ischnura;Ischnura genei;Malta;GenBank;-;MN701505
Odonata;Coenagrionidae;Ischnura;Ischnura genei;Malta;GenBank;-;MN701507
Odonata;Aeshnidae;Anax;Anax imperator;Malta;GenBank;-;MN701510
Odonata;Libellulidae;Sympetrum;Sympetrum fonscolombii;Malta;GenBank;-;MN701513
Odonata;Libellulidae;Sympetrum;Sympetrum fonscolombii;Malta;GenBank;-;MN701518
Odonata;Aeshnidae;Anax;Anax imperator;Malta;GenBank;-;MN701520
Odonata;Coenagrionidae;Ischnura;Ischnura genei;Malta;GenBank;-;MN701521
Odonata;Libellulidae;Sympetrum;Sympetrum fonscolombii;Malta;GenBank;-;MN701526
Odonata;Libellulidae;Orthetrum;Orthetrum coerulescens;Malta;GenBank;-;MN701535
Odonata;Coenagrionidae;Ischnura;Ischnura genei;Malta;GenBank;-;MN701537
Odonata;Libellulidae;Sympetrum;Sympetrum fonscolombii;Malta;GenBank;-;MN701539
Odonata;Coenagrionidae;Ischnura;Ischnura genei;Malta;GenBank;-;MN701541
Odonata;Coenagrionidae;Ischnura;Ischnura genei;Malta;GenBank;-;MN701543
Odonata;Libellulidae;Trithemis;Trithemis annulata;Malta;GenBank;-;MN701545
Odonata;Libellulidae;Crocothemis;Crocothemis erythraea;Malta;GenBank;-;MN701547
Odonata;Aeshnidae;Anax;Anax imperator;Malta;GenBank;-;MN701548
Odonata;Libellulidae;Sympetrum;Sympetrum fonscolombii;Malta;GenBank;-;MN701550
Odonata;Libellulidae;Sympetrum;Sympetrum fonscolombii;Malta;GenBank;-;MN701554
Odonata;Libellulidae;Trithemis;Trithemis annulata;Malta;GenBank;-;MN701557
Odonata;Libellulidae;Sympetrum;Sympetrum fonscolombii;Malta;GenBank;-;MN701559
Odonata;Coenagrionidae;Ischnura;Ischnura genei;Malta;GenBank;-;MN701560
Odonata;Aeshnidae;Aeshna;Aeshna subarctica;Norway;GenBank;-;MN847794
Odonata;Libellulidae;Sympetrum;Sympetrum danae;Denmark;GenBank;-;MN847798
Odonata;Libellulidae;Sympetrum;Sympetrum danae;Denmark;GenBank;-;MN847799
Odonata;Libellulidae;Sympetrum;Sympetrum danae;Denmark;GenBank;-;MN847805
Odonata;Libellulidae;Sympetrum;Sympetrum danae;Denmark;GenBank;-;MN847807
Odonata;Libellulidae;Libellula;Libellula quadrimaculata;Denmark;GenBank;-;MN847820
Odonata;Libellulidae;Libellula;Libellula quadrimaculata;Denmark;GenBank;-;MN847821
Odonata;Libellulidae;Libellula;Libellula quadrimaculata;Denmark;GenBank;-;MN847822
Odonata;Libellulidae;Libellula;Libellula quadrimaculata;Denmark;GenBank;-;MN847823
Odonata;Aeshnidae;Aeshna;Aeshna juncea;Denmark;GenBank;-;MN847870
Odonata;Aeshnidae;Aeshna;Aeshna juncea;Denmark;GenBank;-;MN847873
Odonata;Aeshnidae;Aeshna;Aeshna cyanea;Denmark;GenBank;-;MN939078
Odonata;Aeshnidae;Aeshna;Aeshna cyanea;Denmark;GenBank;-;MN939081
Odonata;Aeshnidae;Aeshna;Aeshna cyanea;Denmark;GenBank;-;MN939083
Odonata;Libellulidae;Orthetrum;Orthetrum coerulescens;Denmark;GenBank;-;MN957914
Odonata;Libellulidae;Orthetrum;Orthetrum coerulescens;Denmark;GenBank;-;MN957915
Odonata;Libellulidae;Orthetrum;Orthetrum coerulescens;Greece;GenBank;-;MN957939
Odonata;Libellulidae;Orthetrum;Orthetrum coerulescens;Spain;GenBank;-;MN957940
Odonata;Libellulidae;Orthetrum;Orthetrum coerulescens;Greece;GenBank;-;MN957946
Odonata;Libellulidae;Orthetrum;Orthetrum coerulescens;Greece;GenBank;-;MN957947
Odonata;Libellulidae;Orthetrum;Orthetrum cancellatum;Denmark;GenBank;-;MN959414
Odonata;Libellulidae;Orthetrum;Orthetrum cancellatum;Denmark;GenBank;-;MN959427
Odonata;Libellulidae;Orthetrum;Orthetrum cancellatum;Denmark;GenBank;-;MN959440
Odonata;Libellulidae;Orthetrum;Orthetrum cancellatum;Sweden;GenBank;-;MN959444
Odonata;Libellulidae;Orthetrum;Orthetrum cancellatum;Sweden;GenBank;-;MN959445
Odonata;Aeshnidae;Aeshna;Aeshna affinis;Italy;GenBank;-;MT298234
Odonata;Aeshnidae;Aeshna;Aeshna isoceles;Italy;GenBank;-;MT298241
Odonata;Aeshnidae;Anax;Anax ephippiger;Italy;GenBank;-;MT298251
Odonata;Coenagrionidae;Ceriagrion;Ceriagrion tenellum;Italy;GenBank;-;MT298299
Odonata;Coenagrionidae;Coenagrion;Coenagrion mercuriale;Italy;GenBank;-;MT298339
Odonata;Coenagrionidae;Erythromma;Erythromma lindenii;Italy;GenBank;-;MT298435
Odonata;Coenagrionidae;Erythromma;Erythromma viridulum;Italy;GenBank;-;MT298455
Odonata;Coenagrionidae;Ischnura;Ischnura genei;Italy;GenBank;-;MT298470
Odonata;Coenagrionidae;Ischnura;Ischnura genei;Italy;GenBank;-;MT298471
Odonata;Coenagrionidae;Ischnura;Ischnura genei;Italy;GenBank;-;MT298472
Odonata;Lestidae;Lestes;Lestes barbarus;Italy;GenBank;-;MT298481
Odonata;Libellulidae;Libellula;Libellula fulva;Italy;Bold;ZPLOD535-20;MT298514
Odonata;Libellulidae;Orthetrum;Orthetrum coerulescens;Italy;GenBank;-;MT298576
Odonata;Libellulidae;Orthetrum;Orthetrum coerulescens;Italy;GenBank;-;MT298579
Odonata;Corduliidae;Somatochlora;Somatochlora flavomaculata;Italy;GenBank;-;MT298616
Odonata;Libellulidae;Sympetrum;Sympetrum fonscolombii;Italy;GenBank;-;MT298652
Odonata;Libellulidae;Sympetrum;Sympetrum fonscolombii;Italy;GenBank;-;MT298653
Odonata;Libellulidae;Sympetrum;Sympetrum meridionale;Italy;GenBank;-;MT298655
Odonata;Cordulegastridae;Cordulegaster;Cordulegaster heros;Austria;GenBank;-;MW490316
Odonata;Cordulegastridae;Cordulegaster;Cordulegaster heros;Austria;GenBank;-;MW490369
Odonata;Coenagrionidae;Erythromma;Erythromma najas;Finland;GenBank;-;MZ657877
Odonata;Aeshnidae;Aeshna;Aeshna mixta;Russia;Bold;GBMNF49890-22;OM089771

Odonata;Aeshnidae;Aeshna;Aeshna mixta;Russia;GenBank;-;OM089772
Odonata;Aeshnidae;Aeshna;Aeshna mixta;Russia;Bold;GBMNF49891-22;OM089773
Odonata;Aeshnidae;Aeshna;Aeshna mixta;Russia;GenBank;-;OM089774
Odonata;Aeshnidae;Aeshna;Aeshna mixta;Russia;GenBank;-;OM089775
Odonata;Aeshnidae;Aeshna;Aeshna mixta;Russia;Bold;GBMNF49887-22;OM089776
Odonata;Aeshnidae;Aeshna;Aeshna affinis;Austria;Bold;AODON115-20;-
Odonata;Aeshnidae;Aeshna;Aeshna cyanea;Austria;Bold;AODON103-20;-
Odonata;Corduliidae;Cordulia;Cordulia aenea;Austria;Bold;AODON002-20;-
Odonata;Aeshnidae;Anax;Anax imperator;United Kingdom;Bold;ANBIO100-19;-
Odonata;Calopterygidae;Calopteryx;Calopteryx splendens;Austria;Bold;AODON451-20;-
Odonata;Calopterygidae;Calopteryx;Calopteryx virgo;Austria;Bold;AODON014-20;-
Odonata;Coenagrionidae;Coenagrion;Coenagrion puella;United Kingdom;Bold;ANBIO202-19;-
Odonata;Coenagrionidae;Coenagrion;Coenagrion pulchellum;Austria;Bold;AODON004-20;-
Odonata;Lestidae;Lestes;Lestes sponsa;Austria;Bold;AODON117-20;-
Odonata;Coenagrionidae;Enallagma;Enallagma cyathigerum;Austria;Bold;AODON025-20;-
Odonata;Coenagrionidae;Erythromma;Erythromma najas;Austria;Bold;AODON032-20;-
Odonata;Gomphidae;Gomphus;Gomphus vulgatissimus;Austria;Bold;AODON436-20;-
Odonata;Coenagrionidae;Ischnura;Ischnura elegans;United Kingdom;Bold;ANBIO150-19;-
Odonata;Coenagrionidae;Ischnura;Ischnura elegans;United Kingdom;Bold;ANBIO203-19;-
Odonata;Coenagrionidae;Ischnura;Ischnura elegans;United Kingdom;Bold;ANBIO195-19;-
Odonata;Libellulidae;Libellula;Libellula depressa;United Kingdom;Bold;ANBIO098-19;-
Odonata;Lestidae;Sympecma;Sympecma fusca;Austria;Bold;AODON006-20;-
Odonata;Libellulidae;Libellula;Libellula quadrimaculata;Austria;Bold;AODON001-20;-
Odonata;Libellulidae;Orthetrum;Orthetrum brunneum;Austria;Bold;AODON454-20;-
Odonata;Libellulidae;Orthetrum;Orthetrum cancellatum;Austria;Bold;AODON102-20;-
Odonata;Coenagrionidae;Pyrrhosoma;Pyrrhosoma nymphula;Austria;Bold;AODON003-20;-
Odonata;Libellulidae;Sympetrum;Sympetrum striolatum;United Kingdom;Bold;ANBIO101-19;-
Odonata;Libellulidae;Sympetrum;Sympetrum striolatum;Austria;Bold;AODON139-20;-
Coleoptera;Lucanidae;Platycerus;Platycerus caprea;Russia;GenBank;-;AB481434
Coleoptera;Lucanidae;Platycerus;Platycerus caraboides;France;GenBank;-;AB481437
Coleoptera;Ciidae;Cis;Cis setiger;France;GenBank;-;FM877939
Coleoptera;Ciidae;Sulcacis;Sulcacis affinis;Germany;GenBank;-;FM877947
Coleoptera;Curculionidae;Onyxacalles;Onyxacalles pyrenaeus;France;GenBank;-;GU981470
Coleoptera;Curculionidae;Kyklioacalles;Kyklioacalles punctaticollis;France;Bold;GBCL11317-12;GU981489
Coleoptera;Curculionidae;Kyklioacalles;Kyklioacalles roboris;Andorra;GenBank;-;GU981498
Coleoptera;Curculionidae;Onyxacalles;Onyxacalles pyrenaeus;France;GenBank;-;GU987762
Coleoptera;Curculionidae;Acallocrates;Acallocrates denticollis;Greece;GenBank;-;GU987875
Coleoptera;Curculionidae;Kyklioacalles;Kyklioacalles punctaticollis;France;Bold;GBMIN29889-13;GU987893
Coleoptera;Curculionidae;Dichromacalles;Dichromacalles rolletii;France;GenBank;-;GU987901
Coleoptera;Pyrochroidae;Pyrochroa;Pyrochroa serraticornis;United Kingdom;Bold;SPMIS348-22;HG995162.1
Coleoptera;Buprestidae;Agrilus;Agrilus graminis;Spain;GenBank;-;HM386452
Coleoptera;Buprestidae;Agrilus;Agrilus viridis;France;GenBank;-;HM417706
Coleoptera;Buprestidae;Agrilus;Agrilus biguttatus;Greece;GenBank;-;HM417714
Coleoptera;Buprestidae;Anthaxia;Anthaxia quadripunctata;Finland;GenBank;-;HM909032
Coleoptera;Cerambycidae;Callidium;Callidium aeneum;Finland;GenBank;-;HM909033
Coleoptera;Cerambycidae;Paracorymbia;Paracorymbia maculicornis;Finland;GenBank;-;HM909037
Coleoptera;Pyrochroidae;Pyrochroa;Pyrochroa coccinea;Finland;GenBank;-;HM909042
Coleoptera;Latridiidae;Cortinicara;Cortinicara gibbosa;Finland;GenBank;-;HM909057
Coleoptera;Staphylinidae;Dinaraea;Dinaraea aequata;Finland;GenBank;-;HM909061
Coleoptera;Mycetophagidae;Litargus;Litargus connexus;Finland;GenBank;-;HM909071
Coleoptera;Mycetophagidae;Mycetophagus;Mycetophagus piceus;Finland;GenBank;-;HM909072
Coleoptera;Zopheridae;Bitoma;Bitoma crenata;Finland;GenBank;-;HM909091
Coleoptera;Tetratomidae;Tetratoma;Tetratoma fungorum;Finland;GenBank;-;HM909108
Coleoptera;Latridiidae;Corticaria;Corticaria abietorum;Finland;Bold;COLFA101-10;HQ559228
Coleoptera;Histeridae;Dendrophilus;Dendrophilus pygmaeus;Finland;GenBank;-;HQ559231
Coleoptera;Curculionidae;Trachodes;Trachodes hispidus;Finland;GenBank;-;HQ559236
Coleoptera;Cerambycidae;Aromia;Aromia moschata;Finland;GenBank;-;HQ559240
Coleoptera;Cerambycidae;Arhopalus;Arhopalus rusticus;Finland;GenBank;-;HQ559250
Coleoptera;Cerambycidae;Judolia;Judolia sexmaculata;Finland;GenBank;-;HQ559267
Coleoptera;Latridiidae;Melanophthalma;Melanophthalma curticollis;Germany;GenBank;-;HQ563322
Coleoptera;Nitidulidae;Epuraea;Epuraea melanocephala;Germany;GenBank;-;HQ948248
Coleoptera;Lymexylidae;Elateroides;Elateroides dermestoides;Germany;Bold;FBCOC023-10;HQ954462
Coleoptera;Buprestidae;Anthaxia;Anthaxia helvetica;Germany;GenBank;-;HQ954476
Coleoptera;Lymexylidae;Elateroides;Elateroides dermestoides;Germany;Bold;FBCOC118-10;HQ954546
Coleoptera;Buprestidae;Agrilus;Agrilus viridis;Germany;GenBank;-;HQ954579
Coleoptera;Cerambycidae;Spondylis;Spondylis buprestoides;Finland;GenBank;-;JN284885
Coleoptera;Ptiliidae;Ptenidium;Ptenidium pusillum;Finland;GenBank;-;JN297959
Coleoptera;Ciidae;Cis;Cis bidentatus;Finland;GenBank;-;JN297960
Coleoptera;Cerambycidae;Herophila;Herophila tristis;Italy;GenBank;-;JX969627
Coleoptera;Scarabaeidae;Calicnemis;Calicnemis sardiniensis;Italy;GenBank;-;KJ492956
Coleoptera;Buprestidae;Agrilus;Agrilus betuleti;Finland;GenBank;-;KJ768192
Coleoptera;Curculionidae;Dichromacalles;Dichromacalles rolletii;Greece;GenBank;-;KJ867575
Coleoptera;Oedemeridae;Chrysanthia;Chrysanthia viridissima;Finland;GenBank;-;KJ961741
Coleoptera;Nitidulidae;Epuraea;Epuraea placida;Finland;GenBank;-;KJ961742
Coleoptera;Mycetophagidae;Typhaea;Typhaea stercorea;Finland;GenBank;-;KJ961744

Coleoptera;Staphylinidae;Leptusa;Leptusa fumida;Finland;GenBank;-;KJ961779
Coleoptera;Anthribidae;Dissoleucas;Dissoleucas niveirostris;Finland;GenBank;-;KJ961782
Coleoptera;Ciidae;Cis;Cis boleti;Finland;GenBank;-;KJ961832
Coleoptera;Elateridae;Ampedus;Ampedus erythrogonus;Finland;GenBank;-;KJ961839
Coleoptera;Ptiliidae;Ptenidium;Ptenidium punctatum;Finland;GenBank;-;KJ961842
Coleoptera;Cerambycidae;Pachyta;Pachyta quadrimaculata;Finland;GenBank;-;KJ961852
Coleoptera;Ptinidae;Cacotemnus;Cacotemnus rufipes;Finland;GenBank;-;KJ961900
Coleoptera;Mycetaeidae;Mycetaea;Mycetaea subterranea;Sweden;GenBank;-;KJ961953
Coleoptera;Monotomidae;Monotoma;Monotoma picipes;Finland;GenBank;-;KJ961980
Coleoptera;Cerambycidae;Gaurotes;Gaurotes virginea;Finland;GenBank;-;KJ961983
Coleoptera;Cerambycidae;Callidium;Callidium aeneum;Finland;GenBank;-;KJ962004
Coleoptera;Tenebrionidae;Mycetochara;Mycetochara axillaris;Finland;GenBank;-;KJ962021
Coleoptera;Tenebrionidae;Diaperis;Diaperis boleti;Finland;GenBank;-;KJ962024
Coleoptera;Ptiliidae;Ptenidium;Ptenidium formicetorum;Sweden;GenBank;-;KJ962041
Coleoptera;Monotomidae;Rhizophagus;Rhizophagus bipustulatus;Finland;GenBank;-;KJ962045
Coleoptera;Zopheridae;Bitoma;Bitoma crenata;Finland;GenBank;-;KJ962118
Coleoptera;Staphylinidae;Scaphidium;Scaphidium quadrimaculatum;Finland;GenBank;-;KJ962145
Coleoptera;Clambidae;Clambus;Clambus armadillo;Finland;Bold;COLFC444-12;KJ962157
Coleoptera;Zopheridae;Bitoma;Bitoma crenata;Finland;GenBank;-;KJ962176
Coleoptera;Latridiidae;Corticaria;Corticaria crenulata;Finland;GenBank;-;KJ962232
Coleoptera;Ciidae;Cis;Cis boleti;Finland;GenBank;-;KJ962322
Coleoptera;Cerambycidae;Pachyta;Pachyta quadrimaculata;Finland;GenBank;-;KJ962331
Coleoptera;Nitidulidae;Epuraea;Epuraea unicolor;Finland;GenBank;-;KJ962357
Coleoptera;Tetratomidae;Hallomenus;Hallomenus binotatus;Finland;GenBank;-;KJ962378
Coleoptera;Cerambycidae;Molorchus;Molorchus minor;Finland;GenBank;-;KJ962384
Coleoptera;Leiodidae;Agathidium;Agathidium seminulum;Finland;GenBank;-;KJ962411
Coleoptera;Aderidae;Anidorus;Anidorus nigrinus;Finland;GenBank;-;KJ962434
Coleoptera;Cerambycidae;Gaurotes;Gaurotes virginea;Finland;GenBank;-;KJ962469
Coleoptera;Ptiliidae;Ptenidium;Ptenidium formicetorum;Sweden;GenBank;-;KJ962489
Coleoptera;Ptiliidae;Ptilium;Ptilium exaratum;Finland;GenBank;-;KJ962499
Coleoptera;Latridiidae;Cortinicara;Cortinicara gibbosa;Finland;GenBank;-;KJ962500
Coleoptera;Buprestidae;Buprestis;Buprestis haemorrhoidalis;Finland;GenBank;-;KJ962529
Coleoptera;Buprestidae;Phaenops;Phaenops cyanea;Finland;GenBank;-;KJ962530
Coleoptera;Sphindidae;Sphindus;Sphindus dubius;Finland;GenBank;-;KJ962540
Coleoptera;Staphylinidae;Trimium;Trimium brevicorne;Finland;GenBank;-;KJ962546
Coleoptera;Cerambycidae;Rutpela;Rutpela maculata;Finland;GenBank;-;KJ962567
Coleoptera;Ptinidae;Anobium;Anobium punctatum;Finland;GenBank;-;KJ962574
Coleoptera;Buprestidae;Phaenops;Phaenops cyanea;Finland;GenBank;-;KJ962596
Coleoptera;Nitidulidae;Cychramus;Cychramus luteus;Finland;GenBank;-;KJ962607
Coleoptera;Mycetophagidae;Litargus;Litargus connexus;Finland;GenBank;-;KJ962610
Coleoptera;Buprestidae;Phaenops;Phaenops cyanea;Finland;GenBank;-;KJ962616
Coleoptera;Tenebrionidae;Diaperis;Diaperis boleti;Finland;GenBank;-;KJ962624
Coleoptera;Scarabaeidae;Protaetia;Protaetia cuprea;Finland;GenBank;-;KJ962659
Coleoptera;Buprestidae;Anthaxia;Anthaxia quadripunctata;Finland;GenBank;-;KJ962721
Coleoptera;Cerambycidae;Callidium;Callidium violaceum;Finland;GenBank;-;KJ962734
Coleoptera;Ptinidae;Ptinomorphus;Ptinomorphus imperialis;Finland;GenBank;-;KJ962811
Coleoptera;Pyrochroidae;Schizotus;Schizotus pectinicornis;Finland;GenBank;-;KJ962829
Coleoptera;Cerambycidae;Stenurella;Stenurella melanura;Finland;GenBank;-;KJ962863
Coleoptera;Mycetophagidae;Typhaea;Typhaea stercorea;Finland;GenBank;-;KJ962864
Coleoptera;Ptinidae;Hadrobregmus;Hadrobregmus pertinax;Finland;GenBank;-;KJ962879
Coleoptera;Scarabaeidae;Trichius;Trichius fasciatus;Finland;GenBank;-;KJ962927
Coleoptera;Pyrochroidae;Schizotus;Schizotus pectinicornis;Finland;GenBank;-;KJ962969
Coleoptera;Latridiidae;Corticaria;Corticaria impressa;Finland;GenBank;-;KJ962971
Coleoptera;Cerambycidae;Gaurotes;Gaurotes virginea;Finland;GenBank;-;KJ963065
Coleoptera;Cerambycidae;Paracorymbia;Paracorymbia maculicornis;Finland;GenBank;-;KJ963072
Coleoptera;Mycetaeidae;Mycetaea;Mycetaea subterranea;Sweden;GenBank;-;KJ963108
Coleoptera;Nitidulidae;Glischrochilus;Glischrochilus hortensis;Finland;GenBank;-;KJ963141
Coleoptera;Tenebrionidae;Pentaphyllus;Pentaphyllus testaceus;Sweden;GenBank;-;KJ963147
Coleoptera;Cerambycidae;Leiopus;Leiopus nebulosus;Finland;GenBank;-;KJ963149
Coleoptera;Curculionidae;Rhyncolus;Rhyncolus ater;Finland;GenBank;-;KJ963150
Coleoptera;Silvanidae;Silvanus;Silvanus bidentatus;Finland;GenBank;-;KJ963197
Coleoptera;Cryptophagidae;Atomaria;Atomaria nigrirostris;Finland;GenBank;-;KJ963261
Coleoptera;Staphylinidae;Phloeonomus;Phloeonomus punctipennis;Finland;GenBank;-;KJ963293
Coleoptera;Cerambycidae;Rhagium;Rhagium mordax;Finland;GenBank;-;KJ963320
Coleoptera;Endomychidae;Endomychus;Endomychus coccineus;Finland;GenBank;-;KJ963347
Coleoptera;Eucnemidae;Melasis;Melasis buprestoides;Sweden;GenBank;-;KJ963367
Coleoptera;Scraptiidae;Anaspis;Anaspis rufilabris;Finland;GenBank;-;KJ963370
Coleoptera;Dermestidae;Ctesias;Ctesias serra;Finland;GenBank;-;KJ963385
Coleoptera;Latridiidae;Corticaria;Corticaria elongata;Finland;GenBank;-;KJ963401
Coleoptera;Cerambycidae;Gaurotes;Gaurotes virginea;Finland;GenBank;-;KJ963449
Coleoptera;Corylophidae;Orthoperus;Orthoperus corticalis;Finland;GenBank;-;KJ963453
Coleoptera;Buprestidae;Anthaxia;Anthaxia quadripunctata;Finland;GenBank;-;KJ963461
Coleoptera;Buprestidae;Buprestis;Buprestis octoguttata;Finland;GenBank;-;KJ963469
Coleoptera;Leiodidae;Anisotoma;Anisotoma axillaris;Finland;GenBank;-;KJ963521
Coleoptera;Curculionidae;Pissodes;Pissodes pini;Finland;GenBank;-;KJ963528
Coleoptera;Nitidulidae;Epuraea;Epuraea pallescens;Finland;GenBank;-;KJ963551
Coleoptera;Tetratomidae;Hallomenus;Hallomenus axillaris;Finland;GenBank;-;KJ963661
Coleoptera;Mordellidae;Mordellistena;Mordellistena humeralis;Finland;GenBank;-;KJ963672

Coleoptera;Tenebrionidae;Bolitophagus;Bolitophagus reticulatus;Estonia;GenBank;-;KJ963690
Coleoptera;Ptinidae;Dorcatoma;Dorcatoma chrysomelina;Sweden;GenBank;-;KJ963701
Coleoptera;Cerambycidae;Stenurella;Stenurella melanura;Finland;GenBank;-;KJ963709
Coleoptera;Erotylidae;Triplax;Triplax rufipes;Finland;GenBank;-;KJ963715
Coleoptera;Staphylinidae;Scaphidium;Scaphidium quadrimaculatum;Finland;GenBank;-;KJ963785
Coleoptera;Ptinidae;Dorcatoma;Dorcatoma chrysomelina;Sweden;GenBank;-;KJ963787
Coleoptera;Ciidae;Sulcacis;Sulcacis fronticornis;Finland;GenBank;-;KJ963808
Coleoptera;Curculionidae;Trachodes;Trachodes hispidus;Finland;GenBank;-;KJ963848
Coleoptera;Elateridae;Melanotus;Melanotus villosus;Estonia;GenBank;-;KJ963853
Coleoptera;Mycetophagidae;Litargus;Litargus connexus;Finland;GenBank;-;KJ963855
Coleoptera;Cerambycidae;Aromia;Aromia moschata;Finland;GenBank;-;KJ963928
Coleoptera;Aderidae;Anidorus;Anidorus nigrinus;Finland;GenBank;-;KJ963945
Coleoptera;Ptiliidae;Ptilium;Ptilium exaratum;Finland;GenBank;-;KJ963999
Coleoptera;Sphindidae;Sphindus;Sphindus dubius;Finland;GenBank;-;KJ964012
Coleoptera;Latridiidae;Corticaria;Corticaria impressa;Finland;GenBank;-;KJ964037
Coleoptera;Buprestidae;Anthaxia;Anthaxia quadripunctata;Finland;GenBank;-;KJ964057
Coleoptera;Nitidulidae;Epuraea;Epuraea variegata;Finland;GenBank;-;KJ964076
Coleoptera;Cerambycidae;Stenurella;Stenurella melanura;Finland;GenBank;-;KJ964096
Coleoptera;Latridiidae;Cortinicara;Cortinicara gibbosa;Finland;GenBank;-;KJ964106
Coleoptera;Curculionidae;Rhyncolus;Rhyncolus ater;Finland;GenBank;-;KJ964123
Coleoptera;Latridiidae;Cartodere;Cartodere nodifer;Finland;GenBank;-;KJ964161
Coleoptera;Scraptiidae;Anaspis;Anaspis rufilabris;Finland;GenBank;-;KJ964162
Coleoptera;Ptinidae;Ptilinus;Ptilinus fuscus;Finland;GenBank;-;KJ964190
Coleoptera;Erotylidae;Triplax;Triplax aenea;Finland;GenBank;-;KJ964262
Coleoptera;Monotomidae;Rhizophagus;Rhizophagus bipustulatus;Finland;GenBank;-;KJ964265
Coleoptera;Cerambycidae;Tetropium;Tetropium fuscum;Finland;GenBank;-;KJ964282
Coleoptera;Cerambycidae;Pogonocherus;Pogonocherus hispidus;Finland;GenBank;-;KJ964297
Coleoptera;Elateridae;Ampedus;Ampedus pomorum;Estonia;GenBank;-;KJ964300
Coleoptera;Elateridae;Ampedus;Ampedus balteatus;Finland;GenBank;-;KJ964318
Coleoptera;Mycetophagidae;Litargus;Litargus connexus;Finland;GenBank;-;KJ964319
Coleoptera;Cerambycidae;Pogonocherus;Pogonocherus fasciculatus;Finland;GenBank;-;KJ964368
Coleoptera;Erotylidae;Tritoma;Tritoma bipustulata;Finland;GenBank;-;KJ964373
Coleoptera;Staphylinidae;Homalota;Homalota plana;Finland;GenBank;-;KJ964375
Coleoptera;Cerambycidae;Arhopalus;Arhopalus rusticus;Finland;GenBank;-;KJ964380
Coleoptera;Cerambycidae;Gaurotes;Gaurotes virginea;Finland;GenBank;-;KJ964393
Coleoptera;Nitidulidae;Glischrochilus;Glischrochilus hortensis;Finland;GenBank;-;KJ964414
Coleoptera;Elateridae;Ampedus;Ampedus balteatus;Finland;GenBank;-;KJ964419
Coleoptera;Curculionidae;Pityogenes;Pityogenes bidentatus;Finland;GenBank;-;KJ964432
Coleoptera;Cerylonidae;Cerylon;Cerylon histeroides;Finland;GenBank;-;KJ964507
Coleoptera;Cerambycidae;Tetropium;Tetropium castaneum;Finland;GenBank;-;KJ964513
Coleoptera;Staphylinidae;Atrecus;Atrecus affinis;Finland;GenBank;-;KJ964531
Coleoptera;Cerambycidae;Spondylis;Spondylis buprestoides;Finland;GenBank;-;KJ964556
Coleoptera;Lycidae;Dictyoptera;Dictyoptera aurora;Finland;GenBank;-;KJ964562
Coleoptera;Cerambycidae;Leiopus;Leiopus nebulosus;Finland;GenBank;-;KJ964583
Coleoptera;Sphindidae;Aspidiphorus;Aspidiphorus orbiculatus;Finland;GenBank;-;KJ964585
Coleoptera;Nitidulidae;Epuraea;Epuraea pygmaea;Finland;GenBank;-;KJ964595
Coleoptera;Mycetophagidae;Litargus;Litargus connexus;Finland;GenBank;-;KJ964606
Coleoptera;Erotylidae;Tritoma;Tritoma bipustulata;Finland;GenBank;-;KJ964727
Coleoptera;Melyridae;Dasytes;Dasytes plumbeus;Finland;GenBank;-;KJ964773
Coleoptera;Oedemeridae;Chrysanthia;Chrysanthia viridissima;Sweden;GenBank;-;KJ964774
Coleoptera;Cerambycidae;Pachyta;Pachyta quadrimaculata;Finland;GenBank;-;KJ964790
Coleoptera;Cerambycidae;Phymatodes;Phymatodes testaceus;Finland;GenBank;-;KJ964796
Coleoptera;Ptinidae;Hadrobregmus;Hadrobregmus pertinax;Finland;GenBank;-;KJ964816
Coleoptera;Nitidulidae;Epuraea;Epuraea unicolor;Finland;GenBank;-;KJ964833
Coleoptera;Mycetophagidae;Mycetophagus;Mycetophagus populi;Finland;GenBank;-;KJ964858
Coleoptera;Buprestidae;Dicerca;Dicerca moesta;Finland;GenBank;-;KJ964870
Coleoptera;Buprestidae;Dicerca;Dicerca moesta;Finland;GenBank;-;KJ964877
Coleoptera;Nitidulidae;Epuraea;Epuraea variegata;Estonia;GenBank;-;KJ964916
Coleoptera;Scarabaeidae;Trichius;Trichius fasciatus;Finland;GenBank;-;KJ964922
Coleoptera;Staphylinidae;Quedius;Quedius brevicornis;Finland;GenBank;-;KJ964951
Coleoptera;Elateridae;Ampedus;Ampedus pomonae;Finland;GenBank;-;KJ964958
Coleoptera;Cerambycidae;Saperda;Saperda carcharias;Finland;GenBank;-;KJ964971
Coleoptera;Curculionidae;Ips;Ips acuminatus;Finland;GenBank;-;KJ965012
Coleoptera;Cerambycidae;Monochamus;Monochamus sutor;Finland;GenBank;-;KJ965030
Coleoptera;Staphylinidae;Quedius;Quedius scitus;Finland;GenBank;-;KJ965037
Coleoptera;Cerambycidae;Molorchus;Molorchus minor;Finland;GenBank;-;KJ965045
Coleoptera;Cerambycidae;Stenurella;Stenurella melanura;Finland;GenBank;-;KJ965077
Coleoptera;Aderidae;Anidorus;Anidorus nigrinus;Finland;GenBank;-;KJ965104
Coleoptera;Endomychidae;Endomychus;Endomychus coccineus;Finland;GenBank;-;KJ965118
Coleoptera;Cerambycidae;Stictoleptura;Stictoleptura rubra;Finland;GenBank;-;KJ965133
Coleoptera;Salpingidae;Salpingus;Salpingus planirostris;Finland;GenBank;-;KJ965219
Coleoptera;Curculionidae;Cryptorhynchus;Cryptorhynchus lapathi;Finland;GenBank;-;KJ965256
Coleoptera;Tenebrionidae;Pseudocistela;Pseudocistela ceramboides;Finland;GenBank;-;KJ965267
Coleoptera;Staphylinidae;Quedius;Quedius cruentus;Finland;GenBank;-;KJ965301
Coleoptera;Leiodidae;Anisotoma;Anisotoma orbicularis;Finland;GenBank;-;KJ965302
Coleoptera;Buprestidae;Dicerca;Dicerca moesta;Finland;GenBank;-;KJ965352
Coleoptera;Cerambycidae;Pachyta;Pachyta quadrimaculata;Finland;GenBank;-;KJ965366
Coleoptera;Elateridae;Denticollis;Denticollis linearis;Finland;GenBank;-;KJ965420

Coleoptera;Scraptiidae;Anaspis;Anaspis rufilabris;Finland;GenBank;-;KJ965432
Coleoptera;Elateridae;Ampedus;Ampedus balteatus;Finland;GenBank;-;KJ965448
Coleoptera;Cerambycidae;Saperda;Saperda carcharias;Finland;GenBank;-;KJ965483
Coleoptera;Staphylinidae;Dinaraea;Dinaraea aequata;Finland;GenBank;-;KJ965484
Coleoptera;Mordellidae;Mordellochroa;Mordellochroa abdominalis;Finland;GenBank;-;KJ965600
Coleoptera;Curculionidae;Cryphalus;Cryphalus saltuarius;Finland;GenBank;-;KJ965606
Coleoptera;Cerambycidae;Alosterna;Alosterna tabacicolor;Finland;GenBank;-;KJ965615
Coleoptera;Cerambycidae;Tetrops;Tetrops starkii;Estonia;GenBank;-;KJ965662
Coleoptera;Nitidulidae;Epuraea;Epuraea marseuli;Finland;GenBank;-;KJ965797
Coleoptera;Staphylinidae;Phloeostiba;Phloeostiba plana;Finland;GenBank;-;KJ965808
Coleoptera;Staphylinidae;Atheta;Atheta picipes;Finland;GenBank;-;KJ965885
Coleoptera;Latridiidae;Cartodere;Cartodere constricta;Finland;GenBank;-;KJ965886
Coleoptera;Latridiidae;Cartodere;Cartodere constricta;Finland;GenBank;-;KJ965912
Coleoptera;Oedemeridae;Chrysanthia;Chrysanthia viridissima;Finland;GenBank;-;KJ965923
Coleoptera;Ciidae;Cis;Cis glabratus;Estonia;GenBank;-;KJ965985
Coleoptera;Elateridae;Stenagostus;Stenagostus rhombeus;Sweden;GenBank;-;KJ966071
Coleoptera;Staphylinidae;Phloeostiba;Phloeostiba plana;Finland;GenBank;-;KJ966112
Coleoptera;Staphylinidae;Homalota;Homalota plana;Finland;GenBank;-;KJ966141
Coleoptera;Erotylidae;Dacne;Dacne bipustulata;Finland;GenBank;-;KJ966156
Coleoptera;Staphylinidae;Sepedophilus;Sepedophilus testaceus;Finland;GenBank;-;KJ966168
Coleoptera;Ciidae;Cis;Cis dentatus;Finland;GenBank;-;KJ966197
Coleoptera;Rhadalidae;Aplocnemus;Aplocnemus nigricornis;Finland;GenBank;-;KJ966215
Coleoptera;Cerambycidae;Stenurella;Stenurella melanura;Finland;GenBank;-;KJ966244
Coleoptera;Erotylidae;Triplax;Triplax aenea;Finland;GenBank;-;KJ966247
Coleoptera;Curculionidae;Trachodes;Trachodes hispidus;Finland;GenBank;-;KJ966283
Coleoptera;Elateridae;Melanotus;Melanotus villosus;Estonia;GenBank;-;KJ966292
Coleoptera;Erotylidae;Dacne;Dacne bipustulata;Finland;GenBank;-;KJ966311
Coleoptera;Cerambycidae;Paracorymbia;Paracorymbia maculicornis;Finland;GenBank;-;KJ966333
Coleoptera;Cerambycidae;Hylotrupes;Hylotrupes bajulus;Finland;GenBank;-;KJ966347
Coleoptera;Mycetophagidae;Mycetophagus;Mycetophagus quadripustulatus;Finland;GenBank;-;KJ966425
Coleoptera;Erotylidae;Dacne;Dacne bipustulata;Finland;GenBank;-;KJ966473
Coleoptera;Staphylinidae;Quedius;Quedius brevicornis;Finland;GenBank;-;KJ966479
Coleoptera;Scraptiidae;Anaspis;Anaspis rufilabris;Finland;GenBank;-;KJ966509
Coleoptera;Lucanidae;Sinodendron;Sinodendron cylindricum;Finland;GenBank;-;KJ966511
Coleoptera;Elateridae;Ampedus;Ampedus pomorum;Finland;GenBank;-;KJ966520
Coleoptera;Curculionidae;Rhyncolus;Rhyncolus ater;Finland;GenBank;-;KJ966539
Coleoptera;Cerambycidae;Anoplodera;Anoplodera sexguttata;Sweden;GenBank;-;KJ966542
Coleoptera;Eucnemidae;Melasis;Melasis buprestoides;Sweden;GenBank;-;KJ966606
Coleoptera;Cerambycidae;Tetropium;Tetropium castaneum;Finland;GenBank;-;KJ966635
Coleoptera;Ciidae;Cis;Cis castaneus;Finland;GenBank;-;KJ966781
Coleoptera;Buprestidae;Phaenops;Phaenops cyanea;Finland;GenBank;-;KJ966789
Coleoptera;Cerambycidae;Pachyta;Pachyta quadrimaculata;Finland;GenBank;-;KJ966803
Coleoptera;Erotylidae;Dacne;Dacne bipustulata;Finland;GenBank;-;KJ966806
Coleoptera;Staphylinidae;Quedius;Quedius brevicornis;Finland;GenBank;-;KJ966821
Coleoptera;Nitidulidae;Cychramus;Cychramus luteus;Finland;GenBank;-;KJ966832
Coleoptera;Elateridae;Ampedus;Ampedus sanguinolentus;Finland;GenBank;-;KJ966894
Coleoptera;Elateridae;Ampedus;Ampedus pomorum;Finland;GenBank;-;KJ966895
Coleoptera;Cerambycidae;Saperda;Saperda carcharias;Finland;GenBank;-;KJ966951
Coleoptera;Oedemeridae;Chrysanthia;Chrysanthia viridissima;Finland;GenBank;-;KJ966956
Coleoptera;Scraptiidae;Anaspis;Anaspis rufilabris;Finland;GenBank;-;KJ966995
Coleoptera;Cerambycidae;Leiopus;Leiopus nebulosus;Finland;GenBank;-;KJ967008
Coleoptera;Staphylinidae;Atheta;Atheta picipes;Finland;GenBank;-;KJ967025
Coleoptera;Silvanidae;Silvanoprus;Silvanoprus fagi;Finland;GenBank;-;KJ967029
Coleoptera;Staphylinidae;Quedius;Quedius scitus;Finland;GenBank;-;KJ967052
Coleoptera;Tenebrionidae;Bolitophagus;Bolitophagus reticulatus;Finland;GenBank;-;KJ967060
Coleoptera;Tenebrionidae;Bolitophagus;Bolitophagus reticulatus;Finland;GenBank;-;KJ967071
Coleoptera;Histeridae;Abraeus;Abraeus perpusillus;Sweden;GenBank;-;KJ967075
Coleoptera;Cerambycidae;Stenurella;Stenurella melanura;Finland;GenBank;-;KJ967156
Coleoptera;Latridiidae;Corticaria;Corticaria elongata;Finland;GenBank;-;KJ967166
Coleoptera;Cerambycidae;Tetrops;Tetrops praeustus;Sweden;GenBank;-;KJ967190
Coleoptera;Scarabaeidae;Protaetia;Protaetia cuprea;Finland;GenBank;-;KJ967217
Coleoptera;Alexiidae;Sphaerosoma;Sphaerosoma pilosum;Finland;GenBank;-;KJ967241
Coleoptera;Cerambycidae;Tetrops;Tetrops praeustus;Finland;GenBank;-;KJ967255
Coleoptera;Scraptiidae;Anaspis;Anaspis rufilabris;Finland;GenBank;-;KJ967269
Coleoptera;Cerambycidae;Stictoleptura;Stictoleptura rubra;Finland;GenBank;-;KJ967273
Coleoptera;Cerambycidae;Paracorymbia;Paracorymbia maculicornis;Finland;GenBank;-;KJ967279
Coleoptera;Cerambycidae;Leiopus;Leiopus nebulosus;Finland;GenBank;-;KJ967320
Coleoptera;Staphylinidae;Tyrus;Tyrus mucronatus;Finland;GenBank;-;KJ967356
Coleoptera;Histeridae;Hololepta;Hololepta plana;Finland;GenBank;-;KJ967462
Coleoptera;Cerambycidae;Monochamus;Monochamus galloprovincialis;Finland;GenBank;-;KJ967472
Coleoptera;Cerambycidae;Alosterna;Alosterna tabacicolor;Finland;GenBank;-;KJ967479
Coleoptera;Elateridae;Ampedus;Ampedus balteatus;Finland;GenBank;-;KJ967483
Coleoptera;Cerambycidae;Molorchus;Molorchus minor;Finland;GenBank;-;KJ967492
Coleoptera;Monotomidae;Monotoma;Monotoma picipes;Finland;GenBank;-;KJ967512
Coleoptera;Curculionidae;Rhyncolus;Rhyncolus ater;Finland;GenBank;-;KJ967520
Coleoptera;Tenebrionidae;Pentaphyllus;Pentaphyllus testaceus;France;GenBank;-;KM285784
Coleoptera;Buprestidae;Anthaxia;Anthaxia umbellatarum;France;GenBank;-;KM285798
Coleoptera;Melandryidae;Conopalpus;Conopalpus brevicollis;France;GenBank;-;KM285804

Coleoptera;Pyrochroidae;Pyrochroa;Pyrochroa serraticornis;France;GenBank;-;KM285809
Coleoptera;Mycetophagidae;Mycetophagus;Mycetophagus piceus;France;GenBank;-;KM285810
Coleoptera;Oedemeridae;Ischnomera;Ischnomera cyanea;France;GenBank;-;KM285824
Coleoptera;Cerambycidae;Grammoptera;Grammoptera ustulata;France;GenBank;-;KM285829
Coleoptera;Elateridae;Ampedus;Ampedus pomonae;France;GenBank;-;KM285831
Coleoptera;Anthribidae;Platyrhinus;Platyrhinus resinosus;France;GenBank;-;KM285849
Coleoptera;Ptinidae;Hadrobregmus;Hadrobregmus denticollis;France;GenBank;-;KM285862
Coleoptera;Cerambycidae;Phymatodes;Phymatodes rufipes;France;GenBank;-;KM285863
Coleoptera;Pyrochroidae;Pyrochroa;Pyrochroa coccinea;France;GenBank;-;KM285880
Coleoptera;Tenebrionidae;Bolitophagus;Bolitophagus reticulatus;France;GenBank;-;KM285900
Coleoptera;Buprestidae;Chrysobothris;Chrysobothris affinis;France;GenBank;-;KM285981
Coleoptera;Lymexylidae;Elateroides;Elateroides dermestoides;France;Bold;PSFOR283-13;KM285992
Coleoptera;Cerambycidae;Prionus;Prionus coriarius;France;GenBank;-;KM286000
Coleoptera;Cerambycidae;Mesosa;Mesosa nebulosa;France;GenBank;-;KM286019
Coleoptera;Cleridae;Opilo;Opilo mollis;France;GenBank;-;KM286038
Coleoptera;Mycetophagidae;Triphyllus;Triphyllus bicolor;France;GenBank;-;KM286043
Coleoptera;Cerambycidae;Exocentrus;Exocentrus adspersus;France;GenBank;-;KM286059
Coleoptera;Lycidae;Dictyoptera;Dictyoptera aurora;France;GenBank;-;KM286113
Coleoptera;Cerambycidae;Dinoptera;Dinoptera collaris;France;GenBank;-;KM286140
Coleoptera;Zopheridae;Coxelus;Coxelus pictus;France;GenBank;-;KM286144
Coleoptera;Ptinidae;Hemicoelus;Hemicoelus canaliculatus;France;GenBank;-;KM286179
Coleoptera;Elateridae;Lacon;Lacon querceus;France;GenBank;-;KM286190
Coleoptera;Ptinidae;Xestobium;Xestobium rufovillosum;France;GenBank;-;KM286197
Coleoptera;Lymexylidae;Elateroides;Elateroides dermestoides;France;Bold;PSFOR1020-14;KM286217
Coleoptera;Cleridae;Thanasimus;Thanasimus femoralis;France;Bold;PSFOR628-13;KM286304
Coleoptera;Cerambycidae;Anoplodera;Anoplodera sexguttata;France;GenBank;-;KM286312
Coleoptera;Cerambycidae;Pyrrhidium;Pyrrhidium sanguineum;France;GenBank;-;KM286354
Coleoptera;Cerambycidae;Stenopterus;Stenopterus rufus;France;GenBank;-;KM286361
Coleoptera;Ptinidae;Ptilinus;Ptilinus pectinicornis;France;GenBank;-;KM286378
Coleoptera;Elateridae;Ampedus;Ampedus nigrinus;France;GenBank;-;KM286384
Coleoptera;Cleridae;Opilo;Opilo mollis;France;GenBank;-;KM286390
Coleoptera;Leiodidae;Agathidium;Agathidium atrum;Belgium;GenBank;-;KM439550
Coleoptera;Bostrichidae;Scobicia;Scobicia chevrieri;Slovenia;GenBank;-;KM440069
Coleoptera;Buprestidae;Agrilus;Agrilus viridis;Germany;GenBank;-;KM440395
Coleoptera;Cerambycidae;Stictoleptura;Stictoleptura cordigera;Greece;GenBank;-;KM441073
Coleoptera;Cerambycidae;Grammoptera;Grammoptera ustulata;Czech Republic;GenBank;-;KM443781
Coleoptera;Cerambycidae;Rutpela;Rutpela maculata;Slovenia;GenBank;-;KM444341
Coleoptera;Mordellidae;Mordellochroa;Mordellochroa abdominalis;Germany;GenBank;-;KM445539
Coleoptera;Lymexylidae;Elateroides;Elateroides dermestoides;Germany;Bold;FBCOD1210-11;KM446388
Coleoptera;Elateridae;Denticollis;Denticollis linearis;Germany;GenBank;-;KM447495
Coleoptera;Cerambycidae;Exocentrus;Exocentrus punctipennis;Slovenia;GenBank;-;KM448299
Coleoptera;Scarabaeidae;Valgus;Valgus hemipterus;Greece;GenBank;-;KM448345
Coleoptera;Pyrochroidae;Pyrochroa;Pyrochroa serraticornis;Germany;GenBank;-;KM448454
Coleoptera;Buprestidae;Anthaxia;Anthaxia helvetica;Slovenia;GenBank;-;KM448847
Coleoptera;Scarabaeidae;Trichius;Trichius sexualis;Slovenia;GenBank;-;KM448908
Coleoptera;Staphylinidae;Dinaraea;Dinaraea aequata;Belgium;GenBank;-;KM449008
Coleoptera;Buprestidae;Agrilus;Agrilus viridis;Germany;GenBank;-;KM450118
Coleoptera;Staphylinidae;Ischnoglossa;Ischnoglossa prolixa;Belgium;GenBank;-;KM450786
Coleoptera;Staphylinidae;Quedius;Quedius maurus;Belgium;GenBank;-;KM450789
Coleoptera;Lymexylidae;Elateroides;Elateroides dermestoides;France;Bold;GBCOU279-13;KM451813
Coleoptera;Buprestidae;Chrysobothris;Chrysobothris affinis;Greece;GenBank;-;KM451934
Coleoptera;Lymexylidae;Elateroides;Elateroides dermestoides;Austria;Bold;GBCOU2419-13;KM452358
Coleoptera;Buprestidae;Agrilus;Agrilus sulcicollis;Slovakia;GenBank;-;KT250459
Coleoptera;Curculionidae;Kyklioacalles;Kyklioacalles punctaticollis;Spain;Bold;GBCL46441-19;KU170190
Coleoptera;Ptinidae;Anobium;Anobium punctatum;France;GenBank;-;KU494028
Coleoptera;Ptinidae;Nicobium;Nicobium castaneum;Germany;GenBank;-;KU494146
Coleoptera;Ptiliidae;Priobium;Priobium carpini;France;GenBank;-;KU494206
Coleoptera;Buprestidae;Anthaxia;Anthaxia nitidula;Germany;GenBank;-;KU906462
Coleoptera;Staphylinidae;Atrecus;Atrecus affinis;France;GenBank;-;KU906787
Coleoptera;Buprestidae;Anthaxia;Anthaxia nitidula;Germany;GenBank;-;KU907409
Coleoptera;Tenebrionidae;Cteniopus;Cteniopus sulphureus;Slovenia;GenBank;-;KU907781
Coleoptera;Buprestidae;Anthaxia;Anthaxia nitidula;Germany;GenBank;-;KU908183
Coleoptera;Scraptiidae;Anaspis;Anaspis pulicaria;Italy;GenBank;-;KU908772
Coleoptera;Buprestidae;Agrilus;Agrilus viridis;Germany;GenBank;-;KU909316
Coleoptera;Scarabaeidae;Cetonia;Cetonia aurata;Germany;GenBank;-;KU909732
Coleoptera;Curculionidae;Ips;Ips acuminatus;Germany;GenBank;-;KU910832
Coleoptera;Buprestidae;Agrilus;Agrilus viridis;Germany;GenBank;-;KU910885
Coleoptera;Lucanidae;Dorcus;Dorcus parallelipipedus;Germany;GenBank;-;KU911240
Coleoptera;Buprestidae;Anthaxia;Anthaxia nitidula;Germany;GenBank;-;KU913055
Coleoptera;Scraptiidae;Anaspis;Anaspis costai;Germany;GenBank;-;KU914019
Coleoptera;Scraptiidae;Anaspis;Anaspis pulicaria;Italy;GenBank;-;KU914057
Coleoptera;Buprestidae;Agrilus;Agrilus viridis;Germany;GenBank;-;KU915192
Coleoptera;Buprestidae;Agrilus;Agrilus viridis;Germany;GenBank;-;KU915193
Coleoptera;Scraptiidae;Anaspis;Anaspis costai;Germany;GenBank;-;KU915666
Coleoptera;Cerambycidae;Stictoleptura;Stictoleptura rubra;Germany;Bold;GCOL560-16;KU916542
Coleoptera;Tenebrionidae;Uloma;Uloma culinaris;Slovenia;GenBank;-;KU916659
Coleoptera;Curculionidae;Ips;Ips acuminatus;Germany;GenBank;-;KU916950

Coleoptera;Scarabaeidae;Oryctes;Oryctes nasicornis;Russia;GenBank;-;MF115595
Coleoptera;Cerambycidae;Cerambyx;Cerambyx miles;Greece;GenBank;-;MF591720
Coleoptera;Pyrochroidae;Schizotus;Schizotus pectinicornis;Russia;GenBank;-;MF776965
Coleoptera;Buprestidae;Agrilus;Agrilus graminis;Slovakia;GenBank;-;MF805187
Coleoptera;Buprestidae;Agrilus;Agrilus angustulus;Slovakia;GenBank;-;MF805200
Coleoptera;Buprestidae;Agrilus;Agrilus graminis;Slovakia;GenBank;-;MF805218
Coleoptera;Buprestidae;Agrilus;Agrilus graminis;Slovakia;GenBank;-;MF805248
Coleoptera;Curculionidae;Dichromacalles;Dichromacalles rolletii;Italy;GenBank;-;MG229717
Coleoptera;Curculionidae;Kyklioacalles;Acalles aubei;Russia;GenBank;-;MG229797
Coleoptera;Curculionidae;Kyklioacalles;Acalles aubei;Russia;GenBank;-;MG229799
Coleoptera;Curculionidae;Mesites;Mesites pallidipennis;Ukraine;GenBank;-;MH618684
Coleoptera;Cucujidae;Cucujus;Cucujus haematodes;Poland;GenBank;-;MK315146
Coleoptera;Cucujidae;Cucujus;Cucujus haematodes;Poland;GenBank;-;MK315147
Coleoptera;Cucujidae;Cucujus;Cucujus haematodes;Poland;GenBank;-;MK315148
Coleoptera;Curculionidae;Ips;Ips sexdentatus;Spain;GenBank;-;MK643361
Coleoptera;Curculionidae;Ips;Ips sexdentatus;Spain;GenBank;-;MK643362
Coleoptera;Curculionidae;Ips;Ips sexdentatus;Spain;GenBank;-;MK643363
Coleoptera;Curculionidae;Ips;Ips sexdentatus;Spain;GenBank;-;MK643364
Coleoptera;Curculionidae;Ips;Ips sexdentatus;Spain;GenBank;-;MK643365
Coleoptera;Curculionidae;Ips;Ips sexdentatus;Spain;GenBank;-;MK643366
Coleoptera;Curculionidae;Ips;Ips sexdentatus;Spain;GenBank;-;MK643367
Coleoptera;Curculionidae;Ips;Ips sexdentatus;Spain;GenBank;-;MK643368
Coleoptera;Curculionidae;Ips;Ips sexdentatus;Spain;GenBank;-;MK643369
Coleoptera;Curculionidae;Ips;Ips sexdentatus;Spain;GenBank;-;MK643370
Coleoptera;Curculionidae;Ips;Ips sexdentatus;Spain;GenBank;-;MK643371
Coleoptera;Curculionidae;Dryocoetes;Dryocoetes villosus;France;Bold;PSFOR1195-17;MN182790
Coleoptera;Scarabaeidae;Protaetia;Protaetia cuprea;France;Bold;PSFOR1500-17;MN182806
Coleoptera;Elateridae;Melanotus;Melanotus villosus;France;GenBank;-;MN182811
Coleoptera;Buprestidae;Chrysobothris;Chrysobothris solieri;France;GenBank;-;MN182819
Coleoptera;Ptinidae;Hemicoelus;Hemicoelus costatus;France;GenBank;-;MN182876
Coleoptera;Histeridae;Gnathoncus;Gnathoncus rotundatus;France;GenBank;-;MN182882
Coleoptera;Curculionidae;Dryocoetes;Dryocoetes villosus;France;Bold;PSFOR1198-17;MN182899
Coleoptera;Alexiidae;Sphaerosoma;Sphaerosoma pilosum;France;GenBank;-;MN182911
Coleoptera;Tenebrionidae;Allecula;Allecula morio;France;GenBank;-;MN182939
Coleoptera;Oedemeridae;Nacerdes;Nacerdes melanura;France;GenBank;-;MN182958
Coleoptera;Tenebrionidae;Eledonoprius;Eledonoprius armatus;France;GenBank;-;MN182976
Coleoptera;Anobiinae;Homophthalmus;Homophthalmus rugicollis;France;GenBank;-;MN182990
Coleoptera;Melandryidae;Osphya;Osphya bipunctata;France;GenBank;-;MN183000
Coleoptera;Curculionidae;Dryocoetes;Dryocoetes villosus;France;Bold;PSFOR1194-17;MN183012
Coleoptera;Scarabaeidae;Protaetia;Protaetia cuprea;France;Bold;PSFOR1503-17;MN183018
Coleoptera;Ptinidae;Nicobium;Nicobium castaneum;France;GenBank;-;MN183033
Coleoptera;Buprestidae;Agrilus;Agrilus laticornis;France;GenBank;-;MN183041
Coleoptera;Oedemeridae;Nacerdes;Nacerdes melanura;France;GenBank;-;MN183047
Coleoptera;Scolytidae;Thamnurgus;Thamnurgus euphorbiae;Italy;GenBank;-;MN344663
Coleoptera;Staphylinidae;Quedius;Quedius truncicola;Germany;GenBank;-;MW259557
Coleoptera;Staphylinidae;Quedius;Quedius truncicola;Germany;GenBank;-;MW259777
Coleoptera;Staphylinidae;Anomognathus;Anomognathus cuspidatus;Finland;GenBank;-;MW661355
Coleoptera;Cerambycidae;Mesosa;Mesosa nebulosa;Slovakia;GenBank;-;MW982722
Coleoptera;Cerambycidae;Mesosa;Mesosa curculionoides;Hungary;GenBank;-;MW982828
Coleoptera;Clambidae;Clambus;Clambus pubescens;Finland;GenBank;-;MZ606858
Coleoptera;Anthribidae;Dissoleucas;Dissoleucas niveirostris;Finland;GenBank;-;MZ606968
Coleoptera;Tenebrionidae;Uloma;Uloma rufa;Finland;GenBank;-;MZ607099
Coleoptera;Cerambycidae;Semanotus;Semanotus undatus;Finland;GenBank;-;MZ607312
Coleoptera;Cerambycidae;Acmaeops;Acmaeops marginatus;Finland;GenBank;-;MZ607850
Coleoptera;Ciidae;Octotemnus;Octotemnus glabriculus;Finland;GenBank;-;MZ608414
Coleoptera;Cerambycidae;Pogonocherus;Pogonocherus hispidus;Finland;GenBank;-;MZ608704
Coleoptera;Melandryidae;Abdera;Abdera flexuosa;Finland;GenBank;-;MZ608807
Coleoptera;Curculionidae;Trypodendron;Trypodendron lineatum;Finland;GenBank;-;MZ609410
Coleoptera;Curculionidae;Trypodendron;Trypodendron domesticum;Finland;GenBank;-;MZ609439
Coleoptera;Staphylinidae;Phloeocharis;Phloeocharis subtilissima;Finland;GenBank;-;MZ609627
Coleoptera;Clambidae;Clambus;Clambus pubescens;Finland;GenBank;-;MZ610301
Coleoptera;Leiodidae;Agathidium;Agathidium atrum;Finland;GenBank;-;MZ610690
Coleoptera;Monotomidae;Monotoma;Monotoma longicollis;Finland;GenBank;-;MZ610851
Coleoptera;Eucnemidae;Microrhagus;Microrhagus pygmaeus;Finland;GenBank;-;MZ610895
Coleoptera;Lophocateridae;Grynocharis;Grynocharis oblonga;Finland;GenBank;-;MZ611151
Coleoptera;Cleridae;Tillus;Tillus elongatus;Finland;GenBank;-;MZ611361
Coleoptera;Elateridae;Ampedus;Ampedus pomonae;Finland;GenBank;-;MZ629286
Coleoptera;Cryptophagidae;Cryptophagus;Cryptophagus populi;Finland;GenBank;-;MZ629287
Coleoptera;Tenebrionidae;Uloma;Uloma rufa;Finland;GenBank;-;MZ629346
Coleoptera;Elateridae;Ampedus;Ampedus pomonae;Finland;GenBank;-;MZ629371
Coleoptera;Lophocateridae;Grynocharis;Grynocharis oblonga;Finland;GenBank;-;MZ629405
Coleoptera;Staphylinidae;Trimium;Trimium brevicorne;Finland;GenBank;-;MZ629533
Coleoptera;Monotomidae;Rhizophagus;Rhizophagus depressus;Finland;GenBank;-;MZ629578
Coleoptera;Leiodidae;Anisotoma;Anisotoma orbicularis;Finland;GenBank;-;MZ629591
Coleoptera;Cryptophagidae;Atomaria;Atomaria umbrina;Finland;GenBank;-;MZ629629
Coleoptera;Ptiliidae;Ptenidium;Ptenidium nitidum;Finland;GenBank;-;MZ629753
Coleoptera;Staphylinidae;Trimium;Trimium brevicorne;Finland;GenBank;-;MZ629763
Coleoptera;Tetratomidae;Tetratoma;Tetratoma fungorum;Finland;GenBank;-;MZ630063

Coleoptera;Melandryidae;Dolotarsus;Dolotarsus lividus;Finland;GenBank;-;MZ630143
Coleoptera;Mycetophagidae;Mycetophagus;Mycetophagus quadripustulatus;Finland;GenBank;-;MZ630177
Coleoptera;Scarabaeidae;Protaetia;Protaetia cuprea;Finland;GenBank;-;MZ630412
Coleoptera;Ptiliidae;Ptenidium;Ptenidium pusillum;Finland;GenBank;-;MZ630461
Coleoptera;Scarabaeidae;Trichius;Trichius fasciatus;Finland;GenBank;-;MZ630476
Coleoptera;Melandryidae;Abdera;Abdera flexuosa;Finland;GenBank;-;MZ630532
Coleoptera;Ptiliidae;Ptilium;Ptilium exaratum;Finland;GenBank;-;MZ630659
Coleoptera;Curculionidae;Phloeotribus;Phloeotribus spinulosus;Finland;GenBank;-;MZ630722
Coleoptera;Lycidae;Pyropterus;Pyropterus nigroruber;Finland;GenBank;-;MZ630748
Coleoptera;Ptinidae;Xestobium;Xestobium rufovillosum;Finland;GenBank;-;MZ630758
Coleoptera;Ptinidae;Xestobium;Xestobium rufovillosum;Finland;GenBank;-;MZ630791
Coleoptera;Tenebrionidae;Mycetochara;Mycetochara flavipes;Finland;GenBank;-;MZ630900
Coleoptera;Staphylinidae;Sepedophilus;Sepedophilus immaculatus;Finland;GenBank;-;MZ630917
Coleoptera;Ciidae;Ennearthron;Ennearthron cornutum;Finland;GenBank;-;MZ630920
Coleoptera;Curculionidae;Crypturgus;Crypturgus hispidulus;Finland;GenBank;-;MZ631086
Coleoptera;Ptinidae;Xestobium;Xestobium rufovillosum;Finland;GenBank;-;MZ631132
Coleoptera;Ciidae;Cis;Cis bidentatus;Finland;GenBank;-;MZ631135
Coleoptera;Cryptophagidae;Cryptophagus;Cryptophagus populi;Finland;GenBank;-;MZ631457
Coleoptera;Histeridae;Dendrophilus;Dendrophilus pygmaeus;Finland;GenBank;-;MZ631586
Coleoptera;Cryptophagidae;Atomaria;Atomaria umbrina;Finland;GenBank;-;MZ632001
Coleoptera;Staphylinidae;Bibloporus;Bibloporus bicolor;Finland;GenBank;-;MZ632046
Coleoptera;Staphylinidae;Phyllodrepa;Phyllodrepa nigra;Finland;GenBank;-;MZ632192
Coleoptera;Leiodidae;Anisotoma;Anisotoma castanea;Finland;GenBank;-;MZ632268
Coleoptera;Staphylinidae;Bibloporus;Bibloporus bicolor;Finland;GenBank;-;MZ632391
Coleoptera;Latridiidae;Corticaria;Corticaria crenulata;Finland;GenBank;-;MZ632468
Coleoptera;Cerambycidae;Tetrops;Tetrops praeustus;Finland;GenBank;-;MZ632603
Coleoptera;Lophocateridae;Grynocharis;Grynocharis oblonga;Finland;GenBank;-;MZ632671
Coleoptera;Ciidae;Cis;Cis bidentatus;Finland;GenBank;-;MZ632759
Coleoptera;Ptiliidae;Ptenidium;Ptenidium nitidum;Finland;GenBank;-;MZ632913
Coleoptera;Histeridae;Plegaderus;Plegaderus caesus;Finland;GenBank;-;MZ632996
Coleoptera;Staphylinidae;Euplectus;Euplectus karsteni;Finland;GenBank;-;MZ633049
Coleoptera;Histeridae;Dendrophilus;Dendrophilus punctatus;Finland;GenBank;-;MZ633277
Coleoptera;Curculionidae;Crypturgus;Crypturgus hispidulus;Finland;GenBank;-;MZ633576
Coleoptera;Ptinidae;Xyletinus;Xyletinus ater;Finland;GenBank;-;MZ633590
Coleoptera;Cerambycidae;Tetrops;Tetrops praeustus;Finland;GenBank;-;MZ633609
Coleoptera;Mycetophagidae;Mycetophagus;Mycetophagus piceus;Finland;GenBank;-;MZ633678
Coleoptera;Ptiliidae;Ptenidium;Ptenidium nitidum;Finland;GenBank;-;MZ633679
Coleoptera;Erotylidae;Tritoma;Tritoma bipustulata;Finland;GenBank;-;MZ633713
Coleoptera;Staphylinidae;Euplectus;Euplectus karsteni;Finland;GenBank;-;MZ633819
Coleoptera;Histeridae;Dendrophilus;Dendrophilus punctatus;Finland;GenBank;-;MZ633871
Coleoptera;Staphylinidae;Bibloporus;Bibloporus bicolor;Finland;GenBank;-;MZ633982
Coleoptera;Staphylinidae;Gabrius;Gabrius splendidulus;Finland;GenBank;-;MZ656159
Coleoptera;Leiodidae;Liodopria;Liodopria serricornis;Finland;GenBank;-;MZ656170
Coleoptera;Ptiliidae;Ptilium;Ptilium exaratum;Finland;GenBank;-;MZ656381
Coleoptera;Lycidae;Pyropterus;Pyropterus nigroruber;Finland;GenBank;-;MZ656484
Coleoptera;Curculionidae;Polygraphus;Polygraphus poligraphus;Finland;GenBank;-;MZ656570
Coleoptera;Monotomidae;Rhizophagus;Rhizophagus depressus;Finland;GenBank;-;MZ656672
Coleoptera;Staphylinidae;Bolitochara;Bolitochara mulsanti;Finland;GenBank;-;MZ656717
Coleoptera;Cerambycidae;Tetrops;Tetrops praeustus;Finland;GenBank;-;MZ656721
Coleoptera;Staphylinidae;Atrecus;Atrecus affinis;Finland;GenBank;-;MZ657130
Coleoptera;Nitidulidae;Glischrochilus;Glischrochilus hortensis;Finland;GenBank;-;MZ657232
Coleoptera;Cerambycidae;Hylotrupes;Hylotrupes bajulus;Finland;GenBank;-;MZ657463
Coleoptera;Latridiidae;Cortinicara;Cortinicara gibbosa;Finland;GenBank;-;MZ657508
Coleoptera;Curculionidae;Orthotomicus;Orthotomicus laricis;Finland;GenBank;-;MZ657602
Coleoptera;Ciidae;Cis;Cis glabratus;Finland;GenBank;-;MZ657607
Coleoptera;Staphylinidae;Leptusa;Leptusa ruficollis;Finland;GenBank;-;MZ657672
Coleoptera;Alexiidae;Sphaerosoma;Sphaerosoma pilosum;Finland;GenBank;-;MZ657774
Coleoptera;Staphylinidae;Sepedophilus;Sepedophilus testaceus;Finland;GenBank;-;MZ657784
Coleoptera;Ciidae;Cis;Cis boleti;Finland;GenBank;-;MZ657860
Coleoptera;Staphylinidae;Euryusa;Euryusa castanoptera;Finland;GenBank;-;MZ657895
Coleoptera;Staphylinidae;Sepedophilus;Sepedophilus testaceus;Finland;GenBank;-;MZ658168
Coleoptera;Cerylonidae;Cerylon;Cerylon fagi;Finland;GenBank;-;MZ658829
Coleoptera;Cerambycidae;Tetrops;Tetrops praeustus;Finland;GenBank;-;MZ658860
Coleoptera;Leiodidae;Liodopria;Liodopria serricornis;Finland;GenBank;-;MZ658933
Coleoptera;Ciidae;Cis;Cis nitidus;Finland;GenBank;-;MZ658990
Coleoptera;Curculionidae;Orthotomicus;Orthotomicus laricis;Finland;GenBank;-;MZ659024
Coleoptera;Leiodidae;Agathidium;Agathidium seminulum;Finland;GenBank;-;MZ659077
Coleoptera;Cerylonidae;Cerylon;Cerylon fagi;Finland;GenBank;-;MZ659320
Coleoptera;Staphylinidae;Euplectus;Euplectus karsteni;Finland;GenBank;-;MZ659575
Coleoptera;Ptinidae;Ptinomorphus;Ptinomorphus imperialis;Finland;GenBank;-;MZ659585
Coleoptera;Staphylinidae;Sepedophilus;Sepedophilus bipunctatus;Finland;GenBank;-;MZ659744
Coleoptera;Latridiidae;Cortinicara;Cortinicara gibbosa;Finland;GenBank;-;MZ659942
Coleoptera;Melandryidae;Orchesia;Orchesia undulata;Finland;GenBank;-;MZ659960
Coleoptera;Nitidulidae;Epuraea;Epuraea melanocephala;Finland;GenBank;-;MZ659976
Coleoptera;Lycidae;Dictyoptera;Dictyoptera aurora;Finland;GenBank;-;MZ660166
Coleoptera;Staphylinidae;Phyllodrepa;Phyllodrepa nigra;Finland;GenBank;-;MZ660225
Coleoptera;Ptiliidae;Ptenidium;Ptenidium punctatum;Finland;GenBank;-;MZ660231
Coleoptera;Staphylinidae;Dinaraea;Dinaraea aequata;Finland;GenBank;-;MZ660236

Coleoptera;Tenebrionidae;Uloma;Uloma rufa;Finland;GenBank;-;MZ660274
Coleoptera;Leiodidae;Agathidium;Agathidium badium;Finland;GenBank;-;MZ660395
Coleoptera;Ptinidae;Ernobius;Ernobius abietis;Finland;GenBank;-;MZ660439
Coleoptera;Monotomidae;Monotoma;Monotoma picipes;Finland;GenBank;-;MZ660504
Coleoptera;Ciidae;Ennearthron;Ennearthron cornutum;Finland;GenBank;-;MZ660544
Coleoptera;Latridiidae;Cortinicara;Cortinicara gibbosa;Finland;GenBank;-;MZ660564
Coleoptera;Staphylinidae;Bolitochara;Bolitochara mulsanti;Finland;GenBank;-;MZ660779
Coleoptera;Staphylinidae;Euryusa;Euryusa castanoptera;Finland;GenBank;-;MZ660885
Coleoptera;Aderidae;Aderus;Aderus populneus;Bulgaria;Bold;GMBUE3135-14;-
Coleoptera;Leiodidae;Agathidium;Agathidium seminulum;Finland;Bold;GMFID067-12;-
Coleoptera;Buprestidae;Agrilus;Agrilus viridis;Austria;Bold;TDAAT982-20;-
Coleoptera;Cerambycidae;Alosterna;Alosterna tabacicolor;Finland;Bold;GMFIF710-12;-
Coleoptera;Elateridae;Ampedus;Ampedus quercicola;Austria;Bold;TDAOE358-21;-
Coleoptera;Elateridae;Ampedus;Ampedus sanguinolentus;Norway;Bold;NOCOL029-12;-
Coleoptera;Elateridae;Ampedus;Ampedus sanguinolentus;Norway;Bold;NOCOL028-12;-
Coleoptera;Nitidulidae;Amphotis;Amphotis marginata;Belarus;Bold;GMBML392-17;-
Coleoptera;Scraptiidae;Anaspis;Anaspis frontalis;Belarus;Bold;GMBMK359-17;-
Coleoptera;Curculionidae;Anisandrus;Anisandrus dispar;Belarus;Bold;GMBMJ1769-17;-
Coleoptera;Oedemeridae;Anogcodes;Anogcodes rufiventris;Austria;Bold;TDAAT1612-20;-
Coleoptera;Anthribidae;Anthribus;Anthribus nebulosus;Belarus;Bold;GMBMJ1807-17;-
Coleoptera;Throscidae;Aulonothroscus;Aulonothroscus brevicollis;Belarus;Bold;GMBMJ1548-17;-
Coleoptera;Throscidae;Aulonothroscus;Aulonothroscus brevicollis;Belarus;Bold;GMBMJ1868-17;-
Coleoptera;Throscidae;Aulonothroscus;Aulonothroscus brevicollis;Belarus;Bold;GMBMJ1869-17;-
Coleoptera;Throscidae;Aulonothroscus;Aulonothroscus brevicollis;Belarus;Bold;GMBMM1075-17;-
Coleoptera;Throscidae;Aulonothroscus;Aulonothroscus brevicollis;Belarus;Bold;GMBMJ1855-17;-
Coleoptera;Throscidae;Aulonothroscus;Aulonothroscus brevicollis;Belarus;Bold;GMBMJ1871-17;-
Coleoptera;Throscidae;Aulonothroscus;Aulonothroscus brevicollis;Belarus;Bold;GMBMJ1545-17;-
Coleoptera;Throscidae;Aulonothroscus;Aulonothroscus brevicollis;Belarus;Bold;GMBMJ1859-17;-
Coleoptera;Throscidae;Aulonothroscus;Aulonothroscus brevicollis;Belarus;Bold;GMBMJ1866-17;-
Coleoptera;Throscidae;Aulonothroscus;Aulonothroscus brevicollis;Belarus;Bold;GMBMJ1541-17;-
Coleoptera;Throscidae;Aulonothroscus;Aulonothroscus brevicollis;Belarus;Bold;GMBMK407-17;-
Coleoptera;Oedemeridae;Chrysanthia;Chrysanthia viridissima;Bulgaria;Bold;GMBUE972-14;-
Coleoptera;Buprestidae;Chrysobothris;Chrysobothris affinis;Austria;Bold;TDAOE351-21;-
Coleoptera;Cleridae;Clerus;Clerus mutillarius;Austria;Bold;TDAOE357-21;-
Coleoptera;Zopheridae;Colobicus;Colobicus hirtus;Austria;Bold;TDAOE363-21;-
Coleoptera;Latridiidae;Corticaria;Corticaria abietorum;Finland;Bold;GMFIK142-12;-
Coleoptera;Latridiidae;Corticarina;Corticarina similata;Bulgaria;Bold;GMBUA1482-14;-
Coleoptera;Latridiidae;Corticarina;Corticarina similata;Bulgaria;Bold;GMBUE3052-14;-
Coleoptera;Latridiidae;Corticarina;Corticarina similata;Bulgaria;Bold;GMBUA1483-14;-
Coleoptera;Latridiidae;Corticarina;Corticarina similata;Bulgaria;Bold;GMBUA1489-14;-
Coleoptera;Latridiidae;Corticarina;Corticarina similata;Belarus;Bold;GMBMH122-17;-
Coleoptera;Latridiidae;Corticarina;Corticarina similata;Belarus;Bold;GMBMH251-17;-
Coleoptera;Latridiidae;Corticarina;Corticarina similata;Belarus;Bold;GMBMH234-17;-
Coleoptera;Latridiidae;Corticarina;Corticarina truncatella;Bulgaria;Bold;GMBUE3069-14;-
Coleoptera;Latridiidae;Corticarina;Corticarina truncatella;Bulgaria;Bold;GMBUE3041-14;-
Coleoptera;Latridiidae;Cortinicara;Cortinicara gibbosa;Belarus;Bold;GMBMK400-17;-
Coleoptera;Latridiidae;Cortinicara;Cortinicara gibbosa;Belarus;Bold;GMBMS885-18;-
Coleoptera;Latridiidae;Cortinicara;Cortinicara gibbosa;Belarus;Bold;GMBMJ1757-17;-
Coleoptera;Latridiidae;Cortinicara;Cortinicara gibbosa;Belarus;Bold;GMBMJ1814-17;-
Coleoptera;Latridiidae;Cortinicara;Cortinicara gibbosa;Belarus;Bold;GMBMH121-17;-
Coleoptera;Latridiidae;Cortinicara;Cortinicara gibbosa;Belarus;Bold;GMBMR1374-17;-
Coleoptera;Latridiidae;Cortinicara;Cortinicara gibbosa;Belarus;Bold;GMBMJ1770-17;-
Coleoptera;Latridiidae;Cortinicara;Cortinicara gibbosa;Belarus;Bold;GMBMP2042-18;-
Coleoptera;Latridiidae;Cortinicara;Cortinicara gibbosa;Belarus;Bold;GMBMI233-17;-
Coleoptera;Latridiidae;Cortinicara;Cortinicara gibbosa;Belarus;Bold;GMBMJ1812-17;-
Coleoptera;Latridiidae;Cortinicara;Cortinicara gibbosa;Belarus;Bold;GMBMK380-17;-
Coleoptera;Latridiidae;Cortinicara;Cortinicara gibbosa;Belarus;Bold;GMBMQ1015-17;-
Coleoptera;Latridiidae;Cortinicara;Cortinicara gibbosa;Belarus;Bold;GMBMR1354-17;-
Coleoptera;Latridiidae;Cortinicara;Cortinicara gibbosa;Belarus;Bold;GMBMM1065-17;-
Coleoptera;Latridiidae;Cortinicara;Cortinicara gibbosa;Belarus;Bold;GMBMT153-18;-
Coleoptera;Latridiidae;Cortinicara;Cortinicara gibbosa;Belarus;Bold;GMBMT123-18;-
Coleoptera;Latridiidae;Cortinicara;Cortinicara gibbosa;Belarus;Bold;GMBMJ1746-17;-
Coleoptera;Latridiidae;Cortinicara;Cortinicara gibbosa;Belarus;Bold;GMBMM1072-17;-
Coleoptera;Latridiidae;Cortinicara;Cortinicara gibbosa;Belarus;Bold;GMBML408-17;-
Coleoptera;Latridiidae;Cortinicara;Cortinicara gibbosa;Belarus;Bold;GMBML410-17;-
Coleoptera;Latridiidae;Cortinicara;Cortinicara gibbosa;Belarus;Bold;GMBMQ1009-17;-
Coleoptera;Latridiidae;Cortinicara;Cortinicara gibbosa;Belarus;Bold;GMBMT161-18;-
Coleoptera;Latridiidae;Cortinicara;Cortinicara gibbosa;Belarus;Bold;GMBMT129-18;-
Coleoptera;Latridiidae;Cortinicara;Cortinicara gibbosa;Belarus;Bold;GMBMS886-18;-
Coleoptera;Latridiidae;Cortinicara;Cortinicara gibbosa;Belarus;Bold;GMBMH127-17;-
Coleoptera;Latridiidae;Cortinicara;Cortinicara gibbosa;Belarus;Bold;GMBMM1055-17;-
Coleoptera;Latridiidae;Cortinicara;Cortinicara gibbosa;Belarus;Bold;GMBMJ1744-17;-
Coleoptera;Latridiidae;Cortinicara;Cortinicara gibbosa;Belarus;Bold;GMBMH129-17;-
Coleoptera;Latridiidae;Cortinicara;Cortinicara gibbosa;Belarus;Bold;GMBMQ1023-17;-
Coleoptera;Latridiidae;Cortinicara;Cortinicara gibbosa;Belarus;Bold;GMBMM1080-17;-
Coleoptera;Latridiidae;Cortinicara;Cortinicara gibbosa;Belarus;Bold;GMBMM1054-17;-
Coleoptera;Latridiidae;Cortinicara;Cortinicara gibbosa;Belarus;Bold;GMBML407-17;-
Coleoptera;Latridiidae;Cortinicara;Cortinicara gibbosa;Belarus;Bold;GMBMQ1037-17;-

Coleoptera;Latridiidae;Cortinicara;Cortinicara gibbosa;Belarus;Bold;GMBMR1356-17;-
Coleoptera;Latridiidae;Cortinicara;Cortinicara gibbosa;Belarus;Bold;GMBMN986-17;-
Coleoptera;Latridiidae;Cortinicara;Cortinicara gibbosa;Belarus;Bold;GMBMK397-17;-
Coleoptera;Latridiidae;Cortinicara;Cortinicara gibbosa;Belarus;Bold;GMBMT136-18;-
Coleoptera;Latridiidae;Cortinicara;Cortinicara gibbosa;Belarus;Bold;GMBMJ1821-17;-
Coleoptera;Latridiidae;Cortinicara;Cortinicara gibbosa;Belarus;Bold;GMBMR1361-17;-
Coleoptera;Latridiidae;Cortinicara;Cortinicara gibbosa;Belarus;Bold;GMBMI223-17;-
Coleoptera;Latridiidae;Cortinicara;Cortinicara gibbosa;Belarus;Bold;GMBML398-17;-
Coleoptera;Latridiidae;Cortinicara;Cortinicara gibbosa;Belarus;Bold;GMBMT159-18;-
Coleoptera;Latridiidae;Cortinicara;Cortinicara gibbosa;Belarus;Bold;GMBMH119-17;-
Coleoptera;Latridiidae;Cortinicara;Cortinicara gibbosa;Belarus;Bold;GMBMI216-17;-
Coleoptera;Latridiidae;Cortinicara;Cortinicara gibbosa;Belarus;Bold;GMBMH118-17;-
Coleoptera;Latridiidae;Cortinicara;Cortinicara gibbosa;Belarus;Bold;GMBMM1066-17;-
Coleoptera;Latridiidae;Cortinicara;Cortinicara gibbosa;Belarus;Bold;GMBMR1365-17;-
Coleoptera;Latridiidae;Cortinicara;Cortinicara gibbosa;Belarus;Bold;GMBMI206-17;-
Coleoptera;Latridiidae;Cortinicara;Cortinicara gibbosa;Belarus;Bold;GMBMR1355-17;-
Coleoptera;Latridiidae;Cortinicara;Cortinicara gibbosa;Belarus;Bold;GMBMK399-17;-
Coleoptera;Latridiidae;Cortinicara;Cortinicara gibbosa;Belarus;Bold;GMBMT149-18;-
Coleoptera;Latridiidae;Cortinicara;Cortinicara gibbosa;Belarus;Bold;GMBMK398-17;-
Coleoptera;Latridiidae;Cortinicara;Cortinicara gibbosa;Belarus;Bold;GMBMK347-17;-
Coleoptera;Latridiidae;Cortinicara;Cortinicara gibbosa;Belarus;Bold;GMBMT154-18;-
Coleoptera;Latridiidae;Cortinicara;Cortinicara gibbosa;Belarus;Bold;GMBMH131-17;-
Coleoptera;Latridiidae;Cortinicara;Cortinicara gibbosa;Belarus;Bold;GMBMJ1768-17;-
Coleoptera;Latridiidae;Cortinicara;Cortinicara gibbosa;Belarus;Bold;GMBMJ1801-17;-
Coleoptera;Latridiidae;Cortinicara;Cortinicara gibbosa;Belarus;Bold;GMBMT162-18;-
Coleoptera;Latridiidae;Cortinicara;Cortinicara gibbosa;Belarus;Bold;GMBMS869-18;-
Coleoptera;Latridiidae;Cortinicara;Cortinicara gibbosa;Belarus;Bold;GMBMP2846-18;-
Coleoptera;Latridiidae;Cortinicara;Cortinicara gibbosa;Belarus;Bold;GMBMQ1054-17;-
Coleoptera;Latridiidae;Cortinicara;Cortinicara gibbosa;Belarus;Bold;GMBMT139-18;-
Coleoptera;Latridiidae;Cortinicara;Cortinicara gibbosa;Belarus;Bold;GMBMJ1817-17;-
Coleoptera;Latridiidae;Cortinicara;Cortinicara gibbosa;Belarus;Bold;GMBMT137-18;-
Coleoptera;Latridiidae;Cortinicara;Cortinicara gibbosa;Belarus;Bold;GMBMQ1018-17;-
Coleoptera;Latridiidae;Cortinicara;Cortinicara gibbosa;Belarus;Bold;GMBMI200-17;-
Coleoptera;Latridiidae;Cortinicara;Cortinicara gibbosa;Belarus;Bold;GMBMT147-18;-
Coleoptera;Latridiidae;Cortinicara;Cortinicara gibbosa;Belarus;Bold;GMBML390-17;-
Coleoptera;Latridiidae;Cortinicara;Cortinicara gibbosa;Belarus;Bold;GMBMS880-18;-
Coleoptera;Latridiidae;Cortinicara;Cortinicara gibbosa;Belarus;Bold;GMBMQ1041-17;-
Coleoptera;Latridiidae;Cortinicara;Cortinicara gibbosa;Belarus;Bold;GMBMQ1010-17;-
Coleoptera;Latridiidae;Cortinicara;Cortinicara gibbosa;Belarus;Bold;GMBMR1405-17;-
Coleoptera;Latridiidae;Cortinicara;Cortinicara gibbosa;Belarus;Bold;GMBMJ1797-17;-
Coleoptera;Latridiidae;Cortinicara;Cortinicara gibbosa;Belarus;Bold;GMBMQ993-17;-
Coleoptera;Latridiidae;Cortinicara;Cortinicara gibbosa;Belarus;Bold;GMBMK371-17;-
Coleoptera;Latridiidae;Cortinicara;Cortinicara gibbosa;Belarus;Bold;GMBMP2038-18;-
Coleoptera;Latridiidae;Cortinicara;Cortinicara gibbosa;Belarus;Bold;GMBMJ1777-17;-
Coleoptera;Latridiidae;Cortinicara;Cortinicara gibbosa;Belarus;Bold;GMBMP2029-18;-
Coleoptera;Latridiidae;Cortinicara;Cortinicara gibbosa;Belarus;Bold;GMBMP2033-18;-
Coleoptera;Latridiidae;Cortinicara;Cortinicara gibbosa;Belarus;Bold;GMBMQ995-17;-
Coleoptera;Latridiidae;Cortinicara;Cortinicara gibbosa;Belarus;Bold;GMBMO808-17;-
Coleoptera;Latridiidae;Cortinicara;Cortinicara gibbosa;Belarus;Bold;GMBMJ1538-17;-
Coleoptera;Latridiidae;Cortinicara;Cortinicara gibbosa;Belarus;Bold;GMBMQ1047-17;-
Coleoptera;Latridiidae;Cortinicara;Cortinicara gibbosa;Belarus;Bold;GMBMM1062-17;-
Coleoptera;Latridiidae;Cortinicara;Cortinicara gibbosa;Belarus;Bold;GMBMH236-17;-
Coleoptera;Latridiidae;Cortinicara;Cortinicara gibbosa;Belarus;Bold;GMBMT160-18;-
Coleoptera;Latridiidae;Cortinicara;Cortinicara gibbosa;Belarus;Bold;GMBMQ1002-17;-
Coleoptera;Latridiidae;Cortinicara;Cortinicara gibbosa;France;Bold;GMFPC184-18;-
Coleoptera;Nitidulidae;Cryptarcha;Cryptarcha undata;Germany;Bold;ULMCA016-19;-
Coleoptera;Laemophloeidae;Cryptolestes;Cryptolestes duplicatus;Austria;Bold;TDAOE347-21;-
Coleoptera;Laemophloeidae;Cryptolestes;Cryptolestes duplicatus;Austria;Bold;TDAOE385-21;-
Coleoptera;Laemophloeidae;Cryptolestes;Cryptolestes ferrugineus;Germany;Bold;CSP049-09;-
Coleoptera;Cryptophagidae;Cryptophagus;Cryptophagus dorsalis;Belarus;Bold;GMBMJ1811-17;-
Coleoptera;Cryptophagidae;Cryptophagus;Cryptophagus scanicus;Bulgaria;Bold;GMBUA1455-14;-
Coleoptera;Curculionidae;Crypturgus;Crypturgus cinereus;Hungary;Bold;SCOL011-12;-
Coleoptera;Curculionidae;Crypturgus;Crypturgus cinereus;Hungary;Bold;SCOL003-12;-
Coleoptera;Curculionidae;Crypturgus;Crypturgus hispidulus;Norway;Bold;SCOL064-12;-
Coleoptera;Melyridae;Dasytes;Dasytes aeratus;Bulgaria;Bold;GMBUA1460-14;-
Coleoptera;Melyridae;Dasytes;Dasytes aeratus;Austria;Bold;TDAOE307-21;-
Coleoptera;Melyridae;Dasytes;Dasytes plumbeus;Belarus;Bold;GMBMN1002-17;-
Coleoptera;Melyridae;Dasytes;Dasytes plumbeus;Belarus;Bold;GMBML402-17;-
Coleoptera;Melyridae;Dasytes;Dasytes plumbeus;Belarus;Bold;GMBMN1011-17;-
Coleoptera;Melyridae;Dasytes;Dasytes plumbeus;Belarus;Bold;GMBMQ1325-17;-
Coleoptera;Melyridae;Dasytes;Dasytes plumbeus;Belarus;Bold;GMBMN1019-17;-
Coleoptera;Melyridae;Dasytes;Dasytes plumbeus;Belarus;Bold;GMBML396-17;-
Coleoptera;Melyridae;Dasytes;Dasytes plumbeus;Austria;Bold;TDAAT1846-20;-
Coleoptera;Melyridae;Dasytes;Dasytes virens;Austria;Bold;TDAOE305-21;-
Coleoptera;Elateridae;Denticollis;Denticollis linearis;Norway;Bold;NOCOL018-12;-
Coleoptera;Lycidae;Dictyoptera;Dictyoptera aurora;Norway;Bold;NOCOL066-12;-
Coleoptera;Staphylinidae;Dinaraea;Dinaraea aequata;Bulgaria;Bold;GMBUA1465-14;-
Coleoptera;Cerambycidae;Dinoptera;Dinoptera collaris;Poland;Bold;COLAT207-08;-

Coleoptera;Cerambycidae;Dinoptera;Dinoptera collaris;Poland;Bold;COLAT208-08;-
Coleoptera;Anthribidae;Dissoleucas;Dissoleucas niveirostris;Belarus;Bold;GMBMI227-17;-
Coleoptera;Lucanidae;Dorcus;Dorcus parallelipipedus;Austria;Bold;TDAOE324-21;-
Coleoptera;Staphylinidae;Dropephylla;Dropephylla gracilicornis;Norway;Bold;NOCLP1802-19;-
Coleoptera;Staphylinidae;Dropephylla;Dropephylla ioptera;Norway;Bold;NOCLP1807-19;-
Coleoptera;Staphylinidae;Dropephylla;Dropephylla ioptera;Bulgaria;Bold;GMBUA1425-14;-
Coleoptera;Staphylinidae;Dropephylla;Dropephylla ioptera;Norway;Bold;NOCLP1806-19;-
Coleoptera;Curculionidae;Dryocoetes;Dryocoetes autographus;Sweden;Bold;SCOL142-12;-
Coleoptera;Curculionidae;Dryocoetes;Dryocoetes autographus;Norway;Bold;SCOL014-12;-
Coleoptera;Elateridae;Elater;Elater ferrugineus;Croatia;Bold;ICRYO045-22;-
Coleoptera;Elateridae;Elater;Elater ferrugineus;Croatia;Bold;ICRYO044-22;-
Coleoptera;Latridiidae;Enicmus;Enicmus transversus;Belarus;Bold;GMBMJ1815-17;-
Coleoptera;Nitidulidae;Epuraea;Epuraea biguttata;Germany;Bold;ULMCA004-19;-
Coleoptera;Nitidulidae;Epuraea;Epuraea biguttata;Germany;Bold;ULMCA006-19;-
Coleoptera;Nitidulidae;Epuraea;Epuraea biguttata;Germany;Bold;ULMCA007-19;-
Coleoptera;Nitidulidae;Epuraea;Epuraea biguttata;Germany;Bold;ULMCA014-19;-
Coleoptera;Nitidulidae;Epuraea;Epuraea melanocephala;Belarus;Bold;GMBMM1051-17;-
Coleoptera;Nitidulidae;Epuraea;Epuraea unicolor;Germany;Bold;ULMCA009-19;-
Coleoptera;Curculionidae;Ernoporus;Ernoporus tiliae;Norway;Bold;SCOL012-12;-
Coleoptera;Curculionidae;Ernoporus;Ernoporus tiliae;Norway;Bold;SCOL043-12;-
Coleoptera;Staphylinidae;Gabrius;Gabrius splendidulus;Austria;Bold;TDAOE336-21;-
Coleoptera;Nitidulidae;Glischrochilus;Glischrochilus quadriguttatus;Germany;Bold;ULMCA001-19;-
Coleoptera;Nitidulidae;Glischrochilus;Glischrochilus quadriguttatus;Germany;Bold;ULMCA012-19;-
Coleoptera;Scarabaeidae;Gnorimus;Gnorimus variabilis;Croatia;Bold;ICRYO043-22;-
Coleoptera;Scarabaeidae;Gnorimus;Gnorimus variabilis;Croatia;Bold;ICRYO046-22;-
Coleoptera;Scarabaeidae;Gnorimus;Gnorimus variabilis;Croatia;Bold;ICRYO047-22;-
Coleoptera;Cerambycidae;Gracilia;Gracilia minuta;Austria;Bold;SICOE925-19;-
Coleoptera;Cerambycidae;Grammoptera;Grammoptera ruficornis;Bulgaria;Bold;GMBUA343-14;-
Coleoptera;Histeridae;Hololepta;Hololepta plana;Croatia;Bold;ICRYO025-22;-
Coleoptera;Curculionidae;Hylastes;Hylastes angustatus;Norway;Bold;NOCLP1735-19;-
Coleoptera;Curculionidae;Hylastes;Hylastes opacus;Norway;Bold;NOCLP1733-19;-
Coleoptera;Curculionidae;Hylesinus;Hylesinus toranio;Norway;Bold;NOCLP1715-19;-
Coleoptera;Curculionidae;Hylesinus;Hylesinus toranio;Norway;Bold;NOCLP1714-19;-
Coleoptera;Curculionidae;Hylesinus;Hylesinus varius;Norway;Bold;NOCLP1721-19;-
Coleoptera;Cerambycidae;Hylotrupes;Hylotrupes bajulus;Austria;Bold;TDAAT1928-20;-
Coleoptera;Curculionidae;Hylurgops;Hylurgops palliatus;Austria;Bold;TDAOE342-21;-
Coleoptera;Curculionidae;Hylurgops;Hylurgops palliatus;Norway;Bold;SCOL015-12;-
Coleoptera;Curculionidae;Hylurgops;Hylurgops palliatus;Hungary;Bold;SCOL006-12;-
Coleoptera;Curculionidae;Ips;Ips acuminatus;Norway;Bold;SCOL023-12;-
Coleoptera;Curculionidae;Ips;Ips acuminatus;Norway;Bold;SCOL026-12;-
Coleoptera;Curculionidae;Ips;Ips acuminatus;Norway;Bold;NOCLP1772-19;-
Coleoptera;Curculionidae;Ips;Ips acuminatus;Norway;Bold;NOCLP1773-19;-
Coleoptera;Curculionidae;Ips;Ips sexdentatus;Norway;Bold;NOCLP1770-19;-
Coleoptera;Curculionidae;Ips;Ips sexdentatus;Spain;Bold;SCOL065-12;-
Coleoptera;Curculionidae;Ips;Ips sexdentatus;Hungary;Bold;SCOL005-12;-
Coleoptera;Curculionidae;Kissophagus;Kissophagus hederae;Austria;Bold;SCOL001-12;-
Coleoptera;Staphylinidae;Leptusa;Leptusa pulchella;Norway;Bold;ZMBN134-16;-
Coleoptera;Staphylinidae;Leptusa;Leptusa pulchella;Finland;Bold;GMFID066-12;-
Coleoptera;Eucnemidae;Microrhagus;Microrhagus pygmaeus;Norway;Bold;NOCLP1712-19;-
Coleoptera;Eucnemidae;Microrhagus;Microrhagus pygmaeus;Norway;Bold;NOCLP1707-19;-
Coleoptera;Mordellidae;Mordellochroa;Mordellochroa abdominalis;Belarus;Bold;GMBMK019-17;-
Coleoptera;Tenebrionidae;Neomida;Neomida haemorrhoidalis;Austria;Bold;TDAOE472-21;-
Coleoptera;Melandryidae;Orchesia;Orchesia minor;Finland;Bold;GMFID065-12;-
Coleoptera;Melandryidae;Orchesia;Orchesia undulata;Norway;Bold;COLHH1572-18;-
Coleoptera;Melandryidae;Orchesia;Orchesia undulata;Belarus;Bold;GMBMJ1745-17;-
Coleoptera;Curculionidae;Orthotomicus;Orthotomicus erosus;Spain;Bold;SCOL066-12;-
Coleoptera;Cucujidae;Pediacus;Pediacus depressus;Germany;Bold;ULMCA002-19;-
Coleoptera;Cucujidae;Pediacus;Pediacus depressus;Germany;Bold;ULMCA010-19;-
Coleoptera;Curculionidae;Phloeophagus;Phloeophagus lignarius;Norway;Bold;NOCLP1404-19;-
Coleoptera;Staphylinidae;Phloeopora;Phloeopora teres;Austria;Bold;TDAOE345-21;-
Coleoptera;Staphylinidae;Phloeopora;Phloeopora teres;Austria;Bold;TDAOE334-21;-
Coleoptera;Staphylinidae;Phloeostiba;Phloeostiba plana;Germany;Bold;ULMCA008-19;-
Coleoptera;Curculionidae;Phloeotribus;Phloeotribus spinulosus;Norway;Bold;NOCLP1741-19;-
Coleoptera;Curculionidae;Pityogenes;Pityogenes bidentatus;Sweden;Bold;SCOL130-12;-
Coleoptera;Curculionidae;Pityogenes;Pityogenes bidentatus;Norway;Bold;NOCLP1763-19;-
Coleoptera;Curculionidae;Pityogenes;Pityogenes bistridentatus;Hungary;Bold;SCOL328-12;-
Coleoptera;Curculionidae;Pityophthorus;Pityophthorus pityographus;Hungary;Bold;SCOL010-12;-
Coleoptera;Curculionidae;Pityophthorus;Pityophthorus pityographus;Hungary;Bold;SCOL009-12;-
Coleoptera;Anthribidae;Platyrhinus;Platyrhinus resinosus;Austria;Bold;TDAOE287-21;-
Coleoptera;Cerambycidae;Pogonocherus;Pogonocherus hispidus;Norway;Bold;COLHH1530-18;-
Coleoptera;Curculionidae;Polygraphus;Polygraphus poligraphus;Norway;Bold;SCOL045-12;-
Coleoptera;Cerambycidae;Prinobius;Prinobius myardi;France;Bold;CERAF123-08;-
Coleoptera;Scirtidae;Prionocyphon;Prionocyphon serricornis;Belarus;Bold;GMBMN1027-17;-
Coleoptera;Curculionidae;Pselactus;Pselactus spadix;Norway;Bold;NOCLP1408-19;-
Coleoptera;Cerambycidae;Pseudovadonia;Pseudovadonia livida;Austria;Bold;TDAOE361-21;-
Coleoptera;Anthribidae;Rhaphitropis;Rhaphitropis marchicus;Bulgaria;Bold;GMBUA1443-14;-
Coleoptera;Salpingidae;Salpingus;Salpingus planirostris;Norway;Bold;ZMBN969-17;-
Coleoptera;Salpingidae;Salpingus;Salpingus ruficollis;Belarus;Bold;GMBMA558-17;-

Coleoptera;Staphylinidae;Scaphisoma;Scaphisoma agaricinum;Austria;Bold;TDAOE382-21;-
Coleoptera;Curculionidae;Scolytus;Scolytus intricatus;Norway;Bold;SCOL044-12;-
Coleoptera;Curculionidae;Scolytus;Scolytus intricatus;Norway;Bold;SCOL016-12;-
Coleoptera;Staphylinidae;Sepedophilus;Sepedophilus testaceus;France;Bold;POLYT173-19;-
Coleoptera;Silvanidae;Silvanus;Silvanus unidentatus;Austria;Bold;TDAOE387-21;-
Coleoptera;Silvanidae;Silvanus;Silvanus unidentatus;Austria;Bold;TDAOE346-21;-
Coleoptera;Nitidulidae;Soronia;Soronia grisea;Germany;Bold;ULMCA003-19;-
Coleoptera;Nitidulidae;Soronia;Soronia grisea;Germany;Bold;ULMCA017-19;-
Coleoptera;Cerambycidae;Spondylis;Spondylis buprestoides;Austria;Bold;TDAAT1929-20;-
Coleoptera;Elateridae;Stenagostus;Stenagostus rhombeus;Croatia;Bold;ICRYO034-22;-
Coleoptera;Elateridae;Stenagostus;Stenagostus rhombeus;Croatia;Bold;ICRYO051-22;-
Coleoptera;Cerambycidae;Stenurella;Stenurella nigra;Bulgaria;Bold;GMBUB183-14;-
Coleoptera;Trogossitidae;Tenebroides;Tenebroides mauritanicus;Germany;Bold;CSP055-09;-
Coleoptera;Trogossitidae;Tenebroides;Tenebroides mauritanicus;Germany;Bold;CSP056-09;-
Coleoptera;Cleridae;Thanasimus;Thanasimus femoralis;Estonia;Bold;COLFH145-14;-
Coleoptera;Curculionidae;Tomicus;Tomicus piniperda;Sweden;Bold;SCOL141-12;-
Coleoptera;Mordellidae;Tomoxia;Tomoxia bucephala;Austria;Bold;TDAOE309-21;-
Coleoptera;Staphylinidae;Trimium;Trimium brevicorne;Belarus;Bold;GMBMJ1780-17;-
Coleoptera;Erotylidae;Tritoma;Tritoma bipustulata;Bulgaria;Bold;GMBUB1629-14;-
Coleoptera;Throscidae;Trixagus;Trixagus carinifrons;Norway;Bold;GMNWJ3053-14;-
Coleoptera;Throscidae;Trixagus;Trixagus carinifrons;Belarus;Bold;GMBMP1912-18;-
Coleoptera;Throscidae;Trixagus;Trixagus carinifrons;Norway;Bold;GMNWF1974-14;-
Coleoptera;Throscidae;Trixagus;Trixagus carinifrons;Norway;Bold;GMNWG3123-14;-
Coleoptera;Throscidae;Trixagus;Trixagus carinifrons;Norway;Bold;GMNWG3146-14;-
Coleoptera;Throscidae;Trixagus;Trixagus carinifrons;Belarus;Bold;GMBMP1929-18;-
Coleoptera;Throscidae;Trixagus;Trixagus carinifrons;Norway;Bold;GMNWF1980-14;-
Coleoptera;Throscidae;Trixagus;Trixagus carinifrons;Belarus;Bold;GMBMO787-17;-
Coleoptera;Throscidae;Trixagus;Trixagus carinifrons;Norway;Bold;GMNWJ3036-14;-
Coleoptera;Throscidae;Trixagus;Trixagus carinifrons;Norway;Bold;GMNWG3131-14;-
Coleoptera;Throscidae;Trixagus;Trixagus carinifrons;Belarus;Bold;GMBMP1939-18;-
Coleoptera;Throscidae;Trixagus;Trixagus carinifrons;Norway;Bold;GMNWI3253-14;-
Coleoptera;Throscidae;Trixagus;Trixagus carinifrons;Belarus;Bold;GMBMJ1852-17;-
Coleoptera;Throscidae;Trixagus;Trixagus carinifrons;Norway;Bold;GMNWI3224-14;-
Coleoptera;Throscidae;Trixagus;Trixagus carinifrons;Norway;Bold;GMNWI3263-14;-
Coleoptera;Throscidae;Trixagus;Trixagus carinifrons;Bulgaria;Bold;GMBUB1586-14;-
Coleoptera;Throscidae;Trixagus;Trixagus carinifrons;Belarus;Bold;GMBMJ1857-17;-
Coleoptera;Throscidae;Trixagus;Trixagus carinifrons;Norway;Bold;GMNWJ3061-14;-
Coleoptera;Throscidae;Trixagus;Trixagus carinifrons;Belarus;Bold;GMBMK365-17;-
Coleoptera;Throscidae;Trixagus;Trixagus carinifrons;Bulgaria;Bold;GMBUB1566-14;-
Coleoptera;Throscidae;Trixagus;Trixagus carinifrons;Norway;Bold;GMNWF2029-14;-
Coleoptera;Throscidae;Trixagus;Trixagus carinifrons;Bulgaria;Bold;GMBUA1457-14;-
Coleoptera;Throscidae;Trixagus;Trixagus carinifrons;Norway;Bold;GMNWK3483-14;-
Coleoptera;Throscidae;Trixagus;Trixagus carinifrons;Norway;Bold;GMNWG3166-14;-
Coleoptera;Throscidae;Trixagus;Trixagus carinifrons;Norway;Bold;GMNWL2542-14;-
Coleoptera;Throscidae;Trixagus;Trixagus carinifrons;Norway;Bold;GMNWI3267-14;-
Coleoptera;Throscidae;Trixagus;Trixagus carinifrons;Norway;Bold;GMNWI3287-14;-
Coleoptera;Throscidae;Trixagus;Trixagus carinifrons;Finland;Bold;COLFH534-15;-
Coleoptera;Throscidae;Trixagus;Trixagus carinifrons;Norway;Bold;GMNWJ3049-14;-
Coleoptera;Throscidae;Trixagus;Trixagus carinifrons;Norway;Bold;GMNWL2590-14;-
Coleoptera;Throscidae;Trixagus;Trixagus carinifrons;Norway;Bold;GMNWG3148-14;-
Coleoptera;Throscidae;Trixagus;Trixagus carinifrons;Norway;Bold;GMNWJ3048-14;-
Coleoptera;Throscidae;Trixagus;Trixagus carinifrons;Norway;Bold;GMNWI3254-14;-
Coleoptera;Throscidae;Trixagus;Trixagus carinifrons;Norway;Bold;GMNWG3127-14;-
Coleoptera;Throscidae;Trixagus;Trixagus carinifrons;Norway;Bold;GMNWI3261-14;-
Coleoptera;Throscidae;Trixagus;Trixagus carinifrons;Belarus;Bold;GMBMJ1850-17;-
Coleoptera;Throscidae;Trixagus;Trixagus carinifrons;Norway;Bold;GMNWG3165-14;-
Coleoptera;Throscidae;Trixagus;Trixagus carinifrons;Belarus;Bold;GMBMP1985-18;-
Coleoptera;Throscidae;Trixagus;Trixagus carinifrons;Norway;Bold;GMNWG3139-14;-
Coleoptera;Throscidae;Trixagus;Trixagus carinifrons;Belarus;Bold;GMBMJ1856-17;-
Coleoptera;Throscidae;Trixagus;Trixagus carinifrons;Belarus;Bold;GMBMP1946-18;-
Coleoptera;Throscidae;Trixagus;Trixagus carinifrons;Belarus;Bold;GMBMK402-17;-
Coleoptera;Throscidae;Trixagus;Trixagus carinifrons;Belarus;Bold;GMBMJ1854-17;-
Coleoptera;Throscidae;Trixagus;Trixagus carinifrons;Belarus;Bold;GMBMK378-17;-
Coleoptera;Throscidae;Trixagus;Trixagus carinifrons;Belarus;Bold;GMBMP1961-18;-
Coleoptera;Throscidae;Trixagus;Trixagus carinifrons;Belarus;Bold;GMBMK373-17;-
Coleoptera;Throscidae;Trixagus;Trixagus carinifrons;Belarus;Bold;GMBMO816-17;-
Coleoptera;Throscidae;Trixagus;Trixagus carinifrons;Belarus;Bold;GMBMM1061-17;-
Coleoptera;Throscidae;Trixagus;Trixagus carinifrons;Belarus;Bold;GMBMP1971-18;-
Coleoptera;Throscidae;Trixagus;Trixagus carinifrons;Belarus;Bold;GMBMP1999-18;-
Coleoptera;Throscidae;Trixagus;Trixagus carinifrons;Belarus;Bold;GMBMP1930-18;-
Coleoptera;Throscidae;Trixagus;Trixagus carinifrons;Belarus;Bold;GMBMQ1006-17;-
Coleoptera;Throscidae;Trixagus;Trixagus carinifrons;Belarus;Bold;GMBMM1068-17;-
Coleoptera;Throscidae;Trixagus;Trixagus carinifrons;Belarus;Bold;GMBMK391-17;-
Coleoptera;Throscidae;Trixagus;Trixagus carinifrons;Belarus;Bold;GMBMP1889-18;-
Coleoptera;Throscidae;Trixagus;Trixagus carinifrons;Belarus;Bold;GMBMK358-17;-
Coleoptera;Throscidae;Trixagus;Trixagus carinifrons;Belarus;Bold;GMBMK369-17;-
Coleoptera;Throscidae;Trixagus;Trixagus carinifrons;Belarus;Bold;GMBMP2006-18;-
Coleoptera;Throscidae;Trixagus;Trixagus carinifrons;Belarus;Bold;GMBMP2002-18;-

Coleoptera;Throscidae;Trixagus;Trixagus carinifrons;Belarus;Bold;GMBMK343-17;-
Coleoptera;Throscidae;Trixagus;Trixagus carinifrons;Belarus;Bold;GMBMK388-17;-
Coleoptera;Throscidae;Trixagus;Trixagus carinifrons;Belarus;Bold;GMBMK337-17;-
Coleoptera;Throscidae;Trixagus;Trixagus carinifrons;Belarus;Bold;GMBMP1920-18;-
Coleoptera;Throscidae;Trixagus;Trixagus carinifrons;Belarus;Bold;GMBMO782-17;-
Coleoptera;Throscidae;Trixagus;Trixagus carinifrons;Belarus;Bold;GMBMK382-17;-
Coleoptera;Throscidae;Trixagus;Trixagus carinifrons;Belarus;Bold;GMBMJ1860-17;-
Coleoptera;Throscidae;Trixagus;Trixagus carinifrons;Belarus;Bold;GMBMJ1826-17;-
Coleoptera;Throscidae;Trixagus;Trixagus carinifrons;Belarus;Bold;GMBMO814-17;-
Coleoptera;Throscidae;Trixagus;Trixagus carinifrons;Belarus;Bold;GMBMP2015-18;-
Coleoptera;Throscidae;Trixagus;Trixagus carinifrons;Belarus;Bold;GMBMK379-17;-
Coleoptera;Throscidae;Trixagus;Trixagus carinifrons;Belarus;Bold;GMBMR1357-17;-
Coleoptera;Throscidae;Trixagus;Trixagus carinifrons;Belarus;Bold;GMBMJ1827-17;-
Coleoptera;Throscidae;Trixagus;Trixagus carinifrons;Belarus;Bold;GMBMP1991-18;-
Coleoptera;Throscidae;Trixagus;Trixagus carinifrons;Belarus;Bold;GMBMP1888-18;-
Coleoptera;Throscidae;Trixagus;Trixagus carinifrons;Belarus;Bold;GMBMP2020-18;-
Coleoptera;Throscidae;Trixagus;Trixagus carinifrons;Belarus;Bold;GMBMP1933-18;-
Coleoptera;Throscidae;Trixagus;Trixagus carinifrons;Belarus;Bold;GMBMP1956-18;-
Coleoptera;Throscidae;Trixagus;Trixagus carinifrons;Belarus;Bold;GMBMO788-17;-
Coleoptera;Throscidae;Trixagus;Trixagus carinifrons;Belarus;Bold;GMBMP2027-18;-
Coleoptera;Throscidae;Trixagus;Trixagus carinifrons;Belarus;Bold;GMBMJ1853-17;-
Coleoptera;Throscidae;Trixagus;Trixagus carinifrons;Belarus;Bold;GMBMO773-17;-
Coleoptera;Throscidae;Trixagus;Trixagus carinifrons;Belarus;Bold;GMBMK390-17;-
Coleoptera;Throscidae;Trixagus;Trixagus carinifrons;Belarus;Bold;GMBMP1976-18;-
Coleoptera;Throscidae;Trixagus;Trixagus carinifrons;Belarus;Bold;GMBMP1915-18;-
Coleoptera;Throscidae;Trixagus;Trixagus carinifrons;Belarus;Bold;GMBMJ1849-17;-
Coleoptera;Throscidae;Trixagus;Trixagus carinifrons;Belarus;Bold;GMBMP1907-18;-
Coleoptera;Throscidae;Trixagus;Trixagus carinifrons;Belarus;Bold;GMBMP1900-18;-
Coleoptera;Throscidae;Trixagus;Trixagus carinifrons;Belarus;Bold;GMBMP1994-18;-
Coleoptera;Throscidae;Trixagus;Trixagus carinifrons;Belarus;Bold;GMBMP2003-18;-
Coleoptera;Throscidae;Trixagus;Trixagus carinifrons;Belarus;Bold;GMBML413-17;-
Coleoptera;Throscidae;Trixagus;Trixagus carinifrons;Norway;Bold;GMNWI3298-14;-
Coleoptera;Throscidae;Trixagus;Trixagus carinifrons;Belarus;Bold;GMBMJ1862-17;-
Coleoptera;Throscidae;Trixagus;Trixagus carinifrons;Belarus;Bold;GMBMP1902-18;-
Coleoptera;Throscidae;Trixagus;Trixagus carinifrons;Norway;Bold;GMNWF2019-14;-
Coleoptera;Throscidae;Trixagus;Trixagus carinifrons;Belarus;Bold;GMBMP1958-18;-
Coleoptera;Throscidae;Trixagus;Trixagus carinifrons;Belarus;Bold;GMBMK376-17;-
Coleoptera;Throscidae;Trixagus;Trixagus carinifrons;Belarus;Bold;GMBMJ1861-17;-
Coleoptera;Throscidae;Trixagus;Trixagus carinifrons;Belarus;Bold;GMBMJ1838-17;-
Coleoptera;Throscidae;Trixagus;Trixagus carinifrons;Belarus;Bold;GMBMP1899-18;-
Coleoptera;Throscidae;Trixagus;Trixagus carinifrons;Belarus;Bold;GMBMP1947-18;-
Coleoptera;Throscidae;Trixagus;Trixagus carinifrons;Belarus;Bold;GMBMK405-17;-
Coleoptera;Throscidae;Trixagus;Trixagus carinifrons;Belarus;Bold;GMBMP1919-18;-
Coleoptera;Throscidae;Trixagus;Trixagus carinifrons;Belarus;Bold;GMBMK339-17;-
Coleoptera;Throscidae;Trixagus;Trixagus carinifrons;Belarus;Bold;GMBMO793-17;-
Coleoptera;Throscidae;Trixagus;Trixagus carinifrons;Belarus;Bold;GMBMR1369-17;-
Coleoptera;Throscidae;Trixagus;Trixagus carinifrons;Belarus;Bold;GMBMJ1843-17;-
Coleoptera;Throscidae;Trixagus;Trixagus carinifrons;Belarus;Bold;GMBMJ1547-17;-
Coleoptera;Throscidae;Trixagus;Trixagus carinifrons;Belarus;Bold;GMBMJ1864-17;-
Coleoptera;Throscidae;Trixagus;Trixagus carinifrons;Belarus;Bold;GMBMP2023-18;-
Coleoptera;Throscidae;Trixagus;Trixagus carinifrons;Belarus;Bold;GMBMK392-17;-
Coleoptera;Throscidae;Trixagus;Trixagus carinifrons;Belarus;Bold;GMBMO844-17;-
Coleoptera;Throscidae;Trixagus;Trixagus carinifrons;Belarus;Bold;GMBMK406-17;-
Coleoptera;Throscidae;Trixagus;Trixagus carinifrons;Belarus;Bold;GMBMJ1851-17;-
Coleoptera;Throscidae;Trixagus;Trixagus carinifrons;Belarus;Bold;GMBMJ1863-17;-
Coleoptera;Throscidae;Trixagus;Trixagus carinifrons;Belarus;Bold;GMBMP1986-18;-
Coleoptera;Throscidae;Trixagus;Trixagus carinifrons;Belarus;Bold;GMBMP1936-18;-
Coleoptera;Throscidae;Trixagus;Trixagus leseigneuri;Belarus;Bold;GMBMJ1825-17;-
Coleoptera;Throscidae;Trixagus;Trixagus leseigneuri;Belarus;Bold;GMBMJ1844-17;-
Coleoptera;Throscidae;Trixagus;Trixagus leseigneuri;Belarus;Bold;GMBMJ1839-17;-
Coleoptera;Throscidae;Trixagus;Trixagus leseigneuri;Belarus;Bold;GMBMJ1829-17;-
Coleoptera;Throscidae;Trixagus;Trixagus leseigneuri;Belarus;Bold;GMBMJ1842-17;-
Coleoptera;Throscidae;Trixagus;Trixagus leseigneuri;Belarus;Bold;GMBMJ1867-17;-
Coleoptera;Throscidae;Trixagus;Trixagus leseigneuri;Belarus;Bold;GMBMJ1845-17;-
Coleoptera;Throscidae;Trixagus;Trixagus leseigneuri;Belarus;Bold;GMBMJ1846-17;-
Coleoptera;Throscidae;Trixagus;Trixagus leseigneuri;Belarus;Bold;GMBMJ1543-17;-
Coleoptera;Throscidae;Trixagus;Trixagus leseigneuri;Belarus;Bold;GMBMJ1542-17;-
Coleoptera;Curculionidae;Trypodendron;Trypodendron lineatum;Norway;Bold;SCOL061-12;-
Coleoptera;Curculionidae;Trypodendron;Trypodendron lineatum;Norway;Bold;SCOL013-12;-
Coleoptera;Tenebrionidae;Uloma;Uloma culinaris;Austria;Bold;TDAOE327-21;-
Coleoptera;Scarabaeidae;Valgus;Valgus hemipterus;Austria;Bold;TDAOE356-21;-
Coleoptera;Curculionidae;Xyleborinus;Xyleborinus saxesenii;Belarus;Bold;GMBMJ1822-17;-
Coleoptera;Curculionidae;Xyleborus;Xyleborus cryptographus;Norway;Bold;NOCLP1788-19;-
Coleoptera;Curculionidae;Xylocleptes;Xylocleptes bispinus;Ukraine;Bold;SCOL206-12;-
;;;;;;;
;;;;;;;

Appendix_5_DataRemoved_after_sequence_assignment;;;;;;
accession_number;order_name;gene;;;;
EF028525;Hymenoptera;COI;;;;
JQ909721;Hymenoptera;COI;;;;
JQ909722;Hymenoptera;COI;;;;
JQ909723;Hymenoptera;COI;;;;
JQ909724;Hymenoptera;COI;;;;
JX256662;Hymenoptera;COI;;;;
JX256691;Hymenoptera;COI;;;;
JX256692;Hymenoptera;COI;;;;
KJ836927;Hymenoptera;COI;;;;
KJ837172;Hymenoptera;COI;;;;
KJ837296;Hymenoptera;COI;;;;
KJ837411;Hymenoptera;COI;;;;
KJ837472;Hymenoptera;COI;;;;
KJ837608;Hymenoptera;COI;;;;
KJ837651;Hymenoptera;COI;;;;
KJ838205;Hymenoptera;COI;;;;
KJ838215;Hymenoptera;COI;;;;
KJ838720;Hymenoptera;COI;;;;
KJ838852;Hymenoptera;COI;;;;
KJ839028;Hymenoptera;COI;;;;
KJ839453;Hymenoptera;COI;;;;
KJ839594;Hymenoptera;COI;;;;
KJ839610;Hymenoptera;COI;;;;
KJ839693;Hymenoptera;COI;;;;
MH271384;Hymenoptera;COI;;;;
MH271385;Hymenoptera;COI;;;;
MH319126;Hymenoptera;COI;;;;
MH458485;Hymenoptera;COI;;;;
MZ628821;Hymenoptera;COI;;;;
AB674372;Lepidoptera;COI;;;;
AB674373;Lepidoptera;COI;;;;
AB674374;Lepidoptera;COI;;;;
AB674375;Lepidoptera;COI;;;;
AB674376;Lepidoptera;COI;;;;
AB674377;Lepidoptera;COI;;;;
AB674380;Lepidoptera;COI;;;;
AB674381;Lepidoptera;COI;;;;
AB674382;Lepidoptera;COI;;;;
AF408187;Lepidoptera;COI;;;;
AF408188;Lepidoptera;COI;;;;
AF408189;Lepidoptera;COI;;;;
AF408191;Lepidoptera;COI;;;;
AF408192;Lepidoptera;COI;;;;
AY346221;Lepidoptera;COI;;;;
AY346222;Lepidoptera;COI;;;;
AY346223;Lepidoptera;COI;;;;
AY346224;Lepidoptera;COI;;;;
AY346225;Lepidoptera;COI;;;;
AY346226;Lepidoptera;COI;;;;
AY346227;Lepidoptera;COI;;;;
AY346228;Lepidoptera;COI;;;;
AY346230;Lepidoptera;COI;;;;
AY350455;Lepidoptera;COI;;;;
AY350456;Lepidoptera;COI;;;;
AY350457;Lepidoptera;COI;;;;
AY350457;Lepidoptera;COI;;;;
AY350458;Lepidoptera;COI;;;;
AY350458;Lepidoptera;COI;;;;
AY350459;Lepidoptera;COI;;;;
AY350459;Lepidoptera;COI;;;;
AY350462;Lepidoptera;COI;;;;
AY350467;Lepidoptera;COI;;;;
AY350468;Lepidoptera;COI;;;;
AY350469;Lepidoptera;COI;;;;
AY350470;Lepidoptera;COI;;;;
AY350471;Lepidoptera;COI;;;;
AY350472;Lepidoptera;COI;;;;
AY350473;Lepidoptera;COI;;;;
AY350474;Lepidoptera;COI;;;;
AY350475;Lepidoptera;COI;;;;
AY350476;Lepidoptera;COI;;;;
AY350477;Lepidoptera;COI;;;;
AY350478;Lepidoptera;COI;;;;
AY350479;Lepidoptera;COI;;;;
AY556945;Lepidoptera;COI;;;;
AY556965;Lepidoptera;COI;;;;

AY556994;Lepidoptera;COI;;;;;
AY557034;Lepidoptera;COI;;;;;
AY557043;Lepidoptera;COI;;;;;
AY557126;Lepidoptera;COI;;;;;
AY557130;Lepidoptera;COI;;;;;
AY557133;Lepidoptera;COI;;;;;
AY557133;Lepidoptera;COI;;;;;
AY585886;Lepidoptera;COI;;;;;
AY585887;Lepidoptera;COI;;;;;
AY585889;Lepidoptera;COI;;;;;
AY585890;Lepidoptera;COI;;;;;
AY585891;Lepidoptera;COI;;;;;
AY675402;Lepidoptera;COI;;;;;
AY675406;Lepidoptera;COI;;;;;
AY675407;Lepidoptera;COI;;;;;
AY675413;Lepidoptera;COI;;;;;
AY675414;Lepidoptera;COI;;;;;
AY675415;Lepidoptera;COI;;;;;
AY675415;Lepidoptera;COI;;;;;
AY675416;Lepidoptera;COI;;;;;
AY675417;Lepidoptera;COI;;;;;
AY675418;Lepidoptera;COI;;;;;
AY675422;Lepidoptera;COI;;;;;
AY675428;Lepidoptera;COI;;;;;
AY675429;Lepidoptera;COI;;;;;
AY675430;Lepidoptera;COI;;;;;
AY675431;Lepidoptera;COI;;;;;
AY675432;Lepidoptera;COI;;;;;
AY675433;Lepidoptera;COI;;;;;
AY675434;Lepidoptera;COI;;;;;
AY675434;Lepidoptera;COI;;;;;
AY675435;Lepidoptera;COI;;;;;
AY675435;Lepidoptera;COI;;;;;
AY675436;Lepidoptera;COI;;;;;
AY675445;Lepidoptera;COI;;;;;
AY675447;Lepidoptera;COI;;;;;
AY675448;Lepidoptera;COI;;;;;
DQ008089;Lepidoptera;COI;;;;;
DQ205105;Lepidoptera;COI;;;;;
DQ351039;Lepidoptera;COI;;;;;
DQ384004;Lepidoptera;COI;;;;;
DQ384007;Lepidoptera;COI;;;;;
DQ384008;Lepidoptera;COI;;;;;
DQ407763;Lepidoptera;COI;;;;;
DQ463398;Lepidoptera;COI;;;;;
DQ875939;Lepidoptera;COI;;;;;
DQ875939;Lepidoptera;COI;;;;;
DQ875940;Lepidoptera;COI;;;;;
EU037851;Lepidoptera;COI;;;;;
EU037852;Lepidoptera;COI;;;;;
EU037853;Lepidoptera;COI;;;;;
EU037854;Lepidoptera;COI;;;;;
EU037855;Lepidoptera;COI;;;;;
EU037856;Lepidoptera;COI;;;;;
EU037857;Lepidoptera;COI;;;;;
EU037858;Lepidoptera;COI;;;;;
EU037859;Lepidoptera;COI;;;;;
EU037860;Lepidoptera;COI;;;;;
EU037861;Lepidoptera;COI;;;;;
EU037862;Lepidoptera;COI;;;;;
EU037863;Lepidoptera;COI;;;;;
EU037864;Lepidoptera;COI;;;;;
EU037865;Lepidoptera;COI;;;;;
EU037866;Lepidoptera;COI;;;;;
EU037884;Lepidoptera;COI;;;;;
EU037885;Lepidoptera;COI;;;;;
EU037886;Lepidoptera;COI;;;;;
EU037887;Lepidoptera;COI;;;;;
EU037889;Lepidoptera;COI;;;;;
EU364380;Lepidoptera;COI;;;;;
EU364381;Lepidoptera;COI;;;;;
EU597131;Lepidoptera;COI;;;;;
EU597132;Lepidoptera;COI;;;;;
EU597133;Lepidoptera;COI;;;;;
EU597136;Lepidoptera;COI;;;;;
EU597137;Lepidoptera;COI;;;;;
EU597138;Lepidoptera;COI;;;;;
EU597139;Lepidoptera;COI;;;;;

EU597140;Lepidoptera;COI;;;;;
EU597141;Lepidoptera;COI;;;;;
EU700961;Lepidoptera;COI;;;;;
EU700962;Lepidoptera;COI;;;;;
EU700963;Lepidoptera;COI;;;;;
EU700964;Lepidoptera;COI;;;;;
EU836659;Lepidoptera;COI;;;;;
EU836662;Lepidoptera;COI;;;;;
EU836663;Lepidoptera;COI;;;;;
EU836669;Lepidoptera;COI;;;;;
EU919314;Lepidoptera;COI;;;;;
EU919315;Lepidoptera;COI;;;;;
FJ428801;Lepidoptera;COI;;;;;
FJ428802;Lepidoptera;COI;;;;;
FJ428803;Lepidoptera;COI;;;;;
FJ428806;Lepidoptera;COI;;;;;
FJ428808;Lepidoptera;COI;;;;;
FJ428809;Lepidoptera;COI;;;;;
FJ428820;Lepidoptera;COI;;;;;
FJ428822;Lepidoptera;COI;;;;;
FJ428825;Lepidoptera;COI;;;;;
FJ628426;Lepidoptera;COI;;;;;
FJ628436;Lepidoptera;COI;;;;;
FJ628445;Lepidoptera;COI;;;;;
FJ628446;Lepidoptera;COI;;;;;
FJ663412;Lepidoptera;COI;;;;;
FJ663413;Lepidoptera;COI;;;;;
FJ663414;Lepidoptera;COI;;;;;
FJ663563;Lepidoptera;COI;;;;;
FJ663564;Lepidoptera;COI;;;;;
FJ663572;Lepidoptera;COI;;;;;
FJ663573;Lepidoptera;COI;;;;;
FJ663746;Lepidoptera;COI;;;;;
FJ663747;Lepidoptera;COI;;;;;
FJ663748;Lepidoptera;COI;;;;;
FJ663757;Lepidoptera;COI;;;;;
FJ663758;Lepidoptera;COI;;;;;
FJ663759;Lepidoptera;COI;;;;;
FJ663760;Lepidoptera;COI;;;;;
FJ663955;Lepidoptera;COI;;;;;
FJ663956;Lepidoptera;COI;;;;;
FJ663957;Lepidoptera;COI;;;;;
FJ663958;Lepidoptera;COI;;;;;
FJ663959;Lepidoptera;COI;;;;;
FJ663976;Lepidoptera;COI;;;;;
FJ663977;Lepidoptera;COI;;;;;
FJ663978;Lepidoptera;COI;;;;;
FJ663979;Lepidoptera;COI;;;;;
FJ943961;Lepidoptera;COI;;;;;
FN601259;Lepidoptera;COI;;;;;
FN601260;Lepidoptera;COI;;;;;
FN601274;Lepidoptera;COI;;;;;
FN601285;Lepidoptera;COI;;;;;
FN601301;Lepidoptera;COI;;;;;
FN601308;Lepidoptera;COI;;;;;
FN601312;Lepidoptera;COI;;;;;
FN601319;Lepidoptera;COI;;;;;
FN601329;Lepidoptera;COI;;;;;
FN601330;Lepidoptera;COI;;;;;
GQ128942;Lepidoptera;COI;;;;;
GQ128945;Lepidoptera;COI;;;;;
GQ128947;Lepidoptera;COI;;;;;
GQ128953;Lepidoptera;COI;;;;;
GQ128976;Lepidoptera;COI;;;;;
GQ128992;Lepidoptera;COI;;;;;
GQ201114;Lepidoptera;COI;;;;;
GQ885173;Lepidoptera;COI;;;;;
GU215766;Lepidoptera;COI;;;;;
GU215767;Lepidoptera;COI;;;;;
GU215768;Lepidoptera;COI;;;;;
GU215769;Lepidoptera;COI;;;;;
GU215770;Lepidoptera;COI;;;;;
GU215771;Lepidoptera;COI;;;;;
GU215772;Lepidoptera;COI;;;;;
GU215773;Lepidoptera;COI;;;;;
GU215774;Lepidoptera;COI;;;;;
GU215775;Lepidoptera;COI;;;;;
GU215776;Lepidoptera;COI;;;;;

GU215777;Lepidoptera;COI;;;;;
GU215778;Lepidoptera;COI;;;;;
GU215779;Lepidoptera;COI;;;;;
GU215780;Lepidoptera;COI;;;;;
GU215781;Lepidoptera;COI;;;;;
GU215782;Lepidoptera;COI;;;;;
GU215783;Lepidoptera;COI;;;;;
GU215784;Lepidoptera;COI;;;;;
GU215785;Lepidoptera;COI;;;;;
GU215786;Lepidoptera;COI;;;;;
GU215787;Lepidoptera;COI;;;;;
GU215788;Lepidoptera;COI;;;;;
GU215789;Lepidoptera;COI;;;;;
GU244492;Lepidoptera;COI;;;;;
GU559737;Lepidoptera;COI;;;;;
GU655006;Lepidoptera;COI;;;;;
GU655007;Lepidoptera;COI;;;;;
GU655025;Lepidoptera;COI;;;;;
GU668031;Lepidoptera;COI;;;;;
GU668032;Lepidoptera;COI;;;;;
GU669600;Lepidoptera;COI;;;;;
GU669600;Lepidoptera;COI;;;;;
GU669601;Lepidoptera;COI;;;;;
GU669601;Lepidoptera;COI;;;;;
GU669602;Lepidoptera;COI;;;;;
GU669602;Lepidoptera;COI;;;;;
GU669603;Lepidoptera;COI;;;;;
GU669603;Lepidoptera;COI;;;;;
GU669609;Lepidoptera;COI;;;;;
GU669610;Lepidoptera;COI;;;;;
GU669616;Lepidoptera;COI;;;;;
GU669624;Lepidoptera;COI;;;;;
GU669739;Lepidoptera;COI;;;;;
GU669740;Lepidoptera;COI;;;;;
GU669741;Lepidoptera;COI;;;;;
GU669818;Lepidoptera;COI;;;;;
GU669819;Lepidoptera;COI;;;;;
GU669823;Lepidoptera;COI;;;;;
GU669824;Lepidoptera;COI;;;;;
GU669825;Lepidoptera;COI;;;;;
GU669837;Lepidoptera;COI;;;;;
GU669837;Lepidoptera;COI;;;;;
GU670786;Lepidoptera;COI;;;;;
GU670791;Lepidoptera;COI;;;;;
GU670792;Lepidoptera;COI;;;;;
GU670793;Lepidoptera;COI;;;;;
GU670798;Lepidoptera;COI;;;;;
GU670799;Lepidoptera;COI;;;;;
GU670801;Lepidoptera;COI;;;;;
GU670804;Lepidoptera;COI;;;;;
GU670809;Lepidoptera;COI;;;;;
GU670813;Lepidoptera;COI;;;;;
GU670815;Lepidoptera;COI;;;;;
GU670817;Lepidoptera;COI;;;;;
GU670818;Lepidoptera;COI;;;;;
GU670821;Lepidoptera;COI;;;;;
GU670822;Lepidoptera;COI;;;;;
GU675633;Lepidoptera;COI;;;;;
GU675671;Lepidoptera;COI;;;;;
GU675671;Lepidoptera;COI;;;;;
GU675672;Lepidoptera;COI;;;;;
GU675672;Lepidoptera;COI;;;;;
GU675677;Lepidoptera;COI;;;;;
GU675696;Lepidoptera;COI;;;;;
GU675700;Lepidoptera;COI;;;;;
GU675700;Lepidoptera;COI;;;;;
GU675712;Lepidoptera;COI;;;;;
GU675742;Lepidoptera;COI;;;;;
GU675743;Lepidoptera;COI;;;;;
GU675744;Lepidoptera;COI;;;;;
GU675758;Lepidoptera;COI;;;;;
GU675759;Lepidoptera;COI;;;;;
GU675764;Lepidoptera;COI;;;;;
GU675774;Lepidoptera;COI;;;;;
GU675800;Lepidoptera;COI;;;;;
GU675825;Lepidoptera;COI;;;;;
GU675826;Lepidoptera;COI;;;;;
GU675851;Lepidoptera;COI;;;;;

GU675852;Lepidoptera;COI;;;;;
GU675896;Lepidoptera;COI;;;;;
GU675896;Lepidoptera;COI;;;;;
GU675936;Lepidoptera;COI;;;;;
GU675938;Lepidoptera;COI;;;;;
GU675957;Lepidoptera;COI;;;;;
GU675960;Lepidoptera;COI;;;;;
GU675975;Lepidoptera;COI;;;;;
GU675976;Lepidoptera;COI;;;;;
GU675977;Lepidoptera;COI;;;;;
GU675977;Lepidoptera;COI;;;;;
GU676017;Lepidoptera;COI;;;;;
GU676020;Lepidoptera;COI;;;;;
GU676041;Lepidoptera;COI;;;;;
GU676041;Lepidoptera;COI;;;;;
GU676088;Lepidoptera;COI;;;;;
GU676091;Lepidoptera;COI;;;;;
GU676169;Lepidoptera;COI;;;;;
GU676209;Lepidoptera;COI;;;;;
GU676228;Lepidoptera;COI;;;;;
GU676228;Lepidoptera;COI;;;;;
GU676236;Lepidoptera;COI;;;;;
GU676236;Lepidoptera;COI;;;;;
GU676277;Lepidoptera;COI;;;;;
GU676304;Lepidoptera;COI;;;;;
GU676351;Lepidoptera;COI;;;;;
GU676352;Lepidoptera;COI;;;;;
GU676362;Lepidoptera;COI;;;;;
GU676363;Lepidoptera;COI;;;;;
GU676368;Lepidoptera;COI;;;;;
GU676368;Lepidoptera;COI;;;;;
GU676373;Lepidoptera;COI;;;;;
GU676373;Lepidoptera;COI;;;;;
GU676377;Lepidoptera;COI;;;;;
GU676377;Lepidoptera;COI;;;;;
GU676383;Lepidoptera;COI;;;;;
GU676383;Lepidoptera;COI;;;;;
GU676408;Lepidoptera;COI;;;;;
GU676408;Lepidoptera;COI;;;;;
GU676425;Lepidoptera;COI;;;;;
GU676490;Lepidoptera;COI;;;;;
GU676490;Lepidoptera;COI;;;;;
GU676500;Lepidoptera;COI;;;;;
GU676516;Lepidoptera;COI;;;;;
GU676556;Lepidoptera;COI;;;;;
GU676558;Lepidoptera;COI;;;;;
GU676716;Lepidoptera;COI;;;;;
GU676734;Lepidoptera;COI;;;;;
GU676825;Lepidoptera;COI;;;;;
GU676826;Lepidoptera;COI;;;;;
GU676831;Lepidoptera;COI;;;;;
GU676832;Lepidoptera;COI;;;;;
GU676892;Lepidoptera;COI;;;;;
GU676893;Lepidoptera;COI;;;;;
GU676910;Lepidoptera;COI;;;;;
GU676956;Lepidoptera;COI;;;;;
GU676970;Lepidoptera;COI;;;;;
GU676970;Lepidoptera;COI;;;;;
GU676990;Lepidoptera;COI;;;;;
GU676991;Lepidoptera;COI;;;;;
GU677010;Lepidoptera;COI;;;;;
GU677010;Lepidoptera;COI;;;;;
GU677011;Lepidoptera;COI;;;;;
GU677011;Lepidoptera;COI;;;;;
GU686975;Lepidoptera;COI;;;;;
GU687045;Lepidoptera;COI;;;;;
GU687046;Lepidoptera;COI;;;;;
GU687048;Lepidoptera;COI;;;;;
GU688446;Lepidoptera;COI;;;;;
GU688446;Lepidoptera;COI;;;;;
GU688453;Lepidoptera;COI;;;;;
GU688454;Lepidoptera;COI;;;;;
GU688456;Lepidoptera;COI;;;;;
GU688470;Lepidoptera;COI;;;;;
GU688507;Lepidoptera;COI;;;;;
GU688510;Lepidoptera;COI;;;;;
GU688532;Lepidoptera;COI;;;;;
GU688532;Lepidoptera;COI;;;;;

GU689149;Lepidoptera;COI;;;;;
GU707036;Lepidoptera;COI;;;;;
GU707088;Lepidoptera;COI;;;;;
GU707090;Lepidoptera;COI;;;;;
GU707115;Lepidoptera;COI;;;;;
GU707134;Lepidoptera;COI;;;;;
GU707145;Lepidoptera;COI;;;;;
GU707194;Lepidoptera;COI;;;;;
GU707266;Lepidoptera;COI;;;;;
GU707371;Lepidoptera;COI;;;;;
GU828690;Lepidoptera;COI;;;;;
GU947444;Lepidoptera;COI;;;;;
GU947445;Lepidoptera;COI;;;;;
GU947447;Lepidoptera;COI;;;;;
GU947448;Lepidoptera;COI;;;;;
GU947449;Lepidoptera;COI;;;;;
GU947450;Lepidoptera;COI;;;;;
GU947451;Lepidoptera;COI;;;;;
GU947452;Lepidoptera;COI;;;;;
GU947453;Lepidoptera;COI;;;;;
GU947454;Lepidoptera;COI;;;;;
GU947455;Lepidoptera;COI;;;;;
GU947456;Lepidoptera;COI;;;;;
GU947458;Lepidoptera;COI;;;;;
GU947459;Lepidoptera;COI;;;;;
GU947460;Lepidoptera;COI;;;;;
GU947461;Lepidoptera;COI;;;;;
GU947462;Lepidoptera;COI;;;;;
GU947463;Lepidoptera;COI;;;;;
GU947464;Lepidoptera;COI;;;;;
GU947468;Lepidoptera;COI;;;;;
GU947473;Lepidoptera;COI;;;;;
GU947474;Lepidoptera;COI;;;;;
GU947475;Lepidoptera;COI;;;;;
GU947478;Lepidoptera;COI;;;;;
GU947487;Lepidoptera;COI;;;;;
GU947488;Lepidoptera;COI;;;;;
GU947489;Lepidoptera;COI;;;;;
GU947490;Lepidoptera;COI;;;;;
GU947491;Lepidoptera;COI;;;;;
GU947492;Lepidoptera;COI;;;;;
GU947493;Lepidoptera;COI;;;;;
GU947494;Lepidoptera;COI;;;;;
GU947495;Lepidoptera;COI;;;;;
GU947496;Lepidoptera;COI;;;;;
GU947497;Lepidoptera;COI;;;;;
GU947498;Lepidoptera;COI;;;;;
GU947499;Lepidoptera;COI;;;;;
GU947501;Lepidoptera;COI;;;;;
GU947502;Lepidoptera;COI;;;;;
GU947503;Lepidoptera;COI;;;;;
GU947504;Lepidoptera;COI;;;;;
GU947505;Lepidoptera;COI;;;;;
GU947506;Lepidoptera;COI;;;;;
GU947507;Lepidoptera;COI;;;;;
GU947508;Lepidoptera;COI;;;;;
GU947509;Lepidoptera;COI;;;;;
GU947510;Lepidoptera;COI;;;;;
GU947511;Lepidoptera;COI;;;;;
GU947512;Lepidoptera;COI;;;;;
GU947513;Lepidoptera;COI;;;;;
GU947514;Lepidoptera;COI;;;;;
GU947515;Lepidoptera;COI;;;;;
GU947516;Lepidoptera;COI;;;;;
GU947517;Lepidoptera;COI;;;;;
GU947518;Lepidoptera;COI;;;;;
GU947519;Lepidoptera;COI;;;;;
GU947520;Lepidoptera;COI;;;;;
GU947521;Lepidoptera;COI;;;;;
GU947522;Lepidoptera;COI;;;;;
GU947523;Lepidoptera;COI;;;;;
GU947524;Lepidoptera;COI;;;;;
GU947525;Lepidoptera;COI;;;;;
GU947526;Lepidoptera;COI;;;;;
GU947527;Lepidoptera;COI;;;;;
GU947528;Lepidoptera;COI;;;;;
GU947529;Lepidoptera;COI;;;;;
GU947530;Lepidoptera;COI;;;;;

GU947531;Lepidoptera;COI;;;;;
GU947532;Lepidoptera;COI;;;;;
GU947533;Lepidoptera;COI;;;;;
GU947534;Lepidoptera;COI;;;;;
GU947535;Lepidoptera;COI;;;;;
GU947536;Lepidoptera;COI;;;;;
GU947537;Lepidoptera;COI;;;;;
GU947538;Lepidoptera;COI;;;;;
GU947539;Lepidoptera;COI;;;;;
GU947540;Lepidoptera;COI;;;;;
GU947541;Lepidoptera;COI;;;;;
GU947542;Lepidoptera;COI;;;;;
GU947543;Lepidoptera;COI;;;;;
GU947544;Lepidoptera;COI;;;;;
GU947545;Lepidoptera;COI;;;;;
GU947546;Lepidoptera;COI;;;;;
GU947547;Lepidoptera;COI;;;;;
GU947548;Lepidoptera;COI;;;;;
GU947549;Lepidoptera;COI;;;;;
GU947550;Lepidoptera;COI;;;;;
GU947551;Lepidoptera;COI;;;;;
GU947552;Lepidoptera;COI;;;;;
GU947553;Lepidoptera;COI;;;;;
GU947554;Lepidoptera;COI;;;;;
GU947555;Lepidoptera;COI;;;;;
GU947556;Lepidoptera;COI;;;;;
GU947557;Lepidoptera;COI;;;;;
GU947558;Lepidoptera;COI;;;;;
GU947559;Lepidoptera;COI;;;;;
GU947560;Lepidoptera;COI;;;;;
GU947561;Lepidoptera;COI;;;;;
GU947562;Lepidoptera;COI;;;;;
GU947563;Lepidoptera;COI;;;;;
GU947564;Lepidoptera;COI;;;;;
GU947565;Lepidoptera;COI;;;;;
GU947566;Lepidoptera;COI;;;;;
GU947567;Lepidoptera;COI;;;;;
GU947568;Lepidoptera;COI;;;;;
GU947569;Lepidoptera;COI;;;;;
GU947570;Lepidoptera;COI;;;;;
GU947571;Lepidoptera;COI;;;;;
GU947572;Lepidoptera;COI;;;;;
GU947573;Lepidoptera;COI;;;;;
GU947574;Lepidoptera;COI;;;;;
GU947575;Lepidoptera;COI;;;;;
GU947583;Lepidoptera;COI;;;;;
GU947584;Lepidoptera;COI;;;;;
GU947585;Lepidoptera;COI;;;;;
GU947586;Lepidoptera;COI;;;;;
GU947587;Lepidoptera;COI;;;;;
GU947588;Lepidoptera;COI;;;;;
GU947589;Lepidoptera;COI;;;;;
GU947591;Lepidoptera;COI;;;;;
GU947592;Lepidoptera;COI;;;;;
GU947593;Lepidoptera;COI;;;;;
GU947594;Lepidoptera;COI;;;;;
GU947595;Lepidoptera;COI;;;;;
GU947596;Lepidoptera;COI;;;;;
GU947597;Lepidoptera;COI;;;;;
GU947598;Lepidoptera;COI;;;;;
GU947599;Lepidoptera;COI;;;;;
GU947600;Lepidoptera;COI;;;;;
GU947614;Lepidoptera;COI;;;;;
GU947615;Lepidoptera;COI;;;;;
GU947618;Lepidoptera;COI;;;;;
GU947619;Lepidoptera;COI;;;;;
GU947622;Lepidoptera;COI;;;;;
GU947627;Lepidoptera;COI;;;;;
GU947632;Lepidoptera;COI;;;;;
GU947633;Lepidoptera;COI;;;;;
GU947634;Lepidoptera;COI;;;;;
GU947635;Lepidoptera;COI;;;;;
GU947637;Lepidoptera;COI;;;;;
HG969225;Lepidoptera;COI;;;;;
HM116932;Lepidoptera;COI;;;;;
HM116935;Lepidoptera;COI;;;;;
HM116939;Lepidoptera;COI;;;;;
HM159431;Lepidoptera;COI;;;;;

HM210163;Lepidoptera;COI;;;;;
HM210163;Lepidoptera;COI;;;;;
HM210164;Lepidoptera;COI;;;;;
HM210164;Lepidoptera;COI;;;;;
HM210167;Lepidoptera;COI;;;;;
HM210167;Lepidoptera;COI;;;;;
HM210168;Lepidoptera;COI;;;;;
HM210168;Lepidoptera;COI;;;;;
HM210172;Lepidoptera;COI;;;;;
HM210172;Lepidoptera;COI;;;;;
HM391783;Lepidoptera;COI;;;;;
HM391817;Lepidoptera;COI;;;;;
HM391889;Lepidoptera;COI;;;;;
HM393176;Lepidoptera;COI;;;;;
HM393185;Lepidoptera;COI;;;;;
HM400986;Lepidoptera;COI;;;;;
HM400986;Lepidoptera;COI;;;;;
HM400987;Lepidoptera;COI;;;;;
HM400987;Lepidoptera;COI;;;;;
HM400988;Lepidoptera;COI;;;;;
HM400988;Lepidoptera;COI;;;;;
HM400989;Lepidoptera;COI;;;;;
HM400989;Lepidoptera;COI;;;;;
HM401009;Lepidoptera;COI;;;;;
HM401010;Lepidoptera;COI;;;;;
HM401011;Lepidoptera;COI;;;;;
HM401012;Lepidoptera;COI;;;;;
HM401018;Lepidoptera;COI;;;;;
HM401019;Lepidoptera;COI;;;;;
HM425866;Lepidoptera;COI;;;;;
HM425928;Lepidoptera;COI;;;;;
HM425954;Lepidoptera;COI;;;;;
HM870991;Lepidoptera;COI;;;;;
HM870992;Lepidoptera;COI;;;;;
HM871926;Lepidoptera;COI;;;;;
HM871926;Lepidoptera;COI;;;;;
HM871927;Lepidoptera;COI;;;;;
HM871927;Lepidoptera;COI;;;;;
HM871931;Lepidoptera;COI;;;;;
HM871931;Lepidoptera;COI;;;;;
HM871939;Lepidoptera;COI;;;;;
HM873306;Lepidoptera;COI;;;;;
HM873544;Lepidoptera;COI;;;;;
HM873572;Lepidoptera;COI;;;;;
HM874159;Lepidoptera;COI;;;;;
HM874826;Lepidoptera;COI;;;;;
HM874827;Lepidoptera;COI;;;;;
HM901257;Lepidoptera;COI;;;;;
HM901290;Lepidoptera;COI;;;;;
HM901344;Lepidoptera;COI;;;;;
HM901344;Lepidoptera;COI;;;;;
HM901378;Lepidoptera;COI;;;;;
HM901378;Lepidoptera;COI;;;;;
HM901380;Lepidoptera;COI;;;;;
HM901380;Lepidoptera;COI;;;;;
HM901382;Lepidoptera;COI;;;;;
HM901382;Lepidoptera;COI;;;;;
HM901419;Lepidoptera;COI;;;;;
HM901419;Lepidoptera;COI;;;;;
HM901420;Lepidoptera;COI;;;;;
HM901420;Lepidoptera;COI;;;;;
HM901546;Lepidoptera;COI;;;;;
HM901617;Lepidoptera;COI;;;;;
HM901721;Lepidoptera;COI;;;;;
HM901728;Lepidoptera;COI;;;;;
HM901777;Lepidoptera;COI;;;;;
HM901791;Lepidoptera;COI;;;;;
HM901837;Lepidoptera;COI;;;;;
HM901838;Lepidoptera;COI;;;;;
HM904299;Lepidoptera;COI;;;;;
HM904301;Lepidoptera;COI;;;;;
HM904306;Lepidoptera;COI;;;;;
HM904312;Lepidoptera;COI;;;;;
HM904315;Lepidoptera;COI;;;;;
HM910016;Lepidoptera;COI;;;;;
HM910016;Lepidoptera;COI;;;;;
HM910017;Lepidoptera;COI;;;;;
HM910017;Lepidoptera;COI;;;;;

HM910019;Lepidoptera;COI;;;;;
HM910019;Lepidoptera;COI;;;;;
HM910574;Lepidoptera;COI;;;;;
HM913944;Lepidoptera;COI;;;;;
HM913962;Lepidoptera;COI;;;;;
HM913964;Lepidoptera;COI;;;;;
HM913965;Lepidoptera;COI;;;;;
HQ004015;Lepidoptera;COI;;;;;
HQ004017;Lepidoptera;COI;;;;;
HQ004021;Lepidoptera;COI;;;;;
HQ004190;Lepidoptera;COI;;;;;
HQ004210;Lepidoptera;COI;;;;;
HQ004211;Lepidoptera;COI;;;;;
HQ004212;Lepidoptera;COI;;;;;
HQ004213;Lepidoptera;COI;;;;;
HQ004214;Lepidoptera;COI;;;;;
HQ004215;Lepidoptera;COI;;;;;
HQ004216;Lepidoptera;COI;;;;;
HQ004217;Lepidoptera;COI;;;;;
HQ004218;Lepidoptera;COI;;;;;
HQ004274;Lepidoptera;COI;;;;;
HQ004275;Lepidoptera;COI;;;;;
HQ004276;Lepidoptera;COI;;;;;
HQ004277;Lepidoptera;COI;;;;;
HQ004278;Lepidoptera;COI;;;;;
HQ004279;Lepidoptera;COI;;;;;
HQ004280;Lepidoptera;COI;;;;;
HQ004281;Lepidoptera;COI;;;;;
HQ004310;Lepidoptera;COI;;;;;
HQ004311;Lepidoptera;COI;;;;;
HQ004312;Lepidoptera;COI;;;;;
HQ004314;Lepidoptera;COI;;;;;
HQ004315;Lepidoptera;COI;;;;;
HQ004316;Lepidoptera;COI;;;;;
HQ004317;Lepidoptera;COI;;;;;
HQ004318;Lepidoptera;COI;;;;;
HQ004362;Lepidoptera;COI;;;;;
HQ004363;Lepidoptera;COI;;;;;
HQ004364;Lepidoptera;COI;;;;;
HQ004365;Lepidoptera;COI;;;;;
HQ004366;Lepidoptera;COI;;;;;
HQ004367;Lepidoptera;COI;;;;;
HQ004368;Lepidoptera;COI;;;;;
HQ004398;Lepidoptera;COI;;;;;
HQ004399;Lepidoptera;COI;;;;;
HQ004400;Lepidoptera;COI;;;;;
HQ004484;Lepidoptera;COI;;;;;
HQ004485;Lepidoptera;COI;;;;;
HQ004486;Lepidoptera;COI;;;;;
HQ004730;Lepidoptera;COI;;;;;
HQ004732;Lepidoptera;COI;;;;;
HQ004733;Lepidoptera;COI;;;;;
HQ004735;Lepidoptera;COI;;;;;
HQ004736;Lepidoptera;COI;;;;;
HQ004737;Lepidoptera;COI;;;;;
HQ004738;Lepidoptera;COI;;;;;
HQ004739;Lepidoptera;COI;;;;;
HQ004839;Lepidoptera;COI;;;;;
HQ004919;Lepidoptera;COI;;;;;
HQ004920;Lepidoptera;COI;;;;;
HQ004921;Lepidoptera;COI;;;;;
HQ004922;Lepidoptera;COI;;;;;
HQ004923;Lepidoptera;COI;;;;;
HQ004924;Lepidoptera;COI;;;;;
HQ004925;Lepidoptera;COI;;;;;
HQ004926;Lepidoptera;COI;;;;;
HQ004927;Lepidoptera;COI;;;;;
HQ004928;Lepidoptera;COI;;;;;
HQ004939;Lepidoptera;COI;;;;;
HQ004951;Lepidoptera;COI;;;;;
HQ004977;Lepidoptera;COI;;;;;
HQ004978;Lepidoptera;COI;;;;;
HQ004979;Lepidoptera;COI;;;;;
HQ004980;Lepidoptera;COI;;;;;
HQ004981;Lepidoptera;COI;;;;;
HQ004982;Lepidoptera;COI;;;;;
HQ004983;Lepidoptera;COI;;;;;
HQ004984;Lepidoptera;COI;;;;;

HQ004985;Lepidoptera;COI;;;;;
HQ004986;Lepidoptera;COI;;;;;
HQ004987;Lepidoptera;COI;;;;;
HQ004988;Lepidoptera;COI;;;;;
HQ004989;Lepidoptera;COI;;;;;
HQ004990;Lepidoptera;COI;;;;;
HQ004991;Lepidoptera;COI;;;;;
HQ004992;Lepidoptera;COI;;;;;
HQ004992;Lepidoptera;COI;;;;;
HQ004993;Lepidoptera;COI;;;;;
HQ004993;Lepidoptera;COI;;;;;
HQ005002;Lepidoptera;COI;;;;;
HQ005003;Lepidoptera;COI;;;;;
HQ005004;Lepidoptera;COI;;;;;
HQ005005;Lepidoptera;COI;;;;;
HQ005006;Lepidoptera;COI;;;;;
HQ005007;Lepidoptera;COI;;;;;
HQ005008;Lepidoptera;COI;;;;;
HQ005010;Lepidoptera;COI;;;;;
HQ005011;Lepidoptera;COI;;;;;
HQ005012;Lepidoptera;COI;;;;;
HQ005013;Lepidoptera;COI;;;;;
HQ005014;Lepidoptera;COI;;;;;
HQ005015;Lepidoptera;COI;;;;;
HQ005017;Lepidoptera;COI;;;;;
HQ005018;Lepidoptera;COI;;;;;
HQ005019;Lepidoptera;COI;;;;;
HQ005030;Lepidoptera;COI;;;;;
HQ005082;Lepidoptera;COI;;;;;
HQ005083;Lepidoptera;COI;;;;;
HQ005084;Lepidoptera;COI;;;;;
HQ005085;Lepidoptera;COI;;;;;
HQ005086;Lepidoptera;COI;;;;;
HQ005087;Lepidoptera;COI;;;;;
HQ005088;Lepidoptera;COI;;;;;
HQ005089;Lepidoptera;COI;;;;;
HQ005090;Lepidoptera;COI;;;;;
HQ176010;Lepidoptera;COI;;;;;
HQ176011;Lepidoptera;COI;;;;;
HQ176012;Lepidoptera;COI;;;;;
HQ176013;Lepidoptera;COI;;;;;
HQ176014;Lepidoptera;COI;;;;;
HQ176015;Lepidoptera;COI;;;;;
HQ176016;Lepidoptera;COI;;;;;
HQ176017;Lepidoptera;COI;;;;;
HQ176018;Lepidoptera;COI;;;;;
HQ176019;Lepidoptera;COI;;;;;
HQ176020;Lepidoptera;COI;;;;;
HQ176021;Lepidoptera;COI;;;;;
HQ224581;Lepidoptera;COI;;;;;
HQ224582;Lepidoptera;COI;;;;;
HQ224583;Lepidoptera;COI;;;;;
HQ224584;Lepidoptera;COI;;;;;
HQ563537;Lepidoptera;COI;;;;;
HQ563539;Lepidoptera;COI;;;;;
HQ563539;Lepidoptera;COI;;;;;
HQ563540;Lepidoptera;COI;;;;;
HQ563542;Lepidoptera;COI;;;;;
HQ563543;Lepidoptera;COI;;;;;
HQ563543;Lepidoptera;COI;;;;;
HQ563553;Lepidoptera;COI;;;;;
HQ563554;Lepidoptera;COI;;;;;
HQ563557;Lepidoptera;COI;;;;;
HQ563557;Lepidoptera;COI;;;;;
HQ563558;Lepidoptera;COI;;;;;
HQ563558;Lepidoptera;COI;;;;;
HQ563561;Lepidoptera;COI;;;;;
HQ563583;Lepidoptera;COI;;;;;
HQ563583;Lepidoptera;COI;;;;;
HQ563585;Lepidoptera;COI;;;;;
HQ563585;Lepidoptera;COI;;;;;
HQ565464;Lepidoptera;COI;;;;;
HQ565468;Lepidoptera;COI;;;;;
HQ565492;Lepidoptera;COI;;;;;
HQ565493;Lepidoptera;COI;;;;;
HQ565495;Lepidoptera;COI;;;;;
HQ570288;Lepidoptera;COI;;;;;
HQ918141;Lepidoptera;COI;;;;;

HQ918142;Lepidoptera;COI;;;;;
HQ918143;Lepidoptera;COI;;;;;
HQ918144;Lepidoptera;COI;;;;;
HQ918145;Lepidoptera;COI;;;;;
HQ918146;Lepidoptera;COI;;;;;
HQ918147;Lepidoptera;COI;;;;;
HQ918148;Lepidoptera;COI;;;;;
HQ918149;Lepidoptera;COI;;;;;
HQ918153;Lepidoptera;COI;;;;;
HQ918153;Lepidoptera;COI;;;;;
HQ918154;Lepidoptera;COI;;;;;
HQ918154;Lepidoptera;COI;;;;;
HQ918155;Lepidoptera;COI;;;;;
HQ918155;Lepidoptera;COI;;;;;
HQ918156;Lepidoptera;COI;;;;;
HQ918156;Lepidoptera;COI;;;;;
HQ918157;Lepidoptera;COI;;;;;
HQ918157;Lepidoptera;COI;;;;;
HQ918159;Lepidoptera;COI;;;;;
HQ918160;Lepidoptera;COI;;;;;
HQ918163;Lepidoptera;COI;;;;;
HQ918164;Lepidoptera;COI;;;;;
HQ918167;Lepidoptera;COI;;;;;
HQ957211;Lepidoptera;COI;;;;;
HQ968391;Lepidoptera;COI;;;;;
HQ968391;Lepidoptera;COI;;;;;
HQ968456;Lepidoptera;COI;;;;;
HQ968457;Lepidoptera;COI;;;;;
HQ968485;Lepidoptera;COI;;;;;
HQ968488;Lepidoptera;COI;;;;;
HQ968514;Lepidoptera;COI;;;;;
HQ968514;Lepidoptera;COI;;;;;
HQ968515;Lepidoptera;COI;;;;;
HQ968515;Lepidoptera;COI;;;;;
HQ968519;Lepidoptera;COI;;;;;
HQ968530;Lepidoptera;COI;;;;;
HQ968531;Lepidoptera;COI;;;;;
JF415712;Lepidoptera;COI;;;;;
JF415724;Lepidoptera;COI;;;;;
JF415724;Lepidoptera;COI;;;;;
JF415725;Lepidoptera;COI;;;;;
JF810413;Lepidoptera;COI;;;;;
JF850429;Lepidoptera;COI;;;;;
JF850432;Lepidoptera;COI;;;;;
JF853624;Lepidoptera;COI;;;;;
JF853632;Lepidoptera;COI;;;;;
JF853653;Lepidoptera;COI;;;;;
JF853654;Lepidoptera;COI;;;;;
JF853665;Lepidoptera;COI;;;;;
JF853832;Lepidoptera;COI;;;;;
JF854493;Lepidoptera;COI;;;;;
JN084688;Lepidoptera;COI;;;;;
JN265786;Lepidoptera;COI;;;;;
JN276895;Lepidoptera;COI;;;;;
JN276895;Lepidoptera;COI;;;;;
JN276897;Lepidoptera;COI;;;;;
JN276897;Lepidoptera;COI;;;;;
JN276902;Lepidoptera;COI;;;;;
JN276904;Lepidoptera;COI;;;;;
JN276904;Lepidoptera;COI;;;;;
JN276908;Lepidoptera;COI;;;;;
JN276910;Lepidoptera;COI;;;;;
JN276911;Lepidoptera;COI;;;;;
JN276911;Lepidoptera;COI;;;;;
JN276914;Lepidoptera;COI;;;;;
JN277563;Lepidoptera;COI;;;;;
JN277563;Lepidoptera;COI;;;;;
JN277571;Lepidoptera;COI;;;;;
JN827879;Lepidoptera;COI;;;;;
JX013970;Lepidoptera;COI;;;;;
JX013971;Lepidoptera;COI;;;;;
JX013972;Lepidoptera;COI;;;;;
JX013973;Lepidoptera;COI;;;;;
JX013974;Lepidoptera;COI;;;;;
JX093461;Lepidoptera;COI;;;;;
JX155755;Lepidoptera;COI;;;;;
JX311069;Lepidoptera;COI;;;;;
JX311075;Lepidoptera;COI;;;;;

JX311078;Lepidoptera;COI;;;;;
JX311083;Lepidoptera;COI;;;;;
JX311084;Lepidoptera;COI;;;;;
JX311086;Lepidoptera;COI;;;;;
JX311087;Lepidoptera;COI;;;;;
JX311093;Lepidoptera;COI;;;;;
JX311096;Lepidoptera;COI;;;;;
JX311100;Lepidoptera;COI;;;;;
JX311101;Lepidoptera;COI;;;;;
JX311102;Lepidoptera;COI;;;;;
JX311103;Lepidoptera;COI;;;;;
JX311104;Lepidoptera;COI;;;;;
JX311105;Lepidoptera;COI;;;;;
JX311106;Lepidoptera;COI;;;;;
JX311107;Lepidoptera;COI;;;;;
JX311108;Lepidoptera;COI;;;;;
JX311109;Lepidoptera;COI;;;;;
JX311110;Lepidoptera;COI;;;;;
JX311111;Lepidoptera;COI;;;;;
JX311113;Lepidoptera;COI;;;;;
JX311114;Lepidoptera;COI;;;;;
JX311116;Lepidoptera;COI;;;;;
JX311117;Lepidoptera;COI;;;;;
JX311118;Lepidoptera;COI;;;;;
JX311119;Lepidoptera;COI;;;;;
JX311120;Lepidoptera;COI;;;;;
JX311121;Lepidoptera;COI;;;;;
JX311122;Lepidoptera;COI;;;;;
JX311126;Lepidoptera;COI;;;;;
JX311128;Lepidoptera;COI;;;;;
JX311129;Lepidoptera;COI;;;;;
JX311130;Lepidoptera;COI;;;;;
JX311134;Lepidoptera;COI;;;;;
JX311135;Lepidoptera;COI;;;;;
JX311136;Lepidoptera;COI;;;;;
JX311137;Lepidoptera;COI;;;;;
JX311138;Lepidoptera;COI;;;;;
JX311139;Lepidoptera;COI;;;;;
JX311157;Lepidoptera;COI;;;;;
JX311162;Lepidoptera;COI;;;;;
JX311163;Lepidoptera;COI;;;;;
JX311164;Lepidoptera;COI;;;;;
JX311165;Lepidoptera;COI;;;;;
JX311167;Lepidoptera;COI;;;;;
JX311168;Lepidoptera;COI;;;;;
JX311169;Lepidoptera;COI;;;;;
JX311182;Lepidoptera;COI;;;;;
JX311183;Lepidoptera;COI;;;;;
JX311189;Lepidoptera;COI;;;;;
JX311190;Lepidoptera;COI;;;;;
JX311193;Lepidoptera;COI;;;;;
JX311194;Lepidoptera;COI;;;;;
JX311195;Lepidoptera;COI;;;;;
JX311196;Lepidoptera;COI;;;;;
JX311197;Lepidoptera;COI;;;;;
JX311198;Lepidoptera;COI;;;;;
JX311199;Lepidoptera;COI;;;;;
JX311200;Lepidoptera;COI;;;;;
JX311201;Lepidoptera;COI;;;;;
JX311202;Lepidoptera;COI;;;;;
JX311207;Lepidoptera;COI;;;;;
JX311208;Lepidoptera;COI;;;;;
JX311209;Lepidoptera;COI;;;;;
JX311210;Lepidoptera;COI;;;;;
JX311211;Lepidoptera;COI;;;;;
JX311213;Lepidoptera;COI;;;;;
JX311214;Lepidoptera;COI;;;;;
JX311215;Lepidoptera;COI;;;;;
JX311216;Lepidoptera;COI;;;;;
JX311217;Lepidoptera;COI;;;;;
JX311223;Lepidoptera;COI;;;;;
JX311225;Lepidoptera;COI;;;;;
JX311226;Lepidoptera;COI;;;;;
JX311227;Lepidoptera;COI;;;;;
JX311229;Lepidoptera;COI;;;;;
JX311230;Lepidoptera;COI;;;;;
JX311231;Lepidoptera;COI;;;;;
JX311232;Lepidoptera;COI;;;;;

JX311250;Lepidoptera;COI;;;;;
JX311254;Lepidoptera;COI;;;;;
JX311255;Lepidoptera;COI;;;;;
JX311260;Lepidoptera;COI;;;;;
JX311261;Lepidoptera;COI;;;;;
JX311262;Lepidoptera;COI;;;;;
JX311263;Lepidoptera;COI;;;;;
JX311264;Lepidoptera;COI;;;;;
JX311265;Lepidoptera;COI;;;;;
JX311266;Lepidoptera;COI;;;;;
JX311267;Lepidoptera;COI;;;;;
JX311268;Lepidoptera;COI;;;;;
JX311269;Lepidoptera;COI;;;;;
JX311270;Lepidoptera;COI;;;;;
JX311271;Lepidoptera;COI;;;;;
JX311272;Lepidoptera;COI;;;;;
JX311273;Lepidoptera;COI;;;;;
JX311275;Lepidoptera;COI;;;;;
KC462786;Lepidoptera;COI;;;;;
KC462787;Lepidoptera;COI;;;;;
KC462797;Lepidoptera;COI;;;;;
KC462798;Lepidoptera;COI;;;;;
KC462799;Lepidoptera;COI;;;;;
KC462803;Lepidoptera;COI;;;;;
KC462812;Lepidoptera;COI;;;;;
KC462813;Lepidoptera;COI;;;;;
KC462814;Lepidoptera;COI;;;;;
KC462822;Lepidoptera;COI;;;;;
KC462827;Lepidoptera;COI;;;;;
KC462832;Lepidoptera;COI;;;;;
KC462833;Lepidoptera;COI;;;;;
KC462840;Lepidoptera;COI;;;;;
KC462841;Lepidoptera;COI;;;;;
KC462847;Lepidoptera;COI;;;;;
KC462848;Lepidoptera;COI;;;;;
KC462854;Lepidoptera;COI;;;;;
KC462855;Lepidoptera;COI;;;;;
KC462856;Lepidoptera;COI;;;;;
KC462857;Lepidoptera;COI;;;;;
KC462858;Lepidoptera;COI;;;;;
KC462859;Lepidoptera;COI;;;;;
KC462860;Lepidoptera;COI;;;;;
KC462861;Lepidoptera;COI;;;;;
KC462862;Lepidoptera;COI;;;;;
KC462863;Lepidoptera;COI;;;;;
KC462864;Lepidoptera;COI;;;;;
KC462867;Lepidoptera;COI;;;;;
KC462868;Lepidoptera;COI;;;;;
KC462872;Lepidoptera;COI;;;;;
KC462873;Lepidoptera;COI;;;;;
KC692330;Lepidoptera;COI;;;;;
KC967227;Lepidoptera;COI;;;;;
KC967227;Lepidoptera;COI;;;;;
KC967228;Lepidoptera;COI;;;;;
KC967228;Lepidoptera;COI;;;;;
KC967229;Lepidoptera;COI;;;;;
KC967229;Lepidoptera;COI;;;;;
KC967230;Lepidoptera;COI;;;;;
KC967230;Lepidoptera;COI;;;;;
KC967231;Lepidoptera;COI;;;;;
KC967231;Lepidoptera;COI;;;;;
KC967232;Lepidoptera;COI;;;;;
KC967232;Lepidoptera;COI;;;;;
KC967233;Lepidoptera;COI;;;;;
KC967233;Lepidoptera;COI;;;;;
KC967234;Lepidoptera;COI;;;;;
KC967234;Lepidoptera;COI;;;;;
KC967235;Lepidoptera;COI;;;;;
KC967235;Lepidoptera;COI;;;;;
KC967236;Lepidoptera;COI;;;;;
KC967236;Lepidoptera;COI;;;;;
KC967237;Lepidoptera;COI;;;;;
KC967237;Lepidoptera;COI;;;;;
KC967238;Lepidoptera;COI;;;;;
KC967238;Lepidoptera;COI;;;;;
KC967239;Lepidoptera;COI;;;;;
KC967239;Lepidoptera;COI;;;;;
KC967240;Lepidoptera;COI;;;;;

KC967240;Lepidoptera;COI;;;;;
KC967241;Lepidoptera;COI;;;;;
KC967241;Lepidoptera;COI;;;;;
KC967242;Lepidoptera;COI;;;;;
KC967242;Lepidoptera;COI;;;;;
KC967243;Lepidoptera;COI;;;;;
KC967243;Lepidoptera;COI;;;;;
KC967244;Lepidoptera;COI;;;;;
KC967244;Lepidoptera;COI;;;;;
KC967245;Lepidoptera;COI;;;;;
KC967245;Lepidoptera;COI;;;;;
KC967246;Lepidoptera;COI;;;;;
KC967246;Lepidoptera;COI;;;;;
KC967247;Lepidoptera;COI;;;;;
KC967247;Lepidoptera;COI;;;;;
KC967248;Lepidoptera;COI;;;;;
KC967248;Lepidoptera;COI;;;;;
KC967249;Lepidoptera;COI;;;;;
KC967249;Lepidoptera;COI;;;;;
KC967250;Lepidoptera;COI;;;;;
KC967250;Lepidoptera;COI;;;;;
KC967251;Lepidoptera;COI;;;;;
KC967251;Lepidoptera;COI;;;;;
KC967252;Lepidoptera;COI;;;;;
KC967252;Lepidoptera;COI;;;;;
KC967253;Lepidoptera;COI;;;;;
KC967253;Lepidoptera;COI;;;;;
KF444470;Lepidoptera;COI;;;;;
KF444471;Lepidoptera;COI;;;;;
KF444472;Lepidoptera;COI;;;;;
KF444473;Lepidoptera;COI;;;;;
KF647233;Lepidoptera;COI;;;;;
KF834369;Lepidoptera;COI;;;;;
KF834369;Lepidoptera;COI;;;;;
KF860859;Lepidoptera;COI;;;;;
KJ648990;Lepidoptera;COI;;;;;
KM020808;Lepidoptera;COI;;;;;
KM020810;Lepidoptera;COI;;;;;
KM020811;Lepidoptera;COI;;;;;
KM020816;Lepidoptera;COI;;;;;
KM020819;Lepidoptera;COI;;;;;
KM020821;Lepidoptera;COI;;;;;
KM020829;Lepidoptera;COI;;;;;
KM020832;Lepidoptera;COI;;;;;
KM020834;Lepidoptera;COI;;;;;
KM020839;Lepidoptera;COI;;;;;
KM020840;Lepidoptera;COI;;;;;
KM020841;Lepidoptera;COI;;;;;
KM020845;Lepidoptera;COI;;;;;
KM020848;Lepidoptera;COI;;;;;
KM020853;Lepidoptera;COI;;;;;
KM020855;Lepidoptera;COI;;;;;
KM020858;Lepidoptera;COI;;;;;
KM020860;Lepidoptera;COI;;;;;
KM020862;Lepidoptera;COI;;;;;
KM020864;Lepidoptera;COI;;;;;
KM020866;Lepidoptera;COI;;;;;
KM020871;Lepidoptera;COI;;;;;
KM020878;Lepidoptera;COI;;;;;
KM020881;Lepidoptera;COI;;;;;
KM033853;Lepidoptera;COI;;;;;
KM033854;Lepidoptera;COI;;;;;
KM033855;Lepidoptera;COI;;;;;
KM033859;Lepidoptera;COI;;;;;
KM033860;Lepidoptera;COI;;;;;
KM033861;Lepidoptera;COI;;;;;
KM033865;Lepidoptera;COI;;;;;
KM033866;Lepidoptera;COI;;;;;
KM033867;Lepidoptera;COI;;;;;
KM033868;Lepidoptera;COI;;;;;
KM033869;Lepidoptera;COI;;;;;
KM033870;Lepidoptera;COI;;;;;
KM033872;Lepidoptera;COI;;;;;
KM033873;Lepidoptera;COI;;;;;
KM033874;Lepidoptera;COI;;;;;
KM033876;Lepidoptera;COI;;;;;
KM033877;Lepidoptera;COI;;;;;
KM033878;Lepidoptera;COI;;;;;

KM033879;Lepidoptera;COI;;;;;
KM033880;Lepidoptera;COI;;;;;
KM033881;Lepidoptera;COI;;;;;
KM033883;Lepidoptera;COI;;;;;
KM033884;Lepidoptera;COI;;;;;
KM033887;Lepidoptera;COI;;;;;
KM033888;Lepidoptera;COI;;;;;
KM033889;Lepidoptera;COI;;;;;
KM033894;Lepidoptera;COI;;;;;
KM033904;Lepidoptera;COI;;;;;
KM033907;Lepidoptera;COI;;;;;
KM033908;Lepidoptera;COI;;;;;
KM033915;Lepidoptera;COI;;;;;
KM033916;Lepidoptera;COI;;;;;
KM033917;Lepidoptera;COI;;;;;
KM033918;Lepidoptera;COI;;;;;
KM033924;Lepidoptera;COI;;;;;
KM033926;Lepidoptera;COI;;;;;
KM033927;Lepidoptera;COI;;;;;
KM033940;Lepidoptera;COI;;;;;
KM196494;Lepidoptera;COI;;;;;
KM196496;Lepidoptera;COI;;;;;
KM287737;Lepidoptera;COI;;;;;
KM287739;Lepidoptera;COI;;;;;
KM287783;Lepidoptera;COI;;;;;
KM287796;Lepidoptera;COI;;;;;
KM287798;Lepidoptera;COI;;;;;
KM459414;Lepidoptera;COI;;;;;
KM517249;Lepidoptera;COI;;;;;
KM517250;Lepidoptera;COI;;;;;
KM517251;Lepidoptera;COI;;;;;
KM517252;Lepidoptera;COI;;;;;
KM517253;Lepidoptera;COI;;;;;
KM517254;Lepidoptera;COI;;;;;
KM517255;Lepidoptera;COI;;;;;
KM517256;Lepidoptera;COI;;;;;
KM517257;Lepidoptera;COI;;;;;
KM517258;Lepidoptera;COI;;;;;
KM517259;Lepidoptera;COI;;;;;
KM517260;Lepidoptera;COI;;;;;
KM517261;Lepidoptera;COI;;;;;
KM517262;Lepidoptera;COI;;;;;
KM517263;Lepidoptera;COI;;;;;
KM517264;Lepidoptera;COI;;;;;
KM517265;Lepidoptera;COI;;;;;
KM517266;Lepidoptera;COI;;;;;
KM517267;Lepidoptera;COI;;;;;
KM517268;Lepidoptera;COI;;;;;
KM517269;Lepidoptera;COI;;;;;
KM517270;Lepidoptera;COI;;;;;
KM517271;Lepidoptera;COI;;;;;
KM517272;Lepidoptera;COI;;;;;
KM517273;Lepidoptera;COI;;;;;
KM517274;Lepidoptera;COI;;;;;
KM517275;Lepidoptera;COI;;;;;
KM517276;Lepidoptera;COI;;;;;
KM517277;Lepidoptera;COI;;;;;
KM517278;Lepidoptera;COI;;;;;
KM517279;Lepidoptera;COI;;;;;
KM517280;Lepidoptera;COI;;;;;
KM517281;Lepidoptera;COI;;;;;
KM517282;Lepidoptera;COI;;;;;
KM517283;Lepidoptera;COI;;;;;
KM517284;Lepidoptera;COI;;;;;
KM517285;Lepidoptera;COI;;;;;
KM517286;Lepidoptera;COI;;;;;
KM517288;Lepidoptera;COI;;;;;
KM517290;Lepidoptera;COI;;;;;
KM517291;Lepidoptera;COI;;;;;
KM517292;Lepidoptera;COI;;;;;
KM517293;Lepidoptera;COI;;;;;
KM517294;Lepidoptera;COI;;;;;
KM517295;Lepidoptera;COI;;;;;
KM517296;Lepidoptera;COI;;;;;
KM517297;Lepidoptera;COI;;;;;
KM572201;Lepidoptera;COI;;;;;
KM572268;Lepidoptera;COI;;;;;
KM572268;Lepidoptera;COI;;;;;

KM572382;Lepidoptera;COI;;;;;
KM572538;Lepidoptera;COI;;;;;
KM572599;Lepidoptera;COI;;;;;
KM572599;Lepidoptera;COI;;;;;
KM573026;Lepidoptera;COI;;;;;
KM573117;Lepidoptera;COI;;;;;
KM972601;Lepidoptera;COI;;;;;
KM972602;Lepidoptera;COI;;;;;
KM972602;Lepidoptera;COI;;;;;
KM972605;Lepidoptera;COI;;;;;
KM972605;Lepidoptera;COI;;;;;
KM972606;Lepidoptera;COI;;;;;
KM972609;Lepidoptera;COI;;;;;
KM972609;Lepidoptera;COI;;;;;
KM972611;Lepidoptera;COI;;;;;
KM972611;Lepidoptera;COI;;;;;
KM972612;Lepidoptera;COI;;;;;
KM972612;Lepidoptera;COI;;;;;
KM972614;Lepidoptera;COI;;;;;
KM972615;Lepidoptera;COI;;;;;
KM972618;Lepidoptera;COI;;;;;
KM972619;Lepidoptera;COI;;;;;
KM972623;Lepidoptera;COI;;;;;
KM972624;Lepidoptera;COI;;;;;
KM972626;Lepidoptera;COI;;;;;
KP032241;Lepidoptera;COI;;;;;
KP032242;Lepidoptera;COI;;;;;
KP032243;Lepidoptera;COI;;;;;
KP032244;Lepidoptera;COI;;;;;
KP032245;Lepidoptera;COI;;;;;
KP032246;Lepidoptera;COI;;;;;
KP032248;Lepidoptera;COI;;;;;
KP032252;Lepidoptera;COI;;;;;
KP032261;Lepidoptera;COI;;;;;
KP032263;Lepidoptera;COI;;;;;
KP032264;Lepidoptera;COI;;;;;
KP032282;Lepidoptera;COI;;;;;
KP032287;Lepidoptera;COI;;;;;
KP032290;Lepidoptera;COI;;;;;
KP032293;Lepidoptera;COI;;;;;
KP032294;Lepidoptera;COI;;;;;
KP032296;Lepidoptera;COI;;;;;
KP032298;Lepidoptera;COI;;;;;
KP032299;Lepidoptera;COI;;;;;
KP032300;Lepidoptera;COI;;;;;
KP032320;Lepidoptera;COI;;;;;
KP032331;Lepidoptera;COI;;;;;
KP032332;Lepidoptera;COI;;;;;
KP253261;Lepidoptera;COI;;;;;
KP253274;Lepidoptera;COI;;;;;
KP253281;Lepidoptera;COI;;;;;
KP253307;Lepidoptera;COI;;;;;
KP253487;Lepidoptera;COI;;;;;
KP253731;Lepidoptera;COI;;;;;
KP870344;Lepidoptera;COI;;;;;
KP870344;Lepidoptera;COI;;;;;
KP870371;Lepidoptera;COI;;;;;
KP870385;Lepidoptera;COI;;;;;
KP870389;Lepidoptera;COI;;;;;
KP870393;Lepidoptera;COI;;;;;
KP870401;Lepidoptera;COI;;;;;
KP870409;Lepidoptera;COI;;;;;
KP870462;Lepidoptera;COI;;;;;
KP870473;Lepidoptera;COI;;;;;
KP870480;Lepidoptera;COI;;;;;
KP870482;Lepidoptera;COI;;;;;
KP870537;Lepidoptera;COI;;;;;
KP870543;Lepidoptera;COI;;;;;
KP870602;Lepidoptera;COI;;;;;
KP870697;Lepidoptera;COI;;;;;
KP870725;Lepidoptera;COI;;;;;
KP870740;Lepidoptera;COI;;;;;
KP870772;Lepidoptera;COI;;;;;
KP870817;Lepidoptera;COI;;;;;
KP870869;Lepidoptera;COI;;;;;
KP870917;Lepidoptera;COI;;;;;
KP870968;Lepidoptera;COI;;;;;
KP871017;Lepidoptera;COI;;;;;

KP871037;Lepidoptera;COI;;;;;
KP871069;Lepidoptera;COI;;;;;
KP871092;Lepidoptera;COI;;;;;
KP871092;Lepidoptera;COI;;;;;
KP871097;Lepidoptera;COI;;;;;
KR007004;Lepidoptera;COI;;;;;
KR007005;Lepidoptera;COI;;;;;
KR007006;Lepidoptera;COI;;;;;
KR007007;Lepidoptera;COI;;;;;
KR007008;Lepidoptera;COI;;;;;
KR007009;Lepidoptera;COI;;;;;
KR007010;Lepidoptera;COI;;;;;
KR007011;Lepidoptera;COI;;;;;
KR007012;Lepidoptera;COI;;;;;
KR007028;Lepidoptera;COI;;;;;
KR007029;Lepidoptera;COI;;;;;
KR007030;Lepidoptera;COI;;;;;
KR007031;Lepidoptera;COI;;;;;
KR007032;Lepidoptera;COI;;;;;
KR007033;Lepidoptera;COI;;;;;
KR007034;Lepidoptera;COI;;;;;
KR007035;Lepidoptera;COI;;;;;
KR007036;Lepidoptera;COI;;;;;
KR007037;Lepidoptera;COI;;;;;
KR007042;Lepidoptera;COI;;;;;
KR007043;Lepidoptera;COI;;;;;
KR007044;Lepidoptera;COI;;;;;
KR007045;Lepidoptera;COI;;;;;
KR007046;Lepidoptera;COI;;;;;
KR007047;Lepidoptera;COI;;;;;
KR007048;Lepidoptera;COI;;;;;
KR007049;Lepidoptera;COI;;;;;
KR007050;Lepidoptera;COI;;;;;
KR007051;Lepidoptera;COI;;;;;
KR007052;Lepidoptera;COI;;;;;
KR007053;Lepidoptera;COI;;;;;
KR007054;Lepidoptera;COI;;;;;
KR007055;Lepidoptera;COI;;;;;
KR007061;Lepidoptera;COI;;;;;
KR007062;Lepidoptera;COI;;;;;
KR007063;Lepidoptera;COI;;;;;
KR007064;Lepidoptera;COI;;;;;
KR007065;Lepidoptera;COI;;;;;
KR007069;Lepidoptera;COI;;;;;
KR007071;Lepidoptera;COI;;;;;
KR007081;Lepidoptera;COI;;;;;
KR007082;Lepidoptera;COI;;;;;
KR007083;Lepidoptera;COI;;;;;
KR007084;Lepidoptera;COI;;;;;
KR007085;Lepidoptera;COI;;;;;
KR007086;Lepidoptera;COI;;;;;
KR007087;Lepidoptera;COI;;;;;
KR007088;Lepidoptera;COI;;;;;
KR007089;Lepidoptera;COI;;;;;
KR007090;Lepidoptera;COI;;;;;
KR007091;Lepidoptera;COI;;;;;
KR007092;Lepidoptera;COI;;;;;
KR007093;Lepidoptera;COI;;;;;
KR007094;Lepidoptera;COI;;;;;
KR007095;Lepidoptera;COI;;;;;
KR007096;Lepidoptera;COI;;;;;
KR007097;Lepidoptera;COI;;;;;
KR007098;Lepidoptera;COI;;;;;
KR007099;Lepidoptera;COI;;;;;
KR007100;Lepidoptera;COI;;;;;
KR007101;Lepidoptera;COI;;;;;
KR007102;Lepidoptera;COI;;;;;
KR007103;Lepidoptera;COI;;;;;
KR007104;Lepidoptera;COI;;;;;
KR007105;Lepidoptera;COI;;;;;
KR007106;Lepidoptera;COI;;;;;
KR007107;Lepidoptera;COI;;;;;
KR007108;Lepidoptera;COI;;;;;
KR007109;Lepidoptera;COI;;;;;
KR007110;Lepidoptera;COI;;;;;
KR007111;Lepidoptera;COI;;;;;
KR007112;Lepidoptera;COI;;;;;
KR007113;Lepidoptera;COI;;;;;

KR007114;Lepidoptera;COI;;;;;
KR007115;Lepidoptera;COI;;;;;
KR007116;Lepidoptera;COI;;;;;
KR007117;Lepidoptera;COI;;;;;
KR007118;Lepidoptera;COI;;;;;
KR007119;Lepidoptera;COI;;;;;
KR007120;Lepidoptera;COI;;;;;
KR007121;Lepidoptera;COI;;;;;
KR007122;Lepidoptera;COI;;;;;
KR007123;Lepidoptera;COI;;;;;
KR007124;Lepidoptera;COI;;;;;
KR007125;Lepidoptera;COI;;;;;
KR007126;Lepidoptera;COI;;;;;
KR007127;Lepidoptera;COI;;;;;
KR007128;Lepidoptera;COI;;;;;
KR007129;Lepidoptera;COI;;;;;
KR007130;Lepidoptera;COI;;;;;
KR007131;Lepidoptera;COI;;;;;
KR007132;Lepidoptera;COI;;;;;
KR007133;Lepidoptera;COI;;;;;
KR007134;Lepidoptera;COI;;;;;
KR007135;Lepidoptera;COI;;;;;
KR007136;Lepidoptera;COI;;;;;
KR007137;Lepidoptera;COI;;;;;
KR007138;Lepidoptera;COI;;;;;
KR007139;Lepidoptera;COI;;;;;
KR007140;Lepidoptera;COI;;;;;
KR007141;Lepidoptera;COI;;;;;
KR007142;Lepidoptera;COI;;;;;
KR007143;Lepidoptera;COI;;;;;
KR007144;Lepidoptera;COI;;;;;
KR007145;Lepidoptera;COI;;;;;
KR007146;Lepidoptera;COI;;;;;
KR007147;Lepidoptera;COI;;;;;
KR007148;Lepidoptera;COI;;;;;
KR007149;Lepidoptera;COI;;;;;
KR007155;Lepidoptera;COI;;;;;
KT230684;Lepidoptera;COI;;;;;
KT782581;Lepidoptera;COI;;;;;
KT782663;Lepidoptera;COI;;;;;
KT792888;Lepidoptera;COI;;;;;
KT792889;Lepidoptera;COI;;;;;
KT792890;Lepidoptera;COI;;;;;
KT792891;Lepidoptera;COI;;;;;
KT792894;Lepidoptera;COI;;;;;
KT792906;Lepidoptera;COI;;;;;
KT907184;Lepidoptera;COI;;;;;
KT907185;Lepidoptera;COI;;;;;
KT989875;Lepidoptera;COI;;;;;
KU577157;Lepidoptera;COI;;;;;
KU577158;Lepidoptera;COI;;;;;
KU577159;Lepidoptera;COI;;;;;
KU577160;Lepidoptera;COI;;;;;
KU577161;Lepidoptera;COI;;;;;
KU577162;Lepidoptera;COI;;;;;
KU577163;Lepidoptera;COI;;;;;
KU577164;Lepidoptera;COI;;;;;
KU577165;Lepidoptera;COI;;;;;
KU577166;Lepidoptera;COI;;;;;
KU577167;Lepidoptera;COI;;;;;
KU577168;Lepidoptera;COI;;;;;
KU577169;Lepidoptera;COI;;;;;
KU577170;Lepidoptera;COI;;;;;
KU577171;Lepidoptera;COI;;;;;
KU577172;Lepidoptera;COI;;;;;
KU577173;Lepidoptera;COI;;;;;
KU577174;Lepidoptera;COI;;;;;
KU577175;Lepidoptera;COI;;;;;
KU577176;Lepidoptera;COI;;;;;
KU577177;Lepidoptera;COI;;;;;
KU577178;Lepidoptera;COI;;;;;
KU577179;Lepidoptera;COI;;;;;
KU577180;Lepidoptera;COI;;;;;
KU577181;Lepidoptera;COI;;;;;
KU577182;Lepidoptera;COI;;;;;
KU577183;Lepidoptera;COI;;;;;
KU577184;Lepidoptera;COI;;;;;
KU577185;Lepidoptera;COI;;;;;

KU577186;Lepidoptera;COI;;;;;
KU577187;Lepidoptera;COI;;;;;
KU577188;Lepidoptera;COI;;;;;
KU577189;Lepidoptera;COI;;;;;
KU577190;Lepidoptera;COI;;;;;
KU577191;Lepidoptera;COI;;;;;
KU577192;Lepidoptera;COI;;;;;
KU577193;Lepidoptera;COI;;;;;
KU973553;Lepidoptera;COI;;;;;
KX011033;Lepidoptera;COI;;;;;
KX039986;Lepidoptera;COI;;;;;
KX039986;Lepidoptera;COI;;;;;
KX040121;Lepidoptera;COI;;;;;
KX040121;Lepidoptera;COI;;;;;
KX040210;Lepidoptera;COI;;;;;
KX040221;Lepidoptera;COI;;;;;
KX040221;Lepidoptera;COI;;;;;
KX040261;Lepidoptera;COI;;;;;
KX040261;Lepidoptera;COI;;;;;
KX040279;Lepidoptera;COI;;;;;
KX040299;Lepidoptera;COI;;;;;
KX040299;Lepidoptera;COI;;;;;
KX040356;Lepidoptera;COI;;;;;
KX040356;Lepidoptera;COI;;;;;
KX040519;Lepidoptera;COI;;;;;
KX040560;Lepidoptera;COI;;;;;
KX040560;Lepidoptera;COI;;;;;
KX040614;Lepidoptera;COI;;;;;
KX040614;Lepidoptera;COI;;;;;
KX040643;Lepidoptera;COI;;;;;
KX040643;Lepidoptera;COI;;;;;
KX040676;Lepidoptera;COI;;;;;
KX040676;Lepidoptera;COI;;;;;
KX040863;Lepidoptera;COI;;;;;
KX040863;Lepidoptera;COI;;;;;
KX040949;Lepidoptera;COI;;;;;
KX040949;Lepidoptera;COI;;;;;
KX041071;Lepidoptera;COI;;;;;
KX041493;Lepidoptera;COI;;;;;
KX041680;Lepidoptera;COI;;;;;
KX041760;Lepidoptera;COI;;;;;
KX041972;Lepidoptera;COI;;;;;
KX041987;Lepidoptera;COI;;;;;
KX044305;Lepidoptera;COI;;;;;
KX044637;Lepidoptera;COI;;;;;
KX044637;Lepidoptera;COI;;;;;
KX044929;Lepidoptera;COI;;;;;
KX044929;Lepidoptera;COI;;;;;
KX045217;Lepidoptera;COI;;;;;
KX045359;Lepidoptera;COI;;;;;
KX045359;Lepidoptera;COI;;;;;
KX045415;Lepidoptera;COI;;;;;
KX045511;Lepidoptera;COI;;;;;
KX045511;Lepidoptera;COI;;;;;
KX045576;Lepidoptera;COI;;;;;
KX045576;Lepidoptera;COI;;;;;
KX046192;Lepidoptera;COI;;;;;
KX046192;Lepidoptera;COI;;;;;
KX046221;Lepidoptera;COI;;;;;
KX046261;Lepidoptera;COI;;;;;
KX047625;Lepidoptera;COI;;;;;
KX047625;Lepidoptera;COI;;;;;
KX047754;Lepidoptera;COI;;;;;
KX048711;Lepidoptera;COI;;;;;
KX048787;Lepidoptera;COI;;;;;
KX049168;Lepidoptera;COI;;;;;
KX049777;Lepidoptera;COI;;;;;
KX049826;Lepidoptera;COI;;;;;
KX049826;Lepidoptera;COI;;;;;
KX051495;Lepidoptera;COI;;;;;
KX071243;Lepidoptera;COI;;;;;
KX071773;Lepidoptera;COI;;;;;
KX130672;Lepidoptera;COI;;;;;
KX130676;Lepidoptera;COI;;;;;
KX130677;Lepidoptera;COI;;;;;
KX130678;Lepidoptera;COI;;;;;
KX130679;Lepidoptera;COI;;;;;
KX130680;Lepidoptera;COI;;;;;

KX130681;Lepidoptera;COI;;;;;
KX130687;Lepidoptera;COI;;;;;
KX130688;Lepidoptera;COI;;;;;
KX130689;Lepidoptera;COI;;;;;
KX130690;Lepidoptera;COI;;;;;
KY112799;Lepidoptera;COI;;;;;
KY112800;Lepidoptera;COI;;;;;
KY112801;Lepidoptera;COI;;;;;
KY112802;Lepidoptera;COI;;;;;
KY112803;Lepidoptera;COI;;;;;
KY112804;Lepidoptera;COI;;;;;
KY112805;Lepidoptera;COI;;;;;
KY112806;Lepidoptera;COI;;;;;
KY112807;Lepidoptera;COI;;;;;
KY112808;Lepidoptera;COI;;;;;
KY112809;Lepidoptera;COI;;;;;
KY112810;Lepidoptera;COI;;;;;
KY112811;Lepidoptera;COI;;;;;
KY112812;Lepidoptera;COI;;;;;
KY112813;Lepidoptera;COI;;;;;
KY112814;Lepidoptera;COI;;;;;
KY112815;Lepidoptera;COI;;;;;
KY112816;Lepidoptera;COI;;;;;
KY112817;Lepidoptera;COI;;;;;
KY112818;Lepidoptera;COI;;;;;
KY112819;Lepidoptera;COI;;;;;
KY112820;Lepidoptera;COI;;;;;
KY112821;Lepidoptera;COI;;;;;
KY112822;Lepidoptera;COI;;;;;
KY112823;Lepidoptera;COI;;;;;
KY112825;Lepidoptera;COI;;;;;
KY112826;Lepidoptera;COI;;;;;
KY112827;Lepidoptera;COI;;;;;
KY112828;Lepidoptera;COI;;;;;
KY112829;Lepidoptera;COI;;;;;
KY112830;Lepidoptera;COI;;;;;
KY112831;Lepidoptera;COI;;;;;
KY112832;Lepidoptera;COI;;;;;
KY112833;Lepidoptera;COI;;;;;
KY112834;Lepidoptera;COI;;;;;
KY112835;Lepidoptera;COI;;;;;
KY112836;Lepidoptera;COI;;;;;
KY112837;Lepidoptera;COI;;;;;
KY112838;Lepidoptera;COI;;;;;
KY112839;Lepidoptera;COI;;;;;
KY112840;Lepidoptera;COI;;;;;
KY112841;Lepidoptera;COI;;;;;
KY112842;Lepidoptera;COI;;;;;
KY112843;Lepidoptera;COI;;;;;
KY112844;Lepidoptera;COI;;;;;
KY112845;Lepidoptera;COI;;;;;
KY112846;Lepidoptera;COI;;;;;
KY112847;Lepidoptera;COI;;;;;
KY112848;Lepidoptera;COI;;;;;
KY112849;Lepidoptera;COI;;;;;
KY112850;Lepidoptera;COI;;;;;
KY112852;Lepidoptera;COI;;;;;
KY112853;Lepidoptera;COI;;;;;
KY128337;Lepidoptera;COI;;;;;
KY128362;Lepidoptera;COI;;;;;
KY128366;Lepidoptera;COI;;;;;
KY128368;Lepidoptera;COI;;;;;
KY128376;Lepidoptera;COI;;;;;
LC155510;Lepidoptera;COI;;;;;
LC340452;Lepidoptera;COI;;;;;
LC340458;Lepidoptera;COI;;;;;
LC340462;Lepidoptera;COI;;;;;
LC340480;Lepidoptera;COI;;;;;
LC340481;Lepidoptera;COI;;;;;
LC340496;Lepidoptera;COI;;;;;
LC340566;Lepidoptera;COI;;;;;
LC340579;Lepidoptera;COI;;;;;
LC340580;Lepidoptera;COI;;;;;
LC340581;Lepidoptera;COI;;;;;
LC340582;Lepidoptera;COI;;;;;
LC340583;Lepidoptera;COI;;;;;
LC340584;Lepidoptera;COI;;;;;
LC340585;Lepidoptera;COI;;;;;

LC340586;Lepidoptera;COI;;;;;
LC340587;Lepidoptera;COI;;;;;
LC340588;Lepidoptera;COI;;;;;
LC340589;Lepidoptera;COI;;;;;
LC340590;Lepidoptera;COI;;;;;
LC340591;Lepidoptera;COI;;;;;
LC340592;Lepidoptera;COI;;;;;
LC340593;Lepidoptera;COI;;;;;
LC340604;Lepidoptera;COI;;;;;
LC340608;Lepidoptera;COI;;;;;
LC340610;Lepidoptera;COI;;;;;
LC340611;Lepidoptera;COI;;;;;
LC340614;Lepidoptera;COI;;;;;
LC340615;Lepidoptera;COI;;;;;
LC340626;Lepidoptera;COI;;;;;
LC340627;Lepidoptera;COI;;;;;
LC340628;Lepidoptera;COI;;;;;
LC340656;Lepidoptera;COI;;;;;
LC340665;Lepidoptera;COI;;;;;
LC340682;Lepidoptera;COI;;;;;
LC340684;Lepidoptera;COI;;;;;
LC340689;Lepidoptera;COI;;;;;
LC340694;Lepidoptera;COI;;;;;
LC340695;Lepidoptera;COI;;;;;
LC340696;Lepidoptera;COI;;;;;
LC340697;Lepidoptera;COI;;;;;
LC471689;Lepidoptera;COI;;;;;
LC471690;Lepidoptera;COI;;;;;
LC471692;Lepidoptera;COI;;;;;
LC471693;Lepidoptera;COI;;;;;
LC471702;Lepidoptera;COI;;;;;
LC471703;Lepidoptera;COI;;;;;
LC471710;Lepidoptera;COI;;;;;
LC471711;Lepidoptera;COI;;;;;
LC471714;Lepidoptera;COI;;;;;
LT219936;Lepidoptera;COI;;;;;
LT219936;Lepidoptera;COI;;;;;
LT219937;Lepidoptera;COI;;;;;
LT219937;Lepidoptera;COI;;;;;
LT219938;Lepidoptera;COI;;;;;
LT219938;Lepidoptera;COI;;;;;
LT219939;Lepidoptera;COI;;;;;
LT219939;Lepidoptera;COI;;;;;
LT219940;Lepidoptera;COI;;;;;
LT219940;Lepidoptera;COI;;;;;
LT219941;Lepidoptera;COI;;;;;
LT219941;Lepidoptera;COI;;;;;
LT219942;Lepidoptera;COI;;;;;
LT219942;Lepidoptera;COI;;;;;
LT219943;Lepidoptera;COI;;;;;
LT219943;Lepidoptera;COI;;;;;
LT219944;Lepidoptera;COI;;;;;
LT219944;Lepidoptera;COI;;;;;
LT219945;Lepidoptera;COI;;;;;
LT219945;Lepidoptera;COI;;;;;
LT219946;Lepidoptera;COI;;;;;
LT219946;Lepidoptera;COI;;;;;
LT219947;Lepidoptera;COI;;;;;
LT219947;Lepidoptera;COI;;;;;
LT219948;Lepidoptera;COI;;;;;
LT219948;Lepidoptera;COI;;;;;
LT219949;Lepidoptera;COI;;;;;
LT219949;Lepidoptera;COI;;;;;
LT219950;Lepidoptera;COI;;;;;
LT219950;Lepidoptera;COI;;;;;
LT219951;Lepidoptera;COI;;;;;
LT219951;Lepidoptera;COI;;;;;
LT219952;Lepidoptera;COI;;;;;
LT219952;Lepidoptera;COI;;;;;
LT219953;Lepidoptera;COI;;;;;
LT219953;Lepidoptera;COI;;;;;
LT219954;Lepidoptera;COI;;;;;
LT219954;Lepidoptera;COI;;;;;
LT219955;Lepidoptera;COI;;;;;
LT219955;Lepidoptera;COI;;;;;
LT219956;Lepidoptera;COI;;;;;
LT219956;Lepidoptera;COI;;;;;
LT219957;Lepidoptera;COI;;;;;

LT219957;Lepidoptera;COI;;;;;
LT219958;Lepidoptera;COI;;;;;
LT219958;Lepidoptera;COI;;;;;
LT219959;Lepidoptera;COI;;;;;
LT219959;Lepidoptera;COI;;;;;
LT219960;Lepidoptera;COI;;;;;
LT219960;Lepidoptera;COI;;;;;
LT219961;Lepidoptera;COI;;;;;
LT219961;Lepidoptera;COI;;;;;
LT219962;Lepidoptera;COI;;;;;
LT219962;Lepidoptera;COI;;;;;
LT219963;Lepidoptera;COI;;;;;
LT219963;Lepidoptera;COI;;;;;
LT219964;Lepidoptera;COI;;;;;
LT219964;Lepidoptera;COI;;;;;
LT219965;Lepidoptera;COI;;;;;
LT219965;Lepidoptera;COI;;;;;
LT219966;Lepidoptera;COI;;;;;
LT219966;Lepidoptera;COI;;;;;
LT219967;Lepidoptera;COI;;;;;
LT219967;Lepidoptera;COI;;;;;
LT219968;Lepidoptera;COI;;;;;
LT219968;Lepidoptera;COI;;;;;
LT219969;Lepidoptera;COI;;;;;
LT219969;Lepidoptera;COI;;;;;
LT219970;Lepidoptera;COI;;;;;
LT219970;Lepidoptera;COI;;;;;
LT219971;Lepidoptera;COI;;;;;
LT219971;Lepidoptera;COI;;;;;
LT219972;Lepidoptera;COI;;;;;
LT219972;Lepidoptera;COI;;;;;
LT219973;Lepidoptera;COI;;;;;
LT219973;Lepidoptera;COI;;;;;
LT219974;Lepidoptera;COI;;;;;
LT219974;Lepidoptera;COI;;;;;
LT219975;Lepidoptera;COI;;;;;
LT219975;Lepidoptera;COI;;;;;
LT219976;Lepidoptera;COI;;;;;
LT219976;Lepidoptera;COI;;;;;
LT219977;Lepidoptera;COI;;;;;
LT219977;Lepidoptera;COI;;;;;
LT219978;Lepidoptera;COI;;;;;
LT219978;Lepidoptera;COI;;;;;
LT219979;Lepidoptera;COI;;;;;
LT219979;Lepidoptera;COI;;;;;
LT219980;Lepidoptera;COI;;;;;
LT219980;Lepidoptera;COI;;;;;
LT219981;Lepidoptera;COI;;;;;
LT219981;Lepidoptera;COI;;;;;
LT219982;Lepidoptera;COI;;;;;
LT219982;Lepidoptera;COI;;;;;
LT219983;Lepidoptera;COI;;;;;
LT219983;Lepidoptera;COI;;;;;
LT219984;Lepidoptera;COI;;;;;
LT219984;Lepidoptera;COI;;;;;
LT219985;Lepidoptera;COI;;;;;
LT219985;Lepidoptera;COI;;;;;
LT219986;Lepidoptera;COI;;;;;
LT219986;Lepidoptera;COI;;;;;
LT219987;Lepidoptera;COI;;;;;
LT219987;Lepidoptera;COI;;;;;
LT219988;Lepidoptera;COI;;;;;
LT219988;Lepidoptera;COI;;;;;
LT219989;Lepidoptera;COI;;;;;
LT219989;Lepidoptera;COI;;;;;
LT219990;Lepidoptera;COI;;;;;
LT219990;Lepidoptera;COI;;;;;
LT219991;Lepidoptera;COI;;;;;
LT219991;Lepidoptera;COI;;;;;
LT219992;Lepidoptera;COI;;;;;
LT219992;Lepidoptera;COI;;;;;
LT219993;Lepidoptera;COI;;;;;
LT219993;Lepidoptera;COI;;;;;
LT219994;Lepidoptera;COI;;;;;
LT219994;Lepidoptera;COI;;;;;
LT219995;Lepidoptera;COI;;;;;
LT219995;Lepidoptera;COI;;;;;
LT219996;Lepidoptera;COI;;;;;

LT219996;Lepidoptera;COI;;;;;
LT219997;Lepidoptera;COI;;;;;
LT219997;Lepidoptera;COI;;;;;
LT219998;Lepidoptera;COI;;;;;
LT219998;Lepidoptera;COI;;;;;
LT219999;Lepidoptera;COI;;;;;
LT219999;Lepidoptera;COI;;;;;
LT220000;Lepidoptera;COI;;;;;
LT220000;Lepidoptera;COI;;;;;
LT220001;Lepidoptera;COI;;;;;
LT220001;Lepidoptera;COI;;;;;
LT220002;Lepidoptera;COI;;;;;
LT220002;Lepidoptera;COI;;;;;
LT220003;Lepidoptera;COI;;;;;
LT220003;Lepidoptera;COI;;;;;
LT220004;Lepidoptera;COI;;;;;
LT220004;Lepidoptera;COI;;;;;
LT220005;Lepidoptera;COI;;;;;
LT220005;Lepidoptera;COI;;;;;
LT220006;Lepidoptera;COI;;;;;
LT220006;Lepidoptera;COI;;;;;
LT220007;Lepidoptera;COI;;;;;
LT220007;Lepidoptera;COI;;;;;
LT220008;Lepidoptera;COI;;;;;
LT220008;Lepidoptera;COI;;;;;
LT220009;Lepidoptera;COI;;;;;
LT220009;Lepidoptera;COI;;;;;
LT220010;Lepidoptera;COI;;;;;
LT220010;Lepidoptera;COI;;;;;
LT220011;Lepidoptera;COI;;;;;
LT220011;Lepidoptera;COI;;;;;
LT220012;Lepidoptera;COI;;;;;
LT220012;Lepidoptera;COI;;;;;
LT220013;Lepidoptera;COI;;;;;
LT220013;Lepidoptera;COI;;;;;
LT220014;Lepidoptera;COI;;;;;
LT220014;Lepidoptera;COI;;;;;
LT220015;Lepidoptera;COI;;;;;
LT220015;Lepidoptera;COI;;;;;
LT220016;Lepidoptera;COI;;;;;
LT220016;Lepidoptera;COI;;;;;
LT220017;Lepidoptera;COI;;;;;
LT220017;Lepidoptera;COI;;;;;
LT628962;Lepidoptera;COI;;;;;
LT628986;Lepidoptera;COI;;;;;
LT628987;Lepidoptera;COI;;;;;
LT628987;Lepidoptera;COI;;;;;
LT628995;Lepidoptera;COI;;;;;
LT628996;Lepidoptera;COI;;;;;
LT628997;Lepidoptera;COI;;;;;
LT986366;Lepidoptera;COI;;;;;
LT986369;Lepidoptera;COI;;;;;
MF141940;Lepidoptera;COI;;;;;
MG779475;Lepidoptera;COI;;;;;
MH006697;Lepidoptera;COI;;;;;
MH235073;Lepidoptera;COI;;;;;
MH235074;Lepidoptera;COI;;;;;
MH235075;Lepidoptera;COI;;;;;
MH235076;Lepidoptera;COI;;;;;
MH235078;Lepidoptera;COI;;;;;
MH235081;Lepidoptera;COI;;;;;
MH235086;Lepidoptera;COI;;;;;
MH235092;Lepidoptera;COI;;;;;
MH235095;Lepidoptera;COI;;;;;
MH235099;Lepidoptera;COI;;;;;
MH235100;Lepidoptera;COI;;;;;
MH235108;Lepidoptera;COI;;;;;
MH235109;Lepidoptera;COI;;;;;
MH235110;Lepidoptera;COI;;;;;
MH235113;Lepidoptera;COI;;;;;
MH235115;Lepidoptera;COI;;;;;
MH235118;Lepidoptera;COI;;;;;
MH235120;Lepidoptera;COI;;;;;
MH235121;Lepidoptera;COI;;;;;
MH235122;Lepidoptera;COI;;;;;
MH235125;Lepidoptera;COI;;;;;
MH235127;Lepidoptera;COI;;;;;
MH235129;Lepidoptera;COI;;;;;

MH235131;Lepidoptera;COI;;;;;
MH235132;Lepidoptera;COI;;;;;
MH235133;Lepidoptera;COI;;;;;
MH235137;Lepidoptera;COI;;;;;
MH235138;Lepidoptera;COI;;;;;
MH235141;Lepidoptera;COI;;;;;
MH235145;Lepidoptera;COI;;;;;
MH235146;Lepidoptera;COI;;;;;
MH235149;Lepidoptera;COI;;;;;
MH235151;Lepidoptera;COI;;;;;
MH235152;Lepidoptera;COI;;;;;
MH235153;Lepidoptera;COI;;;;;
MH235157;Lepidoptera;COI;;;;;
MH235159;Lepidoptera;COI;;;;;
MH235160;Lepidoptera;COI;;;;;
MH235162;Lepidoptera;COI;;;;;
MH418510;Lepidoptera;COI;;;;;
MH418525;Lepidoptera;COI;;;;;
MH418532;Lepidoptera;COI;;;;;
MH418535;Lepidoptera;COI;;;;;
MH418543;Lepidoptera;COI;;;;;
MH418544;Lepidoptera;COI;;;;;
MH418568;Lepidoptera;COI;;;;;
MH418667;Lepidoptera;COI;;;;;
MH418682;Lepidoptera;COI;;;;;
MH418693;Lepidoptera;COI;;;;;
MH418694;Lepidoptera;COI;;;;;
MH418704;Lepidoptera;COI;;;;;
MH418709;Lepidoptera;COI;;;;;
MH418716;Lepidoptera;COI;;;;;
MH418719;Lepidoptera;COI;;;;;
MH418723;Lepidoptera;COI;;;;;
MH418754;Lepidoptera;COI;;;;;
MH418800;Lepidoptera;COI;;;;;
MH418859;Lepidoptera;COI;;;;;
MH418883;Lepidoptera;COI;;;;;
MH418949;Lepidoptera;COI;;;;;
MH418990;Lepidoptera;COI;;;;;
MH419013;Lepidoptera;COI;;;;;
MH419020;Lepidoptera;COI;;;;;
MH419033;Lepidoptera;COI;;;;;
MH419045;Lepidoptera;COI;;;;;
MH419054;Lepidoptera;COI;;;;;
MH419091;Lepidoptera;COI;;;;;
MH419105;Lepidoptera;COI;;;;;
MH419109;Lepidoptera;COI;;;;;
MH419171;Lepidoptera;COI;;;;;
MH419180;Lepidoptera;COI;;;;;
MH419196;Lepidoptera;COI;;;;;
MH419235;Lepidoptera;COI;;;;;
MH419236;Lepidoptera;COI;;;;;
MH419241;Lepidoptera;COI;;;;;
MH419250;Lepidoptera;COI;;;;;
MH419259;Lepidoptera;COI;;;;;
MH419263;Lepidoptera;COI;;;;;
MH419294;Lepidoptera;COI;;;;;
MH419296;Lepidoptera;COI;;;;;
MH419296;Lepidoptera;COI;;;;;
MH419302;Lepidoptera;COI;;;;;
MH419304;Lepidoptera;COI;;;;;
MH419306;Lepidoptera;COI;;;;;
MH419350;Lepidoptera;COI;;;;;
MH419382;Lepidoptera;COI;;;;;
MH419469;Lepidoptera;COI;;;;;
MH419488;Lepidoptera;COI;;;;;
MH419511;Lepidoptera;COI;;;;;
MH419512;Lepidoptera;COI;;;;;
MH419528;Lepidoptera;COI;;;;;
MH419561;Lepidoptera;COI;;;;;
MH419567;Lepidoptera;COI;;;;;
MH419584;Lepidoptera;COI;;;;;
MH419613;Lepidoptera;COI;;;;;
MH419651;Lepidoptera;COI;;;;;
MH419659;Lepidoptera;COI;;;;;
MH419754;Lepidoptera;COI;;;;;
MH419780;Lepidoptera;COI;;;;;
MH419793;Lepidoptera;COI;;;;;
MH419808;Lepidoptera;COI;;;;;

MH419811;Lepidoptera;COI;;;;;
MH419820;Lepidoptera;COI;;;;;
MH419845;Lepidoptera;COI;;;;;
MH419857;Lepidoptera;COI;;;;;
MH419857;Lepidoptera;COI;;;;;
MH419870;Lepidoptera;COI;;;;;
MH419889;Lepidoptera;COI;;;;;
MH419898;Lepidoptera;COI;;;;;
MH419906;Lepidoptera;COI;;;;;
MH419917;Lepidoptera;COI;;;;;
MH419973;Lepidoptera;COI;;;;;
MH420060;Lepidoptera;COI;;;;;
MH420107;Lepidoptera;COI;;;;;
MH420124;Lepidoptera;COI;;;;;
MH420126;Lepidoptera;COI;;;;;
MH420130;Lepidoptera;COI;;;;;
MH420185;Lepidoptera;COI;;;;;
MH420219;Lepidoptera;COI;;;;;
MH420220;Lepidoptera;COI;;;;;
MH420225;Lepidoptera;COI;;;;;
MH420248;Lepidoptera;COI;;;;;
MH420263;Lepidoptera;COI;;;;;
MH420300;Lepidoptera;COI;;;;;
MH420332;Lepidoptera;COI;;;;;
MH420340;Lepidoptera;COI;;;;;
MH420378;Lepidoptera;COI;;;;;
MH420415;Lepidoptera;COI;;;;;
MH670125;Lepidoptera;COI;;;;;
MH670137;Lepidoptera;COI;;;;;
MH670219;Lepidoptera;COI;;;;;
MK186158;Lepidoptera;COI;;;;;
MK186214;Lepidoptera;COI;;;;;
MK186215;Lepidoptera;COI;;;;;
MK186216;Lepidoptera;COI;;;;;
MK186217;Lepidoptera;COI;;;;;
MK186218;Lepidoptera;COI;;;;;
MK186219;Lepidoptera;COI;;;;;
MK186220;Lepidoptera;COI;;;;;
MK186221;Lepidoptera;COI;;;;;
MK186222;Lepidoptera;COI;;;;;
MK186224;Lepidoptera;COI;;;;;
MK186225;Lepidoptera;COI;;;;;
MK186226;Lepidoptera;COI;;;;;
MK186227;Lepidoptera;COI;;;;;
MK186228;Lepidoptera;COI;;;;;
MK186246;Lepidoptera;COI;;;;;
MK186247;Lepidoptera;COI;;;;;
MK186248;Lepidoptera;COI;;;;;
MK186249;Lepidoptera;COI;;;;;
MK186250;Lepidoptera;COI;;;;;
MK186251;Lepidoptera;COI;;;;;
MK186252;Lepidoptera;COI;;;;;
MK186259;Lepidoptera;COI;;;;;
MK186260;Lepidoptera;COI;;;;;
MK186267;Lepidoptera;COI;;;;;
MK186268;Lepidoptera;COI;;;;;
MK186288;Lepidoptera;COI;;;;;
MK186289;Lepidoptera;COI;;;;;
MK186290;Lepidoptera;COI;;;;;
MK186291;Lepidoptera;COI;;;;;
MK186292;Lepidoptera;COI;;;;;
MK186293;Lepidoptera;COI;;;;;
MK186317;Lepidoptera;COI;;;;;
MK186318;Lepidoptera;COI;;;;;
MK186320;Lepidoptera;COI;;;;;
MK186347;Lepidoptera;COI;;;;;
MK186369;Lepidoptera;COI;;;;;
MK186370;Lepidoptera;COI;;;;;
MK186371;Lepidoptera;COI;;;;;
MK186373;Lepidoptera;COI;;;;;
MK186374;Lepidoptera;COI;;;;;
MK186375;Lepidoptera;COI;;;;;
MK186376;Lepidoptera;COI;;;;;
MK186377;Lepidoptera;COI;;;;;
MK186398;Lepidoptera;COI;;;;;
MK186399;Lepidoptera;COI;;;;;
MK186400;Lepidoptera;COI;;;;;
MK186405;Lepidoptera;COI;;;;;

MK186413;Lepidoptera;COI;;;;;
MK186414;Lepidoptera;COI;;;;;
MK186415;Lepidoptera;COI;;;;;
MK186527;Lepidoptera;COI;;;;;
MK186529;Lepidoptera;COI;;;;;
MK186530;Lepidoptera;COI;;;;;
MK186531;Lepidoptera;COI;;;;;
MK186532;Lepidoptera;COI;;;;;
MK186533;Lepidoptera;COI;;;;;
MK186534;Lepidoptera;COI;;;;;
MK186535;Lepidoptera;COI;;;;;
MK186562;Lepidoptera;COI;;;;;
MK186565;Lepidoptera;COI;;;;;
MK186568;Lepidoptera;COI;;;;;
MK186570;Lepidoptera;COI;;;;;
MK186645;Lepidoptera;COI;;;;;
MK186646;Lepidoptera;COI;;;;;
MK186647;Lepidoptera;COI;;;;;
MK186648;Lepidoptera;COI;;;;;
MK186671;Lepidoptera;COI;;;;;
MK186673;Lepidoptera;COI;;;;;
MK186674;Lepidoptera;COI;;;;;
MK186674;Lepidoptera;COI;;;;;
MK186675;Lepidoptera;COI;;;;;
MK186675;Lepidoptera;COI;;;;;
MK186676;Lepidoptera;COI;;;;;
MK186676;Lepidoptera;COI;;;;;
MK186677;Lepidoptera;COI;;;;;
MK186677;Lepidoptera;COI;;;;;
MK186678;Lepidoptera;COI;;;;;
MK186678;Lepidoptera;COI;;;;;
MK186679;Lepidoptera;COI;;;;;
MK186679;Lepidoptera;COI;;;;;
MK186680;Lepidoptera;COI;;;;;
MK186681;Lepidoptera;COI;;;;;
MK186683;Lepidoptera;COI;;;;;
MK186684;Lepidoptera;COI;;;;;
MK186717;Lepidoptera;COI;;;;;
MK186718;Lepidoptera;COI;;;;;
MK186719;Lepidoptera;COI;;;;;
MK186720;Lepidoptera;COI;;;;;
MK186720;Lepidoptera;COI;;;;;
MK186721;Lepidoptera;COI;;;;;
MK186721;Lepidoptera;COI;;;;;
MK186722;Lepidoptera;COI;;;;;
MK186722;Lepidoptera;COI;;;;;
MK186723;Lepidoptera;COI;;;;;
MK186723;Lepidoptera;COI;;;;;
MK186758;Lepidoptera;COI;;;;;
MK186759;Lepidoptera;COI;;;;;
MK186760;Lepidoptera;COI;;;;;
MK186777;Lepidoptera;COI;;;;;
MK186782;Lepidoptera;COI;;;;;
MK186784;Lepidoptera;COI;;;;;
MK541994;Lepidoptera;COI;;;;;
MK567858;Lepidoptera;COI;;;;;
MK567859;Lepidoptera;COI;;;;;
MK567860;Lepidoptera;COI;;;;;
MN138473;Lepidoptera;COI;;;;;
MN138473;Lepidoptera;COI;;;;;
MN138500;Lepidoptera;COI;;;;;
MN138514;Lepidoptera;COI;;;;;
MN138518;Lepidoptera;COI;;;;;
MN138524;Lepidoptera;COI;;;;;
MN138542;Lepidoptera;COI;;;;;
MN138543;Lepidoptera;COI;;;;;
MN138574;Lepidoptera;COI;;;;;
MN138608;Lepidoptera;COI;;;;;
MN138616;Lepidoptera;COI;;;;;
MN138616;Lepidoptera;COI;;;;;
MN138663;Lepidoptera;COI;;;;;
MN138664;Lepidoptera;COI;;;;;
MN138669;Lepidoptera;COI;;;;;
MN138678;Lepidoptera;COI;;;;;
MN138681;Lepidoptera;COI;;;;;
MN138687;Lepidoptera;COI;;;;;
MN138720;Lepidoptera;COI;;;;;
MN138733;Lepidoptera;COI;;;;;

MN138733;Lepidoptera;COI;;;;;
MN138734;Lepidoptera;COI;;;;;
MN138742;Lepidoptera;COI;;;;;
MN138746;Lepidoptera;COI;;;;;
MN138759;Lepidoptera;COI;;;;;
MN138764;Lepidoptera;COI;;;;;
MN138771;Lepidoptera;COI;;;;;
MN138772;Lepidoptera;COI;;;;;
MN138774;Lepidoptera;COI;;;;;
MN138800;Lepidoptera;COI;;;;;
MN138809;Lepidoptera;COI;;;;;
MN138812;Lepidoptera;COI;;;;;
MN138812;Lepidoptera;COI;;;;;
MN138814;Lepidoptera;COI;;;;;
MN138841;Lepidoptera;COI;;;;;
MN138856;Lepidoptera;COI;;;;;
MN138875;Lepidoptera;COI;;;;;
MN138879;Lepidoptera;COI;;;;;
MN138887;Lepidoptera;COI;;;;;
MN138889;Lepidoptera;COI;;;;;
MN138894;Lepidoptera;COI;;;;;
MN138921;Lepidoptera;COI;;;;;
MN138944;Lepidoptera;COI;;;;;
MN138947;Lepidoptera;COI;;;;;
MN138970;Lepidoptera;COI;;;;;
MN138972;Lepidoptera;COI;;;;;
MN138974;Lepidoptera;COI;;;;;
MN138979;Lepidoptera;COI;;;;;
MN138993;Lepidoptera;COI;;;;;
MN138999;Lepidoptera;COI;;;;;
MN138999;Lepidoptera;COI;;;;;
MN139005;Lepidoptera;COI;;;;;
MN139007;Lepidoptera;COI;;;;;
MN139008;Lepidoptera;COI;;;;;
MN139026;Lepidoptera;COI;;;;;
MN139030;Lepidoptera;COI;;;;;
MN139044;Lepidoptera;COI;;;;;
MN139063;Lepidoptera;COI;;;;;
MN139076;Lepidoptera;COI;;;;;
MN139102;Lepidoptera;COI;;;;;
MN139103;Lepidoptera;COI;;;;;
MN139119;Lepidoptera;COI;;;;;
MN139136;Lepidoptera;COI;;;;;
MN139142;Lepidoptera;COI;;;;;
MN139143;Lepidoptera;COI;;;;;
MN139185;Lepidoptera;COI;;;;;
MN139186;Lepidoptera;COI;;;;;
MN139201;Lepidoptera;COI;;;;;
MN139207;Lepidoptera;COI;;;;;
MN139216;Lepidoptera;COI;;;;;
MN139250;Lepidoptera;COI;;;;;
MN139261;Lepidoptera;COI;;;;;
MN139261;Lepidoptera;COI;;;;;
MN139269;Lepidoptera;COI;;;;;
MN139297;Lepidoptera;COI;;;;;
MN139303;Lepidoptera;COI;;;;;
MN139306;Lepidoptera;COI;;;;;
MN139310;Lepidoptera;COI;;;;;
MN139310;Lepidoptera;COI;;;;;
MN139323;Lepidoptera;COI;;;;;
MN139335;Lepidoptera;COI;;;;;
MN139347;Lepidoptera;COI;;;;;
MN139347;Lepidoptera;COI;;;;;
MN139348;Lepidoptera;COI;;;;;
MN139363;Lepidoptera;COI;;;;;
MN139364;Lepidoptera;COI;;;;;
MN139378;Lepidoptera;COI;;;;;
MN139385;Lepidoptera;COI;;;;;
MN139395;Lepidoptera;COI;;;;;
MN139406;Lepidoptera;COI;;;;;
MN139416;Lepidoptera;COI;;;;;
MN139424;Lepidoptera;COI;;;;;
MN139429;Lepidoptera;COI;;;;;
MN139442;Lepidoptera;COI;;;;;
MN139445;Lepidoptera;COI;;;;;
MN139462;Lepidoptera;COI;;;;;
MN139462;Lepidoptera;COI;;;;;
MN139466;Lepidoptera;COI;;;;;

MN139469;Lepidoptera;COI;;;;;
MN139479;Lepidoptera;COI;;;;;
MN139499;Lepidoptera;COI;;;;;
MN139499;Lepidoptera;COI;;;;;
MN139507;Lepidoptera;COI;;;;;
MN139532;Lepidoptera;COI;;;;;
MN139546;Lepidoptera;COI;;;;;
MN139547;Lepidoptera;COI;;;;;
MN139555;Lepidoptera;COI;;;;;
MN139555;Lepidoptera;COI;;;;;
MN139576;Lepidoptera;COI;;;;;
MN139576;Lepidoptera;COI;;;;;
MN139599;Lepidoptera;COI;;;;;
MN139607;Lepidoptera;COI;;;;;
MN139631;Lepidoptera;COI;;;;;
MN139645;Lepidoptera;COI;;;;;
MN139666;Lepidoptera;COI;;;;;
MN139683;Lepidoptera;COI;;;;;
MN139699;Lepidoptera;COI;;;;;
MN139715;Lepidoptera;COI;;;;;
MN139722;Lepidoptera;COI;;;;;
MN139722;Lepidoptera;COI;;;;;
MN139726;Lepidoptera;COI;;;;;
MN139743;Lepidoptera;COI;;;;;
MN139757;Lepidoptera;COI;;;;;
MN139759;Lepidoptera;COI;;;;;
MN139772;Lepidoptera;COI;;;;;
MN139776;Lepidoptera;COI;;;;;
MN139780;Lepidoptera;COI;;;;;
MN139788;Lepidoptera;COI;;;;;
MN139825;Lepidoptera;COI;;;;;
MN139858;Lepidoptera;COI;;;;;
MN139865;Lepidoptera;COI;;;;;
MN139875;Lepidoptera;COI;;;;;
MN139918;Lepidoptera;COI;;;;;
MN139944;Lepidoptera;COI;;;;;
MN139955;Lepidoptera;COI;;;;;
MN139955;Lepidoptera;COI;;;;;
MN139966;Lepidoptera;COI;;;;;
MN139974;Lepidoptera;COI;;;;;
MN139974;Lepidoptera;COI;;;;;
MN139981;Lepidoptera;COI;;;;;
MN139981;Lepidoptera;COI;;;;;
MN139994;Lepidoptera;COI;;;;;
MN139999;Lepidoptera;COI;;;;;
MN140002;Lepidoptera;COI;;;;;
MN140002;Lepidoptera;COI;;;;;
MN140007;Lepidoptera;COI;;;;;
MN140010;Lepidoptera;COI;;;;;
MN140017;Lepidoptera;COI;;;;;
MN140044;Lepidoptera;COI;;;;;
MN140064;Lepidoptera;COI;;;;;
MN140065;Lepidoptera;COI;;;;;
MN140073;Lepidoptera;COI;;;;;
MN140099;Lepidoptera;COI;;;;;
MN140119;Lepidoptera;COI;;;;;
MN140133;Lepidoptera;COI;;;;;
MN140163;Lepidoptera;COI;;;;;
MN140168;Lepidoptera;COI;;;;;
MN140168;Lepidoptera;COI;;;;;
MN140170;Lepidoptera;COI;;;;;
MN140174;Lepidoptera;COI;;;;;
MN140190;Lepidoptera;COI;;;;;
MN140236;Lepidoptera;COI;;;;;
MN140237;Lepidoptera;COI;;;;;
MN140269;Lepidoptera;COI;;;;;
MN140294;Lepidoptera;COI;;;;;
MN140301;Lepidoptera;COI;;;;;
MN140301;Lepidoptera;COI;;;;;
MN140314;Lepidoptera;COI;;;;;
MN140323;Lepidoptera;COI;;;;;
MN140336;Lepidoptera;COI;;;;;
MN140345;Lepidoptera;COI;;;;;
MN140372;Lepidoptera;COI;;;;;
MN140396;Lepidoptera;COI;;;;;
MN140407;Lepidoptera;COI;;;;;
MN140427;Lepidoptera;COI;;;;;
MN140431;Lepidoptera;COI;;;;;

MN140451;Lepidoptera;COI;;;;;
MN140493;Lepidoptera;COI;;;;;
MN140503;Lepidoptera;COI;;;;;
MN140525;Lepidoptera;COI;;;;;
MN140543;Lepidoptera;COI;;;;;
MN140549;Lepidoptera;COI;;;;;
MN140553;Lepidoptera;COI;;;;;
MN140559;Lepidoptera;COI;;;;;
MN140566;Lepidoptera;COI;;;;;
MN140566;Lepidoptera;COI;;;;;
MN140572;Lepidoptera;COI;;;;;
MN140603;Lepidoptera;COI;;;;;
MN140614;Lepidoptera;COI;;;;;
MN140614;Lepidoptera;COI;;;;;
MN140634;Lepidoptera;COI;;;;;
MN140636;Lepidoptera;COI;;;;;
MN140650;Lepidoptera;COI;;;;;
MN140650;Lepidoptera;COI;;;;;
MN140653;Lepidoptera;COI;;;;;
MN140671;Lepidoptera;COI;;;;;
MN140695;Lepidoptera;COI;;;;;
MN140695;Lepidoptera;COI;;;;;
MN140705;Lepidoptera;COI;;;;;
MN140715;Lepidoptera;COI;;;;;
MN140724;Lepidoptera;COI;;;;;
MN140744;Lepidoptera;COI;;;;;
MN140752;Lepidoptera;COI;;;;;
MN140792;Lepidoptera;COI;;;;;
MN140817;Lepidoptera;COI;;;;;
MN140826;Lepidoptera;COI;;;;;
MN140832;Lepidoptera;COI;;;;;
MN140836;Lepidoptera;COI;;;;;
MN140836;Lepidoptera;COI;;;;;
MN140839;Lepidoptera;COI;;;;;
MN140839;Lepidoptera;COI;;;;;
MN140844;Lepidoptera;COI;;;;;
MN140844;Lepidoptera;COI;;;;;
MN140845;Lepidoptera;COI;;;;;
MN140847;Lepidoptera;COI;;;;;
MN140856;Lepidoptera;COI;;;;;
MN140862;Lepidoptera;COI;;;;;
MN140867;Lepidoptera;COI;;;;;
MN140868;Lepidoptera;COI;;;;;
MN140881;Lepidoptera;COI;;;;;
MN140899;Lepidoptera;COI;;;;;
MN140908;Lepidoptera;COI;;;;;
MN140951;Lepidoptera;COI;;;;;
MN140959;Lepidoptera;COI;;;;;
MN140996;Lepidoptera;COI;;;;;
MN140997;Lepidoptera;COI;;;;;
MN140998;Lepidoptera;COI;;;;;
MN140999;Lepidoptera;COI;;;;;
MN141007;Lepidoptera;COI;;;;;
MN141024;Lepidoptera;COI;;;;;
MN141025;Lepidoptera;COI;;;;;
MN141026;Lepidoptera;COI;;;;;
MN141032;Lepidoptera;COI;;;;;
MN141043;Lepidoptera;COI;;;;;
MN141050;Lepidoptera;COI;;;;;
MN141055;Lepidoptera;COI;;;;;
MN141066;Lepidoptera;COI;;;;;
MN141068;Lepidoptera;COI;;;;;
MN141074;Lepidoptera;COI;;;;;
MN141083;Lepidoptera;COI;;;;;
MN141117;Lepidoptera;COI;;;;;
MN141131;Lepidoptera;COI;;;;;
MN141133;Lepidoptera;COI;;;;;
MN141138;Lepidoptera;COI;;;;;
MN141138;Lepidoptera;COI;;;;;
MN141142;Lepidoptera;COI;;;;;
MN141156;Lepidoptera;COI;;;;;
MN141176;Lepidoptera;COI;;;;;
MN141182;Lepidoptera;COI;;;;;
MN141192;Lepidoptera;COI;;;;;
MN141220;Lepidoptera;COI;;;;;
MN141246;Lepidoptera;COI;;;;;
MN141257;Lepidoptera;COI;;;;;
MN141260;Lepidoptera;COI;;;;;

MN141267;Lepidoptera;COI;;;;;
MN141270;Lepidoptera;COI;;;;;
MN141276;Lepidoptera;COI;;;;;
MN141287;Lepidoptera;COI;;;;;
MN141290;Lepidoptera;COI;;;;;
MN141307;Lepidoptera;COI;;;;;
MN141334;Lepidoptera;COI;;;;;
MN141347;Lepidoptera;COI;;;;;
MN141347;Lepidoptera;COI;;;;;
MN141354;Lepidoptera;COI;;;;;
MN141354;Lepidoptera;COI;;;;;
MN141355;Lepidoptera;COI;;;;;
MN141366;Lepidoptera;COI;;;;;
MN141368;Lepidoptera;COI;;;;;
MN141383;Lepidoptera;COI;;;;;
MN141396;Lepidoptera;COI;;;;;
MN141400;Lepidoptera;COI;;;;;
MN141404;Lepidoptera;COI;;;;;
MN141404;Lepidoptera;COI;;;;;
MN141408;Lepidoptera;COI;;;;;
MN141412;Lepidoptera;COI;;;;;
MN141414;Lepidoptera;COI;;;;;
MN141419;Lepidoptera;COI;;;;;
MN141440;Lepidoptera;COI;;;;;
MN141440;Lepidoptera;COI;;;;;
MN141467;Lepidoptera;COI;;;;;
MN141487;Lepidoptera;COI;;;;;
MN141493;Lepidoptera;COI;;;;;
MN141508;Lepidoptera;COI;;;;;
MN141509;Lepidoptera;COI;;;;;
MN141512;Lepidoptera;COI;;;;;
MN141513;Lepidoptera;COI;;;;;
MN141527;Lepidoptera;COI;;;;;
MN141552;Lepidoptera;COI;;;;;
MN141574;Lepidoptera;COI;;;;;
MN141585;Lepidoptera;COI;;;;;
MN141595;Lepidoptera;COI;;;;;
MN141608;Lepidoptera;COI;;;;;
MN141618;Lepidoptera;COI;;;;;
MN141628;Lepidoptera;COI;;;;;
MN141634;Lepidoptera;COI;;;;;
MN141634;Lepidoptera;COI;;;;;
MN141646;Lepidoptera;COI;;;;;
MN141648;Lepidoptera;COI;;;;;
MN141649;Lepidoptera;COI;;;;;
MN141672;Lepidoptera;COI;;;;;
MN141675;Lepidoptera;COI;;;;;
MN141690;Lepidoptera;COI;;;;;
MN141701;Lepidoptera;COI;;;;;
MN141715;Lepidoptera;COI;;;;;
MN141748;Lepidoptera;COI;;;;;
MN141753;Lepidoptera;COI;;;;;
MN141756;Lepidoptera;COI;;;;;
MN141763;Lepidoptera;COI;;;;;
MN141789;Lepidoptera;COI;;;;;
MN141800;Lepidoptera;COI;;;;;
MN141806;Lepidoptera;COI;;;;;
MN141816;Lepidoptera;COI;;;;;
MN141832;Lepidoptera;COI;;;;;
MN141854;Lepidoptera;COI;;;;;
MN141857;Lepidoptera;COI;;;;;
MN141866;Lepidoptera;COI;;;;;
MN141897;Lepidoptera;COI;;;;;
MN141899;Lepidoptera;COI;;;;;
MN141913;Lepidoptera;COI;;;;;
MN141932;Lepidoptera;COI;;;;;
MN141939;Lepidoptera;COI;;;;;
MN141965;Lepidoptera;COI;;;;;
MN141981;Lepidoptera;COI;;;;;
MN141981;Lepidoptera;COI;;;;;
MN141982;Lepidoptera;COI;;;;;
MN141982;Lepidoptera;COI;;;;;
MN141985;Lepidoptera;COI;;;;;
MN141995;Lepidoptera;COI;;;;;
MN141996;Lepidoptera;COI;;;;;
MN142002;Lepidoptera;COI;;;;;
MN142021;Lepidoptera;COI;;;;;
MN142022;Lepidoptera;COI;;;;;

MN142027;Lepidoptera;COI;;;;;
MN142038;Lepidoptera;COI;;;;;
MN142039;Lepidoptera;COI;;;;;
MN142064;Lepidoptera;COI;;;;;
MN142103;Lepidoptera;COI;;;;;
MN142124;Lepidoptera;COI;;;;;
MN142124;Lepidoptera;COI;;;;;
MN142138;Lepidoptera;COI;;;;;
MN142138;Lepidoptera;COI;;;;;
MN142142;Lepidoptera;COI;;;;;
MN142164;Lepidoptera;COI;;;;;
MN142184;Lepidoptera;COI;;;;;
MN142240;Lepidoptera;COI;;;;;
MN142259;Lepidoptera;COI;;;;;
MN142267;Lepidoptera;COI;;;;;
MN142270;Lepidoptera;COI;;;;;
MN142293;Lepidoptera;COI;;;;;
MN142321;Lepidoptera;COI;;;;;
MN142357;Lepidoptera;COI;;;;;
MN142370;Lepidoptera;COI;;;;;
MN142376;Lepidoptera;COI;;;;;
MN142396;Lepidoptera;COI;;;;;
MN142413;Lepidoptera;COI;;;;;
MN142421;Lepidoptera;COI;;;;;
MN142429;Lepidoptera;COI;;;;;
MN142430;Lepidoptera;COI;;;;;
MN142435;Lepidoptera;COI;;;;;
MN142446;Lepidoptera;COI;;;;;
MN142446;Lepidoptera;COI;;;;;
MN142453;Lepidoptera;COI;;;;;
MN142467;Lepidoptera;COI;;;;;
MN142487;Lepidoptera;COI;;;;;
MN142489;Lepidoptera;COI;;;;;
MN142511;Lepidoptera;COI;;;;;
MN142514;Lepidoptera;COI;;;;;
MN142515;Lepidoptera;COI;;;;;
MN142525;Lepidoptera;COI;;;;;
MN142525;Lepidoptera;COI;;;;;
MN142535;Lepidoptera;COI;;;;;
MN142549;Lepidoptera;COI;;;;;
MN142550;Lepidoptera;COI;;;;;
MN142598;Lepidoptera;COI;;;;;
MN142601;Lepidoptera;COI;;;;;
MN142602;Lepidoptera;COI;;;;;
MN142605;Lepidoptera;COI;;;;;
MN142611;Lepidoptera;COI;;;;;
MN142616;Lepidoptera;COI;;;;;
MN142641;Lepidoptera;COI;;;;;
MN142641;Lepidoptera;COI;;;;;
MN142662;Lepidoptera;COI;;;;;
MN142670;Lepidoptera;COI;;;;;
MN142685;Lepidoptera;COI;;;;;
MN142702;Lepidoptera;COI;;;;;
MN142705;Lepidoptera;COI;;;;;
MN142736;Lepidoptera;COI;;;;;
MN142751;Lepidoptera;COI;;;;;
MN142757;Lepidoptera;COI;;;;;
MN142769;Lepidoptera;COI;;;;;
MN142772;Lepidoptera;COI;;;;;
MN142777;Lepidoptera;COI;;;;;
MN142777;Lepidoptera;COI;;;;;
MN142794;Lepidoptera;COI;;;;;
MN142804;Lepidoptera;COI;;;;;
MN142811;Lepidoptera;COI;;;;;
MN142812;Lepidoptera;COI;;;;;
MN142812;Lepidoptera;COI;;;;;
MN142894;Lepidoptera;COI;;;;;
MN142917;Lepidoptera;COI;;;;;
MN142941;Lepidoptera;COI;;;;;
MN142945;Lepidoptera;COI;;;;;
MN142957;Lepidoptera;COI;;;;;
MN142958;Lepidoptera;COI;;;;;
MN142961;Lepidoptera;COI;;;;;
MN142961;Lepidoptera;COI;;;;;
MN142989;Lepidoptera;COI;;;;;
MN142996;Lepidoptera;COI;;;;;
MN143009;Lepidoptera;COI;;;;;
MN143018;Lepidoptera;COI;;;;;

MN143028;Lepidoptera;COI;;;;;
MN143034;Lepidoptera;COI;;;;;
MN143034;Lepidoptera;COI;;;;;
MN143039;Lepidoptera;COI;;;;;
MN143057;Lepidoptera;COI;;;;;
MN143072;Lepidoptera;COI;;;;;
MN143091;Lepidoptera;COI;;;;;
MN143099;Lepidoptera;COI;;;;;
MN143100;Lepidoptera;COI;;;;;
MN143123;Lepidoptera;COI;;;;;
MN143128;Lepidoptera;COI;;;;;
MN143138;Lepidoptera;COI;;;;;
MN143171;Lepidoptera;COI;;;;;
MN143179;Lepidoptera;COI;;;;;
MN143184;Lepidoptera;COI;;;;;
MN143214;Lepidoptera;COI;;;;;
MN143225;Lepidoptera;COI;;;;;
MN143249;Lepidoptera;COI;;;;;
MN143264;Lepidoptera;COI;;;;;
MN143272;Lepidoptera;COI;;;;;
MN143273;Lepidoptera;COI;;;;;
MN143281;Lepidoptera;COI;;;;;
MN143317;Lepidoptera;COI;;;;;
MN143333;Lepidoptera;COI;;;;;
MN143357;Lepidoptera;COI;;;;;
MN143357;Lepidoptera;COI;;;;;
MN143376;Lepidoptera;COI;;;;;
MN143378;Lepidoptera;COI;;;;;
MN143389;Lepidoptera;COI;;;;;
MN143400;Lepidoptera;COI;;;;;
MN143405;Lepidoptera;COI;;;;;
MN143435;Lepidoptera;COI;;;;;
MN143444;Lepidoptera;COI;;;;;
MN143449;Lepidoptera;COI;;;;;
MN143468;Lepidoptera;COI;;;;;
MN143474;Lepidoptera;COI;;;;;
MN143486;Lepidoptera;COI;;;;;
MN143500;Lepidoptera;COI;;;;;
MN143504;Lepidoptera;COI;;;;;
MN143514;Lepidoptera;COI;;;;;
MN143583;Lepidoptera;COI;;;;;
MN143600;Lepidoptera;COI;;;;;
MN143610;Lepidoptera;COI;;;;;
MN143637;Lepidoptera;COI;;;;;
MN143663;Lepidoptera;COI;;;;;
MN143669;Lepidoptera;COI;;;;;
MN143688;Lepidoptera;COI;;;;;
MN143688;Lepidoptera;COI;;;;;
MN143695;Lepidoptera;COI;;;;;
MN143710;Lepidoptera;COI;;;;;
MN143727;Lepidoptera;COI;;;;;
MN143732;Lepidoptera;COI;;;;;
MN143732;Lepidoptera;COI;;;;;
MN143740;Lepidoptera;COI;;;;;
MN143746;Lepidoptera;COI;;;;;
MN143753;Lepidoptera;COI;;;;;
MN143770;Lepidoptera;COI;;;;;
MN143771;Lepidoptera;COI;;;;;
MN143779;Lepidoptera;COI;;;;;
MN143784;Lepidoptera;COI;;;;;
MN143786;Lepidoptera;COI;;;;;
MN143805;Lepidoptera;COI;;;;;
MN143806;Lepidoptera;COI;;;;;
MN143820;Lepidoptera;COI;;;;;
MN143828;Lepidoptera;COI;;;;;
MN143852;Lepidoptera;COI;;;;;
MN143873;Lepidoptera;COI;;;;;
MN143874;Lepidoptera;COI;;;;;
MN143900;Lepidoptera;COI;;;;;
MN143903;Lepidoptera;COI;;;;;
MN143911;Lepidoptera;COI;;;;;
MN143912;Lepidoptera;COI;;;;;
MN143915;Lepidoptera;COI;;;;;
MN143922;Lepidoptera;COI;;;;;
MN143925;Lepidoptera;COI;;;;;
MN143935;Lepidoptera;COI;;;;;
MN143980;Lepidoptera;COI;;;;;
MN144006;Lepidoptera;COI;;;;;

MN144007;Lepidoptera;COI;;;;;
MN144009;Lepidoptera;COI;;;;;
MN144010;Lepidoptera;COI;;;;;
MN144059;Lepidoptera;COI;;;;;
MN144063;Lepidoptera;COI;;;;;
MN144067;Lepidoptera;COI;;;;;
MN144071;Lepidoptera;COI;;;;;
MN144119;Lepidoptera;COI;;;;;
MN144119;Lepidoptera;COI;;;;;
MN144123;Lepidoptera;COI;;;;;
MN144131;Lepidoptera;COI;;;;;
MN144134;Lepidoptera;COI;;;;;
MN144148;Lepidoptera;COI;;;;;
MN144198;Lepidoptera;COI;;;;;
MN144219;Lepidoptera;COI;;;;;
MN144250;Lepidoptera;COI;;;;;
MN144250;Lepidoptera;COI;;;;;
MN144256;Lepidoptera;COI;;;;;
MN144261;Lepidoptera;COI;;;;;
MN144265;Lepidoptera;COI;;;;;
MN144282;Lepidoptera;COI;;;;;
MN144296;Lepidoptera;COI;;;;;
MN144297;Lepidoptera;COI;;;;;
MN144304;Lepidoptera;COI;;;;;
MN144308;Lepidoptera;COI;;;;;
MN144320;Lepidoptera;COI;;;;;
MN144323;Lepidoptera;COI;;;;;
MN144324;Lepidoptera;COI;;;;;
MN144327;Lepidoptera;COI;;;;;
MN144332;Lepidoptera;COI;;;;;
MN144332;Lepidoptera;COI;;;;;
MN144345;Lepidoptera;COI;;;;;
MN144350;Lepidoptera;COI;;;;;
MN144367;Lepidoptera;COI;;;;;
MN144382;Lepidoptera;COI;;;;;
MN144389;Lepidoptera;COI;;;;;
MN144421;Lepidoptera;COI;;;;;
MN144421;Lepidoptera;COI;;;;;
MN144434;Lepidoptera;COI;;;;;
MN144435;Lepidoptera;COI;;;;;
MN144442;Lepidoptera;COI;;;;;
MN144456;Lepidoptera;COI;;;;;
MN144458;Lepidoptera;COI;;;;;
MN144463;Lepidoptera;COI;;;;;
MN144463;Lepidoptera;COI;;;;;
MN144486;Lepidoptera;COI;;;;;
MN144497;Lepidoptera;COI;;;;;
MN144501;Lepidoptera;COI;;;;;
MN144502;Lepidoptera;COI;;;;;
MN144504;Lepidoptera;COI;;;;;
MN144515;Lepidoptera;COI;;;;;
MN144533;Lepidoptera;COI;;;;;
MN144546;Lepidoptera;COI;;;;;
MN144554;Lepidoptera;COI;;;;;
MN144582;Lepidoptera;COI;;;;;
MN144583;Lepidoptera;COI;;;;;
MN144591;Lepidoptera;COI;;;;;
MN144592;Lepidoptera;COI;;;;;
MN144595;Lepidoptera;COI;;;;;
MN144602;Lepidoptera;COI;;;;;
MN144602;Lepidoptera;COI;;;;;
MN144632;Lepidoptera;COI;;;;;
MN144632;Lepidoptera;COI;;;;;
MN144635;Lepidoptera;COI;;;;;
MN144637;Lepidoptera;COI;;;;;
MN144637;Lepidoptera;COI;;;;;
MN144644;Lepidoptera;COI;;;;;
MN144649;Lepidoptera;COI;;;;;
MN144695;Lepidoptera;COI;;;;;
MN144726;Lepidoptera;COI;;;;;
MN144730;Lepidoptera;COI;;;;;
MN144734;Lepidoptera;COI;;;;;
MN144743;Lepidoptera;COI;;;;;
MN144749;Lepidoptera;COI;;;;;
MN144763;Lepidoptera;COI;;;;;
MN144763;Lepidoptera;COI;;;;;
MN144766;Lepidoptera;COI;;;;;
MN144771;Lepidoptera;COI;;;;;

MN144771;Lepidoptera;COI;;;;;
MN144818;Lepidoptera;COI;;;;;
MN144818;Lepidoptera;COI;;;;;
MN144821;Lepidoptera;COI;;;;;
MN144828;Lepidoptera;COI;;;;;
MN144828;Lepidoptera;COI;;;;;
MN144856;Lepidoptera;COI;;;;;
MN144873;Lepidoptera;COI;;;;;
MN144873;Lepidoptera;COI;;;;;
MN144909;Lepidoptera;COI;;;;;
MN144909;Lepidoptera;COI;;;;;
MN144928;Lepidoptera;COI;;;;;
MN144937;Lepidoptera;COI;;;;;
MN144941;Lepidoptera;COI;;;;;
MN144944;Lepidoptera;COI;;;;;
MN144945;Lepidoptera;COI;;;;;
MN144952;Lepidoptera;COI;;;;;
MN144978;Lepidoptera;COI;;;;;
MN144981;Lepidoptera;COI;;;;;
MN144982;Lepidoptera;COI;;;;;
MN145008;Lepidoptera;COI;;;;;
MN145009;Lepidoptera;COI;;;;;
MN145024;Lepidoptera;COI;;;;;
MN145025;Lepidoptera;COI;;;;;
MN145032;Lepidoptera;COI;;;;;
MN145043;Lepidoptera;COI;;;;;
MN145051;Lepidoptera;COI;;;;;
MN145056;Lepidoptera;COI;;;;;
MN145078;Lepidoptera;COI;;;;;
MN145086;Lepidoptera;COI;;;;;
MN145088;Lepidoptera;COI;;;;;
MN145095;Lepidoptera;COI;;;;;
MN145108;Lepidoptera;COI;;;;;
MN145120;Lepidoptera;COI;;;;;
MN145120;Lepidoptera;COI;;;;;
MN145143;Lepidoptera;COI;;;;;
MN145149;Lepidoptera;COI;;;;;
MN145166;Lepidoptera;COI;;;;;
MN145167;Lepidoptera;COI;;;;;
MN145167;Lepidoptera;COI;;;;;
MN145169;Lepidoptera;COI;;;;;
MN145174;Lepidoptera;COI;;;;;
MN145193;Lepidoptera;COI;;;;;
MN145201;Lepidoptera;COI;;;;;
MN145249;Lepidoptera;COI;;;;;
MN145249;Lepidoptera;COI;;;;;
MN145252;Lepidoptera;COI;;;;;
MN145259;Lepidoptera;COI;;;;;
MN145264;Lepidoptera;COI;;;;;
MN145267;Lepidoptera;COI;;;;;
MN145267;Lepidoptera;COI;;;;;
MN145274;Lepidoptera;COI;;;;;
MN145281;Lepidoptera;COI;;;;;
MN145284;Lepidoptera;COI;;;;;
MN145285;Lepidoptera;COI;;;;;
MN145288;Lepidoptera;COI;;;;;
MN145310;Lepidoptera;COI;;;;;
MN145316;Lepidoptera;COI;;;;;
MN145327;Lepidoptera;COI;;;;;
MN145336;Lepidoptera;COI;;;;;
MN145378;Lepidoptera;COI;;;;;
MN145381;Lepidoptera;COI;;;;;
MN145388;Lepidoptera;COI;;;;;
MN145399;Lepidoptera;COI;;;;;
MN145409;Lepidoptera;COI;;;;;
MN145433;Lepidoptera;COI;;;;;
MN145435;Lepidoptera;COI;;;;;
MN145436;Lepidoptera;COI;;;;;
MN145436;Lepidoptera;COI;;;;;
MN145439;Lepidoptera;COI;;;;;
MN829476;Lepidoptera;COI;;;;;
MN829491;Lepidoptera;COI;;;;;
MN888486;Lepidoptera;COI;;;;;
MN888487;Lepidoptera;COI;;;;;
MN888488;Lepidoptera;COI;;;;;
MN888489;Lepidoptera;COI;;;;;
MN992986;Lepidoptera;COI;;;;;
MN993024;Lepidoptera;COI;;;;;

MN993041;Lepidoptera;COI;;;;;
MN993054;Lepidoptera;COI;;;;;
MN993064;Lepidoptera;COI;;;;;
MT151352;Lepidoptera;COI;;;;;
MT151353;Lepidoptera;COI;;;;;
MT151368;Lepidoptera;COI;;;;;
MT210322;Lepidoptera;COI;;;;;
MT407192;Lepidoptera;COI;;;;;
MT407192;Lepidoptera;COI;;;;;
MT407194;Lepidoptera;COI;;;;;
MT407194;Lepidoptera;COI;;;;;
MT407198;Lepidoptera;COI;;;;;
MT407198;Lepidoptera;COI;;;;;
MT762427;Lepidoptera;COI;;;;;
MT762428;Lepidoptera;COI;;;;;
MT762429;Lepidoptera;COI;;;;;
MT762430;Lepidoptera;COI;;;;;
MT762431;Lepidoptera;COI;;;;;
MT762432;Lepidoptera;COI;;;;;
MT762433;Lepidoptera;COI;;;;;
MT762434;Lepidoptera;COI;;;;;
MT762435;Lepidoptera;COI;;;;;
MT762436;Lepidoptera;COI;;;;;
MT762437;Lepidoptera;COI;;;;;
MT762438;Lepidoptera;COI;;;;;
MT762441;Lepidoptera;COI;;;;;
MT762442;Lepidoptera;COI;;;;;
MT762444;Lepidoptera;COI;;;;;
MT762445;Lepidoptera;COI;;;;;
MT762446;Lepidoptera;COI;;;;;
MT762447;Lepidoptera;COI;;;;;
MT762448;Lepidoptera;COI;;;;;
MT762449;Lepidoptera;COI;;;;;
MT762450;Lepidoptera;COI;;;;;
MT762451;Lepidoptera;COI;;;;;
MT762452;Lepidoptera;COI;;;;;
MT762453;Lepidoptera;COI;;;;;
MT762454;Lepidoptera;COI;;;;;
MT762455;Lepidoptera;COI;;;;;
MT762456;Lepidoptera;COI;;;;;
MT762457;Lepidoptera;COI;;;;;
MT762458;Lepidoptera;COI;;;;;
MT762459;Lepidoptera;COI;;;;;
MT762460;Lepidoptera;COI;;;;;
MT762461;Lepidoptera;COI;;;;;
MT762462;Lepidoptera;COI;;;;;
MT762464;Lepidoptera;COI;;;;;
MT762465;Lepidoptera;COI;;;;;
MT762466;Lepidoptera;COI;;;;;
MT762467;Lepidoptera;COI;;;;;
MT762468;Lepidoptera;COI;;;;;
MT762469;Lepidoptera;COI;;;;;
MT762470;Lepidoptera;COI;;;;;
MT762471;Lepidoptera;COI;;;;;
MT762473;Lepidoptera;COI;;;;;
MT762474;Lepidoptera;COI;;;;;
MT762475;Lepidoptera;COI;;;;;
MT762476;Lepidoptera;COI;;;;;
MT762477;Lepidoptera;COI;;;;;
MT762478;Lepidoptera;COI;;;;;
MT762479;Lepidoptera;COI;;;;;
MT762480;Lepidoptera;COI;;;;;
MT762481;Lepidoptera;COI;;;;;
MT762482;Lepidoptera;COI;;;;;
MT762483;Lepidoptera;COI;;;;;
MT762484;Lepidoptera;COI;;;;;
MT762485;Lepidoptera;COI;;;;;
MT762486;Lepidoptera;COI;;;;;
MT762487;Lepidoptera;COI;;;;;
MT762488;Lepidoptera;COI;;;;;
MT762489;Lepidoptera;COI;;;;;
MT762490;Lepidoptera;COI;;;;;
MT762491;Lepidoptera;COI;;;;;
MT762492;Lepidoptera;COI;;;;;
MT762493;Lepidoptera;COI;;;;;
MT762494;Lepidoptera;COI;;;;;
MT762495;Lepidoptera;COI;;;;;
MT762496;Lepidoptera;COI;;;;;

MT762497;Lepidoptera;COI;;;;;
MT762499;Lepidoptera;COI;;;;;
MT762500;Lepidoptera;COI;;;;;
MT762501;Lepidoptera;COI;;;;;
MT762502;Lepidoptera;COI;;;;;
MT762503;Lepidoptera;COI;;;;;
MT762505;Lepidoptera;COI;;;;;
MT762506;Lepidoptera;COI;;;;;
MT762507;Lepidoptera;COI;;;;;
MT762508;Lepidoptera;COI;;;;;
MT762509;Lepidoptera;COI;;;;;
MT762510;Lepidoptera;COI;;;;;
MT762512;Lepidoptera;COI;;;;;
MT762513;Lepidoptera;COI;;;;;
MT762515;Lepidoptera;COI;;;;;
MT762516;Lepidoptera;COI;;;;;
MT762517;Lepidoptera;COI;;;;;
MT762518;Lepidoptera;COI;;;;;
MT762519;Lepidoptera;COI;;;;;
MT762520;Lepidoptera;COI;;;;;
MT762521;Lepidoptera;COI;;;;;
MT762522;Lepidoptera;COI;;;;;
MT762523;Lepidoptera;COI;;;;;
MT762524;Lepidoptera;COI;;;;;
MT762525;Lepidoptera;COI;;;;;
MT762526;Lepidoptera;COI;;;;;
MT762527;Lepidoptera;COI;;;;;
MT762528;Lepidoptera;COI;;;;;
MT762529;Lepidoptera;COI;;;;;
MT762530;Lepidoptera;COI;;;;;
MT762531;Lepidoptera;COI;;;;;
MT762532;Lepidoptera;COI;;;;;
MT762533;Lepidoptera;COI;;;;;
MT762534;Lepidoptera;COI;;;;;
MT762535;Lepidoptera;COI;;;;;
MT762536;Lepidoptera;COI;;;;;
MT762537;Lepidoptera;COI;;;;;
MT762538;Lepidoptera;COI;;;;;
MT762539;Lepidoptera;COI;;;;;
MT762540;Lepidoptera;COI;;;;;
MT762541;Lepidoptera;COI;;;;;
MT762542;Lepidoptera;COI;;;;;
MT762543;Lepidoptera;COI;;;;;
MT762544;Lepidoptera;COI;;;;;
MT762545;Lepidoptera;COI;;;;;
MT762546;Lepidoptera;COI;;;;;
MT762547;Lepidoptera;COI;;;;;
MT762548;Lepidoptera;COI;;;;;
MT762549;Lepidoptera;COI;;;;;
MT762551;Lepidoptera;COI;;;;;
MT762552;Lepidoptera;COI;;;;;
MT762553;Lepidoptera;COI;;;;;
MT762556;Lepidoptera;COI;;;;;
MT762557;Lepidoptera;COI;;;;;
MT762558;Lepidoptera;COI;;;;;
MT762559;Lepidoptera;COI;;;;;
MT762560;Lepidoptera;COI;;;;;
MT762561;Lepidoptera;COI;;;;;
MT762562;Lepidoptera;COI;;;;;
MT762563;Lepidoptera;COI;;;;;
MT762564;Lepidoptera;COI;;;;;
MT762565;Lepidoptera;COI;;;;;
MT762566;Lepidoptera;COI;;;;;
MT762568;Lepidoptera;COI;;;;;
MT762569;Lepidoptera;COI;;;;;
MT762570;Lepidoptera;COI;;;;;
MT762571;Lepidoptera;COI;;;;;
MT762572;Lepidoptera;COI;;;;;
MT762573;Lepidoptera;COI;;;;;
MT762574;Lepidoptera;COI;;;;;
MT762575;Lepidoptera;COI;;;;;
MT762576;Lepidoptera;COI;;;;;
MT762577;Lepidoptera;COI;;;;;
MT762578;Lepidoptera;COI;;;;;
MT762579;Lepidoptera;COI;;;;;
MT762580;Lepidoptera;COI;;;;;
MT762581;Lepidoptera;COI;;;;;
MT762582;Lepidoptera;COI;;;;;

MT762583;Lepidoptera;COI;;;;;
MT762584;Lepidoptera;COI;;;;;
MT762585;Lepidoptera;COI;;;;;
MT762587;Lepidoptera;COI;;;;;
MT762588;Lepidoptera;COI;;;;;
MT762589;Lepidoptera;COI;;;;;
MT762590;Lepidoptera;COI;;;;;
MT762591;Lepidoptera;COI;;;;;
MT762592;Lepidoptera;COI;;;;;
MT762593;Lepidoptera;COI;;;;;
MT762594;Lepidoptera;COI;;;;;
MT762595;Lepidoptera;COI;;;;;
MT762596;Lepidoptera;COI;;;;;
MT762597;Lepidoptera;COI;;;;;
MT762598;Lepidoptera;COI;;;;;
MT762600;Lepidoptera;COI;;;;;
MT762601;Lepidoptera;COI;;;;;
MT762602;Lepidoptera;COI;;;;;
MT878244;Lepidoptera;COI;;;;;
MT878245;Lepidoptera;COI;;;;;
MT878246;Lepidoptera;COI;;;;;
MT878247;Lepidoptera;COI;;;;;
MT878248;Lepidoptera;COI;;;;;
MT878249;Lepidoptera;COI;;;;;
MT878250;Lepidoptera;COI;;;;;
MT878251;Lepidoptera;COI;;;;;
MT878252;Lepidoptera;COI;;;;;
MT878253;Lepidoptera;COI;;;;;
MT878254;Lepidoptera;COI;;;;;
MT878255;Lepidoptera;COI;;;;;
MT878256;Lepidoptera;COI;;;;;
MT878257;Lepidoptera;COI;;;;;
MT878258;Lepidoptera;COI;;;;;
MT878259;Lepidoptera;COI;;;;;
MT878260;Lepidoptera;COI;;;;;
MT878261;Lepidoptera;COI;;;;;
MT878262;Lepidoptera;COI;;;;;
MT878263;Lepidoptera;COI;;;;;
MT878264;Lepidoptera;COI;;;;;
MT878265;Lepidoptera;COI;;;;;
MT878266;Lepidoptera;COI;;;;;
MT878267;Lepidoptera;COI;;;;;
MT878268;Lepidoptera;COI;;;;;
MT878269;Lepidoptera;COI;;;;;
MT878270;Lepidoptera;COI;;;;;
MT878271;Lepidoptera;COI;;;;;
MT878272;Lepidoptera;COI;;;;;
MT878277;Lepidoptera;COI;;;;;
MT878278;Lepidoptera;COI;;;;;
MT878279;Lepidoptera;COI;;;;;
MT878280;Lepidoptera;COI;;;;;
MT878281;Lepidoptera;COI;;;;;
MT878282;Lepidoptera;COI;;;;;
MT878283;Lepidoptera;COI;;;;;
MT878284;Lepidoptera;COI;;;;;
MT878285;Lepidoptera;COI;;;;;
MT878286;Lepidoptera;COI;;;;;
MT878287;Lepidoptera;COI;;;;;
MT878288;Lepidoptera;COI;;;;;
MT878289;Lepidoptera;COI;;;;;
MT878296;Lepidoptera;COI;;;;;
MT878297;Lepidoptera;COI;;;;;
MT878298;Lepidoptera;COI;;;;;
MT878299;Lepidoptera;COI;;;;;
MT878300;Lepidoptera;COI;;;;;
MT878301;Lepidoptera;COI;;;;;
MT878302;Lepidoptera;COI;;;;;
MT878303;Lepidoptera;COI;;;;;
MT878307;Lepidoptera;COI;;;;;
MT878308;Lepidoptera;COI;;;;;
MT878309;Lepidoptera;COI;;;;;
MT878314;Lepidoptera;COI;;;;;
MT878315;Lepidoptera;COI;;;;;
MT878316;Lepidoptera;COI;;;;;
MT878317;Lepidoptera;COI;;;;;
MT878318;Lepidoptera;COI;;;;;
MT878319;Lepidoptera;COI;;;;;
MT878320;Lepidoptera;COI;;;;;

MT878321;Lepidoptera;COI;;;;;
MT878322;Lepidoptera;COI;;;;;
MT878329;Lepidoptera;COI;;;;;
MT878330;Lepidoptera;COI;;;;;
MT878331;Lepidoptera;COI;;;;;
MT878332;Lepidoptera;COI;;;;;
MT878338;Lepidoptera;COI;;;;;
MT878340;Lepidoptera;COI;;;;;
MT878341;Lepidoptera;COI;;;;;
MT878342;Lepidoptera;COI;;;;;
MT878344;Lepidoptera;COI;;;;;
MT878345;Lepidoptera;COI;;;;;
MT878350;Lepidoptera;COI;;;;;
MT878351;Lepidoptera;COI;;;;;
MT878352;Lepidoptera;COI;;;;;
MT878353;Lepidoptera;COI;;;;;
MT878354;Lepidoptera;COI;;;;;
MT878355;Lepidoptera;COI;;;;;
MT878356;Lepidoptera;COI;;;;;
MT878357;Lepidoptera;COI;;;;;
MT878358;Lepidoptera;COI;;;;;
MT878359;Lepidoptera;COI;;;;;
MT878360;Lepidoptera;COI;;;;;
MT916588;Lepidoptera;COI;;;;;
MT916589;Lepidoptera;COI;;;;;
MT916590;Lepidoptera;COI;;;;;
MT916591;Lepidoptera;COI;;;;;
MT916592;Lepidoptera;COI;;;;;
MT916593;Lepidoptera;COI;;;;;
MT916594;Lepidoptera;COI;;;;;
MT916595;Lepidoptera;COI;;;;;
MT916596;Lepidoptera;COI;;;;;
MT916597;Lepidoptera;COI;;;;;
MT916598;Lepidoptera;COI;;;;;
MT916599;Lepidoptera;COI;;;;;
MT916600;Lepidoptera;COI;;;;;
MT916601;Lepidoptera;COI;;;;;
MT916602;Lepidoptera;COI;;;;;
MT916604;Lepidoptera;COI;;;;;
MW018369;Lepidoptera;COI;;;;;
MW018370;Lepidoptera;COI;;;;;
MW018371;Lepidoptera;COI;;;;;
MW018372;Lepidoptera;COI;;;;;
MW018373;Lepidoptera;COI;;;;;
MW018374;Lepidoptera;COI;;;;;
MW018375;Lepidoptera;COI;;;;;
MW018376;Lepidoptera;COI;;;;;
MW018377;Lepidoptera;COI;;;;;
MW018378;Lepidoptera;COI;;;;;
MW018379;Lepidoptera;COI;;;;;
MW018380;Lepidoptera;COI;;;;;
MW018381;Lepidoptera;COI;;;;;
MW018382;Lepidoptera;COI;;;;;
MW018383;Lepidoptera;COI;;;;;
MW018384;Lepidoptera;COI;;;;;
MW018385;Lepidoptera;COI;;;;;
MW018386;Lepidoptera;COI;;;;;
MW018387;Lepidoptera;COI;;;;;
MW018388;Lepidoptera;COI;;;;;
MW018389;Lepidoptera;COI;;;;;
MW018390;Lepidoptera;COI;;;;;
MW018391;Lepidoptera;COI;;;;;
MW018392;Lepidoptera;COI;;;;;
MW018393;Lepidoptera;COI;;;;;
MW018394;Lepidoptera;COI;;;;;
MW018395;Lepidoptera;COI;;;;;
MW018396;Lepidoptera;COI;;;;;
MW018397;Lepidoptera;COI;;;;;
MW018398;Lepidoptera;COI;;;;;
MW018399;Lepidoptera;COI;;;;;
MW020759;Lepidoptera;COI;;;;;
MW022127;Lepidoptera;COI;;;;;
MW022128;Lepidoptera;COI;;;;;
MW022129;Lepidoptera;COI;;;;;
MW441623;Lepidoptera;COI;;;;;
MW441625;Lepidoptera;COI;;;;;
MW498982;Lepidoptera;COI;;;;;
MW498999;Lepidoptera;COI;;;;;

MW498999;Lepidoptera;COI;;;;;
MW499015;Lepidoptera;COI;;;;;
MW499067;Lepidoptera;COI;;;;;
MW499070;Lepidoptera;COI;;;;;
MW499070;Lepidoptera;COI;;;;;
MW499071;Lepidoptera;COI;;;;;
MW499095;Lepidoptera;COI;;;;;
MW499106;Lepidoptera;COI;;;;;
MW499155;Lepidoptera;COI;;;;;
MW499155;Lepidoptera;COI;;;;;
MW499156;Lepidoptera;COI;;;;;
MW499174;Lepidoptera;COI;;;;;
MW499179;Lepidoptera;COI;;;;;
MW499182;Lepidoptera;COI;;;;;
MW499184;Lepidoptera;COI;;;;;
MW499186;Lepidoptera;COI;;;;;
MW499194;Lepidoptera;COI;;;;;
MW499195;Lepidoptera;COI;;;;;
MW499198;Lepidoptera;COI;;;;;
MW499215;Lepidoptera;COI;;;;;
MW499217;Lepidoptera;COI;;;;;
MW499222;Lepidoptera;COI;;;;;
MW499229;Lepidoptera;COI;;;;;
MW499233;Lepidoptera;COI;;;;;
MW499242;Lepidoptera;COI;;;;;
MW499242;Lepidoptera;COI;;;;;
MW499255;Lepidoptera;COI;;;;;
MW499257;Lepidoptera;COI;;;;;
MW499259;Lepidoptera;COI;;;;;
MW499261;Lepidoptera;COI;;;;;
MW499303;Lepidoptera;COI;;;;;
MW499337;Lepidoptera;COI;;;;;
MW499342;Lepidoptera;COI;;;;;
MW499343;Lepidoptera;COI;;;;;
MW499344;Lepidoptera;COI;;;;;
MW499367;Lepidoptera;COI;;;;;
MW499397;Lepidoptera;COI;;;;;
MW499400;Lepidoptera;COI;;;;;
MW499408;Lepidoptera;COI;;;;;
MW499410;Lepidoptera;COI;;;;;
MW499415;Lepidoptera;COI;;;;;
MW499443;Lepidoptera;COI;;;;;
MW499444;Lepidoptera;COI;;;;;
MW499444;Lepidoptera;COI;;;;;
MW499457;Lepidoptera;COI;;;;;
MW499500;Lepidoptera;COI;;;;;
MW499503;Lepidoptera;COI;;;;;
MW499503;Lepidoptera;COI;;;;;
MW499520;Lepidoptera;COI;;;;;
MW499541;Lepidoptera;COI;;;;;
MW499551;Lepidoptera;COI;;;;;
MW499571;Lepidoptera;COI;;;;;
MW499589;Lepidoptera;COI;;;;;
MW499597;Lepidoptera;COI;;;;;
MW499626;Lepidoptera;COI;;;;;
MW499631;Lepidoptera;COI;;;;;
MW499631;Lepidoptera;COI;;;;;
MW499639;Lepidoptera;COI;;;;;
MW499649;Lepidoptera;COI;;;;;
MW499651;Lepidoptera;COI;;;;;
MW499666;Lepidoptera;COI;;;;;
MW499668;Lepidoptera;COI;;;;;
MW499674;Lepidoptera;COI;;;;;
MW499685;Lepidoptera;COI;;;;;
MW499693;Lepidoptera;COI;;;;;
MW499699;Lepidoptera;COI;;;;;
MW499720;Lepidoptera;COI;;;;;
MW499736;Lepidoptera;COI;;;;;
MW499740;Lepidoptera;COI;;;;;
MW499760;Lepidoptera;COI;;;;;
MW499771;Lepidoptera;COI;;;;;
MW499774;Lepidoptera;COI;;;;;
MW499774;Lepidoptera;COI;;;;;
MW499784;Lepidoptera;COI;;;;;
MW499800;Lepidoptera;COI;;;;;
MW499810;Lepidoptera;COI;;;;;
MW499845;Lepidoptera;COI;;;;;
MW499850;Lepidoptera;COI;;;;;

MW499866;Lepidoptera;COI;;;;;
MW499887;Lepidoptera;COI;;;;;
MW499889;Lepidoptera;COI;;;;;
MW499912;Lepidoptera;COI;;;;;
MW499916;Lepidoptera;COI;;;;;
MW499926;Lepidoptera;COI;;;;;
MW499929;Lepidoptera;COI;;;;;
MW499943;Lepidoptera;COI;;;;;
MW499978;Lepidoptera;COI;;;;;
MW499983;Lepidoptera;COI;;;;;
MW499987;Lepidoptera;COI;;;;;
MW499987;Lepidoptera;COI;;;;;
MW500021;Lepidoptera;COI;;;;;
MW500021;Lepidoptera;COI;;;;;
MW500027;Lepidoptera;COI;;;;;
MW500047;Lepidoptera;COI;;;;;
MW500052;Lepidoptera;COI;;;;;
MW500055;Lepidoptera;COI;;;;;
MW500065;Lepidoptera;COI;;;;;
MW500090;Lepidoptera;COI;;;;;
MW500090;Lepidoptera;COI;;;;;
MW500109;Lepidoptera;COI;;;;;
MW500113;Lepidoptera;COI;;;;;
MW500129;Lepidoptera;COI;;;;;
MW500140;Lepidoptera;COI;;;;;
MW500154;Lepidoptera;COI;;;;;
MW500166;Lepidoptera;COI;;;;;
MW500166;Lepidoptera;COI;;;;;
MW500185;Lepidoptera;COI;;;;;
MW500198;Lepidoptera;COI;;;;;
MW500199;Lepidoptera;COI;;;;;
MW500201;Lepidoptera;COI;;;;;
MW500206;Lepidoptera;COI;;;;;
MW500211;Lepidoptera;COI;;;;;
MW500212;Lepidoptera;COI;;;;;
MW500214;Lepidoptera;COI;;;;;
MW500220;Lepidoptera;COI;;;;;
MW500220;Lepidoptera;COI;;;;;
MW500233;Lepidoptera;COI;;;;;
MW500238;Lepidoptera;COI;;;;;
MW500282;Lepidoptera;COI;;;;;
MW500300;Lepidoptera;COI;;;;;
MW500300;Lepidoptera;COI;;;;;
MW500323;Lepidoptera;COI;;;;;
MW500323;Lepidoptera;COI;;;;;
MW500326;Lepidoptera;COI;;;;;
MW500332;Lepidoptera;COI;;;;;
MW500350;Lepidoptera;COI;;;;;
MW500357;Lepidoptera;COI;;;;;
MW500358;Lepidoptera;COI;;;;;
MW500361;Lepidoptera;COI;;;;;
MW500378;Lepidoptera;COI;;;;;
MW500381;Lepidoptera;COI;;;;;
MW500381;Lepidoptera;COI;;;;;
MW500383;Lepidoptera;COI;;;;;
MW500394;Lepidoptera;COI;;;;;
MW500400;Lepidoptera;COI;;;;;
MW500414;Lepidoptera;COI;;;;;
MW500423;Lepidoptera;COI;;;;;
MW500438;Lepidoptera;COI;;;;;
MW500471;Lepidoptera;COI;;;;;
MW500481;Lepidoptera;COI;;;;;
MW500486;Lepidoptera;COI;;;;;
MW500494;Lepidoptera;COI;;;;;
MW500497;Lepidoptera;COI;;;;;
MW500516;Lepidoptera;COI;;;;;
MW500528;Lepidoptera;COI;;;;;
MW500531;Lepidoptera;COI;;;;;
MW500544;Lepidoptera;COI;;;;;
MW500556;Lepidoptera;COI;;;;;
MW500569;Lepidoptera;COI;;;;;
MW500575;Lepidoptera;COI;;;;;
MW500580;Lepidoptera;COI;;;;;
MW500582;Lepidoptera;COI;;;;;
MW500587;Lepidoptera;COI;;;;;
MW500587;Lepidoptera;COI;;;;;
MW500594;Lepidoptera;COI;;;;;
MW500595;Lepidoptera;COI;;;;;

MW500613;Lepidoptera;COI;;;;;
MW500617;Lepidoptera;COI;;;;;
MW500623;Lepidoptera;COI;;;;;
MW500672;Lepidoptera;COI;;;;;
MW500694;Lepidoptera;COI;;;;;
MW500694;Lepidoptera;COI;;;;;
MW500702;Lepidoptera;COI;;;;;
MW500702;Lepidoptera;COI;;;;;
MW500719;Lepidoptera;COI;;;;;
MW500728;Lepidoptera;COI;;;;;
MW500738;Lepidoptera;COI;;;;;
MW500748;Lepidoptera;COI;;;;;
MW500748;Lepidoptera;COI;;;;;
MW500753;Lepidoptera;COI;;;;;
MW500766;Lepidoptera;COI;;;;;
MW500767;Lepidoptera;COI;;;;;
MW500773;Lepidoptera;COI;;;;;
MW500783;Lepidoptera;COI;;;;;
MW500783;Lepidoptera;COI;;;;;
MW500801;Lepidoptera;COI;;;;;
MW500807;Lepidoptera;COI;;;;;
MW500811;Lepidoptera;COI;;;;;
MW500814;Lepidoptera;COI;;;;;
MW500818;Lepidoptera;COI;;;;;
MW500827;Lepidoptera;COI;;;;;
MW500847;Lepidoptera;COI;;;;;
MW500851;Lepidoptera;COI;;;;;
MW500852;Lepidoptera;COI;;;;;
MW500860;Lepidoptera;COI;;;;;
MW500901;Lepidoptera;COI;;;;;
MW500939;Lepidoptera;COI;;;;;
MW500943;Lepidoptera;COI;;;;;
MW500948;Lepidoptera;COI;;;;;
MW500953;Lepidoptera;COI;;;;;
MW500957;Lepidoptera;COI;;;;;
MW500965;Lepidoptera;COI;;;;;
MW500984;Lepidoptera;COI;;;;;
MW500990;Lepidoptera;COI;;;;;
MW500994;Lepidoptera;COI;;;;;
MW501015;Lepidoptera;COI;;;;;
MW501015;Lepidoptera;COI;;;;;
MW501071;Lepidoptera;COI;;;;;
MW501094;Lepidoptera;COI;;;;;
MW501126;Lepidoptera;COI;;;;;
MW501126;Lepidoptera;COI;;;;;
MW501134;Lepidoptera;COI;;;;;
MW501138;Lepidoptera;COI;;;;;
MW501145;Lepidoptera;COI;;;;;
MW501149;Lepidoptera;COI;;;;;
MW501194;Lepidoptera;COI;;;;;
MW501228;Lepidoptera;COI;;;;;
MW501230;Lepidoptera;COI;;;;;
MW501233;Lepidoptera;COI;;;;;
MW501237;Lepidoptera;COI;;;;;
MW501243;Lepidoptera;COI;;;;;
MW501248;Lepidoptera;COI;;;;;
MW501277;Lepidoptera;COI;;;;;
MW501279;Lepidoptera;COI;;;;;
MW501280;Lepidoptera;COI;;;;;
MW501312;Lepidoptera;COI;;;;;
MW501320;Lepidoptera;COI;;;;;
MW501326;Lepidoptera;COI;;;;;
MW501330;Lepidoptera;COI;;;;;
MW501338;Lepidoptera;COI;;;;;
MW501357;Lepidoptera;COI;;;;;
MW501357;Lepidoptera;COI;;;;;
MW501390;Lepidoptera;COI;;;;;
MW501390;Lepidoptera;COI;;;;;
MW501391;Lepidoptera;COI;;;;;
MW501393;Lepidoptera;COI;;;;;
MW501410;Lepidoptera;COI;;;;;
MW501413;Lepidoptera;COI;;;;;
MW501419;Lepidoptera;COI;;;;;
MW501430;Lepidoptera;COI;;;;;
MW501434;Lepidoptera;COI;;;;;
MW501435;Lepidoptera;COI;;;;;
MW501456;Lepidoptera;COI;;;;;
MW501456;Lepidoptera;COI;;;;;

MW501457;Lepidoptera;COI;;;;;
MW501492;Lepidoptera;COI;;;;;
MW501506;Lepidoptera;COI;;;;;
MW501532;Lepidoptera;COI;;;;;
MW501559;Lepidoptera;COI;;;;;
MW501562;Lepidoptera;COI;;;;;
MW501604;Lepidoptera;COI;;;;;
MW501606;Lepidoptera;COI;;;;;
MW501633;Lepidoptera;COI;;;;;
MW501642;Lepidoptera;COI;;;;;
MW501645;Lepidoptera;COI;;;;;
MW501655;Lepidoptera;COI;;;;;
MW501658;Lepidoptera;COI;;;;;
MW501665;Lepidoptera;COI;;;;;
MW501688;Lepidoptera;COI;;;;;
MW501688;Lepidoptera;COI;;;;;
MW501690;Lepidoptera;COI;;;;;
MW501695;Lepidoptera;COI;;;;;
MW501717;Lepidoptera;COI;;;;;
MW501718;Lepidoptera;COI;;;;;
MW501730;Lepidoptera;COI;;;;;
MW501730;Lepidoptera;COI;;;;;
MW501757;Lepidoptera;COI;;;;;
MW501757;Lepidoptera;COI;;;;;
MW501770;Lepidoptera;COI;;;;;
MW501792;Lepidoptera;COI;;;;;
MW501794;Lepidoptera;COI;;;;;
MW501803;Lepidoptera;COI;;;;;
MW501807;Lepidoptera;COI;;;;;
MW501810;Lepidoptera;COI;;;;;
MW501821;Lepidoptera;COI;;;;;
MW501821;Lepidoptera;COI;;;;;
MW501868;Lepidoptera;COI;;;;;
MW501872;Lepidoptera;COI;;;;;
MW501875;Lepidoptera;COI;;;;;
MW501910;Lepidoptera;COI;;;;;
MW501946;Lepidoptera;COI;;;;;
MW501947;Lepidoptera;COI;;;;;
MW501949;Lepidoptera;COI;;;;;
MW501953;Lepidoptera;COI;;;;;
MW501961;Lepidoptera;COI;;;;;
MW501961;Lepidoptera;COI;;;;;
MW501980;Lepidoptera;COI;;;;;
MW501987;Lepidoptera;COI;;;;;
MW501994;Lepidoptera;COI;;;;;
MW501997;Lepidoptera;COI;;;;;
MW502000;Lepidoptera;COI;;;;;
MW502000;Lepidoptera;COI;;;;;
MW502002;Lepidoptera;COI;;;;;
MW502013;Lepidoptera;COI;;;;;
MW502013;Lepidoptera;COI;;;;;
MW502014;Lepidoptera;COI;;;;;
MW502014;Lepidoptera;COI;;;;;
MW502022;Lepidoptera;COI;;;;;
MW502028;Lepidoptera;COI;;;;;
MW502040;Lepidoptera;COI;;;;;
MW502042;Lepidoptera;COI;;;;;
MW502081;Lepidoptera;COI;;;;;
MW502089;Lepidoptera;COI;;;;;
MW502117;Lepidoptera;COI;;;;;
MW502124;Lepidoptera;COI;;;;;
MW502131;Lepidoptera;COI;;;;;
MW502163;Lepidoptera;COI;;;;;
MW502163;Lepidoptera;COI;;;;;
MW502177;Lepidoptera;COI;;;;;
MW502184;Lepidoptera;COI;;;;;
MW502202;Lepidoptera;COI;;;;;
MW502208;Lepidoptera;COI;;;;;
MW502211;Lepidoptera;COI;;;;;
MW502211;Lepidoptera;COI;;;;;
MW502219;Lepidoptera;COI;;;;;
MW502223;Lepidoptera;COI;;;;;
MW502228;Lepidoptera;COI;;;;;
MW502241;Lepidoptera;COI;;;;;
MW502251;Lepidoptera;COI;;;;;
MW502251;Lepidoptera;COI;;;;;
MW502257;Lepidoptera;COI;;;;;
MW502277;Lepidoptera;COI;;;;;

MW502281;Lepidoptera;COI;;;;;
MW502282;Lepidoptera;COI;;;;;
MW502294;Lepidoptera;COI;;;;;
MW502299;Lepidoptera;COI;;;;;
MW502304;Lepidoptera;COI;;;;;
MW502306;Lepidoptera;COI;;;;;
MW502308;Lepidoptera;COI;;;;;
MW502317;Lepidoptera;COI;;;;;
MW502322;Lepidoptera;COI;;;;;
MW502328;Lepidoptera;COI;;;;;
MW502352;Lepidoptera;COI;;;;;
MW502352;Lepidoptera;COI;;;;;
MW502355;Lepidoptera;COI;;;;;
MW502358;Lepidoptera;COI;;;;;
MW502362;Lepidoptera;COI;;;;;
MW502365;Lepidoptera;COI;;;;;
MW502365;Lepidoptera;COI;;;;;
MW502376;Lepidoptera;COI;;;;;
MW502387;Lepidoptera;COI;;;;;
MW502405;Lepidoptera;COI;;;;;
MW502441;Lepidoptera;COI;;;;;
MW502443;Lepidoptera;COI;;;;;
MW502495;Lepidoptera;COI;;;;;
MW502495;Lepidoptera;COI;;;;;
MW502517;Lepidoptera;COI;;;;;
MW502527;Lepidoptera;COI;;;;;
MW502539;Lepidoptera;COI;;;;;
MW502541;Lepidoptera;COI;;;;;
MW502541;Lepidoptera;COI;;;;;
MW502545;Lepidoptera;COI;;;;;
MW502545;Lepidoptera;COI;;;;;
MW502546;Lepidoptera;COI;;;;;
MW502560;Lepidoptera;COI;;;;;
MW502560;Lepidoptera;COI;;;;;
MW502567;Lepidoptera;COI;;;;;
MW502577;Lepidoptera;COI;;;;;
MW502585;Lepidoptera;COI;;;;;
MW502601;Lepidoptera;COI;;;;;
MW502608;Lepidoptera;COI;;;;;
MW502612;Lepidoptera;COI;;;;;
MW502614;Lepidoptera;COI;;;;;
MW502628;Lepidoptera;COI;;;;;
MW502636;Lepidoptera;COI;;;;;
MW502640;Lepidoptera;COI;;;;;
MW502640;Lepidoptera;COI;;;;;
MW502654;Lepidoptera;COI;;;;;
MW502659;Lepidoptera;COI;;;;;
MW502661;Lepidoptera;COI;;;;;
MW502664;Lepidoptera;COI;;;;;
MW502702;Lepidoptera;COI;;;;;
MW502713;Lepidoptera;COI;;;;;
MW502717;Lepidoptera;COI;;;;;
MW502718;Lepidoptera;COI;;;;;
MW502722;Lepidoptera;COI;;;;;
MW502728;Lepidoptera;COI;;;;;
MW502734;Lepidoptera;COI;;;;;
MW502735;Lepidoptera;COI;;;;;
MW502759;Lepidoptera;COI;;;;;
MW502776;Lepidoptera;COI;;;;;
MW502785;Lepidoptera;COI;;;;;
MW502789;Lepidoptera;COI;;;;;
MW502805;Lepidoptera;COI;;;;;
MW502807;Lepidoptera;COI;;;;;
MW502818;Lepidoptera;COI;;;;;
MW502818;Lepidoptera;COI;;;;;
MW502823;Lepidoptera;COI;;;;;
MW502829;Lepidoptera;COI;;;;;
MW502842;Lepidoptera;COI;;;;;
MW502854;Lepidoptera;COI;;;;;
MW502857;Lepidoptera;COI;;;;;
MW502868;Lepidoptera;COI;;;;;
MW502882;Lepidoptera;COI;;;;;
MW502882;Lepidoptera;COI;;;;;
MW502914;Lepidoptera;COI;;;;;
MW502937;Lepidoptera;COI;;;;;
MW502957;Lepidoptera;COI;;;;;
MW502979;Lepidoptera;COI;;;;;
MW502999;Lepidoptera;COI;;;;;

MW503004;Lepidoptera;COI;;;;;
MW503007;Lepidoptera;COI;;;;;
MW503010;Lepidoptera;COI;;;;;
MW503021;Lepidoptera;COI;;;;;
MW503043;Lepidoptera;COI;;;;;
MW503049;Lepidoptera;COI;;;;;
MW503062;Lepidoptera;COI;;;;;
MW503066;Lepidoptera;COI;;;;;
MW503066;Lepidoptera;COI;;;;;
MW503076;Lepidoptera;COI;;;;;
MW503081;Lepidoptera;COI;;;;;
MW503082;Lepidoptera;COI;;;;;
MW503089;Lepidoptera;COI;;;;;
MW503091;Lepidoptera;COI;;;;;
MW503092;Lepidoptera;COI;;;;;
MW503102;Lepidoptera;COI;;;;;
MW503107;Lepidoptera;COI;;;;;
MW503107;Lepidoptera;COI;;;;;
MW503112;Lepidoptera;COI;;;;;
MW503120;Lepidoptera;COI;;;;;
MW503144;Lepidoptera;COI;;;;;
MW503154;Lepidoptera;COI;;;;;
MW503170;Lepidoptera;COI;;;;;
MW503170;Lepidoptera;COI;;;;;
MW503179;Lepidoptera;COI;;;;;
MW503189;Lepidoptera;COI;;;;;
MW503195;Lepidoptera;COI;;;;;
MW503201;Lepidoptera;COI;;;;;
MW503201;Lepidoptera;COI;;;;;
MW503202;Lepidoptera;COI;;;;;
MW503202;Lepidoptera;COI;;;;;
MW503228;Lepidoptera;COI;;;;;
MW503228;Lepidoptera;COI;;;;;
MW503237;Lepidoptera;COI;;;;;
MW503259;Lepidoptera;COI;;;;;
MW503288;Lepidoptera;COI;;;;;
MW503292;Lepidoptera;COI;;;;;
MW503303;Lepidoptera;COI;;;;;
MW503309;Lepidoptera;COI;;;;;
MW503322;Lepidoptera;COI;;;;;
MW503333;Lepidoptera;COI;;;;;
MW503334;Lepidoptera;COI;;;;;
MW503338;Lepidoptera;COI;;;;;
MW503358;Lepidoptera;COI;;;;;
MW503358;Lepidoptera;COI;;;;;
MW503360;Lepidoptera;COI;;;;;
MW503378;Lepidoptera;COI;;;;;
MW503408;Lepidoptera;COI;;;;;
MW503411;Lepidoptera;COI;;;;;
MW503411;Lepidoptera;COI;;;;;
MW503420;Lepidoptera;COI;;;;;
MW503422;Lepidoptera;COI;;;;;
MW503430;Lepidoptera;COI;;;;;
MW503437;Lepidoptera;COI;;;;;
MW503438;Lepidoptera;COI;;;;;
MW503457;Lepidoptera;COI;;;;;
MW503457;Lepidoptera;COI;;;;;
MW503460;Lepidoptera;COI;;;;;
MW503460;Lepidoptera;COI;;;;;
MW503469;Lepidoptera;COI;;;;;
MW503473;Lepidoptera;COI;;;;;
MW503485;Lepidoptera;COI;;;;;
MW503497;Lepidoptera;COI;;;;;
MW503502;Lepidoptera;COI;;;;;
MW503536;Lepidoptera;COI;;;;;
MW503540;Lepidoptera;COI;;;;;
MW503541;Lepidoptera;COI;;;;;
MW503549;Lepidoptera;COI;;;;;
MW503567;Lepidoptera;COI;;;;;
MW503567;Lepidoptera;COI;;;;;
MW503572;Lepidoptera;COI;;;;;
MW503576;Lepidoptera;COI;;;;;
MW503576;Lepidoptera;COI;;;;;
MW503582;Lepidoptera;COI;;;;;
MW503600;Lepidoptera;COI;;;;;
MW503610;Lepidoptera;COI;;;;;
MW503613;Lepidoptera;COI;;;;;
MW503613;Lepidoptera;COI;;;;;

MW503617;Lepidoptera;COI;;;;;
MW503619;Lepidoptera;COI;;;;;
MW503619;Lepidoptera;COI;;;;;
MW503658;Lepidoptera;COI;;;;;
MW503689;Lepidoptera;COI;;;;;
MW653337;Lepidoptera;COI;;;;;
MW661286;Lepidoptera;COI;;;;;
MW661287;Lepidoptera;COI;;;;;
MW661291;Lepidoptera;COI;;;;;
MW661292;Lepidoptera;COI;;;;;
MW661299;Lepidoptera;COI;;;;;
MW661306;Lepidoptera;COI;;;;;
MW661320;Lepidoptera;COI;;;;;
MW661321;Lepidoptera;COI;;;;;
MW661323;Lepidoptera;COI;;;;;
MW661324;Lepidoptera;COI;;;;;
MW661325;Lepidoptera;COI;;;;;
MW661326;Lepidoptera;COI;;;;;
MW857550;Lepidoptera;COI;;;;;
MW861644;Lepidoptera;COI;;;;;
MW861646;Lepidoptera;COI;;;;;
MW861660;Lepidoptera;COI;;;;;
MW861661;Lepidoptera;COI;;;;;
MW861665;Lepidoptera;COI;;;;;
MW861666;Lepidoptera;COI;;;;;
MW861667;Lepidoptera;COI;;;;;
MW861670;Lepidoptera;COI;;;;;
MW861671;Lepidoptera;COI;;;;;
MW861672;Lepidoptera;COI;;;;;
MW861673;Lepidoptera;COI;;;;;
MZ021520;Lepidoptera;COI;;;;;
MZ026164;Lepidoptera;COI;;;;;
MZ026165;Lepidoptera;COI;;;;;
MZ026166;Lepidoptera;COI;;;;;
MZ026167;Lepidoptera;COI;;;;;
MZ026168;Lepidoptera;COI;;;;;
MZ026170;Lepidoptera;COI;;;;;
MZ026171;Lepidoptera;COI;;;;;
MZ026174;Lepidoptera;COI;;;;;
MZ026175;Lepidoptera;COI;;;;;
MZ026191;Lepidoptera;COI;;;;;
MZ026194;Lepidoptera;COI;;;;;
MZ026195;Lepidoptera;COI;;;;;
MZ026232;Lepidoptera;COI;;;;;
MZ026233;Lepidoptera;COI;;;;;
MZ026234;Lepidoptera;COI;;;;;
MZ026235;Lepidoptera;COI;;;;;
MZ026237;Lepidoptera;COI;;;;;
MZ026238;Lepidoptera;COI;;;;;
MZ026239;Lepidoptera;COI;;;;;
MZ026240;Lepidoptera;COI;;;;;
MZ026241;Lepidoptera;COI;;;;;
MZ115048;Lepidoptera;COI;;;;;
MZ128126;Lepidoptera;COI;;;;;
MZ128127;Lepidoptera;COI;;;;;
MZ128128;Lepidoptera;COI;;;;;
MZ626727;Lepidoptera;COI;;;;;
OK037119;Lepidoptera;COI;;;;;
OK037120;Lepidoptera;COI;;;;;
OK037121;Lepidoptera;COI;;;;;
OK037122;Lepidoptera;COI;;;;;
OK037123;Lepidoptera;COI;;;;;
OK037124;Lepidoptera;COI;;;;;
OK037125;Lepidoptera;COI;;;;;
OK037126;Lepidoptera;COI;;;;;
OK037127;Lepidoptera;COI;;;;;
OK037128;Lepidoptera;COI;;;;;
OK037129;Lepidoptera;COI;;;;;
OK037130;Lepidoptera;COI;;;;;
OK037131;Lepidoptera;COI;;;;;
OK037132;Lepidoptera;COI;;;;;
OK037133;Lepidoptera;COI;;;;;
OK037134;Lepidoptera;COI;;;;;
OK037135;Lepidoptera;COI;;;;;
OK037136;Lepidoptera;COI;;;;;
OK037137;Lepidoptera;COI;;;;;
OK037138;Lepidoptera;COI;;;;;
OK037139;Lepidoptera;COI;;;;;

OK037140;Lepidoptera;COI;;;;;
ON436062;Lepidoptera;COI;;;;;
ON436530;Lepidoptera;COI;;;;;
ON437242;Lepidoptera;COI;;;;;
ON437244;Lepidoptera;COI;;;;;
OP644313;Lepidoptera;COI;;;;;
OP715732;Lepidoptera;COI;;;;;
OU723912;Lepidoptera;COI;;;;;
OU723913;Lepidoptera;COI;;;;;
OU723914;Lepidoptera;COI;;;;;
OU723915;Lepidoptera;COI;;;;;
OU723916;Lepidoptera;COI;;;;;
OU723917;Lepidoptera;COI;;;;;
OU723918;Lepidoptera;COI;;;;;
OU723919;Lepidoptera;COI;;;;;
OU723920;Lepidoptera;COI;;;;;
OU723921;Lepidoptera;COI;;;;;
OU724030;Lepidoptera;COI;;;;;
OU724031;Lepidoptera;COI;;;;;
OU724032;Lepidoptera;COI;;;;;
OU724033;Lepidoptera;COI;;;;;
OU724034;Lepidoptera;COI;;;;;
AB709130;Odonata;COI;;;;;
AB709133;Odonata;COI;;;;;
AB709136;Odonata;COI;;;;;
AB709138;Odonata;COI;;;;;
AB709142;Odonata;COI;;;;;
AF228584;Odonata;COI;;;;;
AF228591;Odonata;COI;;;;;
AF228592;Odonata;COI;;;;;
AM180646;Odonata;COI;;;;;
AY300810;Odonata;COI;;;;;
AY300811;Odonata;COI;;;;;
AY300812;Odonata;COI;;;;;
AY300813;Odonata;COI;;;;;
AY300815;Odonata;COI;;;;;
AY300816;Odonata;COI;;;;;
GU682174;Odonata;COI;;;;;
GU682176;Odonata;COI;;;;;
HM901859;Odonata;COI;;;;;
HM901865;Odonata;COI;;;;;
HM901887;Odonata;COI;;;;;
HM901888;Odonata;COI;;;;;
HM901892;Odonata;COI;;;;;
HQ830299;Odonata;COI;;;;;
HQ830300;Odonata;COI;;;;;
HQ830301;Odonata;COI;;;;;
HQ830302;Odonata;COI;;;;;
HQ830303;Odonata;COI;;;;;
HQ830304;Odonata;COI;;;;;
HQ830305;Odonata;COI;;;;;
HQ830306;Odonata;COI;;;;;
HQ830307;Odonata;COI;;;;;
HQ830308;Odonata;COI;;;;;
HQ830309;Odonata;COI;;;;;
HQ830310;Odonata;COI;;;;;
HQ830311;Odonata;COI;;;;;
HQ830312;Odonata;COI;;;;;
HQ830313;Odonata;COI;;;;;
HQ830314;Odonata;COI;;;;;
HQ830315;Odonata;COI;;;;;
HQ830316;Odonata;COI;;;;;
HQ830317;Odonata;COI;;;;;
HQ830318;Odonata;COI;;;;;
HQ830319;Odonata;COI;;;;;
HQ830320;Odonata;COI;;;;;
HQ830321;Odonata;COI;;;;;
KF369349;Odonata;COI;;;;;
KF369350;Odonata;COI;;;;;
KF855908;Odonata;COI;;;;;
KU695838;Odonata;COI;;;;;
KU695840;Odonata;COI;;;;;
KU695841;Odonata;COI;;;;;
KU695842;Odonata;COI;;;;;
KU695843;Odonata;COI;;;;;
KU695844;Odonata;COI;;;;;
KU695845;Odonata;COI;;;;;
KY275437;Odonata;COI;;;;;

KY275438;Odonata;COI;;;;;
KY275439;Odonata;COI;;;;;
KY275440;Odonata;COI;;;;;
KY275441;Odonata;COI;;;;;
KY275442;Odonata;COI;;;;;
KY275443;Odonata;COI;;;;;
KY275444;Odonata;COI;;;;;
KY275445;Odonata;COI;;;;;
KY275446;Odonata;COI;;;;;
KY275447;Odonata;COI;;;;;
KY275448;Odonata;COI;;;;;
KY275449;Odonata;COI;;;;;
KY807688;Odonata;COI;;;;;
KY807689;Odonata;COI;;;;;
KY807690;Odonata;COI;;;;;
KY807691;Odonata;COI;;;;;
KY807692;Odonata;COI;;;;;
KY807693;Odonata;COI;;;;;
KY807694;Odonata;COI;;;;;
KY807695;Odonata;COI;;;;;
KY807696;Odonata;COI;;;;;
KY807697;Odonata;COI;;;;;
KY807698;Odonata;COI;;;;;
KY807699;Odonata;COI;;;;;
KY807700;Odonata;COI;;;;;
KY807701;Odonata;COI;;;;;
KY807702;Odonata;COI;;;;;
KY807703;Odonata;COI;;;;;
KY807704;Odonata;COI;;;;;
KY807705;Odonata;COI;;;;;
KY807706;Odonata;COI;;;;;
KY807707;Odonata;COI;;;;;
KY807708;Odonata;COI;;;;;
KY807709;Odonata;COI;;;;;
KY807710;Odonata;COI;;;;;
KY847569;Odonata;COI;;;;;
KY847570;Odonata;COI;;;;;
KY847574;Odonata;COI;;;;;
KY847575;Odonata;COI;;;;;
KY847576;Odonata;COI;;;;;
KY847577;Odonata;COI;;;;;
LC365774;Odonata;COI;;;;;
LC365775;Odonata;COI;;;;;
LC365776;Odonata;COI;;;;;
LC365777;Odonata;COI;;;;;
LC365778;Odonata;COI;;;;;
LC365779;Odonata;COI;;;;;
LC365780;Odonata;COI;;;;;
LC365781;Odonata;COI;;;;;
LC365782;Odonata;COI;;;;;
LC365783;Odonata;COI;;;;;
LC366768;Odonata;COI;;;;;
LC366769;Odonata;COI;;;;;
LC366842;Odonata;COI;;;;;
MH449990;Odonata;COI;;;;;
MN701490;Odonata;COI;;;;;
MN701509;Odonata;COI;;;;;
MN701511;Odonata;COI;;;;;
MN701527;Odonata;COI;;;;;
MN701529;Odonata;COI;;;;;
MN701538;Odonata;COI;;;;;
MN701544;Odonata;COI;;;;;
MN701553;Odonata;COI;;;;;
MN709115;Odonata;COI;;;;;
MN709116;Odonata;COI;;;;;
MN709117;Odonata;COI;;;;;
MN709118;Odonata;COI;;;;;
MN709119;Odonata;COI;;;;;
MN731356;Odonata;COI;;;;;
MN731357;Odonata;COI;;;;;
MN731358;Odonata;COI;;;;;
MN731359;Odonata;COI;;;;;
MN735553;Odonata;COI;;;;;
MN735554;Odonata;COI;;;;;
MT298340;Odonata;COI;;;;;
MT298341;Odonata;COI;;;;;
MT298342;Odonata;COI;;;;;
MT298343;Odonata;COI;;;;;

MT298344;Odonata;COI;;;;;
MT298345;Odonata;COI;;;;;
MT298346;Odonata;COI;;;;;
MT298347;Odonata;COI;;;;;
MT298348;Odonata;COI;;;;;
MT298349;Odonata;COI;;;;;
MT298350;Odonata;COI;;;;;
MT298351;Odonata;COI;;;;;
MT298352;Odonata;COI;;;;;
MT298353;Odonata;COI;;;;;
MT298354;Odonata;COI;;;;;
MT298355;Odonata;COI;;;;;
MT298356;Odonata;COI;;;;;
MT298357;Odonata;COI;;;;;
MT298360;Odonata;COI;;;;;
MT298361;Odonata;COI;;;;;
MT298362;Odonata;COI;;;;;
MT298363;Odonata;COI;;;;;
MT298364;Odonata;COI;;;;;
MT298365;Odonata;COI;;;;;
MT298366;Odonata;COI;;;;;
MT298367;Odonata;COI;;;;;
MT298368;Odonata;COI;;;;;
MT298369;Odonata;COI;;;;;
MT298370;Odonata;COI;;;;;
MT298371;Odonata;COI;;;;;
MT298372;Odonata;COI;;;;;
MT298373;Odonata;COI;;;;;
MT298374;Odonata;COI;;;;;
MT298375;Odonata;COI;;;;;
MT298376;Odonata;COI;;;;;
MT298377;Odonata;COI;;;;;
MT298378;Odonata;COI;;;;;
MT415343;Odonata;COI;;;;;
MW208371;Odonata;COI;;;;;
MW208386;Odonata;COI;;;;;
MW208387;Odonata;COI;;;;;
MW208388;Odonata;COI;;;;;
MW208389;Odonata;COI;;;;;
MW208390;Odonata;COI;;;;;
MW208392;Odonata;COI;;;;;
MW377804;Odonata;COI;;;;;
MW377805;Odonata;COI;;;;;
MW377806;Odonata;COI;;;;;
MW377807;Odonata;COI;;;;;
MW377808;Odonata;COI;;;;;
MW377809;Odonata;COI;;;;;
MW377810;Odonata;COI;;;;;
MW377811;Odonata;COI;;;;;
MW377812;Odonata;COI;;;;;
MW377813;Odonata;COI;;;;;
MW377814;Odonata;COI;;;;;
MW377815;Odonata;COI;;;;;
MW377816;Odonata;COI;;;;;
MW377817;Odonata;COI;;;;;
MW377818;Odonata;COI;;;;;
MW377819;Odonata;COI;;;;;
MW377820;Odonata;COI;;;;;
MW377821;Odonata;COI;;;;;
MW377822;Odonata;COI;;;;;
MW377823;Odonata;COI;;;;;
MW377824;Odonata;COI;;;;;
MW377825;Odonata;COI;;;;;
MW377826;Odonata;COI;;;;;
MW377827;Odonata;COI;;;;;
MW377828;Odonata;COI;;;;;
MW377829;Odonata;COI;;;;;
MW377830;Odonata;COI;;;;;
MW377831;Odonata;COI;;;;;
MW377832;Odonata;COI;;;;;
MW377833;Odonata;COI;;;;;
MW377834;Odonata;COI;;;;;
MW377835;Odonata;COI;;;;;
MW377836;Odonata;COI;;;;;
MW377837;Odonata;COI;;;;;
MW377839;Odonata;COI;;;;;
MW377840;Odonata;COI;;;;;
MW377841;Odonata;COI;;;;;

MW377842;Odonata;COI;;;;;
MW377843;Odonata;COI;;;;;
MW377844;Odonata;COI;;;;;
MW377845;Odonata;COI;;;;;
MW377846;Odonata;COI;;;;;
MW377847;Odonata;COI;;;;;
MW377848;Odonata;COI;;;;;
MW377849;Odonata;COI;;;;;
MW377850;Odonata;COI;;;;;
MW377851;Odonata;COI;;;;;
MW377852;Odonata;COI;;;;;
MW377853;Odonata;COI;;;;;
MW377854;Odonata;COI;;;;;
MW377855;Odonata;COI;;;;;
MW377859;Odonata;COI;;;;;
MW377860;Odonata;COI;;;;;
MW377861;Odonata;COI;;;;;
MW377862;Odonata;COI;;;;;
MW377863;Odonata;COI;;;;;
MW377864;Odonata;COI;;;;;
MW377865;Odonata;COI;;;;;
MW377866;Odonata;COI;;;;;
MW377867;Odonata;COI;;;;;
MW377868;Odonata;COI;;;;;
MW377869;Odonata;COI;;;;;
MW377870;Odonata;COI;;;;;
MW377871;Odonata;COI;;;;;
MW377872;Odonata;COI;;;;;
MW377873;Odonata;COI;;;;;
MW377874;Odonata;COI;;;;;
MW377875;Odonata;COI;;;;;
MW377876;Odonata;COI;;;;;
MW377877;Odonata;COI;;;;;
MW377878;Odonata;COI;;;;;
MW377879;Odonata;COI;;;;;
MW377880;Odonata;COI;;;;;
MW377881;Odonata;COI;;;;;
MW377882;Odonata;COI;;;;;
MW377883;Odonata;COI;;;;;
MW377884;Odonata;COI;;;;;
MW377885;Odonata;COI;;;;;
MW377886;Odonata;COI;;;;;
MW377887;Odonata;COI;;;;;
MW377888;Odonata;COI;;;;;
MW377889;Odonata;COI;;;;;
MW377890;Odonata;COI;;;;;
MW490099;Odonata;COI;;;;;
MW490107;Odonata;COI;;;;;
MW490111;Odonata;COI;;;;;
MW490114;Odonata;COI;;;;;
MW490120;Odonata;COI;;;;;
MW490123;Odonata;COI;;;;;
MW490139;Odonata;COI;;;;;
MW490149;Odonata;COI;;;;;
MW490159;Odonata;COI;;;;;
MW490165;Odonata;COI;;;;;
MW490176;Odonata;COI;;;;;
MW490181;Odonata;COI;;;;;
MW490183;Odonata;COI;;;;;
MW490184;Odonata;COI;;;;;
MW490198;Odonata;COI;;;;;
MW490204;Odonata;COI;;;;;
MW490206;Odonata;COI;;;;;
MW490214;Odonata;COI;;;;;
MW490219;Odonata;COI;;;;;
MW490226;Odonata;COI;;;;;
MW490233;Odonata;COI;;;;;
MW490240;Odonata;COI;;;;;
MW490247;Odonata;COI;;;;;
MW490269;Odonata;COI;;;;;
MW490279;Odonata;COI;;;;;
MW490282;Odonata;COI;;;;;
MW490289;Odonata;COI;;;;;
MW490306;Odonata;COI;;;;;
MW490307;Odonata;COI;;;;;
MW490329;Odonata;COI;;;;;
MW490336;Odonata;COI;;;;;
MW490340;Odonata;COI;;;;;

MW490347;Odonata;COI;;;;;
MW490363;Odonata;COI;;;;;
MW490381;Odonata;COI;;;;;
MW490397;Odonata;COI;;;;;
MW490406;Odonata;COI;;;;;
MW490436;Odonata;COI;;;;;
MW490437;Odonata;COI;;;;;
MW490440;Odonata;COI;;;;;
MW490442;Odonata;COI;;;;;
MW490446;Odonata;COI;;;;;
MW490447;Odonata;COI;;;;;
MW490454;Odonata;COI;;;;;
MW490467;Odonata;COI;;;;;
MW490484;Odonata;COI;;;;;
MW490488;Odonata;COI;;;;;
MW490493;Odonata;COI;;;;;
MW490498;Odonata;COI;;;;;
MW490524;Odonata;COI;;;;;
MW490540;Odonata;COI;;;;;
MW490553;Odonata;COI;;;;;
MW490562;Odonata;COI;;;;;
MW509059;Odonata;COI;;;;;
MW509060;Odonata;COI;;;;;
MW509061;Odonata;COI;;;;;
MZ508997;Odonata;COI;;;;;
MZ508998;Odonata;COI;;;;;
MZ508999;Odonata;COI;;;;;
MZ509000;Odonata;COI;;;;;
MZ509001;Odonata;COI;;;;;
MZ657068;Odonata;COI;;;;;
MZ657345;Odonata;COI;;;;;
MZ659561;Odonata;COI;;;;;
MZ660534;Odonata;COI;;;;;
MZ660783;Odonata;COI;;;;;
MZ893271;Odonata;COI;;;;;
MZ893272;Odonata;COI;;;;;
MZ893273;Odonata;COI;;;;;
MZ893274;Odonata;COI;;;;;
MZ893275;Odonata;COI;;;;;
MZ893276;Odonata;COI;;;;;
MZ893277;Odonata;COI;;;;;
MZ893278;Odonata;COI;;;;;
MZ893286;Odonata;COI;;;;;
MZ893287;Odonata;COI;;;;;
MZ893288;Odonata;COI;;;;;
MZ893289;Odonata;COI;;;;;
MZ893290;Odonata;COI;;;;;
MZ893291;Odonata;COI;;;;;
MZ893292;Odonata;COI;;;;;
MZ893298;Odonata;COI;;;;;
MZ893299;Odonata;COI;;;;;
MZ893300;Odonata;COI;;;;;
MZ893301;Odonata;COI;;;;;
MZ893302;Odonata;COI;;;;;
MZ893303;Odonata;COI;;;;;
MZ893304;Odonata;COI;;;;;
MZ893305;Odonata;COI;;;;;
MZ893306;Odonata;COI;;;;;
MZ893307;Odonata;COI;;;;;
MZ893308;Odonata;COI;;;;;
MZ893309;Odonata;COI;;;;;
MZ893310;Odonata;COI;;;;;
MZ893311;Odonata;COI;;;;;
MZ893312;Odonata;COI;;;;;
MZ893313;Odonata;COI;;;;;
MZ893314;Odonata;COI;;;;;
MZ893315;Odonata;COI;;;;;
MZ893316;Odonata;COI;;;;;
MZ893317;Odonata;COI;;;;;
MZ893318;Odonata;COI;;;;;
MZ893319;Odonata;COI;;;;;
MZ893320;Odonata;COI;;;;;
MZ893321;Odonata;COI;;;;;
MZ893324;Odonata;COI;;;;;
MZ893325;Odonata;COI;;;;;
MZ893326;Odonata;COI;;;;;
MZ893327;Odonata;COI;;;;;
MZ893328;Odonata;COI;;;;;

MZ893329;Odonata;COI;;;;;
MZ893330;Odonata;COI;;;;;
MZ893331;Odonata;COI;;;;;
OM100714;Odonata;COI;;;;;
OM100716;Odonata;COI;;;;;
OM100717;Odonata;COI;;;;;
ON000165;Odonata;COI;;;;;
ON000166;Odonata;COI;;;;;
ON000167;Odonata;COI;;;;;
ON000168;Odonata;COI;;;;;
ON000169;Odonata;COI;;;;;
ON000170;Odonata;COI;;;;;
ON000171;Odonata;COI;;;;;
AB609581;Coleoptera;COI;;;;;
AB609582;Coleoptera;COI;;;;;
AB609583;Coleoptera;COI;;;;;
AF296554;Coleoptera;COI;;;;;
AF296555;Coleoptera;COI;;;;;
AF296556;Coleoptera;COI;;;;;
AF296557;Coleoptera;COI;;;;;
AF457789;Coleoptera;COI;;;;;
AF457790;Coleoptera;COI;;;;;
AF457791;Coleoptera;COI;;;;;
AF457792;Coleoptera;COI;;;;;
AF457793;Coleoptera;COI;;;;;
AF457794;Coleoptera;COI;;;;;
AF457795;Coleoptera;COI;;;;;
AF457796;Coleoptera;COI;;;;;
AF457797;Coleoptera;COI;;;;;
AF457798;Coleoptera;COI;;;;;
AF457799;Coleoptera;COI;;;;;
AF457800;Coleoptera;COI;;;;;
AF457801;Coleoptera;COI;;;;;
AF457802;Coleoptera;COI;;;;;
AF457803;Coleoptera;COI;;;;;
AF457804;Coleoptera;COI;;;;;
AF457805;Coleoptera;COI;;;;;
AF457806;Coleoptera;COI;;;;;
AF457807;Coleoptera;COI;;;;;
AF457808;Coleoptera;COI;;;;;
AF457809;Coleoptera;COI;;;;;
AF457810;Coleoptera;COI;;;;;
AF457811;Coleoptera;COI;;;;;
AF457812;Coleoptera;COI;;;;;
AF457813;Coleoptera;COI;;;;;
AF457814;Coleoptera;COI;;;;;
AF457815;Coleoptera;COI;;;;;
AF457816;Coleoptera;COI;;;;;
AF457817;Coleoptera;COI;;;;;
AF457818;Coleoptera;COI;;;;;
AF457819;Coleoptera;COI;;;;;
AF457820;Coleoptera;COI;;;;;
AF457821;Coleoptera;COI;;;;;
AF457822;Coleoptera;COI;;;;;
AF457823;Coleoptera;COI;;;;;
AF457824;Coleoptera;COI;;;;;
AF457825;Coleoptera;COI;;;;;
AF457826;Coleoptera;COI;;;;;
AF457827;Coleoptera;COI;;;;;
AF457828;Coleoptera;COI;;;;;
AF457829;Coleoptera;COI;;;;;
AF457830;Coleoptera;COI;;;;;
AF457831;Coleoptera;COI;;;;;
AF457832;Coleoptera;COI;;;;;
AF457833;Coleoptera;COI;;;;;
AF457834;Coleoptera;COI;;;;;
AF457835;Coleoptera;COI;;;;;
AF457836;Coleoptera;COI;;;;;
AF457837;Coleoptera;COI;;;;;
AF457838;Coleoptera;COI;;;;;
AF457839;Coleoptera;COI;;;;;
AF457840;Coleoptera;COI;;;;;
AF457841;Coleoptera;COI;;;;;
AF457842;Coleoptera;COI;;;;;
AF457843;Coleoptera;COI;;;;;
AF457844;Coleoptera;COI;;;;;
AF457845;Coleoptera;COI;;;;;
AF457846;Coleoptera;COI;;;;;

AF457847;Coleoptera;COI;;;;;
AF457848;Coleoptera;COI;;;;;
AF457849;Coleoptera;COI;;;;;
AF457850;Coleoptera;COI;;;;;
AF457851;Coleoptera;COI;;;;;
AF457852;Coleoptera;COI;;;;;
AF457853;Coleoptera;COI;;;;;
AF457854;Coleoptera;COI;;;;;
AF457855;Coleoptera;COI;;;;;
AF457856;Coleoptera;COI;;;;;
AF457857;Coleoptera;COI;;;;;
AF457858;Coleoptera;COI;;;;;
AF457859;Coleoptera;COI;;;;;
AF457860;Coleoptera;COI;;;;;
AF457861;Coleoptera;COI;;;;;
AF457862;Coleoptera;COI;;;;;
AF457863;Coleoptera;COI;;;;;
AF457864;Coleoptera;COI;;;;;
AF457865;Coleoptera;COI;;;;;
AF457866;Coleoptera;COI;;;;;
AF457867;Coleoptera;COI;;;;;
AJ884601;Coleoptera;COI;;;;;
AJ884602;Coleoptera;COI;;;;;
AJ884603;Coleoptera;COI;;;;;
AJ884604;Coleoptera;COI;;;;;
AJ884607;Coleoptera;COI;;;;;
AJ884608;Coleoptera;COI;;;;;
AM283248;Coleoptera;COI;;;;;
AM283249;Coleoptera;COI;;;;;
AM283252;Coleoptera;COI;;;;;
AM423156;Coleoptera;COI;;;;;
AM423157;Coleoptera;COI;;;;;
AY260835;Coleoptera;COI;;;;;
AY260836;Coleoptera;COI;;;;;
AY260837;Coleoptera;COI;;;;;
AY260838;Coleoptera;COI;;;;;
AY260839;Coleoptera;COI;;;;;
AY260840;Coleoptera;COI;;;;;
AY260841;Coleoptera;COI;;;;;
AY260843;Coleoptera;COI;;;;;
AY260844;Coleoptera;COI;;;;;
AY264403;Coleoptera;COI;;;;;
AY389469;Coleoptera;COI;;;;;
AY389470;Coleoptera;COI;;;;;
AY389478;Coleoptera;COI;;;;;
AY389479;Coleoptera;COI;;;;;
AY389480;Coleoptera;COI;;;;;
AY389481;Coleoptera;COI;;;;;
AY389482;Coleoptera;COI;;;;;
AY389483;Coleoptera;COI;;;;;
AY796318;Coleoptera;COI;;;;;
AY796319;Coleoptera;COI;;;;;
AY796320;Coleoptera;COI;;;;;
AY796321;Coleoptera;COI;;;;;
AY796322;Coleoptera;COI;;;;;
AY796323;Coleoptera;COI;;;;;
AY796324;Coleoptera;COI;;;;;
AY796325;Coleoptera;COI;;;;;
AY796326;Coleoptera;COI;;;;;
AY796327;Coleoptera;COI;;;;;
AY796328;Coleoptera;COI;;;;;
AY796329;Coleoptera;COI;;;;;
AY796330;Coleoptera;COI;;;;;
AY796331;Coleoptera;COI;;;;;
AY796332;Coleoptera;COI;;;;;
DQ181217;Coleoptera;COI;;;;;
DQ181290;Coleoptera;COI;;;;;
DQ198545;Coleoptera;COI;;;;;
DQ198568;Coleoptera;COI;;;;;
DQ198573;Coleoptera;COI;;;;;
DQ198576;Coleoptera;COI;;;;;
DQ198589;Coleoptera;COI;;;;;
EF490176;Coleoptera;COI;;;;;
EF490189;Coleoptera;COI;;;;;
EF508056;Coleoptera;COI;;;;;
EF508059;Coleoptera;COI;;;;;
EF589370;Coleoptera;COI;;;;;
EF589372;Coleoptera;COI;;;;;

EF589379;Coleoptera;COI;;;;;
EU011803;Coleoptera;COI;;;;;
EU011804;Coleoptera;COI;;;;;
EU011805;Coleoptera;COI;;;;;
EU011806;Coleoptera;COI;;;;;
EU011814;Coleoptera;COI;;;;;
EU286446;Coleoptera;COI;;;;;
EU286446;Coleoptera;COI;;;;;
EU286447;Coleoptera;COI;;;;;
EU286448;Coleoptera;COI;;;;;
EU286449;Coleoptera;COI;;;;;
EU286451;Coleoptera;COI;;;;;
EU286453;Coleoptera;COI;;;;;
EU286493;Coleoptera;COI;;;;;
EU286507;Coleoptera;COI;;;;;
EU286510;Coleoptera;COI;;;;;
EU286511;Coleoptera;COI;;;;;
EU286512;Coleoptera;COI;;;;;
EU883935;Coleoptera;COI;;;;;
EU883936;Coleoptera;COI;;;;;
EU883937;Coleoptera;COI;;;;;
FJ868695;Coleoptera;COI;;;;;
FJ903955;Coleoptera;COI;;;;;
FJ903956;Coleoptera;COI;;;;;
FJ903957;Coleoptera;COI;;;;;
FJ903961;Coleoptera;COI;;;;;
FJ903965;Coleoptera;COI;;;;;
FJ903981;Coleoptera;COI;;;;;
FJ904002;Coleoptera;COI;;;;;
FJ904003;Coleoptera;COI;;;;;
FJ904013;Coleoptera;COI;;;;;
FJ904038;Coleoptera;COI;;;;;
FJ904039;Coleoptera;COI;;;;;
FJ904041;Coleoptera;COI;;;;;
FJ904042;Coleoptera;COI;;;;;
FJ904043;Coleoptera;COI;;;;;
FJ904044;Coleoptera;COI;;;;;
FJ904048;Coleoptera;COI;;;;;
FJ904053;Coleoptera;COI;;;;;
FJ904054;Coleoptera;COI;;;;;
FJ904055;Coleoptera;COI;;;;;
FJ904056;Coleoptera;COI;;;;;
FJ904057;Coleoptera;COI;;;;;
FJ904059;Coleoptera;COI;;;;;
FJ904065;Coleoptera;COI;;;;;
FJ904067;Coleoptera;COI;;;;;
FJ904069;Coleoptera;COI;;;;;
FJ904080;Coleoptera;COI;;;;;
FJ984570;Coleoptera;COI;;;;;
FJ984571;Coleoptera;COI;;;;;
FJ984572;Coleoptera;COI;;;;;
FJ984573;Coleoptera;COI;;;;;
FJ984574;Coleoptera;COI;;;;;
FM877788;Coleoptera;COI;;;;;
FM877789;Coleoptera;COI;;;;;
FM877790;Coleoptera;COI;;;;;
FM877791;Coleoptera;COI;;;;;
FM877792;Coleoptera;COI;;;;;
FM877795;Coleoptera;COI;;;;;
FM877799;Coleoptera;COI;;;;;
FM877800;Coleoptera;COI;;;;;
FM877802;Coleoptera;COI;;;;;
FM877906;Coleoptera;COI;;;;;
GQ980904;Coleoptera;COI;;;;;
GQ980905;Coleoptera;COI;;;;;
GQ980953;Coleoptera;COI;;;;;
GQ980963;Coleoptera;COI;;;;;
GU003930;Coleoptera;COI;;;;;
GU003934;Coleoptera;COI;;;;;
GU003935;Coleoptera;COI;;;;;
GU981486;Coleoptera;COI;;;;;
GU981501;Coleoptera;COI;;;;;
GU987753;Coleoptera;COI;;;;;
GU987754;Coleoptera;COI;;;;;
GU987755;Coleoptera;COI;;;;;
GU987755;Coleoptera;COI;;;;;
GU987756;Coleoptera;COI;;;;;
GU987758;Coleoptera;COI;;;;;

GU987764;Coleoptera;COI;;;;;
GU987767;Coleoptera;COI;;;;;
GU987770;Coleoptera;COI;;;;;
GU987771;Coleoptera;COI;;;;;
GU987771;Coleoptera;COI;;;;;
GU987772;Coleoptera;COI;;;;;
GU987772;Coleoptera;COI;;;;;
GU987776;Coleoptera;COI;;;;;
GU987777;Coleoptera;COI;;;;;
GU987786;Coleoptera;COI;;;;;
GU987806;Coleoptera;COI;;;;;
GU987808;Coleoptera;COI;;;;;
GU987809;Coleoptera;COI;;;;;
GU987809;Coleoptera;COI;;;;;
GU987810;Coleoptera;COI;;;;;
GU987813;Coleoptera;COI;;;;;
GU987814;Coleoptera;COI;;;;;
GU987816;Coleoptera;COI;;;;;
GU987842;Coleoptera;COI;;;;;
GU987843;Coleoptera;COI;;;;;
GU987845;Coleoptera;COI;;;;;
GU987846;Coleoptera;COI;;;;;
GU987849;Coleoptera;COI;;;;;
GU987850;Coleoptera;COI;;;;;
GU987851;Coleoptera;COI;;;;;
GU987855;Coleoptera;COI;;;;;
GU987870;Coleoptera;COI;;;;;
GU987871;Coleoptera;COI;;;;;
GU987872;Coleoptera;COI;;;;;
GU987873;Coleoptera;COI;;;;;
GU987878;Coleoptera;COI;;;;;
GU987879;Coleoptera;COI;;;;;
GU987880;Coleoptera;COI;;;;;
GU987880;Coleoptera;COI;;;;;
GU987881;Coleoptera;COI;;;;;
GU987882;Coleoptera;COI;;;;;
GU987892;Coleoptera;COI;;;;;
GU987895;Coleoptera;COI;;;;;
GU987896;Coleoptera;COI;;;;;
GU987896;Coleoptera;COI;;;;;
GU987897;Coleoptera;COI;;;;;
GU987898;Coleoptera;COI;;;;;
GU987966;Coleoptera;COI;;;;;
GU987967;Coleoptera;COI;;;;;
GU987970;Coleoptera;COI;;;;;
GU987970;Coleoptera;COI;;;;;
GU987971;Coleoptera;COI;;;;;
GU987972;Coleoptera;COI;;;;;
GU987976;Coleoptera;COI;;;;;
GU987976;Coleoptera;COI;;;;;
GU987977;Coleoptera;COI;;;;;
GU987978;Coleoptera;COI;;;;;
GU987979;Coleoptera;COI;;;;;
GU987981;Coleoptera;COI;;;;;
GU987981;Coleoptera;COI;;;;;
GU987983;Coleoptera;COI;;;;;
GU987988;Coleoptera;COI;;;;;
GU987988;Coleoptera;COI;;;;;
GU987989;Coleoptera;COI;;;;;
GU988028;Coleoptera;COI;;;;;
GU988029;Coleoptera;COI;;;;;
GU988030;Coleoptera;COI;;;;;
GU988031;Coleoptera;COI;;;;;
GU988032;Coleoptera;COI;;;;;
GU988033;Coleoptera;COI;;;;;
GU988034;Coleoptera;COI;;;;;
GU988041;Coleoptera;COI;;;;;
GU988042;Coleoptera;COI;;;;;
GU988042;Coleoptera;COI;;;;;
GU988044;Coleoptera;COI;;;;;
GU988046;Coleoptera;COI;;;;;
GU988080;Coleoptera;COI;;;;;
GU988090;Coleoptera;COI;;;;;
GU988090;Coleoptera;COI;;;;;
GU988151;Coleoptera;COI;;;;;
GU988156;Coleoptera;COI;;;;;
GU988157;Coleoptera;COI;;;;;
HM417705;Coleoptera;COI;;;;;

HM417712;Coleoptera;COI;;;;;
HM909126;Coleoptera;COI;;;;;
HQ333938;Coleoptera;COI;;;;;
HQ333944;Coleoptera;COI;;;;;
HQ333946;Coleoptera;COI;;;;;
HQ333973;Coleoptera;COI;;;;;
HQ334002;Coleoptera;COI;;;;;
HQ619570;Coleoptera;COI;;;;;
HQ619571;Coleoptera;COI;;;;;
HQ619599;Coleoptera;COI;;;;;
HQ619602;Coleoptera;COI;;;;;
HQ619604;Coleoptera;COI;;;;;
HQ619608;Coleoptera;COI;;;;;
HQ727683;Coleoptera;COI;;;;;
HQ727684;Coleoptera;COI;;;;;
HQ727685;Coleoptera;COI;;;;;
HQ727686;Coleoptera;COI;;;;;
HQ727687;Coleoptera;COI;;;;;
HQ727688;Coleoptera;COI;;;;;
HQ953403;Coleoptera;COI;;;;;
HQ953403;Coleoptera;COI;;;;;
HQ953486;Coleoptera;COI;;;;;
HQ953586;Coleoptera;COI;;;;;
HQ954209;Coleoptera;COI;;;;;
HQ954210;Coleoptera;COI;;;;;
HQ954260;Coleoptera;COI;;;;;
HQ954270;Coleoptera;COI;;;;;
JF889494;Coleoptera;COI;;;;;
JF889507;Coleoptera;COI;;;;;
JN299278;Coleoptera;COI;;;;;
JN676037;Coleoptera;COI;;;;;
JN676038;Coleoptera;COI;;;;;
JN676039;Coleoptera;COI;;;;;
JN676040;Coleoptera;COI;;;;;
JN676041;Coleoptera;COI;;;;;
JN676042;Coleoptera;COI;;;;;
JN676043;Coleoptera;COI;;;;;
JN676044;Coleoptera;COI;;;;;
JN676045;Coleoptera;COI;;;;;
JN676046;Coleoptera;COI;;;;;
JN676047;Coleoptera;COI;;;;;
JN676048;Coleoptera;COI;;;;;
JN676049;Coleoptera;COI;;;;;
JN676050;Coleoptera;COI;;;;;
JN676051;Coleoptera;COI;;;;;
JX263829;Coleoptera;COI;;;;;
JX263830;Coleoptera;COI;;;;;
JX263836;Coleoptera;COI;;;;;
JX263846;Coleoptera;COI;;;;;
JX263866;Coleoptera;COI;;;;;
JX626111;Coleoptera;COI;;;;;
KC132741;Coleoptera;COI;;;;;
KC160266;Coleoptera;COI;;;;;
KC160267;Coleoptera;COI;;;;;
KC160268;Coleoptera;COI;;;;;
KC524624;Coleoptera;COI;;;;;
KC593342;Coleoptera;COI;;;;;
KC593343;Coleoptera;COI;;;;;
KC593344;Coleoptera;COI;;;;;
KC758809;Coleoptera;COI;;;;;
KC758811;Coleoptera;COI;;;;;
KC758813;Coleoptera;COI;;;;;
KC758816;Coleoptera;COI;;;;;
KC758824;Coleoptera;COI;;;;;
KC758842;Coleoptera;COI;;;;;
KF625259;Coleoptera;COI;;;;;
KF625271;Coleoptera;COI;;;;;
KF625386;Coleoptera;COI;;;;;
KF625387;Coleoptera;COI;;;;;
KF625407;Coleoptera;COI;;;;;
KF625467;Coleoptera;COI;;;;;
KF680227;Coleoptera;COI;;;;;
KF680229;Coleoptera;COI;;;;;
KF680230;Coleoptera;COI;;;;;
KF680231;Coleoptera;COI;;;;;
KF680232;Coleoptera;COI;;;;;
KF680233;Coleoptera;COI;;;;;
KF680234;Coleoptera;COI;;;;;

KF680235;Coleoptera;COI;;;;;
KF680236;Coleoptera;COI;;;;;
KF680237;Coleoptera;COI;;;;;
KF680241;Coleoptera;COI;;;;;
KF680257;Coleoptera;COI;;;;;
KF737071;Coleoptera;COI;;;;;
KF737072;Coleoptera;COI;;;;;
KF737073;Coleoptera;COI;;;;;
KF737074;Coleoptera;COI;;;;;
KF737075;Coleoptera;COI;;;;;
KF737076;Coleoptera;COI;;;;;
KF737077;Coleoptera;COI;;;;;
KF737078;Coleoptera;COI;;;;;
KF737079;Coleoptera;COI;;;;;
KF737080;Coleoptera;COI;;;;;
KF737081;Coleoptera;COI;;;;;
KF737082;Coleoptera;COI;;;;;
KF737083;Coleoptera;COI;;;;;
KF737084;Coleoptera;COI;;;;;
KF737085;Coleoptera;COI;;;;;
KF737086;Coleoptera;COI;;;;;
KF737087;Coleoptera;COI;;;;;
KF737088;Coleoptera;COI;;;;;
KF737089;Coleoptera;COI;;;;;
KF737090;Coleoptera;COI;;;;;
KF737091;Coleoptera;COI;;;;;
KF737092;Coleoptera;COI;;;;;
KF737093;Coleoptera;COI;;;;;
KF737094;Coleoptera;COI;;;;;
KF737096;Coleoptera;COI;;;;;
KF737097;Coleoptera;COI;;;;;
KF737098;Coleoptera;COI;;;;;
KF737099;Coleoptera;COI;;;;;
KF737100;Coleoptera;COI;;;;;
KF737101;Coleoptera;COI;;;;;
KF737102;Coleoptera;COI;;;;;
KF737103;Coleoptera;COI;;;;;
KF737104;Coleoptera;COI;;;;;
KF737105;Coleoptera;COI;;;;;
KF737106;Coleoptera;COI;;;;;
KF737107;Coleoptera;COI;;;;;
KF737108;Coleoptera;COI;;;;;
KF737109;Coleoptera;COI;;;;;
KF737111;Coleoptera;COI;;;;;
KF737111;Coleoptera;COI;;;;;
KF737112;Coleoptera;COI;;;;;
KF737119;Coleoptera;COI;;;;;
KF737120;Coleoptera;COI;;;;;
KF737121;Coleoptera;COI;;;;;
KF737125;Coleoptera;COI;;;;;
KF737126;Coleoptera;COI;;;;;
KF737127;Coleoptera;COI;;;;;
KF737128;Coleoptera;COI;;;;;
KF737129;Coleoptera;COI;;;;;
KF737130;Coleoptera;COI;;;;;
KF737131;Coleoptera;COI;;;;;
KF737132;Coleoptera;COI;;;;;
KF737133;Coleoptera;COI;;;;;
KJ867571;Coleoptera;COI;;;;;
KJ961815;Coleoptera;COI;;;;;
KJ962265;Coleoptera;COI;;;;;
KJ962306;Coleoptera;COI;;;;;
KJ962490;Coleoptera;COI;;;;;
KJ962569;Coleoptera;COI;;;;;
KJ963055;Coleoptera;COI;;;;;
KJ963200;Coleoptera;COI;;;;;
KJ963477;Coleoptera;COI;;;;;
KJ963506;Coleoptera;COI;;;;;
KJ963622;Coleoptera;COI;;;;;
KJ963907;Coleoptera;COI;;;;;
KJ963981;Coleoptera;COI;;;;;
KJ964069;Coleoptera;COI;;;;;
KJ964213;Coleoptera;COI;;;;;
KJ964357;Coleoptera;COI;;;;;
KJ964482;Coleoptera;COI;;;;;
KJ964551;Coleoptera;COI;;;;;
KJ964578;Coleoptera;COI;;;;;
KJ964683;Coleoptera;COI;;;;;

KJ964792;Coleoptera;COI;;;;;
KJ964805;Coleoptera;COI;;;;;
KJ964900;Coleoptera;COI;;;;;
KJ964934;Coleoptera;COI;;;;;
KJ965043;Coleoptera;COI;;;;;
KJ965102;Coleoptera;COI;;;;;
KJ965130;Coleoptera;COI;;;;;
KJ965259;Coleoptera;COI;;;;;
KJ965629;Coleoptera;COI;;;;;
KJ965642;Coleoptera;COI;;;;;
KJ965661;Coleoptera;COI;;;;;
KJ965930;Coleoptera;COI;;;;;
KJ966019;Coleoptera;COI;;;;;
KJ966266;Coleoptera;COI;;;;;
KJ966621;Coleoptera;COI;;;;;
KJ966648;Coleoptera;COI;;;;;
KJ966656;Coleoptera;COI;;;;;
KJ966881;Coleoptera;COI;;;;;
KJ967076;Coleoptera;COI;;;;;
KJ967152;Coleoptera;COI;;;;;
KJ967219;Coleoptera;COI;;;;;
KJ967263;Coleoptera;COI;;;;;
KJ967420;Coleoptera;COI;;;;;
KJ967511;Coleoptera;COI;;;;;
KM285762;Coleoptera;COI;;;;;
KM285773;Coleoptera;COI;;;;;
KM285812;Coleoptera;COI;;;;;
KM285823;Coleoptera;COI;;;;;
KM285833;Coleoptera;COI;;;;;
KM285839;Coleoptera;COI;;;;;
KM285918;Coleoptera;COI;;;;;
KM285919;Coleoptera;COI;;;;;
KM285954;Coleoptera;COI;;;;;
KM285956;Coleoptera;COI;;;;;
KM285970;Coleoptera;COI;;;;;
KM285988;Coleoptera;COI;;;;;
KM286015;Coleoptera;COI;;;;;
KM286021;Coleoptera;COI;;;;;
KM286036;Coleoptera;COI;;;;;
KM286062;Coleoptera;COI;;;;;
KM286075;Coleoptera;COI;;;;;
KM286086;Coleoptera;COI;;;;;
KM286109;Coleoptera;COI;;;;;
KM286159;Coleoptera;COI;;;;;
KM286203;Coleoptera;COI;;;;;
KM286218;Coleoptera;COI;;;;;
KM286219;Coleoptera;COI;;;;;
KM286252;Coleoptera;COI;;;;;
KM286289;Coleoptera;COI;;;;;
KM286291;Coleoptera;COI;;;;;
KM286310;Coleoptera;COI;;;;;
KM286346;Coleoptera;COI;;;;;
KM286352;Coleoptera;COI;;;;;
KM286353;Coleoptera;COI;;;;;
KM286365;Coleoptera;COI;;;;;
KM286371;Coleoptera;COI;;;;;
KM286401;Coleoptera;COI;;;;;
KM439142;Coleoptera;COI;;;;;
KM439183;Coleoptera;COI;;;;;
KM439224;Coleoptera;COI;;;;;
KM439252;Coleoptera;COI;;;;;
KM439268;Coleoptera;COI;;;;;
KM439442;Coleoptera;COI;;;;;
KM439634;Coleoptera;COI;;;;;
KM439806;Coleoptera;COI;;;;;
KM439943;Coleoptera;COI;;;;;
KM440126;Coleoptera;COI;;;;;
KM440239;Coleoptera;COI;;;;;
KM440295;Coleoptera;COI;;;;;
KM440303;Coleoptera;COI;;;;;
KM440385;Coleoptera;COI;;;;;
KM440742;Coleoptera;COI;;;;;
KM440775;Coleoptera;COI;;;;;
KM440930;Coleoptera;COI;;;;;
KM441117;Coleoptera;COI;;;;;
KM441210;Coleoptera;COI;;;;;
KM441211;Coleoptera;COI;;;;;
KM441574;Coleoptera;COI;;;;;

KM441747;Coleoptera;COI;;;;;
KM441833;Coleoptera;COI;;;;;
KM441833;Coleoptera;COI;;;;;
KM442322;Coleoptera;COI;;;;;
KM443041;Coleoptera;COI;;;;;
KM443070;Coleoptera;COI;;;;;
KM443128;Coleoptera;COI;;;;;
KM443374;Coleoptera;COI;;;;;
KM443481;Coleoptera;COI;;;;;
KM443546;Coleoptera;COI;;;;;
KM443724;Coleoptera;COI;;;;;
KM444049;Coleoptera;COI;;;;;
KM444094;Coleoptera;COI;;;;;
KM444138;Coleoptera;COI;;;;;
KM444190;Coleoptera;COI;;;;;
KM444338;Coleoptera;COI;;;;;
KM444338;Coleoptera;COI;;;;;
KM444452;Coleoptera;COI;;;;;
KM444513;Coleoptera;COI;;;;;
KM444576;Coleoptera;COI;;;;;
KM444613;Coleoptera;COI;;;;;
KM444799;Coleoptera;COI;;;;;
KM445093;Coleoptera;COI;;;;;
KM445093;Coleoptera;COI;;;;;
KM445130;Coleoptera;COI;;;;;
KM445202;Coleoptera;COI;;;;;
KM446024;Coleoptera;COI;;;;;
KM446311;Coleoptera;COI;;;;;
KM446511;Coleoptera;COI;;;;;
KM446591;Coleoptera;COI;;;;;
KM446785;Coleoptera;COI;;;;;
KM446851;Coleoptera;COI;;;;;
KM446871;Coleoptera;COI;;;;;
KM447067;Coleoptera;COI;;;;;
KM447238;Coleoptera;COI;;;;;
KM447620;Coleoptera;COI;;;;;
KM447626;Coleoptera;COI;;;;;
KM447733;Coleoptera;COI;;;;;
KM447747;Coleoptera;COI;;;;;
KM447850;Coleoptera;COI;;;;;
KM447908;Coleoptera;COI;;;;;
KM447951;Coleoptera;COI;;;;;
KM447980;Coleoptera;COI;;;;;
KM448077;Coleoptera;COI;;;;;
KM448128;Coleoptera;COI;;;;;
KM448177;Coleoptera;COI;;;;;
KM448328;Coleoptera;COI;;;;;
KM449044;Coleoptera;COI;;;;;
KM449191;Coleoptera;COI;;;;;
KM449264;Coleoptera;COI;;;;;
KM449356;Coleoptera;COI;;;;;
KM449381;Coleoptera;COI;;;;;
KM449677;Coleoptera;COI;;;;;
KM449706;Coleoptera;COI;;;;;
KM449810;Coleoptera;COI;;;;;
KM450068;Coleoptera;COI;;;;;
KM450093;Coleoptera;COI;;;;;
KM450153;Coleoptera;COI;;;;;
KM450211;Coleoptera;COI;;;;;
KM450280;Coleoptera;COI;;;;;
KM450500;Coleoptera;COI;;;;;
KM450698;Coleoptera;COI;;;;;
KM450755;Coleoptera;COI;;;;;
KM450819;Coleoptera;COI;;;;;
KM450842;Coleoptera;COI;;;;;
KM451116;Coleoptera;COI;;;;;
KM451136;Coleoptera;COI;;;;;
KM451282;Coleoptera;COI;;;;;
KM451343;Coleoptera;COI;;;;;
KM451368;Coleoptera;COI;;;;;
KM451533;Coleoptera;COI;;;;;
KM451925;Coleoptera;COI;;;;;
KM452118;Coleoptera;COI;;;;;
KM452426;Coleoptera;COI;;;;;
KM452605;Coleoptera;COI;;;;;
KM452608;Coleoptera;COI;;;;;
KT259032;Coleoptera;COI;;;;;
KT259033;Coleoptera;COI;;;;;

KT259034;Coleoptera;COI;;;;;
KT259035;Coleoptera;COI;;;;;
KT259036;Coleoptera;COI;;;;;
KT259037;Coleoptera;COI;;;;;
KT259038;Coleoptera;COI;;;;;
KU139606;Coleoptera;COI;;;;;
KU139607;Coleoptera;COI;;;;;
KU139608;Coleoptera;COI;;;;;
KU139608;Coleoptera;COI;;;;;
KU139609;Coleoptera;COI;;;;;
KU139609;Coleoptera;COI;;;;;
KU139610;Coleoptera;COI;;;;;
KU139611;Coleoptera;COI;;;;;
KU139612;Coleoptera;COI;;;;;
KU139613;Coleoptera;COI;;;;;
KU139614;Coleoptera;COI;;;;;
KU139615;Coleoptera;COI;;;;;
KU139616;Coleoptera;COI;;;;;
KU139617;Coleoptera;COI;;;;;
KU139618;Coleoptera;COI;;;;;
KU139619;Coleoptera;COI;;;;;
KU139620;Coleoptera;COI;;;;;
KU139621;Coleoptera;COI;;;;;
KU139622;Coleoptera;COI;;;;;
KU139623;Coleoptera;COI;;;;;
KU139624;Coleoptera;COI;;;;;
KU139625;Coleoptera;COI;;;;;
KU139626;Coleoptera;COI;;;;;
KU139627;Coleoptera;COI;;;;;
KU139628;Coleoptera;COI;;;;;
KU139629;Coleoptera;COI;;;;;
KU139630;Coleoptera;COI;;;;;
KU139631;Coleoptera;COI;;;;;
KU139632;Coleoptera;COI;;;;;
KU139633;Coleoptera;COI;;;;;
KU139634;Coleoptera;COI;;;;;
KU139635;Coleoptera;COI;;;;;
KU139636;Coleoptera;COI;;;;;
KU139637;Coleoptera;COI;;;;;
KU139638;Coleoptera;COI;;;;;
KU139639;Coleoptera;COI;;;;;
KU139639;Coleoptera;COI;;;;;
KU139640;Coleoptera;COI;;;;;
KU139641;Coleoptera;COI;;;;;
KU139641;Coleoptera;COI;;;;;
KU139642;Coleoptera;COI;;;;;
KU139643;Coleoptera;COI;;;;;
KU139644;Coleoptera;COI;;;;;
KU139645;Coleoptera;COI;;;;;
KU139645;Coleoptera;COI;;;;;
KU139646;Coleoptera;COI;;;;;
KU139646;Coleoptera;COI;;;;;
KU139647;Coleoptera;COI;;;;;
KU139648;Coleoptera;COI;;;;;
KU139649;Coleoptera;COI;;;;;
KU139649;Coleoptera;COI;;;;;
KU139650;Coleoptera;COI;;;;;
KU139650;Coleoptera;COI;;;;;
KU139651;Coleoptera;COI;;;;;
KU139651;Coleoptera;COI;;;;;
KU139652;Coleoptera;COI;;;;;
KU139653;Coleoptera;COI;;;;;
KU139653;Coleoptera;COI;;;;;
KU139654;Coleoptera;COI;;;;;
KU139654;Coleoptera;COI;;;;;
KU139655;Coleoptera;COI;;;;;
KU139655;Coleoptera;COI;;;;;
KU139656;Coleoptera;COI;;;;;
KU139656;Coleoptera;COI;;;;;
KU139657;Coleoptera;COI;;;;;
KU139657;Coleoptera;COI;;;;;
KU139658;Coleoptera;COI;;;;;
KU139659;Coleoptera;COI;;;;;
KU139659;Coleoptera;COI;;;;;
KU139660;Coleoptera;COI;;;;;
KU139660;Coleoptera;COI;;;;;
KU139661;Coleoptera;COI;;;;;
KU144883;Coleoptera;COI;;;;;

KU144884;Coleoptera;COI;;;;;
KU906201;Coleoptera;COI;;;;;
KU906345;Coleoptera;COI;;;;;
KU906588;Coleoptera;COI;;;;;
KU906993;Coleoptera;COI;;;;;
KU907426;Coleoptera;COI;;;;;
KU907631;Coleoptera;COI;;;;;
KU907821;Coleoptera;COI;;;;;
KU908106;Coleoptera;COI;;;;;
KU908627;Coleoptera;COI;;;;;
KU908861;Coleoptera;COI;;;;;
KU908901;Coleoptera;COI;;;;;
KU908973;Coleoptera;COI;;;;;
KU909043;Coleoptera;COI;;;;;
KU909393;Coleoptera;COI;;;;;
KU909402;Coleoptera;COI;;;;;
KU909805;Coleoptera;COI;;;;;
KU910325;Coleoptera;COI;;;;;
KU910682;Coleoptera;COI;;;;;
KU910951;Coleoptera;COI;;;;;
KU910964;Coleoptera;COI;;;;;
KU911145;Coleoptera;COI;;;;;
KU911262;Coleoptera;COI;;;;;
KU911413;Coleoptera;COI;;;;;
KU911624;Coleoptera;COI;;;;;
KU911976;Coleoptera;COI;;;;;
KU912074;Coleoptera;COI;;;;;
KU912168;Coleoptera;COI;;;;;
KU912182;Coleoptera;COI;;;;;
KU912417;Coleoptera;COI;;;;;
KU912444;Coleoptera;COI;;;;;
KU912639;Coleoptera;COI;;;;;
KU912807;Coleoptera;COI;;;;;
KU913185;Coleoptera;COI;;;;;
KU913203;Coleoptera;COI;;;;;
KU913342;Coleoptera;COI;;;;;
KU913421;Coleoptera;COI;;;;;
KU913850;Coleoptera;COI;;;;;
KU914416;Coleoptera;COI;;;;;
KU914637;Coleoptera;COI;;;;;
KU914770;Coleoptera;COI;;;;;
KU915174;Coleoptera;COI;;;;;
KU915206;Coleoptera;COI;;;;;
KU915289;Coleoptera;COI;;;;;
KU915307;Coleoptera;COI;;;;;
KU915328;Coleoptera;COI;;;;;
KU915331;Coleoptera;COI;;;;;
KU915448;Coleoptera;COI;;;;;
KU915544;Coleoptera;COI;;;;;
KU915548;Coleoptera;COI;;;;;
KU915794;Coleoptera;COI;;;;;
KU915817;Coleoptera;COI;;;;;
KU915923;Coleoptera;COI;;;;;
KU915924;Coleoptera;COI;;;;;
KU916023;Coleoptera;COI;;;;;
KU916136;Coleoptera;COI;;;;;
KU916261;Coleoptera;COI;;;;;
KU916700;Coleoptera;COI;;;;;
KU916926;Coleoptera;COI;;;;;
KU917113;Coleoptera;COI;;;;;
KU917151;Coleoptera;COI;;;;;
KU917280;Coleoptera;COI;;;;;
KU917478;Coleoptera;COI;;;;;
KU917557;Coleoptera;COI;;;;;
KU917580;Coleoptera;COI;;;;;
KU917704;Coleoptera;COI;;;;;
KU917743;Coleoptera;COI;;;;;
KU918103;Coleoptera;COI;;;;;
KU918384;Coleoptera;COI;;;;;
KU918738;Coleoptera;COI;;;;;
KU919529;Coleoptera;COI;;;;;
KX092766;Coleoptera;COI;;;;;
KX092775;Coleoptera;COI;;;;;
KX092781;Coleoptera;COI;;;;;
KX092792;Coleoptera;COI;;;;;
KX638452;Coleoptera;COI;;;;;
KX638453;Coleoptera;COI;;;;;
KX638454;Coleoptera;COI;;;;;

KX638455;Coleoptera;COI;;;;;
KX638456;Coleoptera;COI;;;;;
KX638457;Coleoptera;COI;;;;;
KX638458;Coleoptera;COI;;;;;
KX638459;Coleoptera;COI;;;;;
KX638460;Coleoptera;COI;;;;;
KX638461;Coleoptera;COI;;;;;
KX638462;Coleoptera;COI;;;;;
KX638463;Coleoptera;COI;;;;;
KX638464;Coleoptera;COI;;;;;
KX638465;Coleoptera;COI;;;;;
KX638466;Coleoptera;COI;;;;;
KX638467;Coleoptera;COI;;;;;
KX638468;Coleoptera;COI;;;;;
KX638469;Coleoptera;COI;;;;;
KX638470;Coleoptera;COI;;;;;
KX638471;Coleoptera;COI;;;;;
KX638472;Coleoptera;COI;;;;;
KX638473;Coleoptera;COI;;;;;
KX638474;Coleoptera;COI;;;;;
KX638475;Coleoptera;COI;;;;;
KX638476;Coleoptera;COI;;;;;
KX638477;Coleoptera;COI;;;;;
KX638478;Coleoptera;COI;;;;;
KX961659;Coleoptera;COI;;;;;
KX961660;Coleoptera;COI;;;;;
KX961661;Coleoptera;COI;;;;;
KX961662;Coleoptera;COI;;;;;
KX961663;Coleoptera;COI;;;;;
KX961664;Coleoptera;COI;;;;;
KX961665;Coleoptera;COI;;;;;
KX961666;Coleoptera;COI;;;;;
KY683691;Coleoptera;COI;;;;;
KY683700;Coleoptera;COI;;;;;
KY683704;Coleoptera;COI;;;;;
KY805887;Coleoptera;COI;;;;;
KY827326;Coleoptera;COI;;;;;
KY827327;Coleoptera;COI;;;;;
KY827328;Coleoptera;COI;;;;;
KY827329;Coleoptera;COI;;;;;
MF286059;Coleoptera;COI;;;;;
MF286060;Coleoptera;COI;;;;;
MF286061;Coleoptera;COI;;;;;
MF286063;Coleoptera;COI;;;;;
MF286066;Coleoptera;COI;;;;;
MF286067;Coleoptera;COI;;;;;
MF286068;Coleoptera;COI;;;;;
MF286069;Coleoptera;COI;;;;;
MF286070;Coleoptera;COI;;;;;
MF286074;Coleoptera;COI;;;;;
MF286075;Coleoptera;COI;;;;;
MF286076;Coleoptera;COI;;;;;
MF286077;Coleoptera;COI;;;;;
MF286078;Coleoptera;COI;;;;;
MF286079;Coleoptera;COI;;;;;
MF286080;Coleoptera;COI;;;;;
MF286081;Coleoptera;COI;;;;;
MF286084;Coleoptera;COI;;;;;
MF286085;Coleoptera;COI;;;;;
MF286094;Coleoptera;COI;;;;;
MF286095;Coleoptera;COI;;;;;
MF286096;Coleoptera;COI;;;;;
MF286097;Coleoptera;COI;;;;;
MF286102;Coleoptera;COI;;;;;
MF286102;Coleoptera;COI;;;;;
MF286103;Coleoptera;COI;;;;;
MF286103;Coleoptera;COI;;;;;
MF286108;Coleoptera;COI;;;;;
MF286109;Coleoptera;COI;;;;;
MF286110;Coleoptera;COI;;;;;
MF286111;Coleoptera;COI;;;;;
MF286112;Coleoptera;COI;;;;;
MF286113;Coleoptera;COI;;;;;
MF286114;Coleoptera;COI;;;;;
MF286115;Coleoptera;COI;;;;;
MF286116;Coleoptera;COI;;;;;
MF286117;Coleoptera;COI;;;;;
MF286118;Coleoptera;COI;;;;;

MF286119;Coleoptera;COI;;;;;
MF286120;Coleoptera;COI;;;;;
MF286121;Coleoptera;COI;;;;;
MF286122;Coleoptera;COI;;;;;
MF286123;Coleoptera;COI;;;;;
MF286124;Coleoptera;COI;;;;;
MF286125;Coleoptera;COI;;;;;
MF286126;Coleoptera;COI;;;;;
MF286129;Coleoptera;COI;;;;;
MF286130;Coleoptera;COI;;;;;
MF286131;Coleoptera;COI;;;;;
MF286132;Coleoptera;COI;;;;;
MF286133;Coleoptera;COI;;;;;
MF286134;Coleoptera;COI;;;;;
MF286135;Coleoptera;COI;;;;;
MF286136;Coleoptera;COI;;;;;
MF286137;Coleoptera;COI;;;;;
MF286149;Coleoptera;COI;;;;;
MF286152;Coleoptera;COI;;;;;
MF286153;Coleoptera;COI;;;;;
MF286169;Coleoptera;COI;;;;;
MF286170;Coleoptera;COI;;;;;
MF286173;Coleoptera;COI;;;;;
MF286184;Coleoptera;COI;;;;;
MF286186;Coleoptera;COI;;;;;
MF286187;Coleoptera;COI;;;;;
MF286204;Coleoptera;COI;;;;;
MF286209;Coleoptera;COI;;;;;
MF286211;Coleoptera;COI;;;;;
MF286216;Coleoptera;COI;;;;;
MF286315;Coleoptera;COI;;;;;
MF327394;Coleoptera;COI;;;;;
MF327395;Coleoptera;COI;;;;;
MF327396;Coleoptera;COI;;;;;
MF327397;Coleoptera;COI;;;;;
MF327398;Coleoptera;COI;;;;;
MF327399;Coleoptera;COI;;;;;
MF327400;Coleoptera;COI;;;;;
MF327401;Coleoptera;COI;;;;;
MF327402;Coleoptera;COI;;;;;
MF327403;Coleoptera;COI;;;;;
MF327404;Coleoptera;COI;;;;;
MF327405;Coleoptera;COI;;;;;
MF327406;Coleoptera;COI;;;;;
MF327407;Coleoptera;COI;;;;;
MF327408;Coleoptera;COI;;;;;
MF327409;Coleoptera;COI;;;;;
MF327410;Coleoptera;COI;;;;;
MF327411;Coleoptera;COI;;;;;
MF327412;Coleoptera;COI;;;;;
MF327413;Coleoptera;COI;;;;;
MF327414;Coleoptera;COI;;;;;
MF327415;Coleoptera;COI;;;;;
MF327416;Coleoptera;COI;;;;;
MF327417;Coleoptera;COI;;;;;
MF327418;Coleoptera;COI;;;;;
MF327419;Coleoptera;COI;;;;;
MF327420;Coleoptera;COI;;;;;
MF327421;Coleoptera;COI;;;;;
MF371175;Coleoptera;COI;;;;;
MF371176;Coleoptera;COI;;;;;
MF371177;Coleoptera;COI;;;;;
MF371178;Coleoptera;COI;;;;;
MF371179;Coleoptera;COI;;;;;
MF371180;Coleoptera;COI;;;;;
MF371181;Coleoptera;COI;;;;;
MF371182;Coleoptera;COI;;;;;
MF371183;Coleoptera;COI;;;;;
MF371184;Coleoptera;COI;;;;;
MF371185;Coleoptera;COI;;;;;
MF371186;Coleoptera;COI;;;;;
MF371187;Coleoptera;COI;;;;;
MF371188;Coleoptera;COI;;;;;
MF371189;Coleoptera;COI;;;;;
MF371190;Coleoptera;COI;;;;;
MF371191;Coleoptera;COI;;;;;
MF371192;Coleoptera;COI;;;;;
MF371193;Coleoptera;COI;;;;;

MF371194;Coleoptera;COI;;;;;
MF371195;Coleoptera;COI;;;;;
MF371196;Coleoptera;COI;;;;;
MF371197;Coleoptera;COI;;;;;
MF371198;Coleoptera;COI;;;;;
MF371199;Coleoptera;COI;;;;;
MF371200;Coleoptera;COI;;;;;
MF371201;Coleoptera;COI;;;;;
MF434579;Coleoptera;COI;;;;;
MF434580;Coleoptera;COI;;;;;
MF434581;Coleoptera;COI;;;;;
MF434582;Coleoptera;COI;;;;;
MF434583;Coleoptera;COI;;;;;
MF434584;Coleoptera;COI;;;;;
MF434585;Coleoptera;COI;;;;;
MF434586;Coleoptera;COI;;;;;
MF434587;Coleoptera;COI;;;;;
MF434588;Coleoptera;COI;;;;;
MF434589;Coleoptera;COI;;;;;
MF434590;Coleoptera;COI;;;;;
MF434591;Coleoptera;COI;;;;;
MF434592;Coleoptera;COI;;;;;
MF434593;Coleoptera;COI;;;;;
MF434594;Coleoptera;COI;;;;;
MF434595;Coleoptera;COI;;;;;
MF434596;Coleoptera;COI;;;;;
MF434597;Coleoptera;COI;;;;;
MF434598;Coleoptera;COI;;;;;
MF434599;Coleoptera;COI;;;;;
MF434600;Coleoptera;COI;;;;;
MF434601;Coleoptera;COI;;;;;
MF434602;Coleoptera;COI;;;;;
MF434603;Coleoptera;COI;;;;;
MF434604;Coleoptera;COI;;;;;
MF434605;Coleoptera;COI;;;;;
MF434606;Coleoptera;COI;;;;;
MF434607;Coleoptera;COI;;;;;
MF434608;Coleoptera;COI;;;;;
MF434609;Coleoptera;COI;;;;;
MF434610;Coleoptera;COI;;;;;
MF434611;Coleoptera;COI;;;;;
MF434612;Coleoptera;COI;;;;;
MF434613;Coleoptera;COI;;;;;
MF434614;Coleoptera;COI;;;;;
MF434615;Coleoptera;COI;;;;;
MF434616;Coleoptera;COI;;;;;
MF434617;Coleoptera;COI;;;;;
MF434618;Coleoptera;COI;;;;;
MF434619;Coleoptera;COI;;;;;
MF434620;Coleoptera;COI;;;;;
MF434621;Coleoptera;COI;;;;;
MF434622;Coleoptera;COI;;;;;
MF434623;Coleoptera;COI;;;;;
MF434624;Coleoptera;COI;;;;;
MF434625;Coleoptera;COI;;;;;
MF434626;Coleoptera;COI;;;;;
MF434627;Coleoptera;COI;;;;;
MF434628;Coleoptera;COI;;;;;
MF434629;Coleoptera;COI;;;;;
MF434630;Coleoptera;COI;;;;;
MF434631;Coleoptera;COI;;;;;
MF434632;Coleoptera;COI;;;;;
MF434633;Coleoptera;COI;;;;;
MF434634;Coleoptera;COI;;;;;
MF434635;Coleoptera;COI;;;;;
MF434636;Coleoptera;COI;;;;;
MF434637;Coleoptera;COI;;;;;
MF470086;Coleoptera;COI;;;;;
MF543038;Coleoptera;COI;;;;;
MF543039;Coleoptera;COI;;;;;
MG229689;Coleoptera;COI;;;;;
MG229689;Coleoptera;COI;;;;;
MG229691;Coleoptera;COI;;;;;
MG229691;Coleoptera;COI;;;;;
MG229695;Coleoptera;COI;;;;;
MG229696;Coleoptera;COI;;;;;
MG229698;Coleoptera;COI;;;;;
MG229699;Coleoptera;COI;;;;;

MG229699;Coleoptera;COI;;;;;
MG229701;Coleoptera;COI;;;;;
MG229743;Coleoptera;COI;;;;;
MG229743;Coleoptera;COI;;;;;
MG229785;Coleoptera;COI;;;;;
MG229790;Coleoptera;COI;;;;;
MG229826;Coleoptera;COI;;;;;
MG229829;Coleoptera;COI;;;;;
MG230706;Coleoptera;COI;;;;;
MG322660;Coleoptera;COI;;;;;
MG322660;Coleoptera;COI;;;;;
MG322662;Coleoptera;COI;;;;;
MG322662;Coleoptera;COI;;;;;
MG322663;Coleoptera;COI;;;;;
MG322663;Coleoptera;COI;;;;;
MG322672;Coleoptera;COI;;;;;
MG322672;Coleoptera;COI;;;;;
MG813270;Coleoptera;COI;;;;;
MG813271;Coleoptera;COI;;;;;
MG813272;Coleoptera;COI;;;;;
MG813274;Coleoptera;COI;;;;;
MG813275;Coleoptera;COI;;;;;
MG813276;Coleoptera;COI;;;;;
MG813277;Coleoptera;COI;;;;;
MG813278;Coleoptera;COI;;;;;
MG813279;Coleoptera;COI;;;;;
MG813280;Coleoptera;COI;;;;;
MG813281;Coleoptera;COI;;;;;
MG813282;Coleoptera;COI;;;;;
MG813283;Coleoptera;COI;;;;;
MG813288;Coleoptera;COI;;;;;
MG813289;Coleoptera;COI;;;;;
MG813290;Coleoptera;COI;;;;;
MG813291;Coleoptera;COI;;;;;
MG813292;Coleoptera;COI;;;;;
MG813293;Coleoptera;COI;;;;;
MG813294;Coleoptera;COI;;;;;
MG813298;Coleoptera;COI;;;;;
MG813299;Coleoptera;COI;;;;;
MG813300;Coleoptera;COI;;;;;
MG813301;Coleoptera;COI;;;;;
MG813302;Coleoptera;COI;;;;;
MG813303;Coleoptera;COI;;;;;
MG813304;Coleoptera;COI;;;;;
MG813305;Coleoptera;COI;;;;;
MG813306;Coleoptera;COI;;;;;
MG813306;Coleoptera;COI;;;;;
MG813307;Coleoptera;COI;;;;;
MG813310;Coleoptera;COI;;;;;
MG813311;Coleoptera;COI;;;;;
MG813313;Coleoptera;COI;;;;;
MG813314;Coleoptera;COI;;;;;
MG813315;Coleoptera;COI;;;;;
MG813316;Coleoptera;COI;;;;;
MG813317;Coleoptera;COI;;;;;
MG813318;Coleoptera;COI;;;;;
MG813320;Coleoptera;COI;;;;;
MG813323;Coleoptera;COI;;;;;
MG813324;Coleoptera;COI;;;;;
MG813325;Coleoptera;COI;;;;;
MG813327;Coleoptera;COI;;;;;
MG813328;Coleoptera;COI;;;;;
MG813329;Coleoptera;COI;;;;;
MG813330;Coleoptera;COI;;;;;
MG813331;Coleoptera;COI;;;;;
MG813332;Coleoptera;COI;;;;;
MH020453;Coleoptera;COI;;;;;
MH020548;Coleoptera;COI;;;;;
MH020549;Coleoptera;COI;;;;;
MH020550;Coleoptera;COI;;;;;
MH020551;Coleoptera;COI;;;;;
MH029245;Coleoptera;COI;;;;;
MH235266;Coleoptera;COI;;;;;
MH235267;Coleoptera;COI;;;;;
MH235268;Coleoptera;COI;;;;;
MH235269;Coleoptera;COI;;;;;
MH235270;Coleoptera;COI;;;;;
MH235271;Coleoptera;COI;;;;;

MH235272;Coleoptera;COI;;;;;
MH235284;Coleoptera;COI;;;;;
MH235299;Coleoptera;COI;;;;;
MH235332;Coleoptera;COI;;;;;
MH235333;Coleoptera;COI;;;;;
MH235334;Coleoptera;COI;;;;;
MH235335;Coleoptera;COI;;;;;
MH235336;Coleoptera;COI;;;;;
MH260900;Coleoptera;COI;;;;;
MH260901;Coleoptera;COI;;;;;
MH271197;Coleoptera;COI;;;;;
MH271198;Coleoptera;COI;;;;;
MH271199;Coleoptera;COI;;;;;
MH271200;Coleoptera;COI;;;;;
MH271201;Coleoptera;COI;;;;;
MH271202;Coleoptera;COI;;;;;
MH984362;Coleoptera;COI;;;;;
MH984363;Coleoptera;COI;;;;;
MH984366;Coleoptera;COI;;;;;
MH984463;Coleoptera;COI;;;;;
MH984479;Coleoptera;COI;;;;;
MK315155;Coleoptera;COI;;;;;
MK315156;Coleoptera;COI;;;;;
MK315157;Coleoptera;COI;;;;;
MK315158;Coleoptera;COI;;;;;
MK315159;Coleoptera;COI;;;;;
MK315160;Coleoptera;COI;;;;;
MK315161;Coleoptera;COI;;;;;
MK315162;Coleoptera;COI;;;;;
MK315163;Coleoptera;COI;;;;;
MK315164;Coleoptera;COI;;;;;
MK315165;Coleoptera;COI;;;;;
MK315166;Coleoptera;COI;;;;;
MK315167;Coleoptera;COI;;;;;
MK315168;Coleoptera;COI;;;;;
MK315169;Coleoptera;COI;;;;;
MK315170;Coleoptera;COI;;;;;
MK315171;Coleoptera;COI;;;;;
MK315172;Coleoptera;COI;;;;;
MK347662;Coleoptera;COI;;;;;
MK347675;Coleoptera;COI;;;;;
MK347684;Coleoptera;COI;;;;;
MK783322;Coleoptera;COI;;;;;
MK783323;Coleoptera;COI;;;;;
MK783324;Coleoptera;COI;;;;;
MK783325;Coleoptera;COI;;;;;
MK783326;Coleoptera;COI;;;;;
MK783327;Coleoptera;COI;;;;;
MK783328;Coleoptera;COI;;;;;
MK783329;Coleoptera;COI;;;;;
MK783330;Coleoptera;COI;;;;;
MK783331;Coleoptera;COI;;;;;
MK783332;Coleoptera;COI;;;;;
MK783333;Coleoptera;COI;;;;;
MK783334;Coleoptera;COI;;;;;
MK783335;Coleoptera;COI;;;;;
MK783336;Coleoptera;COI;;;;;
MK783337;Coleoptera;COI;;;;;
MK783338;Coleoptera;COI;;;;;
MK783339;Coleoptera;COI;;;;;
MK783340;Coleoptera;COI;;;;;
MK783341;Coleoptera;COI;;;;;
MK783342;Coleoptera;COI;;;;;
MK783343;Coleoptera;COI;;;;;
MK783344;Coleoptera;COI;;;;;
MK783345;Coleoptera;COI;;;;;
MK783346;Coleoptera;COI;;;;;
MK783347;Coleoptera;COI;;;;;
MK783348;Coleoptera;COI;;;;;
MK783349;Coleoptera;COI;;;;;
MK783350;Coleoptera;COI;;;;;
MK783351;Coleoptera;COI;;;;;
MK783352;Coleoptera;COI;;;;;
MK783353;Coleoptera;COI;;;;;
MK783354;Coleoptera;COI;;;;;
MK783355;Coleoptera;COI;;;;;
MK783356;Coleoptera;COI;;;;;
MK783357;Coleoptera;COI;;;;;

MK783358;Coleoptera;COI;;;;;
MK783359;Coleoptera;COI;;;;;
MK783360;Coleoptera;COI;;;;;
MK783361;Coleoptera;COI;;;;;
MK783362;Coleoptera;COI;;;;;
MK783363;Coleoptera;COI;;;;;
MK783364;Coleoptera;COI;;;;;
MK783365;Coleoptera;COI;;;;;
MK783366;Coleoptera;COI;;;;;
MK783367;Coleoptera;COI;;;;;
MK783368;Coleoptera;COI;;;;;
MK783369;Coleoptera;COI;;;;;
MK783370;Coleoptera;COI;;;;;
MK783371;Coleoptera;COI;;;;;
MK783372;Coleoptera;COI;;;;;
MK783373;Coleoptera;COI;;;;;
MK783374;Coleoptera;COI;;;;;
MK783375;Coleoptera;COI;;;;;
MK783376;Coleoptera;COI;;;;;
MK783377;Coleoptera;COI;;;;;
MK783378;Coleoptera;COI;;;;;
MK783379;Coleoptera;COI;;;;;
MK783380;Coleoptera;COI;;;;;
MK783381;Coleoptera;COI;;;;;
MK783382;Coleoptera;COI;;;;;
MK783383;Coleoptera;COI;;;;;
MK783384;Coleoptera;COI;;;;;
MK783385;Coleoptera;COI;;;;;
MK783386;Coleoptera;COI;;;;;
MK783387;Coleoptera;COI;;;;;
MK783388;Coleoptera;COI;;;;;
MK783389;Coleoptera;COI;;;;;
MK783390;Coleoptera;COI;;;;;
MK783391;Coleoptera;COI;;;;;
MK783392;Coleoptera;COI;;;;;
MK783393;Coleoptera;COI;;;;;
MK783394;Coleoptera;COI;;;;;
MK783395;Coleoptera;COI;;;;;
MK783396;Coleoptera;COI;;;;;
MK783397;Coleoptera;COI;;;;;
MK783398;Coleoptera;COI;;;;;
MK783399;Coleoptera;COI;;;;;
MK783400;Coleoptera;COI;;;;;
MK783401;Coleoptera;COI;;;;;
MK783402;Coleoptera;COI;;;;;
MK783403;Coleoptera;COI;;;;;
MK783404;Coleoptera;COI;;;;;
MK783405;Coleoptera;COI;;;;;
MK783406;Coleoptera;COI;;;;;
MK783407;Coleoptera;COI;;;;;
MK783408;Coleoptera;COI;;;;;
MK783409;Coleoptera;COI;;;;;
MK783410;Coleoptera;COI;;;;;
MK783411;Coleoptera;COI;;;;;
MK783412;Coleoptera;COI;;;;;
MK783413;Coleoptera;COI;;;;;
MK783414;Coleoptera;COI;;;;;
MK783415;Coleoptera;COI;;;;;
MK783416;Coleoptera;COI;;;;;
MK783417;Coleoptera;COI;;;;;
MK783418;Coleoptera;COI;;;;;
MK783419;Coleoptera;COI;;;;;
MK783420;Coleoptera;COI;;;;;
MK783421;Coleoptera;COI;;;;;
MK783422;Coleoptera;COI;;;;;
MK783423;Coleoptera;COI;;;;;
MK783424;Coleoptera;COI;;;;;
MK783425;Coleoptera;COI;;;;;
MK783426;Coleoptera;COI;;;;;
MK783427;Coleoptera;COI;;;;;
MK783428;Coleoptera;COI;;;;;
MK783429;Coleoptera;COI;;;;;
MK783430;Coleoptera;COI;;;;;
MK783431;Coleoptera;COI;;;;;
MK783432;Coleoptera;COI;;;;;
MK783433;Coleoptera;COI;;;;;
MK783434;Coleoptera;COI;;;;;
MK783435;Coleoptera;COI;;;;;

MK783436;Coleoptera;COI;;;;;
MK783437;Coleoptera;COI;;;;;
MK783438;Coleoptera;COI;;;;;
MK783439;Coleoptera;COI;;;;;
MK783440;Coleoptera;COI;;;;;
MK783441;Coleoptera;COI;;;;;
MK783442;Coleoptera;COI;;;;;
MK783443;Coleoptera;COI;;;;;
MK783444;Coleoptera;COI;;;;;
MK783445;Coleoptera;COI;;;;;
MK783446;Coleoptera;COI;;;;;
MK783447;Coleoptera;COI;;;;;
MK783448;Coleoptera;COI;;;;;
MK783449;Coleoptera;COI;;;;;
MK783450;Coleoptera;COI;;;;;
MK783451;Coleoptera;COI;;;;;
MK783452;Coleoptera;COI;;;;;
MK783453;Coleoptera;COI;;;;;
MK783454;Coleoptera;COI;;;;;
MK783455;Coleoptera;COI;;;;;
MK783456;Coleoptera;COI;;;;;
MK783457;Coleoptera;COI;;;;;
MK783458;Coleoptera;COI;;;;;
MK783459;Coleoptera;COI;;;;;
MK783460;Coleoptera;COI;;;;;
MK783461;Coleoptera;COI;;;;;
MK783462;Coleoptera;COI;;;;;
MK783463;Coleoptera;COI;;;;;
MK783464;Coleoptera;COI;;;;;
MK783465;Coleoptera;COI;;;;;
MK783466;Coleoptera;COI;;;;;
MK783467;Coleoptera;COI;;;;;
MK783468;Coleoptera;COI;;;;;
MK783469;Coleoptera;COI;;;;;
MK783470;Coleoptera;COI;;;;;
MK783471;Coleoptera;COI;;;;;
MK783472;Coleoptera;COI;;;;;
MK783473;Coleoptera;COI;;;;;
MK783474;Coleoptera;COI;;;;;
MK783475;Coleoptera;COI;;;;;
MK783476;Coleoptera;COI;;;;;
MK783477;Coleoptera;COI;;;;;
MK783478;Coleoptera;COI;;;;;
MK783479;Coleoptera;COI;;;;;
MK783480;Coleoptera;COI;;;;;
MK783481;Coleoptera;COI;;;;;
MK783482;Coleoptera;COI;;;;;
MK783483;Coleoptera;COI;;;;;
MK783484;Coleoptera;COI;;;;;
MK783485;Coleoptera;COI;;;;;
MK783486;Coleoptera;COI;;;;;
MK783487;Coleoptera;COI;;;;;
MK783488;Coleoptera;COI;;;;;
MK783489;Coleoptera;COI;;;;;
MK783490;Coleoptera;COI;;;;;
MK783491;Coleoptera;COI;;;;;
MK783492;Coleoptera;COI;;;;;
MK783493;Coleoptera;COI;;;;;
MK783494;Coleoptera;COI;;;;;
MK783495;Coleoptera;COI;;;;;
MK783496;Coleoptera;COI;;;;;
MK783497;Coleoptera;COI;;;;;
MK783498;Coleoptera;COI;;;;;
MK783499;Coleoptera;COI;;;;;
MK783500;Coleoptera;COI;;;;;
MK783501;Coleoptera;COI;;;;;
MK783502;Coleoptera;COI;;;;;
MK783503;Coleoptera;COI;;;;;
MK783504;Coleoptera;COI;;;;;
MK783505;Coleoptera;COI;;;;;
MK783506;Coleoptera;COI;;;;;
MK783507;Coleoptera;COI;;;;;
MK783508;Coleoptera;COI;;;;;
MK783509;Coleoptera;COI;;;;;
MK783510;Coleoptera;COI;;;;;
MK783511;Coleoptera;COI;;;;;
MK783512;Coleoptera;COI;;;;;
MK783513;Coleoptera;COI;;;;;

MK783514;Coleoptera;COI;;;;;
MK783515;Coleoptera;COI;;;;;
MK783516;Coleoptera;COI;;;;;
MK783517;Coleoptera;COI;;;;;
MK783518;Coleoptera;COI;;;;;
MK783519;Coleoptera;COI;;;;;
MK783520;Coleoptera;COI;;;;;
MK783521;Coleoptera;COI;;;;;
MK783522;Coleoptera;COI;;;;;
MK783523;Coleoptera;COI;;;;;
MK783524;Coleoptera;COI;;;;;
MK783525;Coleoptera;COI;;;;;
MK783526;Coleoptera;COI;;;;;
MK783527;Coleoptera;COI;;;;;
MK783528;Coleoptera;COI;;;;;
MK783529;Coleoptera;COI;;;;;
MK783530;Coleoptera;COI;;;;;
MK783531;Coleoptera;COI;;;;;
MK783532;Coleoptera;COI;;;;;
MK783533;Coleoptera;COI;;;;;
MK783534;Coleoptera;COI;;;;;
MK783535;Coleoptera;COI;;;;;
MK783536;Coleoptera;COI;;;;;
MK783537;Coleoptera;COI;;;;;
MK783538;Coleoptera;COI;;;;;
MK783539;Coleoptera;COI;;;;;
MK783540;Coleoptera;COI;;;;;
MK783541;Coleoptera;COI;;;;;
MK783542;Coleoptera;COI;;;;;
MK783543;Coleoptera;COI;;;;;
MK783544;Coleoptera;COI;;;;;
MK783545;Coleoptera;COI;;;;;
MK783546;Coleoptera;COI;;;;;
MK783547;Coleoptera;COI;;;;;
MK783548;Coleoptera;COI;;;;;
MK783549;Coleoptera;COI;;;;;
MK783550;Coleoptera;COI;;;;;
MK783551;Coleoptera;COI;;;;;
MK783552;Coleoptera;COI;;;;;
MK783553;Coleoptera;COI;;;;;
MK783554;Coleoptera;COI;;;;;
MK783555;Coleoptera;COI;;;;;
MK783556;Coleoptera;COI;;;;;
MK783557;Coleoptera;COI;;;;;
MK783558;Coleoptera;COI;;;;;
MK783559;Coleoptera;COI;;;;;
MK783560;Coleoptera;COI;;;;;
MK783561;Coleoptera;COI;;;;;
MK783562;Coleoptera;COI;;;;;
MK783563;Coleoptera;COI;;;;;
MK783564;Coleoptera;COI;;;;;
MK783565;Coleoptera;COI;;;;;
MK783566;Coleoptera;COI;;;;;
MK783567;Coleoptera;COI;;;;;
MK783568;Coleoptera;COI;;;;;
MK783569;Coleoptera;COI;;;;;
MK783570;Coleoptera;COI;;;;;
MK783571;Coleoptera;COI;;;;;
MK783572;Coleoptera;COI;;;;;
MK783573;Coleoptera;COI;;;;;
MK783574;Coleoptera;COI;;;;;
MK783575;Coleoptera;COI;;;;;
MK783576;Coleoptera;COI;;;;;
MK783577;Coleoptera;COI;;;;;
MK783578;Coleoptera;COI;;;;;
MK783579;Coleoptera;COI;;;;;
MK783580;Coleoptera;COI;;;;;
MK783581;Coleoptera;COI;;;;;
MK783582;Coleoptera;COI;;;;;
MK783583;Coleoptera;COI;;;;;
MK783584;Coleoptera;COI;;;;;
MK783585;Coleoptera;COI;;;;;
MK783586;Coleoptera;COI;;;;;
MK783587;Coleoptera;COI;;;;;
MK783588;Coleoptera;COI;;;;;
MK783589;Coleoptera;COI;;;;;
MK783590;Coleoptera;COI;;;;;
MK783591;Coleoptera;COI;;;;;

MK783592;Coleoptera;COI;;;;;
MK783593;Coleoptera;COI;;;;;
MK783594;Coleoptera;COI;;;;;
MK783595;Coleoptera;COI;;;;;
MK783596;Coleoptera;COI;;;;;
MK783597;Coleoptera;COI;;;;;
MK783598;Coleoptera;COI;;;;;
MK783599;Coleoptera;COI;;;;;
MK783600;Coleoptera;COI;;;;;
MK783601;Coleoptera;COI;;;;;
MK783602;Coleoptera;COI;;;;;
MK783603;Coleoptera;COI;;;;;
MK783604;Coleoptera;COI;;;;;
MK783605;Coleoptera;COI;;;;;
MK783606;Coleoptera;COI;;;;;
MK783607;Coleoptera;COI;;;;;
MK783608;Coleoptera;COI;;;;;
MK783609;Coleoptera;COI;;;;;
MK783610;Coleoptera;COI;;;;;
MK783611;Coleoptera;COI;;;;;
MK783612;Coleoptera;COI;;;;;
MK783613;Coleoptera;COI;;;;;
MK783614;Coleoptera;COI;;;;;
MK783615;Coleoptera;COI;;;;;
MK783616;Coleoptera;COI;;;;;
MK783617;Coleoptera;COI;;;;;
MK783618;Coleoptera;COI;;;;;
MK783619;Coleoptera;COI;;;;;
MK783620;Coleoptera;COI;;;;;
MK783621;Coleoptera;COI;;;;;
MK783622;Coleoptera;COI;;;;;
MK783623;Coleoptera;COI;;;;;
MK783624;Coleoptera;COI;;;;;
MK783625;Coleoptera;COI;;;;;
MK783626;Coleoptera;COI;;;;;
MK783627;Coleoptera;COI;;;;;
MK783628;Coleoptera;COI;;;;;
MK783629;Coleoptera;COI;;;;;
MK783630;Coleoptera;COI;;;;;
MK783631;Coleoptera;COI;;;;;
MK783632;Coleoptera;COI;;;;;
MK783633;Coleoptera;COI;;;;;
MK783634;Coleoptera;COI;;;;;
MK783635;Coleoptera;COI;;;;;
MK783636;Coleoptera;COI;;;;;
MK783637;Coleoptera;COI;;;;;
MK783638;Coleoptera;COI;;;;;
MK783639;Coleoptera;COI;;;;;
MK783640;Coleoptera;COI;;;;;
MK783641;Coleoptera;COI;;;;;
MK783642;Coleoptera;COI;;;;;
MK783643;Coleoptera;COI;;;;;
MK783644;Coleoptera;COI;;;;;
MK783645;Coleoptera;COI;;;;;
MK783646;Coleoptera;COI;;;;;
MK783647;Coleoptera;COI;;;;;
MK783648;Coleoptera;COI;;;;;
MK783649;Coleoptera;COI;;;;;
MK783650;Coleoptera;COI;;;;;
MK783651;Coleoptera;COI;;;;;
MK783652;Coleoptera;COI;;;;;
MK783653;Coleoptera;COI;;;;;
MK783654;Coleoptera;COI;;;;;
MK783655;Coleoptera;COI;;;;;
MK783656;Coleoptera;COI;;;;;
MK783657;Coleoptera;COI;;;;;
MK783658;Coleoptera;COI;;;;;
MK783659;Coleoptera;COI;;;;;
MK783660;Coleoptera;COI;;;;;
MK783661;Coleoptera;COI;;;;;
MK783662;Coleoptera;COI;;;;;
MK783663;Coleoptera;COI;;;;;
MK783664;Coleoptera;COI;;;;;
MK783665;Coleoptera;COI;;;;;
MK783666;Coleoptera;COI;;;;;
MK783667;Coleoptera;COI;;;;;
MK783668;Coleoptera;COI;;;;;
MK783669;Coleoptera;COI;;;;;

MK783670;Coleoptera;COI;;;;;
MK783671;Coleoptera;COI;;;;;
MK783672;Coleoptera;COI;;;;;
MK783673;Coleoptera;COI;;;;;
MK783674;Coleoptera;COI;;;;;
MK783675;Coleoptera;COI;;;;;
MK783676;Coleoptera;COI;;;;;
MK783677;Coleoptera;COI;;;;;
MK783678;Coleoptera;COI;;;;;
MK783679;Coleoptera;COI;;;;;
MK891302;Coleoptera;COI;;;;;
MK891391;Coleoptera;COI;;;;;
MK891464;Coleoptera;COI;;;;;
MK891561;Coleoptera;COI;;;;;
MK891638;Coleoptera;COI;;;;;
MK892251;Coleoptera;COI;;;;;
MK892386;Coleoptera;COI;;;;;
MN182769;Coleoptera;COI;;;;;
MN182770;Coleoptera;COI;;;;;
MN182771;Coleoptera;COI;;;;;
MN182773;Coleoptera;COI;;;;;
MN182775;Coleoptera;COI;;;;;
MN182786;Coleoptera;COI;;;;;
MN182789;Coleoptera;COI;;;;;
MN182793;Coleoptera;COI;;;;;
MN182794;Coleoptera;COI;;;;;
MN182801;Coleoptera;COI;;;;;
MN182802;Coleoptera;COI;;;;;
MN182810;Coleoptera;COI;;;;;
MN182814;Coleoptera;COI;;;;;
MN182825;Coleoptera;COI;;;;;
MN182829;Coleoptera;COI;;;;;
MN182831;Coleoptera;COI;;;;;
MN182846;Coleoptera;COI;;;;;
MN182850;Coleoptera;COI;;;;;
MN182859;Coleoptera;COI;;;;;
MN182868;Coleoptera;COI;;;;;
MN182870;Coleoptera;COI;;;;;
MN182873;Coleoptera;COI;;;;;
MN182875;Coleoptera;COI;;;;;
MN182888;Coleoptera;COI;;;;;
MN182889;Coleoptera;COI;;;;;
MN182916;Coleoptera;COI;;;;;
MN182916;Coleoptera;COI;;;;;
MN182934;Coleoptera;COI;;;;;
MN182935;Coleoptera;COI;;;;;
MN182940;Coleoptera;COI;;;;;
MN182942;Coleoptera;COI;;;;;
MN182943;Coleoptera;COI;;;;;
MN182950;Coleoptera;COI;;;;;
MN182962;Coleoptera;COI;;;;;
MN182971;Coleoptera;COI;;;;;
MN182974;Coleoptera;COI;;;;;
MN182994;Coleoptera;COI;;;;;
MN182995;Coleoptera;COI;;;;;
MN183001;Coleoptera;COI;;;;;
MN183017;Coleoptera;COI;;;;;
MN183020;Coleoptera;COI;;;;;
MN183040;Coleoptera;COI;;;;;
MN183043;Coleoptera;COI;;;;;
MN183048;Coleoptera;COI;;;;;
MT140900;Coleoptera;COI;;;;;
MZ569316;Coleoptera;COI;;;;;
MZ569317;Coleoptera;COI;;;;;
MZ569318;Coleoptera;COI;;;;;
MZ569319;Coleoptera;COI;;;;;
MZ569320;Coleoptera;COI;;;;;
MZ569321;Coleoptera;COI;;;;;
MZ569322;Coleoptera;COI;;;;;
MZ569323;Coleoptera;COI;;;;;
MZ569324;Coleoptera;COI;;;;;
MZ569325;Coleoptera;COI;;;;;
MZ569326;Coleoptera;COI;;;;;
MZ569327;Coleoptera;COI;;;;;
MZ569328;Coleoptera;COI;;;;;
MZ569329;Coleoptera;COI;;;;;
MZ569330;Coleoptera;COI;;;;;
MZ569331;Coleoptera;COI;;;;;

MZ569332;Coleoptera;COI;;;;;
MZ569333;Coleoptera;COI;;;;;
MZ569334;Coleoptera;COI;;;;;
MZ569335;Coleoptera;COI;;;;;
MZ569336;Coleoptera;COI;;;;;
MZ569337;Coleoptera;COI;;;;;
MZ569338;Coleoptera;COI;;;;;
MZ569339;Coleoptera;COI;;;;;
MZ569340;Coleoptera;COI;;;;;
MZ569341;Coleoptera;COI;;;;;
MZ569342;Coleoptera;COI;;;;;
MZ569343;Coleoptera;COI;;;;;
MZ569344;Coleoptera;COI;;;;;
MZ569345;Coleoptera;COI;;;;;
MZ569346;Coleoptera;COI;;;;;
MZ569347;Coleoptera;COI;;;;;
MZ569348;Coleoptera;COI;;;;;
MZ569349;Coleoptera;COI;;;;;
MZ569350;Coleoptera;COI;;;;;
MZ569351;Coleoptera;COI;;;;;
MZ569352;Coleoptera;COI;;;;;
MZ569353;Coleoptera;COI;;;;;
MZ569354;Coleoptera;COI;;;;;
MZ569355;Coleoptera;COI;;;;;
MZ569356;Coleoptera;COI;;;;;
MZ569357;Coleoptera;COI;;;;;
MZ569358;Coleoptera;COI;;;;;
MZ569359;Coleoptera;COI;;;;;
MZ569360;Coleoptera;COI;;;;;
MZ569361;Coleoptera;COI;;;;;
MZ569362;Coleoptera;COI;;;;;
MZ569363;Coleoptera;COI;;;;;
MZ569364;Coleoptera;COI;;;;;
MZ569365;Coleoptera;COI;;;;;
MZ607992;Coleoptera;COI;;;;;
MZ608098;Coleoptera;COI;;;;;
MZ608397;Coleoptera;COI;;;;;
MZ608688;Coleoptera;COI;;;;;
MZ608973;Coleoptera;COI;;;;;
MZ609618;Coleoptera;COI;;;;;
MZ610082;Coleoptera;COI;;;;;
MZ610090;Coleoptera;COI;;;;;
MZ610333;Coleoptera;COI;;;;;
MZ610878;Coleoptera;COI;;;;;
MZ629646;Coleoptera;COI;;;;;
MZ629988;Coleoptera;COI;;;;;
MZ630314;Coleoptera;COI;;;;;
MZ631027;Coleoptera;COI;;;;;
MZ631142;Coleoptera;COI;;;;;
MZ631796;Coleoptera;COI;;;;;
MZ632028;Coleoptera;COI;;;;;
MZ632494;Coleoptera;COI;;;;;
MZ633128;Coleoptera;COI;;;;;
MZ634092;Coleoptera;COI;;;;;
MZ634092;Coleoptera;COI;;;;;
MZ656341;Coleoptera;COI;;;;;
MZ656383;Coleoptera;COI;;;;;
MZ657595;Coleoptera;COI;;;;;
MZ658584;Coleoptera;COI;;;;;
MZ658597;Coleoptera;COI;;;;;
MZ658601;Coleoptera;COI;;;;;
MZ658922;Coleoptera;COI;;;;;
MZ659121;Coleoptera;COI;;;;;
MZ659340;Coleoptera;COI;;;;;
MZ659681;Coleoptera;COI;;;;;
MZ660707;Coleoptera;COI;;;;;
MZ660823;Coleoptera;COI;;;;;
MZ661035;Coleoptera;COI;;;;;
MZ661040;Coleoptera;COI;;;;;
OK510537;Coleoptera;COI;;;;;
OK510538;Coleoptera;COI;;;;;
OK510539;Coleoptera;COI;;;;;
OK510540;Coleoptera;COI;;;;;
OK510541;Coleoptera;COI;;;;;
OK510542;Coleoptera;COI;;;;;
OK510543;Coleoptera;COI;;;;;
OK510547;Coleoptera;COI;;;;;
OK510548;Coleoptera;COI;;;;;

OK510549;Coleoptera;COI;;;;;
OK510550;Coleoptera;COI;;;;;
OK510560;Coleoptera;COI;;;;;
OK510561;Coleoptera;COI;;;;;
OK510562;Coleoptera;COI;;;;;
OK510563;Coleoptera;COI;;;;;
OK510564;Coleoptera;COI;;;;;
OK510565;Coleoptera;COI;;;;;
OK510566;Coleoptera;COI;;;;;
OK510567;Coleoptera;COI;;;;;
OK510568;Coleoptera;COI;;;;;
OK510569;Coleoptera;COI;;;;;
OK510570;Coleoptera;COI;;;;;
OK510571;Coleoptera;COI;;;;;
OK510572;Coleoptera;COI;;;;;
OK510573;Coleoptera;COI;;;;;
OK510574;Coleoptera;COI;;;;;
OK510575;Coleoptera;COI;;;;;
OK510576;Coleoptera;COI;;;;;
OK510577;Coleoptera;COI;;;;;
OK510578;Coleoptera;COI;;;;;
OP279134;Coleoptera;COI;;;;;
EF028492;Hymenoptera;COI;;;;;
EF028506;Hymenoptera;COI;;;;;
HQ948038;Hymenoptera;COI;;;;;
HQ948039;Hymenoptera;COI;;;;;
KJ836467;Hymenoptera;COI;;;;;
KJ836696;Hymenoptera;COI;;;;;
KJ837147;Hymenoptera;COI;;;;;
KJ837493;Hymenoptera;COI;;;;;
KJ837659;Hymenoptera;COI;;;;;
KJ837817;Hymenoptera;COI;;;;;
KJ838086;Hymenoptera;COI;;;;;
KJ838106;Hymenoptera;COI;;;;;
KJ838179;Hymenoptera;COI;;;;;
KJ838805;Hymenoptera;COI;;;;;
KY317973;Hymenoptera;COI;;;;;
KY317975;Hymenoptera;COI;;;;;
KY317976;Hymenoptera;COI;;;;;
KY317977;Hymenoptera;COI;;;;;
KY317978;Hymenoptera;COI;;;;;
KY317979;Hymenoptera;COI;;;;;
KY317980;Hymenoptera;COI;;;;;
LR738830;Hymenoptera;COI;;;;;
MH319246;Hymenoptera;COI;;;;;
MT869997;Hymenoptera;COI;;;;;
MZ623000;Hymenoptera;COI;;;;;
MZ626347;Hymenoptera;COI;;;;;
AB674369;Lepidoptera;COI;;;;;
AB674370;Lepidoptera;COI;;;;;
AB674378;Lepidoptera;COI;;;;;
AY346233;Lepidoptera;COI;;;;;
AY556863;Lepidoptera;COI;;;;;
AY556953;Lepidoptera;COI;;;;;
AY556961;Lepidoptera;COI;;;;;
AY557042;Lepidoptera;COI;;;;;
AY557064;Lepidoptera;COI;;;;;
AY557070;Lepidoptera;COI;;;;;
AY557071;Lepidoptera;COI;;;;;
DQ008088;Lepidoptera;COI;;;;;
DQ008090;Lepidoptera;COI;;;;;
DQ008091;Lepidoptera;COI;;;;;
FJ435341;Lepidoptera;COI;;;;;
FJ628432;Lepidoptera;COI;;;;;
FJ628433;Lepidoptera;COI;;;;;
FJ628444;Lepidoptera;COI;;;;;
FJ663252;Lepidoptera;COI;;;;;
FJ663253;Lepidoptera;COI;;;;;
FJ663676;Lepidoptera;COI;;;;;
FJ663677;Lepidoptera;COI;;;;;
FJ663678;Lepidoptera;COI;;;;;
FM196454;Lepidoptera;COI;;;;;
FM196478;Lepidoptera;COI;;;;;
FM196520;Lepidoptera;COI;;;;;
FM196524;Lepidoptera;COI;;;;;
GQ201115;Lepidoptera;COI;;;;;
GQ201119;Lepidoptera;COI;;;;;
GU669369;Lepidoptera;COI;;;;;

GU669707;Lepidoptera;COI;;;;;
GU669726;Lepidoptera;COI;;;;;
GU669727;Lepidoptera;COI;;;;;
GU669777;Lepidoptera;COI;;;;;
GU669794;Lepidoptera;COI;;;;;
GU669795;Lepidoptera;COI;;;;;
GU675634;Lepidoptera;COI;;;;;
GU675663;Lepidoptera;COI;;;;;
GU675680;Lepidoptera;COI;;;;;
GU675773;Lepidoptera;COI;;;;;
GU675930;Lepidoptera;COI;;;;;
GU676006;Lepidoptera;COI;;;;;
GU676148;Lepidoptera;COI;;;;;
GU676161;Lepidoptera;COI;;;;;
GU676163;Lepidoptera;COI;;;;;
GU676170;Lepidoptera;COI;;;;;
GU676241;Lepidoptera;COI;;;;;
GU676242;Lepidoptera;COI;;;;;
GU676244;Lepidoptera;COI;;;;;
GU676309;Lepidoptera;COI;;;;;
GU676562;Lepidoptera;COI;;;;;
GU676750;Lepidoptera;COI;;;;;
GU676751;Lepidoptera;COI;;;;;
GU676756;Lepidoptera;COI;;;;;
GU676878;Lepidoptera;COI;;;;;
GU676899;Lepidoptera;COI;;;;;
GU707096;Lepidoptera;COI;;;;;
GU707267;Lepidoptera;COI;;;;;
GU947500;Lepidoptera;COI;;;;;
HM870990;Lepidoptera;COI;;;;;
HM901240;Lepidoptera;COI;;;;;
HM901259;Lepidoptera;COI;;;;;
HM901297;Lepidoptera;COI;;;;;
HM901402;Lepidoptera;COI;;;;;
HM901454;Lepidoptera;COI;;;;;
HM901470;Lepidoptera;COI;;;;;
HM901532;Lepidoptera;COI;;;;;
HM901538;Lepidoptera;COI;;;;;
HM901539;Lepidoptera;COI;;;;;
HM901540;Lepidoptera;COI;;;;;
HM901772;Lepidoptera;COI;;;;;
HM910514;Lepidoptera;COI;;;;;
HM913972;Lepidoptera;COI;;;;;
HQ003941;Lepidoptera;COI;;;;;
HQ003942;Lepidoptera;COI;;;;;
HQ003943;Lepidoptera;COI;;;;;
HQ003944;Lepidoptera;COI;;;;;
HQ003945;Lepidoptera;COI;;;;;
HQ003946;Lepidoptera;COI;;;;;
HQ004016;Lepidoptera;COI;;;;;
HQ004018;Lepidoptera;COI;;;;;
HQ004019;Lepidoptera;COI;;;;;
HQ004020;Lepidoptera;COI;;;;;
HQ004154;Lepidoptera;COI;;;;;
HQ004166;Lepidoptera;COI;;;;;
HQ004167;Lepidoptera;COI;;;;;
HQ004168;Lepidoptera;COI;;;;;
HQ004169;Lepidoptera;COI;;;;;
HQ004170;Lepidoptera;COI;;;;;
HQ004171;Lepidoptera;COI;;;;;
HQ004172;Lepidoptera;COI;;;;;
HQ004173;Lepidoptera;COI;;;;;
HQ004174;Lepidoptera;COI;;;;;
HQ004397;Lepidoptera;COI;;;;;
HQ004401;Lepidoptera;COI;;;;;
HQ004403;Lepidoptera;COI;;;;;
HQ004404;Lepidoptera;COI;;;;;
HQ004532;Lepidoptera;COI;;;;;
JF415678;Lepidoptera;COI;;;;;
JF850426;Lepidoptera;COI;;;;;
JF853646;Lepidoptera;COI;;;;;
JF853647;Lepidoptera;COI;;;;;
JF860019;Lepidoptera;COI;;;;;
JN160200;Lepidoptera;COI;;;;;
JN160201;Lepidoptera;COI;;;;;
JN160202;Lepidoptera;COI;;;;;
JN274545;Lepidoptera;COI;;;;;
JN581045;Lepidoptera;COI;;;;;

JN581046;Lepidoptera;COI;;;;;
JN581047;Lepidoptera;COI;;;;;
JN581048;Lepidoptera;COI;;;;;
JN581049;Lepidoptera;COI;;;;;
JN581050;Lepidoptera;COI;;;;;
JN581051;Lepidoptera;COI;;;;;
JN581052;Lepidoptera;COI;;;;;
JN581053;Lepidoptera;COI;;;;;
JN581054;Lepidoptera;COI;;;;;
JN581055;Lepidoptera;COI;;;;;
JN581056;Lepidoptera;COI;;;;;
JN581057;Lepidoptera;COI;;;;;
JN581058;Lepidoptera;COI;;;;;
JN581059;Lepidoptera;COI;;;;;
JN581060;Lepidoptera;COI;;;;;
JQ080283;Lepidoptera;COI;;;;;
JX112882;Lepidoptera;COI;;;;;
KC462811;Lepidoptera;COI;;;;;
KF723539;Lepidoptera;COI;;;;;
KM572903;Lepidoptera;COI;;;;;
KM573283;Lepidoptera;COI;;;;;
KM972603;Lepidoptera;COI;;;;;
KM972632;Lepidoptera;COI;;;;;
KP032262;Lepidoptera;COI;;;;;
KP032280;Lepidoptera;COI;;;;;
KP032286;Lepidoptera;COI;;;;;
KP032316;Lepidoptera;COI;;;;;
KP032352;Lepidoptera;COI;;;;;
KP253333;Lepidoptera;COI;;;;;
KP253371;Lepidoptera;COI;;;;;
KP689294;Lepidoptera;COI;;;;;
KP689295;Lepidoptera;COI;;;;;
KP689296;Lepidoptera;COI;;;;;
KP689297;Lepidoptera;COI;;;;;
KP870221;Lepidoptera;COI;;;;;
KP870242;Lepidoptera;COI;;;;;
KP870332;Lepidoptera;COI;;;;;
KP870395;Lepidoptera;COI;;;;;
KP870432;Lepidoptera;COI;;;;;
KP870454;Lepidoptera;COI;;;;;
KP870544;Lepidoptera;COI;;;;;
KP870556;Lepidoptera;COI;;;;;
KP870646;Lepidoptera;COI;;;;;
KP870664;Lepidoptera;COI;;;;;
KP870699;Lepidoptera;COI;;;;;
KP870702;Lepidoptera;COI;;;;;
KP870771;Lepidoptera;COI;;;;;
KP870778;Lepidoptera;COI;;;;;
KP870786;Lepidoptera;COI;;;;;
KP870790;Lepidoptera;COI;;;;;
KP870803;Lepidoptera;COI;;;;;
KP870815;Lepidoptera;COI;;;;;
KP870825;Lepidoptera;COI;;;;;
KP870887;Lepidoptera;COI;;;;;
KP870896;Lepidoptera;COI;;;;;
KP870961;Lepidoptera;COI;;;;;
KP871171;Lepidoptera;COI;;;;;
KT792887;Lepidoptera;COI;;;;;
KT792892;Lepidoptera;COI;;;;;
KT792893;Lepidoptera;COI;;;;;
KT907186;Lepidoptera;COI;;;;;
KT907188;Lepidoptera;COI;;;;;
KT907189;Lepidoptera;COI;;;;;
KX011034;Lepidoptera;COI;;;;;
KX011035;Lepidoptera;COI;;;;;
KX011036;Lepidoptera;COI;;;;;
KX011037;Lepidoptera;COI;;;;;
KX040229;Lepidoptera;COI;;;;;
KX041759;Lepidoptera;COI;;;;;
KX045900;Lepidoptera;COI;;;;;
KX050108;Lepidoptera;COI;;;;;
KX241502;Lepidoptera;COI;;;;;
KX241503;Lepidoptera;COI;;;;;
KY128341;Lepidoptera;COI;;;;;
LC229025;Lepidoptera;COI;;;;;
LC229026;Lepidoptera;COI;;;;;
LC229027;Lepidoptera;COI;;;;;
LC340451;Lepidoptera;COI;;;;;

LC340474;Lepidoptera;COI;;;;;
MG522392;Lepidoptera;COI;;;;;
MG522502;Lepidoptera;COI;;;;;
MG522604;Lepidoptera;COI;;;;;
MH418556;Lepidoptera;COI;;;;;
MH418588;Lepidoptera;COI;;;;;
MH418901;Lepidoptera;COI;;;;;
MH418997;Lepidoptera;COI;;;;;
MH419007;Lepidoptera;COI;;;;;
MH419121;Lepidoptera;COI;;;;;
MH419155;Lepidoptera;COI;;;;;
MH419515;Lepidoptera;COI;;;;;
MH419806;Lepidoptera;COI;;;;;
MH419908;Lepidoptera;COI;;;;;
MH419939;Lepidoptera;COI;;;;;
MH420001;Lepidoptera;COI;;;;;
MH420002;Lepidoptera;COI;;;;;
MH670083;Lepidoptera;COI;;;;;
MH670084;Lepidoptera;COI;;;;;
MH670085;Lepidoptera;COI;;;;;
MH670086;Lepidoptera;COI;;;;;
MH670087;Lepidoptera;COI;;;;;
MH670088;Lepidoptera;COI;;;;;
MH670089;Lepidoptera;COI;;;;;
MH670090;Lepidoptera;COI;;;;;
MH670091;Lepidoptera;COI;;;;;
MH670092;Lepidoptera;COI;;;;;
MH670093;Lepidoptera;COI;;;;;
MH670094;Lepidoptera;COI;;;;;
MH670095;Lepidoptera;COI;;;;;
MH670096;Lepidoptera;COI;;;;;
MH670097;Lepidoptera;COI;;;;;
MH670098;Lepidoptera;COI;;;;;
MH670099;Lepidoptera;COI;;;;;
MH670101;Lepidoptera;COI;;;;;
MH670102;Lepidoptera;COI;;;;;
MH670103;Lepidoptera;COI;;;;;
MH670104;Lepidoptera;COI;;;;;
MH670105;Lepidoptera;COI;;;;;
MH670106;Lepidoptera;COI;;;;;
MH670107;Lepidoptera;COI;;;;;
MH670108;Lepidoptera;COI;;;;;
MH670109;Lepidoptera;COI;;;;;
MH670110;Lepidoptera;COI;;;;;
MH670111;Lepidoptera;COI;;;;;
MH670112;Lepidoptera;COI;;;;;
MH670113;Lepidoptera;COI;;;;;
MH670117;Lepidoptera;COI;;;;;
MH670118;Lepidoptera;COI;;;;;
MH670119;Lepidoptera;COI;;;;;
MH670120;Lepidoptera;COI;;;;;
MH670121;Lepidoptera;COI;;;;;
MH670122;Lepidoptera;COI;;;;;
MH670123;Lepidoptera;COI;;;;;
MH670124;Lepidoptera;COI;;;;;
MH670126;Lepidoptera;COI;;;;;
MH670127;Lepidoptera;COI;;;;;
MH670128;Lepidoptera;COI;;;;;
MH670129;Lepidoptera;COI;;;;;
MH670130;Lepidoptera;COI;;;;;
MH670131;Lepidoptera;COI;;;;;
MH670132;Lepidoptera;COI;;;;;
MH670133;Lepidoptera;COI;;;;;
MH670134;Lepidoptera;COI;;;;;
MH670135;Lepidoptera;COI;;;;;
MH670136;Lepidoptera;COI;;;;;
MH670138;Lepidoptera;COI;;;;;
MH670139;Lepidoptera;COI;;;;;
MH670140;Lepidoptera;COI;;;;;
MH670141;Lepidoptera;COI;;;;;
MH670142;Lepidoptera;COI;;;;;
MH670143;Lepidoptera;COI;;;;;
MH670145;Lepidoptera;COI;;;;;
MH670147;Lepidoptera;COI;;;;;
MH670149;Lepidoptera;COI;;;;;
MH670150;Lepidoptera;COI;;;;;
MH670152;Lepidoptera;COI;;;;;
MH670153;Lepidoptera;COI;;;;;

MH670154;Lepidoptera;COI;;;;;
MH670155;Lepidoptera;COI;;;;;
MH670156;Lepidoptera;COI;;;;;
MH670157;Lepidoptera;COI;;;;;
MH670158;Lepidoptera;COI;;;;;
MH670159;Lepidoptera;COI;;;;;
MH670161;Lepidoptera;COI;;;;;
MH670162;Lepidoptera;COI;;;;;
MH670163;Lepidoptera;COI;;;;;
MH670164;Lepidoptera;COI;;;;;
MH670166;Lepidoptera;COI;;;;;
MH670167;Lepidoptera;COI;;;;;
MH670168;Lepidoptera;COI;;;;;
MH670169;Lepidoptera;COI;;;;;
MH670170;Lepidoptera;COI;;;;;
MH670171;Lepidoptera;COI;;;;;
MH670172;Lepidoptera;COI;;;;;
MH670173;Lepidoptera;COI;;;;;
MH670174;Lepidoptera;COI;;;;;
MH670175;Lepidoptera;COI;;;;;
MH670176;Lepidoptera;COI;;;;;
MH670177;Lepidoptera;COI;;;;;
MH670178;Lepidoptera;COI;;;;;
MH670179;Lepidoptera;COI;;;;;
MH670180;Lepidoptera;COI;;;;;
MH670181;Lepidoptera;COI;;;;;
MH670182;Lepidoptera;COI;;;;;
MH670183;Lepidoptera;COI;;;;;
MH670184;Lepidoptera;COI;;;;;
MH670185;Lepidoptera;COI;;;;;
MH670188;Lepidoptera;COI;;;;;
MH670189;Lepidoptera;COI;;;;;
MH670191;Lepidoptera;COI;;;;;
MH670192;Lepidoptera;COI;;;;;
MH670193;Lepidoptera;COI;;;;;
MH670195;Lepidoptera;COI;;;;;
MH670196;Lepidoptera;COI;;;;;
MH670197;Lepidoptera;COI;;;;;
MH670199;Lepidoptera;COI;;;;;
MH670200;Lepidoptera;COI;;;;;
MH670201;Lepidoptera;COI;;;;;
MH670202;Lepidoptera;COI;;;;;
MH670203;Lepidoptera;COI;;;;;
MH670204;Lepidoptera;COI;;;;;
MH670205;Lepidoptera;COI;;;;;
MH670206;Lepidoptera;COI;;;;;
MH670207;Lepidoptera;COI;;;;;
MH670208;Lepidoptera;COI;;;;;
MH670209;Lepidoptera;COI;;;;;
MH670211;Lepidoptera;COI;;;;;
MH670212;Lepidoptera;COI;;;;;
MH670213;Lepidoptera;COI;;;;;
MH670214;Lepidoptera;COI;;;;;
MH670215;Lepidoptera;COI;;;;;
MH670216;Lepidoptera;COI;;;;;
MH670217;Lepidoptera;COI;;;;;
MH670218;Lepidoptera;COI;;;;;
MH670220;Lepidoptera;COI;;;;;
MH670221;Lepidoptera;COI;;;;;
MH670223;Lepidoptera;COI;;;;;
MH670224;Lepidoptera;COI;;;;;
MH670225;Lepidoptera;COI;;;;;
MH670226;Lepidoptera;COI;;;;;
MH670227;Lepidoptera;COI;;;;;
MH670228;Lepidoptera;COI;;;;;
MH670229;Lepidoptera;COI;;;;;
MH670230;Lepidoptera;COI;;;;;
MH670233;Lepidoptera;COI;;;;;
MH670234;Lepidoptera;COI;;;;;
MH670235;Lepidoptera;COI;;;;;
MH670236;Lepidoptera;COI;;;;;
MH670237;Lepidoptera;COI;;;;;
MH670238;Lepidoptera;COI;;;;;
MH670239;Lepidoptera;COI;;;;;
MH670240;Lepidoptera;COI;;;;;
MH670241;Lepidoptera;COI;;;;;
MK186078;Lepidoptera;COI;;;;;
MK186079;Lepidoptera;COI;;;;;

MK186197;Lepidoptera;COI;;;;;
MK186198;Lepidoptera;COI;;;;;
MK186319;Lepidoptera;COI;;;;;
MK186321;Lepidoptera;COI;;;;;
MK186322;Lepidoptera;COI;;;;;
MK186745;Lepidoptera;COI;;;;;
MK186746;Lepidoptera;COI;;;;;
MK186781;Lepidoptera;COI;;;;;
MK186783;Lepidoptera;COI;;;;;
MK186785;Lepidoptera;COI;;;;;
MK186786;Lepidoptera;COI;;;;;
MK186787;Lepidoptera;COI;;;;;
MK567857;Lepidoptera;COI;;;;;
MN138508;Lepidoptera;COI;;;;;
MN138671;Lepidoptera;COI;;;;;
MN138705;Lepidoptera;COI;;;;;
MN138724;Lepidoptera;COI;;;;;
MN138754;Lepidoptera;COI;;;;;
MN138769;Lepidoptera;COI;;;;;
MN138783;Lepidoptera;COI;;;;;
MN138802;Lepidoptera;COI;;;;;
MN138839;Lepidoptera;COI;;;;;
MN138865;Lepidoptera;COI;;;;;
MN138891;Lepidoptera;COI;;;;;
MN138948;Lepidoptera;COI;;;;;
MN139035;Lepidoptera;COI;;;;;
MN139068;Lepidoptera;COI;;;;;
MN139074;Lepidoptera;COI;;;;;
MN139101;Lepidoptera;COI;;;;;
MN139111;Lepidoptera;COI;;;;;
MN139211;Lepidoptera;COI;;;;;
MN139222;Lepidoptera;COI;;;;;
MN139243;Lepidoptera;COI;;;;;
MN139267;Lepidoptera;COI;;;;;
MN139284;Lepidoptera;COI;;;;;
MN139450;Lepidoptera;COI;;;;;
MN139458;Lepidoptera;COI;;;;;
MN139470;Lepidoptera;COI;;;;;
MN139477;Lepidoptera;COI;;;;;
MN139495;Lepidoptera;COI;;;;;
MN139525;Lepidoptera;COI;;;;;
MN139535;Lepidoptera;COI;;;;;
MN139549;Lepidoptera;COI;;;;;
MN139587;Lepidoptera;COI;;;;;
MN139609;Lepidoptera;COI;;;;;
MN139658;Lepidoptera;COI;;;;;
MN139670;Lepidoptera;COI;;;;;
MN139695;Lepidoptera;COI;;;;;
MN139702;Lepidoptera;COI;;;;;
MN139733;Lepidoptera;COI;;;;;
MN139748;Lepidoptera;COI;;;;;
MN139783;Lepidoptera;COI;;;;;
MN139784;Lepidoptera;COI;;;;;
MN139816;Lepidoptera;COI;;;;;
MN139817;Lepidoptera;COI;;;;;
MN139820;Lepidoptera;COI;;;;;
MN139886;Lepidoptera;COI;;;;;
MN139896;Lepidoptera;COI;;;;;
MN139902;Lepidoptera;COI;;;;;
MN139937;Lepidoptera;COI;;;;;
MN139943;Lepidoptera;COI;;;;;
MN140045;Lepidoptera;COI;;;;;
MN140051;Lepidoptera;COI;;;;;
MN140097;Lepidoptera;COI;;;;;
MN140124;Lepidoptera;COI;;;;;
MN140139;Lepidoptera;COI;;;;;
MN140169;Lepidoptera;COI;;;;;
MN140216;Lepidoptera;COI;;;;;
MN140249;Lepidoptera;COI;;;;;
MN140253;Lepidoptera;COI;;;;;
MN140260;Lepidoptera;COI;;;;;
MN140295;Lepidoptera;COI;;;;;
MN140350;Lepidoptera;COI;;;;;
MN140442;Lepidoptera;COI;;;;;
MN140452;Lepidoptera;COI;;;;;
MN140464;Lepidoptera;COI;;;;;
MN140479;Lepidoptera;COI;;;;;
MN140502;Lepidoptera;COI;;;;;

MN140540;Lepidoptera;COI;;;;;
MN140569;Lepidoptera;COI;;;;;
MN140584;Lepidoptera;COI;;;;;
MN140611;Lepidoptera;COI;;;;;
MN140648;Lepidoptera;COI;;;;;
MN140673;Lepidoptera;COI;;;;;
MN140699;Lepidoptera;COI;;;;;
MN140884;Lepidoptera;COI;;;;;
MN140888;Lepidoptera;COI;;;;;
MN140909;Lepidoptera;COI;;;;;
MN140911;Lepidoptera;COI;;;;;
MN140913;Lepidoptera;COI;;;;;
MN140927;Lepidoptera;COI;;;;;
MN140967;Lepidoptera;COI;;;;;
MN140971;Lepidoptera;COI;;;;;
MN140992;Lepidoptera;COI;;;;;
MN141054;Lepidoptera;COI;;;;;
MN141107;Lepidoptera;COI;;;;;
MN141116;Lepidoptera;COI;;;;;
MN141139;Lepidoptera;COI;;;;;
MN141151;Lepidoptera;COI;;;;;
MN141187;Lepidoptera;COI;;;;;
MN141225;Lepidoptera;COI;;;;;
MN141233;Lepidoptera;COI;;;;;
MN141234;Lepidoptera;COI;;;;;
MN141237;Lepidoptera;COI;;;;;
MN141261;Lepidoptera;COI;;;;;
MN141365;Lepidoptera;COI;;;;;
MN141433;Lepidoptera;COI;;;;;
MN141464;Lepidoptera;COI;;;;;
MN141554;Lepidoptera;COI;;;;;
MN141555;Lepidoptera;COI;;;;;
MN141559;Lepidoptera;COI;;;;;
MN141560;Lepidoptera;COI;;;;;
MN141575;Lepidoptera;COI;;;;;
MN141694;Lepidoptera;COI;;;;;
MN141708;Lepidoptera;COI;;;;;
MN141714;Lepidoptera;COI;;;;;
MN141802;Lepidoptera;COI;;;;;
MN141828;Lepidoptera;COI;;;;;
MN141839;Lepidoptera;COI;;;;;
MN141867;Lepidoptera;COI;;;;;
MN141885;Lepidoptera;COI;;;;;
MN141896;Lepidoptera;COI;;;;;
MN141920;Lepidoptera;COI;;;;;
MN141928;Lepidoptera;COI;;;;;
MN141947;Lepidoptera;COI;;;;;
MN142077;Lepidoptera;COI;;;;;
MN142166;Lepidoptera;COI;;;;;
MN142232;Lepidoptera;COI;;;;;
MN142263;Lepidoptera;COI;;;;;
MN142281;Lepidoptera;COI;;;;;
MN142331;Lepidoptera;COI;;;;;
MN142341;Lepidoptera;COI;;;;;
MN142369;Lepidoptera;COI;;;;;
MN142375;Lepidoptera;COI;;;;;
MN142382;Lepidoptera;COI;;;;;
MN142394;Lepidoptera;COI;;;;;
MN142442;Lepidoptera;COI;;;;;
MN142456;Lepidoptera;COI;;;;;
MN142472;Lepidoptera;COI;;;;;
MN142508;Lepidoptera;COI;;;;;
MN142551;Lepidoptera;COI;;;;;
MN142563;Lepidoptera;COI;;;;;
MN142568;Lepidoptera;COI;;;;;
MN142651;Lepidoptera;COI;;;;;
MN142678;Lepidoptera;COI;;;;;
MN142752;Lepidoptera;COI;;;;;
MN142779;Lepidoptera;COI;;;;;
MN142800;Lepidoptera;COI;;;;;
MN142809;Lepidoptera;COI;;;;;
MN142835;Lepidoptera;COI;;;;;
MN142886;Lepidoptera;COI;;;;;
MN142913;Lepidoptera;COI;;;;;
MN142966;Lepidoptera;COI;;;;;
MN142977;Lepidoptera;COI;;;;;
MN143082;Lepidoptera;COI;;;;;
MN143084;Lepidoptera;COI;;;;;

MN143153;Lepidoptera;COI;;;;;
MN143158;Lepidoptera;COI;;;;;
MN143159;Lepidoptera;COI;;;;;
MN143168;Lepidoptera;COI;;;;;
MN143219;Lepidoptera;COI;;;;;
MN143316;Lepidoptera;COI;;;;;
MN143353;Lepidoptera;COI;;;;;
MN143419;Lepidoptera;COI;;;;;
MN143421;Lepidoptera;COI;;;;;
MN143424;Lepidoptera;COI;;;;;
MN143485;Lepidoptera;COI;;;;;
MN143519;Lepidoptera;COI;;;;;
MN143569;Lepidoptera;COI;;;;;
MN143633;Lepidoptera;COI;;;;;
MN143664;Lepidoptera;COI;;;;;
MN143699;Lepidoptera;COI;;;;;
MN143706;Lepidoptera;COI;;;;;
MN143731;Lepidoptera;COI;;;;;
MN143733;Lepidoptera;COI;;;;;
MN143775;Lepidoptera;COI;;;;;
MN143835;Lepidoptera;COI;;;;;
MN143901;Lepidoptera;COI;;;;;
MN143943;Lepidoptera;COI;;;;;
MN143971;Lepidoptera;COI;;;;;
MN143986;Lepidoptera;COI;;;;;
MN144002;Lepidoptera;COI;;;;;
MN144019;Lepidoptera;COI;;;;;
MN144053;Lepidoptera;COI;;;;;
MN144112;Lepidoptera;COI;;;;;
MN144113;Lepidoptera;COI;;;;;
MN144215;Lepidoptera;COI;;;;;
MN144238;Lepidoptera;COI;;;;;
MN144278;Lepidoptera;COI;;;;;
MN144280;Lepidoptera;COI;;;;;
MN144326;Lepidoptera;COI;;;;;
MN144411;Lepidoptera;COI;;;;;
MN144414;Lepidoptera;COI;;;;;
MN144427;Lepidoptera;COI;;;;;
MN144482;Lepidoptera;COI;;;;;
MN144483;Lepidoptera;COI;;;;;
MN144494;Lepidoptera;COI;;;;;
MN144507;Lepidoptera;COI;;;;;
MN144645;Lepidoptera;COI;;;;;
MN144682;Lepidoptera;COI;;;;;
MN144698;Lepidoptera;COI;;;;;
MN144733;Lepidoptera;COI;;;;;
MN144761;Lepidoptera;COI;;;;;
MN144825;Lepidoptera;COI;;;;;
MN144842;Lepidoptera;COI;;;;;
MN144847;Lepidoptera;COI;;;;;
MN144849;Lepidoptera;COI;;;;;
MN144877;Lepidoptera;COI;;;;;
MN144959;Lepidoptera;COI;;;;;
MN145011;Lepidoptera;COI;;;;;
MN145029;Lepidoptera;COI;;;;;
MN145061;Lepidoptera;COI;;;;;
MN145114;Lepidoptera;COI;;;;;
MN145128;Lepidoptera;COI;;;;;
MN145157;Lepidoptera;COI;;;;;
MN145160;Lepidoptera;COI;;;;;
MN145244;Lepidoptera;COI;;;;;
MN145246;Lepidoptera;COI;;;;;
MN145263;Lepidoptera;COI;;;;;
MN145297;Lepidoptera;COI;;;;;
MN145318;Lepidoptera;COI;;;;;
MN145356;Lepidoptera;COI;;;;;
MN145387;Lepidoptera;COI;;;;;
MN145394;Lepidoptera;COI;;;;;
MN145413;Lepidoptera;COI;;;;;
MN322831;Lepidoptera;COI;;;;;
MN322832;Lepidoptera;COI;;;;;
MN322837;Lepidoptera;COI;;;;;
MN322849;Lepidoptera;COI;;;;;
MN322881;Lepidoptera;COI;;;;;
MT754706;Lepidoptera;COI;;;;;
MT754707;Lepidoptera;COI;;;;;
MT754708;Lepidoptera;COI;;;;;
MT763227;Lepidoptera;COI;;;;;

MT916623;Lepidoptera;COI;;;;;
MT916624;Lepidoptera;COI;;;;;
MT916625;Lepidoptera;COI;;;;;
MT916640;Lepidoptera;COI;;;;;
MT916641;Lepidoptera;COI;;;;;
MT916655;Lepidoptera;COI;;;;;
MT916656;Lepidoptera;COI;;;;;
MT916657;Lepidoptera;COI;;;;;
MW441622;Lepidoptera;COI;;;;;
MW441624;Lepidoptera;COI;;;;;
MW498994;Lepidoptera;COI;;;;;
MW499007;Lepidoptera;COI;;;;;
MW499020;Lepidoptera;COI;;;;;
MW499030;Lepidoptera;COI;;;;;
MW499088;Lepidoptera;COI;;;;;
MW499096;Lepidoptera;COI;;;;;
MW499120;Lepidoptera;COI;;;;;
MW499126;Lepidoptera;COI;;;;;
MW499211;Lepidoptera;COI;;;;;
MW499216;Lepidoptera;COI;;;;;
MW499307;Lepidoptera;COI;;;;;
MW499326;Lepidoptera;COI;;;;;
MW499333;Lepidoptera;COI;;;;;
MW499488;Lepidoptera;COI;;;;;
MW499538;Lepidoptera;COI;;;;;
MW499560;Lepidoptera;COI;;;;;
MW499561;Lepidoptera;COI;;;;;
MW499656;Lepidoptera;COI;;;;;
MW499700;Lepidoptera;COI;;;;;
MW499803;Lepidoptera;COI;;;;;
MW499824;Lepidoptera;COI;;;;;
MW499829;Lepidoptera;COI;;;;;
MW499833;Lepidoptera;COI;;;;;
MW499923;Lepidoptera;COI;;;;;
MW499957;Lepidoptera;COI;;;;;
MW500041;Lepidoptera;COI;;;;;
MW500158;Lepidoptera;COI;;;;;
MW500177;Lepidoptera;COI;;;;;
MW500191;Lepidoptera;COI;;;;;
MW500296;Lepidoptera;COI;;;;;
MW500388;Lepidoptera;COI;;;;;
MW500395;Lepidoptera;COI;;;;;
MW500413;Lepidoptera;COI;;;;;
MW500444;Lepidoptera;COI;;;;;
MW500519;Lepidoptera;COI;;;;;
MW500551;Lepidoptera;COI;;;;;
MW500622;Lepidoptera;COI;;;;;
MW500660;Lepidoptera;COI;;;;;
MW500717;Lepidoptera;COI;;;;;
MW500770;Lepidoptera;COI;;;;;
MW500821;Lepidoptera;COI;;;;;
MW500850;Lepidoptera;COI;;;;;
MW500949;Lepidoptera;COI;;;;;
MW500977;Lepidoptera;COI;;;;;
MW501187;Lepidoptera;COI;;;;;
MW501295;Lepidoptera;COI;;;;;
MW501356;Lepidoptera;COI;;;;;
MW501515;Lepidoptera;COI;;;;;
MW501521;Lepidoptera;COI;;;;;
MW501624;Lepidoptera;COI;;;;;
MW501627;Lepidoptera;COI;;;;;
MW501726;Lepidoptera;COI;;;;;
MW501777;Lepidoptera;COI;;;;;
MW501781;Lepidoptera;COI;;;;;
MW501816;Lepidoptera;COI;;;;;
MW501852;Lepidoptera;COI;;;;;
MW501962;Lepidoptera;COI;;;;;
MW502092;Lepidoptera;COI;;;;;
MW502134;Lepidoptera;COI;;;;;
MW502156;Lepidoptera;COI;;;;;
MW502248;Lepidoptera;COI;;;;;
MW502249;Lepidoptera;COI;;;;;
MW502346;Lepidoptera;COI;;;;;
MW502419;Lepidoptera;COI;;;;;
MW502424;Lepidoptera;COI;;;;;
MW502512;Lepidoptera;COI;;;;;
MW502528;Lepidoptera;COI;;;;;
MW502568;Lepidoptera;COI;;;;;

MW502593;Lepidoptera;COI;;;;;
MW502603;Lepidoptera;COI;;;;;
MW502651;Lepidoptera;COI;;;;;
MW502653;Lepidoptera;COI;;;;;
MW502687;Lepidoptera;COI;;;;;
MW502694;Lepidoptera;COI;;;;;
MW502838;Lepidoptera;COI;;;;;
MW502846;Lepidoptera;COI;;;;;
MW502855;Lepidoptera;COI;;;;;
MW502909;Lepidoptera;COI;;;;;
MW503028;Lepidoptera;COI;;;;;
MW503079;Lepidoptera;COI;;;;;
MW503097;Lepidoptera;COI;;;;;
MW503108;Lepidoptera;COI;;;;;
MW503130;Lepidoptera;COI;;;;;
MW503131;Lepidoptera;COI;;;;;
MW503168;Lepidoptera;COI;;;;;
MW503184;Lepidoptera;COI;;;;;
MW503250;Lepidoptera;COI;;;;;
MW503274;Lepidoptera;COI;;;;;
MW503354;Lepidoptera;COI;;;;;
MW503389;Lepidoptera;COI;;;;;
MW503436;Lepidoptera;COI;;;;;
MW503450;Lepidoptera;COI;;;;;
MW503524;Lepidoptera;COI;;;;;
MW503592;Lepidoptera;COI;;;;;
MW503626;Lepidoptera;COI;;;;;
MW503687;Lepidoptera;COI;;;;;
MW661300;Lepidoptera;COI;;;;;
MW861639;Lepidoptera;COI;;;;;
MW861640;Lepidoptera;COI;;;;;
MW861641;Lepidoptera;COI;;;;;
MW861642;Lepidoptera;COI;;;;;
MW861643;Lepidoptera;COI;;;;;
MW861645;Lepidoptera;COI;;;;;
MW861647;Lepidoptera;COI;;;;;
MW861648;Lepidoptera;COI;;;;;
MW861649;Lepidoptera;COI;;;;;
MW861650;Lepidoptera;COI;;;;;
MW861651;Lepidoptera;COI;;;;;
MW861652;Lepidoptera;COI;;;;;
MW861653;Lepidoptera;COI;;;;;
MW861654;Lepidoptera;COI;;;;;
MW861655;Lepidoptera;COI;;;;;
MW861656;Lepidoptera;COI;;;;;
MW861658;Lepidoptera;COI;;;;;
MW861659;Lepidoptera;COI;;;;;
MW861662;Lepidoptera;COI;;;;;
MW861663;Lepidoptera;COI;;;;;
MW861664;Lepidoptera;COI;;;;;
MW861668;Lepidoptera;COI;;;;;
MW861669;Lepidoptera;COI;;;;;
MW861674;Lepidoptera;COI;;;;;
MW861675;Lepidoptera;COI;;;;;
MW861676;Lepidoptera;COI;;;;;
MW861677;Lepidoptera;COI;;;;;
MW861678;Lepidoptera;COI;;;;;
MW861679;Lepidoptera;COI;;;;;
MW861680;Lepidoptera;COI;;;;;
MZ026159;Lepidoptera;COI;;;;;
MZ026160;Lepidoptera;COI;;;;;
MZ026161;Lepidoptera;COI;;;;;
MZ026176;Lepidoptera;COI;;;;;
MZ026177;Lepidoptera;COI;;;;;
MZ026178;Lepidoptera;COI;;;;;
MZ026179;Lepidoptera;COI;;;;;
MZ026180;Lepidoptera;COI;;;;;
MZ026181;Lepidoptera;COI;;;;;
MZ026182;Lepidoptera;COI;;;;;
MZ026183;Lepidoptera;COI;;;;;
MZ026184;Lepidoptera;COI;;;;;
MZ026185;Lepidoptera;COI;;;;;
MZ026186;Lepidoptera;COI;;;;;
MZ026187;Lepidoptera;COI;;;;;
MZ026188;Lepidoptera;COI;;;;;
MZ026189;Lepidoptera;COI;;;;;
MZ026190;Lepidoptera;COI;;;;;
MZ128131;Lepidoptera;COI;;;;;

MZ128132;Lepidoptera;COI;;;;;
OK345544;Lepidoptera;COI;;;;;
OK345598;Lepidoptera;COI;;;;;
OK345599;Lepidoptera;COI;;;;;
ON435776;Lepidoptera;COI;;;;;
ON436602;Lepidoptera;COI;;;;;
ON436691;Lepidoptera;COI;;;;;
ON436710;Lepidoptera;COI;;;;;
ON436711;Lepidoptera;COI;;;;;
ON436713;Lepidoptera;COI;;;;;
ON437050;Lepidoptera;COI;;;;;
OP430995;Lepidoptera;COI;;;;;
OQ121629;Lepidoptera;COI;;;;;
OQ121630;Lepidoptera;COI;;;;;
OQ121631;Lepidoptera;COI;;;;;
OQ121632;Lepidoptera;COI;;;;;
OQ121633;Lepidoptera;COI;;;;;
OQ121634;Lepidoptera;COI;;;;;
OQ121635;Lepidoptera;COI;;;;;
OQ121636;Lepidoptera;COI;;;;;
OQ121637;Lepidoptera;COI;;;;;
OQ121638;Lepidoptera;COI;;;;;
OQ121639;Lepidoptera;COI;;;;;
OQ121640;Lepidoptera;COI;;;;;
OQ121641;Lepidoptera;COI;;;;;
OQ121642;Lepidoptera;COI;;;;;
OQ121643;Lepidoptera;COI;;;;;
OQ121644;Lepidoptera;COI;;;;;
OQ121645;Lepidoptera;COI;;;;;
OQ121646;Lepidoptera;COI;;;;;
OQ121647;Lepidoptera;COI;;;;;
OQ121648;Lepidoptera;COI;;;;;
OQ121649;Lepidoptera;COI;;;;;
OQ121650;Lepidoptera;COI;;;;;
OQ121651;Lepidoptera;COI;;;;;
OQ121652;Lepidoptera;COI;;;;;
OQ121653;Lepidoptera;COI;;;;;
OQ121654;Lepidoptera;COI;;;;;
OQ121655;Lepidoptera;COI;;;;;
OQ121656;Lepidoptera;COI;;;;;
OQ121657;Lepidoptera;COI;;;;;
OQ182347;Lepidoptera;COI;;;;;
OQ182833;Lepidoptera;COI;;;;;
HM901883;Odonata;COI;;;;;
KF584932;Odonata;COI;;;;;
KF584956;Odonata;COI;;;;;
KF584957;Odonata;COI;;;;;
KF584959;Odonata;COI;;;;;
KF584960;Odonata;COI;;;;;
KF584961;Odonata;COI;;;;;
KF584962;Odonata;COI;;;;;
KF584964;Odonata;COI;;;;;
KF584965;Odonata;COI;;;;;
KF584967;Odonata;COI;;;;;
KF584968;Odonata;COI;;;;;
LC366771;Odonata;COI;;;;;
MN701478;Odonata;COI;;;;;
MN701481;Odonata;COI;;;;;
MN701482;Odonata;COI;;;;;
MN701483;Odonata;COI;;;;;
MN701486;Odonata;COI;;;;;
MN701487;Odonata;COI;;;;;
MN701491;Odonata;COI;;;;;
MN701492;Odonata;COI;;;;;
MN701493;Odonata;COI;;;;;
MN701496;Odonata;COI;;;;;
MN701497;Odonata;COI;;;;;
MN701498;Odonata;COI;;;;;
MN701499;Odonata;COI;;;;;
MN701500;Odonata;COI;;;;;
MN701502;Odonata;COI;;;;;
MN701504;Odonata;COI;;;;;
MN701505;Odonata;COI;;;;;
MN701507;Odonata;COI;;;;;
MN701508;Odonata;COI;;;;;
MN701510;Odonata;COI;;;;;
MN701512;Odonata;COI;;;;;
MN701516;Odonata;COI;;;;;

MN701517;Odonata;COI;;;;;
MN701519;Odonata;COI;;;;;
MN701520;Odonata;COI;;;;;
MN701521;Odonata;COI;;;;;
MN701523;Odonata;COI;;;;;
MN701525;Odonata;COI;;;;;
MN701528;Odonata;COI;;;;;
MN701530;Odonata;COI;;;;;
MN701531;Odonata;COI;;;;;
MN701532;Odonata;COI;;;;;
MN701536;Odonata;COI;;;;;
MN701537;Odonata;COI;;;;;
MN701541;Odonata;COI;;;;;
MN701542;Odonata;COI;;;;;
MN701543;Odonata;COI;;;;;
MN701548;Odonata;COI;;;;;
MN701555;Odonata;COI;;;;;
MN701556;Odonata;COI;;;;;
MN701560;Odonata;COI;;;;;
MN731360;Odonata;COI;;;;;
MN735552;Odonata;COI;;;;;
MN957949;Odonata;COI;;;;;
MT298235;Odonata;COI;;;;;
MT298253;Odonata;COI;;;;;
MT298254;Odonata;COI;;;;;
MT298255;Odonata;COI;;;;;
MT298256;Odonata;COI;;;;;
MT298257;Odonata;COI;;;;;
MT298258;Odonata;COI;;;;;
MT298259;Odonata;COI;;;;;
MT298260;Odonata;COI;;;;;
MT298296;Odonata;COI;;;;;
MT298297;Odonata;COI;;;;;
MT298312;Odonata;COI;;;;;
MT298470;Odonata;COI;;;;;
MT298471;Odonata;COI;;;;;
MT298472;Odonata;COI;;;;;
MT298620;Odonata;COI;;;;;
MT298621;Odonata;COI;;;;;
MT298622;Odonata;COI;;;;;
MT298623;Odonata;COI;;;;;
MT298624;Odonata;COI;;;;;
MT298625;Odonata;COI;;;;;
MT298626;Odonata;COI;;;;;
MT298627;Odonata;COI;;;;;
MT298628;Odonata;COI;;;;;
MT298633;Odonata;COI;;;;;
MW139673;Odonata;COI;;;;;
MW208373;Odonata;COI;;;;;
MW208374;Odonata;COI;;;;;
MW208391;Odonata;COI;;;;;
MW208393;Odonata;COI;;;;;
MW208394;Odonata;COI;;;;;
MW208396;Odonata;COI;;;;;
MW208397;Odonata;COI;;;;;
MW208398;Odonata;COI;;;;;
MW208399;Odonata;COI;;;;;
MW208402;Odonata;COI;;;;;
MW208403;Odonata;COI;;;;;
MW208404;Odonata;COI;;;;;
MW208405;Odonata;COI;;;;;
MW208413;Odonata;COI;;;;;
MW208414;Odonata;COI;;;;;
MW208415;Odonata;COI;;;;;
MW490125;Odonata;COI;;;;;
MW490140;Odonata;COI;;;;;
MW490172;Odonata;COI;;;;;
MW490232;Odonata;COI;;;;;
MW490272;Odonata;COI;;;;;
MW490283;Odonata;COI;;;;;
MW490299;Odonata;COI;;;;;
MW490352;Odonata;COI;;;;;
MW490464;Odonata;COI;;;;;
MW490466;Odonata;COI;;;;;
MW490490;Odonata;COI;;;;;
MW490509;Odonata;COI;;;;;
MW490549;Odonata;COI;;;;;
MW570923;Odonata;COI;;;;;

MW570924;Odonata;COI;;;;;
MW570925;Odonata;COI;;;;;
MZ656557;Odonata;COI;;;;;
NC_031824;Odonata;COI;;;;;
ON003482;Odonata;COI;;;;;
ON003483;Odonata;COI;;;;;
ON003484;Odonata;COI;;;;;
ON003485;Odonata;COI;;;;;
AJ880679;Coleoptera;COI;;;;;
AJ880680;Coleoptera;COI;;;;;
AJ880681;Coleoptera;COI;;;;;
AJ880682;Coleoptera;COI;;;;;
AJ880683;Coleoptera;COI;;;;;
AM412372;Coleoptera;COI;;;;;
AM412373;Coleoptera;COI;;;;;
AM412374;Coleoptera;COI;;;;;
AM412375;Coleoptera;COI;;;;;
AM412376;Coleoptera;COI;;;;;
AM412377;Coleoptera;COI;;;;;
AM412382;Coleoptera;COI;;;;;
AM412383;Coleoptera;COI;;;;;
AM423158;Coleoptera;COI;;;;;
AM423159;Coleoptera;COI;;;;;
EU286487;Coleoptera;COI;;;;;
EU286489;Coleoptera;COI;;;;;
EU286505;Coleoptera;COI;;;;;
EU286509;Coleoptera;COI;;;;;
EU752053;Coleoptera;COI;;;;;
EU752054;Coleoptera;COI;;;;;
FM877915;Coleoptera;COI;;;;;
GQ980935;Coleoptera;COI;;;;;
GU213646;Coleoptera;COI;;;;;
GU981473;Coleoptera;COI;;;;;
GU981476;Coleoptera;COI;;;;;
GU981477;Coleoptera;COI;;;;;
GU981478;Coleoptera;COI;;;;;
GU981480;Coleoptera;COI;;;;;
GU981481;Coleoptera;COI;;;;;
GU981489;Coleoptera;COI;;;;;
GU981497;Coleoptera;COI;;;;;
GU981503;Coleoptera;COI;;;;;
GU981505;Coleoptera;COI;;;;;
GU987824;Coleoptera;COI;;;;;
GU987830;Coleoptera;COI;;;;;
GU987835;Coleoptera;COI;;;;;
GU987893;Coleoptera;COI;;;;;
GU988053;Coleoptera;COI;;;;;
GU988056;Coleoptera;COI;;;;;
HM909037;Coleoptera;COI;;;;;
HQ559243;Coleoptera;COI;;;;;
HQ559244;Coleoptera;COI;;;;;
HQ559246;Coleoptera;COI;;;;;
HQ559265;Coleoptera;COI;;;;;
HQ948196;Coleoptera;COI;;;;;
HQ948261;Coleoptera;COI;;;;;
HQ953393;Coleoptera;COI;;;;;
HQ953425;Coleoptera;COI;;;;;
HQ953467;Coleoptera;COI;;;;;
HQ953500;Coleoptera;COI;;;;;
HQ953648;Coleoptera;COI;;;;;
HQ953774;Coleoptera;COI;;;;;
HQ953775;Coleoptera;COI;;;;;
HQ953825;Coleoptera;COI;;;;;
HQ953942;Coleoptera;COI;;;;;
HQ953943;Coleoptera;COI;;;;;
HQ953981;Coleoptera;COI;;;;;
HQ953997;Coleoptera;COI;;;;;
HQ953998;Coleoptera;COI;;;;;
HQ954089;Coleoptera;COI;;;;;
HQ954277;Coleoptera;COI;;;;;
HQ954583;Coleoptera;COI;;;;;
HQ954593;Coleoptera;COI;;;;;
JF888650;Coleoptera;COI;;;;;
JF888651;Coleoptera;COI;;;;;
JF889535;Coleoptera;COI;;;;;
JF889554;Coleoptera;COI;;;;;
JF889566;Coleoptera;COI;;;;;
JF889567;Coleoptera;COI;;;;;

JF889659;Coleoptera;COI;;;;;
JN262175;Coleoptera;COI;;;;;
JN273792;Coleoptera;COI;;;;;
JN299247;Coleoptera;COI;;;;;
JX263818;Coleoptera;COI;;;;;
JX626112;Coleoptera;COI;;;;;
JX626113;Coleoptera;COI;;;;;
JX626114;Coleoptera;COI;;;;;
JX626115;Coleoptera;COI;;;;;
JX626116;Coleoptera;COI;;;;;
JX626117;Coleoptera;COI;;;;;
JX626118;Coleoptera;COI;;;;;
KC845519;Coleoptera;COI;;;;;
KJ492948;Coleoptera;COI;;;;;
KJ492951;Coleoptera;COI;;;;;
KJ492954;Coleoptera;COI;;;;;
KJ492957;Coleoptera;COI;;;;;
KJ492958;Coleoptera;COI;;;;;
KJ492960;Coleoptera;COI;;;;;
KJ492961;Coleoptera;COI;;;;;
KJ492962;Coleoptera;COI;;;;;
KJ867614;Coleoptera;COI;;;;;
KJ867618;Coleoptera;COI;;;;;
KJ867620;Coleoptera;COI;;;;;
KJ961900;Coleoptera;COI;;;;;
KJ961975;Coleoptera;COI;;;;;
KJ962037;Coleoptera;COI;;;;;
KJ962090;Coleoptera;COI;;;;;
KJ962180;Coleoptera;COI;;;;;
KJ962401;Coleoptera;COI;;;;;
KJ962475;Coleoptera;COI;;;;;
KJ962500;Coleoptera;COI;;;;;
KJ962583;Coleoptera;COI;;;;;
KJ962723;Coleoptera;COI;;;;;
KJ962746;Coleoptera;COI;;;;;
KJ962762;Coleoptera;COI;;;;;
KJ962811;Coleoptera;COI;;;;;
KJ962863;Coleoptera;COI;;;;;
KJ962879;Coleoptera;COI;;;;;
KJ962902;Coleoptera;COI;;;;;
KJ962996;Coleoptera;COI;;;;;
KJ963072;Coleoptera;COI;;;;;
KJ963362;Coleoptera;COI;;;;;
KJ963373;Coleoptera;COI;;;;;
KJ963442;Coleoptera;COI;;;;;
KJ963643;Coleoptera;COI;;;;;
KJ963709;Coleoptera;COI;;;;;
KJ963853;Coleoptera;COI;;;;;
KJ963967;Coleoptera;COI;;;;;
KJ964096;Coleoptera;COI;;;;;
KJ964133;Coleoptera;COI;;;;;
KJ964157;Coleoptera;COI;;;;;
KJ964255;Coleoptera;COI;;;;;
KJ964754;Coleoptera;COI;;;;;
KJ964816;Coleoptera;COI;;;;;
KJ964959;Coleoptera;COI;;;;;
KJ965077;Coleoptera;COI;;;;;
KJ965337;Coleoptera;COI;;;;;
KJ965399;Coleoptera;COI;;;;;
KJ966027;Coleoptera;COI;;;;;
KJ966062;Coleoptera;COI;;;;;
KJ966244;Coleoptera;COI;;;;;
KJ966292;Coleoptera;COI;;;;;
KJ966333;Coleoptera;COI;;;;;
KJ966409;Coleoptera;COI;;;;;
KJ967156;Coleoptera;COI;;;;;
KJ967190;Coleoptera;COI;;;;;
KJ967255;Coleoptera;COI;;;;;
KJ967279;Coleoptera;COI;;;;;
KJ967370;Coleoptera;COI;;;;;
KJ967455;Coleoptera;COI;;;;;
KJ967464;Coleoptera;COI;;;;;
KM285754;Coleoptera;COI;;;;;
KM285770;Coleoptera;COI;;;;;
KM285790;Coleoptera;COI;;;;;
KM285825;Coleoptera;COI;;;;;
KM285826;Coleoptera;COI;;;;;
KM285854;Coleoptera;COI;;;;;

KM285862;Coleoptera;COI;;;;;
KM285871;Coleoptera;COI;;;;;
KM285876;Coleoptera;COI;;;;;
KM285884;Coleoptera;COI;;;;;
KM285895;Coleoptera;COI;;;;;
KM285896;Coleoptera;COI;;;;;
KM285911;Coleoptera;COI;;;;;
KM285916;Coleoptera;COI;;;;;
KM285922;Coleoptera;COI;;;;;
KM285927;Coleoptera;COI;;;;;
KM285933;Coleoptera;COI;;;;;
KM285955;Coleoptera;COI;;;;;
KM285958;Coleoptera;COI;;;;;
KM285961;Coleoptera;COI;;;;;
KM285962;Coleoptera;COI;;;;;
KM285990;Coleoptera;COI;;;;;
KM286008;Coleoptera;COI;;;;;
KM286018;Coleoptera;COI;;;;;
KM286040;Coleoptera;COI;;;;;
KM286044;Coleoptera;COI;;;;;
KM286048;Coleoptera;COI;;;;;
KM286065;Coleoptera;COI;;;;;
KM286072;Coleoptera;COI;;;;;
KM286098;Coleoptera;COI;;;;;
KM286111;Coleoptera;COI;;;;;
KM286123;Coleoptera;COI;;;;;
KM286124;Coleoptera;COI;;;;;
KM286128;Coleoptera;COI;;;;;
KM286130;Coleoptera;COI;;;;;
KM286139;Coleoptera;COI;;;;;
KM286145;Coleoptera;COI;;;;;
KM286170;Coleoptera;COI;;;;;
KM286178;Coleoptera;COI;;;;;
KM286179;Coleoptera;COI;;;;;
KM286201;Coleoptera;COI;;;;;
KM286230;Coleoptera;COI;;;;;
KM286244;Coleoptera;COI;;;;;
KM286257;Coleoptera;COI;;;;;
KM286260;Coleoptera;COI;;;;;
KM286274;Coleoptera;COI;;;;;
KM286280;Coleoptera;COI;;;;;
KM286300;Coleoptera;COI;;;;;
KM286309;Coleoptera;COI;;;;;
KM286311;Coleoptera;COI;;;;;
KM286337;Coleoptera;COI;;;;;
KM286355;Coleoptera;COI;;;;;
KM286359;Coleoptera;COI;;;;;
KM286361;Coleoptera;COI;;;;;
KM286364;Coleoptera;COI;;;;;
KM286382;Coleoptera;COI;;;;;
KM286385;Coleoptera;COI;;;;;
KM286391;Coleoptera;COI;;;;;
KM286395;Coleoptera;COI;;;;;
KM286407;Coleoptera;COI;;;;;
KM439150;Coleoptera;COI;;;;;
KM439242;Coleoptera;COI;;;;;
KM439248;Coleoptera;COI;;;;;
KM439264;Coleoptera;COI;;;;;
KM439286;Coleoptera;COI;;;;;
KM439430;Coleoptera;COI;;;;;
KM439431;Coleoptera;COI;;;;;
KM439473;Coleoptera;COI;;;;;
KM439607;Coleoptera;COI;;;;;
KM439647;Coleoptera;COI;;;;;
KM439667;Coleoptera;COI;;;;;
KM439807;Coleoptera;COI;;;;;
KM440112;Coleoptera;COI;;;;;
KM440221;Coleoptera;COI;;;;;
KM440330;Coleoptera;COI;;;;;
KM440379;Coleoptera;COI;;;;;
KM440402;Coleoptera;COI;;;;;
KM440427;Coleoptera;COI;;;;;
KM440441;Coleoptera;COI;;;;;
KM440468;Coleoptera;COI;;;;;
KM440548;Coleoptera;COI;;;;;
KM440687;Coleoptera;COI;;;;;
KM440920;Coleoptera;COI;;;;;
KM441026;Coleoptera;COI;;;;;

KM441040;Coleoptera;COI;;;;;
KM441079;Coleoptera;COI;;;;;
KM441127;Coleoptera;COI;;;;;
KM441301;Coleoptera;COI;;;;;
KM441453;Coleoptera;COI;;;;;
KM441534;Coleoptera;COI;;;;;
KM441572;Coleoptera;COI;;;;;
KM441741;Coleoptera;COI;;;;;
KM441749;Coleoptera;COI;;;;;
KM441850;Coleoptera;COI;;;;;
KM442023;Coleoptera;COI;;;;;
KM442328;Coleoptera;COI;;;;;
KM442364;Coleoptera;COI;;;;;
KM442509;Coleoptera;COI;;;;;
KM442770;Coleoptera;COI;;;;;
KM442950;Coleoptera;COI;;;;;
KM443253;Coleoptera;COI;;;;;
KM443370;Coleoptera;COI;;;;;
KM443372;Coleoptera;COI;;;;;
KM443460;Coleoptera;COI;;;;;
KM443511;Coleoptera;COI;;;;;
KM443719;Coleoptera;COI;;;;;
KM443740;Coleoptera;COI;;;;;
KM443834;Coleoptera;COI;;;;;
KM443844;Coleoptera;COI;;;;;
KM443869;Coleoptera;COI;;;;;
KM444488;Coleoptera;COI;;;;;
KM444591;Coleoptera;COI;;;;;
KM444596;Coleoptera;COI;;;;;
KM444710;Coleoptera;COI;;;;;
KM444798;Coleoptera;COI;;;;;
KM445044;Coleoptera;COI;;;;;
KM445092;Coleoptera;COI;;;;;
KM445144;Coleoptera;COI;;;;;
KM445160;Coleoptera;COI;;;;;
KM445305;Coleoptera;COI;;;;;
KM445321;Coleoptera;COI;;;;;
KM445412;Coleoptera;COI;;;;;
KM445499;Coleoptera;COI;;;;;
KM445619;Coleoptera;COI;;;;;
KM445722;Coleoptera;COI;;;;;
KM445749;Coleoptera;COI;;;;;
KM445822;Coleoptera;COI;;;;;
KM445977;Coleoptera;COI;;;;;
KM445979;Coleoptera;COI;;;;;
KM445982;Coleoptera;COI;;;;;
KM446033;Coleoptera;COI;;;;;
KM446060;Coleoptera;COI;;;;;
KM446068;Coleoptera;COI;;;;;
KM446120;Coleoptera;COI;;;;;
KM446189;Coleoptera;COI;;;;;
KM446254;Coleoptera;COI;;;;;
KM446613;Coleoptera;COI;;;;;
KM446627;Coleoptera;COI;;;;;
KM446636;Coleoptera;COI;;;;;
KM446650;Coleoptera;COI;;;;;
KM446656;Coleoptera;COI;;;;;
KM446734;Coleoptera;COI;;;;;
KM446739;Coleoptera;COI;;;;;
KM446741;Coleoptera;COI;;;;;
KM446786;Coleoptera;COI;;;;;
KM446847;Coleoptera;COI;;;;;
KM446864;Coleoptera;COI;;;;;
KM446951;Coleoptera;COI;;;;;
KM447220;Coleoptera;COI;;;;;
KM447234;Coleoptera;COI;;;;;
KM447314;Coleoptera;COI;;;;;
KM447419;Coleoptera;COI;;;;;
KM447434;Coleoptera;COI;;;;;
KM447455;Coleoptera;COI;;;;;
KM447639;Coleoptera;COI;;;;;
KM447694;Coleoptera;COI;;;;;
KM447844;Coleoptera;COI;;;;;
KM447881;Coleoptera;COI;;;;;
KM447966;Coleoptera;COI;;;;;
KM448032;Coleoptera;COI;;;;;
KM448148;Coleoptera;COI;;;;;
KM448321;Coleoptera;COI;;;;;

KM448324;Coleoptera;COI;;;;;
KM448343;Coleoptera;COI;;;;;
KM448408;Coleoptera;COI;;;;;
KM448468;Coleoptera;COI;;;;;
KM448542;Coleoptera;COI;;;;;
KM448634;Coleoptera;COI;;;;;
KM448655;Coleoptera;COI;;;;;
KM448674;Coleoptera;COI;;;;;
KM448711;Coleoptera;COI;;;;;
KM448852;Coleoptera;COI;;;;;
KM448870;Coleoptera;COI;;;;;
KM448924;Coleoptera;COI;;;;;
KM448950;Coleoptera;COI;;;;;
KM449354;Coleoptera;COI;;;;;
KM449448;Coleoptera;COI;;;;;
KM449464;Coleoptera;COI;;;;;
KM449562;Coleoptera;COI;;;;;
KM449563;Coleoptera;COI;;;;;
KM449578;Coleoptera;COI;;;;;
KM449732;Coleoptera;COI;;;;;
KM449883;Coleoptera;COI;;;;;
KM449954;Coleoptera;COI;;;;;
KM450044;Coleoptera;COI;;;;;
KM450081;Coleoptera;COI;;;;;
KM450085;Coleoptera;COI;;;;;
KM450097;Coleoptera;COI;;;;;
KM450252;Coleoptera;COI;;;;;
KM450289;Coleoptera;COI;;;;;
KM450340;Coleoptera;COI;;;;;
KM450511;Coleoptera;COI;;;;;
KM450547;Coleoptera;COI;;;;;
KM450616;Coleoptera;COI;;;;;
KM450689;Coleoptera;COI;;;;;
KM450747;Coleoptera;COI;;;;;
KM450844;Coleoptera;COI;;;;;
KM450969;Coleoptera;COI;;;;;
KM451196;Coleoptera;COI;;;;;
KM451230;Coleoptera;COI;;;;;
KM451238;Coleoptera;COI;;;;;
KM451249;Coleoptera;COI;;;;;
KM451541;Coleoptera;COI;;;;;
KM451559;Coleoptera;COI;;;;;
KM451616;Coleoptera;COI;;;;;
KM451718;Coleoptera;COI;;;;;
KM451730;Coleoptera;COI;;;;;
KM451750;Coleoptera;COI;;;;;
KM451956;Coleoptera;COI;;;;;
KM452312;Coleoptera;COI;;;;;
KM452381;Coleoptera;COI;;;;;
KM452392;Coleoptera;COI;;;;;
KM452478;Coleoptera;COI;;;;;
KM452489;Coleoptera;COI;;;;;
KM452694;Coleoptera;COI;;;;;
KU170190;Coleoptera;COI;;;;;
KU906139;Coleoptera;COI;;;;;
KU906180;Coleoptera;COI;;;;;
KU906235;Coleoptera;COI;;;;;
KU906270;Coleoptera;COI;;;;;
KU906295;Coleoptera;COI;;;;;
KU906323;Coleoptera;COI;;;;;
KU906361;Coleoptera;COI;;;;;
KU906428;Coleoptera;COI;;;;;
KU906616;Coleoptera;COI;;;;;
KU906711;Coleoptera;COI;;;;;
KU906771;Coleoptera;COI;;;;;
KU906952;Coleoptera;COI;;;;;
KU906954;Coleoptera;COI;;;;;
KU907158;Coleoptera;COI;;;;;
KU907229;Coleoptera;COI;;;;;
KU907248;Coleoptera;COI;;;;;
KU907272;Coleoptera;COI;;;;;
KU907300;Coleoptera;COI;;;;;
KU907334;Coleoptera;COI;;;;;
KU907343;Coleoptera;COI;;;;;
KU907476;Coleoptera;COI;;;;;
KU907536;Coleoptera;COI;;;;;
KU907587;Coleoptera;COI;;;;;
KU907752;Coleoptera;COI;;;;;

KU907781;Coleoptera;COI;;;;;
KU907805;Coleoptera;COI;;;;;
KU907927;Coleoptera;COI;;;;;
KU907975;Coleoptera;COI;;;;;
KU908166;Coleoptera;COI;;;;;
KU908314;Coleoptera;COI;;;;;
KU908434;Coleoptera;COI;;;;;
KU908589;Coleoptera;COI;;;;;
KU908735;Coleoptera;COI;;;;;
KU908807;Coleoptera;COI;;;;;
KU908820;Coleoptera;COI;;;;;
KU908989;Coleoptera;COI;;;;;
KU909045;Coleoptera;COI;;;;;
KU909073;Coleoptera;COI;;;;;
KU909169;Coleoptera;COI;;;;;
KU909290;Coleoptera;COI;;;;;
KU909323;Coleoptera;COI;;;;;
KU909483;Coleoptera;COI;;;;;
KU909583;Coleoptera;COI;;;;;
KU909639;Coleoptera;COI;;;;;
KU909698;Coleoptera;COI;;;;;
KU909765;Coleoptera;COI;;;;;
KU909825;Coleoptera;COI;;;;;
KU909832;Coleoptera;COI;;;;;
KU910033;Coleoptera;COI;;;;;
KU910422;Coleoptera;COI;;;;;
KU910678;Coleoptera;COI;;;;;
KU910698;Coleoptera;COI;;;;;
KU910793;Coleoptera;COI;;;;;
KU910837;Coleoptera;COI;;;;;
KU911123;Coleoptera;COI;;;;;
KU911153;Coleoptera;COI;;;;;
KU911205;Coleoptera;COI;;;;;
KU911284;Coleoptera;COI;;;;;
KU911352;Coleoptera;COI;;;;;
KU911368;Coleoptera;COI;;;;;
KU911394;Coleoptera;COI;;;;;
KU911462;Coleoptera;COI;;;;;
KU911487;Coleoptera;COI;;;;;
KU911511;Coleoptera;COI;;;;;
KU911598;Coleoptera;COI;;;;;
KU911599;Coleoptera;COI;;;;;
KU911641;Coleoptera;COI;;;;;
KU911744;Coleoptera;COI;;;;;
KU911828;Coleoptera;COI;;;;;
KU911898;Coleoptera;COI;;;;;
KU912029;Coleoptera;COI;;;;;
KU912067;Coleoptera;COI;;;;;
KU912238;Coleoptera;COI;;;;;
KU912320;Coleoptera;COI;;;;;
KU912341;Coleoptera;COI;;;;;
KU912402;Coleoptera;COI;;;;;
KU912448;Coleoptera;COI;;;;;
KU912497;Coleoptera;COI;;;;;
KU912587;Coleoptera;COI;;;;;
KU912590;Coleoptera;COI;;;;;
KU912628;Coleoptera;COI;;;;;
KU912632;Coleoptera;COI;;;;;
KU912765;Coleoptera;COI;;;;;
KU912857;Coleoptera;COI;;;;;
KU912904;Coleoptera;COI;;;;;
KU913033;Coleoptera;COI;;;;;
KU913239;Coleoptera;COI;;;;;
KU913568;Coleoptera;COI;;;;;
KU913603;Coleoptera;COI;;;;;
KU913641;Coleoptera;COI;;;;;
KU913757;Coleoptera;COI;;;;;
KU913831;Coleoptera;COI;;;;;
KU913896;Coleoptera;COI;;;;;
KU913949;Coleoptera;COI;;;;;
KU913976;Coleoptera;COI;;;;;
KU914029;Coleoptera;COI;;;;;
KU914041;Coleoptera;COI;;;;;
KU914257;Coleoptera;COI;;;;;
KU914270;Coleoptera;COI;;;;;
KU914328;Coleoptera;COI;;;;;
KU914402;Coleoptera;COI;;;;;
KU914532;Coleoptera;COI;;;;;

KU914613;Coleoptera;COI;;;;;
KU914853;Coleoptera;COI;;;;;
KU914935;Coleoptera;COI;;;;;
KU915162;Coleoptera;COI;;;;;
KU915172;Coleoptera;COI;;;;;
KU915290;Coleoptera;COI;;;;;
KU915291;Coleoptera;COI;;;;;
KU915695;Coleoptera;COI;;;;;
KU915733;Coleoptera;COI;;;;;
KU915740;Coleoptera;COI;;;;;
KU915770;Coleoptera;COI;;;;;
KU915984;Coleoptera;COI;;;;;
KU916000;Coleoptera;COI;;;;;
KU916070;Coleoptera;COI;;;;;
KU916112;Coleoptera;COI;;;;;
KU916245;Coleoptera;COI;;;;;
KU916299;Coleoptera;COI;;;;;
KU916404;Coleoptera;COI;;;;;
KU916410;Coleoptera;COI;;;;;
KU916495;Coleoptera;COI;;;;;
KU916546;Coleoptera;COI;;;;;
KU916555;Coleoptera;COI;;;;;
KU916626;Coleoptera;COI;;;;;
KU916657;Coleoptera;COI;;;;;
KU916696;Coleoptera;COI;;;;;
KU916762;Coleoptera;COI;;;;;
KU916810;Coleoptera;COI;;;;;
KU916833;Coleoptera;COI;;;;;
KU916860;Coleoptera;COI;;;;;
KU916917;Coleoptera;COI;;;;;
KU916936;Coleoptera;COI;;;;;
KU916982;Coleoptera;COI;;;;;
KU917004;Coleoptera;COI;;;;;
KU917021;Coleoptera;COI;;;;;
KU917033;Coleoptera;COI;;;;;
KU917111;Coleoptera;COI;;;;;
KU917121;Coleoptera;COI;;;;;
KU917150;Coleoptera;COI;;;;;
KU917158;Coleoptera;COI;;;;;
KU917184;Coleoptera;COI;;;;;
KU917356;Coleoptera;COI;;;;;
KU917376;Coleoptera;COI;;;;;
KU917546;Coleoptera;COI;;;;;
KU917632;Coleoptera;COI;;;;;
KU917636;Coleoptera;COI;;;;;
KU917643;Coleoptera;COI;;;;;
KU917736;Coleoptera;COI;;;;;
KU917746;Coleoptera;COI;;;;;
KU917807;Coleoptera;COI;;;;;
KU917891;Coleoptera;COI;;;;;
KU917925;Coleoptera;COI;;;;;
KU917956;Coleoptera;COI;;;;;
KU917971;Coleoptera;COI;;;;;
KU917980;Coleoptera;COI;;;;;
KU918111;Coleoptera;COI;;;;;
KU918206;Coleoptera;COI;;;;;
KU918531;Coleoptera;COI;;;;;
KU918535;Coleoptera;COI;;;;;
KU918552;Coleoptera;COI;;;;;
KU918560;Coleoptera;COI;;;;;
KU918663;Coleoptera;COI;;;;;
KU918763;Coleoptera;COI;;;;;
KU918780;Coleoptera;COI;;;;;
KU918914;Coleoptera;COI;;;;;
KU918938;Coleoptera;COI;;;;;
KU918968;Coleoptera;COI;;;;;
KU918973;Coleoptera;COI;;;;;
KU919015;Coleoptera;COI;;;;;
KU919090;Coleoptera;COI;;;;;
KU919092;Coleoptera;COI;;;;;
KU919168;Coleoptera;COI;;;;;
KU919188;Coleoptera;COI;;;;;
KU919212;Coleoptera;COI;;;;;
KU919268;Coleoptera;COI;;;;;
KU919352;Coleoptera;COI;;;;;
KU919515;Coleoptera;COI;;;;;
KU919565;Coleoptera;COI;;;;;
KU919632;Coleoptera;COI;;;;;

KY683608;Coleoptera;COI;;;;;
KY683651;Coleoptera;COI;;;;;
KY683661;Coleoptera;COI;;;;;
KY683702;Coleoptera;COI;;;;;
MF286233;Coleoptera;COI;;;;;
MF286234;Coleoptera;COI;;;;;
MF286235;Coleoptera;COI;;;;;
MF286236;Coleoptera;COI;;;;;
MF286237;Coleoptera;COI;;;;;
MF286238;Coleoptera;COI;;;;;
MF286242;Coleoptera;COI;;;;;
MF286243;Coleoptera;COI;;;;;
MF286244;Coleoptera;COI;;;;;
MF286256;Coleoptera;COI;;;;;
MF286257;Coleoptera;COI;;;;;
MF286258;Coleoptera;COI;;;;;
MF286259;Coleoptera;COI;;;;;
MF286260;Coleoptera;COI;;;;;
MF286261;Coleoptera;COI;;;;;
MF286262;Coleoptera;COI;;;;;
MF286267;Coleoptera;COI;;;;;
MF286268;Coleoptera;COI;;;;;
MF286273;Coleoptera;COI;;;;;
MF286274;Coleoptera;COI;;;;;
MF286275;Coleoptera;COI;;;;;
MF286276;Coleoptera;COI;;;;;
MF286279;Coleoptera;COI;;;;;
MF286280;Coleoptera;COI;;;;;
MF286281;Coleoptera;COI;;;;;
MF286282;Coleoptera;COI;;;;;
MF286288;Coleoptera;COI;;;;;
MF286294;Coleoptera;COI;;;;;
MF286295;Coleoptera;COI;;;;;
MF286310;Coleoptera;COI;;;;;
MF286316;Coleoptera;COI;;;;;
MF286320;Coleoptera;COI;;;;;
MF286321;Coleoptera;COI;;;;;
MF286324;Coleoptera;COI;;;;;
MF286325;Coleoptera;COI;;;;;
MF286326;Coleoptera;COI;;;;;
MF286330;Coleoptera;COI;;;;;
MF286331;Coleoptera;COI;;;;;
MF543037;Coleoptera;COI;;;;;
MF611995;Coleoptera;COI;;;;;
MF706408;Coleoptera;COI;;;;;
MF706447;Coleoptera;COI;;;;;
MF805176;Coleoptera;COI;;;;;
MF805235;Coleoptera;COI;;;;;
MH020281;Coleoptera;COI;;;;;
MH020282;Coleoptera;COI;;;;;
MH020283;Coleoptera;COI;;;;;
MH020290;Coleoptera;COI;;;;;
MH020301;Coleoptera;COI;;;;;
MH020342;Coleoptera;COI;;;;;
MH020343;Coleoptera;COI;;;;;
MH020450;Coleoptera;COI;;;;;
MH020451;Coleoptera;COI;;;;;
MH020457;Coleoptera;COI;;;;;
MH020458;Coleoptera;COI;;;;;
MH020459;Coleoptera;COI;;;;;
MH020520;Coleoptera;COI;;;;;
MH020521;Coleoptera;COI;;;;;
MH051954;Coleoptera;COI;;;;;
MH115482;Coleoptera;COI;;;;;
MH115573;Coleoptera;COI;;;;;
MH235289;Coleoptera;COI;;;;;
MH235290;Coleoptera;COI;;;;;
MH235291;Coleoptera;COI;;;;;
MH235292;Coleoptera;COI;;;;;
MH235298;Coleoptera;COI;;;;;
MH235302;Coleoptera;COI;;;;;
MH235327;Coleoptera;COI;;;;;
MH618684;Coleoptera;COI;;;;;
MK347673;Coleoptera;COI;;;;;
MK891560;Coleoptera;COI;;;;;
MN182764;Coleoptera;COI;;;;;
MN182776;Coleoptera;COI;;;;;
MN182780;Coleoptera;COI;;;;;

MN182790;Coleoptera;COI;;;;;
MN182803;Coleoptera;COI;;;;;
MN182806;Coleoptera;COI;;;;;
MN182809;Coleoptera;COI;;;;;
MN182811;Coleoptera;COI;;;;;
MN182836;Coleoptera;COI;;;;;
MN182844;Coleoptera;COI;;;;;
MN182849;Coleoptera;COI;;;;;
MN182867;Coleoptera;COI;;;;;
MN182876;Coleoptera;COI;;;;;
MN182882;Coleoptera;COI;;;;;
MN182899;Coleoptera;COI;;;;;
MN182908;Coleoptera;COI;;;;;
MN182911;Coleoptera;COI;;;;;
MN182919;Coleoptera;COI;;;;;
MN182923;Coleoptera;COI;;;;;
MN182969;Coleoptera;COI;;;;;
MN182972;Coleoptera;COI;;;;;
MN182978;Coleoptera;COI;;;;;
MN182990;Coleoptera;COI;;;;;
MN182991;Coleoptera;COI;;;;;
MN182992;Coleoptera;COI;;;;;
MN182993;Coleoptera;COI;;;;;
MN183012;Coleoptera;COI;;;;;
MN183013;Coleoptera;COI;;;;;
MN183018;Coleoptera;COI;;;;;
MN183037;Coleoptera;COI;;;;;
MN183041;Coleoptera;COI;;;;;
MN183049;Coleoptera;COI;;;;;
MN183050;Coleoptera;COI;;;;;
MW259314;Coleoptera;COI;;;;;
MW259342;Coleoptera;COI;;;;;
MW259367;Coleoptera;COI;;;;;
MW259418;Coleoptera;COI;;;;;
MW259458;Coleoptera;COI;;;;;
MW259461;Coleoptera;COI;;;;;
MW259532;Coleoptera;COI;;;;;
MW259546;Coleoptera;COI;;;;;
MW259551;Coleoptera;COI;;;;;
MW259631;Coleoptera;COI;;;;;
MW259684;Coleoptera;COI;;;;;
MW259701;Coleoptera;COI;;;;;
MW259904;Coleoptera;COI;;;;;
MW259908;Coleoptera;COI;;;;;
MW259948;Coleoptera;COI;;;;;
MW520676;Coleoptera;COI;;;;;
MW520677;Coleoptera;COI;;;;;
MZ606899;Coleoptera;COI;;;;;
MZ607295;Coleoptera;COI;;;;;
MZ608576;Coleoptera;COI;;;;;
MZ609847;Coleoptera;COI;;;;;
MZ610834;Coleoptera;COI;;;;;
MZ629289;Coleoptera;COI;;;;;
MZ629296;Coleoptera;COI;;;;;
MZ629629;Coleoptera;COI;;;;;
MZ630288;Coleoptera;COI;;;;;
MZ630316;Coleoptera;COI;;;;;
MZ630374;Coleoptera;COI;;;;;
MZ630724;Coleoptera;COI;;;;;
MZ631986;Coleoptera;COI;;;;;
MZ632001;Coleoptera;COI;;;;;
MZ632551;Coleoptera;COI;;;;;
MZ632603;Coleoptera;COI;;;;;
MZ632747;Coleoptera;COI;;;;;
MZ633042;Coleoptera;COI;;;;;
MZ633589;Coleoptera;COI;;;;;
MZ633609;Coleoptera;COI;;;;;
MZ633936;Coleoptera;COI;;;;;
MZ633989;Coleoptera;COI;;;;;
MZ656180;Coleoptera;COI;;;;;
MZ656272;Coleoptera;COI;;;;;
MZ656302;Coleoptera;COI;;;;;
MZ656356;Coleoptera;COI;;;;;
MZ656659;Coleoptera;COI;;;;;
MZ656721;Coleoptera;COI;;;;;
MZ656926;Coleoptera;COI;;;;;
MZ656962;Coleoptera;COI;;;;;
MZ657235;Coleoptera;COI;;;;;

MZ657508;Coleoptera;COI;;;;;
MZ658038;Coleoptera;COI;;;;;
MZ658860;Coleoptera;COI;;;;;
MZ658978;Coleoptera;COI;;;;;
MZ659029;Coleoptera;COI;;;;;
MZ659069;Coleoptera;COI;;;;;
MZ659211;Coleoptera;COI;;;;;
MZ659585;Coleoptera;COI;;;;;
MZ659864;Coleoptera;COI;;;;;
MZ659942;Coleoptera;COI;;;;;
MZ660210;Coleoptera;COI;;;;;
MZ660564;Coleoptera;COI;;;;;
MZ660637;Coleoptera;COI;;;;;
ON534192;Coleoptera;COI;;;;;
ON534195;Coleoptera;COI;;;;;
;;;;;;;
;;;;;;;

Appendix_6_Compared_assignment_methods;;;;;;
accession_number;Order;GenBank_species_name;SAP_assignment;SAP_Confidence;QIIME_assignment;QIIME_Confidence;
AB481434;Coleoptera;Platycerus caprea;Platycerus caprea;1;Platycerus caprea;1;
AB481435;Coleoptera;Platycerus caraboides;Platycerus caraboides;1;Platycerus caraboides;1;
APPEA017-08;Coleoptera;Pteryx suturalis;Pteryx suturalis;1;Pteryx suturalis;1;
BALA001-22;Coleoptera;Xylotrechus antilope;Xylotrechus antilope;1;Xylotrechus antilope;1;
CERAF087-07;Coleoptera;Prinobius myardi;NA;NA;Prinobius myardi;0.999999694533802;
CERAF088-07;Coleoptera;Prinobius myardi;NA;NA;Prinobius myardi;0.999999694533802;
CERAF089-07;Coleoptera;Prinobius myardi;Prinobius myardi;1;Prinobius myardi;0.99999999883579;
CERAF090-07;Coleoptera;Prinobius myardi;Prinobius myardi;1;Prinobius myardi;0.99999999883579;
CERAF122-08;Coleoptera;Prinobius myardi;Prinobius myardi;NA;Prinobius myardi;0.999999988736079;
CERAF123-08;Coleoptera;Prinobius myardi;Prinobius myardi;NA;Prinobius myardi;0.999999990180811;
CERAF125-08;Coleoptera;Prinobius myardi;Prinobius myardi;1;Prinobius myardi;0.99999986988926;
CERAF127-08;Coleoptera;Prinobius myardi;Prinobius myardi;1;Prinobius myardi;0.999999993592752;
CERAF128-08;Coleoptera;Prinobius myardi;Prinobius myardi;1;Prinobius myardi;0.999999993592752;
CERAF129-08;Coleoptera;Prinobius myardi;Prinobius myardi;1;Prinobius myardi;0.999999974938902;
CERAF130-08;Coleoptera;Prinobius myardi;Prinobius myardi;1;Prinobius myardi;0.999999965199664;
CERAF131-08;Coleoptera;Prinobius myardi;NA;NA;Prinobius myardi;0.999999759602304;
CERAF132-08;Coleoptera;Prinobius myardi;NA;NA;Prinobius myardi;0.999999733549437;
CERAF133-08;Coleoptera;Prinobius myardi;NA;NA;Prinobius myardi;0.999999161821062;
CERAF134-08;Coleoptera;Prinobius myardi;Prinobius myardi;1;Prinobius myardi;0.99999999883579;
CERAF136-08;Coleoptera;Prinobius myardi;Prinobius myardi;1;Prinobius myardi;0.999999999474397;
CERAF147-08;Coleoptera;Prinobius myardi;NA;NA;Prinobius myardi;0.999998804360875;
CERLF460-08;Coleoptera;Herophila tristis;Herophila tristis;1;Herophila tristis;0.999999999999972;
COLFH534-15;Coleoptera;Trixagus carinifrons;Trixagus carinifrons;1;Trixagus carinifrons;1;
COLHH1568-18;Coleoptera;Platycerus caraboides;Platycerus caraboides;1;Platycerus caraboides;1;
COLHH2515-19;Coleoptera;Platystomos albinus;NA;NA;Platystomos albinus;1;
EU752053;Coleoptera;Prinobius myardi;Prinobius myardi;NA;Prinobius myardi;0.999999996562138;
EU752054;Coleoptera;Prinobius myardi;Prinobius myardi;NA;Prinobius myardi;0.999999996562138;
FM877915;Coleoptera;Triplax russica;Triplax russica;0.001;Triplax rufipes;0.99931320990915;
FM877934;Coleoptera;Bitoma crenata;Bitoma crenata;1;Bitoma crenata;1;
FM877936;Coleoptera;Cis boleti;Cis boleti;1;Cis boleti;1;
FM877937;Coleoptera;Cis glabratus;Cis glabratus;1;Cis glabratus;1;
GENHP406-11;Coleoptera;Glischrochilus hortensis;NA;NA;Glischrochilus hortensis;1;
GENHP583-11;Coleoptera;Denticollis linearis;Denticollis linearis;1;Denticollis linearis;1;
GENHP832-11;Coleoptera;Anisandrus dispar;Anisandrus dispar;1;Anisandrus dispar;1;
GMBMJ1541-17;Coleoptera;Aulonothroscus brevicollis;Aulonothroscus brevicollis;1;Aulonothroscus brevicollis;1;
GMBMJ1542-17;Coleoptera;Trixagus leseigneuri;Trixagus leseigneuri;1;Trixagus leseigneuri;1;
GMBMJ1543-17;Coleoptera;Trixagus leseigneuri;Trixagus leseigneuri;1;Trixagus leseigneuri;1;
GMBMJ1545-17;Coleoptera;Aulonothroscus brevicollis;Aulonothroscus brevicollis;1;Aulonothroscus brevicollis;1;
GMBMJ1547-17;Coleoptera;Trixagus carinifrons;Trixagus carinifrons;1;Trixagus carinifrons;1;
GMBMJ1548-17;Coleoptera;Aulonothroscus brevicollis;Aulonothroscus brevicollis;1;Aulonothroscus brevicollis;1;
GMBMJ1769-17;Coleoptera;Anisandrus dispar;Anisandrus dispar;1;Anisandrus dispar;1;
GMBMJ1822-17;Coleoptera;Xyleborinus saxesenii;Xyleborinus saxeseni;1;Xyleborinus saxeseni;1;
GMBMJ1825-17;Coleoptera;Trixagus leseigneuri;Trixagus leseigneuri;1;Trixagus leseigneuri;1;
GMBMJ1826-17;Coleoptera;Trixagus carinifrons;Trixagus carinifrons;1;Trixagus carinifrons;1;
GMBMJ1827-17;Coleoptera;Trixagus carinifrons;Trixagus carinifrons;1;Trixagus carinifrons;1;
GMBMJ1829-17;Coleoptera;Trixagus leseigneuri;Trixagus leseigneuri;1;Trixagus leseigneuri;1;
GMBMJ1838-17;Coleoptera;Trixagus carinifrons;Trixagus carinifrons;1;Trixagus carinifrons;1;
GMBMJ1839-17;Coleoptera;Trixagus leseigneuri;Trixagus leseigneuri;1;Trixagus leseigneuri;1;
GMBMJ1842-17;Coleoptera;Trixagus leseigneuri;Trixagus leseigneuri;1;Trixagus leseigneuri;1;
GMBMJ1843-17;Coleoptera;Trixagus carinifrons;Trixagus carinifrons;1;Trixagus carinifrons;1;
GMBMJ1844-17;Coleoptera;Trixagus leseigneuri;Trixagus leseigneuri;1;Trixagus leseigneuri;1;
GMBMJ1845-17;Coleoptera;Trixagus leseigneuri;Trixagus leseigneuri;1;Trixagus leseigneuri;1;
GMBMJ1846-17;Coleoptera;Trixagus leseigneuri;Trixagus leseigneuri;1;Trixagus leseigneuri;1;
GMBMJ1849-17;Coleoptera;Trixagus carinifrons;Trixagus carinifrons;1;Trixagus carinifrons;1;
GMBMJ1850-17;Coleoptera;Trixagus carinifrons;Trixagus carinifrons;1;Trixagus carinifrons;1;
GMBMJ1851-17;Coleoptera;Trixagus carinifrons;Trixagus carinifrons;1;Trixagus carinifrons;1;
GMBMJ1852-17;Coleoptera;Trixagus carinifrons;Trixagus carinifrons;1;Trixagus carinifrons;1;
GMBMJ1853-17;Coleoptera;Trixagus carinifrons;Trixagus carinifrons;1;Trixagus carinifrons;1;
GMBMJ1854-17;Coleoptera;Trixagus carinifrons;Trixagus carinifrons;1;Trixagus carinifrons;1;
GMBMJ1855-17;Coleoptera;Aulonothroscus brevicollis;Aulonothroscus brevicollis;1;Aulonothroscus brevicollis;1;
GMBMJ1856-17;Coleoptera;Trixagus carinifrons;Trixagus carinifrons;1;Trixagus carinifrons;1;
GMBMJ1857-17;Coleoptera;Trixagus carinifrons;Trixagus carinifrons;1;Trixagus carinifrons;1;
GMBMJ1859-17;Coleoptera;Aulonothroscus brevicollis;Aulonothroscus brevicollis;1;Aulonothroscus brevicollis;1;
GMBMJ1860-17;Coleoptera;Trixagus carinifrons;Trixagus carinifrons;1;Trixagus carinifrons;1;
GMBMJ1861-17;Coleoptera;Trixagus carinifrons;Trixagus carinifrons;1;Trixagus carinifrons;1;
GMBMJ1862-17;Coleoptera;Trixagus carinifrons;Trixagus carinifrons;1;Trixagus carinifrons;1;
GMBMJ1863-17;Coleoptera;Trixagus carinifrons;Trixagus carinifrons;1;Trixagus carinifrons;1;
GMBMJ1864-17;Coleoptera;Trixagus carinifrons;Trixagus carinifrons;1;Trixagus carinifrons;1;

GMBMJ1866-17;Coleoptera;Aulonothroscus brevicollis;Aulonothroscus brevicollis;1;Aulonothroscus brevicollis;1;
GMBMJ1867-17;Coleoptera;Trixagus leseigneuri;Trixagus leseigneuri;1;Trixagus leseigneuri;1;
GMBMJ1868-17;Coleoptera;Aulonothroscus brevicollis;Aulonothroscus brevicollis;1;Aulonothroscus brevicollis;1;
GMBMJ1869-17;Coleoptera;Aulonothroscus brevicollis;Aulonothroscus brevicollis;1;Aulonothroscus brevicollis;1;
GMBMJ1871-17;Coleoptera;Aulonothroscus brevicollis;Aulonothroscus brevicollis;1;Aulonothroscus brevicollis;1;
GMBMK337-17;Coleoptera;Trixagus carinifrons;Trixagus carinifrons;1;Trixagus carinifrons;1;
GMBMK339-17;Coleoptera;Trixagus carinifrons;Trixagus carinifrons;1;Trixagus carinifrons;1;
GMBMK343-17;Coleoptera;Trixagus carinifrons;Trixagus carinifrons;1;Trixagus carinifrons;1;
GMBMK358-17;Coleoptera;Trixagus carinifrons;Trixagus carinifrons;1;Trixagus carinifrons;1;
GMBMK365-17;Coleoptera;Trixagus carinifrons;Trixagus carinifrons;1;Trixagus carinifrons;1;
GMBMK369-17;Coleoptera;Trixagus carinifrons;Trixagus carinifrons;1;Trixagus carinifrons;1;
GMBMK373-17;Coleoptera;Trixagus carinifrons;Trixagus carinifrons;1;Trixagus carinifrons;1;
GMBMK376-17;Coleoptera;Trixagus carinifrons;Trixagus carinifrons;1;Trixagus carinifrons;1;
GMBMK378-17;Coleoptera;Trixagus carinifrons;Trixagus carinifrons;1;Trixagus carinifrons;1;
GMBMK379-17;Coleoptera;Trixagus carinifrons;Trixagus carinifrons;1;Trixagus carinifrons;1;
GMBMK382-17;Coleoptera;Trixagus carinifrons;Trixagus carinifrons;1;Trixagus carinifrons;1;
GMBMK388-17;Coleoptera;Trixagus carinifrons;Trixagus carinifrons;1;Trixagus carinifrons;1;
GMBMK390-17;Coleoptera;Trixagus carinifrons;Trixagus carinifrons;1;Trixagus carinifrons;1;
GMBMK391-17;Coleoptera;Trixagus carinifrons;Trixagus carinifrons;1;Trixagus carinifrons;1;
GMBMK392-17;Coleoptera;Trixagus carinifrons;Trixagus carinifrons;1;Trixagus carinifrons;1;
GMBMK402-17;Coleoptera;Trixagus carinifrons;Trixagus carinifrons;1;Trixagus carinifrons;1;
GMBMK405-17;Coleoptera;Trixagus carinifrons;Trixagus carinifrons;1;Trixagus carinifrons;1;
GMBMK406-17;Coleoptera;Trixagus carinifrons;Trixagus carinifrons;1;Trixagus carinifrons;1;
GMBMK407-17;Coleoptera;Aulonothroscus brevicollis;Aulonothroscus brevicollis;1;Aulonothroscus brevicollis;1;
GMBML413-17;Coleoptera;Trixagus carinifrons;Trixagus carinifrons;1;Trixagus carinifrons;1;
GMBMM1061-17;Coleoptera;Trixagus carinifrons;Trixagus carinifrons;1;Trixagus carinifrons;1;
GMBMM1068-17;Coleoptera;Trixagus carinifrons;Trixagus carinifrons;1;Trixagus carinifrons;1;
GMBMM1075-17;Coleoptera;Aulonothroscus brevicollis;Aulonothroscus brevicollis;1;Aulonothroscus brevicollis;1;
GMBMO773-17;Coleoptera;Trixagus carinifrons;Trixagus carinifrons;1;Trixagus carinifrons;1;
GMBMO782-17;Coleoptera;Trixagus carinifrons;Trixagus carinifrons;1;Trixagus carinifrons;1;
GMBMO787-17;Coleoptera;Trixagus carinifrons;Trixagus carinifrons;1;Trixagus carinifrons;1;
GMBMO788-17;Coleoptera;Trixagus carinifrons;Trixagus carinifrons;1;Trixagus carinifrons;1;
GMBMO793-17;Coleoptera;Trixagus carinifrons;Trixagus carinifrons;1;Trixagus carinifrons;1;
GMBMO814-17;Coleoptera;Trixagus carinifrons;NA;NA;Trixagus carinifrons;1;
GMBMO816-17;Coleoptera;Trixagus carinifrons;Trixagus carinifrons;1;Trixagus carinifrons;1;
GMBMO844-17;Coleoptera;Trixagus carinifrons;Trixagus carinifrons;1;Trixagus carinifrons;1;
GMBMP1888-18;Coleoptera;Trixagus carinifrons;Trixagus carinifrons;1;Trixagus carinifrons;1;
GMBMP1889-18;Coleoptera;Trixagus carinifrons;Trixagus carinifrons;1;Trixagus carinifrons;1;
GMBMP1899-18;Coleoptera;Trixagus carinifrons;Trixagus carinifrons;1;Trixagus carinifrons;1;
GMBMP1900-18;Coleoptera;Trixagus carinifrons;Trixagus carinifrons;1;Trixagus carinifrons;1;
GMBMP1902-18;Coleoptera;Trixagus carinifrons;Trixagus carinifrons;1;Trixagus carinifrons;1;
GMBMP1907-18;Coleoptera;Trixagus carinifrons;Trixagus carinifrons;1;Trixagus carinifrons;1;
GMBMP1912-18;Coleoptera;Trixagus carinifrons;Trixagus carinifrons;1;Trixagus carinifrons;1;
GMBMP1915-18;Coleoptera;Trixagus carinifrons;Trixagus carinifrons;1;Trixagus carinifrons;1;
GMBMP1919-18;Coleoptera;Trixagus carinifrons;Trixagus carinifrons;1;Trixagus carinifrons;1;
GMBMP1920-18;Coleoptera;Trixagus carinifrons;Trixagus carinifrons;1;Trixagus carinifrons;1;
GMBMP1929-18;Coleoptera;Trixagus carinifrons;Trixagus carinifrons;1;Trixagus carinifrons;1;
GMBMP1930-18;Coleoptera;Trixagus carinifrons;Trixagus carinifrons;1;Trixagus carinifrons;1;
GMBMP1933-18;Coleoptera;Trixagus carinifrons;Trixagus carinifrons;1;Trixagus carinifrons;1;
GMBMP1936-18;Coleoptera;Trixagus carinifrons;Trixagus carinifrons;1;Trixagus carinifrons;1;
GMBMP1939-18;Coleoptera;Trixagus carinifrons;Trixagus carinifrons;1;Trixagus carinifrons;1;
GMBMP1946-18;Coleoptera;Trixagus carinifrons;Trixagus carinifrons;1;Trixagus carinifrons;1;
GMBMP1947-18;Coleoptera;Trixagus carinifrons;Trixagus carinifrons;1;Trixagus carinifrons;1;
GMBMP1956-18;Coleoptera;Trixagus carinifrons;Trixagus carinifrons;1;Trixagus carinifrons;1;
GMBMP1958-18;Coleoptera;Trixagus carinifrons;Trixagus carinifrons;1;Trixagus carinifrons;1;
GMBMP1961-18;Coleoptera;Trixagus carinifrons;Trixagus carinifrons;1;Trixagus carinifrons;1;
GMBMP1971-18;Coleoptera;Trixagus carinifrons;Trixagus carinifrons;1;Trixagus carinifrons;1;
GMBMP1976-18;Coleoptera;Trixagus carinifrons;Trixagus carinifrons;1;Trixagus carinifrons;1;
GMBMP1985-18;Coleoptera;Trixagus carinifrons;Trixagus carinifrons;1;Trixagus carinifrons;1;
GMBMP1986-18;Coleoptera;Trixagus carinifrons;Trixagus carinifrons;1;Trixagus carinifrons;1;
GMBMP1991-18;Coleoptera;Trixagus carinifrons;Trixagus carinifrons;1;Trixagus carinifrons;1;
GMBMP1994-18;Coleoptera;Trixagus carinifrons;NA;NA;Trixagus carinifrons;1;
GMBMP1999-18;Coleoptera;Trixagus carinifrons;Trixagus carinifrons;1;Trixagus carinifrons;1;
GMBMP2002-18;Coleoptera;Trixagus carinifrons;Trixagus carinifrons;1;Trixagus carinifrons;1;
GMBMP2003-18;Coleoptera;Trixagus carinifrons;Trixagus carinifrons;1;Trixagus carinifrons;1;
GMBMP2006-18;Coleoptera;Trixagus carinifrons;Trixagus carinifrons;1;Trixagus carinifrons;1;
GMBMP2015-18;Coleoptera;Trixagus carinifrons;NA;NA;Trixagus carinifrons;1;
GMBMP2020-18;Coleoptera;Trixagus carinifrons;Trixagus carinifrons;1;Trixagus carinifrons;1;
GMBMP2023-18;Coleoptera;Trixagus carinifrons;NA;NA;Trixagus carinifrons;1;
GMBMP2027-18;Coleoptera;Trixagus carinifrons;Trixagus carinifrons;1;Trixagus carinifrons;1;
GMBMQ1006-17;Coleoptera;Trixagus carinifrons;Trixagus carinifrons;1;Trixagus carinifrons;1;
GMBMR1357-17;Coleoptera;Trixagus carinifrons;Trixagus carinifrons;1;Trixagus carinifrons;1;

GMBMR1369-17;Coleoptera;Trixagus carinifrons;Trixagus carinifrons;1;Trixagus carinifrons;1;
GMBUA1440-14;Coleoptera;Aulonothroscus brevicollis;Aulonothroscus brevicollis;1;Aulonothroscus brevicollis;1;
GMBUA1456-14;Coleoptera;Aulonothroscus brevicollis;Aulonothroscus brevicollis;1;Aulonothroscus brevicollis;1;
GMBUA1457-14;Coleoptera;Trixagus carinifrons;Trixagus carinifrons;1;Trixagus carinifrons;1;
GMBUA1466-14;Coleoptera;Aulonothroscus brevicollis;Aulonothroscus brevicollis;1;Aulonothroscus brevicollis;1;
GMBUA1475-14;Coleoptera;Aulonothroscus brevicollis;Aulonothroscus brevicollis;1;Aulonothroscus brevicollis;1;
GMBUA1476-14;Coleoptera;Aulonothroscus brevicollis;Aulonothroscus brevicollis;1;Aulonothroscus brevicollis;1;
GMBUA1478-14;Coleoptera;Aulonothroscus brevicollis;Aulonothroscus brevicollis;1;Aulonothroscus brevicollis;1;
GMBUA343-14;Coleoptera;Grammoptera ruficornis;Grammoptera ruficornis;1;Grammoptera ruficornis;0.999999999999261;
GMBUB1566-14;Coleoptera;Trixagus carinifrons;Trixagus carinifrons;1;Trixagus carinifrons;1;
GMBUB1586-14;Coleoptera;Trixagus carinifrons;Trixagus carinifrons;1;Trixagus carinifrons;1;
GMBUB1629-14;Coleoptera;Tritoma bipustulata;NA;1;Tritoma bipustulata;1;
GMBUC1588-14;Coleoptera;Trixagus leseigneuri;Trixagus leseigneuri;1;Trixagus leseigneuri;1;
GMBUC1780-14;Coleoptera;Trixagus leseigneuri;Trixagus leseigneuri;1;Trixagus leseigneuri;1;
GMBUG2178-14;Coleoptera;Aulonothroscus brevicollis;Aulonothroscus brevicollis;1;Aulonothroscus brevicollis;1;
GMFIC247-12;Coleoptera;Placusa tachyporoides;Placusa tachyporoides;1;Placusa tachyporoides;0.999999993023636;
GMFIC382-12;Coleoptera;Atrecus pilicornis;Atrecus pilicornis;1;Atrecus pilicornis;1;
GMFID006-12;Coleoptera;Elateroides dermestoides;Elateroides dermestoides;1;Elateroides dermestoides;1;
GMFID007-12;Coleoptera;Elateroides dermestoides;NA;1;Elateroides dermestoides;1;
GMFID008-12;Coleoptera;Elateroides dermestoides;NA;1;Elateroides dermestoides;1;
GMFID013-12;Coleoptera;Elateroides dermestoides;NA;1;Elateroides dermestoides;1;
GMFID014-12;Coleoptera;Elateroides dermestoides;NA;1;Elateroides dermestoides;1;
GMFID015-12;Coleoptera;Elateroides dermestoides;Elateroides dermestoides;1;Elateroides dermestoides;1;
GMFID017-12;Coleoptera;Elateroides dermestoides;Elateroides dermestoides;1;Elateroides dermestoides;1;
GMFID019-12;Coleoptera;Elateroides dermestoides;Elateroides dermestoides;1;Elateroides dermestoides;1;
GMFID020-12;Coleoptera;Elateroides dermestoides;Elateroides dermestoides;1;Elateroides dermestoides;1;
GMFID021-12;Coleoptera;Elateroides dermestoides;NA;1;Elateroides dermestoides;1;
GMFID022-12;Coleoptera;Elateroides dermestoides;Elateroides dermestoides;1;Elateroides dermestoides;1;
GMFID023-12;Coleoptera;Elateroides dermestoides;NA;1;Elateroides dermestoides;1;
GMFID024-12;Coleoptera;Elateroides dermestoides;NA;1;Elateroides dermestoides;1;
GMFID025-12;Coleoptera;Elateroides dermestoides;NA;1;Elateroides dermestoides;1;
GMFID027-12;Coleoptera;Elateroides dermestoides;Elateroides dermestoides;1;Elateroides dermestoides;1;
GMFID029-12;Coleoptera;Elateroides dermestoides;NA;1;Elateroides dermestoides;1;
GMFID031-12;Coleoptera;Elateroides dermestoides;Elateroides dermestoides;1;Elateroides dermestoides;1;
GMFID032-12;Coleoptera;Elateroides dermestoides;NA;1;Elateroides dermestoides;1;
GMFID033-12;Coleoptera;Elateroides dermestoides;Elateroides dermestoides;1;Elateroides dermestoides;1;
GMFID035-12;Coleoptera;Elateroides dermestoides;NA;1;Elateroides dermestoides;1;
GMFID036-12;Coleoptera;Elateroides dermestoides;Elateroides dermestoides;1;Elateroides dermestoides;1;
GMFID037-12;Coleoptera;Elateroides dermestoides;Elateroides dermestoides;1;Elateroides dermestoides;1;
GMFID038-12;Coleoptera;Elateroides dermestoides;NA;1;Elateroides dermestoides;1;
GMFID039-12;Coleoptera;Elateroides dermestoides;Elateroides dermestoides;1;Elateroides dermestoides;1;
GMFID040-12;Coleoptera;Elateroides dermestoides;NA;1;Elateroides dermestoides;1;
GMFID041-12;Coleoptera;Elateroides dermestoides;Elateroides dermestoides;1;Elateroides dermestoides;1;
GMFID042-12;Coleoptera;Elateroides dermestoides;Elateroides dermestoides;1;Elateroides dermestoides;1;
GMFID043-12;Coleoptera;Elateroides dermestoides;NA;1;Elateroides dermestoides;1;
GMFID044-12;Coleoptera;Elateroides dermestoides;NA;1;Elateroides dermestoides;1;
GMFID046-12;Coleoptera;Elateroides dermestoides;NA;1;Elateroides dermestoides;1;
GMFID047-12;Coleoptera;Elateroides dermestoides;Elateroides dermestoides;1;Elateroides dermestoides;1;
GMFID048-12;Coleoptera;Elateroides dermestoides;NA;1;Elateroides dermestoides;1;
GMFID049-12;Coleoptera;Elateroides dermestoides;Elateroides dermestoides;1;Elateroides dermestoides;1;
GMFID050-12;Coleoptera;Elateroides dermestoides;NA;1;Elateroides dermestoides;1;
GMFID051-12;Coleoptera;Elateroides dermestoides;NA;1;Elateroides dermestoides;1;
GMFID052-12;Coleoptera;Elateroides dermestoides;NA;1;Elateroides dermestoides;1;

GMFID053-12;Coleoptera;Elateroides dermestoides;NA;1;Elateroides dermestoides;1;
GMFID054-12;Coleoptera;Elateroides dermestoides;Elateroides dermestoides;1;Elateroides dermestoides;1;
GMFID057-12;Coleoptera;Elateroides dermestoides;NA;1;Elateroides dermestoides;1;
GMFID058-12;Coleoptera;Elateroides dermestoides;NA;1;Elateroides dermestoides;1;
GMFID061-12;Coleoptera;Elateroides dermestoides;NA;1;Elateroides dermestoides;1;
GMFID063-12;Coleoptera;Elateroides dermestoides;Elateroides dermestoides;1;Elateroides dermestoides;1;
GMFID064-12;Coleoptera;Elateroides dermestoides;Elateroides dermestoides;1;Elateroides dermestoides;1;
GMFID066-12;Coleoptera;Leptusa pulchella;Leptusa pulchella;1;Leptusa pulchella;1;
GMFID080-12;Coleoptera;Elateroides dermestoides;NA;1;Elateroides dermestoides;1;
GMFID082-12;Coleoptera;Elateroides dermestoides;Elateroides dermestoides;1;Elateroides dermestoides;1;
GMFID083-12;Coleoptera;Elateroides dermestoides;Elateroides dermestoides;1;Elateroides dermestoides;1;
GMFID084-12;Coleoptera;Trixagus carinifrons;Trixagus carinifrons;1;Trixagus carinifrons;1;
GMFIE006-12;Coleoptera;Placusa tachyporoides;Placusa tachyporoides;1;Placusa tachyporoides;0.999999996027242;
GMFIE821-12;Coleoptera;Latridius consimilis;Latridius consimilis;1;Latridius consimilis;1;
GMFIE823-12;Coleoptera;Elateroides dermestoides;NA;1;Elateroides dermestoides;1;
GMFIF749-12;Coleoptera;Pteryx suturalis;Pteryx suturalis;1;Pteryx suturalis;1;
GMFIH280-12;Coleoptera;Euplectus punctatus;Euplectus punctatus;1;Euplectus punctatus;1;
GMFIK030-12;Coleoptera;Platycerus caprea;Platycerus caprea;1;Platycerus caprea;1;
GMFIK031-12;Coleoptera;Platycerus caprea;Platycerus caprea;1;Platycerus caprea;1;
GMFIK032-12;Coleoptera;Platycerus caprea;Platycerus caprea;1;Platycerus caprea;1;
GMFIK033-12;Coleoptera;Platycerus caprea;Platycerus caprea;1;Platycerus caprea;1;
GMFIK034-12;Coleoptera;Platycerus caprea;Platycerus caprea;1;Platycerus caprea;1;
GMFIL010-12;Coleoptera;Platycerus caprea;Platycerus caprea;1;Platycerus caprea;1;
GMFIL016-12;Coleoptera;Platycerus caprea;Platycerus caprea;1;Platycerus caprea;1;
GMFIM001-12;Coleoptera;Platycerus caprea;Platycerus caprea;1;Platycerus caprea;1;
GMFIM210-12;Coleoptera;Epuraea pygmaea;Epuraea pygmaea;1;Epuraea pygmaea;0.999999999999574;
GMFIM285-13;Coleoptera;Elateroides dermestoides;Elateroides dermestoides;1;Elateroides dermestoides;1;
GMFIN169-12;Coleoptera;Elateroides dermestoides;NA;1;Elateroides dermestoides;1;
GMFIN170-12;Coleoptera;Elateroides dermestoides;NA;1;Elateroides dermestoides;1;
GMGMA1532-14;Coleoptera;Xylotrechus arvicola;Xylotrechus arvicola;1;Xylotrechus arvicola;0.999999992280607;
GMGMA1550-14;Coleoptera;Anthaxia nitidula;Anthaxia nitidula;1;Anthaxia nitidula;0.999999999999943;
GMGMC759-14;Coleoptera;Tritoma bipustulata;Tritoma bipustulata;1;Tritoma bipustulata;1;
GMGMG1182-14;Coleoptera;Trypodendron domesticum;Trypodendron domesticum;1;Trypodendron domesticum;1;
GMGMG1184-14;Coleoptera;Xyleborinus saxesenii;Xyleborinus saxeseni;1;Xyleborinus saxeseni;1;
GMGMG1187-14;Coleoptera;Ernobius abietis;Ernobius abietis;1;Ernobius abietis;1;
GMGMG1189-14;Coleoptera;Trypodendron domesticum;Trypodendron domesticum;1;Trypodendron domesticum;1;
GMGMG1192-14;Coleoptera;Xyleborinus saxesenii;Xyleborinus saxeseni;1;Xyleborinus saxeseni;1;
GMGMG1193-14;Coleoptera;Xyleborinus saxesenii;Xyleborinus saxeseni;1;Xyleborinus saxeseni;1;
GMGMG1195-14;Coleoptera;Xyleborinus saxesenii;Xyleborinus saxeseni;1;Xyleborinus saxeseni;1;
GMGMG1201-14;Coleoptera;Xyleborinus saxesenii;Xyleborinus saxeseni;1;Xyleborinus saxeseni;1;
GMGMG1202-14;Coleoptera;Xyleborinus saxesenii;Xyleborinus saxeseni;1;Xyleborinus saxeseni;1;
GMGMH1163-14;Coleoptera;Aulonothroscus brevicollis;Aulonothroscus brevicollis;1;Aulonothroscus brevicollis;1;
GMGMH1167-14;Coleoptera;Tritoma bipustulata;Tritoma bipustulata;1;Tritoma bipustulata;1;
GMGMH1559-14;Coleoptera;Platystomos albinus;Platystomos albinus;1;Platystomos albinus;1;
GMGMH1563-14;Coleoptera;Platycerus caraboides;Platycerus caraboides;1;Platycerus caraboides;1;
GMGMH1571-14;Coleoptera;Platystomos albinus;Platystomos albinus;1;Platystomos albinus;1;
GMGMI262-14;Coleoptera;Platystomos albinus;Platystomos albinus;1;Platystomos albinus;1;
GMGMJ1375-14;Coleoptera;Trixagus carinifrons;Trixagus carinifrons;1;Trixagus carinifrons;1;
GMGMJ1500-14;Coleoptera;Xyleborinus saxesenii;Xyleborinus saxeseni;1;Xyleborinus saxeseni;1;
GMGMJ376-14;Coleoptera;Grammoptera ruficornis;Grammoptera ruficornis;1;Grammoptera ruficornis;1;
GMGMJ377-14;Coleoptera;Grammoptera ruficornis;Grammoptera ruficornis;1;Grammoptera ruficornis;1;
GMGMJ869-14;Coleoptera;Denticollis linearis;Denticollis linearis;1;Denticollis linearis;1;
GMGMJ874-14;Coleoptera;Platystomos albinus;Platystomos albinus;1;Platystomos albinus;1;
GMGMK1004-14;Coleoptera;Grammoptera ruficornis;Grammoptera ruficornis;1;Grammoptera ruficornis;1;
GMGMK1005-14;Coleoptera;Anthaxia nitidula;Anthaxia nitidula;1;Anthaxia nitidula;1;
GMGMK1006-14;Coleoptera;Anthaxia nitidula;NA;NA;Anthaxia nitidula;0.999999999997812;
GMGMK1009-14;Coleoptera;Trixagus carinifrons;Trixagus carinifrons;1;Trixagus carinifrons;1;
GMGMK1010-14;Coleoptera;Grammoptera ruficornis;NA;NA;Grammoptera ruficornis;1;
GMGMK1013-14;Coleoptera;Anthaxia nitidula;Anthaxia nitidula;1;Anthaxia nitidula;0.999999999997584;
GMGMK1506-14;Coleoptera;Anthaxia nitidula;Anthaxia nitidula;1;Anthaxia nitidula;1;
GMGMK1507-14;Coleoptera;Trixagus carinifrons;Trixagus carinifrons;1;Trixagus carinifrons;1;
GMGMK1508-14;Coleoptera;Anthaxia nitidula;Anthaxia nitidula;1;Anthaxia nitidula;1;
GMGMK1513-14;Coleoptera;Anthaxia nitidula;Anthaxia nitidula;1;Anthaxia nitidula;1;

GMGMK946-14;Coleoptera;Grammoptera ruficornis;Grammoptera ruficornis;NA;Grammoptera ruficornis;1;
GMGMK954-14;Coleoptera;Xyleborus dryographus;Xyleborus dryographus;1;Xyleborus dryographus;0.999999999999147;
GMGMK973-14;Coleoptera;Grammoptera ruficornis;Grammoptera ruficornis;1;Grammoptera ruficornis;1;
GMGMK990-14;Coleoptera;Anthaxia nitidula;Anthaxia nitidula;1;Anthaxia nitidula;0.999999999999972;
GMGML1294-14;Coleoptera;Anthaxia nitidula;Anthaxia nitidula;1;Anthaxia nitidula;0.999999999999972;
GMGML1295-14;Coleoptera;Anthaxia nitidula;Anthaxia nitidula;1;Anthaxia nitidula;0.999999999999972;
GMGML1296-14;Coleoptera;Anthaxia nitidula;Anthaxia nitidula;1;Anthaxia nitidula;1;
GMGMM1445-14;Coleoptera;Trixagus carinifrons;Trixagus carinifrons;1;Trixagus carinifrons;1;
GMGMM1447-14;Coleoptera;Anthaxia nitidula;Anthaxia nitidula;1;Anthaxia nitidula;0.999999999999972;
GMGMM1458-14;Coleoptera;Anthaxia nitidula;Anthaxia nitidula;1;Anthaxia nitidula;1;
GMGMM1466-14;Coleoptera;Anthaxia nitidula;Anthaxia nitidula;1;Anthaxia nitidula;0.999999999999972;
GMGMM1484-14;Coleoptera;Anthaxia nitidula;Anthaxia nitidula;1;Anthaxia nitidula;1;
GMGMN1150-14;Coleoptera;Trixagus carinifrons;Trixagus carinifrons;1;Trixagus carinifrons;1;
GMGMN772-14;Coleoptera;Anthaxia quadripunctata;Anthaxia quadripunctata;1;Anthaxia quadripunctata;1;
GMGMN803-14;Coleoptera;Trixagus carinifrons;Trixagus carinifrons;1;Trixagus carinifrons;1;
GMGMP1922-18;Coleoptera;Placusa tachyporoides;Placusa tachyporoides;1;Placusa tachyporoides;0.999999999829726;
GMGMP6786-18;Coleoptera;Grammoptera ruficornis;Grammoptera ruficornis;1;Grammoptera ruficornis;1;
GMGMP7064-18;Coleoptera;Grammoptera ruficornis;NA;NA;Grammoptera ruficornis;1;
GMGMP7335-18;Coleoptera;Aulonothroscus brevicollis;Aulonothroscus brevicollis;1;Aulonothroscus brevicollis;1;
GMGMU4995-20;Coleoptera;Hypebaeus flavipes;Hypebaeus flavipes;1;Hypebaeus flavipes;1;
GMGMU5060-20;Coleoptera;Trixagus carinifrons;Trixagus carinifrons;1;Trixagus carinifrons;1;
GMGRA1613-13;Coleoptera;Elateroides dermestoides;NA;1;Elateroides dermestoides;1;
GMGRC2944-13;Coleoptera;Bibloporus bicolor;Bibloporus bicolor;1;Bibloporus bicolor;0.999999999999602;
GMGRD1546-13;Coleoptera;Bibloporus bicolor;Bibloporus bicolor;1;Bibloporus bicolor;0.999999999998522;
GMGRD3652-13;Coleoptera;Atrecus affinis;Atrecus affinis;1;Atrecus affinis;1;
GMGRE3158-13;Coleoptera;Epuraea terminalis;Epuraea terminalis;1;Epuraea terminalis;0.999839541300684;
GMGRE3167-13;Coleoptera;Bibloporus bicolor;Bibloporus bicolor;1;Bibloporus bicolor;0.999999999999773;
GMGRE3186-13;Coleoptera;Bibloporus bicolor;Bibloporus bicolor;1;Bibloporus bicolor;0.99999999997496;
GMGRG3961-13;Coleoptera;Pteryngium crenatum;Pteryngium crenatum;1;Pteryngium crenatum;1;
GMGRG3970-13;Coleoptera;Pteryngium crenatum;Pteryngium crenatum;1;Pteryngium crenatum;1;
GMGRH1959-13;Coleoptera;Anisotoma castanea;Anisotoma castanea;1;Anisotoma castanea;0.999999999999375;
GMNWD521-14;Coleoptera;Trypodendron domesticum;Trypodendron domesticum;1;Trypodendron domesticum;1;
GMNWF1974-14;Coleoptera;Trixagus carinifrons;Trixagus carinifrons;1;Trixagus carinifrons;1;
GMNWF1980-14;Coleoptera;Trixagus carinifrons;Trixagus carinifrons;1;Trixagus carinifrons;1;
GMNWF2019-14;Coleoptera;Trixagus carinifrons;Trixagus carinifrons;1;Trixagus carinifrons;1;
GMNWF2029-14;Coleoptera;Trixagus carinifrons;Trixagus carinifrons;1;Trixagus carinifrons;1;
GMNWG3123-14;Coleoptera;Trixagus carinifrons;Trixagus carinifrons;1;Trixagus carinifrons;1;
GMNWG3127-14;Coleoptera;Trixagus carinifrons;Trixagus carinifrons;1;Trixagus carinifrons;1;
GMNWG3131-14;Coleoptera;Trixagus carinifrons;Trixagus carinifrons;1;Trixagus carinifrons;1;
GMNWG3139-14;Coleoptera;Trixagus carinifrons;Trixagus carinifrons;1;Trixagus carinifrons;1;
GMNWG3146-14;Coleoptera;Trixagus carinifrons;Trixagus carinifrons;1;Trixagus carinifrons;1;
GMNWG3148-14;Coleoptera;Trixagus carinifrons;Trixagus carinifrons;1;Trixagus carinifrons;1;
GMNWG3165-14;Coleoptera;Trixagus carinifrons;Trixagus carinifrons;1;Trixagus carinifrons;1;
GMNWG3166-14;Coleoptera;Trixagus carinifrons;Trixagus carinifrons;1;Trixagus carinifrons;1;
GMNWI3224-14;Coleoptera;Trixagus carinifrons;Trixagus carinifrons;1;Trixagus carinifrons;1;
GMNWI3253-14;Coleoptera;Trixagus carinifrons;Trixagus carinifrons;1;Trixagus carinifrons;1;
GMNWI3254-14;Coleoptera;Trixagus carinifrons;Trixagus carinifrons;1;Trixagus carinifrons;1;
GMNWI3261-14;Coleoptera;Trixagus carinifrons;Trixagus carinifrons;1;Trixagus carinifrons;1;
GMNWI3263-14;Coleoptera;Trixagus carinifrons;Trixagus carinifrons;1;Trixagus carinifrons;1;
GMNWI3267-14;Coleoptera;Trixagus carinifrons;Trixagus carinifrons;1;Trixagus carinifrons;1;
GMNWI3287-14;Coleoptera;Trixagus carinifrons;Trixagus carinifrons;1;Trixagus carinifrons;1;
GMNWI3298-14;Coleoptera;Trixagus carinifrons;Trixagus carinifrons;1;Trixagus carinifrons;1;
GMNWJ3036-14;Coleoptera;Trixagus carinifrons;Trixagus carinifrons;1;Trixagus carinifrons;1;
GMNWJ3048-14;Coleoptera;Trixagus carinifrons;Trixagus carinifrons;1;Trixagus carinifrons;1;
GMNWJ3049-14;Coleoptera;Trixagus carinifrons;Trixagus carinifrons;1;Trixagus carinifrons;1;
GMNWJ3053-14;Coleoptera;Trixagus carinifrons;Trixagus carinifrons;1;Trixagus carinifrons;1;
GMNWJ3061-14;Coleoptera;Trixagus carinifrons;Trixagus carinifrons;1;Trixagus carinifrons;1;
GMNWK3483-14;Coleoptera;Trixagus carinifrons;Trixagus carinifrons;1;Trixagus carinifrons;1;
GMNWL2542-14;Coleoptera;Trixagus carinifrons;Trixagus carinifrons;1;Trixagus carinifrons;1;
GMNWL2590-14;Coleoptera;Trixagus carinifrons;Trixagus carinifrons;1;Trixagus carinifrons;1;

GMNWM677-14;Coleoptera;Epuraea terminalis;Epuraea terminalis;1;Epuraea terminalis;0.999969265387654;
GMNWM722-14;Coleoptera;Epuraea terminalis;Epuraea terminalis;1;Epuraea terminalis;0.999860710938023;
HM422045;Coleoptera;Potamophilus acuminatus;Potamophilus acuminatus;1;Potamophilus acuminatus;1;
HM909032;Coleoptera;Anthaxia quadripunctata;Anthaxia quadripunctata;1;Anthaxia quadripunctata;0.999999998992223;
HM909034;Coleoptera;Gonotropis dorsalis;Gonotropis dorsalis;1;Gonotropis dorsalis;1;
HM909091;Coleoptera;Bitoma crenata;Bitoma crenata;1;Bitoma crenata;1;
HM909106;Coleoptera;Xyleborus cryptographus;Xyleborus cryptographus;1;Xyleborus cryptographus;1;
HM909112;Coleoptera;Agrilus viridis;Agrilus viridis;1;Agrilus viridis;0.999998600333824;
HM909113;Coleoptera;Agrilus viridis;Agrilus viridis;1;Agrilus viridis;0.999999997497753;
HM909114;Coleoptera;Agrilus viridis;Agrilus viridis;1;Agrilus viridis;0.999999994963986;
HM909115;Coleoptera;Agrilus viridis;Agrilus viridis;1;Agrilus viridis;0.999998600333824;
HM909116;Coleoptera;Agrilus viridis;Agrilus viridis;1;Agrilus viridis;0.999998600333824;
HM909117;Coleoptera;Agrilus viridis;Agrilus viridis;1;Agrilus viridis;0.999999404104194;
HM909118;Coleoptera;Agrilus viridis;Agrilus viridis;1;Agrilus viridis;0.999998212903028;
HM909119;Coleoptera;Agrilus viridis;Agrilus viridis;1;Agrilus viridis;0.999999303891926;
HM909125;Coleoptera;Agrilus viridis;Agrilus viridis;1;Agrilus viridis;0.999999994963986;
HM909126;Coleoptera;Agrilus viridis;Agrilus viridis;NA;NA;0.999999999999994;
HM909127;Coleoptera;Agrilus viridis;Agrilus viridis;1;Agrilus viridis;0.999999994963986;
HM909128;Coleoptera;Agrilus viridis;Agrilus viridis;1;Agrilus viridis;0.999999994963986;
HM909129;Coleoptera;Agrilus viridis;Agrilus viridis;1;Agrilus viridis;0.999999994963986;
HM909130;Coleoptera;Agrilus viridis;Agrilus viridis;1;Agrilus viridis;0.999999746205109;
HM909131;Coleoptera;Agrilus viridis;Agrilus viridis;1;Agrilus viridis;0.999999620142163;
HM909132;Coleoptera;Agrilus viridis;Agrilus viridis;1;Agrilus viridis;0.999999897855337;
HM909133;Coleoptera;Agrilus viridis;Agrilus viridis;1;Agrilus viridis;0.999999746205109;
HM909134;Coleoptera;Agrilus viridis;Agrilus viridis;1;Agrilus viridis;0.999999886805916;
HM909135;Coleoptera;Agrilus viridis;Agrilus viridis;1;Agrilus viridis;0.999999886805916;
HM909136;Coleoptera;Agrilus viridis;Agrilus viridis;1;Agrilus viridis;0.999999886805916;
HM909137;Coleoptera;Agrilus viridis;Agrilus viridis;1;Agrilus viridis;0.999999886805916;
HM909138;Coleoptera;Agrilus viridis;Agrilus viridis;1;Agrilus viridis;0.999999910920625;
HM909139;Coleoptera;Agrilus viridis;Agrilus viridis;1;Agrilus viridis;0.999999897855337;
HM909140;Coleoptera;Agrilus viridis;Agrilus viridis;1;Agrilus viridis;0.999999897855337;
HM909141;Coleoptera;Agrilus viridis;Agrilus viridis;1;Agrilus viridis;0.999999719684649;
HM909142;Coleoptera;Agrilus viridis;Agrilus viridis;1;Agrilus viridis;0.999999964495402;
HM909143;Coleoptera;Agrilus viridis;Agrilus viridis;NA;Agrilus viridis;0.999999270966963;
HM909144;Coleoptera;Agrilus viridis;Agrilus viridis;1;Agrilus viridis;0.999999992684138;
HM909145;Coleoptera;Agrilus viridis;Agrilus viridis;1;Agrilus viridis;0.999999993848206;
HM909147;Coleoptera;Agrilus viridis;Agrilus viridis;1;Agrilus viridis;0.999999886805916;
HM909148;Coleoptera;Agrilus viridis;Agrilus viridis;1;Agrilus viridis;0.999999886805916;
HM909149;Coleoptera;Agrilus viridis;Agrilus viridis;1;Agrilus viridis;0.999999897855337;
HM909150;Coleoptera;Agrilus viridis;Agrilus viridis;1;Agrilus viridis;0.999999897855337;
HM909151;Coleoptera;Agrilus viridis;Agrilus viridis;1;Agrilus viridis;0.999999886805916;
HM909152;Coleoptera;Agrilus viridis;Agrilus viridis;1;Agrilus viridis;0.999999886805916;
HM909153;Coleoptera;Agrilus viridis;Agrilus viridis;1;Agrilus viridis;0.999999886805916;
HM909154;Coleoptera;Agrilus viridis;Agrilus viridis;1;Agrilus viridis;0.999999897855337;
HM909155;Coleoptera;Agrilus viridis;Agrilus viridis;1;Agrilus viridis;0.99999970426977;
HQ559231;Coleoptera;Dendrophilus pygmaeus;NA;1;Dendrophilus pygmaeus;1;
HQ559246;Coleoptera;Platycerus caprea;Meloe violaceus;1;Meloe violaceus;0.999999999999829;
HQ563254;Coleoptera;Hylurgops palliatus;Hylurgops palliatus;1;Hylurgops palliatus;1;
HQ948251;Coleoptera;Anisotoma humeralis;Anisotoma humeralis;1;Anisotoma humeralis;0.99999999958203;
HQ948252;Coleoptera;Cis boleti;Cis boleti;1;Cis boleti;1;
HQ948274;Coleoptera;Denticollis linearis;Denticollis linearis;1;Denticollis linearis;1;
HQ953298;Coleoptera;Anisotoma humeralis;Anisotoma humeralis;1;Anisotoma humeralis;0.999999999979735;
HQ953299;Coleoptera;Epuraea variegata;Epuraea variegata;1;Epuraea variegata;0.999999999840242;
HQ953376;Coleoptera;Trixagus leseigneuri;Trixagus leseigneuri;1;Trixagus leseigneuri;1;
HQ953393;Coleoptera;Hypnogyra angularis;Hypnogyra glabra;1;Hypnogyra glabra;1;
HQ953394;Coleoptera;Atrecus affinis;NA;NA;Atrecus affinis;1;
HQ953399;Coleoptera;Euplectus karstenii;Euplectus karstenii;1;Euplectus karstenii;1;
HQ953402;Coleoptera;Batrisodes oculatus;Batrisodes oculatus;1;Batrisodes oculatus;0.999999999999943;
HQ953403;Coleoptera;Euplectus kirbii;NA;0.323;NA;100.000.000.000.001;
HQ953405;Coleoptera;Cis glabratus;Cis glabratus;1;Cis glabratus;1;
HQ953429;Coleoptera;Elateroides dermestoides;NA;1;Elateroides dermestoides;1;
HQ953479;Coleoptera;Euryusa castanoptera;Euryusa castanoptera;NA;Euryusa castanoptera;1;
HQ953481;Coleoptera;Xylostiba bosnica;Xylostiba bosnica;NA;Xylostiba bosnica;1;
HQ953484;Coleoptera;Vincenzellus ruficollis;NA;NA;Vincenzellus ruficollis;1;
HQ953488;Coleoptera;Phloeonomus punctipennis;Phloeonomus punctipennis;1;Phloeonomus punctipennis;1;
HQ953500;Coleoptera;Cis festivus;Orthocis festivus;1;Orthocis festivus;1;
HQ953505;Coleoptera;Homalota plana;NA;NA;Homalota plana;1;
HQ953511;Coleoptera;Trypodendron domesticum;Trypodendron domesticum;1;Trypodendron domesticum;1;
HQ953519;Coleoptera;Placusa tachyporoides;Placusa tachyporoides;1;Placusa tachyporoides;0.999999998163389;
HQ953528;Coleoptera;Euplectus piceus;Euplectus piceus;1;Euplectus piceus;1;

HQ953537;Coleoptera;Valgus hemipterus;Valgus hemipterus;1;Valgus hemipterus;1;
HQ953597;Coleoptera;Berginus tamarisci;NA;1;Berginus tamarisci;1;
HQ953607;Coleoptera;Grammoptera abdominalis;Grammoptera abdominalis;1;Grammoptera abdominalis;1;
HQ953626;Coleoptera;Euplectus infirmus;Euplectus infirmus;1;Euplectus infirmus;1;
HQ953633;Coleoptera;Bibloporus bicolor;Bibloporus bicolor;1;Bibloporus bicolor;0.999999999999972;
HQ953636;Coleoptera;Leptusa fumida;Leptusa fumida;1;Leptusa fumida;1;
HQ953665;Coleoptera;Hypebaeus flavipes;Hypebaeus flavipes;1;Hypebaeus flavipes;1;
HQ953733;Coleoptera;Tritoma bipustulata;NA;1;Tritoma bipustulata;1;
HQ953758;Coleoptera;Pteryngium crenatum;Pteryngium crenatum;1;Pteryngium crenatum;1;
HQ953759;Coleoptera;Pteryngium crenatum;Pteryngium crenatum;1;Pteryngium crenatum;1;
HQ953783;Coleoptera;Platycerus caraboides;Platycerus caraboides;1;Platycerus caraboides;1;
HQ953820;Coleoptera;Plectophloeus erichsoni;Plectophloeus erichsoni;1;Plectophloeus erichsoni;1;
HQ953931;Coleoptera;Grammoptera ustulata;Grammoptera ustulata;1;Grammoptera ustulata;1;
HQ954002;Coleoptera;Latridius hirtus;Latridius hirtus;NA;Latridius hirtus;1;
HQ954006;Coleoptera;Phloeocharis subtilissima;Phloeocharis subtilissima;NA;Phloeocharis subtilissima;1;
HQ954007;Coleoptera;Phloeocharis subtilissima;Phloeocharis subtilissima;1;Phloeocharis subtilissima;1;
HQ954015;Coleoptera;Tritoma bipustulata;Tritoma bipustulata;1;Tritoma bipustulata;1;
HQ954016;Coleoptera;Triplax rufipes;Triplax rufipes;1;Triplax rufipes;1;
HQ954017;Coleoptera;Triplax rufipes;Triplax rufipes;1;Triplax rufipes;1;
HQ954035;Coleoptera;Euryusa sinuata;Euryusa sinuata;NA;Euryusa sinuata;0.999999999020872;
HQ954042;Coleoptera;Latridius pseudominutus;NA;NA;Latridius pseudominutus;0.999806281432518;
HQ954043;Coleoptera;Latridius minutus;Latridius minutus;1;Latridius minutus;0.999999999993065;
HQ954060;Coleoptera;Anisotoma orbicularis;Anisotoma orbicularis;1;Anisotoma orbicularis;1;
HQ954061;Coleoptera;Anisotoma orbicularis;Anisotoma orbicularis;1;Anisotoma orbicularis;1;
HQ954074;Coleoptera;Ips sexdentatus;Ips sexdentatus;1;Ips sexdentatus;1;
HQ954091;Coleoptera;Globicornis nigripes;Globicornis nigripes;1;Globicornis nigripes;1;
HQ954098;Coleoptera;Uloma culinaris;Uloma culinaris;1;Uloma culinaris;1;
HQ954193;Coleoptera;Anthaxia quadripunctata;Anthaxia quadripunctata;1;Anthaxia quadripunctata;1;
HQ954194;Coleoptera;Anthaxia quadripunctata;Anthaxia quadripunctata;1;Anthaxia quadripunctata;1;
HQ954195;Coleoptera;Anthaxia quadripunctata;Anthaxia quadripunctata;1;Anthaxia quadripunctata;1;
HQ954214;Coleoptera;Plectophloeus fischeri;Plectophloeus fischeri;1;Plectophloeus fischeri;1;
HQ954215;Coleoptera;Plectophloeus fischeri;Plectophloeus fischeri;1;Plectophloeus fischeri;1;
HQ954228;Coleoptera;Epuraea rufomarginata;Epuraea rufomarginata;1;Epuraea rufomarginata;1;
HQ954230;Coleoptera;Euryusa pipitzi;Euryusa pipitzi;1;Euryusa pipitzi;1;
HQ954231;Coleoptera;Philothermus evanescens;Philothermus evanescens;1;Philothermus evanescens;1;
HQ954257;Coleoptera;Uloma rufa;Uloma rufa;1;Uloma rufa;1;
HQ954261;Coleoptera;Plectophloeus nitidus;Plectophloeus nitidus;1;Plectophloeus nitidus;1;
HQ954277;Coleoptera;Leiopus nebulosus;NA;0.12;Leiopus linnei;0.911840242395976;
HQ954291;Coleoptera;Anisotoma castanea;Anisotoma castanea;1;Anisotoma castanea;0.999999999997783;
HQ954295;Coleoptera;Phloeonomus minimus;Phloeonomus minimus;1;Phloeonomus minimus;1;
HQ954319;Coleoptera;Euryusa pipitzi;Euryusa pipitzi;1;Euryusa pipitzi;1;
HQ954320;Coleoptera;Cis comptus;Cis comptus;1;Cis comptus;1;
HQ954440;Coleoptera;Gnathotrichus materiarius;Gnathotrichus materiarius;1;Gnathotrichus materiarius;1;
HQ954453;Coleoptera;Anisotoma humeralis;Anisotoma humeralis;1;Anisotoma humeralis;0.999999999865878;
HQ954458;Coleoptera;Anthaxia helvetica;Anthaxia helvetica;1;Anthaxia helvetica;1;
HQ954460;Coleoptera;Anthaxia morio;Anthaxia morio;1;Anthaxia morio;1;
HQ954462;Coleoptera;Elateroides dermestoides;Elateroides dermestoides;1;Elateroides dermestoides;1;
HQ954475;Coleoptera;Glischrochilus quadriguttatus;Glischrochilus quadriguttatus;1;Glischrochilus quadriguttatus;1;
HQ954476;Coleoptera;Anthaxia helvetica;Anthaxia helvetica;1;Anthaxia helvetica;1;
HQ954521;Coleoptera;Tillus elongatus;NA;NA;Tillus elongatus;1;
HQ954522;Coleoptera;Dendrophilus punctatus;NA;1;Dendrophilus punctatus;1;
HQ954533;Coleoptera;Anthaxia helvetica;Anthaxia helvetica;1;Anthaxia helvetica;1;
HQ954546;Coleoptera;Elateroides dermestoides;Elateroides dermestoides;1;Elateroides dermestoides;1;
HQ954549;Coleoptera;Dendrophagus crenatus;NA;1;Dendrophagus crenatus;0.999999999999176;
HQ954567;Coleoptera;Hololepta plana;Hololepta plana;1;Hololepta plana;1;
HQ954578;Coleoptera;Triplax aenea;Triplax aenea;1;Triplax aenea;1;
HQ954581;Coleoptera;Anthaxia helvetica;Anthaxia helvetica;1;Anthaxia helvetica;1;
HQ954589;Coleoptera;Gaurotes virginea;Gaurotes virginea;1;Gaurotes virginea;1;
HQ954598;Coleoptera;Grammoptera ruficornis;Grammoptera ruficornis;1;Grammoptera ruficornis;1;
HQ954600;Coleoptera;Ptilinus pectinicornis;Ptilinus pectinicornis;1;Ptilinus pectinicornis;1;
HQ954603;Coleoptera;Bitoma crenata;Bitoma crenata;1;Bitoma crenata;1;
HQ954605;Coleoptera;Gaurotes virginea;Gaurotes virginea;1;Gaurotes virginea;1;
HQ954607;Coleoptera;Denticollis linearis;Denticollis linearis;1;Denticollis linearis;1;
HQ954612;Coleoptera;Grammoptera ruficornis;NA;NA;Grammoptera ruficornis;1;
ICRYO026-22;Coleoptera;Gnorimus variabilis;Gnorimus variabilis;1;Gnorimus variabilis;1;
ICRYO043-22;Coleoptera;Gnorimus variabilis;Gnorimus variabilis;1;Gnorimus variabilis;1;
ICRYO046-22;Coleoptera;Gnorimus variabilis;Gnorimus variabilis;1;Gnorimus variabilis;1;
ICRYO047-22;Coleoptera;Gnorimus variabilis;Gnorimus variabilis;1;Gnorimus variabilis;1;
JF889475;Coleoptera;Evodinus clathratus;Evodinus clathratus;1;Evodinus clathratus;1;

JF889483;Coleoptera;Bitoma crenata;Bitoma crenata;1;Bitoma crenata;1;
JF889486;Coleoptera;Platystomos albinus;NA;NA;Platystomos albinus;1;
JF889496;Coleoptera;Glischrochilus quadriguttatus;Glischrochilus quadriguttatus;1;Glischrochilus quadriguttatus;1;
JF889509;Coleoptera;Tritoma bipustulata;NA;1;Tritoma bipustulata;1;
JF889510;Coleoptera;Anthaxia quadripunctata;Anthaxia quadripunctata;1;Anthaxia quadripunctata;1;
JF889529;Coleoptera;Anthaxia helvetica;Anthaxia helvetica;1;Anthaxia helvetica;1;
JF889530;Coleoptera;Triplax russica;Triplax russica;1;Triplax russica;1;
JF889541;Coleoptera;Plagionotus arcuatus;Plagionotus arcuatus;1;Plagionotus arcuatus;0.999999999385665;
JF889544;Coleoptera;Gnorimus nobilis;Gnorimus nobilis;1;Gnorimus nobilis;1;
JF889566;Coleoptera;Homophthalmus rugicollis;Dryophilus rugicollis;1;Dryophilus rugicollis;1;
JF889567;Coleoptera;Homophthalmus rugicollis;NA;1;Dryophilus rugicollis;1;
JF889591;Coleoptera;Placusa atrata;Placusa atrata;1;Placusa atrata;1;
JF889605;Coleoptera;Placonotus testaceus;Placonotus testaceus;1;Placonotus testaceus;1;
JF889610;Coleoptera;Polygraphus grandiclava;Polygraphus grandiclava;1;Polygraphus grandiclava;0.999999994686647;
JF889631;Coleoptera;Gnorimus nobilis;Gnorimus nobilis;1;Gnorimus nobilis;1;
JF889662;Coleoptera;Aulonium trisulcum;Aulonium trisulcum;1;Aulonium trisulcum;1;
JF889767;Coleoptera;Trixagus obtusus;Trixagus obtusus;1;Trixagus obtusus;1;
JF889805;Coleoptera;Anisoxya fuscula;NA;NA;Anisoxya fuscula;1;
JF889810;Coleoptera;Uloma culinaris;Uloma culinaris;1;Uloma culinaris;1;
JF889811;Coleoptera;Uloma culinaris;Uloma culinaris;1;Uloma culinaris;1;
JN262175;Coleoptera;Hemicoelus nitidus;NA;1;Anobium nitidum;1;
JN299245;Coleoptera;Cis hispidus;NA;0.317;Cis hispidus;0.99999991378775;
JN299251;Coleoptera;Trixagus atticus;Trixagus atticus;1;Trixagus atticus;1;
JN299252;Coleoptera;Trixagus obtusus;Trixagus obtusus;1;Trixagus obtusus;1;
JN299263;Coleoptera;Exocentrus adspersus;Exocentrus adspersus;1;Exocentrus adspersus;1;
JN299264;Coleoptera;Exocentrus adspersus;Exocentrus adspersus;1;Exocentrus adspersus;1;
JSBIC178-18;Coleoptera;Anthaxia hungarica;NA;NA;Anthaxia hungarica;1;
JX263851;Coleoptera;Polygraphus poligraphus;Polygraphus poligraphus;1;Polygraphus poligraphus;1;
JX263874;Coleoptera;Trypodendron domesticum;Trypodendron domesticum;1;Trypodendron domesticum;1;
JX626111;Coleoptera;Euplectus infirmus;NA;NA;NA;0.863866703233651;
JX626112;Coleoptera;Phloeonomus punctipennis;NA;NA;Paraphloeostiba gayndahensis;0.999851520634078;
JX626113;Coleoptera;Phloeonomus punctipennis;NA;NA;Paraphloeostiba gayndahensis;0.999851520634078;
JX626114;Coleoptera;Phloeonomus punctipennis;NA;NA;Paraphloeostiba gayndahensis;0.999851520634078;
JX626115;Coleoptera;Phloeonomus punctipennis;NA;NA;Paraphloeostiba gayndahensis;0.999851520634078;
JX626116;Coleoptera;Phloeonomus punctipennis;NA;NA;Paraphloeostiba gayndahensis;0.999851520634078;
JX626117;Coleoptera;Phloeonomus punctipennis;NA;NA;Paraphloeostiba gayndahensis;0.999851520634078;
JX626118;Coleoptera;Phloeonomus punctipennis;NA;NA;Paraphloeostiba gayndahensis;0.999851520634078;
JX626121;Coleoptera;Phloeonomus punctipennis;Phloeonomus punctipennis;1;NA;0.714368338948055;
JX969627;Coleoptera;Herophila tristis;Herophila tristis;1;Herophila tristis;1;
KC132742;Coleoptera;Homalota plana;Homalota plana;1;NA;0.742578693066678;
KC132825;Coleoptera;Phloeocharis subtilissima;Phloeocharis subtilissima;1;NA;0.999999999999961;
KC845472;Coleoptera;Hylastes attenuatus;Hylastes attenuatus;1;Hylastes attenuatus;0.999999999993179;
KC845477;Coleoptera;Ips sexdentatus;Ips sexdentatus;NA;Ips sexdentatus;0.999872233445088;
KC845495;Coleoptera;Polygraphus grandiclava;Polygraphus grandiclava;1;Polygraphus grandiclava;1;
KC845496;Coleoptera;Polygraphus poligraphus;Polygraphus poligraphus;1;Polygraphus poligraphus;1;
KC845497;Coleoptera;Polygraphus poligraphus;Polygraphus poligraphus;1;Polygraphus poligraphus;1;
KC845498;Coleoptera;Polygraphus poligraphus;Polygraphus poligraphus;1;Polygraphus poligraphus;1;
KC845499;Coleoptera;Polygraphus poligraphus;Polygraphus poligraphus;1;Polygraphus poligraphus;1;
KC845515;Coleoptera;Tomicus piniperda;NA;NA;Tomicus piniperda;1;
KC845516;Coleoptera;Trypodendron lineatum;Trypodendron lineatum;NA;Trypodendron lineatum;0.999999975336181;
KC845517;Coleoptera;Trypodendron domesticum;Trypodendron domesticum;1;Trypodendron domesticum;0.999999999996163;
KC845519;Coleoptera;Xyleborinus saxesenii;Xyleborinus saxeseni;1;Xyleborinus saxeseni;0.999999956550313;
KJ768196;Coleoptera;Agrilus viridis;Agrilus viridis;1;Agrilus viridis;0.999998600333824;
KJ961779;Coleoptera;Leptusa fumida;Leptusa fumida;1;Leptusa fumida;1;
KJ961810;Coleoptera;Glischrochilus quadripunctatus;Glischrochilus quadripunctatus;1;Glischrochilus quadripunctatus;0.999999919932694;
KJ961811;Coleoptera;Anisotoma axillaris;Anisotoma axillaris;1;Anisotoma axillaris;0.999999999942276;
KJ961860;Coleoptera;Placusa complanata;Placusa complanata;1;Placusa complanata;1;
KJ961988;Coleoptera;Dendrophagus crenatus;NA;1;Dendrophagus crenatus;0.999999999906237;
KJ962118;Coleoptera;Bitoma crenata;Bitoma crenata;1;Bitoma crenata;1;
KJ962141;Coleoptera;Anisotoma humeralis;Anisotoma humeralis;1;Anisotoma humeralis;0.999999999966832;
KJ962176;Coleoptera;Bitoma crenata;Bitoma crenata;1;Bitoma crenata;1;

KJ962257;Coleoptera;Platystomos albinus;NA;NA;Platystomos albinus;1;
KJ962304;Coleoptera;Glischrochilus quadripunctatus;Glischrochilus quadripunctatus;0.001;Glischrochilus quadripunctatus;0.999999973027173;
KJ962357;Coleoptera;Epuraea unicolor;Epuraea unicolor;1;Epuraea unicolor;0.999999999999972;
KJ962400;Coleoptera;Placusa tachyporoides;Placusa tachyporoides;1;Placusa tachyporoides;0.999999995754933;
KJ962435;Coleoptera;Cis comptus;Cis comptus;1;Cis comptus;0.999999999999716;
KJ962449;Coleoptera;Denticollis linearis;Denticollis linearis;1;Denticollis linearis;1;
KJ962530;Coleoptera;Phaenops cyanea;Phaenops cyanea;1;Phaenops cyanea;0.999999999999886;
KJ962580;Coleoptera;Atrecus pilicornis;Atrecus pilicornis;1;Atrecus pilicornis;1;
KJ962596;Coleoptera;Phaenops cyanea;Phaenops cyanea;1;Phaenops cyanea;0.999999999999886;
KJ962616;Coleoptera;Phaenops cyanea;Phaenops cyanea;1;Phaenops cyanea;0.999999999999886;
KJ962690;Coleoptera;Anisotoma humeralis;Anisotoma humeralis;1;Anisotoma humeralis;0.999999999966633;
KJ962719;Coleoptera;Phaenops cyanea;Phaenops cyanea;1;Phaenops cyanea;0.999999999999886;
KJ962721;Coleoptera;Anthaxia quadripunctata;Anthaxia quadripunctata;1;Anthaxia quadripunctata;0.999999998992223;
KJ962762;Coleoptera;Triplax russica;Anidorus nigrinus;1;Anidorus nigrinus;0.999999999999346;
KJ962798;Coleoptera;Ptilinus fuscus;Ptilinus fuscus;1;Ptilinus fuscus;1;
KJ962955;Coleoptera;Anisotoma glabra;Anisotoma glabra;1;Anisotoma glabra;1;
KJ962959;Coleoptera;Tomicus piniperda;NA;NA;Tomicus piniperda;1;
KJ962975;Coleoptera;Anisotoma humeralis;Anisotoma humeralis;1;Anisotoma humeralis;0.99999999995751;
KJ962986;Coleoptera;Plagionotus arcuatus;Plagionotus arcuatus;1;Plagionotus arcuatus;0.999999999542467;
KJ963023;Coleoptera;Platystomos albinus;NA;NA;Platystomos albinus;1;
KJ963035;Coleoptera;Hylurgops palliatus;Hylurgops palliatus;1;Hylurgops palliatus;1;
KJ963054;Coleoptera;Triplax russica;Triplax russica;1;Triplax russica;1;
KJ963086;Coleoptera;Epuraea silacea;Epuraea silacea;1;Epuraea silacea;1;
KJ963141;Coleoptera;Glischrochilus hortensis;Glischrochilus hortensis;1;Glischrochilus hortensis;1;
KJ963149;Coleoptera;Leiopus nebulosus;NA;NA;Leiopus nebulosus;0.920372595577046;
KJ963217;Coleoptera;Platycerus caprea;Platycerus caprea;1;Platycerus caprea;1;
KJ963243;Coleoptera;Elateroides dermestoides;Elateroides dermestoides;1;Elateroides dermestoides;1;
KJ963293;Coleoptera;Phloeonomus punctipennis;Phloeonomus punctipennis;1;Phloeonomus punctipennis;1;
KJ963449;Coleoptera;Gaurotes virginea;Gaurotes virginea;1;Gaurotes virginea;1;
KJ963460;Coleoptera;Epuraea rufomarginata;Epuraea rufomarginata;1;Epuraea rufomarginata;1;
KJ963461;Coleoptera;Anthaxia quadripunctata;Anthaxia quadripunctata;1;Anthaxia quadripunctata;0.999999998992223;
KJ963521;Coleoptera;Anisotoma axillaris;Anisotoma axillaris;1;Anisotoma axillaris;0.999999999931191;
KJ963578;Coleoptera;Cis comptus;Cis comptus;1;Cis comptus;1;
KJ963632;Coleoptera;Denticollis linearis;Denticollis linearis;1;Denticollis linearis;1;
KJ963715;Coleoptera;Triplax rufipes;Triplax rufipes;1;Triplax rufipes;1;
KJ963750;Coleoptera;Glischrochilus quadripunctatus;Glischrochilus quadripunctatus;1;Glischrochilus quadripunctatus;0.999993324385826;
KJ963755;Coleoptera;Exocentrus lusitanus;Exocentrus lusitanus;1;Exocentrus lusitanus;1;
KJ963831;Coleoptera;Platystomos albinus;NA;NA;Platystomos albinus;1;
KJ963913;Coleoptera;Ptiliola kunzei;Ptiliola kunzei;1;Ptiliola kunzei;0.999999999992752;
KJ963915;Coleoptera;Ptiliola kunzei;NA;NA;Ptiliola kunzei;0.999999999999972;
KJ963987;Coleoptera;Cis comptus;Cis comptus;1;Cis comptus;1;
KJ964055;Coleoptera;Anisotoma castanea;Anisotoma castanea;1;Anisotoma castanea;0.99999999999892;
KJ964057;Coleoptera;Anthaxia quadripunctata;Anthaxia quadripunctata;1;Anthaxia quadripunctata;0.999999998992223;
KJ964076;Coleoptera;Epuraea variegata;Epuraea variegata;1;Epuraea variegata;0.999999999840242;
KJ964190;Coleoptera;Ptilinus fuscus;Ptilinus fuscus;1;Ptilinus fuscus;1;
KJ964261;Coleoptera;Triplax russica;Triplax russica;1;Triplax russica;1;
KJ964262;Coleoptera;Triplax aenea;Triplax aenea;1;Triplax aenea;1;
KJ964373;Coleoptera;Tritoma bipustulata;NA;1;Tritoma bipustulata;1;
KJ964375;Coleoptera;Homalota plana;NA;NA;Homalota plana;1;
KJ964393;Coleoptera;Gaurotes virginea;Gaurotes virginea;1;Gaurotes virginea;1;
KJ964414;Coleoptera;Glischrochilus hortensis;Glischrochilus hortensis;1;Glischrochilus hortensis;1;
KJ964440;Coleoptera;Epuraea silacea;Epuraea silacea;1;Epuraea silacea;1;
KJ964522;Coleoptera;Epuraea terminalis;Epuraea terminalis;1;Epuraea terminalis;0.999999449064925;
KJ964531;Coleoptera;Atrecus affinis;NA;NA;Atrecus affinis;1;
KJ964564;Coleoptera;Trypodendron domesticum;Trypodendron domesticum;1;Trypodendron domesticum;1;
KJ964583;Coleoptera;Leiopus nebulosus;NA;NA;Leiopus nebulosus;0.920372595577046;
KJ964694;Coleoptera;Tomicus piniperda;Tomicus piniperda;NA;Tomicus piniperda;1;
KJ964717;Coleoptera;Platycerus caprea;Platycerus caprea;1;Platycerus caprea;1;
KJ964727;Coleoptera;Tritoma bipustulata;NA;1;Tritoma bipustulata;1;
KJ964833;Coleoptera;Epuraea unicolor;Epuraea unicolor;1;Epuraea unicolor;0.999999999999972;
KJ964916;Coleoptera;Epuraea variegata;Epuraea variegata;1;Epuraea variegata;0.999999999840242;
KJ964924;Coleoptera;Latridius consimilis;Latridius consimilis;1;Latridius consimilis;1;
KJ965159;Coleoptera;Latridius consimilis;Latridius consimilis;1;Latridius consimilis;1;

KJ965184;Coleoptera;Epuraea rufomarginata;Epuraea rufomarginata;1;Epuraea rufomarginata;1;
KJ965302;Coleoptera;Anisotoma orbicularis;Anisotoma orbicularis;1;Anisotoma orbicularis;1;
KJ965313;Coleoptera;Platycerus caprea;Platycerus caprea;1;Platycerus caprea;1;
KJ965420;Coleoptera;Denticollis linearis;Denticollis linearis;1;Denticollis linearis;1;
KJ965551;Coleoptera;Epuraea terminalis;Epuraea terminalis;1;Epuraea terminalis;0.999999270360273;
KJ965630;Coleoptera;Xylotrechus antilope;Xylotrechus antilope;1;Xylotrechus antilope;1;
KJ965679;Coleoptera;Atomaria vespertina;Atomaria vespertina;NA;Atomaria vespertina;1;
KJ965985;Coleoptera;Cis glabratus;Cis glabratus;1;Cis glabratus;1;
KJ966084;Coleoptera;Platystomos albinus;NA;NA;Platystomos albinus;1;
KJ966141;Coleoptera;Homalota plana;NA;NA;Homalota plana;1;
KJ966197;Coleoptera;Cis dentatus;Cis dentatus;1;Cis dentatus;1;
KJ966247;Coleoptera;Triplax aenea;Triplax aenea;1;Triplax aenea;1;
KJ966357;Coleoptera;Lepturobosca virens;Lepturobosca virens;NA;Lepturobosca virens;1;
KJ966373;Coleoptera;Anisotoma axillaris;Anisotoma axillaris;1;Anisotoma axillaris;0.999999999942276;
KJ966375;Coleoptera;Pteryx suturalis;Pteryx suturalis;NA;Pteryx suturalis;1;
KJ966407;Coleoptera;Exocentrus lusitanus;Exocentrus lusitanus;1;Exocentrus lusitanus;1;
KJ966409;Coleoptera;Triplax scutellaris;Orthocladius consobrinus;1;Orthocladius consobrinus;0.999999999779263;
KJ966449;Coleoptera;Epuraea silacea;Epuraea silacea;1;Epuraea silacea;1;
KJ966452;Coleoptera;Bibloporus minutus;Bibloporus minutus;1;Bibloporus minutus;1;
KJ966525;Coleoptera;Plagionotus arcuatus;Plagionotus arcuatus;1;Plagionotus arcuatus;0.999999999576602;
KJ966542;Coleoptera;Anoplodera sexguttata;Anoplodera sexguttata;1;Anoplodera sexguttata;1;
KJ966638;Coleoptera;Euryptilium saxonicum;NA;1;Euryptilium saxonicum;1;
KJ966653;Coleoptera;Exocentrus lusitanus;Exocentrus lusitanus;1;Exocentrus lusitanus;1;
KJ966697;Coleoptera;Placusa tachyporoides;Placusa tachyporoides;1;Placusa tachyporoides;0.999999995754933;
KJ966706;Coleoptera;Trypodendron domesticum;Trypodendron domesticum;1;Trypodendron domesticum;1;
KJ966717;Coleoptera;Lepturobosca virens;Lepturobosca virens;NA;Lepturobosca virens;1;
KJ966743;Coleoptera;Platycerus caprea;Platycerus caprea;1;Platycerus caprea;1;
KJ966781;Coleoptera;Cis castaneus;Cis nitidus;1;Cis nitidus;0.999999999999204;
KJ966783;Coleoptera;Epuraea silacea;Epuraea silacea;1;Epuraea silacea;1;
KJ966886;Coleoptera;Triplax russica;Triplax russica;1;Triplax russica;1;
KJ966890;Coleoptera;Ptilinus fuscus;Ptilinus fuscus;1;Ptilinus fuscus;1;
KJ966901;Coleoptera;Triplax russica;Triplax russica;1;Triplax russica;1;
KJ966914;Coleoptera;Anisotoma glabra;Anisotoma glabra;1;Anisotoma glabra;1;
KJ966952;Coleoptera;Plagionotus arcuatus;Plagionotus arcuatus;1;Plagionotus arcuatus;0.999999999777685;
KJ967008;Coleoptera;Leiopus nebulosus;NA;0.122;Leiopus nebulosus;0.950639243882467;
KJ967020;Coleoptera;Anisotoma glabra;Anisotoma glabra;1;Anisotoma glabra;1;
KJ967104;Coleoptera;Anthaxia quadripunctata;Anthaxia quadripunctata;1;Anthaxia quadripunctata;0.999999998992223;
KJ967194;Coleoptera;Elateroides dermestoides;Elateroides dermestoides;1;Elateroides dermestoides;1;
KJ967222;Coleoptera;Elateroides dermestoides;Elateroides dermestoides;1;Elateroides dermestoides;1;
KJ967231;Coleoptera;Lepturobosca virens;Lepturobosca virens;NA;Lepturobosca virens;1;
KJ967299;Coleoptera;Plagionotus detritus;Plagionotus detritus;1;Plagionotus detritus;0.999999998971703;
KJ967318;Coleoptera;Trixagus carinifrons;Trixagus carinifrons;1;Trixagus carinifrons;1;
KJ967320;Coleoptera;Leiopus nebulosus;NA;NA;Leiopus nebulosus;0.933011854612307;
KJ967373;Coleoptera;Anisotoma glabra;Anisotoma glabra;1;Anisotoma glabra;1;
KJ967406;Coleoptera;Platystomos albinus;NA;NA;Platystomos albinus;1;
KJ967452;Coleoptera;Denticollis linearis;Denticollis linearis;1;Denticollis linearis;1;
KJ967467;Coleoptera;Triplax rufipes;Triplax rufipes;1;Triplax rufipes;1;
KM285757;Coleoptera;Tillus elongatus;NA;NA;Tillus elongatus;1;
KM285777;Coleoptera;Gnorimus variabilis;Gnorimus variabilis;1;Gnorimus variabilis;1;
KM285787;Coleoptera;Xyleborus monographus;Xyleborus monographus;1;Xyleborus monographus;0.999999999999858;
KM285789;Coleoptera;Tropideres albirostris;Tropideres albirostris;1;Tropideres albirostris;1;
KM285815;Coleoptera;Plagionotus arcuatus;Plagionotus arcuatus;1;Plagionotus arcuatus;0.999999995534239;
KM285820;Coleoptera;Hypoganus inunctus;Hypoganus inunctus;1;Hypoganus inunctus;1;
KM285829;Coleoptera;Grammoptera ustulata;Grammoptera ustulata;1;Grammoptera ustulata;1;
KM285854;Coleoptera;Hemicoelus fulvicornis;NA;NA;Anobium fulvicorne;1;
KM285858;Coleoptera;Trypodendron domesticum;Trypodendron domesticum;1;Trypodendron domesticum;1;
KM285864;Coleoptera;Plagionotus arcuatus;Plagionotus arcuatus;1;Plagionotus arcuatus;0.999999995534239;
KM285865;Coleoptera;Anisandrus dispar;Anisandrus dispar;1;Anisandrus dispar;1;
KM285873;Coleoptera;Hymenalia rufipes;Hymenalia rufipes;NA;Hymenalia rufipes;1;
KM285894;Coleoptera;Phaenops formaneki;Phaenops formaneki;1;Phaenops formaneki;1;
KM285906;Coleoptera;Triplax lepida;Triplax lepida;1;Triplax lepida;1;
KM285921;Coleoptera;Platystomos albinus;NA;NA;Platystomos albinus;1;
KM285932;Coleoptera;Platystomos albinus;NA;NA;Platystomos albinus;1;
KM285945;Coleoptera;Hypulus quercinus;Hypulus quercinus;1;Hypulus quercinus;1;
KM285950;Coleoptera;Tillus elongatus;Tillus elongatus;1;Tillus elongatus;0.999999999999886;

KM285958;Coleoptera;Xyleborinus saxesenii;Xyleborinus saxeseni;1;Xyleborinus saxeseni;1;
KM285961;Coleoptera;Xyleborinus saxesenii;Xyleborinus saxeseni;1;Xyleborinus saxeseni;0.999999999999972;
KM285986;Coleoptera;Ptilinus fuscus;Ptilinus fuscus;1;Ptilinus fuscus;1;
KM285992;Coleoptera;Elateroides dermestoides;Elateroides dermestoides;1;Elateroides dermestoides;1;
KM285995;Coleoptera;Xyleborus monographus;Xyleborus monographus;1;Xyleborus monographus;0.999999999999744;
KM285996;Coleoptera;Tillus elongatus;Tillus elongatus;NA;Tillus elongatus;0.999999931515733;
KM286027;Coleoptera;Grammoptera abdominalis;Grammoptera abdominalis;1;Grammoptera abdominalis;1;
KM286028;Coleoptera;Denticollis linearis;Denticollis linearis;1;Denticollis linearis;0.999999999999972;
KM286039;Coleoptera;Anisotoma humeralis;Anisotoma humeralis;1;Anisotoma humeralis;0.999999999953076;
KM286049;Coleoptera;Ptilinus pectinicornis;Ptilinus pectinicornis;1;Ptilinus pectinicornis;1;
KM286059;Coleoptera;Exocentrus adspersus;Exocentrus adspersus;1;Exocentrus adspersus;1;
KM286060;Coleoptera;Anisotoma humeralis;Anisotoma humeralis;1;Anisotoma humeralis;0.999999999979735;
KM286061;Coleoptera;Xestobium rufovillosum;NA;1;Xestobium rufovillosum;1;
KM286071;Coleoptera;Tillus elongatus;Tillus elongatus;1;Tillus elongatus;1;
KM286084;Coleoptera;Evodinus clathratus;Evodinus clathratus;1;Evodinus clathratus;0.999999999999858;
KM286085;Coleoptera;Xyleborus dryographus;Xyleborus dryographus;1;Xyleborus dryographus;0.999999999999602;
KM286090;Coleoptera;Leptura aurulenta;Leptura aurulenta;1;Leptura aurulenta;1;
KM286097;Coleoptera;Exocentrus lusitanus;Exocentrus lusitanus;1;Exocentrus lusitanus;1;
KM286103;Coleoptera;Xyleborus dryographus;Xyleborus dryographus;1;Xyleborus dryographus;0.999999999999602;
KM286119;Coleoptera;Prinobius myardi;Prinobius myardi;NA;Prinobius myardi;0.999999989951107;
KM286134;Coleoptera;Trypodendron domesticum;Trypodendron domesticum;1;Trypodendron domesticum;1;
KM286136;Coleoptera;Grynocharis oblonga;NA;1;Grynocharis oblonga;1;
KM286137;Coleoptera;Xyleborus monographus;Xyleborus monographus;1;Xyleborus monographus;0.999999999999972;
KM286147;Coleoptera;Hylastes angustatus;Hylastes angustatus;1;Hylastes angustatus;1;
KM286150;Coleoptera;Grammoptera ruficornis;Grammoptera ruficornis;1;Grammoptera ruficornis;1;
KM286155;Coleoptera;Anisandrus dispar;Anisandrus dispar;1;Anisandrus dispar;1;
KM286157;Coleoptera;Hylastes attenuatus;Hylastes attenuatus;1;Hylastes attenuatus;0.999999999998423;
KM286158;Coleoptera;Hypoganus inunctus;Hypoganus inunctus;1;Hypoganus inunctus;1;
KM286179;Coleoptera;Hemicoelus nitidus;NA;1;Anobium nitidum;1;
KM286193;Coleoptera;Hylastes attenuatus;Hylastes attenuatus;1;Hylastes attenuatus;1;
KM286214;Coleoptera;Xyleborus monographus;Xyleborus monographus;1;Xyleborus monographus;0.999999999999915;
KM286232;Coleoptera;Hypulus quercinus;Hypulus quercinus;1;Hypulus quercinus;1;
KM286240;Coleoptera;Zilora obscura;Zilora obscura;NA;Zilora obscura;1;
KM286242;Coleoptera;Xyleborus monographus;Xyleborus monographus;1;Xyleborus monographus;0.999999999999915;
KM286261;Coleoptera;Eucnemis capucina;Eucnemis capucina;1;Eucnemis capucina;1;
KM286270;Coleoptera;Ptilinus pectinicornis;Ptilinus pectinicornis;1;Ptilinus pectinicornis;0.999999999999972;
KM286279;Coleoptera;Gnorimus nobilis;Gnorimus nobilis;1;Gnorimus nobilis;1;
KM286288;Coleoptera;Etorofus pubescens;Etorofus pubescens;1;Etorofus pubescens;1;
KM286293;Coleoptera;Tritoma bipustulata;NA;1;Tritoma bipustulata;1;
KM286296;Coleoptera;Lepturobosca virens;Lepturobosca virens;1;Lepturobosca virens;1;
KM286312;Coleoptera;Anoplodera sexguttata;Anoplodera sexguttata;1;Anoplodera sexguttata;1;
KM286364;Coleoptera;Xyleborinus saxesenii;Xyleborinus saxeseni;1;Xyleborinus saxeseni;0.999999999999972;
KM286366;Coleoptera;Vincenzellus ruficollis;NA;NA;Vincenzellus ruficollis;0.999999999995921;
KM286378;Coleoptera;Ptilinus pectinicornis;Ptilinus pectinicornis;1;Ptilinus pectinicornis;1;
KM286381;Coleoptera;Anisandrus dispar;Anisandrus dispar;1;Anisandrus dispar;0.999999999999787;
KM286393;Coleoptera;Hypoganus inunctus;Hypoganus inunctus;1;Hypoganus inunctus;1;
KM439130;Coleoptera;Hesperus rufipennis;Hesperus rufipennis;1;Hesperus rufipennis;1;
KM439151;Coleoptera;Leptoplectus spinolae;Leptoplectus spinolae;1;Leptoplectus spinolae;1;
KM439192;Coleoptera;Exocentrus adspersus;Exocentrus adspersus;1;Exocentrus adspersus;1;
KM439195;Coleoptera;Platystomos albinus;Platystomos albinus;1;Platystomos albinus;1;
KM439208;Coleoptera;Grammoptera ruficornis;Grammoptera ruficornis;1;Grammoptera ruficornis;1;
KM439226;Coleoptera;Dendrophilus pygmaeus;Dendrophilus pygmaeus;1;Dendrophilus pygmaeus;1;
KM439289;Coleoptera;Trixagus obtusus;Trixagus obtusus;1;Trixagus obtusus;1;
KM439292;Coleoptera;Gaurotes virginea;Gaurotes virginea;NA;Gaurotes virginea;1;
KM439323;Coleoptera;Bibloporus minutus;Bibloporus minutus;1;Bibloporus minutus;0.999999999999972;
KM439328;Coleoptera;Aulonothroscus brevicollis;Aulonothroscus brevicollis;1;Aulonothroscus brevicollis;1;
KM439345;Coleoptera;Anthaxia candens;Anthaxia candens;1;Anthaxia candens;1;
KM439348;Coleoptera;Trypodendron domesticum;Trypodendron domesticum;1;Trypodendron domesticum;1;
KM439349;Coleoptera;Denticollis linearis;Denticollis linearis;1;Denticollis linearis;1;
KM439373;Coleoptera;Epuraea unicolor;Epuraea unicolor;1;Epuraea unicolor;0.999999999999972;
KM439387;Coleoptera;Hymenalia rufipes;Hymenalia rufipes;NA;Hymenalia rufipes;1;

KM439418;Coleoptera;Euryusa optabilis;Euryusa optabilis;1;Euryusa optabilis;0.999999999941679;
KM439430;Coleoptera;Leiopus nebulosus;NA;0.12;Leiopus linnei;0.911840242395976;
KM439494;Coleoptera;Plectophloeus nubigena;Plectophloeus nubigena;1;Plectophloeus nubigena;1;
KM439519;Coleoptera;Hesperus rufipennis;Hesperus rufipennis;1;Hesperus rufipennis;1;
KM439597;Coleoptera;Trixagus carinifrons;Trixagus carinifrons;1;Trixagus carinifrons;1;
KM439599;Coleoptera;Atrecus affinis;NA;NA;Atrecus affinis;1;
KM439610;Coleoptera;Placusa pumilio;Placusa pumilio;1;Placusa pumilio;1;
KM439634;Coleoptera;Euplectus nanus;NA;0.048;NA;0.999999999999987;
KM439635;Coleoptera;Triplax russica;Triplax russica;1;Triplax russica;1;
KM439643;Coleoptera;Anoplodera sexguttata;Anoplodera sexguttata;1;Anoplodera sexguttata;1;
KM439679;Coleoptera;Potamophilus acuminatus;Potamophilus acuminatus;NA;Potamophilus acuminatus;1;
KM439693;Coleoptera;Platystomos albinus;NA;NA;Platystomos albinus;1;
KM439774;Coleoptera;Philothermus evanescens;NA;1;Philothermus evanescens;1;
KM439798;Coleoptera;Uloma rufa;Uloma rufa;1;Uloma rufa;1;
KM439800;Coleoptera;Valgus hemipterus;Valgus hemipterus;1;Valgus hemipterus;1;
KM439851;Coleoptera;Epuraea unicolor;Epuraea unicolor;1;Epuraea unicolor;0.999999999999972;
KM439901;Coleoptera;Cis hispidus;NA;0.317;Cis hispidus;0.99999991378775;
KM439965;Coleoptera;Tritoma bipustulata;NA;1;Tritoma bipustulata;1;
KM439969;Coleoptera;Denticollis linearis;Denticollis linearis;1;Denticollis linearis;1;
KM439972;Coleoptera;Anisotoma humeralis;Anisotoma humeralis;1;Anisotoma humeralis;0.999999999979735;
KM439982;Coleoptera;Ptilinus pectinicornis;Ptilinus pectinicornis;1;Ptilinus pectinicornis;0.999999999999943;
KM440013;Coleoptera;Phloeocharis subtilissima;Phloeocharis subtilissima;NA;Phloeocharis subtilissima;1;
KM440017;Coleoptera;Polygraphus grandiclava;Polygraphus grandiclava;1;Polygraphus grandiclava;0.999999987585937;
KM440036;Coleoptera;Xyleborus dryographus;Xyleborus dryographus;1;Xyleborus dryographus;0.999999999150049;
KM440075;Coleoptera;Phloeonomus punctipennis;Phloeonomus punctipennis;1;Phloeonomus punctipennis;1;
KM440078;Coleoptera;Latridius minutus;Latridius minutus;1;Latridius minutus;0.999999999993065;
KM440085;Coleoptera;Anthaxia candens;Anthaxia candens;1;Anthaxia candens;1;
KM440092;Coleoptera;Valgus hemipterus;Valgus hemipterus;1;Valgus hemipterus;1;
KM440150;Coleoptera;Philothermus evanescens;Philothermus evanescens;1;Philothermus evanescens;1;
KM440161;Coleoptera;Anthaxia helvetica;Anthaxia helvetica;1;Anthaxia helvetica;1;
KM440169;Coleoptera;Uloma rufa;Uloma rufa;1;Uloma rufa;1;
KM440171;Coleoptera;Uloma culinaris;Uloma culinaris;1;Uloma culinaris;1;
KM440191;Coleoptera;Cis glabratus;Cis glabratus;1;Cis glabratus;1;
KM440209;Coleoptera;Pteryngium crenatum;Pteryngium crenatum;1;Pteryngium crenatum;1;
KM440229;Coleoptera;Exocentrus adspersus;Exocentrus adspersus;1;Exocentrus adspersus;1;
KM440253;Coleoptera;Placusa depressa;Placusa depressa;1;Placusa depressa;0.99999933037358;
KM440262;Coleoptera;Batrisus formicarius;Batrisus formicarius;1;Batrisus formicarius;1;
KM440269;Coleoptera;Anthaxia quadripunctata;Anthaxia quadripunctata;1;Anthaxia quadripunctata;1;
KM440278;Coleoptera;Epuraea rufomarginata;Epuraea rufomarginata;1;Epuraea rufomarginata;0.999999999999829;
KM440283;Coleoptera;Uloma culinaris;Uloma culinaris;1;Uloma culinaris;1;
KM440319;Coleoptera;Hesperus rufipennis;Hesperus rufipennis;1;Hesperus rufipennis;1;
KM440321;Coleoptera;Cis glabratus;Cis glabratus;1;Cis glabratus;1;
KM440331;Coleoptera;Polygraphus grandiclava;Polygraphus grandiclava;1;Polygraphus grandiclava;0.999999989897674;
KM440348;Coleoptera;Placusa depressa;Placusa depressa;1;Placusa depressa;0.999999998185473;
KM440379;Coleoptera;Hypnogyra angularis;Hypnogyra glabra;1;Hypnogyra glabra;1;
KM440380;Coleoptera;Glischrochilus quadripunctatus;Glischrochilus quadripunctatus;0.02;Glischrochilus quadripunctatus;0.999999898249092;
KM440395;Coleoptera;Agrilus viridis;Agrilus viridis;1;Agrilus viridis;0.999999816014269;
KM440408;Coleoptera;Placusa tachyporoides;Placusa tachyporoides;1;Placusa tachyporoides;0.999999995754933;
KM440415;Coleoptera;Tomicus piniperda;NA;NA;Tomicus piniperda;1;
KM440465;Coleoptera;Phloeonomus punctipennis;Phloeonomus punctipennis;1;Phloeonomus punctipennis;1;
KM440476;Coleoptera;Xestobium rufovillosum;NA;1;Xestobium rufovillosum;1;
KM440485;Coleoptera;Hypulus quercinus;Hypulus quercinus;1;Hypulus quercinus;1;
KM440600;Coleoptera;Anthaxia quadripunctata;Anthaxia quadripunctata;1;Anthaxia quadripunctata;1;
KM440605;Coleoptera;Ptiliolum caledonicum;Ptiliolum caledonicum;1;Ptiliolum caledonicum;1;
KM440623;Coleoptera;Berginus tamarisci;NA;1;Berginus tamarisci;1;
KM440628;Coleoptera;Euryusa optabilis;Euryusa optabilis;1;Euryusa optabilis;0.999999999908795;
KM440639;Coleoptera;Trypodendron domesticum;Trypodendron domesticum;1;Trypodendron domesticum;1;
KM440666;Coleoptera;Platystomos albinus;NA;NA;Platystomos albinus;1;
KM440681;Coleoptera;Plectophloeus nubigena;Plectophloeus nubigena;1;Plectophloeus nubigena;1;
KM440702;Coleoptera;Xylostiba bosnica;Xylostiba bosnica;NA;Xylostiba bosnica;1;
KM440746;Coleoptera;Pteryx suturalis;NA;NA;Pteryx suturalis;1;
KM440784;Coleoptera;Uloma culinaris;Uloma culinaris;1;Uloma culinaris;1;
KM440786;Coleoptera;Denticollis linearis;Denticollis linearis;1;Denticollis linearis;1;
KM440802;Coleoptera;Plectophloeus nitidus;Plectophloeus nitidus;1;Plectophloeus nitidus;1;
KM440806;Coleoptera;Euplectus nanus;NA;NA;Euplectus nanus;0.715342734986947;

KM440811;Coleoptera;Glischrochilus quadriguttatus;Glischrochilus quadriguttatus;1;Glischrochilus quadriguttatus;1;
KM440836;Coleoptera;Plectophloeus nitidus;Plectophloeus nitidus;1;Plectophloeus nitidus;1;
KM440844;Coleoptera;Uloma rufa;Uloma rufa;1;Uloma rufa;1;
KM440850;Coleoptera;Dendrophilus pygmaeus;Dendrophilus pygmaeus;1;Dendrophilus pygmaeus;1;
KM440871;Coleoptera;Epuraea thoracica;Epuraea thoracica;1;Epuraea thoracica;0.999982218697291;
KM440887;Coleoptera;Hypebaeus flavipes;Hypebaeus flavipes;1;Hypebaeus flavipes;1;
KM440894;Coleoptera;Epuraea unicolor;Epuraea unicolor;1;Epuraea unicolor;0.999999999999972;
KM440903;Coleoptera;Philothermus evanescens;NA;1;Philothermus evanescens;1;
KM440911;Coleoptera;Epuraea thoracica;Epuraea thoracica;1;Epuraea thoracica;0.999989250075486;
KM440986;Coleoptera;Evodinus clathratus;Evodinus clathratus;1;Evodinus clathratus;1;
KM441027;Coleoptera;Gaurotes virginea;Gaurotes virginea;1;Gaurotes virginea;1;
KM441090;Coleoptera;Leptusa fumida;Leptusa fumida;1;Leptusa fumida;1;
KM441091;Coleoptera;Pteryx suturalis;NA;NA;Pteryx suturalis;1;
KM441107;Coleoptera;Plagionotus detritus;Plagionotus detritus;1;Plagionotus detritus;0.999999999661156;
KM441115;Coleoptera;Plectophloeus nubigena;Plectophloeus nubigena;1;Plectophloeus nubigena;1;
KM441125;Coleoptera;Epuraea unicolor;Epuraea unicolor;1;Epuraea unicolor;1;
KM441126;Coleoptera;Uloma rufa;Uloma rufa;1;Uloma rufa;1;
KM441127;Coleoptera;Euplectus kirbii;NA;0.166;Euplectus kirbyi;0.978419763277652;
KM441149;Coleoptera;Euryusa optabilis;Euryusa optabilis;1;Euryusa optabilis;0.99999999997533;
KM441197;Coleoptera;Anthaxia quadripunctata;Anthaxia quadripunctata;1;Anthaxia quadripunctata;1;
KM441229;Coleoptera;Trixagus obtusus;Trixagus obtusus;1;Trixagus obtusus;1;
KM441236;Coleoptera;Lepturobosca virens;Lepturobosca virens;1;Lepturobosca virens;1;
KM441239;Coleoptera;Anthaxia mendizabali;Anthaxia mendizabali;1;Anthaxia mendizabali;1;
KM441284;Coleoptera;Anthaxia istriana;Anthaxia istriana;1;Anthaxia istriana;1;
KM441311;Coleoptera;Lepturobosca virens;Lepturobosca virens;1;Lepturobosca virens;1;
KM441350;Coleoptera;Anthaxia mendizabali;Anthaxia mendizabali;1;Anthaxia mendizabali;1;
KM441353;Coleoptera;Anthaxia fulgurans;NA;NA;Anthaxia fulgurans;1;
KM441402;Coleoptera;Hypebaeus flavipes;Hypebaeus flavipes;1;Hypebaeus flavipes;0.999999999999858;
KM441405;Coleoptera;Euplectus punctatus;Euplectus punctatus;1;Euplectus punctatus;1;
KM441443;Coleoptera;Euryusa castanoptera;Euryusa castanoptera;NA;Euryusa castanoptera;1;
KM441559;Coleoptera;Euryusa optabilis;Euryusa optabilis;1;Euryusa optabilis;0.999999999966349;
KM441579;Coleoptera;Epuraea variegata;Epuraea variegata;1;Epuraea variegata;0.999999999941821;
KM441583;Coleoptera;Anthaxia candens;Anthaxia candens;1;Anthaxia candens;1;
KM441599;Coleoptera;Euryusa castanoptera;Euryusa castanoptera;NA;Euryusa castanoptera;1;
KM441622;Coleoptera;Glischrochilus quadriguttatus;Glischrochilus quadriguttatus;1;Glischrochilus quadriguttatus;1;
KM441633;Coleoptera;Tomicus piniperda;NA;NA;Tomicus piniperda;1;
KM441637;Coleoptera;Plectophloeus fischeri;Plectophloeus fischeri;1;Plectophloeus fischeri;1;
KM441693;Coleoptera;Bibloporus bicolor;Bibloporus bicolor;1;Bibloporus bicolor;0.999999999999574;
KM441700;Coleoptera;Cis comptus;Cis comptus;1;Cis comptus;1;
KM441702;Coleoptera;Ernobius abietinus;Ernobius abietinus;1;Ernobius abietinus;1;
KM441727;Coleoptera;Anthaxia quadripunctata;Anthaxia quadripunctata;1;Anthaxia quadripunctata;1;
KM441734;Coleoptera;Anthaxia istriana;Anthaxia istriana;1;Anthaxia istriana;1;
KM441761;Coleoptera;Hymenalia rufipes;Hymenalia rufipes;1;Hymenalia rufipes;1;
KM441773;Coleoptera;Dendrophilus pygmaeus;Dendrophilus pygmaeus;1;Dendrophilus pygmaeus;1;
KM441788;Coleoptera;Homalota plana;Homalota plana;NA;Homalota plana;1;
KM441833;Coleoptera;Euplectus kirbii;NA;0.124;NA;1;
KM441862;Coleoptera;Plectophloeus nubigena;Plectophloeus nubigena;1;Plectophloeus nubigena;1;
KM441898;Coleoptera;Placusa pumilio;Placusa pumilio;1;Placusa pumilio;1;
KM441908;Coleoptera;Glischrochilus quadriguttatus;Glischrochilus quadriguttatus;1;Glischrochilus quadriguttatus;1;
KM441926;Coleoptera;Euplectus infirmus;Euplectus infirmus;1;Euplectus infirmus;1;
KM441934;Coleoptera;Uloma culinaris;Uloma culinaris;1;Uloma culinaris;1;
KM441946;Coleoptera;Atrecus affinis;Atrecus affinis;1;Atrecus affinis;1;
KM441984;Coleoptera;Anthaxia salicis;Anthaxia salicis;1;Anthaxia salicis;1;
KM441987;Coleoptera;Dendrophilus pygmaeus;Dendrophilus pygmaeus;1;Dendrophilus pygmaeus;1;
KM441997;Coleoptera;Exocentrus adspersus;NA;NA;Exocentrus adspersus;1;
KM442016;Coleoptera;Cis glabratus;Cis glabratus;1;Cis glabratus;1;
KM442021;Coleoptera;Hypoganus inunctus;Hypoganus inunctus;1;Hypoganus inunctus;1;
KM442034;Coleoptera;Euryusa optabilis;Euryusa optabilis;1;Euryusa optabilis;0.99999999997533;
KM442051;Coleoptera;Trixagus carinifrons;Trixagus carinifrons;1;Trixagus carinifrons;1;
KM442072;Coleoptera;Cis dentatus;Cis dentatus;1;Cis dentatus;1;
KM442098;Coleoptera;Trypodendron domesticum;Trypodendron domesticum;1;Trypodendron domesticum;1;
KM442130;Coleoptera;Exocentrus adspersus;Exocentrus adspersus;1;Exocentrus adspersus;1;
KM442141;Coleoptera;Plectophloeus nitidus;Plectophloeus nitidus;1;Plectophloeus nitidus;1;
KM442161;Coleoptera;Troglops albicans;Troglops albicans;NA;Troglops albicans;1;
KM442177;Coleoptera;Plagionotus detritus;Plagionotus detritus;1;Plagionotus detritus;0.999999998971703;
KM442198;Coleoptera;Leptusa pulchella;Leptusa pulchella;1;Leptusa pulchella;1;
KM442208;Coleoptera;Polygraphus poligraphus;Polygraphus poligraphus;1;Polygraphus poligraphus;0.999999999993719;
KM442239;Coleoptera;Triplax rufipes;Triplax rufipes;1;Triplax rufipes;1;
KM442307;Coleoptera;Hypoganus inunctus;Hypoganus inunctus;1;Hypoganus inunctus;1;
KM442312;Coleoptera;Tritoma bipustulata;NA;1;Tritoma bipustulata;1;

KM442328;Coleoptera;Hypnogyra angularis;Hypnogyra glabra;1;Hypnogyra glabra;1;
KM442418;Coleoptera;Anthaxia quadripunctata;Anthaxia quadripunctata;1;Anthaxia quadripunctata;1;
KM442425;Coleoptera;Gnorimus variabilis;Gnorimus variabilis;1;Gnorimus variabilis;1;
KM442468;Coleoptera;Grammoptera ustulata;Grammoptera ustulata;1;Grammoptera ustulata;1;
KM442472;Coleoptera;Aulonothroscus brevicollis;Aulonothroscus brevicollis;1;Aulonothroscus brevicollis;1;
KM442520;Coleoptera;Cis dentatus;Cis dentatus;1;Cis dentatus;1;
KM442531;Coleoptera;Anisotoma orbicularis;Anisotoma orbicularis;1;Anisotoma orbicularis;1;
KM442582;Coleoptera;Trixagus obtusus;Trixagus obtusus;1;Trixagus obtusus;1;
KM442608;Coleoptera;Anthaxia fulgurans;Anthaxia fulgurans;1;Anthaxia fulgurans;1;
KM442611;Coleoptera;Uloma rufa;Uloma rufa;1;Uloma rufa;1;
KM442629;Coleoptera;Aulonothroscus brevicollis;Aulonothroscus brevicollis;1;Aulonothroscus brevicollis;1;
KM442640;Coleoptera;Bitoma crenata;Bitoma crenata;1;Bitoma crenata;1;
KM442674;Coleoptera;Xylostiba bosnica;Xylostiba bosnica;NA;Xylostiba bosnica;1;
KM442677;Coleoptera;Epuraea unicolor;Epuraea unicolor;1;Epuraea unicolor;0.999999999999972;
KM442680;Coleoptera;Euplectus infirmus;Euplectus infirmus;1;Euplectus infirmus;1;
KM442694;Coleoptera;Exocentrus adspersus;NA;NA;Exocentrus adspersus;1;
KM442703;Coleoptera;Cis glabratus;Cis glabratus;1;Cis glabratus;1;
KM442748;Coleoptera;Leptoplectus spinolae;Leptoplectus spinolae;1;Leptoplectus spinolae;1;
KM442774;Coleoptera;Ptiliolum caledonicum;Ptiliolum caledonicum;1;Ptiliolum caledonicum;1;
KM442801;Coleoptera;Xylotrechus antilope;Xylotrechus antilope;1;Xylotrechus antilope;1;
KM442875;Coleoptera;Hesperus rufipennis;Hesperus rufipennis;1;Hesperus rufipennis;1;
KM442881;Coleoptera;Aulonothroscus brevicollis;Aulonothroscus brevicollis;1;Aulonothroscus brevicollis;1;
KM442885;Coleoptera;Vincenzellus ruficollis;NA;NA;Vincenzellus ruficollis;1;
KM442890;Coleoptera;Trixagus carinifrons;Trixagus carinifrons;1;Trixagus carinifrons;1;
KM442917;Coleoptera;Tillus elongatus;NA;NA;Tillus elongatus;1;
KM442948;Coleoptera;Euplectus infirmus;Euplectus infirmus;1;Euplectus infirmus;1;
KM442950;Coleoptera;Hypnogyra angularis;Hypnogyra glabra;1;Hypnogyra glabra;1;
KM442977;Coleoptera;Denticollis linearis;Denticollis linearis;1;Denticollis linearis;1;
KM442983;Coleoptera;Homalota plana;Homalota plana;1;Homalota plana;1;
KM443017;Coleoptera;Homalota plana;NA;NA;Homalota plana;1;
KM443020;Coleoptera;Leptusa fumida;Leptusa fumida;1;Leptusa fumida;1;
KM443070;Coleoptera;Euplectus nanus;NA;0.319;NA;0.999999999999995;
KM443078;Coleoptera;Euplectus karstenii;Euplectus karstenii;1;Euplectus karstenii;1;
KM443088;Coleoptera;Anisotoma humeralis;Anisotoma humeralis;1;Anisotoma humeralis;0.999999999980901;
KM443097;Coleoptera;Dendrophilus pygmaeus;Dendrophilus pygmaeus;1;Dendrophilus pygmaeus;1;
KM443099;Coleoptera;Anisotoma glabra;Anisotoma glabra;1;Anisotoma glabra;1;
KM443148;Coleoptera;Euplectus piceus;Euplectus piceus;1;Euplectus piceus;1;
KM443202;Coleoptera;Atrecus affinis;NA;NA;Atrecus affinis;1;
KM443212;Coleoptera;Placusa tachyporoides;Placusa tachyporoides;1;Placusa tachyporoides;0.999999999019337;
KM443237;Coleoptera;Uloma culinaris;Uloma culinaris;1;Uloma culinaris;1;
KM443251;Coleoptera;Trixagus obtusus;Trixagus obtusus;1;Trixagus obtusus;1;
KM443269;Coleoptera;Dendrophilus punctatus;NA;1;Dendrophilus punctatus;1;
KM443278;Coleoptera;Plectophloeus fischeri;Plectophloeus fischeri;1;Plectophloeus fischeri;1;
KM443302;Coleoptera;Trypodendron domesticum;Trypodendron domesticum;1;Trypodendron domesticum;1;
KM443322;Coleoptera;Homalota plana;Homalota plana;NA;Homalota plana;1;
KM443334;Coleoptera;Euplectus karstenii;Euplectus karstenii;1;Euplectus karstenii;1;
KM443336;Coleoptera;Leptura aurulenta;Leptura aurulenta;1;Leptura aurulenta;1;
KM443385;Coleoptera;Attalus analis;Attalus analis;1;Attalus analis;1;
KM443392;Coleoptera;Anthaxia istriana;Anthaxia istriana;1;Anthaxia istriana;1;
KM443412;Coleoptera;Triplax rufipes;Triplax rufipes;1;Triplax rufipes;1;
KM443425;Coleoptera;Placusa tachyporoides;Placusa tachyporoides;1;Placusa tachyporoides;0.999999995754933;
KM443426;Coleoptera;Grammoptera ruficornis;Grammoptera ruficornis;1;Grammoptera ruficornis;1;
KM443430;Coleoptera;Plectophloeus nitidus;Plectophloeus nitidus;1;Plectophloeus nitidus;1;
KM443481;Coleoptera;Euplectus nanus;NA;0.135;NA;0.999999999999996;
KM443489;Coleoptera;Plagionotus arcuatus;Plagionotus arcuatus;1;Plagionotus arcuatus;0.999999997790724;
KM443511;Coleoptera;Hypnogyra angularis;Hypnogyra glabra;1;Hypnogyra glabra;1;
KM443540;Coleoptera;Anisotoma orbicularis;Anisotoma orbicularis;1;Anisotoma orbicularis;1;
KM443570;Coleoptera;Euplectus punctatus;Euplectus punctatus;1;Euplectus punctatus;1;
KM443591;Coleoptera;Anisotoma humeralis;Anisotoma humeralis;1;Anisotoma humeralis;0.999999999979735;
KM443603;Coleoptera;Tillus elongatus;NA;NA;Tillus elongatus;1;
KM443617;Coleoptera;Ptiliolum caledonicum;Ptiliolum caledonicum;1;Ptiliolum caledonicum;1;
KM443679;Coleoptera;Gnorimus variabilis;Gnorimus variabilis;1;Gnorimus variabilis;1;
KM443692;Coleoptera;Anthaxia fulgurans;NA;NA;Anthaxia fulgurans;1;
KM443721;Coleoptera;Anthaxia podolica;Anthaxia podolica;1;Anthaxia podolica;1;
KM443734;Coleoptera;Potamophilus acuminatus;Potamophilus acuminatus;NA;Potamophilus acuminatus;1;
KM443736;Coleoptera;Glischrochilus hortensis;Glischrochilus hortensis;1;Glischrochilus hortensis;1;
KM443754;Coleoptera;Atrecus longiceps;NA;NA;Atrecus longiceps;1;
KM443767;Coleoptera;Grammoptera ruficornis;Grammoptera ruficornis;1;Grammoptera ruficornis;1;

KM443781;Coleoptera;Grammoptera ustulata;Grammoptera ustulata;1;Grammoptera ustulata;1;
KM443803;Coleoptera;Cis comptus;Cis comptus;1;Cis comptus;1;
KM443806;Coleoptera;Ptiliolum caledonicum;Ptiliolum caledonicum;1;Ptiliolum caledonicum;1;
KM443818;Coleoptera;Placusa atrata;Placusa atrata;1;Placusa atrata;1;
KM443842;Coleoptera;Atrecus affinis;NA;NA;Atrecus affinis;1;
KM443849;Coleoptera;Plectophloeus nubigena;Plectophloeus nubigena;1;Plectophloeus nubigena;1;
KM443857;Coleoptera;Pteryngium crenatum;Pteryngium crenatum;1;Pteryngium crenatum;1;
KM443884;Coleoptera;Hypulus quercinus;Hypulus quercinus;1;Hypulus quercinus;1;
KM443941;Coleoptera;Leptusa pulchella;Leptusa pulchella;1;Leptusa pulchella;1;
KM443957;Coleoptera;Anisandrus dispar;Anisandrus dispar;1;Anisandrus dispar;1;
KM444004;Coleoptera;Ptenidium pusillum;NA;NA;Ptenidium pusillum;0.998336450701437;
KM444016;Coleoptera;Potamophilus acuminatus;Potamophilus acuminatus;NA;Potamophilus acuminatus;1;
KM444017;Coleoptera;Exocentrus lusitanus;Exocentrus lusitanus;1;Exocentrus lusitanus;1;
KM444055;Coleoptera;Plagionotus arcuatus;Plagionotus arcuatus;1;Plagionotus arcuatus;0.999999999213742;
KM444087;Coleoptera;Denticollis linearis;Denticollis linearis;1;Denticollis linearis;1;
KM444090;Coleoptera;Phloeonomus punctipennis;Phloeonomus punctipennis;1;Phloeonomus punctipennis;1;
KM444132;Coleoptera;Anisotoma castanea;Anisotoma castanea;1;Anisotoma castanea;0.999999999999574;
KM444141;Coleoptera;Plectophloeus nitidus;Plectophloeus nitidus;1;Plectophloeus nitidus;1;
KM444149;Coleoptera;Cis comptus;Cis comptus;1;Cis comptus;1;
KM444168;Coleoptera;Hesperus rufipennis;Hesperus rufipennis;1;Hesperus rufipennis;1;
KM444186;Coleoptera;Lepturobosca virens;Lepturobosca virens;NA;Lepturobosca virens;1;
KM444195;Coleoptera;Cis dentatus;Cis dentatus;1;Cis dentatus;1;
KM444202;Coleoptera;Anisandrus dispar;Anisandrus dispar;1;Anisandrus dispar;1;
KM444221;Coleoptera;Vincenzellus ruficollis;NA;NA;Vincenzellus ruficollis;1;
KM444228;Coleoptera;Anthaxia quadripunctata;Anthaxia quadripunctata;1;Anthaxia quadripunctata;1;
KM444245;Coleoptera;Anthaxia podolica;Anthaxia podolica;1;Anthaxia podolica;1;
KM444268;Coleoptera;Atrecus pilicornis;Atrecus pilicornis;1;Atrecus pilicornis;1;
KM444332;Coleoptera;Pteryx suturalis;Pteryx suturalis;1;Pteryx suturalis;1;
KM444338;Coleoptera;Euplectus kirbii;NA;0.319;NA;0.999999999999995;
KM444347;Coleoptera;Atrecus longiceps;NA;NA;Atrecus longiceps;1;
KM444371;Coleoptera;Phloeonomus minimus;Phloeonomus minimus;NA;Phloeonomus minimus;1;
KM444379;Coleoptera;Xylostiba bosnica;Xylostiba bosnica;NA;Xylostiba bosnica;1;
KM444389;Coleoptera;Atrecus affinis;NA;NA;Atrecus affinis;1;
KM444410;Coleoptera;Hesperus rufipennis;Hesperus rufipennis;1;Hesperus rufipennis;1;
KM444413;Coleoptera;Lepturobosca virens;Lepturobosca virens;1;Lepturobosca virens;1;
KM444440;Coleoptera;Euryusa castanoptera;Euryusa castanoptera;1;Euryusa castanoptera;1;
KM444463;Coleoptera;Euplectus piceus;Euplectus piceus;1;Euplectus piceus;1;
KM444529;Coleoptera;Anthaxia nitidula;Anthaxia nitidula;1;Anthaxia nitidula;1;
KM444534;Coleoptera;Vincenzellus ruficollis;NA;NA;Vincenzellus ruficollis;1;
KM444549;Coleoptera;Phloeonomus punctipennis;Phloeonomus punctipennis;1;Phloeonomus punctipennis;1;
KM444575;Coleoptera;Euplectus karstenii;Euplectus karstenii;1;Euplectus karstenii;1;
KM444581;Coleoptera;Euryusa optabilis;Euryusa optabilis;1;Euryusa optabilis;0.99999999997533;
KM444599;Coleoptera;Vincenzellus ruficollis;NA;NA;Vincenzellus ruficollis;1;
KM444629;Coleoptera;Philothermus evanescens;Philothermus evanescens;1;Philothermus evanescens;1;
KM444647;Coleoptera;Uloma rufa;Uloma rufa;1;Uloma rufa;1;
KM444673;Coleoptera;Globicornis nigripes;Globicornis nigripes;1;Globicornis nigripes;1;
KM444674;Coleoptera;Leptusa fumida;Leptusa fumida;1;Leptusa fumida;0.999999999999972;
KM444708;Coleoptera;Cis dentatus;Cis dentatus;1;Cis dentatus;1;
KM444713;Coleoptera;Epuraea unicolor;Epuraea unicolor;1;Epuraea unicolor;0.999999999999972;
KM444716;Coleoptera;Denticollis linearis;Denticollis linearis;1;Denticollis linearis;1;
KM444748;Coleoptera;Aulonium trisulcum;Aulonium trisulcum;1;Aulonium trisulcum;1;
KM444773;Coleoptera;Plectophloeus nubigena;Plectophloeus nubigena;1;Plectophloeus nubigena;0.999999999999972;
KM444789;Coleoptera;Valgus hemipterus;Valgus hemipterus;1;Valgus hemipterus;1;
KM444799;Coleoptera;Euplectus nanus;NA;0.319;NA;0.999999999999995;
KM444812;Coleoptera;Hypulus quercinus;Hypulus quercinus;1;Hypulus quercinus;1;
KM444817;Coleoptera;Anthaxia quadripunctata;Anthaxia quadripunctata;1;Anthaxia quadripunctata;1;
KM444833;Coleoptera;Glischrochilus quadriguttatus;Glischrochilus quadriguttatus;1;Glischrochilus quadriguttatus;1;
KM444908;Coleoptera;Evodinus clathratus;Evodinus clathratus;1;Evodinus clathratus;1;
KM444915;Coleoptera;Epuraea unicolor;Epuraea unicolor;1;Epuraea unicolor;1;
KM444972;Coleoptera;Ernobius abietinus;Ernobius abietinus;1;Ernobius abietinus;1;
KM444976;Coleoptera;Plectophloeus fischeri;Plectophloeus fischeri;1;Plectophloeus fischeri;1;
KM444978;Coleoptera;Grammoptera ruficornis;NA;NA;Grammoptera ruficornis;1;
KM445036;Coleoptera;Epuraea variegata;Epuraea variegata;1;Epuraea variegata;0.99999999912265;
KM445045;Coleoptera;Trixagus obtusus;Trixagus obtusus;1;Trixagus obtusus;1;
KM445081;Coleoptera;Ptiliolum caledonicum;Ptiliolum caledonicum;1;Ptiliolum caledonicum;1;
KM445088;Coleoptera;Phloeonomus punctipennis;Phloeonomus punctipennis;1;Phloeonomus punctipennis;1;
KM445120;Coleoptera;Philothermus evanescens;Philothermus evanescens;1;Philothermus evanescens;1;
KM445160;Coleoptera;Leiopus nebulosus;NA;0.12;Leiopus linnei;0.911840242395976;
KM445188;Coleoptera;Tropideres albirostris;Tropideres albirostris;1;Tropideres albirostris;1;
KM445218;Coleoptera;Xylostiba bosnica;Xylostiba bosnica;NA;Xylostiba bosnica;1;

KM445276;Coleoptera;Leptusa fumida;Leptusa fumida;1;Leptusa fumida;0.999999999999972;
KM445297;Coleoptera;Euplectus piceus;Euplectus piceus;1;Euplectus piceus;1;
KM445303;Coleoptera;Uloma culinaris;Uloma culinaris;1;Uloma culinaris;1;
KM445313;Coleoptera;Bibloporus bicolor;Bibloporus bicolor;1;Bibloporus bicolor;0.999999999999943;
KM445316;Coleoptera;Euryusa pipitzi;Euryusa pipitzi;1;Euryusa pipitzi;1;
KM445321;Coleoptera;Cis festivus;Orthocis festivus;1;Orthocis festivus;1;
KM445330;Coleoptera;Aulonothroscus brevicollis;Aulonothroscus brevicollis;1;Aulonothroscus brevicollis;1;
KM445339;Coleoptera;Euryusa castanoptera;Euryusa castanoptera;NA;Euryusa castanoptera;1;
KM445354;Coleoptera;Anisotoma castanea;Anisotoma castanea;1;Anisotoma castanea;0.999999999999375;
KM445364;Coleoptera;Cis dentatus;Cis dentatus;1;Cis dentatus;1;
KM445414;Coleoptera;Batrisus formicarius;Batrisus formicarius;1;Batrisus formicarius;1;
KM445429;Coleoptera;Phloeonomus minimus;Phloeonomus minimus;1;Phloeonomus minimus;1;
KM445481;Coleoptera;Philothermus evanescens;Philothermus evanescens;1;Philothermus evanescens;1;
KM445489;Coleoptera;Berginus tamarisci;NA;1;Berginus tamarisci;1;
KM445527;Coleoptera;Gaurotes virginea;Gaurotes virginea;1;Gaurotes virginea;1;
KM445582;Coleoptera;Gaurotes virginea;Gaurotes virginea;1;Gaurotes virginea;1;
KM445623;Coleoptera;Attalus analis;Attalus analis;1;Attalus analis;1;
KM445640;Coleoptera;Anthaxia candens;Anthaxia candens;1;Anthaxia candens;1;
KM445667;Coleoptera;Euryusa castanoptera;Euryusa castanoptera;NA;Euryusa castanoptera;1;
KM445698;Coleoptera;Potamophilus acuminatus;Potamophilus acuminatus;NA;Potamophilus acuminatus;1;
KM445716;Coleoptera;Tillus elongatus;NA;NA;Tillus elongatus;1;
KM445728;Coleoptera;Anthaxia helvetica;Anthaxia helvetica;1;Anthaxia helvetica;1;
KM445734;Coleoptera;Grammoptera ruficornis;Grammoptera ruficornis;1;Grammoptera ruficornis;1;
KM445745;Coleoptera;Anoplodera sexguttata;Anoplodera sexguttata;1;Anoplodera sexguttata;1;
KM445749;Coleoptera;Cis festivus;Orthocis festivus;1;Orthocis festivus;1;
KM445770;Coleoptera;Cis dentatus;Cis dentatus;1;Cis dentatus;1;
KM445812;Coleoptera;Cis boleti;Cis boleti;1;Cis boleti;1;
KM445822;Coleoptera;Xyleborinus saxesenii;Xyleborinus saxeseni;1;Xyleborinus saxeseni;1;
KM445829;Coleoptera;Gnorimus variabilis;Gnorimus variabilis;1;Gnorimus variabilis;1;
KM445866;Coleoptera;Vincenzellus ruficollis;NA;NA;Vincenzellus ruficollis;1;
KM445945;Coleoptera;Euryusa optabilis;Euryusa optabilis;1;Euryusa optabilis;0.999999999966349;
KM445963;Coleoptera;Valgus hemipterus;Valgus hemipterus;1;Valgus hemipterus;1;
KM446009;Coleoptera;Xylostiba bosnica;Xylostiba bosnica;1;Xylostiba bosnica;1;
KM446030;Coleoptera;Euplectus infirmus;Euplectus infirmus;1;Euplectus infirmus;1;
KM446060;Coleoptera;Leiopus nebulosus;NA;0.12;Leiopus linnei;0.911840242395976;
KM446063;Coleoptera;Xylostiba bosnica;Xylostiba bosnica;NA;Xylostiba bosnica;1;
KM446102;Coleoptera;Leptusa pulchella;Leptusa pulchella;1;Leptusa pulchella;1;
KM446120;Coleoptera;Hemicoelus costatus;Anobium fulvicorne;1;Anobium fulvicorne;1;
KM446121;Coleoptera;Uloma rufa;Uloma rufa;1;Uloma rufa;1;
KM446141;Coleoptera;Anthaxia helvetica;Anthaxia helvetica;1;Anthaxia helvetica;1;
KM446163;Coleoptera;Anthaxia morio;Anthaxia morio;1;Anthaxia morio;1;
KM446217;Coleoptera;Tillus elongatus;NA;NA;Tillus elongatus;1;
KM446226;Coleoptera;Plectophloeus nubigena;Plectophloeus nubigena;1;Plectophloeus nubigena;1;
KM446253;Coleoptera;Dendrophilus pygmaeus;Dendrophilus pygmaeus;1;Dendrophilus pygmaeus;1;
KM446256;Coleoptera;Leptusa pulchella;Leptusa pulchella;1;Leptusa pulchella;1;
KM446257;Coleoptera;Euplectus piceus;Euplectus piceus;1;Euplectus piceus;1;
KM446328;Coleoptera;Euplectus karstenii;Euplectus karstenii;1;Euplectus karstenii;1;
KM446332;Coleoptera;Uloma rufa;Uloma rufa;1;Uloma rufa;1;
KM446368;Coleoptera;Xylotrechus antilope;Xylotrechus antilope;1;Xylotrechus antilope;1;
KM446372;Coleoptera;Aulonium trisulcum;Aulonium trisulcum;1;Aulonium trisulcum;1;
KM446388;Coleoptera;Elateroides dermestoides;Elateroides dermestoides;1;Elateroides dermestoides;1;
KM446389;Coleoptera;Epuraea thoracica;Epuraea thoracica;0.001;Epuraea thoracica;0.999951020796953;
KM446397;Coleoptera;Placusa depressa;Placusa depressa;1;Placusa depressa;0.999999760662121;
KM446403;Coleoptera;Valgus hemipterus;Valgus hemipterus;1;Valgus hemipterus;1;
KM446462;Coleoptera;Trypodendron domesticum;Trypodendron domesticum;1;Trypodendron domesticum;1;
KM446499;Coleoptera;Xestobium rufovillosum;NA;1;Xestobium rufovillosum;1;
KM446516;Coleoptera;Cis comptus;Cis comptus;1;Cis comptus;1;
KM446521;Coleoptera;Trypodendron domesticum;Trypodendron domesticum;1;Trypodendron domesticum;1;
KM446528;Coleoptera;Exocentrus adspersus;Exocentrus adspersus;1;Exocentrus adspersus;1;
KM446533;Coleoptera;Atrecus pilicornis;Atrecus pilicornis;1;Atrecus pilicornis;1;
KM446627;Coleoptera;Cis festivus;Orthocis festivus;1;Orthocis festivus;1;
KM446632;Coleoptera;Xylostiba bosnica;Xylostiba bosnica;NA;Xylostiba bosnica;1;
KM446666;Coleoptera;Anisotoma orbicularis;Anisotoma orbicularis;1;Anisotoma orbicularis;1;
KM446672;Coleoptera;Plectophloeus nitidus;Plectophloeus nitidus;1;Plectophloeus nitidus;1;
KM446680;Coleoptera;Trypodendron domesticum;Trypodendron domesticum;1;Trypodendron domesticum;1;
KM446683;Coleoptera;Vincenzellus ruficollis;NA;NA;Vincenzellus ruficollis;1;
KM446725;Coleoptera;Ptilinus fuscus;Ptilinus fuscus;1;Ptilinus fuscus;1;
KM446748;Coleoptera;Phloeonomus punctipennis;Phloeonomus punctipennis;1;Phloeonomus punctipennis;1;
KM446777;Coleoptera;Leptusa fumida;Leptusa fumida;1;Leptusa fumida;1;
KM446779;Coleoptera;Bitoma crenata;Bitoma crenata;1;Bitoma crenata;1;
KM446796;Coleoptera;Latridius minutus;Latridius minutus;1;Latridius minutus;0.99999997678566;

KM446803;Coleoptera;Placusa pumilio;Placusa pumilio;1;Placusa pumilio;1;
KM446847;Coleoptera;Hemicoelus costatus;NA;NA;Anobium fulvicorne;1;
KM446864;Coleoptera;Homophthalmus rugicollis;NA;1;Dryophilus rugicollis;1;
KM446907;Coleoptera;Platystomos albinus;NA;NA;Platystomos albinus;1;
KM446920;Coleoptera;Anoplodera sexguttata;Anoplodera sexguttata;1;Anoplodera sexguttata;1;
KM446926;Coleoptera;Pteryngium crenatum;Pteryngium crenatum;1;Pteryngium crenatum;1;
KM446953;Coleoptera;Uloma culinaris;Uloma culinaris;1;Uloma culinaris;1;
KM446967;Coleoptera;Xylotrechus antilope;Xylotrechus antilope;1;Xylotrechus antilope;1;
KM447033;Coleoptera;Cis castaneus;Cis castaneus;NA;Cis castaneus;1;
KM447034;Coleoptera;Xestobium rufovillosum;NA;1;Xestobium rufovillosum;1;
KM447055;Coleoptera;Pteryx suturalis;Pteryx suturalis;NA;Pteryx suturalis;1;
KM447099;Coleoptera;Phloeonomus punctipennis;Phloeonomus punctipennis;1;Phloeonomus punctipennis;1;
KM447108;Coleoptera;Anthaxia quadripunctata;Anthaxia quadripunctata;1;Anthaxia quadripunctata;1;
KM447112;Coleoptera;Eucnemis capucina;Eucnemis capucina;1;Eucnemis capucina;1;
KM447126;Coleoptera;Anthaxia istriana;Anthaxia istriana;1;Anthaxia istriana;1;
KM447127;Coleoptera;Pteryngium crenatum;Pteryngium crenatum;1;Pteryngium crenatum;1;
KM447171;Coleoptera;Plectophloeus fischeri;Plectophloeus fischeri;1;Plectophloeus fischeri;1;
KM447215;Coleoptera;Anthaxia godeti;Anthaxia godeti;1;Anthaxia godeti;1;
KM447221;Coleoptera;Gnathotrichus materiarius;Gnathotrichus materiarius;1;Gnathotrichus materiarius;1;
KM447260;Coleoptera;Plectophloeus nitidus;Plectophloeus nitidus;1;Plectophloeus nitidus;1;
KM447285;Coleoptera;Anthaxia helvetica;Anthaxia helvetica;1;Anthaxia helvetica;1;
KM447320;Coleoptera;Grammoptera ruficornis;Grammoptera ruficornis;1;Grammoptera ruficornis;1;
KM447330;Coleoptera;Aulonium trisulcum;Aulonium trisulcum;1;Aulonium trisulcum;1;
KM447342;Coleoptera;Euplectus karstenii;Euplectus karstenii;1;Euplectus karstenii;1;
KM447397;Coleoptera;Gnathotrichus materiarius;Gnathotrichus materiarius;1;Gnathotrichus materiarius;1;
KM447404;Coleoptera;Aulonothroscus brevicollis;Aulonothroscus brevicollis;1;Aulonothroscus brevicollis;1;
KM447419;Coleoptera;Hypnogyra angularis;Hypnogyra glabra;1;Hypnogyra glabra;1;
KM447447;Coleoptera;Aulonothroscus brevicollis;Aulonothroscus brevicollis;1;Aulonothroscus brevicollis;1;
KM447482;Coleoptera;Polygraphus poligraphus;Polygraphus poligraphus;1;Polygraphus poligraphus;0.999999999993719;
KM447495;Coleoptera;Denticollis linearis;Denticollis linearis;1;Denticollis linearis;1;
KM447510;Coleoptera;Ptiliolum caledonicum;Ptiliolum caledonicum;1;Ptiliolum caledonicum;1;
KM447526;Coleoptera;Potamophilus acuminatus;Potamophilus acuminatus;NA;Potamophilus acuminatus;1;
KM447529;Coleoptera;Anthaxia helvetica;Anthaxia helvetica;1;Anthaxia helvetica;1;
KM447567;Coleoptera;Placusa pumilio;Placusa pumilio;1;Placusa pumilio;1;
KM447574;Coleoptera;Anthaxia helvetica;Anthaxia helvetica;1;Anthaxia helvetica;1;
KM447576;Coleoptera;Hypoganus inunctus;Hypoganus inunctus;1;Hypoganus inunctus;1;
KM447629;Coleoptera;Phloeocharis subtilissima;Phloeocharis subtilissima;1;Phloeocharis subtilissima;1;
KM447646;Coleoptera;Exocentrus adspersus;Exocentrus adspersus;1;Exocentrus adspersus;1;
KM447649;Coleoptera;Anthaxia morio;Anthaxia morio;1;Anthaxia morio;1;
KM447669;Coleoptera;Xyleborus monographus;Xyleborus monographus;1;Xyleborus monographus;0.999999999999915;
KM447711;Coleoptera;Atrecus affinis;Atrecus affinis;1;Atrecus affinis;1;
KM447719;Coleoptera;Euryusa castanoptera;Euryusa castanoptera;1;Euryusa castanoptera;1;
KM447764;Coleoptera;Plectophloeus fischeri;Plectophloeus fischeri;1;Plectophloeus fischeri;1;
KM447834;Coleoptera;Bitoma crenata;Bitoma crenata;1;Bitoma crenata;1;
KM447903;Coleoptera;Vincenzellus ruficollis;Vincenzellus ruficollis;1;Vincenzellus ruficollis;1;
KM447905;Coleoptera;Anthaxia podolica;Anthaxia podolica;1;Anthaxia podolica;1;
KM447956;Coleoptera;Plectophloeus nitidus;Plectophloeus nitidus;1;Plectophloeus nitidus;1;
KM448007;Coleoptera;Anisotoma orbicularis;Anisotoma orbicularis;1;Anisotoma orbicularis;1;
KM448025;Coleoptera;Pteryngium crenatum;Pteryngium crenatum;1;Pteryngium crenatum;1;
KM448039;Coleoptera;Cis castaneus;Cis castaneus;1;Cis castaneus;1;
KM448088;Coleoptera;Denticollis linearis;Denticollis linearis;1;Denticollis linearis;1;
KM448104;Coleoptera;Hesperus rufipennis;Hesperus rufipennis;1;Hesperus rufipennis;1;
KM448112;Coleoptera;Plectophloeus nubigena;Plectophloeus nubigena;1;Plectophloeus nubigena;1;
KM448137;Coleoptera;Euplectus brunneus;Euplectus brunneus;1;Euplectus brunneus;1;
KM448149;Coleoptera;Polygraphus grandiclava;Polygraphus grandiclava;1;Polygraphus grandiclava;0.99999999772001;
KM448160;Coleoptera;Anthaxia helvetica;Anthaxia helvetica;1;Anthaxia helvetica;1;
KM448165;Coleoptera;Anthaxia godeti;Anthaxia godeti;1;Anthaxia godeti;1;
KM448177;Coleoptera;Euplectus nanus;NA;0.135;NA;0.999999999999996;
KM448250;Coleoptera;Potamophilus acuminatus;Potamophilus acuminatus;NA;Potamophilus acuminatus;1;
KM448257;Coleoptera;Vincenzellus ruficollis;NA;NA;Vincenzellus ruficollis;1;
KM448302;Coleoptera;Vincenzellus ruficollis;NA;NA;Vincenzellus ruficollis;1;
KM448338;Coleoptera;Cis dentatus;Cis dentatus;1;Cis dentatus;1;
KM448345;Coleoptera;Valgus hemipterus;Valgus hemipterus;1;Valgus hemipterus;1;
KM448353;Coleoptera;Euplectus piceus;Euplectus piceus;1;Euplectus piceus;1;
KM448418;Coleoptera;Exocentrus lusitanus;Exocentrus lusitanus;1;Exocentrus lusitanus;1;
KM448444;Coleoptera;Evodinus clathratus;Evodinus clathratus;1;Evodinus clathratus;1;
KM448450;Coleoptera;Ptiliolum marginatum;NA;NA;Ptiliolum marginatum;1;

KM448451;Coleoptera;Epuraea unicolor;Epuraea unicolor;1;Epuraea unicolor;0.999999999999972;
KM448462;Coleoptera;Xylostiba bosnica;Xylostiba bosnica;NA;Xylostiba bosnica;1;
KM448495;Coleoptera;Euplectus nanus;NA;0.132;Euplectus nanus;0.817875111887982;
KM448515;Coleoptera;Berginus tamarisci;NA;1;Berginus tamarisci;1;
KM448546;Coleoptera;Plagionotus arcuatus;Plagionotus arcuatus;1;Plagionotus arcuatus;0.999999999705665;
KM448568;Coleoptera;Euryusa optabilis;Euryusa optabilis;1;Euryusa optabilis;0.999999999908795;
KM448581;Coleoptera;Denticollis linearis;Denticollis linearis;1;Denticollis linearis;1;
KM448599;Coleoptera;Exocentrus adspersus;Exocentrus adspersus;1;Exocentrus adspersus;1;
KM448603;Coleoptera;Epuraea variegata;Epuraea variegata;1;Epuraea variegata;0.999999999704329;
KM448629;Coleoptera;Hypebaeus flavipes;Hypebaeus flavipes;1;Hypebaeus flavipes;1;
KM448634;Coleoptera;Anthaxia millefolii;Anthaxia millefolii;1;Anthaxia millefolii;1;
KM448655;Coleoptera;Hemicoelus costatus;Anobium fulvicorne;1;Anobium fulvicorne;1;
KM448656;Coleoptera;Euplectus karstenii;Euplectus karstenii;1;Euplectus karstenii;1;
KM448674;Coleoptera;Xyleborinus saxesenii;Xyleborinus saxeseni;1;Xyleborinus saxeseni;1;
KM448699;Coleoptera;Anthaxia quadripunctata;Anthaxia quadripunctata;1;Anthaxia quadripunctata;1;
KM448717;Coleoptera;Plectophloeus nubigena;Plectophloeus nubigena;1;Plectophloeus nubigena;0.999999999999972;
KM448771;Coleoptera;Xyleborus monographus;Xyleborus monographus;1;Xyleborus monographus;0.999999999999631;
KM448795;Coleoptera;Anthaxia istriana;Anthaxia istriana;1;Anthaxia istriana;1;
KM448832;Coleoptera;Plectophloeus nitidus;Plectophloeus nitidus;1;Plectophloeus nitidus;1;
KM448841;Coleoptera;Uloma culinaris;Uloma culinaris;1;Uloma culinaris;1;
KM448847;Coleoptera;Anthaxia helvetica;Anthaxia helvetica;1;Anthaxia helvetica;1;
KM448876;Coleoptera;Tritoma bipustulata;NA;1;Tritoma bipustulata;1;
KM448883;Coleoptera;Triplax rufipes;Triplax rufipes;1;Triplax rufipes;1;
KM448891;Coleoptera;Leptusa fumida;Leptusa fumida;1;Leptusa fumida;0.999999999999687;
KM448906;Coleoptera;Hypoganus inunctus;Hypoganus inunctus;1;Hypoganus inunctus;1;
KM448931;Coleoptera;Latridius anthracinus;Latridius anthracinus;1;Latridius anthracinus;1;
KM448989;Coleoptera;Epuraea pygmaea;Epuraea pygmaea;1;Epuraea pygmaea;0.999999999999915;
KM449006;Coleoptera;Atrecus longiceps;NA;NA;Atrecus longiceps;1;
KM449059;Coleoptera;Polygraphus poligraphus;Polygraphus poligraphus;1;Polygraphus poligraphus;0.999999999893191;
KM449070;Coleoptera;Troglops albicans;Troglops albicans;NA;Troglops albicans;1;
KM449098;Coleoptera;Uloma rufa;Uloma rufa;1;Uloma rufa;1;
KM449127;Coleoptera;Hesperus rufipennis;Hesperus rufipennis;1;Hesperus rufipennis;1;
KM449132;Coleoptera;Euplectus karstenii;Euplectus karstenii;1;Euplectus karstenii;0.999999999999972;
KM449160;Coleoptera;Anoplodera sexguttata;Anoplodera sexguttata;1;Anoplodera sexguttata;1;
KM449249;Coleoptera;Epuraea variegata;Epuraea variegata;1;Epuraea variegata;0.999999999840242;
KM449292;Coleoptera;Ernobius abietis;Ernobius abietis;1;Ernobius abietis;1;
KM449293;Coleoptera;Atrecus longiceps;NA;NA;Atrecus longiceps;1;
KM449325;Coleoptera;Polygraphus poligraphus;Polygraphus poligraphus;1;Polygraphus poligraphus;0.99999999999423;
KM449362;Coleoptera;Xestobium rufovillosum;NA;1;Xestobium rufovillosum;1;
KM449379;Coleoptera;Euplectus brunneus;Euplectus brunneus;1;Euplectus brunneus;1;
KM449415;Coleoptera;Cis glabratus;Cis glabratus;1;Cis glabratus;1;
KM449436;Coleoptera;Pteryngium crenatum;Pteryngium crenatum;1;Pteryngium crenatum;1;
KM449481;Coleoptera;Elateroides dermestoides;Elateroides dermestoides;1;Elateroides dermestoides;1;
KM449525;Coleoptera;Cis glabratus;Cis glabratus;1;Cis glabratus;1;
KM449530;Coleoptera;Batrisus formicarius;Batrisus formicarius;1;Batrisus formicarius;1;
KM449571;Coleoptera;Attalus analis;Attalus analis;1;Attalus analis;1;
KM449618;Coleoptera;Anisotoma orbicularis;Anisotoma orbicularis;1;Anisotoma orbicularis;1;
KM449621;Coleoptera;Hymenalia rufipes;Hymenalia rufipes;NA;Hymenalia rufipes;1;
KM449660;Coleoptera;Bibloporus bicolor;Bibloporus bicolor;1;Bibloporus bicolor;0.999999999999886;
KM449752;Coleoptera;Aulonothroscus brevicollis;Aulonothroscus brevicollis;1;Aulonothroscus brevicollis;1;
KM449770;Coleoptera;Placusa depressa;Placusa depressa;1;Placusa depressa;0.999999956008339;
KM449799;Coleoptera;Glischrochilus hortensis;Glischrochilus hortensis;1;Glischrochilus hortensis;1;
KM449800;Coleoptera;Cis comptus;Cis comptus;1;Cis comptus;1;
KM449822;Coleoptera;Tritoma bipustulata;NA;1;Tritoma bipustulata;1;
KM449827;Coleoptera;Glischrochilus hortensis;Glischrochilus hortensis;NA;Glischrochilus hortensis;1;
KM449844;Coleoptera;Ernobius abietis;NA;1;Ernobius abietis;1;
KM449845;Coleoptera;Bitoma crenata;Bitoma crenata;1;Bitoma crenata;1;
KM449883;Coleoptera;Hemicoelus nitidus;Anobium nitidum;1;Anobium nitidum;1;
KM449885;Coleoptera;Xylostiba bosnica;Xylostiba bosnica;NA;Xylostiba bosnica;1;
KM449896;Coleoptera;Hymenalia rufipes;Hymenalia rufipes;NA;Hymenalia rufipes;1;
KM449949;Coleoptera;Bitoma crenata;Bitoma crenata;1;Bitoma crenata;1;
KM449954;Coleoptera;Hypnogyra angularis;Hypnogyra glabra;1;Hypnogyra glabra;1;
KM449998;Coleoptera;Dendrophilus punctatus;NA;1;Dendrophilus punctatus;1;
KM449999;Coleoptera;Philothermus evanescens;Philothermus evanescens;1;Philothermus evanescens;1;
KM450023;Coleoptera;Cis glabratus;Cis glabratus;1;Cis glabratus;1;
KM450071;Coleoptera;Xylostiba bosnica;Xylostiba bosnica;NA;Xylostiba bosnica;1;
KM450072;Coleoptera;Bitoma crenata;Bitoma crenata;1;Bitoma crenata;1;

KM450088;Coleoptera;Platystomos albinus;NA;NA;Platystomos albinus;1;
KM450106;Coleoptera;Plegaderus caesus;Plegaderus caesus;1;Plegaderus caesus;1;
KM450118;Coleoptera;Agrilus viridis;Agrilus viridis;1;Agrilus viridis;0.999999746205109;
KM450143;Coleoptera;Anthaxia helvetica;Anthaxia helvetica;1;Anthaxia helvetica;1;
KM450191;Coleoptera;Anthaxia quadripunctata;Anthaxia quadripunctata;1;Anthaxia quadripunctata;1;
KM450239;Coleoptera;Gnorimus variabilis;Gnorimus variabilis;1;Gnorimus variabilis;1;
KM450289;Coleoptera;Hypnogyra angularis;Hypnogyra glabra;1;Hypnogyra glabra;1;
KM450340;Coleoptera;Euplectus kirbii;NA;0.041;Euplectus kirbyi;0.939713583326614;
KM450355;Coleoptera;Epuraea variegata;Epuraea variegata;1;Epuraea variegata;0.999999999840242;
KM450380;Coleoptera;Euryusa optabilis;Euryusa optabilis;1;Euryusa optabilis;0.99999999997533;
KM450396;Coleoptera;Anthaxia istriana;Anthaxia istriana;1;Anthaxia istriana;1;
KM450403;Coleoptera;Anthaxia fulgurans;Anthaxia fulgurans;1;Anthaxia fulgurans;1;
KM450450;Coleoptera;Bitoma crenata;Bitoma crenata;1;Bitoma crenata;1;
KM450455;Coleoptera;Glischrochilus quadriguttatus;Glischrochilus quadriguttatus;1;Glischrochilus quadriguttatus;1;
KM450469;Coleoptera;Anoplodera sexguttata;Anoplodera sexguttata;1;Anoplodera sexguttata;1;
KM450511;Coleoptera;Leiopus nebulosus;NA;0.12;Leiopus linnei;0.911840242395976;
KM450512;Coleoptera;Plectophloeus nubigena;Plectophloeus nubigena;1;Plectophloeus nubigena;1;
KM450553;Coleoptera;Bibloporus bicolor;Bibloporus bicolor;1;Bibloporus bicolor;0.999999999999972;
KM450556;Coleoptera;Anthaxia fulgurans;NA;NA;Anthaxia fulgurans;1;
KM450575;Coleoptera;Trixagus carinifrons;Trixagus carinifrons;1;Trixagus carinifrons;1;
KM450584;Coleoptera;Anoplodera sexguttata;Anoplodera sexguttata;1;Anoplodera sexguttata;1;
KM450590;Coleoptera;Plegaderus caesus;Plegaderus caesus;1;Plegaderus caesus;1;
KM450621;Coleoptera;Tillus elongatus;NA;NA;Tillus elongatus;1;
KM450643;Coleoptera;Phloeonomus punctipennis;Phloeonomus punctipennis;1;Phloeonomus punctipennis;1;
KM450668;Coleoptera;Exocentrus adspersus;NA;NA;Exocentrus adspersus;1;
KM450683;Coleoptera;Ernobius abietis;Ernobius abietis;1;Ernobius abietis;1;
KM450689;Coleoptera;Cis festivus;Orthocis festivus;1;Orthocis festivus;1;
KM450744;Coleoptera;Hololepta plana;Hololepta plana;1;Hololepta plana;1;
KM450773;Coleoptera;Tillus elongatus;NA;NA;Tillus elongatus;1;
KM450803;Coleoptera;Cis hispidus;NA;NA;Cis hispidus;0.999999793431037;
KM450819;Coleoptera;Euplectus nanus;NA;0.105;NA;0.999999999999987;
KM450841;Coleoptera;Polygraphus poligraphus;Polygraphus poligraphus;1;Polygraphus poligraphus;0.999999999993719;
KM450871;Coleoptera;Bitoma crenata;Bitoma crenata;1;Bitoma crenata;1;
KM450882;Coleoptera;Bitoma crenata;Bitoma crenata;1;Bitoma crenata;1;
KM450996;Coleoptera;Anisotoma castanea;Anisotoma castanea;1;Anisotoma castanea;0.999999999995168;
KM451038;Coleoptera;Anoplodera sexguttata;Anoplodera sexguttata;1;Anoplodera sexguttata;1;
KM451070;Coleoptera;Euplectus karstenii;Euplectus karstenii;1;Euplectus karstenii;1;
KM451104;Coleoptera;Grammoptera ruficornis;Grammoptera ruficornis;1;Grammoptera ruficornis;1;
KM451107;Coleoptera;Exocentrus adspersus;Exocentrus adspersus;1;Exocentrus adspersus;1;
KM451176;Coleoptera;Anisotoma humeralis;Anisotoma humeralis;1;Anisotoma humeralis;0.999999999979991;
KM451263;Coleoptera;Bibloporus bicolor;Bibloporus bicolor;1;Bibloporus bicolor;0.999999999999943;
KM451284;Coleoptera;Euplectus piceus;Euplectus piceus;1;Euplectus piceus;1;
KM451290;Coleoptera;Leiopus femoratus;Leiopus femoratus;1;Leiopus femoratus;1;
KM451321;Coleoptera;Plectophloeus nitidus;Plectophloeus nitidus;1;Plectophloeus nitidus;1;
KM451354;Coleoptera;Pteryx suturalis;Pteryx suturalis;NA;Pteryx suturalis;1;
KM451386;Coleoptera;Leptoplectus spinolae;Leptoplectus spinolae;1;Leptoplectus spinolae;1;
KM451408;Coleoptera;Cis castaneus;Cis castaneus;1;Cis castaneus;1;
KM451425;Coleoptera;Anthaxia helvetica;Anthaxia helvetica;1;Anthaxia helvetica;1;
KM451431;Coleoptera;Cis glabratus;Cis glabratus;1;Cis glabratus;1;
KM451507;Coleoptera;Hypoganus inunctus;Hypoganus inunctus;1;Hypoganus inunctus;1;
KM451512;Coleoptera;Grammoptera ruficornis;Grammoptera ruficornis;1;Grammoptera ruficornis;1;
KM451533;Coleoptera;Euplectus nanus;NA;NA;NA;0.999999999999993;
KM451543;Coleoptera;Euryusa optabilis;Euryusa optabilis;1;Euryusa optabilis;0.99999999997533;
KM451567;Coleoptera;Tillus elongatus;Tillus elongatus;1;Tillus elongatus;1;
KM451588;Coleoptera;Grammoptera ruficornis;Grammoptera ruficornis;1;Grammoptera ruficornis;1;
KM451589;Coleoptera;Glischrochilus quadripunctatus;Glischrochilus quadripunctatus;0.002;Glischrochilus quadripunctatus;0.999999698889542;
KM451591;Coleoptera;Berginus tamarisci;NA;1;Berginus tamarisci;1;
KM451643;Coleoptera;Gnorimus nobilis;Gnorimus nobilis;1;Gnorimus nobilis;1;
KM451646;Coleoptera;Anthaxia istriana;Anthaxia istriana;1;Anthaxia istriana;1;
KM451655;Coleoptera;Placusa tachyporoides;Placusa tachyporoides;1;Placusa tachyporoides;0.999999999886654;
KM451658;Coleoptera;Trixagus obtusus;Trixagus obtusus;1;Trixagus obtusus;1;
KM451674;Coleoptera;Hymenalia rufipes;Hymenalia rufipes;NA;Hymenalia rufipes;1;
KM451676;Coleoptera;Anthaxia mendizabali;Anthaxia mendizabali;1;Anthaxia mendizabali;1;
KM451700;Coleoptera;Hesperus rufipennis;Hesperus rufipennis;1;Hesperus rufipennis;1;
KM451701;Coleoptera;Cis castaneus;Cis castaneus;1;Cis castaneus;1;
KM451710;Coleoptera;Philothermus evanescens;NA;1;Philothermus evanescens;1;
KM451744;Coleoptera;Evodinus clathratus;Evodinus clathratus;1;Evodinus clathratus;1;
KM451763;Coleoptera;Anthaxia godeti;Anthaxia godeti;1;Anthaxia godeti;1;
KM451805;Coleoptera;Anthaxia quadripunctata;Anthaxia quadripunctata;1;Anthaxia quadripunctata;1;

KM451813;Coleoptera;Elateroides dermestoides;NA;1;Elateroides dermestoides;1;
KM451840;Coleoptera;Placusa atrata;Placusa atrata;1;Placusa atrata;1;
KM451864;Coleoptera;Anisandrus dispar;Anisandrus dispar;1;Anisandrus dispar;1;
KM451953;Coleoptera;Leptura aethiops;Leptura aethiops;1;Leptura aethiops;1;
KM451961;Coleoptera;Plagionotus arcuatus;Plagionotus arcuatus;1;Plagionotus
arcuatus;0.999999999038835;
KM451983;Coleoptera;Anisotoma castanea;Anisotoma castanea;1;Anisotoma
castanea;0.999999999999289;
KM452012;Coleoptera;Glischrochilus quadriguttatus;Glischrochilus quadriguttatus;1;Glischrochilus
quadriguttatus;1;
KM452059;Coleoptera;Atrecus affinis;NA;NA;Atrecus affinis;1;
KM452083;Coleoptera;Anthaxia fulgurans;Anthaxia fulgurans;1;Anthaxia fulgurans;1;
KM452085;Coleoptera;Aulonothroscus brevicollis;Aulonothroscus brevicollis;1;Aulonothroscus
brevicollis;1;
KM452105;Coleoptera;Hololepta plana;Hololepta plana;1;Hololepta plana;1;
KM452183;Coleoptera;Lepturobosca virens;Lepturobosca virens;1;Lepturobosca virens;1;
KM452188;Coleoptera;Homalota plana;NA;NA;Homalota plana;1;
KM452233;Coleoptera;Ernobius abietinus;Ernobius abietinus;1;Ernobius abietinus;1;
KM452248;Coleoptera;Exocentrus lusitanus;Exocentrus lusitanus;1;Exocentrus lusitanus;1;
KM452262;Coleoptera;Anisotoma humeralis;Anisotoma humeralis;1;Anisotoma
humeralis;0.999999999912376;
KM452268;Coleoptera;Bitoma crenata;Bitoma crenata;1;Bitoma crenata;1;
KM452273;Coleoptera;Tillus elongatus;NA;NA;Tillus elongatus;1;
KM452282;Coleoptera;Cis dentatus;Cis dentatus;1;Cis dentatus;1;
KM452286;Coleoptera;Vincenzellus ruficollis;NA;NA;Vincenzellus ruficollis;1;
KM452292;Coleoptera;Cis hispidus;NA;0.107;Cis hispidus;0.999999939132836;
KM452358;Coleoptera;Elateroides dermestoides;NA;1;Elateroides dermestoides;1;
KM452376;Coleoptera;Placusa tachyporoides;Placusa tachyporoides;1;Placusa
tachyporoides;0.999999995754933;
KM452390;Coleoptera;Bitoma crenata;Bitoma crenata;1;Bitoma crenata;1;
KM452392;Coleoptera;Xyleborinus saxesenii;Xyleborinus saxeseni;1;Xyleborinus saxeseni;1;
KM452416;Coleoptera;Anthaxia candens;Anthaxia candens;1;Anthaxia candens;1;
KM452423;Coleoptera;Batrisus formicarius;Batrisus formicarius;1;Batrisus formicarius;1;
KM452502;Coleoptera;Uloma rufa;Uloma rufa;1;Uloma rufa;1;
KM452510;Coleoptera;Epuraea unicolor;Epuraea unicolor;1;Epuraea unicolor;0.999999999999943;
KM452520;Coleoptera;Hylastes attenuatus;Hylastes attenuatus;1;Hylastes attenuatus;1;
KM452555;Coleoptera;Euplectus infirmus;Euplectus infirmus;1;Euplectus infirmus;1;
KM452571;Coleoptera;Tropideres albirostris;Tropideres albirostris;1;Tropideres albirostris;1;
KM452588;Coleoptera;Atrecus affinis;Atrecus affinis;1;Atrecus affinis;1;
KM452592;Coleoptera;Hesperus rufipennis;Hesperus rufipennis;NA;Hesperus rufipennis;1;
KM452643;Coleoptera;Uloma rufa;Uloma rufa;1;Uloma rufa;1;
KM452649;Coleoptera;Pteryx suturalis;NA;NA;Pteryx suturalis;1;
KM452657;Coleoptera;Anthaxia quadripunctata;Anthaxia quadripunctata;1;Anthaxia quadripunctata;1;
KM452662;Coleoptera;Anthaxia morio;Anthaxia morio;1;Anthaxia morio;1;
KM452677;Coleoptera;Platystomos albinus;Platystomos albinus;1;Platystomos albinus;1;
KM452678;Coleoptera;Latridius minutus;Latridius minutus;1;Latridius minutus;0.999999999993065;
KM452694;Coleoptera;Xyleborinus saxesenii;Xyleborinus saxeseni;1;Xyleborinus saxeseni;1;
KU494206;Coleoptera;Priobium carpini;Priobium carpini;1;Priobium carpini;1;
KU494207;Coleoptera;Priobium carpini;Priobium carpini;1;Priobium carpini;1;
KU494208;Coleoptera;Priobium carpini;Priobium carpini;1;Priobium carpini;1;
KU494211;Coleoptera;Ptilinus pectinicornis;Ptilinus pectinicornis;1;Ptilinus
pectinicornis;0.999999999999972;
KU494212;Coleoptera;Ptilinus pectinicornis;Ptilinus pectinicornis;1;Ptilinus
pectinicornis;0.999999999999972;
KU494213;Coleoptera;Ptilinus pectinicornis;Ptilinus pectinicornis;1;Ptilinus
pectinicornis;0.999999999999972;
KU494214;Coleoptera;Ptilinus pectinicornis;Ptilinus pectinicornis;1;Ptilinus
pectinicornis;0.999999999999972;
KU494215;Coleoptera;Ptilinus pectinicornis;Ptilinus pectinicornis;1;Ptilinus
pectinicornis;0.999999999999972;
KU494216;Coleoptera;Ptilinus pectinicornis;Ptilinus pectinicornis;1;Ptilinus
pectinicornis;0.999999999999972;
KU906136;Coleoptera;Plagionotus arcuatus;Plagionotus arcuatus;1;Plagionotus
arcuatus;0.999999998658836;
KU906175;Coleoptera;Phloeocharis subtilissima;Phloeocharis subtilissima;NA;Phloeocharis
subtilissima;1;
KU906183;Coleoptera;Pteryx suturalis;Pteryx suturalis;1;Pteryx suturalis;1;
KU906211;Coleoptera;Plectophloeus fischeri;Plectophloeus fischeri;1;Plectophloeus fischeri;1;
KU906214;Coleoptera;Valgus hemipterus;Valgus hemipterus;1;Valgus hemipterus;1;
KU906227;Coleoptera;Evodinus clathratus;Evodinus clathratus;1;Evodinus clathratus;1;
KU906258;Coleoptera;Placusa pumilio;Placusa pumilio;1;Placusa pumilio;1;
KU906284;Coleoptera;Anthaxia morio;Anthaxia morio;1;Anthaxia morio;1;
KU906323;Coleoptera;Hypnogyra angularis;Hypnogyra glabra;1;Hypnogyra glabra;1;
KU906331;Coleoptera;Elateroides dermestoides;Elateroides dermestoides;1;Elateroides
dermestoides;1;
KU906334;Coleoptera;Phloeonomus punctipennis;Phloeonomus punctipennis;1;Phloeonomus
punctipennis;1;

KU906389;Coleoptera;Ptilinus pectinicornis;Ptilinus pectinicornis;1;Ptilinus pectinicornis;0.999999999999972;
KU906419;Coleoptera;Phloeocharis subtilissima;Phloeocharis subtilissima;NA;Phloeocharis subtilissima;1;
KU906462;Coleoptera;Anthaxia nitidula;Anthaxia nitidula;1;Anthaxia nitidula;1;
KU906464;Coleoptera;Plagionotus detritus;Plagionotus detritus;1;Plagionotus detritus;0.999999999609798;
KU906475;Coleoptera;Exocentrus adspersus;Exocentrus adspersus;1;Exocentrus adspersus;1;
KU906506;Coleoptera;Atrecus affinis;Atrecus affinis;1;Atrecus affinis;1;
KU906578;Coleoptera;Ptilinus pectinicornis;Ptilinus pectinicornis;1;Ptilinus pectinicornis;1;
KU906641;Coleoptera;Valgus hemipterus;Valgus hemipterus;1;Valgus hemipterus;1;
KU906649;Coleoptera;Tritoma bipustulata;NA;1;Tritoma bipustulata;1;
KU906666;Coleoptera;Eucnemis capucina;Eucnemis capucina;1;Eucnemis capucina;1;
KU906745;Coleoptera;Vincenzellus ruficollis;NA;NA;Vincenzellus ruficollis;1;
KU906755;Coleoptera;Grammoptera abdominalis;Grammoptera abdominalis;1;Grammoptera abdominalis;1;
KU906760;Coleoptera;Pteryx suturalis;NA;NA;Pteryx suturalis;1;
KU906781;Coleoptera;Anthaxia helvetica;Anthaxia helvetica;1;Anthaxia helvetica;1;
KU906787;Coleoptera;Atrecus affinis;NA;NA;Atrecus affinis;1;
KU906796;Coleoptera;Anoplodera sexguttata;Anoplodera sexguttata;1;Anoplodera sexguttata;1;
KU906824;Coleoptera;Anthaxia godeti;Anthaxia godeti;1;Anthaxia godeti;1;
KU906865;Coleoptera;Aulonothroscus brevicollis;Aulonothroscus brevicollis;1;Aulonothroscus brevicollis;1;
KU906880;Coleoptera;Leiopus femoratus;Leiopus femoratus;1;Leiopus femoratus;1;
KU906905;Coleoptera;Atrecus affinis;Atrecus affinis;1;Atrecus affinis;1;
KU906954;Coleoptera;Hemicoelus nitidus;NA;1;Anobium nitidum;1;
KU906979;Coleoptera;Exocentrus adspersus;Exocentrus adspersus;1;Exocentrus adspersus;1;
KU907014;Coleoptera;Berginus tamarisci;NA;1;Berginus tamarisci;1;
KU907020;Coleoptera;Epuraea unicolor;Epuraea unicolor;1;Epuraea unicolor;0.999999999999972;
KU907022;Coleoptera;Platystomos albinus;NA;NA;Platystomos albinus;1;
KU907042;Coleoptera;Xylostiba monilicornis;Xylostiba monilicornis;1;Xylostiba monilicornis;1;
KU907052;Coleoptera;Bitoma crenata;Bitoma crenata;1;Bitoma crenata;1;
KU907068;Coleoptera;Vincenzellus ruficollis;NA;NA;Vincenzellus ruficollis;1;
KU907092;Coleoptera;Leptusa pulchella;Leptusa pulchella;1;Leptusa pulchella;1;
KU907126;Coleoptera;Phloeocharis subtilissima;Phloeocharis subtilissima;NA;Phloeocharis subtilissima;1;
KU907236;Coleoptera;Exocentrus adspersus;Exocentrus adspersus;1;Exocentrus adspersus;1;
KU907242;Coleoptera;Potamophilus acuminatus;Potamophilus acuminatus;NA;Potamophilus acuminatus;1;
KU907243;Coleoptera;Platystomos albinus;NA;NA;Platystomos albinus;1;
KU907248;Coleoptera;Hemicoelus costatus;NA;NA;Anobium fulvicorne;1;
KU907272;Coleoptera;Homophthalmus rugicollis;NA;1;Dryophilus rugicollis;1;
KU907300;Coleoptera;Xyleborinus saxesenii;Xyleborinus saxeseni;1;Xyleborinus saxeseni;1;
KU907301;Coleoptera;Xylostiba bosnica;Xylostiba bosnica;NA;Xylostiba bosnica;1;
KU907308;Coleoptera;Latridius minutus;Latridius minutus;1;Latridius minutus;0.999999999973738;
KU907327;Coleoptera;Hylurgops palliatus;Hylurgops palliatus;1;Hylurgops palliatus;1;
KU907328;Coleoptera;Cis boleti;Cis boleti;1;Cis boleti;1;
KU907366;Coleoptera;Phloeocharis subtilissima;Phloeocharis subtilissima;NA;Phloeocharis subtilissima;1;
KU907372;Coleoptera;Grammoptera ustulata;Grammoptera ustulata;1;Grammoptera ustulata;1;
KU907395;Coleoptera;Platystomos albinus;NA;NA;Platystomos albinus;1;
KU907399;Coleoptera;Batrisus formicarius;Batrisus formicarius;1;Batrisus formicarius;1;
KU907409;Coleoptera;Anthaxia nitidula;Anthaxia nitidula;1;Anthaxia nitidula;1;
KU907431;Coleoptera;Glischrochilus hortensis;Glischrochilus hortensis;1;Glischrochilus hortensis;1;
KU907510;Coleoptera;Tritoma bipustulata;NA;1;Tritoma bipustulata;1;
KU907519;Coleoptera;Anthaxia podolica;Anthaxia podolica;1;Anthaxia podolica;1;
KU907627;Coleoptera;Aulonothroscus brevicollis;Aulonothroscus brevicollis;1;Aulonothroscus brevicollis;1;
KU907671;Coleoptera;Tritoma bipustulata;NA;1;Tritoma bipustulata;1;
KU907722;Coleoptera;Uloma culinaris;Uloma culinaris;1;Uloma culinaris;1;
KU907738;Coleoptera;Cis boleti;Cis boleti;1;Cis boleti;1;
KU907743;Coleoptera;Cis castaneus;Cis castaneus;1;Cis castaneus;1;
KU907805;Coleoptera;Epuraea terminalis;Epuraea terminalis;NA;Epuraea placida;0.999929985441599;
KU907833;Coleoptera;Bibloporus bicolor;Bibloporus bicolor;1;Bibloporus bicolor;0.999999999999829;
KU907856;Coleoptera;Axinopalpis gracilis;NA;NA;Axinopalpis gracilis;1;
KU907873;Coleoptera;Latridius minutus;Latridius minutus;1;Latridius minutus;0.999999999993065;
KU907927;Coleoptera;Hemicoelus costatus;NA;NA;Anobium fulvicorne;1;
KU907988;Coleoptera;Bibloporus ultimus;Bibloporus ultimus;1;Bibloporus ultimus;0.999999999999943;
KU908000;Coleoptera;Anthaxia helvetica;Anthaxia helvetica;1;Anthaxia helvetica;1;
KU908003;Coleoptera;Glischrochilus quadripunctatus;Glischrochilus quadripunctatus;1;Glischrochilus quadripunctatus;0.999999942615718;
KU908007;Coleoptera;Uloma culinaris;Uloma culinaris;1;Uloma culinaris;1;
KU908045;Coleoptera;Latridius minutus;Latridius minutus;1;Latridius minutus;0.999999999973738;
KU908051;Coleoptera;Vincenzellus ruficollis;NA;NA;Vincenzellus ruficollis;1;
KU908093;Coleoptera;Cis hispidus;NA;0.317;Cis hispidus;0.99999991378775;

KU908097;Coleoptera;Glischrochilus hortensis;Glischrochilus hortensis;1;Glischrochilus hortensis;1;
KU908112;Coleoptera;Potamophilus acuminatus;Potamophilus acuminatus;NA;Potamophilus acuminatus;1;
KU908142;Coleoptera;Polygraphus poligraphus;Polygraphus poligraphus;1;Polygraphus poligraphus;0.999999999993719;
KU908183;Coleoptera;Anthaxia nitidula;Anthaxia nitidula;1;Anthaxia nitidula;1;
KU908206;Coleoptera;Cis comptus;Cis comptus;1;Cis comptus;1;
KU908216;Coleoptera;Phloeocharis subtilissima;Phloeocharis subtilissima;NA;Phloeocharis subtilissima;1;
KU908225;Coleoptera;Tritoma bipustulata;NA;1;Tritoma bipustulata;1;
KU908291;Coleoptera;Tomicus piniperda;NA;NA;Tomicus piniperda;1;
KU908299;Coleoptera;Glischrochilus quadriguttatus;Glischrochilus quadriguttatus;1;Glischrochilus quadriguttatus;1;
KU908368;Coleoptera;Elateroides dermestoides;NA;1;Elateroides dermestoides;1;
KU908381;Coleoptera;Hylastes ater;Hylastes brunneus;1;Hylastes brunneus;0.999999999999972;
KU908395;Coleoptera;Tritoma bipustulata;NA;1;Tritoma bipustulata;1;
KU908450;Coleoptera;Tritoma bipustulata;NA;1;Tritoma bipustulata;1;
KU908459;Coleoptera;Leiopus femoratus;Leiopus femoratus;1;Leiopus femoratus;1;
KU908469;Coleoptera;Tomicus piniperda;NA;NA;Tomicus piniperda;1;
KU908476;Coleoptera;Valgus hemipterus;Valgus hemipterus;1;Valgus hemipterus;1;
KU908483;Coleoptera;Glischrochilus quadriguttatus;Glischrochilus quadriguttatus;1;Glischrochilus quadriguttatus;1;
KU908550;Coleoptera;Hymenalia rufipes;Hymenalia rufipes;NA;Hymenalia rufipes;1;
KU908574;Coleoptera;Trixagus atticus;Trixagus atticus;1;Trixagus atticus;1;
KU908600;Coleoptera;Epuraea variegata;Epuraea variegata;1;Epuraea variegata;0.999999999840242;
KU908672;Coleoptera;Epuraea variegata;Epuraea variegata;1;Epuraea variegata;0.999999999911438;
KU908691;Coleoptera;Evodinus clathratus;Evodinus clathratus;1;Evodinus clathratus;1;
KU908700;Coleoptera;Bibloporus bicolor;Bibloporus bicolor;1;Bibloporus bicolor;0.999999999999858;
KU908709;Coleoptera;Grammoptera ruficornis;Grammoptera ruficornis;1;Grammoptera ruficornis;1;
KU908781;Coleoptera;Elateroides dermestoides;NA;1;Elateroides dermestoides;1;
KU908858;Coleoptera;Bibloporus bicolor;Bibloporus bicolor;1;Bibloporus bicolor;0.999999999999915;
KU908860;Coleoptera;Polygraphus poligraphus;Polygraphus poligraphus;1;Polygraphus poligraphus;0.999999999985704;
KU908874;Coleoptera;Glischrochilus quadriguttatus;Glischrochilus quadriguttatus;1;Glischrochilus quadriguttatus;1;
KU908958;Coleoptera;Tillus elongatus;NA;NA;Tillus elongatus;1;
KU908962;Coleoptera;Anisotoma humeralis;Anisotoma humeralis;1;Anisotoma humeralis;0.999999999910727;
KU908973;Coleoptera;Euplectus nanus;NA;0.319;NA;0.999999999999995;
KU908976;Coleoptera;Glischrochilus hortensis;Glischrochilus hortensis;1;Glischrochilus hortensis;1;
KU908979;Coleoptera;Tillus elongatus;NA;NA;Tillus elongatus;1;
KU909099;Coleoptera;Anthaxia candens;Anthaxia candens;1;Anthaxia candens;1;
KU909150;Coleoptera;Exocentrus adspersus;Exocentrus adspersus;1;Exocentrus adspersus;1;
KU909239;Coleoptera;Grammoptera abdominalis;Grammoptera abdominalis;1;Grammoptera abdominalis;1;
KU909242;Coleoptera;Phloeonomus minimus;Phloeonomus minimus;NA;Phloeonomus minimus;1;
KU909245;Coleoptera;Platystomos albinus;NA;1;Platystomos albinus;1;
KU909274;Coleoptera;Grammoptera abdominalis;Grammoptera abdominalis;1;Grammoptera abdominalis;1;
KU909292;Coleoptera;Platystomos albinus;NA;1;Platystomos albinus;1;
KU909295;Coleoptera;Anthaxia manca;Anthaxia manca;1;Anthaxia manca;1;
KU909316;Coleoptera;Agrilus viridis;Agrilus viridis;1;Agrilus viridis;0.99999967150313;
KU909320;Coleoptera;Dendrophilus punctatus;NA;1;Dendrophilus punctatus;1;
KU909323;Coleoptera;Hemicoelus nitidus;NA;NA;Anobium nitidum;1;
KU909348;Coleoptera;Ptilinus pectinicornis;Ptilinus pectinicornis;1;Ptilinus pectinicornis;1;
KU909370;Coleoptera;Anthaxia quadripunctata;Anthaxia quadripunctata;1;Anthaxia quadripunctata;1;
KU909390;Coleoptera;Ptilinus pectinicornis;Ptilinus pectinicornis;1;Ptilinus pectinicornis;1;
KU909427;Coleoptera;Troglops albicans;Troglops albicans;1;Troglops albicans;1;
KU909452;Coleoptera;Placusa pumilio;Placusa pumilio;1;Placusa pumilio;1;
KU909453;Coleoptera;Grammoptera ruficornis;Grammoptera ruficornis;1;Grammoptera ruficornis;1;
KU909456;Coleoptera;Phloeonomus punctipennis;Phloeonomus punctipennis;1;Phloeonomus punctipennis;1;
KU909486;Coleoptera;Placusa tachyporoides;Placusa tachyporoides;1;Placusa tachyporoides;0.999999995754933;
KU909492;Coleoptera;Exocentrus adspersus;Exocentrus adspersus;1;Exocentrus adspersus;1;
KU909509;Coleoptera;Phloeonomus punctipennis;Phloeonomus punctipennis;1;Phloeonomus punctipennis;1;
KU909537;Coleoptera;Uloma culinaris;Uloma culinaris;1;Uloma culinaris;1;
KU909582;Coleoptera;Anoplodera sexguttata;Anoplodera sexguttata;1;Anoplodera sexguttata;1;
KU909583;Coleoptera;Hypnogyra angularis;Hypnogyra glabra;1;Hypnogyra glabra;1;
KU909617;Coleoptera;Atrecus affinis;NA;NA;Atrecus affinis;1;
KU909673;Coleoptera;Anthaxia nitidula;Anthaxia nitidula;1;Anthaxia nitidula;1;
KU909726;Coleoptera;Troglops albicans;Troglops albicans;1;Troglops albicans;1;
KU909730;Coleoptera;Epuraea unicolor;Epuraea unicolor;1;Epuraea unicolor;0.999999999999972;
KU909737;Coleoptera;Glischrochilus quadripunctatus;Glischrochilus quadripunctatus;0.001;Glischrochilus quadripunctatus;0.9999999497214;

KU909760;Coleoptera;Tillus elongatus;NA;NA;Tillus elongatus;1;
KU909832;Coleoptera;Cis festivus;Orthocis festivus;1;Orthocis festivus;1;
KU909847;Coleoptera;Euryptilium saxonicum;Euryptilium saxonicum;1;Euryptilium saxonicum;1;
KU909849;Coleoptera;Platycerus caraboides;Platycerus caraboides;1;Platycerus caraboides;1;
KU909903;Coleoptera;Cis comptus;Cis comptus;1;Cis comptus;1;
KU909964;Coleoptera;Leptusa fumida;Leptusa fumida;1;Leptusa fumida;0.999999999999972;
KU910110;Coleoptera;Tomicus piniperda;NA;NA;Tomicus piniperda;1;
KU910125;Coleoptera;Latridius minutus;Latridius minutus;1;Latridius minutus;0.999999999973738;
KU910143;Coleoptera;Grammoptera abdominalis;Grammoptera abdominalis;1;Grammoptera abdominalis;1;
KU910200;Coleoptera;Anthaxia quadripunctata;Anthaxia quadripunctata;1;Anthaxia quadripunctata;1;
KU910207;Coleoptera;Anthaxia nitidula;Anthaxia nitidula;1;Anthaxia nitidula;1;
KU910250;Coleoptera;Aulonothroscus brevicollis;Aulonothroscus brevicollis;1;Aulonothroscus brevicollis;1;
KU910276;Coleoptera;Hymenalia rufipes;Hymenalia rufipes;NA;Hymenalia rufipes;1;
KU910307;Coleoptera;Aulonium trisulcum;Aulonium trisulcum;1;Aulonium trisulcum;1;
KU910422;Coleoptera;Hypnogyra angularis;Hypnogyra glabra;NA;Hypnogyra glabra;1;
KU910424;Coleoptera;Bibloporus ultimus;Bibloporus ultimus;1;Bibloporus ultimus;0.999999999999943;
KU910429;Coleoptera;Dendrophilus punctatus;NA;1;Dendrophilus punctatus;1;
KU910436;Coleoptera;Hylurgops palliatus;Hylurgops palliatus;1;Hylurgops palliatus;1;
KU910464;Coleoptera;Atrecus affinis;NA;NA;Atrecus affinis;1;
KU910472;Coleoptera;Dendrophilus punctatus;Dendrophilus punctatus;1;Dendrophilus punctatus;1;
KU910474;Coleoptera;Euryptilium saxonicum;Euryptilium saxonicum;1;Euryptilium saxonicum;1;
KU910477;Coleoptera;Vincenzellus ruficollis;Vincenzellus ruficollis;1;Vincenzellus ruficollis;1;
KU910509;Coleoptera;Xyleborus dryographus;Xyleborus dryographus;1;Xyleborus dryographus;0.999999999999602;
KU910541;Coleoptera;Anisotoma humeralis;Anisotoma humeralis;1;Anisotoma humeralis;0.999999999966832;
KU910553;Coleoptera;Exocentrus adspersus;NA;NA;Exocentrus adspersus;1;
KU910607;Coleoptera;Tritoma bipustulata;NA;1;Tritoma bipustulata;1;
KU910612;Coleoptera;Gnathotrichus materiarius;Gnathotrichus materiarius;1;Gnathotrichus materiarius;1;
KU910664;Coleoptera;Pteryx suturalis;Pteryx suturalis;NA;Pteryx suturalis;1;
KU910678;Coleoptera;Leiopus nebulosus;NA;0.12;Leiopus linnei;0.911840242395976;
KU910701;Coleoptera;Uloma culinaris;Uloma culinaris;1;Uloma culinaris;1;
KU910711;Coleoptera;Homalota plana;NA;NA;Homalota plana;1;
KU910753;Coleoptera;Dendrophilus pygmaeus;NA;1;Dendrophilus pygmaeus;1;
KU910786;Coleoptera;Anthaxia mendizabali;Anthaxia mendizabali;1;Anthaxia mendizabali;1;
KU910789;Coleoptera;Epuraea unicolor;Epuraea unicolor;1;Epuraea unicolor;0.999999999999972;
KU910795;Coleoptera;Aulonium trisulcum;Aulonium trisulcum;1;Aulonium trisulcum;1;
KU910814;Coleoptera;Troglops albicans;Troglops albicans;NA;Troglops albicans;1;
KU910829;Coleoptera;Hololepta plana;Hololepta plana;1;Hololepta plana;1;
KU910839;Coleoptera;Exocentrus lusitanus;Exocentrus lusitanus;1;Exocentrus lusitanus;1;
KU910885;Coleoptera;Agrilus viridis;Agrilus viridis;1;Agrilus viridis;0.999999232154419;
KU910908;Coleoptera;Cis boleti;Cis boleti;1;Cis boleti;1;
KU910934;Coleoptera;Triplax lepida;Triplax lepida;1;Triplax lepida;1;
KU910935;Coleoptera;Aulonothroscus brevicollis;Aulonothroscus brevicollis;1;Aulonothroscus brevicollis;1;
KU910943;Coleoptera;Phloeonomus punctipennis;Phloeonomus punctipennis;1;Phloeonomus punctipennis;1;
KU910945;Coleoptera;Euplectus karstenii;Euplectus karstenii;1;Euplectus karstenii;1;
KU910970;Coleoptera;Tritoma bipustulata;NA;1;Tritoma bipustulata;1;
KU910997;Coleoptera;Vincenzellus ruficollis;NA;NA;Vincenzellus ruficollis;1;
KU911006;Coleoptera;Exocentrus adspersus;NA;NA;Exocentrus adspersus;1;
KU911088;Coleoptera;Hylastes ater;Hylastes ater;1;Hylastes ater;0.999999999999858;
KU911107;Coleoptera;Glischrochilus quadriguttatus;Glischrochilus quadriguttatus;1;Glischrochilus quadriguttatus;1;
KU911123;Coleoptera;Hypnogyra angularis;Hypnogyra glabra;1;Hypnogyra glabra;1;
KU911226;Coleoptera;Xyleborinus saxesenii;NA;0.152;Xyleborinus saxesenii;0.999993637230154;
KU911306;Coleoptera;Vincenzellus ruficollis;Vincenzellus ruficollis;1;Vincenzellus ruficollis;1;
KU911312;Coleoptera;Grammoptera ruficornis;NA;NA;Grammoptera ruficornis;1;
KU911346;Coleoptera;Exocentrus adspersus;Exocentrus adspersus;1;Exocentrus adspersus;1;
KU911347;Coleoptera;Hololepta plana;Hololepta plana;1;Hololepta plana;1;
KU911348;Coleoptera;Glischrochilus hortensis;Glischrochilus hortensis;1;Glischrochilus hortensis;1;
KU911359;Coleoptera;Exocentrus adspersus;Exocentrus adspersus;1;Exocentrus adspersus;1;
KU911366;Coleoptera;Glischrochilus quadriguttatus;Glischrochilus quadriguttatus;1;Glischrochilus quadriguttatus;1;
KU911430;Coleoptera;Anthaxia quadripunctata;Anthaxia quadripunctata;1;Anthaxia quadripunctata;1;
KU911567;Coleoptera;Trixagus leseigneuri;Trixagus leseigneuri;1;Trixagus leseigneuri;1;
KU911568;Coleoptera;Trypodendron domesticum;Trypodendron domesticum;1;Trypodendron domesticum;1;
KU911660;Coleoptera;Grammoptera ruficornis;NA;NA;Grammoptera ruficornis;1;
KU911724;Coleoptera;Dendrophilus pygmaeus;NA;1;Dendrophilus pygmaeus;1;
KU911808;Coleoptera;Grammoptera ustulata;Grammoptera ustulata;1;Grammoptera ustulata;1;
KU911821;Coleoptera;Anthaxia nitidula;Anthaxia nitidula;1;Anthaxia nitidula;1;
KU911936;Coleoptera;Anisotoma humeralis;Anisotoma humeralis;1;Anisotoma humeralis;0.999999999871534;

KU911943;Coleoptera;Anisotoma humeralis;Anisotoma humeralis;1;Anisotoma humeralis;0.999999999966832;
KU911949;Coleoptera;Anthaxia nitidula;Anthaxia nitidula;1;Anthaxia nitidula;1;
KU911952;Coleoptera;Ernobius abietis;NA;1;Ernobius abietis;1;
KU911988;Coleoptera;Ptilinus pectinicornis;Ptilinus pectinicornis;1;Ptilinus pectinicornis;1;
KU912007;Coleoptera;Xylostiba bosnica;Xylostiba bosnica;NA;Xylostiba bosnica;1;
KU912021;Coleoptera;Epuraea unicolor;Epuraea unicolor;1;Epuraea unicolor;1;
KU912046;Coleoptera;Euryusa castanoptera;Euryusa castanoptera;1;Euryusa castanoptera;1;
KU912067;Coleoptera;Xyleborinus saxesenii;Xyleborinus saxeseni;1;Xyleborinus saxeseni;1;
KU912074;Coleoptera;Euplectus nanus;NA;0.319;NA;0.999999999999995;
KU912122;Coleoptera;Platycerus caraboides;Platycerus caraboides;1;Platycerus caraboides;1;
KU912168;Coleoptera;Euplectus nanus;NA;0.319;NA;0.999999999999995;
KU912180;Coleoptera;Hymenalia rufipes;Hymenalia rufipes;NA;Hymenalia rufipes;1;
KU912183;Coleoptera;Cis boleti;Cis boleti;1;Cis boleti;1;
KU912192;Coleoptera;Vincenzellus ruficollis;NA;NA;Vincenzellus ruficollis;1;
KU912220;Coleoptera;Exocentrus adspersus;Exocentrus adspersus;1;Exocentrus adspersus;1;
KU912222;Coleoptera;Hololepta plana;Hololepta plana;1;Hololepta plana;1;
KU912345;Coleoptera;Pteryx suturalis;Pteryx suturalis;1;Pteryx suturalis;1;
KU912414;Coleoptera;Cis boleti;Cis boleti;1;Cis boleti;1;
KU912452;Coleoptera;Xyleborinus saxesenii;NA;0.152;Xyleborinus saxesenii;0.999993637230154;
KU912478;Coleoptera;Anthaxia nitidula;Anthaxia nitidula;1;Anthaxia nitidula;1;
KU912481;Coleoptera;Anisotoma orbicularis;Anisotoma orbicularis;1;Anisotoma orbicularis;1;
KU912524;Coleoptera;Axinopalpis gracilis;NA;NA;Axinopalpis gracilis;1;
KU912535;Coleoptera;Valgus hemipterus;Valgus hemipterus;NA;Valgus hemipterus;1;
KU912555;Coleoptera;Ptilinus fuscus;Ptilinus fuscus;1;Ptilinus fuscus;1;
KU912603;Coleoptera;Placusa tachyporoides;Placusa tachyporoides;1;Placusa tachyporoides;0.999999995754933;
KU912609;Coleoptera;Glischrochilus hortensis;Glischrochilus hortensis;1;Glischrochilus hortensis;1;
KU912613;Coleoptera;Euryusa optabilis;Euryusa optabilis;1;Euryusa optabilis;0.99999999997533;
KU912632;Coleoptera;Hemicoelus nitidus;Anobium nitidum;1;Anobium nitidum;1;
KU912650;Coleoptera;Glischrochilus quadripunctatus;Glischrochilus quadripunctatus;0.002;Glischrochilus quadripunctatus;0.99999985966475;
KU912724;Coleoptera;Trixagus carinifrons;Trixagus carinifrons;1;Trixagus carinifrons;1;
KU912741;Coleoptera;Glischrochilus quadripunctatus;Glischrochilus quadripunctatus;0.002;Glischrochilus quadripunctatus;0.999999938086889;
KU912750;Coleoptera;Grammoptera ruficornis;NA;NA;Grammoptera ruficornis;1;
KU912818;Coleoptera;Gnathotrichus materiarius;Gnathotrichus materiarius;1;Gnathotrichus materiarius;1;
KU912840;Coleoptera;Plegaderus caesus;Plegaderus caesus;1;Plegaderus caesus;1;
KU912848;Coleoptera;Dendrophilus punctatus;NA;1;Dendrophilus punctatus;1;
KU912872;Coleoptera;Phloeonomus punctipennis;Phloeonomus punctipennis;NA;Phloeonomus punctipennis;1;
KU912907;Coleoptera;Grammoptera ustulata;Grammoptera ustulata;1;Grammoptera ustulata;1;
KU912931;Coleoptera;Elateroides dermestoides;NA;1;Elateroides dermestoides;1;
KU912942;Coleoptera;Potamophilus acuminatus;Potamophilus acuminatus;NA;Potamophilus acuminatus;1;
KU912968;Coleoptera;Anthaxia quadripunctata;Anthaxia quadripunctata;1;Anthaxia quadripunctata;1;
KU912981;Coleoptera;Glischrochilus quadriguttatus;Glischrochilus quadriguttatus;1;Glischrochilus quadriguttatus;1;
KU912989;Coleoptera;Polygraphus grandiclava;Polygraphus grandiclava;1;Polygraphus grandiclava;0.999999997098428;
KU913002;Coleoptera;Platystomos albinus;NA;1;Platystomos albinus;1;
KU913055;Coleoptera;Anthaxia nitidula;Anthaxia nitidula;1;Anthaxia nitidula;1;
KU913075;Coleoptera;Hypoganus inunctus;Hypoganus inunctus;1;Hypoganus inunctus;1;
KU913083;Coleoptera;Pteryngium crenatum;Pteryngium crenatum;1;Pteryngium crenatum;1;
KU913104;Coleoptera;Atrecus affinis;NA;NA;Atrecus affinis;1;
KU913178;Coleoptera;Placusa tachyporoides;Placusa tachyporoides;1;Placusa tachyporoides;0.999999995754933;
KU913232;Coleoptera;Elateroides dermestoides;NA;1;Elateroides dermestoides;1;
KU913264;Coleoptera;Grammoptera ruficornis;Grammoptera ruficornis;NA;Grammoptera ruficornis;0.999999999999545;
KU913311;Coleoptera;Tritoma bipustulata;NA;1;Tritoma bipustulata;1;
KU913323;Coleoptera;Triplax russica;Triplax russica;1;Triplax russica;1;
KU913459;Coleoptera;Denticollis linearis;Denticollis linearis;1;Denticollis linearis;1;
KU913483;Coleoptera;Trixagus atticus;Trixagus atticus;1;Trixagus atticus;1;
KU913495;Coleoptera;Exocentrus adspersus;Exocentrus adspersus;1;Exocentrus adspersus;1;
KU913515;Coleoptera;Potamophilus acuminatus;Potamophilus acuminatus;NA;Potamophilus acuminatus;1;
KU913604;Coleoptera;Glischrochilus quadripunctatus;Glischrochilus quadripunctatus;1;Glischrochilus quadripunctatus;0.953174474849839;
KU913803;Coleoptera;Xestobium rufovillosum;NA;1;Xestobium rufovillosum;1;
KU913831;Coleoptera;Cis festivus;NA;NA;Orthocis festivus;1;
KU913834;Coleoptera;Phloeonomus punctipennis;Phloeonomus punctipennis;1;Phloeonomus punctipennis;1;
KU913839;Coleoptera;Plegaderus caesus;Plegaderus caesus;1;Plegaderus caesus;1;
KU913850;Coleoptera;Euplectus nanus;NA;0.319;NA;0.999999999999995;

KU913875;Coleoptera;Phloeocharis subtilissima;Phloeocharis subtilissima;1;Phloeocharis subtilissima;1;
KU913892;Coleoptera;Phloeonomus punctipennis;Phloeonomus punctipennis;NA;Phloeonomus punctipennis;1;
KU913934;Coleoptera;Anisotoma castanea;Anisotoma castanea;1;Anisotoma castanea;0.999999999997783;
KU913941;Coleoptera;Polygraphus poligraphus;Polygraphus poligraphus;1;Polygraphus poligraphus;0.999999999996163;
KU913956;Coleoptera;Dendrophilus punctatus;NA;1;Dendrophilus punctatus;1;
KU913962;Coleoptera;Xylostiba monilicornis;Xylostiba monilicornis;NA;Xylostiba monilicornis;1;
KU913977;Coleoptera;Denticollis linearis;Denticollis linearis;1;Denticollis linearis;1;
KU913994;Coleoptera;Cis glabratus;Cis glabratus;1;Cis glabratus;1;
KU914039;Coleoptera;Epuraea unicolor;Epuraea unicolor;1;Epuraea unicolor;0.999999999999972;
KU914053;Coleoptera;Pteryx suturalis;Pteryx suturalis;1;Pteryx suturalis;1;
KU914083;Coleoptera;Plectophloeus nubigena;Plectophloeus nubigena;1;Plectophloeus nubigena;1;
KU914088;Coleoptera;Anthaxia helvetica;Anthaxia helvetica;1;Anthaxia helvetica;1;
KU914123;Coleoptera;Pteryx suturalis;Pteryx suturalis;1;Pteryx suturalis;1;
KU914127;Coleoptera;Vincenzellus ruficollis;NA;NA;Vincenzellus ruficollis;1;
KU914161;Coleoptera;Exocentrus lusitanus;Exocentrus lusitanus;1;Exocentrus lusitanus;1;
KU914173;Coleoptera;Anthaxia hungarica;Anthaxia hungarica;1;Anthaxia hungarica;1;
KU914189;Coleoptera;Vincenzellus ruficollis;NA;NA;Vincenzellus ruficollis;1;
KU914191;Coleoptera;Leptusa fumida;Leptusa fumida;1;Leptusa fumida;1;
KU914227;Coleoptera;Grammoptera ruficornis;Grammoptera ruficornis;1;Grammoptera ruficornis;1;
KU914233;Coleoptera;Uloma culinaris;Uloma culinaris;1;Uloma culinaris;1;
KU914257;Coleoptera;Cis festivus;Orthocis festivus;1;Orthocis festivus;1;
KU914357;Coleoptera;Euplectus karstenii;Euplectus karstenii;1;Euplectus karstenii;1;
KU914363;Coleoptera;Valgus hemipterus;Valgus hemipterus;NA;Valgus hemipterus;1;
KU914405;Coleoptera;Triplax russica;Triplax russica;1;Triplax russica;1;
KU914485;Coleoptera;Tritoma bipustulata;NA;1;Tritoma bipustulata;1;
KU914569;Coleoptera;Vincenzellus ruficollis;NA;NA;Vincenzellus ruficollis;1;
KU914572;Coleoptera;Elateroides dermestoides;Elateroides dermestoides;1;Elateroides dermestoides;1;
KU914590;Coleoptera;Triplax russica;Triplax russica;1;Triplax russica;1;
KU914597;Coleoptera;Hylastes ater;Hylastes ater;1;Hylastes ater;0.999999999999687;
KU914631;Coleoptera;Polygraphus poligraphus;Polygraphus poligraphus;1;Polygraphus poligraphus;0.999999999995225;
KU914636;Coleoptera;Anthaxia nitidula;Anthaxia nitidula;1;Anthaxia nitidula;1;
KU914686;Coleoptera;Cis hispidus;NA;NA;Cis hispidus;0.999999952719493;
KU914687;Coleoptera;Hypoganus inunctus;Hypoganus inunctus;1;Hypoganus inunctus;1;
KU914714;Coleoptera;Aulonium trisulcum;NA;NA;Aulonium trisulcum;1;
KU914729;Coleoptera;Cis boleti;Cis boleti;1;Cis boleti;1;
KU914735;Coleoptera;Anthaxia helvetica;Anthaxia helvetica;1;Anthaxia helvetica;1;
KU914744;Coleoptera;Cis comptus;Cis comptus;1;Cis comptus;1;
KU914779;Coleoptera;Aulonothroscus brevicollis;Aulonothroscus brevicollis;1;Aulonothroscus brevicollis;1;
KU914837;Coleoptera;Plagionotus arcuatus;Plagionotus arcuatus;1;Plagionotus arcuatus;0.999999998658836;
KU914845;Coleoptera;Anthaxia morio;Anthaxia morio;1;Anthaxia morio;1;
KU914909;Coleoptera;Tritoma bipustulata;NA;1;Tritoma bipustulata;1;
KU914957;Coleoptera;Atrecus affinis;NA;NA;Atrecus affinis;1;
KU915009;Coleoptera;Trypodendron domesticum;Trypodendron domesticum;1;Trypodendron domesticum;1;
KU915028;Coleoptera;Vincenzellus ruficollis;NA;NA;Vincenzellus ruficollis;1;
KU915066;Coleoptera;Dendrophilus punctatus;NA;1;Dendrophilus punctatus;1;
KU915087;Coleoptera;Glischrochilus hortensis;Glischrochilus hortensis;1;Glischrochilus hortensis;1;
KU915090;Coleoptera;Anisotoma humeralis;Anisotoma humeralis;1;Anisotoma humeralis;0.999999999373927;
KU915094;Coleoptera;Vincenzellus ruficollis;NA;NA;Vincenzellus ruficollis;1;
KU915106;Coleoptera;Phloeonomus minimus;Phloeonomus minimus;1;Phloeonomus minimus;1;
KU915167;Coleoptera;Grammoptera ustulata;Grammoptera ustulata;1;Grammoptera ustulata;1;
KU915174;Coleoptera;Euplectus nanus;NA;0.319;NA;0.999999999999995;
KU915192;Coleoptera;Agrilus viridis;Agrilus viridis;1;Agrilus viridis;0.999999824276231;
KU915193;Coleoptera;Agrilus viridis;Agrilus viridis;1;Agrilus viridis;0.999999262134497;
KU915258;Coleoptera;Cis glabratus;Cis glabratus;1;Cis glabratus;1;
KU915262;Coleoptera;Triplax lepida;Triplax lepida;1;Triplax lepida;1;
KU915263;Coleoptera;Glischrochilus hortensis;Glischrochilus hortensis;1;Glischrochilus hortensis;1;
KU915264;Coleoptera;Anthaxia nitidula;Anthaxia nitidula;1;Anthaxia nitidula;1;
KU915290;Coleoptera;Leiopus nebulosus;NA;0.12;Leiopus linnei;0.911840242395976;
KU915302;Coleoptera;Anthaxia candens;Anthaxia candens;1;Anthaxia candens;1;
KU915334;Coleoptera;Euplectus nanus;NA;0.045;Euplectus nanus;0.819242315019459;
KU915338;Coleoptera;Hypulus quercinus;Hypulus quercinus;1;Hypulus quercinus;1;
KU915392;Coleoptera;Ptilinus pectinicornis;Ptilinus pectinicornis;1;Ptilinus pectinicornis;1;
KU915405;Coleoptera;Grammoptera ruficornis;NA;NA;Grammoptera ruficornis;1;
KU915462;Coleoptera;Phloeonomus punctipennis;Phloeonomus punctipennis;1;Phloeonomus punctipennis;1;
KU915464;Coleoptera;Anisotoma orbicularis;Anisotoma orbicularis;1;Anisotoma orbicularis;1;

KU915487;Coleoptera;Ptilinus pectinicornis;Ptilinus pectinicornis;1;Ptilinus pectinicornis;0.999999999999972;
KU915511;Coleoptera;Troglops albicans;Troglops albicans;NA;Troglops albicans;1;
KU915512;Coleoptera;Hypebaeus flavipes;Hypebaeus flavipes;1;Hypebaeus flavipes;1;
KU915537;Coleoptera;Ernobius abietis;Ernobius abietis;1;Ernobius abietis;1;
KU915557;Coleoptera;Xylotrechus arvicola;Xylotrechus arvicola;1;Xylotrechus arvicola;0.999999994925048;
KU915564;Coleoptera;Hymenalia rufipes;Hymenalia rufipes;1;Hymenalia rufipes;1;
KU915577;Coleoptera;Phloeocharis subtilissima;Phloeocharis subtilissima;NA;Phloeocharis subtilissima;1;
KU915586;Coleoptera;Grammoptera ruficornis;Grammoptera ruficornis;1;Grammoptera ruficornis;1;
KU915590;Coleoptera;Pteryx suturalis;Pteryx suturalis;1;Pteryx suturalis;1;
KU915597;Coleoptera;Grammoptera ruficornis;Grammoptera ruficornis;1;Grammoptera ruficornis;1;
KU915600;Coleoptera;Anthaxia helvetica;Anthaxia helvetica;1;Anthaxia helvetica;1;
KU915607;Coleoptera;Exocentrus adspersus;Exocentrus adspersus;1;Exocentrus adspersus;1;
KU915631;Coleoptera;Grammoptera ruficornis;Grammoptera ruficornis;1;Grammoptera ruficornis;1;
KU915676;Coleoptera;Euryusa optabilis;Euryusa optabilis;1;Euryusa optabilis;0.999999999320892;
KU915695;Coleoptera;Hypnogyra angularis;Hypnogyra glabra;NA;Hypnogyra glabra;1;
KU915703;Coleoptera;Triplax lepida;Triplax lepida;1;Triplax lepida;1;
KU915724;Coleoptera;Pteryx suturalis;NA;NA;Pteryx suturalis;1;
KU915794;Coleoptera;Euplectus nanus;NA;0.319;NA;0.999999999999995;
KU915796;Coleoptera;Anthaxia quadripunctata;Anthaxia quadripunctata;1;Anthaxia quadripunctata;1;
KU915799;Coleoptera;Elateroides dermestoides;Elateroides dermestoides;1;Elateroides dermestoides;1;
KU915837;Coleoptera;Plectophloeus nubigena;Plectophloeus nubigena;1;Plectophloeus nubigena;1;
KU915859;Coleoptera;Tillus elongatus;Tillus elongatus;1;Tillus elongatus;1;
KU915923;Coleoptera;Euplectus nanus;NA;0.323;NA;100.000.000.000.001;
KU915979;Coleoptera;Bibloporus bicolor;Bibloporus bicolor;1;Bibloporus bicolor;0.999999999999829;
KU915995;Coleoptera;Pteryx suturalis;NA;NA;Pteryx suturalis;1;
KU916048;Coleoptera;Tomicus piniperda;Tomicus piniperda;NA;Tomicus piniperda;1;
KU916115;Coleoptera;Evodinus clathratus;Evodinus clathratus;1;Evodinus clathratus;1;
KU916116;Coleoptera;Hypebaeus flavipes;Hypebaeus flavipes;1;Hypebaeus flavipes;1;
KU916137;Coleoptera;Anthaxia manca;Anthaxia manca;1;Anthaxia manca;1;
KU916139;Coleoptera;Epuraea unicolor;Epuraea unicolor;1;Epuraea unicolor;0.999999999999972;
KU916158;Coleoptera;Pteryngium crenatum;Pteryngium crenatum;1;Pteryngium crenatum;1;
KU916250;Coleoptera;Anisoxya fuscula;Anisoxya fuscula;1;Anisoxya fuscula;0.999999999445578;
KU916299;Coleoptera;Xyleborinus saxesenii;Xyleborinus saxeseni;1;Xyleborinus saxeseni;1;
KU916300;Coleoptera;Hymenalia rufipes;Hymenalia rufipes;NA;Hymenalia rufipes;1;
KU916352;Coleoptera;Triplax rufipes;Triplax rufipes;1;Triplax rufipes;1;
KU916362;Coleoptera;Vincenzellus ruficollis;NA;NA;Vincenzellus ruficollis;1;
KU916393;Coleoptera;Denticollis linearis;Denticollis linearis;1;Denticollis linearis;1;
KU916410;Coleoptera;Hemicoelus costatus;NA;NA;Anobium fulvicorne;1;
KU916466;Coleoptera;Anisotoma humeralis;Anisotoma humeralis;1;Anisotoma humeralis;0.999999999966832;
KU916489;Coleoptera;Vincenzellus ruficollis;NA;NA;Vincenzellus ruficollis;1;
KU916498;Coleoptera;Euplectus karstenii;Euplectus karstenii;1;Euplectus karstenii;1;
KU916501;Coleoptera;Bitoma crenata;Bitoma crenata;1;Bitoma crenata;1;
KU916515;Coleoptera;Elateroides dermestoides;NA;1;Elateroides dermestoides;1;
KU916540;Coleoptera;Vincenzellus ruficollis;NA;NA;Vincenzellus ruficollis;1;
KU916626;Coleoptera;Hemicoelus nitidus;Anobium nitidum;NA;Anobium nitidum;1;
KU916651;Coleoptera;Priobium carpini;Priobium carpini;1;Priobium carpini;1;
KU916657;Coleoptera;Homophthalmus rugicollis;Dryophilus rugicollis;1;Dryophilus rugicollis;1;
KU916659;Coleoptera;Uloma culinaris;Uloma culinaris;1;Uloma culinaris;1;
KU916692;Coleoptera;Cis hispidus;NA;0.317;Cis hispidus;0.99999991378775;
KU916713;Coleoptera;Denticollis linearis;Denticollis linearis;1;Denticollis linearis;1;
KU916734;Coleoptera;Cis hispidus;NA;0.317;Cis hispidus;0.99999991378775;
KU916743;Coleoptera;Glischrochilus quadriguttatus;Glischrochilus quadriguttatus;1;Glischrochilus quadriguttatus;1;
KU916752;Coleoptera;Anisotoma orbicularis;Anisotoma orbicularis;1;Anisotoma orbicularis;1;
KU916795;Coleoptera;Bibloporus bicolor;Bibloporus bicolor;1;Bibloporus bicolor;0.999999999999943;
KU916854;Coleoptera;Exocentrus lusitanus;Exocentrus lusitanus;1;Exocentrus lusitanus;1;
KU916907;Coleoptera;Latridius minutus;Latridius minutus;1;Latridius minutus;0.999999999986045;
KU916925;Coleoptera;Denticollis linearis;Denticollis linearis;1;Denticollis linearis;1;
KU916936;Coleoptera;Hemicoelus nitidus;NA;1;Anobium nitidum;1;
KU916965;Coleoptera;Valgus hemipterus;Valgus hemipterus;1;Valgus hemipterus;1;
KU916995;Coleoptera;Tritoma bipustulata;NA;1;Tritoma bipustulata;1;
KU917005;Coleoptera;Phloeocharis subtilissima;Phloeocharis subtilissima;1;Phloeocharis subtilissima;1;
KU917015;Coleoptera;Grammoptera ustulata;Grammoptera ustulata;1;Grammoptera ustulata;1;
KU917109;Coleoptera;Tritoma bipustulata;NA;1;Tritoma bipustulata;1;
KU917111;Coleoptera;Leiopus nebulosus;NA;0.12;Leiopus linnei;0.911840242395976;
KU917132;Coleoptera;Anthaxia nitidula;Anthaxia nitidula;1;Anthaxia nitidula;1;
KU917151;Coleoptera;Trixagus duvalii;NA;NA;NA;0.999999999999998;
KU917309;Coleoptera;Zilora obscura;Zilora obscura;1;Zilora obscura;1;
KU917322;Coleoptera;Pteryx suturalis;NA;NA;Pteryx suturalis;1;
KU917336;Coleoptera;Evodinus clathratus;Evodinus clathratus;1;Evodinus clathratus;1;

KU917368;Coleoptera;Cis boleti;Cis boleti;1;Cis boleti;1;
KU917376;Coleoptera;Hypnogyra angularis;Hypnogyra glabra;1;Hypnogyra glabra;1;
KU917408;Coleoptera;Hylastes ater;Hylastes ater;1;Hylastes ater;0.999999999999773;
KU917431;Coleoptera;Pteryngium crenatum;Pteryngium crenatum;1;Pteryngium crenatum;1;
KU917443;Coleoptera;Euplectus karstenii;Euplectus karstenii;1;Euplectus karstenii;1;
KU917458;Coleoptera;Anthaxia quadripunctata;Anthaxia quadripunctata;1;Anthaxia quadripunctata;1;
KU917500;Coleoptera;Tomicus piniperda;NA;NA;Tomicus piniperda;1;
KU917556;Coleoptera;Triplax russica;Triplax russica;1;Triplax russica;1;
KU917564;Coleoptera;Anthaxia quadripunctata;Anthaxia quadripunctata;1;Anthaxia quadripunctata;1;
KU917581;Coleoptera;Dendrophilus pygmaeus;NA;1;Dendrophilus pygmaeus;1;
KU917618;Coleoptera;Placusa tachyporoides;Placusa tachyporoides;1;Placusa tachyporoides;0.999999995754933;
KU917622;Coleoptera;Polygraphus poligraphus;Polygraphus poligraphus;1;Polygraphus poligraphus;0.999999999993719;
KU917652;Coleoptera;Xylostiba monilicornis;Xylostiba monilicornis;1;Xylostiba monilicornis;1;
KU917658;Coleoptera;Valgus hemipterus;Valgus hemipterus;1;Valgus hemipterus;1;
KU917701;Coleoptera;Cis boleti;Cis boleti;1;Cis boleti;1;
KU917709;Coleoptera;Tillus elongatus;NA;NA;Tillus elongatus;1;
KU917759;Coleoptera;Glischrochilus hortensis;Glischrochilus hortensis;1;Glischrochilus hortensis;1;
KU917776;Coleoptera;Platystomos albinus;NA;NA;Platystomos albinus;1;
KU917790;Coleoptera;Phloeonomus punctipennis;Phloeonomus punctipennis;1;Phloeonomus punctipennis;1;
KU917807;Coleoptera;Hemicoelus nitidus;Anobium nitidum;1;Anobium nitidum;1;
KU917873;Coleoptera;Cis hispidus;NA;NA;Cis hispidus;0.999999946277599;
KU917897;Coleoptera;Epuraea variegata;Epuraea variegata;1;Epuraea variegata;0.999999999840242;
KU917925;Coleoptera;Hypnogyra angularis;Hypnogyra glabra;1;Hypnogyra glabra;1;
KU918052;Coleoptera;Trypodendron domesticum;Trypodendron domesticum;1;Trypodendron domesticum;1;
KU918101;Coleoptera;Platycerus caraboides;Platycerus caraboides;1;Platycerus caraboides;1;
KU918126;Coleoptera;Potamophilus acuminatus;Potamophilus acuminatus;NA;Potamophilus acuminatus;1;
KU918175;Coleoptera;Atrecus affinis;NA;NA;Atrecus affinis;1;
KU918177;Coleoptera;Tritoma bipustulata;NA;1;Tritoma bipustulata;1;
KU918269;Coleoptera;Tillus elongatus;NA;NA;Tillus elongatus;1;
KU918318;Coleoptera;Eledona agricola;Eledona agricola;0.014;Eledona agricola;1;
KU918334;Coleoptera;Valgus hemipterus;Valgus hemipterus;1;Valgus hemipterus;1;
KU918353;Coleoptera;Dendrophilus punctatus;Dendrophilus punctatus;1;Dendrophilus punctatus;1;
KU918362;Coleoptera;Anisotoma humeralis;Anisotoma humeralis;1;Anisotoma humeralis;0.999999999871534;
KU918379;Coleoptera;Triplax lepida;Triplax lepida;1;Triplax lepida;1;
KU918380;Coleoptera;Polygraphus poligraphus;Polygraphus poligraphus;1;Polygraphus poligraphus;0.999999999993719;
KU918396;Coleoptera;Ernobius abietinus;Ernobius abietinus;1;Ernobius abietinus;1;
KU918446;Coleoptera;Hymenalia rufipes;Hymenalia rufipes;NA;Hymenalia rufipes;1;
KU918467;Coleoptera;Bibloporus bicolor;Bibloporus bicolor;1;Bibloporus bicolor;0.999999999999915;
KU918508;Coleoptera;Plectophloeus nubigena;Plectophloeus nubigena;1;Plectophloeus nubigena;1;
KU918535;Coleoptera;Epuraea terminalis;Epuraea terminalis;NA;Epuraea placida;0.999963904637439;
KU918562;Coleoptera;Cis boleti;Cis boleti;1;Cis boleti;1;
KU918606;Coleoptera;Plectophloeus nubigena;Plectophloeus nubigena;1;Plectophloeus nubigena;0.999999999999972;
KU918607;Coleoptera;Cis hispidus;NA;0.317;Cis hispidus;0.99999991378775;
KU918636;Coleoptera;Tritoma bipustulata;NA;1;Tritoma bipustulata;1;
KU918649;Coleoptera;Cis dentatus;Cis dentatus;1;Cis dentatus;1;
KU918668;Coleoptera;Euryusa castanoptera;Euryusa castanoptera;NA;Euryusa castanoptera;1;
KU918672;Coleoptera;Anisotoma castanea;Anisotoma castanea;1;Anisotoma castanea;0.999999999999375;
KU918748;Coleoptera;Elateroides dermestoides;Elateroides dermestoides;1;Elateroides dermestoides;1;
KU918795;Coleoptera;Denticollis linearis;Denticollis linearis;1;Denticollis linearis;1;
KU918858;Coleoptera;Denticollis linearis;Denticollis linearis;1;Denticollis linearis;1;
KU918927;Coleoptera;Phloeonomus pusillus;Phloeonomus pusillus;1;Phloeonomus pusillus;1;
KU918937;Coleoptera;Euryusa optabilis;Euryusa optabilis;1;Euryusa optabilis;0.999999999980588;
KU918938;Coleoptera;Hemicoelus nitidus;NA;1;Anobium nitidum;1;
KU918943;Coleoptera;Platystomos albinus;NA;1;Platystomos albinus;1;
KU918944;Coleoptera;Xylotrechus antilope;Xylotrechus antilope;1;Xylotrechus antilope;1;
KU918948;Coleoptera;Tritoma bipustulata;NA;1;Tritoma bipustulata;1;
KU918972;Coleoptera;Platystomos albinus;NA;NA;Platystomos albinus;1;
KU919036;Coleoptera;Platystomos albinus;NA;NA;Platystomos albinus;1;
KU919050;Coleoptera;Elateroides dermestoides;Elateroides dermestoides;1;Elateroides dermestoides;1;
KU919092;Coleoptera;Xyleborinus saxesenii;Xyleborinus saxeseni;1;Xyleborinus saxeseni;1;
KU919100;Coleoptera;Anthaxia helvetica;Anthaxia helvetica;1;Anthaxia helvetica;1;
KU919167;Coleoptera;Ptenidium turgidum;Ptenidium turgidum;1;Ptenidium turgidum;1;
KU919169;Coleoptera;Exocentrus adspersus;Exocentrus adspersus;1;Exocentrus adspersus;1;
KU919207;Coleoptera;Denticollis linearis;Denticollis linearis;1;Denticollis linearis;1;
KU919260;Coleoptera;Anisoxya fuscula;NA;NA;Anisoxya fuscula;1;
KU919312;Coleoptera;Grammoptera ruficornis;Grammoptera ruficornis;1;Grammoptera ruficornis;1;

KU919342;Coleoptera;Anthaxia godeti;Anthaxia godeti;1;Anthaxia godeti;1;
KU919362;Coleoptera;Pteryx suturalis;Pteryx suturalis;1;Pteryx suturalis;1;
KU919407;Coleoptera;Tillus elongatus;NA;NA;Tillus elongatus;1;
KU919416;Coleoptera;Denticollis linearis;Denticollis linearis;1;Denticollis linearis;1;
KU919477;Coleoptera;Hymenalia rufipes;Hymenalia rufipes;NA;Hymenalia rufipes;1;
KU919565;Coleoptera;Xyleborinus saxesenii;Xyleborinus saxeseni;1;Xyleborinus saxeseni;1;
KU919574;Coleoptera;Anthaxia candens;Anthaxia candens;1;Anthaxia candens;1;
KY683603;Coleoptera;Leptura aethiops;Leptura aethiops;1;Leptura aethiops;1;
KY683608;Coleoptera;Gaurotes virginea kozhevnikovi;Gaurotes virginea;NA;Gaurotes virginea;1;
KY683629;Coleoptera;Leptura aethiops;Leptura aethiops;1;Leptura aethiops;1;
KY683651;Coleoptera;Gaurotes virginea kozhevnikovi;Gaurotes virginea;NA;Gaurotes virginea;1;
KY683702;Coleoptera;Gaurotes virginea kozhevnikovi;Gaurotes virginea;NA;Gaurotes virginea;1;
MF286233;Coleoptera;Agrilus viridis;Agrilus viridis;1;Agrilus viridis;0.999997752651974;
MF286234;Coleoptera;Agrilus viridis;Agrilus viridis;1;Agrilus viridis;0.999996214958885;
MF286235;Coleoptera;Agrilus viridis;Agrilus viridis;1;Agrilus viridis;0.998686873422301;
MF286238;Coleoptera;Agrilus viridis;Agrilus viridis;1;Agrilus viridis;0.999999712126267;
MF286256;Coleoptera;Agrilus viridis;Agrilus viridis;1;Agrilus viridis;0.99999718436355;
MF286257;Coleoptera;Agrilus viridis;Agrilus viridis;1;Agrilus viridis;0.993900718357746;
MF286258;Coleoptera;Agrilus viridis;Agrilus viridis;1;Agrilus viridis;0.999973062684815;
MF286260;Coleoptera;Agrilus viridis;Agrilus viridis;1;Agrilus viridis;0.999996532948827;
MF286261;Coleoptera;Agrilus viridis;Agrilus viridis;1;Agrilus viridis;0.999997125034565;
MF286262;Coleoptera;Agrilus viridis;Agrilus viridis;1;Agrilus viridis;0.999997125034565;
MF286320;Coleoptera;Agrilus viridis;Agrilus viridis;1;Agrilus viridis;0.999998254680252;
MF286321;Coleoptera;Agrilus viridis;Agrilus viridis;1;Agrilus viridis;0.999991039621348;
MF286326;Coleoptera;Agrilus viridis;Agrilus viridis;1;Agrilus viridis;0.999997622385463;
MF373734;Coleoptera;Trypodendron domesticum;Trypodendron domesticum;1;Trypodendron domesticum;1;
MF373742;Coleoptera;Trypodendron lineatum;Trypodendron lineatum;0.04;Trypodendron lineatum;1;
MF543033;Coleoptera;Agrilus viridis;Agrilus viridis;1;Agrilus viridis;0.999999478467282;
MF543034;Coleoptera;Agrilus viridis;Agrilus viridis;1;Agrilus viridis;0.999999152067365;
MF543035;Coleoptera;Agrilus viridis;Agrilus viridis;1;Agrilus viridis;0.999999176740713;
MF543036;Coleoptera;Agrilus viridis;Agrilus viridis;1;Agrilus viridis;0.999999503711488;
MF543037;Coleoptera;Agrilus viridis;NA;NA;Agrilus graminis;1;
MF611994;Coleoptera;Xyleborinus saxesenii;NA;0.318;Xyleborinus saxesenii;0.999999074788549;
MF611995;Coleoptera;Xyleborinus saxesenii;Xyleborinus saxeseni;1;Xyleborinus saxeseni;1;
MF611996;Coleoptera;Xyleborinus saxesenii;Xyleborinus saxeseni;0.006;Xyleborinus saxesenii;0.999977291933239;
MF611997;Coleoptera;Xyleborinus saxesenii;Xyleborinus saxeseni;0.006;Xyleborinus saxesenii;0.999977291933239;
MF611998;Coleoptera;Xyleborinus saxesenii;Xyleborinus saxeseni;0.006;Xyleborinus saxesenii;0.999977291933239;
MF611999;Coleoptera;Xyleborus dryographus;Xyleborus dryographus;1;Xyleborus dryographus;1;
MF612000;Coleoptera;Xyleborus dryographus;Xyleborus dryographus;1;Xyleborus dryographus;1;
MF612001;Coleoptera;Xyleborus dryographus;Xyleborus dryographus;1;Xyleborus dryographus;1;
MF612002;Coleoptera;Xyleborus dryographus;Xyleborus dryographus;1;Xyleborus dryographus;1;
MF805176;Coleoptera;Agrilus viridis;Agrilus viridis;NA;Agrilus suvorovi;0.968856705633157;
MH020302;Coleoptera;Bitoma crenata;Bitoma crenata;1;Bitoma crenata;1;
MH020346;Coleoptera;Anoplodera sexguttata;Anoplodera sexguttata;1;Anoplodera sexguttata;1;
MH020440;Coleoptera;Valgus hemipterus;Valgus hemipterus;1;Valgus hemipterus;1;
MH020441;Coleoptera;Valgus hemipterus;Valgus hemipterus;1;Valgus hemipterus;1;
MH115489;Coleoptera;Triplax russica;Triplax russica;1;Triplax russica;1;
MH115490;Coleoptera;Triplax aenea;Triplax aenea;1;Triplax aenea;1;
MH115492;Coleoptera;Tritoma bipustulata;NA;1;Tritoma bipustulata;1;
MH115493;Coleoptera;Tritoma bipustulata;NA;1;Tritoma bipustulata;1;
MH115547;Coleoptera;Plagionotus detritus;Plagionotus detritus;1;Plagionotus detritus;0.99999999874592;
MH115561;Coleoptera;Anthaxia nitidula;Anthaxia nitidula;1;Anthaxia nitidula;1;
MH115574;Coleoptera;Trypodendron lineatum;Trypodendron lineatum;1;Trypodendron lineatum;1;
MK116398;Coleoptera;Prinobius myardi;Prinobius myardi;1;Prinobius myardi;0.99999997937951;
MK116399;Coleoptera;Prinobius myardi;Prinobius myardi;1;Prinobius myardi;0.999999984780715;
MK116400;Coleoptera;Prinobius myardi;Prinobius myardi;1;Prinobius myardi;0.999999960534667;
MK116401;Coleoptera;Prinobius myardi;Prinobius myardi;0.999;Prinobius myardi;0.999999981711312;
MK116402;Coleoptera;Prinobius myardi;Prinobius myardi;1;Prinobius myardi;0.99999997899468;
MK116403;Coleoptera;Prinobius myardi;Prinobius myardi;1;Prinobius myardi;0.999999952710796;
MK116404;Coleoptera;Prinobius myardi;Prinobius myardi;1;Prinobius myardi;0.999999976363768;
MK153066;Coleoptera;Glischrochilus hortensis;Glischrochilus hortensis;1;Glischrochilus hortensis;1;
MK643361;Coleoptera;Ips sexdentatus;Ips sexdentatus;1;Ips sexdentatus;1;
MK643362;Coleoptera;Ips sexdentatus;NA;NA;Ips sexdentatus;1;
MK643363;Coleoptera;Ips sexdentatus;Ips sexdentatus;NA;Ips sexdentatus;1;
MK643364;Coleoptera;Ips sexdentatus;Ips sexdentatus;NA;Ips sexdentatus;1;
MK643365;Coleoptera;Ips sexdentatus;Ips sexdentatus;NA;Ips sexdentatus;1;
MK643366;Coleoptera;Ips sexdentatus;Ips sexdentatus;NA;Ips sexdentatus;1;
MK643367;Coleoptera;Ips sexdentatus;NA;NA;Ips sexdentatus;1;
MK643368;Coleoptera;Ips sexdentatus;NA;NA;Ips sexdentatus;1;
MK643369;Coleoptera;Ips sexdentatus;NA;NA;Ips sexdentatus;1;
MK643371;Coleoptera;Ips sexdentatus;Ips sexdentatus;1;Ips sexdentatus;1;
MK892199;Coleoptera;Hylurgops palliatus;Hylurgops palliatus;1;Hylurgops palliatus;1;
MK892217;Coleoptera;Platystomos albinus;NA;NA;Platystomos albinus;1;

MN182812;Coleoptera;Hesperophanes sericeus;Hesperophanes sericeus;1;Hesperophanes sericeus;1;
MN182826;Coleoptera;Globicornis bifasciata;NA;NA;Globicornis bifasciata;1;
MN182882;Coleoptera;Gnathoncus rotundatus;Gnathoncus buyssoni;NA;Gnathoncus buyssoni;1;
MN182951;Coleoptera;Denops albofasciatus;Denops albofasciatus;1;Denops albofasciatus;1;
MN182954;Coleoptera;Anthaxia confusa;Anthaxia confusa;1;Anthaxia confusa;1;
MN182966;Coleoptera;Denops albofasciatus;Denops albofasciatus;1;Denops albofasciatus;0.999999999986272;
MN182978;Coleoptera;Iphthiminus italicus;NA;NA;Iphthiminus italicus;1;
MN182990;Coleoptera;Homophthalmus rugicollis;Dryophilus rugicollis;1;Dryophilus rugicollis;0.999999999999943;
MN182996;Coleoptera;Denops albofasciatus;Denops albofasciatus;1;Denops albofasciatus;1;
MT313186;Coleoptera;Ips sexdentatus;Ips sexdentatus;1;Ips sexdentatus;1;
MT313187;Coleoptera;Ips sexdentatus;Ips sexdentatus;1;Ips sexdentatus;1;
MT313188;Coleoptera;Ips sexdentatus;NA;NA;Ips sexdentatus;1;
MT313189;Coleoptera;Ips sexdentatus;Ips sexdentatus;1;Ips sexdentatus;1;
MT313190;Coleoptera;Ips sexdentatus;Ips sexdentatus;1;Ips sexdentatus;1;
MT313191;Coleoptera;Ips sexdentatus;Ips sexdentatus;1;Ips sexdentatus;1;
MT313192;Coleoptera;Ips sexdentatus;Ips sexdentatus;1;Ips sexdentatus;1;
MT313193;Coleoptera;Ips sexdentatus;Ips sexdentatus;1;Ips sexdentatus;1;
MT313194;Coleoptera;Ips sexdentatus;Ips sexdentatus;1;Ips sexdentatus;1;
MT920089;Coleoptera;Ips sexdentatus;Ips sexdentatus;1;Ips sexdentatus;1;
MT920090;Coleoptera;Ips sexdentatus;Ips sexdentatus;1;Ips sexdentatus;1;
MT920091;Coleoptera;Ips sexdentatus;Ips sexdentatus;1;Ips sexdentatus;1;
MT920092;Coleoptera;Ips sexdentatus;Ips sexdentatus;1;Ips sexdentatus;1;
MT920093;Coleoptera;Ips sexdentatus;Ips sexdentatus;1;Ips sexdentatus;1;
MT920094;Coleoptera;Ips sexdentatus;Ips sexdentatus;1;Ips sexdentatus;1;
MT920095;Coleoptera;Ips sexdentatus;Ips sexdentatus;1;Ips sexdentatus;1;
MT920096;Coleoptera;Ips sexdentatus;Ips sexdentatus;1;Ips sexdentatus;1;
MT920097;Coleoptera;Ips sexdentatus;Ips sexdentatus;1;Ips sexdentatus;1;
MT920098;Coleoptera;Ips sexdentatus;Ips sexdentatus;1;Ips sexdentatus;1;
MT920099;Coleoptera;Ips sexdentatus;Ips sexdentatus;1;Ips sexdentatus;1;
MT920100;Coleoptera;Ips sexdentatus;Ips sexdentatus;1;Ips sexdentatus;1;
MT920101;Coleoptera;Ips sexdentatus;Ips sexdentatus;1;Ips sexdentatus;1;
MT920102;Coleoptera;Ips sexdentatus;Ips sexdentatus;1;Ips sexdentatus;1;
MT920103;Coleoptera;Ips sexdentatus;Ips sexdentatus;1;Ips sexdentatus;1;
MT920104;Coleoptera;Ips sexdentatus;Ips sexdentatus;1;Ips sexdentatus;1;
MT920105;Coleoptera;Ips sexdentatus;Ips sexdentatus;1;Ips sexdentatus;1;
MT920106;Coleoptera;Ips sexdentatus;Ips sexdentatus;1;Ips sexdentatus;1;
MT920107;Coleoptera;Ips sexdentatus;Ips sexdentatus;1;Ips sexdentatus;1;
MT936905;Coleoptera;Ips sexdentatus;Ips sexdentatus;1;Ips sexdentatus;1;
MT936906;Coleoptera;Ips sexdentatus;Ips sexdentatus;1;Ips sexdentatus;1;
MT936907;Coleoptera;Ips sexdentatus;Ips sexdentatus;1;Ips sexdentatus;1;
MT936908;Coleoptera;Ips sexdentatus;NA;NA;Ips sexdentatus;1;
MT936909;Coleoptera;Ips sexdentatus;Ips sexdentatus;1;Ips sexdentatus;1;
MT936910;Coleoptera;Ips sexdentatus;Ips sexdentatus;1;Ips sexdentatus;1;
MT936911;Coleoptera;Ips sexdentatus;Ips sexdentatus;1;Ips sexdentatus;1;
MT936912;Coleoptera;Ips sexdentatus;Ips sexdentatus;1;Ips sexdentatus;1;
MT936913;Coleoptera;Ips sexdentatus;Ips sexdentatus;1;Ips sexdentatus;1;
MT936917;Coleoptera;Ips sexdentatus;Ips sexdentatus;1;Ips sexdentatus;1;
MT936918;Coleoptera;Ips sexdentatus;Ips sexdentatus;1;Ips sexdentatus;1;
MT936919;Coleoptera;Ips sexdentatus;Ips sexdentatus;1;Ips sexdentatus;1;
MT936920;Coleoptera;Ips sexdentatus;Ips sexdentatus;1;Ips sexdentatus;1;
MT936921;Coleoptera;Ips sexdentatus;Ips sexdentatus;1;Ips sexdentatus;1;
MT936922;Coleoptera;Ips sexdentatus;Ips sexdentatus;1;Ips sexdentatus;1;
MT936923;Coleoptera;Ips sexdentatus;Ips sexdentatus;1;Ips sexdentatus;1;
MT936924;Coleoptera;Ips sexdentatus;Ips sexdentatus;1;Ips sexdentatus;1;
MT936925;Coleoptera;Ips sexdentatus;Ips sexdentatus;1;Ips sexdentatus;1;
MT936926;Coleoptera;Ips sexdentatus;Ips sexdentatus;1;Ips sexdentatus;1;
MT936927;Coleoptera;Ips sexdentatus;Ips sexdentatus;1;Ips sexdentatus;1;
MT936928;Coleoptera;Ips sexdentatus;Ips sexdentatus;1;Ips sexdentatus;1;
MW259333;Coleoptera;Leptusa fumida;Leptusa fumida;1;Leptusa fumida;1;
MW259344;Coleoptera;Hypoganus inunctus;Hypoganus inunctus;1;Hypoganus inunctus;1;
MW259357;Coleoptera;Phloeocharis subtilissima;Phloeocharis subtilissima;1;Phloeocharis subtilissima;1;
MW259363;Coleoptera;Denticollis linearis;Denticollis linearis;1;Denticollis linearis;1;
MW259380;Coleoptera;Phloeocharis subtilissima;Phloeocharis subtilissima;NA;Phloeocharis subtilissima;1;
MW259394;Coleoptera;Denticollis linearis;Denticollis linearis;1;Denticollis linearis;1;
MW259405;Coleoptera;Phloeocharis subtilissima;Phloeocharis subtilissima;NA;Phloeocharis subtilissima;1;
MW259408;Coleoptera;Denticollis linearis;Denticollis linearis;1;Denticollis linearis;1;
MW259423;Coleoptera;Denticollis linearis;Denticollis linearis;1;Denticollis linearis;1;
MW259433;Coleoptera;Leptusa fumida;Leptusa fumida;1;Leptusa fumida;1;
MW259437;Coleoptera;Phloeocharis subtilissima;Phloeocharis subtilissima;NA;Phloeocharis subtilissima;1;
MW259451;Coleoptera;Epuraea variegata;Epuraea variegata;1;Epuraea variegata;0.999999999840242;
MW259460;Coleoptera;Phloeocharis subtilissima;Phloeocharis subtilissima;NA;Phloeocharis subtilissima;1;

MW259468;Coleoptera;Epuraea variegata;Epuraea variegata;1;Epuraea variegata;0.999999999803379;
MW259487;Coleoptera;Denticollis linearis;Denticollis linearis;1;Denticollis linearis;1;
MW259505;Coleoptera;Denticollis linearis;Denticollis linearis;1;Denticollis linearis;1;
MW259610;Coleoptera;Epuraea variegata;Epuraea variegata;1;Epuraea variegata;0.999999999840242;
MW259616;Coleoptera;Hypoganus inunctus;Hypoganus inunctus;1;Hypoganus inunctus;1;
MW259625;Coleoptera;Phloeocharis subtilissima;Phloeocharis subtilissima;NA;Phloeocharis subtilissima;1;
MW259652;Coleoptera;Phloeocharis subtilissima;Phloeocharis subtilissima;NA;Phloeocharis subtilissima;1;
MW259683;Coleoptera;Phloeocharis subtilissima;Phloeocharis subtilissima;NA;Phloeocharis subtilissima;1;
MW259727;Coleoptera;Denticollis linearis;Denticollis linearis;1;Denticollis linearis;1;
MW259734;Coleoptera;Leptusa fumida;Leptusa fumida;1;Leptusa fumida;0.999999999999915;
MW259741;Coleoptera;Denticollis linearis;Denticollis linearis;1;Denticollis linearis;1;
MW259742;Coleoptera;Denticollis linearis;Denticollis linearis;1;Denticollis linearis;1;
MW259763;Coleoptera;Denticollis linearis;Denticollis linearis;1;Denticollis linearis;1;
MW259823;Coleoptera;Tritoma bipustulata;NA;1;Tritoma bipustulata;1;
MW259826;Coleoptera;Leptusa pulchella;Leptusa pulchella;1;Leptusa pulchella;1;
MW259842;Coleoptera;Phloeocharis subtilissima;Phloeocharis subtilissima;NA;Phloeocharis subtilissima;1;
MW259850;Coleoptera;Denticollis linearis;Denticollis linearis;1;Denticollis linearis;1;
MW259863;Coleoptera;Leptusa pulchella;Leptusa pulchella;1;Leptusa pulchella;1;
MW259868;Coleoptera;Leptusa fumida;Leptusa fumida;1;Leptusa fumida;0.999999999999886;
MW259881;Coleoptera;Denticollis linearis;Denticollis linearis;1;Denticollis linearis;1;
MW259892;Coleoptera;Phloeocharis subtilissima;Phloeocharis subtilissima;1;Phloeocharis subtilissima;1;
MW259958;Coleoptera;Triphyllus bicolor;Triphyllus bicolor;1;Triphyllus bicolor;1;
MZ606945;Coleoptera;Epuraea thoracica;Epuraea thoracica;1;Epuraea thoracica;0.99997650276254;
MZ607099;Coleoptera;Uloma rufa;Uloma rufa;1;Uloma rufa;1;
MZ607484;Coleoptera;Gonotropis dorsalis;NA;NA;Gonotropis dorsalis;1;
MZ607976;Coleoptera;Atomaria vespertina;Atomaria vespertina;1;Atomaria vespertina;1;
MZ608248;Coleoptera;Hylurgops palliatus;Hylurgops palliatus;1;Hylurgops palliatus;1;
MZ608479;Coleoptera;Grynocharis oblonga;NA;1;Grynocharis oblonga;1;
MZ608801;Coleoptera;Platycerus caraboides;Platycerus caraboides;1;Platycerus caraboides;1;
MZ609239;Coleoptera;Epuraea thoracica;Epuraea thoracica;1;Epuraea thoracica;0.999982218697291;
MZ609439;Coleoptera;Trypodendron domesticum;Trypodendron domesticum;1;Trypodendron domesticum;1;
MZ609627;Coleoptera;Phloeocharis subtilissima;Phloeocharis subtilissima;1;Phloeocharis subtilissima;1;
MZ609697;Coleoptera;Anisotoma castanea;Anisotoma castanea;1;Anisotoma castanea;0.999999999998835;
MZ609906;Coleoptera;Zilora obscura;NA;NA;Zilora obscura;1;
MZ610748;Coleoptera;Gonotropis dorsalis;NA;NA;Gonotropis dorsalis;1;
MZ611151;Coleoptera;Grynocharis oblonga;Grynocharis oblonga;1;Grynocharis oblonga;1;
MZ611361;Coleoptera;Tillus elongatus;Tillus elongatus;1;Tillus elongatus;0.999999999998181;
MZ629289;Coleoptera;Euplectus mutator;Euplectus karstenii;0.997;Euplectus karstenii;0.999997742400088;
MZ629346;Coleoptera;Uloma rufa;Uloma rufa;1;Uloma rufa;1;
MZ629562;Coleoptera;Ptiliolum caledonicum;Ptiliolum caledonicum;1;Ptiliolum caledonicum;1;
MZ629591;Coleoptera;Anisotoma orbicularis;Anisotoma orbicularis;1;Anisotoma orbicularis;1;
MZ629980;Coleoptera;Phloeonomus pusillus;Phloeonomus pusillus;1;Phloeonomus pusillus;1;
MZ630253;Coleoptera;Epuraea terminalis;Epuraea terminalis;1;Epuraea terminalis;0.999999449064925;
MZ630316;Coleoptera;Euplectus mutator;Euplectus karstenii;0.984;Euplectus karstenii;0.999937468459556;
MZ630440;Coleoptera;Zilora obscura;NA;NA;Zilora obscura;1;
MZ630661;Coleoptera;Epuraea terminalis;Epuraea terminalis;1;Epuraea terminalis;0.999999449064925;
MZ630758;Coleoptera;Xestobium rufovillosum;NA;1;Xestobium rufovillosum;1;
MZ630778;Coleoptera;Epuraea unicolor;Epuraea unicolor;1;Epuraea unicolor;0.999999999999886;
MZ630791;Coleoptera;Xestobium rufovillosum;NA;1;Xestobium rufovillosum;1;
MZ630886;Coleoptera;Bibloporus minutus;Bibloporus minutus;1;Bibloporus minutus;1;
MZ631017;Coleoptera;Bibloporus bicolor;Bibloporus bicolor;1;Bibloporus bicolor;0.999999999999829;
MZ631132;Coleoptera;Xestobium rufovillosum;NA;1;Xestobium rufovillosum;1;
MZ631514;Coleoptera;Pteryx suturalis;Pteryx suturalis;1;Pteryx suturalis;1;
MZ631586;Coleoptera;Dendrophilus pygmaeus;Dendrophilus pygmaeus;1;Dendrophilus pygmaeus;1;
MZ631637;Coleoptera;Euplectus punctatus;Euplectus punctatus;1;Euplectus punctatus;0.999999999998863;
MZ631965;Coleoptera;Cis comptus;Cis comptus;1;Cis comptus;1;
MZ632046;Coleoptera;Bibloporus bicolor;Bibloporus bicolor;1;Bibloporus bicolor;0.999999999999858;
MZ632118;Coleoptera;Bibloporus minutus;Bibloporus minutus;1;Bibloporus minutus;1;
MZ632137;Coleoptera;Dendrophagus crenatus;Dendrophagus crenatus;1;Dendrophagus crenatus;0.999999999982776;
MZ632241;Coleoptera;Euplectus punctatus;Euplectus punctatus;1;Euplectus punctatus;1;
MZ632268;Coleoptera;Anisotoma castanea;Anisotoma castanea;1;Anisotoma castanea;0.999999999999375;

MZ632391;Coleoptera;Bibloporus bicolor;Bibloporus bicolor;1;Bibloporus bicolor;0.999999999999858;
MZ632551;Coleoptera;Cis castaneus;Cis nitidus;1;Cis nitidus;0.999999999996618;
MZ632671;Coleoptera;Grynocharis oblonga;Grynocharis oblonga;1;Grynocharis oblonga;1;
MZ632855;Coleoptera;Bibloporus bicolor;Bibloporus bicolor;1;Bibloporus bicolor;0.999999999999488;
MZ633049;Coleoptera;Euplectus karstenii;Euplectus karstenii;1;Euplectus karstenii;1;
MZ633255;Coleoptera;Ernobius abietinus;Ernobius abietinus;1;Ernobius abietinus;1;
MZ633277;Coleoptera;Dendrophilus punctatus;NA;1;Dendrophilus punctatus;1;
MZ633333;Coleoptera;Euplectus punctatus;Euplectus punctatus;1;Euplectus punctatus;1;
MZ633606;Coleoptera;Euplectus punctatus;Euplectus punctatus;1;Euplectus punctatus;1;
MZ633702;Coleoptera;Phloeonomus pusillus;Phloeonomus pusillus;1;Phloeonomus pusillus;1;
MZ633713;Coleoptera;Tritoma bipustulata;NA;1;Tritoma bipustulata;1;
MZ633819;Coleoptera;Euplectus karstenii;Euplectus karstenii;1;Euplectus karstenii;1;
MZ633871;Coleoptera;Dendrophilus punctatus;NA;1;Dendrophilus punctatus;1;
MZ633982;Coleoptera;Bibloporus bicolor;Bibloporus bicolor;1;Bibloporus bicolor;0.999999999999801;
MZ634002;Coleoptera;Pteryx suturalis;NA;NA;Pteryx suturalis;1;
MZ634066;Coleoptera;Epuraea unicolor;Epuraea unicolor;1;Epuraea unicolor;0.999999999999972;
MZ634092;Coleoptera;Euplectus kirbii;NA;0.319;NA;0.999999999999995;
MZ656272;Coleoptera;Glischrochilus quadripunctatus;Glischrochilus tremulae;0.032;Glischrochilus tremulae;0.99797294682308;
MZ656569;Coleoptera;Latridius consimilis;Latridius consimilis;1;Latridius consimilis;1;
MZ656570;Coleoptera;Polygraphus poligraphus;Polygraphus poligraphus;1;Polygraphus poligraphus;0.999999999998977;
MZ656797;Coleoptera;Epuraea rufomarginata;Epuraea rufomarginata;1;Epuraea rufomarginata;1;
MZ656874;Coleoptera;Anisotoma glabra;Anisotoma glabra;1;Anisotoma glabra;1;
MZ657038;Coleoptera;Pteryx suturalis;NA;NA;Pteryx suturalis;1;
MZ657118;Coleoptera;Zilora obscura;Zilora obscura;1;Zilora obscura;1;
MZ657130;Coleoptera;Atrecus affinis;NA;NA;Atrecus affinis;1;
MZ657131;Coleoptera;Triplax aenea;Triplax aenea;1;Triplax aenea;1;
MZ657232;Coleoptera;Glischrochilus hortensis;Glischrochilus hortensis;1;Glischrochilus hortensis;1;
MZ657577;Coleoptera;Glischrochilus quadripunctatus;Glischrochilus quadripunctatus;0.989;Glischrochilus quadripunctatus;0.999999890836938;
MZ657597;Coleoptera;Atrecus pilicornis;Atrecus pilicornis;1;Atrecus pilicornis;1;
MZ657607;Coleoptera;Cis glabratus;Cis glabratus;1;Cis glabratus;1;
MZ657895;Coleoptera;Euryusa castanoptera;Euryusa castanoptera;1;Euryusa castanoptera;1;
MZ658069;Coleoptera;Atrecus pilicornis;Atrecus pilicornis;1;Atrecus pilicornis;1;
MZ658290;Coleoptera;Hylurgops palliatus;Hylurgops palliatus;1;Hylurgops palliatus;1;
MZ658387;Coleoptera;Xestobium rufovillosum;NA;1;Xestobium rufovillosum;1;
MZ658394;Coleoptera;Epuraea rufomarginata;Epuraea rufomarginata;1;Epuraea rufomarginata;1;
MZ658876;Coleoptera;Anisotoma axillaris;Anisotoma axillaris;1;Anisotoma axillaris;0.999999999963052;
MZ659113;Coleoptera;Epuraea thoracica;Epuraea thoracica;0.005;Epuraea thoracica;0.999962246641901;
MZ659194;Coleoptera;Anisotoma humeralis;Anisotoma humeralis;1;Anisotoma humeralis;0.999999999922693;
MZ659575;Coleoptera;Euplectus karstenii;Euplectus karstenii;1;Euplectus karstenii;0.999999999999972;
MZ659612;Coleoptera;Anisotoma castanea;Anisotoma castanea;1;Anisotoma castanea;0.99999999999892;
MZ659725;Coleoptera;Atrecus pilicornis;Atrecus pilicornis;1;Atrecus pilicornis;1;
MZ659768;Coleoptera;Anisotoma humeralis;Anisotoma humeralis;1;Anisotoma humeralis;0.999999999966832;
MZ659828;Coleoptera;Triplax aenea;Triplax aenea;NA;Triplax aenea;1;
MZ659945;Coleoptera;Platycerus caprea;Platycerus caprea;1;Platycerus caprea;1;
MZ660069;Coleoptera;Dendrophagus crenatus;Dendrophagus crenatus;1;Dendrophagus crenatus;0.999999999998693;
MZ660264;Coleoptera;Elateroides dermestoides;Elateroides dermestoides;1;Elateroides dermestoides;1;
MZ660274;Coleoptera;Uloma rufa;Uloma rufa;1;Uloma rufa;1;
MZ660362;Coleoptera;Grynocharis oblonga;Grynocharis oblonga;1;Grynocharis oblonga;1;
MZ660402;Coleoptera;Anisotoma axillaris;Anisotoma axillaris;1;Anisotoma axillaris;0.999999999931191;
MZ660746;Coleoptera;Placusa atrata;Placusa atrata;1;Placusa atrata;0.999999999999972;
MZ660885;Coleoptera;Euryusa castanoptera;Euryusa castanoptera;1;Euryusa castanoptera;1;
MZ660951;Coleoptera;Epuraea rufomarginata;Epuraea rufomarginata;1;Epuraea rufomarginata;1;
MZ660969;Coleoptera;Gonotropis dorsalis;Gonotropis dorsalis;1;Gonotropis dorsalis;1;
MZ661011;Coleoptera;Epuraea rufomarginata;Epuraea rufomarginata;1;Epuraea rufomarginata;1;
NC_036274;Coleoptera;Vincenzellus ruficollis;NA;NA;NA;0.999999999999916;
NC_036281;Coleoptera;Ips sexdentatus;Ips sexdentatus;1;NA;0.999999999999967;
NC_036286;Coleoptera;Trypodendron domesticum;Trypodendron domesticum;1;Trypodendron domesticum;0.999917621850669;
NC_036290;Coleoptera;Hylastes attenuatus;Hylastes attenuatus;1;Hylastes attenuatus;0.999803724873253;
NC_036293;Coleoptera;Anisandrus dispar;Anisandrus dispar;1;Anisandrus dispar;0.857065340434243;
NC_036294;Coleoptera;Gnathotrichus materiarius;Gnathotrichus materiarius;1;Gnathotrichus materiarius;0.999999650013994;

NOBAR005-10;Coleoptera;Trypodendron lineatum;Trypodendron lineatum;0.003;Trypodendron lineatum;1;
NOCLP1686-19;Coleoptera;Denticollis linearis;Denticollis linearis;1;Denticollis linearis;1;
NOCLP1687-19;Coleoptera;Denticollis linearis;Denticollis linearis;1;Denticollis linearis;1;
NOCLP1693-19;Coleoptera;Trixagus atticus;Trixagus atticus;1;Trixagus atticus;1;
NOCLP1694-19;Coleoptera;Trixagus atticus;Trixagus atticus;1;Trixagus atticus;1;
NOCLP1703-19;Coleoptera;Trixagus carinifrons;Trixagus carinifrons;1;Trixagus carinifrons;1;
NOCLP1735-19;Coleoptera;Hylastes angustatus;Hylastes angustatus;1;Hylastes angustatus;1;
NOCLP1770-19;Coleoptera;Ips sexdentatus;Ips sexdentatus;1;Ips sexdentatus;1;
NOCLP1775-19;Coleoptera;Xyleborinus saxesenii;Xyleborinus saxeseni;1;Xyleborinus saxeseni;1;
NOCLP1783-19;Coleoptera;Trypodendron lineatum;Trypodendron lineatum;0.013;Trypodendron lineatum;1;
NOCLP1788-19;Coleoptera;Xyleborus cryptographus;Xyleborus cryptographus;1;Xyleborus cryptographus;1;
NOCLP1789-19;Coleoptera;Xyleborus cryptographus;Xyleborus cryptographus;1;Xyleborus cryptographus;1;
NOCLP1791-19;Coleoptera;Anisandrus dispar;Anisandrus dispar;1;Anisandrus dispar;1;
NOCLP1793-19;Coleoptera;Anisandrus dispar;Anisandrus dispar;1;Anisandrus dispar;1;
NOCOL015-12;Coleoptera;Denticollis linearis;Denticollis linearis;1;Denticollis linearis;1;
NOCOL016-12;Coleoptera;Denticollis linearis;Denticollis linearis;1;Denticollis linearis;1;
NOCOL018-12;Coleoptera;Denticollis linearis;Denticollis linearis;1;Denticollis linearis;1;
OK139664;Coleoptera;Xestobium rufovillosum;NA;1;Xestobium rufovillosum;1;
OK142732;Coleoptera;Xestobium rufovillosum;Xestobium rufovillosum;1;Xestobium rufovillosum;1;
OK142733;Coleoptera;Xestobium rufovillosum;Xestobium rufovillosum;1;Xestobium rufovillosum;1;
OK142771;Coleoptera;Xestobium rufovillosum;NA;1;Xestobium rufovillosum;1;
OK144134;Coleoptera;Xestobium rufovillosum;NA;1;Xestobium rufovillosum;1;
OK144142;Coleoptera;Xestobium rufovillosum;Xestobium rufovillosum;1;Xestobium rufovillosum;1;
POLYT005-19;Coleoptera;Glischrochilus quadripunctatus;NA;0.012;Glischrochilus tremulae;0.967840601585585;
SCOL018-12;Coleoptera;Trypodendron lineatum;Trypodendron lineatum;0.034;Trypodendron lineatum;0.999999160498288;
SCOL029-12;Coleoptera;Trypodendron domesticum;Trypodendron domesticum;1;Trypodendron domesticum;0.999999999966434;
SCOL039-12;Coleoptera;Trypodendron domesticum;Trypodendron domesticum;1;Trypodendron domesticum;0.999999999996277;
SCOL045-12;Coleoptera;Polygraphus poligraphus;Polygraphus poligraphus;1;Polygraphus poligraphus;1;
SCOL049-12;Coleoptera;Trypodendron lineatum;Trypodendron lineatum;0.028;Trypodendron lineatum;0.999999944681322;
SCOL050-12;Coleoptera;Hylastes attenuatus;Hylastes attenuatus;1;Hylastes attenuatus;0.999999999963421;
SCOL061-12;Coleoptera;Trypodendron lineatum;Trypodendron lineatum;0.003;Trypodendron lineatum;0.999999788061694;
SCOL065-12;Coleoptera;Ips sexdentatus;Ips sexdentatus;NA;Ips sexdentatus;0.999999992695166;
SCOL110-12;Coleoptera;Trypodendron lineatum;Trypodendron lineatum;0.002;Trypodendron lineatum;0.999999913940716;
SCOL153-12;Coleoptera;Trypodendron lineatum;Trypodendron lineatum;0.002;Trypodendron lineatum;0.999999913940716;
SCOL158-12;Coleoptera;Trypodendron lineatum;Trypodendron lineatum;0.002;Trypodendron lineatum;0.999999913940716;
SCOL162-12;Coleoptera;Trypodendron lineatum;Trypodendron lineatum;0.003;Trypodendron lineatum;0.999999999999972;
SCOL171-12;Coleoptera;Hylurgops palliatus;Hylurgops palliatus;1;Hylurgops palliatus;0.999999997941359;
SCOL172-12;Coleoptera;Trypodendron lineatum;Trypodendron lineatum;0.025;Trypodendron lineatum;0.999998012066059;
SCOL174-12;Coleoptera;Ips typographus;NA;0.022;Ips typographus;0.999999998631893;
SCOL178-12;Coleoptera;Trypodendron lineatum;Trypodendron lineatum;NA;Trypodendron lineatum;0.999999892616052;
SCOL193-12;Coleoptera;Trypodendron lineatum;Trypodendron lineatum;0.002;Trypodendron lineatum;0.999999913940716;
SCOL203-12;Coleoptera;Trypodendron lineatum;Trypodendron lineatum;0.024;Trypodendron lineatum;0.999999941982908;
SCOL219-12;Coleoptera;Xyleborinus saxesenii;Xyleborinus saxeseni;1;Xyleborinus saxeseni;0.999999948542326;
SCOL271-12;Coleoptera;Ips typographus;NA;0.043;Ips typographus;0.999923923145452;
SCOL277-12;Coleoptera;Trypodendron lineatum;Trypodendron lineatum;0.024;Trypodendron lineatum;0.999999376350696;
SCOL301-12;Coleoptera;Trypodendron domesticum;Trypodendron domesticum;1;Trypodendron domesticum;0.999999999992838;
SCOL313-12;Coleoptera;Ips typographus;NA;NA;Ips typographus;0.999976345733228;
SCOL324-12;Coleoptera;Trypodendron lineatum;Trypodendron lineatum;0.027;Trypodendron lineatum;0.999999740451448;
SICOC487-18;Coleoptera;Ergates faber;Trichocnemis spiculatus;1;Trichocnemis spiculatus;0.999999999999957;
TDAAT012-19;Coleoptera;Tillus elongatus;NA;NA;Tillus elongatus;1;
TDAAT1086-20;Coleoptera;Plagionotus arcuatus;Plagionotus arcuatus;1;Plagionotus arcuatus;0.999999982287932;

TDAAT1088-20;Coleoptera;Valgus hemipterus;Valgus hemipterus;1;Valgus hemipterus;1;
TDAAT1101-20;Coleoptera;Leiopus nebulosus;NA;0.042;Leiopus linnei;0.921884459848507;
TDAAT1116-20;Coleoptera;Ptilinus pectinicornis;Ptilinus pectinicornis;1;Ptilinus pectinicornis;1;
TDAAT1118-20;Coleoptera;Xylotrechus antilope;Xylotrechus antilope;1;Xylotrechus antilope;1;
TDAAT1120-20;Coleoptera;Exocentrus adspersus;Exocentrus adspersus;1;Exocentrus adspersus;1;
TDAAT1121-20;Coleoptera;Axinopalpis gracilis;NA;NA;Axinopalpis gracilis;1;
TDAAT1223-20;Coleoptera;Platystomos albinus;NA;NA;Platystomos albinus;1;
TDAAT1244-20;Coleoptera;Grammoptera ruficornis;Grammoptera ruficornis;1;Grammoptera ruficornis;1;
TDAAT1526-20;Coleoptera;Grammoptera ruficornis;NA;NA;Grammoptera ruficornis;0.999999999999289;
TDAAT1534-20;Coleoptera;Exocentrus adspersus;Exocentrus adspersus;1;Exocentrus adspersus;1;
TDAAT1564-20;Coleoptera;Anthaxia helvetica;Anthaxia helvetica;1;Anthaxia helvetica;1;
TDAAT1565-20;Coleoptera;Anthaxia quadripunctata;Anthaxia quadripunctata;1;Anthaxia quadripunctata;1;
TDAAT1567-20;Coleoptera;Anthaxia morio;Anthaxia morio;1;Anthaxia morio;1;
TDAAT1614-20;Coleoptera;Bitoma crenata;Bitoma crenata;1;Bitoma crenata;1;
TDAAT1738-20;Coleoptera;Anthaxia helvetica;Anthaxia helvetica;1;Anthaxia helvetica;1;
TDAAT1744-20;Coleoptera;Anthaxia quadripunctata;Anthaxia quadripunctata;1;Anthaxia quadripunctata;1;
TDAAT1921-20;Coleoptera;Gnorimus nobilis;Gnorimus nobilis;1;Gnorimus nobilis;1;
TDAAT1927-20;Coleoptera;Tillus elongatus;Tillus elongatus;1;Tillus elongatus;0.99999999999784;
TDAAT950-20;Coleoptera;Valgus hemipterus;Valgus hemipterus;NA;Valgus hemipterus;1;
TDAAT981-20;Coleoptera;Anthaxia podolica;Anthaxia podolica;1;Anthaxia podolica;1;
TDAAT982-20;Coleoptera;Agrilus viridis;Agrilus viridis;1;Agrilus viridis;0.999999877940049;
TDAAT983-20;Coleoptera;Ptilinus pectinicornis;Ptilinus pectinicornis;1;Ptilinus pectinicornis;1;
TDAOE185-21;Coleoptera;Grammoptera ruficornis;Grammoptera ruficornis;1;Grammoptera ruficornis;0.999999999999545;
TDAOE214-21;Coleoptera;Uloma rufa;Uloma rufa;1;Uloma rufa;1;
TDAOE356-21;Coleoptera;Valgus hemipterus;Valgus hemipterus;1;Valgus hemipterus;1;
ULMCA001-19;Coleoptera;Glischrochilus quadriguttatus;Glischrochilus quadriguttatus;1;Glischrochilus quadriguttatus;1;
ULMCA009-19;Coleoptera;Epuraea unicolor;Epuraea unicolor;1;Epuraea unicolor;0.999999999999943;
ULMCA012-19;Coleoptera;Glischrochilus quadriguttatus;Glischrochilus quadriguttatus;1;Glischrochilus quadriguttatus;1;
ZMBN134-16;Coleoptera;Leptusa pulchella;Leptusa pulchella;1;Leptusa pulchella;1;
ZMBN761-17;Coleoptera;Anthaxia quadripunctata;Anthaxia quadripunctata;1;Anthaxia quadripunctata;0.999999998992223;
ABEE003-17;Hymenoptera;Andrena hattorfiana;Andrena hattorfiana;1;Andrena hattorfiana;1;
ABEE026-17;Hymenoptera;Halictus quadricinctus;Halictus quadricinctus;1;Halictus quadricinctus;1;
ABEE080-17;Hymenoptera;Sphecodes cristatus;Sphecodes cristatus;1;Sphecodes cristatus;0.999999999999204;
ABEE103-17;Hymenoptera;Andrena hattorfiana;Andrena hattorfiana;1;Andrena hattorfiana;1;
ABEE104-17;Hymenoptera;Halictus quadricinctus;Halictus quadricinctus;1;Halictus quadricinctus;0.999999999999716;
ABEE140-17;Hymenoptera;Andrena ovatula;Andrena ovatula;1;Andrena ovatula;0.999999996163041;
ABEE162-17;Hymenoptera;Andrena hattorfiana;Andrena hattorfiana;1;Andrena hattorfiana;1;
ABEE225-17;Hymenoptera;Lasioglossum clypeare;Lasioglossum clypeare;1;Lasioglossum clypeare;0.99999999976194;
ACUFI035-12;Hymenoptera;Andrena argentata;Andrena barbilabris;1;Andrena barbilabris;0.999999038526206;
AF104653;Hymenoptera;Lasioglossum majus;Lasioglossum majus;1;Lasioglossum majus;0.999307085647598;
BBBO067-10;Hymenoptera;Bombus alpinus;Bombus alpinus;1;Bombus alpinus;0.999999963317294;
BEEEE116-15;Hymenoptera;Bombus monticola;Bombus monticola;1;Bombus monticola;0.999720698703334;
BEEEE130-15;Hymenoptera;Lasioglossum laevigatum;Lasioglossum laevigatum;1;Lasioglossum laevigatum;0.999999999999972;
BEEEE139-15;Hymenoptera;Colletes succinctus;NA;NA;NA;0.999999999998211;
BEEEE144-15;Hymenoptera;Andrena ovatula;Andrena ovatula;1;Andrena ovatula;0.999999698955139;
BEEEE183-15;Hymenoptera;Lasioglossum laevigatum;Lasioglossum laevigatum;1;Lasioglossum laevigatum;1;
BEEEE208-15;Hymenoptera;Andrena hattorfiana;Andrena hattorfiana;1;Andrena hattorfiana;1;
BEEEE212-15;Hymenoptera;Lasioglossum xanthopus;Lasioglossum xanthopus;1;Lasioglossum xanthopus;0.999999999984652;
BEEEE216-15;Hymenoptera;Colletes fodiens;Colletes fodiens;1;Colletes fodiens;0.996980773551553;
BEEEE235-15;Hymenoptera;Colletes succinctus;NA;NA;NA;0.999999999997973;
BEEEE237-15;Hymenoptera;Andrena ovatula;Andrena ovatula;1;Andrena ovatula;0.999999937493444;
BEEEE242-15;Hymenoptera;Sphecodes hyalinatus;Sphecodes hyalinatus;0.003;Sphecodes hyalinatus;0.999557194637488;
BEEEE245-15;Hymenoptera;Melitta tricincta;Melitta tricincta;NA;Melitta tricincta;0.999999999999858;
BEEEE252-15;Hymenoptera;Epeolus cruciger;NA;0.048;Epeolus cruciger;0.975988992906172;
BEEEE311-16;Hymenoptera;Bombus monticola;Bombus monticola;1;Bombus monticola;0.998981344571331;
BEEEE312-16;Hymenoptera;Bombus monticola;Bombus monticola;1;Bombus monticola;0.998981344571331;
BEEEE335-16;Hymenoptera;Bombus monticola;Bombus monticola;1;Bombus monticola;0.998981344571331;
BOMNI066-13;Hymenoptera;Bombus alpinus;Bombus alpinus;1;Bombus alpinus;0.999999996600849;
BOWGF169-08;Hymenoptera;Sphecodes rubicundus;Sphecodes rubicundus;1;Sphecodes rubicundus;0.999999974458234;

EF028492;Hymenoptera;Colletes albomaculatus;Colletes nasutus;1;Colletes nasutus;0.998441153984403;
EF028495;Hymenoptera;Colletes collaris;Colletes collaris;1;Colletes collaris;0.99995031961704;
EF028504;Hymenoptera;Colletes fodiens;Colletes fodiens;1;Colletes fodiens;0.996966267414801;
EF028508;Hymenoptera;Colletes graeffei;Colletes graeffei;1;Colletes graeffei;0.999999624648253;
EF028525;Hymenoptera;Colletes succinctus;NA;NA;NA;0.999999999996005;
EF028527;Hymenoptera;Colletes wolfi;Colletes wolfi;1;Colletes wolfi;0.999999953084427;
EF218722;Hymenoptera;Colletes graeffei;Colletes graeffei;1;Colletes graeffei;0.999999624648253;
GMBUD1100-14;Hymenoptera;Lasioglossum angusticeps;Lasioglossum angusticeps;1;Lasioglossum angusticeps;0.999999999988546;
GMBUD183-14;Hymenoptera;Andrena ovatula;Andrena ovatula;1;Andrena ovatula;0.999999999478717;
GMBUE1926-14;Hymenoptera;Lasioglossum pygmaeum;Lasioglossum pygmaeum;1;Lasioglossum pygmaeum;0.999999998804384;
GMBUE2034-14;Hymenoptera;Lasioglossum angusticeps;Lasioglossum angusticeps;1;Lasioglossum angusticeps;0.999999999985903;
GMBUE2049-14;Hymenoptera;Lasioglossum angusticeps;Lasioglossum angusticeps;1;Lasioglossum angusticeps;0.999999999941565;
GMBUG400-14;Hymenoptera;Lasioglossum angusticeps;Lasioglossum angusticeps;1;Lasioglossum angusticeps;0.999999999971749;
GMGMA1092-14;Hymenoptera;Lasioglossum minutulum;Lasioglossum minutulum;1;Lasioglossum minutulum;1;
GMGMD182-14;Hymenoptera;Lasioglossum costulatum;Lasioglossum costulatum;1;Lasioglossum costulatum;1;
GMGMN298-14;Hymenoptera;Andrena hattorfiana;Andrena hattorfiana;1;Andrena hattorfiana;1;
GMRSK019-15;Hymenoptera;Colletes collaris;Colletes collaris;NA;Colletes collaris;0.999804101688157;
GMRSU159-15;Hymenoptera;Sphecodes hyalinatus;NA;0.069;Sphecodes hyalinatus;0.991349472308359;
GMRUB009-16;Hymenoptera;Colletes collaris;Colletes collaris;0.982;Colletes collaris;0.99996441429844;
GMRUB010-16;Hymenoptera;Colletes collaris;Colletes collaris;0.999;Colletes collaris;0.999943628042431;
GMRUB011-16;Hymenoptera;Colletes collaris;Colletes collaris;1;Colletes collaris;0.999926724150448;
GU705902;Hymenoptera;Bombus gerstaeckeri;Bombus gerstaeckeri;1;Bombus gerstaeckeri;0.999999996373589;
GU705913;Hymenoptera;Bombus monticola;Bombus monticola;1;Bombus monticola;0.99982316997635;
GU705914;Hymenoptera;Bombus mendax;Bombus mendax;1;Bombus mendax;0.99999999999406;
GU706050;Hymenoptera;Melitta tricincta;Melitta tricincta;NA;Melitta tricincta;1;
HM376238;Hymenoptera;Andrena hattorfiana;Andrena hattorfiana;1;Andrena hattorfiana;1;
HM401035;Hymenoptera;Sphecodes schenckii;Sphecodes schenckii;1;Sphecodes schenckii;0.999999904504169;
HM401038;Hymenoptera;Nomada armata;Nomada armata;1;Nomada armata;0.999999999724196;
HM401047;Hymenoptera;Andrena fulvida;Andrena fulvida;1;Andrena fulvida;0.99999999996146;
HM401093;Hymenoptera;Halictus quadricinctus;Halictus quadricinctus;1;Halictus quadricinctus;1;
HM401098;Hymenoptera;Lasioglossum angusticeps;Lasioglossum angusticeps;1;Lasioglossum angusticeps;0.999999999984624;
HM401100;Hymenoptera;Lasioglossum costulatum;Lasioglossum costulatum;1;Lasioglossum costulatum;0.999999999999915;
HM401151;Hymenoptera;Dufourea dentiventris;Dufourea dentiventris;1;Dufourea dentiventris;1;
HM401156;Hymenoptera;Colletes fodiens;Colletes fodiens;1;Colletes fodiens;0.997405602727073;
HM401180;Hymenoptera;Hylaeus pfankuchi;Hylaeus pfankuchi;1;Hylaeus pfankuchi;0.999722272576945;
HM401181;Hymenoptera;Hylaeus pfankuchi;Hylaeus pfankuchi;1;Hylaeus pfankuchi;0.999722272576945;
HM401182;Hymenoptera;Hylaeus pfankuchi;Hylaeus pfankuchi;NA;Hylaeus pfankuchi;0.999456633066967;
HM401183;Hymenoptera;Hylaeus pfankuchi;Hylaeus pfankuchi;1;Hylaeus pfankuchi;0.999238980743741;
HM401257;Hymenoptera;Lasioglossum pygmaeum;Lasioglossum pygmaeum;1;Lasioglossum pygmaeum;0.999999999938325;
HM401267;Hymenoptera;Bombus gerstaeckeri;Bombus gerstaeckeri;1;Bombus gerstaeckeri;0.999999996373589;
HM401268;Hymenoptera;Bombus gerstaeckeri;Bombus gerstaeckeri;1;Bombus gerstaeckeri;0.999999996373589;
HQ563095;Hymenoptera;Sphecodes rubicundus;Sphecodes rubicundus;1;Sphecodes rubicundus;0.999999981305081;
HQ563096;Hymenoptera;Sphecodes rubicundus;Sphecodes rubicundus;1;Sphecodes rubicundus;0.999999973263556;
HQ563099;Hymenoptera;Sphecodes schenckii;Sphecodes schenckii;1;Sphecodes schenckii;0.999999994175511;
HQ563101;Hymenoptera;Nomada argentata;Nomada argentata;1;Nomada argentata;0.999999917757382;
HQ563801;Hymenoptera;Bombus mendax;Bombus mendax;1;Bombus mendax;0.999999999995282;
HQ563802;Hymenoptera;Bombus mendax;Bombus mendax;1;Bombus mendax;0.99999999999406;
HQ948038;Hymenoptera;Nomada roberjeotiana;Nomada tormentillae;NA;Nomada tormentillae;0.990757204907176;
HQ948039;Hymenoptera;Nomada roberjeotiana;Nomada tormentillae;NA;Nomada tormentillae;0.996173290371019;
HQ948040;Hymenoptera;Nomada mutica;Nomada mutica;1;Nomada mutica;0.99999918674023;
HQ948051;Hymenoptera;Lasioglossum angusticeps;Lasioglossum angusticeps;1;Lasioglossum angusticeps;0.999999999996845;
HQ948052;Hymenoptera;Lasioglossum brevicorne;NA;0.323;Lasioglossum brevicorne;0.999999995313345;

HQ948053;Hymenoptera;Lasioglossum brevicorne;Lasioglossum brevicorne;1;Lasioglossum brevicorne;0.999999999104915;
HQ948059;Hymenoptera;Epeolus cruciger;Epeolus cruciger;1;Epeolus cruciger;0.999989764568256;
HQ948069;Hymenoptera;Melitta tricincta;Melitta tricincta;NA;Melitta tricincta;0.999999999999829;
HQ948113;Hymenoptera;Lasioglossum quadrinotatulum;Lasioglossum quadrinotatulum;1;Lasioglossum quadrinotatulum;0.999999999999886;
HQ948115;Hymenoptera;Colletes floralis;Colletes floralis;1;Colletes floralis;0.999999985844284;
HQ948116;Hymenoptera;Colletes floralis;Colletes floralis;1;Colletes floralis;0.999999971142075;
HQ948117;Hymenoptera;Colletes floralis;Colletes floralis;1;Colletes floralis;0.99999998327894;
HQ948121;Hymenoptera;Bombus alpinus;Bombus alpinus;1;Bombus alpinus;0.999999926677336;
HQ954752;Hymenoptera;Lasioglossum laevigatum;Lasioglossum laevigatum;1;Lasioglossum laevigatum;1;
HQ954754;Hymenoptera;Lasioglossum majus;Lasioglossum majus;1;Lasioglossum majus;0.999321175089311;
HQ954759;Hymenoptera;Lasioglossum quadrinotatulum;Lasioglossum quadrinotatulum;1;Lasioglossum quadrinotatulum;0.999999999998721;
HQ954760;Hymenoptera;Lasioglossum quadrinotatulum;Lasioglossum quadrinotatulum;1;Lasioglossum quadrinotatulum;0.999999999999176;
HQ954766;Hymenoptera;Andrena fulvida;Andrena fulvida;1;Andrena fulvida;0.999999999935;
HYMAA253-21;Hymenoptera;Andrena ovatula;Andrena ovatula;1;Andrena ovatula;0.999993325987407;
HYMAA254-21;Hymenoptera;Andrena ovatula;Andrena ovatula;1;Andrena ovatula;0.999999780094096;
JQ909690;Hymenoptera;Andrena ovatula;Andrena ovatula;1;Andrena ovatula;0.999999869136852;
JQ909712;Hymenoptera;Colletes floralis;Colletes floralis;1;Colletes floralis;0.999999996590304;
JQ909713;Hymenoptera;Colletes floralis;Colletes floralis;1;Colletes floralis;0.999999996590304;
JQ909714;Hymenoptera;Colletes floralis;Colletes floralis;1;Colletes floralis;0.999999996590304;
JQ909715;Hymenoptera;Colletes floralis;Colletes floralis;1;Colletes floralis;0.999999996590304;
JQ909721;Hymenoptera;Colletes succinctus;NA;NA;NA;0.999999999949464;
JQ909722;Hymenoptera;Colletes succinctus;NA;NA;NA;0.999999999999541;
JQ909723;Hymenoptera;Colletes succinctus;NA;NA;NA;0.99999999999886;
JQ909724;Hymenoptera;Colletes succinctus;NA;NA;NA;0.999999999949464;
JQ909874;Hymenoptera;Sphecodes hyalinatus;Sphecodes hyalinatus;0.013;Sphecodes hyalinatus;0.989257894811799;
JQ909875;Hymenoptera;Sphecodes hyalinatus;NA;0.024;Sphecodes hyalinatus;0.963725437528646;
JQ909876;Hymenoptera;Sphecodes hyalinatus;Sphecodes hyalinatus;0.013;Sphecodes hyalinatus;0.989257894811799;
JQ909877;Hymenoptera;Sphecodes hyalinatus;Sphecodes hyalinatus;0.013;Sphecodes hyalinatus;0.989257894811799;
JQ909878;Hymenoptera;Sphecodes hyalinatus;Sphecodes hyalinatus;0.013;Sphecodes hyalinatus;0.989257894811799;
JX256660;Hymenoptera;Sphecodes schenckii;Sphecodes schenckii;0.002;Sphecodes schenckii;0.999366092568888;
JX256667;Hymenoptera;Sphecodes cristatus;Sphecodes cristatus;1;Sphecodes cristatus;0.999998851670659;
JX256703;Hymenoptera;Sphecodes majalis;Sphecodes majalis;1;Sphecodes majalis;0.99999792053916;
JX256721;Hymenoptera;Sphecodes spinulosus;Sphecodes spinulosus;1;Sphecodes spinulosus;0.999999814916424;
JX256722;Hymenoptera;Sphecodes spinulosus;Sphecodes spinulosus;1;Sphecodes spinulosus;0.999993801749408;
JX256725;Hymenoptera;Sphecodes rubicundus;Sphecodes rubicundus;1;Sphecodes rubicundus;0.999996646819278;
KF434334;Hymenoptera;Bombus monticola;Bombus monticola;1;Bombus monticola;0.99978633943506;
KF434335;Hymenoptera;Bombus monticola;Bombus monticola;1;Bombus monticola;0.99992968678245;
KF434336;Hymenoptera;Bombus monticola;Bombus monticola;1;Bombus monticola;0.99992968678245;
KF434337;Hymenoptera;Bombus monticola;Bombus monticola;1;Bombus monticola;0.99992968678245;
KF434338;Hymenoptera;Bombus monticola;Bombus monticola;1;Bombus monticola;0.99978633943506;
KF434339;Hymenoptera;Bombus monticola;Bombus monticola;1;Bombus monticola;0.99992968678245;
KF434340;Hymenoptera;Bombus monticola;Bombus monticola;1;Bombus monticola;0.99992968678245;
KF434341;Hymenoptera;Bombus monticola;Bombus monticola;1;Bombus monticola;0.99978633943506;
KJ836415;Hymenoptera;Sphecodes croaticus;Sphecodes croaticus;1;Sphecodes croaticus;0.999998146129527;
KJ836433;Hymenoptera;Sphecodes croaticus;Sphecodes croaticus;1;Sphecodes croaticus;0.999638472217986;
KJ836467;Hymenoptera;Nomada villosa;NA;NA;Nomada striata;0.894506833279756;
KJ836474;Hymenoptera;Epeolus cruciger;Epeolus cruciger;1;Epeolus cruciger;0.999991025773648;
KJ836480;Hymenoptera;Halictus leucaheneus;Halictus leucaheneus;1;Halictus leucaheneus;1;
KJ836497;Hymenoptera;Lasioglossum costulatum;Lasioglossum costulatum;1;Lasioglossum costulatum;1;
KJ836503;Hymenoptera;Dufourea dentiventris;Dufourea dentiventris;1;Dufourea dentiventris;1;
KJ836586;Hymenoptera;Halictus quadricinctus;Halictus quadricinctus;1;Halictus quadricinctus;1;
KJ836601;Hymenoptera;Colletes fodiens;Colletes fodiens;1;Colletes fodiens;0.995561860742147;
KJ836607;Hymenoptera;Melitturga clavicornis;Melitturga clavicornis;1;Melitturga clavicornis;1;
KJ836625;Hymenoptera;Lasioglossum xanthopus;Lasioglossum xanthopus;1;Lasioglossum xanthopus;0.999999999996703;
KJ836647;Hymenoptera;Sphecodes croaticus;Sphecodes croaticus;1;Sphecodes croaticus;0.999626107200707;
KJ836696;Hymenoptera;Nomada villosa;NA;NA;Nomada striata;0.894506833279756;
KJ836750;Hymenoptera;Epeolus cruciger;NA;0.16;Epeolus cruciger;0.968204513358466;

KJ836759;Hymenoptera;Lasioglossum costulatum;Lasioglossum costulatum;1;Lasioglossum costulatum;1;
KJ836762;Hymenoptera;Sphecodes schenckii;Sphecodes schenckii;1;Sphecodes schenckii;0.999999987608362;
KJ836795;Hymenoptera;Bombus confusus;Bombus confusus;1;Bombus confusus;0.999999998595996;
KJ836805;Hymenoptera;Colletes fodiens;Colletes fodiens;1;Colletes fodiens;0.998407648597494;
KJ836819;Hymenoptera;Systropha curvicornis;NA;NA;Systropha curvicornis;1;
KJ836821;Hymenoptera;Halictus leucaheneus;Halictus leucaheneus;1;Halictus leucaheneus;1;
KJ836829;Hymenoptera;Halictus quadricinctus;Halictus quadricinctus;1;Halictus quadricinctus;1;
KJ836861;Hymenoptera;Halictus quadricinctus;Halictus quadricinctus;1;Halictus quadricinctus;1;
KJ836882;Hymenoptera;Nomada armata;Nomada armata;1;Nomada armata;0.99999999960724;
KJ836900;Hymenoptera;Lasioglossum pygmaeum;Lasioglossum pygmaeum;1;Lasioglossum pygmaeum;1;
KJ836931;Hymenoptera;Lasioglossum pygmaeum;Lasioglossum pygmaeum;1;Lasioglossum pygmaeum;0.999999999999972;
KJ836940;Hymenoptera;Halictus quadricinctus;Halictus quadricinctus;1;Halictus quadricinctus;1;
KJ836971;Hymenoptera;Lasioglossum pygmaeum;Lasioglossum pygmaeum;1;Lasioglossum pygmaeum;0.999999999999972;
KJ836978;Hymenoptera;Sphecodes schenckii;Sphecodes schenckii;1;Sphecodes schenckii;0.999999914431076;
KJ836981;Hymenoptera;Andrena hattorfiana;Andrena hattorfiana;1;Andrena hattorfiana;1;
KJ836993;Hymenoptera;Melitturga clavicornis;Melitturga clavicornis;1;Melitturga clavicornis;1;
KJ837051;Hymenoptera;Sphecodes majalis;Sphecodes majalis;1;Sphecodes majalis;1;
KJ837055;Hymenoptera;Halictus quadricinctus;Halictus quadricinctus;1;Halictus quadricinctus;1;
KJ837071;Hymenoptera;Nomada argentata;Nomada argentata;1;Nomada argentata;0.999999793404861;
KJ837086;Hymenoptera;Lasioglossum costulatum;Lasioglossum costulatum;1;Lasioglossum costulatum;1;
KJ837113;Hymenoptera;Anthophora balneorum;Anthophora balneorum;1;Anthophora balneorum;1;
KJ837131;Hymenoptera;Bombus monticola;Bombus monticola;1;Bombus monticola;0.99982316997635;
KJ837145;Hymenoptera;Nomada armata;Nomada armata;1;Nomada armata;0.99999999960724;
KJ837146;Hymenoptera;Dufourea dentiventris;Dufourea dentiventris;1;Dufourea dentiventris;1;
KJ837151;Hymenoptera;Dufourea minuta;Dufourea minuta;1;Dufourea minuta;1;
KJ837195;Hymenoptera;Halictus quadricinctus;Halictus quadricinctus;1;Halictus quadricinctus;1;
KJ837235;Hymenoptera;Sphecodes croaticus;Sphecodes croaticus;1;Sphecodes croaticus;0.999626107200707;
KJ837294;Hymenoptera;Sphecodes hyalinatus;Sphecodes hyalinatus;1;Sphecodes hyalinatus;0.999840844068618;
KJ837312;Hymenoptera;Dufourea minuta;Dufourea minuta;1;Dufourea minuta;1;
KJ837357;Hymenoptera;Lasioglossum costulatum;Lasioglossum costulatum;1;Lasioglossum costulatum;1;
KJ837367;Hymenoptera;Dufourea dentiventris;Dufourea dentiventris;1;Dufourea dentiventris;1;
KJ837407;Hymenoptera;Andrena hattorfiana;Andrena hattorfiana;1;Andrena hattorfiana;1;
KJ837418;Hymenoptera;Halictus leucaheneus;Halictus leucaheneus;1;Halictus leucaheneus;1;
KJ837424;Hymenoptera;Andrena hattorfiana;Andrena hattorfiana;1;Andrena hattorfiana;1;
KJ837444;Hymenoptera;Dufourea inermis;Dufourea inermis;1;Dufourea inermis;1;
KJ837459;Hymenoptera;Andrena ovatula;Andrena ovatula;1;Andrena ovatula;0.999999999948159;
KJ837462;Hymenoptera;Lasioglossum sexnotatum;Lasioglossum sexnotatum;1;Lasioglossum sexnotatum;1;
KJ837472;Hymenoptera;Colletes succinctus;NA;NA;NA;0.999999999999249;
KJ837501;Hymenoptera;Bombus gerstaeckeri;Bombus gerstaeckeri;1;Bombus gerstaeckeri;0.999999996373589;
KJ837507;Hymenoptera;Nomada roberjeotiana;Nomada roberjeotiana;NA;Nomada roberjeotiana;0.999200188838259;
KJ837522;Hymenoptera;Andrena hattorfiana;Andrena hattorfiana;1;Andrena hattorfiana;1;
KJ837543;Hymenoptera;Bombus monticola;Bombus monticola;1;Bombus monticola;0.999853543369091;
KJ837571;Hymenoptera;Lasioglossum pygmaeum;Lasioglossum pygmaeum;1;Lasioglossum pygmaeum;0.999999999999943;
KJ837646;Hymenoptera;Sphecodes croaticus;Sphecodes croaticus;1;Sphecodes croaticus;0.999626107200707;
KJ837657;Hymenoptera;Sphecodes schenckii;Sphecodes schenckii;1;Sphecodes schenckii;0.999999987608362;
KJ837659;Hymenoptera;Lasioglossum intermedium;Lasioglossum semilucens;1;Lasioglossum semilucens;0.999968997288772;
KJ837664;Hymenoptera;Colletes fodiens;Colletes fodiens;1;Colletes fodiens;0.997405602727073;
KJ837682;Hymenoptera;Anthidium montanum;Anthidium montanum;1;Anthidium montanum;1;
KJ837685;Hymenoptera;Halictus quadricinctus;Halictus quadricinctus;1;Halictus quadricinctus;1;
KJ837694;Hymenoptera;Anthidium montanum;Anthidium montanum;1;Anthidium montanum;1;
KJ837759;Hymenoptera;Lasioglossum costulatum;Lasioglossum costulatum;1;Lasioglossum costulatum;1;
KJ837817;Hymenoptera;Nomada roberjeotiana;Nomada tormentillae;0.001;Nomada tormentillae;0.99441350524657;
KJ837889;Hymenoptera;Lasioglossum xanthopus;Lasioglossum xanthopus;1;Lasioglossum xanthopus;0.999999999996703;
KJ837905;Hymenoptera;Epeolus cruciger;Epeolus cruciger;0.006;Epeolus cruciger;0.999990871561691;
KJ837909;Hymenoptera;Sphecodes majalis;Sphecodes majalis;1;Sphecodes majalis;1;
KJ837921;Hymenoptera;Sphecodes hyalinatus;Sphecodes hyalinatus;0.003;Sphecodes hyalinatus;0.999309335480466;
KJ837937;Hymenoptera;Lasioglossum costulatum;Lasioglossum costulatum;1;Lasioglossum costulatum;1;

KJ837955;Hymenoptera;Dufourea minuta;Dufourea minuta;1;Dufourea minuta;1;
KJ837961;Hymenoptera;Lasioglossum clypeare;Lasioglossum clypeare;1;Lasioglossum clypeare;0.99999999976194;
KJ837980;Hymenoptera;Halictus quadricinctus;Halictus quadricinctus;1;Halictus quadricinctus;1;
KJ837984;Hymenoptera;Lasioglossum xanthopus;Lasioglossum xanthopus;1;Lasioglossum xanthopus;0.999999999996703;
KJ838013;Hymenoptera;Colletes fodiens;Colletes fodiens;1;Colletes fodiens;0.998407648597494;
KJ838016;Hymenoptera;Lasioglossum minutulum;Lasioglossum minutulum;1;Lasioglossum minutulum;1;
KJ838052;Hymenoptera;Epeolus cruciger;NA;0.444;Epeolus cruciger;0.97602694074939;
KJ838072;Hymenoptera;Lasioglossum costulatum;Lasioglossum costulatum;1;Lasioglossum costulatum;1;
KJ838083;Hymenoptera;Halictus quadricinctus;Halictus quadricinctus;1;Halictus quadricinctus;1;
KJ838091;Hymenoptera;Lasioglossum sexnotatum;Lasioglossum sexnotatum;1;Lasioglossum sexnotatum;1;
KJ838094;Hymenoptera;Colletes fodiens;Colletes fodiens;1;Colletes fodiens;0.996818485731277;
KJ838122;Hymenoptera;Andrena ovatula;Andrena ovatula;1;Andrena ovatula;0.999999999948159;
KJ838139;Hymenoptera;Lasioglossum sexnotatum;Lasioglossum sexnotatum;1;Lasioglossum sexnotatum;0.999999999999972;
KJ838146;Hymenoptera;Epeolus cruciger;NA;0.113;Epeolus cruciger;0.989220250066161;
KJ838166;Hymenoptera;Colletes succinctus;Colletes succinctus;NA;Colletes succinctus;0.998404218350284;
KJ838182;Hymenoptera;Halictus leucaheneus;Halictus leucaheneus;1;Halictus leucaheneus;1;
KJ838212;Hymenoptera;Lasioglossum intermedium;Lasioglossum intermedium;1;Lasioglossum intermedium;0.999999999999346;
KJ838262;Hymenoptera;Lasioglossum intermedium;Lasioglossum intermedium;1;Lasioglossum intermedium;0.999999999999176;
KJ838272;Hymenoptera;Halictus leucaheneus;Halictus leucaheneus;1;Halictus leucaheneus;1;
KJ838279;Hymenoptera;Lasioglossum sexnotatum;Lasioglossum sexnotatum;1;Lasioglossum sexnotatum;1;
KJ838299;Hymenoptera;Lasioglossum sexnotatum;Lasioglossum sexnotatum;1;Lasioglossum sexnotatum;1;
KJ838315;Hymenoptera;Colletes floralis;Colletes floralis;1;Colletes floralis;0.999999992581081;
KJ838327;Hymenoptera;Lasioglossum subfasciatum;Lasioglossum subfasciatum;1;Lasioglossum subfasciatum;1;
KJ838335;Hymenoptera;Lasioglossum pygmaeum;Lasioglossum pygmaeum;1;Lasioglossum pygmaeum;0.999999999999972;
KJ838347;Hymenoptera;Andrena ovatula;Andrena ovatula;1;Andrena ovatula;0.999999999906834;
KJ838349;Hymenoptera;Bombus monticola;Bombus monticola;1;Bombus monticola;0.99982316997635;
KJ838355;Hymenoptera;Andrena hattorfiana;Andrena hattorfiana;1;Andrena hattorfiana;1;
KJ838360;Hymenoptera;Nomada argentata;Nomada argentata;1;Nomada argentata;0.999999113245446;
KJ838365;Hymenoptera;Epeolus cruciger;Epeolus cruciger;0.007;Epeolus cruciger;0.999740282277555;
KJ838382;Hymenoptera;Halictus leucaheneus;Halictus leucaheneus;1;Halictus leucaheneus;1;
KJ838387;Hymenoptera;Lasioglossum costulatum;Lasioglossum costulatum;1;Lasioglossum costulatum;1;
KJ838397;Hymenoptera;Andrena ovatula;Andrena ovatula;1;Andrena ovatula;0.999999999973568;
KJ838401;Hymenoptera;Colletes floralis;Colletes floralis;1;Colletes floralis;0.999999992581081;
KJ838431;Hymenoptera;Melitturga clavicornis;Melitturga clavicornis;1;Melitturga clavicornis;1;
KJ838435;Hymenoptera;Colletes fodiens;Colletes fodiens;1;Colletes fodiens;0.996818485731277;
KJ838456;Hymenoptera;Bombus monticola;Bombus monticola;1;Bombus monticola;0.99982316997635;
KJ838494;Hymenoptera;Lasioglossum subfasciatum;Lasioglossum subfasciatum;1;Lasioglossum subfasciatum;0.999999999999915;
KJ838502;Hymenoptera;Dasypoda suripes;Dasypoda suripes;1;Dasypoda suripes;1;
KJ838510;Hymenoptera;Halictus quadricinctus;Halictus quadricinctus;1;Halictus quadricinctus;1;
KJ838551;Hymenoptera;Colletes fodiens;Colletes fodiens;1;Colletes fodiens;0.997405602727073;
KJ838554;Hymenoptera;Sphecodes majalis;Sphecodes majalis;1;Sphecodes majalis;1;
KJ838566;Hymenoptera;Halictus leucaheneus;Halictus leucaheneus;1;Halictus leucaheneus;1;
KJ838574;Hymenoptera;Halictus quadricinctus;Halictus quadricinctus;1;Halictus quadricinctus;1;
KJ838610;Hymenoptera;Dufourea minuta;Dufourea minuta;1;Dufourea minuta;1;
KJ838622;Hymenoptera;Dufourea dentiventris;Dufourea dentiventris;1;Dufourea dentiventris;1;
KJ838657;Hymenoptera;Colletes graeffei;Colletes graeffei;1;Colletes graeffei;0.999994471118605;
KJ838659;Hymenoptera;Andrena hattorfiana;Andrena hattorfiana;1;Andrena hattorfiana;1;
KJ838670;Hymenoptera;Halictus quadricinctus;Halictus quadricinctus;1;Halictus quadricinctus;1;
KJ838713;Hymenoptera;Nomada mutica;Nomada mutica;1;Nomada mutica;0.999999788779314;
KJ838749;Hymenoptera;Melitta tricincta;Melitta tricincta;NA;Melitta tricincta;1;
KJ838754;Hymenoptera;Lasioglossum marginellum;Lasioglossum marginellum;1;Lasioglossum marginellum;0.999999999966604;
KJ838761;Hymenoptera;Halictus quadricinctus;Halictus quadricinctus;1;Halictus quadricinctus;1;
KJ838797;Hymenoptera;Bombus confusus;Bombus confusus;1;Bombus confusus;0.999999998595996;
KJ838805;Hymenoptera;Sphecodes hyalinatus;Sphecodes ferruginatus;1;Sphecodes ferruginatus;0.997700529625535;
KJ838806;Hymenoptera;Lasioglossum marginellum;Lasioglossum marginellum;1;Lasioglossum marginellum;0.999999999989882;
KJ838824;Hymenoptera;Sphecodes hyalinatus;Sphecodes hyalinatus;1;Sphecodes hyalinatus;0.999905579082925;
KJ838837;Hymenoptera;Nomada roberjeotiana;Nomada roberjeotiana;NA;Nomada roberjeotiana;0.995138562210576;
KJ838844;Hymenoptera;Colletes fodiens;Colletes fodiens;1;Colletes fodiens;0.999099301831391;
KJ838873;Hymenoptera;Dufourea minuta;Dufourea minuta;1;Dufourea minuta;1;

KJ838918;Hymenoptera;Colletes fodiens;Colletes fodiens;1;Colletes fodiens;0.998407648597494;
KJ838972;Hymenoptera;Sphecodes hyalinatus;Sphecodes hyalinatus;0.004;Sphecodes hyalinatus;0.997030930666154;
KJ839017;Hymenoptera;Bombus mucidus;Bombus mucidus;1;Bombus mucidus;0.999986904807707;
KJ839032;Hymenoptera;Andrena fulvida;Andrena fulvida;1;Andrena fulvida;0.999999999969646;
KJ839051;Hymenoptera;Sphecodes schenckii;Sphecodes schenckii;1;Sphecodes schenckii;0.999999987608362;
KJ839066;Hymenoptera;Halictus leucaheneus;Halictus leucaheneus;1;Halictus leucaheneus;1;
KJ839075;Hymenoptera;Halictus leucaheneus;Halictus leucaheneus;1;Halictus leucaheneus;1;
KJ839082;Hymenoptera;Halictus quadricinctus;Halictus quadricinctus;1;Halictus quadricinctus;1;
KJ839125;Hymenoptera;Halictus quadricinctus;Halictus quadricinctus;1;Halictus quadricinctus;1;
KJ839146;Hymenoptera;Anthidium montanum;Anthidium montanum;1;Anthidium montanum;1;
KJ839149;Hymenoptera;Lasioglossum majus;Lasioglossum majus;1;Lasioglossum majus;0.999337899533515;
KJ839169;Hymenoptera;Lasioglossum costulatum;Lasioglossum costulatum;1;Lasioglossum costulatum;1;
KJ839220;Hymenoptera;Lasioglossum costulatum;Lasioglossum costulatum;1;Lasioglossum costulatum;1;
KJ839232;Hymenoptera;Epeolus cruciger;NA;0.444;Epeolus cruciger;0.97602694074939;
KJ839248;Hymenoptera;Lasioglossum costulatum;Lasioglossum costulatum;1;Lasioglossum costulatum;1;
KJ839257;Hymenoptera;Epeolus cruciger;Epeolus cruciger;1;Epeolus cruciger;0.999991025773648;
KJ839281;Hymenoptera;Nomada roberjeotiana;Nomada roberjeotiana;NA;Nomada roberjeotiana;0.998244023166367;
KJ839283;Hymenoptera;Lasioglossum sexnotatum;Lasioglossum sexnotatum;1;Lasioglossum sexnotatum;1;
KJ839292;Hymenoptera;Halictus quadricinctus;Halictus quadricinctus;1;Halictus quadricinctus;1;
KJ839303;Hymenoptera;Andrena ovatula;Andrena ovatula;1;Andrena ovatula;0.999999996087297;
KJ839342;Hymenoptera;Anthophora balneorum;Anthophora balneorum;1;Anthophora balneorum;1;
KJ839343;Hymenoptera;Halictus quadricinctus;Halictus quadricinctus;1;Halictus quadricinctus;1;
KJ839352;Hymenoptera;Andrena ovatula;Andrena ovatula;1;Andrena ovatula;0.999999999948159;
KJ839357;Hymenoptera;Sphecodes hyalinatus;Sphecodes hyalinatus;0.002;Sphecodes hyalinatus;0.999725249832498;
KJ839367;Hymenoptera;Colletes fodiens;Colletes fodiens;1;Colletes fodiens;0.997718007726496;
KJ839403;Hymenoptera;Lasioglossum minutulum;Lasioglossum minutulum;1;Lasioglossum minutulum;1;
KJ839413;Hymenoptera;Lasioglossum costulatum;Lasioglossum costulatum;1;Lasioglossum costulatum;1;
KJ839438;Hymenoptera;Sphecodes hyalinatus;Sphecodes hyalinatus;1;Sphecodes hyalinatus;0.999684471272531;
KJ839448;Hymenoptera;Nomada symphyti;Nomada symphyti;0.001;Nomada symphyti;0.83870428014373;
KJ839457;Hymenoptera;Halictus quadricinctus;Halictus quadricinctus;1;Halictus quadricinctus;1;
KJ839462;Hymenoptera;Epeolus cruciger;Epeolus cruciger;1;Epeolus cruciger;0.999987310741053;
KJ839467;Hymenoptera;Andrena hattorfiana;Andrena hattorfiana;1;Andrena hattorfiana;1;
KJ839488;Hymenoptera;Lasioglossum costulatum;Lasioglossum costulatum;1;Lasioglossum costulatum;1;
KJ839506;Hymenoptera;Dufourea dentiventris;Dufourea dentiventris;1;Dufourea dentiventris;1;
KJ839509;Hymenoptera;Andrena fulvida;Andrena fulvida;1;Andrena fulvida;0.999999991289314;
KJ839532;Hymenoptera;Epeolus cruciger;NA;0.053;Epeolus cruciger;0.98986705678984;
KJ839568;Hymenoptera;Andrena hattorfiana;Andrena hattorfiana;1;Andrena hattorfiana;1;
KJ839581;Hymenoptera;Halictus quadricinctus;Halictus quadricinctus;1;Halictus quadricinctus;1;
KJ839600;Hymenoptera;Sphecodes croaticus;Sphecodes croaticus;1;Sphecodes croaticus;0.999626107200707;
KJ839604;Hymenoptera;Lasioglossum xanthopus;Lasioglossum xanthopus;1;Lasioglossum xanthopus;0.999999999994827;
KJ839614;Hymenoptera;Colletes graeffei;Colletes graeffei;1;Colletes graeffei;0.999997885499217;
KJ839622;Hymenoptera;Lasioglossum quadrinotatulum;Lasioglossum quadrinotatulum;1;Lasioglossum quadrinotatulum;0.999999999999773;
KJ839643;Hymenoptera;Lasioglossum pygmaeum;Lasioglossum pygmaeum;1;Lasioglossum pygmaeum;0.999999999999972;
KJ839683;Hymenoptera;Halictus quadricinctus;Halictus quadricinctus;1;Halictus quadricinctus;1;
KJ839709;Hymenoptera;Lasioglossum costulatum;Lasioglossum costulatum;1;Lasioglossum costulatum;1;
KJ839712;Hymenoptera;Sphecodes schenckii;Sphecodes schenckii;1;Sphecodes schenckii;0.999999993159065;
KJ839748;Hymenoptera;Colletes fodiens;Colletes fodiens;1;Colletes fodiens;0.997405602727073;
KJ839760;Hymenoptera;Sphecodes hyalinatus;Sphecodes hyalinatus;0.013;Sphecodes hyalinatus;0.994070562207568;
KJ839765;Hymenoptera;Colletes fodiens;Colletes fodiens;1;Colletes fodiens;0.998407648597494;
KJ839776;Hymenoptera;Halictus leucaheneus;Halictus leucaheneus;1;Halictus leucaheneus;1;
KJ839801;Hymenoptera;Lasioglossum majus;Lasioglossum majus;1;Lasioglossum majus;0.999337899533515;
KJ839806;Hymenoptera;Andrena hattorfiana;Andrena hattorfiana;1;Andrena hattorfiana;1;
KJ839822;Hymenoptera;Melitturga clavicornis;Melitturga clavicornis;1;Melitturga clavicornis;1;
KJ839824;Hymenoptera;Lasioglossum subfasciatum;Lasioglossum subfasciatum;1;Lasioglossum subfasciatum;0.999999999999972;
KJ839830;Hymenoptera;Epeolus cruciger;Epeolus cruciger;0.999;Epeolus cruciger;0.999984954461876;
KT074041;Hymenoptera;Colletes fodiens;Colletes fodiens;1;Colletes fodiens;0.99729303447681;

KT334300;Hymenoptera;Bombus gerstaeckeri;Bombus gerstaeckeri;1;Bombus gerstaeckeri;0.999999995486064;
KX374788;Hymenoptera;Lasioglossum sexnotatum;Lasioglossum sexnotatum;1;Lasioglossum sexnotatum;1;
KX374792;Hymenoptera;Lasioglossum brevicorne;Lasioglossum brevicorne;1;Lasioglossum brevicorne;0.999999997608256;
KY317973;Hymenoptera;Bombus konradini;NA;NA;Bombus glacialis;0.915229021264775;
KY317975;Hymenoptera;Bombus monticola;Bombus monticola;0.002;Bombus glacialis;0.954328173808283;
KY317976;Hymenoptera;Bombus monticola;Bombus monticola;0.001;Bombus glacialis;0.971450548213942;
KY317977;Hymenoptera;Bombus monticola;Bombus monticola;0.001;Bombus glacialis;0.95553046739416;
KY317978;Hymenoptera;Bombus monticola;Bombus monticola;0.002;Bombus glacialis;0.945259030457934;
KY317979;Hymenoptera;Bombus monticola;Bombus monticola;0.005;Bombus glacialis;0.97163987978333;
KY317980;Hymenoptera;Bombus monticola;Bombus monticola;0.002;Bombus glacialis;0.954328173808283;
LASNA304-08;Hymenoptera;Lasioglossum xanthopus;Lasioglossum xanthopus;1;Lasioglossum xanthopus;0.999999999995936;
LASNA398-08;Hymenoptera;Lasioglossum xanthopus;Lasioglossum xanthopus;1;Lasioglossum xanthopus;0.999999999995936;
MG309777;Hymenoptera;Bombus mucidus;Bombus mucidus;1;Bombus mucidus;0.999989597318141;
MG846706;Hymenoptera;Lasioglossum laevigatum;Lasioglossum laevigatum;1;Lasioglossum laevigatum;0.999999999999915;
MK268705;Hymenoptera;Sphecodes hyalinatus;Sphecodes hyalinatus;1;Sphecodes hyalinatus;0.999357142058941;
MN342315;Hymenoptera;Epeolus tarsalis;Epeolus tarsalis;1;Epeolus tarsalis;0.999967462753482;
MN342316;Hymenoptera;Triepeolus tristis;Triepeolus tristis;1;Triepeolus tristis;0.999999999999289;
MN342321;Hymenoptera;Epeolus cruciger;Epeolus cruciger;1;Epeolus cruciger;0.999993974779177;
MT869002;Hymenoptera;Halictus quadricinctus;Halictus quadricinctus;1;Halictus quadricinctus;1;
MT869193;Hymenoptera;Halictus quadricinctus;Halictus quadricinctus;1;Halictus quadricinctus;1;
MT869239;Hymenoptera;Halictus quadricinctus;Halictus quadricinctus;1;Halictus quadricinctus;1;
MT869247;Hymenoptera;Halictus quadricinctus;Halictus quadricinctus;1;Halictus quadricinctus;1;
MT869460;Hymenoptera;Halictus quadricinctus;Halictus quadricinctus;1;Halictus quadricinctus;0.999999999999687;
MT869542;Hymenoptera;Halictus quadricinctus;Halictus quadricinctus;1;Halictus quadricinctus;1;
MT869551;Hymenoptera;Colletes fodiens;Colletes fodiens;1;Colletes fodiens;0.999261790595922;
MT869558;Hymenoptera;Halictus quadricinctus;Halictus quadricinctus;1;Halictus quadricinctus;1;
MT869605;Hymenoptera;Halictus quadricinctus;Halictus quadricinctus;1;Halictus quadricinctus;1;
MT869656;Hymenoptera;Lasioglossum pygmaeum;Lasioglossum pygmaeum;1;Lasioglossum pygmaeum;0.999999999999972;
MT869675;Hymenoptera;Halictus quadricinctus;Halictus quadricinctus;1;Halictus quadricinctus;1;
MT869719;Hymenoptera;Lasioglossum intermedium;Lasioglossum intermedium;1;Lasioglossum intermedium;0.999999999999744;
MT869798;Hymenoptera;Halictus quadricinctus;Halictus quadricinctus;1;Halictus quadricinctus;1;
MT869810;Hymenoptera;Halictus quadricinctus;Halictus quadricinctus;1;Halictus quadricinctus;1;
MT869939;Hymenoptera;Halictus quadricinctus;Halictus quadricinctus;1;Halictus quadricinctus;0.999999999999972;
MT869997;Hymenoptera;Andrena ovatula;Andrena albofasciata;1;Andrena albofasciata;0.999820113441725;
MT870176;Hymenoptera;Halictus quadricinctus;Halictus quadricinctus;1;Halictus quadricinctus;1;
MT870247;Hymenoptera;Halictus quadricinctus;Halictus quadricinctus;1;Halictus quadricinctus;1;
MT870277;Hymenoptera;Lasioglossum sexnotatum;Lasioglossum sexnotatum;1;Lasioglossum sexnotatum;1;
MT870298;Hymenoptera;Lasioglossum sexnotatum;Lasioglossum sexnotatum;1;Lasioglossum sexnotatum;1;
MT870325;Hymenoptera;Lasioglossum xanthopus;Lasioglossum xanthopus;1;Lasioglossum xanthopus;0.999999999996618;
MT870353;Hymenoptera;Halictus quadricinctus;Halictus quadricinctus;1;Halictus quadricinctus;1;
MT870361;Hymenoptera;Lasioglossum xanthopus;Lasioglossum xanthopus;1;Lasioglossum xanthopus;0.999999999996618;
MT870444;Hymenoptera;Lasioglossum sexnotatum;Lasioglossum sexnotatum;1;Lasioglossum sexnotatum;1;
MT870531;Hymenoptera;Halictus quadricinctus;Halictus quadricinctus;1;Halictus quadricinctus;1;
MT870680;Hymenoptera;Sphecodes majalis;Sphecodes majalis;1;Sphecodes majalis;1;
MT870742;Hymenoptera;Halictus quadricinctus;Halictus quadricinctus;1;Halictus quadricinctus;1;
MT870786;Hymenoptera;Lasioglossum sexnotatum;Lasioglossum sexnotatum;1;Lasioglossum sexnotatum;1;
MT870845;Hymenoptera;Halictus quadricinctus;Halictus quadricinctus;1;Halictus quadricinctus;1;
MT870991;Hymenoptera;Lasioglossum sexnotatum;Lasioglossum sexnotatum;1;Lasioglossum sexnotatum;1;
MT871060;Hymenoptera;Lasioglossum majus;Lasioglossum majus;1;Lasioglossum majus;0.99927239707714;
MT871075;Hymenoptera;Halictus quadricinctus;Halictus quadricinctus;1;Halictus quadricinctus;1;
MT871127;Hymenoptera;Halictus quadricinctus;Halictus quadricinctus;1;Halictus quadricinctus;1;
MT871182;Hymenoptera;Lasioglossum sexnotatum;Lasioglossum sexnotatum;1;Lasioglossum sexnotatum;1;
MT871225;Hymenoptera;Halictus quadricinctus;Halictus quadricinctus;1;Halictus quadricinctus;1;
MT871278;Hymenoptera;Halictus quadricinctus;Halictus quadricinctus;1;Halictus quadricinctus;1;
MT871459;Hymenoptera;Halictus quadricinctus;Halictus quadricinctus;1;Halictus quadricinctus;1;

MT871538;Hymenoptera;Lasioglossum sexnotatum;Lasioglossum sexnotatum;1;Lasioglossum sexnotatum;1;
MT871640;Hymenoptera;Halictus quadricinctus;Halictus quadricinctus;1;Halictus quadricinctus;1;
MT871678;Hymenoptera;Halictus quadricinctus;Halictus quadricinctus;1;Halictus quadricinctus;1;
MT871821;Hymenoptera;Halictus quadricinctus;Halictus quadricinctus;1;Halictus quadricinctus;1;
MT871840;Hymenoptera;Lasioglossum sexnotatum;Lasioglossum sexnotatum;1;Lasioglossum sexnotatum;1;
MZ607516;Hymenoptera;Bombus monticola;Bombus monticola;0.987;Bombus monticola;0.891228347514907;
MZ608606;Hymenoptera;Bombus monticola;Bombus monticola;1;Bombus monticola;0.99978633943506;
MZ609007;Hymenoptera;Bombus monticola;Bombus monticola;1;Bombus monticola;0.999746210653439;
MZ609350;Hymenoptera;Bombus monticola;Bombus monticola;0.99;Bombus monticola;0.789282477969724;
MZ622896;Hymenoptera;Colletes floralis;Colletes floralis;1;Colletes floralis;0.999999932107075;
MZ623000;Hymenoptera;Nomada roberjeotiana;Nomada tormentillae;0.993;Nomada tormentillae;0.924350479033569;
MZ623535;Hymenoptera;Andrena hattorfiana;Andrena hattorfiana;1;Andrena hattorfiana;1;
MZ624205;Hymenoptera;Nomada roberjeotiana;Nomada roberjeotiana;NA;Nomada roberjeotiana;0.998244023166367;
MZ624361;Hymenoptera;Bombus monticola;NA;0.001;Bombus monticola;0.805007038902856;
MZ624859;Hymenoptera;Nomada armata;Nomada armata;1;Nomada armata;0.999999999472777;
MZ625293;Hymenoptera;Nomada roberjeotiana;NA;NA;Nomada roberjeotiana;0.98474768386597;
MZ625426;Hymenoptera;Epeolus cruciger;Epeolus cruciger;1;Epeolus cruciger;0.999991286625705;
MZ625443;Hymenoptera;Epeolus cruciger;Epeolus cruciger;1;Epeolus cruciger;0.999995772456612;
MZ625473;Hymenoptera;Andrena fulvida;Andrena fulvida;1;Andrena fulvida;0.999999696975965;
MZ625851;Hymenoptera;Dufourea minuta;Dufourea minuta;1;Dufourea minuta;1;
MZ625877;Hymenoptera;Andrena fulvida;Andrena fulvida;1;Andrena fulvida;0.999999999911324;
MZ625887;Hymenoptera;Epeolus cruciger;Epeolus cruciger;0.001;Epeolus cruciger;0.99997288199024;
MZ626347;Hymenoptera;Bombus muscorum;NA;0.166;Bombus humilis;0.998047355529146;
MZ626543;Hymenoptera;Dufourea minuta;Dufourea minuta;1;Dufourea minuta;1;
MZ626598;Hymenoptera;Sphecodes hyalinatus;NA;0.222;Sphecodes hyalinatus;0.997362228792556;
MZ626770;Hymenoptera;Hylaeus pfankuchi;NA;0.056;Hylaeus pfankuchi;0.998085912521369;
MZ627178;Hymenoptera;Nomada roberjeotiana;Nomada roberjeotiana;NA;Nomada roberjeotiana;0.998244023166367;
MZ627860;Hymenoptera;Andrena fulvida;Andrena fulvida;1;Andrena fulvida;0.999999999911324;
MZ627897;Hymenoptera;Epeolus cruciger;Epeolus cruciger;0.998;Epeolus cruciger;0.999991684417338;
MZ628204;Hymenoptera;Nomada roberjeotiana;Nomada roberjeotiana;NA;Nomada roberjeotiana;0.998244023166367;
MZ628487;Hymenoptera;Bombus alpinus;Bombus alpinus;1;Bombus alpinus;0.999999924887081;
MZ628607;Hymenoptera;Bombus monticola;Bombus monticola;0.987;Bombus monticola;0.891228347514907;
MZ628725;Hymenoptera;Lasioglossum quadrinotatulum;Lasioglossum quadrinotatulum;1;Lasioglossum quadrinotatulum;0.999999999999773;
MZ628821;Hymenoptera;Colletes succinctus;NA;NA;NA;0.999999999999502;
NOBEE070-09;Hymenoptera;Epeolus cruciger;NA;0.321;Epeolus cruciger;0.975892562359686;
NOBEE071-09;Hymenoptera;Epeolus cruciger;NA;0.444;Epeolus cruciger;0.97602694074939;
TDAAT1041-20;Hymenoptera;Nomada armata;Nomada armata;1;Nomada armata;0.99999999960724;
TDAAT1044-20;Hymenoptera;Andrena hattorfiana;Andrena hattorfiana;1;Andrena hattorfiana;1;
TDAAT1324-20;Hymenoptera;Bombus mucidus;Bombus mucidus;1;Bombus mucidus;0.999992778697884;
TDAAT1325-20;Hymenoptera;Bombus mendax;Bombus mendax;1;Bombus mendax;0.99999999999406;
TDAAT1329-20;Hymenoptera;Bombus alpinus;Bombus alpinus;1;Bombus alpinus;0.999999963060674;
TDAAT1330-20;Hymenoptera;Bombus alpinus;Bombus alpinus;1;Bombus alpinus;0.999999963060674;
TDAAT1331-20;Hymenoptera;Bombus monticola;Bombus monticola;1;Bombus monticola;0.99982316997635;
TDAAT1332-20;Hymenoptera;Bombus monticola;Bombus monticola;1;Bombus monticola;0.99982316997635;
TDAAT1344-20;Hymenoptera;Bombus monticola;Bombus monticola;1;Bombus monticola;0.99982316997635;
TDAAT1491-20;Hymenoptera;Andrena hattorfiana;Andrena hattorfiana;1;Andrena hattorfiana;1;
TDAAT2093-20;Hymenoptera;Bombus mucidus;Bombus mucidus;1;Bombus mucidus;0.9999961618278;
TDAOE069-21;Hymenoptera;Andrena ovatula;Andrena wilkella;NA;Andrena wilkella;0.999999449020417;
TDAOE081-21;Hymenoptera;Andrena hattorfiana;Andrena hattorfiana;1;Andrena hattorfiana;1;
TDAOE493-21;Hymenoptera;Andrena ovatula;Andrena ovatula;1;Andrena ovatula;0.999999999813497;
VWB014-18;Hymenoptera;Nomada armata;Nomada armata;1;Nomada armata;0.999999999350706;
VWB016-18;Hymenoptera;Andrena hattorfiana;Andrena hattorfiana;1;Andrena hattorfiana;1;
VWB028-18;Hymenoptera;Halictus quadricinctus;Halictus quadricinctus;1;Halictus quadricinctus;1;
VWB034-18;Hymenoptera;Lasioglossum xanthopus;Lasioglossum xanthopus;0.001;Lasioglossum xanthopus;0.994637020414442;
VWB037-18;Hymenoptera;Andrena ovatula;Andrena ovatula;1;Andrena ovatula;0.999999968230043;
WASPS382-14;Hymenoptera;Bombus alpinus;Bombus alpinus;1;Bombus alpinus;0.999999961371487;
ZMBN809-17;Hymenoptera;Bombus alpinus;NA;0.013;Bombus pyrrhopygus;0.997688466041621;
ZPLBI007-22;Hymenoptera;Bombus muscorum;Bombus humilis;1;Bombus humilis;0.999136246437524;
AB674372;Lepidoptera;Erebia euryale;NA;0.301;NA;0.999999999962217;
AB674384;Lepidoptera;Erebia medusa;NA;0.01;Erebia medusa;0.972811569416705;
AB674386;Lepidoptera;Erebia medusa;NA;0.018;Erebia medusa;0.982727105014107;
AB674387;Lepidoptera;Erebia medusa;NA;0.013;Erebia medusa;0.984905305072752;
AB674388;Lepidoptera;Erebia oeme;NA;0.28;Erebia oeme;0.99999999890909;
AB674389;Lepidoptera;Erebia oeme;Erebia oeme;0.003;Erebia oeme;0.999999990988101;
AB674390;Lepidoptera;Erebia oeme;Erebia oeme;0.001;Erebia oeme;0.999999993492906;
AB674391;Lepidoptera;Erebia oeme;Erebia oeme;0.006;Erebia oeme;0.999999998598241;
AB674392;Lepidoptera;Erebia oeme;Erebia oeme;0.006;Erebia oeme;0.999999998598241;
AB674393;Lepidoptera;Erebia ottomana;Erebia ottomana;1;Erebia ottomana;0.99999999688427;
AB674394;Lepidoptera;Erebia ottomana;Erebia ottomana;1;Erebia ottomana;0.99999999688427;
AB674395;Lepidoptera;Erebia ottomana;Erebia ottomana;1;Erebia ottomana;0.999999998814303;

AB674396;Lepidoptera;Erebia ottomana;Erebia ottomana;1;Erebia ottomana;0.999999997330434;
AB674397;Lepidoptera;Erebia ottomana;Erebia ottomana;1;Erebia ottomana;0.999999997165077;
AB674398;Lepidoptera;Erebia ottomana;Erebia ottomana;1;Erebia ottomana;0.999999997165077;
AB674399;Lepidoptera;Erebia pandrose;Erebia pandrose;0.004;Erebia pandrose;0.976358963490405;
AB674400;Lepidoptera;Erebia pandrose;Erebia pandrose;0.027;Erebia pandrose;0.9864806252672;
ABOLD439-16;Lepidoptera;Satyrium pruni;NA;NA;Satyrium pruni;0.978161568775537;
ABOLD489-16;Lepidoptera;Lycaena thersamon;Thersamonia thersamon;0.035;Lycaena thersamon;0.956733690681528;
ABOLD490-16;Lepidoptera;Satyrium pruni;NA;NA;Satyrium pruni;0.969814295341849;
ABOLD492-16;Lepidoptera;Cupido osiris;Cupido osiris;0.999;Cupido osiris;0.999864248064952;
ABOLD494-16;Lepidoptera;Cupido alcetas;NA;0.002;Cupido alcetas;0.801754491963039;
ABOLD495-16;Lepidoptera;Coenonympha oedippus;Coenonympha oedippus;1;Coenonympha oedippus;0.999999999471498;
ABOLD497-16;Lepidoptera;Melitaea britomartis;NA;NA;Melitaea britomartis;0.996328395979252;
ABOLD502-16;Lepidoptera;Melitaea varia;NA;0.001;Melitaea varia;0.976287436532261;
ABOLD506-16;Lepidoptera;Limenitis populi;Limenitis populi;1;Limenitis populi;0.999995827628958;
AF153920;Lepidoptera;Euphydryas aurinia;NA;0.307;Euphydryas aurinia;0.918993679737925;
AF153933;Lepidoptera;Euphydryas maturna;NA;0.125;Euphydryas maturna;0.873338270148348;
AM262789;Lepidoptera;Anthocharis cardamines;Anthocharis cardamines;1;Anthocharis cardamines;0.999989701606745;
AM262790;Lepidoptera;Anthocharis cardamines;Anthocharis cardamines;1;Anthocharis cardamines;0.99999907808788;
AM262796;Lepidoptera;Anthocharis cardamines;Anthocharis cardamines;1;Anthocharis cardamines;0.99999907808788;
AM262798;Lepidoptera;Anthocharis cardamines;Anthocharis cardamines;1;Anthocharis cardamines;0.99999907808788;
AM262802;Lepidoptera;Anthocharis cardamines;Anthocharis cardamines;1;Anthocharis cardamines;0.99999907808788;
AM262803;Lepidoptera;Anthocharis cardamines;Anthocharis cardamines;1;Anthocharis cardamines;0.999993720783938;
AM262804;Lepidoptera;Anthocharis damone;Anthocharis damone;0.999;Anthocharis damone;0.99999654698177;
AM262806;Lepidoptera;Anthocharis cardamines;NA;0.163;Anthocharis cardamines;0.999498436246391;
AM262810;Lepidoptera;Anthocharis damone;Anthocharis damone;NA;Anthocharis damone;0.99999780755971;
AM262811;Lepidoptera;Anthocharis damone;Anthocharis damone;0.001;Anthocharis damone;0.999996577763313;
AMTPC042-15;Lepidoptera;Aporia crataegi;NA;0.005;Aporia crataegi;0.999985516587155;
AMTPC045-15;Lepidoptera;Boloria euphrosyne;Boloria euphrosyne;1;Boloria euphrosyne;0.99997773695286;
AMTPC046-15;Lepidoptera;Melitaea diamina;NA;0.157;Melitaea diamina;0.999999981720549;
AMTPC047-15;Lepidoptera;Melitaea diamina;NA;0.16;Melitaea diamina;0.999999979251413;
AMTPC048-15;Lepidoptera;Lasiommata maera;Lasiommata maera;0.029;Lasiommata maera;0.999987056003009;
AMTPC049-15;Lepidoptera;Pyrgus warrenensis;NA;0.002;NA;0.9999999999984;
AMTPC050-15;Lepidoptera;Ochlodes sylvanus;NA;NA;Ochlodes sylvanus;0.995823680926199;
AMTPC051-15;Lepidoptera;Cyaniris semiargus;NA;NA;Cyaniris semiargus;0.999101499375485;
AMTPC052-15;Lepidoptera;Cupido minimus;NA;0.004;Cupido minimus;0.997479807037898;
AMTPC084-15;Lepidoptera;Erynnis tages;Erynnis tages;1;Erynnis tages;0.999955880826156;
AMTPC122-15;Lepidoptera;Boloria euphrosyne;Boloria euphrosyne;1;Boloria euphrosyne;0.999976943686819;
AMTPC123-15;Lepidoptera;Lasiommata petropolitana;Lasiommata petropolitana;1;Lasiommata petropolitana;0.999997871383917;
AMTPC124-15;Lepidoptera;Cupido minimus;NA;0.003;Cupido minimus;0.995871121583884;
AMTPC125-15;Lepidoptera;Aglais urticae;NA;0.001;Aglais urticae;0.986745888835628;
AMTPC126-15;Lepidoptera;Boloria euphrosyne;Boloria euphrosyne;1;Boloria euphrosyne;0.99997773695286;
AMTPC127-15;Lepidoptera;Cupido minimus;NA;0.008;Cupido minimus;0.998438286349197;
AMTPC128-15;Lepidoptera;Cupido minimus;NA;0.001;Cupido minimus;0.998843602978539;
AMTPC129-15;Lepidoptera;Boloria euphrosyne;Boloria euphrosyne;0.998;Boloria euphrosyne;0.999973474063339;
AMTPC130-15;Lepidoptera;Pyrgus warrenensis;NA;0.002;NA;0.9999999999984;
AMTPC132-15;Lepidoptera;Callophrys rubi;NA;0.009;Callophrys rubi;0.996682294679977;
AMTPC133-15;Lepidoptera;Boloria euphrosyne;Boloria euphrosyne;0.001;Boloria euphrosyne;0.999641955959878;
AMTPC138-15;Lepidoptera;Gonepteryx rhamni;NA;0.487;Gonepteryx rhamni;0.986058728586698;
AMTPC139-15;Lepidoptera;Pyrgus malvae;Pyrgus malvae;0.005;Pyrgus malvae;0.986140979485564;
AMTPC153-15;Lepidoptera;Pyrgus malvae;Pyrgus malvae;0.005;Pyrgus malvae;0.986140979485564;
AMTPC191-15;Lepidoptera;Erebia pharte;Erebia pharte;1;Erebia pharte;0.999999980780018;
AMTPC192-15;Lepidoptera;Erebia pharte;Erebia pharte;1;Erebia pharte;0.999999991026073;
AMTPC193-15;Lepidoptera;Coenonympha gardetta;NA;0.002;Coenonympha gardetta;0.76122108155261;
AMTPC204-15;Lepidoptera;Aricia artaxerxes;NA;0.493;Aricia artaxerxes;0.999564449613157;
AMTPC214-15;Lepidoptera;Erebia manto;Erebia manto;0.003;Erebia manto;0.999979531388322;
AMTPC222-15;Lepidoptera;Cupido minimus;NA;0.013;Cupido minimus;0.998077282612956;
AMTPC226-15;Lepidoptera;Cyaniris semiargus;NA;NA;Cyaniris semiargus;0.999101499375485;
AMTPC227-15;Lepidoptera;Erebia oeme;NA;0.244;Erebia oeme;0.999999999877048;
AMTPC228-15;Lepidoptera;Erebia meolans;Erebia meolans;NA;Erebia meolans;0.999872284309413;
AMTPC229-15;Lepidoptera;Erebia oeme;NA;0.244;Erebia oeme;0.999999999877048;

AMTPC231-15;Lepidoptera;Lasiommata maera;Lasiommata maera;0.998;Lasiommata maera;0.999987703988518;
AMTPC232-15;Lepidoptera;Lasiommata maera;Lasiommata maera;0.998;Lasiommata maera;0.999987703988518;
AMTPC234-15;Lepidoptera;Melitaea diamina;NA;0.157;Melitaea diamina;0.999999981720549;
AMTPC236-15;Lepidoptera;Melitaea diamina;NA;0.157;Melitaea diamina;0.999999981720549;
AMTPC247-15;Lepidoptera;Erebia pronoe;Erebia pronoe;NA;Erebia pronoe;0.834226812415793;
AMTPC261-15;Lepidoptera;Cupido minimus;NA;0.004;Cupido minimus;0.998215728074331;
AMTPC263-15;Lepidoptera;Erebia oeme;NA;0.244;Erebia oeme;0.999999999877048;
AMTPC265-15;Lepidoptera;Erebia tyndarus;NA;NA;NA;0.999999999999949;
AMTPC300-15;Lepidoptera;Pieris napi;NA;0.013;Pieris napi;0.764655619178569;
AMTPC302-15;Lepidoptera;Anthocharis cardamines;Anthocharis cardamines;1;Anthocharis cardamines;0.999999596425763;
AMTPC304-15;Lepidoptera;Erebia manto;Erebia manto;0.003;Erebia manto;0.999979531388322;
AMTPC305-15;Lepidoptera;Erebia manto;Erebia manto;0.003;Erebia manto;0.999979531388322;
AMTPC333-15;Lepidoptera;Melitaea diamina;NA;0.16;Melitaea diamina;0.999999978877298;
AMTPC365-15;Lepidoptera;Erebia eriphyle;Erebia eriphyle;1;Erebia eriphyle;0.999999974010791;
AMTPC375-15;Lepidoptera;Erebia oeme;NA;0.244;Erebia oeme;0.999999999877048;
AMTPC380-15;Lepidoptera;Erebia oeme;NA;0.244;Erebia oeme;0.999999999877048;
AMTPC394-15;Lepidoptera;Aricia artaxerxes;NA;0.002;Aricia artaxerxes;0.992637870166738;
AMTPC405-15;Lepidoptera;Melitaea diamina;NA;0.151;Melitaea diamina;0.999999944912021;
AMTPC432-15;Lepidoptera;Boloria euphrosyne;Boloria euphrosyne;NA;Boloria euphrosyne;0.999637709581655;
AMTPC433-15;Lepidoptera;Melitaea diamina;NA;0.176;Melitaea diamina;0.999999944597734;
AMTPC434-15;Lepidoptera;Maculinea arion;NA;0.002;Phengaris arion;0.840187680703552;
AMTPC449-15;Lepidoptera;Coenonympha pamphilus;Coenonympha pamphilus;1;Coenonympha pamphilus;0.999999999995225;
AMTPC450-15;Lepidoptera;Cyaniris semiargus;NA;NA;Cyaniris semiargus;0.998918074728235;
AMTPC451-15;Lepidoptera;Pyrgus malvae;Pyrgus malvae;0.006;Pyrgus malvae;0.991284437961241;
AMTPC453-15;Lepidoptera;Maculinea arion;NA;0.001;Phengaris arion;0.875469653948688;
AMTPC454-15;Lepidoptera;Callophrys rubi;NA;0.017;Callophrys rubi;0.98618411141067;
AMTPC491-15;Lepidoptera;Erebia tyndarus;NA;0.001;Erebia tyndarus;0.758216264275257;
ANBIO196-19;Lepidoptera;Vanessa atalanta;Vanessa atalanta;1;Vanessa atalanta;0.9999999993762;
ANBIO330-19;Lepidoptera;Thymelicus sylvestris;NA;0.324;Thymelicus sylvestris;0.999940778442454;
ATLAS002-22;Lepidoptera;Aglais urticae;NA;0.002;Aglais urticae;0.987497593219019;
ATLAS003-22;Lepidoptera;Agriades glandon;NA;0.001;Agriades glandon;0.985149519522682;
ATLAS004-22;Lepidoptera;Agriades glandon;NA;0.001;Agriades glandon;0.985149519522682;
ATLAS007-22;Lepidoptera;Aphantopus hyperantus;Aphantopus hyperantus;0.004;Aphantopus hyperantus;0.999598909345147;
ATLAS011-22;Lepidoptera;Aricia agestis;NA;0.253;Aricia agestis;0.996350017387504;
ATLAS012-22;Lepidoptera;Aricia agestis;NA;0.001;Aricia artaxerxes;0.998546425606378;
ATLAS013-22;Lepidoptera;Aricia agestis;NA;0.067;Aricia agestis;0.999509743227786;
ATLAS014-22;Lepidoptera;Aricia artaxerxes;NA;0.001;Aricia artaxerxes;0.999429422213984;
ATLAS015-22;Lepidoptera;Aricia cramera;Aricia cramera;1;Aricia cramera;0.999993731037033;
ATLAS016-22;Lepidoptera;Aricia cramera;Aricia cramera;1;Aricia cramera;0.999993731037033;
ATLAS018-22;Lepidoptera;Aricia cramera;Aricia cramera;1;Aricia cramera;0.999993731037033;
ATLAS019-22;Lepidoptera;Aricia cramera;Aricia cramera;1;Aricia cramera;0.999993731037033;
ATLAS020-22;Lepidoptera;Aricia cramera;Aricia cramera;1;Aricia cramera;0.999993731037033;
ATLAS021-22;Lepidoptera;Aricia cramera;Aricia cramera;1;Aricia cramera;0.999993731037033;
ATLAS022-22;Lepidoptera;Aricia cramera;Aricia cramera;1;Aricia cramera;0.999993731037033;
ATLAS023-22;Lepidoptera;Aricia cramera;Aricia cramera;1;Aricia cramera;0.999993731037033;
ATLAS024-22;Lepidoptera;Aricia cramera;Aricia cramera;1;Aricia cramera;0.999993731037033;
ATLAS032-22;Lepidoptera;Boloria dia;Boloria dia;1;Boloria dia;0.999999999963421;
ATLAS033-22;Lepidoptera;Boloria dia;Boloria dia;1;Boloria dia;0.999999999980503;
ATLAS034-22;Lepidoptera;Boloria dia;Boloria dia;1;Boloria dia;0.999999999980503;
ATLAS038-22;Lepidoptera;Boloria selene;NA;0.092;Boloria selene;0.99999957723188;
ATLAS039-22;Lepidoptera;Brenthis daphne;Brenthis daphne;0.001;Brenthis daphne;0.994571844722107;
ATLAS040-22;Lepidoptera;Brenthis daphne;NA;0.102;Brenthis daphne;0.717134521611551;
ATLAS041-22;Lepidoptera;Brenthis daphne;Brenthis ino;0.007;Brenthis ino;0.977157381961834;
ATLAS042-22;Lepidoptera;Brenthis hecate;NA;0.072;Brenthis hecate;0.999803056289114;
ATLAS043-22;Lepidoptera;Brenthis hecate;NA;0.068;Brenthis hecate;0.999053551498733;
ATLAS044-22;Lepidoptera;Brintesia circe;Brintesia circe;NA;Brintesia circe;0.999999706538049;
ATLAS045-22;Lepidoptera;Brintesia circe;Brintesia circe;1;Brintesia circe;0.99999951254154;
ATLAS051-22;Lepidoptera;Callophrys avis;Callophrys avis;1;Callophrys avis;0.992335035373355;
ATLAS052-22;Lepidoptera;Callophrys avis;Callophrys avis;1;Callophrys avis;0.992335035373355;
ATLAS053-22;Lepidoptera;Callophrys rubi;NA;0.02;Callophrys rubi;0.999082687464383;
ATLAS055-22;Lepidoptera;Carcharodus alceae;Carcharodus alceae;1;Carcharodus alceae;0.999999998465938;
ATLAS056-22;Lepidoptera;Carcharodus alceae;Carcharodus alceae;NA;Carcharodus alceae;0.999999984059485;
ATLAS057-22;Lepidoptera;Carcharodus alceae;Carcharodus alceae;0.999;Carcharodus alceae;0.999999996339511;
ATLAS060-22;Lepidoptera;Carterocephalus palaemon;NA;0.052;Carterocephalus palaemon;0.728445051795325;
ATLAS061-22;Lepidoptera;Carterocephalus palaemon;NA;0.052;Carterocephalus palaemon;0.728445051795325;
ATLAS062-22;Lepidoptera;Carterocephalus palaemon;NA;0.052;Carterocephalus palaemon;0.728445051795325;

ATLAS065-22;Lepidoptera;Celastrina argiolus;Celastrina argiolus;1;Celastrina argiolus;0.999527385150531;
ATLAS066-22;Lepidoptera;Celastrina argiolus;Celastrina argiolus;1;Celastrina argiolus;0.999527385150531;
ATLAS067-22;Lepidoptera;Charaxes jasius;Charaxes jasius;NA;Charaxes jasius;0.988941612024336;
ATLAS068-22;Lepidoptera;Charaxes jasius;Charaxes jasius;NA;Charaxes jasius;0.990397821479112;
ATLAS069-22;Lepidoptera;Charaxes jasius;Charaxes jasius;NA;Charaxes jasius;0.982882460966838;
ATLAS071-22;Lepidoptera;Charaxes jasius;Charaxes jasius;NA;Charaxes jasius;0.995860724722804;
ATLAS072-22;Lepidoptera;Charaxes jasius;Charaxes jasius;NA;Charaxes jasius;0.949854280059292;
ATLAS073-22;Lepidoptera;Charaxes jasius;Charaxes jasius;NA;Charaxes jasius;0.995860724722804;
ATLAS085-22;Lepidoptera;Coenonympha arcania;NA;0.172;Coenonympha arcania;0.963036309473038;
ATLAS086-22;Lepidoptera;Coenonympha arcania;NA;0.273;Coenonympha arcania;0.948490192015929;
ATLAS089-22;Lepidoptera;Coenonympha pamphilus;Coenonympha pamphilus;1;Coenonympha pamphilus;0.999999999999744;
ATLAS092-22;Lepidoptera;Colias alfacariensis;Erynnis tages;1;Erynnis tages;0.999999281987018;
ATLAS093-22;Lepidoptera;Colias alfacariensis;Colias alfacariensis;1;Colias alfacariensis;0.999894613238469;
ATLAS094-22;Lepidoptera;Colias alfacariensis;Colias alfacariensis;0.001;Colias alfacariensis;0.999926026317526;
ATLAS095-22;Lepidoptera;Colias alfacariensis;Colias alfacariensis;0.001;Colias alfacariensis;0.999926026317526;
ATLAS096-22;Lepidoptera;Colias alfacariensis;Colias alfacariensis;0.001;Colias alfacariensis;0.999926026317526;
ATLAS097-22;Lepidoptera;Colias alfacariensis;Colias alfacariensis;0.001;Colias alfacariensis;0.999926026317526;
ATLAS098-22;Lepidoptera;Colias alfacariensis;Colias alfacariensis;0.001;Colias alfacariensis;0.999926026317526;
ATLAS099-22;Lepidoptera;Colias alfacariensis;Colias alfacariensis;0.001;Colias alfacariensis;0.999926026317526;
ATLAS100-22;Lepidoptera;Colias alfacariensis;Colias alfacariensis;0.001;Colias alfacariensis;0.999926026317526;
ATLAS101-22;Lepidoptera;Colias alfacariensis;Colias alfacariensis;0.001;Colias alfacariensis;0.999926026317526;
ATLAS102-22;Lepidoptera;Colias alfacariensis;Colias alfacariensis;0.001;Colias alfacariensis;0.999926026317526;
ATLAS103-22;Lepidoptera;Colias alfacariensis;Colias alfacariensis;0.001;Colias alfacariensis;0.999926026317526;
ATLAS104-22;Lepidoptera;Colias alfacariensis;Colias alfacariensis;0.001;Colias alfacariensis;0.999926026317526;
ATLAS105-22;Lepidoptera;Colias alfacariensis;Colias alfacariensis;0.001;Colias alfacariensis;0.999926026317526;
ATLAS106-22;Lepidoptera;Colias alfacariensis;Colias alfacariensis;0.001;Colias alfacariensis;0.999926026317526;
ATLAS107-22;Lepidoptera;Colias alfacariensis;Colias alfacariensis;0.001;Colias alfacariensis;0.999926026317526;
ATLAS108-22;Lepidoptera;Colias alfacariensis;Colias alfacariensis;1;Colias alfacariensis;0.999986994131001;
ATLAS109-22;Lepidoptera;Colias alfacariensis;Colias alfacariensis;0.001;Colias alfacariensis;0.999857790810452;
ATLAS110-22;Lepidoptera;Colias alfacariensis;Colias alfacariensis;0.001;Colias alfacariensis;0.999857790810452;
ATLAS111-22;Lepidoptera;Colias alfacariensis;Colias alfacariensis;0.001;Colias alfacariensis;0.999926026317526;
ATLAS113-22;Lepidoptera;Colias crocea;NA;0.059;NA;0.999999999999738;
ATLAS118-22;Lepidoptera;Colias crocea;NA;0.059;NA;0.999999999999738;
ATLAS119-22;Lepidoptera;Colias crocea;NA;0.059;NA;0.999999999999738;
ATLAS120-22;Lepidoptera;Colias crocea;NA;NA;NA;0.999999999999751;
ATLAS121-22;Lepidoptera;Colias crocea;NA;NA;NA;0.999999999999751;
ATLAS122-22;Lepidoptera;Colias crocea;NA;NA;NA;0.999999999999751;
ATLAS123-22;Lepidoptera;Colias crocea;NA;NA;NA;0.999999999999751;
ATLAS124-22;Lepidoptera;Colias crocea;NA;NA;NA;0.999999999999751;
ATLAS125-22;Lepidoptera;Colias crocea;NA;NA;NA;0.999999999999715;
ATLAS126-22;Lepidoptera;Colias crocea;NA;NA;NA;0.999999999999751;
ATLAS127-22;Lepidoptera;Colias crocea;NA;NA;NA;0.999999999999741;
ATLAS128-22;Lepidoptera;Colias crocea;NA;NA;NA;0.999999999999334;
ATLAS129-22;Lepidoptera;Colias crocea;NA;NA;NA;0.999999999999707;
ATLAS130-22;Lepidoptera;Colias crocea;NA;NA;NA;0.999999999999751;
ATLAS131-22;Lepidoptera;Colias crocea;NA;NA;NA;0.999999999999751;
ATLAS132-22;Lepidoptera;Colias crocea;NA;NA;NA;0.99999999999975;
ATLAS133-22;Lepidoptera;Colias crocea;NA;NA;NA;0.99999999999975;
ATLAS134-22;Lepidoptera;Colias crocea;NA;NA;NA;0.999999999999751;
ATLAS135-22;Lepidoptera;Colias crocea;NA;NA;NA;0.999999999988337;
ATLAS136-22;Lepidoptera;Colias crocea;NA;NA;NA;0.999999999999751;
ATLAS137-22;Lepidoptera;Colias crocea;NA;NA;NA;0.999999999999751;
ATLAS138-22;Lepidoptera;Colias crocea;NA;NA;NA;0.999999999999751;
ATLAS139-22;Lepidoptera;Colias crocea;NA;NA;NA;0.999999999999751;
ATLAS140-22;Lepidoptera;Colias crocea;NA;NA;NA;0.999999999999751;
ATLAS141-22;Lepidoptera;Colias crocea;NA;NA;NA;0.999999999999751;

ATLAS142-22;Lepidoptera;Colias crocea;NA;0.059;NA;0.999999999999738;
ATLAS143-22;Lepidoptera;Colias crocea;NA;0.059;NA;0.999999999999738;
ATLAS144-22;Lepidoptera;Colias crocea;NA;0.059;NA;0.999999999999738;
ATLAS145-22;Lepidoptera;Colias crocea;NA;0.059;NA;0.999999999999738;
ATLAS146-22;Lepidoptera;Colias crocea;NA;0.059;NA;0.999999999999738;
ATLAS147-22;Lepidoptera;Colias crocea;NA;0.059;NA;0.999999999999738;
ATLAS148-22;Lepidoptera;Colias hyale;Colias hyale;0.015;Colias hyale;0.999997884296128;
ATLAS149-22;Lepidoptera;Colias hyale;Colias hyale;0.015;Colias hyale;0.999997884296128;
ATLAS150-22;Lepidoptera;Colias hyale;Colias hyale;0.015;Colias hyale;0.999997884296128;
ATLAS151-22;Lepidoptera;Colias hyale;Colias hyale;0.015;Colias hyale;0.999997884296128;
ATLAS152-22;Lepidoptera;Colias hyale;Colias hyale;0.015;Colias hyale;0.999997884296128;
ATLAS153-22;Lepidoptera;Colias hyale;Colias hyale;0.016;Colias hyale;0.999997994514011;
ATLAS154-22;Lepidoptera;Colias hyale;Colias hyale;0.015;Colias hyale;0.999997923550718;
ATLAS155-22;Lepidoptera;Cupido alcetas;NA;0.068;Cupido alcetas;0.88988907876118;
ATLAS157-22;Lepidoptera;Cupido argiades;NA;0.499;NA;0.999999999984682;
ATLAS160-22;Lepidoptera;Cupido minimus;NA;0.001;Cupido minimus;0.99992423685743;
ATLAS161-22;Lepidoptera;Cupido osiris;Cupido osiris;1;Cupido osiris;0.999999351858474;
ATLAS162-22;Lepidoptera;Cyaniris semiargus;NA;NA;NA;0.999998909142239;
ATLAS168-22;Lepidoptera;Erebia epiphron;NA;NA;Erebia epiphron;0.950006471153786;
ATLAS169-22;Lepidoptera;Erebia epiphron;NA;NA;Erebia epiphron;0.950006471153786;
ATLAS170-22;Lepidoptera;Erebia epiphron;NA;NA;Erebia epiphron;0.950006471153786;
ATLAS173-22;Lepidoptera;Erebia euryale;NA;NA;Erebia euryale;0.935077349534533;
ATLAS183-22;Lepidoptera;Erebia meolans;Erebia meolans;NA;Erebia meolans;0.99976373317804;
ATLAS184-22;Lepidoptera;Erebia meolans;Erebia meolans;NA;Erebia meolans;0.999835953426128;
ATLAS185-22;Lepidoptera;Erebia neoridas;NA;NA;Erebia neoridas;0.997677227800323;
ATLAS187-22;Lepidoptera;Erynnis tages;Erynnis tages;1;Erynnis tages;0.999999281987018;
ATLAS227-22;Lepidoptera;Euphydryas aurinia;Euphydryas aurinia;0.016;Euphydryas aurinia;0.997751503369639;
ATLAS228-22;Lepidoptera;Euphydryas aurinia;Euphydryas aurinia;0.016;Euphydryas aurinia;0.997751503369639;
ATLAS232-22;Lepidoptera;Gegenes nostrodamus;Gegenes nostrodamus;1;Gegenes nostrodamus;0.999999997215411;
ATLAS233-22;Lepidoptera;Gegenes nostrodamus;Gegenes nostrodamus;1;Gegenes nostrodamus;0.999999996883645;
ATLAS234-22;Lepidoptera;Gegenes nostrodamus;Gegenes nostrodamus;1;Gegenes nostrodamus;0.999999996883645;
ATLAS235-22;Lepidoptera;Gegenes nostrodamus;Gegenes nostrodamus;1;Gegenes nostrodamus;0.999999996883645;
ATLAS236-22;Lepidoptera;Gegenes nostrodamus;Gegenes nostrodamus;1;Gegenes nostrodamus;0.999999996883645;
ATLAS237-22;Lepidoptera;Gegenes nostrodamus;Gegenes nostrodamus;1;Gegenes nostrodamus;0.999999996883645;
ATLAS256-22;Lepidoptera;Gonepteryx rhamni;NA;0.474;Gonepteryx rhamni;0.982076396127858;
ATLAS257-22;Lepidoptera;Hipparchia aristaeus;NA;0.001;Hipparchia semele;0.86935938851355;
ATLAS296-22;Lepidoptera;Iolana iolas;NA;NA;Iolana iolas;0.97259403345821;
ATLAS297-22;Lepidoptera;Iolana iolas;NA;NA;Iolana iolas;0.998335020541118;
ATLAS299-22;Lepidoptera;Issoria lathonia;Issoria lathonia;1;Issoria lathonia;0.999999999989257;
ATLAS300-22;Lepidoptera;Issoria lathonia;Issoria lathonia;1;Issoria lathonia;0.999999999989257;
ATLAS303-22;Lepidoptera;Lampides boeticus;Lampides boeticus;1;Lampides boeticus;0.999999999545906;
ATLAS304-22;Lepidoptera;Lampides boeticus;Lampides boeticus;1;Lampides boeticus;0.999999999545906;
ATLAS305-22;Lepidoptera;Lampides boeticus;Lampides boeticus;1;Lampides boeticus;0.99999999876789;
ATLAS308-22;Lepidoptera;Lampides boeticus;Lampides boeticus;1;Lampides boeticus;0.999999999545906;
ATLAS309-22;Lepidoptera;Lampides boeticus;Lampides boeticus;1;Lampides boeticus;0.999999999691909;
ATLAS310-22;Lepidoptera;Lampides boeticus;Lampides boeticus;1;Lampides boeticus;0.999999999699043;
ATLAS311-22;Lepidoptera;Lampides boeticus;Lampides boeticus;1;Lampides boeticus;0.999999939889337;
ATLAS312-22;Lepidoptera;Lampides boeticus;Lampides boeticus;1;Lampides boeticus;0.999999999691909;
ATLAS313-22;Lepidoptera;Lampides boeticus;Lampides boeticus;1;Lampides boeticus;0.999999999545906;
ATLAS314-22;Lepidoptera;Lampides boeticus;Lampides boeticus;1;Lampides boeticus;0.999999999545906;
ATLAS315-22;Lepidoptera;Lampides boeticus;Lampides boeticus;1;Lampides boeticus;0.999999999545906;
ATLAS316-22;Lepidoptera;Lampides boeticus;Lampides boeticus;1;Lampides boeticus;0.999999999545906;
ATLAS317-22;Lepidoptera;Lampides boeticus;Lampides boeticus;1;Lampides boeticus;0.999999999545906;
ATLAS318-22;Lepidoptera;Lampides boeticus;Lampides boeticus;1;Lampides boeticus;0.999999999690317;
ATLAS319-22;Lepidoptera;Lampides boeticus;Lampides boeticus;1;Lampides boeticus;0.99999999872125;

ATLAS320-22;Lepidoptera;Lasiommata maera;Lasiommata maera;1;Lasiommata maera;0.999997945450226;
ATLAS321-22;Lepidoptera;Lasiommata megera;Lasiommata megera;1;Lasiommata megera;0.999928528239676;
ATLAS322-22;Lepidoptera;Lasiommata megera;Lasiommata megera;1;Lasiommata megera;0.999928528239676;
ATLAS392-22;Lepidoptera;Lycaena alciphron;Lycaena alciphron;1;Lycaena alciphron;0.999999738488788;
ATLAS393-22;Lepidoptera;Lycaena hippothoe;NA;NA;Lycaena hippothoe;0.928973453460131;
ATLAS394-22;Lepidoptera;Lycaena phlaeas;Lycaena phlaeas;1;Lycaena phlaeas;0.999999996363186;
ATLAS397-22;Lepidoptera;Lycaena phlaeas;Lycaena phlaeas;1;Lycaena phlaeas;0.999999996363186;
ATLAS403-22;Lepidoptera;Maniola jurtina;NA;0.038;NA;0.999999999999524;
ATLAS404-22;Lepidoptera;Maniola jurtina;NA;0.001;Maniola jurtina;0.939220105860875;
ATLAS405-22;Lepidoptera;Maniola jurtina;NA;NA;Maniola jurtina;0.969611756132048;
ATLAS406-22;Lepidoptera;Maniola jurtina;NA;0.001;Maniola jurtina;0.939220105860875;
ATLAS407-22;Lepidoptera;Maniola jurtina;NA;0.001;Maniola jurtina;0.939220105860875;
ATLAS408-22;Lepidoptera;Maniola jurtina;NA;NA;Maniola jurtina;0.982233585563106;
ATLAS409-22;Lepidoptera;Maniola jurtina;NA;0.001;Maniola jurtina;0.939220105860875;
ATLAS411-22;Lepidoptera;Maniola jurtina;NA;0.001;Maniola jurtina;0.939220105860875;
ATLAS412-22;Lepidoptera;Maniola jurtina;NA;0.001;Maniola jurtina;0.939220105860875;
ATLAS442-22;Lepidoptera;Melitaea deione;Melitaea deione;1;Melitaea deione;0.999997357311937;
ATLAS443-22;Lepidoptera;Melitaea deione;Melitaea deione;1;Melitaea deione;0.999998609499699;
ATLAS444-22;Lepidoptera;Melitaea deione;Melitaea deione;1;Melitaea deione;0.999999066084409;
ATLAS445-22;Lepidoptera;Melitaea deione;Melitaea deione;1;Melitaea deione;0.999999999302304;
ATLAS446-22;Lepidoptera;Melitaea deione;Melitaea deione;1;Melitaea deione;0.999999998407247;
ATLAS447-22;Lepidoptera;Melitaea deione;Melitaea deione;1;Melitaea deione;0.999999990313768;
ATLAS448-22;Lepidoptera;Melitaea diamina;NA;0.149;Melitaea diamina;0.999999996592464;
ATLAS449-22;Lepidoptera;Melitaea diamina;Melitaea diamina;0.002;Melitaea diamina;0.999999850355675;
ATLAS450-22;Lepidoptera;Melitaea diamina;NA;0.066;Melitaea diamina;0.999999985294579;
ATLAS451-22;Lepidoptera;Melitaea diamina;NA;0.066;Melitaea diamina;0.999999985294579;
ATLAS452-22;Lepidoptera;Melitaea didyma;Melitaea didyma;0.995;Melitaea didyma;0.994792486381848;
ATLAS461-22;Lepidoptera;Melitaea didyma;Melitaea didyma;1;Melitaea didyma;0.996366765827355;
ATLAS466-22;Lepidoptera;Melitaea parthenoides;Melitaea parthenoides;1;Melitaea parthenoides;0.999999968152765;
ATLAS467-22;Lepidoptera;Melitaea parthenoides;Melitaea parthenoides;1;Melitaea parthenoides;0.999999993715846;
ATLAS468-22;Lepidoptera;Melitaea parthenoides;Melitaea parthenoides;1;Melitaea parthenoides;0.999999969741709;
ATLAS469-22;Lepidoptera;Melitaea parthenoides;Melitaea parthenoides;1;Melitaea parthenoides;0.999998278417568;
ATLAS470-22;Lepidoptera;Melitaea parthenoides;Melitaea parthenoides;1;Melitaea parthenoides;0.999999984713213;
ATLAS471-22;Lepidoptera;Melitaea parthenoides;Melitaea parthenoides;1;Melitaea parthenoides;0.999999995982648;
ATLAS472-22;Lepidoptera;Melitaea parthenoides;Melitaea parthenoides;1;Melitaea parthenoides;0.999999995457472;
ATLAS473-22;Lepidoptera;Melitaea parthenoides;Melitaea parthenoides;1;Melitaea parthenoides;0.999999980399821;
ATLAS474-22;Lepidoptera;Melitaea parthenoides;Melitaea parthenoides;1;Melitaea parthenoides;0.999998904055136;
ATLAS475-22;Lepidoptera;Melitaea parthenoides;Melitaea parthenoides;1;Melitaea parthenoides;0.999999993530452;
ATLAS483-22;Lepidoptera;Minois dryas;NA;0.025;Minois dryas;0.850746026247397;
ATLAS503-22;Lepidoptera;Ochlodes sylvanus;NA;NA;Ochlodes sylvanus;0.996982382831402;
ATLAS512-22;Lepidoptera;Pararge aegeria;Pararge aegeria;1;Pararge aegeria;0.999999999963165;
ATLAS513-22;Lepidoptera;Pararge aegeria;Pararge aegeria;1;Pararge aegeria;0.999999999963165;
ATLAS514-22;Lepidoptera;Pararge aegeria;Pararge aegeria;1;Pararge aegeria;0.999999999963165;
ATLAS528-22;Lepidoptera;Parnassius mnemosyne;Parnassius mnemosyne;1;Parnassius mnemosyne;0.999999269897625;
ATLAS570-22;Lepidoptera;Polygonia c-album;NA;0.215;NA;0.999999192918557;
ATLAS572-22;Lepidoptera;Polygonia egea;Polygonia egea;1;Polygonia egea;0.999999758078475;
ATLAS577-22;Lepidoptera;Polyommatus celina;NA;0.304;Polyommatus celina;0.999999999853685;
ATLAS578-22;Lepidoptera;Polyommatus celina;NA;0.004;Polyommatus celina;0.999999999587999;
ATLAS579-22;Lepidoptera;Polyommatus celina;NA;0.003;Polyommatus celina;0.999999999914678;
ATLAS580-22;Lepidoptera;Polyommatus celina;Polyommatus celina;0.009;Polyommatus celina;0.999999999949694;
ATLAS581-22;Lepidoptera;Polyommatus celina;NA;0.072;Polyommatus celina;0.999999999953559;
ATLAS582-22;Lepidoptera;Polyommatus celina;NA;0.072;Polyommatus celina;0.999999999953559;
ATLAS583-22;Lepidoptera;Polyommatus celina;Polyommatus celina;0.009;Polyommatus celina;0.999999999949694;
ATLAS584-22;Lepidoptera;Polyommatus celina;Polyommatus celina;0.04;Polyommatus celina;0.999999999133223;
ATLAS585-22;Lepidoptera;Polyommatus celina;Polyommatus celina;0.04;Polyommatus celina;0.999999999590585;
ATLAS588-22;Lepidoptera;Polyommatus celina;NA;0.072;Polyommatus celina;0.999999999953559;
ATLAS589-22;Lepidoptera;Polyommatus celina;NA;0.004;Polyommatus celina;0.999999999587999;
ATLAS590-22;Lepidoptera;Polyommatus celina;NA;0.072;Polyommatus celina;0.999999999953559;

ATLAS591-22;Lepidoptera;Polyommatus damon;Polyommatus damon;1;Polyommatus damon;0.999999933777959;
ATLAS592-22;Lepidoptera;Polyommatus damon;Polyommatus damon;1;Polyommatus damon;0.999999887832281;
ATLAS593-22;Lepidoptera;Polyommatus damon;Polyommatus damon;1;Polyommatus damon;0.999998403245054;
ATLAS594-22;Lepidoptera;Polyommatus dolus;Polyommatus dolus;0.002;Polyommatus dolus;0.974976778379616;
ATLAS595-22;Lepidoptera;Polyommatus dorylas;Polyommatus dorylas;0.005;Polyommatus dorylas;0.987518562410738;
ATLAS596-22;Lepidoptera;Polyommatus dorylas;Polyommatus dorylas;0.01;Polyommatus dorylas;0.986535405601042;
ATLAS597-22;Lepidoptera;Polyommatus eros;NA;0.005;Polyommatus eros;0.975783510541378;
ATLAS598-22;Lepidoptera;Polyommatus eros;NA;0.005;Polyommatus eros;0.975783510541378;
ATLAS599-22;Lepidoptera;Polyommatus eros;NA;0.001;Polyommatus eros;0.975240035293052;
ATLAS600-22;Lepidoptera;Polyommatus eros;NA;NA;Polyommatus eros;0.865495727112908;
ATLAS601-22;Lepidoptera;Polyommatus escheri;Polyommatus escheri;1;Polyommatus escheri;0.999999989429227;
ATLAS608-22;Lepidoptera;Polyommatus icarus;NA;0.004;Polyommatus icarus;0.999481683747521;
ATLAS610-22;Lepidoptera;Polyommatus icarus;Polyommatus icarus;0.001;Polyommatus icarus;0.999851522482238;
ATLAS611-22;Lepidoptera;Polyommatus icarus;NA;0.009;Polyommatus icarus;0.99934079538001;
ATLAS612-22;Lepidoptera;Polyommatus icarus;Polyommatus icarus;0.001;Polyommatus icarus;0.999851522482238;
ATLAS613-22;Lepidoptera;Polyommatus icarus;NA;0.001;Polyommatus icarus;0.971665478142064;
ATLAS614-22;Lepidoptera;Polyommatus icarus;Polyommatus icarus;0.001;Polyommatus icarus;0.999851522482238;
ATLAS615-22;Lepidoptera;Polyommatus icarus;NA;0.004;Polyommatus icarus;0.999481683747521;
ATLAS616-22;Lepidoptera;Polyommatus icarus;Polyommatus icarus;0.001;Polyommatus icarus;0.999851522482238;
ATLAS617-22;Lepidoptera;Polyommatus icarus;NA;0.001;Polyommatus icarus;0.971665478142064;
ATLAS618-22;Lepidoptera;Polyommatus icarus;Polyommatus icarus;0.003;Polyommatus icarus;0.999808014773309;
ATLAS619-22;Lepidoptera;Polyommatus icarus;NA;0.004;Polyommatus icarus;0.999481683747521;
ATLAS620-22;Lepidoptera;Polyommatus icarus;Polyommatus icarus;0.001;Polyommatus icarus;0.999848859399272;
ATLAS632-22;Lepidoptera;Polyommatus thersites;Polyommatus thersites;0.999;Polyommatus thersites;0.999999916395897;
ATLAS633-22;Lepidoptera;Polyommatus thersites;Polyommatus thersites;0.001;Polyommatus thersites;0.99999962890986;
ATLAS635-22;Lepidoptera;Pontia callidice;Pontia callidice;1;Pontia callidice;0.999999998637406;
ATLAS690-22;Lepidoptera;Pyrgus alveus;NA;NA;Pyrgus alveus;0.931326683565012;
ATLAS691-22;Lepidoptera;Pyrgus alveus;NA;NA;Pyrgus alveus;0.927306391770666;
ATLAS692-22;Lepidoptera;Pyrgus alveus;NA;NA;Pyrgus alveus;0.917812816884131;
ATLAS693-22;Lepidoptera;Pyrgus alveus;NA;NA;Pyrgus alveus;0.931326683565012;
ATLAS694-22;Lepidoptera;Pyrgus alveus;NA;NA;Pyrgus alveus;0.902591602214502;
ATLAS695-22;Lepidoptera;Pyrgus andromedae;NA;0.001;Pyrgus andromedae;0.975733909551987;
ATLAS696-22;Lepidoptera;Pyrgus andromedae;Pyrgus andromedae;1;Pyrgus andromedae;0.993824993204341;
ATLAS697-22;Lepidoptera;Pyrgus armoricanus;Pyrgus armoricanus;1;Pyrgus armoricanus;0.998895076657973;
ATLAS698-22;Lepidoptera;Pyrgus armoricanus;Pyrgus armoricanus;1;Pyrgus armoricanus;0.999997353848817;
ATLAS699-22;Lepidoptera;Pyrgus armoricanus;Pyrgus armoricanus;1;Pyrgus armoricanus;0.997478680096412;
ATLAS700-22;Lepidoptera;Pyrgus armoricanus;Pyrgus armoricanus;1;Pyrgus armoricanus;0.997478680096412;
ATLAS701-22;Lepidoptera;Pyrgus armoricanus;Pyrgus armoricanus;1;Pyrgus armoricanus;0.997478680096412;
ATLAS702-22;Lepidoptera;Pyrgus armoricanus;Pyrgus armoricanus;1;Pyrgus armoricanus;0.997478680096412;
ATLAS703-22;Lepidoptera;Pyrgus armoricanus;Pyrgus armoricanus;1;Pyrgus armoricanus;0.999989012351076;
ATLAS704-22;Lepidoptera;Pyrgus armoricanus;Pyrgus armoricanus;1;Pyrgus armoricanus;0.999997353848817;
ATLAS705-22;Lepidoptera;Pyrgus armoricanus;Pyrgus armoricanus;1;Pyrgus armoricanus;0.999997353848817;
ATLAS706-22;Lepidoptera;Pyrgus armoricanus;Pyrgus armoricanus;1;Pyrgus armoricanus;0.999997353848817;
ATLAS707-22;Lepidoptera;Pyrgus armoricanus;Pyrgus armoricanus;1;Pyrgus armoricanus;0.999997353848817;
ATLAS710-22;Lepidoptera;Pyrgus malvae;Pyrgus malvae;0.999;Pyrgus malvae;0.993531783407179;
ATLAS711-22;Lepidoptera;Pyrgus malvae;Pyrgus malvae;NA;Pyrgus malvae;0.980121140374433;
ATLAS712-22;Lepidoptera;Pyrgus malvae;Pyrgus malvae;NA;Pyrgus malvae;0.980121140374433;
ATLAS713-22;Lepidoptera;Pyrgus malvae;Pyrgus malvae;NA;Pyrgus malvae;0.979843680166459;
ATLAS714-22;Lepidoptera;Pyrgus malvoides;NA;0.076;Pyrgus malvoides;0.887854315566122;
ATLAS715-22;Lepidoptera;Pyrgus malvoides;NA;0.076;Pyrgus malvoides;0.887854315566122;
ATLAS716-22;Lepidoptera;Pyrgus malvoides;NA;0.076;Pyrgus malvoides;0.887854315566122;

ATLAS717-22;Lepidoptera;Pyrgus malvoides;NA;0.176;Pyrgus malvoides;0.940490890379026;
ATLAS718-22;Lepidoptera;Pyrgus malvoides;NA;0.111;Pyrgus malvoides;0.968399374774442;
ATLAS719-22;Lepidoptera;Pyrgus malvoides;NA;0.001;Pyrgus malvoides;0.864662411153201;
ATLAS720-22;Lepidoptera;Pyrgus malvoides;NA;0.076;Pyrgus malvoides;0.887854315566122;
ATLAS721-22;Lepidoptera;Pyrgus malvoides;NA;0.076;Pyrgus malvoides;0.887854315566122;
ATLAS722-22;Lepidoptera;Pyrgus malvoides;NA;0.041;Pyrgus malvoides;0.932850168236208;
ATLAS723-22;Lepidoptera;Pyrgus onopordi;Pyrgus onopordi;1;Pyrgus onopordi;0.999994513194469;
ATLAS724-22;Lepidoptera;Pyrgus onopordi;Pyrgus onopordi;1;Pyrgus onopordi;0.999997111229755;
ATLAS730-22;Lepidoptera;Pyronia cecilia;Pyronia cecilia;NA;Pyronia cecilia;1;
ATLAS732-22;Lepidoptera;Pyronia tithonus;Pyronia tithonus;1;Pyronia tithonus;1;
ATLAS733-22;Lepidoptera;Satyrium esculi;Satyrium esculi;1;Satyrium esculi;0.999999957367948;
ATLAS734-22;Lepidoptera;Satyrium pruni;NA;0.076;Satyrium pruni;0.990322364021655;
ATLAS735-22;Lepidoptera;Satyrium spini;Satyrium spini;1;Satyrium spini;0.999998560564576;
ATLAS736-22;Lepidoptera;Satyrium spini;Satyrium spini;1;Satyrium spini;0.999999721054916;
ATLAS737-22;Lepidoptera;Satyrus ferula;Satyrus ferula;0.001;Satyrus ferula;0.989623920673219;
ATLAS738-22;Lepidoptera;Argynnis aglaja;NA;0.001;NA;0.999999999999586;
ATLAS739-22;Lepidoptera;Argynnis aglaja;NA;NA;NA;0.999999999998051;
ATLAS741-22;Lepidoptera;Spialia sertorius;Spialia sertorius;1;Spialia sertorius;0.99999994250456;
ATLAS744-22;Lepidoptera;Spialia sertorius;Spialia sertorius;1;Spialia sertorius;0.999999932771518;
ATLAS745-22;Lepidoptera;Spialia sertorius;Spialia sertorius;1;Spialia sertorius;0.99999994250456;
ATLAS753-22;Lepidoptera;Spialia sertorius;Spialia sertorius;1;Spialia sertorius;0.999999957643241;
ATLAS755-22;Lepidoptera;Spialia sertorius;Spialia sertorius;1;Spialia sertorius;0.999999975508246;
ATLAS756-22;Lepidoptera;Spialia sertorius;Spialia sertorius;1;Spialia sertorius;0.999999926892772;
ATLAS759-22;Lepidoptera;Spialia sertorius;Spialia sertorius;1;Spialia sertorius;0.999999957643241;
ATLAS763-22;Lepidoptera;Spialia sertorius;Spialia sertorius;1;Spialia sertorius;0.999999932771518;
ATLAS766-22;Lepidoptera;Thecla betulae;Thecla betulae;1;Thecla betulae;0.999999999972886;
ATLAS768-22;Lepidoptera;Thymelicus acteon;Thymelicus acteon;1;Thymelicus acteon;0.999999962833841;
ATLAS769-22;Lepidoptera;Thymelicus acteon;Thymelicus acteon;1;Thymelicus acteon;0.999999976833664;
ATLAS779-22;Lepidoptera;Thymelicus lineola;Thymelicus lineola;0.002;Thymelicus lineola;0.999992153884563;
ATLAS780-22;Lepidoptera;Thymelicus sylvestris;Thymelicus sylvestris;1;Thymelicus sylvestris;0.999999985935205;
ATLAS789-22;Lepidoptera;Vanessa atalanta;Vanessa atalanta;1;Vanessa atalanta;0.999999999757762;
ATLAS791-22;Lepidoptera;Vanessa atalanta;Vanessa atalanta;1;Vanessa atalanta;0.999999999669399;
ATLAS792-22;Lepidoptera;Vanessa atalanta;Vanessa atalanta;1;Vanessa atalanta;0.999999999793403;
ATLAS793-22;Lepidoptera;Vanessa atalanta;Vanessa atalanta;1;Vanessa atalanta;0.999999999725759;
ATLAS794-22;Lepidoptera;Vanessa atalanta;Vanessa atalanta;1;Vanessa atalanta;0.999999999793403;
ATLAS795-22;Lepidoptera;Vanessa atalanta;Vanessa atalanta;1;Vanessa atalanta;0.999999999381544;
ATLAS796-22;Lepidoptera;Vanessa atalanta;Vanessa atalanta;1;Vanessa atalanta;0.999999999790589;
ATLAS797-22;Lepidoptera;Vanessa atalanta;Vanessa atalanta;1;Vanessa atalanta;0.999999996750603;
ATLAS798-22;Lepidoptera;Vanessa cardui;Vanessa cardui;1;Vanessa cardui;0.999999459983318;
ATLAS799-22;Lepidoptera;Vanessa cardui;Vanessa cardui;1;Vanessa cardui;0.999999116402754;
ATLAS800-22;Lepidoptera;Vanessa cardui;Vanessa cardui;1;Vanessa cardui;0.999999803450484;
ATLAS805-22;Lepidoptera;Vanessa cardui;Vanessa cardui;1;Vanessa cardui;0.999999459983318;
ATLAS806-22;Lepidoptera;Vanessa cardui;Vanessa cardui;1;Vanessa cardui;0.999999803450484;
ATLAS808-22;Lepidoptera;Vanessa cardui;Vanessa cardui;1;Vanessa cardui;0.999999427581593;
AY346221;Lepidoptera;Erebia euryale;NA;NA;NA;0.871436907054897;
AY346223;Lepidoptera;Erebia meolans;Erebia meolans;NA;NA;0.822853759048436;
AY346225;Lepidoptera;Erebia epiphron;NA;NA;NA;0.898369892379513;
AY346226;Lepidoptera;Erebia pandrose;NA;0.045;NA;0.870510983426716;
AY346227;Lepidoptera;Erebia oeme;Erebia oeme;0.003;NA;0.841979908687587;
AY346230;Lepidoptera;Coenonympha arcania;Coenonympha arcania;0.004;NA;0.863693349039585;
AY346231;Lepidoptera;Coenonympha glycerion;Coenonympha glycerion;1;NA;0.836719866219981;
AY346232;Lepidoptera;Coenonympha pamphilus;Coenonympha pamphilus;1;NA;0.83509472629056;
AY346233;Lepidoptera;Maniola jurtina;NA;0.009;Maniola megala;0.84548447398376;
AY346251;Lepidoptera;Erebia triaria;NA;NA;Erebia triaria;0.817907988878753;
AY346252;Lepidoptera;Arethusana arethusa;Arethusana arethusa;1;Arethusana arethusa;0.99994982536517;
AY346253;Lepidoptera;Aphantopus hyperantus;NA;0.102;Aphantopus hyperantus;0.9990049472058;
AY346254;Lepidoptera;Parnassius apollo;Parnassius apollo;1;Parnassius apollo;0.989969291602239;
AY346255;Lepidoptera;Maniola tithonus;Pyronia tithonus;1;Pyronia tithonus;0.999999999836604;
AY350455;Lepidoptera;Erebia meolans;Erebia meolans;NA;NA;0.822053333153082;
AY350456;Lepidoptera;Aricia agestis;NA;0.001;NA;0.876484833993336;
AY350457;Lepidoptera;Plebejus argus;Plebejus argus;0.016;NA;0.891200580206937;
AY350458;Lepidoptera;Plebejus argus;Plebejus argus;0.016;NA;0.901251123961532;
AY350459;Lepidoptera;Plebejus argus;Plebejus argus;0.016;NA;0.889897037311938;
AY350462;Lepidoptera;Erebia triaria;NA;0.122;NA;0.999999999266715;

AY556855;Lepidoptera;Polyommatus escheri;Polyommatus escheri;1;Polyommatus escheri;0.999957262319021;
AY556862;Lepidoptera;Aricia artaxerxes;NA;0.064;Aricia artaxerxes;0.999307805158988;
AY556863;Lepidoptera;Aricia artaxerxes;NA;0.052;Aricia agestis;0.985516448333493;
AY556865;Lepidoptera;Aricia agestis;NA;0.129;Aricia agestis;0.964968588620611;
AY556942;Lepidoptera;Polyommatus escheri;Polyommatus escheri;1;Polyommatus escheri;0.999999722390992;
AY556945;Lepidoptera;Lysandra coridon;NA;NA;NA;0.997738326705677;
AY556946;Lepidoptera;Polyommatus dorylas;Polyommatus dorylas;0.016;Polyommatus dorylas;0.985037180771894;
AY556947;Lepidoptera;Lampides boeticus;Lampides boeticus;1;Lampides boeticus;0.999999990742936;
AY556949;Lepidoptera;Polyommatus icarus;Polyommatus icarus;0.003;Polyommatus icarus;0.978006664750656;
AY556950;Lepidoptera;Satyrium esculi;Satyrium esculi;1;Satyrium esculi;0.99999991992124;
AY556953;Lepidoptera;Aricia agestis;NA;0.046;Aricia montensis;0.998635282041986;
AY556956;Lepidoptera;Aricia cramera;Aricia cramera;1;Aricia cramera;0.99997650674376;
AY556961;Lepidoptera;Lysandra coridon;NA;NA;Lysandra hispana;0.90983622858907;
AY556965;Lepidoptera;Lysandra coridon;NA;NA;NA;0.954573556903198;
AY556979;Lepidoptera;Cupido osiris;Cupido osiris;1;Cupido osiris;0.999903158680877;
AY556983;Lepidoptera;Polyommatus daphnis;Polyommatus daphnis;0.999;Polyommatus daphnis;0.727435480614624;
AY556988;Lepidoptera;Lycaena thersamon;NA;0.359;Lycaena thersamon;0.967121964182512;
AY556992;Lepidoptera;Polyommatus dorylas;Polyommatus dorylas;0.001;Polyommatus dorylas;0.992051531812644;
AY556994;Lepidoptera;Polyommatus icarus;NA;0.002;NA;0.999998064332033;
AY557011;Lepidoptera;Lycaena tityrus;Lycaena tityrus;1;Lycaena tityrus;0.999985055494962;
AY557016;Lepidoptera;Lampides boeticus;Lampides boeticus;1;Lampides boeticus;0.999999964282325;
AY557018;Lepidoptera;Polyommatus daphnis;Polyommatus daphnis;0.996;Polyommatus daphnis;0.929450889194657;
AY557029;Lepidoptera;Polyommatus dorylas;Polyommatus dorylas;1;Polyommatus dorylas;0.995684102725221;
AY557031;Lepidoptera;Aricia agestis;NA;0.065;Aricia agestis;0.989063108666142;
AY557034;Lepidoptera;Maculinea arion;NA;0.045;NA;0.999999996881548;
AY557035;Lepidoptera;Polyommatus amandus;Polyommatus amandus;0.007;Polyommatus amandus;0.999697391945753;
AY557044;Lepidoptera;Celastrina argiolus;Celastrina argiolus;0.999;Celastrina argiolus;0.992752024611529;
AY557053;Lepidoptera;Aricia eumedon;Aricia eumedon;0.001;Aricia eumedon;0.999936680633228;
AY557115;Lepidoptera;Lycaena virgaureae;NA;0.225;Lycaena virgaureae;0.98729247149122;
AY557121;Lepidoptera;Polyommatus damon;Polyommatus damon;1;Polyommatus damon;0.999996415198725;
AY557126;Lepidoptera;Polyommatus eros;NA;0.002;NA;0.999980855413135;
AY557127;Lepidoptera;Polyommatus humedasae;Polyommatus humedasae;1;Polyommatus humedasae;0.999488547236434;
AY557128;Lepidoptera;Polyommatus humedasae;Polyommatus humedasae;1;Polyommatus humedasae;0.99877461416058;
AY557130;Lepidoptera;Lysandra coridon;Lysandra coridon;0.001;NA;0.799193154522841;
AY557131;Lepidoptera;Polyommatus damon;Polyommatus damon;1;Polyommatus damon;0.999989506599664;
AY557133;Lepidoptera;Lysandra coridon gennargenti;NA;NA;NA;0.998624971615247;
AY585886;Lepidoptera;Carterocephalus palaemon;NA;0.038;NA;0.999999997282839;
AY585887;Lepidoptera;Carterocephalus palaemon;NA;0.038;NA;0.999999997282839;
AY585889;Lepidoptera;Carterocephalus palaemon;NA;0.037;NA;0.999999994913945;
AY585890;Lepidoptera;Carterocephalus palaemon;NA;0.038;NA;0.999999997282839;
AY585891;Lepidoptera;Carterocephalus palaemon;NA;0.038;NA;0.999999997282839;
AY675402;Lepidoptera;Maculinea arion;NA;0.001;NA;0.751343203187268;
AY675406;Lepidoptera;Maculinea alcon;NA;0.201;NA;0.761398063497889;
AY675407;Lepidoptera;Maculinea arion;NA;0.002;NA;0.721737654212504;
AY675413;Lepidoptera;Glaucopsyche alexis;NA;0.001;NA;0.723075189423239;
AY675414;Lepidoptera;Iolana iolas;NA;NA;NA;0.786125520931428;
AY675415;Lepidoptera;Maculinea rebeli;NA;0.298;NA;0.741235044277175;
AY675417;Lepidoptera;Maculinea alcon;NA;0.126;NA;0.812865253726509;
AY675418;Lepidoptera;Maculinea teleius;NA;0.001;NA;0.834938625273714;
AY675422;Lepidoptera;Maculinea arion;NA;0.001;NA;0.82099423712478;
AY675428;Lepidoptera;Maculinea teleius;NA;NA;NA;0.730449573632859;
AY675429;Lepidoptera;Maculinea teleius;NA;0.001;NA;0.728492652184826;
AY675430;Lepidoptera;Maculinea alcon;NA;0.119;NA;0.707211510265174;
AY675431;Lepidoptera;Maculinea alcon;NA;0.114;NA;0.750606888784308;
AY675432;Lepidoptera;Maculinea arion;NA;0.066;NA;0.706297269727053;
AY675433;Lepidoptera;Maculinea arion;NA;NA;NA;0.776239577469599;
AY675434;Lepidoptera;Maculinea rebeli;NA;0.126;NA;0.707791312282221;
AY675435;Lepidoptera;Maculinea rebeli;NA;0.001;NA;0.727473166227636;
AY675436;Lepidoptera;Maculinea alcon;NA;0.278;NA;0.999999999916248;
AY675445;Lepidoptera;Maculinea arion;NA;0.001;NA;0.744975014406251;
AY675447;Lepidoptera;Maculinea arion;NA;0.001;NA;0.999999999986271;
AY675448;Lepidoptera;Maculinea arion;NA;0.001;NA;0.737076953517932;
BCLEP122-17;Lepidoptera;Pyronia tithonus;Pyronia tithonus;1;Pyronia tithonus;0.999999999999972;
BDE009-18;Lepidoptera;Euphydryas aurinia;NA;0.001;Euphydryas aurinia;0.995576749826865;
BDE010-18;Lepidoptera;Parnassius mnemosyne;Parnassius mnemosyne;1;Parnassius mnemosyne;0.999999761646734;

BDE011-18;Lepidoptera;Euphydryas aurinia;Euphydryas aurinia;0.02;Euphydryas aurinia;0.99668249939786;
BDE015-18;Lepidoptera;Euphydryas aurinia;Euphydryas aurinia;0.02;Euphydryas aurinia;0.99668249939786;
BDE016-18;Lepidoptera;Parnassius mnemosyne;Parnassius mnemosyne;1;Parnassius mnemosyne;0.999999600553986;
BDE017-18;Lepidoptera;Euphydryas aurinia;Euphydryas aurinia;0.012;Euphydryas aurinia;0.997780875235948;
BDE018-18;Lepidoptera;Euphydryas aurinia;NA;0.31;Euphydryas aurinia;0.993387708761386;
BDE021-18;Lepidoptera;Euphydryas aurinia;NA;0.008;Euphydryas aurinia;0.997216897249492;
BDE023-18;Lepidoptera;Euphydryas aurinia;NA;0.001;Euphydryas aurinia;0.995576749826865;
BDE025-18;Lepidoptera;Euphydryas aurinia;NA;0.31;Euphydryas aurinia;0.993387708761386;
BDE027-18;Lepidoptera;Euphydryas aurinia;NA;0.001;Euphydryas aurinia;0.995576749826865;
BDE028-18;Lepidoptera;Parnassius mnemosyne;Parnassius mnemosyne;1;Parnassius mnemosyne;0.99999987497234;
BDE029-18;Lepidoptera;Parnassius mnemosyne;Parnassius mnemosyne;1;Parnassius mnemosyne;0.99999987497234;
BDE030-18;Lepidoptera;Euphydryas aurinia;Euphydryas aurinia;0.016;Euphydryas aurinia;0.997751503369639;
BDE031-18;Lepidoptera;Euphydryas aurinia;Euphydryas aurinia;0.016;Euphydryas aurinia;0.997751503369639;
BDE036-18;Lepidoptera;Euphydryas aurinia;NA;0.31;Euphydryas aurinia;0.993387708761386;
BDE037-18;Lepidoptera;Euphydryas aurinia;NA;0.31;Euphydryas aurinia;0.993387708761386;
BDE038-18;Lepidoptera;Parnassius mnemosyne;Parnassius mnemosyne;1;Parnassius mnemosyne;0.999999761646734;
BDE039-18;Lepidoptera;Parnassius mnemosyne;Parnassius mnemosyne;1;Parnassius mnemosyne;0.999999761646734;
BDE042-18;Lepidoptera;Parnassius mnemosyne;Parnassius mnemosyne;1;Parnassius mnemosyne;0.999999416965636;
BDE043-18;Lepidoptera;Parnassius mnemosyne;Parnassius mnemosyne;1;Parnassius mnemosyne;0.999999416965636;
BDE045-18;Lepidoptera;Parnassius mnemosyne;Parnassius mnemosyne;1;Parnassius mnemosyne;0.999999416965636;
BDE046-18;Lepidoptera;Parnassius mnemosyne;Parnassius mnemosyne;1;Parnassius mnemosyne;0.999999131309189;
BDE048-18;Lepidoptera;Parnassius mnemosyne;Parnassius mnemosyne;1;Parnassius mnemosyne;0.999996958841953;
BDE049-18;Lepidoptera;Parnassius mnemosyne;Parnassius mnemosyne;1;Parnassius mnemosyne;0.999998906020325;
BDE050-18;Lepidoptera;Parnassius mnemosyne;Parnassius mnemosyne;1;Parnassius mnemosyne;0.999999416965636;
BDE051-18;Lepidoptera;Parnassius mnemosyne;Parnassius mnemosyne;1;Parnassius mnemosyne;0.999999416965636;
BDE052-18;Lepidoptera;Parnassius mnemosyne;Parnassius mnemosyne;0.999;Parnassius mnemosyne;0.999999453050897;
BDE054-18;Lepidoptera;Parnassius mnemosyne;Parnassius mnemosyne;1;Parnassius mnemosyne;0.999999416965636;
BDE055-18;Lepidoptera;Parnassius mnemosyne;Parnassius mnemosyne;1;Parnassius mnemosyne;0.999999416965636;
BDE057-18;Lepidoptera;Parnassius mnemosyne;Parnassius mnemosyne;1;Parnassius mnemosyne;0.999999416965636;
BDE062-18;Lepidoptera;Parnassius mnemosyne;Parnassius mnemosyne;1;Parnassius mnemosyne;0.999999761646734;
BDE1039-22;Lepidoptera;Gonepteryx rhamni;Gonepteryx rhamni;1;Gonepteryx rhamni;0.99964616790002;
BDE1040-22;Lepidoptera;Gonepteryx rhamni;Gonepteryx rhamni;1;Gonepteryx rhamni;0.99964616790002;
BDE1041-22;Lepidoptera;Gonepteryx rhamni;NA;0.474;Gonepteryx rhamni;0.982076396127858;
BDE1042-22;Lepidoptera;Gonepteryx rhamni;NA;0.474;Gonepteryx rhamni;0.982076396127858;
BDE1043-22;Lepidoptera;Gonepteryx rhamni;Gonepteryx rhamni;1;Gonepteryx rhamni;0.99964616790002;
BDE1044-22;Lepidoptera;Gonepteryx rhamni;Gonepteryx rhamni;1;Gonepteryx rhamni;0.99964616790002;
BDE1045-22;Lepidoptera;Gonepteryx rhamni;Gonepteryx rhamni;1;Gonepteryx rhamni;0.99964616790002;
BDE1046-22;Lepidoptera;Gonepteryx rhamni;NA;0.474;Gonepteryx rhamni;0.982076396127858;
BDE1047-22;Lepidoptera;Gonepteryx rhamni;NA;0.474;Gonepteryx rhamni;0.982076396127858;
BDE1048-22;Lepidoptera;Gonepteryx rhamni;Gonepteryx rhamni;1;Gonepteryx rhamni;0.999850170258883;
BDE1049-22;Lepidoptera;Gonepteryx rhamni;Gonepteryx rhamni;1;Gonepteryx rhamni;0.99964616790002;
BDE1050-22;Lepidoptera;Gonepteryx rhamni;Gonepteryx rhamni;1;Gonepteryx rhamni;0.99964616790002;
BDE206-19;Lepidoptera;Aglais urticae;NA;0.002;Aglais urticae;0.987497593219019;
BDE207-19;Lepidoptera;Anthocharis euphenoides;NA;0.172;Anthocharis euphenoides;0.943741664916385;
BDE208-19;Lepidoptera;Aphantopus hyperantus;Aphantopus hyperantus;0.003;Aphantopus hyperantus;0.999999455871523;
BDE209-19;Lepidoptera;Argynnis adippe;Fabriciana adippe;1;Fabriciana adippe;0.999722411724441;
BDE210-19;Lepidoptera;Argynnis aglaja;NA;NA;NA;0.999999999998776;
BDE211-19;Lepidoptera;Aricia cramera;NA;0.001;Aricia montensis;0.99557916540038;
BDE213-19;Lepidoptera;Boloria dia;Boloria dia;1;Boloria dia;0.999999999980503;
BDE215-19;Lepidoptera;Brenthis daphne;Brenthis daphne;0.001;Brenthis daphne;0.997781825264304;
BDE217-19;Lepidoptera;Brenthis ino;NA;0.061;Brenthis ino;0.791997215074462;
BDE219-19;Lepidoptera;Boloria euphrosyne;NA;NA;Boloria euphrosyne;0.999936593845194;

BDE222-19;Lepidoptera;Coenonympha arcania;NA;0.022;Coenonympha arcania;0.972706499739185;
BDE223-19;Lepidoptera;Coenonympha dorus;Coenonympha dorus;1;Coenonympha dorus;0.999999999915957;
BDE224-19;Lepidoptera;Coenonympha glycerion;NA;0.055;Coenonympha glycerion;0.99999566036386;
BDE225-19;Lepidoptera;Colias alfacariensis;Colias alfacariensis;0.001;Colias alfacariensis;0.999857790810452;
BDE226-19;Lepidoptera;Colias phicomone;NA;0.064;Colias phicomone;0.992122721786916;
BDE227-19;Lepidoptera;Cupido argiades;NA;0.498;NA;0.999999999965209;
BDE228-19;Lepidoptera;Cupido minimus;NA;0.001;Cupido minimus;0.999751488575214;
BDE230-19;Lepidoptera;Erebia epiphron;NA;NA;Erebia euryale;0.93493911427573;
BDE231-19;Lepidoptera;Erebia euryale;NA;NA;Erebia euryale;0.902406174360953;
BDE233-19;Lepidoptera;Erebia manto;NA;0.091;Erebia manto;0.99940430793667;
BDE234-19;Lepidoptera;Erebia meolans;Erebia meolans;NA;Erebia meolans;0.999670624550747;
BDE236-19;Lepidoptera;Erebia triaria;NA;0.051;Erebia triarius;0.840521161239895;
BDE238-19;Lepidoptera;Glaucopsyche melanops;Glaucopsyche melanops;1;Glaucopsyche melanops;0.999860230433135;
BDE239-19;Lepidoptera;Gonepteryx cleopatra;Gonepteryx cleopatra;0.998;Gonepteryx cleopatra;0.999118636582266;
BDE240-19;Lepidoptera;Hamearis lucina;NA;NA;Hamearis lucina;0.999999999979167;
BDE243-19;Lepidoptera;Hipparchia semele;NA;0.003;Hipparchia semele;0.855676008628351;
BDE246-19;Lepidoptera;Brintesia circe;Brintesia circe;1;Brintesia circe;0.9999954171695;
BDE249-19;Lepidoptera;Lycaena tityrus;Lycaena tityrus;1;Lycaena tityrus;0.999997855580031;
BDE250-19;Lepidoptera;Lycaena virgaureae;NA;0.176;Lycaena virgaureae;0.999417010436143;
BDE251-19;Lepidoptera;Melanargia galathea;Melanargia galathea;0.009;Melanargia galathea;0.985492042252146;
BDE255-19;Lepidoptera;Melitaea cinxia;Melitaea cinxia;1;Melitaea cinxia;0.999999999999602;
BDE256-19;Lepidoptera;Parnassius apollo;Parnassius apollo;1;Parnassius apollo;0.997639119991203;
BDE258-19;Lepidoptera;Pieris napi;NA;NA;Pieris napi;0.744610646821454;
BDE259-19;Lepidoptera;Plebejus argus;Plebejus argus;0.014;Plebejus argus;0.979849220658559;
BDE260-19;Lepidoptera;Plebejus idas;NA;0.007;NA;0.999546324331549;
BDE261-19;Lepidoptera;Polygonia c-album;NA;0.215;NA;0.999999192918557;
BDE262-19;Lepidoptera;Polyommatus dorylas;Polyommatus dorylas;0.025;Polyommatus dorylas;0.973229109265438;
BDE267-19;Lepidoptera;Satyrium acaciae;Satyrium acaciae;1;Satyrium acaciae;0.99999991343697;
BDE268-19;Lepidoptera;Satyrium esculi;Satyrium esculi;1;Satyrium esculi;0.999999957210833;
BDE269-19;Lepidoptera;Satyrium ilicis;Satyrium ilicis;1;Satyrium ilicis;0.999999981931609;
BDE270-19;Lepidoptera;Satyrium spini;Satyrium spini;1;Satyrium spini;0.999998173309697;
BDE271-19;Lepidoptera;Spialia sertorius;Spialia sertorius;1;Spialia sertorius;0.999999984019468;
BDE273-19;Lepidoptera;Favonius quercus;NA;NA;NA;0.999999999996165;
BDE299-19;Lepidoptera;Anthocharis euphenoides;NA;0.048;Anthocharis euphenoides;0.964164577868293;
BDE301-19;Lepidoptera;Argynnis aglaja;NA;0.005;Speyeria aglaja;0.896438572419641;
BDE302-19;Lepidoptera;Erebia triaria;NA;0.182;NA;1;
BDE310-19;Lepidoptera;Satyrium esculi;Satyrium esculi;1;Satyrium esculi;0.999999957367948;
BDE313-19;Lepidoptera;Coenonympha dorus;Coenonympha dorus;1;Coenonympha dorus;0.99999999988259;
BDE317-19;Lepidoptera;Satyrium spini;Satyrium spini;1;Satyrium spini;0.999987432167351;
BDE321-19;Lepidoptera;Lasiommata megera;Lasiommata megera;1;Lasiommata megera;0.999928528239676;
BDE322-19;Lepidoptera;Maniola jurtina;NA;0.001;Maniola jurtina;0.939220105860875;
BDE323-19;Lepidoptera;Pieris napi;NA;0.011;Pieris napi;0.801985394724814;
BDE326-19;Lepidoptera;Pyronia cecilia;Pyronia cecilia;1;Pyronia cecilia;1;
BDE327-19;Lepidoptera;Hipparchia semele;NA;0.001;Hipparchia semele;0.802446092830354;
BDE328-19;Lepidoptera;Colias alfacariensis;Colias alfacariensis;0.001;Colias alfacariensis;0.999857790810452;
BDE331-19;Lepidoptera;Polygonia c-album;NA;0.215;NA;0.999999192918557;
BDE332-19;Lepidoptera;Pyronia cecilia;Pyronia cecilia;1;Pyronia cecilia;1;
BDE333-19;Lepidoptera;Polyommatus celina;NA;0.098;Polyommatus celina;0.999999999912802;
BDE334-19;Lepidoptera;Pieris napi;NA;0.028;Pieris napi;0.888205720664699;
BDE335-19;Lepidoptera;Satyrium esculi;Satyrium esculi;1;Satyrium esculi;0.999999957367948;
BDE336-19;Lepidoptera;Gonepteryx rhamni;NA;0.271;Gonepteryx rhamni;0.99904249910688;
BDE338-19;Lepidoptera;Gonepteryx rhamni;Gonepteryx rhamni;1;Gonepteryx rhamni;0.999850170258883;
BDE340-19;Lepidoptera;Pyronia tithonus;Pyronia tithonus;NA;Pyronia tithonus;1;
BDE341-19;Lepidoptera;Pyronia cecilia;Pyronia cecilia;NA;Pyronia cecilia;1;
BDE342-19;Lepidoptera;Satyrium esculi;Satyrium esculi;1;Satyrium esculi;0.999999957367948;
BDE343-19;Lepidoptera;Polyommatus celina;Polyommatus celina;0.012;Polyommatus celina;0.999999999936477;
BDE348-19;Lepidoptera;Gonepteryx cleopatra;Gonepteryx cleopatra;0.001;Gonepteryx cleopatra;0.998922340478632;
BDE349-19;Lepidoptera;Satyrium esculi;Satyrium esculi;1;Satyrium esculi;0.999999957367948;
BDE351-19;Lepidoptera;Melitaea nevadensis;Melitaea celadussa;0.999;Melitaea celadussa;0.998819206090668;
BDE352-19;Lepidoptera;Gonepteryx rhamni;Gonepteryx rhamni;1;Gonepteryx rhamni;0.999850170258883;
BDE353-19;Lepidoptera;Polygonia c-album;NA;0.214;NA;0.999999114245056;
BDE354-19;Lepidoptera;Polyommatus icarus;Polyommatus icarus;0.001;Polyommatus icarus;0.999851522482238;
BDE357-19;Lepidoptera;Coenonympha dorus;Coenonympha dorus;1;Coenonympha dorus;0.999999999259217;
BDE358-19;Lepidoptera;Pyronia tithonus;Pyronia tithonus;1;Pyronia tithonus;1;
BDE360-19;Lepidoptera;Polyommatus celina;Polyommatus celina;0.009;Polyommatus celina;0.999999999949694;
BDE362-19;Lepidoptera;Coenonympha dorus;Coenonympha dorus;1;Coenonympha dorus;0.99999999988259;

BDE363-19;Lepidoptera;Hipparchia statilinus;Hipparchia statilinus;1;Hipparchia statilinus;0.999999999999801;
BDE364-19;Lepidoptera;Polyommatus celina;NA;0.072;Polyommatus celina;0.999999999953559;
BDE365-19;Lepidoptera;Hipparchia statilinus;Hipparchia statilinus;1;Hipparchia statilinus;0.999999999999318;
BDE368-19;Lepidoptera;Polyommatus celina;NA;0.025;Polyommatus celina;0.999999999946397;
BDE371-19;Lepidoptera;Polyommatus celina;NA;0.067;Polyommatus celina;0.999999999867015;
BDE372-19;Lepidoptera;Polyommatus celina;Polyommatus celina;0.012;Polyommatus celina;0.999999999969816;
BDE373-19;Lepidoptera;Pyronia cecilia;Pyronia cecilia;1;Pyronia cecilia;0.999999999999972;
BDE374-19;Lepidoptera;Hipparchia statilinus;Hipparchia statilinus;1;Hipparchia statilinus;0.999999999999801;
BDE375-19;Lepidoptera;Polyommatus celina;NA;0.072;Polyommatus celina;0.999999999953559;
BDE376-19;Lepidoptera;Pyronia cecilia;Pyronia cecilia;1;Pyronia cecilia;1;
BDE378-19;Lepidoptera;Hipparchia statilinus;Hipparchia statilinus;1;Hipparchia statilinus;0.999999999999545;
BDE384-19;Lepidoptera;Pyronia tithonus;Pyronia tithonus;1;Pyronia tithonus;1;
BDE386-19;Lepidoptera;Minois dryas;NA;0.025;Minois dryas;0.850746026247397;
BDE389-19;Lepidoptera;Gonepteryx rhamni;Gonepteryx rhamni;0.008;Gonepteryx rhamni;0.999576796993255;
BDE390-19;Lepidoptera;Pieris napi;NA;NA;Pieris napi;0.860244891707633;
BDE391-19;Lepidoptera;Lycaena tityrus;Lycaena tityrus;1;Lycaena tityrus;0.999997855580031;
BDE392-19;Lepidoptera;Pyronia tithonus;Pyronia tithonus;1;Pyronia tithonus;1;
BDE393-19;Lepidoptera;Polygonia c-album;NA;0.215;NA;0.999999192918557;
BDE397-19;Lepidoptera;Araschnia levana;Araschnia levana;1;Araschnia levana;0.999999772187832;
BDE399-19;Lepidoptera;Aricia agestis;NA;0.067;Aricia agestis;0.999509743227786;
BDE400-19;Lepidoptera;Pyronia tithonus;Pyronia tithonus;1;Pyronia tithonus;0.999999999999972;
BDE403-19;Lepidoptera;Melitaea trivia;Melitaea trivia;1;Melitaea trivia;0.999999999923034;
BDE404-19;Lepidoptera;Pieris napi;NA;NA;Pieris napi;0.783894617328234;
BDE405-19;Lepidoptera;Lasiommata megera;Lasiommata megera;1;Lasiommata megera;0.999928528239676;
BDE406-19;Lepidoptera;Maniola jurtina;NA;0.014;Maniola jurtina;0.792979225803034;
BDE407-19;Lepidoptera;Lasiommata maera;Lasiommata maera;1;Lasiommata maera;0.999997945450226;
BDE408-19;Lepidoptera;Hipparchia fidia;Hipparchia fidia;1;Hipparchia fidia;1;
BDE409-19;Lepidoptera;Hipparchia fidia;Hipparchia fidia;1;Hipparchia fidia;1;
BDE410-19;Lepidoptera;Hipparchia hermione;Hipparchia hermione;1;Hipparchia hermione;0.999950501544645;
BDE412-19;Lepidoptera;Pyronia tithonus;Pyronia tithonus;1;Pyronia tithonus;1;
BDE413-19;Lepidoptera;Satyrus actaea;Satyrus actaea;0.002;Satyrus actaea;0.995392408098466;
BDE414-19;Lepidoptera;Pyronia tithonus;Pyronia tithonus;1;Pyronia tithonus;1;
BDE416-19;Lepidoptera;Polyommatus icarus;NA;0.004;Polyommatus icarus;0.999481683747521;
BDE419-19;Lepidoptera;Coenonympha pamphilus;Coenonympha pamphilus;1;Coenonympha pamphilus;0.999999999999744;
BDE420-19;Lepidoptera;Brintesia circe;Brintesia circe;1;Brintesia circe;0.999995749349661;
BDE421-19;Lepidoptera;Colias crocea;NA;NA;0.999999999999712;
BDE422-19;Lepidoptera;Satyrium esculi;Satyrium esculi;1;Satyrium esculi;0.999999957367948;
BDE424-19;Lepidoptera;Lampides boeticus;Lampides boeticus;1;Lampides boeticus;0.999999999637112;
BDE427-19;Lepidoptera;Hipparchia hermione;Hipparchia hermione;1;Hipparchia hermione;0.99988238204256;
BDE430-19;Lepidoptera;Gonepteryx rhamni;Gonepteryx rhamni;0.003;Gonepteryx rhamni;0.999853350566569;
BDE431-19;Lepidoptera;Hipparchia semele;NA;0.005;Hipparchia semele;0.811250086847141;
BDE432-19;Lepidoptera;Satyrium ilicis;Satyrium ilicis;1;Satyrium ilicis;0.999999985717665;
BDE433-19;Lepidoptera;Satyrium spini;Satyrium esculi;1;Satyrium esculi;0.999999957367948;
BDE434-19;Lepidoptera;Lycaena tityrus;Lycaena bleusei;1;Lycaena bleusei;0.999986494424373;
BDE435-19;Lepidoptera;Pyronia tithonus;Pyronia tithonus;1;Pyronia tithonus;1;
BDE437-19;Lepidoptera;Melitaea didyma;Melitaea didyma;NA;Melitaea didyma;0.980272836986251;
BDE440-19;Lepidoptera;Colias crocea;NA;0.059;NA;0.999999999999738;
BDE444-19;Lepidoptera;Pyronia cecilia;Pyronia cecilia;NA;Pyronia cecilia;1;
BDE446-19;Lepidoptera;Melitaea didyma;Melitaea didyma;1;Melitaea didyma;0.996477093809569;
BDE447-19;Lepidoptera;Lycaena virgaureae;NA;0.113;Lycaena virgaureae;0.999506208964591;
BDE448-19;Lepidoptera;Melanargia galathea;Melanargia galathea;NA;Melanargia galathea;0.97229026078718;
BDE449-19;Lepidoptera;Argynnis aglaja;NA;0.002;Speyeria aglaja;0.87239079736947;
BDE450-19;Lepidoptera;Pararge aegeria;Pararge aegeria;1;Pararge aegeria;0.999999999963165;
BDE452-19;Lepidoptera;Lycaena phlaeas;Lycaena phlaeas;1;Lycaena phlaeas;0.999999996363186;
BDE453-19;Lepidoptera;Hipparchia fidia;Hipparchia fidia;1;Hipparchia fidia;1;
BDE455-19;Lepidoptera;Celastrina argiolus;Celastrina argiolus;1;Celastrina argiolus;0.999527385150531;
BDE457-19;Lepidoptera;Glaucopsyche melanops;Glaucopsyche melanops;1;Glaucopsyche melanops;0.999952924254607;
BDE459-19;Lepidoptera;Issoria lathonia;Issoria lathonia;1;Issoria lathonia;0.999999999989257;
BDE463-19;Lepidoptera;Pyronia cecilia;Pyronia cecilia;1;Pyronia cecilia;1;
BDE464-19;Lepidoptera;Glaucopsyche melanops;Glaucopsyche melanops;1;Glaucopsyche melanops;0.999952924254607;
BDE467-19;Lepidoptera;Pyronia cecilia;NA;NA;Pyronia cecilia;0.999999999999943;
BDE480-19;Lepidoptera;Pyronia cecilia;Pyronia cecilia;1;Pyronia cecilia;1;
BDE482-19;Lepidoptera;Satyrium esculi;Satyrium esculi;1;Satyrium esculi;0.99999975269745;

BDE486-19;Lepidoptera;Coenonympha pamphilus;Coenonympha pamphilus;1;Coenonympha
pamphilus;0.999999999999744;
BDE489-19;Lepidoptera;Polyommatus celina;Polyommatus celina;0.009;Polyommatus
celina;0.999999999949694;
BDE491-19;Lepidoptera;Pyronia cecilia;Pyronia cecilia;1;Pyronia cecilia;0.999999999999943;
BDE495-19;Lepidoptera;Satyrus actaea;NA;0.001;Satyrus actaea;0.990911087649489;
BDE496-19;Lepidoptera;Satyrium spini;Satyrium spini;1;Satyrium spini;0.999997334596599;
BDE498-19;Lepidoptera;Pieris napi;NA;0.011;Pieris napi;0.801985394724814;
BDE499-19;Lepidoptera;Anthocharis cardamines;Anthocharis cardamines;0.002;Anthocharis
cardamines;0.999999951714672;
BDE500-19;Lepidoptera;Gonepteryx rhamni;NA;0.474;Gonepteryx rhamni;0.982076396127858;
BDE501-19;Lepidoptera;Cupido argiades;NA;0.499;NA;0.999999999984682;
BDE502-19;Lepidoptera;Lycaena phlaeas;Lycaena phlaeas;1;Lycaena phlaeas;0.999999996363186;
BDE506-19;Lepidoptera;Boloria dia;Boloria dia;1;Boloria dia;0.999999999980503;
BDE508-19;Lepidoptera;Araschnia levana;Araschnia levana;1;Araschnia levana;0.999999772187832;
BDE511-19;Lepidoptera;Papilio machaon;Papilio machaon;0.003;Papilio machaon;0.999482752749035;
BDE513-19;Lepidoptera;Araschnia levana;Araschnia levana;1;Araschnia levana;0.999999772187832;
BDE514-19;Lepidoptera;Pieris napi;NA;0.011;Pieris napi;0.801985394724814;
BDE515-19;Lepidoptera;Aglais urticae;Aglais urticae;0.001;Aglais urticae;0.990690600684087;
BDE516-19;Lepidoptera;Melitaea cinxia;Melitaea cinxia;1;Melitaea cinxia;0.999999999999943;
BDE520-19;Lepidoptera;Anthocharis euphenoides;NA;NA;Anthocharis euphenoides;0.960146267594346;
BDE521-19;Lepidoptera;Aricia agestis;Aricia cramera;1;Aricia cramera;0.999993731037033;
BDE522-19;Lepidoptera;Lycaena phlaeas;Lycaena phlaeas;1;Lycaena phlaeas;0.999999991873125;
BDE525-19;Lepidoptera;Callophrys rubi;NA;0.001;Callophrys rubi;0.99870518510598;
BDE526-19;Lepidoptera;Glaucopsyche melanops;Glaucopsyche melanops;1;Glaucopsyche
melanops;0.999957693018609;
BDE527-19;Lepidoptera;Lasiommata maera;Lasiommata maera;1;Lasiommata maera;0.99999857497297;
BDE533-19;Lepidoptera;Polyommatus icarus;NA;0.026;Polyommatus icarus;0.826249354925025;
BDE537-19;Lepidoptera;Gonepteryx rhamni;Gonepteryx rhamni;0.005;Gonepteryx
rhamni;0.9997748982174;
BDE538-19;Lepidoptera;Polyommatus celina;NA;0.025;Polyommatus celina;0.999999999946397;
BDE542-19;Lepidoptera;Euphydryas aurinia;Euphydryas aurinia;0.02;Euphydryas
aurinia;0.99668249939786;
BDE543-19;Lepidoptera;Lycaena phlaeas;Lycaena phlaeas;1;Lycaena phlaeas;0.999999996363186;
BDE544-19;Lepidoptera;Satyrium spini;Satyrium spini;1;Satyrium spini;0.99998759142769;
BDE546-19;Lepidoptera;Satyrium esculi;Satyrium esculi;1;Satyrium esculi;0.999999975795589;
BDE547-19;Lepidoptera;Satyrium spini;Satyrium spini;1;Satyrium spini;0.999990225283962;
BDE551-19;Lepidoptera;Satyrium esculi;Satyrium esculi;1;Satyrium esculi;0.999999836089486;
BDE555-19;Lepidoptera;Gonepteryx cleopatra;Gonepteryx cleopatra;0.998;Gonepteryx
cleopatra;0.999118636582266;
BDE556-19;Lepidoptera;Polyommatus celina;Polyommatus celina;0.009;Polyommatus
celina;0.999999999949694;
BDE563-19;Lepidoptera;Maniola jurtina;NA;0.001;Maniola jurtina;0.939220105860875;
BDE564-19;Lepidoptera;Aricia cramera;Aricia cramera;1;Aricia cramera;0.999993731037033;
BDE565-19;Lepidoptera;Colias crocea;NA;0.059;NA;0.999999999999738;
BDE571-19;Lepidoptera;Lasiommata megera;Lasiommata megera;1;Lasiommata megera;0.999928528239676;
BDE572-19;Lepidoptera;Lampides boeticus;Lampides boeticus;1;Lampides boeticus;0.999999999545906;
BDE573-19;Lepidoptera;Polyommatus celina;NA;0.022;Polyommatus celina;0.999999999893788;
BDE606-19;Lepidoptera;Euphydryas aurinia;Euphydryas aurinia;0.015;Euphydryas
aurinia;0.995469278399974;
BDE734-21;Lepidoptera;Aglais urticae;Aglais urticae;0.001;Aglais urticae;0.990690600684087;
BDE735-21;Lepidoptera;Aglais urticae;NA;0.004;Aglais urticae;0.980243655259103;
BDE736-21;Lepidoptera;Aglais urticae;NA;0.002;Aglais urticae;0.987497593219019;
BDE737-21;Lepidoptera;Aglais urticae;NA;0.004;Aglais urticae;0.976209345945922;
BDE738-21;Lepidoptera;Aglais urticae;NA;0.002;Aglais urticae;0.987497593219019;
BDE739-21;Lepidoptera;Aglais urticae;NA;0.002;Aglais urticae;0.987497593219019;
BDE740-21;Lepidoptera;Aglais urticae;Aglais urticae;0.001;Aglais urticae;0.994396335177485;
BDE741-21;Lepidoptera;Aglais urticae;NA;0.002;Aglais urticae;0.987497593219019;
BDE742-21;Lepidoptera;Aglais urticae;NA;0.002;Aglais urticae;0.987497593219019;
BDE743-21;Lepidoptera;Aglais urticae;NA;0.117;Aglais urticae;0.986519693221239;
BDE744-21;Lepidoptera;Aglais urticae;Aglais urticae;0.001;Aglais urticae;0.994396335177485;
BDE745-21;Lepidoptera;Aglais urticae;Aglais urticae;0.967;Aglais urticae;0.992839340839702;
BDE746-21;Lepidoptera;Aglais urticae;Aglais urticae;0.001;Aglais urticae;0.989513941242214;
BDE747-21;Lepidoptera;Aglais urticae;Aglais urticae;0.001;Aglais urticae;0.994396335177485;
BDE748-21;Lepidoptera;Aglais urticae;Aglais urticae;0.001;Aglais urticae;0.994396335177485;
BDE749-21;Lepidoptera;Aglais urticae;Aglais urticae;1;Aglais urticae;0.996080669636376;
BDE750-21;Lepidoptera;Aglais urticae;Aglais urticae;0.004;Aglais urticae;0.995016750255472;
BDE751-21;Lepidoptera;Aglais urticae;Aglais urticae;0.001;Aglais urticae;0.994396335177485;
BDE752-21;Lepidoptera;Aglais urticae;Aglais urticae;0.001;Aglais urticae;0.990690600684087;
BDE753-21;Lepidoptera;Aglais urticae;Aglais urticae;0.001;Aglais urticae;0.990690600684087;
BDE754-21;Lepidoptera;Aglais urticae;Aglais urticae;0.001;Aglais urticae;0.994966452807995;
BDE755-21;Lepidoptera;Aglais urticae;NA;0.002;Aglais urticae;0.987497593219019;
BDE756-21;Lepidoptera;Aglais urticae;Aglais urticae;0.001;Aglais urticae;0.990690600684087;
BDE757-21;Lepidoptera;Aglais urticae;Aglais urticae;0.001;Aglais urticae;0.990690600684087;
BDE760-21;Lepidoptera;Aglais urticae;NA;0.001;Aglais urticae;0.983431756290712;
BDE761-21;Lepidoptera;Aglais urticae;NA;0.002;Aglais urticae;0.987497593219019;
BDE762-21;Lepidoptera;Aglais urticae;NA;0.002;Aglais urticae;0.987497593219019;
BDE763-21;Lepidoptera;Aglais ichnusa;Aglais ichnusa;0.998;NA;0.999999996206876;

BDE764-21;Lepidoptera;Aglais urticae;Aglais urticae;0.001;Aglais urticae;0.994396335177485;
BDE765-21;Lepidoptera;Aglais ichnusa;Aglais ichnusa;0.998;NA;0.999999996206876;
BIBSA128-15;Lepidoptera;Pyronia tithonus;Pyronia tithonus;1;Pyronia tithonus;0.999999999999119;
BIBSA134-15;Lepidoptera;Hyponephele lycaon;Hyponephele lycaon;1;Hyponephele lycaon;0.999999996686455;
BIBSA1465-16;Lepidoptera;Erebia melampus;NA;0.384;Erebia melampus;0.806595181092398;
BIBSA1489-16;Lepidoptera;Lycaena dispar;Lycaena dispar;0.001;Lycaena dispar;0.999999996828279;
BIBSA1901-19;Lepidoptera;Polyommatus bellargus;NA;NA;Lysandra bellargus;0.995544920417663;
BIBSA1903-19;Lepidoptera;Plebejus argus;NA;0.11;Plebejus argus;0.967857858868143;
BIBSA1904-19;Lepidoptera;Maculinea arion;NA;0.026;Phengaris arion;0.932223897575527;
BIBSA1905-19;Lepidoptera;Maculinea arion;NA;0.054;Phengaris arion;0.938397095316026;
BIBSA1907-19;Lepidoptera;Cupido argiades;NA;0.485;NA;0.999999999982933;
BIBSA1908-19;Lepidoptera;Plebejus idas;NA;0.297;Plebejus argus;0.966815475875377;
BIBSA1910-19;Lepidoptera;Thymelicus lineola;Thymelicus lineola;1;Thymelicus lineola;0.999996457070319;
BIBSA1911-19;Lepidoptera;Thymelicus sylvestris;Thymelicus sylvestris;1;Thymelicus sylvestris;0.999999991232329;
BIBSA1912-19;Lepidoptera;Maniola jurtina;NA;0.038;NA;0.999999999999524;
BIBSA1913-19;Lepidoptera;Thymelicus acteon;Thymelicus acteon;1;Thymelicus acteon;0.999999993688419;
BIBSA1914-19;Lepidoptera;Lycaena phlaeas;Lycaena phlaeas;1;Lycaena phlaeas;0.999999976990381;
BIBSA1915-19;Lepidoptera;Satyrium ilicis;Satyrium ilicis;1;Satyrium ilicis;0.999993003728494;
BIBSA1916-19;Lepidoptera;Satyrium ilicis;Satyrium ilicis;1;Satyrium ilicis;0.999999984678283;
BIBSA1917-19;Lepidoptera;Colias crocea;NA;0.059;NA;0.999999999999738;
BIBSA1918-19;Lepidoptera;Lasiommata megera;Lasiommata megera;0.001;Lasiommata megera;0.999466241471132;
BIBSA1919-19;Lepidoptera;Pararge aegeria;Pararge aegeria;1;Pararge aegeria;0.999999999197257;
BIBSA1921-19;Lepidoptera;Lasiommata maera;Lasiommata maera;1;Lasiommata maera;0.999995042435739;
BIBSA1922-19;Lepidoptera;Lasiommata maera;Lasiommata maera;0.003;Lasiommata maera;0.999986871370403;
BIBSA1923-19;Lepidoptera;Coenonympha arcania;NA;0.105;NA;0.999999999999971;
BIBSA1924-19;Lepidoptera;Plebejus idas;Polyommatus escheri;1;Polyommatus escheri;0.999999967858628;
BIBSA1928-19;Lepidoptera;Polyommatus escheri;Polyommatus escheri;1;Polyommatus escheri;0.999999986318471;
BIBSA1929-19;Lepidoptera;Cupido minimus;NA;0.009;Cupido minimus;0.999687653269194;
BIBSA1930-19;Lepidoptera;Melitaea cinxia;Melitaea cinxia;1;Melitaea cinxia;0.999999999999062;
BIBSA1933-19;Lepidoptera;Pararge aegeria;Pararge aegeria;1;Pararge aegeria;0.999999999963165;
BIBSA1934-19;Lepidoptera;Pararge aegeria;Pararge aegeria;1;Pararge aegeria;0.99999999119342;
BIBSA1935-19;Lepidoptera;Maniola jurtina;NA;NA;Maniola jurtina;0.890930381730166;
BIBSA1936-19;Lepidoptera;Maniola jurtina;NA;0.011;Maniola jurtina;0.919381504201813;
BIBSA1937-19;Lepidoptera;Maniola jurtina;NA;0.001;Maniola jurtina;0.939220105860875;
BIBSA1938-19;Lepidoptera;Pyronia tithonus;Pyronia tithonus;1;Pyronia tithonus;1;
BIBSA1939-19;Lepidoptera;Pyronia tithonus;Pyronia tithonus;1;Pyronia tithonus;0.999999999999972;
BIBSA1940-19;Lepidoptera;Callophrys rubi;NA;0.009;Callophrys rubi;0.996682294679977;
BIBSA1942-19;Lepidoptera;Aricia agestis;NA;0.231;Aricia agestis;0.992802054683313;
BIBSA1943-19;Lepidoptera;Aricia agestis;NA;NA;Aricia agestis;0.999226097983055;
BIBSA1944-19;Lepidoptera;Aricia agestis;NA;0.081;Aricia agestis;0.999290860039936;
BIBSA1945-19;Lepidoptera;Cupido argiades;NA;0.499;NA;0.999999999984682;
BIBSA1946-19;Lepidoptera;Cupido argiades;NA;0.499;NA;0.999999999984682;
BIBSA1947-19;Lepidoptera;Thymelicus acteon;Thymelicus acteon;1;Thymelicus acteon;0.999999998066187;
BIBSA1948-19;Lepidoptera;Thymelicus acteon;Thymelicus acteon;1;Thymelicus acteon;0.99999999752248;
BIBSA1949-19;Lepidoptera;Thymelicus acteon;Thymelicus acteon;1;Thymelicus acteon;0.999999998099099;
BIBSA1950-19;Lepidoptera;Heteropterus morpheus;Heteropterus morpheus;1;Heteropterus morpheus;0.999999999999687;
BIBSA1951-19;Lepidoptera;Heteropterus morpheus;NA;NA;Heteropterus morpheus;0.999999999999943;
BIBSA1952-19;Lepidoptera;Coenonympha pamphilus;Coenonympha pamphilus;1;Coenonympha pamphilus;0.999999999999886;
BIBSA1953-19;Lepidoptera;Coenonympha pamphilus;Coenonympha pamphilus;1;Coenonympha pamphilus;0.999999999999886;
BIBSA1961-19;Lepidoptera;Polyommatus icarus;NA;0.002;Polyommatus icarus;0.998485237110805;
BIBSA1962-19;Lepidoptera;Lasiommata megera;Lasiommata megera;1;Lasiommata megera;0.999937484427266;
BIBSA1963-19;Lepidoptera;Ochlodes sylvanus;NA;0.003;Ochlodes sylvanus;0.995120982651897;
BIBSA1964-19;Lepidoptera;Vanessa atalanta;Vanessa atalanta;1;Vanessa atalanta;0.999999999757762;
BIBSA1965-19;Lepidoptera;Colias crocea;NA;NA;NA;0.999999999999584;
BIBSA1966-19;Lepidoptera;Pararge aegeria;Pararge aegeria;1;Pararge aegeria;0.999999999963165;
BIBSA1967-19;Lepidoptera;Maniola jurtina;NA;0.001;Maniola jurtina;0.901401754054888;
BIBSA1968-19;Lepidoptera;Maniola jurtina;NA;0.006;Maniola jurtina;0.941584460807617;
BIBSA1969-19;Lepidoptera;Polyommatus icarus;NA;0.002;Polyommatus icarus;0.998502314961965;
BIBSA1970-19;Lepidoptera;Polyommatus icarus;NA;0.005;Polyommatus icarus;0.971371333533657;
BIBSA1971-19;Lepidoptera;Lasiommata megera;Lasiommata megera;1;Lasiommata megera;0.999928528239676;
BIBSA1972-19;Lepidoptera;Lasiommata megera;Lasiommata megera;1;Lasiommata megera;0.999945664728349;

BIBSA1974-19;Lepidoptera;Pyronia tithonus;Pyronia tithonus;1;Pyronia tithonus;1;
BIBSA1975-19;Lepidoptera;Pararge aegeria;Pararge aegeria;1;Pararge aegeria;0.999999999963165;
BIBSA1976-19;Lepidoptera;Pararge aegeria;Pararge aegeria;1;Pararge aegeria;0.99999999953738;
BIBSA1977-19;Lepidoptera;Coenonympha pamphilus;Coenonympha pamphilus;1;Coenonympha pamphilus;0.999999999999886;
BIBSA1978-19;Lepidoptera;Coenonympha pamphilus;Coenonympha pamphilus;1;Coenonympha pamphilus;0.999999999999886;
BIBSA1981-19;Lepidoptera;Argynnis paphia;Argynnis paphia;1;Argynnis paphia;0.999999991389927;
BIBSA1983-19;Lepidoptera;Thymelicus acteon;Thymelicus acteon;1;Thymelicus acteon;0.99999999752248;
BIBSA1984-19;Lepidoptera;Thymelicus acteon;Thymelicus acteon;1;Thymelicus acteon;0.99999999752248;
BIBSA1985-19;Lepidoptera;Hipparchia semele;NA;0.002;Hipparchia semele;0.767063813608107;
BIBSA1986-19;Lepidoptera;Hipparchia semele;NA;0.004;Hipparchia semele;0.748325879155071;
BIBSA1987-19;Lepidoptera;Aricia agestis;NA;0.067;Aricia agestis;0.999509743227786;
BIBSA1988-19;Lepidoptera;Aricia agestis;NA;NA;Aricia agestis;0.998706664933933;
BIBSA1989-19;Lepidoptera;Polyommatus icarus;NA;0.001;Polyommatus icarus;0.981285528541973;
BIBSA1991-19;Lepidoptera;Thymelicus acteon;Thymelicus acteon;1;Thymelicus acteon;0.99999999752248;
BIBSA1992-19;Lepidoptera;Lycaena phlaeas;Lycaena phlaeas;1;Lycaena phlaeas;0.999999997146887;
BIBSA1993-19;Lepidoptera;Coenonympha pamphilus;Coenonympha pamphilus;1;Coenonympha pamphilus;0.999999999921442;
BIBSA1994-19;Lepidoptera;Maniola jurtina;NA;NA;NA;0.99999999985062;
BIBSA1995-19;Lepidoptera;Thymelicus lineola;Thymelicus lineola;1;Thymelicus lineola;0.999990451952522;
BIBSA2012-19;Lepidoptera;Erynnis tages;Erynnis tages;1;Erynnis tages;0.999997762421119;
BIBSA2013-19;Lepidoptera;Polygonia c-album;NA;0.215;NA;0.999999192918557;
BIBSA2014-19;Lepidoptera;Cupido alcetas;NA;0.068;Cupido alcetas;0.88988907876118;
BIBSA2015-19;Lepidoptera;Pieris napi;NA;0.008;Pieris napi;0.840314219469586;
BIBSA2016-19;Lepidoptera;Colias crocea;NA;0.059;NA;0.999999999999738;
BIBSA2019-19;Lepidoptera;Lampides boeticus;Lampides boeticus;1;Lampides boeticus;0.999999999545906;
BIBSA2021-19;Lepidoptera;Colias crocea;NA;0.059;NA;0.999999999999738;
BIBSA2025-19;Lepidoptera;Lampides boeticus;Lampides boeticus;1;Lampides boeticus;0.999999999545906;
BIBSA2026-19;Lepidoptera;Colias crocea;NA;0.059;NA;0.999999999999738;
BIBSA2027-19;Lepidoptera;Colias crocea;NA;0.059;NA;0.999999999999738;
BIBSA2028-19;Lepidoptera;Lampides boeticus;Lampides boeticus;1;Lampides boeticus;0.999999999545906;
BIBSA2030-19;Lepidoptera;Polyommatus thersites;Polyommatus icarus;0.001;Polyommatus icarus;0.999851522482238;
BIBSA2032-19;Lepidoptera;Polygonia egea;Polygonia egea;1;Polygonia egea;0.999999758078475;
BIBSA2033-19;Lepidoptera;Gonepteryx rhamni;NA;0.192;Gonepteryx rhamni;0.991002175992451;
BIBSA2034-19;Lepidoptera;Lasiommata maera;Lasiommata maera;1;Lasiommata maera;0.99999510792253;
BIBSA2035-19;Lepidoptera;Thymelicus acteon;Thymelicus acteon;1;Thymelicus acteon;0.99999999752248;
BIBSA2038-19;Lepidoptera;Pyronia cecilia;Pyronia cecilia;1;Pyronia cecilia;0.999999999999943;
BIBSA2039-19;Lepidoptera;Polyommatus amandus;Polyommatus amandus;0.002;Polyommatus amandus;0.99998879592523;
BIBSA2040-19;Lepidoptera;Polyommatus escheri;Polyommatus escheri;1;Polyommatus escheri;0.999999995379966;
BIBSA2041-19;Lepidoptera;Plebejus idas;NA;0.004;NA;0.999605698719885;
BIBSA2042-19;Lepidoptera;Cupido osiris;Cupido osiris;1;Cupido osiris;0.999999791352473;
BIBSA2044-19;Lepidoptera;Callophrys rubi;NA;0.019;Callophrys rubi;0.998201459637982;
BIBSA2045-19;Lepidoptera;Thymelicus lineola;Thymelicus lineola;1;Thymelicus lineola;0.999988258187826;
BIBSA2047-19;Lepidoptera;Lampides boeticus;Lampides boeticus;1;Lampides boeticus;0.999999999545906;
BIBSA2048-19;Lepidoptera;Lycaena phlaeas;Lycaena phlaeas;1;Lycaena phlaeas;0.999999996363186;
BIBSA2049-19;Lepidoptera;Colias alfacariensis;Colias alfacariensis;0.001;Colias alfacariensis;0.999926026317526;
BIBSA2050-19;Lepidoptera;Aricia agestis;NA;NA;Aricia agestis;0.996930365249266;
BIBSA2052-19;Lepidoptera;Heteropterus morpheus;NA;NA;Heteropterus morpheus;0.999999999999943;
BIBSA2053-19;Lepidoptera;Cupido minimus;NA;0.001;Cupido minimus;0.999803920701362;
BIBSA2054-19;Lepidoptera;Argynnis adippe;NA;NA;Fabriciana adippe;0.946794248611187;
BIBSA2055-19;Lepidoptera;Satyrium acaciae;Satyrium acaciae;1;Satyrium acaciae;0.999999754684667;
BIBSA2056-19;Lepidoptera;Brenthis daphne;Brenthis daphne;0.006;Brenthis daphne;0.984559944066479;
BIBSA2057-19;Lepidoptera;Aporia crataegi;NA;NA;Aporia crataegi;0.99999814099686;
BIBSA2058-19;Lepidoptera;Thymelicus sylvestris;Thymelicus sylvestris;1;Thymelicus sylvestris;0.999999960888005;
BIBSA2059-19;Lepidoptera;Melitaea nevadensis;NA;0.007;Melitaea celadussa;0.857896833450963;
BIBSA2061-19;Lepidoptera;Satyrium ilicis;Satyrium ilicis;1;Satyrium ilicis;0.999999987901305;
BIBSA2062-19;Lepidoptera;Lasiommata maera;Lasiommata maera;1;Lasiommata maera;0.99999510792253;
BIBSA2064-19;Lepidoptera;Melitaea cinxia;Melitaea cinxia;1;Melitaea cinxia;0.999999999999631;
BIBSA2065-19;Lepidoptera;Melitaea didyma;Melitaea didyma;1;Melitaea didyma;0.999931198646483;
BIBSA2066-19;Lepidoptera;Cyaniris semiargus;NA;NA;Cyaniris semiargus;0.999051841766953;
BIBSA2067-19;Lepidoptera;Plebejus argus;NA;NA;Plebejus argus;0.977793255361976;

BIBSA2069-19;Lepidoptera;Vanessa atalanta;Vanessa atalanta;1;Vanessa atalanta;0.999999999567706;
BIBSA2074-19;Lepidoptera;Boloria euphrosyne;Boloria euphrosyne;1;Boloria euphrosyne;0.99999665665472;
BIBSA2075-19;Lepidoptera;Pyrgus armoricanus;Pyrgus armoricanus;1;Pyrgus armoricanus;0.999997353848817;
BIBSA2076-19;Lepidoptera;Pyronia cecilia;Pyronia cecilia;1;Pyronia cecilia;1;
BIBSA2078-19;Lepidoptera;Maniola jurtina;NA;0.038;NA;0.999999999999524;
BIBSA2079-19;Lepidoptera;Coenonympha pamphilus;Coenonympha pamphilus;1;Coenonympha pamphilus;0.999999999998636;
BIBSA2080-19;Lepidoptera;Lasiommata megera;Lasiommata megera;1;Lasiommata megera;0.999379144055441;
BIBSA2081-19;Lepidoptera;Callophrys rubi;NA;0.019;Callophrys rubi;0.998201459637982;
BIBSA2083-19;Lepidoptera;Anthocharis cardamines;Anthocharis cardamines;1;Anthocharis cardamines;0.999999984875757;
BIBSA2086-19;Lepidoptera;Anthocharis cardamines;Anthocharis cardamines;1;Anthocharis cardamines;0.999999964875315;
BIBSA2092-20;Lepidoptera;Brintesia circe;Brintesia circe;1;Brintesia circe;0.999999696306634;
BIBSA2093-20;Lepidoptera;Issoria lathonia;Issoria lathonia;1;Issoria lathonia;0.999999999989257;
BIBSA2094-20;Lepidoptera;Carcharodus alceae;Carcharodus alceae;0.999;Carcharodus alceae;0.999999995579515;
BIBSA2095-20;Lepidoptera;Carcharodus alceae;Carcharodus alceae;0.999;Carcharodus alceae;0.999999996339511;
BIBSA2096-20;Lepidoptera;Colias crocea;NA;0.024;NA;0.999999999998621;
BIBSA2097-20;Lepidoptera;Callophrys rubi;NA;0.001;Callophrys rubi;0.998885705938309;
BIBSA2100-20;Lepidoptera;Brintesia circe;Brintesia circe;1;Brintesia circe;0.999998734635459;
BIBSA2102-20;Lepidoptera;Callophrys rubi;NA;0.001;Callophrys rubi;0.998885705938309;
BIBSA2103-20;Lepidoptera;Callophrys rubi;NA;0.001;Callophrys rubi;0.998885705938309;
BIBSA2104-20;Lepidoptera;Anthocharis cardamines;Anthocharis cardamines;1;Anthocharis cardamines;0.999999954741555;
BIBSA2105-20;Lepidoptera;Anthocharis cardamines;Anthocharis cardamines;1;Anthocharis cardamines;0.99999997953563;
BIBSA2106-20;Lepidoptera;Anthocharis cardamines;Anthocharis cardamines;1;Anthocharis cardamines;0.999999961354121;
BIBSA2107-20;Lepidoptera;Charaxes jasius;Charaxes jasius;NA;Charaxes jasius;0.990397821479112;
BIBSA2109-20;Lepidoptera;Vanessa atalanta;Vanessa atalanta;1;Vanessa atalanta;0.999999999757762;
BIBSA2110-20;Lepidoptera;Satyrium ilicis;Satyrium ilicis;1;Satyrium ilicis;0.999999987901305;
BIBSA2111-20;Lepidoptera;Satyrium ilicis;Satyrium ilicis;1;Satyrium ilicis;0.999999987901305;
BIBSA2116-20;Lepidoptera;Maniola jurtina;NA;0.001;Maniola jurtina;0.939220105860875;
BIBSA2117-20;Lepidoptera;Lycaena virgaureae;NA;0.186;Lycaena virgaureae;0.999491227342155;
BIBSA2118-20;Lepidoptera;Melitaea diamina;NA;0.149;Melitaea diamina;0.999999996592464;
BIBSA2119-20;Lepidoptera;Issoria lathonia;Issoria lathonia;1;Issoria lathonia;0.999999999989257;
BIBSA2120-20;Lepidoptera;Lycaena hippothoe;NA;NA;Lycaena hippothoe;0.901524795943211;
BIBSA2121-20;Lepidoptera;Ochlodes sylvanus;NA;0.316;Ochlodes sylvanus;0.996318754838625;
BIBSA2122-20;Lepidoptera;Aglais urticae;NA;0.002;Aglais urticae;0.987497593219019;
BIBSA2123-20;Lepidoptera;Aporia crataegi;NA;NA;Aporia crataegi;0.999995661287277;
BIBSA2124-20;Lepidoptera;Boloria titania;Boloria titania;0.001;Boloria titania;0.99983358374111;
BIBSA2125-20;Lepidoptera;Pararge aegeria;Pararge aegeria;1;Pararge aegeria;0.999999999963165;
BIBSA2126-20;Lepidoptera;Aphantopus hyperantus;Aphantopus hyperantus;0.004;Aphantopus hyperantus;0.999598909345147;
BIBSA2127-20;Lepidoptera;Brenthis daphne;NA;0.102;Brenthis daphne;0.717134521611551;
BIBSA2128-20;Lepidoptera;Issoria lathonia;Issoria lathonia;1;Issoria lathonia;0.999999999989257;
BIBSA2132-20;Lepidoptera;Euphydryas aurinia;NA;0.31;Euphydryas aurinia;0.993387708761386;
BIBSA2134-20;Lepidoptera;Cupido argiades;NA;0.499;NA;0.999999999984682;
BIBSA2136-20;Lepidoptera;Brenthis ino;Brenthis ino;1;Brenthis ino;0.997823940101957;
BIBSA2137-20;Lepidoptera;Boloria selene;NA;0.275;Boloria selene;0.999999702697396;
BIBSA2140-20;Lepidoptera;Melitaea varia;Melitaea varia;0.999;Melitaea varia;0.99999725909709;
BIBSA2144-20;Lepidoptera;Pyrgus carthami;Pyrgus carthami;1;Pyrgus carthami;0.999998997209607;
BIBSA2146-20;Lepidoptera;Parnassius mnemosyne;Parnassius mnemosyne;1;Parnassius mnemosyne;0.999999378711061;
BIBSA2148-20;Lepidoptera;Coenonympha glycerion;NA;0.004;Coenonympha rhodopensis;0.923784712175037;
BIBSA2149-20;Lepidoptera;Erebia pandrose;NA;NA;Erebia pandrose;0.8186015174923;
BIBSA2150-20;Lepidoptera;Erebia pandrose;NA;NA;Erebia pandrose;0.8186015174923;
BIBSA2152-20;Lepidoptera;Aporia crataegi;NA;NA;Aporia crataegi;0.999997860566808;
BIBSA2154-20;Lepidoptera;Brintesia circe;Brintesia circe;1;Brintesia circe;0.99999969821626;
BIBSA2155-20;Lepidoptera;Pararge aegeria;Pararge aegeria;1;Pararge aegeria;0.999999999963165;
BIBSA2158-20;Lepidoptera;Ochlodes sylvanus;NA;0.316;Ochlodes sylvanus;0.996318754838625;
BIBSA2161-20;Lepidoptera;Issoria lathonia;Issoria lathonia;1;Issoria lathonia;0.999999999967599;
BIBSA2162-20;Lepidoptera;Melitaea didyma;Melitaea didyma;0.999;Melitaea didyma;0.999760747572586;
BIBSA2163-20;Lepidoptera;Cupido minimus;NA;0.006;Cupido minimus;0.999768371759258;
BIBSA2164-20;Lepidoptera;Polygonia c-album;NA;0.215;NA;0.999999192918557;
BIBSA2165-20;Lepidoptera;Gonepteryx cleopatra;Gonepteryx cleopatra;0.998;Gonepteryx cleopatra;0.999118636582266;
BIBSA2166-20;Lepidoptera;Maniola jurtina;NA;0.023;Maniola jurtina;0.732198397368727;
BIBSA2167-20;Lepidoptera;Coenonympha corinna;Coenonympha corinna;1;Coenonympha corinna;0.999999999995879;
BIBSA2168-20;Lepidoptera;Pyronia cecilia;Pyronia cecilia;1;Pyronia cecilia;0.999999999999972;

BIBSA2171-20;Lepidoptera;Polyommatus celina;NA;0.044;Polyommatus celina;0.999999994953214;
BIBSA2173-20;Lepidoptera;Coenonympha corinna;Coenonympha corinna;1;Coenonympha corinna;0.999999869776141;
BIBSA2175-20;Lepidoptera;Celastrina argiolus;Celastrina argiolus;1;Celastrina argiolus;0.999527385150531;
BIBSA2177-20;Lepidoptera;Carcharodus alceae;Carcharodus alceae;1;Carcharodus alceae;0.999999998465938;
BIBSA2178-20;Lepidoptera;Lampides boeticus;Lampides boeticus;1;Lampides boeticus;0.999999999545906;
BIBSA2179-20;Lepidoptera;Thymelicus acteon;Thymelicus acteon;1;Thymelicus acteon;0.99999999752248;
BIBSA2180-20;Lepidoptera;Pyronia cecilia;Pyronia cecilia;NA;Pyronia cecilia;1;
BIBSA2182-20;Lepidoptera;Glaucopsyche alexis;Glaucopsyche alexis;0.001;Glaucopsyche alexis;0.94042380088325;
BIBSA2183-20;Lepidoptera;Melanargia galathea;Melanargia galathea;NA;Melanargia galathea;0.959429932872284;
BIBSA2184-20;Lepidoptera;Melanargia galathea;Melanargia galathea;NA;Melanargia galathea;0.959429932872284;
BIBSA2190-20;Lepidoptera;Lasiommata megera;Lasiommata megera;1;Lasiommata megera;0.999379144055441;
BIBSA2191-20;Lepidoptera;Lasiommata megera;Lasiommata megera;1;Lasiommata megera;0.999379144055441;
BIBSA2192-20;Lepidoptera;Lasiommata megera;Lasiommata megera;1;Lasiommata megera;0.999379144055441;
BIBSA2193-20;Lepidoptera;Colias crocea;NA;0.059;NA;0.999999999999738;
BIBSA2195-20;Lepidoptera;Lasiommata megera;Lasiommata megera;1;Lasiommata megera;0.999928528239676;
BIBSA2196-20;Lepidoptera;Gonepteryx cleopatra;Gonepteryx cleopatra;0.998;Gonepteryx cleopatra;0.999118636582266;
BIBSA2197-20;Lepidoptera;Lasiommata paramegaera;Lasiommata paramegaera;0.999;Lasiommata paramegaera;0.995283446287531;
BIBSA2198-20;Lepidoptera;Celastrina argiolus;Celastrina argiolus;1;Celastrina argiolus;0.999037794018796;
BIBSA2199-20;Lepidoptera;Gonepteryx cleopatra;Gonepteryx cleopatra;0.998;Gonepteryx cleopatra;0.999118636582266;
BIBSA2200-20;Lepidoptera;Coenonympha pamphilus;Coenonympha pamphilus;1;Coenonympha pamphilus;0.999999999999744;
BIBSA2202-20;Lepidoptera;Colias crocea;NA;0.059;NA;0.999999999999738;
BIBSA2203-20;Lepidoptera;Charaxes jasius;Charaxes jasius;NA;Charaxes jasius;0.990397821479112;
BIBSA2205-20;Lepidoptera;Thymelicus sylvestris;NA;0.324;Thymelicus sylvestris;0.999940778442454;
BIBSA2207-20;Lepidoptera;Lycaena phlaeas;Lycaena phlaeas;1;Lycaena phlaeas;0.999999996363186;
BIBSA2209-20;Lepidoptera;Lampides boeticus;Lampides boeticus;1;Lampides boeticus;0.99999999876789;
BIBSA2210-20;Lepidoptera;Celastrina argiolus;Celastrina argiolus;1;Celastrina argiolus;0.999527385150531;
BIBSA2211-20;Lepidoptera;Lycaena phlaeas;Lycaena phlaeas;1;Lycaena phlaeas;0.999999996363186;
BIBSA2212-20;Lepidoptera;Celastrina argiolus;Celastrina argiolus;1;Celastrina argiolus;0.999527385150531;
BIBSA2215-20;Lepidoptera;Pyronia cecilia;Pyronia cecilia;NA;Pyronia cecilia;1;
BIBSA2216-20;Lepidoptera;Lasiommata paramegaera;Lasiommata paramegaera;0.999;Lasiommata paramegaera;0.995283446287531;
BIBSA2217-20;Lepidoptera;Lasiommata paramegaera;Lasiommata paramegaera;0.999;Lasiommata paramegaera;0.995283446287531;
BIBSA2218-20;Lepidoptera;Colias crocea;NA;0.059;NA;0.999999999999738;
BIBSA2219-20;Lepidoptera;Pyronia tithonus;Pyronia tithonus;1;Pyronia tithonus;1;
BIBSA2220-20;Lepidoptera;Coenonympha corinna;Coenonympha corinna;1;Coenonympha corinna;0.999999999995879;
BIBSA2221-20;Lepidoptera;Lampides boeticus;Lampides boeticus;1;Lampides boeticus;0.999999999545906;
BIBSA2222-20;Lepidoptera;Melitaea nevadensis;NA;0.003;Melitaea celadussa;0.889046332903503;
BIBSA2223-20;Lepidoptera;Melitaea nevadensis;NA;0.003;Melitaea celadussa;0.918635002680887;
BIBSA2224-20;Lepidoptera;Celastrina argiolus;Celastrina argiolus;1;Celastrina argiolus;0.999037794018796;
BIBSA2225-20;Lepidoptera;Colias crocea;NA;0.059;NA;0.999999999999738;
BIBSA2228-20;Lepidoptera;Colias crocea;NA;0.059;NA;0.999999999999738;
BIBSA2229-20;Lepidoptera;Celastrina argiolus;Celastrina argiolus;1;Celastrina argiolus;0.999527385150531;
BIBSA2234-20;Lepidoptera;Pyronia cecilia;Pyronia cecilia;NA;Pyronia cecilia;0.999999999999943;
BIBSA2245-20;Lepidoptera;Vanessa cardui;Vanessa cardui;1;Vanessa cardui;0.999999459983318;
BIBSA2246-20;Lepidoptera;Aricia agestis;NA;0.253;Aricia agestis;0.996350017387504;
BIBSA2248-20;Lepidoptera;Carcharodus alceae;Carcharodus alceae;1;Carcharodus alceae;0.999999998465938;
BIBSA2250-20;Lepidoptera;Colias crocea;NA;0.059;NA;0.999999999999738;
BIBSA2251-20;Lepidoptera;Pyronia cecilia;Pyronia cecilia;NA;Pyronia cecilia;0.999999999999972;
BIBSA2252-20;Lepidoptera;Favonius quercus;NA;NA;NA;0.999999999998742;
BIBSA2253-20;Lepidoptera;Pyronia tithonus;Pyronia tithonus;1;Pyronia tithonus;1;
BIBSA2254-20;Lepidoptera;Pyronia tithonus;Pyronia tithonus;1;Pyronia tithonus;1;

BIBSA2255-20;Lepidoptera;Carcharodus alceae;Carcharodus alceae;0.001;Carcharodus alceae;0.999999995618566;
BIBSA2256-20;Lepidoptera;Pyrgus armoricanus;Pyrgus armoricanus;1;Pyrgus armoricanus;0.999997353848817;
BIBSA2257-20;Lepidoptera;Lampides boeticus;Lampides boeticus;1;Lampides boeticus;0.99999999876789;
BIBSA2258-20;Lepidoptera;Iphiclides podalirius;NA;0.313;Iphiclides podalirius;0.889198863100098;
BIBSA2259-20;Lepidoptera;Polygonia c-album;NA;0.215;NA;0.999999192918557;
BIBSA2261-20;Lepidoptera;Hipparchia neomiris;Hipparchia neomiris;1;Hipparchia neomiris;0.999999999998977;
BIBSA2262-20;Lepidoptera;Hipparchia aristaeus;Hipparchia aristaeus;1;Hipparchia aristaeus;0.999999889765866;
BIBSA2266-20;Lepidoptera;Coenonympha corinna;Coenonympha corinna;1;Coenonympha corinna;0.999999999975955;
BIBSA2268-20;Lepidoptera;Anthocharis cardamines;Anthocharis cardamines;1;Anthocharis cardamines;0.999999961354121;
BIBSA2270-20;Lepidoptera;Pyrgus armoricanus;Pyrgus armoricanus;1;Pyrgus armoricanus;0.999997353848817;
BIBSA2271-20;Lepidoptera;Iphiclides podalirius;NA;0.302;Iphiclides podalirius;0.931082412606632;
BIBSA2272-20;Lepidoptera;Coenonympha pamphilus;Coenonympha pamphilus;1;Coenonympha pamphilus;0.999999999999744;
BIBSA2273-20;Lepidoptera;Aricia agestis;NA;0.253;Aricia agestis;0.996350017387504;
BIBSA2274-20;Lepidoptera;Pararge aegeria;Pararge aegeria;1;Pararge aegeria;0.999999999963165;
BIBSA2275-20;Lepidoptera;Celastrina argiolus;Celastrina argiolus;1;Celastrina argiolus;0.999527385150531;
BIBSA2282-20;Lepidoptera;Colias crocea;NA;0.059;NA;0.999999999999738;
BIBSA2284-20;Lepidoptera;Lasiommata megera;Lasiommata megera;1;Lasiommata megera;0.999379144055441;
BIBSA2285-20;Lepidoptera;Colias crocea;NA;0.059;NA;0.999999999999738;
BIBSA2286-20;Lepidoptera;Colias crocea;NA;0.059;NA;0.999999999999738;
BIBSA2287-20;Lepidoptera;Lampides boeticus;Lampides boeticus;1;Lampides boeticus;0.999999999690317;
BIBSA2288-20;Lepidoptera;Lampides boeticus;Lampides boeticus;1;Lampides boeticus;0.999999999690317;
BIBSA2289-20;Lepidoptera;Celastrina argiolus;Celastrina argiolus;1;Celastrina argiolus;0.999527385150531;
BIBSA2291-20;Lepidoptera;Lasiommata megera;Lasiommata megera;1;Lasiommata megera;0.999379144055441;
BIBSA2293-20;Lepidoptera;Anthocharis cardamines;Anthocharis cardamines;1;Anthocharis cardamines;0.999999984567239;
BIBSA2294-20;Lepidoptera;Aricia agestis;NA;0.253;Aricia agestis;0.996350017387504;
BIBSA2295-20;Lepidoptera;Thymelicus acteon;Thymelicus acteon;1;Thymelicus acteon;0.99999999752248;
BIBSA2296-20;Lepidoptera;Pararge aegeria;Pararge aegeria;1;Pararge aegeria;0.999999999963165;
BIBSA2298-20;Lepidoptera;Satyrium w-album;NA;0.1;Satyrium w-album;0.979163594724737;
BIBSA2299-20;Lepidoptera;Polygonia c-album;NA;0.215;NA;0.999999192918557;
BIBSA2301-20;Lepidoptera;Pararge aegeria;Pararge aegeria;1;Pararge aegeria;0.999999999850246;
BIBSA2302-20;Lepidoptera;Erebia mnestra;Erebia mnestra;0.002;Erebia mnestra;0.999916977821046;
BIBSA2306-20;Lepidoptera;Agriades glandon;NA;0.001;Agriades glandon;0.986645583198728;
BIBSA2307-20;Lepidoptera;Erebia epiphron;Erebia epiphron;NA;Erebia epiphron;0.998243429957028;
BIBSA2310-20;Lepidoptera;Melitaea nevadensis;Melitaea parthenoides;1;Melitaea parthenoides;0.999999993530452;
BIBSA2312-20;Lepidoptera;Colias crocea;NA;0.059;NA;0.999999999999738;
BIBSA2316-20;Lepidoptera;Celastrina argiolus;Celastrina argiolus;1;Celastrina argiolus;0.999527385150531;
BIBSA2317-20;Lepidoptera;Colias crocea;NA;0.059;NA;0.999999999999738;
BIBSA2318-20;Lepidoptera;Pyronia cecilia;Pyronia cecilia;1;Pyronia cecilia;1;
BIBSA2320-20;Lepidoptera;Chazara briseis;Chazara briseis;0.004;Chazara briseis;0.999942158527271;
BIBSA2322-20;Lepidoptera;Anthocharis cardamines;Anthocharis cardamines;1;Anthocharis cardamines;0.999999988534341;
BIBSA2323-20;Lepidoptera;Colias crocea;NA;0.021;NA;0.999999999999351;
BIBSA2327-20;Lepidoptera;Gonepteryx cleopatra;Gonepteryx cleopatra;0.998;Gonepteryx cleopatra;0.999118636582266;
BIBSA2328-20;Lepidoptera;Vanessa cardui;Vanessa cardui;1;Vanessa cardui;0.999999459983318;
BIBSA2329-20;Lepidoptera;Plebejus idas;NA;0.001;NA;0.994620093546684;
BIBSA2333-20;Lepidoptera;Lasiommata megera;Lasiommata megera;1;Lasiommata megera;0.997946594306567;
BIBSA2334-20;Lepidoptera;Issoria lathonia;Issoria lathonia;1;Issoria lathonia;0.999999999989257;
BIBSA2335-20;Lepidoptera;Satyrium ilicis;Satyrium ilicis;1;Satyrium ilicis;0.999999987901305;
BIBSA2336-20;Lepidoptera;Thymelicus sylvestris;NA;0.324;Thymelicus sylvestris;0.999940778442454;
BIBSA2338-20;Lepidoptera;Pararge aegeria;Pararge aegeria;1;Pararge aegeria;0.999999999963165;
BIBSA2342-20;Lepidoptera;Issoria lathonia;Issoria lathonia;1;Issoria lathonia;0.999999999989257;
BIBSA2344-20;Lepidoptera;Polyommatus celina;Polyommatus celina;0.009;Polyommatus celina;0.999999999739714;
BIBSA2345-20;Lepidoptera;Gonepteryx cleopatra;Gonepteryx cleopatra;0.998;Gonepteryx cleopatra;0.999118636582266;
BIBSA2346-20;Lepidoptera;Lycaena tityrus;Lycaena tityrus;1;Lycaena tityrus;0.999998928238034;

BIBSA2347-20;Lepidoptera;Callophrys rubi;NA;0.001;Callophrys rubi;0.99870518510598;
BIBSA2348-20;Lepidoptera;Pararge aegeria;Pararge aegeria;1;Pararge aegeria;0.999999999963165;
BIBSA2349-20;Lepidoptera;Gonepteryx cleopatra;Gonepteryx cleopatra;0.969;Gonepteryx cleopatra;0.998560379901668;
BIBSA2352-20;Lepidoptera;Polyommatus icarus;NA;0.001;Polyommatus icarus;0.981285528541973;
BIBSA2354-20;Lepidoptera;Lasiommata megera;Lasiommata megera;1;Lasiommata megera;0.999928528239676;
BIBSA2355-20;Lepidoptera;Anthocharis cardamines;Anthocharis cardamines;0.001;Anthocharis cardamines;0.999999939290634;
BIBSA2357-20;Lepidoptera;Satyrium ilicis;Satyrium ilicis;1;Satyrium ilicis;0.999999987901305;
BIBSA2358-20;Lepidoptera;Pyrgus malvoides;NA;0.176;Pyrgus malvoides;0.940490890379026;
BIBSA2359-20;Lepidoptera;Lampides boeticus;Lampides boeticus;1;Lampides boeticus;0.999999999545906;
BIBSA2360-20;Lepidoptera;Lasiommata megera;Lasiommata megera;1;Lasiommata megera;0.999379144055441;
BIBSA2361-20;Lepidoptera;Lasiommata megera;Lasiommata megera;1;Lasiommata megera;0.999379144055441;
BIBSA2362-20;Lepidoptera;Lasiommata megera;Lasiommata megera;1;Lasiommata megera;0.999379144055441;
BIBSA2365-20;Lepidoptera;Hipparchia fagi;NA;NA;Hipparchia fagi;0.984048543688953;
BIBSA2366-20;Lepidoptera;Cupido argiades;NA;0.499;NA;0.999999999984682;
BIBSA2368-20;Lepidoptera;Pyrgus armoricanus;Pyrgus armoricanus;1;Pyrgus armoricanus;0.999997353848817;
BIBSA2370-20;Lepidoptera;Erebia oeme;NA;0.002;Erebia medusa;0.991052367627986;
BIBSA2373-20;Lepidoptera;Lasiommata megera;Lasiommata megera;1;Lasiommata megera;0.999379144055441;
BIBSA2374-20;Lepidoptera;Pararge aegeria;Pararge aegeria;1;Pararge aegeria;0.999999999963165;
BIBSA2376-20;Lepidoptera;Polyommatus dolus;NA;0.001;Polyommatus dolus;0.945161985609982;
BIBSA2377-20;Lepidoptera;Pyrgus armoricanus;Pyrgus armoricanus;1;Pyrgus armoricanus;0.999997353848817;
BIBSA2378-20;Lepidoptera;Polyommatus thersites;Polyommatus thersites;0.001;Polyommatus thersites;0.999999883559219;
BIBSA2379-20;Lepidoptera;Polyommatus daphnis;Polyommatus daphnis;1;Polyommatus daphnis;0.999949599958755;
BIBSA2381-20;Lepidoptera;Lycaena virgaureae;NA;0.209;Lycaena virgaureae;0.999669745100572;
BIBSA2383-20;Lepidoptera;Gonepteryx cleopatra;Gonepteryx cleopatra;0.998;Gonepteryx cleopatra;0.999118636582266;
BIBSA2385-20;Lepidoptera;Colias crocea;NA;0.059;NA;0.999999999999738;
BIBSA2386-20;Lepidoptera;Colias crocea;NA;0.059;NA;0.999999999999738;
BIBSA2389-20;Lepidoptera;Vanessa cardui;Vanessa cardui;1;Vanessa cardui;0.999999459983318;
BIBSA2391-20;Lepidoptera;Charaxes jasius;Charaxes jasius;0.001;Charaxes jasius;0.988304809129503;
BIBSA2395-20;Lepidoptera;Hipparchia neapolitana;NA;0.004;Hipparchia semele;0.780671715710997;
BIBSA2396-20;Lepidoptera;Hipparchia neapolitana;NA;0.001;NA;0.999999999999843;
BIBSA2399-20;Lepidoptera;Colias crocea;NA;0.059;NA;0.999999999999738;
BIBSA2400-20;Lepidoptera;Colias crocea;NA;0.059;NA;0.999999999999738;
BIBSA2404-20;Lepidoptera;Lycaena phlaeas;Lycaena phlaeas;1;Lycaena phlaeas;0.999999996363186;
BIBSA2405-20;Lepidoptera;Lycaena phlaeas;Lycaena phlaeas;1;Lycaena phlaeas;0.999999996363186;
BIBSA2407-20;Lepidoptera;Hipparchia fagi;NA;NA;Hipparchia fagi;0.959251275816445;
BIBSA2408-20;Lepidoptera;Lycaena phlaeas;Lycaena phlaeas;1;Lycaena phlaeas;0.999999996363186;
BIBSA2412-20;Lepidoptera;Vanessa atalanta;Vanessa atalanta;1;Vanessa atalanta;0.999999999669399;
BIBSA2415-20;Lepidoptera;Lampides boeticus;Lampides boeticus;1;Lampides boeticus;0.999999999690317;
BIBSA2416-20;Lepidoptera;Lampides boeticus;Lampides boeticus;1;Lampides boeticus;0.999999999455639;
BIBSA2419-20;Lepidoptera;Polyommatus icarus;Polyommatus icarus;0.001;Polyommatus icarus;0.999851522482238;
BIBSA2420-20;Lepidoptera;Vanessa atalanta;Vanessa atalanta;1;Vanessa atalanta;0.999999999410704;
BIBSA2421-20;Lepidoptera;Vanessa cardui;Vanessa cardui;1;Vanessa cardui;0.999999850530696;
BIBSA2427-20;Lepidoptera;Colias crocea;NA;0.059;NA;0.999999999999738;
BIBSA2428-20;Lepidoptera;Colias crocea;NA;0.006;NA;0.999999999999492;
BIBSA2429-20;Lepidoptera;Gonepteryx cleopatra;Gonepteryx cleopatra;1;Gonepteryx cleopatra;0.996726219900688;
BIBSA2430-20;Lepidoptera;Lampides boeticus;Lampides boeticus;1;Lampides boeticus;0.999999999545906;
BIBSA2431-20;Lepidoptera;Lampides boeticus;Lampides boeticus;1;Lampides boeticus;0.999999999545906;
BIBSA2439-20;Lepidoptera;Thecla betulae;Thecla betulae;1;Thecla betulae;0.999999999972886;
BIBSA2440-20;Lepidoptera;Satyrium w-album;NA;0.056;Satyrium w-album;0.989378230996316;
BIBSA2441-20;Lepidoptera;Apatura ilia;NA;0.165;Apatura ilia;0.956796812031481;
BIBSA2442-20;Lepidoptera;Lycaena virgaureae;NA;0.209;Lycaena virgaureae;0.999669745100572;
BIBSA2443-20;Lepidoptera;Satyrium w-album;NA;0.183;Satyrium w-album;0.94958214537494;
BIBSA2447-20;Lepidoptera;Erebia pandrose;NA;NA;Erebia pandrose;0.8186015174923;
BIBSA2448-20;Lepidoptera;Erebia meolans;NA;NA;Erebia meolans;0.998020712377682;
BIBSA2449-20;Lepidoptera;Parnassius mnemosyne;Parnassius mnemosyne;1;Parnassius mnemosyne;0.999999378711061;
BIBSA2453-20;Lepidoptera;Polyommatus daphnis;Polyommatus daphnis;1;Polyommatus daphnis;0.999937221723078;

BIBSA2454-20;Lepidoptera;Erebia meolans;Erebia meolans;NA;Erebia meolans;0.997425474109132;
BIBSA2456-20;Lepidoptera;Issoria lathonia;Issoria lathonia;1;Issoria lathonia;0.999999999989257;
BIBSA2458-20;Lepidoptera;Carcharodus lavatherae;NA;0.333;Carcharodus lavatherae;0.99839243348919;
BIBSA2459-20;Lepidoptera;Polygonia egea;Polygonia egea;1;Polygonia egea;0.999999758078475;
BIBSA2460-20;Lepidoptera;Favonius quercus;NA;NA;NA;0.999999999998728;
BIBSA2461-20;Lepidoptera;Hipparchia fagi;NA;NA;Hipparchia fagi;0.952646862594835;
BIBSA2463-20;Lepidoptera;Parnassius mnemosyne;Parnassius mnemosyne;1;Parnassius mnemosyne;0.9999989662036;
BIBSA2465-20;Lepidoptera;Melitaea diamina;Melitaea diamina;0.001;Melitaea diamina;0.999999991551789;
BIBSA2466-20;Lepidoptera;Polyommatus thersites;NA;0.824;Polyommatus thersites;0.999999764108565;
BIBSA2469-20;Lepidoptera;Cupido minimus;NA;0.009;Cupido minimus;0.998365728131086;
BIBSA2470-20;Lepidoptera;Argynnis adippe;NA;NA;Fabriciana adippe;0.922280384106682;
BIBSA2472-20;Lepidoptera;Erebia meolans;Erebia meolans;NA;Erebia meolans;0.997425474109132;
BIBSA2474-20;Lepidoptera;Erebia neoridas;NA;NA;Erebia neoridas;0.987404576493131;
BIBSA2475-20;Lepidoptera;Erebia meolans;Erebia meolans;0.993;Erebia meolans;0.996967089964336;
BIBSA2480-20;Lepidoptera;Erebia pluto;Erebia gorge;1;Erebia gorge;0.999999998936204;
BIBSA2482-20;Lepidoptera;Erebia pluto;Erebia pluto;1;Erebia pluto;0.999999186851643;
BIBSA2483-20;Lepidoptera;Erebia pluto;Erebia pluto;1;Erebia pluto;0.9999995980348;
BIBSA2489-20;Lepidoptera;Erebia meolans;Erebia meolans;NA;Erebia meolans;0.999880195584624;
BIBSA2491-20;Lepidoptera;Erebia meolans;NA;0.489;Erebia meolans;0.972815650293517;
BIBSA2496-20;Lepidoptera;Erebia pluto;Erebia pluto;1;Erebia pluto;0.999999419144556;
BIBSA2497-20;Lepidoptera;Erebia pluto;Erebia pluto;1;Erebia pluto;0.999999419144556;
BIBSA2499-20;Lepidoptera;Erebia pluto;Erebia pluto;1;Erebia pluto;0.999999419144556;
BIBSA2509-20;Lepidoptera;Erebia meolans;Erebia meolans;1;Erebia meolans;0.999164444873577;
BIBSA2510-20;Lepidoptera;Erebia epiphron;NA;NA;Erebia epiphron;0.997605378796729;
BIBSA2514-20;Lepidoptera;Erebia meolans;Erebia meolans;1;Erebia meolans;0.999231432693854;
BIBSA2516-20;Lepidoptera;Erebia epiphron;NA;NA;Erebia epiphron;0.9975214976346;
BIBSA2517-20;Lepidoptera;Erebia epiphron;NA;NA;Erebia epiphron;0.997527149345302;
BIBSA2523-20;Lepidoptera;Erebia meolans;Erebia meolans;1;Erebia meolans;0.999231432693854;
BIBSA2524-20;Lepidoptera;Erebia meolans;NA;NA;Erebia neoridas;0.985497901839785;
BIBSA2525-20;Lepidoptera;Erebia neoridas;NA;NA;Erebia neoridas;0.994117947216123;
BIBSA2526-20;Lepidoptera;Erebia neoridas;NA;NA;Erebia neoridas;0.994117947216123;
BIBSA2527-20;Lepidoptera;Erebia neoridas;NA;NA;Erebia neoridas;0.987404576493131;
BIBSA2528-20;Lepidoptera;Erebia neoridas;NA;NA;Erebia neoridas;0.994117947216123;
BIBSA2529-20;Lepidoptera;Erebia neoridas;NA;NA;Erebia neoridas;0.994117947216123;
BIBSA2530-20;Lepidoptera;Erebia neoridas;NA;NA;Erebia neoridas;0.994117947216123;
BIBSA2532-20;Lepidoptera;Erebia neoridas;NA;NA;Erebia neoridas;0.99150073021036;
BIBSA2533-20;Lepidoptera;Erebia neoridas;NA;NA;Erebia neoridas;0.994117947216123;
BIBSA2536-20;Lepidoptera;Carcharodus alceae;Carcharodus alceae;0.999;Carcharodus alceae;0.999999996339511;
BIBSA2537-20;Lepidoptera;Colias crocea;NA;0.059;NA;0.999999999999738;
BIBSA2538-20;Lepidoptera;Pyronia tithonus;Pyronia tithonus;1;Pyronia tithonus;1;
BIBSA2539-20;Lepidoptera;Gonepteryx rhamni;NA;0.194;Gonepteryx rhamni;0.99583141915324;
BIBSA2540-20;Lepidoptera;Pararge aegeria;Pararge aegeria;1;Pararge aegeria;0.999999999958789;
BIBSA2542-20;Lepidoptera;Papilio machaon;Papilio machaon;0.003;Papilio machaon;0.999482752749035;
BIBSA2544-20;Lepidoptera;Vanessa cardui;Vanessa cardui;1;Vanessa cardui;0.999999459983318;
BIBSA2548-20;Lepidoptera;Gonepteryx cleopatra;Gonepteryx cleopatra;0.998;Gonepteryx cleopatra;0.999118636582266;
BIBSA2549-20;Lepidoptera;Gonepteryx cleopatra;Gonepteryx cleopatra;0.998;Gonepteryx cleopatra;0.999118636582266;
BIBSA2550-20;Lepidoptera;Gonepteryx cleopatra;Gonepteryx cleopatra;0.998;Gonepteryx cleopatra;0.999118636582266;
BIBSA2554-20;Lepidoptera;Thymelicus acteon;Thymelicus acteon;1;Thymelicus acteon;0.99999999752248;
BIBSA2556-20;Lepidoptera;Papilio machaon;Papilio machaon;0.003;Papilio machaon;0.999482752749035;
BIBSA2559-20;Lepidoptera;Celastrina argiolus;Celastrina argiolus;1;Celastrina argiolus;0.999037794018796;
BIBSA2560-20;Lepidoptera;Colias crocea;NA;0.059;NA;0.999999999999738;
BIBSA2562-20;Lepidoptera;Lampides boeticus;Lampides boeticus;1;Lampides boeticus;0.999999999545906;
BIBSA2563-20;Lepidoptera;Hipparchia neomiris;Hipparchia neomiris;1;Hipparchia neomiris;0.999999999998977;
BIBSA2565-20;Lepidoptera;Charaxes jasius;Charaxes jasius;NA;Charaxes jasius;0.990397821479112;
BIBSA2566-20;Lepidoptera;Hipparchia aristaeus;Hipparchia aristaeus;1;Hipparchia aristaeus;0.999999943814517;
BIBSA2568-20;Lepidoptera;Hipparchia aristaeus;Hipparchia aristaeus;1;Hipparchia aristaeus;0.999999993824275;
BIBSA2570-20;Lepidoptera;Lasiommata megera;Lasiommata megera;1;Lasiommata megera;0.999379144055441;
BIBSA2571-20;Lepidoptera;Argynnis paphia;Argynnis paphia;1;Argynnis paphia;0.999999992970402;
BIBSA2572-20;Lepidoptera;Melitaea didyma;Melitaea didyma;1;Melitaea didyma;0.999945816868155;
BIBSA2573-20;Lepidoptera;Gegenes nostrodamus;Gegenes nostrodamus;1;Gegenes nostrodamus;0.999999996883645;

BIBSA2576-20;Lepidoptera;Gonepteryx cleopatra;Gonepteryx cleopatra;0.998;Gonepteryx cleopatra;0.999118636582266;
BIBSA2577-20;Lepidoptera;Erebia neoridas;NA;NA;Erebia neoridas;0.997112686054603;
BIBSA2578-20;Lepidoptera;Erebia neoridas;NA;NA;Erebia neoridas;0.997112686054603;
BIBSA2579-20;Lepidoptera;Erebia montana;Erebia montana;1;Erebia montana;0.99978250115925;
BIBSA2580-20;Lepidoptera;Erebia neoridas;NA;NA;Erebia neoridas;0.990464121514464;
BIBSA2581-20;Lepidoptera;Erebia medusa;NA;0.009;Erebia medusa;0.986275612856827;
BIBSA2582-20;Lepidoptera;Erebia medusa;NA;0.009;Erebia medusa;0.986275612856827;
BIBSA2583-20;Lepidoptera;Erebia medusa;NA;0.009;Erebia medusa;0.986275612856827;
BIBSA2584-20;Lepidoptera;Erebia pandrose;Erebia pandrose;NA;Erebia pandrose;0.956509272964271;
BIBSA2585-20;Lepidoptera;Erebia meolans;Erebia meolans;1;Erebia meolans;0.999164444873577;
BIBSA2590-20;Lepidoptera;Erebia epiphron;Erebia epiphron;NA;Erebia epiphron;0.997087969435667;
BIBSA2592-20;Lepidoptera;Erebia neoridas;Erebia neoridas;NA;Erebia neoridas;0.998631936578793;
BIBSA2594-20;Lepidoptera;Erebia neoridas;NA;NA;Erebia neoridas;0.994368810769763;
BIBSA2595-20;Lepidoptera;Erebia medusa;NA;0.009;Erebia medusa;0.986275612856827;
BIBSA2598-20;Lepidoptera;Erebia pandrose;Erebia pandrose;0.013;Erebia pandrose;0.974388092470716;
BIBSA2599-20;Lepidoptera;Erebia neoridas;Erebia neoridas;NA;Erebia neoridas;0.998631936578793;
BIBSA2600-20;Lepidoptera;Erebia neoridas;Erebia neoridas;NA;Erebia neoridas;0.998631936578793;
BIBSA2601-20;Lepidoptera;Erebia epiphron;Erebia epiphron;NA;Erebia epiphron;0.997087969435667;
BIBSA2602-20;Lepidoptera;Erebia montana;Erebia montana;1;Erebia montana;0.999855571743067;
BIBSA2603-20;Lepidoptera;Erebia epiphron;Erebia epiphron;NA;Erebia epiphron;0.998243429957028;
BIBSA2604-20;Lepidoptera;Erebia pluto;Erebia pluto;1;Erebia pluto;0.999999696335028;
BIBSA2605-20;Lepidoptera;Erebia epiphron;NA;NA;Erebia epiphron;0.997605378796729;
BIBSA2608-20;Lepidoptera;Erebia montana;Erebia montana;1;Erebia montana;0.999047244700624;
BIBSA2609-20;Lepidoptera;Erebia montana;Erebia montana;1;Erebia montana;0.999047244700624;
BIBSA2614-20;Lepidoptera;Erebia montana;Erebia montana;1;Erebia montana;0.99905080170195;
BIBSA2616-20;Lepidoptera;Erebia pandrose;Erebia pandrose;0.003;Erebia pandrose;0.993608645577247;
BIBSA2617-20;Lepidoptera;Erebia oeme;NA;0.096;Erebia oeme;0.999999999806249;
BIBSA2619-20;Lepidoptera;Erebia epiphron;NA;NA;Erebia epiphron;0.98984833968802;
BIBSA2626-20;Lepidoptera;Erebia neoridas;NA;NA;Erebia neoridas;0.97412860899077;
BIBSA2627-20;Lepidoptera;Erebia meolans;Erebia meolans;NA;Erebia meolans;0.999835953426128;
BIBSA2628-20;Lepidoptera;Erebia montana;Erebia montana;1;Erebia montana;0.999855571743067;
BIBSA2696-21;Lepidoptera;Brenthis hecate;NA;0.072;Brenthis hecate;0.999803056289114;
BIBSA2709-21;Lepidoptera;Polygonia egea;Polygonia egea;1;Polygonia egea;0.999999903367585;
BIBSA437-15;Lepidoptera;Coenonympha arcania;NA;0.091;Coenonympha arcania;0.908376923930217;
CM002851.1;Lepidoptera;Melitaea cinxia;NA;0.324;Melitaea cinxia;0.999999999995623;
DMAZ107-09;Lepidoptera;Lasiommata megera;Lasiommata megera;1;Lasiommata megera;0.999928528239676;
DMAZ108-09;Lepidoptera;Brenthis daphne;Brenthis daphne;0.001;Brenthis daphne;0.997781825264304;
DMAZ109-09;Lepidoptera;Brenthis daphne;Brenthis daphne;0.001;Brenthis daphne;0.997781825264304;
DMAZ110-09;Lepidoptera;Lasiommata maera;Lasiommata maera;1;Lasiommata maera;0.999997945450226;
DMAZ111-09;Lepidoptera;Lasiommata maera;Lasiommata maera;1;Lasiommata maera;0.999997945450226;
DMAZ112-09;Lepidoptera;Brenthis ino;NA;0.061;Brenthis ino;0.791997215074462;
DMAZ114-09;Lepidoptera;Aphantopus hyperantus;Aphantopus hyperantus;0.003;Aphantopus hyperantus;0.999999455871523;
DMAZ119-09;Lepidoptera;Erebia meolans;Erebia meolans;NA;Erebia meolans;0.999835953426128;
DMAZ120-09;Lepidoptera;Erebia meolans;Erebia meolans;NA;Erebia meolans;0.999835953426128;
DMAZ122-09;Lepidoptera;Lasiommata megera;Lasiommata megera;1;Lasiommata megera;0.999928528239676;
DQ008096;Lepidoptera;Maniola nurag;Maniola nurag;0.991;Maniola nurag;0.983874803370865;
DQ008097;Lepidoptera;Maniola nurag;Maniola nurag;0.991;Maniola nurag;0.983874803370865;
DQ008098;Lepidoptera;Maniola nurag;NA;0.003;Maniola nurag;0.948160948668927;
DQ008099;Lepidoptera;Maniola nurag;NA;0.003;Maniola nurag;0.948160948668927;
DQ008100;Lepidoptera;Maniola nurag;NA;0.003;Maniola nurag;0.948160948668927;
DQ008103;Lepidoptera;Maniola cecilia;Pyronia cecilia;NA;Pyronia cecilia;0.999999999995794;
DQ205111;Lepidoptera;Limenitis camilla;Limenitis camilla;0.998;NA;0.999999999999951;
DQ407763;Lepidoptera;Parnassius apollo;NA;0.001;NA;0.999999945436759;
DQ407769;Lepidoptera;Parnassius mnemosyne;Parnassius mnemosyne;1;Parnassius mnemosyne;0.999992614958542;
DQ463376;Lepidoptera;Aporia crataegi;NA;0.005;Aporia crataegi;0.999927154833558;
EF514429;Lepidoptera;Parnassius apollo;Parnassius apollo;0.999;Parnassius apollo;0.986977990307296;
EF514457;Lepidoptera;Parnassius apollo;Parnassius apollo;1;Parnassius apollo;0.99930702366574;
EU597125;Lepidoptera;Polyommatus amandus;Polyommatus amandus;0.009;Polyommatus amandus;0.999899545629377;
EU597126;Lepidoptera;Polyommatus amandus;Polyommatus amandus;0.009;Polyommatus amandus;0.999899545629377;
EU597131;Lepidoptera;Polyommatus eros;NA;0.001;NA;0.999997124762555;
EU597132;Lepidoptera;Polyommatus eros;NA;0.001;NA;0.999997124762555;
EU597133;Lepidoptera;Polyommatus eros;NA;0.001;NA;0.999997548457523;
EU597136;Lepidoptera;Polyommatus eros;NA;0.001;NA;0.99999798124352;
EU597137;Lepidoptera;Polyommatus eros;NA;0.001;NA;0.99999798124352;
EU597138;Lepidoptera;Polyommatus eros;NA;NA;NA;0.999997071831858;
EU597139;Lepidoptera;Polyommatus icarus;NA;0.001;NA;0.999999868591263;
EU597140;Lepidoptera;Polyommatus icarus;NA;0.002;NA;0.999999916681562;
EU597141;Lepidoptera;Polyommatus icarus;NA;0.002;NA;0.999999916681562;

EU836659;Lepidoptera;Parnassius mnemosyne;Parnassius mnemosyne;NA;NA;0.993531071388157;
EU836660;Lepidoptera;Parnassius mnemosyne;Parnassius mnemosyne;1;NA;0.991699445878714;
EU836661;Lepidoptera;Parnassius mnemosyne;Parnassius mnemosyne;1;NA;0.990370981718783;
EU836662;Lepidoptera;Parnassius mnemosyne;Parnassius mnemosyne;NA;NA;0.993414156509127;
EU836663;Lepidoptera;Parnassius mnemosyne;Parnassius mnemosyne;NA;NA;0.994340756388564;
EU836664;Lepidoptera;Parnassius mnemosyne;Parnassius mnemosyne;1;NA;0.992199494304769;
EU836665;Lepidoptera;Parnassius mnemosyne;Parnassius mnemosyne;1;NA;0.987655159599278;
EU836666;Lepidoptera;Parnassius mnemosyne;Parnassius mnemosyne;1;NA;0.986942554821084;
EU836667;Lepidoptera;Parnassius mnemosyne;Parnassius mnemosyne;1;NA;0.990617919362508;
EU836668;Lepidoptera;Parnassius mnemosyne;Parnassius mnemosyne;1;NA;0.991111110687633;
EU836669;Lepidoptera;Parnassius mnemosyne;Parnassius mnemosyne;NA;NA;0.993240577424131;
EU836670;Lepidoptera;Parnassius mnemosyne;Parnassius mnemosyne;1;NA;0.982406714779643;
EU836671;Lepidoptera;Parnassius mnemosyne;Parnassius mnemosyne;1;NA;0.985834905775943;
EU836672;Lepidoptera;Parnassius mnemosyne;Parnassius mnemosyne;1;NA;0.98899361194782;
EU836673;Lepidoptera;Parnassius mnemosyne;Parnassius mnemosyne;0.999;NA;0.99332427093159;
EU836674;Lepidoptera;Parnassius mnemosyne;Parnassius mnemosyne;0.999;NA;0.992057296611131;
EU836675;Lepidoptera;Parnassius mnemosyne;Parnassius mnemosyne;1;NA;0.711473166529364;
EU836676;Lepidoptera;Parnassius mnemosyne;Parnassius mnemosyne;1;NA;0.988689274388334;
EU836677;Lepidoptera;Parnassius mnemosyne;Parnassius mnemosyne;1;NA;0.989758536204545;
EU836678;Lepidoptera;Parnassius mnemosyne;Parnassius mnemosyne;1;NA;0.988898603530705;
EU836679;Lepidoptera;Parnassius mnemosyne;Parnassius mnemosyne;1;NA;0.988920405611335;
EU836680;Lepidoptera;Parnassius mnemosyne;Parnassius mnemosyne;1;NA;0.988687360273623;
EU836681;Lepidoptera;Parnassius mnemosyne;Parnassius mnemosyne;1;NA;0.982182510600399;
EU836682;Lepidoptera;Parnassius mnemosyne;Parnassius mnemosyne;1;NA;0.987335499667498;
EU919281;Lepidoptera;Lampides boeticus;Lampides boeticus;1;NA;0.933856907629784;
EU919314;Lepidoptera;Lampides boeticus;Lampides boeticus;NA;NA;0.936571568153291;
EU919315;Lepidoptera;Lampides boeticus;Lampides boeticus;NA;NA;0.929041409027339;
EU919331;Lepidoptera;Lampides boeticus;Lampides boeticus;1;NA;0.92755033012778;
EU919338;Lepidoptera;Lampides boeticus;Lampides boeticus;1;NA;0.93145283590753;
EULEP1224-15;Lepidoptera;Lycaena thersamon;Thersamonia thersamon;NA;Thersamonia thersamon;0.996061635185203;
EULEP1408-15;Lepidoptera;Hipparchia fagi;NA;NA;Hipparchia fagi;0.942857264035062;
EULEP1610-15;Lepidoptera;Hipparchia fagi;NA;NA;Hipparchia fagi;0.922352347358008;
EULEP1640-15;Lepidoptera;Limenitis populi;Limenitis populi;1;Limenitis populi;0.999999999913513;
EULEP1725-15;Lepidoptera;Cyaniris semiargus;NA;NA;Cyaniris semiargus;0.998830591470926;
EULEP2356-15;Lepidoptera;Hamearis lucina;Hamearis lucina;1;Hamearis lucina;0.999999998041574;
EULEP2588-15;Lepidoptera;Aglais ichnusa;Aglais ichnusa;0.001;NA;0.999999997028814;
EULEP2681-15;Lepidoptera;Boloria titania;NA;0.075;Boloria chariclea;0.875902760689287;
EULEP2806-15;Lepidoptera;Erebia neoridas;NA;NA;Erebia neoridas;0.990014687080988;
EULEP2818-15;Lepidoptera;Erebia manto;Erebia manto;0.014;Erebia manto;0.999902936830928;
EULEP2819-15;Lepidoptera;Erebia manto;Erebia manto;0.014;Erebia manto;0.999902936830928;
EULEP2821-15;Lepidoptera;Erebia manto;NA;0.176;Erebia manto;0.990266020044635;
EULEP2826-15;Lepidoptera;Erebia meolans;NA;0.03;Erebia meolans;0.989471197046052;
EULEP2827-15;Lepidoptera;Erebia meolans;NA;0.079;Erebia meolans;0.962039703443954;
EULEP2833-15;Lepidoptera;Erebia ottomana;Erebia ottomana;1;Erebia ottomana;0.999999997177142;
EULEP2858-15;Lepidoptera;Euphydryas intermedia;NA;0.001;Euphydryas intermedia;0.746327555155385;
EULEP3518-16;Lepidoptera;Boloria euphrosyne;Boloria euphrosyne;0.003;Boloria euphrosyne;0.999972659970609;
EULEP3729-16;Lepidoptera;Cupido alcetas;NA;0.001;Cupido alcetas;0.966087411023027;
EULEP4127-16;Lepidoptera;Lasiommata maera;Lasiommata maera;1;Lasiommata maera;0.999769586492687;
EULEP4133-16;Lepidoptera;Limenitis camilla;Limenitis camilla;0.001;Limenitis camilla;0.999843171870627;
EULEP4451-16;Lepidoptera;Pieris napi;NA;NA;Pieris napi;0.823232114003236;
EULEP4477-16;Lepidoptera;Plebejus idas;NA;0.007;NA;0.999546324331549;
EULEP4492-16;Lepidoptera;Plebejus idas;NA;0.001;NA;0.999033031509377;
EULEP4493-16;Lepidoptera;Plebejus idas;NA;0.001;NA;0.999430735322623;
EULEP4913-16;Lepidoptera;Brenthis ino;Brenthis ino;0.013;Brenthis ino;0.973702144793219;
EULEP5151-17;Lepidoptera;Parnassius apollo;Parnassius apollo;NA;Parnassius apollo;0.986316317299342;
EULEP5158-17;Lepidoptera;Limenitis camilla;Limenitis camilla;0.001;Limenitis camilla;0.999780663060643;
EULEP5159-17;Lepidoptera;Limenitis camilla;NA;0.001;Limenitis camilla;0.998716281294493;
EULEP576-15;Lepidoptera;Pyrgus carlinae;NA;0.001;NA;0.99999999640157;
EULEP5940-18;Lepidoptera;Erebia medusa;NA;0.04;Erebia medusa;0.984042649596477;
EULEP5941-18;Lepidoptera;Erebia medusa;NA;0.04;Erebia medusa;0.984042649596477;
EULEP5942-18;Lepidoptera;Erebia medusa;NA;0.04;Erebia medusa;0.984042649596477;
EULEP5943-18;Lepidoptera;Erebia medusa;NA;0.04;Erebia medusa;0.984042649596477;
EULEP5944-18;Lepidoptera;Erebia medusa;NA;0.04;Erebia medusa;0.984042649596477;
EULEP6004-20;Lepidoptera;Plebejus argus;NA;0.328;Plebejus argus;0.969789734199889;
EULEP6005-20;Lepidoptera;Hipparchia semele;NA;0.004;NA;0.999999999999872;
EULEP6006-20;Lepidoptera;Pyrgus malvae;Pyrgus malvae;NA;Pyrgus malvae;0.980121140374433;
EULEP6008-20;Lepidoptera;Favonius quercus;NA;NA;Favonius quercus;0.745528601618086;
EULEP6009-20;Lepidoptera;Aricia agestis;NA;0.073;Aricia agestis;0.999293749830595;
EULEP6010-20;Lepidoptera;Maniola jurtina;NA;0.001;Maniola jurtina;0.762537481133735;
EULEP6013-20;Lepidoptera;Lycaena phlaeas;Lycaena phlaeas;1;Lycaena phlaeas;0.999999996363186;
EULEP6014-20;Lepidoptera;Lycaena phlaeas;Lycaena phlaeas;1;Lycaena phlaeas;0.999999996363186;
EULEP6015-20;Lepidoptera;Araschnia levana;Araschnia levana;1;Araschnia levana;0.999999772187832;

EULEP6016-20;Lepidoptera;Polyommatus amandus;Polyommatus amandus;0.004;Polyommatus amandus;0.999986259154429;
EULEP6017-20;Lepidoptera;Coenonympha arcania;NA;0.057;Coenonympha arcania;0.983992716466119;
EULEP6019-20;Lepidoptera;Thymelicus lineola;Thymelicus lineola;1;Thymelicus lineola;0.999982844978955;
EULEP6020-20;Lepidoptera;Coenonympha arcania;NA;0.057;Coenonympha arcania;0.983992716466119;
EULEP6021-20;Lepidoptera;Argynnis aglaja;NA;NA;NA;0.999999999996955;
EULEP6022-20;Lepidoptera;Polyommatus thersites;NA;NA;Polyommatus thersites;0.999999041958266;
EULEP6024-20;Lepidoptera;Maniola jurtina;NA;NA;Maniola jurtina;0.746095236282775;
EULEP6025-20;Lepidoptera;Lycaena dispar;Lycaena dispar;1;Lycaena dispar;0.999999990743959;
EULEP6028-20;Lepidoptera;Polyommatus amandus;Polyommatus amandus;0.004;Polyommatus amandus;0.999986259154429;
EULEP6029-20;Lepidoptera;Lasiommata maera;Lasiommata maera;1;Lasiommata maera;0.99999942908402;
EULEP6030-20;Lepidoptera;Cyaniris semiargus;NA;NA;Cyaniris semiargus;0.998005169409319;
EULEP6031-20;Lepidoptera;Ochlodes sylvanus;NA;0.316;Ochlodes sylvanus;0.996318754838625;
EULEP6032-20;Lepidoptera;Cupido minimus;NA;0.009;Cupido minimus;0.999791453712643;
EULEP6033-20;Lepidoptera;Lycaena phlaeas;Lycaena phlaeas;1;Lycaena phlaeas;0.999999996363186;
EULEP6034-20;Lepidoptera;Coenonympha arcania;NA;0.057;Coenonympha arcania;0.983992716466119;
EULEP6036-20;Lepidoptera;Glaucopsyche alexis;NA;NA;Glaucopsyche alexis;0.979381169624069;
EULEP6037-20;Lepidoptera;Callophrys rubi;NA;0.01;Callophrys rubi;0.997394826446521;
EULEP6038-20;Lepidoptera;Glaucopsyche alexis;NA;NA;Glaucopsyche alexis;0.979381169624069;
EULEP6039-20;Lepidoptera;Aphantopus hyperantus;Aphantopus hyperantus;NA;Aphantopus hyperantus;0.997869678773523;
EULEP6040-20;Lepidoptera;Aglais urticae;Aglais urticae;0.001;Aglais urticae;0.994966452807995;
EULEP6041-20;Lepidoptera;Pieris napi;NA;0.008;Pieris napi;0.840314219469586;
EULEP6042-20;Lepidoptera;Melitaea diamina;NA;0.149;Melitaea diamina;0.999999996592464;
EULEP6043-20;Lepidoptera;Pararge aegeria;Pararge aegeria;1;Pararge aegeria;0.999999999963165;
EULEP6045-20;Lepidoptera;Gonepteryx rhamni;NA;0.474;Gonepteryx rhamni;0.982076396127858;
EULEP6046-20;Lepidoptera;Polygonia c-album;NA;0.215;NA;0.999999192918557;
EULEP6048-20;Lepidoptera;Erynnis tages;Erynnis tages;1;Erynnis tages;0.999999477791329;
EULEP6049-20;Lepidoptera;Lasiommata maera;Lasiommata maera;0.998;Lasiommata maera;0.999999191101026;
EULEP6050-20;Lepidoptera;Aricia artaxerxes;NA;0.383;Aricia artaxerxes;0.999814231048968;
EULEP6051-20;Lepidoptera;Celastrina argiolus;Celastrina argiolus;1;Celastrina argiolus;0.999708846309046;
EULEP6052-20;Lepidoptera;Boloria euphrosyne;Boloria euphrosyne;0.008;Boloria euphrosyne;0.999964281413411;
EULEP6054-20;Lepidoptera;Callophrys rubi;NA;0.003;Callophrys rubi;0.997328639918919;
EULEP6057-20;Lepidoptera;Erynnis tages;Erynnis tages;1;Erynnis tages;0.999995431835206;
EULEP6058-20;Lepidoptera;Anthocharis cardamines;Anthocharis cardamines;1;Anthocharis cardamines;0.999999940675226;
EULEP6059-20;Lepidoptera;Pieris napi;NA;0.066;Pieris napi;0.832895481558215;
EULEP6060-20;Lepidoptera;Boloria euphrosyne;Boloria euphrosyne;0.008;Boloria euphrosyne;0.999964281413411;
EULEP6062-20;Lepidoptera;Callophrys rubi;NA;0.003;Callophrys rubi;0.997328639918919;
EULEP6066-20;Lepidoptera;Lycaena phlaeas;Lycaena phlaeas;1;Lycaena phlaeas;0.999999998167567;
EULEP6067-20;Lepidoptera;Carterocephalus palaemon;Carterocephalus palaemon;0.016;Carterocephalus palaemon;0.930544560040029;
EULEP6068-20;Lepidoptera;Lycaena hippothoe;NA;NA;Lycaena hippothoe;0.928973453460131;
EULEP6070-20;Lepidoptera;Colias palaeno;NA;0.002;NA;0.999999999983681;
EULEP6071-20;Lepidoptera;Boloria euphrosyne;Boloria euphrosyne;0.036;Boloria euphrosyne;0.999968054449582;
EULEP6075-20;Lepidoptera;Colias palaeno;NA;0.002;NA;0.999999999983681;
EULEP6076-20;Lepidoptera;Cyaniris semiargus;NA;NA;Cyaniris semiargus;0.999299367533383;
EULEP6077-20;Lepidoptera;Aglais urticae;Aglais urticae;0.001;Aglais urticae;0.994966452807995;
EULEP6078-20;Lepidoptera;Ochlodes sylvanus;NA;0.315;Ochlodes sylvanus;0.99610625702004;
EULEP6079-20;Lepidoptera;Lycaena virgaureae;NA;0.186;Lycaena virgaureae;0.999491227342155;
EULEP6080-20;Lepidoptera;Coenonympha pamphilus;Coenonympha pamphilus;1;Coenonympha pamphilus;0.999999999999886;
EULEP6081-20;Lepidoptera;Brenthis ino;Brenthis ino;0.997;Brenthis ino;0.996195711592226;
EULEP6082-20;Lepidoptera;Argynnis aglaja;NA;0.005;NA;0.999999999994961;
EULEP6084-20;Lepidoptera;Plebejus idas;NA;0.001;NA;0.999237786552083;
EULEP6088-20;Lepidoptera;Aphantopus hyperantus;Aphantopus hyperantus;NA;Aphantopus hyperantus;0.998450183669756;
EULEP6090-20;Lepidoptera;Lycaena virgaureae;NA;0.186;Lycaena virgaureae;0.999491227342155;
EULEP6091-20;Lepidoptera;Apatura ilia;NA;0.165;Apatura ilia;0.956796812031481;
EULEP6092-20;Lepidoptera;Araschnia levana;Araschnia levana;1;Araschnia levana;0.999999772187832;
EULEP6094-20;Lepidoptera;Ochlodes sylvanus;NA;0.316;Ochlodes sylvanus;0.996318754838625;
EULEP6096-20;Lepidoptera;Coenonympha arcania;NA;0.067;Coenonympha arcania;0.978811371852141;
EULEP6097-20;Lepidoptera;Melanargia galathea;NA;NA;Melanargia galathea;0.943593560237409;
EULEP6098-20;Lepidoptera;Lycaena virgaureae;NA;0.076;Lycaena virgaureae;0.999570679451439;
EULEP6099-20;Lepidoptera;Issoria lathonia;Issoria lathonia;1;Issoria lathonia;0.999999999993747;
EULEP6100-20;Lepidoptera;Pieris napi;NA;0.011;Pieris napi;0.801985394724814;
EULEP6101-20;Lepidoptera;Maniola jurtina;NA;0.001;Maniola jurtina;0.762537481133735;
EULEP6104-20;Lepidoptera;Melanargia galathea;Melanargia galathea;NA;Melanargia galathea;0.970417110336134;
EULEP6105-20;Lepidoptera;Thymelicus lineola;Thymelicus lineola;1;Thymelicus lineola;0.999952080918693;

EULEP6106-20;Lepidoptera;Lycaena phlaeas;Lycaena phlaeas;1;Lycaena phlaeas;0.999999996363186;
EULEP6107-20;Lepidoptera;Celastrina argiolus;Celastrina argiolus;1;Celastrina argiolus;0.999527385150531;
EULEP6110-20;Lepidoptera;Pararge aegeria;Pararge aegeria;1;Pararge aegeria;0.999999999530445;
EULEP6111-20;Lepidoptera;Cupido argiades;NA;NA;NA;0.999999999899952;
EULEP6112-20;Lepidoptera;Lycaena tityrus;Lycaena tityrus;1;Lycaena tityrus;0.999998928238034;
EULEP6114-20;Lepidoptera;Aricia agestis;NA;0.253;Aricia agestis;0.996350017387504;
EULEP6115-20;Lepidoptera;Melanargia galathea;NA;NA;Melanargia galathea;0.943593560237409;
EULEP6116-20;Lepidoptera;Thymelicus lineola;Thymelicus lineola;1;Thymelicus lineola;0.999887623258534;
EULEP6118-20;Lepidoptera;Pararge aegeria;Pararge aegeria;1;Pararge aegeria;0.999999999963165;
EULEP6119-20;Lepidoptera;Lycaena tityrus;Lycaena tityrus;1;Lycaena tityrus;0.999998928238034;
EULEP6121-20;Lepidoptera;Thymelicus lineola;Thymelicus lineola;1;Thymelicus lineola;0.999951047783181;
EULEP6122-20;Lepidoptera;Boloria dia;Boloria dia;1;Boloria dia;0.999999999980503;
EULEP6123-20;Lepidoptera;Aglais urticae;NA;0.002;Aglais urticae;0.987497593219019;
EULEP6124-20;Lepidoptera;Boloria selene;NA;0.098;Boloria selene;0.999999661291981;
EULEP6128-20;Lepidoptera;Aphantopus hyperantus;Aphantopus hyperantus;0.996;Aphantopus hyperantus;0.999999185470491;
EULEP6129-20;Lepidoptera;Pieris rapae;NA;NA;Pieris rapae;0.999980812404001;
EULEP6130-20;Lepidoptera;Lasiommata megera;Lasiommata megera;1;Lasiommata megera;0.999928528239676;
EULEP6131-20;Lepidoptera;Coenonympha pamphilus;Coenonympha pamphilus;1;Coenonympha pamphilus;0.999999999999744;
EULEP6132-20;Lepidoptera;Gonepteryx rhamni;NA;0.474;Gonepteryx rhamni;0.982076396127858;
EULEP6133-20;Lepidoptera;Cyaniris semiargus;NA;NA;Cyaniris semiargus;0.999299367533383;
EULEP6134-20;Lepidoptera;Lycaena dispar;Lycaena dispar;1;Lycaena dispar;0.999999990743959;
EULEP6135-20;Lepidoptera;Maniola jurtina;NA;0.001;Maniola jurtina;0.762537481133735;
EULEP6136-20;Lepidoptera;Thymelicus lineola;Thymelicus lineola;1;Thymelicus lineola;0.999952080918693;
EULEP6137-20;Lepidoptera;Boloria selene;NA;0.001;Boloria selene;0.999995370701811;
EULEP6138-20;Lepidoptera;Lasiommata megera;Lasiommata megera;1;Lasiommata megera;0.999928528239676;
EULEP6139-20;Lepidoptera;Vanessa atalanta;Vanessa atalanta;1;Vanessa atalanta;0.999999999757762;
EULEP6140-20;Lepidoptera;Pararge aegeria;Pararge aegeria;1;Pararge aegeria;0.999999999963165;
EULEP6142-20;Lepidoptera;Colias hyale;Colias hyale;0.015;Colias hyale;0.999997884296128;
EULEP6143-20;Lepidoptera;Coenonympha pamphilus;Coenonympha pamphilus;1;Coenonympha pamphilus;0.999999999999744;
EULEP6144-20;Lepidoptera;Melanargia galathea;Melanargia galathea;NA;Melanargia galathea;0.981855578470499;
EULEP6146-20;Lepidoptera;Lycaena phlaeas;Lycaena phlaeas;1;Lycaena phlaeas;0.999999996363186;
EULEP6148-20;Lepidoptera;Brenthis ino;Brenthis ino;0.006;Brenthis ino;0.977279137111276;
EULEP6149-20;Lepidoptera;Pyronia tithonus;Pyronia tithonus;1;Pyronia tithonus;1;
EULEP6150-20;Lepidoptera;Lycaena phlaeas;Lycaena phlaeas;1;Lycaena phlaeas;0.999999996363186;
EULEP6155-20;Lepidoptera;Anthocharis cardamines;Anthocharis cardamines;1;Anthocharis cardamines;0.999999940675226;
EULEP6156-20;Lepidoptera;Cupido minimus;NA;0.001;Cupido minimus;0.999803920701362;
EULEP6157-20;Lepidoptera;Glaucopsyche alexis;NA;NA;Glaucopsyche alexis;0.976271367951777;
EULEP6158-20;Lepidoptera;Gonepteryx rhamni;NA;0.192;Gonepteryx rhamni;0.991002175992451;
EULEP6159-20;Lepidoptera;Melitaea cinxia;Melitaea cinxia;1;Melitaea cinxia;0.999999999999829;
EULEP6161-20;Lepidoptera;Euphydryas aurinia;NA;0.31;Euphydryas aurinia;0.993387708761386;
EULEP6162-20;Lepidoptera;Pyrgus armoricanus;Pyrgus armoricanus;1;Pyrgus armoricanus;0.997478680096412;
EULEP6163-20;Lepidoptera;Lycaena phlaeas;Lycaena phlaeas;1;Lycaena phlaeas;0.999999986385689;
EULEP6164-20;Lepidoptera;Pyrgus armoricanus;Pyrgus armoricanus;1;Pyrgus armoricanus;0.997478680096412;
EULEP6165-20;Lepidoptera;Pararge aegeria;Pararge aegeria;1;Pararge aegeria;0.999999999836319;
EULEP6166-20;Lepidoptera;Parnassius mnemosyne;Parnassius mnemosyne;1;Parnassius mnemosyne;0.99999987497234;
EULEP6167-20;Lepidoptera;Scolitantides orion;Scolitantides orion;1;Scolitantides orion;0.999999991449329;
EULEP6168-20;Lepidoptera;Callophrys rubi;NA;0.003;Callophrys rubi;0.997328639918919;
EULEP6169-20;Lepidoptera;Anthocharis cardamines;Anthocharis cardamines;1;Anthocharis cardamines;0.999999940675226;
EULEP6170-20;Lepidoptera;Carcharodus alceae;Carcharodus alceae;0.998;Carcharodus alceae;0.999999996978374;
EULEP6171-20;Lepidoptera;Gonepteryx rhamni;NA;0.474;Gonepteryx rhamni;0.982076396127858;
EULEP6172-20;Lepidoptera;Brenthis daphne;Brenthis daphne;0.001;Brenthis daphne;0.997781825264304;
EULEP6173-20;Lepidoptera;Coenonympha arcania;NA;0.001;Coenonympha arcania;0.956328802179621;
EULEP6174-20;Lepidoptera;Aporia crataegi;NA;NA;Aporia crataegi;0.999997351946131;
EULEP6175-20;Lepidoptera;Thymelicus sylvestris;Thymelicus sylvestris;1;Thymelicus sylvestris;0.999999985935205;
EULEP6177-20;Lepidoptera;Coenonympha glycerion;NA;0.389;Coenonympha glycerion;0.999998566525765;
EULEP6180-20;Lepidoptera;Coenonympha glycerion;NA;0.264;Coenonympha glycerion;0.999998370144304;
EULEP6181-20;Lepidoptera;Brenthis daphne;NA;0.102;Brenthis daphne;0.717134521611551;
EULEP6182-20;Lepidoptera;Satyrium ilicis;Satyrium ilicis;1;Satyrium ilicis;0.999999974300067;
EULEP6183-20;Lepidoptera;Satyrium acaciae;Satyrium acaciae;1;Satyrium acaciae;0.999999951077798;

EULEP6184-20;Lepidoptera;Melitaea diamina;NA;0.315;Melitaea diamina;0.999999923615721;
EULEP6185-20;Lepidoptera;Apatura ilia;NA;0.165;Apatura ilia;0.956796812031481;
EULEP6186-20;Lepidoptera;Melitaea cinxia;Melitaea cinxia;1;Melitaea cinxia;0.999999999999801;
EULEP6187-20;Lepidoptera;Melanargia galathea;Melanargia galathea;NA;Melanargia
galathea;0.981855578470499;
EULEP6188-20;Lepidoptera;Argynnis aglaja;NA;NA;Speyeria aglaja;0.872991147917853;
EULEP6189-20;Lepidoptera;Thymelicus lineola;Thymelicus lineola;1;Thymelicus
lineola;0.999993723282816;
EULEP6190-20;Lepidoptera;Coenonympha arcania;NA;0.096;Coenonympha arcania;0.962481905162994;
EULEP6191-20;Lepidoptera;Aphantopus hyperantus;Aphantopus hyperantus;0.003;Aphantopus
hyperantus;0.999999455871523;
EULEP6193-20;Lepidoptera;Brenthis daphne;Brenthis daphne;0.001;Brenthis
daphne;0.998611735797805;
EULEP6195-20;Lepidoptera;Apatura iris;Apatura iris;1;Apatura iris;0.999999999999972;
EULEP6196-20;Lepidoptera;Melitaea didyma;Melitaea didyma;NA;Melitaea didyma;0.987668646124956;
EULEP6198-20;Lepidoptera;Satyrium spini;Satyrium spini;1;Satyrium spini;0.999999835426407;
EULEP6200-20;Lepidoptera;Coenonympha glycerion;NA;0.264;Coenonympha glycerion;0.999998370144304;
EULEP6201-20;Lepidoptera;Thymelicus lineola;Thymelicus lineola;1;Thymelicus
lineola;0.999995927238524;
EULEP6202-20;Lepidoptera;Coenonympha arcania;NA;0.022;Coenonympha arcania;0.987735012529952;
EULEP6204-20;Lepidoptera;Lasiommata maera;Lasiommata maera;1;Lasiommata maera;0.999999140721485;
EULEP6206-20;Lepidoptera;Pararge aegeria;Pararge aegeria;1;Pararge aegeria;0.999999999836319;
EULEP6207-20;Lepidoptera;Boloria euphrosyne;Boloria euphrosyne;1;Boloria
euphrosyne;0.999998514406196;
EULEP6208-20;Lepidoptera;Lasiommata maera;Lasiommata maera;1;Lasiommata maera;0.999999181072746;
EULEP6209-20;Lepidoptera;Euphydryas aurinia;NA;0.31;Euphydryas aurinia;0.993387708761386;
EULEP6210-20;Lepidoptera;Cupido minimus;NA;0.002;Cupido minimus;0.999532194246603;
EULEP6211-20;Lepidoptera;Erebia epiphron;NA;NA;Erebia epiphron;0.994401488812176;
EULEP6214-20;Lepidoptera;Satyrus ferula;NA;0.001;Satyrus ferula;0.971317945932266;
EULEP6215-20;Lepidoptera;Boloria graeca;Boloria graeca;1;Boloria graeca;0.999999974197039;
EULEP6217-20;Lepidoptera;Brenthis ino;Brenthis ino;0.007;Brenthis ino;0.977157381961834;
EULEP6218-20;Lepidoptera;Lycaena tityrus;Lycaena tityrus;1;Lycaena tityrus;0.999998928238034;
EULEP6220-20;Lepidoptera;Polyommatus amandus;Polyommatus amandus;0.002;Polyommatus
amandus;0.999976189132416;
EULEP6221-20;Lepidoptera;Lasiommata megera;Lasiommata megera;0.006;Lasiommata
megera;0.992588117195958;
EULEP6222-20;Lepidoptera;Melanargia galathea;Melanargia galathea;NA;Melanargia
galathea;0.981855578470499;
EULEP6223-20;Lepidoptera;Gonepteryx rhamni;NA;0.39;Gonepteryx rhamni;0.997611156919627;
EULEP6224-20;Lepidoptera;Coenonympha arcania;NA;0.001;NA;0.999999999999975;
EULEP6225-20;Lepidoptera;Favonius quercus;NA;NA;NA;0.999999999999574;
EULEP6226-20;Lepidoptera;Thymelicus sylvestris;NA;0.131;Thymelicus sylvestris;0.999961574491035;
EULEP6227-20;Lepidoptera;Lycaena phlaeas;Lycaena phlaeas;1;Lycaena phlaeas;0.999999994740023;
EULEP6228-20;Lepidoptera;Carterocephalus palaemon;NA;0.052;Carterocephalus
palaemon;0.728445051795325;
EULEP6229-20;Lepidoptera;Cupido osiris;Cupido osiris;0.002;Cupido osiris;0.999919806355472;
EULEP6230-20;Lepidoptera;Hamearis lucina;NA;NA;Hamearis lucina;0.999999999972005;
EULEP6231-20;Lepidoptera;Melitaea diamina;NA;0.13;Melitaea diamina;0.999999982010166;
EULEP6232-20;Lepidoptera;Boloria titania;Boloria titania;0.97;Boloria titania;0.999611961598441;
EULEP6233-20;Lepidoptera;Lycaena thersamon;NA;NA;Lycaena thersamon;0.9170475904551;
EULEP6237-20;Lepidoptera;Vanessa cardui;Vanessa cardui;1;Vanessa cardui;0.999999803450484;
EULEP6239-20;Lepidoptera;Parnassius mnemosyne;Parnassius mnemosyne;1;Parnassius
mnemosyne;0.999999416965636;
EULEP6242-20;Lepidoptera;Callophrys rubi;NA;0.002;Callophrys rubi;0.995660099455287;
EULEP6243-20;Lepidoptera;Polyommatus amandus;Polyommatus amandus;0.004;Polyommatus
amandus;0.999986259154429;
EULEP6244-20;Lepidoptera;Aphantopus hyperantus;Aphantopus hyperantus;0.004;Aphantopus
hyperantus;0.999598909345147;
EULEP6245-20;Lepidoptera;Pyrgus malvae;Pyrgus malvae;NA;Pyrgus malvae;0.980121140374433;
EULEP6247-20;Lepidoptera;Carterocephalus palaemon;NA;0.052;Carterocephalus
palaemon;0.728445051795325;
EULEP6248-20;Lepidoptera;Lycaena phlaeas;Lycaena phlaeas;1;Lycaena phlaeas;0.999999996363186;
EULEP6249-20;Lepidoptera;Thecla betulae;Thecla betulae;1;Thecla betulae;0.999999999972886;
EULEP6250-20;Lepidoptera;Colias palaeno;NA;0.002;NA;0.999999999983681;
EULEP6253-20;Lepidoptera;Celastrina argiolus;Celastrina argiolus;1;Celastrina
argiolus;0.999708846309046;
EULEP6254-20;Lepidoptera;Callophrys rubi;NA;0.003;Callophrys rubi;0.997328639918919;
EULEP6256-20;Lepidoptera;Boloria euphrosyne;Boloria euphrosyne;0.991;Boloria
euphrosyne;0.999987993057807;
EULEP6259-20;Lepidoptera;Pararge aegeria;Pararge aegeria;1;Pararge aegeria;0.999999999846608;
EULEP6261-20;Lepidoptera;Polygonia c-album;NA;0.215;NA;0.999999192918557;
EULEP6262-20;Lepidoptera;Callophrys rubi;NA;0.003;Callophrys rubi;0.997328639918919;
EULEP6263-20;Lepidoptera;Polyommatus amandus;Polyommatus amandus;0.003;Polyommatus
amandus;0.999980071833264;
EULEP6264-20;Lepidoptera;Callophrys rubi;NA;0.003;Callophrys rubi;0.997328639918919;
EULEP6265-20;Lepidoptera;Lycaena tityrus;Lycaena tityrus;1;Lycaena tityrus;0.999999137688152;
EULEP6268-20;Lepidoptera;Pyrgus armoricanus;Pyrgus armoricanus;1;Pyrgus
armoricanus;0.997478680096412;

EULEP6270-20;Lepidoptera;Glaucopsyche alexis;NA;NA;Glaucopsyche alexis;0.969998277832517;
EULEP6271-20;Lepidoptera;Anthocharis cardamines;Anthocharis cardamines;0.001;Anthocharis cardamines;0.999999896812517;
EULEP6272-20;Lepidoptera;Glaucopsyche alexis;NA;NA;Glaucopsyche alexis;0.970914290380378;
EULEP6273-20;Lepidoptera;Polyommatus thersites;NA;NA;Polyommatus thersites;0.999999041958266;
EULEP6274-20;Lepidoptera;Cupido osiris;Cupido osiris;0.001;Cupido osiris;0.99999920729954;
EULEP6277-20;Lepidoptera;Coenonympha arcania;NA;0.001;NA;0.999999999999975;
EULEP6278-20;Lepidoptera;Glaucopsyche alexis;NA;NA;Glaucopsyche alexis;0.979381169624069;
EULEP6279-20;Lepidoptera;Polyommatus amandus;Polyommatus amandus;0.006;Polyommatus amandus;0.999984603640236;
EULEP6280-20;Lepidoptera;Euphydryas aurinia;NA;0.31;Euphydryas aurinia;0.993387708761386;
EULEP6281-20;Lepidoptera;Aporia crataegi;NA;NA;Aporia crataegi;0.999997266436607;
EULEP6283-20;Lepidoptera;Polyommatus thersites;NA;0.019;Polyommatus thersites;0.999997614558399;
EULEP6286-20;Lepidoptera;Hamearis lucina;NA;NA;Hamearis lucina;0.999999999984141;
EULEP6287-20;Lepidoptera;Gonepteryx rhamni;NA;0.474;Gonepteryx rhamni;0.982076396127858;
EULEP6288-20;Lepidoptera;Coenonympha pamphilus;Coenonympha pamphilus;1;Coenonympha pamphilus;0.999999999999744;
EULEP6289-20;Lepidoptera;Pyrgus armoricanus;Pyrgus armoricanus;1;Pyrgus armoricanus;0.99700549775422;
EULEP6290-20;Lepidoptera;Thymelicus lineola;Thymelicus lineola;1;Thymelicus lineola;0.999920011109672;
EULEP6291-20;Lepidoptera;Aricia artaxerxes;Plebejus argus;1;Aricia artaxerxes;0.999743187199922;
EULEP6292-20;Lepidoptera;Vanessa atalanta;Vanessa atalanta;1;Vanessa atalanta;0.999999998808789;
EULEP6293-20;Lepidoptera;Arethusana arethusa;NA;0.062;Arethusana arethusa;0.999962489703143;
EULEP6296-20;Lepidoptera;Satyrium ilicis;Satyrium ilicis;1;Satyrium ilicis;0.999999963901758;
EULEP6297-20;Lepidoptera;Cyaniris semiargus;NA;NA;Cyaniris semiargus;0.999051841766953;
EULEP6298-20;Lepidoptera;Cupido osiris;Cupido osiris;1;Cupido osiris;0.999998710927265;
EULEP6299-20;Lepidoptera;Polyommatus daphnis;NA;0.014;Polyommatus daphnis;0.986744184248688;
EULEP6300-20;Lepidoptera;Issoria lathonia;Issoria lathonia;1;Issoria lathonia;0.999999999989257;
EULEP6302-20;Lepidoptera;Colias hyale;NA;0.488;Colias hyale;0.999915488936852;
EULEP6303-20;Lepidoptera;Gonepteryx rhamni;NA;0.474;Gonepteryx rhamni;0.982076396127858;
EULEP6304-20;Lepidoptera;Aglais urticae;Aglais urticae;0.001;Aglais urticae;0.990690600684087;
EULEP6306-20;Lepidoptera;Anthocharis euphenoides;NA;0.007;Anthocharis euphenoides;0.97843137547761;
EULEP6309-20;Lepidoptera;Cyaniris semiargus;NA;NA;Cyaniris semiargus;0.998898909490417;
EULEP6311-20;Lepidoptera;Boloria euphrosyne;Boloria euphrosyne;0.018;Boloria euphrosyne;0.999995315694861;
EULEP6312-20;Lepidoptera;Argynnis aglaja;NA;NA;NA;0.999999999997818;
EULEP6313-20;Lepidoptera;Boloria titania;Boloria titania;0.001;Boloria titania;0.99983358374111;
EULEP6314-20;Lepidoptera;Coenonympha arcania;NA;0.076;Coenonympha arcania;0.970852850065578;
EULEP6315-20;Lepidoptera;Erebia meolans;NA;NA;Erebia meolans;0.999558897580846;
EULEP6316-20;Lepidoptera;Melanargia galathea;Melanargia galathea;NA;Melanargia galathea;0.981855578470499;
EULEP6317-20;Lepidoptera;Pyrgus serratulae;Pyrgus serratulae;1;Pyrgus serratulae;0.999999694697398;
EULEP6318-20;Lepidoptera;Carcharodus alceae;Carcharodus alceae;1;Carcharodus alceae;0.99999999711855;
EULEP6319-20;Lepidoptera;Erebia ottomana;Erebia ottomana;1;Erebia ottomana;0.999999994283371;
EULEP6320-20;Lepidoptera;Thecla betulae;Thecla betulae;1;Thecla betulae;0.999999999931561;
EULEP6322-20;Lepidoptera;Favonius quercus;NA;NA;Favonius quercus;0.73046069582187;
EULEP6324-20;Lepidoptera;Aphantopus hyperantus;Aphantopus hyperantus;0.007;Aphantopus hyperantus;0.999716519423631;
EULEP6326-20;Lepidoptera;Melitaea didyma;Melitaea didyma;1;Melitaea didyma;0.98897548959593;
EULEP6327-20;Lepidoptera;Thecla betulae;Thecla betulae;1;Thecla betulae;0.999999999965127;
EULEP6331-20;Lepidoptera;Lycaena virgaureae;NA;0.095;Lycaena virgaureae;0.99947625902545;
EULEP6332-20;Lepidoptera;Thymelicus lineola;Thymelicus lineola;1;Thymelicus lineola;0.999971535093703;
EULEP6333-20;Lepidoptera;Brenthis ino;Brenthis ino;0.001;Brenthis ino;0.994711912827522;
EULEP6334-20;Lepidoptera;Argynnis aglaja;NA;NA;NA;0.999999999998051;
EULEP6335-20;Lepidoptera;Erynnis tages;Erynnis tages;1;Erynnis tages;0.999999281987018;
EULEP6338-20;Lepidoptera;Lasiommata maera;Lasiommata maera;0.999;Lasiommata maera;0.999996386594878;
EULEP6339-20;Lepidoptera;Pieris rapae;NA;0.038;Pieris rapae;0.999969767806059;
EULEP6340-20;Lepidoptera;Erebia pandrose;Erebia pandrose;0.998;Erebia pandrose;0.997117103851311;
EULEP6341-20;Lepidoptera;Aglais urticae;Aglais urticae;0.003;Aglais urticae;0.991278660373755;
EULEP6342-20;Lepidoptera;Coenonympha pamphilus;Coenonympha pamphilus;1;Coenonympha pamphilus;0.999999999999886;
EULEP6344-20;Lepidoptera;Pieris napi;NA;0.013;Pieris napi;0.798118615202961;
EULEP6345-20;Lepidoptera;Boloria euphrosyne;Boloria euphrosyne;0.999;Boloria euphrosyne;0.999990576157994;
EULEP6346-20;Lepidoptera;Callophrys rubi;NA;0.003;Callophrys rubi;0.997328639918919;
EULEP6349-20;Lepidoptera;Colias palaeno;NA;0.002;NA;0.999999999983681;
EULEP6351-20;Lepidoptera;Boloria selene;NA;0.019;Boloria selene;0.999998063439647;
EULEP6352-20;Lepidoptera;Pieris napi;NA;0.011;Pieris napi;0.801985394724814;
EULEP6354-20;Lepidoptera;Ochlodes sylvanus;NA;0.316;Ochlodes sylvanus;0.996318754838625;
EULEP6355-20;Lepidoptera;Pararge aegeria;Pararge aegeria;1;Pararge aegeria;0.999999999963165;
EULEP6357-20;Lepidoptera;Aglais urticae;NA;0.002;Aglais urticae;0.987497593219019;

EULEP6361-20;Lepidoptera;Lycaena virgaureae;Lycaena virgaureae;0.027;Lycaena virgaureae;0.999582921361102;
EULEP6363-20;Lepidoptera;Thecla betulae;Thecla betulae;1;Thecla betulae;0.999999999955577;
EULEP6364-20;Lepidoptera;Pieris napi;NA;0.011;Pieris napi;0.801985394724814;
EULEP6365-20;Lepidoptera;Anthocharis cardamines;Anthocharis cardamines;1;Anthocharis cardamines;0.999999843941806;
EULEP6366-20;Lepidoptera;Boloria euphrosyne;Boloria euphrosyne;0.001;Boloria euphrosyne;0.999985640170951;
EULEP6367-20;Lepidoptera;Pieris napi;NA;0.028;Pieris napi;0.888205720664699;
EULEP6369-20;Lepidoptera;Boloria euphrosyne;Boloria euphrosyne;1;Boloria euphrosyne;0.999989902710144;
EULEP6370-20;Lepidoptera;Aporia crataegi;NA;NA;Aporia crataegi;0.999996781126733;
EULEP6372-20;Lepidoptera;Vanessa atalanta;Vanessa atalanta;1;Vanessa atalanta;0.999999999805453;
EULEP6375-20;Lepidoptera;Cyaniris semiargus;NA;NA;Cyaniris semiargus;0.999299367533383;
EULEP6378-20;Lepidoptera;Ochlodes sylvanus;NA;0.316;Ochlodes sylvanus;0.996318754838625;
EULEP6379-20;Lepidoptera;Aphantopus hyperantus;Aphantopus hyperantus;0.003;Aphantopus hyperantus;0.999999455871523;
EULEP6381-20;Lepidoptera;Boloria thore;Boloria thore;1;Boloria thore;0.999999996930853;
EULEP955-15;Lepidoptera;Limenitis camilla;Limenitis camilla;0.999;Limenitis camilla;0.999930476876987;
EZCHB293-09;Lepidoptera;Charaxes jasius;Charaxes jasius;NA;Charaxes jasius;0.949854280059292;
EZHBA001-07;Lepidoptera;Arethusana arethusa;Arethusana arethusa;0.025;Arethusana arethusa;0.999964979600124;
EZHBA068-07;Lepidoptera;Brenthis ino;Brenthis ino;0.005;Brenthis ino;0.960910255966063;
EZHBA070-07;Lepidoptera;Brenthis ino;Brenthis ino;0.003;Brenthis ino;0.99471031617257;
EZHBA079-07;Lepidoptera;Melitaea cinxia;Melitaea cinxia;0.044;Melitaea cinxia;0.999999999992383;
EZHBA080-07;Lepidoptera;Melitaea cinxia;NA;0.16;Melitaea cinxia;0.999999999999176;
EZHBA081-07;Lepidoptera;Melitaea cinxia;NA;0.057;Melitaea cinxia;0.999999999999545;
EZHBA206-07;Lepidoptera;Pieris napi;NA;0.011;Pieris napi;0.801985394724814;
EZHBA208-07;Lepidoptera;Pieris napi;NA;NA;NA;0.99999999999991;
EZHBA210-07;Lepidoptera;Pieris napi;NA;0.008;Pieris napi;0.840314219469586;
EZHBA211-07;Lepidoptera;Pieris napi;NA;0.084;Pieris napi;0.885343420349066;
EZHBA215-07;Lepidoptera;Pararge aegeria;Pararge aegeria;1;Pararge aegeria;0.999999999809319;
EZHBA216-07;Lepidoptera;Pieris napi;NA;0.011;Pieris napi;0.801985394724814;
EZHBA229-07;Lepidoptera;Erebia medusa;NA;0.009;Erebia medusa;0.986275612856827;
EZHBA230-07;Lepidoptera;Erebia medusa;NA;0.009;Erebia medusa;0.986275612856827;
EZHBA236-07;Lepidoptera;Aphantopus hyperantus;Aphantopus hyperantus;1;Aphantopus hyperantus;0.999999429853367;
EZHBA238-07;Lepidoptera;Boloria titania;Boloria titania;0.001;Boloria titania;0.99983358374111;
EZHBA240-07;Lepidoptera;Boloria titania;Boloria titania;0.001;Boloria titania;0.99983358374111;
EZHBA242-07;Lepidoptera;Erebia medusa;NA;0.092;Erebia oeme;0.999999999421647;
EZHBA243-07;Lepidoptera;Erebia medusa;NA;0.092;Erebia oeme;0.999999999421647;
EZHBA244-07;Lepidoptera;Erebia pandrose;Erebia pandrose;0.005;Erebia pandrose;0.994914940562747;
EZHBA245-07;Lepidoptera;Euphydryas aurinia;NA;0.31;Euphydryas aurinia;0.993387708761386;
EZHBA261-07;Lepidoptera;Thecla betulae;Thecla betulae;1;Thecla betulae;0.99999999996885;
EZHBA279-07;Lepidoptera;Erebia pandrose;Erebia pandrose;0.008;Erebia pandrose;0.992131426961598;
EZHBA291-07;Lepidoptera;Plebejus idas;NA;0.001;NA;0.998019917903297;
EZHBA292-07;Lepidoptera;Plebejus idas;NA;0.004;NA;0.999073942160048;
EZHBA308-07;Lepidoptera;Melitaea didyma;NA;0.165;Melitaea latonigena;0.997364814828811;
EZHBA329-07;Lepidoptera;Pieris napi;NA;0.001;Pieris virginiensis;0.797762237336339;
EZHBA331-07;Lepidoptera;Maculinea teleius;NA;0.498;Phengaris teleius;0.707377020793528;
EZHBA336-07;Lepidoptera;Coenonympha glycerion;Coenonympha hero;0.007;Coenonympha glycerion;0.952889470375269;
EZHBA337-07;Lepidoptera;Chazara briseis;Chazara briseis;0.001;Chazara briseis;0.999984130678865;
EZHBA338-07;Lepidoptera;Vanessa cardui;Vanessa cardui;1;Vanessa cardui;0.999999803450484;
EZHBA347-07;Lepidoptera;Plebejus argus;Plebejus argus;1;Aricia artaxerxes;0.999744916772824;
EZHBA352-07;Lepidoptera;Lampides boeticus;Lampides boeticus;1;Lampides boeticus;0.999999999304265;
EZHBA361-07;Lepidoptera;Callophrys rubi;NA;0.002;Callophrys rubi;0.996854375168945;
EZHBA362-07;Lepidoptera;Callophrys rubi;NA;0.002;Callophrys rubi;0.996854375168945;
EZHBA363-07;Lepidoptera;Araschnia levana;Araschnia levana;1;Araschnia levana;0.999999757979766;
EZHBA370-07;Lepidoptera;Aporia crataegi;NA;0.002;Aporia crataegi;0.999998160741186;
EZHBA371-07;Lepidoptera;Aporia crataegi;NA;NA;Aporia crataegi;0.999995760621692;
EZHBA372-07;Lepidoptera;Aporia crataegi;NA;NA;Aporia crataegi;0.999995760621692;
EZHBA375-07;Lepidoptera;Celastrina argiolus;Celastrina argiolus;1;Celastrina argiolus;0.999659368369645;
EZHBA376-07;Lepidoptera;Celastrina argiolus;Celastrina argiolus;1;Celastrina argiolus;0.999659368369645;
EZHBA385-07;Lepidoptera;Thymelicus lineola;Thymelicus lineola;1;Thymelicus lineola;0.999972112780985;
EZHBA387-07;Lepidoptera;Plebejus idas;NA;0.001;NA;0.9992305574893;
EZHBA393-07;Lepidoptera;Scolitantides orion;Scolitantides orion;NA;Scolitantides orion;0.999999987418505;
EZHBA394-07;Lepidoptera;Scolitantides orion;Scolitantides orion;1;Scolitantides orion;0.999999985205363;
EZHBA410-07;Lepidoptera;Boloria thore;Boloria thore;1;Boloria thore;0.999999683688479;
EZHBA416-07;Lepidoptera;Euphydryas intermedia;NA;0.001;Euphydryas aurinia;0.829740071784734;
EZHBA417-07;Lepidoptera;Euphydryas intermedia;NA;0.324;Euphydryas intermedia;0.954108568236655;

EZHBA419-07;Lepidoptera;Thecla betulae;Thecla betulae;1;Thecla betulae;0.99999999996885;
EZHBA462-07;Lepidoptera;Lycaena phlaeas;Lycaena phlaeas;0.012;NA;0.772489731589761;
EZHBA466-07;Lepidoptera;Carterocephalus palaemon;NA;0.036;Carterocephalus palaemon;0.875682325584506;
EZHBA467-07;Lepidoptera;Carterocephalus palaemon;NA;0.034;Carterocephalus palaemon;0.841107463891872;
EZHBA473-07;Lepidoptera;Erynnis tages;Erynnis tages;1;Erynnis tages;0.999998746428095;
EZHBA474-07;Lepidoptera;Erynnis tages;Erynnis tages;1;Erynnis tages;0.999997668555569;
EZHBA475-07;Lepidoptera;Araschnia levana;NA;0.186;Neptis sappho;0.999987268767013;
EZHBA479-07;Lepidoptera;Glaucopsyche alexis;Glaucopsyche alexis;0.001;Glaucopsyche alexis;0.996228899058554;
EZHBA480-07;Lepidoptera;Carterocephalus palaemon;Carterocephalus palaemon;0.011;Carterocephalus palaemon;0.924877521328362;
EZHBA484-07;Lepidoptera;Pyrgus malvae;Pyrgus malvae;NA;Pyrgus malvae;0.981848255193114;
EZHBA487-07;Lepidoptera;Lasiommata maera;Lasiommata maera;0.999;Lasiommata maera;0.999991974294436;
EZHBA517-07;Lepidoptera;Boloria napaea;NA;0.004;Boloria napaea;0.863893254514718;
EZHBA533-07;Lepidoptera;Argynnis paphia;Argynnis paphia;1;Argynnis paphia;0.999999990374505;
EZHBA534-07;Lepidoptera;Argynnis paphia;Argynnis paphia;1;Argynnis paphia;0.999999990374505;
EZHBA536-07;Lepidoptera;Aglais urticae;Aglais urticae;1;Aglais urticae;0.789513097787554;
EZHBA538-07;Lepidoptera;Issoria lathonia;Issoria lathonia;1;Issoria lathonia;0.999999999987949;
EZHBA539-07;Lepidoptera;Polyommatus icarus;NA;0.02;Polyommatus icarus;0.995583946382704;
EZHBA540-07;Lepidoptera;Polyommatus icarus;NA;0.02;Polyommatus icarus;0.995583946382704;
EZHBA541-07;Lepidoptera;Polyommatus icarus;NA;0.006;Polyommatus icarus;0.987670691800453;
EZHBA545-07;Lepidoptera;Satyrus ferula;Satyrus ferula;0.001;Satyrus ferula;0.969457022045489;
EZHBA546-07;Lepidoptera;Satyrus ferula;Satyrus ferula;0.006;Satyrus ferula;0.982914749148686;
EZHBA547-07;Lepidoptera;Satyrus ferula;NA;0.001;Satyrus ferula;0.96895623361784;
EZHBA550-07;Lepidoptera;Chazara briseis;Chazara briseis;0.001;Chazara briseis;0.999949744657138;
EZHBA551-07;Lepidoptera;Chazara briseis;Chazara briseis;0.993;Chazara briseis;0.999983113709829;
EZHBA555-07;Lepidoptera;Satyrus ferula;Satyrus ferula;0.998;Satyrus ferula;0.981324344453409;
EZHBA559-07;Lepidoptera;Polyommatus damon;NA;0.006;Polyommatus damone;0.987572346924968;
EZHBA560-07;Lepidoptera;Melitaea didyma;NA;NA;Melitaea latonigena;0.998520661242043;
EZHBA561-07;Lepidoptera;Melitaea didyma;NA;0.047;Melitaea latonigena;0.996493345168523;
EZHBA567-07;Lepidoptera;Euphydryas intermedia;NA;NA;Euphydryas intermedia;0.948800493926842;
EZHBA589-07;Lepidoptera;Erebia pandrose;Erebia pandrose;1;Erebia pandrose;0.991059440949696;
EZHBA602-07;Lepidoptera;Aglais urticae;Aglais urticae;0.001;Aglais urticae;0.990690600684087;
EZHBA614-07;Lepidoptera;Argynnis paphia;Argynnis paphia;1;Argynnis paphia;0.999999991389927;
EZHBA618-07;Lepidoptera;Celastrina argiolus;Celastrina argiolus;1;Celastrina argiolus;0.999527385150531;
EZHBA623-07;Lepidoptera;Argynnis paphia;Argynnis paphia;1;Argynnis paphia;0.999999991389927;
EZHBA624-07;Lepidoptera;Thymelicus lineola;Thymelicus lineola;0.997;Thymelicus lineola;0.999986912199643;
EZHBA625-07;Lepidoptera;Issoria lathonia;Issoria lathonia;1;Issoria lathonia;0.999999999989257;
EZHBA629-07;Lepidoptera;Gonepteryx rhamni;Gonepteryx rhamni;0.008;Gonepteryx rhamni;0.999527533936108;
EZHBA631-07;Lepidoptera;Hyponephele lycaon;Hyponephele lycaon;1;Hyponephele lycaon;0.999313352955397;
EZHBA636-07;Lepidoptera;Chazara briseis;Chazara briseis;1;Chazara briseis;0.962546535505063;
EZHBA637-07;Lepidoptera;Pieris napi;NA;NA;Pontia edusa;0.999987430880858;
EZHBA639-07;Lepidoptera;Plebejus argus;NA;NA;NA;0.958175512956666;
EZHBA641-07;Lepidoptera;Hyponephele lycaon;Hyponephele lycaon;1;Hyponephele lycaon;0.999999999877474;
EZHBA643-07;Lepidoptera;Arethusana arethusa;NA;0.055;Arethusana arethusa;0.999975657084744;
EZHBA651-07;Lepidoptera;Polyommatus damon;Polyommatus damon;1;Polyommatus damon;0.999999832375622;
EZHBA652-07;Lepidoptera;Boloria euphrosyne;Boloria euphrosyne;0.001;Boloria euphrosyne;0.999983620845843;
EZHBA656-07;Lepidoptera;Pieris napi;NA;0.487;Colias hyale;0.999917608048945;
EZHBA663-07;Lepidoptera;Brenthis daphne;Brenthis daphne;0.003;Brenthis daphne;0.981250390036139;
EZHBA665-07;Lepidoptera;Plebejus idas;NA;0.001;NA;0.999244503269316;
EZHBA666-07;Lepidoptera;Plebejus idas;NA;0.001;NA;0.998336170537544;
EZHBA667-07;Lepidoptera;Plebejus idas;NA;0.001;NA;0.999244503269316;
EZHBA668-07;Lepidoptera;Plebejus idas;NA;0.001;NA;0.999244503269316;
EZHBA669-07;Lepidoptera;Plebejus idas;NA;0.005;NA;0.996437398744577;
EZHBA671-07;Lepidoptera;Plebejus argus;NA;0.001;NA;0.999992802367651;
EZHBA678-07;Lepidoptera;Cupido minimus;NA;0.002;Cupido minimus;0.998300525877831;
EZHBA695-07;Lepidoptera;Euphydryas aurinia;NA;0.079;Euphydryas aurinia;0.898458207627416;
EZHBA709-07;Lepidoptera;Pieris napi;NA;NA;NA;100.000.000.000.001;
EZHBA711-07;Lepidoptera;Polyommatus icarus;NA;0.002;Polyommatus icarus;0.980427239137624;
EZHBA725-07;Lepidoptera;Callophrys rubi;NA;0.002;Callophrys rubi;0.996854375168945;
EZHBA726-07;Lepidoptera;Callophrys rubi;NA;0.002;Callophrys rubi;0.996854375168945;
EZHBA727-07;Lepidoptera;Callophrys rubi;NA;0.002;Callophrys rubi;0.997253508922544;
EZHBA728-07;Lepidoptera;Araschnia levana;Araschnia levana;1;Araschnia levana;0.999999696225348;
EZHBA729-07;Lepidoptera;Araschnia levana;Araschnia levana;1;Araschnia levana;0.999999757979766;
EZHBA730-07;Lepidoptera;Araschnia levana;Araschnia levana;1;Araschnia levana;0.999999757979766;
EZHBA732-07;Lepidoptera;Pieris rapae;Pieris rapae;NA;Pieris rapae;0.999994908102199;
EZHBA733-07;Lepidoptera;Pieris rapae;NA;NA;Pieris rapae;0.999953011121959;

EZHBA734-07;Lepidoptera;Coenonympha pamphilus;Coenonympha pamphilus;1;Coenonympha pamphilus;0.999999999999687;
EZHBA735-07;Lepidoptera;Coenonympha pamphilus;Coenonympha pamphilus;1;Coenonympha pamphilus;0.999999999999602;
EZHBA736-07;Lepidoptera;Pieris rapae;NA;0.022;Pontia edusa;0.99997255838741;
EZHBA744-07;Lepidoptera;Hyponephele lycaon;Hyponephele lycaon;1;Hyponephele lycaon;0.999999999131063;
EZHBA756-07;Lepidoptera;Aporia crataegi;NA;NA;Aporia crataegi;0.999995760621692;
EZHBA757-07;Lepidoptera;Aporia crataegi;NA;NA;Aporia crataegi;0.999995760621692;
EZHBA758-07;Lepidoptera;Limenitis populi;Limenitis populi;1;Limenitis populi;0.999999993386808;
EZHBA766-07;Lepidoptera;Brenthis ino;Brenthis ino;0.001;Brenthis ino;0.995262064014781;
EZHBA767-07;Lepidoptera;Brenthis ino;Brenthis ino;0.001;Brenthis ino;0.995262064014781;
EZHBA768-07;Lepidoptera;Issoria lathonia;Issoria lathonia;1;Issoria lathonia;0.999999999987949;
EZHBA769-07;Lepidoptera;Plebejus argus;NA;0.321;Plebejus argus;0.961323643580747;
EZHBA770-07;Lepidoptera;Plebejus argus;NA;0.421;Plebejus argus;0.940147304682971;
EZHBA776-07;Lepidoptera;Lasiommata maera;Lasiommata maera;1;Lasiommata maera;0.999940541293278;
EZHBA777-07;Lepidoptera;Lasiommata maera;Lasiommata maera;1;Lasiommata maera;0.999998059696345;
EZHBA778-07;Lepidoptera;Lasiommata maera;Lasiommata maera;1;Lasiommata maera;0.999998059696345;
EZHBA779-07;Lepidoptera;Lasiommata maera;Lasiommata maera;1;Lasiommata maera;0.999999556477077;
EZHBA780-07;Lepidoptera;Lasiommata maera;Lasiommata maera;1;Lasiommata maera;0.999999064930119;
EZHBA817-07;Lepidoptera;Pieris napi;NA;NA;Euchloe creusa;0.999995578632533;
EZHBA827-07;Lepidoptera;Erebia medusa;NA;0.009;Erebia medusa;0.986275612856827;
EZHBA828-07;Lepidoptera;Erebia medusa;NA;0.009;Erebia medusa;0.986275612856827;
EZHBA844-07;Lepidoptera;Boloria euphrosyne;Boloria euphrosyne;0.005;Boloria euphrosyne;0.999947951173974;
EZHBA845-07;Lepidoptera;Boloria euphrosyne;Boloria euphrosyne;0.001;Boloria euphrosyne;0.999985020608944;
EZHBA846-07;Lepidoptera;Boloria euphrosyne;Boloria euphrosyne;0.001;Boloria euphrosyne;0.999985020608944;
EZHBA883-07;Lepidoptera;Boloria thore;Boloria thore;1;Boloria thore;0.999877250611715;
EZHBA886-07;Lepidoptera;Pyrgus alveus;NA;NA;Pyrgus alveus;0.890529793922276;
EZHBA887-07;Lepidoptera;Pyrgus alveus;NA;NA;Pyrgus alveus;0.910320969135436;
EZHBA888-07;Lepidoptera;Pyrgus alveus;NA;NA;Pyrgus alveus;0.890529793922276;
EZHBA889-07;Lepidoptera;Pyrgus alveus;NA;NA;Pyrgus alveus;0.890529793922276;
EZHBA890-07;Lepidoptera;Pyrgus alveus;NA;NA;Pyrgus alveus;0.890529793922276;
EZHBA891-07;Lepidoptera;Pyrgus alveus;NA;NA;Pyrgus alveus;0.890529793922276;
EZSPC017-09;Lepidoptera;Pyrgus serratulae;Pyrgus serratulae;1;Pyrgus serratulae;0.99999989567508;
EZSPC099-09;Lepidoptera;Pyrgus armoricanus;Pyrgus armoricanus;1;Pyrgus armoricanus;0.998895076657973;
EZSPC120-09;Lepidoptera;Pyrgus armoricanus;Pyrgus armoricanus;1;Pyrgus armoricanus;0.998895076657973;
EZSPC121-09;Lepidoptera;Pyrgus armoricanus;Pyrgus armoricanus;1;Pyrgus armoricanus;0.998895076657973;
EZSPC122-09;Lepidoptera;Pyrgus armoricanus;Pyrgus armoricanus;1;Pyrgus armoricanus;0.998895076657973;
EZSPC123-09;Lepidoptera;Pyrgus armoricanus;Pyrgus armoricanus;1;Pyrgus armoricanus;0.998895076657973;
EZSPC144-09;Lepidoptera;Pyrgus armoricanus;Pyrgus armoricanus;1;Pyrgus armoricanus;0.998895076657973;
EZSPC145-09;Lepidoptera;Pyrgus armoricanus;Pyrgus armoricanus;1;Pyrgus armoricanus;0.998895076657973;
EZSPC146-09;Lepidoptera;Pyrgus armoricanus;Pyrgus armoricanus;1;Pyrgus armoricanus;0.998895076657973;
FJ428801;Lepidoptera;Polyommatus icarus;NA;NA;NA;0.999999509666107;
FJ428802;Lepidoptera;Polyommatus icarus;NA;NA;NA;0.999999509666107;
FJ428803;Lepidoptera;Polyommatus icarus;NA;NA;NA;0.999999509666107;
FJ428806;Lepidoptera;Polyommatus eros;NA;0.001;NA;0.99999798124352;
FJ428808;Lepidoptera;Polyommatus eros;NA;0.001;NA;0.99999798124352;
FJ428809;Lepidoptera;Polyommatus eros;NA;0.001;NA;0.999996856346662;
FJ428817;Lepidoptera;Polyommatus icarus;NA;0.004;Polyommatus icarus;0.787321449983069;
FJ428818;Lepidoptera;Polyommatus icarus;NA;0.001;Polyommatus icarus;0.75979356402232;
FJ428819;Lepidoptera;Polyommatus icarus;NA;0.001;Polyommatus icarus;0.773101494790543;
FJ428820;Lepidoptera;Polyommatus icarus;NA;NA;NA;0.999999509666107;
FJ428821;Lepidoptera;Polyommatus icarus;NA;0.001;Polyommatus icarus;0.748264214277749;
FJ428822;Lepidoptera;Polyommatus icarus;NA;NA;NA;0.999999509666107;
FJ428825;Lepidoptera;Polyommatus icarus;NA;0.003;NA;0.99999992766227;
FJ435341;Lepidoptera;Polyommatus eros;NA;0.001;Polyommatus erotides;0.76185179953082;
FJ628423;Lepidoptera;Erebia euryale;NA;NA;Erebia euryale;0.885865842941319;
FJ628424;Lepidoptera;Erebia euryale;NA;NA;Erebia euryale;0.887705354255096;
FJ628425;Lepidoptera;Erebia medusa;Erebia medusa;1;Erebia medusa;0.776430243115076;
FJ628426;Lepidoptera;Erebia medusa;NA;0.012;NA;0.999999999065301;
FJ628427;Lepidoptera;Erebia manto;Erebia manto;1;Erebia manto;0.931687947306853;
FJ628428;Lepidoptera;Erebia euryale;NA;NA;Erebia euryale;0.887705354255096;
FJ628429;Lepidoptera;Erebia manto;Erebia manto;1;Erebia manto;0.941275656507444;
FJ628432;Lepidoptera;Erebia epiphron;Erebia epiphron;1;Erebia serotina;0.770552571202559;
FJ628433;Lepidoptera;Erebia epiphron;NA;0.198;Erebia serotina;0.759927913749334;
FJ628435;Lepidoptera;Erebia pronoe;Erebia pronoe;1;Erebia pronoe;0.980058449947045;

FJ628436;Lepidoptera;Erebia pandrose;NA;NA;NA;0.992852600839316;
FJ628437;Lepidoptera;Erebia euryale;NA;NA;Erebia euryale;0.887705354255096;
FJ628438;Lepidoptera;Erebia euryale;Erebia euryale;0.001;Erebia euryale;0.995143815428805;
FJ628439;Lepidoptera;Erebia pronoe;Erebia pronoe;1;Erebia pronoe;0.980792957115092;
FJ628440;Lepidoptera;Erebia euryale;NA;NA;Erebia euryale;0.887705354255096;
FJ628442;Lepidoptera;Erebia euryale;NA;NA;Erebia euryale;0.887705354255096;
FJ628443;Lepidoptera;Erebia euryale;Erebia euryale;NA;Erebia euryale;0.992102328748505;
FJ628444;Lepidoptera;Erebia medusa;NA;0.009;Erebia polaris;0.841187497895335;
FJ628445;Lepidoptera;Erebia medusa;Erebia medusa;0.006;NA;0.999999997702201;
FJ628446;Lepidoptera;Erebia medusa;NA;0.012;NA;0.999999999065301;
FJ628447;Lepidoptera;Erebia pharte;Erebia pharte;1;Erebia pharte;0.999994865861956;
FJ663212;Lepidoptera;Aglais urticae;Aglais urticae;0.996;Aglais urticae;0.86007262958937;
FJ663213;Lepidoptera;Aglais urticae;Aglais urticae;0.002;Aglais urticae;0.904340834515854;
FJ663252;Lepidoptera;Agriades orbitulus sajana;NA;NA;Agriades orbitulus;0.978209678948238;
FJ663253;Lepidoptera;Agriades orbitulus sajana;NA;NA;Agriades orbitulus;0.978209678948238;
FJ663264;Lepidoptera;Apatura ilia;NA;0.039;Apatura ilia;0.977274423026401;
FJ663265;Lepidoptera;Apatura ilia;Apatura ilia;0.018;Apatura ilia;0.978454420945423;
FJ663283;Lepidoptera;Araschnia levana;Araschnia levana;1;Araschnia levana;0.999999772187832;
FJ663284;Lepidoptera;Araschnia levana;Araschnia levana;1;Araschnia levana;0.999999772187832;
FJ663334;Lepidoptera;Boloria napaea;NA;0.152;Boloria napaea;0.81642421149858;
FJ663336;Lepidoptera;Brenthis daphne;Brenthis daphne;0.004;Brenthis daphne;0.969469542867473;
FJ663337;Lepidoptera;Brenthis ino;Brenthis ino;0.001;Brenthis ino;0.994711912827522;
FJ663338;Lepidoptera;Brenthis ino;Brenthis ino;0.001;Brenthis ino;0.994711912827522;
FJ663364;Lepidoptera;Boloria euphrosyne;Boloria euphrosyne;1;Boloria euphrosyne;0.9999883312184;
FJ663365;Lepidoptera;Boloria euphrosyne;Boloria euphrosyne;1;Boloria euphrosyne;0.999970074469922;
FJ663412;Lepidoptera;Colias crocea;NA;0.059;NA;0.999999999999738;
FJ663413;Lepidoptera;Colias crocea;NA;0.059;NA;0.999999999999738;
FJ663414;Lepidoptera;Colias crocea;NA;0.059;NA;0.999999999999738;
FJ663418;Lepidoptera;Colias hyale;NA;0.488;Colias hyale;0.999915488936852;
FJ663439;Lepidoptera;Cupido minimus;NA;0.002;Cupido minimus;0.991793750212648;
FJ663440;Lepidoptera;Cupido minimus;NA;0.019;Cupido minimus;0.997099017544134;
FJ663441;Lepidoptera;Cupido minimus;NA;0.008;Cupido minimus;0.999818742396574;
FJ663445;Lepidoptera;Cupido osiris;Cupido osiris;1;Cupido osiris;0.999999477553951;
FJ663516;Lepidoptera;Erebia pandrose;Erebia pandrose;0.006;Erebia pandrose;0.995149791063603;
FJ663563;Lepidoptera;Cupido argiades;NA;0.499;NA;0.999999999984682;
FJ663564;Lepidoptera;Cupido argiades;NA;0.499;NA;0.999999999984682;
FJ663572;Lepidoptera;Favonius quercus;NA;NA;NA;0.999999999996153;
FJ663573;Lepidoptera;Favonius quercus;NA;NA;NA;0.999999999996893;
FJ663594;Lepidoptera;Gonepteryx rhamni;NA;0.181;Gonepteryx rhamni;0.985430786358543;
FJ663595;Lepidoptera;Gonepteryx rhamni;NA;0.474;Gonepteryx rhamni;0.982076396127858;
FJ663596;Lepidoptera;Lycaena tityrus;Lycaena tityrus;1;Lycaena tityrus;0.999998928238034;
FJ663686;Lepidoptera;Issoria lathonia;Issoria lathonia;1;Issoria lathonia;0.999999999962967;
FJ663724;Lepidoptera;Limenitis camilla;Limenitis camilla;0.009;Limenitis camilla;0.9999828626808;
FJ663725;Lepidoptera;Limenitis camilla;NA;0.054;Limenitis camilla;0.99998730864279;
FJ663736;Lepidoptera;Helleia helle;Lycaena helle;1;Lycaena helle;0.999999984510879;
FJ663737;Lepidoptera;Helleia helle;Lycaena helle;1;Lycaena helle;0.999999984510879;
FJ663738;Lepidoptera;Lycaena phlaeas;Lycaena phlaeas;1;Lycaena phlaeas;0.999999996363186;
FJ663739;Lepidoptera;Lycaena phlaeas;Lycaena phlaeas;1;Lycaena phlaeas;0.9999999859225;
FJ663749;Lepidoptera;Lysandra coridon;NA;NA;Lysandra coridon;0.754269905766234;
FJ663750;Lepidoptera;Lysandra coridon;Lysandra coridon;NA;Lysandra coridon;0.735779770406819;
FJ663756;Lepidoptera;Maniola jurtina;NA;0.005;Maniola jurtina;0.714129421849189;
FJ663757;Lepidoptera;Maniola jurtina;NA;0.161;NA;0.999999999999418;
FJ663758;Lepidoptera;Maniola jurtina;NA;0.038;NA;0.999999999999524;
FJ663759;Lepidoptera;Maniola jurtina;NA;0.01;NA;0.999999999998599;
FJ663760;Lepidoptera;Maniola jurtina;NA;0.038;NA;0.999999999999524;
FJ663769;Lepidoptera;Polyommatus daphnis;NA;0.001;Polyommatus daphnis;0.942172634976058;
FJ663770;Lepidoptera;Polyommatus daphnis;NA;0.001;Polyommatus daphnis;0.994093721308241;
FJ663771;Lepidoptera;Polyommatus daphnis;Polyommatus daphnis;0.008;Polyommatus daphnis;0.997264841617925;
FJ663772;Lepidoptera;Polyommatus daphnis;NA;0.098;Polyommatus daphnis;0.996542893341734;
FJ663773;Lepidoptera;Polyommatus daphnis;NA;0.001;Polyommatus daphnis;0.942172634976058;
FJ663784;Lepidoptera;Melitaea cinxia;Melitaea cinxia;0.021;Melitaea cinxia;0.999999999999176;
FJ663785;Lepidoptera;Melitaea cinxia;Melitaea cinxia;0.05;Melitaea cinxia;0.999999999999119;
FJ663854;Lepidoptera;Nymphalis antiopa;NA;0.23;Nymphalis antiopa;0.999999944198096;
FJ663885;Lepidoptera;Pararge aegeria;Pararge aegeria;1;Pararge aegeria;0.999999999730363;
FJ663932;Lepidoptera;Pieris napi;NA;0.013;Pieris napi;0.798949532876188;
FJ663951;Lepidoptera;Plebejus argus;NA;0.048;Plebejus argus;0.982596100749942;
FJ663952;Lepidoptera;Plebejus argus;NA;0.044;Plebejus argus;0.975622330747864;
FJ663953;Lepidoptera;Plebejus argyrognomon;Plebejus argyrognomon;0.003;Plebejus argyrognomon;0.927010765218643;
FJ663954;Lepidoptera;Plebejus argyrognomon;NA;0.001;Plebejus argyrognomon;0.822886398456556;
FJ663955;Lepidoptera;Plebejus argyrognomon;NA;0.002;NA;0.998323393743912;
FJ663956;Lepidoptera;Plebejus argyrognomon;NA;0.001;NA;0.997791535991658;
FJ663957;Lepidoptera;Plebejus argyrognomon;NA;0.001;NA;0.998508017873438;
FJ663958;Lepidoptera;Plebejus argyrognomon;NA;0.001;NA;0.994620093546684;
FJ663959;Lepidoptera;Plebejus argyrognomon;NA;0.001;NA;0.994620093546684;

FJ663976;Lepidoptera;Nymphalis c-album;NA;0.085;NA;0.999999279624904;
FJ663977;Lepidoptera;Nymphalis c-album;NA;0.215;NA;0.999999192918557;
FJ663978;Lepidoptera;Nymphalis c-album;NA;0.215;NA;0.999999192918557;
FJ663979;Lepidoptera;Nymphalis c-album;NA;0.215;NA;0.999999192918557;
FJ664034;Lepidoptera;Satyrus ferula;Satyrus ferula;0.001;Satyrus ferula;0.993398569985402;
FJ664047;Lepidoptera;Thecla betulae;Thecla betulae;1;Thecla betulae;0.999999999967031;
FJ664048;Lepidoptera;Thecla betulae;Thecla betulae;1;Thecla betulae;0.999999999889212;
FJ664075;Lepidoptera;Agriades optilete;Agriades optilete;1;Agriades optilete;0.999999990133006;
FJ664076;Lepidoptera;Agriades optilete;Agriades optilete;1;Agriades optilete;0.999999990133006;
FJ664077;Lepidoptera;Agriades optilete;Agriades optilete;1;Agriades optilete;0.99999999183251;
FJ664078;Lepidoptera;Vanessa atalanta;Vanessa atalanta;1;Vanessa atalanta;0.999999999739629;
FJ664079;Lepidoptera;Vanessa atalanta;Vanessa atalanta;1;Vanessa atalanta;0.999999999673548;
FJ938179;Lepidoptera;Erebia medusa;NA;0.009;Erebia medusa;0.986275612856827;
FJ938180;Lepidoptera;Erebia medusa;NA;0.035;Erebia medusa;0.984273665813791;
FJ938181;Lepidoptera;Erebia medusa;NA;0.009;Erebia medusa;0.986275612856827;
FJ938182;Lepidoptera;Erebia medusa;NA;0.009;Erebia medusa;0.986275612856827;
FJ938183;Lepidoptera;Erebia medusa;NA;0.04;Erebia medusa;0.984042649596477;
FJ938184;Lepidoptera;Erebia medusa;NA;0.022;Erebia medusa;0.986197124545351;
FJ938185;Lepidoptera;Erebia medusa;NA;0.009;Erebia medusa;0.986275612856827;
FJ938186;Lepidoptera;Erebia medusa;NA;0.009;Erebia medusa;0.986275612856827;
FJ938187;Lepidoptera;Erebia medusa;NA;0.04;Erebia medusa;0.984042649596477;
FJ938188;Lepidoptera;Erebia medusa;NA;0.009;Erebia medusa;0.986275612856827;
FJ938189;Lepidoptera;Erebia medusa;NA;0.016;Erebia medusa;0.988942863975822;
FJ938190;Lepidoptera;Erebia medusa;NA;0.009;Erebia medusa;0.986275612856827;
FJ938191;Lepidoptera;Erebia medusa;NA;0.04;Erebia medusa;0.984042649596477;
FJ938192;Lepidoptera;Erebia oeme;NA;0.031;Erebia oeme;0.999999999935056;
FJ938193;Lepidoptera;Erebia oeme;Erebia oeme;0.005;Erebia oeme;0.999999999877474;
FJ938194;Lepidoptera;Erebia oeme;Erebia oeme;0.003;Erebia oeme;0.999999999681421;
FJ938195;Lepidoptera;Erebia oeme;Erebia oeme;0.003;Erebia oeme;0.999999999681421;
FJ938196;Lepidoptera;Erebia oeme;Erebia oeme;0.003;Erebia oeme;0.999999999681421;
FJ943961;Lepidoptera;Lysandra coridon;Lysandra coridon;NA;NA;0.997782410243321;
FN601259;Lepidoptera;Callophrys rubi;NA;0.001;NA;0.999999982170419;
FN601260;Lepidoptera;Callophrys rubi;NA;0.001;NA;0.999999982170419;
FN601274;Lepidoptera;Callophrys avis;NA;0.001;NA;0.807759286975261;
FN601282;Lepidoptera;Callophrys rubi;NA;0.001;Callophrys rubi;0.705657102729907;
FN601285;Lepidoptera;Callophrys rubi;NA;0.001;NA;0.999999982170419;
FN601301;Lepidoptera;Callophrys rubi;NA;0.001;NA;0.999999982170419;
FN601308;Lepidoptera;Callophrys rubi;NA;0.001;NA;0.999999982170419;
FN601312;Lepidoptera;Callophrys rubi;NA;0.001;NA;0.999999982170419;
FN601319;Lepidoptera;Callophrys rubi;NA;0.001;NA;0.999999982170419;
FN601329;Lepidoptera;Callophrys rubi;NA;0.001;NA;0.999999982170419;
FN601330;Lepidoptera;Callophrys rubi;NA;0.001;NA;0.999999982170419;
FR989949.1;Lepidoptera;Plebejus argus;NA;0.123;Plebejus argus;0.980927835351298;
FR990065.1;Lepidoptera;Glaucopsyche alexis;NA;NA;Glaucopsyche alexis;0.991019528246159;
FR990152.1;Lepidoptera;Ochlodes sylvanus;NA;0.108;Ochlodes sylvanus;0.996827249893169;
GBLAA1157-15;Lepidoptera;Cyaniris semiargus;NA;NA;Cyaniris semiargus;0.999051841766953;
GBLAA1166-15;Lepidoptera;Thymelicus sylvestris;Thymelicus sylvestris;1;Thymelicus sylvestris;0.99999997998853;
GBLAA1167-15;Lepidoptera;Thymelicus sylvestris;Thymelicus sylvestris;1;Thymelicus sylvestris;0.999999981568351;
GBLAA133-14;Lepidoptera;Maniola jurtina;NA;NA;NA;0.99999999991;
GBLAA372-14;Lepidoptera;Aphantopus hyperantus;Aphantopus hyperantus;0.004;Aphantopus hyperantus;0.999598909345147;
GBLAA407-14;Lepidoptera;Thymelicus lineola;Thymelicus lineola;1;Thymelicus lineola;0.999945767337001;
GBLAB121-13;Lepidoptera;Aricia agestis;NA;0.253;Aricia agestis;0.996350017387504;
GBLAB123-13;Lepidoptera;Polyommatus icarus;NA;0.001;Polyommatus icarus;0.984622665164294;
GBLAB137-13;Lepidoptera;Thymelicus sylvestris;Thymelicus sylvestris;1;Thymelicus sylvestris;0.999999985935205;
GBLAB138-13;Lepidoptera;Thymelicus lineola;Thymelicus lineola;1;Thymelicus lineola;0.999992304433643;
GBLAB144-13;Lepidoptera;Aphantopus hyperantus;Aphantopus hyperantus;0.004;Aphantopus hyperantus;0.999598909345147;
GBLAB145-13;Lepidoptera;Maniola jurtina;NA;NA;NA;0.99999999991;
GBLAB149-13;Lepidoptera;Coenonympha arcania;NA;0.172;Coenonympha arcania;0.963036309473038;
GBLAB318-13;Lepidoptera;Melitaea cinxia;Melitaea cinxia;1;Melitaea cinxia;0.999999999999858;
GBLAC266-13;Lepidoptera;Polyommatus icarus;NA;0.001;Polyommatus icarus;0.96905816849887;
GBLAC281-13;Lepidoptera;Thymelicus sylvestris;NA;0.324;Thymelicus sylvestris;0.999940778442454;
GBLAC282-13;Lepidoptera;Thymelicus lineola;Thymelicus lineola;1;Thymelicus lineola;0.999993723282816;
GBLAC941-13;Lepidoptera;Pieris napi;NA;NA;Pieris napi;0.807328036910527;
GBLAD064-14;Lepidoptera;Pieris napi;NA;0.028;Pieris napi;0.888205720664699;
GBLAD096-14;Lepidoptera;Pieris napi;NA;0.011;Pieris napi;0.801985394724814;
GBLAD098-14;Lepidoptera;Maniola jurtina;NA;0.074;NA;0.999999999996465;
GBLAD101-14;Lepidoptera;Aphantopus hyperantus;Aphantopus hyperantus;1;Aphantopus hyperantus;0.999998423174411;
GBLAD257-14;Lepidoptera;Boloria euphrosyne;Boloria euphrosyne;0.999;Boloria euphrosyne;0.999987921564426;

GBLAD931-14;Lepidoptera;Pieris napi;NA;0.011;Pieris napi;0.801985394724814;
GBLAD949-14;Lepidoptera;Pyrgus cacaliae;Pyrgus cacaliae;0.001;Pyrgus cacaliae;0.99354498208327;
GBLAF147-14;Lepidoptera;Polyommatus icarus;Polyommatus icarus;0.001;Polyommatus icarus;0.999848859399272;
GBLAF396-14;Lepidoptera;Pieris napi;NA;0.028;Pieris napi;0.888205720664699;
GBLAF397-14;Lepidoptera;Aphantopus hyperantus;Aphantopus hyperantus;0.004;Aphantopus hyperantus;0.999598909345147;
GBLAF778-14;Lepidoptera;Aricia agestis;NA;0.067;Aricia agestis;0.999509743227786;
GMBMR047-17;Lepidoptera;Pieris rapae;NA;NA;Pieris rapae;0.999943665698249;
GMBUA102-14;Lepidoptera;Lasiommata maera;Lasiommata maera;1;Lasiommata maera;0.999995059298625;
GMBUB1924-14;Lepidoptera;Hamearis lucina;NA;NA;Hamearis lucina;0.999999999841009;
GMBUB1935-14;Lepidoptera;Satyrium pruni;NA;0.486;Satyrium pruni;0.988466558320733;
GMBUD002-14;Lepidoptera;Brintesia circe;Brintesia circe;1;Brintesia circe;0.999999511269385;
GMBUD003-14;Lepidoptera;Maniola jurtina;NA;0.001;NA;0.999999999874528;
GMBUD007-14;Lepidoptera;Maniola jurtina;NA;0.007;NA;0.999999999950132;
GMBUD008-14;Lepidoptera;Lasiommata megera;Lasiommata megera;1;Lasiommata megera;0.999969474203344;
GMBUD009-14;Lepidoptera;Pieris rapae;NA;0.121;Pieris rapae;0.999977523517787;
GMBUD010-14;Lepidoptera;Lasiommata megera;Lasiommata megera;1;Lasiommata megera;0.999966665008664;
GMBUD013-14;Lepidoptera;Maniola jurtina;NA;0.001;NA;0.99999999995215;
GMBUD014-14;Lepidoptera;Maniola jurtina;NA;0.004;NA;0.999999999974408;
GMBUD125-14;Lepidoptera;Maniola jurtina;NA;0.006;NA;0.999999999971332;
GMBUD128-14;Lepidoptera;Coenonympha pamphilus;Coenonympha pamphilus;1;Coenonympha pamphilus;0.999999999978996;
GMBUD129-14;Lepidoptera;Ochlodes sylvanus;NA;NA;Ochlodes sylvanus;0.994343320156993;
GMBUD131-14;Lepidoptera;Maniola jurtina;NA;0.009;NA;0.999999999976504;
GMBUD134-14;Lepidoptera;Aphantopus hyperantus;Aphantopus hyperantus;0.019;Aphantopus hyperantus;0.999994626926675;
GMBUD135-14;Lepidoptera;Lasiommata megera;Lasiommata megera;0.999;Lasiommata megera;0.999966844083951;
GMBUD136-14;Lepidoptera;Aphantopus hyperantus;Aphantopus hyperantus;0.003;Aphantopus hyperantus;0.999998902508542;
GMBUD137-14;Lepidoptera;Maniola jurtina;NA;0.009;NA;0.999999999976504;
GMBUD138-14;Lepidoptera;Aphantopus hyperantus;Aphantopus hyperantus;0.003;Aphantopus hyperantus;0.999998902508542;
GMBUE016-14;Lepidoptera;Plebejus argus;NA;0.126;Plebejus argus;0.981193877291878;
GMBUE047-14;Lepidoptera;Coenonympha pamphilus;Coenonympha pamphilus;1;Coenonympha pamphilus;0.999999999977632;
GMBUE052-14;Lepidoptera;Coenonympha pamphilus;Coenonympha pamphilus;1;Coenonympha pamphilus;0.999999999995083;
GMBUE053-14;Lepidoptera;Lasiommata megera;Lasiommata megera;1;Lasiommata megera;0.999969653368234;
GMBUF002-14;Lepidoptera;Coenonympha pamphilus;Coenonympha pamphilus;1;Coenonympha pamphilus;0.999999999982208;
GMBUF081-14;Lepidoptera;Coenonympha pamphilus;Coenonympha pamphilus;1;Coenonympha pamphilus;0.999999999994742;
GMBUG001-14;Lepidoptera;Maniola jurtina;NA;0.009;NA;0.999999999976504;
GMBUG002-14;Lepidoptera;Maniola jurtina;NA;0.001;NA;0.999999999946141;
GMBUG003-14;Lepidoptera;Arethusana arethusa;NA;0.488;Arethusana arethusa;0.937870288270298;
GMBUG022-14;Lepidoptera;Coenonympha pamphilus;Coenonympha pamphilus;1;Coenonympha pamphilus;0.999999999983373;
GMBUG2254-14;Lepidoptera;Lasiommata maera;Lasiommata maera;1;Lasiommata maera;0.999996028148232;
GMGMA487-14;Lepidoptera;Celastrina argiolus;Celastrina argiolus;1;Celastrina argiolus;0.999330495887851;
GMGMA491-14;Lepidoptera;Celastrina argiolus;Celastrina argiolus;0.997;Celastrina argiolus;0.99933493633429;
GMGMA501-14;Lepidoptera;Thymelicus sylvestris;Thymelicus sylvestris;1;Thymelicus sylvestris;0.999999924993293;
GMGMA518-14;Lepidoptera;Celastrina argiolus;Celastrina argiolus;0.997;Celastrina argiolus;0.999579184234861;
GMGMA529-14;Lepidoptera;Thymelicus sylvestris;Thymelicus sylvestris;1;Thymelicus sylvestris;0.999999762854258;
GMGMA536-14;Lepidoptera;Maniola jurtina;NA;0.003;NA;0.999999999430614;
GMGMB017-14;Lepidoptera;Celastrina argiolus;Celastrina argiolus;0.999;Celastrina argiolus;0.999636359687375;
GMGMB018-14;Lepidoptera;Celastrina argiolus;Celastrina argiolus;0.999;Celastrina argiolus;0.999622090292273;
GMGMB019-14;Lepidoptera;Celastrina argiolus;Celastrina argiolus;0.999;Celastrina argiolus;0.999636359687375;
GMGMB470-14;Lepidoptera;Celastrina argiolus;Celastrina argiolus;1;Celastrina argiolus;0.999686094051245;
GMGMC014-14;Lepidoptera;Celastrina argiolus;Celastrina argiolus;1;Celastrina argiolus;0.999686094051245;
GMGMC027-14;Lepidoptera;Thymelicus sylvestris;Thymelicus sylvestris;1;Thymelicus sylvestris;0.999999950165774;
GMGMC030-14;Lepidoptera;Vanessa cardui;Vanessa cardui;1;Vanessa cardui;0.999999257400808;

GMGMC239-14;Lepidoptera;Lasiommata megera;Lasiommata megera;1;Lasiommata megera;0.999966948144719;
GMGMC240-14;Lepidoptera;Maniola jurtina;NA;NA;NA;0.999999998961499;
GMGMC250-14;Lepidoptera;Celastrina argiolus;Celastrina argiolus;0.999;Celastrina argiolus;0.999666716978783;
GMGMH1425-14;Lepidoptera;Araschnia levana;Araschnia levana;1;Araschnia levana;0.999999832664983;
GMGMH1431-14;Lepidoptera;Lasiommata megera;Lasiommata megera;1;Lasiommata megera;0.999970342842984;
GMGMI201-14;Lepidoptera;Lasiommata megera;Lasiommata megera;0.999;Lasiommata megera;0.999966844083951;
GMGMI210-14;Lepidoptera;Lasiommata megera;Lasiommata megera;0.999;Lasiommata megera;0.999966844083951;
GMGMI217-14;Lepidoptera;Pararge aegeria;Pararge aegeria;1;Pararge aegeria;0.999999999959414;
GMGMI218-14;Lepidoptera;Lasiommata megera;Lasiommata megera;0.999;Lasiommata megera;0.999966844083951;
GMGMI229-14;Lepidoptera;Lasiommata megera;Lasiommata megera;1;Lasiommata megera;0.999966665008664;
GMGMJ032-14;Lepidoptera;Satyrium pruni;NA;0.497;Satyrium pruni;0.989233904372907;
GMGMJ033-14;Lepidoptera;Lasiommata megera;Lasiommata megera;1;Lasiommata megera;0.999975631755706;
GMGMJ045-14;Lepidoptera;Lasiommata megera;Lasiommata megera;1;Lasiommata megera;0.999969653368234;
GMGMK191-14;Lepidoptera;Thymelicus sylvestris;Thymelicus sylvestris;1;Thymelicus sylvestris;0.999999949110021;
GMGMK192-14;Lepidoptera;Maniola jurtina;NA;0.004;NA;0.999999999974408;
GMGMK193-14;Lepidoptera;Thymelicus sylvestris;Thymelicus sylvestris;1;Thymelicus sylvestris;0.999999949110021;
GMGML016-14;Lepidoptera;Maniola jurtina;NA;NA;NA;0.999999999997274;
GMGML017-14;Lepidoptera;Maniola jurtina;NA;0.004;NA;0.999999999865405;
GMGML020-14;Lepidoptera;Maniola jurtina;NA;0.009;Maniola jurtina;0.939166605292266;
GMGML021-14;Lepidoptera;Maniola jurtina;NA;0.009;NA;0.999999999976504;
GMGML022-14;Lepidoptera;Maniola jurtina;NA;0.009;NA;0.999999999976504;
GMGML025-14;Lepidoptera;Maniola jurtina;NA;0.007;NA;0.999999999941172;
GMGML026-14;Lepidoptera;Maniola jurtina;NA;NA;NA;0.999999998779753;
GMGML027-14;Lepidoptera;Maniola jurtina;NA;0.005;Maniola chia;0.741043173259956;
GMGML028-14;Lepidoptera;Maniola jurtina;Maniola jurtina;0.023;Maniola jurtina;0.877189324384157;
GMGML029-14;Lepidoptera;Maniola jurtina;NA;0.021;NA;0.999999999949024;
GMGML031-14;Lepidoptera;Maniola jurtina;NA;NA;NA;0.999999999936473;
GMGML032-14;Lepidoptera;Maniola jurtina;NA;0.001;NA;0.999999999987881;
GMGML033-14;Lepidoptera;Maniola jurtina;NA;0.018;NA;0.999999998934134;
GMGML043-14;Lepidoptera;Thymelicus lineola;Thymelicus lineola;1;Thymelicus lineola;0.999992047013555;
GMGML046-14;Lepidoptera;Thymelicus lineola;Thymelicus lineola;1;Thymelicus lineola;0.999977655372067;
GMGML051-14;Lepidoptera;Thymelicus sylvestris;Thymelicus sylvestris;1;Thymelicus sylvestris;0.999999942320842;
GMGML056-14;Lepidoptera;Thymelicus sylvestris;Thymelicus sylvestris;1;Thymelicus sylvestris;0.999999937939352;
GMGML063-14;Lepidoptera;Thymelicus sylvestris;Thymelicus sylvestris;1;Thymelicus sylvestris;0.99999996158249;
GMGMM009-14;Lepidoptera;Maniola jurtina;NA;0.002;Maniola jurtina;0.774995587786062;
GMGMM012-14;Lepidoptera;Maniola jurtina;NA;0.005;Maniola jurtina;0.946979799614752;
GMGMM014-14;Lepidoptera;Maniola jurtina;NA;0.003;NA;0.999999999990033;
GMGMM015-14;Lepidoptera;Maniola jurtina;Maniola jurtina;0.009;Maniola jurtina;0.829226790520797;
GMGMM016-14;Lepidoptera;Maniola jurtina;NA;0.003;NA;0.999999999990033;
GMGMM018-14;Lepidoptera;Maniola jurtina;NA;NA;Maniola jurtina;0.704272131898308;
GMGMM019-14;Lepidoptera;Maniola jurtina;NA;0.116;NA;0.999999999987256;
GMGMM020-14;Lepidoptera;Maniola jurtina;NA;0.007;NA;0.999999999987721;
GMGMM021-14;Lepidoptera;Lasiommata megera;Lasiommata megera;1;Lasiommata megera;0.999969474203344;
GMGMM050-14;Lepidoptera;Lasiommata megera;Lasiommata megera;0.999;Lasiommata megera;0.999949012846764;
GMGMM058-14;Lepidoptera;Maniola jurtina;NA;0.003;NA;0.999999999990033;
GMGMM060-14;Lepidoptera;Maniola jurtina;NA;0.003;NA;0.999999999990033;
GMGMM061-14;Lepidoptera;Maniola jurtina;NA;0.003;NA;0.999999999990033;
GMGMM062-14;Lepidoptera;Maniola jurtina;NA;0.003;NA;0.999999999990033;
GMGMM063-14;Lepidoptera;Maniola jurtina;NA;0.003;NA;0.999999999990033;
GMGMM064-14;Lepidoptera;Lasiommata megera;Lasiommata megera;1;Lasiommata megera;0.999955870837446;
GMGMM065-14;Lepidoptera;Lasiommata megera;Lasiommata megera;1;Lasiommata megera;0.999939218657025;
GMGMM067-14;Lepidoptera;Lasiommata megera;Lasiommata megera;1;Lasiommata megera;0.999969474203344;
GMGMM069-14;Lepidoptera;Maniola jurtina;NA;0.003;NA;0.999999999990033;
GMGMM070-14;Lepidoptera;Maniola jurtina;NA;0.003;NA;0.999999999990033;
GMGMM071-14;Lepidoptera;Maniola jurtina;NA;0.003;NA;0.999999999990033;
GMGMM072-14;Lepidoptera;Maniola jurtina;NA;0.003;NA;0.999999999990033;
GMGMM073-14;Lepidoptera;Maniola jurtina;NA;0.002;Maniola jurtina;0.860414212487598;

GMGMM074-14;Lepidoptera;Maniola jurtina;NA;0.004;NA;0.999999999974408;
GMGMM075-14;Lepidoptera;Lasiommata megera;Lasiommata megera;1;Lasiommata megera;0.999969653368234;
GMGMM076-14;Lepidoptera;Maniola jurtina;NA;0.003;NA;0.999999999990033;
GMGMM077-14;Lepidoptera;Thymelicus sylvestris;Thymelicus sylvestris;1;Thymelicus sylvestris;0.99999993699595;
GMGMM078-14;Lepidoptera;Lasiommata megera;Lasiommata megera;1;Lasiommata megera;0.999969474203344;
GMGMM080-14;Lepidoptera;Maniola jurtina;NA;0.006;NA;0.999999999871436;
GMGMM088-14;Lepidoptera;Lasiommata megera;Lasiommata megera;1;Lasiommata megera;0.999969474203344;
GMGMM095-14;Lepidoptera;Thymelicus sylvestris;Thymelicus sylvestris;1;Thymelicus sylvestris;0.999999938532882;
GMGMM097-14;Lepidoptera;Maniola jurtina;NA;0.004;NA;0.999999999974408;
GMGMN052-14;Lepidoptera;Maniola jurtina;NA;0.004;NA;0.999999999974408;
GMGMN054-14;Lepidoptera;Lasiommata megera;Lasiommata megera;0.001;Lasiommata megera;0.999905026489642;
GMGMN056-14;Lepidoptera;Maniola jurtina;NA;0.009;NA;0.999999999976504;
GMGMN068-14;Lepidoptera;Lasiommata megera;Lasiommata megera;0.999;Lasiommata megera;0.999966844083951;
GMGMN069-14;Lepidoptera;Issoria lathonia;Issoria lathonia;1;Issoria lathonia;0.99999999987412;
GMGMN071-14;Lepidoptera;Maniola jurtina;NA;0.008;NA;0.999999999876443;
GMGMN073-14;Lepidoptera;Pyronia tithonus;Pyronia tithonus;1;Pyronia tithonus;1;
GMGMN090-14;Lepidoptera;Lasiommata megera;Lasiommata megera;0.999;Lasiommata megera;0.999952224561591;
GMGMN091-14;Lepidoptera;Maniola jurtina;NA;0.009;NA;0.999999999976504;
GMGMN094-14;Lepidoptera;Maniola jurtina;NA;0.009;NA;0.999999999976504;
GMGMN096-14;Lepidoptera;Lasiommata megera;Lasiommata megera;0.999;Lasiommata megera;0.999966844083951;
GMGMN097-14;Lepidoptera;Maniola jurtina;NA;0.005;Maniola jurtina;0.934616755277837;
GMGMN100-14;Lepidoptera;Maniola jurtina;NA;0.001;NA;0.999999999987881;
GMGMN102-14;Lepidoptera;Maniola jurtina;NA;NA;NA;0.999999999796435;
GMGMN103-14;Lepidoptera;Maniola jurtina;NA;0.005;Maniola jurtina;0.934616755277837;
GMGMN104-14;Lepidoptera;Maniola jurtina;NA;0.002;NA;0.999999999903496;
GMGMN105-14;Lepidoptera;Maniola jurtina;NA;0.002;NA;0.999999999903496;
GMGMN107-14;Lepidoptera;Maniola jurtina;NA;0.009;NA;0.999999999976504;
GMGMN108-14;Lepidoptera;Lasiommata megera;Lasiommata megera;1;Lasiommata megera;0.999899207286718;
GMGMN109-14;Lepidoptera;Lasiommata megera;Lasiommata megera;1;Lasiommata megera;0.99997012617639;
GMGMN113-14;Lepidoptera;Lasiommata megera;Lasiommata megera;1;Lasiommata megera;0.99996989668155;
GMGMN117-14;Lepidoptera;Maniola jurtina;NA;0.009;NA;0.999999999976504;
GMGMN120-14;Lepidoptera;Lasiommata megera;Lasiommata megera;1;Lasiommata megera;0.999969653368234;
GMGMN121-14;Lepidoptera;Maniola jurtina;NA;0.009;NA;0.999999999976504;
GMGMN124-14;Lepidoptera;Lasiommata megera;Lasiommata megera;1;Lasiommata megera;0.999969653368234;
GMGMN127-14;Lepidoptera;Thymelicus lineola;Thymelicus lineola;1;Thymelicus lineola;0.999986878711549;
GMGMN141-14;Lepidoptera;Lasiommata megera;Lasiommata megera;1;Lasiommata megera;0.999973871152596;
GMGMN143-14;Lepidoptera;Maniola jurtina;NA;0.009;NA;0.999999999976504;
GMGMN144-14;Lepidoptera;Maniola jurtina;NA;0.009;NA;0.999999999976504;
GMGMU3192-20;Lepidoptera;Pieris napi;NA;0.017;Pieris napi;0.805583713910701;
GMGMU3193-20;Lepidoptera;Pieris napi;NA;NA;Pieris napi;0.917869117371985;
GMGMV2344-20;Lepidoptera;Pieris napi;NA;0.025;Pieris napi;0.890466414022189;
GMGRE2270-13;Lepidoptera;Lasiommata maera;Lasiommata maera;1;Lasiommata maera;0.999997723969845;
GMGRE2271-13;Lepidoptera;Lasiommata maera;Lasiommata maera;1;Lasiommata maera;0.999996875417757;
GMGRE2272-13;Lepidoptera;Lasiommata maera;Lasiommata maera;1;Lasiommata maera;0.999997723969845;
GMGRF5621-13;Lepidoptera;Lasiommata maera;Lasiommata maera;1;Lasiommata maera;0.999997516733302;
GMRSF081-14;Lepidoptera;Araschnia levana;Araschnia levana;1;Araschnia levana;0.999999236241685;
GMRSU120-15;Lepidoptera;Heteropterus morpheus;NA;NA;Heteropterus morpheus;0.999999999999801;
GMRSW019-15;Lepidoptera;Aphantopus hyperantus;Aphantopus hyperantus;0.002;Aphantopus hyperantus;0.999998684236415;
GQ128942;Lepidoptera;Agriades glandon;Agriades glandon;NA;NA;0.860288767951466;
GQ128945;Lepidoptera;Agriades orbitulus;NA;0.002;NA;0.934236298474903;
GQ128947;Lepidoptera;Cupido minimus;NA;0.017;NA;0.878333322986603;
GQ128953;Lepidoptera;Aricia eumedon;NA;0.487;NA;0.864612642815733;
GQ128976;Lepidoptera;Lysandra coridon;NA;NA;NA;0.819281743141762;
GQ129011;Lepidoptera;Agriades optilete optilete;Agriades optilete;1;NA;0.88395019607177;
GQ200937;Lepidoptera;Melanargia galathea;Melanargia galathea;NA;Melanargia galathea;0.981855578470499;
GQ200938;Lepidoptera;Melanargia galathea;Melanargia galathea;NA;Melanargia galathea;0.981855578470499;
GQ200939;Lepidoptera;Melanargia galathea;Melanargia galathea;NA;Melanargia galathea;0.987561356582775;

GQ200940;Lepidoptera;Melanargia galathea;Melanargia galathea;NA;Melanargia galathea;0.987561356582775;
GQ200942;Lepidoptera;Melanargia galathea;Melanargia galathea;NA;Melanargia galathea;0.985355101480436;
GQ200943;Lepidoptera;Melanargia galathea;Melanargia galathea;NA;Melanargia galathea;0.981855578470499;
GQ200944;Lepidoptera;Melanargia galathea;Melanargia galathea;0.999;Melanargia galathea;0.991824592076933;
GQ200945;Lepidoptera;Melanargia galathea;Melanargia galathea;0.002;Melanargia galathea;0.987025289954079;
GQ885173;Lepidoptera;Polyommatus icarus;NA;NA;NA;0.999999777000389;
GU244492;Lepidoptera;Polyommatus eros;NA;0.003;NA;0.999956428652965;
GU559737;Lepidoptera;Polyommatus celina;NA;0.009;NA;0.763308217155514;
GU559741;Lepidoptera;Polyommatus dolus;Polyommatus dolus;0.002;Polyommatus dolus;0.948273592698561;
GU655002;Lepidoptera;Pyrgus malvae;Pyrgus malvae;NA;Pyrgus malvae;0.980121140374433;
GU655003;Lepidoptera;Pyrgus malvae;Pyrgus malvae;NA;Pyrgus malvae;0.980121140374433;
GU655004;Lepidoptera;Pyrgus alveus;NA;NA;Pyrgus alveus;0.827119616974978;
GU655005;Lepidoptera;Polyommatus icarus;NA;0.002;Polyommatus icarus;0.997871588783533;
GU655007;Lepidoptera;Plebejus argyrognomon;NA;0.001;NA;0.994620093546684;
GU655008;Lepidoptera;Pararge aegeria;Pararge aegeria;1;Pararge aegeria;0.999999999963165;
GU655009;Lepidoptera;Pararge aegeria;Pararge aegeria;1;Pararge aegeria;0.999999999963165;
GU655010;Lepidoptera;Ochlodes sylvanus;NA;0.316;Ochlodes sylvanus;0.996318754838625;
GU655011;Lepidoptera;Melitaea cinxia;Melitaea cinxia;1;Melitaea cinxia;0.999999999999403;
GU655015;Lepidoptera;Lasiommata megera;Lasiommata megera;1;Lasiommata megera;0.999928528239676;
GU655016;Lepidoptera;Gonepteryx rhamni;NA;0.474;Gonepteryx rhamni;0.982076396127858;
GU655017;Lepidoptera;Glaucopsyche alexis;NA;NA;Glaucopsyche alexis;0.979004200903647;
GU655018;Lepidoptera;Erebia medusa;NA;0.037;Erebia medusa;0.967935760207416;
GU655019;Lepidoptera;Cupido minimus;NA;0.004;Cupido minimus;0.99987402211542;
GU655020;Lepidoptera;Coenonympha pamphilus;Coenonympha pamphilus;1;Coenonympha pamphilus;0.999999999999744;
GU655022;Lepidoptera;Carterocephalus palaemon;NA;0.052;Carterocephalus palaemon;0.728445051795325;
GU655023;Lepidoptera;Carcharodus alceae;Carcharodus alceae;1;Carcharodus alceae;0.999999996515299;
GU655025;Lepidoptera;Argynnis aglaja;NA;0.001;NA;0.999999999999586;
GU655026;Lepidoptera;Araschnia levana;Araschnia levana;1;Araschnia levana;0.999999772187832;
GU655027;Lepidoptera;Aphantopus hyperantus;Aphantopus hyperantus;0.006;Aphantopus hyperantus;0.999998615504572;
GU668027;Lepidoptera;Cupido minimus;NA;0.001;Cupido minimus;0.99992423685743;
GU668028;Lepidoptera;Cupido minimus;NA;0.009;Cupido minimus;0.999791453712643;
GU668029;Lepidoptera;Cupido osiris;Cupido osiris;1;Cupido osiris;0.999999944757265;
GU668031;Lepidoptera;Colias crocea;NA;0.059;NA;0.999999999999738;
GU668032;Lepidoptera;Colias crocea;NA;0.059;NA;0.999999999999738;
GU668033;Lepidoptera;Cupido minimus;NA;0.006;Cupido minimus;0.999768371759258;
GU668034;Lepidoptera;Colias alfacariensis;Colias alfacariensis;1;Colias alfacariensis;0.999857768905063;
GU668035;Lepidoptera;Colias alfacariensis;Colias alfacariensis;0.001;Colias alfacariensis;0.999857790810452;
GU668036;Lepidoptera;Colias alfacariensis;Colias alfacariensis;0.001;Colias alfacariensis;0.999857790810452;
GU668037;Lepidoptera;Colias alfacariensis;Colias alfacariensis;0.001;Colias alfacariensis;0.999857790810452;
GU668038;Lepidoptera;Coenonympha pamphilus;Coenonympha pamphilus;1;Coenonympha pamphilus;0.999999999999744;
GU668039;Lepidoptera;Coenonympha pamphilus;Coenonympha pamphilus;1;Coenonympha pamphilus;0.999999999999744;
GU668040;Lepidoptera;Coenonympha pamphilus;Coenonympha pamphilus;1;Coenonympha pamphilus;0.999999999999744;
GU668041;Lepidoptera;Coenonympha pamphilus;Coenonympha pamphilus;1;Coenonympha pamphilus;0.999999999999744;
GU668042;Lepidoptera;Coenonympha glycerion;NA;0.28;Coenonympha glycerion;0.999998036263913;
GU668043;Lepidoptera;Coenonympha glycerion;NA;0.28;Coenonympha glycerion;0.999998036263913;
GU669369;Lepidoptera;Lycaena phlaeas;Archiearis parthenias;1;Archiearis parthenias;0.999998637442094;
GU669600;Lepidoptera;Plebejus idas;NA;0.007;NA;0.999546324331549;
GU669601;Lepidoptera;Plebejus idas;NA;0.007;NA;0.999546324331549;
GU669602;Lepidoptera;Plebejus idas;NA;0.007;NA;0.999546324331549;
GU669603;Lepidoptera;Plebejus idas;NA;0.007;NA;0.999546324331549;
GU669604;Lepidoptera;Brintesia circe;Brintesia circe;1;Brintesia circe;0.99999972858607;
GU669605;Lepidoptera;Polyommatus dorylas;Polyommatus dorylas;0.007;Polyommatus dorylas;0.987428194236461;
GU669606;Lepidoptera;Polyommatus dorylas;Polyommatus dorylas;0.007;Polyommatus dorylas;0.987428194236461;
GU669609;Lepidoptera;Nymphalis c-album;NA;0.215;NA;0.999999192918557;
GU669610;Lepidoptera;Nymphalis c-album;NA;0.215;NA;0.999999192918557;
GU669611;Lepidoptera;Plebejus argus;Plebejus argus;0.003;Plebejus argus;0.990564896508373;
GU669614;Lepidoptera;Pyrgus carthami;Pyrgus carthami;1;Pyrgus carthami;0.999993533091689;

GU669616;Lepidoptera;Nymphalis c-album;NA;0.215;NA;0.999999192918557;
GU669617;Lepidoptera;Polyommatus icarus;Polyommatus icarus;0.001;Polyommatus icarus;0.999851522482238;
GU669618;Lepidoptera;Plebejus argus;Plebejus argus;0.013;Plebejus argus;0.99029909759821;
GU669621;Lepidoptera;Plebejus argus;NA;NA;Plebejus argus;0.969283117748927;
GU669622;Lepidoptera;Polyommatus icarus;NA;0.004;Polyommatus icarus;0.999481683747521;
GU669623;Lepidoptera;Polyommatus dorylas;Polyommatus dorylas;0.007;Polyommatus dorylas;0.987428194236461;
GU669624;Lepidoptera;Nymphalis c-album;NA;0.215;NA;0.999999192918557;
GU669625;Lepidoptera;Polyommatus icarus;Polyommatus icarus;0.001;Polyommatus icarus;0.999851522482238;
GU669627;Lepidoptera;Euphydryas aurinia;NA;0.216;Euphydryas aurinia;0.994446100640531;
GU669628;Lepidoptera;Brintesia circe;Brintesia circe;1;Brintesia circe;0.999999209802354;
GU669629;Lepidoptera;Plebejus argus;NA;0.036;Plebejus argus;0.988160277018267;
GU669630;Lepidoptera;Boloria euphrosyne;Boloria euphrosyne;1;Boloria euphrosyne;0.999998093616766;
GU669631;Lepidoptera;Cyaniris semiargus;NA;NA;Cyaniris semiargus;0.999097907036339;
GU669633;Lepidoptera;Parnassius mnemosyne;Parnassius mnemosyne;1;Parnassius mnemosyne;0.999999336880698;
GU669634;Lepidoptera;Boloria euphrosyne;Boloria euphrosyne;0.002;Boloria euphrosyne;0.999959672696798;
GU669635;Lepidoptera;Boloria euphrosyne;Boloria euphrosyne;0.003;Boloria euphrosyne;0.999970094877776;
GU669637;Lepidoptera;Apatura ilia;NA;0.165;Apatura ilia;0.956796812031481;
GU669638;Lepidoptera;Pyrgus carthami;Pyrgus carthami;1;Pyrgus carthami;0.999981615717954;
GU669642;Lepidoptera;Satyrium w-album;NA;0.056;Satyrium w-album;0.989378230996316;
GU669643;Lepidoptera;Polyommatus daphnis;Polyommatus daphnis;0.001;Polyommatus daphnis;0.989092184788836;
GU669646;Lepidoptera;Cupido alcetas;Cupido alcetas;0.999;Cupido alcetas;0.995696397591104;
GU669647;Lepidoptera;Hipparchia semele;NA;0.001;Hipparchia semele;0.802446092830354;
GU669649;Lepidoptera;Erebia manto;NA;0.063;Erebia manto;0.998375472639544;
GU669650;Lepidoptera;Erebia manto;Erebia manto;NA;Erebia manto;0.999129075172383;
GU669651;Lepidoptera;Erebia euryale;NA;NA;Erebia euryale;0.919518760623792;
GU669652;Lepidoptera;Lasiommata maera;Lasiommata maera;1;Lasiommata maera;0.999999229574554;
GU669655;Lepidoptera;Lasiommata maera;Lasiommata maera;1;Lasiommata maera;0.999999181072746;
GU669656;Lepidoptera;Erebia euryale;NA;NA;Erebia euryale;0.919518760623792;
GU669659;Lepidoptera;Lasiommata maera;Lasiommata maera;1;Lasiommata maera;0.999999181072746;
GU669660;Lepidoptera;Callophrys rubi;NA;0.003;Callophrys rubi;0.997328639918919;
GU669662;Lepidoptera;Lasiommata maera;Lasiommata maera;1;Lasiommata maera;0.999999483871809;
GU669664;Lepidoptera;Erebia euryale;NA;NA;Erebia euryale;0.919518760623792;
GU669665;Lepidoptera;Pieris napi;NA;0.084;Pieris napi;0.885343420349066;
GU669667;Lepidoptera;Erebia epiphron;Erebia epiphron;NA;Erebia epiphron;0.998021437243156;
GU669668;Lepidoptera;Pieris napi;NA;0.028;Pieris napi;0.888205720664699;
GU669671;Lepidoptera;Pieris napi;NA;0.011;Pieris napi;0.801985394724814;
GU669672;Lepidoptera;Pieris napi;NA;0.011;Pieris napi;0.801985394724814;
GU669673;Lepidoptera;Pieris napi;NA;0.011;Pieris napi;0.801985394724814;
GU669679;Lepidoptera;Parnassius mnemosyne;Parnassius mnemosyne;1;Parnassius mnemosyne;0.999999269897625;
GU669680;Lepidoptera;Pararge aegeria;Pararge aegeria;1;Pararge aegeria;0.999999999963165;
GU669681;Lepidoptera;Pararge aegeria;Pararge aegeria;1;Pararge aegeria;0.999999999963165;
GU669683;Lepidoptera;Papilio machaon;Papilio machaon;NA;Papilio machaon;0.998259418780604;
GU669684;Lepidoptera;Pararge aegeria;Pararge aegeria;1;Pararge aegeria;0.999999999963165;
GU669685;Lepidoptera;Pararge aegeria;Pararge aegeria;1;Pararge aegeria;0.999999999963165;
GU669686;Lepidoptera;Ochlodes sylvanus;NA;0.316;Ochlodes sylvanus;0.996318754838625;
GU669687;Lepidoptera;Ochlodes sylvanus;NA;0.316;Ochlodes sylvanus;0.996318754838625;
GU669689;Lepidoptera;Papilio machaon;Papilio machaon;0.001;Papilio machaon;0.999463129150422;
GU669691;Lepidoptera;Ochlodes sylvanus;NA;0.316;Ochlodes sylvanus;0.996318754838625;
GU669692;Lepidoptera;Ochlodes sylvanus;NA;0.316;Ochlodes sylvanus;0.996318754838625;
GU669693;Lepidoptera;Ochlodes sylvanus;NA;0.316;Ochlodes sylvanus;0.996318754838625;
GU669698;Lepidoptera;Favonius quercus;NA;NA;Favonius quercus;0.73046069582187;
GU669699;Lepidoptera;Favonius quercus;NA;NA;Favonius quercus;0.73046069582187;
GU669702;Lepidoptera;Favonius quercus;NA;NA;Favonius quercus;0.73046069582187;
GU669703;Lepidoptera;Favonius quercus;NA;NA;Favonius quercus;0.73046069582187;
GU669704;Lepidoptera;Melitaea trivia;Melitaea trivia;1;Melitaea trivia;0.999999999977604;
GU669705;Lepidoptera;Melitaea trivia;Melitaea trivia;1;Melitaea trivia;0.999999999977604;
GU669706;Lepidoptera;Melitaea trivia;Melitaea trivia;1;Melitaea trivia;0.999999999977604;
GU669707;Lepidoptera;Melitaea nevadensis;Melitaea celadussa;1;Melitaea celadussa;0.999267524572691;
GU669712;Lepidoptera;Melitaea didyma;Melitaea didyma;1;Melitaea didyma;0.978209045677336;
GU669713;Lepidoptera;Melitaea didyma;Melitaea didyma;1;Melitaea didyma;0.978209045677336;
GU669714;Lepidoptera;Melitaea didyma;Melitaea didyma;NA;Melitaea didyma;0.980272836986251;
GU669715;Lepidoptera;Melitaea didyma;Melitaea didyma;1;Melitaea didyma;0.979092139543137;
GU669716;Lepidoptera;Melitaea diamina;NA;0.149;Melitaea diamina;0.999999996592464;
GU669717;Lepidoptera;Melitaea diamina;NA;0.066;Melitaea diamina;0.999999985294579;
GU669718;Lepidoptera;Melitaea diamina;NA;0.149;Melitaea diamina;0.999999996592464;
GU669719;Lepidoptera;Melitaea diamina;NA;0.149;Melitaea diamina;0.999999996592464;
GU669720;Lepidoptera;Melitaea cinxia;Melitaea cinxia;1;Melitaea cinxia;0.999999999999318;
GU669721;Lepidoptera;Melitaea cinxia;Melitaea cinxia;1;Melitaea cinxia;0.999999999999545;

GU669722;Lepidoptera;Melitaea cinxia;Melitaea cinxia;1;Melitaea cinxia;0.999999999999773;
GU669723;Lepidoptera;Melitaea cinxia;Melitaea cinxia;1;Melitaea cinxia;0.999999999999659;
GU669724;Lepidoptera;Polyommatus daphnis;Polyommatus daphnis;0.003;Polyommatus daphnis;0.99269461877028;
GU669725;Lepidoptera;Polyommatus daphnis;Polyommatus daphnis;0.001;Polyommatus daphnis;0.98724811249498;
GU669726;Lepidoptera;Melitaea nevadensis;Melitaea celadussa;1;Melitaea celadussa;0.999267524572691;
GU669727;Lepidoptera;Melitaea nevadensis;Melitaea celadussa;0.999;Melitaea celadussa;0.991309474236601;
GU669729;Lepidoptera;Polyommatus daphnis;Polyommatus daphnis;0.004;Polyommatus daphnis;0.98580375378789;
GU669730;Lepidoptera;Polyommatus daphnis;Polyommatus daphnis;0.001;Polyommatus daphnis;0.985613807163386;
GU669731;Lepidoptera;Maniola jurtina;NA;0.001;Maniola jurtina;0.939220105860875;
GU669732;Lepidoptera;Maniola jurtina;NA;NA;Maniola jurtina;0.953827931447544;
GU669733;Lepidoptera;Maniola jurtina;NA;NA;Maniola jurtina;0.953827931447544;
GU669734;Lepidoptera;Maniola jurtina;NA;NA;Maniola jurtina;0.953827931447544;
GU669739;Lepidoptera;Lysandra coridon;NA;NA;NA;0.999824213859773;
GU669740;Lepidoptera;Lysandra coridon;NA;NA;NA;0.999974416879064;
GU669741;Lepidoptera;Lysandra coridon;NA;NA;NA;0.99980755553816;
GU669742;Lepidoptera;Lysandra coridon;NA;NA;Lysandra coridon;0.719434738962092;
GU669746;Lepidoptera;Lysandra coridon;NA;NA;Lysandra coridon;0.719434738962092;
GU669748;Lepidoptera;Lycaena virgaureae;NA;0.077;Lycaena virgaureae;0.999717833371003;
GU669749;Lepidoptera;Lycaena virgaureae;NA;0.077;Lycaena virgaureae;0.999717833371003;
GU669751;Lepidoptera;Lycaena tityrus;Lycaena tityrus;1;Lycaena tityrus;0.999998453088497;
GU669752;Lepidoptera;Lycaena tityrus;Lycaena tityrus;1;Lycaena tityrus;0.999999121938815;
GU669753;Lepidoptera;Lycaena virgaureae;NA;0.063;Lycaena virgaureae;0.999609671520472;
GU669754;Lepidoptera;Lycaena virgaureae;NA;0.077;Lycaena virgaureae;0.999717833371003;
GU669755;Lepidoptera;Lycaena phlaeas;Lycaena phlaeas;1;Lycaena phlaeas;0.999999996363186;
GU669756;Lepidoptera;Lycaena tityrus;Lycaena tityrus;1;Lycaena tityrus;0.999998928238034;
GU669757;Lepidoptera;Lycaena tityrus;Lycaena tityrus;1;Lycaena tityrus;0.999998928238034;
GU669758;Lepidoptera;Lycaena tityrus;Lycaena tityrus;1;Lycaena tityrus;0.999998928238034;
GU669759;Lepidoptera;Lycaena phlaeas;Lycaena phlaeas;1;Lycaena phlaeas;0.999999995026627;
GU669760;Lepidoptera;Lycaena phlaeas;Lycaena phlaeas;1;Lycaena phlaeas;0.999999989808146;
GU669761;Lepidoptera;Lycaena phlaeas;Lycaena phlaeas;1;Lycaena phlaeas;0.999999996363186;
GU669762;Lepidoptera;Lycaena alciphron;Lycaena alciphron;1;Lycaena alciphron;0.99999978186528;
GU669763;Lepidoptera;Lycaena alciphron;Lycaena alciphron;1;Lycaena alciphron;0.999999794606957;
GU669766;Lepidoptera;Lycaena alciphron;Lycaena alciphron;1;Lycaena alciphron;0.999999852025592;
GU669767;Lepidoptera;Lycaena alciphron;Lycaena alciphron;1;Lycaena alciphron;0.999999716612377;
GU669768;Lepidoptera;Limenitis camilla;Limenitis camilla;0.009;Limenitis camilla;0.9999828626808;
GU669776;Lepidoptera;Lasiommata megera;Lasiommata megera;1;Lasiommata megera;0.999928528239676;
GU669777;Lepidoptera;Erebia pandrose;NA;0.002;Leptidea sinapis;0.938927273709494;
GU669778;Lepidoptera;Lasiommata maera;Lasiommata maera;1;Lasiommata maera;0.999997945450226;
GU669779;Lepidoptera;Lasiommata megera;Lasiommata megera;1;Lasiommata megera;0.999928528239676;
GU669780;Lepidoptera;Lasiommata megera;Lasiommata megera;1;Lasiommata megera;0.999944542420381;
GU669781;Lepidoptera;Lasiommata megera;Lasiommata megera;1;Lasiommata megera;0.999928528239676;
GU669782;Lepidoptera;Lampides boeticus;Lampides boeticus;1;Lampides boeticus;0.999999999545906;
GU669783;Lepidoptera;Lampides boeticus;Lampides boeticus;1;Lampides boeticus;0.999999999545906;
GU669784;Lepidoptera;Lasiommata maera;Lasiommata maera;1;Lasiommata maera;0.999997945450226;
GU669785;Lepidoptera;Lasiommata maera;Lasiommata maera;1;Lasiommata maera;0.999998751575999;
GU669788;Lepidoptera;Lampides boeticus;Lampides boeticus;1;Lampides boeticus;0.999999999293607;
GU669789;Lepidoptera;Lampides boeticus;Lampides boeticus;1;Lampides boeticus;0.999999999616279;
GU669792;Lepidoptera;Issoria lathonia;Issoria lathonia;1;Issoria lathonia;0.999999999989257;
GU669802;Lepidoptera;Hipparchia semele;NA;0.001;Hipparchia semele;0.859606265929303;
GU669803;Lepidoptera;Hipparchia semele;NA;0.002;Hipparchia semele;0.767063813608107;
GU669804;Lepidoptera;Hipparchia semele;NA;0.002;Hipparchia semele;0.767063813608107;
GU669805;Lepidoptera;Hipparchia semele;NA;0.002;Hipparchia semele;0.784603069619426;
GU669806;Lepidoptera;Hipparchia fagi;Hipparchia fagi;NA;Hipparchia fagi;0.986566456150817;
GU669807;Lepidoptera;Hipparchia fagi;Hipparchia fagi;NA;Hipparchia fagi;0.986566456150817;
GU669808;Lepidoptera;Hipparchia fagi;Hipparchia fagi;NA;Hipparchia fagi;0.986566456150817;
GU669809;Lepidoptera;Hipparchia fagi;Hipparchia fagi;NA;Hipparchia fagi;0.986566456150817;
GU669810;Lepidoptera;Gonepteryx rhamni;Gonepteryx rhamni;1;Gonepteryx rhamni;0.99964616790002;
GU669811;Lepidoptera;Gonepteryx rhamni;Gonepteryx rhamni;1;Gonepteryx rhamni;0.99964616790002;
GU669812;Lepidoptera;Hamearis lucina;NA;NA;Hamearis lucina;0.999999999984141;
GU669813;Lepidoptera;Hamearis lucina;NA;NA;Hamearis lucina;0.999999999984141;
GU669814;Lepidoptera;Glaucopsyche alexis;Glaucopsyche alexis;0.001;Glaucopsyche alexis;0.994667256195263;
GU669815;Lepidoptera;Glaucopsyche alexis;Glaucopsyche alexis;0.001;Glaucopsyche alexis;0.994667256195263;
GU669816;Lepidoptera;Gonepteryx rhamni;NA;0.474;Gonepteryx rhamni;0.982076396127858;
GU669817;Lepidoptera;Gonepteryx rhamni;NA;0.474;Gonepteryx rhamni;0.982076396127858;
GU669818;Lepidoptera;Cupido argiades;NA;0.471;NA;0.999999999949778;
GU669819;Lepidoptera;Cupido argiades;NA;0.499;NA;0.999999999984682;
GU669820;Lepidoptera;Glaucopsyche alexis;Glaucopsyche alexis;0.001;Glaucopsyche alexis;0.994667256195263;

GU669821;Lepidoptera;Glaucopsyche alexis;Glaucopsyche alexis;0.001;Glaucopsyche alexis;0.992129691008676;
GU669822;Lepidoptera;Cupido alcetas;NA;0.068;Cupido alcetas;0.88988907876118;
GU669823;Lepidoptera;Cupido argiades;NA;0.499;NA;0.999999999984682;
GU669824;Lepidoptera;Cupido argiades;NA;0.499;NA;0.999999999984682;
GU669825;Lepidoptera;Cupido argiades;NA;0.499;NA;0.999999999984682;
GU669826;Lepidoptera;Cupido alcetas;NA;0.068;Cupido alcetas;0.88988907876118;
GU669827;Lepidoptera;Cupido alcetas;NA;0.068;Cupido alcetas;0.88988907876118;
GU669828;Lepidoptera;Cupido alcetas;NA;0.068;Cupido alcetas;0.88988907876118;
GU669829;Lepidoptera;Cupido alcetas;NA;0.068;Cupido alcetas;0.88988907876118;
GU669830;Lepidoptera;Euphydryas aurinia;Euphydryas aurinia;0.001;Euphydryas aurinia;0.998892818330584;
GU669831;Lepidoptera;Euphydryas aurinia;Euphydryas aurinia;0.02;Euphydryas aurinia;0.99668249939786;
GU669832;Lepidoptera;Euphydryas aurinia;Euphydryas aurinia;0.02;Euphydryas aurinia;0.99668249939786;
GU669833;Lepidoptera;Euphydryas aurinia;Euphydryas aurinia;0.02;Euphydryas aurinia;0.99668249939786;
GU669834;Lepidoptera;Erynnis tages;Erynnis tages;0.999;Erynnis tages;0.999956830266092;
GU669835;Lepidoptera;Erynnis tages;Erynnis tages;1;Erynnis tages;0.999999160434197;
GU669837;Lepidoptera;Plebejus idas;NA;0.007;NA;0.999546324331549;
GU669838;Lepidoptera;Erebia manto;Erebia manto;0.009;Erebia manto;0.999861807193394;
GU669840;Lepidoptera;Erebia manto;Erebia manto;0.009;Erebia manto;0.999861807193394;
GU669841;Lepidoptera;Erebia manto;Erebia manto;0.009;Erebia manto;0.999861807193394;
GU669842;Lepidoptera;Erebia manto;Erebia manto;NA;Erebia manto;0.999930125474235;
GU669843;Lepidoptera;Erebia manto;Erebia manto;NA;Erebia manto;0.999926924199631;
GU669844;Lepidoptera;Erebia euryale;NA;NA;Erebia euryale;0.93493911427573;
GU669845;Lepidoptera;Erebia euryale;NA;NA;Erebia euryale;0.93493911427573;
GU669846;Lepidoptera;Erebia euryale;NA;NA;Erebia euryale;0.965689685361429;
GU669848;Lepidoptera;Erebia epiphron;NA;NA;Erebia epiphron;0.950006471153786;
GU669849;Lepidoptera;Erebia epiphron;NA;NA;Erebia epiphron;0.950006471153786;
GU669850;Lepidoptera;Erebia euryale;NA;NA;Erebia euryale;0.93493911427573;
GU669851;Lepidoptera;Erebia euryale;NA;NA;Erebia euryale;0.93493911427573;
GU669852;Lepidoptera;Vanessa cardui;Vanessa cardui;1;Vanessa cardui;0.999999459983318;
GU669853;Lepidoptera;Erebia epiphron;NA;NA;Erebia epiphron;0.950006471153786;
GU669854;Lepidoptera;Erebia epiphron;NA;NA;Erebia epiphron;0.930026054867432;
GU669855;Lepidoptera;Erebia epiphron;NA;NA;Erebia epiphron;0.950006471153786;
GU669856;Lepidoptera;Cyaniris semiargus;NA;NA;Cyaniris semiargus;0.999097907036339;
GU669857;Lepidoptera;Vanessa cardui;Vanessa cardui;1;Vanessa cardui;0.999999803450484;
GU669858;Lepidoptera;Vanessa cardui;Vanessa cardui;1;Vanessa cardui;0.999999459983318;
GU669859;Lepidoptera;Vanessa cardui;Vanessa cardui;1;Vanessa cardui;0.999999459983318;
GU669860;Lepidoptera;Cupido osiris;Cupido osiris;1;Cupido osiris;0.999999944757265;
GU669861;Lepidoptera;Cyaniris semiargus;NA;NA;Cyaniris semiargus;0.999051841766953;
GU669862;Lepidoptera;Cyaniris semiargus;NA;NA;Cyaniris semiargus;0.999097907036339;
GU669863;Lepidoptera;Cyaniris semiargus;NA;NA;Cyaniris semiargus;0.999097907036339;
GU669864;Lepidoptera;Cupido minimus;NA;0.001;Cupido minimus;0.99992423685743;
GU675630;Lepidoptera;Thecla betulae;Thecla betulae;1;Thecla betulae;0.999999999972886;
GU675633;Lepidoptera;Plebejus argyrognomon;NA;0.001;NA;0.994620093546684;
GU675636;Lepidoptera;Polyommatus amandus;Polyommatus amandus;0.003;Polyommatus amandus;0.999983287111715;
GU675637;Lepidoptera;Polyommatus amandus;Polyommatus amandus;0.003;Polyommatus amandus;0.999980071833264;
GU675638;Lepidoptera;Polyommatus amandus;Polyommatus amandus;0.003;Polyommatus amandus;0.999980071833264;
GU675641;Lepidoptera;Pyrgus sidae;Pyrgus sidae;1;Pyrgus sidae;0.99996750457282;
GU675642;Lepidoptera;Pyrgus sidae;Pyrgus sidae;1;Pyrgus sidae;0.99999311394709;
GU675648;Lepidoptera;Pyrgus sidae;Pyrgus sidae;1;Pyrgus sidae;0.999981962201329;
GU675661;Lepidoptera;Erebia pandrose;Erebia pandrose;0.998;Erebia pandrose;0.99797994145198;
GU675662;Lepidoptera;Erebia pandrose;Erebia pandrose;0.998;Erebia pandrose;0.998086832694793;
GU675664;Lepidoptera;Aglais urticae;NA;0.001;Aglais urticae;0.990689645759264;
GU675665;Lepidoptera;Aporia crataegi;NA;NA;Aporia crataegi;0.999996781126733;
GU675670;Lepidoptera;Pyrgus foulquieri;Pyrgus foulquieri;0.005;Pyrgus foulquieri;0.741885925757585;
GU675674;Lepidoptera;Pyrgus armoricanus;Pyrgus armoricanus;1;Pyrgus armoricanus;0.999997353848817;
GU675675;Lepidoptera;Pyrgus armoricanus;Pyrgus armoricanus;1;Pyrgus armoricanus;0.999997353848817;
GU675677;Lepidoptera;Colias crocea;NA;0.059;NA;0.999999999999738;
GU675678;Lepidoptera;Polyommatus dorylas;Polyommatus dorylas;0.007;Polyommatus dorylas;0.987428194236461;
GU675682;Lepidoptera;Satyrium spini;Satyrium spini;1;Satyrium spini;0.999998222517791;
GU675683;Lepidoptera;Polyommatus eros;Polyommatus eros;0.001;Polyommatus eros;0.990287248915798;
GU675684;Lepidoptera;Colias alfacariensis;Colias alfacariensis;1;Colias alfacariensis;0.999620674460243;
GU675685;Lepidoptera;Argynnis paphia;Argynnis paphia;1;Argynnis paphia;0.999999992970402;
GU675687;Lepidoptera;Satyrium spini;Satyrium spini;1;Satyrium spini;0.999996354401924;
GU675689;Lepidoptera;Lycaena tityrus;Lycaena tityrus;1;Lycaena tityrus;0.999997855580031;
GU675690;Lepidoptera;Lycaena tityrus;Lycaena tityrus;1;Lycaena tityrus;0.999995271914181;

GU675691;Lepidoptera;Vanessa atalanta;Vanessa atalanta;1;Vanessa atalanta;0.999999999757762;
GU675694;Lepidoptera;Melitaea trivia;Melitaea trivia;1;Melitaea trivia;0.99999999997172;
GU675695;Lepidoptera;Maniola jurtina;NA;0.012;Maniola jurtina;0.91755716175482;
GU675696;Lepidoptera;Nymphalis c-album;NA;0.215;NA;0.999999192918557;
GU675699;Lepidoptera;Pieris napi;NA;0.012;Pieris napi;0.918936415764965;
GU675700;Lepidoptera;Plebejus idas;NA;0.007;NA;0.999546324331549;
GU675701;Lepidoptera;Pieris napi;NA;0.011;Pieris napi;0.801985394724814;
GU675702;Lepidoptera;Lasiommata maera;Lasiommata maera;1;Lasiommata maera;0.999997945450226;
GU675703;Lepidoptera;Pieris napi;NA;0.028;Pieris napi;0.888205720664699;
GU675704;Lepidoptera;Melitaea deione;Melitaea deione;1;Melitaea deione;0.999999999676987;
GU675705;Lepidoptera;Fabriciana adippe;Fabriciana adippe;0.001;Fabriciana adippe;0.999538788643547;
GU675706;Lepidoptera;Melitaea parthenoides;Melitaea parthenoides;1;Melitaea parthenoides;0.999999980399821;
GU675707;Lepidoptera;Coenonympha arcania;Coenonympha arcania;0.019;Coenonympha arcania;0.986942135735679;
GU675709;Lepidoptera;Coenonympha pamphilus;Coenonympha pamphilus;1;Coenonympha pamphilus;0.999999999999289;
GU675710;Lepidoptera;Aglais urticae;NA;0.002;Aglais urticae;0.987497593219019;
GU675711;Lepidoptera;Erebia meolans;Erebia meolans;NA;Erebia meolans;0.999835953426128;
GU675713;Lepidoptera;Erebia meolans;Erebia meolans;NA;Erebia meolans;0.999835953426128;
GU675714;Lepidoptera;Erebia meolans;Erebia meolans;NA;Erebia meolans;0.999835953426128;
GU675715;Lepidoptera;Erebia euryale;NA;NA;Erebia euryale;0.926347729741663;
GU675721;Lepidoptera;Satyrus actaea;NA;0.9;Satyrus actaea;0.998537709015806;
GU675722;Lepidoptera;Chazara briseis;Chazara briseis;1;Chazara briseis;0.999980393060755;
GU675723;Lepidoptera;Hipparchia semele;NA;0.001;Hipparchia semele;0.852517984111014;
GU675724;Lepidoptera;Satyrus ferula;Satyrus ferula;0.001;Satyrus ferula;0.994329228962786;
GU675725;Lepidoptera;Hipparchia fidia;Hipparchia fidia;1;Hipparchia fidia;1;
GU675726;Lepidoptera;Hipparchia hermione;Hipparchia hermione;1;Hipparchia hermione;0.99958099561668;
GU675727;Lepidoptera;Hipparchia fidia;Hipparchia fidia;1;Hipparchia fidia;1;
GU675728;Lepidoptera;Arethusana arethusa;Arethusana arethusa;0.003;Arethusana arethusa;0.999976174545094;
GU675729;Lepidoptera;Hipparchia statilinus;Hipparchia statilinus;1;Hipparchia statilinus;0.999999999999801;
GU675735;Lepidoptera;Arethusana arethusa;NA;0.062;Arethusana arethusa;0.999962489703143;
GU675737;Lepidoptera;Pyrgus serratulae;Pyrgus serratulae;1;Pyrgus serratulae;0.999999877361184;
GU675738;Lepidoptera;Pyrgus carthami;Pyrgus carthami;1;Pyrgus carthami;0.999997085657879;
GU675740;Lepidoptera;Pyrgus alveus;NA;NA;Pyrgus alveus;0.931326683565012;
GU675742;Lepidoptera;Cupido argiades;NA;0.499;NA;0.999999999984682;
GU675743;Lepidoptera;Cupido argiades;NA;0.499;NA;0.999999999984682;
GU675744;Lepidoptera;Cupido argiades;NA;0.499;NA;0.999999999984682;
GU675745;Lepidoptera;Celastrina argiolus;Celastrina argiolus;1;Celastrina argiolus;0.999527385150531;
GU675747;Lepidoptera;Celastrina argiolus;Celastrina argiolus;1;Celastrina argiolus;0.999527385150531;
GU675748;Lepidoptera;Lampides boeticus;Lampides boeticus;1;Lampides boeticus;0.999999999411585;
GU675755;Lepidoptera;Polyommatus icarus;NA;0.004;Polyommatus icarus;0.999481683747521;
GU675755;Lepidoptera;Polyommatus icarus;NA;0.004;Polyommatus icarus;0.999481683747521;
GU675758;Lepidoptera;Lysandra coridon;NA;NA;NA;0.999976559473762;
GU675759;Lepidoptera;Lysandra coridon;NA;NA;NA;0.999969135579956;
GU675761;Lepidoptera;Polyommatus daphnis;NA;0.031;Polyommatus daphnis;0.989661132749926;
GU675762;Lepidoptera;Polyommatus dorylas;Polyommatus dorylas;0.025;Polyommatus dorylas;0.973229109265438;
GU675763;Lepidoptera;Polyommatus dorylas;Polyommatus dorylas;0.025;Polyommatus dorylas;0.973229109265438;
GU675764;Lepidoptera;Argynnis aglaja;NA;NA;NA;0.999999999998776;
GU675769;Lepidoptera;Euphydryas aurinia;Euphydryas aurinia;0.005;Euphydryas aurinia;0.998570950148478;
GU675770;Lepidoptera;Melitaea deione;Melitaea deione;1;Melitaea deione;0.999999998710706;
GU675771;Lepidoptera;Vanessa atalanta;Vanessa atalanta;1;Vanessa atalanta;0.999999999297358;
GU675774;Lepidoptera;Nymphalis c-album;NA;0.215;NA;0.999999192918557;
GU675775;Lepidoptera;Charaxes jasius;Charaxes jasius;NA;Charaxes jasius;0.988941612024336;
GU675776;Lepidoptera;Aglais urticae;NA;0.145;Aglais urticae;0.986365595304464;
GU675777;Lepidoptera;Charaxes jasius;Charaxes jasius;NA;Charaxes jasius;0.988941612024336;
GU675778;Lepidoptera;Charaxes jasius;Charaxes jasius;NA;Charaxes jasius;0.988941612024336;
GU675779;Lepidoptera;Satyrium esculi;Satyrium esculi;1;Satyrium esculi;0.999999957367948;
GU675780;Lepidoptera;Satyrium ilicis;Satyrium ilicis;1;Satyrium ilicis;0.999999987901305;
GU675781;Lepidoptera;Callophrys rubi;NA;0.003;Callophrys rubi;0.996429498210922;
GU675782;Lepidoptera;Callophrys rubi;NA;0.001;Callophrys rubi;0.99870518510598;
GU675783;Lepidoptera;Glaucopsyche alexis;NA;NA;Glaucopsyche alexis;0.980579683000946;
GU675784;Lepidoptera;Glaucopsyche alexis;NA;NA;Glaucopsyche alexis;0.97933749849394;
GU675785;Lepidoptera;Glaucopsyche melanops;Glaucopsyche melanops;1;Glaucopsyche melanops;0.999952924254607;
GU675786;Lepidoptera;Glaucopsyche melanops;Glaucopsyche melanops;1;Glaucopsyche melanops;0.999952924254607;
GU675789;Lepidoptera;Celastrina argiolus;Celastrina argiolus;1;Celastrina argiolus;0.999527385150531;

GU675790;Lepidoptera;Celastrina argiolus;Celastrina argiolus;1;Celastrina argiolus;0.999527385150531;
GU675792;Lepidoptera;Polyommatus icarus;Polyommatus icarus;0.001;Polyommatus icarus;0.999503294805987;
GU675792;Lepidoptera;Polyommatus icarus;Polyommatus icarus;0.001;Polyommatus icarus;0.999503294805987;
GU675793;Lepidoptera;Polyommatus eros;NA;0.003;Polyommatus eros;0.985402505884526;
GU675794;Lepidoptera;Polyommatus eros;NA;0.003;Polyommatus eros;0.985402505884526;
GU675795;Lepidoptera;Polyommatus damon;Polyommatus damon;1;Polyommatus damon;0.999999961804208;
GU675796;Lepidoptera;Polyommatus dorylas;Polyommatus dorylas;0.007;Polyommatus dorylas;0.975640696264455;
GU675797;Lepidoptera;Cupido minimus;NA;0.009;Cupido minimus;0.999791453712643;
GU675800;Lepidoptera;Cupido argiades;NA;0.499;NA;0.999999999984682;
GU675801;Lepidoptera;Gonepteryx rhamni;Gonepteryx rhamni;1;Gonepteryx rhamni;0.999850170258883;
GU675802;Lepidoptera;Lycaena tityrus;Lycaena tityrus;1;Lycaena tityrus;0.999997855580031;
GU675807;Lepidoptera;Limenitis camilla;Limenitis camilla;0.009;Limenitis camilla;0.9999828626808;
GU675809;Lepidoptera;Lasiommata megera;Lasiommata megera;1;Lasiommata megera;0.999943327299082;
GU675810;Lepidoptera;Lasiommata megera;Lasiommata megera;1;Lasiommata megera;0.999928528239676;
GU675811;Lepidoptera;Pararge aegeria;Pararge aegeria;1;Pararge aegeria;0.999999999963165;
GU675812;Lepidoptera;Lasiommata maera;Lasiommata maera;1;Lasiommata maera;0.999997945450226;
GU675814;Lepidoptera;Pararge aegeria;Pararge aegeria;1;Pararge aegeria;0.999999999963165;
GU675815;Lepidoptera;Erebia epiphron;NA;NA;Erebia epiphron;0.950006471153786;
GU675818;Lepidoptera;Erebia epiphron;NA;NA;Erebia epiphron;0.950006471153786;
GU675822;Lepidoptera;Erebia euryale;NA;NA;Erebia euryale;0.93493911427573;
GU675835;Lepidoptera;Anthocharis cardamines;Anthocharis cardamines;1;Anthocharis cardamines;0.999999940675226;
GU675837;Lepidoptera;Anthocharis euphenoides;NA;NA;Anthocharis euphenoides;0.960146267594346;
GU675838;Lepidoptera;Anthocharis cardamines;Anthocharis cardamines;1;Anthocharis cardamines;0.999999940675226;
GU675839;Lepidoptera;Pieris napi;NA;NA;Pieris napi;0.860244891707633;
GU675842;Lepidoptera;Pieris napi;NA;NA;Pieris napi;0.93839557695923;
GU675845;Lepidoptera;Coenonympha pamphilus;Coenonympha pamphilus;1;Coenonympha pamphilus;0.999999999999915;
GU675847;Lepidoptera;Coenonympha arcania;NA;0.172;Coenonympha arcania;0.963036309473038;
GU675848;Lepidoptera;Coenonympha pamphilus;Coenonympha pamphilus;1;Coenonympha pamphilus;0.999999999999915;
GU675849;Lepidoptera;Colias phicomone;Colias phicomone;0.001;Colias phicomone;0.986802434025645;
GU675850;Lepidoptera;Colias phicomone;Colias phicomone;0.001;Colias phicomone;0.986802434025645;
GU675851;Lepidoptera;Colias crocea;NA;0.059;NA;0.999999999999738;
GU675852;Lepidoptera;Colias crocea;NA;0.059;NA;0.999999999999738;
GU675853;Lepidoptera;Aporia crataegi;NA;NA;Aporia crataegi;0.999996781126733;
GU675854;Lepidoptera;Maniola tithonus;Pyronia tithonus;1;Pyronia tithonus;1;
GU675856;Lepidoptera;Papilio machaon;Papilio machaon;0.007;Papilio machaon;0.998348042131142;
GU675858;Lepidoptera;Maniola jurtina;NA;0.001;Maniola jurtina;0.939220105860875;
GU675860;Lepidoptera;Lycaena phlaeas;Lycaena phlaeas;1;Lycaena phlaeas;0.999999996363186;
GU675861;Lepidoptera;Thymelicus acteon;Thymelicus acteon;1;Thymelicus acteon;0.99999999752248;
GU675862;Lepidoptera;Coenonympha glycerion;NA;0.156;Coenonympha glycerion;0.999998559347134;
GU675863;Lepidoptera;Pieris napi;NA;NA;Pieris napi;0.928562853666922;
GU675864;Lepidoptera;Ochlodes sylvanus;NA;0.316;Ochlodes sylvanus;0.996318754838625;
GU675865;Lepidoptera;Brintesia circe;Brintesia circe;1;Brintesia circe;0.999999898802064;
GU675867;Lepidoptera;Lampides boeticus;Lampides boeticus;1;Lampides boeticus;0.999999999545906;
GU675868;Lepidoptera;Pararge aegeria;Pararge aegeria;1;Pararge aegeria;0.999999999963165;
GU675869;Lepidoptera;Lasiommata maera;Lasiommata maera;1;Lasiommata maera;0.999997945450226;
GU675870;Lepidoptera;Carcharodus alceae;Carcharodus alceae;0.999;Carcharodus alceae;0.999999996339511;
GU675872;Lepidoptera;Papilio machaon;Papilio machaon;0.003;Papilio machaon;0.997288237409893;
GU675873;Lepidoptera;Cupido alcetas;NA;0.218;Cupido alcetas;0.833501983138177;
GU675875;Lepidoptera;Satyrium esculi;Satyrium esculi;1;Satyrium esculi;0.999999957367948;
GU675877;Lepidoptera;Callophrys rubi;NA;0.001;Callophrys rubi;0.99870518510598;
GU675878;Lepidoptera;Carcharodus alceae;Carcharodus alceae;0.999;Carcharodus alceae;0.999999996339511;
GU675881;Lepidoptera;Melitaea didyma;Melitaea didyma;1;Melitaea didyma;0.990041801198236;
GU675882;Lepidoptera;Pyrgus carthami;Pyrgus carthami;1;Pyrgus carthami;0.999998862764095;
GU675885;Lepidoptera;Maniola jurtina;NA;NA;Maniola jurtina;0.962772432769033;
GU675887;Lepidoptera;Hipparchia semele;NA;0.001;Hipparchia semele;0.812142709977226;
GU675888;Lepidoptera;Pyrgus carthami;Pyrgus carthami;1;Pyrgus carthami;0.999998623758851;
GU675890;Lepidoptera;Gonepteryx cleopatra;Gonepteryx cleopatra;0.998;Gonepteryx cleopatra;0.999118636582266;
GU675891;Lepidoptera;Polyommatus icarus;Polyommatus icarus;0.001;Polyommatus icarus;0.999851522482238;
GU675891;Lepidoptera;Polyommatus icarus;Polyommatus icarus;0.001;Polyommatus icarus;0.999851522482238;
GU675893;Lepidoptera;Pieris napi;NA;0.028;Pieris napi;0.888205720664699;
GU675896;Lepidoptera;Lysandra hispana;NA;NA;NA;0.999964115886992;
GU675907;Lepidoptera;Pyrgus armoricanus;Pyrgus armoricanus;1;Pyrgus armoricanus;0.999997353848817;
GU675908;Lepidoptera;Melitaea deione;Melitaea deione;1;Melitaea deione;0.999999999611504;

GU675909;Lepidoptera;Colias alfacariensis;Colias alfacariensis;0.001;Colias alfacariensis;0.999857790810452;
GU675912;Lepidoptera;Colias alfacariensis;Colias alfacariensis;0.001;Colias alfacariensis;0.999857790810452;
GU675914;Lepidoptera;Coenonympha pamphilus;Coenonympha pamphilus;1;Coenonympha pamphilus;0.999999999999886;
GU675925;Lepidoptera;Coenonympha glycerion;NA;0.083;Coenonympha glycerion;0.999999074492111;
GU675926;Lepidoptera;Maniola cecilia;Pyronia cecilia;1;Pyronia cecilia;1;
GU675927;Lepidoptera;Plebejus argus;Plebejus argus;0.014;Plebejus argus;0.979849220658559;
GU675928;Lepidoptera;Colias alfacariensis;Colias alfacariensis;0.001;Colias alfacariensis;0.999857790810452;
GU675929;Lepidoptera;Hipparchia hermione;Hipparchia hermione;1;Hipparchia hermione;0.999905429250471;
GU675930;Lepidoptera;Melitaea nevadensis;Melitaea celadussa;0.999;Melitaea celadussa;0.998819206090668;
GU675931;Lepidoptera;Lycaena virgaureae;NA;0.184;Lycaena virgaureae;0.999653824584039;
GU675935;Lepidoptera;Lycaena tityrus;Lycaena tityrus;1;Lycaena tityrus;0.999997855580031;
GU675936;Lepidoptera;Lysandra coridon;NA;NA;NA;0.999969135579956;
GU675937;Lepidoptera;Erebia euryale;NA;NA;Erebia euryale;0.971435689232499;
GU675942;Lepidoptera;Cupido alcetas;NA;0.218;Cupido alcetas;0.833501983138177;
GU675944;Lepidoptera;Favonius quercus;NA;NA;Favonius quercus;0.73046069582187;
GU675946;Lepidoptera;Hipparchia fagi;Hipparchia fagi;NA;Hipparchia fagi;0.973330290902851;
GU675947;Lepidoptera;Euphydryas aurinia;Euphydryas aurinia;0.014;Euphydryas aurinia;0.996180211940517;
GU675949;Lepidoptera;Coenonympha arcania;NA;0.06;Coenonympha arcania;0.960519110135242;
GU675950;Lepidoptera;Gonepteryx cleopatra;Gonepteryx cleopatra;0.001;Gonepteryx cleopatra;0.998992887085358;
GU675953;Lepidoptera;Brenthis ino;Brenthis ino;0.001;Brenthis ino;0.997741439264217;
GU675954;Lepidoptera;Charaxes jasius;Charaxes jasius;NA;Charaxes jasius;0.988941612024336;
GU675957;Lepidoptera;Colias crocea;NA;0.059;NA;0.999999999999738;
GU675958;Lepidoptera;Anthocharis euphenoides;NA;0.057;Anthocharis euphenoides;0.951171242535209;
GU675959;Lepidoptera;Colias phicomone;Colias phicomone;0.001;Colias phicomone;0.986802434025645;
GU675960;Lepidoptera;Colias crocea;NA;NA;NA;0.999999999999568;
GU675961;Lepidoptera;Celastrina argiolus;Celastrina argiolus;1;Celastrina argiolus;0.999527385150531;
GU675962;Lepidoptera;Colias alfacariensis;Colias alfacariensis;0.001;Colias alfacariensis;0.999857790810452;
GU675963;Lepidoptera;Lampides boeticus;Lampides boeticus;1;Lampides boeticus;0.999999999545906;
GU675964;Lepidoptera;Celastrina argiolus;Celastrina argiolus;1;Celastrina argiolus;0.999527385150531;
GU675965;Lepidoptera;Satyrium esculi;Satyrium esculi;1;Satyrium esculi;0.999999957367948;
GU675966;Lepidoptera;Satyrium ilicis;Satyrium ilicis;1;Satyrium ilicis;0.999999987901305;
GU675968;Lepidoptera;Satyrium spini;Satyrium spini;1;Satyrium spini;0.999998173309697;
GU675971;Lepidoptera;Polyommatus damon;Polyommatus damon;1;Polyommatus damon;0.999999959192594;
GU675975;Lepidoptera;Lysandra coridon;NA;NA;NA;0.999969135579956;
GU675976;Lepidoptera;Lysandra coridon;NA;NA;NA;0.999969135579956;
GU675977;Lepidoptera;Plebejus idas;NA;0.007;NA;0.999546324331549;
GU675979;Lepidoptera;Polyommatus celina;Polyommatus celina;0.001;Polyommatus celina;0.999999999717033;
GU675980;Lepidoptera;Polyommatus escheri;Polyommatus escheri;1;Polyommatus escheri;0.999999995351544;
GU675981;Lepidoptera;Lycaena phlaeas;Lycaena phlaeas;1;Lycaena phlaeas;0.999999996363186;
GU675982;Lepidoptera;Polyommatus celina;Polyommatus celina;0.001;Polyommatus celina;0.999999999717033;
GU675983;Lepidoptera;Lycaena phlaeas;Lycaena phlaeas;1;Lycaena phlaeas;0.999999996363186;
GU675984;Lepidoptera;Lycaena phlaeas;Lycaena phlaeas;1;Lycaena phlaeas;0.999999996363186;
GU675986;Lepidoptera;Lycaena virgaureae;NA;0.184;Lycaena virgaureae;0.999653824584039;
GU675993;Lepidoptera;Gonepteryx cleopatra;Gonepteryx cleopatra;0.998;Gonepteryx cleopatra;0.999118636582266;
GU675994;Lepidoptera;Gonepteryx cleopatra;Gonepteryx cleopatra;0.998;Gonepteryx cleopatra;0.999118636582266;
GU676001;Lepidoptera;Euphydryas aurinia;NA;0.001;Euphydryas aurinia;0.986569933454;
GU676003;Lepidoptera;Euphydryas aurinia;NA;0.001;Euphydryas aurinia;0.984263569279661;
GU676004;Lepidoptera;Euphydryas aurinia;NA;0.001;Euphydryas aurinia;0.984263569279661;
GU676005;Lepidoptera;Lasiommata megera;Lasiommata megera;0.995;Lasiommata megera;0.999279716854958;
GU676006;Lepidoptera;Melitaea nevadensis;Melitaea celadussa;1;Melitaea celadussa;0.999267524572691;
GU676007;Lepidoptera;Lasiommata megera;Lasiommata megera;1;Lasiommata megera;0.999928528239676;
GU676008;Lepidoptera;Lasiommata megera;Lasiommata megera;1;Lasiommata megera;0.999928528239676;
GU676009;Lepidoptera;Pararge aegeria;Pararge aegeria;1;Pararge aegeria;0.999999999365514;
GU676010;Lepidoptera;Pararge aegeria;Pararge aegeria;1;Pararge aegeria;0.999999999365514;
GU676011;Lepidoptera;Erebia neoridas;NA;NA;Erebia neoridas;0.994368810769763;
GU676013;Lepidoptera;Erebia euryale;NA;NA;Erebia euryale;0.93493911427573;
GU676014;Lepidoptera;Erebia neoridas;NA;NA;Erebia neoridas;0.994368810769763;
GU676015;Lepidoptera;Erebia oeme;NA;0.114;Erebia oeme;0.999999999830635;
GU676016;Lepidoptera;Erebia meolans;Erebia meolans;NA;Erebia meolans;0.999835953426128;
GU676018;Lepidoptera;Erebia oeme;NA;0.114;Erebia oeme;0.999999999830635;

GU676021;Lepidoptera;Satyrus actaea;Satyrus actaea;0.002;Satyrus actaea;0.998246235973604;
GU676022;Lepidoptera;Satyrus ferula;Satyrus ferula;0.001;Satyrus ferula;0.995920305204187;
GU676023;Lepidoptera;Aphantopus hyperantus;Aphantopus hyperantus;0.006;Aphantopus hyperantus;0.999998615504572;
GU676024;Lepidoptera;Maniola jurtina;NA;0.168;Maniola jurtina;0.934856736346661;
GU676025;Lepidoptera;Maniola cecilia;Pyronia cecilia;1;Pyronia cecilia;1;
GU676026;Lepidoptera;Maniola cecilia;Pyronia cecilia;1;Pyronia cecilia;1;
GU676027;Lepidoptera;Maniola cecilia;Pyronia cecilia;1;Pyronia cecilia;0.999999999999972;
GU676028;Lepidoptera;Maniola cecilia;Pyronia cecilia;1;Pyronia cecilia;1;
GU676029;Lepidoptera;Coenonympha arcania;Coenonympha arcania;0.022;Coenonympha arcania;0.98373278454791;
GU676030;Lepidoptera;Maniola tithonus;Pyronia tithonus;1;Pyronia tithonus;1;
GU676031;Lepidoptera;Coenonympha pamphilus;Coenonympha pamphilus;1;Coenonympha pamphilus;0.999999999995282;
GU676032;Lepidoptera;Coenonympha dorus;Coenonympha dorus;1;Coenonympha dorus;0.99999999988259;
GU676033;Lepidoptera;Coenonympha pamphilus;Coenonympha pamphilus;1;Coenonympha pamphilus;0.999999999995282;
GU676034;Lepidoptera;Coenonympha pamphilus;Coenonympha pamphilus;1;Coenonympha pamphilus;0.999999999995282;
GU676035;Lepidoptera;Hipparchia fidia;Hipparchia fidia;1;Hipparchia fidia;0.999999999999972;
GU676036;Lepidoptera;Hipparchia fidia;Hipparchia fidia;1;Hipparchia fidia;1;
GU676038;Lepidoptera;Hipparchia fidia;Hipparchia fidia;1;Hipparchia fidia;1;
GU676041;Lepidoptera;Plebejus idas;NA;0.009;NA;0.99929488087288;
GU676042;Lepidoptera;Celastrina argiolus;Celastrina argiolus;1;Celastrina argiolus;0.999537223294636;
GU676047;Lepidoptera;Pyrgus alveus;NA;NA;Pyrgus alveus;0.931326683565012;
GU676048;Lepidoptera;Pyrgus armoricanus;Pyrgus armoricanus;1;Pyrgus armoricanus;0.997478680096412;
GU676049;Lepidoptera;Pyrgus armoricanus;Pyrgus armoricanus;1;Pyrgus armoricanus;0.999997353848817;
GU676050;Lepidoptera;Pyrgus sidae;Pyrgus sidae;1;Pyrgus sidae;0.99996750457282;
GU676051;Lepidoptera;Pyrgus armoricanus;Pyrgus armoricanus;1;Pyrgus armoricanus;0.997478680096412;
GU676053;Lepidoptera;Pyrgus sidae;Pyrgus sidae;1;Pyrgus sidae;0.999973057661669;
GU676060;Lepidoptera;Gonepteryx cleopatra;Gonepteryx cleopatra;1;Gonepteryx cleopatra;0.999518782695122;
GU676065;Lepidoptera;Charaxes jasius;Charaxes jasius;0.001;Charaxes jasius;0.997689374666591;
GU676066;Lepidoptera;Iphiclides podalirius;NA;0.302;Iphiclides podalirius;0.931082412606632;
GU676070;Lepidoptera;Polyommatus eros;NA;0.001;Polyommatus eros;0.972333425517021;
GU676078;Lepidoptera;Polyommatus celina;NA;0.072;Polyommatus celina;0.999999999953559;
GU676086;Lepidoptera;Plebejus argus;Plebejus argus;0.001;Plebejus argus;0.967036594263956;
GU676087;Lepidoptera;Plebejus argus;Plebejus argus;0.008;Plebejus argus;0.978603339047252;
GU676088;Lepidoptera;Cupido argiades;NA;0.499;NA;0.999999999984682;
GU676089;Lepidoptera;Plebejus argus;Plebejus argus;0.003;Plebejus argus;0.990726105541344;
GU676090;Lepidoptera;Cupido alcetas;NA;0.068;Cupido alcetas;0.88988907876118;
GU676091;Lepidoptera;Cupido argiades;NA;0.499;NA;0.999999999984682;
GU676094;Lepidoptera;Favonius quercus;NA;NA;Favonius quercus;0.73046069582187;
GU676095;Lepidoptera;Favonius quercus;Favonius quercus;1;Favonius quercus;0.998074872649888;
GU676096;Lepidoptera;Colias alfacariensis;Colias alfacariensis;0.001;Colias alfacariensis;0.999857790810452;
GU676097;Lepidoptera;Colias alfacariensis;Colias alfacariensis;1;Colias alfacariensis;0.99986120827666;
GU676098;Lepidoptera;Pieris napi;NA;0.028;Pieris napi;0.888205720664699;
GU676102;Lepidoptera;Erebia euryale;NA;NA;Erebia euryale;0.93493911427573;
GU676103;Lepidoptera;Maniola jurtina;Maniola jurtina;0.004;Maniola jurtina;0.985235775650841;
GU676104;Lepidoptera;Satyrus actaea;Satyrus actaea;0.002;Satyrus actaea;0.998246235973604;
GU676105;Lepidoptera;Satyrus actaea;Satyrus actaea;0.01;Satyrus actaea;0.94366382029787;
GU676108;Lepidoptera;Hipparchia fidia;Hipparchia fidia;1;Hipparchia fidia;1;
GU676110;Lepidoptera;Maniola tithonus;Pyronia tithonus;NA;Pyronia tithonus;1;
GU676111;Lepidoptera;Maniola tithonus;Pyronia tithonus;1;Pyronia tithonus;1;
GU676112;Lepidoptera;Lasiommata megera;Lasiommata megera;0.995;Lasiommata megera;0.999279716854958;
GU676114;Lepidoptera;Melitaea parthenoides;Melitaea parthenoides;1;Melitaea parthenoides;0.999999993530452;
GU676115;Lepidoptera;Lasiommata maera;Lasiommata maera;1;Lasiommata maera;0.999995306565851;
GU676116;Lepidoptera;Melitaea parthenoides;Melitaea parthenoides;1;Melitaea parthenoides;0.99999999620502;
GU676117;Lepidoptera;Melitaea parthenoides;Melitaea parthenoides;1;Melitaea parthenoides;0.999999993473381;
GU676118;Lepidoptera;Euphydryas aurinia;Euphydryas aurinia;0.02;Euphydryas aurinia;0.99668249939786;
GU676119;Lepidoptera;Araschnia levana;Araschnia levana;1;Araschnia levana;0.999999772187832;
GU676120;Lepidoptera;Araschnia levana;Araschnia levana;1;Araschnia levana;0.999999772187832;
GU676127;Lepidoptera;Coenonympha dorus;Coenonympha dorus;1;Coenonympha dorus;0.999999999743835;
GU676129;Lepidoptera;Coenonympha dorus;Coenonympha dorus;1;Coenonympha dorus;0.999999999943213;
GU676130;Lepidoptera;Brenthis daphne;Brenthis daphne;1;Brenthis daphne;0.997627940680404;
GU676134;Lepidoptera;Erebia euryale;NA;NA;Erebia euryale;0.93493911427573;
GU676135;Lepidoptera;Satyrium ilicis;Satyrium ilicis;1;Satyrium ilicis;0.999999987901305;

GU676137;Lepidoptera;Brenthis daphne;Brenthis daphne;1;Brenthis daphne;0.997172799742872;
GU676138;Lepidoptera;Melitaea didyma;Melitaea didyma;NA;Melitaea didyma;0.980272836986251;
GU676139;Lepidoptera;Hipparchia semele;Hipparchia semele;0.001;Hipparchia semele;0.88547266660975;
GU676140;Lepidoptera;Polyommatus escheri;Polyommatus escheri;1;Polyommatus escheri;0.999999984088305;
GU676141;Lepidoptera;Lampides boeticus;Lampides boeticus;1;Lampides boeticus;0.999999998557229;
GU676142;Lepidoptera;Maniola jurtina;NA;0.001;Maniola jurtina;0.939220105860875;
GU676144;Lepidoptera;Satyrium esculi;Satyrium esculi;1;Satyrium esculi;0.999999957367948;
GU676147;Lepidoptera;Phengaris arion;NA;0.009;Phengaris arion;0.834433860638034;
GU676148;Lepidoptera;Melitaea nevadensis;Melitaea celadussa;0.994;Melitaea celadussa;0.995853651157716;
GU676149;Lepidoptera;Polyommatus escheri;Polyommatus escheri;1;Polyommatus escheri;0.999999989429227;
GU676150;Lepidoptera;Melitaea deione;Melitaea deione;1;Melitaea deione;0.999999998728185;
GU676151;Lepidoptera;Satyrus ferula;Satyrus ferula;0.001;Satyrus ferula;0.9958095881137;
GU676153;Lepidoptera;Cupido osiris;Cupido osiris;1;Cupido osiris;0.999974988645513;
GU676154;Lepidoptera;Ochlodes sylvanus;NA;0.316;Ochlodes sylvanus;0.996318754838625;
GU676155;Lepidoptera;Hipparchia semele;NA;NA;Hipparchia semele;0.845949489688888;
GU676156;Lepidoptera;Gonepteryx cleopatra;Gonepteryx cleopatra;0.998;Gonepteryx cleopatra;0.999118636582266;
GU676157;Lepidoptera;Hipparchia fidia;Hipparchia fidia;1;Hipparchia fidia;1;
GU676159;Lepidoptera;Melitaea deione;Melitaea deione;1;Melitaea deione;0.99999999580632;
GU676160;Lepidoptera;Carcharodus lavatherae;Carcharodus lavatherae;1;Carcharodus lavatherae;0.999975293871709;
GU676161;Lepidoptera;Melitaea nevadensis;Melitaea celadussa;0.999;Melitaea celadussa;0.998819206090668;
GU676163;Lepidoptera;Argynnis aglaja;NA;0.005;Speyeria aglaja;0.896438572419641;
GU676164;Lepidoptera;Melitaea parthenoides;Melitaea parthenoides;1;Melitaea parthenoides;0.999999968152765;
GU676165;Lepidoptera;Satyrium acaciae;Satyrium acaciae;1;Satyrium acaciae;0.99999991343697;
GU676166;Lepidoptera;Cercyonis lupina;Hyponephele lupina;1;Hyponephele lupina;0.99999998018663;
GU676167;Lepidoptera;Thymelicus lineola;Thymelicus lineola;1;Thymelicus lineola;0.999999096983497;
GU676168;Lepidoptera;Thymelicus lineola;Thymelicus lineola;1;Thymelicus lineola;0.999999080121537;
GU676169;Lepidoptera;Nymphalis c-album;NA;0.207;NA;0.999998867685115;
GU676172;Lepidoptera;Carcharodus alceae;Carcharodus alceae;1;Carcharodus alceae;0.999999997554852;
GU676173;Lepidoptera;Brintesia circe;Brintesia circe;1;Brintesia circe;0.999999572713085;
GU676174;Lepidoptera;Plebejus argus;Plebejus argus;1;Plebejus argus;0.996350867978009;
GU676175;Lepidoptera;Ochlodes sylvanus;NA;0.316;Ochlodes sylvanus;0.996318754838625;
GU676176;Lepidoptera;Satyrium esculi;Satyrium esculi;1;Satyrium esculi;0.999999957367948;
GU676178;Lepidoptera;Melitaea cinxia;Melitaea cinxia;1;Melitaea cinxia;0.999999999999659;
GU676179;Lepidoptera;Thymelicus sylvestris;Thymelicus sylvestris;1;Thymelicus sylvestris;0.999999985935205;
GU676180;Lepidoptera;Cercyonis lupina;Hyponephele lupina;1;Hyponephele lupina;0.999999952251103;
GU676181;Lepidoptera;Gegenes nostrodamus;Gegenes nostrodamus;1;Gegenes nostrodamus;0.999999996883645;
GU676182;Lepidoptera;Gegenes nostrodamus;Gegenes nostrodamus;1;Gegenes nostrodamus;0.999999996883645;
GU676183;Lepidoptera;Thymelicus acteon;Thymelicus acteon;1;Thymelicus acteon;0.99999999752248;
GU676184;Lepidoptera;Lasiommata maera;Lasiommata maera;1;Lasiommata maera;0.999997945450226;
GU676186;Lepidoptera;Lasiommata maera;Lasiommata maera;1;Lasiommata maera;0.999997945450226;
GU676190;Lepidoptera;Lasiommata maera;Lasiommata maera;1;Lasiommata maera;0.999997945450226;
GU676191;Lepidoptera;Aglais urticae;NA;0.002;Aglais urticae;0.987497593219019;
GU676195;Lepidoptera;Brenthis daphne;Brenthis daphne;0.001;Brenthis daphne;0.997781825264304;
GU676197;Lepidoptera;Lasiommata maera;Lasiommata maera;1;Lasiommata maera;0.999997660206086;
GU676198;Lepidoptera;Lycaena virgaureae;Lycaena virgaureae;0.025;Lycaena virgaureae;0.999833961481754;
GU676200;Lepidoptera;Satyrium spini;Satyrium spini;1;Satyrium spini;0.999997876872024;
GU676201;Lepidoptera;Erebia meolans;Erebia meolans;NA;Erebia meolans;0.99957934552327;
GU676203;Lepidoptera;Argynnis paphia;Argynnis paphia;1;Argynnis paphia;0.999999991389927;
GU676204;Lepidoptera;Hipparchia semele;Hipparchia semele;0.001;Hipparchia semele;0.926224616831104;
GU676205;Lepidoptera;Chazara briseis;Chazara briseis;0.005;Chazara briseis;0.999971123216223;
GU676206;Lepidoptera;Hipparchia semele;NA;0.004;Hipparchia semele;0.752871175214561;
GU676207;Lepidoptera;Satyrium ilicis;Satyrium ilicis;1;Satyrium ilicis;0.999999987901305;
GU676208;Lepidoptera;Lycaena virgaureae;Lycaena virgaureae;0.025;Lycaena virgaureae;0.999833961481754;
GU676209;Lepidoptera;Nymphalis c-album;NA;0.08;NA;0.999996025433302;
GU676210;Lepidoptera;Aphantopus hyperantus;Aphantopus hyperantus;0.003;Aphantopus hyperantus;0.999999455871523;
GU676211;Lepidoptera;Brenthis hecate;NA;0.068;Brenthis hecate;0.999053551498733;
GU676215;Lepidoptera;Fabriciana adippe;Fabriciana adippe;0.001;Fabriciana adippe;0.999538788643547;
GU676216;Lepidoptera;Coenonympha dorus;Coenonympha dorus;1;Coenonympha dorus;0.999999999915957;

GU676217;Lepidoptera;Carcharodus lavatherae;Carcharodus lavatherae;1;Carcharodus lavatherae;0.999975293871709;
GU676219;Lepidoptera;Satyrium spini;Satyrium spini;1;Satyrium spini;0.999997334596599;
GU676221;Lepidoptera;Brenthis ino;Brenthis ino;0.001;Brenthis ino;0.997741439264217;
GU676223;Lepidoptera;Polyommatus damon;Polyommatus damon;1;Polyommatus damon;0.999999939976279;
GU676224;Lepidoptera;Polyommatus damon;Polyommatus damon;1;Polyommatus damon;0.999999924215334;
GU676228;Lepidoptera;Plebejus idas;NA;0.001;NA;0.999367461111577;
GU676231;Lepidoptera;Brenthis ino;Brenthis ino;0.999;Brenthis ino;0.998672633091632;
GU676232;Lepidoptera;Satyrium acaciae;Satyrium acaciae;1;Satyrium acaciae;0.99999991343697;
GU676233;Lepidoptera;Satyrium ilicis;Satyrium ilicis;1;Satyrium ilicis;0.999999987901305;
GU676236;Lepidoptera;Plebejus idas;NA;0.001;NA;0.999590911470354;
GU676237;Lepidoptera;Boloria dia;Boloria dia;1;Boloria dia;0.999999999980503;
GU676238;Lepidoptera;Satyrium acaciae;Satyrium acaciae;1;Satyrium acaciae;0.999999453316214;
GU676239;Lepidoptera;Satyrium spini;Satyrium spini;1;Satyrium spini;0.999997334596599;
GU676244;Lepidoptera;Melitaea nevadensis;Melitaea celadussa;0.999;Melitaea celadussa;0.998819206090668;
GU676245;Lepidoptera;Boloria dia;Boloria dia;1;Boloria dia;0.999999999980503;
GU676246;Lepidoptera;Brenthis daphne;Brenthis daphne;0.001;Brenthis daphne;0.997781825264304;
GU676247;Lepidoptera;Melitaea didyma;Melitaea didyma;1;Melitaea didyma;0.996477093809569;
GU676248;Lepidoptera;Brenthis hecate;NA;0.068;Brenthis hecate;0.999053551498733;
GU676249;Lepidoptera;Maniola tithonus;Pyronia tithonus;1;Pyronia tithonus;0.999999999999972;
GU676250;Lepidoptera;Satyrium esculi;Satyrium esculi;1;Satyrium esculi;0.999999957367948;
GU676251;Lepidoptera;Fabriciana niobe;NA;0.001;Fabriciana niobe;0.924679661444846;
GU676252;Lepidoptera;Coenonympha arcania;NA;0.172;Coenonympha arcania;0.963036309473038;
GU676253;Lepidoptera;Coenonympha arcania;NA;0.172;Coenonympha arcania;0.963036309473038;
GU676256;Lepidoptera;Melitaea didyma;Melitaea didyma;1;Melitaea didyma;0.996366765827355;
GU676259;Lepidoptera;Hipparchia hermione;Hipparchia hermione;1;Hipparchia hermione;0.999765856753897;
GU676260;Lepidoptera;Polyommatus amandus;Polyommatus amandus;0.001;Polyommatus amandus;0.999982520055691;
GU676271;Lepidoptera;Melitaea parthenoides;Melitaea parthenoides;1;Melitaea parthenoides;0.999999968152765;
GU676272;Lepidoptera;Pieris napi;NA;0.011;Pieris napi;0.801985394724814;
GU676273;Lepidoptera;Melitaea cinxia;Melitaea cinxia;1;Melitaea cinxia;0.999999999999659;
GU676276;Lepidoptera;Polyommatus dorylas;Polyommatus dorylas;0.025;Polyommatus dorylas;0.973229109265438;
GU676277;Lepidoptera;Argynnis aglaja;NA;NA;NA;0.999999999998776;
GU676278;Lepidoptera;Polyommatus amandus;Polyommatus amandus;0.002;Polyommatus amandus;0.999895064798667;
GU676279;Lepidoptera;Polyommatus escheri;Polyommatus escheri;1;Polyommatus escheri;0.999997480524106;
GU676281;Lepidoptera;Satyrium acaciae;Satyrium acaciae;1;Satyrium acaciae;0.99999991343697;
GU676282;Lepidoptera;Plebejus argus;NA;0.04;Plebejus argus;0.972686995581077;
GU676283;Lepidoptera;Melitaea cinxia;Melitaea cinxia;1;Melitaea cinxia;0.999999999999659;
GU676284;Lepidoptera;Coenonympha glycerion;NA;0.221;Coenonympha glycerion;0.999998704466507;
GU676286;Lepidoptera;Hamearis lucina;NA;NA;Hamearis lucina;0.999999999984141;
GU676290;Lepidoptera;Melitaea trivia;Melitaea trivia;1;Melitaea trivia;0.999999999908766;
GU676291;Lepidoptera;Plebejus argus;Plebejus argus;0.01;Plebejus argus;0.979195638958128;
GU676292;Lepidoptera;Satyrium esculi;Satyrium esculi;1;Satyrium esculi;0.999999957367948;
GU676296;Lepidoptera;Lasiommata megera;Lasiommata megera;1;Lasiommata megera;0.99994902516581;
GU676297;Lepidoptera;Pieris napi;NA;0.028;Pieris napi;0.843942654364311;
GU676298;Lepidoptera;Glaucopsyche alexis;Glaucopsyche alexis;0.001;Glaucopsyche alexis;0.994667256195263;
GU676299;Lepidoptera;Euphydryas aurinia;Euphydryas aurinia;0.016;Euphydryas aurinia;0.997751503369639;
GU676304;Lepidoptera;Argynnis aglaja;NA;NA;NA;0.999999999995822;
GU676305;Lepidoptera;Gonepteryx rhamni;Gonepteryx rhamni;0.002;Gonepteryx rhamni;0.999788876860678;
GU676306;Lepidoptera;Glaucopsyche melanops;Glaucopsyche melanops;1;Glaucopsyche melanops;0.999952924254607;
GU676308;Lepidoptera;Pyrgus serratulae;Pyrgus serratulae;1;Pyrgus serratulae;0.99999949307896;
GU676313;Lepidoptera;Melitaea deione;Melitaea deione;1;Melitaea deione;0.999999983690515;
GU676314;Lepidoptera;Aporia crataegi;NA;0.002;Aporia crataegi;0.999996938385945;
GU676315;Lepidoptera;Maniola tithonus;Pyronia tithonus;1;Pyronia tithonus;1;
GU676320;Lepidoptera;Melitaea parthenoides;Melitaea parthenoides;1;Melitaea parthenoides;0.999999978339219;
GU676321;Lepidoptera;Melitaea cinxia;Melitaea cinxia;1;Melitaea cinxia;0.999999999999659;
GU676322;Lepidoptera;Melitaea trivia;Melitaea trivia;1;Melitaea trivia;0.999999999938666;
GU676323;Lepidoptera;Aglais urticae;NA;0.002;Aglais urticae;0.987497593219019;
GU676324;Lepidoptera;Coenonympha glycerion;NA;0.134;Coenonympha glycerion;0.999998730379228;
GU676325;Lepidoptera;Fabriciana niobe;NA;0.035;Fabriciana niobe;0.949480222958778;
GU676326;Lepidoptera;Aricia eumedon;Eumedonia eumedon;1;Eumedonia eumedon;0.993730847153741;
GU676327;Lepidoptera;Aricia eumedon;Eumedonia eumedon;1;Eumedonia eumedon;0.993730847153741;
GU676329;Lepidoptera;Cyaniris semiargus;NA;NA;Cyaniris semiargus;0.999066914949168;
GU676330;Lepidoptera;Lasiommata megera;Lasiommata megera;1;Lasiommata megera;0.999928528239676;
GU676331;Lepidoptera;Polyommatus celina;NA;0.072;Polyommatus celina;0.999999999953559;
GU676333;Lepidoptera;Celastrina argiolus;Celastrina argiolus;1;Celastrina argiolus;0.999527385150531;

GU676341;Lepidoptera;Maniola jurtina;NA;0.001;Maniola jurtina;0.939220105860875;
GU676342;Lepidoptera;Satyrium esculi;Satyrium esculi;1;Satyrium esculi;0.999999957367948;
GU676343;Lepidoptera;Maniola cecilia;Pyronia cecilia;NA;Pyronia cecilia;1;
GU676344;Lepidoptera;Carcharodus baeticus;Carcharodus baeticus;1;Carcharodus baeticus;0.999983899664537;
GU676345;Lepidoptera;Coenonympha dorus;Coenonympha dorus;1;Coenonympha dorus;0.999999999041165;
GU676346;Lepidoptera;Callophrys avis;Callophrys avis;1;Callophrys avis;0.992335035373355;
GU676347;Lepidoptera;Callophrys avis;Callophrys avis;1;Callophrys avis;0.992335035373355;
GU676348;Lepidoptera;Aricia eumedon;Eumedonia eumedon;0.002;Eumedonia eumedon;0.995876443425412;
GU676350;Lepidoptera;Pyrgus serratulae;Pyrgus serratulae;1;Pyrgus serratulae;0.999999822628057;
GU676353;Lepidoptera;Gegenes nostrodamus;Gegenes nostrodamus;1;Gegenes nostrodamus;0.999999996883645;
GU676355;Lepidoptera;Arethusana arethusa;Arethusana arethusa;1;Arethusana arethusa;0.999999801603556;
GU676359;Lepidoptera;Aricia eumedon;Eumedonia eumedon;0.001;Eumedonia eumedon;0.997136200888682;
GU676362;Lepidoptera;Lysandra coridon;NA;NA;NA;0.999976559473762;
GU676363;Lepidoptera;Lysandra coridon;NA;NA;NA;0.999976559473762;
GU676367;Lepidoptera;Polyommatus daphnis;NA;0.031;Polyommatus daphnis;0.989661132749926;
GU676368;Lepidoptera;Lysandra hispana;NA;NA;NA;0.999978182264605;
GU676370;Lepidoptera;Hipparchia fidia;Hipparchia fidia;1;Hipparchia fidia;1;
GU676372;Lepidoptera;Polyommatus icarus;NA;0.029;Polyommatus icarus;0.828260567531005;
GU676373;Lepidoptera;Lysandra hispana;NA;NA;NA;0.999963114385083;
GU676374;Lepidoptera;Maniola jurtina;NA;0.001;Maniola jurtina;0.939220105860875;
GU676375;Lepidoptera;Hipparchia fidia;Hipparchia fidia;1;Hipparchia fidia;1;
GU676377;Lepidoptera;Lysandra hispana;NA;NA;NA;0.99981082296412;
GU676379;Lepidoptera;Lysandra hispana;Lysandra hispana;NA;Lysandra hispana;0.712402577741674;
GU676380;Lepidoptera;Maniola tithonus;Pyronia tithonus;NA;Pyronia tithonus;1;
GU676381;Lepidoptera;Hipparchia semele;NA;0.002;Hipparchia semele;0.767063813608107;
GU676382;Lepidoptera;Satyrus actaea;Satyrus actaea;0.999;Satyrus actaea;0.999013870742133;
GU676383;Lepidoptera;Lysandra hispana;NA;NA;NA;0.999883930469524;
GU676384;Lepidoptera;Colias alfacariensis;Colias alfacariensis;0.001;Colias alfacariensis;0.999857790810452;
GU676385;Lepidoptera;Brenthis hecate;NA;0.068;Brenthis hecate;0.999053551498733;
GU676388;Lepidoptera;Coenonympha dorus;Coenonympha dorus;1;Coenonympha dorus;0.99999999988259;
GU676392;Lepidoptera;Brenthis ino;Brenthis ino;0.998;Brenthis ino;0.995325118916732;
GU676393;Lepidoptera;Polyommatus damon;Polyommatus damon;1;Polyommatus damon;0.999999435300384;
GU676394;Lepidoptera;Arethusana arethusa;Arethusana arethusa;0.045;Arethusana arethusa;0.999962297609568;
GU676395;Lepidoptera;Melitaea didyma;Melitaea didyma;1;Melitaea didyma;0.998761608110557;
GU676396;Lepidoptera;Brenthis ino;Brenthis ino;0.998;Brenthis ino;0.995325118916732;
GU676399;Lepidoptera;Polyommatus escheri;Polyommatus escheri;1;Polyommatus escheri;0.999999984824285;
GU676402;Lepidoptera;Polyommatus amandus;Polyommatus amandus;0.001;Polyommatus amandus;0.999984793725812;
GU676404;Lepidoptera;Melitaea parthenoides;Melitaea parthenoides;1;Melitaea parthenoides;0.999999993530452;
GU676405;Lepidoptera;Polyommatus damon;Polyommatus damon;1;Polyommatus damon;0.999999721622043;
GU676407;Lepidoptera;Hipparchia semele;NA;0.001;Hipparchia semele;0.862655722502182;
GU676408;Lepidoptera;Plebejus idas;NA;0.001;NA;0.999163905769696;
GU676409;Lepidoptera;Plebejus argus;Plebejus argus;0.008;Plebejus argus;0.982355058519351;
GU676415;Lepidoptera;Satyrus actaea;NA;0.001;Satyrus actaea;0.992438087901691;
GU676417;Lepidoptera;Fabriciana niobe;NA;0.035;Fabriciana niobe;0.949480222958778;
GU676419;Lepidoptera;Colias alfacariensis;Colias alfacariensis;0.001;Colias alfacariensis;0.999857790810452;
GU676421;Lepidoptera;Plebejus argus;Plebejus argus;0.003;Plebejus argus;0.990726105541344;
GU676423;Lepidoptera;Aglais urticae;Aglais urticae;0.004;Aglais urticae;0.995016750255472;
GU676424;Lepidoptera;Polyommatus amandus;Polyommatus amandus;0.001;Polyommatus amandus;0.999982544243848;
GU676425;Lepidoptera;Argynnis aglaja;NA;NA;NA;0.999999999998776;
GU676426;Lepidoptera;Coenonympha arcania;NA;0.172;Coenonympha arcania;0.963036309473038;
GU676427;Lepidoptera;Lycaena phlaeas;Lycaena phlaeas;1;Lycaena phlaeas;0.999999996363186;
GU676429;Lepidoptera;Plebejus argus;Plebejus argus;1;Plebejus argus;0.995486594467474;
GU676430;Lepidoptera;Polyommatus escheri;Polyommatus escheri;1;Polyommatus escheri;0.999999989738654;
GU676431;Lepidoptera;Glaucopsyche alexis;NA;NA;Glaucopsyche alexis;0.991537790028883;
GU676432;Lepidoptera;Cupido osiris;Cupido osiris;1;Cupido osiris;0.999999815277948;
GU676433;Lepidoptera;Lasiommata megera;Lasiommata megera;1;Lasiommata megera;0.999928528239676;
GU676436;Lepidoptera;Euphydryas aurinia;Euphydryas aurinia;0.02;Euphydryas aurinia;0.99668249939786;
GU676438;Lepidoptera;Polyommatus celina;Polyommatus celina;0.009;Polyommatus celina;0.999999999949694;
GU676439;Lepidoptera;Coenonympha pamphilus;Coenonympha pamphilus;1;Coenonympha pamphilus;0.999999999999488;
GU676440;Lepidoptera;Satyrium spini;Satyrium spini;1;Satyrium spini;0.999999026999281;
GU676444;Lepidoptera;Lampides boeticus;Lampides boeticus;1;Lampides boeticus;0.999999999545906;
GU676445;Lepidoptera;Satyrium spini;Satyrium spini;1;Satyrium spini;0.999999026999281;
GU676447;Lepidoptera;Aricia cramera;Aricia cramera;1;Aricia cramera;0.999993731037033;
GU676448;Lepidoptera;Anthocharis euphenoides;NA;0.172;Anthocharis euphenoides;0.943741664916385;

GU676453;Lepidoptera;Anthocharis cardamines;Anthocharis cardamines;1;Anthocharis cardamines;0.999999951562672;
GU676455;Lepidoptera;Lycaena phlaeas;Lycaena phlaeas;1;Lycaena phlaeas;0.999999996363186;
GU676457;Lepidoptera;Callophrys rubi;NA;0.002;Callophrys rubi;0.998152239914699;
GU676458;Lepidoptera;Glaucopsyche melanops;Glaucopsyche melanops;1;Glaucopsyche melanops;0.999953769170809;
GU676463;Lepidoptera;Maniola jurtina;NA;NA;Maniola jurtina;0.951182761228822;
GU676464;Lepidoptera;Melitaea didyma;Melitaea didyma;1;Melitaea didyma;0.999727166931204;
GU676465;Lepidoptera;Aporia crataegi;NA;0.002;Aporia crataegi;0.999996497444636;
GU676466;Lepidoptera;Cyaniris semiargus;NA;NA;Cyaniris semiargus;0.998973546847411;
GU676472;Lepidoptera;Cupido minimus;NA;0.009;Cupido minimus;0.999791453712643;
GU676477;Lepidoptera;Polyommatus escheri;Polyommatus escheri;1;Polyommatus escheri;0.999999994765119;
GU676478;Lepidoptera;Polyommatus escheri;Polyommatus escheri;1;Polyommatus escheri;0.999999998529631;
GU676479;Lepidoptera;Polyommatus escheri;Polyommatus escheri;1;Polyommatus escheri;0.999999998529631;
GU676481;Lepidoptera;Polyommatus escheri;Polyommatus escheri;1;Polyommatus escheri;0.999998281574049;
GU676482;Lepidoptera;Polyommatus escheri;Polyommatus escheri;1;Polyommatus escheri;0.999999997873005;
GU676484;Lepidoptera;Polyommatus icarus;Polyommatus icarus;1;Polyommatus icarus;0.988670826309563;
GU676487;Lepidoptera;Anthocharis euphenoides;NA;0.12;Anthocharis euphenoides;0.931402619863286;
GU676489;Lepidoptera;Polyommatus icarus;Polyommatus icarus;0.001;Polyommatus icarus;0.977842229419285;
GU676490;Lepidoptera;Plebejus idas;NA;0.001;NA;0.999163905769696;
GU676491;Lepidoptera;Fabriciana niobe;NA;0.035;Fabriciana niobe;0.949480222958778;
GU676492;Lepidoptera;Melitaea parthenoides;Melitaea parthenoides;1;Melitaea parthenoides;0.999999993530452;
GU676494;Lepidoptera;Melitaea parthenoides;Melitaea parthenoides;1;Melitaea parthenoides;0.99999997819765;
GU676499;Lepidoptera;Plebejus argus;Plebejus argus;0.003;Plebejus argus;0.991225116398388;
GU676500;Lepidoptera;Colias crocea;NA;0.059;NA;0.999999999999738;
GU676501;Lepidoptera;Coenonympha glycerion;NA;0.157;Coenonympha glycerion;0.999999004304256;
GU676504;Lepidoptera;Maniola jurtina;NA;0.001;Maniola jurtina;0.939220105860875;
GU676505;Lepidoptera;Callophrys rubi;NA;0.001;Callophrys rubi;0.99870518510598;
GU676506;Lepidoptera;Cupido minimus;NA;0.009;Cupido minimus;0.999791453712643;
GU676508;Lepidoptera;Coenonympha glycerion;NA;0.284;Coenonympha glycerion;0.999998676061348;
GU676509;Lepidoptera;Cupido osiris;Cupido osiris;1;Cupido osiris;0.999999791352473;
GU676510;Lepidoptera;Glaucopsyche alexis;Glaucopsyche alexis;0.001;Glaucopsyche alexis;0.994667256195263;
GU676511;Lepidoptera;Euphydryas aurinia;Euphydryas aurinia;0.016;Euphydryas aurinia;0.997751503369639;
GU676512;Lepidoptera;Anthocharis cardamines;Anthocharis cardamines;1;Anthocharis cardamines;0.999999895074103;
GU676516;Lepidoptera;Argynnis aglaja;NA;NA;NA;0.999999999998776;
GU676517;Lepidoptera;Lampides boeticus;Lampides boeticus;1;Lampides boeticus;0.999999999411585;
GU676519;Lepidoptera;Anthocharis euphenoides;NA;0.12;Anthocharis euphenoides;0.931402619863286;
GU676520;Lepidoptera;Cupido minimus;NA;0.026;Cupido minimus;0.999181550941374;
GU676521;Lepidoptera;Hamearis lucina;NA;NA;Hamearis lucina;0.999999999984141;
GU676522;Lepidoptera;Callophrys avis;Callophrys avis;1;Callophrys avis;0.992335035373355;
GU676524;Lepidoptera;Aporia crataegi;NA;0.009;Aporia crataegi;0.999996613335036;
GU676527;Lepidoptera;Fabriciana adippe;Fabriciana adippe;1;Fabriciana adippe;0.999446572009028;
GU676528;Lepidoptera;Lasiommata megera;Lasiommata megera;1;Lasiommata megera;0.999943800289156;
GU676532;Lepidoptera;Callophrys avis;Callophrys avis;1;Callophrys avis;0.994873005421139;
GU676533;Lepidoptera;Callophrys avis;Callophrys avis;1;Callophrys avis;0.992335035373355;
GU676536;Lepidoptera;Anthocharis euphenoides;NA;0.172;Anthocharis euphenoides;0.943741664916385;
GU676540;Lepidoptera;Thymelicus sylvestris;Thymelicus sylvestris;1;Thymelicus sylvestris;0.999999985935205;
GU676541;Lepidoptera;Lampides boeticus;Lampides boeticus;1;Lampides boeticus;0.999999999545906;
GU676542;Lepidoptera;Plebejus argus;Plebejus argus;0.014;Plebejus argus;0.979849220658559;
GU676543;Lepidoptera;Lasiommata megera;Lasiommata megera;1;Lasiommata megera;0.999928528239676;
GU676547;Lepidoptera;Lycaena phlaeas;Lycaena phlaeas;1;Lycaena phlaeas;0.999999996363186;
GU676548;Lepidoptera;Hipparchia hermione;Hipparchia hermione;0.001;Hipparchia hermione;0.999954661253629;
GU676549;Lepidoptera;Coenonympha dorus;Coenonympha dorus;1;Coenonympha dorus;0.999999999798803;
GU676550;Lepidoptera;Coenonympha glycerion;NA;0.176;Coenonympha glycerion;0.999998747950729;
GU676551;Lepidoptera;Coenonympha glycerion;NA;0.083;Coenonympha glycerion;0.999999102623609;
GU676552;Lepidoptera;Maniola jurtina;NA;0.001;Maniola jurtina;0.939220105860875;
GU676553;Lepidoptera;Maniola tithonus;Pyronia tithonus;NA;Pyronia tithonus;1;
GU676556;Lepidoptera;Nymphalis c-album;NA;0.215;NA;0.999999192918557;
GU676557;Lepidoptera;Ochlodes sylvanus;NA;0.316;Ochlodes sylvanus;0.996318754838625;
GU676558;Lepidoptera;Colias crocea;NA;NA;NA;0.999999999999584;
GU676559;Lepidoptera;Gonepteryx rhamni;Gonepteryx rhamni;1;Gonepteryx rhamni;0.999850170258883;
GU676561;Lepidoptera;Fabriciana adippe;Fabriciana adippe;0.001;Fabriciana adippe;0.999538788643547;

GU676562;Lepidoptera;Melitaea nevadensis;Melitaea celadussa;0.999;Melitaea celadussa;0.998819206090668;
GU676563;Lepidoptera;Satyrium spini;Satyrium spini;1;Satyrium spini;0.999994528861254;
GU676565;Lepidoptera;Thymelicus acteon;Thymelicus acteon;1;Thymelicus acteon;0.99999999752248;
GU676567;Lepidoptera;Brintesia circe;Brintesia circe;1;Brintesia circe;0.999995749349661;
GU676569;Lepidoptera;Polyommatus icarus;Polyommatus icarus;0.001;Polyommatus icarus;0.999851522482238;
GU676570;Lepidoptera;Melitaea parthenoides;Melitaea parthenoides;1;Melitaea parthenoides;0.999999953314018;
GU676571;Lepidoptera;Euphydryas aurinia;Euphydryas aurinia;0.016;Euphydryas aurinia;0.997751503369639;
GU676572;Lepidoptera;Euphydryas aurinia;Euphydryas aurinia;0.016;Euphydryas aurinia;0.997751503369639;
GU676573;Lepidoptera;Lycaena tityrus;Lycaena tityrus;1;Lycaena tityrus;0.999997855580031;
GU676574;Lepidoptera;Glaucopsyche melanops;Glaucopsyche melanops;1;Glaucopsyche melanops;0.999952924254607;
GU676575;Lepidoptera;Pararge aegeria;Pararge aegeria;1;Pararge aegeria;0.999999999963165;
GU676577;Lepidoptera;Pieris napi;NA;0.009;Pieris napi;0.896514330886707;
GU676579;Lepidoptera;Aporia crataegi;NA;NA;Aporia crataegi;0.999977873310658;
GU676581;Lepidoptera;Coenonympha pamphilus;Coenonympha pamphilus;1;Coenonympha pamphilus;0.999999999999631;
GU676583;Lepidoptera;Favonius quercus;Favonius quercus;0.001;Favonius quercus;0.998640139520663;
GU676585;Lepidoptera;Fabriciana adippe;Fabriciana adippe;1;Fabriciana adippe;0.997025694784552;
GU676586;Lepidoptera;Brintesia circe;Brintesia circe;1;Brintesia circe;0.999999905095796;
GU676589;Lepidoptera;Lampides boeticus;Lampides boeticus;1;Lampides boeticus;0.999999999545906;
GU676590;Lepidoptera;Anthocharis euphenoides;NA;0.073;Anthocharis euphenoides;0.954712885635666;
GU676592;Lepidoptera;Glaucopsyche melanops;Glaucopsyche melanops;1;Glaucopsyche melanops;0.999952924254607;
GU676593;Lepidoptera;Polyommatus celina;NA;0.029;Polyommatus celina;0.999999999960295;
GU676595;Lepidoptera;Vanessa atalanta;Vanessa atalanta;1;Vanessa atalanta;0.999999999469395;
GU676596;Lepidoptera;Colias crocea;NA;0.021;Colias crocea;0.749631165399089;
GU676597;Lepidoptera;Glaucopsyche melanops;Glaucopsyche melanops;1;Glaucopsyche melanops;0.999981879020163;
GU676600;Lepidoptera;Anthocharis euphenoides;Anthocharis euphenoides;0.008;Anthocharis euphenoides;0.985036987582027;
GU676603;Lepidoptera;Coenonympha pamphilus;Coenonympha pamphilus;1;Coenonympha pamphilus;0.999999999999744;
GU676604;Lepidoptera;Lycaena phlaeas;Lycaena phlaeas;1;Lycaena phlaeas;0.999999996363186;
GU676605;Lepidoptera;Carcharodus alceae;Carcharodus alceae;0.999;Carcharodus alceae;0.999999996339511;
GU676609;Lepidoptera;Pararge aegeria;Pararge aegeria;1;Pararge aegeria;0.999999999963165;
GU676611;Lepidoptera;Polyommatus icarus;Polyommatus icarus;0.001;Polyommatus icarus;0.999851522482238;
GU676613;Lepidoptera;Pieris napi;NA;0.028;Pieris napi;0.888205720664699;
GU676615;Lepidoptera;Parnassius mnemosyne;Parnassius mnemosyne;1;Parnassius mnemosyne;0.999999269897625;
GU676616;Lepidoptera;Parnassius mnemosyne;Parnassius mnemosyne;1;Parnassius mnemosyne;0.999999269897625;
GU676617;Lepidoptera;Pyrgus malvoides;NA;0.076;Pyrgus malvoides;0.887854315566122;
GU676621;Lepidoptera;Glaucopsyche alexis;Glaucopsyche alexis;0.001;Glaucopsyche alexis;0.994667256195263;
GU676622;Lepidoptera;Cupido minimus;NA;0.001;Cupido minimus;0.999420080317202;
GU676624;Lepidoptera;Anthocharis euphenoides;NA;NA;Anthocharis euphenoides;0.948387107617061;
GU676627;Lepidoptera;Gonepteryx rhamni;Gonepteryx rhamni;1;Gonepteryx rhamni;0.999844409790237;
GU676629;Lepidoptera;Celastrina argiolus;Celastrina argiolus;1;Celastrina argiolus;0.999527385150531;
GU676630;Lepidoptera;Gonepteryx cleopatra;Gonepteryx cleopatra;0.998;Gonepteryx cleopatra;0.999118636582266;
GU676632;Lepidoptera;Colias alfacariensis;Colias alfacariensis;0.001;Colias alfacariensis;0.999857790810452;
GU676634;Lepidoptera;Carcharodus alceae;Carcharodus alceae;0.999;Carcharodus alceae;0.999999996339511;
GU676638;Lepidoptera;Vanessa atalanta;Vanessa atalanta;1;Vanessa atalanta;0.999999999757762;
GU676639;Lepidoptera;Maniola cecilia;Pyronia cecilia;1;Pyronia cecilia;1;
GU676640;Lepidoptera;Callophrys rubi;NA;0.001;Callophrys rubi;0.99870518510598;
GU676648;Lepidoptera;Lycaena tityrus;Lycaena tityrus;1;Lycaena tityrus;0.999997855580031;
GU676649;Lepidoptera;Aporia crataegi;NA;NA;Aporia crataegi;0.999996781126733;
GU676651;Lepidoptera;Maniola jurtina;NA;NA;Maniola jurtina;0.949518227999457;
GU676654;Lepidoptera;Polyommatus icarus;Polyommatus icarus;0.001;Polyommatus icarus;0.999851522482238;
GU676660;Lepidoptera;Coenonympha pamphilus;Coenonympha pamphilus;1;Coenonympha pamphilus;0.999999999999744;
GU676663;Lepidoptera;Anthocharis cardamines;Anthocharis cardamines;1;Anthocharis cardamines;0.99999998453049;
GU676666;Lepidoptera;Glaucopsyche melanops;Glaucopsyche melanops;1;Glaucopsyche melanops;0.999952924254607;
GU676668;Lepidoptera;Glaucopsyche melanops;Glaucopsyche melanops;1;Glaucopsyche melanops;0.999977090845591;

GU676669;Lepidoptera;Lycaena phlaeas;Lycaena phlaeas;1;Lycaena phlaeas;0.999999996363186;
GU676671;Lepidoptera;Vanessa atalanta;Vanessa atalanta;1;Vanessa atalanta;0.999999999757762;
GU676674;Lepidoptera;Lasiommata megera;Lasiommata megera;1;Lasiommata megera;0.999951812067954;
GU676675;Lepidoptera;Anthocharis cardamines;Anthocharis cardamines;1;Anthocharis cardamines;0.99999998453049;
GU676676;Lepidoptera;Pararge aegeria;Pararge aegeria;1;Pararge aegeria;0.999999999963165;
GU676679;Lepidoptera;Callophrys rubi;NA;0.001;Callophrys rubi;0.99870518510598;
GU676680;Lepidoptera;Plebejus argus;Plebejus argus;1;Plebejus argus;0.995353400367624;
GU676685;Lepidoptera;Aglais urticae;NA;0.002;Aglais urticae;0.987497593219019;
GU676687;Lepidoptera;Lycaena phlaeas;Lycaena phlaeas;1;Lycaena phlaeas;0.999999996363186;
GU676689;Lepidoptera;Anthocharis euphenoides;NA;0.172;Anthocharis euphenoides;0.943741664916385;
GU676691;Lepidoptera;Colias alfacariensis;Colias alfacariensis;0.001;Colias alfacariensis;0.999857790810452;
GU676693;Lepidoptera;Anthocharis cardamines;Anthocharis cardamines;1;Anthocharis cardamines;0.99999998453049;
GU676694;Lepidoptera;Pyrgus onopordi;Pyrgus onopordi;1;Pyrgus onopordi;0.999998134499953;
GU676700;Lepidoptera;Pyrgus onopordi;Pyrgus onopordi;1;Pyrgus onopordi;0.999997111229755;
GU676701;Lepidoptera;Gonepteryx cleopatra;Gonepteryx cleopatra;0.998;Gonepteryx cleopatra;0.999118636582266;
GU676702;Lepidoptera;Gonepteryx cleopatra;Gonepteryx cleopatra;0.998;Gonepteryx cleopatra;0.999118636582266;
GU676703;Lepidoptera;Carcharodus alceae;Carcharodus alceae;0.999;Carcharodus alceae;0.999999996339511;
GU676710;Lepidoptera;Pararge aegeria;Pararge aegeria;1;Pararge aegeria;0.999999999365514;
GU676712;Lepidoptera;Celastrina argiolus;Celastrina argiolus;1;Celastrina argiolus;0.999527385150531;
GU676713;Lepidoptera;Colias alfacariensis;Colias alfacariensis;0.001;Colias alfacariensis;0.999857790810452;
GU676714;Lepidoptera;Maniola jurtina;NA;0.001;Maniola jurtina;0.939220105860875;
GU676716;Lepidoptera;Colias crocea;NA;0.059;NA;0.999999999999738;
GU676717;Lepidoptera;Lycaena phlaeas;Lycaena phlaeas;1;Lycaena phlaeas;0.999999996363186;
GU676720;Lepidoptera;Maniola cecilia;Pyronia cecilia;1;Pyronia cecilia;1;
GU676721;Lepidoptera;Polyommatus celina;Polyommatus celina;0.001;Polyommatus celina;0.999999999499352;
GU676723;Lepidoptera;Melitaea deione;Melitaea deione;1;Melitaea deione;0.999999999627732;
GU676724;Lepidoptera;Vanessa cardui;Vanessa cardui;1;Vanessa cardui;0.999999459983318;
GU676725;Lepidoptera;Gonepteryx cleopatra;Gonepteryx cleopatra;0.998;Gonepteryx cleopatra;0.999118636582266;
GU676726;Lepidoptera;Polyommatus celina;Polyommatus celina;0.001;Polyommatus celina;0.999999999717033;
GU676727;Lepidoptera;Lycaena phlaeas;Lycaena phlaeas;1;Lycaena phlaeas;0.999999996363186;
GU676729;Lepidoptera;Celastrina argiolus;Celastrina argiolus;1;Celastrina argiolus;0.999527385150531;
GU676730;Lepidoptera;Gonepteryx cleopatra;Gonepteryx cleopatra;0.998;Gonepteryx cleopatra;0.999118636582266;
GU676731;Lepidoptera;Coenonympha pamphilus;Coenonympha pamphilus;1;Coenonympha pamphilus;0.999999999995282;
GU676732;Lepidoptera;Coenonympha pamphilus;Coenonympha pamphilus;1;Coenonympha pamphilus;0.999999999995424;
GU676733;Lepidoptera;Vanessa cardui;Vanessa cardui;1;Vanessa cardui;0.999999803450484;
GU676734;Lepidoptera;Colias crocea;NA;0.059;NA;0.999999999999738;
GU676735;Lepidoptera;Vanessa atalanta;Vanessa atalanta;1;Vanessa atalanta;0.999999999757762;
GU676738;Lepidoptera;Pararge aegeria;Pararge aegeria;1;Pararge aegeria;0.999999999365514;
GU676740;Lepidoptera;Scolitantides orion;Scolitantides orion;1;Scolitantides orion;0.999999953757596;
GU676741;Lepidoptera;Melitaea parthenoides;Melitaea parthenoides;1;Melitaea parthenoides;0.999999426287269;
GU676742;Lepidoptera;Vanessa atalanta;Vanessa atalanta;1;Vanessa atalanta;0.999999999757762;
GU676743;Lepidoptera;Pyrgus cacaliae;Pyrgus cacaliae;0.999;Pyrgus cacaliae;0.997229839348598;
GU676744;Lepidoptera;Thymelicus sylvestris;Thymelicus sylvestris;1;Thymelicus sylvestris;0.999999985935205;
GU676747;Lepidoptera;Chazara briseis;Chazara briseis;1;Chazara briseis;0.999980393060755;
GU676752;Lepidoptera;Polyommatus damon;Polyommatus damon;1;Polyommatus damon;0.999999502567344;
GU676754;Lepidoptera;Limenitis camilla;Limenitis camilla;0.009;Limenitis camilla;0.9999828626808;
GU676755;Lepidoptera;Apatura ilia;NA;0.165;Apatura ilia;0.956796812031481;
GU676757;Lepidoptera;Carcharodus alceae;Carcharodus alceae;0.999;Carcharodus alceae;0.999999996339511;
GU676758;Lepidoptera;Polyommatus eros;NA;0.003;Polyommatus eros;0.985402505884526;
GU676759;Lepidoptera;Polyommatus eros;NA;0.003;Polyommatus eros;0.985402505884526;
GU676762;Lepidoptera;Pyrgus carthami;Pyrgus carthami;1;Pyrgus carthami;0.999997353719783;
GU676764;Lepidoptera;Colias phicomone;Colias phicomone;0.001;Colias phicomone;0.986802434025645;
GU676765;Lepidoptera;Colias phicomone;Colias phicomone;0.001;Colias phicomone;0.986802434025645;
GU676766;Lepidoptera;Cyaniris semiargus;Cyaniris semiargus;0.001;Cyaniris semiargus;0.998806431837647;
GU676767;Lepidoptera;Polyommatus icarus;NA;0.004;Polyommatus icarus;0.999481683747521;
GU676768;Lepidoptera;Polyommatus icarus;NA;NA;Polyommatus icarus;0.984400584728957;
GU676772;Lepidoptera;Polyommatus celina;NA;0.072;Polyommatus celina;0.999999999953559;

GU676778;Lepidoptera;Carcharodus baeticus;Carcharodus baeticus;1;Carcharodus baeticus;0.999983899664537;
GU676779;Lepidoptera;Pyrgus malvoides;NA;0.211;Pyrgus malvoides;0.811806911877104;
GU676781;Lepidoptera;Satyrium acaciae;Satyrium acaciae;1;Satyrium acaciae;0.99999991343697;
GU676786;Lepidoptera;Cupido osiris;Cupido osiris;1;Cupido osiris;0.999999791352473;
GU676791;Lepidoptera;Anthocharis euphenoides;NA;0.131;Anthocharis euphenoides;0.934740202775498;
GU676794;Lepidoptera;Colias alfacariensis;Colias alfacariensis;0.001;Colias alfacariensis;0.999857790810452;
GU676796;Lepidoptera;Carcharodus baeticus;Carcharodus baeticus;1;Carcharodus baeticus;0.999956286218202;
GU676797;Lepidoptera;Polyommatus icarus;Polyommatus icarus;0.001;Polyommatus icarus;0.999851522482238;
GU676800;Lepidoptera;Carcharodus alceae;Carcharodus alceae;0.001;Carcharodus alceae;0.999999970193983;
GU676801;Lepidoptera;Satyrium spini;Satyrium spini;1;Satyrium spini;0.999998173309697;
GU676803;Lepidoptera;Melitaea deione;Melitaea deione;1;Melitaea deione;0.999999996865029;
GU676806;Lepidoptera;Carcharodus alceae;Carcharodus alceae;0.999;Carcharodus alceae;0.999999996339511;
GU676809;Lepidoptera;Thymelicus sylvestris;Thymelicus sylvestris;1;Thymelicus sylvestris;0.999999985935205;
GU676810;Lepidoptera;Gegenes nostrodamus;Gegenes nostrodamus;1;Gegenes nostrodamus;0.999999996883645;
GU676812;Lepidoptera;Gegenes nostrodamus;Gegenes nostrodamus;1;Gegenes nostrodamus;0.999999996883645;
GU676813;Lepidoptera;Gegenes nostrodamus;Gegenes nostrodamus;1;Gegenes nostrodamus;0.999999887376482;
GU676814;Lepidoptera;Pyrgus malvoides;NA;0.076;Pyrgus malvoides;0.887854315566122;
GU676815;Lepidoptera;Thymelicus acteon;Thymelicus acteon;1;Thymelicus acteon;0.99999999752248;
GU676816;Lepidoptera;Pyrgus malvoides;NA;0.076;Pyrgus malvoides;0.887854315566122;
GU676817;Lepidoptera;Pyrgus carthami;Pyrgus carthami;1;Pyrgus carthami;0.999997085657879;
GU676819;Lepidoptera;Pyrgus serratulae;Pyrgus serratulae;1;Pyrgus serratulae;0.999999877361184;
GU676820;Lepidoptera;Carcharodus lavatherae;Carcharodus lavatherae;1;Carcharodus lavatherae;0.999975293871709;
GU676821;Lepidoptera;Pyrgus serratulae;Pyrgus serratulae;1;Pyrgus serratulae;0.99999949307896;
GU676824;Lepidoptera;Ochlodes sylvanus;NA;0.316;Ochlodes sylvanus;0.996318754838625;
GU676825;Lepidoptera;Lysandra coridon;NA;NA;NA;0.999974015575741;
GU676826;Lepidoptera;Lysandra coridon;NA;NA;NA;0.999936262862373;
GU676827;Lepidoptera;Carcharodus alceae;Carcharodus alceae;0.999;Carcharodus alceae;0.999999996339511;
GU676828;Lepidoptera;Gegenes nostrodamus;Gegenes nostrodamus;1;Gegenes nostrodamus;0.999999996883645;
GU676829;Lepidoptera;Melitaea deione;Melitaea deione;1;Melitaea deione;0.999999832903242;
GU676830;Lepidoptera;Pyrgus onopordi;Pyrgus onopordi;1;Pyrgus onopordi;0.999997111229755;
GU676831;Lepidoptera;Lysandra coridon;NA;NA;NA;0.999974015575741;
GU676832;Lepidoptera;Lysandra coridon;NA;NA;NA;0.999974015575741;
GU676833;Lepidoptera;Lampides boeticus;Lampides boeticus;1;Lampides boeticus;0.999999999281187;
GU676834;Lepidoptera;Aglais urticae;Aglais urticae;0.004;Aglais urticae;0.995016750255472;
GU676838;Lepidoptera;Glaucopsyche melanops;Glaucopsyche melanops;1;Glaucopsyche melanops;0.999953769170809;
GU676840;Lepidoptera;Aglais urticae;Aglais urticae;0.001;Aglais urticae;0.986884738893991;
GU676841;Lepidoptera;Thymelicus acteon;Thymelicus acteon;1;Thymelicus acteon;0.99999999752248;
GU676843;Lepidoptera;Anthocharis euphenoides;NA;0.172;Anthocharis euphenoides;0.943741664916385;
GU676844;Lepidoptera;Celastrina argiolus;Celastrina argiolus;1;Celastrina argiolus;0.999527385150531;
GU676845;Lepidoptera;Lasiommata megera;Lasiommata megera;1;Lasiommata megera;0.999928528239676;
GU676846;Lepidoptera;Glaucopsyche melanops;Glaucopsyche melanops;1;Glaucopsyche melanops;0.999953769170809;
GU676847;Lepidoptera;Lycaena phlaeas;Lycaena phlaeas;1;Lycaena phlaeas;0.999999996363186;
GU676849;Lepidoptera;Melitaea deione;Melitaea deione;1;Melitaea deione;0.999999998728185;
GU676853;Lepidoptera;Charaxes jasius;Charaxes jasius;0.001;Charaxes jasius;0.997689374666591;
GU676869;Lepidoptera;Aporia crataegi;NA;NA;Aporia crataegi;0.999996445569743;
GU676870;Lepidoptera;Gonepteryx rhamni;NA;0.122;Gonepteryx rhamni;0.999344210758396;
GU676871;Lepidoptera;Hipparchia semele;NA;0.001;Hipparchia semele;0.792351581988454;
GU676872;Lepidoptera;Brintesia circe;Brintesia circe;1;Brintesia circe;0.999999922895629;
GU676873;Lepidoptera;Arethusana arethusa;NA;0.062;Arethusana arethusa;0.999946829736877;
GU676874;Lepidoptera;Fabriciana adippe;Fabriciana adippe;0.001;Fabriciana adippe;0.99939671801438;
GU676875;Lepidoptera;Maniola tithonus;Pyronia tithonus;NA;Pyronia tithonus;1;
GU676876;Lepidoptera;Brenthis ino;Brenthis ino;0.998;Brenthis ino;0.995325118916732;
GU676878;Lepidoptera;Melitaea nevadensis;Melitaea celadussa;1;Melitaea celadussa;0.996871309289245;
GU676881;Lepidoptera;Euphydryas aurinia;Euphydryas aurinia;0.016;Euphydryas aurinia;0.997751503369639;
GU676884;Lepidoptera;Coenonympha arcania;NA;0.172;Coenonympha arcania;0.963036309473038;
GU676885;Lepidoptera;Lampides boeticus;Lampides boeticus;1;Lampides boeticus;0.999999999469992;
GU676886;Lepidoptera;Euphydryas aurinia;Euphydryas aurinia;0.021;Euphydryas aurinia;0.996482336387469;
GU676888;Lepidoptera;Pieris napi;NA;0.028;Pieris napi;0.888205720664699;

GU676889;Lepidoptera;Pieris napi;NA;0.028;Pieris napi;0.888205720664699;
GU676891;Lepidoptera;Celastrina argiolus;Celastrina argiolus;1;Celastrina argiolus;0.999527385150531;
GU676892;Lepidoptera;Colias crocea;NA;0.059;NA;0.999999999999738;
GU676893;Lepidoptera;Colias crocea;NA;0.059;NA;0.999999999999738;
GU676895;Lepidoptera;Vanessa atalanta;Vanessa atalanta;1;Vanessa atalanta;0.999999999757762;
GU676896;Lepidoptera;Melitaea trivia;Melitaea trivia;1;Melitaea trivia;0.999999999977604;
GU676897;Lepidoptera;Anthocharis cardamines;Anthocharis cardamines;1;Anthocharis cardamines;0.999999940675226;
GU676899;Lepidoptera;Melitaea nevadensis;Melitaea celadussa;1;Melitaea celadussa;0.996871309289245;
GU676901;Lepidoptera;Cyaniris semiargus;NA;NA;Cyaniris semiargus;0.999051841766953;
GU676902;Lepidoptera;Lasiommata megera;Lasiommata megera;1;Lasiommata megera;0.999928528239676;
GU676904;Lepidoptera;Melitaea cinxia;Melitaea cinxia;1;Melitaea cinxia;0.999999999999659;
GU676905;Lepidoptera;Aporia crataegi;NA;0.007;Aporia crataegi;0.999997331828531;
GU676906;Lepidoptera;Satyrus actaea;Satyrus actaea;1;Satyrus actaea;0.998655924692805;
GU676908;Lepidoptera;Hipparchia hermione;Hipparchia hermione;1;Hipparchia hermione;0.999929600232128;
GU676909;Lepidoptera;Satyrus actaea;Satyrus actaea;1;Satyrus actaea;0.998400754517542;
GU676910;Lepidoptera;Lysandra coridon;NA;NA;NA;0.999938937260851;
GU676911;Lepidoptera;Coenonympha arcania;NA;0.172;Coenonympha arcania;0.963036309473038;
GU676913;Lepidoptera;Maniola tithonus;Pyronia tithonus;1;Pyronia tithonus;1;
GU676915;Lepidoptera;Vanessa cardui;Vanessa cardui;1;Vanessa cardui;0.999999459983318;
GU676919;Lepidoptera;Polyommatus celina;Polyommatus celina;0.021;Polyommatus celina;0.999999999963336;
GU676924;Lepidoptera;Satyrium esculi;Satyrium esculi;1;Satyrium esculi;0.999999957367948;
GU676928;Lepidoptera;Cupido argiades;NA;0.187;Cupido argiades;0.750699947178303;
GU676929;Lepidoptera;Cupido osiris;Cupido osiris;1;Cupido osiris;0.999999908943356;
GU676930;Lepidoptera;Fabriciana niobe;NA;0.035;Fabriciana niobe;0.949480222958778;
GU676932;Lepidoptera;Melitaea cinxia;Melitaea cinxia;1;Melitaea cinxia;0.999999999999517;
GU676933;Lepidoptera;Phengaris arion;NA;0.054;Phengaris arion;0.938397095316026;
GU676936;Lepidoptera;Cupido osiris;Cupido osiris;1;Cupido osiris;0.999999170120762;
GU676938;Lepidoptera;Cupido minimus;NA;0.009;Cupido minimus;0.999791453712643;
GU676940;Lepidoptera;Erynnis tages;Erynnis tages;1;Erynnis tages;0.999999281987018;
GU676941;Lepidoptera;Carcharodus alceae;Carcharodus alceae;0.001;Carcharodus alceae;0.999999970193983;
GU676942;Lepidoptera;Cupido osiris;Cupido osiris;1;Cupido osiris;0.999999791352473;
GU676943;Lepidoptera;Melitaea didyma;Melitaea didyma;NA;Melitaea didyma;0.97469276593809;
GU676945;Lepidoptera;Coenonympha pamphilus;Coenonympha pamphilus;1;Coenonympha pamphilus;0.999999999999744;
GU676946;Lepidoptera;Carcharodus alceae;Carcharodus alceae;0.999;Carcharodus alceae;0.999999996339511;
GU676947;Lepidoptera;Ochlodes sylvanus;NA;0.316;Ochlodes sylvanus;0.996318754838625;
GU676948;Lepidoptera;Cercyonis lupina;Hyponephele lupina;1;Hyponephele lupina;0.999999992730324;
GU676950;Lepidoptera;Hipparchia hermione;Hipparchia hermione;1;Hipparchia hermione;0.999921864017579;
GU676951;Lepidoptera;Satyrus actaea;Satyrus actaea;0.999;Satyrus actaea;0.998319392230218;
GU676952;Lepidoptera;Lysandra hispana;Lysandra hispana;NA;Lysandra hispana;0.788541986725807;
GU676954;Lepidoptera;Coenonympha dorus;Coenonympha dorus;1;Coenonympha dorus;0.999999999757307;
GU676956;Lepidoptera;Brenthis daphne;NA;0.151;NA;0.999999999489116;
GU676958;Lepidoptera;Ochlodes sylvanus;NA;0.316;Ochlodes sylvanus;0.996318754838625;
GU676959;Lepidoptera;Thymelicus acteon;Thymelicus acteon;1;Thymelicus acteon;0.99999999752248;
GU676961;Lepidoptera;Ochlodes sylvanus;NA;0.316;Ochlodes sylvanus;0.996318754838625;
GU676962;Lepidoptera;Pyrgus serratulae;Pyrgus serratulae;1;Pyrgus serratulae;0.999999694697398;
GU676964;Lepidoptera;Polyommatus eros;Polyommatus eros;0.001;Polyommatus eros;0.986004783752008;
GU676965;Lepidoptera;Cyaniris semiargus;NA;NA;Cyaniris semiargus;0.999097907036339;
GU676966;Lepidoptera;Boloria euphrosyne;Boloria euphrosyne;0.002;Boloria euphrosyne;0.999970544424708;
GU676967;Lepidoptera;Polyommatus eros;NA;0.003;Polyommatus eros;0.985402505884526;
GU676969;Lepidoptera;Polyommatus amandus;Polyommatus amandus;0.003;Polyommatus amandus;0.999980071833264;
GU676970;Lepidoptera;Lysandra hispana;NA;NA;NA;0.999905175238227;
GU676971;Lepidoptera;Pyrgus carthami;Pyrgus carthami;1;Pyrgus carthami;0.999981615717954;
GU676972;Lepidoptera;Satyrus ferula;Satyrus ferula;0.001;Satyrus ferula;0.994329228962786;
GU676973;Lepidoptera;Coenonympha glycerion;NA;0.28;Coenonympha glycerion;0.999998036263913;
GU676974;Lepidoptera;Pontia callidice;Pontia callidice;1;Pontia callidice;0.999999998637406;
GU676975;Lepidoptera;Satyrus ferula;Satyrus ferula;0.001;Satyrus ferula;0.994329228962786;
GU676977;Lepidoptera;Agriades glandon;NA;0.002;Agriades glandon;0.987149722429845;
GU676980;Lepidoptera;Ochlodes sylvanus;NA;0.316;Ochlodes sylvanus;0.996318754838625;
GU676981;Lepidoptera;Pyrgus malvoides;NA;0.076;Pyrgus malvoides;0.887854315566122;
GU676982;Lepidoptera;Thymelicus lineola;Thymelicus lineola;1;Thymelicus lineola;0.999999292515094;
GU676985;Lepidoptera;Thymelicus lineola;Thymelicus lineola;1;Thymelicus lineola;0.999997636840483;
GU676986;Lepidoptera;Pyrgus carthami;Pyrgus carthami;1;Pyrgus carthami;0.999997085657879;
GU676987;Lepidoptera;Thymelicus lineola;Thymelicus lineola;1;Thymelicus lineola;0.999998106572607;
GU676989;Lepidoptera;Pyrgus onopordi;Pyrgus onopordi;1;Pyrgus onopordi;0.999997111229755;

GU676992;Lepidoptera;Carcharodus alceae;Carcharodus alceae;0.999;Carcharodus alceae;0.999999996339511;
GU676993;Lepidoptera;Erynnis tages;Erynnis tages;1;Erynnis tages;0.999999281987018;
GU676994;Lepidoptera;Carterocephalus palaemon;NA;0.052;Carterocephalus palaemon;0.728445051795325;
GU676995;Lepidoptera;Carterocephalus palaemon;NA;0.052;Carterocephalus palaemon;0.728445051795325;
GU676997;Lepidoptera;Carterocephalus palaemon;NA;0.052;Carterocephalus palaemon;0.728445051795325;
GU677003;Lepidoptera;Coenonympha dorus;Coenonympha dorus;1;Coenonympha dorus;0.999999999269562;
GU677004;Lepidoptera;Pyrgus malvoides;NA;0.076;Pyrgus malvoides;0.887854315566122;
GU677006;Lepidoptera;Pyrgus onopordi;Pyrgus onopordi;1;Pyrgus onopordi;0.999997111229755;
GU677010;Lepidoptera;Lysandra hispana;Lysandra hispana;NA;NA;0.999950101635271;
GU677011;Lepidoptera;Lysandra hispana;NA;NA;NA;0.999986194928665;
GU677014;Lepidoptera;Coenonympha dorus;Coenonympha dorus;1;Coenonympha dorus;0.99999999988259;
GU677022;Lepidoptera;Polyommatus escheri;Polyommatus escheri;1;Polyommatus escheri;0.999999972395529;
GU677023;Lepidoptera;Helleia helle;Lycaena helle;1;Lycaena helle;0.999999987876606;
GU677024;Lepidoptera;Lysandra coridon;Lysandra coridon;NA;Lysandra coridon;0.892412957467058;
GU677027;Lepidoptera;Lysandra coridon;Lysandra coridon;NA;Lysandra coridon;0.835615654600914;
GU677028;Lepidoptera;Satyrus actaea;Satyrus actaea;1;Satyrus actaea;0.998519952537604;
GU677030;Lepidoptera;Aglais urticae;NA;0.002;Aglais urticae;0.987497593219019;
GU677032;Lepidoptera;Erebia neoridas;NA;NA;Erebia neoridas;0.996118011663822;
GU677034;Lepidoptera;Erebia neoridas;NA;NA;Erebia neoridas;0.997866159969085;
GU677035;Lepidoptera;Cupido alcetas;NA;0.068;Cupido alcetas;0.88988907876118;
GU677037;Lepidoptera;Melitaea trivia;Melitaea trivia;1;Melitaea trivia;0.999999999977604;
GU677039;Lepidoptera;Cupido minimus;Cupido minimus;0.001;Cupido minimus;0.999926360783759;
GU677040;Lepidoptera;Melitaea trivia;Melitaea trivia;1;Melitaea trivia;0.999999999977604;
GU677042;Lepidoptera;Polyommatus damon;Polyommatus damon;1;Polyommatus damon;0.999999571555669;
GU677047;Lepidoptera;Chazara briseis;Chazara briseis;1;Chazara briseis;0.999982519182705;
GU677048;Lepidoptera;Euphydryas aurinia;Euphydryas aurinia;0.016;Euphydryas aurinia;0.997751503369639;
GU677051;Lepidoptera;Lasiommata megera;Lasiommata megera;1;Lasiommata megera;0.999928528239676;
GU677052;Lepidoptera;Celastrina argiolus;Celastrina argiolus;1;Celastrina argiolus;0.999527385150531;
GU686843;Lepidoptera;Thymelicus lineola;Thymelicus lineola;1;Thymelicus lineola;0.999971535093703;
GU686862;Lepidoptera;Cupido minimus;NA;0.006;Cupido minimus;0.999768371759258;
GU686877;Lepidoptera;Celastrina argiolus;Celastrina argiolus;1;Celastrina argiolus;0.999708846309046;
GU686947;Lepidoptera;Erebia pronoe;Erebia pronoe;1;Erebia pronoe;0.999733321385675;
GU686972;Lepidoptera;Pyrgus andromedae;Pyrgus andromedae;0.001;Pyrgus andromedae;0.998828669843251;
GU686973;Lepidoptera;Boloria euphrosyne;Boloria euphrosyne;0.002;Boloria euphrosyne;0.999982756586458;
GU686974;Lepidoptera;Erebia pandrose;Erebia pandrose;0.013;Erebia pandrose;0.974388092470716;
GU686979;Lepidoptera;Aricia agestis;NA;0.253;Aricia agestis;0.996350017387504;
GU686980;Lepidoptera;Aricia agestis;NA;0.253;Aricia agestis;0.996350017387504;
GU686981;Lepidoptera;Aricia agestis;NA;0.253;Aricia agestis;0.996350017387504;
GU686982;Lepidoptera;Aricia agestis;NA;NA;Aricia agestis;0.99525963650756;
GU686983;Lepidoptera;Aricia agestis;NA;0.253;Aricia agestis;0.996350017387504;
GU686984;Lepidoptera;Aricia agestis;NA;0.253;Aricia agestis;0.996350017387504;
GU686985;Lepidoptera;Aricia agestis;NA;0.253;Aricia agestis;0.996350017387504;
GU686986;Lepidoptera;Aricia agestis;NA;0.253;Aricia agestis;0.996350017387504;
GU686988;Lepidoptera;Aricia agestis;NA;0.253;Aricia agestis;0.996350017387504;
GU686990;Lepidoptera;Aricia agestis;NA;0.253;Aricia agestis;0.996350017387504;
GU686991;Lepidoptera;Pyrgus carthami;Pyrgus carthami;1;Pyrgus carthami;0.999998997209607;
GU686992;Lepidoptera;Aricia agestis;NA;0.253;Aricia agestis;0.996350017387504;
GU686993;Lepidoptera;Pyrgus armoricanus;Pyrgus armoricanus;1;Pyrgus armoricanus;0.999997353848817;
GU686994;Lepidoptera;Aricia agestis;NA;0.253;Aricia agestis;0.996350017387504;
GU686995;Lepidoptera;Aricia agestis;NA;0.253;Aricia agestis;0.996350017387504;
GU686997;Lepidoptera;Aricia agestis;NA;0.253;Aricia agestis;0.996350017387504;
GU686998;Lepidoptera;Aricia agestis;NA;0.253;Aricia agestis;0.996350017387504;
GU686999;Lepidoptera;Pyrgus alveus;NA;NA;Pyrgus alveus;0.941697735568124;
GU687000;Lepidoptera;Aricia agestis;NA;0.161;Aricia agestis;0.995266819041939;
GU687001;Lepidoptera;Pyrgus alveus;NA;NA;Pyrgus alveus;0.941697735568124;
GU687002;Lepidoptera;Pyrgus armoricanus;Pyrgus armoricanus;1;Pyrgus armoricanus;0.999997353848817;
GU687003;Lepidoptera;Aricia agestis;NA;0.253;Aricia agestis;0.996350017387504;
GU687004;Lepidoptera;Pyrgus alveus;NA;NA;Pyrgus alveus;0.941697735568124;
GU687005;Lepidoptera;Aricia agestis;NA;0.253;Aricia agestis;0.996350017387504;
GU687006;Lepidoptera;Pyrgus alveus;NA;NA;Pyrgus alveus;0.941697735568124;
GU687007;Lepidoptera;Aricia agestis;NA;0.253;Aricia agestis;0.996350017387504;
GU687008;Lepidoptera;Aricia agestis;NA;0.253;Aricia agestis;0.996350017387504;
GU687009;Lepidoptera;Aricia agestis;NA;0.253;Aricia agestis;0.996350017387504;
GU687010;Lepidoptera;Pyrgus alveus;NA;NA;Pyrgus alveus;0.941697735568124;
GU687011;Lepidoptera;Aricia agestis;NA;0.161;Aricia agestis;0.995266819041939;

GU687012;Lepidoptera;Pyrgus alveus;NA;NA;Pyrgus alveus;0.962152887824497;
GU687013;Lepidoptera;Pyrgus alveus;NA;NA;Pyrgus alveus;0.941697735568124;
GU687014;Lepidoptera;Pyrgus alveus;NA;NA;Pyrgus alveus;0.827119616974978;
GU687015;Lepidoptera;Pyrgus alveus;NA;NA;Pyrgus alveus;0.941697735568124;
GU687016;Lepidoptera;Aricia agestis;NA;0.253;Aricia agestis;0.996350017387504;
GU687017;Lepidoptera;Pyrgus alveus;NA;NA;Pyrgus alveus;0.941697735568124;
GU687018;Lepidoptera;Pyrgus alveus;NA;NA;Pyrgus alveus;0.941697735568124;
GU687019;Lepidoptera;Pyrgus alveus;NA;NA;Pyrgus alveus;0.941697735568124;
GU687020;Lepidoptera;Aricia agestis;NA;0.253;Aricia agestis;0.996350017387504;
GU687021;Lepidoptera;Pyrgus alveus;NA;NA;Pyrgus alveus;0.918895626779646;
GU687022;Lepidoptera;Aricia agestis;NA;0.253;Aricia agestis;0.996350017387504;
GU687023;Lepidoptera;Pyrgus alveus;NA;NA;Pyrgus alveus;0.827119616974978;
GU687024;Lepidoptera;Aricia agestis;NA;0.253;Aricia agestis;0.996350017387504;
GU687025;Lepidoptera;Pyrgus alveus;NA;0.205;Pyrgus alveus;0.796584470642115;
GU687026;Lepidoptera;Aricia agestis;NA;0.253;Aricia agestis;0.996350017387504;
GU687027;Lepidoptera;Pyrgus alveus;NA;NA;Pyrgus alveus;0.827119616974978;
GU687028;Lepidoptera;Aricia agestis;NA;0.253;Aricia agestis;0.996350017387504;
GU687029;Lepidoptera;Pyrgus alveus;NA;NA;Pyrgus alveus;0.827119616974978;
GU687030;Lepidoptera;Aricia agestis;NA;0.253;Aricia agestis;0.996350017387504;
GU687031;Lepidoptera;Pyrgus alveus;NA;NA;Pyrgus alveus;0.827119616974978;
GU687032;Lepidoptera;Aricia agestis;NA;0.253;Aricia agestis;0.996350017387504;
GU687033;Lepidoptera;Pyrgus alveus;NA;NA;Pyrgus alveus;0.827119616974978;
GU687034;Lepidoptera;Aricia agestis;NA;0.231;Aricia agestis;0.992802054683313;
GU687035;Lepidoptera;Pyrgus alveus;NA;NA;Pyrgus alveus;0.827119616974978;
GU687036;Lepidoptera;Aricia artaxerxes;Plebejus argus;1;Aricia artaxerxes;0.999743187199922;
GU687037;Lepidoptera;Pyrgus alveus;NA;NA;Pyrgus alveus;0.827119616974978;
GU687038;Lepidoptera;Aricia artaxerxes;Plebejus argus;1;Aricia artaxerxes;0.999743187199922;
GU687039;Lepidoptera;Aricia agestis;NA;0.253;Aricia agestis;0.996350017387504;
GU687040;Lepidoptera;Pyrgus alveus;NA;NA;Pyrgus alveus;0.827119616974978;
GU687041;Lepidoptera;Pyrgus alveus;NA;NA;Pyrgus alveus;0.827119616974978;
GU687042;Lepidoptera;Aricia artaxerxes;Plebejus argus;1;Aricia artaxerxes;0.999743187199922;
GU687043;Lepidoptera;Aricia agestis;NA;0.083;Aricia agestis;0.999021338679561;
GU687044;Lepidoptera;Aricia agestis;NA;NA;Aricia agestis;0.996930365249266;
GU687045;Lepidoptera;Pyrgus warrenensis;NA;0.106;NA;0.999999999994992;
GU687046;Lepidoptera;Pyrgus warrenensis;NA;0.003;NA;0.999999999997732;
GU687047;Lepidoptera;Aricia agestis;NA;0.083;Aricia agestis;0.999021338679561;
GU687048;Lepidoptera;Pyrgus warrenensis;NA;0.003;NA;0.999999999997732;
GU688437;Lepidoptera;Callophrys rubi;NA;0.003;Callophrys rubi;0.997328639918919;
GU688440;Lepidoptera;Callophrys rubi;NA;0.003;Callophrys rubi;0.996753822785804;
GU688441;Lepidoptera;Cupido minimus;NA;0.006;Cupido minimus;0.999768371759258;
GU688442;Lepidoptera;Satyrium spini;Satyrium spini;1;Satyrium spini;0.999999835426407;
GU688445;Lepidoptera;Plebejus argus;NA;0.328;Plebejus argus;0.969789734199889;
GU688446;Lepidoptera;Plebejus idas;NA;0.007;NA;0.999546324331549;
GU688448;Lepidoptera;Cyaniris semiargus;NA;NA;Cyaniris semiargus;0.999077874641703;
GU688451;Lepidoptera;Polyommatus daphnis;Polyommatus daphnis;0.008;Polyommatus
daphnis;0.99732138840566;
GU688452;Lepidoptera;Polyommatus dorylas;Polyommatus dorylas;0.022;Polyommatus
dorylas;0.975812216500382;
GU688454;Lepidoptera;Lysandra coridon;NA;NA;NA;0.998070184737648;
GU688455;Lepidoptera;Agriades optilete;Agriades optilete;1;Agriades optilete;0.999999991917434;
GU688457;Lepidoptera;Celastrina argiolus;Celastrina argiolus;1;Celastrina
argiolus;0.999607797738003;
GU688458;Lepidoptera;Thecla betulae;Thecla betulae;1;Thecla betulae;0.999999999923688;
GU688459;Lepidoptera;Lycaena tityrus;Lycaena tityrus;1;Lycaena tityrus;0.999999147518929;
GU688460;Lepidoptera;Lycaena virgaureae;NA;0.209;Lycaena virgaureae;0.999669745100572;
GU688463;Lepidoptera;Helleia helle;Lycaena helle;1;Lycaena helle;0.999999986154194;
GU688465;Lepidoptera;Lycaena phlaeas;Lycaena phlaeas;1;Lycaena phlaeas;0.999999996363186;
GU688466;Lepidoptera;Helleia helle;Lycaena helle;1;Lycaena helle;0.999999986154194;
GU688468;Lepidoptera;Fabriciana niobe;NA;0.005;Fabriciana niobe;0.992125835358755;
GU688470;Lepidoptera;Argynnis aglaja;NA;0.001;NA;0.999999999999586;
GU688471;Lepidoptera;Fabriciana adippe;Fabriciana adippe;0.028;Fabriciana
adippe;0.969769198942097;
GU688473;Lepidoptera;Boloria thore;Boloria thore;1;Boloria thore;0.999999998359726;
GU688476;Lepidoptera;Anthocharis cardamines;Anthocharis cardamines;1;Anthocharis
cardamines;0.999999361582729;
GU688503;Lepidoptera;Pieris napi;NA;0.013;Pieris napi;0.946576653115347;
GU688507;Lepidoptera;Colias crocea;NA;0.059;NA;0.999999999999738;
GU688508;Lepidoptera;Aporia crataegi;NA;NA;Aporia crataegi;0.999998459680561;
GU688510;Lepidoptera;Colias palaeno;NA;0.001;NA;0.999999999982869;
GU688531;Lepidoptera;Aricia agestis;NA;0.253;Aricia agestis;0.996350017387504;
GU688532;Lepidoptera;Plebejus idas;NA;0.007;NA;0.999546324331549;
GU688536;Lepidoptera;Pararge aegeria;Pararge aegeria;1;Pararge aegeria;0.999999999963165;
GU688538;Lepidoptera;Melitaea didyma;Melitaea didyma;1;Melitaea didyma;0.999768602965124;
GU688539;Lepidoptera;Melitaea didyma;Melitaea didyma;1;Melitaea didyma;0.999870115406424;
GU688541;Lepidoptera;Pyrgus armoricanus;Pyrgus armoricanus;1;Pyrgus
armoricanus;0.999997353848817;
GU688544;Lepidoptera;Lampides boeticus;Lampides boeticus;1;Lampides boeticus;0.999999999545906;
GU689149;Lepidoptera;Erebia cassioides;NA;0.003;NA;0.999999999999934;

GU689150;Lepidoptera;Colias phicomone;NA;0.001;Colias phicomone;0.985598879697681;
GU689154;Lepidoptera;Agriades orbitulus;Agriades orbitulus;0.002;Agriades orbitulus;0.999286783448229;
GU689196;Lepidoptera;Erebia pronoe;Erebia pronoe;1;Erebia pronoe;0.999718339357544;
GU707034;Lepidoptera;Satyrium pruni;NA;0.076;Satyrium pruni;0.990322364021655;
GU707035;Lepidoptera;Satyrium acaciae;Satyrium acaciae;1;Satyrium acaciae;0.999999951077798;
GU707037;Lepidoptera;Satyrium pruni;NA;0.076;Satyrium pruni;0.990322364021655;
GU707038;Lepidoptera;Ochlodes sylvanus;NA;0.316;Ochlodes sylvanus;0.996318754838625;
GU707039;Lepidoptera;Thymelicus sylvestris;Thymelicus sylvestris;1;Thymelicus sylvestris;0.99999876153052;
GU707041;Lepidoptera;Melitaea britomartis;NA;NA;Melitaea britomartis;0.960348127420398;
GU707085;Lepidoptera;Cupido minimus;NA;0.008;Cupido minimus;0.999319672482489;
GU707087;Lepidoptera;Vanessa cardui;Vanessa cardui;1;Vanessa cardui;0.999996792914969;
GU707088;Lepidoptera;Nymphalis c-album;NA;0.215;NA;0.999999192918557;
GU707089;Lepidoptera;Lycaena phlaeas;Lycaena phlaeas;1;Lycaena phlaeas;0.999999996363186;
GU707091;Lepidoptera;Fabriciana adippe;Fabriciana adippe;0.004;Fabriciana adippe;0.991347020421632;
GU707092;Lepidoptera;Araschnia levana;Araschnia levana;1;Araschnia levana;0.999999772187832;
GU707095;Lepidoptera;Limenitis camilla;Limenitis camilla;1;Limenitis camilla;0.999951160156017;
GU707097;Lepidoptera;Thymelicus lineola;Thymelicus lineola;1;Thymelicus lineola;0.999971535093703;
GU707098;Lepidoptera;Satyrium w-album;NA;0.056;Satyrium w-album;0.989378230996316;
GU707100;Lepidoptera;Celastrina argiolus;Celastrina argiolus;1;Celastrina argiolus;0.999708846309046;
GU707102;Lepidoptera;Lasiommata maera;Lasiommata maera;1;Lasiommata maera;0.99999510792253;
GU707115;Lepidoptera;Argynnis aglaja;NA;0.001;NA;0.999999999999586;
GU707127;Lepidoptera;Fabriciana adippe;NA;0.001;Fabriciana adippe;0.957131879909897;
GU707134;Lepidoptera;Plebejus argyrognomon;NA;0.001;NA;0.994620093546684;
GU707145;Lepidoptera;Plebejus argyrognomon;NA;0.001;NA;0.994620093546684;
GU707146;Lepidoptera;Coenonympha glycerion;NA;0.153;Coenonympha glycerion;0.999998464013956;
GU707147;Lepidoptera;Coenonympha oedippus;Coenonympha oedippus;1;Coenonympha oedippus;0.999999999999659;
GU707174;Lepidoptera;Coenonympha pamphilus;Coenonympha pamphilus;1;Coenonympha pamphilus;0.999999999999744;
GU707175;Lepidoptera;Pyrgus malvae;Pyrgus malvae;0.999;Pyrgus malvae;0.993531783407179;
GU707177;Lepidoptera;Melanargia galathea;NA;NA;Melanargia galathea;0.943593560237409;
GU707194;Lepidoptera;Argynnis aglaja;NA;0.001;NA;0.999999999999586;
GU707196;Lepidoptera;Apatura ilia;NA;0.165;Apatura ilia;0.956796812031481;
GU707265;Lepidoptera;Plebejus argus;Plebejus argus;0.013;Plebejus argus;0.99029909759821;
GU707266;Lepidoptera;Plebejus argyrognomon;NA;0.001;NA;0.994620093546684;
GU707268;Lepidoptera;Polyommatus daphnis;Polyommatus daphnis;0.008;Polyommatus daphnis;0.99732138840566;
GU707270;Lepidoptera;Phengaris arion;NA;0.054;Phengaris arion;0.938397095316026;
GU707272;Lepidoptera;Lycaena phlaeas;Lycaena phlaeas;1;Lycaena phlaeas;0.999999996363186;
GU707297;Lepidoptera;Coenonympha glycerion;NA;0.1;Coenonympha glycerion;0.99999728912966;
GU707298;Lepidoptera;Minois dryas;NA;0.025;Minois dryas;0.850746026247397;
GU707335;Lepidoptera;Colias alfacariensis;Colias alfacariensis;1;Colias alfacariensis;0.999986994131001;
GU828583;Lepidoptera;Pyrgus malvae;Pyrgus malvae;NA;Pyrgus malvae;0.982672823248561;
GU828690;Lepidoptera;Colias palaeno;NA;0.001;NA;0.999999999978266;
GU828706;Lepidoptera;Favonius quercus;Favonius quercus;NA;Favonius quercus;0.77732345775836;
GU947444;Lepidoptera;Parnassius apollo;Parnassius apollo;0.001;NA;0.954009296748392;
GU947445;Lepidoptera;Parnassius apollo;Parnassius apollo;0.001;NA;0.954009296748392;
GU947446;Lepidoptera;Parnassius apollo;Parnassius apollo;1;NA;0.874350707747126;
GU947447;Lepidoptera;Parnassius apollo;Parnassius apollo;NA;NA;0.884165190246321;
GU947448;Lepidoptera;Parnassius apollo;Parnassius apollo;NA;NA;0.884165190246321;
GU947449;Lepidoptera;Parnassius apollo;Parnassius apollo;NA;NA;0.884165190246321;
GU947450;Lepidoptera;Parnassius apollo;NA;NA;NA;0.838906008365956;
GU947451;Lepidoptera;Parnassius apollo;Parnassius apollo;NA;NA;0.891902714123732;
GU947452;Lepidoptera;Parnassius apollo;Parnassius apollo;NA;NA;0.990986284674012;
GU947453;Lepidoptera;Parnassius apollo;Parnassius apollo;NA;NA;0.990986284674012;
GU947454;Lepidoptera;Parnassius apollo;Parnassius apollo;NA;NA;0.985398750065504;
GU947455;Lepidoptera;Parnassius apollo;Parnassius apollo;NA;NA;0.980153071244881;
GU947456;Lepidoptera;Parnassius apollo;Parnassius apollo;NA;NA;0.980153071244881;
GU947457;Lepidoptera;Parnassius apollo;Parnassius apollo;0.999;NA;0.989661638427511;
GU947458;Lepidoptera;Parnassius apollo;Parnassius apollo;NA;NA;0.982628925458211;
GU947459;Lepidoptera;Parnassius apollo;Parnassius apollo;NA;NA;0.99112001391321;
GU947460;Lepidoptera;Parnassius apollo;Parnassius apollo;NA;NA;0.990986284674012;
GU947461;Lepidoptera;Parnassius apollo;NA;0.001;NA;0.987665686594832;
GU947462;Lepidoptera;Parnassius apollo;NA;0.001;NA;0.987665686594832;
GU947463;Lepidoptera;Parnassius apollo;Parnassius apollo;NA;NA;0.990986284674012;
GU947464;Lepidoptera;Parnassius apollo;Parnassius apollo;NA;NA;0.990986284674012;
GU947465;Lepidoptera;Parnassius apollo;Parnassius apollo;0.999;NA;0.989624310088282;
GU947466;Lepidoptera;Parnassius apollo;Parnassius apollo;0.999;NA;0.989661638427511;
GU947467;Lepidoptera;Parnassius apollo;Parnassius apollo;0.999;NA;0.989476314717979;
GU947468;Lepidoptera;Parnassius apollo;Parnassius apollo;NA;NA;0.990738781901465;
GU947469;Lepidoptera;Parnassius apollo;Parnassius apollo;0.999;NA;0.989661638427511;
GU947470;Lepidoptera;Parnassius apollo;Parnassius apollo;1;NA;0.881724007313126;

GU947471;Lepidoptera;Parnassius apollo;Parnassius apollo;1;NA;0.881724007313126;
GU947472;Lepidoptera;Parnassius apollo;Parnassius apollo;1;NA;0.877066671005351;
GU947473;Lepidoptera;Parnassius apollo;NA;NA;NA;0.989622139411186;
GU947474;Lepidoptera;Parnassius apollo;NA;NA;NA;0.989622139411186;
GU947475;Lepidoptera;Parnassius apollo;NA;NA;NA;0.989622139411186;
GU947476;Lepidoptera;Parnassius apollo;Parnassius apollo;1;NA;0.892878752965805;
GU947477;Lepidoptera;Parnassius apollo;Parnassius apollo;1;NA;0.892878752965805;
GU947478;Lepidoptera;Parnassius apollo;Parnassius apollo;NA;NA;0.901196708092871;
GU947479;Lepidoptera;Parnassius apollo;Parnassius apollo;1;NA;0.884930209631634;
GU947480;Lepidoptera;Parnassius apollo;Parnassius apollo;1;NA;0.885473976746205;
GU947481;Lepidoptera;Parnassius apollo;Parnassius apollo;1;NA;0.880313379921548;
GU947482;Lepidoptera;Parnassius apollo;Parnassius apollo;1;NA;0.885473976746205;
GU947483;Lepidoptera;Parnassius apollo;Parnassius apollo;1;NA;0.885473976746205;
GU947484;Lepidoptera;Parnassius apollo;Parnassius apollo;0.999;NA;0.864243075312234;
GU947485;Lepidoptera;Parnassius apollo;Parnassius apollo;0.999;NA;0.864243075312234;
GU947486;Lepidoptera;Parnassius apollo;Parnassius apollo;1;NA;0.884930209631634;
GU947487;Lepidoptera;Parnassius apollo;Parnassius apollo;NA;NA;0.991630921520101;
GU947488;Lepidoptera;Parnassius apollo;Parnassius apollo;NA;NA;0.991630921520101;
GU947489;Lepidoptera;Parnassius apollo;Parnassius apollo;NA;NA;0.990986284674012;
GU947490;Lepidoptera;Parnassius apollo;Parnassius apollo;NA;NA;0.990986284674012;
GU947491;Lepidoptera;Parnassius apollo;Parnassius apollo;NA;NA;0.990986284674012;
GU947492;Lepidoptera;Parnassius apollo;Parnassius apollo;NA;NA;0.989345334613694;
GU947493;Lepidoptera;Parnassius apollo;Parnassius apollo;NA;NA;0.990986284674012;
GU947494;Lepidoptera;Parnassius apollo;Parnassius apollo;NA;NA;0.990986284674012;
GU947498;Lepidoptera;Parnassius apollo;Parnassius apollo;NA;NA;0.990986284674012;
GU947499;Lepidoptera;Parnassius apollo;NA;NA;NA;0.99104307983794;
GU947500;Lepidoptera;Parnassius apollo;Parnassius apollo;0.001;Parnassius jacquemonti;0.724382339654484;
GU947501;Lepidoptera;Parnassius apollo;Parnassius apollo;NA;NA;0.990986284674012;
GU947502;Lepidoptera;Parnassius apollo;Parnassius apollo;NA;NA;0.990986284674012;
GU947503;Lepidoptera;Parnassius apollo;Parnassius apollo;NA;NA;0.990986284674012;
GU947504;Lepidoptera;Parnassius apollo;Parnassius apollo;NA;NA;0.990986284674012;
GU947505;Lepidoptera;Parnassius apollo;Parnassius apollo;NA;NA;0.990986284674012;
GU947506;Lepidoptera;Parnassius apollo;Parnassius apollo;NA;NA;0.990986284674012;
GU947507;Lepidoptera;Parnassius apollo;Parnassius apollo;NA;NA;0.990986284674012;
GU947508;Lepidoptera;Parnassius apollo;Parnassius apollo;NA;NA;0.990986284674012;
GU947509;Lepidoptera;Parnassius apollo;NA;NA;NA;0.99063758966645;
GU947510;Lepidoptera;Parnassius apollo;Parnassius apollo;NA;NA;0.990986284674012;
GU947511;Lepidoptera;Parnassius apollo;Parnassius apollo;NA;NA;0.990986284674012;
GU947512;Lepidoptera;Parnassius apollo;Parnassius apollo;NA;NA;0.990986284674012;
GU947513;Lepidoptera;Parnassius apollo;Parnassius apollo;NA;NA;0.968271506029224;
GU947514;Lepidoptera;Parnassius apollo;Parnassius apollo;NA;NA;0.990986284674012;
GU947515;Lepidoptera;Parnassius apollo;Parnassius apollo;NA;NA;0.990986284674012;
GU947516;Lepidoptera;Parnassius apollo;Parnassius apollo;NA;NA;0.990986284674012;
GU947517;Lepidoptera;Parnassius apollo;Parnassius apollo;NA;NA;0.990006916560279;
GU947518;Lepidoptera;Parnassius apollo;Parnassius apollo;NA;NA;0.990006916560279;
GU947519;Lepidoptera;Parnassius apollo;Parnassius apollo;NA;NA;0.989198282108868;
GU947520;Lepidoptera;Parnassius apollo;Parnassius apollo;NA;NA;0.990986284674012;
GU947521;Lepidoptera;Parnassius apollo;NA;0.001;NA;0.989131741916346;
GU947522;Lepidoptera;Parnassius apollo;Parnassius apollo;NA;NA;0.990986284674012;
GU947523;Lepidoptera;Parnassius apollo;NA;0.001;NA;0.989131741916346;
GU947524;Lepidoptera;Parnassius apollo;Parnassius apollo;NA;NA;0.992468773499913;
GU947525;Lepidoptera;Parnassius apollo;Parnassius apollo;NA;NA;0.990986284674012;
GU947526;Lepidoptera;Parnassius apollo;NA;0.001;NA;0.989131741916346;
GU947527;Lepidoptera;Parnassius apollo;NA;NA;NA;0.98930649699472;
GU947528;Lepidoptera;Parnassius apollo;NA;0.001;NA;0.989131741916346;
GU947529;Lepidoptera;Parnassius apollo;Parnassius apollo;NA;NA;0.988735135315133;
GU947530;Lepidoptera;Parnassius apollo;NA;NA;NA;0.989526577070085;
GU947531;Lepidoptera;Parnassius apollo;NA;NA;NA;0.989526577070085;
GU947532;Lepidoptera;Parnassius apollo;NA;NA;NA;0.989526577070085;
GU947533;Lepidoptera;Parnassius apollo;NA;NA;NA;0.989526577070085;
GU947534;Lepidoptera;Parnassius apollo;Parnassius apollo;NA;NA;0.988735135315133;
GU947535;Lepidoptera;Parnassius apollo;NA;NA;NA;0.989526577070085;
GU947536;Lepidoptera;Parnassius apollo;NA;NA;NA;0.989526577070085;
GU947537;Lepidoptera;Parnassius apollo;NA;NA;NA;0.989526577070085;
GU947538;Lepidoptera;Parnassius apollo;Parnassius apollo;NA;NA;0.988735135315133;
GU947539;Lepidoptera;Parnassius apollo;NA;NA;NA;0.991271529358124;
GU947540;Lepidoptera;Parnassius apollo;NA;NA;NA;0.990875901582542;
GU947541;Lepidoptera;Parnassius apollo;Parnassius apollo;NA;NA;0.990986284674012;
GU947542;Lepidoptera;Parnassius apollo;Parnassius apollo;NA;NA;0.990986284674012;
GU947543;Lepidoptera;Parnassius apollo;Parnassius apollo;NA;NA;0.990986284674012;
GU947544;Lepidoptera;Parnassius apollo;Parnassius apollo;NA;NA;0.990986284674012;
GU947545;Lepidoptera;Parnassius apollo;Parnassius apollo;NA;NA;0.990986284674012;
GU947546;Lepidoptera;Parnassius apollo;Parnassius apollo;NA;NA;0.990986284674012;
GU947547;Lepidoptera;Parnassius apollo;Parnassius apollo;NA;NA;0.990986284674012;
GU947548;Lepidoptera;Parnassius apollo;Parnassius apollo;NA;NA;0.990986284674012;
GU947549;Lepidoptera;Parnassius apollo;Parnassius apollo;NA;NA;0.990986284674012;
GU947550;Lepidoptera;Parnassius apollo;NA;NA;NA;0.989526577070085;

GU947551;Lepidoptera;Parnassius apollo;NA;NA;NA;0.989526577070085;
GU947552;Lepidoptera;Parnassius apollo;NA;NA;NA;0.989526577070085;
GU947553;Lepidoptera;Parnassius apollo;NA;NA;NA;0.989526577070085;
GU947554;Lepidoptera;Parnassius apollo;NA;NA;NA;0.989526577070085;
GU947555;Lepidoptera;Parnassius apollo;NA;NA;NA;0.989526577070085;
GU947556;Lepidoptera;Parnassius apollo;Parnassius apollo;NA;NA;0.990738781901465;
GU947557;Lepidoptera;Parnassius apollo;Parnassius apollo;NA;NA;0.990986284674012;
GU947558;Lepidoptera;Parnassius apollo;NA;0.001;NA;0.989131741916346;
GU947559;Lepidoptera;Parnassius apollo;NA;0.001;NA;0.989131741916346;
GU947560;Lepidoptera;Parnassius apollo;NA;0.001;NA;0.989131741916346;
GU947561;Lepidoptera;Parnassius apollo;NA;0.001;NA;0.989131741916346;
GU947562;Lepidoptera;Parnassius apollo;NA;0.001;NA;0.989131741916346;
GU947563;Lepidoptera;Parnassius apollo;NA;0.001;NA;0.989131741916346;
GU947564;Lepidoptera;Parnassius apollo;NA;0.001;NA;0.989131741916346;
GU947565;Lepidoptera;Parnassius apollo;NA;0.001;NA;0.989131741916346;
GU947566;Lepidoptera;Parnassius apollo;NA;0.001;NA;0.989131741916346;
GU947567;Lepidoptera;Parnassius apollo;NA;0.001;NA;0.989131741916346;
GU947568;Lepidoptera;Parnassius apollo;NA;0.001;NA;0.989131741916346;
GU947569;Lepidoptera;Parnassius apollo;NA;0.001;NA;0.989131741916346;
GU947570;Lepidoptera;Parnassius apollo;NA;NA;NA;0.987397047156887;
GU947571;Lepidoptera;Parnassius apollo;NA;0.001;NA;0.990780251461868;
GU947572;Lepidoptera;Parnassius apollo;NA;0.001;NA;0.988853415268291;
GU947573;Lepidoptera;Parnassius apollo;NA;NA;NA;0.988517365719056;
GU947574;Lepidoptera;Parnassius apollo;NA;NA;NA;0.931742877733348;
GU947575;Lepidoptera;Parnassius apollo;NA;NA;NA;0.931742877733348;
GU947583;Lepidoptera;Parnassius apollo;NA;0.001;NA;0.982466026230959;
GU947584;Lepidoptera;Parnassius apollo;NA;0.001;NA;0.982466026230959;
GU947585;Lepidoptera;Parnassius apollo;NA;0.001;NA;0.982326083382435;
GU947586;Lepidoptera;Parnassius apollo;NA;0.001;NA;0.982466026230959;
GU947587;Lepidoptera;Parnassius apollo;NA;0.001;NA;0.982466026230959;
GU947588;Lepidoptera;Parnassius apollo;NA;0.001;NA;0.978298787359085;
GU947589;Lepidoptera;Parnassius apollo;NA;0.001;NA;0.982466026230959;
GU947591;Lepidoptera;Parnassius apollo;NA;0.003;NA;0.987369084481944;
GU947592;Lepidoptera;Parnassius apollo;NA;NA;NA;0.979874532524271;
GU947593;Lepidoptera;Parnassius apollo;NA;0.001;NA;0.859092122961634;
GU947594;Lepidoptera;Parnassius apollo;Parnassius apollo;0.001;NA;0.826902666720126;
GU947595;Lepidoptera;Parnassius apollo;NA;NA;NA;0.979874532524271;
GU947596;Lepidoptera;Parnassius apollo;NA;NA;NA;0.978391058808047;
GU947597;Lepidoptera;Parnassius apollo;NA;NA;NA;0.979874532524271;
GU947598;Lepidoptera;Parnassius apollo;NA;NA;NA;0.979874532524271;
GU947599;Lepidoptera;Parnassius apollo;NA;NA;NA;0.979874532524271;
GU947600;Lepidoptera;Parnassius apollo;Parnassius apollo;0.003;NA;0.85448355982222;
GU947601;Lepidoptera;Parnassius apollo;Parnassius apollo;1;NA;0.877066671005351;
GU947602;Lepidoptera;Parnassius apollo;Parnassius apollo;1;NA;0.877066671005351;
GU947603;Lepidoptera;Parnassius apollo;Parnassius apollo;1;NA;0.877066671005351;
GU947604;Lepidoptera;Parnassius apollo;Parnassius apollo;1;NA;0.877066671005351;
GU947605;Lepidoptera;Parnassius apollo;Parnassius apollo;1;NA;0.877066671005351;
GU947606;Lepidoptera;Parnassius apollo;Parnassius apollo;1;NA;0.877066671005351;
GU947607;Lepidoptera;Parnassius apollo;Parnassius apollo;1;NA;0.877066671005351;
GU947608;Lepidoptera;Parnassius apollo;Parnassius apollo;1;NA;0.877066671005351;
GU947609;Lepidoptera;Parnassius apollo;Parnassius apollo;1;NA;0.877066671005351;
GU947610;Lepidoptera;Parnassius apollo;Parnassius apollo;1;NA;0.877066671005351;
GU947611;Lepidoptera;Parnassius apollo;Parnassius apollo;1;NA;0.877066671005351;
GU947612;Lepidoptera;Parnassius apollo;Parnassius apollo;1;NA;0.877066671005351;
GU947614;Lepidoptera;Parnassius apollo;NA;0.001;NA;0.982672372363842;
GU947615;Lepidoptera;Parnassius apollo;NA;0.001;NA;0.982672372363842;
GU947616;Lepidoptera;Parnassius apollo;Parnassius apollo;0.999;NA;0.8780739642792;
GU947617;Lepidoptera;Parnassius apollo;Parnassius apollo;0.999;NA;0.839977154997076;
GU947618;Lepidoptera;Parnassius apollo;NA;0.001;NA;0.984034011587861;
GU947619;Lepidoptera;Parnassius apollo;NA;0.001;NA;0.984010646751428;
GU947620;Lepidoptera;Parnassius apollo;Parnassius apollo;0.999;NA;0.859789013545169;
GU947621;Lepidoptera;Parnassius apollo;Parnassius apollo;0.999;NA;0.859789013545169;
GU947623;Lepidoptera;Parnassius apollo;Parnassius apollo;0.999;NA;0.860891429270674;
GU947624;Lepidoptera;Parnassius apollo;Parnassius apollo;0.999;NA;0.87199384371568;
GU947625;Lepidoptera;Parnassius apollo;Parnassius apollo;0.999;NA;0.872528875466083;
GU947626;Lepidoptera;Parnassius apollo;Parnassius apollo;0.999;NA;0.87199384371568;
GU947627;Lepidoptera;Parnassius apollo;Parnassius apollo;NA;NA;0.796513812421955;
GU947628;Lepidoptera;Parnassius apollo;Parnassius apollo;0.999;NA;0.872528875466083;
GU947629;Lepidoptera;Parnassius apollo;Parnassius apollo;0.999;NA;0.87199384371568;
GU947630;Lepidoptera;Parnassius apollo;Parnassius apollo;0.999;NA;0.87199384371568;
GU947631;Lepidoptera;Parnassius apollo;Parnassius apollo;0.999;NA;0.867370424573712;
GU947632;Lepidoptera;Parnassius apollo;NA;0.001;NA;0.984034011587861;
GU947633;Lepidoptera;Parnassius apollo;NA;0.001;NA;0.984034011587861;
GU947634;Lepidoptera;Parnassius apollo;NA;0.001;NA;0.984034011587861;
GU947635;Lepidoptera;Parnassius apollo;NA;0.001;NA;0.984034011587861;
GU947636;Lepidoptera;Parnassius apollo;Parnassius apollo;0.999;NA;0.87199384371568;
GU947637;Lepidoptera;Parnassius apollo;NA;0.001;NA;0.982672372363842;
GU947642;Lepidoptera;Parnassius mnemosyne;Parnassius mnemosyne;1;NA;0.994080593898526;

GUPPY161-17;Lepidoptera;Pieris rapae;NA;NA;NA;0.999999999999999;
GWOTL182-13;Lepidoptera;Limenitis camilla;Limenitis camilla;0.005;Limenitis camilla;0.999978382657506;
GWOUC014-19;Lepidoptera;Thymelicus lineola;Thymelicus lineola;1;Thymelicus lineola;0.999971535093703;
HBNK074-07;Lepidoptera;Boloria thore;Boloria thore;1;Boloria thore;0.999999717205594;
HBNK075-07;Lepidoptera;Boloria thore;Boloria thore;1;Boloria thore;0.999999717205594;
HBNK077-07;Lepidoptera;Boloria thore;Boloria thore;1;Boloria thore;0.999999425077642;
HBNK292-07;Lepidoptera;Colias palaeno;NA;0.002;NA;0.999999999983681;
HBNKB355-07;Lepidoptera;Polygonia c-album;NA;0.08;Polygonia calbum;0.847312432756229;
HBNKB361-07;Lepidoptera;Polygonia c-album;NA;0.215;NA;0.999999192918557;
HBOK031-08;Lepidoptera;Hipparchia semele;NA;0.001;Hipparchia semele;0.708426894198838;
HBOK101-08;Lepidoptera;Erebia eriphyle;Erebia eriphyle;1;Erebia eriphyle;0.870864608181857;
HBOK175-08;Lepidoptera;Polyommatus icarus;Polyommatus icarus;NA;Dissomorphia australiaria;0.985686791525344;
HE614681;Lepidoptera;Erebia pronoe;Erebia pronoe;1;Erebia pronoe;0.999627827275307;
HE614682;Lepidoptera;Erebia manto;Erebia manto;0.999;Erebia manto;0.999814324036993;
HE614683;Lepidoptera;Erebia epiphron;NA;NA;Erebia epiphron;0.887560949368923;
HE775142;Lepidoptera;Colias hyale;Colias hyale;0.043;Colias hyale;0.999998111229864;
HE775143;Lepidoptera;Colias hyale;Colias hyale;0.043;Colias hyale;0.999998111229864;
HG326621;Lepidoptera;Phengaris arion;NA;0.021;NA;0.791339524332038;
HG326623;Lepidoptera;Phengaris arion;NA;0.021;NA;0.791339524332038;
HG326624;Lepidoptera;Phengaris arion;NA;0.021;NA;0.791339524332038;
HG326625;Lepidoptera;Phengaris arion;NA;0.021;NA;0.791339524332038;
HG326626;Lepidoptera;Phengaris arion;NA;0.021;NA;0.791339524332038;
HG326627;Lepidoptera;Phengaris arion;NA;0.021;NA;0.791339524332038;
HG326628;Lepidoptera;Phengaris arion;NA;0.021;NA;0.791339524332038;
HG326629;Lepidoptera;Phengaris arion;NA;0.021;NA;0.791339524332038;
HG326630;Lepidoptera;Phengaris arion;NA;0.021;NA;0.790855775694374;
HG326631;Lepidoptera;Phengaris arion;NA;0.021;NA;0.791339524332038;
HG326632;Lepidoptera;Phengaris arion;NA;0.021;NA;0.791339524332038;
HG326633;Lepidoptera;Phengaris arion;NA;0.021;NA;0.791339524332038;
HG326634;Lepidoptera;Phengaris arion;NA;0.021;NA;0.792894606077744;
HG326635;Lepidoptera;Phengaris arion;NA;0.021;NA;0.791339524332038;
HG326636;Lepidoptera;Phengaris arion;NA;0.021;NA;0.79148828313844;
HG326637;Lepidoptera;Phengaris arion;NA;0.021;NA;0.79148828313844;
HG326638;Lepidoptera;Phengaris arion;NA;0.021;NA;0.793579063388615;
HG326639;Lepidoptera;Phengaris arion;NA;0.021;NA;0.793579063388615;
HG326640;Lepidoptera;Phengaris arion;NA;0.021;NA;0.791620191442083;
HG326641;Lepidoptera;Phengaris arion;NA;0.002;NA;0.801921507536285;
HG326642;Lepidoptera;Phengaris arion;NA;0.021;NA;0.791620191442083;
HM116932;Lepidoptera;Polyommatus eros;NA;NA;NA;0.999998891555751;
HM116935;Lepidoptera;Polyommatus icarus;NA;0.001;NA;0.999999549662548;
HM116936;Lepidoptera;Polyommatus icarus;Polyommatus icarus;0.002;Polyommatus icarus;0.809019347212362;
HM116937;Lepidoptera;Polyommatus icarus;Polyommatus icarus;0.001;Polyommatus icarus;0.973614065192442;
HM116939;Lepidoptera;Polyommatus icarus;NA;NA;NA;0.999999509666107;
HM116940;Lepidoptera;Polyommatus icarus;NA;0.001;Polyommatus icarus;0.748264214277749;
HM159431;Lepidoptera;Plebejus argyrognomon;NA;0.001;NA;0.996739256512481;
HM210169;Lepidoptera;Polyommatus humedasae;Polyommatus humedasae;1;NA;0.946645845760546;
HM386883;Lepidoptera;Boloria thore;Boloria thore;1;Boloria thore;0.999999998160007;
HM386884;Lepidoptera;Boloria thore;Boloria thore;1;Boloria thore;0.99999999245631;
HM386892;Lepidoptera;Pyrgus andromedae;Pyrgus andromedae;1;Pyrgus andromedae;0.999854960792946;
HM391781;Lepidoptera;Scolitantides orion;Scolitantides orion;1;Scolitantides orion;0.999999988125126;
HM391783;Lepidoptera;Polyommatus icarus;NA;0.001;NA;0.999994270558956;
HM391816;Lepidoptera;Anthocharis cardamines;Anthocharis cardamines;1;Anthocharis cardamines;0.999999871062621;
HM391817;Lepidoptera;Colias palaeno;NA;0.001;NA;0.999999999982869;
HM391818;Lepidoptera;Apatura ilia;NA;0.165;Apatura ilia;0.956796812031481;
HM391819;Lepidoptera;Apatura ilia;NA;0.133;Apatura ilia;0.891986785422262;
HM391821;Lepidoptera;Boloria selene;NA;0.253;Boloria selene;0.999999785347507;
HM391822;Lepidoptera;Boloria titania;Boloria titania;0.001;Boloria titania;0.99983358374111;
HM391827;Lepidoptera;Maniola jurtina;NA;0.174;Maniola jurtina;0.716224233181708;
HM391828;Lepidoptera;Coenonympha pamphilus;Coenonympha pamphilus;1;Coenonympha pamphilus;0.999999999999744;
HM391830;Lepidoptera;Coenonympha glycerion;NA;0.153;Coenonympha glycerion;0.999998464013956;
HM391831;Lepidoptera;Pararge aegeria;Pararge aegeria;1;Pararge aegeria;0.999999999963165;
HM391832;Lepidoptera;Pararge aegeria;Pararge aegeria;1;Pararge aegeria;0.999999999963165;
HM391834;Lepidoptera;Lycaena phlaeas;Lycaena phlaeas;1;Lycaena phlaeas;0.999999967867325;
HM391835;Lepidoptera;Lycaena tityrus;Lycaena tityrus;1;Lycaena tityrus;0.999999375548977;
HM391836;Lepidoptera;Plebejus argus;NA;0.045;Plebejus argus;0.989189327190115;
HM391837;Lepidoptera;Plebejus argus;NA;0.106;Plebejus argus;0.979424269542147;
HM391838;Lepidoptera;Ochlodes sylvanus;NA;0.316;Ochlodes sylvanus;0.996318754838625;
HM391840;Lepidoptera;Erynnis tages;Erynnis tages;1;Erynnis tages;0.999999281987018;
HM391841;Lepidoptera;Thymelicus acteon;Thymelicus acteon;1;Thymelicus acteon;0.99999999752248;

HM391842;Lepidoptera;Thymelicus lineola;Thymelicus lineola;1;Thymelicus lineola;0.999952218376157;
HM393172;Lepidoptera;Parnassius apollo;Parnassius apollo;1;Parnassius apollo;0.992704703916692;
HM393173;Lepidoptera;Parnassius apollo;Parnassius apollo;1;Parnassius apollo;0.992704703916692;
HM393174;Lepidoptera;Parnassius apollo;Parnassius apollo;1;Parnassius apollo;0.992704703916692;
HM393176;Lepidoptera;Colias crocea;NA;0.059;NA;0.999999999999738;
HM393177;Lepidoptera;Colias alfacariensis;Colias alfacariensis;1;Colias alfacariensis;0.999944653442302;
HM393178;Lepidoptera;Colias phicomone;NA;0.001;Colias phicomone;0.985598879697681;
HM393180;Lepidoptera;Aporia crataegi;NA;0.003;Aporia crataegi;0.999998466875677;
HM393184;Lepidoptera;Gonepteryx rhamni;NA;0.192;Gonepteryx rhamni;0.991002175992451;
HM393185;Lepidoptera;Lysandra coridon;NA;NA;NA;0.998070184737648;
HM393186;Lepidoptera;Phengaris arion;NA;0.054;Phengaris arion;0.938397095316026;
HM393189;Lepidoptera;Polyommatus amandus;Polyommatus amandus;0.003;Polyommatus amandus;0.999980071833264;
HM393190;Lepidoptera;Cupido minimus;NA;0.006;Cupido minimus;0.999768371759258;
HM393191;Lepidoptera;Lycaena virgaureae;NA;0.186;Lycaena virgaureae;0.999491227342155;
HM393192;Lepidoptera;Lycaena tityrus;Lycaena tityrus;1;Lycaena tityrus;0.999998928238034;
HM393193;Lepidoptera;Favonius quercus;NA;NA;Favonius quercus;0.73046069582187;
HM393194;Lepidoptera;Favonius quercus;NA;NA;Favonius quercus;0.850630352400423;
HM393195;Lepidoptera;Favonius quercus;NA;NA;Favonius quercus;0.73046069582187;
HM393196;Lepidoptera;Melanargia galathea;Melanargia galathea;NA;Melanargia galathea;0.970417110336134;
HM393197;Lepidoptera;Pararge aegeria;Pararge aegeria;1;Pararge aegeria;0.999999999963165;
HM393198;Lepidoptera;Pararge aegeria;Pararge aegeria;1;Pararge aegeria;0.999999997581313;
HM393199;Lepidoptera;Aphantopus hyperantus;Aphantopus hyperantus;0.009;Aphantopus hyperantus;0.999523934516809;
HM393200;Lepidoptera;Aphantopus hyperantus;Aphantopus hyperantus;0.006;Aphantopus hyperantus;0.999998615504572;
HM393201;Lepidoptera;Aphantopus hyperantus;Aphantopus hyperantus;0.01;Aphantopus hyperantus;0.999997523774024;
HM393203;Lepidoptera;Coenonympha arcania;NA;0.067;Coenonympha arcania;0.978811371852141;
HM393204;Lepidoptera;Erebia medusa;NA;0.009;Erebia medusa;0.986275612856827;
HM393205;Lepidoptera;Erebia medusa;NA;0.009;Erebia medusa;0.986275612856827;
HM393206;Lepidoptera;Erebia pronoe;Erebia pronoe;1;Erebia pronoe;0.999045603074731;
HM393207;Lepidoptera;Thymelicus sylvestris;NA;0.324;Thymelicus sylvestris;0.999940778442454;
HM393209;Lepidoptera;Carterocephalus palaemon;NA;0.052;Carterocephalus palaemon;0.728445051795325;
HM393210;Lepidoptera;Pyrgus malvae;Pyrgus malvae;NA;Pyrgus malvae;0.980121140374433;
HM393211;Lepidoptera;Euphydryas cynthia;Euphydryas cynthia;1;Euphydryas cynthia;0.999999999707427;
HM393212;Lepidoptera;Brenthis ino;Brenthis ino;0.002;Brenthis ino;0.994632032669749;
HM393213;Lepidoptera;Boloria selene;NA;0.275;Boloria selene;0.999999702697396;
HM393214;Lepidoptera;Boloria titania;Boloria titania;0.001;Boloria titania;0.99983358374111;
HM393216;Lepidoptera;Melitaea diamina;NA;0.305;Melitaea diamina;0.999999994065661;
HM393217;Lepidoptera;Issoria lathonia;Issoria lathonia;1;Issoria lathonia;0.999999999989257;
HM393220;Lepidoptera;Fabriciana niobe;NA;0.005;Fabriciana niobe;0.992125835358755;
HM393222;Lepidoptera;Nymphalis antiopa;NA;0.23;Nymphalis antiopa;0.999999944198096;
HM393225;Lepidoptera;Vanessa atalanta;Vanessa atalanta;1;Vanessa atalanta;0.999999999757762;
HM393226;Lepidoptera;Apatura iris;Apatura iris;1;Apatura iris;0.999999999999972;
HM393227;Lepidoptera;Apatura iris;Apatura iris;1;Apatura iris;0.999999999999972;
HM393228;Lepidoptera;Apatura iris;Apatura iris;1;Apatura iris;0.99999999999946;
HM393229;Lepidoptera;Apatura ilia;NA;0.165;Apatura ilia;0.956796812031481;
HM393232;Lepidoptera;Cyaniris semiargus;NA;NA;Cyaniris semiargus;0.999051841766953;
HM396370;Lepidoptera;Erebia pandrose;Erebia pandrose;0.998;Erebia pandrose;0.997117103851311;
HM396374;Lepidoptera;Aporia crataegi;NA;NA;Aporia crataegi;0.999996899001592;
HM400990;Lepidoptera;Pyrgus sidae;Pyrgus sidae;1;Pyrgus sidae;0.999995233751538;
HM400991;Lepidoptera;Pyrgus armoricanus;Pyrgus armoricanus;1;Pyrgus armoricanus;0.997335970003131;
HM400995;Lepidoptera;Agriades glandon;NA;0.026;Agriades glandon;0.973567310186356;
HM400996;Lepidoptera;Agriades glandon;NA;0.004;Agriades glandon;0.972363327373725;
HM400997;Lepidoptera;Agriades glandon;NA;NA;Agriades glandon;0.986958609491942;
HM401000;Lepidoptera;Agriades glandon;NA;0.001;Agriades glandon;0.978966919354654;
HM401001;Lepidoptera;Agriades glandon;NA;0.019;Agriades glandon;0.971626349703295;
HM401002;Lepidoptera;Agriades glandon;Agriades glandon;0.019;Agriades glandon;0.990080177943521;
HM401003;Lepidoptera;Aricia nicias;Aricia nicias;0.005;Aricia nicias;0.999451683966392;
HM401005;Lepidoptera;Aricia nicias;Aricia nicias;0.005;Aricia nicias;0.999451683966392;
HM401006;Lepidoptera;Aricia nicias;Aricia nicias;0.003;Aricia nicias;0.99891072569394;
HM401007;Lepidoptera;Aricia nicias;Aricia nicias;0.006;Aricia nicias;0.999121647973837;
HM401008;Lepidoptera;Aricia nicias;Aricia nicias;0.008;Aricia nicias;0.999405599877345;
HM425834;Lepidoptera;Erebia pluto;Erebia pluto;1;Erebia pluto;0.999999988858747;
HM425835;Lepidoptera;Erebia pluto;Erebia pluto;1;Erebia pluto;0.999999988858747;
HM425836;Lepidoptera;Erebia pandrose;Erebia pandrose;NA;Erebia pandrose;0.956509272964271;
HM425867;Lepidoptera;Erebia epiphron;NA;NA;Erebia epiphron;0.994401488812176;
HM425868;Lepidoptera;Polyommatus eros;NA;0.002;Polyommatus eros;0.966901467598208;
HM425889;Lepidoptera;Erebia epiphron;Erebia epiphron;NA;Erebia epiphron;0.998243429957028;
HM425891;Lepidoptera;Erebia pandrose;Erebia pandrose;0.005;Erebia pandrose;0.976530044823327;
HM425892;Lepidoptera;Agriades glandon;Agriades glandon;0.998;Agriades glandon;0.939962083086514;

HM425911;Lepidoptera;Oeneis glacialis;Oeneis glacialis;NA;Oeneis glacialis;0.974851412684016;
HM425927;Lepidoptera;Erebia epiphron;Erebia epiphron;NA;Erebia epiphron;0.995580956478864;
HM425931;Lepidoptera;Melitaea varia;Melitaea varia;1;Melitaea varia;0.999999652249276;
HM425936;Lepidoptera;Colias phicomone;NA;0.001;Colias phicomone;0.985598879697681;
HM425943;Lepidoptera;Erebia pluto;Erebia pluto;1;Erebia pluto;0.999999984692352;
HM425944;Lepidoptera;Erebia pluto;Erebia pluto;1;Erebia pluto;0.999999976671887;
HM425945;Lepidoptera;Polyommatus damon;Polyommatus damon;1;Polyommatus damon;0.999999882210126;
HM425953;Lepidoptera;Thymelicus acteon;Thymelicus acteon;1;Thymelicus acteon;0.99999999752248;
HM425954;Lepidoptera;Nymphalis c-album;NA;0.215;NA;0.999999192918557;
HM426042;Lepidoptera;Coenonympha glycerion;NA;0.154;Coenonympha glycerion;0.999998359070115;
HM426043;Lepidoptera;Coenonympha glycerion;NA;0.28;Coenonympha glycerion;0.999998036263913;
HM426044;Lepidoptera;Coenonympha gardetta;NA;0.031;Coenonympha gardetta;0.700970820226565;
HM426045;Lepidoptera;Lycaena tityrus;Lycaena tityrus;1;Lycaena tityrus;0.999995729087172;
HM426046;Lepidoptera;Thymelicus lineola;Thymelicus lineola;1;Thymelicus lineola;0.999995927238524;
HM426082;Lepidoptera;Boloria titania;Boloria titania;1;Boloria titania;0.999855929452882;
HM426083;Lepidoptera;Melitaea diamina;NA;0.149;Melitaea diamina;0.999999996592464;
HM426102;Lepidoptera;Boloria titania;Boloria titania;0.001;Boloria titania;0.99983358374111;
HM426105;Lepidoptera;Coenonympha gardetta;NA;0.001;Coenonympha gardetta;0.755947323088537;
HM426106;Lepidoptera;Coenonympha gardetta;NA;0.001;Coenonympha gardetta;0.755947323088537;
HM870914;Lepidoptera;Favonius quercus;NA;NA;Favonius quercus;0.73046069582187;
HM870926;Lepidoptera;Pyrgus malvae;Pyrgus malvae;NA;Pyrgus malvae;0.988055481360344;
HM870947;Lepidoptera;Lycaena virgaureae;Lycaena virgaureae;0.048;Lycaena virgaureae;0.999409200513189;
HM870974;Lepidoptera;Apatura iris;Apatura iris;1;Apatura iris;0.999999999999972;
HM870988;Lepidoptera;Aglais urticae;Aglais urticae;0.01;Aglais urticae;0.991470569735649;
HM870989;Lepidoptera;Aglais urticae;NA;0.002;Aglais urticae;0.987497593219019;
HM870991;Lepidoptera;Nymphalis c-album;NA;0.215;NA;0.999999192918557;
HM870992;Lepidoptera;Nymphalis c-album;NA;0.2;NA;0.99999889990099;
HM870993;Lepidoptera;Callophrys rubi;NA;0.003;Callophrys rubi;0.997328639918919;
HM871030;Lepidoptera;Celastrina argiolus;Celastrina argiolus;1;Celastrina argiolus;0.999708846309046;
HM871035;Lepidoptera;Anthocharis cardamines;Anthocharis cardamines;1;Anthocharis cardamines;0.999999940675226;
HM871037;Lepidoptera;Anthocharis cardamines;Anthocharis cardamines;1;Anthocharis cardamines;0.999999843941806;
HM871043;Lepidoptera;Pieris napi;NA;NA;Pieris napi;0.929162374195885;
HM871044;Lepidoptera;Carterocephalus palaemon;Carterocephalus palaemon;0.016;Carterocephalus palaemon;0.930544560040029;
HM871046;Lepidoptera;Pieris napi;NA;NA;Pieris napi;0.970569233032173;
HM871158;Lepidoptera;Papilio machaon;Papilio machaon;0.003;Papilio machaon;0.997288237409893;
HM871218;Lepidoptera;Melitaea cinxia;Melitaea cinxia;0.02;Melitaea cinxia;0.999999999999432;
HM871219;Lepidoptera;Pyrgus malvae;Pyrgus malvae;NA;Pyrgus malvae;0.980121140374433;
HM871230;Lepidoptera;Favonius quercus;NA;NA;Favonius quercus;0.73046069582187;
HM871271;Lepidoptera;Gonepteryx rhamni;NA;0.474;Gonepteryx rhamni;0.982076396127858;
HM871277;Lepidoptera;Vanessa cardui;Vanessa cardui;1;Vanessa cardui;0.999999547500397;
HM871278;Lepidoptera;Vanessa atalanta;Vanessa atalanta;1;Vanessa atalanta;0.999999999790276;
HM871746;Lepidoptera;Ochlodes sylvanus;NA;0.332;Ochlodes sylvanus;0.995966031721723;
HM871747;Lepidoptera;Ochlodes sylvanus;NA;NA;Ochlodes sylvanus;0.997133711363483;
HM871867;Lepidoptera;Polyommatus amandus;Polyommatus amandus;0.003;Polyommatus amandus;0.999980071833264;
HM871879;Lepidoptera;Aricia eumedon;Eumedonia eumedon;0.001;Eumedonia eumedon;0.997942473254099;
HM871895;Lepidoptera;Aphantopus hyperantus;Aphantopus hyperantus;0.003;Aphantopus hyperantus;0.999999455871523;
HM871925;Lepidoptera;Plebejus argus;NA;0.114;Plebejus argus;0.984076165564578;
HM871926;Lepidoptera;Plebejus idas;NA;0.002;NA;0.999633477464423;
HM871927;Lepidoptera;Plebejus idas;NA;0.007;NA;0.999546324331549;
HM871929;Lepidoptera;Agriades optilete;Agriades optilete;1;Agriades optilete;0.99999999183251;
HM871930;Lepidoptera;Agriades optilete;Agriades optilete;1;Agriades optilete;0.99999999183251;
HM871931;Lepidoptera;Plebejus idas;NA;0.007;NA;0.999546324331549;
HM871932;Lepidoptera;Aricia artaxerxes;Plebejus argus;1;Aricia artaxerxes;0.999743187199922;
HM871933;Lepidoptera;Polyommatus amandus;Polyommatus amandus;0.003;Polyommatus amandus;0.999980071833264;
HM871937;Lepidoptera;Brenthis ino;Brenthis ino;0.001;Brenthis ino;0.994711912827522;
HM871938;Lepidoptera;Brenthis ino;Brenthis ino;0.998;Brenthis ino;0.992517169157773;
HM871939;Lepidoptera;Argynnis aglaja;NA;NA;NA;0.999999999998051;
HM872111;Lepidoptera;Lycaena phlaeas;Lycaena phlaeas;1;Lycaena phlaeas;0.999999996363186;
HM872188;Lepidoptera;Glaucopsyche alexis;NA;NA;Glaucopsyche alexis;0.979381169624069;
HM872343;Lepidoptera;Glaucopsyche alexis;NA;NA;Glaucopsyche alexis;0.979381169624069;
HM872811;Lepidoptera;Satyrium w-album;NA;0.122;Satyrium w-album;0.97166364363414;
HM872822;Lepidoptera;Callophrys rubi;NA;0.003;Callophrys rubi;0.997328639918919;
HM872835;Lepidoptera;Papilio machaon;Papilio machaon;NA;Papilio machaon;0.998259418780604;
HM872901;Lepidoptera;Papilio machaon;Papilio machaon;NA;Papilio machaon;0.998428074612506;
HM872932;Lepidoptera;Lycaena tityrus;Lycaena tityrus;1;Lycaena tityrus;0.999998928238034;
HM872936;Lepidoptera;Euphydryas aurinia;NA;0.001;Euphydryas aurinia;0.995576749826865;
HM872958;Lepidoptera;Apatura iris;Apatura iris;1;Apatura iris;0.999999999999972;
HM872960;Lepidoptera;Apatura ilia;Apatura ilia;NA;Apatura ilia;0.988792300608896;
HM873003;Lepidoptera;Araschnia levana;Araschnia levana;1;Araschnia levana;0.999999772187832;

HM873004;Lepidoptera;Araschnia levana;Araschnia levana;1;Araschnia levana;0.999999772187832;
HM873036;Lepidoptera;Colias hyale;NA;NA;Colias hyale;0.999975737287978;
HM873051;Lepidoptera;Thecla betulae;Thecla betulae;1;Thecla betulae;0.999999999961119;
HM873110;Lepidoptera;Coenonympha glycerion;NA;0.295;Coenonympha glycerion;0.999998958157621;
HM873189;Lepidoptera;Erebia pandrose;Erebia pandrose;0.998;Erebia pandrose;0.997117103851311;
HM873260;Lepidoptera;Boloria euphrosyne;Boloria euphrosyne;1;Boloria euphrosyne;0.999989902710144;
HM873306;Lepidoptera;Argynnis aglaja;NA;NA;NA;0.999999999998051;
HM873358;Lepidoptera;Lasiommata maera;Lasiommata maera;1;Lasiommata maera;0.999999181072746;
HM873490;Lepidoptera;Pyrgus alveus;NA;NA;Pyrgus alveus;0.922887984165083;
HM873544;Lepidoptera;Maniola jurtina;NA;NA;NA;0.99999999991;
HM873568;Lepidoptera;Hipparchia semele;NA;0.002;Hipparchia semele;0.81627604446986;
HM873571;Lepidoptera;Maniola jurtina;NA;0.001;Maniola jurtina;0.939220105860875;
HM873572;Lepidoptera;Maniola jurtina;NA;0.038;NA;0.999999999999524;
HM873620;Lepidoptera;Gonepteryx rhamni;NA;0.474;Gonepteryx rhamni;0.982076396127858;
HM874159;Lepidoptera;Colias croceus;NA;0.059;NA;0.999999999999738;
HM874490;Lepidoptera;Plebejus argus;NA;0.328;Plebejus argus;0.969789734199889;
HM874491;Lepidoptera;Aricia artaxerxes;Plebejus argus;1;Aricia artaxerxes;0.999743187199922;
HM874569;Lepidoptera;Coenonympha glycerion;NA;0.264;Coenonympha glycerion;0.999998370144304;
HM874610;Lepidoptera;Coenonympha pamphilus;Coenonympha pamphilus;1;Coenonympha pamphilus;0.999999999999858;
HM874632;Lepidoptera;Lycaena phlaeas;Lycaena phlaeas;1;Lycaena phlaeas;0.999999996363186;
HM874652;Lepidoptera;Carterocephalus palaemon;NA;0.052;Carterocephalus palaemon;0.728445051795325;
HM874653;Lepidoptera;Carterocephalus palaemon;NA;0.052;Carterocephalus palaemon;0.728445051795325;
HM874822;Lepidoptera;Helleia helle;Lycaena helle;1;Lycaena helle;0.999999993533947;
HM874823;Lepidoptera;Helleia helle;Lycaena helle;1;Lycaena helle;0.999999993533947;
HM874824;Lepidoptera;Cupido minimus;NA;0.009;Cupido minimus;0.999791453712643;
HM874825;Lepidoptera;Cupido minimus;NA;0.004;Cupido minimus;0.999720131986281;
HM874826;Lepidoptera;Cupido argiades;NA;0.499;NA;0.999999999984682;
HM874827;Lepidoptera;Cupido argiades;NA;0.499;NA;0.999999999984682;
HM875785;Lepidoptera;Issoria lathonia;Issoria lathonia;1;Issoria lathonia;0.999999999981071;
HM875813;Lepidoptera;Vanessa cardui;Vanessa cardui;1;Vanessa cardui;0.999999803450484;
HM875908;Lepidoptera;Aricia eumedon;Eumedonia eumedon;0.001;Eumedonia eumedon;0.997532320863849;
HM875917;Lepidoptera;Araschnia levana;Araschnia levana;1;Araschnia levana;0.999999772187832;
HM875940;Lepidoptera;Vanessa atalanta;Vanessa atalanta;1;Vanessa atalanta;0.999999999757762;
HM876030;Lepidoptera;Issoria lathonia;Issoria lathonia;1;Issoria lathonia;0.999999999989257;
HM876033;Lepidoptera;Coenonympha pamphilus;Coenonympha pamphilus;1;Coenonympha pamphilus;0.999999999999716;
HM876061;Lepidoptera;Fabriciana adippe;Fabriciana adippe;0.028;Fabriciana adippe;0.969769198942097;
HM876062;Lepidoptera;Coenonympha glycerion;NA;0.194;Coenonympha glycerion;0.999999237873431;
HM876085;Lepidoptera;Thecla betulae;Thecla betulae;1;Thecla betulae;0.999999999977774;
HM876171;Lepidoptera;Erebia pandrose;Erebia pandrose;0.998;Erebia pandrose;0.997117103851311;
HM876206;Lepidoptera;Lycaena virgaureae;Lycaena virgaureae;0.027;Lycaena virgaureae;0.999582921361102;
HM876209;Lepidoptera;Thymelicus lineola;Thymelicus lineola;1;Thymelicus lineola;0.999971535093703;
HM901207;Lepidoptera;Carcharodus lavatherae;Carcharodus lavatherae;1;Carcharodus lavatherae;0.999975293871709;
HM901208;Lepidoptera;Pyrgus onopordi;Pyrgus onopordi;1;Pyrgus onopordi;0.999997111229755;
HM901209;Lepidoptera;Aporia crataegi;NA;NA;Aporia crataegi;0.999996781126733;
HM901221;Lepidoptera;Melitaea didyma;Melitaea didyma;1;Melitaea didyma;0.998043552633492;
HM901224;Lepidoptera;Hipparchia hermione;Hipparchia hermione;1;Hipparchia hermione;0.999892638915927;
HM901225;Lepidoptera;Hipparchia hermione;Hipparchia hermione;1;Hipparchia hermione;0.999900116531443;
HM901227;Lepidoptera;Carcharodus lavatherae;Carcharodus lavatherae;1;Carcharodus lavatherae;0.999975293871709;
HM901228;Lepidoptera;Pyrgus onopordi;Pyrgus onopordi;1;Pyrgus onopordi;0.999997111229755;
HM901229;Lepidoptera;Pyrgus onopordi;Pyrgus onopordi;1;Pyrgus onopordi;0.999997111229755;
HM901230;Lepidoptera;Pyrgus onopordi;Pyrgus onopordi;1;Pyrgus onopordi;0.999997111229755;
HM901232;Lepidoptera;Maniola cecilia;Pyronia cecilia;NA;Pyronia cecilia;1;
HM901233;Lepidoptera;Hipparchia fidia;Hipparchia fidia;1;Hipparchia fidia;1;
HM901234;Lepidoptera;Favonius quercus;NA;0.19;Favonius quercus;0.798566809308261;
HM901235;Lepidoptera;Melitaea parthenoides;Melitaea parthenoides;1;Melitaea parthenoides;0.999999956653455;
HM901236;Lepidoptera;Melitaea parthenoides;Melitaea parthenoides;1;Melitaea parthenoides;0.999999980399821;
HM901238;Lepidoptera;Coenonympha dorus;Coenonympha dorus;1;Coenonympha dorus;0.99999999988259;
HM901239;Lepidoptera;Glaucopsyche alexis;Glaucopsyche alexis;NA;Glaucopsyche alexis;0.989960894737762;
HM901240;Lepidoptera;Argynnis aglaja;NA;NA;Speyeria aglaja;0.785160490959768;
HM901244;Lepidoptera;Erebia pronoe;Erebia pronoe;1;Erebia pronoe;0.99980482908169;
HM901246;Lepidoptera;Scolitantides orion;Scolitantides orion;1;Scolitantides orion;0.999999953757596;
HM901247;Lepidoptera;Erebia meolans;Erebia meolans;0.999;Erebia meolans;0.99980804531561;

HM901248;Lepidoptera;Glaucopsyche alexis;Glaucopsyche alexis;0.001;Glaucopsyche alexis;0.994667256195263;
HM901249;Lepidoptera;Melitaea didyma;Melitaea didyma;1;Melitaea didyma;0.989721238423191;
HM901250;Lepidoptera;Melitaea didyma;Melitaea didyma;0.015;Melitaea didyma;0.979252256105077;
HM901251;Lepidoptera;Melitaea didyma;Melitaea didyma;1;Melitaea didyma;0.989721238423191;
HM901252;Lepidoptera;Papilio machaon;Papilio machaon;NA;Papilio machaon;0.998489747694706;
HM901253;Lepidoptera;Thymelicus sylvestris;Thymelicus sylvestris;1;Thymelicus sylvestris;0.999999985935205;
HM901254;Lepidoptera;Erebia pronoe;Erebia pronoe;1;Erebia pronoe;0.99980482908169;
HM901256;Lepidoptera;Euphydryas aurinia;Euphydryas aurinia;0.02;Euphydryas aurinia;0.99668249939786;
HM901257;Lepidoptera;Nymphalis c-album;NA;0.215;NA;0.999999192918557;
HM901258;Lepidoptera;Erebia pronoe;Erebia pronoe;1;Erebia pronoe;0.99980482908169;
HM901259;Lepidoptera;Melitaea nevadensis;Melitaea celadussa;1;Melitaea celadussa;0.996894039568781;
HM901260;Lepidoptera;Erebia pronoe;Erebia pronoe;1;Erebia pronoe;0.99980482908169;
HM901261;Lepidoptera;Thymelicus sylvestris;Thymelicus sylvestris;1;Thymelicus sylvestris;0.999999985935205;
HM901266;Lepidoptera;Glaucopsyche alexis;NA;NA;Glaucopsyche alexis;0.991019528246159;
HM901267;Lepidoptera;Erebia pronoe;Erebia pronoe;1;Erebia pronoe;0.99980482908169;
HM901268;Lepidoptera;Polyommatus dorylas;Polyommatus dorylas;0.025;Polyommatus dorylas;0.973229109265438;
HM901269;Lepidoptera;Melitaea parthenoides;Melitaea parthenoides;1;Melitaea parthenoides;0.999999993530452;
HM901270;Lepidoptera;Melitaea didyma;Melitaea didyma;1;Melitaea didyma;0.987834979325531;
HM901271;Lepidoptera;Hipparchia fidia;Hipparchia fidia;1;Hipparchia fidia;1;
HM901272;Lepidoptera;Brenthis hecate;NA;0.068;Brenthis hecate;0.999053551498733;
HM901273;Lepidoptera;Brenthis hecate;NA;0.068;Brenthis hecate;0.999053551498733;
HM901274;Lepidoptera;Carcharodus alceae;Carcharodus alceae;0.999;Carcharodus alceae;0.999999996339511;
HM901277;Lepidoptera;Satyrium esculi;Satyrium esculi;1;Satyrium esculi;0.999999982056039;
HM901278;Lepidoptera;Erebia meolans;Erebia meolans;NA;Erebia meolans;0.999835953426128;
HM901279;Lepidoptera;Lycaena alciphron;Lycaena alciphron;1;Lycaena alciphron;0.999999822789549;
HM901280;Lepidoptera;Maniola cecilia;Pyronia cecilia;1;Pyronia cecilia;0.999999999999972;
HM901281;Lepidoptera;Melitaea deione;Melitaea deione;1;Melitaea deione;0.999999999420709;
HM901282;Lepidoptera;Melitaea deione;Melitaea deione;1;Melitaea deione;0.999999999467633;
HM901283;Lepidoptera;Melitaea didyma;Melitaea didyma;1;Melitaea didyma;0.998043552633492;
HM901284;Lepidoptera;Fabriciana niobe;NA;0.001;Fabriciana niobe;0.924679661444846;
HM901285;Lepidoptera;Lycaena alciphron;Lycaena alciphron;1;Lycaena alciphron;0.999999822789549;
HM901287;Lepidoptera;Brenthis hecate;NA;0.068;Brenthis hecate;0.999053551498733;
HM901288;Lepidoptera;Brenthis hecate;NA;0.068;Brenthis hecate;0.999053551498733;
HM901290;Lepidoptera;Nymphalis c-album;NA;0.215;NA;0.999999192918557;
HM901291;Lepidoptera;Brintesia circe;Brintesia circe;1;Brintesia circe;0.999999898349165;
HM901292;Lepidoptera;Melitaea didyma;Melitaea didyma;1;Melitaea didyma;0.996364124392483;
HM901293;Lepidoptera;Melitaea didyma;Melitaea didyma;1;Melitaea didyma;0.998043552633492;
HM901294;Lepidoptera;Cercyonis lupina;Hyponephele lupina;1;Hyponephele lupina;0.999999964177108;
HM901295;Lepidoptera;Favonius quercus;Favonius quercus;1;Favonius quercus;0.998960636885624;
HM901296;Lepidoptera;Favonius quercus;NA;NA;Favonius quercus;0.73046069582187;
HM901298;Lepidoptera;Cyaniris semiargus;NA;NA;Cyaniris semiargus;0.999051841766953;
HM901299;Lepidoptera;Cyaniris semiargus;NA;NA;Cyaniris semiargus;0.999051841766953;
HM901300;Lepidoptera;Melitaea didyma;Melitaea didyma;1;Melitaea didyma;0.996366765827355;
HM901301;Lepidoptera;Lycaena virgaureae;Lycaena virgaureae;0.025;Lycaena virgaureae;0.999833961481754;
HM901303;Lepidoptera;Erebia meolans;Erebia meolans;NA;Erebia meolans;0.99957934552327;
HM901304;Lepidoptera;Erebia meolans;Erebia meolans;NA;Erebia meolans;0.99957934552327;
HM901305;Lepidoptera;Polyommatus damon;Polyommatus damon;1;Polyommatus damon;0.999999650121315;
HM901309;Lepidoptera;Cercyonis lupina;Hyponephele lupina;1;Hyponephele lupina;0.999999987915601;
HM901310;Lepidoptera;Cercyonis lupina;Hyponephele lupina;1;Hyponephele lupina;0.999999969007007;
HM901311;Lepidoptera;Gegenes nostrodamus;Gegenes nostrodamus;1;Gegenes nostrodamus;0.999999996883645;
HM901312;Lepidoptera;Cupido osiris;Cupido osiris;1;Cupido osiris;0.999974988645513;
HM901313;Lepidoptera;Cupido osiris;Cupido osiris;1;Cupido osiris;0.999999944757265;
HM901314;Lepidoptera;Erebia epiphron;NA;NA;Erebia epiphron;0.950006471153786;
HM901315;Lepidoptera;Erebia epiphron;NA;NA;Erebia epiphron;0.950006471153786;
HM901317;Lepidoptera;Polyommatus damon;Polyommatus damon;1;Polyommatus damon;0.999999933777959;
HM901318;Lepidoptera;Erebia manto;Erebia manto;0.009;Erebia manto;0.999861807193394;
HM901320;Lepidoptera;Charaxes jasius;Charaxes jasius;NA;Charaxes jasius;0.985950745410897;
HM901324;Lepidoptera;Hipparchia fidia;Hipparchia fidia;1;Hipparchia fidia;1;
HM901325;Lepidoptera;Polyommatus daphnis;Polyommatus damon;1;Polyommatus damon;0.999999390653429;
HM901326;Lepidoptera;Polyommatus daphnis;NA;0.031;Polyommatus daphnis;0.989661132749926;
HM901327;Lepidoptera;Hipparchia fidia;Hipparchia fidia;1;Hipparchia fidia;0.999999999999886;
HM901334;Lepidoptera;Chazara briseis;Chazara briseis;1;Chazara briseis;0.999980393060755;
HM901340;Lepidoptera;Satyrium pruni;NA;0.076;Satyrium pruni;0.990322364021655;
HM901341;Lepidoptera;Satyrium pruni;NA;0.076;Satyrium pruni;0.990322364021655;
HM901342;Lepidoptera;Satyrium pruni;NA;0.076;Satyrium pruni;0.990322364021655;
HM901343;Lepidoptera;Satyrium pruni;NA;0.076;Satyrium pruni;0.990322364021655;
HM901344;Lepidoptera;Plebejus idas;NA;0.007;NA;0.999546324331549;

HM901345;Lepidoptera;Lycaena virgaureae;Lycaena virgaureae;0.009;Lycaena virgaureae;0.999860459836935;
HM901348;Lepidoptera;Gonepteryx cleopatra;Gonepteryx cleopatra;0.998;Gonepteryx cleopatra;0.999118636582266;
HM901349;Lepidoptera;Melitaea didyma;Melitaea didyma;NA;Melitaea didyma;0.980272836986251;
HM901350;Lepidoptera;Pyrgus onopordi;Pyrgus onopordi;1;Pyrgus onopordi;0.999997111229755;
HM901357;Lepidoptera;Erebia epiphron;NA;NA;Erebia epiphron;0.950006471153786;
HM901359;Lepidoptera;Pyrgus onopordi;Pyrgus onopordi;1;Pyrgus onopordi;0.999998677585716;
HM901360;Lepidoptera;Pyrgus armoricanus;Pyrgus armoricanus;1;Pyrgus armoricanus;0.999997353848817;
HM901361;Lepidoptera;Pyrgus armoricanus;Pyrgus armoricanus;1;Pyrgus armoricanus;0.999997353848817;
HM901362;Lepidoptera;Pyrgus serratulae;Pyrgus serratulae;1;Pyrgus serratulae;0.999999079739408;
HM901363;Lepidoptera;Pyrgus serratulae;Pyrgus serratulae;1;Pyrgus serratulae;0.999999877361184;
HM901364;Lepidoptera;Hamearis lucina;NA;NA;Hamearis lucina;0.999999999984141;
HM901366;Lepidoptera;Polyommatus eros;NA;0.005;Polyommatus eros;0.975783510541378;
HM901367;Lepidoptera;Gonepteryx rhamni;NA;0.474;Gonepteryx rhamni;0.982076396127858;
HM901370;Lepidoptera;Papilio machaon;Papilio machaon;0.001;Papilio machaon;0.999463129150422;
HM901373;Lepidoptera;Melitaea diamina;NA;0.149;Melitaea diamina;0.999999996592464;
HM901374;Lepidoptera;Melitaea diamina;NA;0.149;Melitaea diamina;0.999999996592464;
HM901375;Lepidoptera;Melitaea parthenoides;Melitaea parthenoides;1;Melitaea parthenoides;0.999999971905084;
HM901376;Lepidoptera;Hamearis lucina;NA;NA;Hamearis lucina;0.999999999984141;
HM901377;Lepidoptera;Aricia eumedon;Eumedonia eumedon;1;Eumedonia eumedon;0.994829162794983;
HM901378;Lepidoptera;Plebejus idas;NA;0.007;NA;0.999546324331549;
HM901380;Lepidoptera;Plebejus idas;NA;0.007;NA;0.999546324331549;
HM901381;Lepidoptera;Pyrgus serratulae;Pyrgus serratulae;1;Pyrgus serratulae;0.999999877361184;
HM901382;Lepidoptera;Plebejus idas;NA;0.001;NA;0.999590911470354;
HM901384;Lepidoptera;Gonepteryx rhamni;Gonepteryx rhamni;1;Gonepteryx rhamni;0.999850170258883;
HM901386;Lepidoptera;Carcharodus lavatherae;Carcharodus lavatherae;1;Carcharodus lavatherae;0.999975293871709;
HM901388;Lepidoptera;Scolitantides orion;Scolitantides orion;1;Scolitantides orion;0.999999953757596;
HM901389;Lepidoptera;Melitaea diamina;Melitaea diamina;0.022;Melitaea diamina;0.999999970612266;
HM901392;Lepidoptera;Gonepteryx rhamni;Gonepteryx rhamni;1;Gonepteryx rhamni;0.99964616790002;
HM901400;Lepidoptera;Brintesia circe;Brintesia circe;1;Brintesia circe;0.999999625395716;
HM901401;Lepidoptera;Fabriciana niobe;NA;0.035;Fabriciana niobe;0.949480222958778;
HM901402;Lepidoptera;Argynnis aglaja;NA;NA;Speyeria aglaja;0.785160490959768;
HM901403;Lepidoptera;Thymelicus sylvestris;NA;0.324;Thymelicus sylvestris;0.999940778442454;
HM901404;Lepidoptera;Thymelicus sylvestris;NA;0.324;Thymelicus sylvestris;0.999940778442454;
HM901405;Lepidoptera;Euphydryas aurinia;Euphydryas aurinia;0.02;Euphydryas aurinia;0.99668249939786;
HM901406;Lepidoptera;Carcharodus lavatherae;Carcharodus lavatherae;1;Carcharodus lavatherae;0.999975293871709;
HM901407;Lepidoptera;Pyrgus onopordi;Pyrgus onopordi;1;Pyrgus onopordi;0.999997111229755;
HM901408;Lepidoptera;Pyrgus onopordi;Pyrgus onopordi;1;Pyrgus onopordi;0.999997111229755;
HM901409;Lepidoptera;Cupido alcetas;NA;0.086;Cupido alcetas;0.896940516362126;
HM901410;Lepidoptera;Thecla betulae;Thecla betulae;1;Thecla betulae;0.999999999972886;
HM901411;Lepidoptera;Cercyonis lupina;Hyponephele lupina;1;Hyponephele lupina;0.999999989342968;
HM901414;Lepidoptera;Maniola cecilia;Pyronia cecilia;1;Pyronia cecilia;1;
HM901417;Lepidoptera;Fabriciana niobe;NA;0.035;Fabriciana niobe;0.949480222958778;
HM901419;Lepidoptera;Plebejus idas;NA;0.001;NA;0.999163905769696;
HM901420;Lepidoptera;Plebejus idas;NA;0.001;NA;0.999163905769696;
HM901421;Lepidoptera;Pyrgus onopordi;Pyrgus onopordi;1;Pyrgus onopordi;0.999989958084683;
HM901423;Lepidoptera;Favonius quercus;NA;NA;Favonius quercus;0.73046069582187;
HM901424;Lepidoptera;Aricia eumedon;Eumedonia eumedon;0.003;Eumedonia eumedon;0.998278637613531;
HM901425;Lepidoptera;Aricia eumedon;Eumedonia eumedon;0.001;Eumedonia eumedon;0.998288650724713;
HM901426;Lepidoptera;Gonepteryx rhamni;Gonepteryx rhamni;1;Gonepteryx rhamni;0.99964616790002;
HM901427;Lepidoptera;Polyommatus dorylas;Polyommatus dorylas;0.007;Polyommatus dorylas;0.987428194236461;
HM901428;Lepidoptera;Scolitantides orion;Scolitantides orion;NA;Scolitantides orion;0.99999985538032;
HM901429;Lepidoptera;Scolitantides orion;Scolitantides orion;1;Scolitantides orion;0.999999953757596;
HM901430;Lepidoptera;Lasiommata maera;Lasiommata maera;1;Lasiommata maera;0.999997945450226;
HM901431;Lepidoptera;Polyommatus dorylas;Polyommatus dorylas;0.025;Polyommatus dorylas;0.973229109265438;
HM901433;Lepidoptera;Maniola cecilia;Pyronia cecilia;1;Pyronia cecilia;1;
HM901434;Lepidoptera;Lasiommata maera;Lasiommata maera;1;Lasiommata maera;0.999997945450226;
HM901435;Lepidoptera;Lycaena virgaureae;NA;0.077;Lycaena virgaureae;0.999717833371003;
HM901436;Lepidoptera;Erebia meolans;Erebia meolans;NA;Erebia meolans;0.999885150395991;
HM901437;Lepidoptera;Erebia meolans;NA;NA;Erebia meolans;0.999492139374011;
HM901439;Lepidoptera;Melitaea diamina;NA;0.149;Melitaea diamina;0.999999996592464;
HM901441;Lepidoptera;Aricia eumedon;Eumedonia eumedon;0.002;Eumedonia eumedon;0.997589529763314;
HM901442;Lepidoptera;Aricia eumedon;Eumedonia eumedon;0.003;Eumedonia eumedon;0.998278637613531;
HM901444;Lepidoptera;Cupido alcetas;NA;0.068;Cupido alcetas;0.88988907876118;
HM901445;Lepidoptera;Erebia euryale;NA;NA;Erebia euryale;0.93493911427573;
HM901446;Lepidoptera;Polyommatus eros;NA;0.002;Polyommatus eros;0.987468418234804;

HM901447;Lepidoptera;Pyrgus serratulae;Pyrgus serratulae;1;Pyrgus serratulae;0.999999822628057;
HM901448;Lepidoptera;Erebia meolans;Erebia meolans;NA;Erebia meolans;0.999835953426128;
HM901449;Lepidoptera;Erebia euryale;NA;NA;Erebia euryale;0.963541074418532;
HM901450;Lepidoptera;Erebia euryale;NA;NA;Erebia euryale;0.963541074418532;
HM901451;Lepidoptera;Melitaea parthenoides;Melitaea parthenoides;1;Melitaea parthenoides;0.999999993530452;
HM901452;Lepidoptera;Melitaea diamina;Melitaea diamina;1;Melitaea diamina;0.999999955078466;
HM901454;Lepidoptera;Melitaea nevadensis;Melitaea celadussa;1;Melitaea celadussa;0.998596140127316;
HM901455;Lepidoptera;Melitaea deione;Melitaea deione;1;Melitaea deione;0.999999999978741;
HM901456;Lepidoptera;Melitaea deione;Melitaea deione;1;Melitaea deione;0.999999999985903;
HM901459;Lepidoptera;Satyrium esculi;Satyrium esculi;1;Satyrium esculi;0.999999957367948;
HM901461;Lepidoptera;Melitaea trivia;Melitaea trivia;1;Melitaea trivia;0.999999999977604;
HM901462;Lepidoptera;Callophrys avis;Callophrys avis;1;Callophrys avis;0.992335035373355;
HM901463;Lepidoptera;Melitaea parthenoides;Melitaea parthenoides;1;Melitaea parthenoides;0.999999993530452;
HM901464;Lepidoptera;Melitaea deione;Melitaea deione;1;Melitaea deione;0.999999999735309;
HM901465;Lepidoptera;Plebejus argus;Plebejus argus;1;Plebejus argus;0.996068373368906;
HM901466;Lepidoptera;Satyrium acaciae;Satyrium acaciae;1;Satyrium acaciae;0.99999991343697;
HM901468;Lepidoptera;Satyrium esculi;Satyrium esculi;1;Satyrium esculi;0.999999957367948;
HM901469;Lepidoptera;Satyrium acaciae;Satyrium acaciae;1;Satyrium acaciae;0.99999991343697;
HM901470;Lepidoptera;Melitaea nevadensis;Melitaea celadussa;1;Melitaea celadussa;0.998596140127316;
HM901471;Lepidoptera;Euphydryas aurinia;Euphydryas aurinia;0.02;Euphydryas aurinia;0.99668249939786;
HM901472;Lepidoptera;Satyrium acaciae;Satyrium acaciae;1;Satyrium acaciae;0.999999811388779;
HM901475;Lepidoptera;Melitaea parthenoides;Melitaea parthenoides;1;Melitaea parthenoides;0.999999993530452;
HM901478;Lepidoptera;Chazara briseis;Chazara briseis;1;Chazara briseis;0.999980393060755;
HM901481;Lepidoptera;Polyommatus escheri;Polyommatus escheri;1;Polyommatus escheri;0.999999989429227;
HM901485;Lepidoptera;Chazara briseis;Chazara briseis;1;Chazara briseis;0.999980393060755;
HM901486;Lepidoptera;Gegenes nostrodamus;Gegenes nostrodamus;1;Gegenes nostrodamus;0.999999996883645;
HM901487;Lepidoptera;Melitaea cinxia;Melitaea cinxia;1;Melitaea cinxia;0.999999999999659;
HM901490;Lepidoptera;Satyrium acaciae;Satyrium acaciae;1;Satyrium acaciae;0.99999991343697;
HM901491;Lepidoptera;Lycaena alciphron;Lycaena alciphron;1;Lycaena alciphron;0.99999971794291;
HM901492;Lepidoptera;Brintesia circe;Brintesia circe;NA;Brintesia circe;0.999999752169404;
HM901494;Lepidoptera;Satyrium esculi;Satyrium esculi;1;Satyrium esculi;0.999999957367948;
HM901495;Lepidoptera;Thymelicus lineola;Thymelicus lineola;1;Thymelicus lineola;0.999999096983497;
HM901497;Lepidoptera;Erebia euryale;NA;NA;Erebia euryale;0.93493911427573;
HM901499;Lepidoptera;Erebia epiphron;NA;NA;Erebia epiphron;0.950006471153786;
HM901502;Lepidoptera;Chazara briseis;Chazara briseis;1;Chazara briseis;0.999982402025926;
HM901503;Lepidoptera;Erebia neoridas;NA;NA;Erebia neoridas;0.994368810769763;
HM901504;Lepidoptera;Erebia neoridas;NA;NA;Erebia neoridas;0.994368810769763;
HM901505;Lepidoptera;Erebia oeme;NA;0.114;Erebia oeme;0.999999999830635;
HM901507;Lepidoptera;Callophrys rubi;NA;0.001;Callophrys rubi;0.99870518510598;
HM901508;Lepidoptera;Melitaea didyma;NA;NA;Melitaea didyma;0.981993619455166;
HM901509;Lepidoptera;Euphydryas aurinia;Euphydryas aurinia;0.017;Euphydryas aurinia;0.997183742560994;
HM901510;Lepidoptera;Euphydryas aurinia;Euphydryas aurinia;0.017;Euphydryas aurinia;0.997183742560994;
HM901511;Lepidoptera;Araschnia levana;Araschnia levana;1;Araschnia levana;0.999999772187832;
HM901512;Lepidoptera;Fabriciana adippe;Fabriciana adippe;0.001;Fabriciana adippe;0.999538788643547;
HM901513;Lepidoptera;Polyommatus damon;Polyommatus damon;1;Polyommatus damon;0.999999390653429;
HM901514;Lepidoptera;Coenonympha dorus;Coenonympha dorus;1;Coenonympha dorus;0.99999999988259;
HM901517;Lepidoptera;Polyommatus dorylas;Polyommatus dorylas;0.025;Polyommatus dorylas;0.973229109265438;
HM901518;Lepidoptera;Lasiommata maera;Lasiommata maera;1;Lasiommata maera;0.999997945450226;
HM901520;Lepidoptera;Erebia meolans;Erebia meolans;NA;Erebia meolans;0.999866655133868;
HM901521;Lepidoptera;Polyommatus dorylas;Polyommatus dorylas;0.025;Polyommatus dorylas;0.973229109265438;
HM901522;Lepidoptera;Maniola cecilia;Pyronia cecilia;1;Pyronia cecilia;1;
HM901523;Lepidoptera;Coenonympha arcania;NA;0.172;Coenonympha arcania;0.963036309473038;
HM901524;Lepidoptera;Polyommatus dorylas;Polyommatus dorylas;0.025;Polyommatus dorylas;0.973229109265438;
HM901529;Lepidoptera;Hipparchia hermione;Hipparchia hermione;1;Hipparchia hermione;0.999958624563657;
HM901530;Lepidoptera;Arethusana arethusa;Arethusana arethusa;1;Arethusana arethusa;0.999999801603556;
HM901531;Lepidoptera;Arethusana arethusa;Arethusana arethusa;1;Arethusana arethusa;0.999998574660502;
HM901532;Lepidoptera;Cupido minimus;NA;0.216;Cupido lorquinii;0.726278995315528;
HM901533;Lepidoptera;Cupido osiris;Cupido osiris;1;Cupido osiris;0.999999791352473;
HM901534;Lepidoptera;Cupido osiris;Cupido osiris;1;Cupido osiris;0.999999791352473;
HM901535;Lepidoptera;Cupido osiris;Cupido osiris;1;Cupido osiris;0.999999791352473;

HM901536;Lepidoptera;Cupido osiris;Cupido osiris;1;Cupido osiris;0.999999791352473;
HM901539;Lepidoptera;Melitaea nevadensis;Melitaea celadussa;0.997;Melitaea celadussa;0.996554884093156;
HM901543;Lepidoptera;Arethusana arethusa;Arethusana arethusa;1;Arethusana arethusa;0.999999801603556;
HM901544;Lepidoptera;Arethusana arethusa;Arethusana arethusa;1;Arethusana arethusa;0.999999801603556;
HM901546;Lepidoptera;Cupido minimus;NA;0.219;NA;0.999997407214946;
HM901547;Lepidoptera;Pyrgus armoricanus;Pyrgus armoricanus;0.999;Pyrgus armoricanus;0.999972901730941;
HM901552;Lepidoptera;Colias alfacariensis;Colias alfacariensis;0.001;Colias alfacariensis;0.999857790810452;
HM901553;Lepidoptera;Polyommatus celina;NA;0.072;Polyommatus celina;0.999999999953559;
HM901554;Lepidoptera;Erebia neoridas;NA;NA;Erebia neoridas;0.994368810769763;
HM901555;Lepidoptera;Erebia neoridas;NA;NA;Erebia neoridas;0.994368810769763;
HM901557;Lepidoptera;Colias alfacariensis;Colias alfacariensis;0.001;Colias alfacariensis;0.999857790810452;
HM901558;Lepidoptera;Polyommatus amandus;Polyommatus amandus;0.003;Polyommatus amandus;0.999980071833264;
HM901560;Lepidoptera;Hipparchia hermione;Hipparchia hermione;1;Hipparchia hermione;0.999929600232128;
HM901561;Lepidoptera;Thymelicus lineola;Thymelicus lineola;1;Thymelicus lineola;0.999998106572607;
HM901562;Lepidoptera;Aricia cramera;Aricia cramera;1;Aricia cramera;0.999993731037033;
HM901563;Lepidoptera;Aricia cramera;Aricia cramera;1;Aricia cramera;0.999993731037033;
HM901564;Lepidoptera;Coenonympha glycerion;NA;0.28;Coenonympha glycerion;0.999998036263913;
HM901565;Lepidoptera;Coenonympha glycerion;NA;0.28;Coenonympha glycerion;0.999998036263913;
HM901566;Lepidoptera;Colias phicomone;Colias phicomone;0.001;Colias phicomone;0.986802434025645;
HM901569;Lepidoptera;Coenonympha pamphilus;Coenonympha pamphilus;1;Coenonympha pamphilus;0.999999999981924;
HM901573;Lepidoptera;Thymelicus lineola;Thymelicus lineola;1;Thymelicus lineola;0.999998787978423;
HM901574;Lepidoptera;Thymelicus sylvestris;Thymelicus sylvestris;1;Thymelicus sylvestris;0.999999985935205;
HM901584;Lepidoptera;Polyommatus celina;Polyommatus celina;0.009;Polyommatus celina;0.999999999949694;
HM901588;Lepidoptera;Brenthis daphne;Brenthis daphne;0.001;Brenthis daphne;0.997781825264304;
HM901589;Lepidoptera;Brenthis daphne;Brenthis daphne;0.001;Brenthis daphne;0.997781825264304;
HM901591;Lepidoptera;Thymelicus lineola;Thymelicus lineola;1;Thymelicus lineola;0.999998126710004;
HM901594;Lepidoptera;Coenonympha pamphilus;Coenonympha pamphilus;1;Coenonympha pamphilus;0.99999999999946;
HM901595;Lepidoptera;Arethusana arethusa;Arethusana arethusa;1;Arethusana arethusa;0.999999785474722;
HM901596;Lepidoptera;Aphantopus hyperantus;Aphantopus hyperantus;0.003;Aphantopus hyperantus;0.999999455871523;
HM901597;Lepidoptera;Polyommatus escheri;Polyommatus escheri;1;Polyommatus escheri;0.9999999988855;
HM901605;Lepidoptera;Aglais urticae;Aglais urticae;0.004;Aglais urticae;0.995016750255472;
HM901607;Lepidoptera;Pararge aegeria;Pararge aegeria;1;Pararge aegeria;0.999999999963165;
HM901608;Lepidoptera;Aricia cramera;Aricia cramera;1;Aricia cramera;0.999993731037033;
HM901611;Lepidoptera;Aricia cramera;Aricia cramera;1;Aricia cramera;0.999993731037033;
HM901613;Lepidoptera;Hipparchia hermione;Hipparchia hermione;1;Hipparchia hermione;0.999958624563657;
HM901615;Lepidoptera;Colias alfacariensis;Colias alfacariensis;0.001;Colias alfacariensis;0.999857790810452;
HM901616;Lepidoptera;Colias alfacariensis;Colias alfacariensis;0.001;Colias alfacariensis;0.999857790810452;
HM901619;Lepidoptera;Brenthis daphne;Brenthis daphne;1;Brenthis daphne;0.997172799742872;
HM901620;Lepidoptera;Limenitis camilla;Limenitis camilla;0.009;Limenitis camilla;0.9999828626808;
HM901624;Lepidoptera;Aglais urticae;NA;0.002;Aglais urticae;0.987497593219019;
HM901626;Lepidoptera;Coenonympha glycerion;NA;0.156;Coenonympha glycerion;0.999998559347134;
HM901627;Lepidoptera;Coenonympha glycerion;NA;0.284;Coenonympha glycerion;0.999998676061348;
HM901628;Lepidoptera;Cyaniris semiargus;NA;NA;Cyaniris semiargus;0.999051841766953;
HM901629;Lepidoptera;Polyommatus escheri;Polyommatus escheri;1;Polyommatus escheri;0.999999998501636;
HM901630;Lepidoptera;Polyommatus escheri;Polyommatus escheri;1;Polyommatus escheri;0.999998281574049;
HM901634;Lepidoptera;Coenonympha glycerion;NA;0.157;Coenonympha glycerion;0.999999004304256;
HM901636;Lepidoptera;Brenthis daphne;Brenthis daphne;0.001;Brenthis daphne;0.997781825264304;
HM901637;Lepidoptera;Aglais urticae;NA;0.002;Aglais urticae;0.987497593219019;
HM901638;Lepidoptera;Pyrgus serratulae;Pyrgus serratulae;1;Pyrgus serratulae;0.999999694697398;
HM901639;Lepidoptera;Polyommatus escheri;Polyommatus escheri;1;Polyommatus escheri;0.999999335270609;
HM901640;Lepidoptera;Polyommatus escheri;Polyommatus escheri;1;Polyommatus escheri;0.999980529280732;

HM901642;Lepidoptera;Thymelicus lineola;Thymelicus lineola;1;Thymelicus lineola;0.999999096983497;
HM901643;Lepidoptera;Pyrgus serratulae;Pyrgus serratulae;1;Pyrgus serratulae;0.999999694697398;
HM901644;Lepidoptera;Aphantopus hyperantus;Aphantopus hyperantus;0.003;Aphantopus hyperantus;0.999999455871523;
HM901645;Lepidoptera;Coenonympha glycerion;NA;0.406;Coenonympha glycerion;0.99999824609387;
HM901646;Lepidoptera;Pararge aegeria;Pararge aegeria;1;Pararge aegeria;0.999999999365514;
HM901647;Lepidoptera;Polyommatus celina;Polyommatus celina;0.001;Polyommatus celina;0.999999999717033;
HM901648;Lepidoptera;Pararge aegeria;Pararge aegeria;1;Pararge aegeria;0.999999999365514;
HM901649;Lepidoptera;Polyommatus celina;Polyommatus celina;0.001;Polyommatus celina;0.999999999717033;
HM901650;Lepidoptera;Aphantopus hyperantus;Aphantopus hyperantus;0.006;Aphantopus hyperantus;0.999998615504572;
HM901651;Lepidoptera;Arethusana arethusa;Arethusana arethusa;0.003;Arethusana arethusa;0.999976174545094;
HM901652;Lepidoptera;Arethusana arethusa;Arethusana arethusa;0.001;Arethusana arethusa;0.999978196934222;
HM901660;Lepidoptera;Lysandra coridon;Lysandra coridon;NA;Lysandra coridon;0.878290303545515;
HM901661;Lepidoptera;Lysandra coridon;Lysandra coridon;NA;Lysandra coridon;0.892412957467058;
HM901665;Lepidoptera;Arethusana arethusa;NA;0.062;Arethusana arethusa;0.999962489703143;
HM901666;Lepidoptera;Pararge aegeria;Pararge aegeria;1;Pararge aegeria;0.999999999963165;
HM901667;Lepidoptera;Cupido minimus;NA;0.009;Cupido minimus;0.999791453712643;
HM901668;Lepidoptera;Cupido minimus;NA;0.009;Cupido minimus;0.999791453712643;
HM901671;Lepidoptera;Thymelicus lineola;Thymelicus lineola;1;Thymelicus lineola;0.999998231984392;
HM901673;Lepidoptera;Aphantopus hyperantus;Aphantopus hyperantus;0.006;Aphantopus hyperantus;0.999998615504572;
HM901674;Lepidoptera;Aphantopus hyperantus;Aphantopus hyperantus;0.006;Aphantopus hyperantus;0.999998615504572;
HM901677;Lepidoptera;Hipparchia hermione;Hipparchia hermione;1;Hipparchia hermione;0.999791304970896;
HM901682;Lepidoptera;Aphantopus hyperantus;Aphantopus hyperantus;0.003;Aphantopus hyperantus;0.99999864068927;
HM901686;Lepidoptera;Agriades glandon;NA;NA;Agriades glandon;0.986958609491942;
HM901687;Lepidoptera;Colias phicomone;Colias phicomone;0.001;Colias phicomone;0.986802434025645;
HM901688;Lepidoptera;Colias phicomone;Colias phicomone;0.001;Colias phicomone;0.986802434025645;
HM901697;Lepidoptera;Pararge aegeria;Pararge aegeria;1;Pararge aegeria;0.999999999963165;
HM901698;Lepidoptera;Coenonympha pamphilus;Coenonympha pamphilus;1;Coenonympha pamphilus;0.999999999999829;
HM901699;Lepidoptera;Cupido minimus;NA;0.009;Cupido minimus;0.999791453712643;
HM901700;Lepidoptera;Cupido minimus;NA;0.009;Cupido minimus;0.999791453712643;
HM901701;Lepidoptera;Limenitis camilla;Limenitis camilla;0.009;Limenitis camilla;0.9999828626808;
HM901702;Lepidoptera;Colias phicomone;Colias phicomone;0.001;Colias phicomone;0.986802434025645;
HM901703;Lepidoptera;Colias phicomone;Colias phicomone;0.001;Colias phicomone;0.986802434025645;
HM901704;Lepidoptera;Brenthis ino;Brenthis ino;0.001;Brenthis ino;0.993162660176952;
HM901710;Lepidoptera;Erebia pronoe;Erebia pronoe;1;Erebia pronoe;0.997847273843868;
HM901711;Lepidoptera;Erebia pronoe;Erebia pronoe;1;Erebia pronoe;0.997847273843868;
HM901712;Lepidoptera;Apatura ilia;NA;0.031;Apatura ilia;0.977379808055049;
HM901713;Lepidoptera;Apatura iris;Apatura iris;1;Apatura iris;0.999999999999972;
HM901714;Lepidoptera;Apatura ilia;Apatura ilia;NA;Apatura ilia;0.988792300608896;
HM901717;Lepidoptera;Colias phicomone;NA;0.064;Colias phicomone;0.992122721786916;
HM901718;Lepidoptera;Colias phicomone;NA;0.064;Colias phicomone;0.992122721786916;
HM901719;Lepidoptera;Boloria euphrosyne;Boloria euphrosyne;1;Boloria euphrosyne;0.999996593742825;
HM901720;Lepidoptera;Boloria euphrosyne;Boloria euphrosyne;1;Boloria euphrosyne;0.999996593742825;
HM901726;Lepidoptera;Fabriciana niobe;NA;0.035;Fabriciana niobe;0.949480222958778;
HM901734;Lepidoptera;Hipparchia hermione;Hipparchia hermione;1;Hipparchia hermione;0.999923234126642;
HM901740;Lepidoptera;Polyommatus escheri;Polyommatus escheri;1;Polyommatus escheri;0.999999997780122;
HM901744;Lepidoptera;Coenonympha pamphilus;Coenonympha pamphilus;1;Coenonympha pamphilus;0.999999999999744;
HM901746;Lepidoptera;Polyommatus amandus;Polyommatus amandus;0.003;Polyommatus amandus;0.999980071833264;
HM901747;Lepidoptera;Boloria euphrosyne;Boloria euphrosyne;0.008;Boloria euphrosyne;0.999964281413411;
HM901749;Lepidoptera;Limenitis camilla;Limenitis camilla;0.009;Limenitis camilla;0.9999828626808;
HM901750;Lepidoptera;Aphantopus hyperantus;Aphantopus hyperantus;0.003;Aphantopus hyperantus;0.999999455871523;
HM901751;Lepidoptera;Limenitis camilla;Limenitis camilla;0.009;Limenitis camilla;0.9999828626808;
HM901756;Lepidoptera;Brenthis ino;NA;0.061;Brenthis ino;0.791997215074462;
HM901757;Lepidoptera;Apatura iris;Apatura iris;1;Apatura iris;0.999999999999972;

HM901762;Lepidoptera;Polyommatus escheri;Polyommatus escheri;1;Polyommatus escheri;0.999999996684522;
HM901763;Lepidoptera;Polyommatus escheri;Polyommatus escheri;1;Polyommatus escheri;0.999999996299948;
HM901764;Lepidoptera;Fabriciana adippe;Fabriciana adippe;1;Fabriciana adippe;0.999486158851074;
HM901766;Lepidoptera;Satyrium esculi;Satyrium esculi;1;Satyrium esculi;0.999999957367948;
HM901767;Lepidoptera;Coenonympha glycerion;NA;0.28;Coenonympha glycerion;0.999998036263913;
HM901768;Lepidoptera;Brenthis ino;Brenthis ino;0.001;Brenthis ino;0.998434780405276;
HM901769;Lepidoptera;Coenonympha glycerion;NA;0.28;Coenonympha glycerion;0.999998036263913;
HM901770;Lepidoptera;Carcharodus lavatherae;Carcharodus lavatherae;1;Carcharodus lavatherae;0.999975293871709;
HM901771;Lepidoptera;Thymelicus sylvestris;Thymelicus sylvestris;1;Thymelicus sylvestris;0.999999985935205;
HM901773;Lepidoptera;Thymelicus sylvestris;Thymelicus sylvestris;1;Thymelicus sylvestris;0.999999985935205;
HM901778;Lepidoptera;Iolana iolas;Iolana iolas;1;Iolana iolas;0.999999158433083;
HM901779;Lepidoptera;Arethusana arethusa;NA;0.062;Arethusana arethusa;0.999962489703143;
HM901780;Lepidoptera;Pararge aegeria;Pararge aegeria;1;Pararge aegeria;0.999999775819728;
HM901781;Lepidoptera;Boloria euphrosyne;Boloria euphrosyne;0.003;Boloria euphrosyne;0.999970094877776;
HM901782;Lepidoptera;Boloria pales;Boloria pales;1;Boloria pales;0.99847060885702;
HM901783;Lepidoptera;Erebia pronoe;Erebia pronoe;1;Erebia pronoe;0.99980482908169;
HM901784;Lepidoptera;Erebia pronoe;Erebia pronoe;1;Erebia pronoe;0.999546444749081;
HM901785;Lepidoptera;Erebia pronoe;Erebia pronoe;1;Erebia pronoe;0.99980482908169;
HM901786;Lepidoptera;Erebia pronoe;Erebia pronoe;1;Erebia pronoe;0.99980482908169;
HM901787;Lepidoptera;Erebia pronoe;Erebia pronoe;1;Erebia pronoe;0.99980482908169;
HM901791;Lepidoptera;Lysandra coridon;Lysandra coridon;NA;NA;0.999914532655271;
HM901792;Lepidoptera;Arethusana arethusa;NA;0.062;Arethusana arethusa;0.999953862792797;
HM901793;Lepidoptera;Aglais urticae;Aglais urticae;1;Aglais urticae;0.995854684772509;
HM901795;Lepidoptera;Hipparchia hermione;Hipparchia hermione;1;Hipparchia hermione;0.999941679724737;
HM901799;Lepidoptera;Aglais urticae;NA;0.002;Aglais urticae;0.987497593219019;
HM901802;Lepidoptera;Brenthis ino;Brenthis ino;0.002;Brenthis ino;0.984749809551735;
HM901804;Lepidoptera;Carcharodus baeticus;Carcharodus baeticus;1;Carcharodus baeticus;0.999983899664537;
HM901805;Lepidoptera;Pyrgus serratulae;Pyrgus serratulae;1;Pyrgus serratulae;0.999999877361184;
HM901806;Lepidoptera;Pyrgus serratulae;Pyrgus serratulae;1;Pyrgus serratulae;0.999999877361184;
HM901809;Lepidoptera;Melitaea didyma;Melitaea didyma;NA;Melitaea didyma;0.980272836986251;
HM901810;Lepidoptera;Melitaea didyma;Melitaea didyma;NA;Melitaea didyma;0.980272836986251;
HM901811;Lepidoptera;Carcharodus baeticus;Carcharodus baeticus;1;Carcharodus baeticus;0.999956286218202;
HM901812;Lepidoptera;Erebia neoridas;NA;NA;Erebia neoridas;0.994368810769763;
HM901813;Lepidoptera;Erebia neoridas;NA;NA;Erebia neoridas;0.994368810769763;
HM901814;Lepidoptera;Erebia neoridas;NA;NA;Erebia neoridas;0.994368810769763;
HM901815;Lepidoptera;Erebia neoridas;NA;NA;Erebia neoridas;0.997677227800323;
HM901816;Lepidoptera;Erebia neoridas;NA;NA;Erebia neoridas;0.996460951256266;
HM901817;Lepidoptera;Colias phicomone;Colias phicomone;0.001;Colias phicomone;0.986802434025645;
HM901818;Lepidoptera;Colias phicomone;Colias phicomone;0.029;Colias phicomone;0.960754705901041;
HM901819;Lepidoptera;Colias phicomone;Colias phicomone;0.001;Colias phicomone;0.986802434025645;
HM901820;Lepidoptera;Melitaea parthenoides;Melitaea parthenoides;1;Melitaea parthenoides;0.999998904055136;
HM901821;Lepidoptera;Melitaea diamina;NA;0.149;Melitaea diamina;0.999999996592464;
HM901822;Lepidoptera;Coenonympha dorus;Coenonympha dorus;1;Coenonympha dorus;0.99999999988259;
HM901824;Lepidoptera;Carcharodus baeticus;Carcharodus baeticus;1;Carcharodus baeticus;0.999983899664537;
HM901825;Lepidoptera;Melitaea didyma;Melitaea didyma;1;Melitaea didyma;0.987834979325531;
HM901826;Lepidoptera;Chazara briseis;Chazara briseis;0.001;Chazara briseis;0.999986616075242;
HM901827;Lepidoptera;Charaxes jasius;Charaxes jasius;NA;Charaxes jasius;0.995860724722804;
HM901828;Lepidoptera;Charaxes jasius;Charaxes jasius;0.001;Charaxes jasius;0.997689374666591;
HM901829;Lepidoptera;Erebia oeme;NA;0.114;Erebia oeme;0.999999999830635;
HM901830;Lepidoptera;Erebia oeme;NA;0.12;Erebia oeme;0.999999999885944;
HM901833;Lepidoptera;Thymelicus sylvestris;NA;0.324;Thymelicus sylvestris;0.999940778442454;
HM901834;Lepidoptera;Melitaea parthenoides;Melitaea parthenoides;1;Melitaea parthenoides;0.999999426287269;
HM902067;Lepidoptera;Minois dryas;Minois dryas;0.007;Minois dryas;0.948207146728008;
HM902068;Lepidoptera;Brintesia circe;Brintesia circe;1;Brintesia circe;0.999998969116339;
HM902070;Lepidoptera;Erebia euryale;NA;NA;Erebia euryale;0.919518760623792;
HM903321;Lepidoptera;Aricia agestis;NA;NA;Aricia agestis;0.979669910324124;
HM904288;Lepidoptera;Melitaea didyma;Melitaea didyma;1;Melitaea didyma;0.999391232011314;
HM904290;Lepidoptera;Coenonympha pamphilus;Coenonympha pamphilus;1;Coenonympha pamphilus;0.999999999999744;
HM904291;Lepidoptera;Ochlodes sylvanus;NA;0.119;Ochlodes sylvanus;0.997373902608318;
HM904293;Lepidoptera;Coenonympha pamphilus;Coenonympha pamphilus;1;Coenonympha pamphilus;0.999999999997954;
HM904298;Lepidoptera;Vanessa cardui;Vanessa cardui;1;Vanessa cardui;0.999999459983318;
HM904299;Lepidoptera;Lysandra coridon;Lysandra coridon;NA;NA;0.999039865644651;
HM904301;Lepidoptera;Cupido argiades;NA;0.499;NA;0.999999999984682;
HM904305;Lepidoptera;Cupido alcetas;NA;0.207;Cupido alcetas;0.841248270372301;

HM904306;Lepidoptera;Cupido argiades;NA;NA;NA;0.999999999855364;
HM904308;Lepidoptera;Brintesia circe;Brintesia circe;1;Brintesia circe;0.99999951254154;
HM904309;Lepidoptera;Vanessa atalanta;Vanessa atalanta;1;Vanessa atalanta;0.999999999757762;
HM904310;Lepidoptera;Colias alfacariensis;Colias alfacariensis;0.001;Colias alfacariensis;0.999926026317526;
HM904312;Lepidoptera;Colias crocea;NA;NA;NA;0.999999999999491;
HM904315;Lepidoptera;Maniola jurtina;NA;0.038;NA;0.999999999999524;
HM904316;Lepidoptera;Pieris napi;NA;0.011;Pieris napi;0.801985394724814;
HM909999;Lepidoptera;Colias alfacariensis;Colias alfacariensis;1;Colias alfacariensis;0.999944653442302;
HM910000;Lepidoptera;Colias alfacariensis;Colias alfacariensis;1;Colias alfacariensis;0.999944653442302;
HM910001;Lepidoptera;Colias alfacariensis;Colias alfacariensis;1;Colias alfacariensis;0.999944653442302;
HM910002;Lepidoptera;Colias alfacariensis;Colias alfacariensis;1;Colias alfacariensis;0.999944653442302;
HM910004;Lepidoptera;Parnassius mnemosyne;Parnassius mnemosyne;1;Parnassius mnemosyne;0.999999688766982;
HM910006;Lepidoptera;Lasiommata maera;Lasiommata maera;1;Lasiommata maera;0.99999942908402;
HM910007;Lepidoptera;Pararge aegeria;Pararge aegeria;1;Pararge aegeria;0.999999999963165;
HM910008;Lepidoptera;Coenonympha arcania;NA;0.067;Coenonympha arcania;0.978811371852141;
HM910010;Lepidoptera;Coenonympha glycerion;NA;0.28;Coenonympha glycerion;0.999998036263913;
HM910012;Lepidoptera;Coenonympha glycerion;NA;0.104;Coenonympha glycerion;0.999997457415382;
HM910014;Lepidoptera;Minois dryas;NA;0.025;Minois dryas;0.850746026247397;
HM910015;Lepidoptera;Minois dryas;NA;0.025;Minois dryas;0.850746026247397;
HM910019;Lepidoptera;Pyrgus alveus;NA;NA;NA;0.999999999995496;
HM910498;Lepidoptera;Papilio machaon;Papilio machaon;NA;Papilio machaon;0.99889956649962;
HM910501;Lepidoptera;Anthocharis cardamines;Anthocharis cardamines;1;Anthocharis cardamines;0.999999988033608;
HM910506;Lepidoptera;Aporia crataegi;NA;NA;Aporia crataegi;0.999998459680561;
HM910507;Lepidoptera;Gonepteryx rhamni;Gonepteryx rhamni;1;Gonepteryx rhamni;0.999673119534565;
HM910510;Lepidoptera;Melitaea trivia;Melitaea trivia;1;Melitaea trivia;0.999999999955975;
HM910511;Lepidoptera;Melitaea didyma;Melitaea didyma;1;Melitaea didyma;0.999870115406424;
HM910512;Lepidoptera;Melitaea cinxia;Melitaea cinxia;1;Melitaea cinxia;0.999999999999631;
HM910514;Lepidoptera;Melitaea nevadensis;NA;0.005;Melitaea celadussa;0.873944840950458;
HM910573;Lepidoptera;Issoria lathonia;Issoria lathonia;1;Issoria lathonia;0.999999999989257;
HM910574;Lepidoptera;Lysandra coridon;Lysandra coridon;NA;NA;0.999039865644651;
HM910579;Lepidoptera;Vanessa atalanta;Vanessa atalanta;1;Vanessa atalanta;0.999999999746649;
HM910580;Lepidoptera;Fabriciana adippe;NA;NA;Fabriciana adippe;0.922280384106682;
HM910581;Lepidoptera;Hipparchia semele;NA;0.002;Hipparchia semele;0.887078465943553;
HM910583;Lepidoptera;Chazara briseis;Chazara briseis;0.001;Chazara briseis;0.999984130678865;
HM910594;Lepidoptera;Pieris napi;NA;0.011;Pieris napi;0.801985394724814;
HM910600;Lepidoptera;Maniola cecilia;Pyronia cecilia;1;Pyronia cecilia;0.999999999998863;
HM913938;Lepidoptera;Boloria euphrosyne;Boloria euphrosyne;1;Boloria euphrosyne;0.999999910115296;
HM913939;Lepidoptera;Issoria lathonia;Issoria lathonia;1;Issoria lathonia;0.999999999989257;
HM913940;Lepidoptera;Fabriciana adippe;NA;NA;Fabriciana adippe;0.922280384106682;
HM913943;Lepidoptera;Nymphalis egea;Polygonia egea;1;Polygonia egea;0.999999758078475;
HM913944;Lepidoptera;Nymphalis c-album;NA;0.08;NA;0.999996025433302;
HM913946;Lepidoptera;Pararge aegeria;Pararge aegeria;1;Pararge aegeria;0.999999999963165;
HM913947;Lepidoptera;Lasiommata megera;Lasiommata megera;1;Lasiommata megera;0.999498474862377;
HM913948;Lepidoptera;Lasiommata maera;Lasiommata maera;1;Lasiommata maera;0.99999510792253;
HM913950;Lepidoptera;Maniola cecilia;Pyronia cecilia;1;Pyronia cecilia;1;
HM913951;Lepidoptera;Chazara briseis;Chazara briseis;0.001;Chazara briseis;0.999984130678865;
HM913952;Lepidoptera;Hipparchia semele;NA;NA;Hipparchia semele;0.748345401572611;
HM913953;Lepidoptera;Lycaena tityrus;Lycaena tityrus;1;Lycaena tityrus;0.999998928238034;
HM913954;Lepidoptera;Lycaena phlaeas;Lycaena phlaeas;1;Lycaena phlaeas;0.99999999060276;
HM913955;Lepidoptera;Satyrium ilicis;Satyrium ilicis;1;Satyrium ilicis;0.999999987901305;
HM913956;Lepidoptera;Callophrys rubi;Callophrys rubi;0.007;Callophrys rubi;0.99851401092179;
HM913958;Lepidoptera;Lampides boeticus;Lampides boeticus;1;Lampides boeticus;0.999999999545906;
HM913959;Lepidoptera;Cupido alcetas;NA;0.218;Cupido alcetas;0.833501983138177;
HM913960;Lepidoptera;Cupido alcetas;NA;0.218;Cupido alcetas;0.833501983138177;
HM913961;Lepidoptera;Cupido minimus;NA;0.009;Cupido minimus;0.999791453712643;
HM913963;Lepidoptera;Glaucopsyche alexis;Glaucopsyche alexis;0.001;Glaucopsyche alexis;0.94042380088325;
HM913964;Lepidoptera;Plebejus argyrognomon;NA;0.001;NA;0.998518811781132;
HM913965;Lepidoptera;Plebejus argyrognomon;NA;0.001;NA;0.998518811781132;
HM913966;Lepidoptera;Aricia agestis;NA;0.253;Aricia agestis;0.996350017387504;
HM913967;Lepidoptera;Aricia agestis;NA;NA;Aricia agestis;0.993980512246517;
HM913969;Lepidoptera;Polyommatus amandus;Polyommatus amandus;0.002;Polyommatus amandus;0.99998879592523;
HM913970;Lepidoptera;Hamearis lucina;NA;NA;Hamearis lucina;0.999999999984141;
HM913973;Lepidoptera;Pyrgus onopordi;Pyrgus onopordi;1;Pyrgus onopordi;0.999997111229755;
HM913974;Lepidoptera;Pyrgus carthami;Pyrgus carthami;1;Pyrgus carthami;0.999999568765909;
HM913975;Lepidoptera;Pyrgus armoricanus;Pyrgus armoricanus;1;Pyrgus armoricanus;0.999997353848817;
HM913977;Lepidoptera;Thymelicus sylvestris;Thymelicus sylvestris;1;Thymelicus sylvestris;0.999999981568351;

HM913978;Lepidoptera;Thymelicus sylvestris;Thymelicus sylvestris;1;Thymelicus sylvestris;0.999999981568351;
HM913979;Lepidoptera;Ochlodes sylvanus;NA;0.316;Ochlodes sylvanus;0.996318754838625;
HM913980;Lepidoptera;Thymelicus acteon;Thymelicus acteon;1;Thymelicus acteon;0.99999999752248;
HM913982;Lepidoptera;Gegenes pumilio;Gegenes pumilio;1;Gegenes pumilio;0.999986726679266;
HMYZ021-19;Lepidoptera;Pieris rapae;NA;0.22;Pieris rapae;0.999885170635921;
HQ003947;Lepidoptera;Aglais urticae;NA;0.002;Aglais urticae;0.987497593219019;
HQ003948;Lepidoptera;Aglais urticae;NA;NA;Aglais urticae;0.924150107699752;
HQ003949;Lepidoptera;Aglais urticae;Aglais urticae;0.006;Aglais urticae;0.994059189651409;
HQ003950;Lepidoptera;Aglais urticae;NA;0.002;Aglais urticae;0.987497593219019;
HQ003951;Lepidoptera;Aglais urticae;Aglais urticae;0.001;Aglais urticae;0.990690600684087;
HQ003952;Lepidoptera;Aglais urticae;NA;0.002;Aglais urticae;0.987497593219019;
HQ003954;Lepidoptera;Anthocharis cardamines;Anthocharis cardamines;1;Anthocharis cardamines;0.999999940675226;
HQ003955;Lepidoptera;Anthocharis cardamines;Anthocharis cardamines;1;Anthocharis cardamines;0.999999940675226;
HQ003956;Lepidoptera;Anthocharis cardamines;Anthocharis cardamines;1;Anthocharis cardamines;0.999999936991374;
HQ003957;Lepidoptera;Anthocharis cardamines;Anthocharis cardamines;1;Anthocharis cardamines;0.999999940675226;
HQ003958;Lepidoptera;Anthocharis cardamines;Anthocharis cardamines;1;Anthocharis cardamines;0.999999948434409;
HQ003959;Lepidoptera;Anthocharis cardamines;Anthocharis cardamines;1;Anthocharis cardamines;0.999999940675226;
HQ003960;Lepidoptera;Anthocharis cardamines;Anthocharis cardamines;1;Anthocharis cardamines;0.999999979606855;
HQ003961;Lepidoptera;Anthocharis cardamines;Anthocharis cardamines;1;Anthocharis cardamines;0.999999940675226;
HQ003962;Lepidoptera;Anthocharis cardamines;Anthocharis cardamines;1;Anthocharis cardamines;0.999999951917603;
HQ003963;Lepidoptera;Apatura ilia;NA;0.039;Apatura ilia;0.977274423026401;
HQ003964;Lepidoptera;Apatura ilia;NA;0.165;Apatura ilia;0.956796812031481;
HQ003965;Lepidoptera;Apatura ilia;NA;0.165;Apatura ilia;0.956796812031481;
HQ003966;Lepidoptera;Apatura ilia;NA;0.243;Apatura ilia;0.940982441998145;
HQ003967;Lepidoptera;Apatura ilia;NA;0.165;Apatura ilia;0.956796812031481;
HQ003968;Lepidoptera;Apatura iris;Apatura iris;1;Apatura iris;0.999999999999915;
HQ003969;Lepidoptera;Apatura iris;Apatura iris;1;Apatura iris;0.999999999999943;
HQ003970;Lepidoptera;Apatura iris;Apatura iris;1;Apatura iris;0.999999999999943;
HQ003971;Lepidoptera;Apatura iris;Apatura iris;1;Apatura iris;0.999999999999915;
HQ003972;Lepidoptera;Apatura iris;Apatura iris;1;Apatura iris;0.999999999999943;
HQ003973;Lepidoptera;Apatura iris;Apatura iris;1;Apatura iris;0.999999999999631;
HQ003974;Lepidoptera;Apatura iris;Apatura iris;1;Apatura iris;0.999999999999943;
HQ003980;Lepidoptera;Aphantopus hyperantus;Aphantopus hyperantus;0.006;Aphantopus hyperantus;0.999998615504572;
HQ003981;Lepidoptera;Aphantopus hyperantus;Aphantopus hyperantus;0.003;Aphantopus hyperantus;0.999999455871523;
HQ003982;Lepidoptera;Aphantopus hyperantus;Aphantopus hyperantus;0.003;Aphantopus hyperantus;0.999999455871523;
HQ003983;Lepidoptera;Aphantopus hyperantus;Aphantopus hyperantus;0.003;Aphantopus hyperantus;0.999999455871523;
HQ003984;Lepidoptera;Aphantopus hyperantus;Aphantopus hyperantus;0.003;Aphantopus hyperantus;0.999999455871523;
HQ003985;Lepidoptera;Aphantopus hyperantus;Aphantopus hyperantus;0.002;Aphantopus hyperantus;0.999999020907804;
HQ003986;Lepidoptera;Aphantopus hyperantus;Aphantopus hyperantus;0.003;Aphantopus hyperantus;0.999999455871523;
HQ003987;Lepidoptera;Aporia crataegi;NA;0.074;Aporia crataegi;0.999879638454693;
HQ003988;Lepidoptera;Aporia crataegi;NA;NA;Aporia crataegi;0.999996899001592;
HQ003989;Lepidoptera;Aporia crataegi;NA;NA;Aporia crataegi;0.999996781126733;
HQ003990;Lepidoptera;Aporia crataegi;NA;NA;Aporia crataegi;0.999996781126733;
HQ003991;Lepidoptera;Aporia crataegi;NA;NA;Aporia crataegi;0.999996781126733;
HQ003992;Lepidoptera;Aporia crataegi;NA;0.002;Aporia crataegi;0.999995898341125;
HQ003993;Lepidoptera;Aporia crataegi;NA;NA;Aporia crataegi;0.999996781126733;
HQ003994;Lepidoptera;Araschnia levana;Araschnia levana;1;Araschnia levana;0.999999772187832;
HQ003995;Lepidoptera;Araschnia levana;Araschnia levana;1;Araschnia levana;0.999999772187832;
HQ003996;Lepidoptera;Araschnia levana;Araschnia levana;1;Araschnia levana;0.999999772187832;
HQ003997;Lepidoptera;Araschnia levana;Araschnia levana;1;Araschnia levana;0.999999772187832;
HQ003998;Lepidoptera;Araschnia levana;Araschnia levana;1;Araschnia levana;0.999999772187832;
HQ003999;Lepidoptera;Araschnia levana;Araschnia levana;1;Araschnia levana;0.999999772187832;
HQ004000;Lepidoptera;Araschnia levana;Araschnia levana;1;Araschnia levana;0.999999734047783;
HQ004001;Lepidoptera;Arethusana arethusa;NA;0.153;Arethusana arethusa;0.999652039046744;
HQ004002;Lepidoptera;Arethusana arethusa;Arethusana arethusa;0.026;Arethusana arethusa;0.999926983375418;
HQ004003;Lepidoptera;Arethusana arethusa;NA;0.153;Arethusana arethusa;0.999652039046744;
HQ004004;Lepidoptera;Arethusana arethusa;Arethusana arethusa;0.024;Arethusana arethusa;0.999978283522486;
HQ004005;Lepidoptera;Arethusana arethusa;NA;0.153;Arethusana arethusa;0.999652039046744;
HQ004006;Lepidoptera;Arethusana arethusa;NA;0.153;Arethusana arethusa;0.999652039046744;

HQ004007;Lepidoptera;Arethusana arethusa;NA;0.153;Arethusana arethusa;0.999652039046744;
HQ004008;Lepidoptera;Arethusana arethusa;Arethusana arethusa;0.023;Arethusana arethusa;0.999971494862782;
HQ004009;Lepidoptera;Fabriciana adippe;Fabriciana adippe;0.012;Fabriciana adippe;0.987124560355947;
HQ004010;Lepidoptera;Fabriciana adippe;Fabriciana adippe;0.003;Fabriciana adippe;0.993461634804659;
HQ004011;Lepidoptera;Fabriciana adippe;Fabriciana adippe;0.002;Fabriciana adippe;0.995619139154349;
HQ004012;Lepidoptera;Fabriciana adippe;NA;NA;Fabriciana adippe;0.98103293393153;
HQ004013;Lepidoptera;Fabriciana adippe;Fabriciana adippe;0.012;Fabriciana adippe;0.987124560355947;
HQ004014;Lepidoptera;Fabriciana adippe;Fabriciana adippe;NA;Fabriciana adippe;0.98186989364118;
HQ004015;Lepidoptera;Argynnis aglaja;NA;0.001;NA;0.999999999999586;
HQ004016;Lepidoptera;Argynnis aglaja;NA;NA;Speyeria aglaja;0.872991147917853;
HQ004017;Lepidoptera;Argynnis aglaja;NA;0.001;NA;0.999999999999586;
HQ004018;Lepidoptera;Argynnis aglaja;NA;NA;Speyeria aglaja;0.872991147917853;
HQ004019;Lepidoptera;Argynnis aglaja;NA;0.004;Speyeria aglaja;0.785084763582602;
HQ004020;Lepidoptera;Argynnis aglaja;NA;0.004;Speyeria aglaja;0.785084763582602;
HQ004021;Lepidoptera;Argynnis aglaja;NA;0.001;NA;0.999999999999586;
HQ004026;Lepidoptera;Fabriciana niobe;NA;0.005;Fabriciana niobe;0.992125835358755;
HQ004027;Lepidoptera;Fabriciana niobe;NA;0.002;Fabriciana niobe;0.977304369806308;
HQ004028;Lepidoptera;Fabriciana niobe;Fabriciana niobe;0.001;Fabriciana niobe;0.985635797870942;
HQ004029;Lepidoptera;Fabriciana niobe;NA;0.001;Fabriciana niobe;0.977553955749008;
HQ004030;Lepidoptera;Fabriciana niobe;Fabriciana niobe;0.001;Fabriciana niobe;0.985635797870942;
HQ004031;Lepidoptera;Fabriciana niobe;NA;0.005;Fabriciana niobe;0.992125835358755;
HQ004032;Lepidoptera;Fabriciana niobe;NA;0.007;Fabriciana niobe;0.984627988609443;
HQ004044;Lepidoptera;Argynnis paphia;Argynnis paphia;1;Argynnis paphia;0.999999992110673;
HQ004045;Lepidoptera;Aricia agestis;NA;0.081;Aricia agestis;0.998857812762894;
HQ004046;Lepidoptera;Aricia agestis;NA;0.081;Aricia agestis;0.998857812762894;
HQ004047;Lepidoptera;Aricia agestis;NA;0.081;Aricia agestis;0.998857812762894;
HQ004048;Lepidoptera;Aricia agestis;NA;0.067;Aricia agestis;0.999509743227786;
HQ004049;Lepidoptera;Aricia agestis;NA;0.081;Aricia agestis;0.998857812762894;
HQ004050;Lepidoptera;Aricia agestis;NA;0.081;Aricia agestis;0.998857812762894;
HQ004051;Lepidoptera;Aricia agestis;NA;0.081;Aricia agestis;0.998857812762894;
HQ004052;Lepidoptera;Aricia agestis;NA;0.067;Aricia agestis;0.999509743227786;
HQ004053;Lepidoptera;Aricia agestis;NA;0.081;Aricia agestis;0.998857812762894;
HQ004054;Lepidoptera;Aricia agestis;NA;0.081;Aricia agestis;0.998857812762894;
HQ004055;Lepidoptera;Aricia agestis;NA;0.03;Aricia agestis;0.998027685685265;
HQ004056;Lepidoptera;Aricia agestis;NA;0.039;Aricia agestis;0.99882588429926;
HQ004057;Lepidoptera;Aricia artaxerxes;NA;0.394;Aricia artaxerxes;0.999502890976379;
HQ004058;Lepidoptera;Aricia artaxerxes;NA;0.394;Aricia artaxerxes;0.999502890976379;
HQ004059;Lepidoptera;Aricia artaxerxes;NA;0.394;Aricia artaxerxes;0.999502890976379;
HQ004060;Lepidoptera;Aricia artaxerxes;NA;0.394;Aricia artaxerxes;0.999502890976379;
HQ004061;Lepidoptera;Aricia artaxerxes;NA;0.394;Aricia artaxerxes;0.999502890976379;
HQ004062;Lepidoptera;Aricia artaxerxes;NA;0.151;Aricia artaxerxes;0.999531539195816;
HQ004063;Lepidoptera;Aricia artaxerxes;NA;0.394;Aricia artaxerxes;0.999502890976379;
HQ004064;Lepidoptera;Aricia artaxerxes;NA;0.394;Aricia artaxerxes;0.999502890976379;
HQ004065;Lepidoptera;Aricia artaxerxes;NA;0.394;Aricia artaxerxes;0.999502890976379;
HQ004076;Lepidoptera;Boloria dia;Boloria dia;1;Boloria dia;0.999999999980503;
HQ004077;Lepidoptera;Boloria dia;Boloria dia;1;Boloria dia;0.999999999980503;
HQ004079;Lepidoptera;Boloria dia;Boloria dia;1;Boloria dia;0.999999999980503;
HQ004080;Lepidoptera;Boloria dia;Boloria dia;1;Boloria dia;0.999999999980503;
HQ004081;Lepidoptera;Boloria dia;Boloria dia;1;Boloria dia;0.999999999980503;
HQ004082;Lepidoptera;Boloria euphrosyne;Boloria euphrosyne;1;Boloria euphrosyne;0.999998102693392;
HQ004083;Lepidoptera;Boloria euphrosyne;Boloria euphrosyne;1;Boloria euphrosyne;0.999998552914401;
HQ004084;Lepidoptera;Boloria euphrosyne;Boloria euphrosyne;1;Boloria euphrosyne;0.99999841672292;
HQ004085;Lepidoptera;Boloria euphrosyne;Boloria euphrosyne;1;Boloria euphrosyne;0.999997763335159;
HQ004086;Lepidoptera;Boloria euphrosyne;Boloria euphrosyne;1;Boloria euphrosyne;0.999998552914401;
HQ004087;Lepidoptera;Boloria euphrosyne;Boloria euphrosyne;1;Boloria euphrosyne;0.999998514406196;
HQ004088;Lepidoptera;Boloria euphrosyne;Boloria euphrosyne;1;Boloria euphrosyne;0.999998552914401;
HQ004089;Lepidoptera;Boloria euphrosyne;Boloria euphrosyne;1;Boloria euphrosyne;0.999998102693392;
HQ004090;Lepidoptera;Boloria pales;Boloria pales;0.998;Boloria pales;0.996520205418986;
HQ004091;Lepidoptera;Boloria pales;Boloria pales;0.998;Boloria pales;0.996520205418986;
HQ004092;Lepidoptera;Boloria pales;Boloria pales;0.998;Boloria pales;0.996520205418986;
HQ004095;Lepidoptera;Boloria pales;Boloria pales;0.998;Boloria pales;0.996520205418986;
HQ004096;Lepidoptera;Boloria pales;Boloria pales;0.989;Boloria pales;0.995496736606565;
HQ004106;Lepidoptera;Boloria titania;Boloria titania;0.001;Boloria titania;0.99990849163814;
HQ004107;Lepidoptera;Boloria titania;Boloria titania;0.001;Boloria titania;0.99990849163814;
HQ004108;Lepidoptera;Boloria titania;Boloria titania;0.001;Boloria titania;0.99990849163814;

HQ004109;Lepidoptera;Boloria titania;Boloria titania;0.001;Boloria titania;0.99990849163814;
HQ004110;Lepidoptera;Boloria titania;Boloria titania;0.001;Boloria titania;0.99990849163814;
HQ004111;Lepidoptera;Boloria titania;Boloria titania;0.001;Boloria titania;0.99990849163814;
HQ004112;Lepidoptera;Brenthis daphne;NA;0.102;Brenthis daphne;0.717134521611551;
HQ004113;Lepidoptera;Brenthis daphne;NA;0.102;Brenthis daphne;0.717134521611551;
HQ004114;Lepidoptera;Brenthis daphne;NA;0.102;Brenthis daphne;0.717134521611551;
HQ004115;Lepidoptera;Brenthis daphne;NA;0.063;Brenthis daphne;0.854461977744429;
HQ004116;Lepidoptera;Brenthis daphne;NA;0.102;Brenthis daphne;0.717134521611551;
HQ004117;Lepidoptera;Brenthis daphne;NA;0.102;Brenthis daphne;0.717134521611551;
HQ004118;Lepidoptera;Brenthis daphne;Brenthis daphne;1;Brenthis daphne;0.998500858925195;
HQ004119;Lepidoptera;Brenthis daphne;NA;0.043;Brenthis daphne;0.722020100159206;
HQ004120;Lepidoptera;Brenthis daphne;NA;0.102;Brenthis daphne;0.717134521611551;
HQ004121;Lepidoptera;Brenthis hecate;Brenthis hecate;0.015;Brenthis hecate;0.999871793261773;
HQ004122;Lepidoptera;Brenthis hecate;NA;0.034;Brenthis hecate;0.99987292514345;
HQ004123;Lepidoptera;Brenthis hecate;NA;0.061;Brenthis hecate;0.999698739882756;
HQ004124;Lepidoptera;Brenthis hecate;NA;0.001;Brenthis hecate;0.9996055733577;
HQ004125;Lepidoptera;Brenthis hecate;NA;0.072;Brenthis hecate;0.999803056289114;
HQ004126;Lepidoptera;Brenthis hecate;NA;0.083;Brenthis hecate;0.999707638996184;
HQ004127;Lepidoptera;Brenthis hecate;NA;0.083;Brenthis hecate;0.999707638996184;
HQ004128;Lepidoptera;Brenthis hecate;Brenthis hecate;0.015;Brenthis hecate;0.999840709488841;
HQ004129;Lepidoptera;Brenthis ino;Brenthis ino;0.998;Brenthis ino;0.995325118916732;
HQ004130;Lepidoptera;Brenthis ino;Brenthis ino;0.998;Brenthis ino;0.995325118916732;
HQ004131;Lepidoptera;Brenthis ino;Brenthis ino;1;Brenthis ino;0.99882211566857;
HQ004132;Lepidoptera;Brenthis ino;Brenthis ino;0.998;Brenthis ino;0.995325118916732;
HQ004133;Lepidoptera;Brenthis ino;Brenthis ino;0.007;Brenthis ino;0.977157381961834;
HQ004134;Lepidoptera;Brenthis ino;Brenthis ino;1;Brenthis ino;0.997823940101957;
HQ004135;Lepidoptera;Brenthis ino;Brenthis ino;1;Brenthis ino;0.99882211566857;
HQ004136;Lepidoptera;Brenthis ino;Brenthis ino;1;Brenthis ino;0.997823940101957;
HQ004137;Lepidoptera;Brintesia circe;Brintesia circe;NA;Brintesia circe;0.999999813082031;
HQ004138;Lepidoptera;Brintesia circe;Brintesia circe;1;Brintesia circe;0.999999566571953;
HQ004139;Lepidoptera;Brintesia circe;Brintesia circe;NA;Brintesia circe;0.999999592702691;
HQ004140;Lepidoptera;Brintesia circe;Brintesia circe;NA;Brintesia circe;0.999999823544969;
HQ004141;Lepidoptera;Brintesia circe;Brintesia circe;NA;Brintesia circe;0.999999592702691;
HQ004142;Lepidoptera;Brintesia circe;Brintesia circe;NA;Brintesia circe;0.999999592702691;
HQ004143;Lepidoptera;Brintesia circe;Brintesia circe;1;Brintesia circe;0.999999111118765;
HQ004144;Lepidoptera;Brintesia circe;Brintesia circe;NA;Brintesia circe;0.999999823544969;
HQ004145;Lepidoptera;Brintesia circe;Brintesia circe;1;Brintesia circe;0.999999264057679;
HQ004146;Lepidoptera;Callophrys rubi;NA;0.003;Callophrys rubi;0.996753822785804;
HQ004147;Lepidoptera;Callophrys rubi;NA;0.003;Callophrys rubi;0.997328639918919;
HQ004148;Lepidoptera;Callophrys rubi;NA;0.003;Callophrys rubi;0.996753822785804;
HQ004149;Lepidoptera;Callophrys rubi;NA;0.003;Callophrys rubi;0.997328639918919;
HQ004150;Lepidoptera;Callophrys rubi;NA;0.002;Callophrys rubi;0.996052483231556;
HQ004151;Lepidoptera;Callophrys rubi;NA;0.003;Callophrys rubi;0.997328639918919;
HQ004152;Lepidoptera;Callophrys rubi;NA;0.002;Callophrys rubi;0.993800423545913;
HQ004153;Lepidoptera;Callophrys rubi;NA;0.002;Callophrys rubi;0.997663599924675;
HQ004154;Lepidoptera;Callophrys rubi;NA;0.001;Ahlbergia frivaldszkyi;0.774173868731941;
HQ004155;Lepidoptera;Carcharodus alceae;Carcharodus alceae;NA;Carcharodus alceae;0.999998131544272;
HQ004156;Lepidoptera;Carcharodus alceae;Carcharodus alceae;0.999;Carcharodus alceae;0.99999999752319;
HQ004157;Lepidoptera;Carcharodus alceae;Carcharodus alceae;0.999;Carcharodus alceae;0.999999998008462;
HQ004158;Lepidoptera;Carcharodus alceae;Carcharodus alceae;0.001;Carcharodus alceae;0.999999998054733;
HQ004159;Lepidoptera;Carcharodus alceae;Carcharodus alceae;0.999;Carcharodus alceae;0.999999998008462;
HQ004160;Lepidoptera;Carcharodus alceae;Carcharodus alceae;0.999;Carcharodus alceae;0.999999998008462;
HQ004161;Lepidoptera;Carcharodus alceae;Carcharodus alceae;NA;Carcharodus alceae;0.999998131544272;
HQ004162;Lepidoptera;Carcharodus alceae;Carcharodus alceae;0.999;Carcharodus alceae;0.999999996339511;
HQ004163;Lepidoptera;Carcharodus alceae;Carcharodus alceae;NA;Carcharodus alceae;0.999998131544272;
HQ004164;Lepidoptera;Carcharodus alceae;Carcharodus alceae;0.999;Carcharodus alceae;0.999999998008462;
HQ004165;Lepidoptera;Carcharodus alceae;Carcharodus alceae;0.999;Carcharodus alceae;0.999999998321016;
HQ004175;Lepidoptera;Carcharodus lavatherae;Carcharodus lavatherae;0.047;Carcharodus lavatherae;0.998322815497744;
HQ004176;Lepidoptera;Carcharodus lavatherae;NA;0.109;Carcharodus lavatherae;0.998655193590746;
HQ004177;Lepidoptera;Carcharodus lavatherae;NA;0.108;Carcharodus lavatherae;0.998927718400682;
HQ004178;Lepidoptera;Carcharodus lavatherae;NA;0.108;Carcharodus lavatherae;0.998927718400682;
HQ004188;Lepidoptera;Carterocephalus palaemon;Carterocephalus palaemon;0.011;Carterocephalus palaemon;0.826779617779824;
HQ004189;Lepidoptera;Carterocephalus palaemon;Carterocephalus palaemon;0.013;Carterocephalus palaemon;0.795574350711358;
HQ004190;Lepidoptera;Carterocephalus palaemon;NA;0.028;NA;0.999999999999333;

HQ004191;Lepidoptera;Carterocephalus palaemon;NA;0.052;Carterocephalus palaemon;0.728445051795325;
HQ004192;Lepidoptera;Carterocephalus palaemon;Carterocephalus palaemon;0.015;Carterocephalus palaemon;0.803403729950902;
HQ004193;Lepidoptera;Carterocephalus palaemon;NA;0.052;Carterocephalus palaemon;0.728445051795325;
HQ004194;Lepidoptera;Carterocephalus palaemon;NA;0.052;Carterocephalus palaemon;0.728445051795325;
HQ004195;Lepidoptera;Celastrina argiolus;Celastrina argiolus;1;Celastrina argiolus;0.999631157817044;
HQ004196;Lepidoptera;Celastrina argiolus;Celastrina argiolus;1;Celastrina argiolus;0.999631157817044;
HQ004197;Lepidoptera;Celastrina argiolus;Celastrina argiolus;1;Celastrina argiolus;0.999527385150531;
HQ004198;Lepidoptera;Celastrina argiolus;Celastrina argiolus;1;Celastrina argiolus;0.999527385150531;
HQ004199;Lepidoptera;Celastrina argiolus;Celastrina argiolus;0.998;Celastrina argiolus;0.999557774480579;
HQ004200;Lepidoptera;Celastrina argiolus;Celastrina argiolus;1;Celastrina argiolus;0.999631157817044;
HQ004201;Lepidoptera;Celastrina argiolus;Celastrina argiolus;1;Celastrina argiolus;0.999527385150531;
HQ004202;Lepidoptera;Celastrina argiolus;Celastrina argiolus;1;Celastrina argiolus;0.999527385150531;
HQ004203;Lepidoptera;Celastrina argiolus;Celastrina argiolus;1;Celastrina argiolus;0.999527385150531;
HQ004204;Lepidoptera;Chazara briseis;Chazara briseis;0.001;Chazara briseis;0.999984130678865;
HQ004205;Lepidoptera;Chazara briseis;Chazara briseis;0.001;Chazara briseis;0.999984130678865;
HQ004206;Lepidoptera;Chazara briseis;Chazara briseis;0.001;Chazara briseis;0.999984130678865;
HQ004207;Lepidoptera;Chazara briseis;Chazara briseis;0.001;Chazara briseis;0.999960914898745;
HQ004208;Lepidoptera;Chazara briseis;Chazara briseis;0.001;Chazara briseis;0.999984130678865;
HQ004209;Lepidoptera;Chazara briseis;Chazara briseis;0.001;Chazara briseis;0.999944132711476;
HQ004210;Lepidoptera;Coenonympha arcania;NA;0.002;NA;0.999999999999975;
HQ004211;Lepidoptera;Coenonympha arcania;NA;0.058;NA;0.999999999999967;
HQ004212;Lepidoptera;Coenonympha arcania;NA;0.001;NA;0.999999999999973;
HQ004213;Lepidoptera;Coenonympha arcania;NA;0.001;NA;0.999999999999975;
HQ004214;Lepidoptera;Coenonympha arcania;NA;0.001;NA;0.999999999999975;
HQ004215;Lepidoptera;Coenonympha arcania;NA;0.001;NA;0.999999999999975;
HQ004216;Lepidoptera;Coenonympha arcania;NA;0.058;NA;0.999999999999967;
HQ004217;Lepidoptera;Coenonympha arcania;NA;0.001;NA;0.999999999999975;
HQ004218;Lepidoptera;Coenonympha arcania;NA;0.001;NA;0.999999999999965;
HQ004219;Lepidoptera;Coenonympha glycerion;NA;0.206;Coenonympha glycerion;0.999999087964472;
HQ004220;Lepidoptera;Coenonympha glycerion;NA;0.28;Coenonympha glycerion;0.999998036263913;
HQ004221;Lepidoptera;Coenonympha glycerion;NA;0.199;Coenonympha glycerion;0.999998758999797;
HQ004222;Lepidoptera;Coenonympha glycerion;NA;0.064;Coenonympha glycerion;0.999999129312964;
HQ004223;Lepidoptera;Coenonympha glycerion;NA;0.295;Coenonympha glycerion;0.999998958157621;
HQ004224;Lepidoptera;Coenonympha glycerion;NA;0.28;Coenonympha glycerion;0.999998036263913;
HQ004225;Lepidoptera;Coenonympha glycerion;NA;0.295;Coenonympha glycerion;0.999998958157621;
HQ004226;Lepidoptera;Coenonympha glycerion;NA;0.295;Coenonympha glycerion;0.999998958157621;
HQ004227;Lepidoptera;Coenonympha glycerion;NA;0.32;Coenonympha glycerion;0.999996720049446;
HQ004233;Lepidoptera;Coenonympha pamphilus;Coenonympha pamphilus;1;Coenonympha pamphilus;0.999999999999545;
HQ004234;Lepidoptera;Coenonympha pamphilus;Coenonympha pamphilus;1;Coenonympha pamphilus;0.999999999999744;
HQ004235;Lepidoptera;Coenonympha pamphilus;Coenonympha pamphilus;1;Coenonympha pamphilus;0.999999999999744;
HQ004236;Lepidoptera;Coenonympha pamphilus;Coenonympha pamphilus;1;Coenonympha pamphilus;0.999999999999432;
HQ004237;Lepidoptera;Coenonympha pamphilus;Coenonympha pamphilus;1;Coenonympha pamphilus;0.999999999999488;
HQ004238;Lepidoptera;Coenonympha pamphilus;Coenonympha pamphilus;1;Coenonympha pamphilus;0.999999999999744;
HQ004239;Lepidoptera;Coenonympha pamphilus;Coenonympha pamphilus;1;Coenonympha pamphilus;0.999999999999744;
HQ004240;Lepidoptera;Coenonympha pamphilus;Coenonympha pamphilus;1;Coenonympha pamphilus;0.999999999999744;
HQ004241;Lepidoptera;Coenonympha pamphilus;Coenonympha pamphilus;1;Coenonympha pamphilus;0.999999999999744;
HQ004242;Lepidoptera;Coenonympha rhodopensis;NA;0.019;Coenonympha rhodopensis;0.872457011198986;
HQ004243;Lepidoptera;Coenonympha rhodopensis;NA;0.019;Coenonympha rhodopensis;0.872457011198986;
HQ004244;Lepidoptera;Coenonympha rhodopensis;NA;0.035;Coenonympha rhodopensis;0.853098489717182;
HQ004245;Lepidoptera;Coenonympha rhodopensis;NA;0.019;Coenonympha rhodopensis;0.872457011198986;
HQ004246;Lepidoptera;Coenonympha rhodopensis;NA;0.035;Coenonympha rhodopensis;0.853098489717182;
HQ004252;Lepidoptera;Colias alfacariensis;Colias alfacariensis;0.001;Colias alfacariensis;0.999926026317526;
HQ004253;Lepidoptera;Colias alfacariensis;Colias alfacariensis;0.001;Colias alfacariensis;0.999926026317526;

HQ004254;Lepidoptera;Colias alfacariensis;Colias alfacariensis;1;Colias alfacariensis;0.999958999251201;
HQ004255;Lepidoptera;Colias alfacariensis;Colias alfacariensis;1;Colias alfacariensis;0.999986994131001;
HQ004256;Lepidoptera;Colias alfacariensis;Colias alfacariensis;1;Colias alfacariensis;0.999986994131001;
HQ004257;Lepidoptera;Colias alfacariensis;Colias alfacariensis;1;Colias alfacariensis;0.999986994131001;
HQ004258;Lepidoptera;Colias alfacariensis;Colias alfacariensis;1;Colias alfacariensis;0.999958999251201;
HQ004259;Lepidoptera;Colias alfacariensis;Colias alfacariensis;1;Colias alfacariensis;0.999958999251201;
HQ004260;Lepidoptera;Colias alfacariensis;Colias alfacariensis;1;Colias alfacariensis;0.999986994131001;
HQ004261;Lepidoptera;Colias alfacariensis;Colias alfacariensis;1;Colias alfacariensis;0.999986994131001;
HQ004262;Lepidoptera;Colias alfacariensis;Colias alfacariensis;1;Colias alfacariensis;0.999985834946241;
HQ004263;Lepidoptera;Colias alfacariensis;Colias alfacariensis;1;Colias alfacariensis;0.999958999251201;
HQ004264;Lepidoptera;Colias alfacariensis;Colias alfacariensis;1;Colias alfacariensis;0.999986994131001;
HQ004265;Lepidoptera;Colias alfacariensis;Colias alfacariensis;1;Colias alfacariensis;0.999966879080062;
HQ004266;Lepidoptera;Colias alfacariensis;Colias alfacariensis;1;Colias alfacariensis;0.999959811913174;
HQ004267;Lepidoptera;Colias alfacariensis;Colias alfacariensis;1;Colias alfacariensis;0.999987100165369;
HQ004268;Lepidoptera;Colias alfacariensis;Colias alfacariensis;1;Colias alfacariensis;0.999986994131001;
HQ004269;Lepidoptera;Colias alfacariensis;Colias alfacariensis;0.001;Colias alfacariensis;0.999966522684745;
HQ004274;Lepidoptera;Colias crocea;NA;0.059;NA;0.999999999999738;
HQ004275;Lepidoptera;Colias crocea;NA;0.059;NA;0.999999999999738;
HQ004276;Lepidoptera;Colias croceus;NA;NA;NA;0.999999999999568;
HQ004277;Lepidoptera;Colias crocea;NA;NA;NA;0.999999999999568;
HQ004278;Lepidoptera;Colias crocea;NA;0.059;NA;0.999999999999738;
HQ004279;Lepidoptera;Colias crocea;NA;NA;NA;0.999999999999751;
HQ004280;Lepidoptera;Colias crocea;NA;0.059;NA;0.999999999999738;
HQ004281;Lepidoptera;Colias crocea;NA;0.059;NA;0.999999999999738;
HQ004282;Lepidoptera;Colias crocea;NA;NA;Colias crocea;0.708512483346918;
HQ004297;Lepidoptera;Colias hyale;Colias hyale;0.012;Colias hyale;0.999998203311315;
HQ004298;Lepidoptera;Colias hyale;Colias hyale;0.012;Colias hyale;0.999998203311315;
HQ004299;Lepidoptera;Colias hyale;Colias hyale;0.015;Colias hyale;0.999997884296128;
HQ004300;Lepidoptera;Colias hyale;Colias hyale;0.015;Colias hyale;0.999997884296128;
HQ004301;Lepidoptera;Colias hyale;Colias hyale;0.015;Colias hyale;0.999997884296128;
HQ004302;Lepidoptera;Colias hyale;Colias hyale;0.015;Colias hyale;0.999997884296128;
HQ004307;Lepidoptera;Cupido alcetas;Cupido alcetas;0.998;Cupido alcetas;0.996143733014087;
HQ004308;Lepidoptera;Cupido alcetas;Cupido alcetas;0.998;Cupido alcetas;0.996143733014087;
HQ004309;Lepidoptera;Cupido alcetas;Cupido alcetas;0.995;Cupido alcetas;0.996047865612985;
HQ004310;Lepidoptera;Cupido argiades;NA;0.499;NA;0.999999999984682;
HQ004311;Lepidoptera;Cupido argiades;NA;0.499;NA;0.999999999984682;
HQ004312;Lepidoptera;Cupido argiades;NA;0.499;NA;0.999999999984682;
HQ004313;Lepidoptera;Cupido argiades;NA;0.183;Cupido argiades;0.779615330961106;
HQ004314;Lepidoptera;Cupido argiades;NA;0.497;NA;0.999999999985171;
HQ004315;Lepidoptera;Cupido argiades;NA;0.499;NA;0.999999999984682;
HQ004316;Lepidoptera;Cupido argiades;NA;0.499;NA;0.999999999984682;
HQ004317;Lepidoptera;Cupido argiades;NA;0.499;NA;0.999999999984682;
HQ004318;Lepidoptera;Cupido argiades;NA;0.499;NA;0.999999999984682;
HQ004328;Lepidoptera;Cupido minimus;NA;0.016;Cupido minimus;0.999712342982067;
HQ004329;Lepidoptera;Cupido minimus;NA;0.001;Cupido minimus;0.997732398012586;
HQ004330;Lepidoptera;Cupido minimus;NA;0.001;Cupido minimus;0.997621299333221;
HQ004331;Lepidoptera;Cupido minimus;NA;0.009;Cupido minimus;0.999791453712643;
HQ004332;Lepidoptera;Cupido minimus;NA;0.009;Cupido minimus;0.999791453712643;
HQ004333;Lepidoptera;Cupido minimus;NA;0.004;Cupido minimus;0.99982784666808;
HQ004334;Lepidoptera;Cupido minimus;NA;0.003;Cupido minimus;0.999787792962518;
HQ004335;Lepidoptera;Cupido minimus;NA;0.004;Cupido minimus;0.99987402211542;
HQ004336;Lepidoptera;Cupido minimus;NA;0.003;Cupido minimus;0.99984089771194;
HQ004337;Lepidoptera;Cupido minimus;NA;0.001;Cupido minimus;0.999892569949957;
HQ004338;Lepidoptera;Cupido osiris;Cupido osiris;1;Cupido osiris;0.999998710927265;
HQ004339;Lepidoptera;Cupido osiris;Cupido osiris;1;Cupido osiris;0.999998710927265;
HQ004340;Lepidoptera;Cupido osiris;Cupido osiris;1;Cupido osiris;0.999999920248743;
HQ004341;Lepidoptera;Cupido osiris;Cupido osiris;1;Cupido osiris;0.999999920248743;
HQ004342;Lepidoptera;Cupido osiris;Cupido osiris;1;Cupido osiris;0.99999991590116;
HQ004343;Lepidoptera;Cupido osiris;Cupido osiris;1;Cupido osiris;0.999998710927265;
HQ004344;Lepidoptera;Cupido osiris;Cupido osiris;1;Cupido osiris;0.999999837166924;
HQ004345;Lepidoptera;Cyaniris semiargus;NA;NA;Cyaniris semiargus;0.999069195783418;

HQ004346;Lepidoptera;Cyaniris semiargus;Cyaniris semiargus;NA;Cyaniris semiargus;0.999717779236788;
HQ004347;Lepidoptera;Cyaniris semiargus;NA;NA;Cyaniris semiargus;0.998474211845289;
HQ004348;Lepidoptera;Cyaniris semiargus;Cyaniris semiargus;NA;Cyaniris semiargus;0.999707733485007;
HQ004349;Lepidoptera;Cyaniris semiargus;NA;NA;Cyaniris semiargus;0.999196155013845;
HQ004350;Lepidoptera;Cyaniris semiargus;NA;NA;Cyaniris semiargus;0.997097672202902;
HQ004351;Lepidoptera;Cyaniris semiargus;NA;NA;Cyaniris semiargus;0.999051841766953;
HQ004352;Lepidoptera;Cyaniris semiargus;NA;NA;Cyaniris semiargus;0.999051841766953;
HQ004369;Lepidoptera;Erebia epiphron;Erebia epiphron;NA;Erebia epiphron;0.998021437243156;
HQ004370;Lepidoptera;Erebia epiphron;Erebia epiphron;NA;Erebia epiphron;0.995130554397791;
HQ004371;Lepidoptera;Erebia epiphron;Erebia epiphron;NA;Erebia epiphron;0.998021437243156;
HQ004372;Lepidoptera;Erebia epiphron;Erebia epiphron;NA;Erebia epiphron;0.996812763610257;
HQ004373;Lepidoptera;Erebia epiphron;Erebia epiphron;NA;Erebia epiphron;0.998021437243156;
HQ004374;Lepidoptera;Erebia epiphron;Erebia epiphron;NA;Erebia epiphron;0.995130554397791;
HQ004375;Lepidoptera;Erebia epiphron;Erebia epiphron;NA;Erebia epiphron;0.998021437243156;
HQ004376;Lepidoptera;Erebia epiphron;Erebia epiphron;NA;Erebia epiphron;0.998021437243156;
HQ004377;Lepidoptera;Erebia epiphron;Erebia epiphron;NA;Erebia epiphron;0.998021437243156;
HQ004378;Lepidoptera;Erebia euryale;NA;NA;Erebia euryale;0.914169138470041;
HQ004379;Lepidoptera;Erebia euryale;NA;NA;Erebia euryale;0.919518760623792;
HQ004380;Lepidoptera;Erebia euryale;NA;NA;Erebia euryale;0.919518760623792;
HQ004381;Lepidoptera;Erebia euryale;NA;NA;Erebia euryale;0.919518760623792;
HQ004382;Lepidoptera;Erebia euryale;NA;NA;Erebia euryale;0.919518760623792;
HQ004383;Lepidoptera;Erebia euryale;NA;NA;Erebia euryale;0.919518760623792;
HQ004384;Lepidoptera;Erebia euryale;NA;NA;Erebia euryale;0.919518760623792;
HQ004386;Lepidoptera;Erebia euryale;NA;NA;Erebia euryale;0.919518760623792;
HQ004387;Lepidoptera;Erebia euryale;NA;NA;Erebia euryale;0.919518760623792;
HQ004405;Lepidoptera;Erebia manto;NA;0.109;Erebia manto;0.996739694842059;
HQ004406;Lepidoptera;Erebia manto;Erebia manto;NA;Erebia manto;0.999129075172383;
HQ004407;Lepidoptera;Erebia manto;NA;0.109;Erebia manto;0.996739694842059;
HQ004408;Lepidoptera;Erebia manto;NA;0.063;Erebia manto;0.998375472639544;
HQ004409;Lepidoptera;Erebia manto;NA;NA;Erebia manto;0.997239343105498;
HQ004410;Lepidoptera;Erebia manto;NA;0.063;Erebia manto;0.998375472639544;
HQ004411;Lepidoptera;Erebia manto;NA;NA;Erebia manto;0.997239343105498;
HQ004412;Lepidoptera;Erebia manto;NA;0.109;Erebia manto;0.996739694842059;
HQ004413;Lepidoptera;Erebia manto;NA;0.063;Erebia manto;0.998375472639544;
HQ004431;Lepidoptera;Erebia pandrose;Erebia pandrose;0.003;Erebia pandrose;0.993608645577247;
HQ004432;Lepidoptera;Erebia pandrose;Erebia pandrose;0.998;Erebia pandrose;0.997117103851311;
HQ004433;Lepidoptera;Erebia pandrose;Erebia pandrose;0.999;Erebia pandrose;0.994023911585754;
HQ004434;Lepidoptera;Erebia pandrose;Erebia pandrose;0.999;Erebia pandrose;0.994023911585754;
HQ004435;Lepidoptera;Erebia pharte;Erebia pharte;1;Erebia pharte;0.999999973572898;
HQ004436;Lepidoptera;Erebia pharte;Erebia pharte;1;Erebia pharte;0.999999973572898;
HQ004437;Lepidoptera;Erebia pronoe;Erebia pronoe;1;Erebia pronoe;0.998721231437303;
HQ004438;Lepidoptera;Erebia pronoe;Erebia pronoe;0.001;Erebia pronoe;0.997621582415603;
HQ004439;Lepidoptera;Erebia pronoe;Erebia pronoe;0.999;Erebia pronoe;0.995193627523185;
HQ004440;Lepidoptera;Erebia pronoe;Erebia pronoe;0.001;Erebia pronoe;0.997621582415603;
HQ004441;Lepidoptera;Erebia pronoe;Erebia pronoe;0.001;Erebia pronoe;0.998200106450327;
HQ004442;Lepidoptera;Erebia pronoe;Erebia pronoe;0.001;Erebia pronoe;0.997621582415603;
HQ004443;Lepidoptera;Erebia pronoe;Erebia pronoe;1;Erebia pronoe;0.999110793248495;
HQ004444;Lepidoptera;Erebia pronoe;Erebia pronoe;1;Erebia pronoe;0.998721231437303;
HQ004456;Lepidoptera;Erynnis tages;Erynnis tages;1;Erynnis tages;0.999998697902579;
HQ004457;Lepidoptera;Erynnis tages;Erynnis tages;1;Erynnis tages;0.999998697902579;
HQ004458;Lepidoptera;Erynnis tages;Erynnis tages;1;Erynnis tages;0.999998697902579;
HQ004459;Lepidoptera;Erynnis tages;Erynnis tages;1;Erynnis tages;0.999998697902579;
HQ004460;Lepidoptera;Erynnis tages;Erynnis tages;1;Erynnis tages;0.999999281987018;
HQ004461;Lepidoptera;Erynnis tages;Erynnis tages;1;Erynnis tages;0.999999281987018;
HQ004470;Lepidoptera;Aricia eumedon;Eumedonia eumedon;0.012;Eumedonia eumedon;0.993624989649652;
HQ004471;Lepidoptera;Aricia eumedon;Eumedonia eumedon;0.003;Eumedonia eumedon;0.996145222741686;
HQ004472;Lepidoptera;Aricia eumedon;Eumedonia eumedon;0.004;Eumedonia eumedon;0.995179062090393;
HQ004473;Lepidoptera;Aricia eumedon;Eumedonia eumedon;0.02;Eumedonia eumedon;0.994743149109561;
HQ004479;Lepidoptera;Euphydryas aurinia;NA;0.31;Euphydryas aurinia;0.993387708761386;
HQ004480;Lepidoptera;Euphydryas aurinia;NA;0.31;Euphydryas aurinia;0.993387708761386;
HQ004481;Lepidoptera;Euphydryas aurinia;NA;0.052;Euphydryas aurinia;0.996086749472406;
HQ004482;Lepidoptera;Euphydryas maturna;NA;0.125;Euphydryas maturna;0.861122566117855;
HQ004483;Lepidoptera;Euphydryas maturna;NA;0.067;Euphydryas maturna;0.719365084631966;
HQ004484;Lepidoptera;Euphydryas maturna;NA;NA;NA;0.999999999996973;
HQ004485;Lepidoptera;Euphydryas maturna;NA;NA;NA;0.999999999996973;
HQ004486;Lepidoptera;Euphydryas maturna;NA;NA;NA;0.999999999996973;
HQ004487;Lepidoptera;Euphydryas maturna;NA;0.043;Euphydryas maturna;0.800951977353662;
HQ004488;Lepidoptera;Euphydryas maturna;NA;0.043;Euphydryas maturna;0.800951977353662;
HQ004489;Lepidoptera;Euphydryas maturna;NA;NA;Euphydryas maturna;0.957925789833158;
HQ004490;Lepidoptera;Glaucopsyche alexis;NA;NA;Glaucopsyche alexis;0.979381169624069;
HQ004491;Lepidoptera;Glaucopsyche alexis;NA;NA;Glaucopsyche alexis;0.979381169624069;
HQ004492;Lepidoptera;Glaucopsyche alexis;NA;NA;Glaucopsyche alexis;0.979381169624069;
HQ004493;Lepidoptera;Glaucopsyche alexis;NA;NA;Glaucopsyche alexis;0.979381169624069;
HQ004494;Lepidoptera;Glaucopsyche alexis;NA;NA;Glaucopsyche alexis;0.979381169624069;
HQ004495;Lepidoptera;Glaucopsyche alexis;NA;NA;Glaucopsyche alexis;0.979381169624069;
HQ004496;Lepidoptera;Glaucopsyche alexis;NA;NA;Glaucopsyche alexis;0.979381169624069;

HQ004497;Lepidoptera;Glaucopsyche alexis;NA;NA;Glaucopsyche alexis;0.979381169624069;
HQ004498;Lepidoptera;Glaucopsyche alexis;NA;NA;Glaucopsyche alexis;0.979381169624069;
HQ004499;Lepidoptera;Glaucopsyche alexis;NA;0.13;Glaucopsyche alexis;0.982273953020636;
HQ004500;Lepidoptera;Glaucopsyche alexis;NA;NA;Glaucopsyche alexis;0.979381169624069;
HQ004501;Lepidoptera;Glaucopsyche alexis;NA;NA;Glaucopsyche alexis;0.899237597871183;
HQ004502;Lepidoptera;Gonepteryx rhamni;Gonepteryx rhamni;0.008;Gonepteryx rhamni;0.999527533936108;
HQ004503;Lepidoptera;Gonepteryx rhamni;NA;0.474;Gonepteryx rhamni;0.982076396127858;
HQ004504;Lepidoptera;Gonepteryx rhamni;NA;0.474;Gonepteryx rhamni;0.982076396127858;
HQ004505;Lepidoptera;Gonepteryx rhamni;Gonepteryx rhamni;0.02;Gonepteryx rhamni;0.994554206399161;
HQ004506;Lepidoptera;Gonepteryx rhamni;NA;0.474;Gonepteryx rhamni;0.982076396127858;
HQ004507;Lepidoptera;Gonepteryx rhamni;NA;0.474;Gonepteryx rhamni;0.982076396127858;
HQ004508;Lepidoptera;Hamearis lucina;NA;NA;Hamearis lucina;0.999999999984141;
HQ004509;Lepidoptera;Hamearis lucina;NA;NA;Hamearis lucina;0.999999999984141;
HQ004510;Lepidoptera;Hamearis lucina;NA;NA;Hamearis lucina;0.999999999964501;
HQ004511;Lepidoptera;Hamearis lucina;Hamearis lucina;1;Hamearis lucina;0.999999999922693;
HQ004512;Lepidoptera;Hamearis lucina;Hamearis lucina;1;Hamearis lucina;0.99999999996885;
HQ004513;Lepidoptera;Hamearis lucina;NA;NA;Hamearis lucina;0.999999999984141;
HQ004514;Lepidoptera;Hamearis lucina;NA;NA;Hamearis lucina;0.999999999984141;
HQ004515;Lepidoptera;Hamearis lucina;NA;NA;Hamearis lucina;0.999999999984141;
HQ004518;Lepidoptera;Heteropterus morpheus;NA;NA;Heteropterus morpheus;0.999999999999943;
HQ004520;Lepidoptera;Heteropterus morpheus;NA;NA;Heteropterus morpheus;0.999999999999943;
HQ004521;Lepidoptera;Hipparchia fagi;NA;NA;Hipparchia fagi;0.977048494324972;
HQ004522;Lepidoptera;Hipparchia fagi;NA;NA;Hipparchia fagi;0.97466470298983;
HQ004523;Lepidoptera;Hipparchia fagi;NA;NA;Hipparchia fagi;0.981425325990758;
HQ004524;Lepidoptera;Hipparchia fagi;NA;NA;Hipparchia fagi;0.977048494324972;
HQ004525;Lepidoptera;Hipparchia fagi;NA;NA;Hipparchia fagi;0.979223379082023;
HQ004526;Lepidoptera;Hipparchia fagi;NA;NA;Hipparchia fagi;0.977048494324972;
HQ004527;Lepidoptera;Hipparchia fagi;NA;NA;Hipparchia fagi;0.977048494324972;
HQ004528;Lepidoptera;Hipparchia semele;NA;0.001;Hipparchia semele;0.721904492156862;
HQ004529;Lepidoptera;Hipparchia semele;NA;0.002;Hipparchia semele;0.768561939711135;
HQ004530;Lepidoptera;Hipparchia semele;NA;0.002;Hipparchia semele;0.807780383202924;
HQ004531;Lepidoptera;Hipparchia semele;NA;0.002;Hipparchia semele;0.784603069619426;
HQ004532;Lepidoptera;Hipparchia semele;NA;NA;Hipparchia senthes;0.963652907198623;
HQ004533;Lepidoptera;Hipparchia semele;NA;0.002;Hipparchia semele;0.709178647127069;
HQ004534;Lepidoptera;Hipparchia semele;NA;0.002;Hipparchia semele;0.784603069619426;
HQ004535;Lepidoptera;Hipparchia semele;NA;0.002;Hipparchia semele;0.784603069619426;
HQ004536;Lepidoptera;Hipparchia semele;NA;0.002;Hipparchia semele;0.807780383202924;
HQ004537;Lepidoptera;Hipparchia statilinus;Hipparchia statilinus;1;Hipparchia statilinus;0.999999999999858;
HQ004538;Lepidoptera;Hipparchia statilinus;Hipparchia statilinus;1;Hipparchia statilinus;0.999999999999773;
HQ004558;Lepidoptera;Issoria lathonia;Issoria lathonia;1;Issoria lathonia;0.999999999989257;
HQ004559;Lepidoptera;Issoria lathonia;Issoria lathonia;1;Issoria lathonia;0.999999999989257;
HQ004560;Lepidoptera;Issoria lathonia;Issoria lathonia;1;Issoria lathonia;0.999999999982947;
HQ004561;Lepidoptera;Issoria lathonia;Issoria lathonia;1;Issoria lathonia;0.999999999989257;
HQ004562;Lepidoptera;Issoria lathonia;Issoria lathonia;1;Issoria lathonia;0.999999999989257;
HQ004563;Lepidoptera;Issoria lathonia;Issoria lathonia;1;Issoria lathonia;0.9999999999892;
HQ004564;Lepidoptera;Issoria lathonia;Issoria lathonia;1;Issoria lathonia;0.999999999989257;
HQ004565;Lepidoptera;Issoria lathonia;Issoria lathonia;1;Issoria lathonia;0.999999999989257;
HQ004566;Lepidoptera;Issoria lathonia;Issoria lathonia;1;Issoria lathonia;0.999999999989257;
HQ004567;Lepidoptera;Issoria lathonia;Issoria lathonia;1;Issoria lathonia;0.999999999989257;
HQ004572;Lepidoptera;Lampides boeticus;Lampides boeticus;1;Lampides boeticus;0.999999999545906;
HQ004573;Lepidoptera;Lampides boeticus;Lampides boeticus;1;Lampides boeticus;0.999999999545906;
HQ004574;Lepidoptera;Lampides boeticus;Lampides boeticus;1;Lampides boeticus;0.99999999942554;
HQ004575;Lepidoptera;Lasiommata maera;Lasiommata maera;1;Lasiommata maera;0.999962725146629;
HQ004576;Lepidoptera;Lasiommata maera;Lasiommata maera;1;Lasiommata maera;0.999999612368602;
HQ004577;Lepidoptera;Lasiommata maera;Lasiommata maera;1;Lasiommata maera;0.999997752443814;
HQ004578;Lepidoptera;Lasiommata maera;Lasiommata maera;1;Lasiommata maera;0.999998694479899;
HQ004579;Lepidoptera;Lasiommata maera;Lasiommata maera;1;Lasiommata maera;0.999997474408764;
HQ004580;Lepidoptera;Lasiommata maera;Lasiommata maera;1;Lasiommata maera;0.999999181072746;
HQ004581;Lepidoptera;Lasiommata maera;Lasiommata maera;1;Lasiommata maera;0.999999181072746;
HQ004582;Lepidoptera;Lasiommata maera;Lasiommata maera;1;Lasiommata maera;0.999999181072746;
HQ004583;Lepidoptera;Lasiommata megera;Lasiommata megera;1;Lasiommata megera;0.99994250542913;
HQ004584;Lepidoptera;Lasiommata megera;Lasiommata megera;1;Lasiommata megera;0.999928528239676;
HQ004585;Lepidoptera;Lasiommata megera;Lasiommata megera;1;Lasiommata megera;0.999928528239676;
HQ004586;Lepidoptera;Lasiommata megera;Lasiommata megera;1;Lasiommata megera;0.999928528239676;
HQ004587;Lepidoptera;Lasiommata megera;Lasiommata megera;1;Lasiommata megera;0.999923974308681;
HQ004588;Lepidoptera;Lasiommata megera;Lasiommata megera;1;Lasiommata megera;0.999928528239676;
HQ004589;Lepidoptera;Lasiommata megera;Lasiommata megera;1;Lasiommata megera;0.999928528239676;
HQ004622;Lepidoptera;Limenitis camilla;Limenitis camilla;0.01;Limenitis camilla;0.999988164408943;
HQ004623;Lepidoptera;Limenitis camilla;Limenitis camilla;0.01;Limenitis camilla;0.999988164408943;
HQ004624;Lepidoptera;Limenitis camilla;Limenitis camilla;0.01;Limenitis camilla;0.999988164408943;

HQ004625;Lepidoptera;Limenitis camilla;Limenitis camilla;0.009;Limenitis camilla;0.9999828626808;
HQ004627;Lepidoptera;Limenitis camilla;Limenitis camilla;0.001;Limenitis camilla;0.999982124723693;
HQ004635;Lepidoptera;Lycaena alciphron;Lycaena alciphron;1;Lycaena alciphron;0.999999768423036;
HQ004636;Lepidoptera;Lycaena alciphron;Lycaena alciphron;1;Lycaena alciphron;0.999999852270048;
HQ004637;Lepidoptera;Lycaena alciphron;Lycaena alciphron;1;Lycaena alciphron;0.999999860607612;
HQ004638;Lepidoptera;Lycaena alciphron;Lycaena alciphron;1;Lycaena alciphron;0.999999856207079;
HQ004639;Lepidoptera;Lycaena alciphron;Lycaena alciphron;1;Lycaena alciphron;0.999999860607612;
HQ004643;Lepidoptera;Lycaena dispar;Lycaena dispar;1;Lycaena dispar;0.999999990743959;
HQ004644;Lepidoptera;Lycaena dispar;Lycaena dispar;1;Lycaena dispar;0.999999992566444;
HQ004645;Lepidoptera;Lycaena dispar;Lycaena dispar;1;Lycaena dispar;0.999999990743959;
HQ004646;Lepidoptera;Lycaena dispar;Lycaena dispar;1;Lycaena dispar;0.999999990743959;
HQ004647;Lepidoptera;Lycaena dispar;Lycaena dispar;1;Lycaena dispar;0.999999992566444;
HQ004648;Lepidoptera;Lycaena dispar;Lycaena dispar;1;Lycaena dispar;0.999999990743959;
HQ004649;Lepidoptera;Lycaena dispar;Lycaena dispar;1;Lycaena dispar;0.999999990514283;
HQ004650;Lepidoptera;Lycaena dispar;Lycaena dispar;1;Lycaena dispar;0.999999990743959;
HQ004651;Lepidoptera;Lycaena dispar;Lycaena dispar;1;Lycaena dispar;0.999999990743959;
HQ004652;Lepidoptera;Helleia helle;Lycaena helle;1;Lycaena helle;0.999999993533947;
HQ004653;Lepidoptera;Helleia helle;Lycaena helle;1;Lycaena helle;0.999999993533947;
HQ004654;Lepidoptera;Helleia helle;Lycaena helle;1;Lycaena helle;0.999999993533947;
HQ004655;Lepidoptera;Helleia helle;Lycaena helle;1;Lycaena helle;0.999999993533947;
HQ004656;Lepidoptera;Helleia helle;Lycaena helle;1;Lycaena helle;0.999999993533947;
HQ004657;Lepidoptera;Helleia helle;Lycaena helle;1;Lycaena helle;0.999999982106601;
HQ004658;Lepidoptera;Lycaena hippothoe;NA;NA;Lycaena hippothoe;0.929268253497542;
HQ004659;Lepidoptera;Lycaena hippothoe;NA;NA;Lycaena hippothoe;0.929268253497542;
HQ004663;Lepidoptera;Lycaena phlaeas;Lycaena phlaeas;1;Lycaena phlaeas;0.999999994740023;
HQ004664;Lepidoptera;Lycaena phlaeas;Lycaena phlaeas;1;Lycaena phlaeas;0.999999996363186;
HQ004665;Lepidoptera;Lycaena phlaeas;Lycaena phlaeas;1;Lycaena phlaeas;0.999999994740023;
HQ004666;Lepidoptera;Lycaena phlaeas;Lycaena phlaeas;1;Lycaena phlaeas;0.999999994740023;
HQ004667;Lepidoptera;Lycaena phlaeas;Lycaena phlaeas;1;Lycaena phlaeas;0.999999994740023;
HQ004668;Lepidoptera;Lycaena phlaeas;Lycaena phlaeas;1;Lycaena phlaeas;0.99999998549427;
HQ004669;Lepidoptera;Lycaena phlaeas;Lycaena phlaeas;1;Lycaena phlaeas;0.999999995251216;
HQ004670;Lepidoptera;Lycaena phlaeas;Lycaena phlaeas;1;Lycaena phlaeas;0.999999994740023;
HQ004671;Lepidoptera;Lycaena phlaeas;Lycaena phlaeas;1;Lycaena phlaeas;0.999999996363186;
HQ004672;Lepidoptera;Lycaena thersamon;NA;NA;Lycaena thersamon;0.961302670565805;
HQ004673;Lepidoptera;Lycaena thersamon;NA;NA;Lycaena thersamon;0.961302670565805;
HQ004674;Lepidoptera;Lycaena thersamon;NA;NA;Lycaena thersamon;0.969135324846213;
HQ004675;Lepidoptera;Lycaena thersamon;NA;NA;Lycaena thersamon;0.95609400375869;
HQ004676;Lepidoptera;Lycaena thersamon;NA;NA;Lycaena thersamon;0.961302670565805;
HQ004677;Lepidoptera;Lycaena thersamon;NA;NA;Lycaena thersamon;0.961302670565805;
HQ004678;Lepidoptera;Lycaena thersamon;NA;NA;Lycaena thersamon;0.961302670565805;
HQ004679;Lepidoptera;Lycaena tityrus;Lycaena tityrus;1;Lycaena tityrus;0.999998928238034;
HQ004680;Lepidoptera;Lycaena tityrus;Lycaena tityrus;1;Lycaena tityrus;0.999999311170153;
HQ004681;Lepidoptera;Lycaena tityrus;Lycaena tityrus;1;Lycaena tityrus;0.999998928238034;
HQ004682;Lepidoptera;Lycaena tityrus;Lycaena tityrus;1;Lycaena tityrus;0.999999137688152;
HQ004683;Lepidoptera;Lycaena tityrus;Lycaena tityrus;1;Lycaena tityrus;0.999999137688152;
HQ004684;Lepidoptera;Lycaena tityrus;Lycaena tityrus;1;Lycaena tityrus;0.999998928238034;
HQ004685;Lepidoptera;Lycaena tityrus;Lycaena tityrus;1;Lycaena tityrus;0.999999137688152;
HQ004686;Lepidoptera;Lycaena tityrus;Lycaena tityrus;1;Lycaena tityrus;0.999997855580031;
HQ004687;Lepidoptera;Lycaena tityrus;Lycaena tityrus;1;Lycaena tityrus;0.999998928238034;
HQ004688;Lepidoptera;Lycaena virgaureae;NA;0.186;Lycaena virgaureae;0.999491227342155;
HQ004689;Lepidoptera;Lycaena virgaureae;Lycaena virgaureae;0.022;Lycaena virgaureae;0.998289731831646;
HQ004690;Lepidoptera;Lycaena virgaureae;NA;0.073;Lycaena virgaureae;0.987335569692494;
HQ004691;Lepidoptera;Lycaena virgaureae;NA;0.096;Lycaena virgaureae;0.998044906563439;
HQ004692;Lepidoptera;Lycaena virgaureae;Lycaena virgaureae;0.025;Lycaena virgaureae;0.999659709924644;
HQ004693;Lepidoptera;Lycaena virgaureae;NA;0.067;Lycaena virgaureae;0.999579233710412;
HQ004694;Lepidoptera;Lycaena virgaureae;Lycaena virgaureae;0.035;Lycaena virgaureae;0.999385274911075;
HQ004695;Lepidoptera;Lycaena virgaureae;Lycaena virgaureae;0.019;Lycaena virgaureae;0.999127655949914;
HQ004707;Lepidoptera;Phengaris arion;NA;0.021;Phengaris arion;0.956663122359086;
HQ004708;Lepidoptera;Phengaris arion;NA;0.054;Phengaris arion;0.938397095316026;
HQ004709;Lepidoptera;Phengaris arion;NA;0.054;Phengaris arion;0.938397095316026;
HQ004710;Lepidoptera;Phengaris arion;NA;0.021;Phengaris arion;0.956663122359086;
HQ004711;Lepidoptera;Phengaris arion;NA;0.054;Phengaris arion;0.938397095316026;
HQ004712;Lepidoptera;Phengaris arion;NA;0.054;Phengaris arion;0.938397095316026;
HQ004713;Lepidoptera;Phengaris arion;NA;0.054;Phengaris arion;0.938397095316026;
HQ004730;Lepidoptera;Maniola jurtina;NA;0.038;NA;0.999999999999524;
HQ004731;Lepidoptera;Maniola jurtina;NA;0.074;Maniola jurtina;0.734176111085427;
HQ004732;Lepidoptera;Maniola jurtina;NA;0.001;NA;0.999999999997362;
HQ004733;Lepidoptera;Maniola jurtina;NA;0.169;NA;0.999999999999202;
HQ004740;Lepidoptera;Melanargia galathea;Melanargia galathea;NA;Melanargia galathea;0.981855578470499;
HQ004741;Lepidoptera;Melanargia galathea;Melanargia galathea;NA;Melanargia galathea;0.981855578470499;

HQ004742;Lepidoptera;Melanargia galathea;Melanargia galathea;NA;Melanargia galathea;0.981855578470499;
HQ004743;Lepidoptera;Melanargia galathea;Melanargia galathea;NA;Melanargia galathea;0.981855578470499;
HQ004772;Lepidoptera;Melitaea britomartis;NA;NA;Melitaea britomartis;0.965147943123453;
HQ004773;Lepidoptera;Melitaea britomartis;NA;NA;Melitaea britomartis;0.964318779323939;
HQ004774;Lepidoptera;Melitaea cinxia;Melitaea cinxia;1;Melitaea cinxia;0.999999999999886;
HQ004775;Lepidoptera;Melitaea cinxia;Melitaea cinxia;1;Melitaea cinxia;0.999999999999488;
HQ004776;Lepidoptera;Melitaea cinxia;Melitaea cinxia;1;Melitaea cinxia;0.999999999999801;
HQ004777;Lepidoptera;Melitaea cinxia;Melitaea cinxia;1;Melitaea cinxia;0.999999999999574;
HQ004778;Lepidoptera;Melitaea cinxia;Melitaea cinxia;1;Melitaea cinxia;0.999999999999488;
HQ004779;Lepidoptera;Melitaea cinxia;Melitaea cinxia;1;Melitaea cinxia;0.999999999999915;
HQ004780;Lepidoptera;Melitaea cinxia;Melitaea cinxia;1;Melitaea cinxia;0.999999999999943;
HQ004781;Lepidoptera;Melitaea cinxia;Melitaea cinxia;1;Melitaea cinxia;0.999999999999488;
HQ004782;Lepidoptera;Melitaea diamina;NA;0.13;Melitaea diamina;0.999999982010166;
HQ004783;Lepidoptera;Melitaea diamina;NA;0.048;Melitaea diamina;0.999999987986001;
HQ004784;Lepidoptera;Melitaea diamina;NA;0.13;Melitaea diamina;0.999999982010166;
HQ004785;Lepidoptera;Melitaea diamina;NA;0.13;Melitaea diamina;0.999999982010166;
HQ004786;Lepidoptera;Melitaea diamina;NA;0.13;Melitaea diamina;0.999999982010166;
HQ004787;Lepidoptera;Melitaea diamina;NA;0.13;Melitaea diamina;0.999999982010166;
HQ004788;Lepidoptera;Melitaea diamina;NA;0.13;Melitaea diamina;0.999999982010166;
HQ004789;Lepidoptera;Melitaea didyma;Melitaea didyma;1;Melitaea didyma;0.994623940987323;
HQ004790;Lepidoptera;Melitaea didyma;Melitaea didyma;1;Melitaea didyma;0.993439843713843;
HQ004791;Lepidoptera;Melitaea didyma;Melitaea didyma;1;Melitaea didyma;0.993439843713843;
HQ004792;Lepidoptera;Melitaea didyma;Melitaea didyma;1;Melitaea didyma;0.993439843713843;
HQ004793;Lepidoptera;Melitaea didyma;Melitaea didyma;1;Melitaea didyma;0.993439843713843;
HQ004811;Lepidoptera;Melitaea trivia;Melitaea trivia;1;Melitaea trivia;0.999999999960949;
HQ004812;Lepidoptera;Melitaea trivia;Melitaea trivia;1;Melitaea trivia;0.999999999907146;
HQ004813;Lepidoptera;Melitaea trivia;Melitaea trivia;1;Melitaea trivia;0.999999999960949;
HQ004814;Lepidoptera;Melitaea trivia;Melitaea trivia;1;Melitaea trivia;0.999999999877389;
HQ004815;Lepidoptera;Melitaea trivia;Melitaea trivia;1;Melitaea trivia;0.999999999956572;
HQ004817;Lepidoptera;Melitaea trivia;Melitaea trivia;1;Melitaea trivia;0.999999999877389;
HQ004819;Lepidoptera;Minois dryas;NA;0.025;Minois dryas;0.850746026247397;
HQ004820;Lepidoptera;Minois dryas;NA;0.025;Minois dryas;0.850746026247397;
HQ004821;Lepidoptera;Minois dryas;NA;0.025;Minois dryas;0.850746026247397;
HQ004822;Lepidoptera;Minois dryas;NA;0.025;Minois dryas;0.850746026247397;
HQ004823;Lepidoptera;Minois dryas;NA;0.025;Minois dryas;0.850746026247397;
HQ004824;Lepidoptera;Minois dryas;NA;0.025;Minois dryas;0.850746026247397;
HQ004825;Lepidoptera;Minois dryas;NA;0.025;Minois dryas;0.850746026247397;
HQ004836;Lepidoptera;Favonius quercus;NA;NA;Favonius quercus;0.738637895579293;
HQ004837;Lepidoptera;Favonius quercus;NA;NA;Favonius quercus;0.729218423035651;
HQ004838;Lepidoptera;Favonius quercus;NA;NA;Favonius quercus;0.70740331639258;
HQ004839;Lepidoptera;Favonius quercus;NA;NA;NA;0.999999999999574;
HQ004858;Lepidoptera;Nymphalis antiopa;NA;0.23;Nymphalis antiopa;0.999999944198096;
HQ004874;Lepidoptera;Ochlodes sylvanus;NA;0.316;Ochlodes sylvanus;0.996318754838625;
HQ004875;Lepidoptera;Ochlodes sylvanus;NA;0.316;Ochlodes sylvanus;0.996318754838625;
HQ004876;Lepidoptera;Ochlodes sylvanus;NA;0.316;Ochlodes sylvanus;0.996318754838625;
HQ004877;Lepidoptera;Ochlodes sylvanus;NA;0.316;Ochlodes sylvanus;0.996318754838625;
HQ004878;Lepidoptera;Ochlodes sylvanus;NA;0.316;Ochlodes sylvanus;0.996318754838625;
HQ004879;Lepidoptera;Ochlodes sylvanus;NA;0.316;Ochlodes sylvanus;0.996318754838625;
HQ004880;Lepidoptera;Ochlodes sylvanus;NA;0.316;Ochlodes sylvanus;0.996318754838625;
HQ004881;Lepidoptera;Ochlodes sylvanus;NA;0.316;Ochlodes sylvanus;0.996318754838625;
HQ004882;Lepidoptera;Ochlodes sylvanus;NA;0.316;Ochlodes sylvanus;0.996318754838625;
HQ004883;Lepidoptera;Ochlodes sylvanus;NA;0.316;Ochlodes sylvanus;0.996318754838625;
HQ004891;Lepidoptera;Pararge aegeria;Pararge aegeria;1;Pararge aegeria;0.999999999806874;
HQ004892;Lepidoptera;Pararge aegeria;Pararge aegeria;1;Pararge aegeria;0.999999999836319;
HQ004893;Lepidoptera;Pararge aegeria;Pararge aegeria;1;Pararge aegeria;0.999999999963165;
HQ004894;Lepidoptera;Pararge aegeria;Pararge aegeria;1;Pararge aegeria;0.999999999930424;
HQ004895;Lepidoptera;Pararge aegeria;Pararge aegeria;1;Pararge aegeria;0.999999999836319;
HQ004896;Lepidoptera;Pararge aegeria;Pararge aegeria;1;Pararge aegeria;0.999999999963165;
HQ004897;Lepidoptera;Pararge aegeria;Pararge aegeria;1;Pararge aegeria;0.999999999904645;
HQ004898;Lepidoptera;Pararge aegeria;Pararge aegeria;1;Pararge aegeria;0.999999999963165;
HQ004899;Lepidoptera;Pararge aegeria;Pararge aegeria;1;Pararge aegeria;0.999999999836319;
HQ004900;Lepidoptera;Parnassius apollo;Parnassius apollo;1;Parnassius apollo;0.999611380084422;
HQ004901;Lepidoptera;Parnassius apollo;Parnassius apollo;0.001;Parnassius apollo;0.9652975019949;
HQ004902;Lepidoptera;Parnassius mnemosyne;Parnassius mnemosyne;1;Parnassius mnemosyne;0.999999789823726;
HQ004903;Lepidoptera;Parnassius mnemosyne;Parnassius mnemosyne;1;Parnassius mnemosyne;0.999999761646734;
HQ004904;Lepidoptera;Parnassius mnemosyne;Parnassius mnemosyne;1;Parnassius mnemosyne;0.999999761646734;
HQ004905;Lepidoptera;Parnassius mnemosyne;Parnassius mnemosyne;1;Parnassius mnemosyne;0.999999761646734;
HQ004906;Lepidoptera;Parnassius mnemosyne;Parnassius mnemosyne;1;Parnassius mnemosyne;0.999999761646734;
HQ004907;Lepidoptera;Parnassius mnemosyne;Parnassius mnemosyne;1;Parnassius mnemosyne;0.999999458035068;

HQ004908;Lepidoptera;Parnassius mnemosyne;Parnassius mnemosyne;1;Parnassius mnemosyne;0.999999766845291;
HQ004909;Lepidoptera;Parnassius mnemosyne;Parnassius mnemosyne;1;Parnassius mnemosyne;0.999999751412392;
HQ004910;Lepidoptera;Parnassius mnemosyne;Parnassius mnemosyne;1;Parnassius mnemosyne;0.999999761646734;
HQ004911;Lepidoptera;Parnassius mnemosyne;Parnassius mnemosyne;1;Parnassius mnemosyne;0.999999761646734;
HQ004939;Lepidoptera;Pieris napi;NA;NA;NA;100.000.000.000.001;
HQ004940;Lepidoptera;Pieris napi;NA;0.011;Pieris napi;0.801985394724814;
HQ004941;Lepidoptera;Pieris napi;NA;0.011;Pieris napi;0.801985394724814;
HQ004942;Lepidoptera;Pieris napi;NA;0.028;Pieris napi;0.888205720664699;
HQ004943;Lepidoptera;Pieris napi;NA;0.066;Pieris napi;0.832895481558215;
HQ004944;Lepidoptera;Pieris napi;NA;0.011;Pieris napi;0.911140198650123;
HQ004945;Lepidoptera;Pieris napi;NA;0.028;Pieris napi;0.888205720664699;
HQ004946;Lepidoptera;Pieris napi;NA;0.028;Pieris napi;0.888205720664699;
HQ004947;Lepidoptera;Pieris napi;NA;0.084;Pieris napi;0.885343420349066;
HQ004948;Lepidoptera;Pieris napi;NA;0.011;Pieris napi;0.801985394724814;
HQ004949;Lepidoptera;Pieris napi;NA;0.004;Pieris napi;0.759767822852292;
HQ004950;Lepidoptera;Pieris napi;NA;0.011;Pieris napi;0.801985394724814;
HQ004951;Lepidoptera;Pieris napi;NA;0.001;NA;0.999999999999955;
HQ004952;Lepidoptera;Pieris napi;NA;0.084;Pieris napi;0.885343420349066;
HQ004963;Lepidoptera;Plebejus argus;NA;0.123;Plebejus argus;0.980927835351298;
HQ004964;Lepidoptera;Plebejus argus;NA;0.123;Plebejus argus;0.980927835351298;
HQ004965;Lepidoptera;Plebejus argus;NA;0.123;Plebejus argus;0.980927835351298;
HQ004966;Lepidoptera;Plebejus argus;NA;NA;Plebejus argus;0.973283205133445;
HQ004967;Lepidoptera;Plebejus argus;NA;0.123;Plebejus argus;0.980927835351298;
HQ004968;Lepidoptera;Plebejus argus;NA;0.123;Plebejus argus;0.980927835351298;
HQ004969;Lepidoptera;Plebejus argus;NA;0.049;Plebejus argus;0.964369635730701;
HQ004970;Lepidoptera;Plebejus argus;NA;0.123;Plebejus argus;0.980927835351298;
HQ004971;Lepidoptera;Plebejus argus;NA;0.123;Plebejus argus;0.980927835351298;
HQ004972;Lepidoptera;Plebejus argus;NA;0.123;Plebejus argus;0.980927835351298;
HQ004973;Lepidoptera;Plebejus argus;NA;0.328;Plebejus argus;0.969789734199889;
HQ004974;Lepidoptera;Plebejus argus;NA;0.04;Plebejus argus;0.980859624104987;
HQ004975;Lepidoptera;Plebejus argus;NA;0.177;Plebejus argus;0.976301485545538;
HQ004976;Lepidoptera;Plebejus argus;NA;0.177;Plebejus argus;0.976301485545538;
HQ004977;Lepidoptera;Plebejus argyrognomon;NA;0.001;NA;0.994620093546684;
HQ004978;Lepidoptera;Plebejus argyrognomon;NA;0.002;NA;0.996348696700873;
HQ004979;Lepidoptera;Plebejus argyrognomon;NA;0.001;NA;0.994620093546684;
HQ004980;Lepidoptera;Plebejus argyrognomon;NA;0.001;NA;0.994620093546684;
HQ004981;Lepidoptera;Plebejus argyrognomon;NA;0.001;NA;0.994620093546684;
HQ004982;Lepidoptera;Plebejus argyrognomon;NA;0.001;NA;0.994620093546684;
HQ004983;Lepidoptera;Plebejus argyrognomon;NA;0.001;NA;0.994620093546684;
HQ004984;Lepidoptera;Plebejus argyrognomon;NA;0.001;NA;0.994620093546684;
HQ004985;Lepidoptera;Plebejus argyrognomon;NA;0.001;NA;0.994620093546684;
HQ004986;Lepidoptera;Plebejus argyrognomon;NA;0.001;NA;0.994620093546684;
HQ004987;Lepidoptera;Plebejus argyrognomon;NA;0.001;NA;0.994620093546684;
HQ004988;Lepidoptera;Plebejus argyrognomon;NA;0.001;NA;0.994620093546684;
HQ004989;Lepidoptera;Plebejus argyrognomon;NA;0.001;NA;0.994620093546684;
HQ004990;Lepidoptera;Plebejus argyrognomon;NA;0.001;NA;0.994620093546684;
HQ004991;Lepidoptera;Plebejus argyrognomon;NA;0.001;NA;0.994620093546684;
HQ004992;Lepidoptera;Plebejus idas;NA;0.007;NA;0.999546324331549;
HQ004993;Lepidoptera;Plebejus idas;NA;0.007;NA;0.999546324331549;
HQ005002;Lepidoptera;Nymphalis c-album;NA;0.215;NA;0.999999192918557;
HQ005003;Lepidoptera;Nymphalis c-album;NA;0.215;NA;0.999999192918557;
HQ005004;Lepidoptera;Nymphalis c-album;NA;0.215;NA;0.999999192918557;
HQ005005;Lepidoptera;Nymphalis c-album;NA;0.215;NA;0.999999192918557;
HQ005006;Lepidoptera;Nymphalis c-album;NA;0.215;NA;0.999999192918557;
HQ005007;Lepidoptera;Nymphalis c-album;NA;0.215;NA;0.999999192918557;
HQ005008;Lepidoptera;Nymphalis c-album;NA;0.215;NA;0.999999192918557;
HQ005009;Lepidoptera;Polyommatus amandus;Polyommatus amandus;0.002;Polyommatus amandus;0.999986997208719;
HQ005020;Lepidoptera;Lysandra coridon;Lysandra coridon;NA;Lysandra coridon;0.735779770406819;
HQ005021;Lepidoptera;Lysandra coridon;NA;NA;Lysandra coridon;0.754269905766234;
HQ005022;Lepidoptera;Lysandra coridon;NA;NA;Lysandra coridon;0.754269905766234;
HQ005023;Lepidoptera;Lysandra coridon;Lysandra coridon;NA;Lysandra coridon;0.735779770406819;
HQ005024;Lepidoptera;Lysandra coridon;Lysandra coridon;NA;Lysandra coridon;0.735779770406819;
HQ005025;Lepidoptera;Lysandra coridon;Lysandra coridon;NA;Lysandra coridon;0.868131209180917;
HQ005026;Lepidoptera;Lysandra coridon;NA;NA;Lysandra coridon;0.792716649139017;
HQ005027;Lepidoptera;Lysandra coridon;Lysandra coridon;NA;Lysandra coridon;0.735779770406819;
HQ005028;Lepidoptera;Lysandra coridon;Lysandra coridon;NA;Lysandra coridon;0.730574181508556;
HQ005029;Lepidoptera;Lysandra coridon;Lysandra coridon;NA;Lysandra coridon;0.735779770406819;
HQ005030;Lepidoptera;Lysandra coridon;NA;NA;NA;0.998946018519232;
HQ005032;Lepidoptera;Polyommatus daphnis;NA;0.06;Polyommatus daphnis;0.994947425490863;
HQ005033;Lepidoptera;Polyommatus daphnis;NA;0.062;Polyommatus daphnis;0.995724017767077;
HQ005034;Lepidoptera;Polyommatus daphnis;Polyommatus daphnis;0.028;Polyommatus daphnis;0.994856508659724;

HQ005035;Lepidoptera;Polyommatus daphnis;Polyommatus daphnis;0.023;Polyommatus daphnis;0.997907000385637;
HQ005036;Lepidoptera;Polyommatus daphnis;NA;0.002;Polyommatus daphnis;0.991545923418825;
HQ005037;Lepidoptera;Polyommatus daphnis;NA;0.163;Polyommatus daphnis;0.992967582982541;
HQ005038;Lepidoptera;Polyommatus dorylas;Polyommatus dorylas;0.001;Polyommatus dorylas;0.994481388857129;
HQ005039;Lepidoptera;Polyommatus dorylas;Polyommatus dorylas;0.008;Polyommatus dorylas;0.991281865899703;
HQ005040;Lepidoptera;Polyommatus dorylas;Polyommatus dorylas;0.008;Polyommatus dorylas;0.991281865899703;
HQ005041;Lepidoptera;Polyommatus dorylas;Polyommatus dorylas;0.008;Polyommatus dorylas;0.991281865899703;
HQ005042;Lepidoptera;Polyommatus dorylas;NA;0.002;Polyommatus dorylas;0.994430327300235;
HQ005043;Lepidoptera;Polyommatus dorylas;Polyommatus dorylas;0.008;Polyommatus dorylas;0.991281865899703;
HQ005044;Lepidoptera;Polyommatus dorylas;Polyommatus dorylas;0.008;Polyommatus dorylas;0.991281865899703;
HQ005045;Lepidoptera;Polyommatus dorylas;Polyommatus dorylas;0.008;Polyommatus dorylas;0.991281865899703;
HQ005046;Lepidoptera;Polyommatus icarus;NA;0.13;Polyommatus icarus;0.979762695748748;
HQ005047;Lepidoptera;Polyommatus icarus;NA;0.003;Polyommatus icarus;0.998348220031726;
HQ005048;Lepidoptera;Polyommatus icarus;NA;0.001;Polyommatus icarus;0.974455446201716;
HQ005049;Lepidoptera;Polyommatus icarus;NA;0.003;Polyommatus icarus;0.998348220031726;
HQ005050;Lepidoptera;Polyommatus icarus;NA;0.13;Polyommatus icarus;0.979762695748748;
HQ005051;Lepidoptera;Polyommatus icarus;NA;0.002;Polyommatus icarus;0.988825959464389;
HQ005052;Lepidoptera;Polyommatus icarus;NA;0.003;Polyommatus icarus;0.998348220031726;
HQ005053;Lepidoptera;Polyommatus icarus;NA;0.13;Polyommatus icarus;0.979762695748748;
HQ005054;Lepidoptera;Polyommatus icarus;NA;0.002;Polyommatus icarus;0.997043403297368;
HQ005055;Lepidoptera;Polyommatus icarus;Polyommatus icarus;0.027;Polyommatus icarus;0.985218928807075;
HQ005056;Lepidoptera;Polyommatus icarus;NA;0.13;Polyommatus icarus;0.979762695748748;
HQ005057;Lepidoptera;Polyommatus thersites;NA;0.11;Polyommatus thersites;0.999999518928236;
HQ005058;Lepidoptera;Polyommatus thersites;NA;0.33;Polyommatus thersites;0.999999477278829;
HQ005059;Lepidoptera;Polyommatus thersites;NA;0.066;Polyommatus thersites;0.999998480644439;
HQ005060;Lepidoptera;Polyommatus thersites;NA;NA;Polyommatus thersites;0.999999041958266;
HQ005061;Lepidoptera;Polyommatus thersites;Polyommatus thersites;0.001;Polyommatus thersites;0.999999937179697;
HQ005062;Lepidoptera;Polyommatus thersites;NA;NA;Polyommatus thersites;0.999999041958266;
HQ005091;Lepidoptera;Pyrgus alveus;NA;NA;Pyrgus alveus;0.941697735568124;
HQ005092;Lepidoptera;Pyrgus alveus;NA;NA;Pyrgus alveus;0.941697735568124;
HQ005093;Lepidoptera;Pyrgus alveus;NA;NA;Pyrgus alveus;0.941697735568124;
HQ005094;Lepidoptera;Pyrgus alveus;NA;NA;Pyrgus alveus;0.941697735568124;
HQ005095;Lepidoptera;Pyrgus alveus;NA;NA;Pyrgus alveus;0.941697735568124;
HQ005096;Lepidoptera;Pyrgus andromedae;Pyrgus andromedae;1;Pyrgus andromedae;0.999808671605833;
HQ005097;Lepidoptera;Pyrgus andromedae;Pyrgus andromedae;0.001;Pyrgus andromedae;0.997740560982143;
HQ005098;Lepidoptera;Pyrgus andromedae;Pyrgus andromedae;1;Pyrgus andromedae;0.999980225955564;
HQ005099;Lepidoptera;Pyrgus armoricanus;Pyrgus armoricanus;1;Pyrgus armoricanus;0.99999858288849;
HQ005100;Lepidoptera;Pyrgus armoricanus;Pyrgus armoricanus;1;Pyrgus armoricanus;0.999997353848817;
HQ005101;Lepidoptera;Pyrgus armoricanus;Pyrgus armoricanus;1;Pyrgus armoricanus;0.999997353848817;
HQ005102;Lepidoptera;Pyrgus armoricanus;Pyrgus armoricanus;1;Pyrgus armoricanus;0.999997353848817;
HQ005103;Lepidoptera;Pyrgus armoricanus;Pyrgus armoricanus;1;Pyrgus armoricanus;0.999997353848817;
HQ005104;Lepidoptera;Pyrgus armoricanus;Pyrgus armoricanus;1;Pyrgus armoricanus;0.999997353848817;
HQ005105;Lepidoptera;Pyrgus armoricanus;Pyrgus armoricanus;1;Pyrgus armoricanus;0.999997353848817;
HQ005106;Lepidoptera;Pyrgus armoricanus;Pyrgus armoricanus;1;Pyrgus armoricanus;0.997478680096412;
HQ005107;Lepidoptera;Pyrgus armoricanus;Pyrgus armoricanus;1;Pyrgus armoricanus;0.999997353848817;
HQ005108;Lepidoptera;Pyrgus armoricanus;Pyrgus armoricanus;1;Pyrgus armoricanus;0.99999858288849;
HQ005109;Lepidoptera;Pyrgus armoricanus;Pyrgus armoricanus;1;Pyrgus armoricanus;0.997478680096412;
HQ005110;Lepidoptera;Pyrgus armoricanus;Pyrgus armoricanus;1;Pyrgus armoricanus;0.999997637345223;
HQ005111;Lepidoptera;Pyrgus armoricanus;Pyrgus armoricanus;1;Pyrgus armoricanus;0.999997353848817;
HQ005112;Lepidoptera;Pyrgus armoricanus;Pyrgus armoricanus;1;Pyrgus armoricanus;0.997478680096412;
HQ005113;Lepidoptera;Pyrgus armoricanus;Pyrgus armoricanus;1;Pyrgus armoricanus;0.999997353848817;

HQ005114;Lepidoptera;Pyrgus armoricanus;Pyrgus armoricanus;1;Pyrgus armoricanus;0.999997353848817;
HQ005115;Lepidoptera;Pyrgus armoricanus;Pyrgus armoricanus;1;Pyrgus armoricanus;0.999997353848817;
HQ005116;Lepidoptera;Pyrgus armoricanus;Pyrgus armoricanus;1;Pyrgus armoricanus;0.999997353848817;
HQ005117;Lepidoptera;Pyrgus armoricanus;Pyrgus armoricanus;1;Pyrgus armoricanus;0.999997353848817;
HQ005118;Lepidoptera;Pyrgus armoricanus;Pyrgus armoricanus;1;Pyrgus armoricanus;0.999997353848817;
HQ005119;Lepidoptera;Pyrgus armoricanus;Pyrgus armoricanus;1;Pyrgus armoricanus;0.999997353848817;
HQ005120;Lepidoptera;Pyrgus armoricanus;Pyrgus armoricanus;1;Pyrgus armoricanus;0.999997353848817;
HQ005121;Lepidoptera;Pyrgus armoricanus;Pyrgus armoricanus;1;Pyrgus armoricanus;0.997478680096412;
HQ005122;Lepidoptera;Pyrgus cacaliae;Pyrgus cacaliae;1;Pyrgus cacaliae;0.996034079665744;
HQ005123;Lepidoptera;Pyrgus cacaliae;Pyrgus cacaliae;1;Pyrgus cacaliae;0.996034079665744;
HQ005124;Lepidoptera;Pyrgus cacaliae;Pyrgus cacaliae;1;Pyrgus cacaliae;0.998409529296952;
HQ005125;Lepidoptera;Pyrgus carthami;Pyrgus carthami;1;Pyrgus carthami;0.999947683373648;
HQ005126;Lepidoptera;Pyrgus carthami;Pyrgus carthami;1;Pyrgus carthami;0.999990719819382;
HQ005127;Lepidoptera;Pyrgus carthami;Pyrgus carthami;1;Pyrgus carthami;0.999990719819382;
HQ005128;Lepidoptera;Pyrgus carthami;Pyrgus carthami;1;Pyrgus carthami;0.999990719819382;
HQ005129;Lepidoptera;Pyrgus carthami;Pyrgus carthami;1;Pyrgus carthami;0.999990719819382;
HQ005130;Lepidoptera;Pyrgus carthami;Pyrgus carthami;1;Pyrgus carthami;0.999990719819382;
HQ005131;Lepidoptera;Pyrgus carthami;Pyrgus carthami;1;Pyrgus carthami;0.999990719819382;
HQ005132;Lepidoptera;Pyrgus malvae;Pyrgus malvae;0.999;Pyrgus malvae;0.993531783407179;
HQ005133;Lepidoptera;Pyrgus malvae;Pyrgus malvae;0.999;Pyrgus malvae;0.993531783407179;
HQ005134;Lepidoptera;Pyrgus malvae;Pyrgus malvae;0.003;Pyrgus malvae;0.993890103151852;
HQ005135;Lepidoptera;Pyrgus malvae;Pyrgus malvae;NA;Pyrgus malvae;0.980121140374433;
HQ005136;Lepidoptera;Pyrgus malvae;Pyrgus malvae;0.999;Pyrgus malvae;0.993531783407179;
HQ005137;Lepidoptera;Pyrgus malvae;Pyrgus malvae;0.999;Pyrgus malvae;0.993531783407179;
HQ005138;Lepidoptera;Pyrgus malvae;NA;NA;Pyrgus malvae;0.992160992679828;
HQ005139;Lepidoptera;Pyrgus malvae;Pyrgus malvae;0.999;Pyrgus malvae;0.993531783407179;
HQ005140;Lepidoptera;Pyrgus malvae;Pyrgus malvae;0.999;Pyrgus malvae;0.993531783407179;
HQ005143;Lepidoptera;Pyrgus sidae;Pyrgus sidae;1;Pyrgus sidae;0.99996750457282;
HQ005145;Lepidoptera;Pyrgus sidae;Pyrgus sidae;1;Pyrgus sidae;0.99996750457282;
HQ005146;Lepidoptera;Pyrgus sidae;Pyrgus sidae;1;Pyrgus sidae;0.99996750457282;
HQ005147;Lepidoptera;Pyrgus sidae;Pyrgus sidae;1;Pyrgus sidae;0.999993489463113;
HQ005148;Lepidoptera;Maniola tithonus;Pyronia tithonus;1;Pyronia tithonus;1;
HQ005149;Lepidoptera;Maniola tithonus;Pyronia tithonus;1;Pyronia tithonus;1;
HQ005150;Lepidoptera;Maniola tithonus;Pyronia tithonus;1;Pyronia tithonus;1;
HQ005151;Lepidoptera;Maniola tithonus;Pyronia tithonus;NA;Pyronia tithonus;1;
HQ005152;Lepidoptera;Maniola tithonus;Pyronia tithonus;1;Pyronia tithonus;1;
HQ005153;Lepidoptera;Maniola tithonus;Pyronia tithonus;1;Pyronia tithonus;1;
HQ005154;Lepidoptera;Maniola tithonus;Pyronia tithonus;NA;Pyronia tithonus;1;
HQ005155;Lepidoptera;Maniola tithonus;Pyronia tithonus;1;Pyronia tithonus;1;
HQ005156;Lepidoptera;Satyrium acaciae;Satyrium acaciae;1;Satyrium acaciae;0.999999965189347;
HQ005157;Lepidoptera;Satyrium acaciae;Satyrium acaciae;1;Satyrium acaciae;0.999999951077798;
HQ005158;Lepidoptera;Satyrium acaciae;Satyrium acaciae;1;Satyrium acaciae;0.999999906035616;
HQ005159;Lepidoptera;Satyrium acaciae;Satyrium acaciae;1;Satyrium acaciae;0.999999951077798;
HQ005160;Lepidoptera;Satyrium acaciae;Satyrium acaciae;1;Satyrium acaciae;0.999999951077798;
HQ005161;Lepidoptera;Satyrium acaciae;Satyrium acaciae;1;Satyrium acaciae;0.999999951077798;
HQ005162;Lepidoptera;Satyrium acaciae;Satyrium acaciae;1;Satyrium acaciae;0.999999951077798;
HQ005163;Lepidoptera;Satyrium acaciae;Satyrium acaciae;1;Satyrium acaciae;0.999999951077798;
HQ005164;Lepidoptera;Satyrium acaciae;Satyrium acaciae;1;Satyrium acaciae;0.999999951077798;
HQ005165;Lepidoptera;Satyrium acaciae;Satyrium acaciae;1;Satyrium acaciae;0.999999951077798;
HQ005166;Lepidoptera;Satyrium acaciae;Satyrium acaciae;1;Satyrium acaciae;0.999999951077798;
HQ005167;Lepidoptera;Satyrium ilicis;Satyrium ilicis;1;Satyrium ilicis;0.999999939891298;
HQ005168;Lepidoptera;Satyrium ilicis;Satyrium ilicis;1;Satyrium ilicis;0.999999991601669;
HQ005169;Lepidoptera;Satyrium ilicis;Satyrium ilicis;1;Satyrium ilicis;0.999999987901305;
HQ005170;Lepidoptera;Satyrium ilicis;Satyrium ilicis;1;Satyrium ilicis;0.999999963901758;
HQ005171;Lepidoptera;Satyrium ilicis;Satyrium ilicis;1;Satyrium ilicis;0.999999963901758;
HQ005172;Lepidoptera;Satyrium ilicis;Satyrium ilicis;1;Satyrium ilicis;0.999999991394333;
HQ005173;Lepidoptera;Satyrium ilicis;Satyrium ilicis;1;Satyrium ilicis;0.999999987901305;
HQ005174;Lepidoptera;Satyrium pruni;NA;0.076;Satyrium pruni;0.990322364021655;
HQ005175;Lepidoptera;Satyrium pruni;NA;0.32;Satyrium pruni;0.993871422369024;
HQ005176;Lepidoptera;Satyrium pruni;NA;0.076;Satyrium pruni;0.990322364021655;
HQ005177;Lepidoptera;Satyrium pruni;NA;0.076;Satyrium pruni;0.990322364021655;
HQ005178;Lepidoptera;Satyrium pruni;NA;0.32;Satyrium pruni;0.993871422369024;
HQ005179;Lepidoptera;Satyrium pruni;NA;0.32;Satyrium pruni;0.993871422369024;
HQ005180;Lepidoptera;Satyrium pruni;NA;0.076;Satyrium pruni;0.990322364021655;
HQ005181;Lepidoptera;Satyrium pruni;NA;0.076;Satyrium pruni;0.990322364021655;
HQ005182;Lepidoptera;Satyrium spini;Satyrium spini;1;Satyrium spini;0.999998550843001;
HQ005183;Lepidoptera;Satyrium spini;Satyrium spini;1;Satyrium spini;0.999999403222667;
HQ005184;Lepidoptera;Satyrium spini;Satyrium spini;1;Satyrium spini;0.999996396739488;
HQ005185;Lepidoptera;Satyrium spini;Satyrium spini;1;Satyrium spini;0.999998891040736;
HQ005186;Lepidoptera;Satyrium spini;Satyrium spini;1;Satyrium spini;0.999998428727123;

HQ005187;Lepidoptera;Satyrium spini;Satyrium spini;1;Satyrium spini;0.999999390354945;
HQ005188;Lepidoptera;Satyrium spini;Satyrium spini;1;Satyrium spini;0.999999352481961;
HQ005189;Lepidoptera;Satyrium spini;Satyrium spini;1;Satyrium spini;0.999998550843001;
HQ005190;Lepidoptera;Satyrium w-album;NA;0.056;Satyrium w-album;0.989378230996316;
HQ005191;Lepidoptera;Satyrium w-album;NA;0.056;Satyrium w-album;0.989378230996316;
HQ005192;Lepidoptera;Satyrium w-album;NA;0.056;Satyrium w-album;0.989378230996316;
HQ005193;Lepidoptera;Satyrium w-album;NA;0.056;Satyrium w-album;0.989378230996316;
HQ005194;Lepidoptera;Satyrium w-album;NA;0.056;Satyrium w-album;0.989378230996316;
HQ005195;Lepidoptera;Scolitantides orion;Scolitantides orion;1;Scolitantides orion;0.999999984804305;
HQ005196;Lepidoptera;Scolitantides orion;Scolitantides orion;1;Scolitantides orion;0.99999997690378;
HQ005197;Lepidoptera;Scolitantides orion;Scolitantides orion;1;Scolitantides orion;0.999999984804305;
HQ005198;Lepidoptera;Scolitantides orion;Scolitantides orion;1;Scolitantides orion;0.999999984804305;
HQ005199;Lepidoptera;Scolitantides orion;Scolitantides orion;1;Scolitantides orion;0.999999984804305;
HQ005200;Lepidoptera;Scolitantides orion;Scolitantides orion;1;Scolitantides orion;0.999999984804305;
HQ005201;Lepidoptera;Scolitantides orion;Scolitantides orion;1;Scolitantides orion;0.999999984804305;
HQ005212;Lepidoptera;Thecla betulae;Thecla betulae;1;Thecla betulae;0.999999999955833;
HQ005213;Lepidoptera;Thecla betulae;Thecla betulae;1;Thecla betulae;0.999999999896204;
HQ005214;Lepidoptera;Thecla betulae;Thecla betulae;1;Thecla betulae;0.999999999974392;
HQ005215;Lepidoptera;Thecla betulae;Thecla betulae;1;Thecla betulae;0.999999999974392;
HQ005216;Lepidoptera;Thecla betulae;Thecla betulae;1;Thecla betulae;0.999999999956742;
HQ005217;Lepidoptera;Thecla betulae;Thecla betulae;1;Thecla betulae;0.999999999961119;
HQ005218;Lepidoptera;Thecla betulae;Thecla betulae;1;Thecla betulae;0.999999999974818;
HQ005219;Lepidoptera;Thymelicus acteon;Thymelicus acteon;1;Thymelicus acteon;0.999999997710063;
HQ005220;Lepidoptera;Thymelicus acteon;Thymelicus acteon;1;Thymelicus acteon;0.99999999752248;
HQ005221;Lepidoptera;Thymelicus acteon;Thymelicus acteon;1;Thymelicus acteon;0.999999995105014;
HQ005222;Lepidoptera;Thymelicus lineola;Thymelicus lineola;1;Thymelicus lineola;0.999982844978955;
HQ005223;Lepidoptera;Thymelicus lineola;Thymelicus lineola;1;Thymelicus lineola;0.999982844978955;
HQ005224;Lepidoptera;Thymelicus lineola;Thymelicus lineola;1;Thymelicus lineola;0.999982844978955;
HQ005225;Lepidoptera;Thymelicus lineola;Thymelicus lineola;1;Thymelicus lineola;0.999971535093703;
HQ005226;Lepidoptera;Thymelicus lineola;Thymelicus lineola;1;Thymelicus lineola;0.99996903931991;
HQ005227;Lepidoptera;Thymelicus lineola;Thymelicus lineola;1;Thymelicus lineola;0.99996416095097;
HQ005228;Lepidoptera;Thymelicus sylvestris;Thymelicus sylvestris;1;Thymelicus sylvestris;0.99999876153052;
HQ005229;Lepidoptera;Thymelicus sylvestris;Thymelicus sylvestris;1;Thymelicus sylvestris;0.99999665642118;
HQ005230;Lepidoptera;Thymelicus sylvestris;NA;0.324;Thymelicus sylvestris;0.999940778442454;
HQ005231;Lepidoptera;Thymelicus sylvestris;Thymelicus sylvestris;1;Thymelicus sylvestris;0.99999876153052;
HQ005232;Lepidoptera;Thymelicus sylvestris;Thymelicus sylvestris;1;Thymelicus sylvestris;0.99999665642118;
HQ005233;Lepidoptera;Thymelicus sylvestris;Thymelicus sylvestris;1;Thymelicus sylvestris;0.99999665642118;
HQ005234;Lepidoptera;Thymelicus sylvestris;NA;0.324;Thymelicus sylvestris;0.999940778442454;
HQ005235;Lepidoptera;Thymelicus sylvestris;NA;0.324;Thymelicus sylvestris;0.999940778442454;
HQ005236;Lepidoptera;Thymelicus sylvestris;Thymelicus sylvestris;1;Thymelicus sylvestris;0.99999876153052;
HQ005237;Lepidoptera;Thymelicus sylvestris;NA;0.324;Thymelicus sylvestris;0.999940778442454;
HQ005238;Lepidoptera;Thymelicus sylvestris;NA;0.324;Thymelicus sylvestris;0.999940778442454;
HQ005239;Lepidoptera;Thymelicus sylvestris;NA;0.324;Thymelicus sylvestris;0.999940778442454;
HQ005240;Lepidoptera;Thymelicus sylvestris;Thymelicus sylvestris;1;Thymelicus sylvestris;0.999998759716989;
HQ005241;Lepidoptera;Thymelicus sylvestris;NA;0.135;Thymelicus sylvestris;0.999944477624781;
HQ005242;Lepidoptera;Thymelicus sylvestris;NA;0.131;Thymelicus sylvestris;0.999961574491035;
HQ005243;Lepidoptera;Thymelicus sylvestris;Thymelicus sylvestris;1;Thymelicus sylvestris;0.99999876153052;
HQ005244;Lepidoptera;Thymelicus sylvestris;NA;0.131;Thymelicus sylvestris;0.999961574491035;
HQ005247;Lepidoptera;Vanessa atalanta;Vanessa atalanta;1;Vanessa atalanta;0.999999999757762;
HQ005248;Lepidoptera;Vanessa atalanta;Vanessa atalanta;1;Vanessa atalanta;0.999999999757762;
HQ005249;Lepidoptera;Vanessa atalanta;Vanessa atalanta;1;Vanessa atalanta;0.999999999757762;
HQ005250;Lepidoptera;Vanessa atalanta;Vanessa atalanta;1;Vanessa atalanta;0.999999999757762;
HQ005251;Lepidoptera;Vanessa atalanta;Vanessa atalanta;1;Vanessa atalanta;0.99999999952658;
HQ005252;Lepidoptera;Vanessa atalanta;Vanessa atalanta;1;Vanessa atalanta;0.999999999669399;
HQ005253;Lepidoptera;Vanessa atalanta;Vanessa atalanta;1;Vanessa atalanta;0.999999999751964;
HQ005254;Lepidoptera;Vanessa atalanta;Vanessa atalanta;1;Vanessa atalanta;0.999999999383476;
HQ005255;Lepidoptera;Vanessa atalanta;Vanessa atalanta;1;Vanessa atalanta;0.999999999757762;

HQ005256;Lepidoptera;Vanessa cardui;Vanessa cardui;1;Vanessa cardui;0.999999803450484;
HQ005257;Lepidoptera;Vanessa cardui;Vanessa cardui;1;Vanessa cardui;0.999999459983318;
HQ005258;Lepidoptera;Vanessa cardui;Vanessa cardui;1;Vanessa cardui;0.999999803450484;
HQ005259;Lepidoptera;Vanessa cardui;Vanessa cardui;1;Vanessa cardui;0.999999459983318;
HQ005260;Lepidoptera;Vanessa cardui;Vanessa cardui;1;Vanessa cardui;0.999999803450484;
HQ562781;Lepidoptera;Melitaea didyma;Melitaea didyma;1;Melitaea didyma;0.99976480414171;
HQ562782;Lepidoptera;Melitaea didyma;Melitaea didyma;1;Melitaea didyma;0.99976480414171;
HQ562783;Lepidoptera;Melitaea didyma;Melitaea didyma;1;Melitaea didyma;0.999515490426369;
HQ562784;Lepidoptera;Favonius quercus;NA;NA;Favonius quercus;0.754098981100012;
HQ562785;Lepidoptera;Carcharodus alceae;Carcharodus alceae;1;Carcharodus alceae;0.999999998465938;
HQ562786;Lepidoptera;Carterocephalus palaemon;NA;0.052;Carterocephalus palaemon;0.728445051795325;
HQ563537;Lepidoptera;Erebia tyndarus;NA;NA;NA;0.999999999999923;
HQ563538;Lepidoptera;Aricia agestis;NA;0.253;Aricia agestis;0.996350017387504;
HQ563539;Lepidoptera;Pyrgus alveus;NA;0.003;NA;0.999999999997732;
HQ563540;Lepidoptera;Erebia tyndarus;NA;NA;NA;0.999999999999923;
HQ563541;Lepidoptera;Aricia artaxerxes;Plebejus argus;1;Aricia artaxerxes;0.999743187199922;
HQ563542;Lepidoptera;Erebia tyndarus;NA;NA;NA;0.999999999999923;
HQ563543;Lepidoptera;Pyrgus alveus;NA;0.003;NA;0.999999999997732;
HQ563544;Lepidoptera;Pyrgus alveus;NA;NA;Pyrgus alveus;0.902591602214502;
HQ563545;Lepidoptera;Pyrgus alveus;NA;NA;Pyrgus alveus;0.827119616974978;
HQ563549;Lepidoptera;Aricia agestis;NA;0.253;Aricia agestis;0.996350017387504;
HQ563550;Lepidoptera;Polyommatus amandus;NA;0.249;Polyommatus amandus;0.997279404062746;
HQ563551;Lepidoptera;Aricia artaxerxes;Plebejus argus;1;Aricia artaxerxes;0.999743187199922;
HQ563552;Lepidoptera;Cyaniris semiargus;NA;NA;Cyaniris semiargus;0.997097672202902;
HQ563553;Lepidoptera;Lysandra coridon;NA;NA;NA;0.998070184737648;
HQ563554;Lepidoptera;Lysandra coridon;NA;NA;NA;0.998070184737648;
HQ563555;Lepidoptera;Polyommatus icarus;NA;0.002;Polyommatus icarus;0.997871588783533;
HQ563556;Lepidoptera;Cyaniris semiargus;NA;NA;Cyaniris semiargus;0.999051841766953;
HQ563557;Lepidoptera;Pyrgus alveus;NA;0.003;NA;0.999999999997732;
HQ563558;Lepidoptera;Pyrgus alveus;NA;0.003;NA;0.999999999997732;
HQ563559;Lepidoptera;Pyrgus alveus;NA;NA;Pyrgus alveus;0.827119616974978;
HQ563560;Lepidoptera;Thymelicus acteon;Thymelicus acteon;1;Thymelicus acteon;0.99999999752248;
HQ563561;Lepidoptera;Pyrgus warrenensis;NA;0.003;NA;0.999999999997732;
HQ563563;Lepidoptera;Thymelicus lineola;Thymelicus lineola;1;Thymelicus lineola;0.999995927238524;
HQ563564;Lepidoptera;Carterocephalus palaemon;NA;0.052;Carterocephalus palaemon;0.728445051795325;
HQ563565;Lepidoptera;Pyrgus malvae;Pyrgus malvae;NA;Pyrgus malvae;0.980121140374433;
HQ563566;Lepidoptera;Pyrgus carthami;Pyrgus carthami;1;Pyrgus carthami;0.999997085657879;
HQ563569;Lepidoptera;Aricia artaxerxes;Plebejus argus;1;Aricia artaxerxes;0.999743187199922;
HQ563570;Lepidoptera;Pyrgus alveus;NA;NA;Pyrgus alveus;0.962152887824497;
HQ563571;Lepidoptera;Pyrgus alveus;NA;NA;Pyrgus alveus;0.827119616974978;
HQ563572;Lepidoptera;Aricia artaxerxes;Plebejus argus;1;Aricia artaxerxes;0.999743187199922;
HQ563574;Lepidoptera;Aricia artaxerxes;Plebejus argus;1;Aricia artaxerxes;0.999743187199922;
HQ563576;Lepidoptera;Aricia artaxerxes;Plebejus argus;1;Aricia artaxerxes;0.999743187199922;
HQ563577;Lepidoptera;Pyrgus alveus;NA;NA;Pyrgus alveus;0.902591602214502;
HQ563578;Lepidoptera;Aricia agestis;NA;0.067;Aricia agestis;0.999509743227786;
HQ563579;Lepidoptera;Pyrgus alveus;NA;NA;Pyrgus alveus;0.902591602214502;
HQ563580;Lepidoptera;Pyrgus alveus;NA;NA;Pyrgus alveus;0.827119616974978;
HQ563581;Lepidoptera;Aricia artaxerxes;Plebejus argus;0.004;Aricia artaxerxes;0.99904771879725;
HQ563582;Lepidoptera;Pyrgus alveus;NA;NA;Pyrgus alveus;0.827119616974978;
HQ563583;Lepidoptera;Pyrgus alveus;NA;NA;NA;0.999999999887642;
HQ563584;Lepidoptera;Pyrgus alveus;NA;NA;Pyrgus alveus;0.902591602214502;
HQ563585;Lepidoptera;Pyrgus alveus;NA;0.003;NA;0.999999999997732;
HQ563608;Lepidoptera;Issoria lathonia;Issoria lathonia;1;Issoria lathonia;0.999999999989257;
HQ565464;Lepidoptera;Colias palaeno;NA;0.001;NA;0.999999999982869;
HQ565465;Lepidoptera;Apatura iris;Apatura iris;1;Apatura iris;0.999999999999972;
HQ565466;Lepidoptera;Apatura ilia;NA;0.165;Apatura ilia;0.956796812031481;
HQ565468;Lepidoptera;Argynnis aglaja;NA;0.001;NA;0.999999999999586;
HQ565472;Lepidoptera;Melitaea britomartis;Mellicta britomartis;NA;Melitaea britomartis;0.933869879268478;
HQ565491;Lepidoptera;Euphydryas aurinia;NA;0.194;Euphydryas aurinia;0.985201226652523;
HQ565493;Lepidoptera;Coenonympha arcania;NA;0.004;NA;0.999999999999983;
HQ565495;Lepidoptera;Erebia tyndarus;NA;NA;NA;0.999999999999923;
HQ565496;Lepidoptera;Oeneis glacialis;NA;NA;Oeneis glacialis;0.892833742436513;
HQ570286;Lepidoptera;Boloria thore;Boloria thore;1;Boloria thore;0.999999998091482;
HQ570288;Lepidoptera;Colias palaeno;NA;0.002;NA;0.999999999983681;
HQ570386;Lepidoptera;Pyrgus andromedae;Pyrgus andromedae;1;Pyrgus andromedae;0.999945721009741;
HQ570388;Lepidoptera;Erebia medusa;NA;0.009;Erebia medusa;0.986275612856827;
HQ570389;Lepidoptera;Erebia medusa;NA;0.009;Erebia medusa;0.986275612856827;
HQ955418;Lepidoptera;Satyrium spini;Satyrium spini;1;Satyrium spini;0.999999835426407;
HQ957206;Lepidoptera;Anthocharis cardamines;Anthocharis cardamines;1;Anthocharis cardamines;0.999999904052946;
HQ957207;Lepidoptera;Anthocharis cardamines;Anthocharis cardamines;1;Anthocharis cardamines;0.999999962791663;
HQ957209;Lepidoptera;Gonepteryx rhamni;NA;0.474;Gonepteryx rhamni;0.982076396127858;

HQ957210;Lepidoptera;Polyommatus dorylas;Polyommatus dorylas;1;Polyommatus dorylas;0.998759837233869;
HQ957212;Lepidoptera;Aricia artaxerxes;Plebejus argus;1;Aricia artaxerxes;0.999743187199922;
HQ957213;Lepidoptera;Pyrgus cacaliae;Pyrgus cacaliae;0.003;Pyrgus cacaliae;0.998434964230306;
HQ957216;Lepidoptera;Limenitis camilla;Limenitis camilla;0.999;Limenitis camilla;0.999953690065568;
HQ957217;Lepidoptera;Limenitis camilla;Limenitis camilla;1;Limenitis camilla;0.999939679225788;
HQ957267;Lepidoptera;Aricia agestis;NA;0.253;Aricia agestis;0.996350017387504;
HQ957268;Lepidoptera;Aricia agestis;NA;0.253;Aricia agestis;0.996350017387504;
HQ957269;Lepidoptera;Aricia agestis;NA;0.253;Aricia agestis;0.996350017387504;
HQ957270;Lepidoptera;Aricia agestis;NA;0.415;Aricia agestis;0.995575163376746;
HQ957502;Lepidoptera;Hamearis lucina;NA;NA;Hamearis lucina;0.999999999984141;
HQ957503;Lepidoptera;Limenitis camilla;Limenitis camilla;1;Limenitis camilla;0.999962628579717;
HQ968286;Lepidoptera;Agriades glandon;NA;0.002;Agriades glandon;0.987189585708506;
HQ968287;Lepidoptera;Agriades glandon;NA;0.002;Agriades glandon;0.987189585708506;
HQ968288;Lepidoptera;Agriades glandon;Agriades glandon;NA;Agriades glandon;0.989560338766428;
HQ968289;Lepidoptera;Agriades optilete;Agriades optilete;1;Agriades optilete;0.9999999864568;
HQ968293;Lepidoptera;Boloria thore;Boloria thore;1;Boloria thore;0.999999998359726;
HQ968296;Lepidoptera;Boloria titania;Boloria titania;0.001;Boloria titania;0.99983358374111;
HQ968297;Lepidoptera;Boloria titania;Boloria titania;0.001;Boloria titania;0.99983358374111;
HQ968298;Lepidoptera;Boloria titania;Boloria titania;0.001;Boloria titania;0.99983358374111;
HQ968299;Lepidoptera;Erebia pandrose;Erebia pandrose;0.013;Erebia pandrose;0.974388092470716;
HQ968300;Lepidoptera;Boloria pales;Boloria pales;1;Boloria pales;0.998071007441061;
HQ968301;Lepidoptera;Boloria pales;Boloria pales;1;Boloria pales;0.997102300597465;
HQ968302;Lepidoptera;Boloria pales;Boloria pales;1;Boloria pales;0.999550793226151;
HQ968430;Lepidoptera;Melitaea varia;Melitaea varia;1;Melitaea varia;0.999997686496646;
HQ968431;Lepidoptera;Melitaea varia;Melitaea varia;1;Melitaea varia;0.999996867427246;
HQ968432;Lepidoptera;Coenonympha glycerion;NA;0.28;Coenonympha glycerion;0.999998036263913;
HQ968433;Lepidoptera;Erebia epiphron;NA;NA;Erebia epiphron;0.997605378796729;
HQ968454;Lepidoptera;Lycaena tityrus;Lycaena tityrus;1;Lycaena tityrus;0.999991689073969;
HQ968455;Lepidoptera;Oeneis glacialis;NA;NA;Oeneis glacialis;0.894225823747824;
HQ968460;Lepidoptera;Boloria titania;Boloria titania;0.001;Boloria titania;0.99983358374111;
HQ968461;Lepidoptera;Euphydryas aurinia;NA;0.31;Euphydryas aurinia;0.993387708761386;
HQ968462;Lepidoptera;Euphydryas aurinia;NA;0.298;Euphydryas aurinia;0.981888414352726;
HQ968463;Lepidoptera;Callophrys rubi;NA;0.001;Callophrys rubi;0.99870518510598;
HQ968464;Lepidoptera;Callophrys rubi;NA;0.001;Callophrys rubi;0.99870518510598;
HQ968465;Lepidoptera;Coenonympha gardetta;NA;0.001;Coenonympha gardetta;0.731095812176462;
HQ968466;Lepidoptera;Cupido minimus;NA;0.001;Cupido minimus;0.999828420231565;
HQ968472;Lepidoptera;Aricia artaxerxes;Plebejus argus;1;Aricia artaxerxes;0.999671471677394;
HQ968473;Lepidoptera;Pyrgus andromedae;Pyrgus andromedae;0.001;Pyrgus andromedae;0.998828669843251;
HQ968474;Lepidoptera;Aricia eumedon;Eumedonia eumedon;0.003;Eumedonia eumedon;0.997682558007858;
HQ968475;Lepidoptera;Aricia eumedon;Eumedonia eumedon;0.006;Eumedonia eumedon;0.997094629310374;
HQ968476;Lepidoptera;Lycaena tityrus;Lycaena tityrus;1;Lycaena tityrus;0.999998928238034;
HQ968477;Lepidoptera;Erebia epiphron;Erebia epiphron;NA;Erebia epiphron;0.998837748029833;
HQ968478;Lepidoptera;Erebia medusa;NA;0.037;Erebia medusa;0.967935760207416;
HQ968479;Lepidoptera;Erebia meolans;Erebia meolans;NA;Erebia meolans;0.999888168778318;
HQ968480;Lepidoptera;Erebia meolans;Erebia meolans;0.999;Erebia meolans;0.999893587788713;
HQ968481;Lepidoptera;Erebia meolans;Erebia meolans;NA;Erebia meolans;0.999888168778318;
HQ968482;Lepidoptera;Erebia epiphron;Erebia epiphron;NA;Erebia epiphron;0.998837748029833;
HQ968483;Lepidoptera;Erebia epiphron;Erebia epiphron;NA;Erebia epiphron;0.998837748029833;
HQ968484;Lepidoptera;Erebia melampus;Erebia melampus;0.011;Erebia melampus;0.975499903768874;
HQ968485;Lepidoptera;Coenonympha gardetta;NA;0.12;NA;0.999999999999993;
HQ968486;Lepidoptera;Aricia artaxerxes;Plebejus argus;1;Aricia artaxerxes;0.999707730914532;
HQ968489;Lepidoptera;Araschnia levana;Araschnia levana;1;Araschnia levana;0.999999772187832;
HQ968506;Lepidoptera;Euphydryas aurinia;NA;0.31;Euphydryas aurinia;0.993387708761386;
HQ968508;Lepidoptera;Erebia manto;NA;0.179;Erebia manto;0.9978825939314;
HQ968509;Lepidoptera;Erebia manto;NA;0.192;Erebia manto;0.997710988231194;
HQ968510;Lepidoptera;Erebia pronoe;Erebia pronoe;NA;Erebia pronoe;0.906909765449499;
HQ968511;Lepidoptera;Erebia medusa;NA;0.037;Erebia medusa;0.967935760207416;
HQ968512;Lepidoptera;Erebia epiphron;Erebia epiphron;NA;Erebia epiphron;0.998837748029833;
HQ968516;Lepidoptera;Melitaea cinxia;Melitaea cinxia;1;Melitaea cinxia;0.999999999999801;
HQ968517;Lepidoptera;Aricia eumedon;Eumedonia eumedon;0.002;Eumedonia eumedon;0.997434540282035;
HQ968520;Lepidoptera;Lycaena tityrus;Lycaena tityrus;1;Lycaena tityrus;0.999997027007808;
HQ968522;Lepidoptera;Erebia epiphron;NA;NA;Erebia epiphron;0.994401488812176;
HQ968523;Lepidoptera;Euphydryas aurinia;NA;0.31;Euphydryas aurinia;0.993387708761386;
HQ968524;Lepidoptera;Aricia artaxerxes;NA;0.394;Aricia artaxerxes;0.999502890976379;
HQ968525;Lepidoptera;Carterocephalus palaemon;Carterocephalus palaemon;0.01;Carterocephalus palaemon;0.817246099879953;
HQ968526;Lepidoptera;Erebia euryale;NA;NA;Erebia euryale;0.907478661508288;
HQ968527;Lepidoptera;Colias phicomone;NA;0.001;Colias phicomone;0.985598879697681;
HQ968528;Lepidoptera;Erebia pandrose;Erebia pandrose;0.013;Erebia pandrose;0.974388092470716;
HQ968529;Lepidoptera;Euphydryas aurinia;NA;0.31;Euphydryas aurinia;0.993387708761386;
HQ968530;Lepidoptera;Coenonympha gardetta;NA;0.001;NA;0.99999999999997;
HQ968531;Lepidoptera;Erebia tyndarus;NA;NA;NA;0.999999999999923;
HQ968532;Lepidoptera;Melitaea varia;Melitaea varia;1;Melitaea varia;0.999992527121135;
HQ968533;Lepidoptera;Erebia mnestra;Erebia mnestra;0.002;Erebia mnestra;0.999848876047927;
HQ968534;Lepidoptera;Erebia mnestra;Erebia mnestra;0.002;Erebia mnestra;0.999845899963913;

HQ968535;Lepidoptera;Melitaea varia;Melitaea varia;1;Melitaea varia;0.999992527121135;
HQ996661;Lepidoptera;Pyrgus sidae;Pyrgus sidae;1;Pyrgus sidae;0.999993672336597;
HQ996662;Lepidoptera;Pyrgus sidae;Pyrgus sidae;1;Pyrgus sidae;0.999993672336597;
HQ996664;Lepidoptera;Pyrgus sidae;Pyrgus sidae;1;Pyrgus sidae;0.999968332724737;
ISSWG153-21;Lepidoptera;Thymelicus lineola;Thymelicus lineola;1;Thymelicus lineola;0.999991813825713;
ISSWG245-21;Lepidoptera;Thymelicus lineola;Thymelicus lineola;1;Thymelicus lineola;0.999975085149331;
JF262055;Lepidoptera;Glaucopsyche melanops;Glaucopsyche melanops;1;NA;0.700009170980357;
JF283325;Lepidoptera;Pyrgus carthami;Pyrgus carthami;1;Pyrgus carthami;0.999997085657879;
JF283344;Lepidoptera;Pyrgus sidae;Pyrgus sidae;1;Pyrgus sidae;0.99999495957445;
JF283347;Lepidoptera;Pyrgus sidae;Pyrgus sidae;1;Pyrgus sidae;0.999959757681902;
JF283350;Lepidoptera;Pyrgus sidae;Pyrgus sidae;1;Pyrgus sidae;0.999994277634217;
JF283351;Lepidoptera;Pyrgus sidae;Pyrgus sidae;1;Pyrgus sidae;0.99996750457282;
JF283352;Lepidoptera;Pyrgus sidae;Pyrgus sidae;1;Pyrgus sidae;0.999994277634217;
JF283353;Lepidoptera;Pyrgus sidae;Pyrgus sidae;1;Pyrgus sidae;0.999994277634217;
JF283354;Lepidoptera;Pyrgus sidae;Pyrgus sidae;1;Pyrgus sidae;0.999994277634217;
JF283355;Lepidoptera;Pyrgus sidae;Pyrgus sidae;1;Pyrgus sidae;0.999994277634217;
JF283356;Lepidoptera;Pyrgus sidae;Pyrgus sidae;1;Pyrgus sidae;0.999984781588847;
JF283357;Lepidoptera;Pyrgus sidae;Pyrgus sidae;1;Pyrgus sidae;0.999994277634217;
JF283371;Lepidoptera;Pyrgus sidae;Pyrgus sidae;1;Pyrgus sidae;0.99999311394709;
JF283372;Lepidoptera;Pyrgus sidae;Pyrgus sidae;1;Pyrgus sidae;0.99999311394709;
JF283376;Lepidoptera;Pyrgus sidae;Pyrgus sidae;1;Pyrgus sidae;0.999991149939742;
JF283382;Lepidoptera;Pyrgus sidae;Pyrgus sidae;1;Pyrgus sidae;0.999996542615443;
JF283389;Lepidoptera;Pyrgus sidae;Pyrgus sidae;1;Pyrgus sidae;0.999994277634217;
JF283390;Lepidoptera;Pyrgus sidae;Pyrgus sidae;1;Pyrgus sidae;0.999994277634217;
JF283391;Lepidoptera;Pyrgus sidae;Pyrgus sidae;1;Pyrgus sidae;0.999994277634217;
JF283392;Lepidoptera;Pyrgus sidae;Pyrgus sidae;1;Pyrgus sidae;0.999994277634217;
JF283393;Lepidoptera;Pyrgus sidae;Pyrgus sidae;1;Pyrgus sidae;0.999994277634217;
JF415668;Lepidoptera;Aporia crataegi;NA;NA;Aporia crataegi;0.999998459680561;
JF415669;Lepidoptera;Fabriciana adippe;Fabriciana adippe;1;Fabriciana adippe;0.995947626017421;
JF415670;Lepidoptera;Fabriciana adippe;Fabriciana adippe;1;Fabriciana adippe;0.995429620697469;
JF415671;Lepidoptera;Aricia agestis;NA;0.253;Aricia agestis;0.996350017387504;
JF415672;Lepidoptera;Aricia agestis;NA;0.253;Aricia agestis;0.996350017387504;
JF415673;Lepidoptera;Aricia agestis;NA;0.253;Aricia agestis;0.996350017387504;
JF415674;Lepidoptera;Aricia agestis;NA;0.253;Aricia agestis;0.996350017387504;
JF415675;Lepidoptera;Aricia agestis;NA;0.253;Aricia agestis;0.996350017387504;
JF415676;Lepidoptera;Aricia agestis;NA;0.253;Aricia agestis;0.996350017387504;
JF415677;Lepidoptera;Aricia artaxerxes;Plebejus argus;1;Aricia artaxerxes;0.999743187199922;
JF415678;Lepidoptera;Aricia eumedon;Eumedonia eumedon;0.017;Aricia eumedon;0.990022718798503;
JF415680;Lepidoptera;Boloria dia;Boloria dia;1;Boloria dia;0.99999999997425;
JF415683;Lepidoptera;Boloria euphrosyne;Boloria euphrosyne;1;Boloria euphrosyne;0.999998552914401;
JF415684;Lepidoptera;Boloria euphrosyne;Boloria euphrosyne;1;Boloria euphrosyne;0.999997047227918;
JF415689;Lepidoptera;Boloria titania;Boloria titania;0.001;Boloria titania;0.99983358374111;
JF415690;Lepidoptera;Brenthis ino;Brenthis ino;0.007;Brenthis ino;0.977157381961834;
JF415691;Lepidoptera;Celastrina argiolus;Celastrina argiolus;1;Celastrina argiolus;0.999708846309046;
JF415694;Lepidoptera;Colias alfacariensis;Colias alfacariensis;0.004;Colias alfacariensis;0.99974939016745;
JF415696;Lepidoptera;Erebia euryale;NA;NA;Erebia euryale;0.907156911257842;
JF415697;Lepidoptera;Erebia euryale;NA;NA;Erebia euryale;0.919518760623792;
JF415700;Lepidoptera;Erebia medusa;NA;0.009;Erebia medusa;0.986275612856827;
JF415701;Lepidoptera;Erebia medusa;NA;0.009;Erebia medusa;0.986275612856827;
JF415702;Lepidoptera;Erebia oeme;NA;0.114;Erebia oeme;0.999999999830635;
JF415703;Lepidoptera;Erebia oeme;NA;0.161;Erebia oeme;0.999999999457856;
JF415704;Lepidoptera;Erebia pronoe;Erebia pronoe;1;Erebia pronoe;0.999567958198288;
JF415705;Lepidoptera;Erebia pronoe;Erebia pronoe;1;Erebia pronoe;0.999567958198288;
JF415706;Lepidoptera;Euphydryas aurinia;NA;0.001;Euphydryas aurinia;0.995576749826865;
JF415709;Lepidoptera;Lasiommata maera;Lasiommata maera;1;Lasiommata maera;0.99999510792253;
JF415711;Lepidoptera;Lycaena tityrus;Lycaena tityrus;1;Lycaena tityrus;0.999998928238034;
JF415713;Lepidoptera;Melitaea cinxia;Melitaea cinxia;1;Melitaea cinxia;0.999999999999488;
JF415714;Lepidoptera;Melitaea cinxia;Melitaea cinxia;1;Melitaea cinxia;0.999999999999375;
JF415715;Lepidoptera;Melitaea diamina;NA;0.305;Melitaea diamina;0.999999994065661;
JF415716;Lepidoptera;Melitaea diamina;NA;0.305;Melitaea diamina;0.999999994065661;
JF415717;Lepidoptera;Melitaea didyma;Melitaea didyma;0.021;Melitaea didyma;0.9742722334097;
JF415719;Lepidoptera;Ochlodes sylvanus;NA;0.316;Ochlodes sylvanus;0.996318754838625;
JF415721;Lepidoptera;Parnassius apollo;Parnassius apollo;1;Parnassius apollo;0.992704703916692;
JF415724;Lepidoptera;Plebejus idas;NA;0.007;NA;0.999546324331549;
JF415725;Lepidoptera;Lysandra coridon;NA;NA;NA;0.992959117456688;
JF415726;Lepidoptera;Polyommatus damon;Polyommatus damon;1;Polyommatus damon;0.999999381161721;
JF415727;Lepidoptera;Polyommatus icarus;NA;NA;Polyommatus icarus;0.847406848705006;
JF415728;Lepidoptera;Pyrgus malvae;Pyrgus malvae;NA;Pyrgus malvae;0.980121140374433;
JF415729;Lepidoptera;Pyrgus malvae;Pyrgus malvae;NA;Pyrgus malvae;0.980121140374433;
JF415730;Lepidoptera;Pyrgus malvae;Pyrgus malvae;NA;Pyrgus malvae;0.980121140374433;
JF415731;Lepidoptera;Thymelicus sylvestris;NA;0.324;Thymelicus sylvestris;0.999940778442454;
JF810413;Lepidoptera;Callophrys rubi;NA;0.002;NA;0.999729984229093;

JF847976;Lepidoptera;Apatura ilia;NA;NA;Apatura ilia;0.935728859550908;
JF847980;Lepidoptera;Apatura iris;Apatura iris;1;Apatura iris;0.999999999999972;
JF847985;Lepidoptera;Erebia epiphron;NA;NA;Erebia epiphron;0.930026054867432;
JF847986;Lepidoptera;Hamearis lucina;NA;NA;Hamearis lucina;0.999999999984141;
JF847989;Lepidoptera;Pyrgus serratulae;Pyrgus serratulae;1;Pyrgus serratulae;0.99999989567508;
JF847990;Lepidoptera;Pyrgus serratulae;Pyrgus serratulae;1;Pyrgus serratulae;0.99999989567508;
JF847991;Lepidoptera;Pyrgus serratulae;Pyrgus serratulae;1;Pyrgus serratulae;0.999999908955122;
JF847992;Lepidoptera;Pyrgus serratulae;Pyrgus serratulae;1;Pyrgus serratulae;0.99999989567508;
JF847993;Lepidoptera;Pyrgus serratulae;Pyrgus serratulae;1;Pyrgus serratulae;0.99999989567508;
JF847994;Lepidoptera;Euphydryas aurinia;NA;0.432;Euphydryas aurinia;0.986888557954672;
JF847995;Lepidoptera;Helleia helle;Lycaena helle;1;Lycaena helle;0.999999994999371;
JF847996;Lepidoptera;Helleia helle;Lycaena helle;1;Lycaena helle;0.999999994999371;
JF847997;Lepidoptera;Helleia helle;Lycaena helle;1;Lycaena helle;0.999999994999371;
JF847998;Lepidoptera;Helleia helle;Lycaena helle;1;Lycaena helle;0.999999994999371;
JF847999;Lepidoptera;Helleia helle;Lycaena helle;1;Lycaena helle;0.999999996162757;
JF848000;Lepidoptera;Cupido osiris;Cupido osiris;1;Cupido osiris;0.999999944757265;
JF848001;Lepidoptera;Euphydryas aurinia;Euphydryas aurinia;0.012;Euphydryas aurinia;0.997780875235948;
JF848004;Lepidoptera;Aricia eumedon;Eumedonia eumedon;0.003;Eumedonia eumedon;0.998278637613531;
JF848005;Lepidoptera;Boloria selene;NA;NA;Boloria selene;0.999999920739274;
JF848006;Lepidoptera;Apatura iris;Apatura iris;1;Apatura iris;0.999999999999972;
JF848007;Lepidoptera;Pyrgus carthami;Pyrgus carthami;1;Pyrgus carthami;0.999998802660492;
JF848008;Lepidoptera;Cupido osiris;Cupido osiris;1;Cupido osiris;0.999997440696525;
JF850424;Lepidoptera;Parnassius apollo;Parnassius apollo;1;Parnassius apollo;0.992704703916692;
JF850425;Lepidoptera;Parnassius mnemosyne;Parnassius mnemosyne;1;Parnassius mnemosyne;0.999999326633058;
JF850427;Lepidoptera;Lycaena tityrus;Lycaena tityrus;1;Lycaena tityrus;0.999997027007808;
JF850428;Lepidoptera;Cupido minimus;NA;0.006;Cupido minimus;0.999768371759258;
JF850429;Lepidoptera;Cupido argiades;NA;0.499;NA;0.999999999984682;
JF850430;Lepidoptera;Cupido argiades;NA;0.189;Cupido argiades;0.713277686893393;
JF850431;Lepidoptera;Lycaena tityrus;Lycaena tityrus;1;Lycaena tityrus;0.999995729087172;
JF850432;Lepidoptera;Cupido argiades;NA;0.499;NA;0.999999999984682;
JF853516;Lepidoptera;Fabriciana niobe;NA;0.001;Fabriciana niobe;0.948797566470808;
JF853517;Lepidoptera;Fabriciana niobe;NA;0.001;Fabriciana niobe;0.948797566470808;
JF853545;Lepidoptera;Lycaena dispar;Lycaena dispar;1;Lycaena dispar;0.999999990743959;
JF853606;Lepidoptera;Carterocephalus palaemon;NA;0.052;Carterocephalus palaemon;0.728445051795325;
JF853607;Lepidoptera;Carterocephalus palaemon;Carterocephalus palaemon;0.016;Carterocephalus palaemon;0.930544560040029;
JF853612;Lepidoptera;Ochlodes sylvanus;NA;0.316;Ochlodes sylvanus;0.996318754838625;
JF853616;Lepidoptera;Anthocharis cardamines;Anthocharis cardamines;1;Anthocharis cardamines;0.999999843941806;
JF853617;Lepidoptera;Satyrium pruni;NA;0.076;Satyrium pruni;0.990322364021655;
JF853618;Lepidoptera;Callophrys rubi;NA;0.003;Callophrys rubi;0.997328639918919;
JF853619;Lepidoptera;Lycaena phlaeas;Lycaena phlaeas;1;Lycaena phlaeas;0.999999996363186;
JF853620;Lepidoptera;Lycaena virgaureae;NA;0.067;Lycaena virgaureae;0.999579233710412;
JF853622;Lepidoptera;Lycaena hippothoe;NA;NA;Lycaena hippothoe;0.929268253497542;
JF853623;Lepidoptera;Gonepteryx rhamni;NA;0.183;Gonepteryx rhamni;0.985836621117961;
JF853624;Lepidoptera;Pieris napi;NA;NA;NA;100.000.000.000.001;
JF853628;Lepidoptera;Aporia crataegi;NA;NA;Aporia crataegi;0.999997266436607;
JF853629;Lepidoptera;Aporia crataegi;NA;NA;Aporia crataegi;0.999997266436607;
JF853631;Lepidoptera;Colias hyale;Colias hyale;0.015;Colias hyale;0.999997884296128;
JF853632;Lepidoptera;Colias palaeno;NA;0.002;NA;0.999999999983681;
JF853633;Lepidoptera;Celastrina argiolus;Celastrina argiolus;1;Celastrina argiolus;0.999708846309046;
JF853634;Lepidoptera;Celastrina argiolus;Celastrina argiolus;1;Celastrina argiolus;0.999708846309046;
JF853635;Lepidoptera;Glaucopsyche alexis;NA;NA;Glaucopsyche alexis;0.979381169624069;
JF853636;Lepidoptera;Aricia artaxerxes;Plebejus argus;1;Aricia artaxerxes;0.999743187199922;
JF853637;Lepidoptera;Aricia eumedon;Eumedonia eumedon;0.001;Eumedonia eumedon;0.997942473254099;
JF853638;Lepidoptera;Plebejus argus;NA;0.328;Plebejus argus;0.969789734199889;
JF853639;Lepidoptera;Agriades optilete;Agriades optilete;1;Agriades optilete;0.999999991149878;
JF853641;Lepidoptera;Polyommatus icarus;NA;0.001;Polyommatus icarus;0.971665478142064;
JF853641;Lepidoptera;Polyommatus icarus;NA;0.001;Polyommatus icarus;0.971665478142064;
JF853642;Lepidoptera;Polyommatus amandus;Polyommatus amandus;0.003;Polyommatus amandus;0.999983287111715;
JF853643;Lepidoptera;Thecla betulae;Thecla betulae;1;Thecla betulae;0.999999999961119;
JF853644;Lepidoptera;Vanessa cardui;Vanessa cardui;1;Vanessa cardui;0.999999459983318;
JF853645;Lepidoptera;Vanessa atalanta;Vanessa atalanta;1;Vanessa atalanta;0.99999999957015;
JF853648;Lepidoptera;Aglais urticae;Aglais urticae;0.001;Aglais urticae;0.990690600684087;
JF853652;Lepidoptera;Apatura ilia;Apatura ilia;NA;Apatura ilia;0.988792300608896;
JF853653;Lepidoptera;Nymphalis c-album;Polygonia c-album;0.015;NA;0.99999926882126;
JF853654;Lepidoptera;Argynnis aglaja;NA;NA;NA;0.999999999998051;
JF853655;Lepidoptera;Fabriciana adippe;Fabriciana adippe;0.028;Fabriciana adippe;0.969769198942097;
JF853656;Lepidoptera;Fabriciana adippe;Fabriciana adippe;0.001;Fabriciana adippe;0.985680812514845;
JF853657;Lepidoptera;Fabriciana niobe;NA;0.001;Fabriciana niobe;0.948797566470808;

JF853658;Lepidoptera;Issoria lathonia;Issoria lathonia;1;Issoria lathonia;0.999999999989257;
JF853659;Lepidoptera;Brenthis ino;Brenthis ino;0.001;Brenthis ino;0.994711912827522;
JF853661;Lepidoptera;Boloria selene;NA;0.281;Boloria selene;0.999999606392712;
JF853663;Lepidoptera;Boloria euphrosyne;Boloria euphrosyne;1;Boloria euphrosyne;0.999998552914401;
JF853665;Lepidoptera;Euphydryas maturna;NA;NA;NA;0.999999999996973;
JF853666;Lepidoptera;Hipparchia semele;NA;0.002;Hipparchia semele;0.807780383202924;
JF853667;Lepidoptera;Hipparchia semele;NA;0.002;Hipparchia semele;0.784603069619426;
JF853668;Lepidoptera;Erebia medusa;NA;0.009;Erebia medusa;0.986275612856827;
JF853673;Lepidoptera;Coenonympha pamphilus;Coenonympha pamphilus;1;Coenonympha pamphilus;0.999999999999744;
JF853674;Lepidoptera;Aphantopus hyperantus;Aphantopus hyperantus;0.004;Aphantopus hyperantus;0.999598909345147;
JF853675;Lepidoptera;Pararge aegeria;Pararge aegeria;1;Pararge aegeria;0.999999999963165;
JF853676;Lepidoptera;Pararge aegeria;Pararge aegeria;1;Pararge aegeria;0.999999999963165;
JF853677;Lepidoptera;Lasiommata maera;Lasiommata maera;1;Lasiommata maera;0.999993981691807;
JF853678;Lepidoptera;Lasiommata maera;Lasiommata maera;1;Lasiommata maera;0.999999181072746;
JF853798;Lepidoptera;Thymelicus lineola;Thymelicus lineola;1;Thymelicus lineola;0.999932511874613;
JF853803;Lepidoptera;Apatura iris;Apatura iris;1;Apatura iris;0.999999999999972;
JF853804;Lepidoptera;Apatura ilia;Apatura ilia;NA;Apatura ilia;0.988792300608896;
JF853830;Lepidoptera;Satyrium pruni;NA;0.076;Satyrium pruni;0.990322364021655;
JF853831;Lepidoptera;Glaucopsyche alexis;NA;NA;Glaucopsyche alexis;0.979381169624069;
JF853832;Lepidoptera;Euphydryas maturna;NA;NA;NA;0.999999999996973;
JF854415;Lepidoptera;Lycaena dispar;Lycaena dispar;1;Lycaena dispar;0.999999990743959;
JF854416;Lepidoptera;Lycaena dispar;Lycaena dispar;1;Lycaena dispar;0.999999990743959;
JF854417;Lepidoptera;Scolitantides orion;Scolitantides orion;NA;Scolitantides orion;0.999999987418505;
JF854419;Lepidoptera;Erebia euryale;NA;NA;Erebia euryale;0.911037472312872;
JF854420;Lepidoptera;Erebia euryale;NA;NA;Erebia euryale;0.919518760623792;
JF854491;Lepidoptera;Lasiommata megera;Lasiommata megera;0.006;Lasiommata megera;0.992588117195958;
JF854492;Lepidoptera;Lasiommata megera;Lasiommata megera;1;Lasiommata megera;0.999928528239676;
JF854493;Lepidoptera;Colias croceus;NA;0.059;NA;0.999999999999738;
JF854496;Lepidoptera;Iphiclides podalirius;NA;0.313;Iphiclides podalirius;0.889198863100098;
JF854581;Lepidoptera;Euphydryas maturna;NA;0.126;Euphydryas maturna;0.885705216462044;
JF859941;Lepidoptera;Pararge aegeria;Pararge aegeria;1;Pararge aegeria;0.999999996428699;
JF859942;Lepidoptera;Erebia medusa;NA;0.01;Erebia medusa;0.979621232162315;
JF859943;Lepidoptera;Coenonympha pamphilus;Coenonympha pamphilus;1;Coenonympha pamphilus;0.999999999999744;
JF859976;Lepidoptera;Carterocephalus palaemon;NA;0.052;Carterocephalus palaemon;0.728445051795325;
JF860019;Lepidoptera;Argynnis aglaja;NA;NA;Speyeria aglaja;0.872991147917853;
JF860243;Lepidoptera;Boloria titania;Boloria titania;0.001;Boloria titania;0.99983358374111;
JF860261;Lepidoptera;Pyrgus andromedae;Pyrgus andromedae;0.001;Pyrgus andromedae;0.998828669843251;
JF860268;Lepidoptera;Agriades orbitulus;NA;0.01;Agriades orbitulus;0.998991302227708;
JN084662;Lepidoptera;Polyommatus celina;Polyommatus celina;0.001;Polyommatus celina;0.999999998406594;
JN084663;Lepidoptera;Polyommatus celina;Polyommatus celina;0.002;Polyommatus celina;0.999999998632774;
JN084664;Lepidoptera;Polyommatus celina;Polyommatus celina;0.001;Polyommatus celina;0.999999998295038;
JN084665;Lepidoptera;Polyommatus celina;Polyommatus celina;0.002;Polyommatus celina;0.999999998098872;
JN084668;Lepidoptera;Polyommatus celina;Polyommatus celina;0.011;Polyommatus celina;0.999999999815316;
JN084670;Lepidoptera;Polyommatus celina;Polyommatus celina;0.009;Polyommatus celina;0.999999999846835;
JN084672;Lepidoptera;Polyommatus celina;NA;0.066;Polyommatus celina;0.999999999820375;
JN084673;Lepidoptera;Polyommatus celina;NA;0.026;Polyommatus celina;0.999999999742386;
JN084675;Lepidoptera;Polyommatus celina;Polyommatus celina;0.003;Polyommatus celina;0.999999999495316;
JN084676;Lepidoptera;Polyommatus celina;NA;0.031;Polyommatus celina;0.999999999162497;
JN084678;Lepidoptera;Polyommatus celina;Polyommatus celina;0.009;Polyommatus celina;0.99999999744972;
JN084681;Lepidoptera;Polyommatus celina;Polyommatus celina;0.007;Polyommatus celina;0.999999999839019;
JN084682;Lepidoptera;Polyommatus eros;NA;0.002;Polyommatus eros;0.844998768384732;
JN084683;Lepidoptera;Polyommatus eros;NA;0.002;Polyommatus eros;0.834423002542038;
JN084684;Lepidoptera;Polyommatus eros;NA;0.003;Polyommatus eros;0.920592712495623;
JN084685;Lepidoptera;Polyommatus eros;NA;0.001;Polyommatus eros;0.952926802692268;
JN084688;Lepidoptera;Polyommatus icarus;NA;0.032;NA;0.999986483378367;
JN084690;Lepidoptera;Polyommatus eros;NA;0.005;Polyommatus eros;0.962873614249808;
JN084691;Lepidoptera;Polyommatus icarus;Polyommatus icarus;1;Polyommatus icarus;0.929306371740776;
JN084692;Lepidoptera;Polyommatus icarus;Polyommatus icarus;1;Polyommatus icarus;0.973480145147418;

JN084693;Lepidoptera;Polyommatus icarus;NA;0.122;Polyommatus icarus;0.924129158400787;
JN084694;Lepidoptera;Polyommatus icarus;NA;0.004;Polyommatus icarus;0.897338824938527;
JN084695;Lepidoptera;Polyommatus icarus;NA;0.003;Polyommatus icarus;0.876367325848701;
JN084696;Lepidoptera;Polyommatus icarus;NA;0.001;Polyommatus icarus;0.988079488562884;
JN084697;Lepidoptera;Polyommatus icarus;NA;0.001;Polyommatus icarus;0.993273500584892;
JN084698;Lepidoptera;Polyommatus icarus;NA;0.001;Polyommatus icarus;0.994084407316265;
JN084699;Lepidoptera;Polyommatus icarus;NA;0.005;Polyommatus icarus;0.991751251654024;
JN084700;Lepidoptera;Polyommatus icarus;Polyommatus icarus;0.001;Polyommatus icarus;0.999326557615001;
JN084701;Lepidoptera;Polyommatus icarus;Polyommatus icarus;0.002;Polyommatus icarus;0.999346502768464;
JN084702;Lepidoptera;Polyommatus icarus;Polyommatus icarus;0.005;Polyommatus icarus;0.999275111324919;
JN084703;Lepidoptera;Polyommatus icarus;Polyommatus icarus;0.001;Polyommatus icarus;0.999526751169551;
JN084704;Lepidoptera;Polyommatus icarus;Polyommatus icarus;0.007;Polyommatus icarus;0.999488788177924;
JN084705;Lepidoptera;Polyommatus icarus;NA;0.003;Polyommatus icarus;0.996721942207079;
JN084706;Lepidoptera;Polyommatus icarus;Polyommatus icarus;0.001;Polyommatus icarus;0.99737489720609;
JN084707;Lepidoptera;Polyommatus icarus;NA;0.003;Polyommatus icarus;0.997760766612533;
JN084708;Lepidoptera;Polyommatus icarus;NA;0.001;Polyommatus icarus;0.947786269007611;
JN084709;Lepidoptera;Polyommatus icarus;Polyommatus icarus;1;Polyommatus icarus;0.942188845781506;
JN114414;Lepidoptera;Polyommatus icarus;NA;0.004;Polyommatus icarus;0.999481683747521;
JN114415;Lepidoptera;Polyommatus icarus;Polyommatus icarus;0.001;Polyommatus icarus;0.999851522482238;
JN114416;Lepidoptera;Polyommatus icarus;NA;0.004;Polyommatus icarus;0.999481683747521;
JN114417;Lepidoptera;Polyommatus icarus;NA;0.002;Polyommatus icarus;0.998502314961965;
JN262235;Lepidoptera;Apatura iris;Apatura iris;1;Apatura iris;0.999999999999972;
JN264880;Lepidoptera;Charaxes jasius;Charaxes jasius;NA;Charaxes jasius;0.990397821479112;
JN265784;Lepidoptera;Gonepteryx cleopatra;Gonepteryx cleopatra;0.998;Gonepteryx cleopatra;0.999118636582266;
JN265785;Lepidoptera;Gonepteryx cleopatra;Gonepteryx cleopatra;0.998;Gonepteryx cleopatra;0.999118636582266;
JN265786;Lepidoptera;Colias crocea;NA;0.059;NA;0.999999999999738;
JN272495;Lepidoptera;Fabriciana adippe;Fabriciana adippe;0.001;Fabriciana adippe;0.999538788643547;
JN272496;Lepidoptera;Fabriciana adippe;Fabriciana adippe;0.001;Fabriciana adippe;0.999095724865568;
JN272497;Lepidoptera;Boloria euphrosyne;Boloria euphrosyne;0.003;Boloria euphrosyne;0.999970094877776;
JN272499;Lepidoptera;Fabriciana adippe;NA;0.001;Fabriciana adippe;0.924751459079782;
JN272500;Lepidoptera;Brenthis hecate;NA;0.068;Brenthis hecate;0.999053551498733;
JN272502;Lepidoptera;Fabriciana adippe;Fabriciana adippe;1;Fabriciana adippe;0.999684800144869;
JN272503;Lepidoptera;Fabriciana adippe;Fabriciana adippe;0.001;Fabriciana adippe;0.999538788643547;
JN272504;Lepidoptera;Fabriciana adippe;Fabriciana adippe;1;Fabriciana adippe;0.999722411724441;
JN272505;Lepidoptera;Boloria thore;Boloria thore;1;Boloria thore;0.999999999251798;
JN272506;Lepidoptera;Brenthis ino;Brenthis ino;1;Brenthis ino;0.995903086085865;
JN272507;Lepidoptera;Boloria thore;Boloria thore;1;Boloria thore;0.999999998359726;
JN272539;Lepidoptera;Boloria euphrosyne;Boloria euphrosyne;1;Boloria euphrosyne;0.999998552914401;
JN273363;Lepidoptera;Thymelicus sylvestris;NA;0.324;Thymelicus sylvestris;0.999940778442454;
JN273364;Lepidoptera;Thymelicus acteon;Thymelicus acteon;1;Thymelicus acteon;0.99999999752248;
JN273482;Lepidoptera;Thymelicus lineola;Thymelicus lineola;1;Thymelicus lineola;0.999971535093703;
JN273485;Lepidoptera;Thymelicus lineola;Thymelicus lineola;1;Thymelicus lineola;0.999971535093703;
JN274546;Lepidoptera;Melitaea cinxia;Melitaea cinxia;1;Melitaea cinxia;0.999999999999602;
JN274547;Lepidoptera;Aglais urticae;Aglais urticae;0.999;Aglais urticae;0.996396169943103;
JN274549;Lepidoptera;Euphydryas aurinia;Euphydryas aurinia;0.005;Euphydryas aurinia;0.998570950148478;
JN274551;Lepidoptera;Melitaea britomartis;Mellicta britomartis;NA;Melitaea britomartis;0.933869879268478;
JN274552;Lepidoptera;Melitaea cinxia;Melitaea cinxia;1;Melitaea cinxia;0.999999999999488;
JN274553;Lepidoptera;Melitaea cinxia;Melitaea cinxia;1;Melitaea cinxia;0.999999999999488;
JN274554;Lepidoptera;Euphydryas aurinia;NA;0.001;Euphydryas aurinia;0.995576749826865;
JN274555;Lepidoptera;Euphydryas aurinia;NA;0.001;Euphydryas aurinia;0.995576749826865;
JN274637;Lepidoptera;Melitaea diamina;NA;0.149;Melitaea diamina;0.999999996592464;
JN274638;Lepidoptera;Melitaea diamina;NA;0.149;Melitaea diamina;0.999999996592464;
JN275717;Lepidoptera;Parnassius mnemosyne;Parnassius mnemosyne;1;Parnassius mnemosyne;0.999999556844826;
JN276236;Lepidoptera;Anthocharis euphenoides;NA;0.172;Anthocharis euphenoides;0.943741664916385;
JN276237;Lepidoptera;Anthocharis cardamines;Anthocharis cardamines;1;Anthocharis cardamines;0.999999940675226;
JN276244;Lepidoptera;Anthocharis euphenoides;NA;0.172;Anthocharis euphenoides;0.943741664916385;
JN276246;Lepidoptera;Pieris napi;NA;NA;Pieris napi;0.880626338564953;

JN276880;Lepidoptera;Polyommatus escheri;Polyommatus escheri;1;Polyommatus escheri;0.999998281574049;
JN276884;Lepidoptera;Plebejus argus;Plebejus argus;1;Plebejus argus;0.997339600942735;
JN276886;Lepidoptera;Polyommatus escheri;Polyommatus escheri;1;Polyommatus escheri;0.999998281574049;
JN276887;Lepidoptera;Glaucopsyche melanops;Glaucopsyche melanops;1;Glaucopsyche melanops;0.999952924254607;
JN276888;Lepidoptera;Glaucopsyche melanops;Glaucopsyche melanops;1;Glaucopsyche melanops;0.999976648921708;
JN276889;Lepidoptera;Plebejus argus;NA;NA;Plebejus argus;0.969283117748927;
JN276890;Lepidoptera;Glaucopsyche melanops;Glaucopsyche melanops;1;Glaucopsyche melanops;0.999952924254607;
JN276891;Lepidoptera;Plebejus argus;Plebejus argus;0.014;Plebejus argus;0.979849220658559;
JN276892;Lepidoptera;Cupido osiris;Cupido osiris;1;Cupido osiris;0.99999988431427;
JN276893;Lepidoptera;Cupido osiris;Cupido osiris;1;Cupido osiris;0.999974988645513;
JN276895;Lepidoptera;Plebejus idas;NA;0.001;NA;0.999590911470354;
JN276897;Lepidoptera;Plebejus idas;Plebejus idas;0.001;NA;0.999655983096466;
JN276901;Lepidoptera;Plebejus argus;Plebejus argus;0.014;Plebejus argus;0.979849220658559;
JN276902;Lepidoptera;Cupido argiades;NA;0.499;NA;0.999999999984682;
JN276903;Lepidoptera;Aricia eumedon;NA;0.069;Eumedonia eumedon;0.937041919865327;
JN276904;Lepidoptera;Plebejus idas;NA;0.001;NA;0.998603327696059;
JN276907;Lepidoptera;Aricia eumedon;Eumedonia eumedon;1;Eumedonia eumedon;0.924507020284958;
JN276908;Lepidoptera;Cupido argiades;NA;0.499;NA;0.999999999984682;
JN276909;Lepidoptera;Polyommatus amandus;Polyommatus amandus;1;Polyommatus amandus;0.999993368166194;
JN276910;Lepidoptera;Lysandra coridon;NA;NA;NA;0.998070184737648;
JN276911;Lepidoptera;Plebejus idas;NA;0.007;NA;0.999546324331549;
JN276912;Lepidoptera;Aricia artaxerxes;Plebejus argus;1;Aricia artaxerxes;0.999743187199922;
JN276913;Lepidoptera;Polyommatus daphnis;Polyommatus daphnis;0.008;Polyommatus daphnis;0.99732138840566;
JN276914;Lepidoptera;Lysandra coridon;NA;NA;NA;0.998070184737648;
JN276915;Lepidoptera;Aricia agestis;NA;0.253;Aricia agestis;0.996350017387504;
JN276916;Lepidoptera;Polyommatus damon;Polyommatus damon;1;Polyommatus damon;0.999999955377263;
JN276922;Lepidoptera;Cupido minimus;NA;0.003;Cupido minimus;0.999871667634294;
JN276923;Lepidoptera;Aricia artaxerxes;NA;0.001;Aricia artaxerxes;0.999793077258486;
JN277556;Lepidoptera;Carcharodus baeticus;Carcharodus baeticus;1;Carcharodus baeticus;0.999983899664537;
JN277557;Lepidoptera;Pyrgus malvoides;NA;0.076;Pyrgus malvoides;0.887854315566122;
JN277558;Lepidoptera;Pyrgus malvoides;NA;0.03;Pyrgus malvoides;0.934050489634307;
JN277562;Lepidoptera;Pyrgus cacaliae;Pyrgus cacaliae;0.001;Pyrgus cacaliae;0.997779968725098;
JN277563;Lepidoptera;Pyrgus alveus;NA;0.003;NA;0.999999999997732;
JN277564;Lepidoptera;Pyrgus alveus;NA;NA;Pyrgus alveus;0.827119616974978;
JN277565;Lepidoptera;Pyrgus alveus;NA;NA;Pyrgus alveus;0.827119616974978;
JN277570;Lepidoptera;Pyrgus armoricanus;Pyrgus armoricanus;1;Pyrgus armoricanus;0.999997353848817;
JN277571;Lepidoptera;Pyrgus warrenensis;NA;0.003;NA;0.999999999997732;
JN277572;Lepidoptera;Pyrgus alveus;NA;NA;Pyrgus alveus;0.941697735568124;
JN277573;Lepidoptera;Pyrgus serratulae;Pyrgus serratulae;1;Pyrgus serratulae;0.99999989567508;
JN277575;Lepidoptera;Pyrgus alveus;NA;NA;Pyrgus alveus;0.827119616974978;
JN277577;Lepidoptera;Pyrgus serratulae;Pyrgus serratulae;1;Pyrgus serratulae;0.99999989567508;
JN277580;Lepidoptera;Erynnis tages;Erynnis tages;1;Erynnis tages;0.999999281987018;
JN277845;Lepidoptera;Pyrgus andromedae;Pyrgus andromedae;1;Pyrgus andromedae;0.99960427425013;
JN277846;Lepidoptera;Pyrgus andromedae;Pyrgus andromedae;1;Pyrgus andromedae;0.999845626259229;
JN277847;Lepidoptera;Pyrgus alveus;NA;NA;Pyrgus alveus;0.922887984165083;
JN278861;Lepidoptera;Erebia meolans;Erebia meolans;NA;Erebia meolans;0.999835953426128;
JN278862;Lepidoptera;Cercyonis lupina;Hyponephele lupina;1;Hyponephele lupina;0.999999953670284;
JN278872;Lepidoptera;Coenonympha dorus;Coenonympha dorus;1;Coenonympha dorus;0.999999999703419;
JN278874;Lepidoptera;Hipparchia fagi;Hipparchia fagi;NA;Hipparchia fagi;0.973330290902851;
JN278875;Lepidoptera;Cercyonis lupina;Hyponephele lupina;1;Hyponephele lupina;0.99999998018663;
JN278877;Lepidoptera;Cercyonis lupina;Hyponephele lupina;1;Hyponephele lupina;0.99999998018663;
JN278879;Lepidoptera;Hipparchia hermione;Hipparchia hermione;1;Hipparchia hermione;0.99988238204256;
JN278880;Lepidoptera;Hipparchia semele;NA;0.008;Hipparchia semele;0.74726780401631;
JN278882;Lepidoptera;Satyrus ferula;Satyrus ferula;0.001;Satyrus ferula;0.994329228962786;
JN278883;Lepidoptera;Hipparchia semele;NA;0.001;Hipparchia semele;0.862655722502182;
JN278884;Lepidoptera;Satyrus ferula;Satyrus ferula;0.001;Satyrus ferula;0.997389091702265;
JN278891;Lepidoptera;Hipparchia statilinus;Hipparchia statilinus;1;Hipparchia statilinus;0.99999999999946;
JN278892;Lepidoptera;Arethusana arethusa;Arethusana arethusa;1;Arethusana arethusa;0.999999733827174;
JN278893;Lepidoptera;Arethusana arethusa;Arethusana arethusa;1;Arethusana arethusa;0.999999733827174;
JN278894;Lepidoptera;Coenonympha dorus;Coenonympha dorus;1;Coenonympha dorus;0.999999999915957;
JN278895;Lepidoptera;Coenonympha dorus;Coenonympha dorus;1;Coenonympha dorus;0.999999999861814;
JN278896;Lepidoptera;Lasiommata megera;Lasiommata megera;1;Lasiommata megera;0.999770424843767;
JN278897;Lepidoptera;Minois dryas;NA;0.025;Minois dryas;0.850746026247397;
JN278898;Lepidoptera;Minois dryas;NA;0.025;Minois dryas;0.850746026247397;
JN278899;Lepidoptera;Lasiommata maera;Lasiommata maera;1;Lasiommata maera;0.99999942908402;

JN278901;Lepidoptera;Erebia pharte;Erebia pharte;1;Erebia pharte;0.99999999630495;
JN278902;Lepidoptera;Erebia manto;NA;0.192;Erebia manto;0.997710988231194;
JN278903;Lepidoptera;Erebia oeme;NA;0.114;Erebia oeme;0.999999999830635;
JN278904;Lepidoptera;Erebia euryale;NA;NA;Erebia euryale;0.891793339631426;
JN278905;Lepidoptera;Erebia pronoe;Erebia pronoe;1;Erebia pronoe;0.999567958198288;
JN278906;Lepidoptera;Erebia styx;NA;NA;Erebia styx;0.964967594538516;
JN278907;Lepidoptera;Erebia pronoe;Erebia pronoe;1;Erebia pronoe;0.999567958198288;
JN278908;Lepidoptera;Erebia pronoe;Erebia pronoe;1;Erebia pronoe;0.999567958198288;
JN278909;Lepidoptera;Minois dryas;NA;0.025;Minois dryas;0.850746026247397;
JN278910;Lepidoptera;Brintesia circe;Brintesia circe;1;Brintesia circe;0.99999951254154;
JN278927;Lepidoptera;Erebia euryale;NA;NA;Erebia euryale;0.896073040775925;
JN280062;Lepidoptera;Limenitis camilla;NA;0.049;Limenitis camilla;0.999921324781941;
JN280740;Lepidoptera;Favonius quercus;NA;NA;Favonius quercus;0.73046069582187;
JN280794;Lepidoptera;Lycaena alciphron;Lycaena alciphron;1;Lycaena alciphron;0.999999802956713;
JN280795;Lepidoptera;Lycaena dispar;Lycaena dispar;1;Lycaena dispar;0.999999990743959;
JN280797;Lepidoptera;Lycaena dispar;Lycaena dispar;1;Lycaena dispar;0.999999990743959;
JN283987;Lepidoptera;Hamearis lucina;NA;NA;Hamearis lucina;0.999999999984141;
JN283988;Lepidoptera;Hamearis lucina;NA;NA;Hamearis lucina;0.999999999984141;
JN286153;Lepidoptera;Satyrium w-album;NA;0.056;Satyrium w-album;0.989378230996316;
JN581045;Lepidoptera;Plebejus argus;NA;0.001;Plebejus artaxerxes;0.783192910822683;
JN581046;Lepidoptera;Plebejus argus;NA;0.329;Plebejus artaxerxes;0.861065871161998;
JN581047;Lepidoptera;Plebejus argus;NA;0.119;Plebejus artaxerxes;0.777287514901365;
JN581048;Lepidoptera;Plebejus argus;NA;0.329;Plebejus artaxerxes;0.87533599385847;
JN581049;Lepidoptera;Plebejus argus;NA;0.002;Plebejus artaxerxes;0.867913751762474;
JN581050;Lepidoptera;Plebejus argus;NA;0.329;Plebejus artaxerxes;0.863590204914929;
JN581051;Lepidoptera;Plebejus argus;NA;0.001;Plebejus artaxerxes;0.771297277867841;
JN581052;Lepidoptera;Plebejus argus;NA;0.329;Plebejus artaxerxes;0.898756221079625;
JN581053;Lepidoptera;Plebejus argus;NA;0.329;Plebejus artaxerxes;0.902874876101085;
JN581054;Lepidoptera;Plebejus argus;NA;0.329;Plebejus artaxerxes;0.887561036013298;
JN581055;Lepidoptera;Plebejus argus;NA;0.001;Plebejus artaxerxes;0.782539669648728;
JN581056;Lepidoptera;Plebejus argus;NA;0.329;Plebejus artaxerxes;0.878270880288244;
JN581057;Lepidoptera;Plebejus argus;NA;0.001;Plebejus artaxerxes;0.7849926493968;
JN581058;Lepidoptera;Plebejus argus;NA;0.001;Plebejus artaxerxes;0.791969059784226;
JN581059;Lepidoptera;Plebejus argus;NA;0.001;Plebejus artaxerxes;0.769066857349353;
JN581060;Lepidoptera;Plebejus argus;NA;0.123;Plebejus artaxerxes;0.860415772996577;
JN820118;Lepidoptera;Polyommatus icarus;NA;0.002;Polyommatus icarus;0.977063739380834;
JN827862;Lepidoptera;Aporia crataegi;NA;NA;Aporia crataegi;0.999997694051005;
JN827863;Lepidoptera;Aporia crataegi;NA;NA;Aporia crataegi;0.999996781126733;
JN827864;Lepidoptera;Aporia crataegi;NA;NA;Aporia crataegi;0.999996781126733;
JN827865;Lepidoptera;Aporia crataegi;NA;NA;Aporia crataegi;0.999995283373874;
JN827866;Lepidoptera;Aricia eumedon;Eumedonia eumedon;1;Eumedonia eumedon;0.998912717557217;
JN827867;Lepidoptera;Aricia eumedon;Eumedonia eumedon;1;Eumedonia eumedon;0.997792722086228;
JN827868;Lepidoptera;Aricia eumedon;Eumedonia eumedon;1;Eumedonia eumedon;0.998912717557217;
JN827869;Lepidoptera;Aricia eumedon;Eumedonia eumedon;0.001;Eumedonia eumedon;0.997779785094791;
JN827871;Lepidoptera;Boloria pales;Boloria pales;1;Boloria pales;0.999024569652783;
JN827872;Lepidoptera;Boloria pales;Boloria pales;1;Boloria pales;0.998507647164222;
JN827873;Lepidoptera;Celastrina argiolus;Celastrina argiolus;0.999;Celastrina argiolus;0.997763693175195;
JN827874;Lepidoptera;Celastrina argiolus;Celastrina argiolus;0.999;Celastrina argiolus;0.999500479526489;
JN827875;Lepidoptera;Celastrina argiolus;Celastrina argiolus;0.997;Celastrina argiolus;0.999539235304467;
JN827876;Lepidoptera;Celastrina argiolus;Celastrina argiolus;0.999;Celastrina argiolus;0.999500479526489;
JN827877;Lepidoptera;Celastrina argiolus;Celastrina argiolus;1;Celastrina argiolus;0.999485186737738;
JN827878;Lepidoptera;Coenonympha pamphilus;Coenonympha pamphilus;1;Coenonympha pamphilus;0.999999999999744;
JN827879;Lepidoptera;Colias crocea;NA;0.059;NA;0.999999999999738;
JN827880;Lepidoptera;Erebia oeme;NA;0.114;Erebia oeme;0.999999999830635;
JN827881;Lepidoptera;Erebia oeme;NA;0.114;Erebia oeme;0.999999999830635;
JN827882;Lepidoptera;Erebia oeme;NA;0.114;Erebia oeme;0.999999999830635;
JN827883;Lepidoptera;Erebia oeme;NA;0.114;Erebia oeme;0.999999999830635;
JN827884;Lepidoptera;Erebia oeme;NA;0.114;Erebia oeme;0.999999999830635;
JN827889;Lepidoptera;Vanessa atalanta;Vanessa atalanta;1;Vanessa atalanta;0.999999999757762;
JN827890;Lepidoptera;Vanessa atalanta;Vanessa atalanta;1;Vanessa atalanta;0.999999999757762;
JN827891;Lepidoptera;Vanessa atalanta;Vanessa atalanta;1;Vanessa atalanta;0.999999999383476;
JN827892;Lepidoptera;Vanessa atalanta;Vanessa atalanta;1;Vanessa atalanta;0.999999999757762;
JN827893;Lepidoptera;Vanessa atalanta;Vanessa atalanta;1;Vanessa atalanta;0.999999999017149;
JX013970;Lepidoptera;Erebia medusa;NA;0.017;NA;0.99999999819795;
JX013971;Lepidoptera;Erebia medusa;NA;0.017;NA;0.99999999819795;
JX013972;Lepidoptera;Erebia medusa;NA;0.017;NA;0.99999999819795;
JX013973;Lepidoptera;Erebia medusa;NA;0.017;NA;0.99999999819795;
JX013974;Lepidoptera;Erebia medusa;NA;0.004;NA;0.999999998506986;
JX034611;Lepidoptera;Erebia medusa;NA;0.009;Erebia medusa;0.986275612856827;
JX034616;Lepidoptera;Erebia pandrose;Erebia pandrose;0.017;Erebia pandrose;0.979533383298681;
JX034634;Lepidoptera;Erebia pandrose;Erebia pandrose;0.999;Erebia pandrose;0.99815402817964;
JX034650;Lepidoptera;Erebia pandrose;Erebia pandrose;0.999;Erebia pandrose;0.99815402817964;

JX034685;Lepidoptera;Boloria thore;Boloria thore;1;Boloria thore;0.99999994695486;
JX093461;Lepidoptera;Polyommatus daphnis;NA;0.058;NA;0.962780253148182;
JX093465;Lepidoptera;Polyommatus escheri;Polyommatus escheri;1;NA;0.849301216595283;
JX112880;Lepidoptera;Satyrium pruni;NA;0.347;Satyrium pruni;0.991870503237268;
JX112881;Lepidoptera;Satyrium spini;Satyrium spini;1;Satyrium spini;0.999992332933321;
JX112882;Lepidoptera;Satyrium w-album;NA;0.142;Satyrium walbum;0.704884797922891;
JX112886;Lepidoptera;Satyrium acaciae;Satyrium acaciae;1;Satyrium acaciae;0.999999726506227;
JX112887;Lepidoptera;Satyrium ilicis;Satyrium ilicis;1;Satyrium ilicis;0.999999980777432;
JX155755;Lepidoptera;Erebia pronoe;NA;0.002;NA;0.999991228117973;
JX155756;Lepidoptera;Erebia pronoe;Erebia pronoe;0.001;Erebia pronoe;0.837490350528977;
JX311250;Lepidoptera;Phengaris teleius;NA;0.001;NA;0.830140871479552;
JX311251;Lepidoptera;Phengaris teleius;Phengaris teleius;1;NA;0.767566236602233;
JX311254;Lepidoptera;Phengaris teleius;NA;0.192;NA;0.822313378426264;
JX311255;Lepidoptera;Phengaris teleius;NA;0.488;NA;0.814102909812003;
JX311260;Lepidoptera;Phengaris teleius;NA;0.495;NA;0.815636694559594;
JX311261;Lepidoptera;Phengaris teleius;NA;0.488;NA;0.823545101572831;
JX311262;Lepidoptera;Phengaris teleius;NA;0.488;NA;0.922548956175661;
JX311263;Lepidoptera;Phengaris teleius;NA;0.493;NA;0.823421349863848;
JX311264;Lepidoptera;Phengaris teleius;NA;0.488;NA;0.922548956175661;
JX311265;Lepidoptera;Phengaris teleius;NA;0.488;NA;0.900697937585587;
JX311266;Lepidoptera;Phengaris teleius;NA;0.488;NA;0.900697937585587;
JX678023;Lepidoptera;Aricia cramera;Aricia cramera;1;Aricia cramera;0.999994694491994;
JX678024;Lepidoptera;Aricia cramera;Aricia cramera;1;Aricia cramera;0.999994694491994;
JX678025;Lepidoptera;Aricia cramera;Aricia cramera;1;Aricia cramera;0.999994694491994;
JX678026;Lepidoptera;Aricia cramera;Aricia cramera;1;Aricia cramera;0.999994694491994;
JX678027;Lepidoptera;Aricia cramera;Aricia cramera;1;Aricia cramera;0.999993970273909;
JX678028;Lepidoptera;Aricia cramera;Aricia cramera;1;Aricia cramera;0.999993970273909;
JX678030;Lepidoptera;Aricia cramera;Aricia cramera;1;Aricia cramera;0.999993970273909;
JX678031;Lepidoptera;Aricia cramera;Aricia cramera;1;Aricia cramera;0.999993970273909;
JX678032;Lepidoptera;Aricia cramera;Aricia cramera;1;Aricia cramera;0.999993970273909;
JX678033;Lepidoptera;Aricia cramera;Aricia cramera;1;Aricia cramera;0.999996928694627;
JX678034;Lepidoptera;Aricia cramera;Aricia cramera;1;Aricia cramera;0.999996928694627;
JX678035;Lepidoptera;Aricia cramera;Aricia cramera;1;Aricia cramera;0.999993970273909;
JX678036;Lepidoptera;Aricia cramera;Aricia cramera;1;Aricia cramera;0.999993970273909;
JX678039;Lepidoptera;Aricia cramera;Aricia cramera;1;Aricia cramera;0.99999593445832;
JX678040;Lepidoptera;Aricia cramera;Aricia cramera;1;Aricia cramera;0.99999644604876;
JX678041;Lepidoptera;Aricia cramera;Aricia cramera;1;Aricia cramera;0.999995501900228;
JX678042;Lepidoptera;Aricia cramera;Aricia cramera;1;Aricia cramera;0.999993970273909;
JX678043;Lepidoptera;Aricia cramera;Aricia cramera;1;Aricia cramera;0.999991041622588;
JX678044;Lepidoptera;Aricia cramera;Aricia cramera;1;Aricia cramera;0.999994694491994;
JX678045;Lepidoptera;Aricia cramera;Aricia cramera;1;Aricia cramera;0.999994694491994;
JX678048;Lepidoptera;Aricia cramera;Aricia cramera;1;Aricia cramera;0.999994694491994;
JX678049;Lepidoptera;Aricia cramera;Aricia cramera;1;Aricia cramera;0.999993993168339;
JX678050;Lepidoptera;Aricia cramera;Aricia cramera;1;Aricia cramera;0.999993993168339;
JX678052;Lepidoptera;Aricia artaxerxes;NA;0.412;Aricia artaxerxes;0.999555436728994;
JX678053;Lepidoptera;Aricia artaxerxes;NA;0.412;Aricia artaxerxes;0.999555436728994;
JX678054;Lepidoptera;Aricia artaxerxes;NA;0.412;Aricia artaxerxes;0.999555436728994;
JX678055;Lepidoptera;Aricia artaxerxes;Plebejus argus;1;Aricia artaxerxes;0.99976599124702;
JX678056;Lepidoptera;Aricia artaxerxes;Plebejus argus;1;Aricia artaxerxes;0.99976599124702;
JX678058;Lepidoptera;Aricia artaxerxes;NA;0.143;Aricia artaxerxes;0.999805001123268;
JX678063;Lepidoptera;Aricia agestis;NA;0.034;Aricia agestis;0.999538578806676;
JX678064;Lepidoptera;Aricia agestis;NA;0.034;Aricia agestis;0.999538578806676;
JX678071;Lepidoptera;Aricia agestis;NA;0.064;Aricia agestis;0.995841086018724;
JX678072;Lepidoptera;Aricia agestis;NA;0.064;Aricia agestis;0.995841086018724;
JX678078;Lepidoptera;Aricia artaxerxes;Plebejus argus;1;Aricia artaxerxes;0.99976599124702;
JX678080;Lepidoptera;Aricia artaxerxes;Plebejus argus;1;Aricia artaxerxes;0.99976599124702;
JX678081;Lepidoptera;Aricia artaxerxes;Plebejus argus;1;Aricia artaxerxes;0.99976599124702;
JX678082;Lepidoptera;Aricia artaxerxes;Plebejus argus;1;Aricia artaxerxes;0.99976599124702;
JX678083;Lepidoptera;Aricia artaxerxes;Plebejus argus;1;Aricia artaxerxes;0.99976599124702;
JX678084;Lepidoptera;Aricia artaxerxes;Plebejus argus;1;Aricia artaxerxes;0.99976599124702;
JX678085;Lepidoptera;Aricia artaxerxes;Plebejus argus;1;Aricia artaxerxes;0.99976599124702;
JX678087;Lepidoptera;Aricia artaxerxes;NA;0.383;Aricia artaxerxes;0.999829424590954;
JX678088;Lepidoptera;Aricia artaxerxes;NA;0.383;Aricia artaxerxes;0.999829424590954;
JX678089;Lepidoptera;Aricia artaxerxes;NA;0.412;Aricia artaxerxes;0.999555436728994;
JX678090;Lepidoptera;Aricia artaxerxes;NA;0.412;Aricia artaxerxes;0.999555436728994;
JX678093;Lepidoptera;Polyommatus thersites;Polyommatus thersites;0.003;Polyommatus thersites;0.999999936691014;
JX678097;Lepidoptera;Aricia artaxerxes;NA;0.001;Aricia artaxerxes;0.998800871462615;
JX678098;Lepidoptera;Aricia artaxerxes;NA;0.001;Aricia artaxerxes;0.998800871462615;
JX678100;Lepidoptera;Aricia artaxerxes;NA;0.062;Aricia artaxerxes;0.999560771556993;
JX678105;Lepidoptera;Aricia artaxerxes;NA;0.057;Aricia artaxerxes;0.998733206230977;
JX678106;Lepidoptera;Aricia artaxerxes;NA;0.136;Aricia artaxerxes;0.999466097136126;
JX678115;Lepidoptera;Aricia agestis;NA;0.091;Aricia agestis;0.998135413168303;
JX678118;Lepidoptera;Aricia agestis;NA;0.306;Aricia agestis;0.993846455426407;
JX678119;Lepidoptera;Aricia agestis;NA;0.306;Aricia agestis;0.993846455426407;
JX678120;Lepidoptera;Aricia agestis;NA;0.306;Aricia agestis;0.993846455426407;
JX678121;Lepidoptera;Aricia agestis;NA;0.306;Aricia agestis;0.993846455426407;
JX678122;Lepidoptera;Aricia agestis;NA;0.306;Aricia agestis;0.993846455426407;

JX678123;Lepidoptera;Aricia agestis;NA;NA;Aricia agestis;0.996270377984369;
JX678124;Lepidoptera;Aricia agestis;NA;NA;Aricia agestis;0.996270377984369;
JX678125;Lepidoptera;Aricia agestis;NA;NA;Aricia agestis;0.996270377984369;
JX678127;Lepidoptera;Aricia agestis;NA;NA;Aricia agestis;0.996270377984369;
JX678128;Lepidoptera;Aricia agestis;NA;NA;Aricia agestis;0.996270377984369;
JX678129;Lepidoptera;Aricia agestis;NA;NA;Aricia agestis;0.996270377984369;
JX678130;Lepidoptera;Aricia agestis;NA;0.073;Aricia agestis;0.999504421887332;
JX678131;Lepidoptera;Aricia agestis;NA;0.073;Aricia agestis;0.999504421887332;
JX678132;Lepidoptera;Aricia agestis;NA;0.085;Aricia agestis;0.998833911707261;
JX678134;Lepidoptera;Aricia agestis;Aricia agestis;1;Aricia agestis;0.971536179996815;
JX678135;Lepidoptera;Aricia agestis;NA;0.1;Aricia agestis;0.990272297705663;
JX678137;Lepidoptera;Aricia agestis;Aricia agestis;1;Aricia agestis;0.990793357852564;
JX678138;Lepidoptera;Aricia agestis;Aricia agestis;1;Aricia agestis;0.978864665329184;
JX678140;Lepidoptera;Aricia agestis;NA;0.073;Aricia agestis;0.999504421887332;
JX678144;Lepidoptera;Aricia agestis;NA;0.037;Aricia agestis;0.997482346124557;
JX678145;Lepidoptera;Aricia agestis;NA;0.039;Aricia agestis;0.996032662733461;
JX678146;Lepidoptera;Aricia agestis;NA;0.169;Aricia agestis;0.996445434287717;
JX678148;Lepidoptera;Aricia agestis;NA;0.075;Aricia agestis;0.998336166156409;
JX678149;Lepidoptera;Aricia agestis;NA;0.001;Aricia agestis;0.996102156007606;
JX678150;Lepidoptera;Aricia cramera;Aricia cramera;1;Aricia cramera;0.999994172405776;
JX678151;Lepidoptera;Aricia agestis;NA;0.12;Aricia agestis;0.991347816891068;
JX678152;Lepidoptera;Aricia agestis;NA;0.07;Aricia agestis;0.987216246615008;
JX678166;Lepidoptera;Polyommatus icarus;Polyommatus icarus;0.003;Polyommatus icarus;0.999850577436282;
JX678168;Lepidoptera;Aricia artaxerxes;NA;0.002;Aricia artaxerxes;0.99972102469214;
KC208818;Lepidoptera;Helleia helle;Lycaena helle;1;Lycaena helle;0.999999988262175;
KC208819;Lepidoptera;Helleia helle;Lycaena helle;1;Lycaena helle;0.999999988262175;
KC208820;Lepidoptera;Helleia helle;Lycaena helle;1;Lycaena helle;0.999999988262175;
KC208821;Lepidoptera;Helleia helle;Lycaena helle;1;Lycaena helle;0.999999988262175;
KC208822;Lepidoptera;Helleia helle;Lycaena helle;1;Lycaena helle;0.999999988262175;
KC208823;Lepidoptera;Helleia helle;Lycaena helle;1;Lycaena helle;0.999999988262175;
KC208846;Lepidoptera;Helleia helle;Lycaena helle;1;Lycaena helle;0.999999988262175;
KC208847;Lepidoptera;Helleia helle;Lycaena helle;1;Lycaena helle;0.999999988262175;
KC208848;Lepidoptera;Helleia helle;Lycaena helle;1;Lycaena helle;0.999999988262175;
KC208849;Lepidoptera;Helleia helle;Lycaena helle;1;Lycaena helle;0.999999988262175;
KC462812;Lepidoptera;Pieris napi;NA;NA;NA;0.999987701561402;
KC462816;Lepidoptera;Pieris rapae;NA;NA;Pieris rapae;0.999992588119495;
KC462817;Lepidoptera;Pieris rapae;NA;0.206;Pieris rapae;0.999929773639513;
KC462818;Lepidoptera;Pieris rapae;Pieris rapae;0.005;Pieris rapae;0.999986343554325;
KC465909;Lepidoptera;Melitaea cinxia;Melitaea cinxia;0.02;Melitaea cinxia;0.99999999996777;
KC465910;Lepidoptera;Melitaea cinxia;Melitaea cinxia;NA;Melitaea cinxia;0.999999999998977;
KC465911;Lepidoptera;Melitaea cinxia;Melitaea cinxia;0.014;Melitaea cinxia;0.999999999991047;
KC465912;Lepidoptera;Melitaea cinxia;Melitaea cinxia;0.014;Melitaea cinxia;0.999999999991047;
KC465913;Lepidoptera;Melitaea cinxia;NA;0.069;Melitaea cinxia;0.999999999934317;
KC465914;Lepidoptera;Melitaea cinxia;NA;0.069;Melitaea cinxia;0.999999999934317;
KC465915;Lepidoptera;Melitaea cinxia;NA;0.076;Melitaea cinxia;0.999999999980759;
KC465916;Lepidoptera;Melitaea cinxia;Melitaea cinxia;0.002;Melitaea cinxia;0.999999999940655;
KC465917;Lepidoptera;Melitaea cinxia;Melitaea cinxia;1;Melitaea cinxia;0.999999999947676;
KC618686;Lepidoptera;Lycaena dispar;Lycaena dispar;1;Lycaena dispar;0.999999897700212;
KC660004;Lepidoptera;Lycaena thersamon;NA;NA;Lycaena thersamon;0.921022252798935;
KC676695;Lepidoptera;Lycaena phlaeas;Lycaena phlaeas;1;Lycaena phlaeas;0.999999637440036;
KC676696;Lepidoptera;Thecla betulae;Thecla betulae;1;Thecla betulae;0.999999999219568;
KC676699;Lepidoptera;Cupido argiades;NA;0.164;Cupido argiades;0.775010632974806;
KC676700;Lepidoptera;Cupido osiris;Cupido osiris;1;Cupido osiris;0.999968122272701;
KC676703;Lepidoptera;Aricia agestis;NA;0.05;Aricia agestis;0.992504108972964;
KC676704;Lepidoptera;Aricia artaxerxes;NA;0.002;Aricia artaxerxes;0.999288496025259;
KC692329;Lepidoptera;Scolitantides orion;Scolitantides orion;1;Scolitantides orion;0.999999636228049;
KF491989;Lepidoptera;Parnassius apollo;Parnassius apollo;1;Parnassius apollo;0.995413498609845;
KF647226;Lepidoptera;Lycaena virgaureae;NA;0.147;Lycaena virgaureae;0.998843375757078;
KF647230;Lepidoptera;Plebejus idas;Plebejus idas;0.002;Plebejus idas;0.85449597634936;
KF647231;Lepidoptera;Helleia helle;Lycaena helle;1;Lycaena helle;0.99999997384478;
KF647233;Lepidoptera;Polyommatus eros;NA;NA;NA;0.999999155867694;
KF647235;Lepidoptera;Polyommatus dorylas;Polyommatus dorylas;0.001;Polyommatus dorylas;0.977714590772569;
KF647237;Lepidoptera;Polyommatus damon;Polyommatus damon;1;Polyommatus damon;0.999983061722399;
KF647242;Lepidoptera;Celastrina argiolus;Celastrina argiolus;NA;Celastrina argiolus;0.999014219778089;
KF647245;Lepidoptera;Glaucopsyche alexis;NA;NA;Glaucopsyche alexis;0.95304363372556;
KF647247;Lepidoptera;Cupido alcetas;Cupido alcetas;0.001;Cupido alcetas;0.985475764062565;
KF709970;Lepidoptera;Aporia crataegi;NA;NA;Aporia crataegi;0.99999593396151;
KF709971;Lepidoptera;Aporia crataegi;NA;NA;Aporia crataegi;0.99999593396151;
KF709972;Lepidoptera;Aporia crataegi;NA;NA;Aporia crataegi;0.99999593396151;
KF723539;Lepidoptera;Melitaea britomartis;NA;0.294;Melitaea ambigua;0.925280550686365;
KF723544;Lepidoptera;Minois dryas;Minois dryas;0.012;Minois dryas;0.932618420387345;
KF723548;Lepidoptera;Fabriciana niobe;NA;0.035;Fabriciana niobe;0.949480222958778;
KF723550;Lepidoptera;Brenthis ino;Brenthis ino;0.998;Brenthis ino;0.995325118916732;
KF834369;Lepidoptera;Lysandra hispana;Lysandra hispana;NA;NA;0.732361207330183;

KF860851;Lepidoptera;Polyommatus amandus;Polyommatus amandus;0.021;Polyommatus amandus;0.999824792434497;
KF860852;Lepidoptera;Plebejus argus;NA;0.001;Plebejus argus;0.851872820025762;
KF860853;Lepidoptera;Lampides boeticus;Lampides boeticus;1;Lampides boeticus;0.99999997755657;
KF860855;Lepidoptera;Polyommatus daphnis;NA;0.004;Polyommatus daphnis;0.899187085008227;
KF860856;Lepidoptera;Aricia eumedon;NA;0.058;Aricia eumedon;0.989248156947127;
KF860857;Lepidoptera;Cupido minimus;NA;0.002;Cupido minimus;0.966044066419617;
KF860860;Lepidoptera;Cyaniris semiargus;Cyaniris semiargus;NA;Cyaniris semiargus;0.99543201085201;
KF860861;Lepidoptera;Polyommatus thersites;Polyommatus thersites;0.011;Polyommatus thersites;0.999991576769764;
KJ547676;Lepidoptera;Pararge aegeria;Pararge aegeria;1;NA;0.876928955314701;
KJ638698;Lepidoptera;Melitaea trivia;Melitaea trivia;1;Melitaea trivia;0.999999999975302;
KJ638699;Lepidoptera;Melitaea trivia;Melitaea trivia;1;Melitaea trivia;0.999999999973596;
KJ648990;Lepidoptera;Nymphalis c-album;NA;0.006;NA;0.999996732695115;
KJ649004;Lepidoptera;Vanessa atalanta;Vanessa atalanta;1;Vanessa atalanta;0.999999999698275;
KJ649005;Lepidoptera;Aglais urticae;NA;0.002;Aglais urticae;0.987437630127445;
KJ649006;Lepidoptera;Araschnia levana;Araschnia levana;1;Araschnia levana;0.999999641700306;
KJ649007;Lepidoptera;Vanessa cardui;Vanessa cardui;1;Vanessa cardui;0.99999203779212;
KM020827;Lepidoptera;Maniola jurtina;NA;NA;Maniola jurtina;0.970049180822518;
KM020828;Lepidoptera;Maniola jurtina;NA;NA;Maniola jurtina;0.953827931447544;
KM020829;Lepidoptera;Maniola jurtina;NA;0.165;NA;0.999999999999109;
KM020830;Lepidoptera;Maniola jurtina;NA;0.001;Maniola jurtina;0.939220105860875;
KM020831;Lepidoptera;Maniola jurtina;Maniola jurtina;0.001;Maniola jurtina;0.953764054001816;
KM020832;Lepidoptera;Maniola jurtina;NA;0.005;NA;0.999999999998626;
KM020833;Lepidoptera;Maniola jurtina;Maniola jurtina;0.001;Maniola jurtina;0.953764054001816;
KM020834;Lepidoptera;Maniola jurtina;NA;0.038;NA;0.999999999999524;
KM020835;Lepidoptera;Maniola jurtina;NA;0.001;Maniola jurtina;0.939220105860875;
KM020836;Lepidoptera;Maniola jurtina;NA;0.001;Maniola jurtina;0.939220105860875;
KM020837;Lepidoptera;Maniola jurtina;NA;0.153;Maniola jurtina;0.959231900673445;
KM020838;Lepidoptera;Maniola jurtina;NA;0.001;Maniola jurtina;0.939220105860875;
KM020839;Lepidoptera;Maniola jurtina;NA;0.038;NA;0.999999999999524;
KM020840;Lepidoptera;Maniola jurtina;NA;0.038;NA;0.999999999999524;
KM020841;Lepidoptera;Maniola jurtina;NA;0.038;NA;0.999999999999524;
KM020842;Lepidoptera;Maniola jurtina;NA;0.001;Maniola jurtina;0.939220105860875;
KM020843;Lepidoptera;Maniola jurtina;NA;0.001;Maniola jurtina;0.922911334333417;
KM020844;Lepidoptera;Maniola jurtina;NA;0.001;Maniola jurtina;0.939220105860875;
KM020845;Lepidoptera;Maniola jurtina;NA;0.163;NA;0.999999999999256;
KM020847;Lepidoptera;Maniola jurtina;NA;NA;Maniola jurtina;0.932842454558237;
KM020848;Lepidoptera;Maniola jurtina;NA;0.038;NA;0.999999999999524;
KM020850;Lepidoptera;Maniola jurtina;NA;0.001;Maniola jurtina;0.939220105860875;
KM020851;Lepidoptera;Maniola jurtina;NA;0.119;Maniola jurtina;0.853294047700275;
KM020852;Lepidoptera;Maniola jurtina;NA;0.119;Maniola jurtina;0.853294047700275;
KM020853;Lepidoptera;Maniola jurtina;NA;0.038;NA;0.999999999999524;
KM020855;Lepidoptera;Maniola jurtina;NA;0.038;NA;0.999999999999524;
KM020858;Lepidoptera;Maniola jurtina;NA;0.038;NA;0.999999999999524;
KM020860;Lepidoptera;Maniola jurtina;NA;0.038;NA;0.999999999999524;
KM020861;Lepidoptera;Maniola jurtina;NA;0.119;Maniola jurtina;0.853294047700275;
KM020862;Lepidoptera;Maniola jurtina;NA;0.038;NA;0.999999999999524;
KM020864;Lepidoptera;Maniola jurtina;NA;0.038;NA;0.999999999999524;
KM020866;Lepidoptera;Maniola jurtina;NA;0.038;NA;0.999999999999524;
KM020867;Lepidoptera;Maniola jurtina;NA;0.157;Maniola jurtina;0.72935823378201;
KM020868;Lepidoptera;Maniola jurtina;NA;NA;Maniola jurtina;0.940008002647499;
KM020870;Lepidoptera;Maniola jurtina;NA;0.119;Maniola jurtina;0.853294047700275;
KM020871;Lepidoptera;Maniola jurtina;NA;0.038;NA;0.999999999999524;
KM033847;Lepidoptera;Maniola jurtina;NA;0.003;Maniola jurtina;0.93622146239045;
KM033848;Lepidoptera;Maniola jurtina;NA;0.005;Maniola jurtina;0.714129421849189;
KM033849;Lepidoptera;Maniola jurtina;NA;0.005;Maniola jurtina;0.714129421849189;
KM033850;Lepidoptera;Maniola jurtina;NA;0.001;Maniola jurtina;0.939220105860875;
KM033852;Lepidoptera;Maniola jurtina;NA;0.001;Maniola jurtina;0.939220105860875;
KM033853;Lepidoptera;Maniola jurtina;NA;0.038;NA;0.999999999999524;
KM033855;Lepidoptera;Maniola jurtina;NA;0.146;NA;0.999999999999341;
KM033858;Lepidoptera;Maniola jurtina;NA;0.157;Maniola jurtina;0.72935823378201;
KM033859;Lepidoptera;Maniola jurtina;NA;0.015;NA;0.999999999999554;
KM033860;Lepidoptera;Maniola jurtina;NA;0.038;NA;0.999999999999524;
KM033861;Lepidoptera;Maniola jurtina;NA;0.038;NA;0.999999999999524;
KM033862;Lepidoptera;Maniola jurtina;NA;0.001;Maniola jurtina;0.939220105860875;
KM033864;Lepidoptera;Maniola jurtina;NA;NA;Maniola jurtina;0.746095236282775;
KM033865;Lepidoptera;Maniola jurtina;NA;0.038;NA;0.999999999999524;
KM033866;Lepidoptera;Maniola jurtina;NA;0.038;NA;0.999999999999524;
KM033872;Lepidoptera;Maniola jurtina;NA;0.038;NA;0.999999999999524;
KM033873;Lepidoptera;Maniola jurtina;NA;NA;NA;0.999999999998784;
KM033874;Lepidoptera;Maniola jurtina;NA;0.038;NA;0.999999999999524;
KM033875;Lepidoptera;Maniola jurtina;NA;0.008;Maniola jurtina;0.769922062887517;
KM033876;Lepidoptera;Maniola jurtina;NA;0.038;NA;0.999999999999524;
KM033877;Lepidoptera;Maniola jurtina;NA;0.038;NA;0.999999999999524;
KM033878;Lepidoptera;Maniola jurtina;NA;0.038;NA;0.999999999999524;
KM033879;Lepidoptera;Maniola jurtina;NA;0.038;NA;0.999999999999524;

KM033880;Lepidoptera;Maniola jurtina;NA;0.007;NA;0.999999999999192;
KM033881;Lepidoptera;Maniola jurtina;NA;0.038;NA;0.999999999999524;
KM033882;Lepidoptera;Maniola jurtina;NA;0.018;Maniola jurtina;0.718000045815069;
KM033883;Lepidoptera;Maniola jurtina;NA;0.169;NA;0.999999999999053;
KM033884;Lepidoptera;Maniola jurtina;NA;0.038;NA;0.999999999999524;
KM033885;Lepidoptera;Maniola jurtina;NA;0.032;Maniola jurtina;0.713438248133508;
KM033886;Lepidoptera;Maniola jurtina;NA;0.025;Maniola jurtina;0.705010888933137;
KM033888;Lepidoptera;Maniola jurtina;NA;0.02;NA;0.999999999999197;
KM033889;Lepidoptera;Maniola jurtina;NA;NA;NA;0.999999999998784;
KM033893;Lepidoptera;Maniola jurtina;NA;0.008;Maniola jurtina;0.945994690405052;
KM033896;Lepidoptera;Maniola jurtina;NA;0.001;Maniola jurtina;0.939220105860875;
KM033897;Lepidoptera;Maniola jurtina;NA;0.001;Maniola jurtina;0.939220105860875;
KM033898;Lepidoptera;Maniola jurtina;NA;0.013;Maniola jurtina;0.936595709375125;
KM033899;Lepidoptera;Maniola jurtina;NA;0.009;Maniola jurtina;0.952535358390331;
KM033901;Lepidoptera;Maniola jurtina;NA;0.001;Maniola jurtina;0.939220105860875;
KM033902;Lepidoptera;Maniola jurtina;NA;0.157;Maniola jurtina;0.72935823378201;
KM033904;Lepidoptera;Maniola jurtina;NA;0.169;NA;0.999999999999053;
KM033905;Lepidoptera;Maniola jurtina;NA;0.063;Maniola jurtina;0.770025363931686;
KM033906;Lepidoptera;Maniola jurtina;NA;0.119;Maniola jurtina;0.853294047700275;
KM033907;Lepidoptera;Maniola jurtina;NA;0.038;NA;0.999999999999524;
KM033908;Lepidoptera;Maniola jurtina;NA;0.038;NA;0.999999999999524;
KM033909;Lepidoptera;Maniola jurtina;NA;0.001;Maniola jurtina;0.939220105860875;
KM033910;Lepidoptera;Maniola jurtina;NA;0.001;Maniola jurtina;0.939220105860875;
KM033911;Lepidoptera;Maniola jurtina;NA;0.001;Maniola jurtina;0.939220105860875;
KM033912;Lepidoptera;Maniola jurtina;NA;0.001;Maniola jurtina;0.939220105860875;
KM033914;Lepidoptera;Maniola jurtina;NA;0.001;Maniola jurtina;0.939220105860875;
KM033915;Lepidoptera;Maniola jurtina;NA;0.146;NA;0.999999999999341;
KM033916;Lepidoptera;Maniola jurtina;NA;0.038;NA;0.999999999999524;
KM033917;Lepidoptera;Maniola jurtina;NA;0.038;NA;0.999999999999524;
KM033918;Lepidoptera;Maniola jurtina;NA;0.038;NA;0.999999999999524;
KM033919;Lepidoptera;Maniola jurtina;NA;0.157;Maniola jurtina;0.72935823378201;
KM033920;Lepidoptera;Maniola jurtina;NA;0.001;Maniola jurtina;0.939220105860875;
KM033922;Lepidoptera;Maniola jurtina;NA;0.004;Maniola jurtina;0.86894499343177;
KM033923;Lepidoptera;Maniola jurtina;NA;0.001;Maniola jurtina;0.939220105860875;
KM033924;Lepidoptera;Maniola jurtina;NA;0.038;NA;0.999999999999524;
KM033925;Lepidoptera;Maniola jurtina;NA;0.001;Maniola jurtina;0.939220105860875;
KM033926;Lepidoptera;Maniola jurtina;NA;0.038;NA;0.999999999999524;
KM033927;Lepidoptera;Maniola jurtina;NA;0.022;NA;0.999999999999563;
KM033928;Lepidoptera;Maniola jurtina;NA;0.001;Maniola jurtina;0.939220105860875;
KM033929;Lepidoptera;Maniola jurtina;NA;0.001;Maniola jurtina;0.939220105860875;
KM033930;Lepidoptera;Maniola jurtina;NA;0.001;Maniola jurtina;0.939220105860875;
KM033935;Lepidoptera;Maniola jurtina;NA;0.023;Maniola jurtina;0.732198397368727;
KM033936;Lepidoptera;Maniola jurtina;NA;0.001;Maniola jurtina;0.939220105860875;
KM033937;Lepidoptera;Maniola jurtina;NA;0.008;Maniola jurtina;0.721530652332321;
KM033939;Lepidoptera;Maniola jurtina;NA;0.008;Maniola jurtina;0.721530652332321;
KM033940;Lepidoptera;Maniola jurtina;NA;0.038;NA;0.999999999999524;
KM033941;Lepidoptera;Maniola jurtina;NA;0.001;Maniola jurtina;0.939220105860875;
KM196530;Lepidoptera;Erebia euryale;NA;NA;Erebia euryale;0.911949726023156;
KM459029;Lepidoptera;Aricia agestis;NA;0.067;Aricia agestis;0.999509743227786;
KM459030;Lepidoptera;Aricia agestis;NA;0.253;Aricia agestis;0.996350017387504;
KM459031;Lepidoptera;Aricia agestis;NA;0.067;Aricia agestis;0.999509743227786;
KM459032;Lepidoptera;Aricia agestis;NA;NA;Aricia agestis;0.993980512246517;
KM459033;Lepidoptera;Aricia agestis;NA;0.253;Aricia agestis;0.996350017387504;
KM459034;Lepidoptera;Aricia agestis;NA;0.253;Aricia agestis;0.996350017387504;
KM459035;Lepidoptera;Aricia agestis;NA;0.253;Aricia agestis;0.996350017387504;
KM459036;Lepidoptera;Aricia agestis;NA;0.003;Aricia agestis;0.998094186768341;
KM459037;Lepidoptera;Aricia agestis;NA;0.253;Aricia agestis;0.996350017387504;
KM459038;Lepidoptera;Aricia agestis;NA;0.069;Aricia agestis;0.995918114091591;
KM459039;Lepidoptera;Aricia agestis;NA;0.253;Aricia agestis;0.996350017387504;
KM459040;Lepidoptera;Aricia agestis;NA;0.389;Aricia agestis;0.995319636523153;
KM459041;Lepidoptera;Aricia agestis;NA;NA;Aricia agestis;0.993980512246517;
KM459042;Lepidoptera;Aricia agestis;Aricia agestis;1;Aricia agestis;0.985110124660693;
KM459043;Lepidoptera;Aricia agestis;NA;0.253;Aricia agestis;0.996350017387504;
KM459044;Lepidoptera;Aricia agestis;NA;0.004;Aricia agestis;0.994726192422807;
KM459045;Lepidoptera;Aricia agestis;NA;NA;Aricia agestis;0.993980512246517;
KM459046;Lepidoptera;Aricia agestis;Aricia agestis;0.002;Aricia agestis;0.99978486076089;
KM459047;Lepidoptera;Aricia agestis;Aricia agestis;1;Aricia agestis;0.979315827401608;
KM459048;Lepidoptera;Aricia agestis;Aricia agestis;1;Aricia agestis;0.979315827401608;
KM459049;Lepidoptera;Aricia agestis;Aricia agestis;1;Aricia agestis;0.979315827401608;
KM459050;Lepidoptera;Aricia agestis;Aricia agestis;1;Aricia agestis;0.979315827401608;
KM459051;Lepidoptera;Aricia agestis;Aricia agestis;1;Aricia agestis;0.979315827401608;
KM459052;Lepidoptera;Aricia agestis;NA;NA;Aricia agestis;0.990477247955767;
KM459053;Lepidoptera;Aricia agestis;Aricia agestis;0.001;Aricia agestis;0.972120797608056;
KM459054;Lepidoptera;Aricia agestis;Aricia agestis;1;Aricia agestis;0.983952687998024;
KM459055;Lepidoptera;Aricia agestis;NA;NA;Aricia agestis;0.990477247955767;
KM459056;Lepidoptera;Aricia agestis;NA;0.038;Aricia agestis;0.993235151962273;
KM459057;Lepidoptera;Aricia agestis;Aricia agestis;0.002;Aricia agestis;0.962305544143764;
KM459058;Lepidoptera;Aricia agestis;Aricia agestis;0.001;Aricia agestis;0.972120797608056;

KM459059;Lepidoptera;Aricia agestis;Aricia agestis;0.001;Aricia agestis;0.972120797608056;
KM459060;Lepidoptera;Aricia agestis;Aricia agestis;1;Aricia agestis;0.979315827401608;
KM459061;Lepidoptera;Aricia agestis;Aricia agestis;1;Aricia agestis;0.979315827401608;
KM459062;Lepidoptera;Aricia agestis;Aricia agestis;1;Aricia agestis;0.988437755993415;
KM459063;Lepidoptera;Aricia agestis;Aricia agestis;1;Aricia agestis;0.979315827401608;
KM459064;Lepidoptera;Aricia agestis;Aricia agestis;1;Aricia agestis;0.979315827401608;
KM459065;Lepidoptera;Aricia agestis;Aricia agestis;1;Aricia agestis;0.979315827401608;
KM459066;Lepidoptera;Aricia agestis;Aricia agestis;1;Aricia agestis;0.988018465556624;
KM459067;Lepidoptera;Aricia agestis;Aricia agestis;1;Aricia agestis;0.979315827401608;
KM459068;Lepidoptera;Aricia agestis;NA;0.067;Aricia agestis;0.999509743227786;
KM459069;Lepidoptera;Aricia agestis;NA;NA;Aricia agestis;0.996930365249266;
KM459070;Lepidoptera;Aricia agestis;Aricia agestis;0.001;Aricia agestis;0.972120797608056;
KM459071;Lepidoptera;Aricia agestis;NA;0.099;Aricia agestis;0.994857168775148;
KM459072;Lepidoptera;Aricia agestis;NA;0.253;Aricia agestis;0.996350017387504;
KM459073;Lepidoptera;Aricia agestis;NA;0.067;Aricia agestis;0.999509743227786;
KM459074;Lepidoptera;Aricia agestis;NA;0.046;Aricia agestis;0.999133913416159;
KM459075;Lepidoptera;Aricia agestis;NA;0.253;Aricia agestis;0.996350017387504;
KM459076;Lepidoptera;Aricia agestis;NA;0.253;Aricia agestis;0.996350017387504;
KM459077;Lepidoptera;Aricia agestis;NA;0.003;Aricia agestis;0.998094186768341;
KM459078;Lepidoptera;Aricia agestis;NA;0.278;Aricia agestis;0.993115831599498;
KM459079;Lepidoptera;Aricia agestis;Aricia agestis;1;Aricia agestis;0.988018465556624;
KM459080;Lepidoptera;Aricia agestis;Aricia agestis;1;Aricia agestis;0.988018465556624;
KM459081;Lepidoptera;Aricia agestis;NA;0.253;Aricia agestis;0.996350017387504;
KM459082;Lepidoptera;Aricia agestis;NA;0.253;Aricia agestis;0.996350017387504;
KM459083;Lepidoptera;Aricia agestis;Aricia agestis;0.001;Aricia agestis;0.972120797608056;
KM459084;Lepidoptera;Aricia agestis;Aricia agestis;0.001;Aricia agestis;0.972120797608056;
KM459085;Lepidoptera;Aricia agestis;Aricia agestis;0.002;Aricia agestis;0.99978486076089;
KM459086;Lepidoptera;Aricia agestis;NA;0.253;Aricia agestis;0.996350017387504;
KM459087;Lepidoptera;Aricia agestis;NA;0.253;Aricia agestis;0.996350017387504;
KM459088;Lepidoptera;Aricia agestis;NA;0.253;Aricia agestis;0.996350017387504;
KM459089;Lepidoptera;Aricia agestis;NA;0.03;Aricia agestis;0.999295990875121;
KM459090;Lepidoptera;Aricia agestis;NA;0.101;Aricia agestis;0.993514047526397;
KM459091;Lepidoptera;Aricia agestis;Aricia agestis;1;Aricia agestis;0.979315827401608;
KM459092;Lepidoptera;Aricia agestis;Aricia agestis;1;Aricia agestis;0.979315827401608;
KM459093;Lepidoptera;Aricia agestis;Aricia agestis;1;Aricia agestis;0.978996764450968;
KM459094;Lepidoptera;Aricia agestis;Aricia agestis;1;Aricia agestis;0.988018465556624;
KM459095;Lepidoptera;Aricia agestis;NA;0.253;Aricia agestis;0.996350017387504;
KM459096;Lepidoptera;Aricia agestis;NA;0.101;Aricia agestis;0.998279476999417;
KM459097;Lepidoptera;Aricia agestis;NA;0.101;Aricia agestis;0.998279476999417;
KM459098;Lepidoptera;Aricia agestis;NA;0.101;Aricia agestis;0.998279476999417;
KM459099;Lepidoptera;Aricia agestis;NA;0.253;Aricia agestis;0.996350017387504;
KM459100;Lepidoptera;Aricia agestis;NA;0.253;Aricia agestis;0.996350017387504;
KM459101;Lepidoptera;Aricia agestis;Aricia agestis;1;Aricia agestis;0.979315827401608;
KM459102;Lepidoptera;Aricia agestis;Aricia agestis;1;Aricia agestis;0.978864665329184;
KM459103;Lepidoptera;Aricia agestis;NA;0.067;Aricia agestis;0.999509743227786;
KM459104;Lepidoptera;Aricia agestis;Aricia agestis;0.001;Aricia agestis;0.972120797608056;
KM459105;Lepidoptera;Aricia cramera;Aricia cramera;1;Aricia cramera;0.999993731037033;
KM459106;Lepidoptera;Aricia cramera;Aricia cramera;1;Aricia cramera;0.999993731037033;
KM459107;Lepidoptera;Aricia cramera;Aricia cramera;1;Aricia cramera;0.999993731037033;
KM459108;Lepidoptera;Aricia cramera;Aricia cramera;1;Aricia cramera;0.999993731037033;
KM459109;Lepidoptera;Aricia cramera;Aricia cramera;1;Aricia cramera;0.999996788515018;
KM459110;Lepidoptera;Aricia cramera;Aricia cramera;1;Aricia cramera;0.999993731037033;
KM459111;Lepidoptera;Aricia cramera;Aricia cramera;1;Aricia cramera;0.999994105496263;
KM459114;Lepidoptera;Aricia cramera;Aricia cramera;1;Aricia cramera;0.999993731037033;
KM459115;Lepidoptera;Aricia cramera;Aricia cramera;1;Aricia cramera;0.999996788515018;
KM459116;Lepidoptera;Aricia cramera;Aricia cramera;1;Aricia cramera;0.999996788515018;
KM459117;Lepidoptera;Aricia cramera;Aricia cramera;1;Aricia cramera;0.999993731037033;
KM459118;Lepidoptera;Aricia cramera;Aricia cramera;1;Aricia cramera;0.999993731037033;
KM459119;Lepidoptera;Aricia cramera;Aricia cramera;1;Aricia cramera;0.999993731037033;
KM459120;Lepidoptera;Aricia cramera;Aricia cramera;1;Aricia cramera;0.999993731037033;
KM459121;Lepidoptera;Aricia cramera;Aricia cramera;1;Aricia cramera;0.999993731037033;
KM459122;Lepidoptera;Aricia cramera;Aricia cramera;1;Aricia cramera;0.999993731037033;
KM459123;Lepidoptera;Aricia cramera;Aricia cramera;1;Aricia cramera;0.999996788515018;
KM459124;Lepidoptera;Aricia cramera;Aricia cramera;1;Aricia cramera;0.999996788515018;
KM459125;Lepidoptera;Aricia cramera;Aricia cramera;1;Aricia cramera;0.999993731037033;
KM459126;Lepidoptera;Aricia cramera;Aricia cramera;1;Aricia cramera;0.999993731037033;
KM459127;Lepidoptera;Aricia cramera;Aricia cramera;1;Aricia cramera;0.999993731037033;
KM459128;Lepidoptera;Aricia cramera;Aricia cramera;1;Aricia cramera;0.999993731037033;
KM459129;Lepidoptera;Aricia cramera;Aricia cramera;1;Aricia cramera;0.999993731037033;
KM459130;Lepidoptera;Aricia cramera;Aricia cramera;1;Aricia cramera;0.999993731037033;
KM459131;Lepidoptera;Aricia cramera;Aricia cramera;1;Aricia cramera;0.999993731037033;
KM459132;Lepidoptera;Aricia cramera;Aricia cramera;1;Aricia cramera;0.999996278250043;
KM459133;Lepidoptera;Aricia cramera;Aricia cramera;1;Aricia cramera;0.999996442931848;
KM459134;Lepidoptera;Aricia cramera;Aricia cramera;1;Aricia cramera;0.999993731037033;
KM459135;Lepidoptera;Aricia cramera;Aricia cramera;1;Aricia cramera;0.999990596811572;
KM459142;Lepidoptera;Aricia cramera;Aricia cramera;1;Aricia cramera;0.999993731037033;
KM459144;Lepidoptera;Aricia cramera;Aricia cramera;1;Aricia cramera;0.999969295454852;
KM459145;Lepidoptera;Aricia cramera;Aricia cramera;1;Aricia cramera;0.999993731037033;

KM459146;Lepidoptera;Aricia cramera;Aricia cramera;1;Aricia cramera;0.999993731037033;
KM459149;Lepidoptera;Aricia cramera;Aricia cramera;1;Aricia cramera;0.999993731037033;
KM459150;Lepidoptera;Aricia cramera;Aricia cramera;1;Aricia cramera;0.999996278250043;
KM459151;Lepidoptera;Aricia cramera;Aricia cramera;1;Aricia cramera;0.999993731037033;
KM459152;Lepidoptera;Aricia cramera;Aricia cramera;1;Aricia cramera;0.999996911644581;
KM459153;Lepidoptera;Aricia cramera;Aricia cramera;1;Aricia cramera;0.999993731037033;
KM459154;Lepidoptera;Aricia cramera;Aricia cramera;1;Aricia cramera;0.999995777202847;
KM459155;Lepidoptera;Aricia cramera;Aricia cramera;1;Aricia cramera;0.999995777202847;
KM459156;Lepidoptera;Aricia cramera;Aricia cramera;1;Aricia cramera;0.999993731037033;
KM459157;Lepidoptera;Aricia cramera;Aricia cramera;1;Aricia cramera;0.999993731037033;
KM459158;Lepidoptera;Aricia cramera;Aricia cramera;1;Aricia cramera;0.999993731037033;
KM459159;Lepidoptera;Aricia cramera;Aricia cramera;1;Aricia cramera;0.999993731037033;
KM459160;Lepidoptera;Aricia cramera;Aricia cramera;1;Aricia cramera;0.999993731037033;
KM459161;Lepidoptera;Aricia cramera;Aricia cramera;1;Aricia cramera;0.999995777202847;
KM459162;Lepidoptera;Aricia cramera;Aricia cramera;1;Aricia cramera;0.999993731037033;
KM459163;Lepidoptera;Aricia cramera;Aricia cramera;1;Aricia cramera;0.999993731037033;
KM459164;Lepidoptera;Aricia cramera;Aricia cramera;1;Aricia cramera;0.999993731037033;
KM459165;Lepidoptera;Aricia cramera;Aricia cramera;1;Aricia cramera;0.999995199703488;
KM459166;Lepidoptera;Aricia cramera;Aricia cramera;1;Aricia cramera;0.999993731037033;
KM459167;Lepidoptera;Aricia cramera;Aricia cramera;1;Aricia cramera;0.999993731037033;
KM459168;Lepidoptera;Aricia cramera;Aricia cramera;1;Aricia cramera;0.999993731037033;
KM459169;Lepidoptera;Aricia cramera;Aricia cramera;1;Aricia cramera;0.999993731037033;
KM459170;Lepidoptera;Aricia cramera;Aricia cramera;1;Aricia cramera;0.999993731037033;
KM459171;Lepidoptera;Aricia cramera;Aricia cramera;1;Aricia cramera;0.999993731037033;
KM459172;Lepidoptera;Aricia cramera;Aricia cramera;1;Aricia cramera;0.999993731037033;
KM459173;Lepidoptera;Aricia cramera;Aricia cramera;1;Aricia cramera;0.999993731037033;
KM459174;Lepidoptera;Aricia cramera;Aricia cramera;1;Aricia cramera;0.999993731037033;
KM459175;Lepidoptera;Aricia cramera;Aricia cramera;1;Aricia cramera;0.999993731037033;
KM459176;Lepidoptera;Polyommatus celina;NA;0.072;Polyommatus celina;0.999999999953559;
KM459177;Lepidoptera;Polyommatus celina;NA;0.072;Polyommatus celina;0.999999999953559;
KM459181;Lepidoptera;Polyommatus celina;Polyommatus celina;0.002;Polyommatus celina;0.999999998632774;
KM459182;Lepidoptera;Polyommatus celina;Polyommatus celina;0.002;Polyommatus celina;0.999999998632774;
KM459184;Lepidoptera;Polyommatus celina;Polyommatus celina;0.009;Polyommatus celina;0.999999999949694;
KM459186;Lepidoptera;Polyommatus celina;Polyommatus celina;0.009;Polyommatus celina;0.999999999949694;
KM459187;Lepidoptera;Polyommatus celina;Polyommatus celina;1;Polyommatus celina;0.999999999654818;
KM459188;Lepidoptera;Polyommatus celina;Polyommatus celina;0.999;Polyommatus celina;0.999999999674429;
KM459190;Lepidoptera;Polyommatus celina;NA;0.072;Polyommatus celina;0.999999999953559;
KM459191;Lepidoptera;Polyommatus celina;NA;0.005;Polyommatus celina;0.999999999881851;
KM459195;Lepidoptera;Polyommatus celina;NA;0.072;Polyommatus celina;0.999999999953559;
KM459196;Lepidoptera;Polyommatus celina;NA;0.072;Polyommatus celina;0.999999999953559;
KM459197;Lepidoptera;Polyommatus celina;Polyommatus celina;0.009;Polyommatus celina;0.999999999949694;
KM459198;Lepidoptera;Polyommatus celina;Polyommatus celina;0.009;Polyommatus celina;0.999999999949694;
KM459199;Lepidoptera;Polyommatus celina;Polyommatus celina;0.977;Polyommatus celina;0.999999999949722;
KM459200;Lepidoptera;Polyommatus celina;NA;0.072;Polyommatus celina;0.999999999953559;
KM459201;Lepidoptera;Polyommatus celina;Polyommatus celina;0.007;Polyommatus celina;0.999999999909249;
KM459203;Lepidoptera;Polyommatus celina;Polyommatus celina;0.999;Polyommatus celina;0.999999999963705;
KM459209;Lepidoptera;Polyommatus celina;Polyommatus celina;0.009;Polyommatus celina;0.999999999739714;
KM459210;Lepidoptera;Polyommatus celina;Polyommatus celina;0.009;Polyommatus celina;0.999999999739714;
KM459211;Lepidoptera;Polyommatus celina;Polyommatus celina;0.996;Polyommatus celina;0.999999999866759;
KM459212;Lepidoptera;Polyommatus celina;NA;0.021;Polyommatus celina;0.999999999814662;
KM459213;Lepidoptera;Polyommatus celina;Polyommatus celina;0.009;Polyommatus celina;0.999999999739714;
KM459214;Lepidoptera;Polyommatus celina;Polyommatus celina;0.001;Polyommatus celina;0.999999999833364;
KM459215;Lepidoptera;Polyommatus celina;Polyommatus celina;0.996;Polyommatus celina;0.999999999752333;
KM459216;Lepidoptera;Polyommatus celina;Polyommatus celina;0.996;Polyommatus celina;0.999999999866759;
KM459217;Lepidoptera;Polyommatus celina;Polyommatus celina;0.002;Polyommatus celina;0.999999998632774;
KM459219;Lepidoptera;Polyommatus celina;Polyommatus celina;0.002;Polyommatus celina;0.999999998632774;
KM459220;Lepidoptera;Polyommatus celina;Polyommatus celina;0.002;Polyommatus celina;0.999999998632774;

KM459221;Lepidoptera;Polyommatus celina;Polyommatus celina;0.002;Polyommatus celina;0.999999998632774;
KM459222;Lepidoptera;Polyommatus celina;NA;0.072;Polyommatus celina;0.999999999953559;
KM459223;Lepidoptera;Polyommatus celina;Polyommatus celina;0.009;Polyommatus celina;0.999999999949694;
KM459224;Lepidoptera;Polyommatus celina;Polyommatus celina;0.006;Polyommatus celina;0.99999999979326;
KM459225;Lepidoptera;Polyommatus celina;Polyommatus celina;0.009;Polyommatus celina;0.999999999949694;
KM459226;Lepidoptera;Polyommatus celina;Polyommatus celina;0.001;Polyommatus celina;0.999999999717033;
KM459227;Lepidoptera;Polyommatus celina;Polyommatus celina;0.001;Polyommatus celina;0.999999999717033;
KM459228;Lepidoptera;Polyommatus celina;NA;0.072;Polyommatus celina;0.999999999953559;
KM459229;Lepidoptera;Polyommatus celina;Polyommatus celina;0.007;Polyommatus celina;0.999999999839019;
KM459230;Lepidoptera;Polyommatus celina;NA;0.943;Polyommatus celina;0.999999998954848;
KM459231;Lepidoptera;Polyommatus celina;Polyommatus celina;0.989;Polyommatus celina;0.999999999759865;
KM459232;Lepidoptera;Polyommatus celina;Polyommatus celina;0.001;Polyommatus celina;0.999999999717033;
KM459233;Lepidoptera;Polyommatus celina;Polyommatus celina;0.007;Polyommatus celina;0.999999999839019;
KM459234;Lepidoptera;Polyommatus celina;NA;0.021;Polyommatus celina;0.999999999814662;
KM459235;Lepidoptera;Polyommatus celina;Polyommatus celina;0.994;Polyommatus celina;0.999999999362757;
KM459236;Lepidoptera;Polyommatus celina;Polyommatus celina;0.994;Polyommatus celina;0.999999999766857;
KM459237;Lepidoptera;Polyommatus celina;Polyommatus celina;0.994;Polyommatus celina;0.999999999362757;
KM459238;Lepidoptera;Polyommatus celina;Polyommatus celina;0.007;Polyommatus celina;0.999999999839019;
KM459239;Lepidoptera;Polyommatus celina;Polyommatus celina;0.994;Polyommatus celina;0.999999999362757;
KM459240;Lepidoptera;Polyommatus celina;Polyommatus celina;0.999;Polyommatus celina;0.999999999331862;
KM459241;Lepidoptera;Polyommatus celina;Polyommatus celina;0.001;Polyommatus celina;0.999999999717033;
KM459242;Lepidoptera;Polyommatus celina;Polyommatus celina;0.992;Polyommatus celina;0.999999999676362;
KM459243;Lepidoptera;Polyommatus celina;Polyommatus celina;0.999;Polyommatus celina;0.99999999991357;
KM459244;Lepidoptera;Polyommatus celina;Polyommatus celina;0.001;Polyommatus celina;0.999999999717033;
KM459245;Lepidoptera;Polyommatus celina;Polyommatus celina;0.001;Polyommatus celina;0.999999999717033;
KM459254;Lepidoptera;Polyommatus celina;Polyommatus celina;0.007;Polyommatus celina;0.999999999839019;
KM459255;Lepidoptera;Polyommatus celina;Polyommatus celina;0.007;Polyommatus celina;0.999999999839019;
KM459256;Lepidoptera;Polyommatus celina;Polyommatus celina;0.003;Polyommatus celina;0.99999999996129;
KM459257;Lepidoptera;Polyommatus celina;Polyommatus celina;0.997;Polyommatus celina;0.99999999996291;
KM459258;Lepidoptera;Polyommatus celina;Polyommatus celina;0.003;Polyommatus celina;0.99999999996129;
KM459259;Lepidoptera;Polyommatus celina;Polyommatus celina;0.009;Polyommatus celina;0.999999999949694;
KM459260;Lepidoptera;Polyommatus celina;NA;0.072;Polyommatus celina;0.999999999953559;
KM459261;Lepidoptera;Polyommatus celina;Polyommatus celina;0.009;Polyommatus celina;0.999999999949694;
KM459262;Lepidoptera;Polyommatus celina;NA;0.072;Polyommatus celina;0.999999999953559;
KM459263;Lepidoptera;Polyommatus celina;NA;0.028;Polyommatus celina;0.99999999996308;
KM459264;Lepidoptera;Polyommatus celina;NA;0.004;Polyommatus celina;0.999999999787462;
KM459265;Lepidoptera;Polyommatus celina;Polyommatus celina;0.009;Polyommatus celina;0.999999999949694;
KM459266;Lepidoptera;Polyommatus celina;NA;0.072;Polyommatus celina;0.999999999953559;
KM459267;Lepidoptera;Polyommatus celina;NA;0.072;Polyommatus celina;0.999999999953559;
KM459268;Lepidoptera;Polyommatus celina;NA;0.021;Polyommatus celina;0.999999999814662;
KM459269;Lepidoptera;Polyommatus celina;Polyommatus celina;0.007;Polyommatus celina;0.999999999839019;
KM459270;Lepidoptera;Polyommatus celina;Polyommatus celina;0.007;Polyommatus celina;0.999999999839019;
KM459271;Lepidoptera;Polyommatus celina;NA;0.021;Polyommatus celina;0.999999999814662;
KM459272;Lepidoptera;Polyommatus celina;NA;0.021;Polyommatus celina;0.999999999814662;
KM459273;Lepidoptera;Polyommatus celina;Polyommatus celina;0.007;Polyommatus celina;0.999999999839019;

KM459274;Lepidoptera;Polyommatus celina;Polyommatus celina;0.982;Polyommatus celina;0.999999999352411;
KM459275;Lepidoptera;Polyommatus celina;Polyommatus celina;0.983;Polyommatus celina;0.999999999342975;
KM459276;Lepidoptera;Polyommatus celina;Polyommatus celina;0.007;Polyommatus celina;0.999999999839019;
KM459277;Lepidoptera;Polyommatus celina;NA;0.021;Polyommatus celina;0.999999999814662;
KM459278;Lepidoptera;Polyommatus celina;Polyommatus celina;0.009;Polyommatus celina;0.999999999739714;
KM459279;Lepidoptera;Polyommatus celina;Polyommatus celina;0.001;Polyommatus celina;0.99999999974969;
KM459280;Lepidoptera;Polyommatus celina;NA;0.021;Polyommatus celina;0.999999999814662;
KM459281;Lepidoptera;Polyommatus celina;Polyommatus celina;0.007;Polyommatus celina;0.999999999839019;
KM459282;Lepidoptera;Polyommatus celina;Polyommatus celina;0.983;Polyommatus celina;0.999999999619718;
KM459283;Lepidoptera;Polyommatus celina;Polyommatus celina;0.983;Polyommatus celina;0.999999999379384;
KM459284;Lepidoptera;Polyommatus celina;Polyommatus celina;0.009;Polyommatus celina;0.999999999739714;
KM459285;Lepidoptera;Polyommatus celina;Polyommatus celina;0.023;Polyommatus celina;0.999999999971379;
KM459288;Lepidoptera;Polyommatus celina;Polyommatus celina;0.001;Polyommatus celina;0.999999999717033;
KM459289;Lepidoptera;Polyommatus celina;Polyommatus celina;0.007;Polyommatus celina;0.999999999909107;
KM459290;Lepidoptera;Polyommatus celina;NA;0.067;Polyommatus celina;0.999999999867015;
KM459291;Lepidoptera;Polyommatus celina;NA;0.072;Polyommatus celina;0.999999999953559;
KM459295;Lepidoptera;Polyommatus celina;Polyommatus celina;0.997;Polyommatus celina;0.999999998146563;
KM459296;Lepidoptera;Polyommatus celina;Polyommatus celina;0.001;Polyommatus celina;0.999999999623128;
KM459297;Lepidoptera;Polyommatus celina;Polyommatus celina;0.995;Polyommatus celina;0.999999999552301;
KM459298;Lepidoptera;Polyommatus celina;Polyommatus celina;1;Polyommatus celina;0.999999999639783;
KM459299;Lepidoptera;Polyommatus celina;Polyommatus celina;0.007;Polyommatus celina;0.999999999839019;
KM459300;Lepidoptera;Polyommatus celina;Polyommatus celina;0.999;Polyommatus celina;0.999999999287951;
KM459301;Lepidoptera;Polyommatus celina;NA;0.021;Polyommatus celina;0.999999999814662;
KM459302;Lepidoptera;Polyommatus celina;NA;0.021;Polyommatus celina;0.999999999814662;
KM459303;Lepidoptera;Polyommatus celina;Polyommatus celina;0.992;Polyommatus celina;0.99999999963805;
KM459304;Lepidoptera;Polyommatus celina;Polyommatus celina;0.983;Polyommatus celina;0.999999999652459;
KM459305;Lepidoptera;Polyommatus celina;Polyommatus celina;0.998;Polyommatus celina;0.99999999949253;
KM459306;Lepidoptera;Polyommatus celina;Polyommatus celina;0.001;Polyommatus celina;0.999999999358465;
KM459307;Lepidoptera;Polyommatus celina;Polyommatus celina;0.001;Polyommatus celina;0.999999999841947;
KM459308;Lepidoptera;Polyommatus celina;NA;0.011;Polyommatus celina;0.999999999924682;
KM459309;Lepidoptera;Polyommatus celina;Polyommatus celina;1;Polyommatus celina;0.999999999721325;
KM459310;Lepidoptera;Polyommatus celina;Polyommatus celina;0.001;Polyommatus celina;0.999999999717033;
KM459311;Lepidoptera;Polyommatus celina;Polyommatus celina;0.001;Polyommatus celina;0.999999999717033;
KM459312;Lepidoptera;Polyommatus celina;Polyommatus celina;0.999;Polyommatus celina;0.99999999964686;
KM459313;Lepidoptera;Polyommatus celina;Polyommatus celina;1;Polyommatus celina;0.999999999721325;
KM459314;Lepidoptera;Polyommatus celina;Polyommatus celina;0.009;Polyommatus celina;0.999999999739714;
KM459315;Lepidoptera;Polyommatus celina;Polyommatus celina;0.983;Polyommatus celina;0.999999999652459;
KM459316;Lepidoptera;Polyommatus celina;Polyommatus celina;0.007;Polyommatus celina;0.999999999839019;
KM459317;Lepidoptera;Polyommatus celina;NA;0.021;Polyommatus celina;0.999999999814662;
KM459318;Lepidoptera;Polyommatus celina;Polyommatus celina;0.001;Polyommatus celina;0.99999999974969;
KM459319;Lepidoptera;Polyommatus celina;Polyommatus celina;0.993;Polyommatus celina;0.999999999801673;
KM459320;Lepidoptera;Polyommatus celina;Polyommatus celina;0.001;Polyommatus celina;0.99999999974969;
KM459321;Lepidoptera;Polyommatus icarus;Polyommatus icarus;0.001;Polyommatus icarus;0.998856113719376;

KM459330;Lepidoptera;Polyommatus icarus;Polyommatus icarus;0.002;Polyommatus icarus;0.999346502768464;
KM459331;Lepidoptera;Polyommatus icarus;Polyommatus icarus;0.002;Polyommatus icarus;0.999346502768464;
KM459332;Lepidoptera;Polyommatus icarus;Polyommatus icarus;0.002;Polyommatus icarus;0.999346502768464;
KM459333;Lepidoptera;Polyommatus icarus;Polyommatus icarus;0.002;Polyommatus icarus;0.999346502768464;
KM459335;Lepidoptera;Polyommatus icarus;Polyommatus icarus;0.002;Polyommatus icarus;0.999346502768464;
KM459336;Lepidoptera;Polyommatus icarus;Polyommatus icarus;0.979;Polyommatus icarus;0.999558582389478;
KM459337;Lepidoptera;Polyommatus icarus;Polyommatus icarus;0.002;Polyommatus icarus;0.999346502768464;
KM459341;Lepidoptera;Polyommatus icarus;Polyommatus icarus;0.002;Polyommatus icarus;0.999346502768464;
KM459342;Lepidoptera;Polyommatus icarus;Polyommatus icarus;0.002;Polyommatus icarus;0.999346502768464;
KM459344;Lepidoptera;Polyommatus icarus;Polyommatus icarus;0.999;Polyommatus icarus;0.971790103343284;
KM459346;Lepidoptera;Polyommatus icarus;Polyommatus icarus;0.002;Polyommatus icarus;0.999346502768464;
KM459351;Lepidoptera;Polyommatus icarus;NA;0.003;Polyommatus icarus;0.997760766612533;
KM459356;Lepidoptera;Polyommatus icarus;Polyommatus icarus;0.001;Polyommatus icarus;0.999848859399272;
KM459359;Lepidoptera;Polyommatus icarus;Polyommatus icarus;0.002;Polyommatus icarus;0.999346502768464;
KM459360;Lepidoptera;Polyommatus icarus;NA;0.003;Polyommatus icarus;0.997760766612533;
KM459361;Lepidoptera;Polyommatus icarus;Polyommatus icarus;0.999;Polyommatus icarus;0.971790103343284;
KM459362;Lepidoptera;Polyommatus icarus;NA;0.003;Polyommatus icarus;0.997760766612533;
KM459363;Lepidoptera;Polyommatus icarus;NA;0.003;Polyommatus icarus;0.997760766612533;
KM459366;Lepidoptera;Polyommatus icarus;NA;0.002;Polyommatus icarus;0.998502314961965;
KM459367;Lepidoptera;Polyommatus icarus;NA;0.003;Polyommatus icarus;0.997760766612533;
KM459368;Lepidoptera;Polyommatus icarus;NA;0.006;Polyommatus icarus;0.997965308245128;
KM459369;Lepidoptera;Polyommatus icarus;NA;0.006;Polyommatus icarus;0.997965308245128;
KM459370;Lepidoptera;Polyommatus icarus;NA;0.004;Polyommatus icarus;0.999481683747521;
KM459371;Lepidoptera;Polyommatus icarus;NA;0.006;Polyommatus icarus;0.997965308245128;
KM459372;Lepidoptera;Polyommatus icarus;NA;0.006;Polyommatus icarus;0.997965308245128;
KM459373;Lepidoptera;Polyommatus icarus;NA;0.006;Polyommatus icarus;0.997965308245128;
KM459374;Lepidoptera;Polyommatus icarus;Polyommatus icarus;0.002;Polyommatus icarus;0.999346502768464;
KM459375;Lepidoptera;Polyommatus icarus;Polyommatus icarus;0.001;Polyommatus icarus;0.999851522482238;
KM459376;Lepidoptera;Polyommatus icarus;Polyommatus icarus;0.002;Polyommatus icarus;0.999725320070896;
KM459377;Lepidoptera;Polyommatus icarus;NA;0.004;Polyommatus icarus;0.999481683747521;
KM459378;Lepidoptera;Polyommatus icarus;NA;0.001;Polyommatus icarus;0.994660939114793;
KM459379;Lepidoptera;Polyommatus icarus;Polyommatus icarus;0.001;Polyommatus icarus;0.999851522482238;
KM459380;Lepidoptera;Polyommatus icarus;Polyommatus icarus;0.001;Polyommatus icarus;0.999851522482238;
KM459381;Lepidoptera;Polyommatus icarus;Polyommatus icarus;0.001;Polyommatus icarus;0.999851522482238;
KM459382;Lepidoptera;Polyommatus icarus;Polyommatus icarus;0.001;Polyommatus icarus;0.999851522482238;
KM459383;Lepidoptera;Polyommatus icarus;Polyommatus icarus;0.001;Polyommatus icarus;0.999847102144222;
KM459384;Lepidoptera;Polyommatus icarus;Polyommatus icarus;0.001;Polyommatus icarus;0.999851522482238;
KM459385;Lepidoptera;Polyommatus icarus;Polyommatus icarus;0.001;Polyommatus icarus;0.999851522482238;
KM459386;Lepidoptera;Polyommatus icarus;NA;0.002;Polyommatus icarus;0.998502314961965;
KM459387;Lepidoptera;Polyommatus icarus;NA;0.068;Polyommatus icarus;0.764483966403937;
KM459388;Lepidoptera;Polyommatus icarus;Polyommatus icarus;0.001;Polyommatus icarus;0.999851522482238;
KM459389;Lepidoptera;Polyommatus icarus;Polyommatus icarus;0.995;Polyommatus icarus;0.999841416213754;
KM459390;Lepidoptera;Polyommatus icarus;Polyommatus icarus;0.995;Polyommatus icarus;0.999841416213754;
KM459391;Lepidoptera;Polyommatus icarus;Polyommatus icarus;0.001;Polyommatus icarus;0.999834630680561;
KM459392;Lepidoptera;Polyommatus icarus;Polyommatus icarus;0.996;Polyommatus icarus;0.999718674711025;
KM459393;Lepidoptera;Polyommatus icarus;Polyommatus icarus;0.001;Polyommatus icarus;0.999851522482238;
KM459394;Lepidoptera;Polyommatus icarus;Polyommatus icarus;0.001;Polyommatus icarus;0.999851522482238;

KM459395;Lepidoptera;Polyommatus icarus;Polyommatus icarus;0.002;Polyommatus icarus;0.999653930279044;
KM459396;Lepidoptera;Polyommatus icarus;Polyommatus icarus;0.001;Polyommatus icarus;0.999851522482238;
KM459397;Lepidoptera;Polyommatus icarus;Polyommatus icarus;0.001;Polyommatus icarus;0.999851522482238;
KM459399;Lepidoptera;Polyommatus icarus;Polyommatus icarus;0.001;Polyommatus icarus;0.999811574177771;
KM459401;Lepidoptera;Polyommatus icarus;Polyommatus icarus;0.001;Polyommatus icarus;0.999851522482238;
KM459402;Lepidoptera;Polyommatus icarus;Polyommatus icarus;0.001;Polyommatus icarus;0.999851522482238;
KM459403;Lepidoptera;Polyommatus icarus;Polyommatus icarus;0.001;Polyommatus icarus;0.999851522482238;
KM459404;Lepidoptera;Polyommatus icarus;Polyommatus icarus;0.001;Polyommatus icarus;0.999851522482238;
KM459405;Lepidoptera;Polyommatus icarus;Polyommatus icarus;0.001;Polyommatus icarus;0.999851522482238;
KM459406;Lepidoptera;Polyommatus icarus;Polyommatus icarus;0.001;Polyommatus icarus;0.999851522482238;
KM459408;Lepidoptera;Polyommatus icarus;NA;0.003;Polyommatus icarus;0.998348220031726;
KM459409;Lepidoptera;Polyommatus icarus;Polyommatus icarus;1;Polyommatus icarus;0.988670826309563;
KM459410;Lepidoptera;Polyommatus icarus;Polyommatus icarus;1;Polyommatus icarus;0.988670826309563;
KM459411;Lepidoptera;Polyommatus icarus;Polyommatus icarus;1;Polyommatus icarus;0.988670826309563;
KM459412;Lepidoptera;Polyommatus icarus;Polyommatus icarus;1;Polyommatus icarus;0.988670826309563;
KM459413;Lepidoptera;Polyommatus icarus;Polyommatus icarus;1;Polyommatus icarus;0.988670826309563;
KM459414;Lepidoptera;Polyommatus icarus;NA;0.001;NA;0.999994270558956;
KM459415;Lepidoptera;Polyommatus icarus;Polyommatus icarus;0.001;Polyommatus icarus;0.999851522482238;
KM459416;Lepidoptera;Polyommatus icarus;Polyommatus icarus;0.001;Polyommatus icarus;0.999851522482238;
KM459418;Lepidoptera;Polyommatus icarus;Polyommatus icarus;0.001;Polyommatus icarus;0.999851522482238;
KM459419;Lepidoptera;Polyommatus icarus;Polyommatus icarus;0.001;Polyommatus icarus;0.999851522482238;
KM459420;Lepidoptera;Polyommatus icarus;Polyommatus icarus;0.001;Polyommatus icarus;0.999851522482238;
KM459421;Lepidoptera;Polyommatus icarus;Polyommatus icarus;0.001;Polyommatus icarus;0.999851522482238;
KM459422;Lepidoptera;Polyommatus icarus;NA;0.13;Polyommatus icarus;0.979762695748748;
KM459423;Lepidoptera;Polyommatus icarus;Polyommatus icarus;0.001;Polyommatus icarus;0.999860626666977;
KM459424;Lepidoptera;Polyommatus icarus;Polyommatus icarus;0.995;Polyommatus icarus;0.999841416213754;
KM459425;Lepidoptera;Polyommatus icarus;Polyommatus icarus;0.001;Polyommatus icarus;0.999851522482238;
KM459426;Lepidoptera;Polyommatus icarus;Polyommatus icarus;0.001;Polyommatus icarus;0.999851522482238;
KM459427;Lepidoptera;Polyommatus icarus;NA;0.003;Polyommatus icarus;0.998348220031726;
KM459428;Lepidoptera;Polyommatus icarus;NA;0.004;Polyommatus icarus;0.999481683747521;
KM459429;Lepidoptera;Polyommatus icarus;Polyommatus icarus;0.001;Polyommatus icarus;0.999851522482238;
KM459431;Lepidoptera;Polyommatus icarus;Polyommatus icarus;0.001;Polyommatus icarus;0.999765839960799;
KM459432;Lepidoptera;Polyommatus icarus;Polyommatus icarus;0.001;Polyommatus icarus;0.999851522482238;
KM459433;Lepidoptera;Polyommatus icarus;Polyommatus icarus;0.001;Polyommatus icarus;0.999851522482238;
KM459435;Lepidoptera;Polyommatus icarus;Polyommatus icarus;0.001;Polyommatus icarus;0.999851522482238;
KM459438;Lepidoptera;Polyommatus icarus;Polyommatus icarus;0.998;Polyommatus icarus;0.999597220201636;
KM459439;Lepidoptera;Polyommatus icarus;Polyommatus icarus;0.993;Polyommatus icarus;0.999862835392831;
KM517249;Lepidoptera;Phengaris arion;NA;0.019;NA;0.873942246502214;
KM517284;Lepidoptera;Phengaris teleius;NA;0.491;NA;0.854630020337126;
KM517286;Lepidoptera;Phengaris teleius;NA;0.491;NA;0.854630020337126;
KM517287;Lepidoptera;Phengaris teleius;Phengaris teleius;1;NA;0.854794865866951;
KM517288;Lepidoptera;Phengaris teleius;NA;0.491;NA;0.858264794752326;
KM517289;Lepidoptera;Phengaris teleius;Phengaris teleius;1;NA;0.850887794441426;
KM517290;Lepidoptera;Phengaris teleius;NA;0.491;NA;0.857245891064942;
KM517291;Lepidoptera;Phengaris teleius;NA;0.491;NA;0.854630020337126;
KM517292;Lepidoptera;Phengaris teleius;NA;0.491;NA;0.854630020337126;

KM517293;Lepidoptera;Phengaris teleius;NA;0.491;NA;0.854460238073035;
KM517294;Lepidoptera;Phengaris teleius;NA;0.491;NA;0.857245891064942;
KM517295;Lepidoptera;Phengaris teleius;NA;0.491;NA;0.854630020337126;
KM517296;Lepidoptera;Phengaris teleius;NA;0.491;NA;0.855949517859573;
KM517297;Lepidoptera;Phengaris teleius;NA;0.491;NA;0.854630020337126;
KM517329;Lepidoptera;Phengaris teleius;Phengaris teleius;0.012;Phengaris
teleius;0.938293075893268;
KM517330;Lepidoptera;Phengaris teleius;NA;0.493;Phengaris teleius;0.926779528630756;
KM517331;Lepidoptera;Phengaris teleius;NA;0.493;Phengaris teleius;0.926779528630756;
KM517835;Lepidoptera;Aricia agestis;Aricia agestis;1;Aricia agestis;0.979315827401608;
KM517836;Lepidoptera;Aricia agestis;Aricia agestis;1;Aricia agestis;0.978996764450968;
KM517838;Lepidoptera;Aricia agestis;Aricia agestis;1;Aricia agestis;0.979315827401608;
KM517839;Lepidoptera;Aricia agestis;Aricia agestis;1;Aricia agestis;0.979315827401608;
KM517841;Lepidoptera;Aricia agestis;NA;NA;Aricia agestis;0.993980512246517;
KM517842;Lepidoptera;Aricia agestis;Aricia agestis;1;Aricia agestis;0.988437755993415;
KM517846;Lepidoptera;Aricia agestis;Aricia agestis;1;Aricia agestis;0.979315827401608;
KM517847;Lepidoptera;Aricia agestis;Aricia agestis;1;Aricia agestis;0.979315827401608;
KM517848;Lepidoptera;Aricia cramera;Aricia cramera;1;Aricia cramera;0.999993153527516;
KM517849;Lepidoptera;Polyommatus celina;NA;0.072;Polyommatus celina;0.999999999953559;
KM517850;Lepidoptera;Aricia cramera;Aricia cramera;1;Aricia cramera;0.999992327687311;
KM517853;Lepidoptera;Aricia cramera;Aricia cramera;1;Aricia cramera;0.999993731037033;
KM517854;Lepidoptera;Aricia cramera;Aricia cramera;1;Aricia cramera;0.999993731037033;
KM517855;Lepidoptera;Aricia cramera;Aricia cramera;1;Aricia cramera;0.999991524313082;
KM517859;Lepidoptera;Polyommatus icarus;NA;0.001;Polyommatus icarus;0.999367894299281;
KM517860;Lepidoptera;Aricia cramera;Aricia cramera;1;Aricia cramera;0.999993731037033;
KM517861;Lepidoptera;Polyommatus celina;Polyommatus celina;0.002;Polyommatus
celina;0.999999999641517;
KM517863;Lepidoptera;Aricia cramera;Aricia cramera;1;Aricia cramera;0.999993731037033;
KM517864;Lepidoptera;Polyommatus celina;Polyommatus celina;0.009;Polyommatus
celina;0.999999999949694;
KM571976;Lepidoptera;Aricia artaxerxes;Plebejus argus;1;Aricia artaxerxes;0.999743187199922;
KM571986;Lepidoptera;Aglais urticae;NA;0.002;Aglais urticae;0.987497593219019;
KM572099;Lepidoptera;Lycaena phlaeas;Lycaena phlaeas;1;Lycaena phlaeas;0.999999986385689;
KM572133;Lepidoptera;Lasiommata maera;Lasiommata maera;1;Lasiommata maera;0.999995617120616;
KM572165;Lepidoptera;Aricia eumedon;Eumedonia eumedon;0.002;Eumedonia eumedon;0.997434540282035;
KM572201;Lepidoptera;Nymphalis c-album;NA;0.076;NA;0.999999389450531;
KM572268;Lepidoptera;Pyrgus alveus;NA;0.003;NA;0.999999999997732;
KM572272;Lepidoptera;Aricia artaxerxes;Plebejus argus;1;Aricia artaxerxes;0.999743187199922;
KM572320;Lepidoptera;Aricia artaxerxes;Plebejus argus;1;Aricia artaxerxes;0.999743187199922;
KM572347;Lepidoptera;Fabriciana niobe;NA;0.001;Fabriciana niobe;0.989214650993052;
KM572370;Lepidoptera;Gonepteryx rhamni;NA;0.474;Gonepteryx rhamni;0.982076396127858;
KM572382;Lepidoptera;Colias palaeno;NA;0.002;NA;0.999999999983681;
KM572384;Lepidoptera;Brenthis ino;Brenthis ino;1;Brenthis ino;0.999238356896675;
KM572430;Lepidoptera;Colias hyale;Colias hyale;0.015;Colias hyale;0.999997884296128;
KM572441;Lepidoptera;Boloria euphrosyne;Boloria euphrosyne;0.001;Boloria
euphrosyne;0.999985640170951;
KM572512;Lepidoptera;Pyrgus malvae;Pyrgus malvae;NA;Pyrgus malvae;0.980121140374433;
KM572513;Lepidoptera;Aricia artaxerxes;Plebejus argus;1;Aricia artaxerxes;0.999743187199922;
KM572519;Lepidoptera;Anthocharis cardamines;Anthocharis cardamines;1;Anthocharis
cardamines;0.999999904052946;
KM572538;Lepidoptera;Colias palaeno;NA;0.002;NA;0.999999999983681;
KM572581;Lepidoptera;Lycaena hippothoe;NA;NA;Lycaena hippothoe;0.928973453460131;
KM572599;Lepidoptera;Plebejus idas;NA;0.007;NA;0.999546324331549;
KM572712;Lepidoptera;Melitaea diamina;NA;0.149;Melitaea diamina;0.999999996592464;
KM572728;Lepidoptera;Ochlodes sylvanus;NA;0.325;Ochlodes sylvanus;0.995935281765954;
KM572761;Lepidoptera;Pyrgus malvae;Pyrgus malvae;NA;Pyrgus malvae;0.980121140374433;
KM572766;Lepidoptera;Apatura iris;Apatura iris;1;Apatura iris;0.999999999999972;
KM572775;Lepidoptera;Callophrys rubi;NA;0.003;Callophrys rubi;0.997328639918919;
KM572807;Lepidoptera;Aphantopus hyperantus;Aphantopus hyperantus;0.002;Aphantopus
hyperantus;0.999999556235408;
KM572834;Lepidoptera;Coenonympha pamphilus;Coenonympha pamphilus;1;Coenonympha
pamphilus;0.999999999999744;
KM572903;Lepidoptera;Argynnis aglaja;NA;NA;Speyeria aglaja;0.872991147917853;
KM572979;Lepidoptera;Pararge aegeria;Pararge aegeria;1;Pararge aegeria;0.999999999963165;
KM573008;Lepidoptera;Cyaniris semiargus;NA;NA;Cyaniris semiargus;0.999051841766953;
KM573033;Lepidoptera;Aricia artaxerxes;Plebejus argus;1;Aricia artaxerxes;0.999743187199922;
KM573077;Lepidoptera;Lycaena hippothoe;NA;NA;Lycaena hippothoe;0.96171722036274;
KM573204;Lepidoptera;Aphantopus hyperantus;Aphantopus hyperantus;0.001;Aphantopus
hyperantus;0.999990984082303;
KM573218;Lepidoptera;Aporia crataegi;NA;0.008;Aporia crataegi;0.999995700720296;
KM573234;Lepidoptera;Apatura ilia;NA;0.165;Apatura ilia;0.956796812031481;
KM573246;Lepidoptera;Carterocephalus palaemon;NA;0.052;Carterocephalus
palaemon;0.728445051795325;
KM573281;Lepidoptera;Glaucopsyche alexis;NA;NA;Glaucopsyche alexis;0.979381169624069;
KM573316;Lepidoptera;Cyaniris semiargus;NA;NA;Cyaniris semiargus;0.999299367533383;
KM573324;Lepidoptera;Aricia artaxerxes;Plebejus argus;1;Aricia artaxerxes;0.999743187199922;
KM573345;Lepidoptera;Pararge aegeria;Pararge aegeria;1;Pararge aegeria;0.999999999963165;

KM573383;Lepidoptera;Thymelicus lineola;Thymelicus lineola;1;Thymelicus lineola;0.999995927238524;
KM573397;Lepidoptera;Coenonympha glycerion;NA;0.162;Coenonympha glycerion;0.999998620111782;
KM573493;Lepidoptera;Plebejus argus;NA;0.036;Plebejus argus;0.988160277018267;
KM573513;Lepidoptera;Aricia artaxerxes;Plebejus argus;1;Aricia artaxerxes;0.999743187199922;
KM573527;Lepidoptera;Pyrgus andromedae;Pyrgus andromedae;0.001;Pyrgus andromedae;0.998828669843251;
KM573528;Lepidoptera;Aricia eumedon;Eumedonia eumedon;0.002;Eumedonia eumedon;0.997434540282035;
KM573540;Lepidoptera;Erebia pandrose;Erebia pandrose;0.003;Erebia pandrose;0.993608645577247;
KM573555;Lepidoptera;Lycaena virgaureae;NA;0.133;Lycaena virgaureae;0.999273473352577;
KM573575;Lepidoptera;Thymelicus lineola;Thymelicus lineola;1;Thymelicus lineola;0.999962055342616;
KM573621;Lepidoptera;Vanessa atalanta;Vanessa atalanta;1;Vanessa atalanta;0.999999999757762;
KM573649;Lepidoptera;Parnassius mnemosyne;Parnassius mnemosyne;1;Parnassius mnemosyne;0.999999584180132;
KM573651;Lepidoptera;Cupido minimus;NA;0.006;Cupido minimus;0.999768371759258;
KM573699;Lepidoptera;Aricia artaxerxes;Plebejus argus;1;Aricia artaxerxes;0.999743187199922;
KM573714;Lepidoptera;Fabriciana adippe;NA;NA;Fabriciana adippe;0.900255826442102;
KM573716;Lepidoptera;Celastrina argiolus;Celastrina argiolus;1;Celastrina argiolus;0.999527385150531;
KM592969;Lepidoptera;Aphantopus hyperantus;Aphantopus hyperantus;0.999;NA;0.868572173240311;
KM972602;Lepidoptera;Lysandra coridon gennargenti;NA;NA;NA;0.992233859749513;
KM972603;Lepidoptera;Lysandra coridon nufrellensis;Lysandra coridon;0.001;Lysandra coridon;0.874063524667043;
KM972605;Lepidoptera;Lysandra hispana;NA;NA;NA;0.957190060882237;
KM972606;Lepidoptera;Lysandra coridon;Lysandra coridon;NA;NA;0.995479627820194;
KM972607;Lepidoptera;Lysandra coridon;Lysandra coridon;NA;Lysandra coridon;0.801648170307457;
KM972609;Lepidoptera;Lysandra hispana;NA;NA;NA;0.997938263226617;
KM972611;Lepidoptera;Lysandra hispana;NA;NA;NA;0.930118546253334;
KM972612;Lepidoptera;Lysandra hispana;NA;NA;NA;0.909098462050209;
KM972615;Lepidoptera;Lysandra coridon;Lysandra coridon;NA;NA;0.953008903890644;
KM972617;Lepidoptera;Lysandra coridon;NA;NA;Lysandra coridon;0.811340269227995;
KM972618;Lepidoptera;Lysandra coridon;NA;NA;NA;0.966495575681232;
KM972632;Lepidoptera;Lysandra coridon gennargenti;NA;NA;Lysandra caelestissimus;0.746618874262524;
KP032261;Lepidoptera;Maniola nurag;NA;NA;NA;0.999999999969646;
KP032262;Lepidoptera;Maniola nurag;NA;NA;Maniola jurtina;0.91247810881561;
KP032263;Lepidoptera;Maniola nurag;NA;NA;NA;0.999999999969646;
KP032264;Lepidoptera;Maniola nurag;NA;NA;NA;0.999999999969646;
KP032265;Lepidoptera;Maniola nurag;Maniola nurag;1;Maniola nurag;0.944268434745869;
KP032266;Lepidoptera;Maniola nurag;Maniola nurag;0.999;Maniola nurag;0.944634067496311;
KP032268;Lepidoptera;Maniola nurag;Maniola nurag;1;Maniola nurag;0.975702243612751;
KP032280;Lepidoptera;Maniola nurag;NA;0.002;Maniola jurtina;0.930521714939346;
KP032281;Lepidoptera;Maniola nurag;NA;NA;Maniola nurag;0.735088611024579;
KP032282;Lepidoptera;Maniola nurag;NA;0.007;NA;0.999999999998573;
KP032283;Lepidoptera;Maniola nurag;NA;NA;Maniola nurag;0.984865455196247;
KP032284;Lepidoptera;Maniola nurag;Maniola nurag;0.995;Maniola nurag;0.998920097011014;
KP032285;Lepidoptera;Maniola nurag;NA;NA;Maniola nurag;0.963281872445319;
KP032287;Lepidoptera;Maniola nurag;NA;0.001;NA;0.999999999994698;
KP032288;Lepidoptera;Maniola nurag;NA;NA;Maniola nurag;0.963281872445319;
KP032298;Lepidoptera;Maniola jurtina;NA;0.003;NA;0.999999999997295;
KP032299;Lepidoptera;Maniola jurtina;NA;0.007;NA;0.999999999998573;
KP032300;Lepidoptera;Maniola jurtina;NA;0.014;NA;0.999999999999311;
KP032316;Lepidoptera;Maniola nurag;NA;0.002;Maniola jurtina;0.930521714939346;
KP032317;Lepidoptera;Maniola jurtina;NA;0.002;Maniola jurtina;0.930521714939346;
KP032346;Lepidoptera;Maniola cecilia;Pyronia cecilia;1;Pyronia cecilia;1;
KP032347;Lepidoptera;Maniola cecilia;Pyronia cecilia;1;Pyronia cecilia;1;
KP032352;Lepidoptera;Maniola nurag;NA;0.002;Maniola jurtina;0.930521714939346;
KP032353;Lepidoptera;Maniola nurag;Maniola nurag;0.999;Maniola nurag;0.944634067496311;
KP032355;Lepidoptera;Maniola nurag;Maniola nurag;1;Maniola nurag;0.975702243612751;
KP052710;Lepidoptera;Aricia agestis;NA;NA;Aricia agestis;0.993844765181105;
KP253125;Lepidoptera;Pyrgus malvoides;NA;0.176;Pyrgus malvoides;0.940490890379026;
KP253136;Lepidoptera;Polyommatus thersites;Polyommatus thersites;0.005;Polyommatus thersites;0.999999807789199;
KP253157;Lepidoptera;Erebia pharte;Erebia pharte;1;Erebia pharte;0.99999999630495;
KP253167;Lepidoptera;Erebia melampus;Erebia melampus;NA;Erebia melampus;0.950990028966024;
KP253180;Lepidoptera;Melitaea didyma;Melitaea didyma;0.001;Melitaea didyma;0.988955981346734;
KP253190;Lepidoptera;Erebia oeme;NA;0.114;Erebia oeme;0.999999999830635;
KP253200;Lepidoptera;Boloria pales;Boloria pales;0.998;Boloria pales;0.996520205418986;
KP253207;Lepidoptera;Pyrgus serratulae;Pyrgus serratulae;1;Pyrgus serratulae;0.99999989567508;
KP253223;Lepidoptera;Coenonympha gardetta;NA;0.003;Coenonympha gardetta;0.773402540734451;
KP253248;Lepidoptera;Lycaena tityrus;Lycaena tityrus;1;Lycaena tityrus;0.999998928238034;
KP253255;Lepidoptera;Minois dryas;NA;0.025;Minois dryas;0.850746026247397;
KP253256;Lepidoptera;Erebia epiphron;NA;NA;Erebia epiphron;0.994401488812176;
KP253261;Lepidoptera;Colias crocea;NA;0.059;NA;0.999999999999738;
KP253264;Lepidoptera;Colias alfacariensis;Colias alfacariensis;0.001;Colias alfacariensis;0.999926026317526;
KP253275;Lepidoptera;Agriades optilete;Agriades optilete;1;Agriades optilete;0.999999983195778;

KP253281;Lepidoptera;Erebia tyndarus;NA;NA;NA;0.999999999999923;
KP253294;Lepidoptera;Erebia pluto;Erebia pluto;1;Erebia pluto;0.999999977588772;
KP253307;Lepidoptera;Euphydryas intermedia;NA;0.121;NA;0.999999999995334;
KP253333;Lepidoptera;Carcharodus alceae;NA;NA;Carcharodus tripolinus;0.998261082574014;
KP253349;Lepidoptera;Erebia montana;Erebia montana;1;Erebia montana;0.999034068085633;
KP253354;Lepidoptera;Erebia oeme;NA;0.114;Erebia oeme;0.999999999830635;
KP253361;Lepidoptera;Erebia medusa;NA;0.018;Erebia medusa;0.988349516821371;
KP253373;Lepidoptera;Erebia manto;Erebia manto;0.999;Erebia manto;0.999976956795746;
KP253383;Lepidoptera;Colias phicomone;NA;0.001;Colias phicomone;0.985598879697681;
KP253384;Lepidoptera;Euphydryas cynthia;Euphydryas cynthia;1;Euphydryas cynthia;0.999999243242402;
KP253390;Lepidoptera;Erebia montana;Erebia montana;1;Erebia montana;0.99905080170195;
KP253393;Lepidoptera;Erebia euryale;NA;NA;Erebia euryale;0.919518760623792;
KP253411;Lepidoptera;Erynnis tages;Erynnis tages;1;Erynnis tages;0.999999281987018;
KP253465;Lepidoptera;Erebia pronoe;Erebia pronoe;1;Erebia pronoe;0.999567958198288;
KP253469;Lepidoptera;Colias alfacariensis;Colias alfacariensis;0.001;Colias alfacariensis;0.999926026317526;
KP253472;Lepidoptera;Erebia montana;Erebia montana;1;Erebia montana;0.99905080170195;
KP253476;Lepidoptera;Aricia eumedon;Eumedonia eumedon;0.007;Eumedonia eumedon;0.996933802230206;
KP253480;Lepidoptera;Lasiommata megera;Lasiommata megera;1;Lasiommata megera;0.999798408437094;
KP253487;Lepidoptera;Lysandra coridon;NA;NA;NA;0.997949615104622;
KP253494;Lepidoptera;Thymelicus sylvestris;Thymelicus sylvestris;1;Thymelicus sylvestris;0.999999981568351;
KP253515;Lepidoptera;Polyommatus thersites;Polyommatus thersites;0.005;Polyommatus thersites;0.999999807789199;
KP253531;Lepidoptera;Phengaris arion;NA;0.054;Phengaris arion;0.938397095316026;
KP253543;Lepidoptera;Pyrgus cacaliae;Pyrgus cacaliae;1;Pyrgus cacaliae;0.998243937509632;
KP253572;Lepidoptera;Satyrium w-album;NA;0.056;Satyrium w-album;0.989378230996316;
KP253586;Lepidoptera;Lycaena tityrus;Lycaena tityrus;1;Lycaena tityrus;0.999997027007808;
KP253591;Lepidoptera;Erebia meolans;NA;NA;Erebia meolans;0.999558897580846;
KP253597;Lepidoptera;Limenitis camilla;Limenitis camilla;0.009;Limenitis camilla;0.9999828626808;
KP253616;Lepidoptera;Erebia melampus;Erebia melampus;NA;Erebia melampus;0.950990028966024;
KP253641;Lepidoptera;Thecla betulae;Thecla betulae;1;Thecla betulae;0.999999999972886;
KP253700;Lepidoptera;Erebia eriphyle;Erebia eriphyle;1;Erebia eriphyle;0.999999983979336;
KP253710;Lepidoptera;Erebia pronoe;Erebia pronoe;1;Erebia pronoe;0.999567958198288;
KP253740;Lepidoptera;Hamearis lucina;NA;NA;Hamearis lucina;0.999999999984141;
KP253746;Lepidoptera;Oeneis glacialis;NA;NA;Oeneis glacialis;0.858599451249677;
KP689268;Lepidoptera;Euphydryas aurinia;NA;0.087;Euphydryas aurinia;0.771256060199022;
KP689269;Lepidoptera;Euphydryas aurinia;NA;0.087;Euphydryas aurinia;0.771256060199022;
KP689275;Lepidoptera;Euphydryas aurinia;NA;0.001;Euphydryas aurinia;0.827324190002093;
KP689276;Lepidoptera;Euphydryas aurinia;NA;0.001;Euphydryas aurinia;0.827324190002093;
KP689294;Lepidoptera;Euphydryas aurinia;NA;0.218;Euphydryas merope;0.770676462833303;
KP689295;Lepidoptera;Euphydryas aurinia;NA;0.218;Euphydryas merope;0.770676462833303;
KP689296;Lepidoptera;Euphydryas aurinia;NA;0.218;Euphydryas merope;0.770676462833303;
KP689297;Lepidoptera;Euphydryas aurinia;NA;0.218;Euphydryas merope;0.770676462833303;
KP851156;Lepidoptera;Iphiclides podalirius;NA;0.313;Iphiclides podalirius;0.889198863100098;
KP851157;Lepidoptera;Iphiclides podalirius;NA;0.313;Iphiclides podalirius;0.9030679504823;
KP851159;Lepidoptera;Iphiclides podalirius;NA;0.302;Iphiclides podalirius;0.931082412606632;
KP851160;Lepidoptera;Iphiclides podalirius;NA;0.302;Iphiclides podalirius;0.931082412606632;
KP851162;Lepidoptera;Iphiclides podalirius;NA;0.313;Iphiclides podalirius;0.889198863100098;
KP870212;Lepidoptera;Coenonympha dorus;Coenonympha dorus;1;Coenonympha dorus;0.999999999915957;
KP870213;Lepidoptera;Hamearis lucina;NA;NA;Hamearis lucina;0.999999999984141;
KP870214;Lepidoptera;Lycaena phlaeas;Lycaena phlaeas;1;Lycaena phlaeas;0.999999996363186;
KP870216;Lepidoptera;Coenonympha pamphilus;Coenonympha pamphilus;1;Coenonympha pamphilus;0.999999999999744;
KP870217;Lepidoptera;Araschnia levana;Araschnia levana;1;Araschnia levana;0.99999980307634;
KP870218;Lepidoptera;Thymelicus acteon;Thymelicus acteon;1;Thymelicus acteon;0.99999999752248;
KP870219;Lepidoptera;Pyrgus foulquieri;Pyrgus foulquieri;0.005;Pyrgus foulquieri;0.741885925757585;
KP870220;Lepidoptera;Carcharodus baeticus;Carcharodus baeticus;0.004;Carcharodus baeticus;0.999229183442549;
KP870221;Lepidoptera;Argynnis aglaja;NA;NA;Speyeria aglaja;0.889159762971514;
KP870223;Lepidoptera;Carcharodus lavatherae;Carcharodus lavatherae;1;Carcharodus lavatherae;0.999975293871709;
KP870225;Lepidoptera;Satyrium pruni;NA;0.076;Satyrium pruni;0.990322364021655;
KP870226;Lepidoptera;Carcharodus alceae;Carcharodus alceae;0.998;Carcharodus alceae;0.999999994860701;
KP870227;Lepidoptera;Thymelicus acteon;Thymelicus acteon;1;Thymelicus acteon;0.99999999752248;
KP870228;Lepidoptera;Lycaena phlaeas;Lycaena phlaeas;1;Lycaena phlaeas;0.999999996363186;
KP870231;Lepidoptera;Plebejus argus;Plebejus argus;0.001;Plebejus argus;0.995411334945632;
KP870232;Lepidoptera;Aricia eumedon;Eumedonia eumedon;0.034;Eumedonia eumedon;0.978796818650431;
KP870233;Lepidoptera;Scolitantides orion;Scolitantides orion;1;Scolitantides orion;0.999999953757596;
KP870234;Lepidoptera;Erebia meolans;Erebia meolans;NA;Erebia meolans;0.999771037232439;
KP870235;Lepidoptera;Papilio machaon;Papilio machaon;0.003;Papilio machaon;0.997288237409893;
KP870236;Lepidoptera;Araschnia levana;Araschnia levana;1;Araschnia levana;0.999999772187832;
KP870238;Lepidoptera;Brenthis daphne;Brenthis daphne;0.001;Brenthis daphne;0.996233668169668;

KP870240;Lepidoptera;Gegenes pumilio;Gegenes pumilio;1;Gegenes pumilio;0.999986726679266;
KP870241;Lepidoptera;Boloria euphrosyne;Boloria euphrosyne;0.001;Boloria euphrosyne;0.999975174156152;
KP870242;Lepidoptera;Argynnis aglaja;NA;0.003;Speyeria aglaja;0.829040804758583;
KP870245;Lepidoptera;Thymelicus sylvestris;Thymelicus sylvestris;1;Thymelicus sylvestris;0.999999985935205;
KP870247;Lepidoptera;Carcharodus lavatherae;Carcharodus lavatherae;1;Carcharodus lavatherae;0.999938147857335;
KP870248;Lepidoptera;Maniola tithonus;Pyronia tithonus;NA;Pyronia tithonus;1;
KP870249;Lepidoptera;Araschnia levana;Araschnia levana;1;Araschnia levana;0.999999751605545;
KP870251;Lepidoptera;Carcharodus lavatherae;Carcharodus lavatherae;1;Carcharodus lavatherae;0.999984716552214;
KP870252;Lepidoptera;Lycaena phlaeas;Lycaena phlaeas;1;Lycaena phlaeas;0.999999996363186;
KP870253;Lepidoptera;Gonepteryx rhamni;NA;0.194;Gonepteryx rhamni;0.985761968681459;
KP870254;Lepidoptera;Thymelicus sylvestris;NA;0.324;Thymelicus sylvestris;0.999940778442454;
KP870255;Lepidoptera;Anthocharis cardamines;Anthocharis cardamines;1;Anthocharis cardamines;0.999999988995796;
KP870257;Lepidoptera;Pyrgus alveus;NA;NA;Pyrgus alveus;0.931326683565012;
KP870258;Lepidoptera;Hipparchia fagi;Hipparchia fagi;NA;Hipparchia fagi;0.986566456150817;
KP870259;Lepidoptera;Callophrys rubi;NA;0.014;Callophrys rubi;0.998620746648187;
KP870260;Lepidoptera;Boloria pales;Boloria pales;1;Boloria pales;0.998536542463107;
KP870262;Lepidoptera;Brenthis daphne;Brenthis daphne;1;Brenthis daphne;0.997172799742872;
KP870263;Lepidoptera;Fabriciana niobe;NA;0.012;Fabriciana niobe;0.984414140592274;
KP870264;Lepidoptera;Lampides boeticus;Lampides boeticus;1;Lampides boeticus;0.999999999545906;
KP870265;Lepidoptera;Hipparchia statilinus;Hipparchia statilinus;1;Hipparchia statilinus;0.999999999999005;
KP870266;Lepidoptera;Fabriciana niobe;NA;0.007;Fabriciana niobe;0.984280831332444;
KP870267;Lepidoptera;Pyrgus alveus;NA;0.002;Pyrgus alveus;0.952935075824246;
KP870268;Lepidoptera;Cupido osiris;Cupido osiris;1;Cupido osiris;0.999999170120762;
KP870269;Lepidoptera;Araschnia levana;Araschnia levana;1;Araschnia levana;0.999999774674759;
KP870270;Lepidoptera;Celastrina argiolus;Celastrina argiolus;1;Celastrina argiolus;0.999527385150531;
KP870271;Lepidoptera;Satyrium spini;Satyrium spini;1;Satyrium spini;0.999997733330997;
KP870273;Lepidoptera;Papilio machaon;Papilio machaon;0.003;Papilio machaon;0.997288237409893;
KP870274;Lepidoptera;Lycaena virgaureae;NA;0.184;Lycaena virgaureae;0.999653824584039;
KP870275;Lepidoptera;Satyrium spini;Satyrium spini;1;Satyrium spini;0.999987432167351;
KP870277;Lepidoptera;Hipparchia hermione;Hipparchia hermione;1;Hipparchia hermione;0.999958720745028;
KP870282;Lepidoptera;Charaxes jasius;Charaxes jasius;NA;Charaxes jasius;0.990397821479112;
KP870283;Lepidoptera;Pyrgus cacaliae;Pyrgus cacaliae;0.999;Pyrgus cacaliae;0.997229839348598;
KP870284;Lepidoptera;Colias phicomone;Colias phicomone;0.001;Colias phicomone;0.986802434025645;
KP870286;Lepidoptera;Pieris napi;NA;0.028;Pieris napi;0.888205720664699;
KP870288;Lepidoptera;Gonepteryx cleopatra;Gonepteryx cleopatra;0.998;Gonepteryx cleopatra;0.999118636582266;
KP870290;Lepidoptera;Coenonympha arcania;NA;0.153;Coenonympha arcania;0.966428236827255;
KP870291;Lepidoptera;Aricia eumedon;Eumedonia eumedon;0.049;Eumedonia eumedon;0.980035355423569;
KP870293;Lepidoptera;Minois dryas;NA;0.025;Minois dryas;0.850746026247397;
KP870295;Lepidoptera;Aglais urticae;NA;0.002;Aglais urticae;0.987497593219019;
KP870297;Lepidoptera;Thymelicus lineola;Thymelicus lineola;1;Thymelicus lineola;0.999995927238524;
KP870298;Lepidoptera;Colias alfacariensis;Colias alfacariensis;0.001;Colias alfacariensis;0.999857790810452;
KP870299;Lepidoptera;Apatura iris;Apatura iris;1;Apatura iris;0.999999999999972;
KP870304;Lepidoptera;Lycaena phlaeas;Lycaena phlaeas;1;Lycaena phlaeas;0.999999996363186;
KP870306;Lepidoptera;Cyaniris semiargus;NA;NA;Cyaniris semiargus;0.999065557065321;
KP870307;Lepidoptera;Aphantopus hyperantus;Aphantopus hyperantus;0.003;Aphantopus hyperantus;0.999999455871523;
KP870310;Lepidoptera;Polyommatus amandus;Polyommatus amandus;0.003;Polyommatus amandus;0.999951656678735;
KP870311;Lepidoptera;Vanessa atalanta;Vanessa atalanta;1;Vanessa atalanta;0.999999999757762;
KP870312;Lepidoptera;Issoria lathonia;Issoria lathonia;1;Issoria lathonia;0.999999999989257;
KP870313;Lepidoptera;Satyrium acaciae;Satyrium acaciae;1;Satyrium acaciae;0.99999991343697;
KP870315;Lepidoptera;Cupido minimus;NA;0.006;Cupido minimus;0.999768371759258;
KP870316;Lepidoptera;Lasiommata maera;Lasiommata maera;1;Lasiommata maera;0.999998722471466;
KP870317;Lepidoptera;Minois dryas;NA;0.025;Minois dryas;0.850746026247397;
KP870318;Lepidoptera;Lasiommata maera;Lasiommata maera;1;Lasiommata maera;0.9999936429582;
KP870319;Lepidoptera;Polyommatus thersites;Polyommatus thersites;0.999;Polyommatus thersites;0.999999916395897;
KP870320;Lepidoptera;Thecla betulae;Thecla betulae;1;Thecla betulae;0.999999999972886;
KP870324;Lepidoptera;Pyrgus foulquieri;Pyrgus foulquieri;0.005;Pyrgus foulquieri;0.741885925757585;
KP870325;Lepidoptera;Cupido osiris;Cupido osiris;1;Cupido osiris;0.999999238096456;
KP870326;Lepidoptera;Thymelicus acteon;Thymelicus acteon;1;Thymelicus acteon;0.99999999752248;
KP870327;Lepidoptera;Satyrus ferula;Satyrus ferula;0.001;Satyrus ferula;0.993138467028266;
KP870328;Lepidoptera;Coenonympha pamphilus;Coenonympha pamphilus;1;Coenonympha pamphilus;0.999999999999744;
KP870329;Lepidoptera;Satyrium ilicis;Satyrium ilicis;1;Satyrium ilicis;0.999999987901305;
KP870330;Lepidoptera;Brenthis ino;Brenthis ino;0.002;Brenthis ino;0.984749809551735;

KP870331;Lepidoptera;Pyrgus alveus;NA;NA;Pyrgus alveus;0.931326683565012;
KP870334;Lepidoptera;Cupido minimus;NA;0.009;Cupido minimus;0.999791453712643;
KP870337;Lepidoptera;Apatura iris;Apatura iris;1;Apatura iris;0.999999999999972;
KP870339;Lepidoptera;Thymelicus lineola;Thymelicus lineola;1;Thymelicus lineola;0.999994567418986;
KP870340;Lepidoptera;Minois dryas;NA;0.025;Minois dryas;0.850746026247397;
KP870341;Lepidoptera;Erebia pandrose;Erebia pandrose;0.003;Erebia pandrose;0.993608645577247;
KP870342;Lepidoptera;Glaucopsyche alexis;NA;NA;Glaucopsyche alexis;0.991019528246159;
KP870344;Lepidoptera;Plebejus idas;NA;0.001;NA;0.999938863896795;
KP870345;Lepidoptera;Pyrgus foulquieri;Pyrgus foulquieri;0.005;Pyrgus foulquieri;0.741885925757585;
KP870349;Lepidoptera;Coenonympha pamphilus;Coenonympha pamphilus;1;Coenonympha pamphilus;0.999999999999744;
KP870350;Lepidoptera;Gegenes pumilio;Gegenes pumilio;1;Gegenes pumilio;0.999986726679266;
KP870352;Lepidoptera;Arethusana arethusa;Arethusana arethusa;0.045;Arethusana arethusa;0.999962297609568;
KP870353;Lepidoptera;Maniola cecilia;Pyronia cecilia;1;Pyronia cecilia;1;
KP870354;Lepidoptera;Chazara briseis;Chazara briseis;0.001;Chazara briseis;0.999962563201771;
KP870355;Lepidoptera;Boloria pales;Boloria pales;1;Boloria pales;0.998536542463107;
KP870356;Lepidoptera;Brenthis ino;Brenthis ino;0.998;Brenthis ino;0.995325118916732;
KP870357;Lepidoptera;Brenthis daphne;Brenthis daphne;0.001;Brenthis daphne;0.996649664069688;
KP870358;Lepidoptera;Thymelicus sylvestris;Thymelicus sylvestris;1;Thymelicus sylvestris;0.999999985935205;
KP870359;Lepidoptera;Satyrium w-album;NA;0.185;Satyrium w-album;0.994665799693619;
KP870360;Lepidoptera;Hipparchia hermione;Hipparchia hermione;1;Hipparchia hermione;0.999958624563657;
KP870361;Lepidoptera;Gegenes nostrodamus;Gegenes nostrodamus;1;Gegenes nostrodamus;0.999999996883645;
KP870363;Lepidoptera;Satyrium acaciae;Satyrium acaciae;1;Satyrium acaciae;0.99999991343697;
KP870364;Lepidoptera;Satyrium acaciae;Satyrium acaciae;1;Satyrium acaciae;0.999999915972158;
KP870366;Lepidoptera;Scolitantides orion;Scolitantides orion;1;Scolitantides orion;0.999999953757596;
KP870368;Lepidoptera;Minois dryas;NA;0.025;Minois dryas;0.850746026247397;
KP870371;Lepidoptera;Hipparchia hermione;NA;NA;NA;0.999999999999991;
KP870373;Lepidoptera;Arethusana arethusa;Arethusana arethusa;0.023;Arethusana arethusa;0.999940049962276;
KP870375;Lepidoptera;Brenthis ino;Brenthis ino;0.007;Brenthis ino;0.977157381961834;
KP870376;Lepidoptera;Limenitis camilla;Limenitis camilla;0.003;Limenitis camilla;0.999815165838915;
KP870377;Lepidoptera;Parnassius apollo;Parnassius apollo;1;Parnassius apollo;0.99641937826143;
KP870379;Lepidoptera;Polyommatus daphnis;NA;0.06;Polyommatus daphnis;0.994947425490863;
KP870380;Lepidoptera;Arethusana arethusa;Arethusana arethusa;1;Arethusana arethusa;0.999985516708968;
KP870381;Lepidoptera;Coenonympha gardetta;NA;0.039;Coenonympha gardetta;0.768574698743531;
KP870384;Lepidoptera;Satyrium spini;Satyrium spini;1;Satyrium spini;0.999998221707433;
KP870385;Lepidoptera;Argynnis aglaja;NA;NA;NA;0.999999999998776;
KP870386;Lepidoptera;Gegenes nostrodamus;Gegenes nostrodamus;1;Gegenes nostrodamus;0.999999996883645;
KP870387;Lepidoptera;Coenonympha arcania;NA;0.179;Coenonympha arcania;0.96703617676693;
KP870388;Lepidoptera;Arethusana arethusa;NA;0.062;Arethusana arethusa;0.999962489703143;
KP870389;Lepidoptera;Argynnis aglaja;NA;0.005;NA;0.999999999994961;
KP870392;Lepidoptera;Satyrium esculi;Satyrium esculi;1;Satyrium esculi;0.999999957367948;
KP870393;Lepidoptera;Argynnis aglaja;NA;NA;NA;0.999999999998776;
KP870394;Lepidoptera;Callophrys rubi;NA;0.002;Callophrys rubi;0.999056774120503;
KP870396;Lepidoptera;Satyrium spini;Satyrium spini;1;Satyrium spini;0.999997778937137;
KP870397;Lepidoptera;Pyrgus alveus;NA;NA;Pyrgus alveus;0.931326683565012;
KP870398;Lepidoptera;Maniola cecilia;Pyronia cecilia;1;Pyronia cecilia;1;
KP870399;Lepidoptera;Pyrgus andromedae;Pyrgus andromedae;1;Pyrgus andromedae;0.997371767954981;
KP870400;Lepidoptera;Boloria euphrosyne;Boloria euphrosyne;1;Boloria euphrosyne;0.999987528582416;
KP870401;Lepidoptera;Lysandra coridon;NA;NA;NA;0.998202549747544;
KP870404;Lepidoptera;Polyommatus thersites;Polyommatus thersites;0.999;Polyommatus thersites;0.99999985539095;
KP870405;Lepidoptera;Pyrgus foulquieri;Pyrgus foulquieri;0.005;Pyrgus foulquieri;0.741885925757585;
KP870406;Lepidoptera;Carterocephalus palaemon;NA;0.05;Carterocephalus palaemon;0.720518487271686;
KP870407;Lepidoptera;Erebia manto;Erebia manto;0.003;Erebia manto;0.999947286129868;
KP870408;Lepidoptera;Brenthis ino;Brenthis ino;0.001;Brenthis ino;0.998434780405276;
KP870409;Lepidoptera;Nymphalis c-album;NA;0.215;NA;0.999999192918557;
KP870411;Lepidoptera;Polyommatus daphnis;Polyommatus daphnis;0.007;Polyommatus daphnis;0.995698888179315;
KP870414;Lepidoptera;Carcharodus alceae;Carcharodus alceae;0.999;Carcharodus alceae;0.999999996339511;
KP870417;Lepidoptera;Aglais urticae;Aglais urticae;0.001;Aglais urticae;0.994396335177485;
KP870419;Lepidoptera;Aglais urticae;Aglais urticae;0.999;Aglais urticae;0.996396169943103;
KP870420;Lepidoptera;Minois dryas;NA;0.025;Minois dryas;0.850746026247397;
KP870421;Lepidoptera;Lampides boeticus;Lampides boeticus;1;Lampides boeticus;0.99999999876789;

KP870422;Lepidoptera;Thecla betulae;Thecla betulae;1;Thecla betulae;0.999999999972886;
KP870423;Lepidoptera;Arethusana arethusa;Arethusana arethusa;1;Arethusana arethusa;0.999999787144355;
KP870424;Lepidoptera;Aglais urticae;NA;0.002;Aglais urticae;0.987497593219019;
KP870428;Lepidoptera;Fabriciana niobe;NA;0.007;Fabriciana niobe;0.984627988609443;
KP870429;Lepidoptera;Hipparchia fagi;Hipparchia fagi;NA;Hipparchia fagi;0.986566456150817;
KP870430;Lepidoptera;Coenonympha pamphilus;Coenonympha pamphilus;1;Coenonympha pamphilus;0.999999999999744;
KP870433;Lepidoptera;Polyommatus amandus;Polyommatus amandus;0.003;Polyommatus amandus;0.999951656678735;
KP870435;Lepidoptera;Boloria pales;Boloria pales;1;Boloria pales;0.998536542463107;
KP870436;Lepidoptera;Hipparchia hermione;Hipparchia hermione;1;Hipparchia hermione;0.999912393907121;
KP870441;Lepidoptera;Pieris napi;NA;0.028;Pieris napi;0.888205720664699;
KP870442;Lepidoptera;Cupido argiades;NA;0.49;Cupido argiades;0.727122840051529;
KP870443;Lepidoptera;Minois dryas;NA;0.025;Minois dryas;0.850746026247397;
KP870444;Lepidoptera;Hamearis lucina;NA;NA;Hamearis lucina;0.999999999984141;
KP870445;Lepidoptera;Erebia epiphron;NA;NA;Erebia epiphron;0.994401488812176;
KP870446;Lepidoptera;Cupido minimus;NA;0.009;Cupido minimus;0.999791453712643;
KP870447;Lepidoptera;Maniola cecilia;Pyronia cecilia;1;Pyronia cecilia;1;
KP870449;Lepidoptera;Pieris napi;NA;0.028;Pieris napi;0.888205720664699;
KP870451;Lepidoptera;Thymelicus acteon;Thymelicus acteon;1;Thymelicus acteon;0.99999999752248;
KP870453;Lepidoptera;Lysandra hispana;NA;NA;Lysandra hispana;0.906698563865667;
KP870455;Lepidoptera;Scolitantides orion;Scolitantides orion;1;Scolitantides orion;0.999999953757596;
KP870459;Lepidoptera;Pyrgus foulquieri;Pyrgus foulquieri;0.005;Pyrgus foulquieri;0.741885925757585;
KP870460;Lepidoptera;Pyrgus cacaliae;Pyrgus cacaliae;0.999;Pyrgus cacaliae;0.997229839348598;
KP870461;Lepidoptera;Lasiommata megera;Lasiommata megera;0.995;Lasiommata megera;0.999279716854958;
KP870462;Lepidoptera;Coenonympha arcania;Coenonympha arcania;0.002;NA;0.999999999999943;
KP870464;Lepidoptera;Thymelicus sylvestris;Thymelicus sylvestris;1;Thymelicus sylvestris;0.999999974418415;
KP870465;Lepidoptera;Cupido osiris;Cupido osiris;1;Cupido osiris;0.999997440696525;
KP870466;Lepidoptera;Boloria dia;Boloria dia;1;Boloria dia;0.999999999979764;
KP870469;Lepidoptera;Vanessa cardui;Vanessa cardui;1;Vanessa cardui;0.999999459983318;
KP870470;Lepidoptera;Thymelicus sylvestris;Thymelicus sylvestris;1;Thymelicus sylvestris;0.999999993098527;
KP870471;Lepidoptera;Thymelicus acteon;Thymelicus acteon;1;Thymelicus acteon;0.999999962833841;
KP870472;Lepidoptera;Pyrgus foulquieri;Pyrgus foulquieri;0.005;Pyrgus foulquieri;0.741885925757585;
KP870473;Lepidoptera;Colias crocea;NA;0.059;NA;0.999999999999738;
KP870476;Lepidoptera;Cupido osiris;Cupido osiris;1;Cupido osiris;0.999999238096456;
KP870477;Lepidoptera;Melitaea diamina;NA;0.149;Melitaea diamina;0.999999996592464;
KP870478;Lepidoptera;Apatura ilia;NA;0.165;Apatura ilia;0.956796812031481;
KP870480;Lepidoptera;Colias crocea;NA;NA;NA;0.999999999999005;
KP870482;Lepidoptera;Colias crocea;NA;0.059;NA;0.999999999999738;
KP870483;Lepidoptera;Ochlodes sylvanus;NA;0.054;Ochlodes sylvanus;0.997764762465093;
KP870484;Lepidoptera;Pyrgus cacaliae;Pyrgus cacaliae;1;Pyrgus cacaliae;0.996034079665744;
KP870485;Lepidoptera;Hipparchia semele;NA;0.001;Hipparchia semele;0.831933308520053;
KP870486;Lepidoptera;Satyrium esculi;Satyrium esculi;1;Satyrium esculi;0.999999973565906;
KP870488;Lepidoptera;Satyrium ilicis;Satyrium ilicis;1;Satyrium ilicis;0.999999963901758;
KP870490;Lepidoptera;Brintesia circe;Brintesia circe;1;Brintesia circe;0.99999951254154;
KP870491;Lepidoptera;Lasiommata megera;Lasiommata megera;1;Lasiommata megera;0.999770424843767;
KP870492;Lepidoptera;Lasiommata megera;Lasiommata megera;0.013;Lasiommata megera;0.999252637809699;
KP870495;Lepidoptera;Limenitis camilla;Limenitis camilla;0.009;Limenitis camilla;0.9999828626808;
KP870496;Lepidoptera;Satyrium w-album;NA;0.056;Satyrium w-album;0.989378230996316;
KP870497;Lepidoptera;Aglais urticae;Aglais urticae;0.001;Aglais urticae;0.990690600684087;
KP870498;Lepidoptera;Pararge aegeria;Pararge aegeria;1;Pararge aegeria;0.999999997297948;
KP870500;Lepidoptera;Hipparchia semele;NA;0.001;Hipparchia semele;0.824269221281445;
KP870503;Lepidoptera;Thecla betulae;Thecla betulae;1;Thecla betulae;0.999999999972886;
KP870504;Lepidoptera;Brenthis ino;NA;0.054;Brenthis ino;0.812147075315208;
KP870505;Lepidoptera;Hamearis lucina;Hamearis lucina;1;Hamearis lucina;0.999999999986215;
KP870506;Lepidoptera;Melitaea cinxia;Melitaea cinxia;1;Melitaea cinxia;0.999999999999659;
KP870507;Lepidoptera;Gegenes nostrodamus;Gegenes nostrodamus;1;Gegenes nostrodamus;0.999999996883645;
KP870509;Lepidoptera;Thymelicus lineola;Thymelicus lineola;1;Thymelicus lineola;0.999997716763366;
KP870510;Lepidoptera;Brenthis ino;Brenthis ino;0.004;Brenthis ino;0.977399410491198;
KP870511;Lepidoptera;Helleia helle;Lycaena helle;1;Lycaena helle;0.999999995066105;
KP870512;Lepidoptera;Thymelicus acteon;Thymelicus acteon;1;Thymelicus acteon;0.999999899039215;
KP870516;Lepidoptera;Plebejus argus;NA;NA;Plebejus argus;0.969283117748927;
KP870518;Lepidoptera;Aphantopus hyperantus;Aphantopus hyperantus;0.009;Aphantopus hyperantus;0.999523934516809;
KP870519;Lepidoptera;Chazara briseis;Chazara briseis;0.001;Chazara briseis;0.999991285807394;

KP870520;Lepidoptera;Hipparchia hermione;Hipparchia hermione;NA;Hipparchia hermione;0.99478921057068;
KP870521;Lepidoptera;Pyrgus alveus;NA;NA;Pyrgus alveus;0.944546694610372;
KP870522;Lepidoptera;Melitaea britomartis;Mellicta britomartis;NA;Melitaea britomartis;0.93596070790707;
KP870524;Lepidoptera;Cupido minimus;NA;0.001;Cupido minimus;0.999751488575214;
KP870525;Lepidoptera;Gegenes nostrodamus;Gegenes nostrodamus;1;Gegenes nostrodamus;0.999999996883645;
KP870527;Lepidoptera;Erynnis tages;Erynnis tages;1;Erynnis tages;0.999999281987018;
KP870529;Lepidoptera;Pyrgus armoricanus;Pyrgus armoricanus;1;Pyrgus armoricanus;0.999997353848817;
KP870530;Lepidoptera;Boloria pales;Boloria pales;0.998;Boloria pales;0.996520205418986;
KP870531;Lepidoptera;Pyrgus cacaliae;Pyrgus cacaliae;0.999;Pyrgus cacaliae;0.997229839348598;
KP870532;Lepidoptera;Pyrgus malvoides;NA;0.076;Pyrgus malvoides;0.887854315566122;
KP870533;Lepidoptera;Boloria euphrosyne;Boloria euphrosyne;1;Boloria euphrosyne;0.999998102693392;
KP870535;Lepidoptera;Hipparchia semele;NA;0.002;Hipparchia semele;0.767063813608107;
KP870536;Lepidoptera;Lycaena phlaeas;Lycaena phlaeas;1;Lycaena phlaeas;0.999999990751576;
KP870537;Lepidoptera;Argynnis aglaja;NA;0.001;NA;0.999999999999586;
KP870538;Lepidoptera;Pyrgus andromedae;Pyrgus andromedae;1;Pyrgus andromedae;0.997371767954981;
KP870540;Lepidoptera;Vanessa atalanta;Vanessa atalanta;1;Vanessa atalanta;0.999999999790589;
KP870541;Lepidoptera;Papilio machaon;Papilio machaon;0.003;Papilio machaon;0.99836194854697;
KP870542;Lepidoptera;Pyrgus foulquieri;Pyrgus foulquieri;0.005;Pyrgus foulquieri;0.741885925757585;
KP870543;Lepidoptera;Colias crocea;NA;0.059;NA;0.999999999999738;
KP870544;Lepidoptera;Argynnis aglaja;NA;NA;Speyeria aglaja;0.879701754260687;
KP870547;Lepidoptera;Satyrium w-album;NA;0.056;Satyrium w-album;0.989378230996316;
KP870550;Lepidoptera;Coenonympha dorus;Coenonympha dorus;1;Coenonympha dorus;0.999999999517769;
KP870551;Lepidoptera;Apatura iris;Apatura iris;1;Apatura iris;0.999999999999972;
KP870552;Lepidoptera;Cercyonis lupina;Hyponephele lupina;1;Hyponephele lupina;0.999999983109973;
KP870554;Lepidoptera;Thymelicus lineola;Thymelicus lineola;1;Thymelicus lineola;0.999995927238524;
KP870556;Lepidoptera;Cupido minimus;NA;0.216;Cupido lorquinii;0.726278995315528;
KP870557;Lepidoptera;Lycaena hippothoe;NA;NA;Lycaena hippothoe;0.929268253497542;
KP870558;Lepidoptera;Boloria dia;Boloria dia;1;Boloria dia;0.999999999979764;
KP870559;Lepidoptera;Coenonympha dorus;Coenonympha dorus;1;Coenonympha dorus;0.99999999988259;
KP870560;Lepidoptera;Aglais urticae;Aglais urticae;0.004;Aglais urticae;0.995016750255472;
KP870561;Lepidoptera;Thecla betulae;Thecla betulae;1;Thecla betulae;0.999999999972886;
KP870562;Lepidoptera;Pararge aegeria;Pararge aegeria;1;Pararge aegeria;0.999999999963165;
KP870563;Lepidoptera;Brenthis ino;Brenthis ino;0.001;Brenthis ino;0.998434780405276;
KP870564;Lepidoptera;Cupido minimus;NA;0.009;Cupido minimus;0.999791453712643;
KP870565;Lepidoptera;Thymelicus lineola;Thymelicus lineola;1;Thymelicus lineola;0.999883815000502;
KP870566;Lepidoptera;Lasiommata maera;Lasiommata maera;1;Lasiommata maera;0.99999385225814;
KP870567;Lepidoptera;Lycaena phlaeas;Lycaena phlaeas;1;Lycaena phlaeas;0.999999996363186;
KP870568;Lepidoptera;Lasiommata maera;Lasiommata maera;1;Lasiommata maera;0.999996744651964;
KP870569;Lepidoptera;Cupido minimus;NA;0.006;Cupido minimus;0.999768371759258;
KP870570;Lepidoptera;Callophrys rubi;Callophrys rubi;0.001;Callophrys rubi;0.998606378892944;
KP870571;Lepidoptera;Heteropterus morpheus;NA;NA;Heteropterus morpheus;0.999999999999943;
KP870573;Lepidoptera;Thymelicus sylvestris;Thymelicus sylvestris;1;Thymelicus sylvestris;0.999999985350172;
KP870574;Lepidoptera;Papilio machaon;Papilio machaon;0.001;Papilio machaon;0.999463129150422;
KP870576;Lepidoptera;Boloria pales;Boloria pales;1;Boloria pales;0.998536542463107;
KP870577;Lepidoptera;Erebia epiphron;Erebia epiphron;0.996;Erebia epiphron;0.998796590633588;
KP870578;Lepidoptera;Hipparchia semele;NA;0.001;Hipparchia semele;0.802446092830354;
KP870580;Lepidoptera;Erebia epiphron;Erebia epiphron;NA;Erebia epiphron;0.998837748029833;
KP870581;Lepidoptera;Carcharodus alceae;Carcharodus alceae;0.999;Carcharodus alceae;0.999999996339511;
KP870583;Lepidoptera;Satyrium ilicis;Satyrium ilicis;1;Satyrium ilicis;0.999999963901758;
KP870584;Lepidoptera;Papilio machaon;Papilio machaon;0.003;Papilio machaon;0.997288237409893;
KP870586;Lepidoptera;Erebia manto;Erebia manto;NA;Erebia manto;0.999926924199631;
KP870587;Lepidoptera;Erebia epiphron;Erebia epiphron;0.996;Erebia epiphron;0.998796590633588;
KP870591;Lepidoptera;Gonepteryx rhamni;NA;0.474;Gonepteryx rhamni;0.982076396127858;
KP870592;Lepidoptera;Lycaena alciphron;Lycaena alciphron;1;Lycaena alciphron;0.999999842864254;
KP870593;Lepidoptera;Melitaea didyma;Melitaea didyma;1;Melitaea didyma;0.999515490426369;
KP870594;Lepidoptera;Hyponephele lycaon;Hyponephele lycaon;1;Hyponephele lycaon;0.99999999927789;
KP870595;Lepidoptera;Pyrgus armoricanus;Pyrgus armoricanus;1;Pyrgus armoricanus;0.999997353848817;
KP870596;Lepidoptera;Coenonympha rhodopensis;Coenonympha rhodopensis;0.002;Coenonympha rhodopensis;0.972179742721853;
KP870598;Lepidoptera;Hipparchia semele;NA;0.002;Hipparchia semele;0.767063813608107;
KP870600;Lepidoptera;Satyrium ilicis;Satyrium ilicis;1;Satyrium ilicis;0.999999989733169;
KP870601;Lepidoptera;Thecla betulae;Thecla betulae;1;Thecla betulae;0.999999999972886;
KP870602;Lepidoptera;Nymphalis c-album;NA;0.215;NA;0.999999192918557;
KP870603;Lepidoptera;Carcharodus baeticus;Carcharodus baeticus;1;Carcharodus baeticus;0.999983899664537;

KP870604;Lepidoptera;Thymelicus sylvestris;Thymelicus sylvestris;1;Thymelicus sylvestris;0.999999985935205;
KP870605;Lepidoptera;Brintesia circe;Brintesia circe;1;Brintesia circe;0.999999659617999;
KP870606;Lepidoptera;Cupido minimus;NA;0.001;NA;0.999999109483477;
KP870607;Lepidoptera;Thymelicus lineola;Thymelicus lineola;1;Thymelicus lineola;0.999999168558962;
KP870608;Lepidoptera;Fabriciana adippe;NA;NA;Fabriciana adippe;0.900255826442102;
KP870610;Lepidoptera;Pyrgus cacaliae;Pyrgus cacaliae;0.999;Pyrgus cacaliae;0.997366941689678;
KP870611;Lepidoptera;Heteropterus morpheus;NA;NA;Heteropterus morpheus;0.999999999999943;
KP870613;Lepidoptera;Pyrgus carthami;Pyrgus carthami;1;Pyrgus carthami;0.999990719819382;
KP870614;Lepidoptera;Maniola cecilia;Pyronia cecilia;1;Pyronia cecilia;1;
KP870615;Lepidoptera;Pyrgus alveus;NA;NA;Pyrgus alveus;0.941697735568124;
KP870616;Lepidoptera;Erebia epiphron;Erebia epiphron;NA;Erebia epiphron;0.998837748029833;
KP870617;Lepidoptera;Brenthis hecate;NA;0.068;Brenthis hecate;0.999053551498733;
KP870618;Lepidoptera;Pyrgus alveus;NA;NA;Pyrgus alveus;0.957305240793981;
KP870619;Lepidoptera;Apatura iris;Apatura iris;1;Apatura iris;0.999999999999972;
KP870620;Lepidoptera;Thymelicus acteon;Thymelicus acteon;1;Thymelicus acteon;0.99999999752248;
KP870621;Lepidoptera;Arethusana arethusa;Arethusana arethusa;0.03;Arethusana arethusa;0.999965341832138;
KP870622;Lepidoptera;Hipparchia statilinus;Hipparchia statilinus;1;Hipparchia statilinus;0.999999999399932;
KP870623;Lepidoptera;Scolitantides orion;Scolitantides orion;1;Scolitantides orion;0.999999893797684;
KP870624;Lepidoptera;Satyrium spini;Satyrium spini;1;Satyrium spini;0.999997666151069;
KP870625;Lepidoptera;Erebia epiphron;Erebia epiphron;NA;Erebia epiphron;0.998837748029833;
KP870626;Lepidoptera;Pyrgus alveus;NA;NA;Pyrgus alveus;0.927745497730628;
KP870628;Lepidoptera;Coenonympha pamphilus;Coenonympha pamphilus;1;Coenonympha pamphilus;0.999999999992554;
KP870629;Lepidoptera;Hipparchia semele;NA;0.001;Hipparchia semele;0.802446092830354;
KP870630;Lepidoptera;Thymelicus lineola;Thymelicus lineola;1;Thymelicus lineola;0.999986269120677;
KP870631;Lepidoptera;Pyrgus alveus;NA;NA;Pyrgus alveus;0.931326683565012;
KP870632;Lepidoptera;Aphantopus hyperantus;Aphantopus hyperantus;0.003;Aphantopus hyperantus;0.999999455871523;
KP870633;Lepidoptera;Thymelicus sylvestris;Thymelicus sylvestris;1;Thymelicus sylvestris;0.999999985935205;
KP870634;Lepidoptera;Pyrgus alveus;NA;NA;Pyrgus alveus;0.931326683565012;
KP870635;Lepidoptera;Thymelicus acteon;Thymelicus acteon;1;Thymelicus acteon;0.999999995080373;
KP870636;Lepidoptera;Aricia artaxerxes;Plebejus argus;1;Aricia artaxerxes;0.999743187199922;
KP870637;Lepidoptera;Aglais urticae;NA;0.002;Aglais urticae;0.987497593219019;
KP870640;Lepidoptera;Coenonympha arcania;Coenonympha arcania;0.018;Coenonympha arcania;0.986973789478505;
KP870641;Lepidoptera;Apatura ilia;NA;0.165;Apatura ilia;0.956796812031481;
KP870642;Lepidoptera;Carterocephalus palaemon;NA;0.052;Carterocephalus palaemon;0.728445051795325;
KP870643;Lepidoptera;Thymelicus acteon;Thymelicus acteon;1;Thymelicus acteon;0.99999999752248;
KP870644;Lepidoptera;Lasiommata megera;Lasiommata megera;1;Lasiommata megera;0.999928528239676;
KP870647;Lepidoptera;Carcharodus lavatherae;Carcharodus lavatherae;1;Carcharodus lavatherae;0.999958116204794;
KP870649;Lepidoptera;Hipparchia hermione;Hipparchia hermione;1;Hipparchia hermione;0.999977229560459;
KP870650;Lepidoptera;Erebia pronoe;Erebia pronoe;1;Erebia pronoe;0.997847273843868;
KP870652;Lepidoptera;Erebia euryale;NA;NA;Erebia euryale;0.971435689232499;
KP870656;Lepidoptera;Cercyonis lupina;Hyponephele lupina;1;Hyponephele lupina;0.999999981841427;
KP870657;Lepidoptera;Aglais urticae;NA;0.002;Aglais urticae;0.987497593219019;
KP870659;Lepidoptera;Melitaea didyma;Melitaea didyma;1;Melitaea didyma;0.999727166931204;
KP870660;Lepidoptera;Lycaena phlaeas;Lycaena phlaeas;1;Lycaena phlaeas;0.999999996363186;
KP870661;Lepidoptera;Boloria euphrosyne;Boloria euphrosyne;1;Boloria euphrosyne;0.999998552914401;
KP870662;Lepidoptera;Aglais urticae;NA;0.002;Aglais urticae;0.987497593219019;
KP870663;Lepidoptera;Coenonympha pamphilus;Coenonympha pamphilus;1;Coenonympha pamphilus;0.999999999999744;
KP870665;Lepidoptera;Thymelicus acteon;Thymelicus acteon;1;Thymelicus acteon;0.99999999752248;
KP870667;Lepidoptera;Aphantopus hyperantus;Aphantopus hyperantus;NA;Aphantopus hyperantus;0.999996892516745;
KP870668;Lepidoptera;Lasiommata megera;Lasiommata megera;1;Lasiommata megera;0.999928528239676;
KP870669;Lepidoptera;Pyrgus foulquieri;Pyrgus foulquieri;0.005;Pyrgus foulquieri;0.741885925757585;
KP870670;Lepidoptera;Pyrgus foulquieri;Pyrgus foulquieri;0.005;Pyrgus foulquieri;0.741885925757585;
KP870672;Lepidoptera;Hipparchia semele;NA;0.001;Hipparchia semele;0.77443093634494;
KP870675;Lepidoptera;Pyrgus onopordi;Pyrgus onopordi;1;Pyrgus onopordi;0.999997111229755;
KP870676;Lepidoptera;Minois dryas;NA;0.025;Minois dryas;0.850746026247397;
KP870678;Lepidoptera;Callophrys rubi;NA;0.002;Callophrys rubi;0.998841790162773;
KP870679;Lepidoptera;Cupido minimus;NA;0.009;Cupido minimus;0.999791453712643;
KP870681;Lepidoptera;Erebia pandrose;Erebia pandrose;0.998;Erebia pandrose;0.99797994145198;
KP870683;Lepidoptera;Lasiommata maera;Lasiommata maera;1;Lasiommata maera;0.999997945450226;
KP870685;Lepidoptera;Maniola tithonus;Pyronia tithonus;1;Pyronia tithonus;1;

KP870687;Lepidoptera;Fabriciana adippe;NA;0.392;Fabriciana adippe;0.917232439736753;
KP870688;Lepidoptera;Polyommatus escheri;Polyommatus escheri;1;Polyommatus escheri;0.999998418369956;
KP870689;Lepidoptera;Arethusana arethusa;Arethusana arethusa;0.045;Arethusana arethusa;0.999962297609568;
KP870690;Lepidoptera;Gonepteryx rhamni;NA;0.474;Gonepteryx rhamni;0.982076396127858;
KP870692;Lepidoptera;Lycaena hippothoe;NA;NA;Lycaena hippothoe;0.928973453460131;
KP870694;Lepidoptera;Plebejus argus;Plebejus argus;0.006;Plebejus argus;0.988245271162348;
KP870695;Lepidoptera;Issoria lathonia;Issoria lathonia;1;Issoria lathonia;0.999999999989257;
KP870696;Lepidoptera;Aglais urticae;NA;0.002;Aglais urticae;0.987497593219019;
KP870697;Lepidoptera;Colias crocea;NA;0.059;NA;0.999999999999738;
KP870700;Lepidoptera;Apatura iris;Apatura iris;1;Apatura iris;0.999999999999972;
KP870701;Lepidoptera;Celastrina argiolus;Celastrina argiolus;1;Celastrina argiolus;0.999037794018796;
KP870702;Lepidoptera;Coenonympha lyllus;Coenonympha pamphilus;1;Coenonympha pamphilus;0.999999999996902;
KP870704;Lepidoptera;Melitaea didyma;Melitaea didyma;1;Melitaea didyma;0.994281215815027;
KP870705;Lepidoptera;Coenonympha pamphilus;Coenonympha pamphilus;1;Coenonympha pamphilus;0.999999999999744;
KP870706;Lepidoptera;Brenthis daphne;Brenthis daphne;1;Brenthis daphne;0.997172799742872;
KP870707;Lepidoptera;Boloria euphrosyne;Boloria euphrosyne;0.001;Boloria euphrosyne;0.999965608361016;
KP870708;Lepidoptera;Minois dryas;NA;0.025;Minois dryas;0.850746026247397;
KP870710;Lepidoptera;Boloria titania;Boloria titania;0.001;Boloria titania;0.999933039420112;
KP870711;Lepidoptera;Apatura iris;Apatura iris;1;Apatura iris;0.999999999999972;
KP870712;Lepidoptera;Pyrgus andromedae;Pyrgus andromedae;1;Pyrgus andromedae;0.997371767954981;
KP870713;Lepidoptera;Thymelicus sylvestris;Thymelicus sylvestris;1;Thymelicus sylvestris;0.999999985935205;
KP870714;Lepidoptera;Maniola cecilia;Pyronia cecilia;1;Pyronia cecilia;1;
KP870715;Lepidoptera;Apatura iris;Apatura iris;1;Apatura iris;0.999999999999972;
KP870718;Lepidoptera;Minois dryas;NA;0.011;Minois dryas;0.911200988453291;
KP870719;Lepidoptera;Carcharodus alceae;Carcharodus alceae;0.002;Carcharodus alceae;0.999999995967272;
KP870723;Lepidoptera;Helleia helle;Lycaena helle;1;Lycaena helle;0.999999995066105;
KP870724;Lepidoptera;Gegenes nostrodamus;Gegenes nostrodamus;1;Gegenes nostrodamus;0.999999996883645;
KP870725;Lepidoptera;Favonius quercus;NA;0.135;NA;0.999999999364868;
KP870726;Lepidoptera;Lasiommata megera;Lasiommata megera;1;Lasiommata megera;0.999928528239676;
KP870727;Lepidoptera;Pyrgus alveus;NA;NA;Pyrgus alveus;0.931326683565012;
KP870728;Lepidoptera;Minois dryas;NA;0.025;Minois dryas;0.850746026247397;
KP870730;Lepidoptera;Arethusana arethusa;NA;0.062;Arethusana arethusa;0.999962489703143;
KP870732;Lepidoptera;Thecla betulae;Thecla betulae;1;Thecla betulae;0.999999999961346;
KP870733;Lepidoptera;Maniola tithonus;Pyronia tithonus;1;Pyronia tithonus;1;
KP870735;Lepidoptera;Lampides boeticus;Lampides boeticus;1;Lampides boeticus;0.999999998557229;
KP870737;Lepidoptera;Pyrgus cacaliae;Pyrgus cacaliae;0.999;Pyrgus cacaliae;0.997229839348598;
KP870740;Lepidoptera;Brenthis daphne;NA;0.111;NA;0.999999999817875;
KP870743;Lepidoptera;Minois dryas;NA;0.025;Minois dryas;0.850746026247397;
KP870744;Lepidoptera;Melitaea didyma;Melitaea didyma;NA;Melitaea didyma;0.980272836986251;
KP870745;Lepidoptera;Apatura ilia;NA;0.165;Apatura ilia;0.956796812031481;
KP870747;Lepidoptera;Chazara briseis;Chazara briseis;0.002;Chazara briseis;0.999962623085466;
KP870748;Lepidoptera;Pyrgus alveus;NA;NA;Pyrgus alveus;0.931326683565012;
KP870749;Lepidoptera;Melitaea didyma;Melitaea didyma;1;Melitaea didyma;0.993439843713843;
KP870750;Lepidoptera;Hipparchia fagi;NA;NA;Hipparchia fagi;0.987735485715459;
KP870751;Lepidoptera;Thymelicus sylvestris;NA;0.324;Thymelicus sylvestris;0.999940778442454;
KP870752;Lepidoptera;Oeneis glacialis;NA;NA;Oeneis glacialis;0.835029434446287;
KP870753;Lepidoptera;Lasiommata megera;Lasiommata megera;1;Lasiommata megera;0.999928528239676;
KP870757;Lepidoptera;Thymelicus acteon;Thymelicus acteon;1;Thymelicus acteon;0.999999963752175;
KP870759;Lepidoptera;Minois dryas;NA;0.025;Minois dryas;0.850746026247397;
KP870760;Lepidoptera;Melitaea parthenoides;Melitaea parthenoides;1;Melitaea parthenoides;0.999999993530452;
KP870762;Lepidoptera;Melitaea deione;Melitaea deione;1;Melitaea deione;0.999999999588169;
KP870763;Lepidoptera;Fabriciana niobe;NA;0.001;Fabriciana niobe;0.97642748807868;
KP870764;Lepidoptera;Pyrgus foulquieri;Pyrgus foulquieri;0.005;Pyrgus foulquieri;0.741885925757585;
KP870768;Lepidoptera;Brenthis daphne;Brenthis daphne;1;Brenthis daphne;0.997172799742872;
KP870769;Lepidoptera;Phengaris arion;NA;0.054;Phengaris arion;0.938397095316026;
KP870770;Lepidoptera;Coenonympha rhodopensis;Coenonympha rhodopensis;0.01;Coenonympha rhodopensis;0.912323431995304;
KP870772;Lepidoptera;Hipparchia hermione;NA;NA;NA;0.999999999999991;
KP870774;Lepidoptera;Carterocephalus palaemon;NA;0.033;Carterocephalus palaemon;0.744872231374349;
KP870777;Lepidoptera;Thymelicus sylvestris;Thymelicus sylvestris;1;Thymelicus sylvestris;0.999999985935205;
KP870778;Lepidoptera;Erebia triaria;NA;0.116;Erebia triarius;0.702820281716756;
KP870779;Lepidoptera;Satyrium esculi;Satyrium esculi;1;Satyrium esculi;0.999999957367948;
KP870780;Lepidoptera;Erebia oeme;NA;0.175;Erebia oeme;0.99999999991374;
KP870781;Lepidoptera;Scolitantides orion;Scolitantides orion;1;Scolitantides orion;0.999999953757596;

KP870789;Lepidoptera;Fabriciana niobe;NA;0.007;Fabriciana niobe;0.984627988609443;
KP870792;Lepidoptera;Cupido minimus;NA;0.009;Cupido minimus;0.999791453712643;
KP870794;Lepidoptera;Phengaris arion;NA;0.054;Phengaris arion;0.938397095316026;
KP870797;Lepidoptera;Melitaea trivia;Melitaea trivia;1;Melitaea trivia;0.999999999960949;
KP870798;Lepidoptera;Polyommatus thersites;NA;0.121;Polyommatus thersites;0.999999047413893;
KP870801;Lepidoptera;Boloria dia;Boloria dia;1;Boloria dia;0.999999999980503;
KP870802;Lepidoptera;Melitaea trivia;Melitaea trivia;1;Melitaea trivia;0.999999999977604;
KP870803;Lepidoptera;Cupido minimus;NA;0.216;Cupido lorquinii;0.726278995315528;
KP870805;Lepidoptera;Melitaea cinxia;Melitaea cinxia;1;Melitaea cinxia;0.999999999999488;
KP870806;Lepidoptera;Satyrium esculi;Satyrium esculi;1;Satyrium esculi;0.999999957367948;
KP870807;Lepidoptera;Satyrium ilicis;Satyrium ilicis;1;Satyrium ilicis;0.999999987901305;
KP870808;Lepidoptera;Chazara briseis;Chazara briseis;0.001;Chazara briseis;0.999984130678865;
KP870809;Lepidoptera;Thymelicus acteon;Thymelicus acteon;1;Thymelicus acteon;0.99999999752248;
KP870810;Lepidoptera;Lampides boeticus;Lampides boeticus;NA;Lampides boeticus;0.999999999307221;
KP870811;Lepidoptera;Vanessa atalanta;Vanessa atalanta;1;Vanessa atalanta;0.999999999757762;
KP870812;Lepidoptera;Erebia meolans;Erebia meolans;NA;Erebia meolans;0.999791585691543;
KP870813;Lepidoptera;Satyrium w-album;NA;0.158;Satyrium w-album;0.991135458498042;
KP870815;Lepidoptera;Cupido minimus;NA;0.089;Cupido lorquinii;0.728383684109358;
KP870817;Lepidoptera;Hipparchia hermione;NA;NA;NA;0.999999999999991;
KP870818;Lepidoptera;Fabriciana niobe;NA;0.014;Fabriciana niobe;0.980110477277841;
KP870819;Lepidoptera;Pyrgus armoricanus;Pyrgus armoricanus;1;Pyrgus armoricanus;0.999998858647755;
KP870820;Lepidoptera;Carcharodus baeticus;Carcharodus baeticus;1;Carcharodus baeticus;0.999983899664537;
KP870822;Lepidoptera;Chazara briseis;Chazara briseis;0.003;Chazara briseis;0.999944393154726;
KP870823;Lepidoptera;Pyrgus armoricanus;Pyrgus armoricanus;1;Pyrgus armoricanus;0.999997353848817;
KP870824;Lepidoptera;Pararge aegeria;Pararge aegeria;1;Pararge aegeria;0.999999999963165;
KP870825;Lepidoptera;Cupido minimus;NA;0.024;Cupido lorquinii;0.822471829137295;
KP870827;Lepidoptera;Satyrium esculi;Satyrium esculi;1;Satyrium esculi;0.999999991927865;
KP870829;Lepidoptera;Minois dryas;NA;0.025;Minois dryas;0.850746026247397;
KP870830;Lepidoptera;Anthocharis cardamines;Anthocharis cardamines;1;Anthocharis cardamines;0.999999988995796;
KP870831;Lepidoptera;Pyrgus alveus;NA;NA;Pyrgus alveus;0.944546694610372;
KP870832;Lepidoptera;Pyrgus cacaliae;Pyrgus cacaliae;0.999;Pyrgus cacaliae;0.997229839348598;
KP870834;Lepidoptera;Arethusana arethusa;Arethusana arethusa;0.045;Arethusana arethusa;0.999962297609568;
KP870836;Lepidoptera;Melitaea didyma;Melitaea didyma;1;Melitaea didyma;0.978209045677336;
KP870838;Lepidoptera;Carcharodus baeticus;Carcharodus baeticus;1;Carcharodus baeticus;0.999983899664537;
KP870839;Lepidoptera;Heteropterus morpheus;NA;NA;Heteropterus morpheus;0.999999999999943;
KP870841;Lepidoptera;Melitaea cinxia;Melitaea cinxia;1;Melitaea cinxia;0.999999999999517;
KP870842;Lepidoptera;Satyrium spini;Satyrium spini;1;Satyrium spini;0.999998173309697;
KP870843;Lepidoptera;Cercyonis lupina;Hyponephele lupina;1;Hyponephele lupina;0.999999973380085;
KP870848;Lepidoptera;Pyrgus armoricanus;Pyrgus armoricanus;1;Pyrgus armoricanus;0.999998326740418;
KP870849;Lepidoptera;Coenonympha pamphilus;Coenonympha pamphilus;1;Coenonympha pamphilus;0.99999999999946;
KP870851;Lepidoptera;Phengaris arion;NA;0.048;Phengaris arion;0.925121569803617;
KP870853;Lepidoptera;Satyrium spini;Satyrium spini;1;Satyrium spini;0.999997334596599;
KP870854;Lepidoptera;Celastrina argiolus;Celastrina argiolus;1;Celastrina argiolus;0.999527385150531;
KP870857;Lepidoptera;Carcharodus alceae;Carcharodus alceae;1;Carcharodus alceae;0.999999996479374;
KP870860;Lepidoptera;Carcharodus alceae;Carcharodus alceae;0.001;Carcharodus alceae;0.999999970193983;
KP870861;Lepidoptera;Brenthis ino;Brenthis ino;0.001;Brenthis ino;0.993162660176952;
KP870862;Lepidoptera;Pyrgus carthami;Pyrgus carthami;1;Pyrgus carthami;0.999981615717954;
KP870864;Lepidoptera;Pyrgus alveus;NA;NA;Pyrgus alveus;0.931326683565012;
KP870869;Lepidoptera;Hipparchia hermione;NA;NA;NA;0.999999999999991;
KP870870;Lepidoptera;Satyrium spini;Satyrium spini;1;Satyrium spini;0.999997869643319;
KP870871;Lepidoptera;Arethusana arethusa;Arethusana arethusa;1;Arethusana arethusa;0.999999787144355;
KP870872;Lepidoptera;Fabriciana adippe;Fabriciana adippe;0.001;Fabriciana adippe;0.999538788643547;
KP870873;Lepidoptera;Parnassius apollo;Parnassius apollo;1;Parnassius apollo;0.998823981816303;
KP870874;Lepidoptera;Plebejus argus;Plebejus argus;1;Plebejus argus;0.997297027003937;
KP870877;Lepidoptera;Apatura iris;Apatura iris;1;Apatura iris;0.999999999999972;
KP870879;Lepidoptera;Brenthis ino;Brenthis ino;0.998;Brenthis ino;0.995325118916732;
KP870880;Lepidoptera;Polyommatus thersites;Polyommatus thersites;0.999;Polyommatus thersites;0.99999985539095;
KP870881;Lepidoptera;Maniola tithonus;Pyronia tithonus;1;Pyronia tithonus;1;
KP870882;Lepidoptera;Celastrina argiolus;Celastrina argiolus;1;Celastrina argiolus;0.999527385150531;
KP870883;Lepidoptera;Coenonympha rhodopensis;Coenonympha rhodopensis;0.001;Coenonympha rhodopensis;0.945819137649883;
KP870884;Lepidoptera;Pyrgus foulquieri;Pyrgus foulquieri;0.005;Pyrgus foulquieri;0.741885925757585;

KP870885;Lepidoptera;Polyommatus thersites;Polyommatus thersites;0.999;Polyommatus thersites;0.999999935754092;
KP870887;Lepidoptera;Cupido minimus;NA;0.216;Cupido lorquinii;0.726278995315528;
KP870888;Lepidoptera;Cupido minimus;NA;0.083;NA;0.999998549281497;
KP870891;Lepidoptera;Brenthis daphne;Brenthis daphne;0.001;Brenthis daphne;0.997781825264304;
KP870894;Lepidoptera;Satyrium esculi;Satyrium esculi;1;Satyrium esculi;0.999999836089486;
KP870898;Lepidoptera;Brenthis ino;NA;0.074;Brenthis ino;0.799697987139591;
KP870899;Lepidoptera;Aglais urticae;NA;0.002;Aglais urticae;0.987497593219019;
KP870900;Lepidoptera;Coenonympha dorus;Coenonympha dorus;1;Coenonympha dorus;0.999999999766146;
KP870901;Lepidoptera;Euphydryas aurinia;NA;0.31;Euphydryas aurinia;0.993387708761386;
KP870902;Lepidoptera;Thymelicus sylvestris;Thymelicus sylvestris;1;Thymelicus sylvestris;0.999999985935205;
KP870903;Lepidoptera;Pyrgus andromedae;Pyrgus andromedae;1;Pyrgus andromedae;0.999808671605833;
KP870906;Lepidoptera;Thymelicus acteon;Thymelicus acteon;1;Thymelicus acteon;0.99999999752248;
KP870907;Lepidoptera;Favonius quercus;NA;NA;Favonius quercus;0.82037508280549;
KP870910;Lepidoptera;Aglais urticae;Aglais urticae;0.004;Aglais urticae;0.995016750255472;
KP870911;Lepidoptera;Aglais urticae;NA;0.002;Aglais urticae;0.987497593219019;
KP870913;Lepidoptera;Fabriciana adippe;NA;NA;Fabriciana adippe;0.885713811096646;
KP870915;Lepidoptera;Pyrgus carthami;Pyrgus carthami;1;Pyrgus carthami;0.999981615717954;
KP870916;Lepidoptera;Erebia epiphron;Erebia epiphron;0.996;Erebia epiphron;0.998796590633588;
KP870917;Lepidoptera;Hipparchia hermione;NA;NA;NA;0.999999999999991;
KP870918;Lepidoptera;Satyrium w-album;NA;0.056;Satyrium w-album;0.989378230996316;
KP870919;Lepidoptera;Satyrium spini;Satyrium spini;1;Satyrium spini;0.999993192602438;
KP870920;Lepidoptera;Arethusana arethusa;Arethusana arethusa;0.047;Arethusana arethusa;0.999974033031615;
KP870921;Lepidoptera;Glaucopsyche melanops;Glaucopsyche melanops;1;Glaucopsyche melanops;0.999952924254607;
KP870923;Lepidoptera;Satyrium acaciae;Satyrium acaciae;1;Satyrium acaciae;0.999999938005745;
KP870924;Lepidoptera;Thymelicus sylvestris;NA;0.324;Thymelicus sylvestris;0.999940778442454;
KP870927;Lepidoptera;Papilio machaon;Papilio machaon;0.001;Papilio machaon;0.999463129150422;
KP870928;Lepidoptera;Aglais urticae;NA;0.002;Aglais urticae;0.987497593219019;
KP870929;Lepidoptera;Satyrium acaciae;Satyrium acaciae;1;Satyrium acaciae;0.99999991343697;
KP870930;Lepidoptera;Satyrium esculi;Satyrium esculi;1;Satyrium esculi;0.99999997326492;
KP870931;Lepidoptera;Erebia epiphron;Erebia epiphron;0.996;Erebia epiphron;0.998796590633588;
KP870932;Lepidoptera;Erynnis tages;Erynnis tages;1;Erynnis tages;0.999996685244108;
KP870933;Lepidoptera;Callophrys rubi;NA;0.002;Callophrys rubi;0.998841790162773;
KP870934;Lepidoptera;Hipparchia statilinus;Hipparchia statilinus;1;Hipparchia statilinus;0.99999999999784;
KP870935;Lepidoptera;Polyommatus thersites;Polyommatus thersites;0.999;Polyommatus thersites;0.999999916395897;
KP870936;Lepidoptera;Lycaena virgaureae;NA;0.125;Lycaena virgaureae;0.999002077309996;
KP870937;Lepidoptera;Thymelicus sylvestris;NA;0.324;Thymelicus sylvestris;0.999940778442454;
KP870938;Lepidoptera;Aphantopus hyperantus;Aphantopus hyperantus;0.003;Aphantopus hyperantus;0.999999455871523;
KP870941;Lepidoptera;Hipparchia hermione;Hipparchia hermione;1;Hipparchia hermione;0.999783016818011;
KP870942;Lepidoptera;Hipparchia hermione;Hipparchia hermione;1;Hipparchia hermione;0.999905429250471;
KP870944;Lepidoptera;Melitaea deione;Melitaea deione;1;Melitaea deione;0.999999999503189;
KP870945;Lepidoptera;Fabriciana niobe;NA;0.005;Fabriciana niobe;0.992125835358755;
KP870949;Lepidoptera;Vanessa cardui;Vanessa cardui;1;Vanessa cardui;0.999997579965112;
KP870951;Lepidoptera;Satyrium ilicis;Satyrium ilicis;1;Satyrium ilicis;0.999999987901305;
KP870953;Lepidoptera;Melitaea deione;Melitaea deione;1;Melitaea deione;0.999999999503189;
KP870955;Lepidoptera;Euphydryas maturna;NA;0.126;Euphydryas maturna;0.885705216462044;
KP870956;Lepidoptera;Thymelicus acteon;Thymelicus acteon;1;Thymelicus acteon;0.99999999752248;
KP870957;Lepidoptera;Thymelicus sylvestris;NA;0.324;Thymelicus sylvestris;0.999940778442454;
KP870958;Lepidoptera;Fabriciana adippe;NA;0.001;Fabriciana adippe;0.924751459079782;
KP870959;Lepidoptera;Maniola cecilia;Pyronia cecilia;1;Pyronia cecilia;1;
KP870960;Lepidoptera;Lycaena phlaeas;Lycaena phlaeas;1;Lycaena phlaeas;0.999999996363186;
KP870962;Lepidoptera;Celastrina argiolus;Celastrina argiolus;1;Celastrina argiolus;0.999037794018796;
KP870964;Lepidoptera;Callophrys rubi;NA;0.001;Callophrys rubi;0.998898923869396;
KP870966;Lepidoptera;Pararge aegeria;Pararge aegeria;1;Pararge aegeria;0.999999999963165;
KP870968;Lepidoptera;Colias crocea;NA;0.059;NA;0.999999999999738;
KP870969;Lepidoptera;Erebia meolans;Erebia meolans;NA;Erebia meolans;0.999708273694177;
KP870970;Lepidoptera;Thymelicus acteon;Thymelicus acteon;1;Thymelicus acteon;0.99999999752248;
KP870971;Lepidoptera;Brenthis daphne;Brenthis daphne;1;Brenthis daphne;0.997172799742872;
KP870972;Lepidoptera;Pyrgus alveus;NA;NA;Pyrgus alveus;0.931326683565012;
KP870973;Lepidoptera;Pyrgus andromedae;Pyrgus andromedae;1;Pyrgus andromedae;0.998809971311763;
KP870977;Lepidoptera;Maniola cecilia;Pyronia cecilia;NA;Pyronia cecilia;1;
KP870979;Lepidoptera;Satyrus ferula;Satyrus ferula;0.001;Satyrus ferula;0.993138467028266;
KP870980;Lepidoptera;Erebia epiphron;Erebia epiphron;NA;Erebia epiphron;0.998837748029833;
KP870981;Lepidoptera;Scolitantides orion;Scolitantides orion;1;Scolitantides orion;0.999999984804305;
KP870984;Lepidoptera;Pyrgus armoricanus;Pyrgus armoricanus;1;Pyrgus armoricanus;0.999997353848817;
KP870985;Lepidoptera;Limenitis camilla;Limenitis camilla;0.009;Limenitis camilla;0.9999828626808;

KP870986;Lepidoptera;Pyrgus cacaliae;Pyrgus cacaliae;0.999;Pyrgus cacaliae;0.997229839348598;
KP870987;Lepidoptera;Pyrgus andromedae;Pyrgus andromedae;1;Pyrgus andromedae;0.997371767954981;
KP870989;Lepidoptera;Apatura iris;Apatura iris;1;Apatura iris;0.999999999999972;
KP870990;Lepidoptera;Brintesia circe;Brintesia circe;1;Brintesia circe;0.99999744550143;
KP870992;Lepidoptera;Minois dryas;NA;0.025;Minois dryas;0.850746026247397;
KP870993;Lepidoptera;Pyrgus alveus;NA;NA;Pyrgus alveus;0.931326683565012;
KP870995;Lepidoptera;Minois dryas;NA;0.025;Minois dryas;0.850746026247397;
KP870996;Lepidoptera;Aglais urticae;Aglais urticae;0.001;Aglais urticae;0.994396335177485;
KP870998;Lepidoptera;Lasiommata maera;Lasiommata maera;1;Lasiommata maera;0.999997945450226;
KP870999;Lepidoptera;Hipparchia hermione;Hipparchia hermione;1;Hipparchia hermione;0.999867997302225;
KP871001;Lepidoptera;Pyrgus andromedae;Pyrgus andromedae;1;Pyrgus andromedae;0.997371767954981;
KP871002;Lepidoptera;Satyrium spini;Satyrium spini;1;Satyrium spini;0.999990225283962;
KP871003;Lepidoptera;Pyrgus alveus;NA;NA;Pyrgus alveus;0.944546694610372;
KP871004;Lepidoptera;Gonepteryx rhamni;Gonepteryx rhamni;0.002;Gonepteryx rhamni;0.999788876860678;
KP871005;Lepidoptera;Plebejus argus;NA;NA;Plebejus argus;0.969283117748927;
KP871007;Lepidoptera;Cupido minimus;NA;0.009;Cupido minimus;0.999791453712643;
KP871008;Lepidoptera;Minois dryas;NA;0.025;Minois dryas;0.850746026247397;
KP871009;Lepidoptera;Vanessa cardui;Vanessa cardui;1;Vanessa cardui;0.999999459983318;
KP871010;Lepidoptera;Cupido minimus;NA;0.009;Cupido minimus;0.999791453712643;
KP871012;Lepidoptera;Lycaena tityrus;Lycaena tityrus;1;Lycaena tityrus;0.999997855580031;
KP871015;Lepidoptera;Apatura ilia;NA;0.165;Apatura ilia;0.956796812031481;
KP871017;Lepidoptera;Colias crocea;NA;0.059;NA;0.999999999999738;
KP871018;Lepidoptera;Hipparchia semele;NA;0.001;Hipparchia semele;0.862655722502182;
KP871020;Lepidoptera;Agriades glandon;NA;0.002;Agriades glandon;0.987149722429845;
KP871021;Lepidoptera;Aglais urticae;NA;0.001;Aglais urticae;0.98874782597203;
KP871023;Lepidoptera;Cupido osiris;Cupido osiris;1;Cupido osiris;0.999998710927265;
KP871024;Lepidoptera;Thymelicus acteon;Thymelicus acteon;1;Thymelicus acteon;0.999999998000504;
KP871027;Lepidoptera;Brenthis hecate;NA;0.005;Brenthis hecate;0.947748679440882;
KP871029;Lepidoptera;Satyrium w-album;NA;0.056;Satyrium w-album;0.989378230996316;
KP871031;Lepidoptera;Pyrgus foulquieri;Pyrgus foulquieri;0.005;Pyrgus foulquieri;0.741885925757585;
KP871032;Lepidoptera;Polyommatus escheri;Polyommatus escheri;1;Polyommatus escheri;0.999999994183696;
KP871033;Lepidoptera;Erebia euryale;NA;NA;Erebia euryale;0.93493911427573;
KP871035;Lepidoptera;Thecla betulae;Thecla betulae;1;Thecla betulae;0.999999999972886;
KP871036;Lepidoptera;Satyrium spini;Satyrium spini;1;Satyrium spini;0.999998173309697;
KP871037;Lepidoptera;Pieris napi;NA;NA;NA;100.000.000.000.001;
KP871039;Lepidoptera;Cupido minimus;NA;0.009;Cupido minimus;0.999791453712643;
KP871040;Lepidoptera;Vanessa cardui;Vanessa cardui;1;Vanessa cardui;0.999999803450484;
KP871041;Lepidoptera;Brenthis ino;Brenthis ino;0.998;Brenthis ino;0.995325118916732;
KP871042;Lepidoptera;Lampides boeticus;Lampides boeticus;1;Lampides boeticus;0.999999999545906;
KP871046;Lepidoptera;Pyrgus alveus;NA;NA;Pyrgus alveus;0.931326683565012;
KP871047;Lepidoptera;Callophrys rubi;NA;0.002;Callophrys rubi;0.998841790162773;
KP871049;Lepidoptera;Heteropterus morpheus;NA;NA;Heteropterus morpheus;0.999999999999943;
KP871051;Lepidoptera;Brenthis hecate;NA;0.031;Brenthis hecate;0.99924976100958;
KP871052;Lepidoptera;Satyrium acaciae;Satyrium acaciae;1;Satyrium acaciae;0.999999951077798;
KP871054;Lepidoptera;Pyrgus alveus;NA;NA;Pyrgus alveus;0.931326683565012;
KP871055;Lepidoptera;Erebia meolans;Erebia meolans;NA;Erebia meolans;0.999835953426128;
KP871059;Lepidoptera;Boloria pales;Boloria pales;1;Boloria pales;0.998536542463107;
KP871061;Lepidoptera;Apatura iris;Apatura iris;1;Apatura iris;0.999999999999972;
KP871063;Lepidoptera;Gonepteryx rhamni;Gonepteryx rhamni;1;Gonepteryx rhamni;0.999865258729401;
KP871065;Lepidoptera;Callophrys rubi;NA;0.001;Callophrys rubi;0.99870518510598;
KP871069;Lepidoptera;Lysandra coridon;Lysandra coridon;NA;NA;0.999966047347831;
KP871070;Lepidoptera;Arethusana arethusa;NA;0.053;Arethusana arethusa;0.999947559376708;
KP871071;Lepidoptera;Charaxes jasius;Charaxes jasius;NA;Charaxes jasius;0.988941612024336;
KP871072;Lepidoptera;Agriades glandon;NA;0.002;Agriades glandon;0.987149722429845;
KP871074;Lepidoptera;Apatura ilia;NA;0.165;Apatura ilia;0.956796812031481;
KP871075;Lepidoptera;Chazara briseis;Chazara briseis;1;Chazara briseis;0.999985122765388;
KP871079;Lepidoptera;Lycaena phlaeas;Lycaena phlaeas;1;Lycaena phlaeas;0.999999996363186;
KP871082;Lepidoptera;Thecla betulae;Thecla betulae;1;Thecla betulae;0.999999999974676;
KP871084;Lepidoptera;Coenonympha pamphilus;Coenonympha pamphilus;1;Coenonympha pamphilus;0.999999999999744;
KP871089;Lepidoptera;Anthocharis cardamines;Anthocharis cardamines;0.001;Anthocharis cardamines;0.999999816482432;
KP871090;Lepidoptera;Pyrgus cacaliae;Pyrgus cacaliae;0.999;Pyrgus cacaliae;0.997229839348598;
KP871091;Lepidoptera;Callophrys rubi;NA;0.003;Callophrys rubi;0.998769313902836;
KP871092;Lepidoptera;Plebejus idas;NA;0.007;NA;0.999546324331549;
KP871093;Lepidoptera;Thymelicus acteon;Thymelicus acteon;1;Thymelicus acteon;0.99999999752248;
KP871094;Lepidoptera;Coenonympha dorus;Coenonympha dorus;1;Coenonympha dorus;0.99999999988259;
KP871096;Lepidoptera;Aglais urticae;Aglais urticae;0.001;Aglais urticae;0.994396335177485;
KP871098;Lepidoptera;Chazara briseis;Chazara briseis;0.001;Chazara briseis;0.999942611553846;
KP871099;Lepidoptera;Limenitis camilla;Limenitis camilla;0.01;Limenitis camilla;0.999988164408943;
KP871103;Lepidoptera;Pyrgus armoricanus;Pyrgus armoricanus;1;Pyrgus armoricanus;0.999997353848817;
KP871105;Lepidoptera;Cupido minimus;NA;0.009;Cupido minimus;0.999791453712643;

KP871107;Lepidoptera;Satyrium w-album;NA;0.056;Satyrium w-album;0.989378230996316;
KP871109;Lepidoptera;Thymelicus lineola;Thymelicus lineola;1;Thymelicus lineola;0.999930684106799;
KP871110;Lepidoptera;Pyrgus onopordi;Pyrgus onopordi;1;Pyrgus onopordi;0.999997111229755;
KP871111;Lepidoptera;Gonepteryx cleopatra;Gonepteryx cleopatra;0.998;Gonepteryx cleopatra;0.999118636582266;
KP871113;Lepidoptera;Brintesia circe;Brintesia circe;1;Brintesia circe;0.999999527019638;
KP871119;Lepidoptera;Pyrgus alveus;NA;NA;Pyrgus alveus;0.944546694610372;
KP871121;Lepidoptera;Coenonympha arcania;NA;0.161;Coenonympha arcania;0.962300604949033;
KP871124;Lepidoptera;Maniola tithonus;Pyronia tithonus;NA;Pyronia tithonus;1;
KP871125;Lepidoptera;Aricia eumedon;Eumedonia eumedon;NA;Eumedonia eumedon;0.99134759589959;
KP871126;Lepidoptera;Araschnia levana;Araschnia levana;1;Araschnia levana;0.999999772187832;
KP871127;Lepidoptera;Pyrgus carthami;Pyrgus carthami;1;Pyrgus carthami;0.999981615717954;
KP871129;Lepidoptera;Brenthis ino;Brenthis ino;0.001;Brenthis ino;0.998434780405276;
KP871130;Lepidoptera;Hipparchia hermione;Hipparchia hermione;NA;Hipparchia hermione;0.995161599525584;
KP871134;Lepidoptera;Araschnia levana;Araschnia levana;1;Araschnia levana;0.99999980307634;
KP871137;Lepidoptera;Erynnis tages;Erynnis tages;1;Erynnis tages;0.999999281987018;
KP871139;Lepidoptera;Thecla betulae;Thecla betulae;1;Thecla betulae;0.999999999972886;
KP871140;Lepidoptera;Thymelicus sylvestris;Thymelicus sylvestris;1;Thymelicus sylvestris;0.999999985935205;
KP871141;Lepidoptera;Coenonympha pamphilus;Coenonympha pamphilus;1;Coenonympha pamphilus;0.999999999999744;
KP871142;Lepidoptera;Fabriciana adippe;Fabriciana adippe;0.012;Fabriciana adippe;0.987124560355947;
KP871143;Lepidoptera;Cercyonis lupina;Hyponephele lupina;1;Hyponephele lupina;0.999999983699496;
KP871144;Lepidoptera;Satyrium w-album;NA;0.056;Satyrium w-album;0.989378230996316;
KP871145;Lepidoptera;Satyrus actaea;Satyrus actaea;0.002;Satyrus actaea;0.996459824853088;
KP871146;Lepidoptera;Arethusana arethusa;Arethusana arethusa;0.018;Arethusana arethusa;0.999964729588135;
KP871147;Lepidoptera;Carcharodus lavatherae;Carcharodus lavatherae;1;Carcharodus lavatherae;0.999958116204794;
KP871148;Lepidoptera;Arethusana arethusa;Arethusana arethusa;0.012;Arethusana arethusa;0.999966518960972;
KP871149;Lepidoptera;Lampides boeticus;Lampides boeticus;1;Lampides boeticus;0.999999999545906;
KP871150;Lepidoptera;Anthocharis cardamines;Anthocharis cardamines;1;Anthocharis cardamines;0.999999988995796;
KP871151;Lepidoptera;Thymelicus acteon;Thymelicus acteon;1;Thymelicus acteon;0.99999999752248;
KP871152;Lepidoptera;Carcharodus alceae;Carcharodus alceae;0.001;Carcharodus alceae;0.999999998102538;
KP871155;Lepidoptera;Pyrgus foulquieri;Pyrgus foulquieri;0.005;Pyrgus foulquieri;0.741885925757585;
KP871156;Lepidoptera;Thymelicus sylvestris;Thymelicus sylvestris;1;Thymelicus sylvestris;0.999999979812401;
KP871157;Lepidoptera;Anthocharis cardamines;Anthocharis cardamines;1;Anthocharis cardamines;0.999999936583011;
KP871159;Lepidoptera;Erebia pandrose;Erebia pandrose;0.998;Erebia pandrose;0.998086832694793;
KP871163;Lepidoptera;Melitaea diamina;NA;0.149;Melitaea diamina;0.999999996592464;
KP871164;Lepidoptera;Erebia pronoe;Erebia pronoe;1;Erebia pronoe;0.997847273843868;
KP871165;Lepidoptera;Coenonympha arcania;NA;0.179;Coenonympha arcania;0.96703617676693;
KP871168;Lepidoptera;Arethusana arethusa;NA;0.062;Arethusana arethusa;0.999962489703143;
KP871169;Lepidoptera;Gegenes nostrodamus;Gegenes nostrodamus;1;Gegenes nostrodamus;0.999999996883645;
KP871170;Lepidoptera;Thymelicus sylvestris;NA;0.324;Thymelicus sylvestris;0.999940778442454;
KP871171;Lepidoptera;Cupido minimus;NA;0.216;Cupido lorquinii;0.726278995315528;
KP871172;Lepidoptera;Pyrgus foulquieri;Pyrgus foulquieri;0.005;Pyrgus foulquieri;0.741885925757585;
KP871173;Lepidoptera;Satyrium w-album;NA;0.056;Satyrium w-album;0.989378230996316;
KP871175;Lepidoptera;Polyommatus thersites;Polyommatus thersites;1;Polyommatus thersites;0.999999914332992;
KP871178;Lepidoptera;Issoria lathonia;Issoria lathonia;1;Issoria lathonia;0.999999999962967;
KR007004;Lepidoptera;Lysandra coridon;Lysandra coridon;NA;NA;0.988319761639796;
KR007005;Lepidoptera;Lysandra coridon;Lysandra coridon;NA;NA;0.985116416974902;
KR007006;Lepidoptera;Lysandra coridon;Lysandra coridon;NA;NA;0.985116416974902;
KR007007;Lepidoptera;Lysandra coridon;Lysandra coridon;NA;NA;0.985116416974902;
KR007008;Lepidoptera;Lysandra coridon;Lysandra coridon;NA;NA;0.985116416974902;
KR007009;Lepidoptera;Lysandra coridon;Lysandra coridon;NA;NA;0.986430321219049;
KR007010;Lepidoptera;Lysandra coridon;NA;NA;NA;0.988257507033511;
KR007011;Lepidoptera;Lysandra coridon;Lysandra coridon;NA;NA;0.986430321219049;
KR007012;Lepidoptera;Lysandra coridon;Lysandra coridon;NA;NA;0.986430321219049;
KR007013;Lepidoptera;Lysandra coridon;Lysandra coridon;NA;Lysandra coridon;0.758899829784951;
KR007014;Lepidoptera;Lysandra coridon;Lysandra coridon;NA;Lysandra coridon;0.758899829784951;
KR007015;Lepidoptera;Lysandra coridon;Lysandra coridon;NA;Lysandra coridon;0.758899829784951;
KR007016;Lepidoptera;Lysandra coridon;Lysandra coridon;NA;Lysandra coridon;0.758899829784951;
KR007017;Lepidoptera;Lysandra coridon;Lysandra coridon;NA;Lysandra coridon;0.758899829784951;
KR007018;Lepidoptera;Lysandra coridon;Lysandra coridon;NA;Lysandra coridon;0.758899829784951;
KR007019;Lepidoptera;Lysandra coridon;Lysandra coridon;NA;Lysandra coridon;0.758899829784951;
KR007020;Lepidoptera;Lysandra coridon;Lysandra coridon;NA;Lysandra coridon;0.758899829784951;

KR007021;Lepidoptera;Lysandra coridon;Lysandra coridon;NA;Lysandra coridon;0.758899829784951;
KR007022;Lepidoptera;Lysandra coridon;Lysandra coridon;NA;Lysandra coridon;0.758899829784951;
KR007023;Lepidoptera;Lysandra coridon;Lysandra coridon;NA;Lysandra coridon;0.758899829784951;
KR007024;Lepidoptera;Lysandra coridon;Lysandra coridon;NA;Lysandra coridon;0.758899829784951;
KR007025;Lepidoptera;Lysandra coridon;Lysandra coridon;NA;Lysandra coridon;0.758899829784951;
KR007026;Lepidoptera;Lysandra coridon;Lysandra coridon;NA;Lysandra coridon;0.758899829784951;
KR007027;Lepidoptera;Lysandra coridon;Lysandra coridon;NA;Lysandra coridon;0.758899829784951;
KR007028;Lepidoptera;Lysandra coridon;NA;NA;NA;0.964953375329878;
KR007029;Lepidoptera;Lysandra coridon;NA;NA;NA;0.964953375329878;
KR007030;Lepidoptera;Lysandra coridon;NA;NA;NA;0.964953375329878;
KR007031;Lepidoptera;Lysandra coridon;NA;NA;NA;0.964953375329878;
KR007032;Lepidoptera;Lysandra coridon;NA;NA;NA;0.964953375329878;
KR007033;Lepidoptera;Lysandra coridon;NA;NA;NA;0.986461703775788;
KR007034;Lepidoptera;Lysandra coridon;NA;NA;NA;0.967826704628015;
KR007035;Lepidoptera;Lysandra coridon;NA;NA;NA;0.985885474451966;
KR007036;Lepidoptera;Lysandra coridon;NA;NA;NA;0.985885474451966;
KR007037;Lepidoptera;Lysandra coridon;NA;NA;NA;0.985885474451966;
KR007038;Lepidoptera;Lysandra coridon;Lysandra coridon;NA;Lysandra coridon;0.758899829784951;
KR007039;Lepidoptera;Lysandra coridon;Lysandra coridon;NA;Lysandra coridon;0.758899829784951;
KR007040;Lepidoptera;Lysandra coridon;Lysandra coridon;NA;Lysandra coridon;0.758899829784951;
KR007041;Lepidoptera;Lysandra coridon;Lysandra coridon;NA;Lysandra coridon;0.758899829784951;
KR007042;Lepidoptera;Lysandra coridon;Lysandra coridon;NA;NA;0.990151960898731;
KR007043;Lepidoptera;Lysandra coridon;Lysandra coridon;NA;NA;0.986672563819842;
KR007044;Lepidoptera;Lysandra coridon;Lysandra coridon;NA;NA;0.988779225441574;
KR007045;Lepidoptera;Lysandra coridon;Lysandra coridon;NA;NA;0.988779225441574;
KR007046;Lepidoptera;Lysandra coridon;NA;NA;NA;0.988257507033511;
KR007047;Lepidoptera;Lysandra coridon;NA;NA;NA;0.988257507033511;
KR007048;Lepidoptera;Lysandra coridon;NA;NA;NA;0.988257507033511;
KR007049;Lepidoptera;Lysandra coridon;NA;NA;NA;0.988257507033511;
KR007050;Lepidoptera;Lysandra coridon;NA;NA;NA;0.988257507033511;
KR007051;Lepidoptera;Lysandra coridon;NA;NA;NA;0.979138191572473;
KR007052;Lepidoptera;Lysandra coridon;NA;NA;NA;0.979138191572473;
KR007053;Lepidoptera;Lysandra coridon;NA;NA;NA;0.97847310073614;
KR007054;Lepidoptera;Lysandra coridon;NA;NA;NA;0.97847310073614;
KR007055;Lepidoptera;Lysandra coridon;NA;NA;NA;0.961243119472709;
KR007056;Lepidoptera;Lysandra coridon;Lysandra coridon;NA;Lysandra coridon;0.758899829784951;
KR007057;Lepidoptera;Lysandra coridon;Lysandra coridon;NA;Lysandra coridon;0.776897982541322;
KR007058;Lepidoptera;Lysandra coridon;Lysandra coridon;NA;Lysandra coridon;0.758899829784951;
KR007059;Lepidoptera;Lysandra coridon;Lysandra coridon;NA;Lysandra coridon;0.758899829784951;
KR007060;Lepidoptera;Lysandra coridon;Lysandra coridon;NA;Lysandra coridon;0.758899829784951;
KR007061;Lepidoptera;Lysandra coridon;NA;NA;NA;0.961243119472709;
KR007062;Lepidoptera;Lysandra coridon;NA;NA;NA;0.961243119472709;
KR007063;Lepidoptera;Lysandra coridon;NA;NA;NA;0.961243119472709;
KR007064;Lepidoptera;Lysandra coridon;NA;NA;NA;0.961243119472709;
KR007065;Lepidoptera;Lysandra coridon;NA;NA;NA;0.961243119472709;
KR007066;Lepidoptera;Lysandra coridon;Lysandra coridon;NA;Lysandra coridon;0.758899829784951;
KR007067;Lepidoptera;Lysandra coridon;Lysandra coridon;NA;Lysandra coridon;0.758899829784951;
KR007068;Lepidoptera;Lysandra coridon;Lysandra coridon;NA;Lysandra coridon;0.758899829784951;
KR007069;Lepidoptera;Lysandra coridon;Lysandra coridon;NA;NA;0.99575954286468;
KR007070;Lepidoptera;Lysandra coridon;Lysandra coridon;NA;Lysandra coridon;0.758899829784951;
KR007071;Lepidoptera;Lysandra coridon;Lysandra coridon;NA;NA;0.998374405346466;
KR007072;Lepidoptera;Lysandra coridon;Lysandra coridon;NA;Lysandra coridon;0.770755339120282;
KR007073;Lepidoptera;Lysandra coridon;Lysandra coridon;NA;Lysandra coridon;0.770755339120282;
KR007155;Lepidoptera;Polyommatus icarus;NA;0.139;NA;0.999999367496427;
KT782365;Lepidoptera;Scolitantides orion;Scolitantides orion;1;Scolitantides orion;0.999999981949031;
KT782419;Lepidoptera;Limenitis camilla;Limenitis camilla;0.009;Limenitis camilla;0.9999828626808;
KT782440;Lepidoptera;Hipparchia hermione;Hipparchia hermione;1;Hipparchia hermione;0.999791304970896;
KT782523;Lepidoptera;Coenonympha arcania;NA;0.067;Coenonympha arcania;0.978811371852141;
KT782578;Lepidoptera;Polyommatus damon;Polyommatus damon;1;Polyommatus damon;0.999999972583709;
KT782663;Lepidoptera;Plebejus argyrognomon;NA;0.001;NA;0.998518811781132;
KT782677;Lepidoptera;Thymelicus sylvestris;NA;0.042;Thymelicus sylvestris;0.9999626292829;
KT782725;Lepidoptera;Satyrium w-album;NA;0.056;Satyrium w-album;0.989378230996316;
KT792887;Lepidoptera;Melitaea britomartis;NA;0.187;Mellicta britomartis;0.790714973899495;
KT792888;Lepidoptera;Melitaea britomartis;NA;0.19;NA;0.999999999997893;
KT792889;Lepidoptera;Melitaea britomartis;NA;0.498;NA;0.999999999996363;
KT792890;Lepidoptera;Melitaea britomartis;NA;0.496;NA;0.999999999997713;
KT792891;Lepidoptera;Melitaea britomartis;NA;0.498;NA;0.999999999996363;
KT792892;Lepidoptera;Melitaea britomartis;NA;0.175;Mellicta britomartis;0.80458915409834;
KT792893;Lepidoptera;Melitaea britomartis;NA;0.184;Mellicta britomartis;0.785539669548814;
KT792894;Lepidoptera;Melitaea britomartis;NA;0.19;NA;0.999999999998544;
KT792895;Lepidoptera;Melitaea cinxia;NA;NA;Melitaea cinxia;0.999999999987381;
KT792896;Lepidoptera;Melitaea cinxia;NA;0.23;Melitaea cinxia;0.999999999994742;
KT792897;Lepidoptera;Melitaea diamina;NA;0.327;Melitaea diamina;0.999999935406637;
KT792898;Lepidoptera;Melitaea didyma;Melitaea didyma;1;Melitaea didyma;0.967711726169338;
KT792899;Lepidoptera;Melitaea didyma;Melitaea didyma;1;Melitaea didyma;0.979298151718851;

KT792900;Lepidoptera;Melitaea didyma;Melitaea didyma;1;Melitaea didyma;0.994538950390651;
KT792901;Lepidoptera;Melitaea didyma;Melitaea didyma;1;Melitaea didyma;0.994159805105065;
KT792902;Lepidoptera;Melitaea didyma;Melitaea didyma;1;Melitaea didyma;0.988846550427704;
KT792903;Lepidoptera;Melitaea didyma;Melitaea didyma;1;Melitaea didyma;0.993166498245832;
KT792904;Lepidoptera;Melitaea didyma;Melitaea didyma;1;Melitaea didyma;0.996293536181993;
KT792905;Lepidoptera;Melitaea didyma;Melitaea didyma;1;Melitaea didyma;0.969595766253483;
KT792908;Lepidoptera;Melitaea didyma;Melitaea didyma;0.999;Melitaea didyma;0.954220159771322;
KT874713;Lepidoptera;Melitaea didyma;Melitaea didyma;1;Melitaea didyma;0.991123500108873;
KT874716;Lepidoptera;Melitaea didyma;Melitaea didyma;0.001;Melitaea didyma;0.99813805816878;
KT874720;Lepidoptera;Melitaea didyma;Melitaea didyma;1;Melitaea didyma;0.992165146696479;
KT874721;Lepidoptera;Melitaea didyma;Melitaea didyma;0.008;Melitaea didyma;0.989890661413771;
KT874730;Lepidoptera;Melitaea didyma;Melitaea didyma;0.995;Melitaea didyma;0.975560702519337;
KT874733;Lepidoptera;Melitaea didyma;Melitaea didyma;0.008;Melitaea didyma;0.989890661413771;
KT874743;Lepidoptera;Melitaea didyma;Melitaea didyma;0.008;Melitaea didyma;0.989890661413771;
KT989872;Lepidoptera;Euphydryas intermedia;Euphydryas intermedia;0.995;Euphydryas intermedia;0.812587411154132;
KT989875;Lepidoptera;Euphydryas intermedia;NA;0.013;NA;0.999999999988838;
KU246383;Lepidoptera;Parnassius mnemosyne;Parnassius mnemosyne;1;NA;0.990051275615589;
KU246384;Lepidoptera;Parnassius mnemosyne;Parnassius mnemosyne;1;NA;0.990454591825374;
KU246385;Lepidoptera;Parnassius mnemosyne;Parnassius mnemosyne;1;NA;0.990454591825374;
KU246386;Lepidoptera;Parnassius mnemosyne;Parnassius mnemosyne;1;NA;0.989823203753365;
KU246387;Lepidoptera;Parnassius mnemosyne;Parnassius mnemosyne;1;NA;0.989823203753365;
KU246388;Lepidoptera;Parnassius mnemosyne;Parnassius mnemosyne;1;NA;0.990051275615589;
KU246389;Lepidoptera;Parnassius mnemosyne;Parnassius mnemosyne;0.999;NA;0.992724686477204;
KU246390;Lepidoptera;Parnassius mnemosyne;Parnassius mnemosyne;1;NA;0.990454591825374;
KU246391;Lepidoptera;Parnassius mnemosyne;Parnassius mnemosyne;1;NA;0.991415026297799;
KU246392;Lepidoptera;Parnassius mnemosyne;Parnassius mnemosyne;1;NA;0.990051275615589;
KU246393;Lepidoptera;Parnassius mnemosyne;Parnassius mnemosyne;1;NA;0.990051275615589;
KU246394;Lepidoptera;Parnassius mnemosyne;Parnassius mnemosyne;1;NA;0.989823203753365;
KU246395;Lepidoptera;Parnassius mnemosyne;Parnassius mnemosyne;1;NA;0.990051275615589;
KU246396;Lepidoptera;Parnassius mnemosyne;Parnassius mnemosyne;1;NA;0.990454591825374;
KU246397;Lepidoptera;Parnassius mnemosyne;Parnassius mnemosyne;1;NA;0.990454591825374;
KU246398;Lepidoptera;Parnassius mnemosyne;Parnassius mnemosyne;1;NA;0.990454591825374;
KU246399;Lepidoptera;Parnassius mnemosyne;Parnassius mnemosyne;1;NA;0.990454591825374;
KU246400;Lepidoptera;Parnassius mnemosyne;Parnassius mnemosyne;0.999;NA;0.992812218749276;
KU246401;Lepidoptera;Parnassius mnemosyne;Parnassius mnemosyne;1;NA;0.990051275615589;
KU246402;Lepidoptera;Parnassius mnemosyne;Parnassius mnemosyne;1;NA;0.990454591825374;
KU379779;Lepidoptera;Satyrium ilicis;Satyrium ilicis;1;Satyrium ilicis;0.999999987901305;
KU707843;Lepidoptera;Erebia oeme;NA;0.114;Erebia oeme;0.999999999830635;
KU905362;Lepidoptera;Spialia therapne;Spialia therapne;1;Spialia therapne;0.99999999437938;
KU905370;Lepidoptera;Spialia therapne;Spialia therapne;1;Spialia therapne;0.99999999437938;
KU905509;Lepidoptera;Spialia therapne;Spialia therapne;1;Spialia therapne;0.999999998225945;
KX011032;Lepidoptera;Fabriciana adippe;NA;NA;Fabriciana adippe;0.853915001000558;
KX011033;Lepidoptera;Fabriciana adippe;NA;0.006;NA;0.999999999999074;
KX011034;Lepidoptera;Fabriciana niobe;NA;0.002;Argynnis adippe;0.718182190495788;
KX011035;Lepidoptera;Fabriciana niobe;NA;0.005;Fabriciana adippe;0.814339656239357;
KX011036;Lepidoptera;Argynnis aglaja;NA;NA;Speyeria aglaja;0.833152886629393;
KX011037;Lepidoptera;Argynnis aglaja;NA;NA;Speyeria aglaja;0.83275547848188;
KX039986;Lepidoptera;Pyrgus alveus;NA;0.003;NA;0.999999999997732;
KX040002;Lepidoptera;Erynnis tages;Erynnis tages;1;Erynnis tages;0.999999281987018;
KX040032;Lepidoptera;Brenthis ino;Brenthis ino;0.007;Brenthis ino;0.977157381961834;
KX040040;Lepidoptera;Erebia pharte;Erebia pharte;1;Erebia pharte;0.99999999630495;
KX040042;Lepidoptera;Erebia pandrose;Erebia pandrose;0.998;Erebia pandrose;0.997117103851311;
KX040049;Lepidoptera;Agriades optilete;Agriades optilete;1;Agriades optilete;0.999999991227838;
KX040063;Lepidoptera;Cupido minimus;NA;0.006;Cupido minimus;0.999768371759258;
KX040081;Lepidoptera;Parnassius mnemosyne;Parnassius mnemosyne;1;Parnassius mnemosyne;0.999999584180132;
KX040086;Lepidoptera;Aricia artaxerxes;Plebejus argus;1;Aricia artaxerxes;0.999743187199922;
KX040089;Lepidoptera;Celastrina argiolus;Celastrina argiolus;1;Celastrina argiolus;0.999527385150531;
KX040091;Lepidoptera;Pyrgus malvae;Pyrgus malvae;NA;Pyrgus malvae;0.980121140374433;
KX040103;Lepidoptera;Pontia callidice;Pontia callidice;1;Pontia callidice;0.999999997508183;
KX040111;Lepidoptera;Lycaena tityrus;Lycaena tityrus;1;Lycaena tityrus;0.999997018148077;
KX040121;Lepidoptera;Pyrgus alveus;NA;0.003;NA;0.999999999997732;
KX040126;Lepidoptera;Erebia pronoe;Erebia pronoe;1;Erebia pronoe;0.999567958198288;
KX040143;Lepidoptera;Aricia eumedon;Eumedonia eumedon;0.005;Eumedonia eumedon;0.997725914077716;
KX040149;Lepidoptera;Erebia melampus;Erebia melampus;0.014;Erebia melampus;0.970458013540357;
KX040169;Lepidoptera;Boloria pales;Boloria pales;0.998;Boloria pales;0.996520205418986;
KX040209;Lepidoptera;Pyrgus cacaliae;Pyrgus cacaliae;0.001;Pyrgus cacaliae;0.986004413030866;
KX040210;Lepidoptera;Erebia tyndarus;NA;NA;NA;0.999999999999885;
KX040220;Lepidoptera;Lycaena hippothoe;Lycaena hippothoe;NA;Lycaena hippothoe;0.943512461175779;
KX040221;Lepidoptera;Pyrgus alveus;NA;NA;NA;0.999999999995496;
KX040230;Lepidoptera;Colias phicomone;NA;0.001;Colias phicomone;0.985598879697681;
KX040236;Lepidoptera;Callophrys rubi;NA;0.001;Callophrys rubi;0.99870518510598;
KX040239;Lepidoptera;Lycaena alciphron;Lycaena alciphron;1;Lycaena alciphron;0.999999863088457;
KX040240;Lepidoptera;Erebia pronoe;Erebia pronoe;NA;Erebia pronoe;0.906909765449499;
KX040246;Lepidoptera;Lycaena dispar;Lycaena dispar;1;Lycaena dispar;0.999999987474467;
KX040255;Lepidoptera;Pyrgus alveus;NA;NA;Pyrgus alveus;0.827119616974978;

KX040257;Lepidoptera;Agriades orbitulus;NA;0.01;Agriades orbitulus;0.998991302227708;
KX040261;Lepidoptera;Pyrgus alveus;NA;0.003;NA;0.999999999997732;
KX040279;Lepidoptera;Colias palaeno;NA;0.001;NA;0.999999999982869;
KX040280;Lepidoptera;Aricia artaxerxes;Plebejus argus;1;Aricia artaxerxes;0.999743187199922;
KX040299;Lepidoptera;Pyrgus alveus;NA;0.003;NA;0.999999999997732;
KX040316;Lepidoptera;Pyrgus malvae;Pyrgus malvae;NA;Pyrgus malvae;0.980121140374433;
KX040317;Lepidoptera;Phengaris arion;NA;0.046;Phengaris arion;0.925974249765429;
KX040345;Lepidoptera;Aricia agestis;NA;0.067;Aricia agestis;0.999509743227786;
KX040346;Lepidoptera;Aricia artaxerxes;Plebejus argus;1;Aricia artaxerxes;0.999743187199922;
KX040349;Lepidoptera;Agriades glandon;NA;0.002;Agriades glandon;0.987149722429845;
KX040356;Lepidoptera;Pyrgus alveus;NA;0.003;NA;0.999999999997732;
KX040362;Lepidoptera;Thecla betulae;Thecla betulae;1;Thecla betulae;0.99999999996561;
KX040372;Lepidoptera;Polyommatus icarus;NA;0.13;Polyommatus icarus;0.97976269574874 8;
KX040404;Lepidoptera;Pyrgus serratulae;Pyrgus serratulae;1;Pyrgus serratulae;0.99999989567508;
KX040421;Lepidoptera;Erebia oeme;NA;0.114;Erebia oeme;0.999999999830635;
KX040435;Lepidoptera;Callophrys rubi;NA;0.001;Callophrys rubi;0.99870518510598;
KX040437;Lepidoptera;Erebia medusa;NA;0.004;Erebia medusa;0.98682001175274;
KX040462;Lepidoptera;Polyommatus icarus;NA;0.003;Polyommatus icarus;0.986125651677522;
KX040463;Lepidoptera;Erebia medusa;NA;0.01;Erebia medusa;0.979621232162315;
KX040473;Lepidoptera;Erebia styx;NA;NA;Erebia styx;0.890739244956497;
KX040486;Lepidoptera;Erebia pharte;Erebia pharte;1;Erebia pharte;0.99999999630495;
KX040494;Lepidoptera;Cupido minimus;NA;0.001;Cupido minimus;0.99973296330718;
KX040499;Lepidoptera;Pyrgus alveus;NA;NA;Pyrgus alveus;0.902591602214502;
KX040502;Lepidoptera;Aricia artaxerxes;NA;0.5;Aricia artaxerxes;0.999618624034396;
KX040533;Lepidoptera;Carcharodus alceae;Carcharodus alceae;1;Carcharodus alceae;0.99999999647568;
KX040537;Lepidoptera;Erebia pharte;Erebia pharte;1;Erebia pharte;0.99999999630495;
KX040560;Lepidoptera;Pyrgus alveus;NA;0.003;NA;0.999999999997732;
KX040603;Lepidoptera;Erebia manto;NA;0.068;Erebia manto;0.991765428936011;
KX040612;Lepidoptera;Hamearis lucina;NA;NA;Hamearis lucina;0.999999999984141;
KX040614;Lepidoptera;Pyrgus alveus;NA;0.003;NA;0.999999999997732;
KX040618;Lepidoptera;Erebia pluto;Erebia pluto;1;Erebia pluto;0.999999988858747;
KX040619;Lepidoptera;Aricia artaxerxes;Plebejus argus;0.004;Aricia artaxerxes;0.99904771879725;
KX040625;Lepidoptera;Erebia pronoe;Erebia pronoe;1;Erebia pronoe;0.999567958198288;
KX040628;Lepidoptera;Phengaris arion;NA;0.054;Phengaris arion;0.938397095316026;
KX040630;Lepidoptera;Erebia manto;Erebia manto;0.999;Erebia manto;0.999986127236517;
KX040631;Lepidoptera;Lycaena phlaeas;Lycaena phlaeas;1;Lycaena phlaeas;0.9999999959837;
KX040636;Lepidoptera;Aricia artaxerxes;Plebejus argus;1;Aricia artaxerxes;0.999743187199922;
KX040643;Lepidoptera;Pyrgus alveus;NA;0.003;NA;0.999999999997732;
KX040650;Lepidoptera;Aricia artaxerxes;Plebejus argus;1;Aricia artaxerxes;0.999743187199922;
KX040667;Lepidoptera;Erebia styx;NA;0.123;Erebia styx;0.889337301851033;
KX040676;Lepidoptera;Pyrgus alveus;NA;0.003;NA;0.999999999997732;
KX040685;Lepidoptera;Pyrgus malvae;Pyrgus malvae;NA;Pyrgus malvae;0.980121140374433;
KX040688;Lepidoptera;Pyrgus malvoides;NA;0.107;Pyrgus malvoides;0.970799101353076;
KX040691;Lepidoptera;Pyrgus serratulae;Pyrgus serratulae;1;Pyrgus serratulae;0.99999989567508;
KX040697;Lepidoptera;Erebia oeme;NA;0.178;Erebia oeme;0.999999999838849;
KX040736;Lepidoptera;Coenonympha arcania;NA;0.32;Coenonympha arcania;0.715629089660113;
KX040743;Lepidoptera;Erebia pluto;Erebia pluto;1;Erebia pluto;0.999999932368498;
KX040759;Lepidoptera;Euphydryas maturna;NA;0.128;Euphydryas maturna;0.882179886670326;
KX040769;Lepidoptera;Erynnis tages;Erynnis tages;1;Erynnis tages;0.999999281987018;
KX040774;Lepidoptera;Aricia artaxerxes;Plebejus argus;1;Aricia artaxerxes;0.999743187199922;
KX040793;Lepidoptera;Aricia eumedon;Eumedonia eumedon;0.012;Eumedonia eumedon;0.996977243476777;
KX040833;Lepidoptera;Aricia artaxerxes;Plebejus argus;0.004;Aricia artaxerxes;0.99904771879725;
KX040839;Lepidoptera;Hamearis lucina;NA;NA;Hamearis lucina;0.999999999984141;
KX040846;Lepidoptera;Pyrgus alveus;NA;NA;Pyrgus alveus;0.827119616974978;
KX040852;Lepidoptera;Aricia artaxerxes;Plebejus argus;1;Aricia artaxerxes;0.999743187199922;
KX040861;Lepidoptera;Hipparchia semele;NA;0.002;Hipparchia semele;0.767063813608107;
KX040863;Lepidoptera;Pyrgus alveus;NA;0.003;NA;0.999999999997732;
KX040894;Lepidoptera;Aricia agestis;NA;0.253;Aricia agestis;0.996350017387504;
KX040949;Lepidoptera;Pyrgus alveus;NA;NA;NA;0.999999999992155;
KX040988;Lepidoptera;Hipparchia semele;NA;0.001;Hipparchia semele;0.862655722502182;
KX041017;Lepidoptera;Aricia artaxerxes;Plebejus argus;1;Aricia artaxerxes;0.999743187199922;
KX041027;Lepidoptera;Hipparchia semele;Hipparchia semele;0.001;Hipparchia semele;0.849754858703295;
KX041037;Lepidoptera;Aricia agestis;NA;0.253;Aricia agestis;0.996350017387504;
KX041071;Lepidoptera;Pyrgus warrenensis;NA;0.003;NA;0.99999999999828;
KX041097;Lepidoptera;Erebia eriphyle;Erebia eriphyle;1;Erebia eriphyle;0.999999982347646;
KX041111;Lepidoptera;Erebia eriphyle;Erebia eriphyle;1;Erebia eriphyle;0.999999983979336;
KX041120;Lepidoptera;Erebia epiphron;NA;NA;Erebia epiphron;0.994401488812176;
KX041140;Lepidoptera;Aricia artaxerxes;Plebejus argus;1;Aricia artaxerxes;0.999743187199922;
KX041150;Lepidoptera;Erebia epiphron;NA;NA;Erebia epiphron;0.994401488812176;
KX041159;Lepidoptera;Erebia eriphyle;Erebia eriphyle;1;Erebia eriphyle;0.999999992447471;
KX041163;Lepidoptera;Erebia styx;NA;NA;Erebia styx;0.947012795007953;
KX041171;Lepidoptera;Erebia manto;Erebia manto;0.999;Erebia manto;0.999986127236517;
KX041219;Lepidoptera;Satyrium pruni;NA;0.076;Satyrium pruni;0.990322364021655;
KX041293;Lepidoptera;Satyrium w-album;NA;0.056;Satyrium w-album;0.989378230996316;
KX041493;Lepidoptera;Euphydryas maturna;NA;NA;NA;0.999999999996973;
KX041606;Lepidoptera;Thymelicus sylvestris;NA;0.324;Thymelicus sylvestris;0.999940778442454;

KX041612;Lepidoptera;Araschnia levana;Araschnia levana;1;Araschnia levana;0.999999539033034;
KX041617;Lepidoptera;Coenonympha arcania;NA;0.125;Coenonympha arcania;0.946375678620991;
KX041632;Lepidoptera;Satyrium spini;Satyrium spini;1;Satyrium spini;0.999999894425036;
KX041654;Lepidoptera;Cupido minimus;NA;0.003;Cupido minimus;0.999787792962518;
KX041670;Lepidoptera;Arethusana arethusa;NA;NA;Arethusana arethusa;0.998746634985211;
KX041680;Lepidoptera;Nymphalis c-album;NA;0.215;NA;0.999999192918557;
KX041682;Lepidoptera;Celastrina argiolus;Celastrina argiolus;0.999;Celastrina argiolus;0.999741253489753;
KX041730;Lepidoptera;Colias alfacariensis;Colias alfacariensis;0.001;Colias alfacariensis;0.999857790810452;
KX041743;Lepidoptera;Pieris napi;NA;0.028;Pieris napi;0.888205720664699;
KX041746;Lepidoptera;Araschnia levana;Araschnia levana;1;Araschnia levana;0.999999772187832;
KX041748;Lepidoptera;Colias alfacariensis;Colias alfacariensis;0.001;Colias alfacariensis;0.999926026317526;
KX041750;Lepidoptera;Vanessa cardui;Vanessa cardui;1;Vanessa cardui;0.999999160346886;
KX041755;Lepidoptera;Pararge aegeria;Pararge aegeria;1;Pararge aegeria;0.999999999903309;
KX041760;Lepidoptera;Favonius quercus;NA;NA;NA;0.999999999995578;
KX041777;Lepidoptera;Melitaea parthenoides;Melitaea parthenoides;1;Melitaea parthenoides;0.999999993530452;
KX041778;Lepidoptera;Melitaea parthenoides;Melitaea parthenoides;1;Melitaea parthenoides;0.999999993530452;
KX041812;Lepidoptera;Pararge aegeria;Pararge aegeria;1;Pararge aegeria;0.999999999946255;
KX041825;Lepidoptera;Coenonympha pamphilus;Coenonympha pamphilus;1;Coenonympha pamphilus;0.999999999999659;
KX041853;Lepidoptera;Callophrys rubi;NA;0.003;Callophrys rubi;0.996753822785804;
KX041881;Lepidoptera;Aglais urticae;NA;0.001;Aglais urticae;0.987391064814553;
KX041927;Lepidoptera;Cupido minimus;NA;0.013;Cupido minimus;0.998077282612956;
KX041955;Lepidoptera;Hamearis lucina;NA;NA;Hamearis lucina;0.999999999984141;
KX041965;Lepidoptera;Hamearis lucina;NA;NA;Hamearis lucina;0.999999999982975;
KX041968;Lepidoptera;Thymelicus sylvestris;NA;0.324;Thymelicus sylvestris;0.999940778442454;
KX042293;Lepidoptera;Agriades glandon;Agriades glandon;0.001;Agriades glandon;0.982364968564748;
KX042580;Lepidoptera;Erebia pandrose;NA;0.006;Erebia pandrose;0.984874134171668;
KX043044;Lepidoptera;Favonius quercus;NA;NA;Favonius quercus;0.786853301434446;
KX044025;Lepidoptera;Pieris napi;NA;0.011;Pieris napi;0.801985394724814;
KX044297;Lepidoptera;Aricia agestis;NA;0.253;Aricia agestis;0.996350017387504;
KX044305;Lepidoptera;Erebia nivalis;NA;NA;NA;0.999999999999947;
KX044357;Lepidoptera;Pyrgus alveus;NA;NA;Pyrgus alveus;0.827119616974978;
KX044428;Lepidoptera;Pontia callidice;Pontia callidice;1;Pontia callidice;0.999999997508183;
KX044438;Lepidoptera;Erebia pronoe;Erebia pronoe;NA;Erebia pronoe;0.906909765449499;
KX044496;Lepidoptera;Aricia eumedon;Eumedonia eumedon;0.003;Eumedonia eumedon;0.997225950391515;
KX044537;Lepidoptera;Euphydryas cynthia;Euphydryas cynthia;1;Euphydryas cynthia;0.999999999705039;
KX044556;Lepidoptera;Aricia artaxerxes;Plebejus argus;1;Aricia artaxerxes;0.999743187199922;
KX044564;Lepidoptera;Erebia medusa;NA;0.008;Erebia medusa;0.980649728371849;
KX044592;Lepidoptera;Parnassius mnemosyne;Parnassius mnemosyne;1;Parnassius mnemosyne;0.999999416965636;
KX044620;Lepidoptera;Thymelicus sylvestris;Thymelicus sylvestris;1;Thymelicus sylvestris;0.999999985935205;
KX044637;Lepidoptera;Pyrgus alveus;NA;NA;NA;0.999999999992155;
KX044652;Lepidoptera;Oeneis glacialis;NA;NA;Oeneis glacialis;0.892833742436513;
KX044653;Lepidoptera;Pyrgus alveus;Pyrgus alveus;0.001;Pyrgus alveus;0.968972839336243;
KX044665;Lepidoptera;Pyrgus alveus;NA;NA;Pyrgus alveus;0.962152887824497;
KX044703;Lepidoptera;Scolitantides orion;Scolitantides orion;1;Scolitantides orion;0.999999984804305;
KX044717;Lepidoptera;Erebia manto;Erebia manto;0.999;Erebia manto;0.999986127236517;
KX044818;Lepidoptera;Aricia agestis;NA;0.253;Aricia agestis;0.996350017387504;
KX044894;Lepidoptera;Euphydryas aurinia;NA;0.001;Euphydryas aurinia;0.995576749826865;
KX044900;Lepidoptera;Brenthis daphne;Brenthis daphne;1;Brenthis daphne;0.997257262539705;
KX044907;Lepidoptera;Lycaena tityrus;Lycaena tityrus;1;Lycaena tityrus;0.999995729087172;
KX044929;Lepidoptera;Pyrgus alveus;NA;0.003;NA;0.999999999997732;
KX044940;Lepidoptera;Aricia artaxerxes;Plebejus argus;1;Aricia artaxerxes;0.999743187199922;
KX044969;Lepidoptera;Pyrgus alveus;NA;NA;Pyrgus alveus;0.827119616974978;
KX045038;Lepidoptera;Aricia agestis;NA;0.253;Aricia agestis;0.996350017387504;
KX045208;Lepidoptera;Aricia agestis;NA;0.253;Aricia agestis;0.996350017387504;
KX045217;Lepidoptera;Cupido argiades;NA;0.499;NA;0.999999999984682;
KX045326;Lepidoptera;Agriades glandon;NA;0.002;Agriades glandon;0.987149722429845;
KX045359;Lepidoptera;Pyrgus alveus;NA;0.003;NA;0.999999999997732;
KX045370;Lepidoptera;Pyrgus alveus;NA;NA;Pyrgus alveus;0.827119616974978;
KX045461;Lepidoptera;Boloria titania;Boloria titania;0.001;Boloria titania;0.99983358374111;
KX045511;Lepidoptera;Pyrgus alveus;NA;0.003;NA;0.999999999997732;
KX045542;Lepidoptera;Boloria pales;Boloria pales;0.998;Boloria pales;0.996520205418986;
KX045576;Lepidoptera;Pyrgus alveus;NA;NA;NA;0.999999999995496;
KX045675;Lepidoptera;Colias alfacariensis;Colias alfacariensis;0.998;Colias alfacariensis;0.999914373031199;
KX045705;Lepidoptera;Brenthis ino;Brenthis ino;1;Brenthis ino;0.995903086085865;
KX045718;Lepidoptera;Parnassius mnemosyne;Parnassius mnemosyne;1;Parnassius mnemosyne;0.999999416965636;
KX045810;Lepidoptera;Aricia agestis;NA;0.312;Aricia agestis;0.996478252474614;

KX045814;Lepidoptera;Pyrgus alveus;NA;NA;Pyrgus alveus;0.941697735568124;
KX045862;Lepidoptera;Colias phicomone;NA;0.001;Colias phicomone;0.985598879697681;
KX045900;Lepidoptera;Argynnis aglaja;NA;0.001;Speyeria aglaja;0.796630797745259;
KX045970;Lepidoptera;Agriades glandon;NA;0.002;Agriades glandon;0.987189585708506;
KX046037;Lepidoptera;Polyommatus icarus;NA;0.13;Polyommatus icarus;0.979762695748748;
KX046038;Lepidoptera;Agriades glandon;NA;0.014;Agriades glandon;0.988102942468088;
KX046171;Lepidoptera;Erebia pandrose;Erebia pandrose;0.013;Erebia pandrose;0.974388092470716;
KX046181;Lepidoptera;Lasiommata maera;Lasiommata maera;1;Lasiommata maera;0.999997752443814;
KX046189;Lepidoptera;Lycaena dispar;Lycaena dispar;1;Lycaena dispar;0.999999990743959;
KX046192;Lepidoptera;Pyrgus alveus;NA;NA;NA;0.999999999992155;
KX046261;Lepidoptera;Erebia nivalis;NA;NA;NA;0.999999999999947;
KX046268;Lepidoptera;Aricia agestis;NA;0.253;Aricia agestis;0.996350017387504;
KX046366;Lepidoptera;Boloria pales;Boloria pales;0.001;Boloria pales;0.994313470451071;
KX046408;Lepidoptera;Plebejus argus;Plebejus argus;0.002;Plebejus argus;0.985485404767793;
KX046541;Lepidoptera;Agriades glandon;NA;0.002;Agriades glandon;0.987149722429845;
KX046550;Lepidoptera;Erebia styx;NA;NA;Erebia styx;0.947012795007953;
KX046652;Lepidoptera;Pyrgus alveus;NA;0.003;Pyrgus alveus;0.938585943089171;
KX046734;Lepidoptera;Maniola jurtina;NA;NA;Maniola jurtina;0.710927730171155;
KX046778;Lepidoptera;Melitaea cinxia;Melitaea cinxia;1;Melitaea cinxia;0.999999999999432;
KX046788;Lepidoptera;Lycaena alciphron;Lycaena alciphron;1;Lycaena alciphron;0.999999768423036;
KX046826;Lepidoptera;Aricia agestis;NA;0.253;Aricia agestis;0.996350017387504;
KX046928;Lepidoptera;Erebia epiphron;NA;NA;Erebia epiphron;0.994401488812176;
KX046932;Lepidoptera;Pyrgus serratulae;Pyrgus serratulae;1;Pyrgus serratulae;0.999999905727099;
KX046955;Lepidoptera;Cyaniris semiargus;NA;NA;Cyaniris semiargus;0.999044642832579;
KX047054;Lepidoptera;Brenthis hecate;NA;0.093;Brenthis hecate;0.999579571062591;
KX047114;Lepidoptera;Pyrgus alveus;NA;NA;Pyrgus alveus;0.827119616974978;
KX047207;Lepidoptera;Aricia eumedon;Eumedonia eumedon;0.004;Eumedonia eumedon;0.99844803714109;
KX047263;Lepidoptera;Aricia artaxerxes;Plebejus argus;1;Aricia artaxerxes;0.999743187199922;
KX047279;Lepidoptera;Melitaea diamina;NA;0.305;Melitaea diamina;0.999999994065661;
KX047325;Lepidoptera;Pyrgus alveus;Pyrgus alveus;0.001;Pyrgus alveus;0.968972839336243;
KX047367;Lepidoptera;Satyrium ilicis;Satyrium ilicis;1;Satyrium ilicis;0.999999987901305;
KX047412;Lepidoptera;Pyrgus alveus;NA;NA;Pyrgus alveus;0.827119616974978;
KX047480;Lepidoptera;Agriades glandon;NA;0.002;Agriades glandon;0.987189585708506;
KX047625;Lepidoptera;Plebejus idas;NA;0.007;NA;0.999546324331549;
KX047634;Lepidoptera;Cupido minimus;NA;0.01;Cupido minimus;0.999797157600087;
KX047641;Lepidoptera;Agriades optilete;Agriades optilete;1;Agriades optilete;0.999999948697736;
KX047652;Lepidoptera;Vanessa atalanta;Vanessa atalanta;1;Vanessa atalanta;0.999999999514898;
KX047683;Lepidoptera;Lasiommata megera;Lasiommata megera;1;Lasiommata megera;0.999890372422668;
KX047716;Lepidoptera;Boloria euphrosyne;Boloria euphrosyne;0.008;Boloria euphrosyne;0.999964281413411;
KX047756;Lepidoptera;Gonepteryx rhamni;NA;0.497;Gonepteryx rhamni;0.980035687766745;
KX047799;Lepidoptera;Cyaniris semiargus;NA;NA;Cyaniris semiargus;0.999051841766953;
KX047808;Lepidoptera;Argynnis paphia;Argynnis paphia;1;Argynnis paphia;0.999999991067568;
KX047828;Lepidoptera;Lycaena phlaeas;Lycaena phlaeas;1;Lycaena phlaeas;0.999999996067032;
KX047857;Lepidoptera;Parnassius apollo;Parnassius apollo;NA;Parnassius apollo;0.99956833515036;
KX047929;Lepidoptera;Boloria euphrosyne;Boloria euphrosyne;0.004;Boloria euphrosyne;0.99997090132607;
KX048020;Lepidoptera;Plebejus argus;NA;0.13;Plebejus argus;0.972634369319029;
KX048053;Lepidoptera;Lycaena virgaureae;NA;0.067;Lycaena virgaureae;0.999579233710412;
KX048063;Lepidoptera;Boloria dia;Boloria dia;1;Boloria dia;0.999999999979764;
KX048159;Lepidoptera;Pieris napi;NA;0.079;Pieris napi;0.829470145096553;
KX048161;Lepidoptera;Anthocharis cardamines;Anthocharis cardamines;1;Anthocharis cardamines;0.999999940675226;
KX048233;Lepidoptera;Parnassius mnemosyne;Parnassius mnemosyne;1;Parnassius mnemosyne;0.999999416965636;
KX048257;Lepidoptera;Carterocephalus palaemon;NA;0.051;Carterocephalus palaemon;0.753998671629088;
KX048275;Lepidoptera;Boloria euphrosyne;Boloria euphrosyne;0.008;Boloria euphrosyne;0.999964281413411;
KX048399;Lepidoptera;Parnassius apollo;Parnassius apollo;NA;Parnassius apollo;0.99956833515036;
KX048426;Lepidoptera;Agriades optilete;Agriades optilete;1;Agriades optilete;0.999999991532263;
KX048470;Lepidoptera;Scolitantides orion;Scolitantides orion;1;Scolitantides orion;0.999999962663367;
KX048480;Lepidoptera;Parnassius apollo;Parnassius apollo;NA;Parnassius apollo;0.99956833515036;
KX048495;Lepidoptera;Erebia pandrose;Erebia pandrose;0.004;Erebia pandrose;0.997336976016485;
KX048500;Lepidoptera;Parnassius apollo;Parnassius apollo;1;Parnassius apollo;0.99966565105155;
KX048509;Lepidoptera;Scolitantides orion;Scolitantides orion;1;Scolitantides orion;0.999999962663367;
KX048523;Lepidoptera;Lycaena tityrus;Lycaena tityrus;1;Lycaena tityrus;0.999998928238034;
KX048601;Lepidoptera;Pieris napi;NA;NA;Pieris napi;0.954337147758801;
KX048626;Lepidoptera;Ochlodes sylvanus;NA;0.138;Ochlodes sylvanus;0.997351494363929;
KX048709;Lepidoptera;Cupido minimus;NA;0.009;Cupido minimus;0.999791453712643;
KX048711;Lepidoptera;Plebejus argyrognomon;NA;0.001;NA;0.994620093546684;
KX048776;Lepidoptera;Aricia nicias;Aricia nicias;1;Aricia nicias;0.999760056790492;
KX048787;Lepidoptera;Colias palaeno;NA;0.002;NA;0.99999999799021;
KX048803;Lepidoptera;Lasiommata megera;Lasiommata megera;1;Lasiommata megera;0.99989806235896;
KX048848;Lepidoptera;Coenonympha arcania;Coenonympha arcania;0.014;Coenonympha arcania;0.986375706914077;

KX048864;Lepidoptera;Aricia eumedon;Eumedonia eumedon;0.002;Eumedonia eumedon;0.998062893323354;
KX048905;Lepidoptera;Aphantopus hyperantus;Aphantopus hyperantus;0.002;Aphantopus hyperantus;0.999999300149444;
KX048958;Lepidoptera;Scolitantides orion;Scolitantides orion;1;Scolitantides orion;0.999999962663367;
KX048972;Lepidoptera;Aricia artaxerxes;Plebejus argus;1;Aricia artaxerxes;0.999743187199922;
KX049178;Lepidoptera;Aricia eumedon;Eumedonia eumedon;1;Eumedonia eumedon;0.998306244105872;
KX049217;Lepidoptera;Pyrgus andromedae;Pyrgus andromedae;1;Pyrgus andromedae;0.999798526295844;
KX049222;Lepidoptera;Glaucopsyche alexis;NA;NA;Glaucopsyche alexis;0.978116759463985;
KX049250;Lepidoptera;Melitaea cinxia;Melitaea cinxia;1;Melitaea cinxia;0.999999999999403;
KX049293;Lepidoptera;Thymelicus sylvestris;NA;0.042;Thymelicus sylvestris;0.9999626292829;
KX049522;Lepidoptera;Erebia pandrose;Erebia pandrose;0.004;Erebia pandrose;0.997336976016485;
KX049579;Lepidoptera;Erebia medusa;NA;0.007;Erebia medusa;0.987140153764329;
KX049658;Lepidoptera;Coenonympha pamphilus;Coenonympha pamphilus;1;Coenonympha pamphilus;0.999999999999744;
KX049723;Lepidoptera;Lycaena phlaeas;Lycaena phlaeas;1;Lycaena phlaeas;0.999999996363186;
KX049735;Lepidoptera;Coenonympha pamphilus;Coenonympha pamphilus;1;Coenonympha pamphilus;0.999999999999886;
KX049777;Lepidoptera;Nymphalis io;NA;0.489;NA;0.99999999999788;
KX049826;Lepidoptera;Plebejus idas;NA;0.001;NA;0.999551141554281;
KX049843;Lepidoptera;Coenonympha pamphilus;Coenonympha pamphilus;1;Coenonympha pamphilus;0.999999999999886;
KX049869;Lepidoptera;Pararge aegeria;Pararge aegeria;1;Pararge aegeria;0.999999999963165;
KX049889;Lepidoptera;Vanessa atalanta;Vanessa atalanta;1;Vanessa atalanta;0.999999999805453;
KX049935;Lepidoptera;Lasiommata megera;Lasiommata megera;1;Lasiommata megera;0.999890372422668;
KX049957;Lepidoptera;Thecla betulae;Thecla betulae;1;Thecla betulae;0.999999999935426;
KX050013;Lepidoptera;Aricia artaxerxes;Plebejus argus;1;Aricia artaxerxes;0.99976599124702;
KX050108;Lepidoptera;Nymphalis io;NA;0.488;Aglais io;0.924256600536712;
KX071222;Lepidoptera;Plebejus argus;NA;0.036;Plebejus argus;0.988160277018267;
KX071243;Lepidoptera;Nymphalis c-album;NA;0.215;NA;0.999999192918557;
KX071306;Lepidoptera;Erebia medusa;NA;0.033;Erebia medusa;0.952039959141035;
KX071376;Lepidoptera;Pyrgus alveus;NA;NA;Pyrgus alveus;0.784892790140425;
KX071442;Lepidoptera;Anthocharis cardamines;Anthocharis cardamines;1;Anthocharis cardamines;0.999999951562672;
KX071706;Lepidoptera;Hipparchia hermione;Hipparchia hermione;1;Hipparchia hermione;0.999965771098254;
KX071761;Lepidoptera;Euphydryas maturna;NA;0.126;Euphydryas maturna;0.885705216462044;
KX071773;Lepidoptera;Nymphalis c-album;NA;0.215;NA;0.999999192918557;
KX071814;Lepidoptera;Hipparchia hermione;NA;NA;NA;0.999999999999999;
KX072019;Lepidoptera;Anthocharis cardamines;Anthocharis cardamines;1;Anthocharis cardamines;0.999999933384063;
KX072125;Lepidoptera;Euphydryas maturna;Euphydryas maturna;1;Euphydryas maturna;0.893551409890867;
KX130682;Lepidoptera;Parnassius mnemosyne;Parnassius mnemosyne;1;NA;0.982385387973762;
KX130683;Lepidoptera;Parnassius mnemosyne;Parnassius mnemosyne;1;NA;0.980300870248416;
KX130684;Lepidoptera;Parnassius mnemosyne;Parnassius mnemosyne;1;NA;0.978273651399484;
KX130685;Lepidoptera;Parnassius mnemosyne;Parnassius mnemosyne;1;NA;0.888574208472778;
KX130686;Lepidoptera;Parnassius mnemosyne;Parnassius mnemosyne;1;NA;0.980588002105575;
KX241493;Lepidoptera;Lycaena dispar;Lycaena dispar;0.005;Lycaena dispar;0.999999999949267;
KX241500;Lepidoptera;Euphydryas aurinia;NA;0.006;Euphydryas aurinia;0.756425138720062;
KX241501;Lepidoptera;Euphydryas provincialis;NA;0.311;Euphydryas provincialis;0.832304786723762;
KX241502;Lepidoptera;Euphydryas provincialis;NA;0.335;Euphydryas aurinia;0.962236452818543;
KX241503;Lepidoptera;Euphydryas provincialis;NA;0.307;Euphydryas aurinia;0.932882591269762;
KX277936;Lepidoptera;Erebia pronoe;Erebia pronoe;1;Erebia pronoe;0.999652510310187;
KX277937;Lepidoptera;Erebia pronoe;Erebia pronoe;1;Erebia pronoe;0.999652510310187;
KX277938;Lepidoptera;Erebia pronoe;Erebia pronoe;0.001;Erebia pronoe;0.999597058595386;
KX277939;Lepidoptera;Erebia pronoe;Erebia pronoe;1;Erebia pronoe;0.999652510310187;
KX277940;Lepidoptera;Erebia pronoe;Erebia pronoe;1;Erebia pronoe;0.999652510310187;
KX277941;Lepidoptera;Erebia pronoe;Erebia pronoe;0.999;Erebia pronoe;0.999433014122848;
KX277942;Lepidoptera;Erebia pronoe;Erebia pronoe;1;Erebia pronoe;0.999652510310187;
KX277943;Lepidoptera;Erebia pronoe;Erebia pronoe;0.999;Erebia pronoe;0.999585863843868;
KX277944;Lepidoptera;Erebia pronoe;Erebia pronoe;1;Erebia pronoe;0.999652510310187;
KX277945;Lepidoptera;Erebia pronoe;Erebia pronoe;1;Erebia pronoe;0.999652510310187;
KX277946;Lepidoptera;Erebia pronoe;Erebia pronoe;1;Erebia pronoe;0.999652510310187;
KX277947;Lepidoptera;Erebia pronoe;Erebia pronoe;0.999;Erebia pronoe;0.999585863843868;
KX277948;Lepidoptera;Erebia pronoe;Erebia pronoe;1;Erebia pronoe;0.999652510310187;
KX277949;Lepidoptera;Erebia pronoe;Erebia pronoe;1;Erebia pronoe;0.999837399753529;
KX277950;Lepidoptera;Erebia pronoe;Erebia pronoe;NA;Erebia pronoe;0.999498950011637;
KX277951;Lepidoptera;Erebia pronoe;Erebia pronoe;1;Erebia pronoe;0.999813984712597;
KX277952;Lepidoptera;Erebia pronoe;Erebia pronoe;1;Erebia pronoe;0.999828527312903;
KX277953;Lepidoptera;Erebia pronoe;Erebia pronoe;1;Erebia pronoe;0.99991290589748;
KX277954;Lepidoptera;Erebia pronoe;Erebia pronoe;1;Erebia pronoe;0.999868410095852;
KX277955;Lepidoptera;Erebia pronoe;Erebia pronoe;1;Erebia pronoe;0.99942306672371;
KX277956;Lepidoptera;Erebia pronoe;Erebia pronoe;1;Erebia pronoe;0.999902522806916;
KX277957;Lepidoptera;Erebia pronoe;Erebia pronoe;1;Erebia pronoe;0.999851167246661;
KX277958;Lepidoptera;Erebia pronoe;Erebia pronoe;1;Erebia pronoe;0.999807306062552;
KX277959;Lepidoptera;Erebia pronoe;Erebia pronoe;1;Erebia pronoe;0.999914728208209;
KX277960;Lepidoptera;Erebia pronoe;Erebia pronoe;1;Erebia pronoe;0.999607121951299;

KX277961;Lepidoptera;Erebia pronoe;Erebia pronoe;1;Erebia pronoe;0.999851167246661;
KX277962;Lepidoptera;Erebia pronoe;Erebia pronoe;1;Erebia pronoe;0.999856083117195;
KX377635;Lepidoptera;Polyommatus damon;Polyommatus damon;1;Polyommatus damon;0.999999794290794;
KX951944;Lepidoptera;Polyommatus celina;NA;0.075;Polyommatus celina;0.999999999646178;
KY000505;Lepidoptera;Satyrus ferula;Satyrus ferula;0.988;Satyrus ferula;0.993221685107162;
KY000506;Lepidoptera;Satyrus ferula;Satyrus ferula;0.001;Satyrus ferula;0.992881646523949;
KY000507;Lepidoptera;Satyrus ferula;NA;0.001;Satyrus ferula;0.963611208448215;
KY000508;Lepidoptera;Satyrus ferula;NA;0.003;Satyrus ferula;0.984337909759304;
KY000509;Lepidoptera;Satyrus ferula;Satyrus ferula;0.001;Satyrus ferula;0.993531109275661;
KY000510;Lepidoptera;Satyrus ferula;Satyrus ferula;0.001;Satyrus ferula;0.985590051567424;
KY000511;Lepidoptera;Satyrus ferula;NA;0.012;Satyrus ferula;0.974869552654809;
KY000512;Lepidoptera;Satyrus ferula;Satyrus ferula;0.001;Satyrus ferula;0.988785780171169;
KY066730;Lepidoptera;Polyommatus damon;Polyommatus damon;1;Polyommatus damon;0.99999996733564;
KY066731;Lepidoptera;Polyommatus damon;Polyommatus damon;1;Polyommatus damon;0.999999983729595;
KY112814;Lepidoptera;Melitaea ornata;NA;NA;NA;0.91981127253977;
KY112815;Lepidoptera;Melitaea ornata;NA;NA;NA;0.91981127253977;
KY112816;Lepidoptera;Melitaea ornata;NA;NA;NA;0.91981127253977;
KY112817;Lepidoptera;Melitaea ornata;NA;NA;NA;0.91981127253977;
KY112818;Lepidoptera;Melitaea ornata;NA;NA;NA;0.90855109807123;
KY112819;Lepidoptera;Melitaea ornata;NA;NA;NA;0.915231925209144;
KY112820;Lepidoptera;Melitaea ornata;NA;NA;NA;0.915231925209144;
KY112821;Lepidoptera;Melitaea ornata;NA;NA;NA;0.91981127253977;
KY112822;Lepidoptera;Melitaea ornata;NA;NA;NA;0.90855109807123;
KY112823;Lepidoptera;Melitaea ornata;NA;NA;NA;0.90855109807123;
KY112825;Lepidoptera;Melitaea ornata;NA;NA;NA;0.911459175804093;
KY112826;Lepidoptera;Melitaea ornata;NA;NA;NA;0.916096871865532;
KY112827;Lepidoptera;Melitaea ornata;NA;0.027;NA;0.901087625782827;
KY112828;Lepidoptera;Melitaea ornata;NA;0.027;NA;0.901087625782827;
KY112829;Lepidoptera;Melitaea ornata;NA;0.027;NA;0.901087625782827;
KY112830;Lepidoptera;Melitaea ornata;NA;0.027;NA;0.901087625782827;
KY128336;Lepidoptera;Aglais ichnusa;Aglais ichnusa;0.996;NA;0.999999994680505;
KY128337;Lepidoptera;Nymphalis io;NA;0.487;NA;0.812507864455466;
KY128341;Lepidoptera;Aglais urticae;Aglais urticae;0.001;Aglais nixa;0.98024379709347;
KY128345;Lepidoptera;Nymphalis antiopa;NA;0.114;Nymphalis antiopa;0.999999904320139;
KY128346;Lepidoptera;Nymphalis antiopa;Nymphalis antiopa;0.031;Nymphalis antiopa;0.999999964251004;
KY569451;Lepidoptera;Erebia pronoe;Erebia pronoe;1;Erebia pronoe;0.999586503683627;
KY569452;Lepidoptera;Erebia pronoe;Erebia pronoe;1;Erebia pronoe;0.999586503683627;
KY569453;Lepidoptera;Erebia pronoe;Erebia pronoe;1;Erebia pronoe;0.999586503683627;
KY569454;Lepidoptera;Erebia pronoe;Erebia pronoe;1;Erebia pronoe;0.999586503683627;
KY569455;Lepidoptera;Erebia pronoe;Erebia pronoe;1;Erebia pronoe;0.999586503683627;
KY569456;Lepidoptera;Erebia pronoe;Erebia pronoe;1;Erebia pronoe;0.999849627513594;
KY569457;Lepidoptera;Erebia pronoe;Erebia pronoe;1;Erebia pronoe;0.999586503683627;
KY569458;Lepidoptera;Erebia pronoe;Erebia pronoe;1;Erebia pronoe;0.999586503683627;
LC155510;Lepidoptera;Oeneis glacialis;Oeneis glacialis;NA;NA;0.999999999999959;
LC340450;Lepidoptera;Erebia euryale;NA;NA;Erebia euryale;0.869647226981521;
LC340453;Lepidoptera;Erebia epiphron;NA;0.048;Erebia epiphron;0.984236140438357;
LC340455;Lepidoptera;Erebia manto;NA;0.112;Erebia manto;0.994130514247749;
LC340456;Lepidoptera;Erebia mnestra;Erebia mnestra;0.997;Erebia mnestra;0.999829234762559;
LC340457;Lepidoptera;Erebia montana;Erebia montana;1;Erebia montana;0.997503353757874;
LC340458;Lepidoptera;Erebia nivalis;NA;0.001;NA;0.999999999999821;
LC340459;Lepidoptera;Erebia oeme;NA;0.11;Erebia oeme;0.999999999958959;
LC340460;Lepidoptera;Erebia pharte;Erebia pharte;1;Erebia pharte;0.999999995286061;
LC340461;Lepidoptera;Erebia pronoe;NA;NA;Erebia pronoe;0.835310357693488;
LC340462;Lepidoptera;Erebia tyndarus;NA;0.011;NA;0.999999999999136;
LC340463;Lepidoptera;Erebia melampus;NA;0.042;Erebia melampus;0.944389887194667;
LC340478;Lepidoptera;Erebia euryale;NA;NA;Erebia euryale;0.869647226981521;
LC340481;Lepidoptera;Erebia tyndarus;NA;0.001;NA;0.999999999999841;
LC340484;Lepidoptera;Erebia neoridas;Erebia neoridas;NA;Erebia neoridas;0.994364286581965;
LC340485;Lepidoptera;Erebia ottomana;Erebia ottomana;1;Erebia ottomana;0.999999988838795;
LC340496;Lepidoptera;Erebia triaria;NA;0.296;NA;0.999999999999993;
LC340498;Lepidoptera;Erebia pandrose;Erebia pandrose;0.005;Erebia pandrose;0.997196148347696;
LC340526;Lepidoptera;Erebia pluto;Erebia pluto;1;Erebia pluto;0.999999771864933;
LC340535;Lepidoptera;Erebia meolans;Erebia meolans;0.01;Erebia meolans;0.99940279413839;
LC340552;Lepidoptera;Erebia styx;Erebia styx;0.002;Erebia styx;0.971927006399762;
LC340554;Lepidoptera;Erebia pandrose;Erebia pandrose;0.005;Erebia pandrose;0.998473564469949;
LC340559;Lepidoptera;Erebia scipio;Erebia scipio;1;Erebia scipio;0.999999992333755;
LC340564;Lepidoptera;Erebia medusa;NA;0.012;Erebia medusa;0.970383261328537;
LC340565;Lepidoptera;Erebia eriphyle;Erebia eriphyle;1;Erebia eriphyle;0.99999992267752;
LC340566;Lepidoptera;Erebia stirius;NA;0.001;NA;0.999999999784577;
LC340567;Lepidoptera;Erebia christi;Erebia christi;1;Erebia christi;0.999999999964643;
LC471678;Lepidoptera;Anthocharis cardamines;Anthocharis cardamines;0.974;Anthocharis cardamines;0.999995816682112;
LC471679;Lepidoptera;Anthocharis cardamines;Anthocharis cardamines;0.979;Anthocharis cardamines;0.99999858536342;
LC471680;Lepidoptera;Anthocharis cardamines;Anthocharis cardamines;0.002;Anthocharis cardamines;0.999996829877341;

LC471681;Lepidoptera;Anthocharis cardamines;Anthocharis cardamines;0.001;Anthocharis cardamines;0.999991670577019;
LC471682;Lepidoptera;Anthocharis cardamines;NA;0.001;Anthocharis cardamines;0.999995060166591;
LC471683;Lepidoptera;Anthocharis cardamines;Anthocharis cardamines;0.002;Anthocharis cardamines;0.99997643130059;
LC471684;Lepidoptera;Anthocharis cardamines;Anthocharis cardamines;0.995;Anthocharis cardamines;0.999997328760899;
LC471689;Lepidoptera;Colias palaeno;NA;NA;NA;0.999999999831314;
LC471692;Lepidoptera;Colias palaeno;NA;NA;NA;0.999999999831314;
LC471701;Lepidoptera;Aglais urticae;NA;0.001;Aglais urticae;0.761522218140768;
LC471702;Lepidoptera;Aglais urticae;NA;NA;NA;0.999999997238419;
LC471703;Lepidoptera;Aglais urticae;NA;0.006;NA;0.999999998205741;
LC471710;Lepidoptera;Carterocephalus palaemon;NA;0.046;NA;0.999999999424184;
LC471711;Lepidoptera;Carterocephalus palaemon;NA;0.01;NA;0.999999993667149;
LC471712;Lepidoptera;Carterocephalus palaemon;Carterocephalus palaemon;0.01;Carterocephalus palaemon;0.739646793110812;
LC471714;Lepidoptera;Carterocephalus palaemon;NA;0.009;NA;0.999999996135473;
LC507810;Lepidoptera;Aporia crataegi;NA;NA;Aporia crataegi;0.998009684501193;
LEASS1001-17;Lepidoptera;Brenthis daphne;NA;0.183;Brenthis daphne;0.828231658877439;
LEASS1010-17;Lepidoptera;Melitaea phoebe;NA;0.002;Melitaea phoebe;0.809634755947941;
LEASS1025-17;Lepidoptera;Thecla betulae;Thecla betulae;1;Thecla betulae;0.99991876000087;
LEASS1031-17;Lepidoptera;Satyrium ilicis;NA;0.183;Satyrium ilicis;0.996615181211911;
LEASS1040-17;Lepidoptera;Polyommatus daphnis;NA;0.049;Polyommatus daphnis;0.961194861034674;
LEASS1041-17;Lepidoptera;Polyommatus daphnis;NA;0.049;Polyommatus daphnis;0.961194861034674;
LEASS537-17;Lepidoptera;Polyommatus dorylas;NA;NA;Polyommatus dorylas;0.989832543295548;
LEASS705-17;Lepidoptera;Carcharodus alceae;NA;NA;Carcharodus alceae;0.999941172693733;
LEASS890-17;Lepidoptera;Limenitis camilla;Limenitis camilla;1;Limenitis camilla;0.999906475273725;
LEASS905-17;Lepidoptera;Satyrium ilicis;NA;0.196;Satyrium ilicis;0.995167698244269;
LEASS912-17;Lepidoptera;Polyommatus amandus;Polyommatus amandus;0.023;Polyommatus amandus;0.999915179944342;
LEASS913-17;Lepidoptera;Polyommatus dorylas;NA;0.037;Polyommatus dorylas;0.81501858849305;
LEASS921-17;Lepidoptera;Carcharodus alceae;NA;NA;Carcharodus alceae;0.999969715441795;
LEASS965-17;Lepidoptera;Colias hyale;NA;0.003;Colias hyale;0.999739891795678;
LEASS979-17;Lepidoptera;Cupido argiades;NA;0.002;NA;0.999999409969707;
LEASS984-17;Lepidoptera;Hipparchia fagi;NA;NA;Hipparchia fagi;0.732661841087221;
LEASS991-17;Lepidoptera;Oeneis glacialis;Oeneis glacialis;NA;Oeneis glacialis;0.97674082091352;
LEATG401-14;Lepidoptera;Carcharodus lavatherae;NA;0.146;Carcharodus lavatherae;0.997727542590474;
LEATG422-14;Lepidoptera;Celastrina argiolus;Celastrina argiolus;NA;Celastrina argiolus;0.998081449161059;
LEATG430-14;Lepidoptera;Scolitantides orion;Scolitantides orion;1;Scolitantides orion;0.999999972689921;
LEATG450-14;Lepidoptera;Melitaea didyma;Melitaea didyma;NA;Melitaea didyma;0.997888427653926;
LEATG471-14;Lepidoptera;Cupido alcetas;NA;0.184;Cupido alcetas;0.836771345436072;
LEATG476-14;Lepidoptera;Polyommatus coridon;Polyommatus coridon;1;Polyommatus coridon;0.999999764885927;
LEATG481-14;Lepidoptera;Iolana iolas;Iolana iolas;1;Iolana iolas;0.999988730040341;
LEATG486-14;Lepidoptera;Lampides boeticus;Lampides boeticus;NA;Lampides boeticus;0.999998765013184;
LEATG488-14;Lepidoptera;Polyommatus thersites;NA;0.046;Polyommatus thersites;0.999993261717002;
LEATG509-14;Lepidoptera;Lasiommata megera;Lasiommata megera;0.019;Lasiommata megera;0.996962677013203;
LEATG510-14;Lepidoptera;Lasiommata megera;Lasiommata megera;0.001;Lasiommata megera;0.99561575060372;
LEATG528-14;Lepidoptera;Colias hyale;NA;0.103;Colias hyale;0.999989328684744;
LEPMT010-22;Lepidoptera;Polyommatus celina;Polyommatus celina;0.963;Polyommatus celina;0.99999960735365;
LEPMT011-22;Lepidoptera;Polyommatus celina;Polyommatus celina;0.002;Polyommatus celina;0.999999885384632;
LEPMT012-22;Lepidoptera;Polyommatus celina;Polyommatus celina;0.02;Polyommatus celina;0.999999999277776;
LEPMT013-22;Lepidoptera;Polyommatus celina;Polyommatus celina;0.009;Polyommatus celina;0.999999999421163;
LEPMT015-22;Lepidoptera;Polyommatus celina;NA;0.076;Polyommatus celina;0.999999999607354;
LEPMT016-22;Lepidoptera;Polyommatus celina;Polyommatus celina;0.001;Polyommatus celina;0.99999998649551;
LEPMT017-22;Lepidoptera;Polyommatus celina;NA;0.017;Polyommatus celina;0.999999999020844;
LEPMT019-22;Lepidoptera;Polyommatus celina;Polyommatus celina;0.012;Polyommatus celina;0.99999999885614;
LEPMT020-22;Lepidoptera;Polyommatus celina;NA;0.17;Polyommatus celina;0.99999999819812;
LEPMT023-22;Lepidoptera;Polyommatus celina;NA;0.004;Polyommatus celina;0.999999999006064;
LEPPK089-13;Lepidoptera;Apatura iris;Apatura iris;1;Apatura iris;0.999999999999744;
LON1068-12;Lepidoptera;Coenonympha pamphilus;Coenonympha pamphilus;1;Coenonympha pamphilus;0.999999999999886;
LON1079-12;Lepidoptera;Ochlodes sylvanus;NA;0.138;Ochlodes sylvanus;0.997351494363929;
LON1097-12;Lepidoptera;Satyrium w-album;NA;0.056;Satyrium w-album;0.989378230996316;
LON1107-12;Lepidoptera;Melitaea cinxia;Melitaea cinxia;1;Melitaea cinxia;0.999999999999403;

LON1124-12;Lepidoptera;Aphantopus hyperantus;Aphantopus hyperantus;0.002;Aphantopus hyperantus;0.999999300149444;
LON1125-12;Lepidoptera;Brenthis ino;Brenthis ino;0.001;Brenthis ino;0.998434780405276;
LON1185-14;Lepidoptera;Colias palaeno;NA;0.002;NA;0.999999999983681;
LON1257-14;Lepidoptera;Colias palaeno;NA;0.002;NA;0.999999999983681;
LON1313-14;Lepidoptera;Plebejus idas;NA;0.007;NA;0.999546324331549;
LON2681-16;Lepidoptera;Parnassius apollo;Parnassius apollo;NA;Parnassius apollo;0.99956833515036;
LON2691-16;Lepidoptera;Aglais urticae;Aglais urticae;0.001;Aglais urticae;0.994966452807995;
LON2711-16;Lepidoptera;Coenonympha pamphilus;Coenonympha pamphilus;1;Coenonympha pamphilus;0.999999999999886;
LON2714-16;Lepidoptera;Anthocharis cardamines;Anthocharis cardamines;1;Anthocharis cardamines;0.999999940675226;
LON2716-16;Lepidoptera;Pieris napi;NA;0.013;Pieris napi;0.798118615202961;
LON2740-16;Lepidoptera;Parnassius mnemosyne;Parnassius mnemosyne;1;Parnassius mnemosyne;0.999999416965636;
LON2741-16;Lepidoptera;Parnassius apollo;Parnassius apollo;NA;Parnassius apollo;0.99956833515036;
LON4305-16;Lepidoptera;Glaucopsyche alexis;NA;NA;Glaucopsyche alexis;0.979381169624069;
LON4501-16;Lepidoptera;Aphantopus hyperantus;Aphantopus hyperantus;0.002;Aphantopus hyperantus;0.999999300149444;
LON4526-16;Lepidoptera;Melitaea diamina;Melitaea diamina;0.021;Melitaea diamina;0.999999995493909;
LON4527-16;Lepidoptera;Lasiommata maera;Lasiommata maera;1;Lasiommata maera;0.999996420657379;
LON4528-16;Lepidoptera;Lasiommata maera;Lasiommata maera;1;Lasiommata maera;0.99999942908402;
LON4576-16;Lepidoptera;Colias palaeno;NA;0.002;NA;0.999999999983681;
LON4620-16;Lepidoptera;Pyrgus malvae;Pyrgus malvae;0.005;Pyrgus malvae;0.989643682630206;
LON4624-16;Lepidoptera;Cupido minimus;NA;0.009;Cupido minimus;0.999791453712643;
LON4628-16;Lepidoptera;Glaucopsyche alexis;NA;NA;Glaucopsyche alexis;0.979381169624069;
LON4629-16;Lepidoptera;Argynnis aglaja;NA;NA;NA;0.999999999998051;
LON4630-16;Lepidoptera;Boloria euphrosyne;Boloria euphrosyne;1;Boloria euphrosyne;0.999988204772551;
LON4634-16;Lepidoptera;Aphantopus hyperantus;Aphantopus hyperantus;0.002;Aphantopus hyperantus;0.999999300149444;
LON4635-16;Lepidoptera;Aphantopus hyperantus;Aphantopus hyperantus;0.002;Aphantopus hyperantus;0.999999300149444;
LON5642-17;Lepidoptera;Polygonia c-album;NA;0.215;NA;0.999999192918557;
LON5646-17;Lepidoptera;Hamearis lucina;Hamearis lucina;NA;Hamearis lucina;0.999999998835278;
LON5647-17;Lepidoptera;Celastrina argiolus;Celastrina argiolus;1;Celastrina argiolus;0.999708846309046;
LON5651-17;Lepidoptera;Lasiommata maera;Lasiommata maera;1;Lasiommata maera;0.99999942908402;
LON5652-17;Lepidoptera;Lasiommata maera;Lasiommata maera;1;Lasiommata maera;0.99999942908402;
LON5653-17;Lepidoptera;Lasiommata maera;Lasiommata maera;1;Lasiommata maera;0.99999942908402;
LON5663-17;Lepidoptera;Argynnis aglaja;NA;0.005;NA;0.999999999994961;
LON5664-17;Lepidoptera;Argynnis aglaja;NA;NA;NA;0.999999999998051;
LON5668-17;Lepidoptera;Favonius quercus;NA;NA;Favonius quercus;0.73046069582187;
LON5669-17;Lepidoptera;Favonius quercus;NA;NA;Favonius quercus;0.73046069582187;
LON5670-17;Lepidoptera;Callophrys rubi;NA;0.007;Callophrys rubi;0.997836671768354;
LON5671-17;Lepidoptera;Callophrys rubi;NA;0.003;Callophrys rubi;0.997328639918919;
LON5673-17;Lepidoptera;Lycaena hippothoe;NA;NA;NA;0.999999468478795;
LON5676-17;Lepidoptera;Limenitis populi;Limenitis populi;1;Limenitis populi;0.999999999859654;
LON5685-17;Lepidoptera;Pyrgus malvae;Pyrgus malvae;NA;Pyrgus malvae;0.980121140374433;
LON5686-17;Lepidoptera;Pyrgus malvae;Pyrgus malvae;NA;Pyrgus malvae;0.980121140374433;
LON5687-17;Lepidoptera;Pyrgus malvae;Pyrgus malvae;NA;Pyrgus malvae;0.980121140374433;
LON5688-17;Lepidoptera;Pyrgus alveus;NA;NA;Pyrgus alveus;0.922887984165083;
LON5722-17;Lepidoptera;Colias palaeno;NA;0.002;NA;0.999999999983681;
LON6142-17;Lepidoptera;Maniola jurtina;NA;0.038;NA;0.999999999999524;
LON6143-17;Lepidoptera;Maniola jurtina;NA;NA;NA;0.99999999985062;
LON6407-17;Lepidoptera;Parnassius apollo;Parnassius apollo;NA;Parnassius apollo;0.99956833515036;
LON6412-17;Lepidoptera;Ochlodes sylvanus;NA;0.316;Ochlodes sylvanus;0.996318754838625;
LON6413-17;Lepidoptera;Pararge aegeria;Pararge aegeria;1;Pararge aegeria;0.999999999963165;
LON6434-17;Lepidoptera;Parnassius mnemosyne;Parnassius mnemosyne;1;Parnassius mnemosyne;0.999999416965636;
LON6435-17;Lepidoptera;Parnassius mnemosyne;Parnassius mnemosyne;1;Parnassius mnemosyne;0.999999416965636;
LON6575-17;Lepidoptera;Aricia artaxerxes;Plebejus argus;1;Aricia artaxerxes;0.999743187199922;
LON6811-18;Lepidoptera;Aricia artaxerxes;Plebejus argus;1;Aricia artaxerxes;0.999743187199922;
LON6812-18;Lepidoptera;Parnassius mnemosyne;Parnassius mnemosyne;1;Parnassius mnemosyne;0.999999416965636;
LON6813-18;Lepidoptera;Parnassius mnemosyne;Parnassius mnemosyne;1;Parnassius mnemosyne;0.999999416965636;
LON6827-18;Lepidoptera;Maniola jurtina;NA;NA;NA;0.99999999985062;
LON6829-18;Lepidoptera;Maniola jurtina;NA;0.038;NA;0.999999999999524;
LON6830-18;Lepidoptera;Maniola jurtina;NA;0.038;NA;0.999999999999524;
LON6831-18;Lepidoptera;Maniola jurtina;NA;NA;NA;0.99999999985062;
LON7245-18;Lepidoptera;Pyrgus malvae;Pyrgus malvae;0.999;Pyrgus malvae;0.993531783407179;
LON7246-18;Lepidoptera;Pyrgus malvae;Pyrgus malvae;0.001;Pyrgus malvae;0.996288466450643;

LON7247-18;Lepidoptera;Pyrgus malvae;Pyrgus malvae;0.999;Pyrgus malvae;0.993531783407179;
LON7262-18;Lepidoptera;Polyommatus amandus;Polyommatus amandus;0.007;Polyommatus amandus;0.999944246955852;
LON7263-18;Lepidoptera;Melitaea cinxia;Melitaea cinxia;1;Melitaea cinxia;0.999999999999574;
LON7264-18;Lepidoptera;Pyrgus armoricanus;Pyrgus armoricanus;1;Pyrgus armoricanus;0.997478680096412;
LON7266-18;Lepidoptera;Pyrgus malvae;Pyrgus malvae;0.999;Pyrgus malvae;0.993531783407179;
LON7267-18;Lepidoptera;Pyrgus carthami;Pyrgus carthami;1;Pyrgus carthami;0.99999339129648;
LON7268-18;Lepidoptera;Pyrgus malvae;Pyrgus malvae;0.999;Pyrgus malvae;0.993531783407179;
LON7271-18;Lepidoptera;Melitaea didyma;Melitaea didyma;1;Melitaea didyma;0.992165146696479;
LON7273-18;Lepidoptera;Erebia medusa;NA;0.015;Erebia medusa;0.987529305377852;
LOWAB166-09;Lepidoptera;Aricia artaxerxes;NA;0.069;Aricia artaxerxes;0.998975227462761;
LOWAB167-09;Lepidoptera;Polyommatus icarus;NA;0.002;Polyommatus icarus;0.997043403297368;
LOWAB170-09;Lepidoptera;Polyommatus damon;Polyommatus damon;1;Polyommatus damon;0.999999887140439;
LOWAB171-09;Lepidoptera;Aricia artaxerxes;Aricia artaxerxes;0.013;Aricia artaxerxes;0.998150484524344;
LOWAB172-09;Lepidoptera;Polyommatus damon;Polyommatus damon;1;Polyommatus damon;0.999999887140439;
LOWAB173-09;Lepidoptera;Aricia artaxerxes;NA;0.069;Aricia artaxerxes;0.998975227462761;
LOWAB176-09;Lepidoptera;Aricia artaxerxes;Aricia artaxerxes;0.017;Aricia artaxerxes;0.999222547145315;
LOWAB177-09;Lepidoptera;Aricia artaxerxes;Aricia artaxerxes;0.017;Aricia artaxerxes;0.999222547145315;
LOWAB180-09;Lepidoptera;Polyommatus daphnis;NA;0.033;Polyommatus daphnis;0.995567728273577;
LOWAB182-09;Lepidoptera;Aricia artaxerxes;NA;0.069;Aricia artaxerxes;0.998975227462761;
LOWAB183-09;Lepidoptera;Polyommatus damon;Polyommatus damon;1;Polyommatus damon;0.999999876500006;
LOWAB185-09;Lepidoptera;Polyommatus daphnis;NA;0.001;Polyommatus daphnis;0.961407528082872;
LOWAB187-09;Lepidoptera;Polyommatus daphnis;NA;0.33;Polyommatus thersites;0.999999477278829;
LOWAB188-09;Lepidoptera;Polyommatus daphnis;Polyommatus daphnis;0.017;Polyommatus daphnis;0.987159579554665;
LOWAB189-09;Lepidoptera;Polyommatus damon;Polyommatus damon;1;Polyommatus damon;0.999999887140439;
LOWAB191-09;Lepidoptera;Plebejus argus;NA;0.001;NA;0.999346672923103;
LOWAB193-09;Lepidoptera;Cyaniris semiargus;NA;NA;Cyaniris semiargus;0.999673907732824;
LOWAB196-09;Lepidoptera;Polyommatus daphnis;Polyommatus daphnis;0.01;Polyommatus daphnis;0.989729743734613;
LOWAB203-09;Lepidoptera;Polyommatus damon;Polyommatus damon;1;Polyommatus damon;0.999999949118207;
LOWAB204-09;Lepidoptera;Aricia artaxerxes;NA;0.069;Aricia artaxerxes;0.998975227462761;
LOWAB209-09;Lepidoptera;Thymelicus lineola;NA;0.324;Thymelicus sylvestris;0.999940778442454;
LOWAB216-09;Lepidoptera;Polyommatus daphnis;Polyommatus daphnis;0.017;Polyommatus daphnis;0.987159579554665;
LOWAB220-09;Lepidoptera;Polyommatus daphnis;Polyommatus daphnis;0.01;Polyommatus daphnis;0.989729743734613;
LOWAB221-09;Lepidoptera;Polyommatus damon;Polyommatus damon;1;Polyommatus damon;0.999999947147872;
LOWAB224-09;Lepidoptera;Polyommatus daphnis;Polyommatus daphnis;0.011;Polyommatus daphnis;0.952134006498833;
LOWAB225-09;Lepidoptera;Polyommatus daphnis;Polyommatus daphnis;0.01;Polyommatus daphnis;0.989729743734613;
LOWAB236-09;Lepidoptera;Issoria lathonia;Issoria lathonia;1;Issoria lathonia;0.999999999962967;
LOWAB237-09;Lepidoptera;Arethusana arethusa;Arethusana arethusa;0.008;Arethusana arethusa;0.999986743724591;
LOWAB238-09;Lepidoptera;Arethusana arethusa;Arethusana arethusa;0.008;Arethusana arethusa;0.999986743724591;
LOWAB241-09;Lepidoptera;Arethusana arethusa;Arethusana arethusa;0.008;Arethusana arethusa;0.999986743724591;
LOWAB273-09;Lepidoptera;Arethusana arethusa;Arethusana arethusa;0.01;Arethusana arethusa;0.999980185447415;
LOWAB278-09;Lepidoptera;Argynnis paphia;Argynnis paphia;1;Argynnis paphia;0.999999977920595;
LOWAB280-09;Lepidoptera;Lasiommata megera;Lasiommata megera;0.006;Lasiommata megera;0.992588117195958;
LOWAB283-09;Lepidoptera;Aricia artaxerxes;NA;0.069;Aricia artaxerxes;0.998975227462761;
LOWAB284-09;Lepidoptera;Aricia artaxerxes;NA;0.069;Aricia artaxerxes;0.998975227462761;
LOWAB286-09;Lepidoptera;Thymelicus lineola;Thymelicus lineola;1;Thymelicus lineola;0.999948200943492;
LOWAB287-09;Lepidoptera;Polyommatus icarus;NA;0.002;Polyommatus icarus;0.997043403297368;
LR990102.1;Lepidoptera;Erynnis tages;Erynnis tages;1;Erynnis tages;0.999999281987018;
LR990279.1;Lepidoptera;Aricia agestis;NA;0.081;Aricia agestis;0.998857812762894;
LR990581.1;Lepidoptera;Vanessa atalanta;Vanessa atalanta;1;Vanessa atalanta;0.999999998834312;
LR990920.1;Lepidoptera;Pararge aegeria;Pararge aegeria;1;Pararge aegeria;0.999999999963165;
LR994570.1;Lepidoptera;Cyaniris semiargus;NA;NA;Cyaniris semiargus;0.999340901980295;
LT219471;Lepidoptera;Agriades optilete;Agriades optilete;1;Agriades optilete;0.999999921887539;
LT219472;Lepidoptera;Agriades optilete;Agriades optilete;1;Agriades optilete;0.999999964347467;
LT219473;Lepidoptera;Agriades optilete;Agriades optilete;1;Agriades optilete;0.999999964347467;
LT219474;Lepidoptera;Agriades optilete;Agriades optilete;1;Agriades optilete;0.999999964347467;

LT219475;Lepidoptera;Agriades optilete;Agriades optilete;1;Agriades optilete;0.999999964347467;
LT219476;Lepidoptera;Agriades optilete;Agriades optilete;1;Agriades optilete;0.999999964347467;
LT219477;Lepidoptera;Agriades optilete;Agriades optilete;1;Agriades optilete;0.999999964347467;
LT219478;Lepidoptera;Agriades optilete;Agriades optilete;1;Agriades optilete;0.999999964347467;
LT219479;Lepidoptera;Agriades optilete;Agriades optilete;1;Agriades optilete;0.999999964347467;
LT219480;Lepidoptera;Agriades optilete;Agriades optilete;1;Agriades optilete;0.999999964347467;
LT219481;Lepidoptera;Agriades optilete;Agriades optilete;1;Agriades optilete;0.999999964347467;
LT219482;Lepidoptera;Agriades optilete;Agriades optilete;1;Agriades optilete;0.999999964347467;
LT219483;Lepidoptera;Agriades optilete;Agriades optilete;1;Agriades optilete;0.999999964347467;
LT219484;Lepidoptera;Agriades optilete;Agriades optilete;1;Agriades optilete;0.999999964347467;
LT219485;Lepidoptera;Agriades optilete;Agriades optilete;1;Agriades optilete;0.999999964347467;
LT219486;Lepidoptera;Agriades optilete;Agriades optilete;1;Agriades optilete;0.999999992866407;
LT219487;Lepidoptera;Agriades optilete;Agriades optilete;1;Agriades optilete;0.999999963075738;
LT219488;Lepidoptera;Agriades optilete;Agriades optilete;1;Agriades optilete;0.999999964347467;
LT219489;Lepidoptera;Agriades optilete;Agriades optilete;1;Agriades optilete;0.999999964347467;
LT219490;Lepidoptera;Agriades optilete;Agriades optilete;1;Agriades optilete;0.999999991168806;
LT219491;Lepidoptera;Agriades optilete;Agriades optilete;1;Agriades optilete;0.999999940941509;
LT219492;Lepidoptera;Agriades optilete;Agriades optilete;1;Agriades optilete;0.999999940941509;
LT219493;Lepidoptera;Agriades optilete;Agriades optilete;1;Agriades optilete;0.999999940941509;
LT219494;Lepidoptera;Agriades optilete;Agriades optilete;1;Agriades optilete;0.999999940941509;
LT219495;Lepidoptera;Agriades optilete;Agriades optilete;1;Agriades optilete;0.999999940941509;
LT219496;Lepidoptera;Agriades optilete;Agriades optilete;1;Agriades optilete;0.999999940941509;
LT219497;Lepidoptera;Agriades optilete;Agriades optilete;1;Agriades optilete;0.999999964347467;
LT219498;Lepidoptera;Agriades optilete;Agriades optilete;1;Agriades optilete;0.999999964347467;
LT219499;Lepidoptera;Agriades optilete;Agriades optilete;1;Agriades optilete;0.999999964347467;
LT219500;Lepidoptera;Agriades optilete;Agriades optilete;1;Agriades optilete;0.999999964347467;
LT219501;Lepidoptera;Agriades optilete;Agriades optilete;1;Agriades optilete;0.999999964347467;
LT219502;Lepidoptera;Agriades optilete;Agriades optilete;1;Agriades optilete;0.999999964347467;
LT219503;Lepidoptera;Agriades optilete;Agriades optilete;1;Agriades optilete;0.999999964347467;
LT219504;Lepidoptera;Agriades optilete;Agriades optilete;1;Agriades optilete;0.999999967927693;
LT219505;Lepidoptera;Agriades optilete;Agriades optilete;1;Agriades optilete;0.999999967927693;
LT219506;Lepidoptera;Agriades optilete;Agriades optilete;1;Agriades optilete;0.999999964347467;
LT219507;Lepidoptera;Agriades optilete;Agriades optilete;1;Agriades optilete;0.999999964347467;
LT219508;Lepidoptera;Agriades optilete;Agriades optilete;1;Agriades optilete;0.999999964347467;
LT219509;Lepidoptera;Agriades optilete;Agriades optilete;1;Agriades optilete;0.999999964347467;
LT219510;Lepidoptera;Agriades optilete;Agriades optilete;1;Agriades optilete;0.999999964347467;
LT219511;Lepidoptera;Agriades optilete;Agriades optilete;1;Agriades optilete;0.999999964347467;
LT219512;Lepidoptera;Agriades optilete;Agriades optilete;1;Agriades optilete;0.999999964347467;
LT219513;Lepidoptera;Agriades optilete;Agriades optilete;1;Agriades optilete;0.999999964347467;
LT219514;Lepidoptera;Agriades optilete;Agriades optilete;1;Agriades optilete;0.999999947321727;
LT219515;Lepidoptera;Agriades optilete;Agriades optilete;1;Agriades optilete;0.999999964347467;
LT219516;Lepidoptera;Agriades optilete;Agriades optilete;1;Agriades optilete;0.999999964347467;
LT219517;Lepidoptera;Agriades optilete;Agriades optilete;1;Agriades optilete;0.999999964347467;
LT219518;Lepidoptera;Agriades optilete;Agriades optilete;1;Agriades optilete;0.999999964347467;
LT219519;Lepidoptera;Agriades optilete;Agriades optilete;1;Agriades optilete;0.999999964347467;
LT219520;Lepidoptera;Agriades optilete;Agriades optilete;1;Agriades optilete;0.999999964347467;
LT219521;Lepidoptera;Agriades optilete;Agriades optilete;1;Agriades optilete;0.999999964347467;
LT219522;Lepidoptera;Agriades optilete;Agriades optilete;1;Agriades optilete;0.999999964347467;
LT219523;Lepidoptera;Agriades optilete;Agriades optilete;1;Agriades optilete;0.999999888325312;
LT219524;Lepidoptera;Agriades optilete;Agriades optilete;1;Agriades optilete;0.999999964347467;
LT219525;Lepidoptera;Agriades optilete;Agriades optilete;1;Agriades optilete;0.999999964347467;
LT219526;Lepidoptera;Agriades optilete;Agriades optilete;1;Agriades optilete;0.999999964347467;
LT219527;Lepidoptera;Agriades optilete;Agriades optilete;1;Agriades optilete;0.999999964347467;
LT219528;Lepidoptera;Agriades optilete;Agriades optilete;1;Agriades optilete;0.999999964347467;
LT219529;Lepidoptera;Agriades optilete;Agriades optilete;1;Agriades optilete;0.999999964347467;
LT219530;Lepidoptera;Agriades optilete;Agriades optilete;1;Agriades optilete;0.999999964347467;
LT219531;Lepidoptera;Agriades optilete;Agriades optilete;1;Agriades optilete;0.999999964347467;
LT219532;Lepidoptera;Agriades optilete;Agriades optilete;1;Agriades optilete;0.999999964347467;
LT219533;Lepidoptera;Agriades optilete;Agriades optilete;1;Agriades optilete;0.999999964347467;
LT219534;Lepidoptera;Agriades optilete;Agriades optilete;1;Agriades optilete;0.999999964347467;
LT219535;Lepidoptera;Agriades optilete;Agriades optilete;1;Agriades optilete;0.999999964347467;
LT219536;Lepidoptera;Agriades optilete;Agriades optilete;1;Agriades optilete;0.999999964347467;
LT219537;Lepidoptera;Agriades optilete;Agriades optilete;1;Agriades optilete;0.999999964347467;
LT219538;Lepidoptera;Agriades optilete;Agriades optilete;1;Agriades optilete;0.999999964347467;
LT219539;Lepidoptera;Agriades optilete;Agriades optilete;1;Agriades optilete;0.999999964347467;
LT219540;Lepidoptera;Agriades optilete;Agriades optilete;1;Agriades optilete;0.999999964347467;
LT219541;Lepidoptera;Agriades optilete;Agriades optilete;1;Agriades optilete;0.999999964347467;
LT219542;Lepidoptera;Agriades optilete;Agriades optilete;1;Agriades optilete;0.999999964347467;
LT219543;Lepidoptera;Agriades optilete;Agriades optilete;1;Agriades optilete;0.999999964347467;
LT219544;Lepidoptera;Agriades optilete;Agriades optilete;1;Agriades optilete;0.999999964347467;
LT219545;Lepidoptera;Agriades optilete;Agriades optilete;1;Agriades optilete;0.999999947321727;
LT219546;Lepidoptera;Agriades optilete;Agriades optilete;1;Agriades optilete;0.999999954047469;
LT219547;Lepidoptera;Agriades optilete;Agriades optilete;1;Agriades optilete;0.999999964347467;
LT219548;Lepidoptera;Agriades optilete;Agriades optilete;1;Agriades optilete;0.999999964347467;
LT219549;Lepidoptera;Agriades optilete;Agriades optilete;1;Agriades optilete;0.999999964347467;
LT219550;Lepidoptera;Agriades optilete;Agriades optilete;1;Agriades optilete;0.999999964347467;
LT219551;Lepidoptera;Agriades optilete;Agriades optilete;1;Agriades optilete;0.999999964347467;
LT219552;Lepidoptera;Agriades optilete;Agriades optilete;1;Agriades optilete;0.999999964347467;

LT219553;Lepidoptera;Agriades optilete;Agriades optilete;1;Agriades optilete;0.999999964347467;
LT219554;Lepidoptera;Agriades optilete;Agriades optilete;1;Agriades optilete;0.999999964347467;
LT219555;Lepidoptera;Agriades optilete;Agriades optilete;1;Agriades optilete;0.999999964347467;
LT219556;Lepidoptera;Agriades optilete;Agriades optilete;1;Agriades optilete;0.999999964347467;
LT219557;Lepidoptera;Agriades optilete;Agriades optilete;1;Agriades optilete;0.999999964347467;
LT219558;Lepidoptera;Agriades optilete;Agriades optilete;1;Agriades optilete;0.999999964347467;
LT219559;Lepidoptera;Agriades optilete;Agriades optilete;1;Agriades optilete;0.999999964347467;
LT219560;Lepidoptera;Agriades optilete;Agriades optilete;1;Agriades optilete;0.999999958368989;
LT219561;Lepidoptera;Agriades optilete;Agriades optilete;1;Agriades optilete;0.999999358970292;
LT219713;Lepidoptera;Glaucopsyche alexis;NA;NA;Glaucopsyche alexis;0.972762900740308;
LT219714;Lepidoptera;Glaucopsyche alexis;NA;NA;Glaucopsyche alexis;0.972762900740308;
LT219715;Lepidoptera;Glaucopsyche alexis;NA;NA;Glaucopsyche alexis;0.972762900740308;
LT219716;Lepidoptera;Glaucopsyche alexis;NA;NA;Glaucopsyche alexis;0.972762900740308;
LT219717;Lepidoptera;Glaucopsyche alexis;NA;NA;Glaucopsyche alexis;0.972762900740308;
LT219718;Lepidoptera;Glaucopsyche alexis;NA;NA;Glaucopsyche alexis;0.972762900740308;
LT219719;Lepidoptera;Glaucopsyche alexis;NA;NA;Glaucopsyche alexis;0.963411140125619;
LT219720;Lepidoptera;Glaucopsyche alexis;NA;NA;Glaucopsyche alexis;0.963411140125619;
LT219721;Lepidoptera;Glaucopsyche alexis;NA;NA;Glaucopsyche alexis;0.963411140125619;
LT219722;Lepidoptera;Glaucopsyche alexis;NA;NA;Glaucopsyche alexis;0.963411140125619;
LT219723;Lepidoptera;Glaucopsyche alexis;NA;NA;Glaucopsyche alexis;0.963411140125619;
LT219724;Lepidoptera;Glaucopsyche alexis;NA;NA;Glaucopsyche alexis;0.963411140125619;
LT219725;Lepidoptera;Glaucopsyche alexis;NA;NA;Glaucopsyche alexis;0.976715242672232;
LT219726;Lepidoptera;Glaucopsyche alexis;NA;NA;Glaucopsyche alexis;0.963411140125619;
LT219727;Lepidoptera;Glaucopsyche alexis;NA;NA;Glaucopsyche alexis;0.972762900740308;
LT219728;Lepidoptera;Glaucopsyche alexis;NA;NA;Glaucopsyche alexis;0.972762900740308;
LT219729;Lepidoptera;Glaucopsyche alexis;NA;NA;Glaucopsyche alexis;0.963411140125619;
LT219730;Lepidoptera;Glaucopsyche alexis;NA;NA;Glaucopsyche alexis;0.963411140125619;
LT219731;Lepidoptera;Glaucopsyche alexis;NA;NA;Glaucopsyche alexis;0.963411140125619;
LT219732;Lepidoptera;Glaucopsyche alexis;NA;NA;Glaucopsyche alexis;0.963411140125619;
LT219733;Lepidoptera;Glaucopsyche alexis;NA;NA;Glaucopsyche alexis;0.972762900740308;
LT219734;Lepidoptera;Glaucopsyche alexis;NA;NA;Glaucopsyche alexis;0.963411140125619;
LT219735;Lepidoptera;Glaucopsyche alexis;NA;NA;Glaucopsyche alexis;0.963411140125619;
LT219736;Lepidoptera;Glaucopsyche alexis;NA;NA;Glaucopsyche alexis;0.963411140125619;
LT219737;Lepidoptera;Glaucopsyche alexis;NA;NA;Glaucopsyche alexis;0.963411140125619;
LT219738;Lepidoptera;Glaucopsyche alexis;NA;NA;Glaucopsyche alexis;0.972762900740308;
LT219739;Lepidoptera;Glaucopsyche alexis;NA;NA;Glaucopsyche alexis;0.972762900740308;
LT219740;Lepidoptera;Glaucopsyche alexis;NA;NA;Glaucopsyche alexis;0.972762900740308;
LT219741;Lepidoptera;Glaucopsyche alexis;NA;NA;Glaucopsyche alexis;0.972762900740308;
LT219742;Lepidoptera;Glaucopsyche alexis;NA;NA;Glaucopsyche alexis;0.972762900740308;
LT219743;Lepidoptera;Glaucopsyche alexis;NA;NA;Glaucopsyche alexis;0.972762900740308;
LT219744;Lepidoptera;Glaucopsyche alexis;NA;NA;Glaucopsyche alexis;0.972762900740308;
LT219745;Lepidoptera;Glaucopsyche alexis;NA;NA;Glaucopsyche alexis;0.972762900740308;
LT219746;Lepidoptera;Glaucopsyche alexis;NA;NA;Glaucopsyche alexis;0.972762900740308;
LT219747;Lepidoptera;Glaucopsyche alexis;NA;NA;Glaucopsyche alexis;0.972762900740308;
LT219748;Lepidoptera;Glaucopsyche alexis;NA;NA;Glaucopsyche alexis;0.972762900740308;
LT219749;Lepidoptera;Glaucopsyche alexis;NA;NA;Glaucopsyche alexis;0.972762900740308;
LT219750;Lepidoptera;Glaucopsyche alexis;NA;NA;Glaucopsyche alexis;0.972762900740308;
LT219751;Lepidoptera;Glaucopsyche alexis;NA;NA;Glaucopsyche alexis;0.972762900740308;
LT219752;Lepidoptera;Glaucopsyche alexis;NA;NA;Glaucopsyche alexis;0.972762900740308;
LT219753;Lepidoptera;Glaucopsyche alexis;NA;NA;Glaucopsyche alexis;0.963411140125619;
LT219754;Lepidoptera;Glaucopsyche alexis;NA;NA;Glaucopsyche alexis;0.963411140125619;
LT219755;Lepidoptera;Glaucopsyche alexis;NA;NA;Glaucopsyche alexis;0.963411140125619;
LT219756;Lepidoptera;Glaucopsyche alexis;NA;NA;Glaucopsyche alexis;0.963411140125619;
LT219757;Lepidoptera;Glaucopsyche alexis;NA;NA;Glaucopsyche alexis;0.963411140125619;
LT219758;Lepidoptera;Glaucopsyche alexis;NA;NA;Glaucopsyche alexis;0.963411140125619;
LT219759;Lepidoptera;Glaucopsyche alexis;NA;NA;Glaucopsyche alexis;0.963411140125619;
LT219760;Lepidoptera;Glaucopsyche alexis;NA;NA;Glaucopsyche alexis;0.963411140125619;
LT219761;Lepidoptera;Glaucopsyche alexis;NA;NA;Glaucopsyche alexis;0.972762900740308;
LT219762;Lepidoptera;Glaucopsyche alexis;NA;NA;Glaucopsyche alexis;0.972762900740308;
LT219763;Lepidoptera;Glaucopsyche alexis;NA;NA;Glaucopsyche alexis;0.972762900740308;
LT219764;Lepidoptera;Glaucopsyche alexis;NA;NA;Glaucopsyche alexis;0.972762900740308;
LT219765;Lepidoptera;Glaucopsyche alexis;NA;NA;Glaucopsyche alexis;0.972762900740308;
LT219766;Lepidoptera;Glaucopsyche alexis;NA;NA;Glaucopsyche alexis;0.972762900740308;
LT219767;Lepidoptera;Glaucopsyche alexis;NA;NA;Glaucopsyche alexis;0.972762900740308;
LT219768;Lepidoptera;Glaucopsyche alexis;NA;NA;Glaucopsyche alexis;0.972762900740308;
LT219769;Lepidoptera;Glaucopsyche alexis;NA;NA;Glaucopsyche alexis;0.972762900740308;
LT219770;Lepidoptera;Glaucopsyche alexis;NA;NA;Glaucopsyche alexis;0.972762900740308;
LT219771;Lepidoptera;Glaucopsyche alexis;NA;NA;Glaucopsyche alexis;0.963411140125619;
LT219772;Lepidoptera;Glaucopsyche alexis;NA;NA;Glaucopsyche alexis;0.963411140125619;
LT219773;Lepidoptera;Glaucopsyche alexis;NA;NA;Glaucopsyche alexis;0.963411140125619;
LT219774;Lepidoptera;Glaucopsyche alexis;NA;NA;Glaucopsyche alexis;0.963411140125619;
LT219775;Lepidoptera;Glaucopsyche alexis;NA;NA;Glaucopsyche alexis;0.963411140125619;
LT219776;Lepidoptera;Glaucopsyche alexis;NA;NA;Glaucopsyche alexis;0.963411140125619;
LT219777;Lepidoptera;Glaucopsyche alexis;NA;NA;Glaucopsyche alexis;0.963411140125619;
LT219778;Lepidoptera;Glaucopsyche alexis;NA;NA;Glaucopsyche alexis;0.972762900740308;
LT219780;Lepidoptera;Glaucopsyche alexis;NA;NA;Glaucopsyche alexis;0.965843543377355;
LT219781;Lepidoptera;Glaucopsyche alexis;NA;NA;Glaucopsyche alexis;0.965843543377355;
LT219782;Lepidoptera;Glaucopsyche alexis;NA;NA;Glaucopsyche alexis;0.965843543377355;

LT219783;Lepidoptera;Glaucopsyche alexis;NA;NA;Glaucopsyche alexis;0.965843543377355;
LT219784;Lepidoptera;Glaucopsyche alexis;NA;NA;Glaucopsyche alexis;0.965843543377355;
LT219785;Lepidoptera;Glaucopsyche alexis;NA;NA;Glaucopsyche alexis;0.965843543377355;
LT219786;Lepidoptera;Glaucopsyche alexis;NA;NA;Glaucopsyche alexis;0.966529405371757;
LT219787;Lepidoptera;Glaucopsyche alexis;NA;NA;Glaucopsyche alexis;0.977215812206823;
LT219788;Lepidoptera;Glaucopsyche alexis;NA;NA;Glaucopsyche alexis;0.957275461081428;
LT219790;Lepidoptera;Glaucopsyche alexis;NA;NA;Glaucopsyche alexis;0.968840264023661;
LT219791;Lepidoptera;Glaucopsyche alexis;NA;NA;Glaucopsyche alexis;0.963411140125619;
LT219792;Lepidoptera;Glaucopsyche alexis;NA;NA;Glaucopsyche alexis;0.963411140125619;
LT219793;Lepidoptera;Glaucopsyche alexis;NA;NA;Glaucopsyche alexis;0.972762900740308;
LT219794;Lepidoptera;Glaucopsyche alexis;NA;NA;Glaucopsyche alexis;0.972762900740308;
LT219795;Lepidoptera;Glaucopsyche alexis;NA;NA;Glaucopsyche alexis;0.972762900740308;
LT219796;Lepidoptera;Glaucopsyche alexis;NA;NA;Glaucopsyche alexis;0.972762900740308;
LT219797;Lepidoptera;Glaucopsyche alexis;NA;NA;Glaucopsyche alexis;0.972762900740308;
LT219798;Lepidoptera;Glaucopsyche alexis;NA;NA;Glaucopsyche alexis;0.972762900740308;
LT219799;Lepidoptera;Glaucopsyche alexis;NA;NA;Glaucopsyche alexis;0.972762900740308;
LT219800;Lepidoptera;Glaucopsyche alexis;NA;NA;Glaucopsyche alexis;0.972762900740308;
LT219801;Lepidoptera;Glaucopsyche alexis;NA;NA;Glaucopsyche alexis;0.972762900740308;
LT219802;Lepidoptera;Glaucopsyche alexis;NA;NA;Glaucopsyche alexis;0.972762900740308;
LT219803;Lepidoptera;Glaucopsyche alexis;NA;NA;Glaucopsyche alexis;0.972762900740308;
LT219804;Lepidoptera;Glaucopsyche alexis;NA;NA;Glaucopsyche alexis;0.972762900740308;
LT219805;Lepidoptera;Glaucopsyche alexis;NA;NA;Glaucopsyche alexis;0.972762900740308;
LT219806;Lepidoptera;Plebejus argus;NA;0.001;Plebejus argus;0.964830489107857;
LT219807;Lepidoptera;Plebejus argus;NA;0.037;Plebejus argus;0.967686914533617;
LT219808;Lepidoptera;Plebejus argus;Plebejus argus;0.016;Plebejus argus;0.977770143991648;
LT219809;Lepidoptera;Plebejus argus;NA;0.037;Plebejus argus;0.967686914533617;
LT219810;Lepidoptera;Plebejus argus;NA;0.037;Plebejus argus;0.967686914533617;
LT219811;Lepidoptera;Plebejus argus;NA;0.056;Plebejus argus;0.960383357130779;
LT219812;Lepidoptera;Plebejus argus;NA;0.037;Plebejus argus;0.967686914533617;
LT219813;Lepidoptera;Plebejus argus;NA;0.037;Plebejus argus;0.967686914533617;
LT219814;Lepidoptera;Plebejus argus;NA;0.001;Plebejus argus;0.964830489107857;
LT219816;Lepidoptera;Plebejus argus;NA;0.037;Plebejus argus;0.967686914533617;
LT219817;Lepidoptera;Plebejus argus;NA;0.323;Plebejus argus;0.921546344013766;
LT219818;Lepidoptera;Plebejus argus;NA;0.323;Plebejus argus;0.921546344013766;
LT219819;Lepidoptera;Plebejus argus;NA;0.323;Plebejus argus;0.921546344013766;
LT219827;Lepidoptera;Plebejus argus;NA;0.003;Plebejus argus;0.960055152697393;
LT219828;Lepidoptera;Plebejus argus;NA;0.129;Plebejus argus;0.922646956033098;
LT219829;Lepidoptera;Plebejus argus;NA;0.119;Plebejus argus;0.948498951406236;
LT219830;Lepidoptera;Plebejus argus;NA;0.162;Plebejus argus;0.902098365042469;
LT219831;Lepidoptera;Plebejus argus;NA;0.04;Plebejus argus;0.95690508933728;
LT219835;Lepidoptera;Plebejus argus;NA;0.133;Plebejus argus;0.979302527358729;
LT219836;Lepidoptera;Plebejus argus;NA;0.001;Plebejus argus;0.964830489107857;
LT219837;Lepidoptera;Plebejus argus;NA;0.323;Plebejus argus;0.921546344013766;
LT219838;Lepidoptera;Plebejus argus;NA;0.323;Plebejus argus;0.921546344013766;
LT219839;Lepidoptera;Plebejus argus;NA;0.037;Plebejus argus;0.967686914533617;
LT219840;Lepidoptera;Plebejus argus;Plebejus argus;0.01;Plebejus argus;0.96041037009845;
LT219841;Lepidoptera;Plebejus argus;NA;0.037;Plebejus argus;0.967686914533617;
LT219844;Lepidoptera;Plebejus argus;NA;0.001;Plebejus argus;0.964830489107857;
LT219855;Lepidoptera;Plebejus argus;NA;0.312;Plebejus argus;0.969776741393822;
LT219856;Lepidoptera;Plebejus argus;NA;0.042;Plebejus argus;0.948155414552817;
LT219857;Lepidoptera;Plebejus argus;Plebejus argus;0.017;Plebejus argus;0.968169272119379;
LT219858;Lepidoptera;Plebejus argus;NA;0.119;Plebejus argus;0.948498951406236;
LT219859;Lepidoptera;Plebejus argus;NA;0.323;Plebejus argus;0.921546344013766;
LT219860;Lepidoptera;Plebejus argus;NA;0.119;Plebejus argus;0.948498951406236;
LT219861;Lepidoptera;Plebejus argus;NA;0.119;Plebejus argus;0.948498951406236;
LT219862;Lepidoptera;Plebejus argus;NA;0.119;Plebejus argus;0.948498951406236;
LT219863;Lepidoptera;Plebejus argus;NA;0.119;Plebejus argus;0.948498951406236;
LT219864;Lepidoptera;Plebejus argus;NA;0.323;Plebejus argus;0.921546344013766;
LT219865;Lepidoptera;Plebejus argus;NA;0.119;Plebejus argus;0.948498951406236;
LT219866;Lepidoptera;Plebejus argus;NA;0.323;Plebejus argus;0.921546344013766;
LT219867;Lepidoptera;Plebejus argus;NA;0.323;Plebejus argus;0.921546344013766;
LT219868;Lepidoptera;Plebejus argus;NA;0.133;Plebejus argus;0.918655685827247;
LT219869;Lepidoptera;Plebejus argus;NA;0.133;Plebejus argus;0.918655685827247;
LT219870;Lepidoptera;Plebejus argus;NA;0.323;Plebejus argus;0.921546344013766;
LT219871;Lepidoptera;Plebejus argus;NA;0.323;Plebejus argus;0.921546344013766;
LT219872;Lepidoptera;Plebejus argus;NA;0.323;Plebejus argus;0.921546344013766;
LT219873;Lepidoptera;Plebejus argus;NA;0.323;Plebejus argus;0.921546344013766;
LT219874;Lepidoptera;Plebejus argus;NA;0.323;Plebejus argus;0.921546344013766;
LT219875;Lepidoptera;Plebejus argus;NA;0.323;Plebejus argus;0.921546344013766;
LT219876;Lepidoptera;Plebejus argus;NA;0.323;Plebejus argus;0.921546344013766;
LT219877;Lepidoptera;Plebejus argus;NA;0.323;Plebejus argus;0.921546344013766;
LT219878;Lepidoptera;Plebejus argus;NA;0.133;Plebejus argus;0.918655685827247;
LT219879;Lepidoptera;Plebejus argus;NA;0.133;Plebejus argus;0.918655685827247;
LT219880;Lepidoptera;Plebejus argus;NA;0.133;Plebejus argus;0.918655685827247;
LT219881;Lepidoptera;Plebejus argus;NA;0.323;Plebejus argus;0.921546344013766;
LT219882;Lepidoptera;Plebejus argus;NA;0.323;Plebejus argus;0.921546344013766;
LT219883;Lepidoptera;Plebejus argus;NA;0.041;Plebejus argus;0.979288970293412;
LT219884;Lepidoptera;Plebejus argus;NA;0.133;Plebejus argus;0.979302527358729;

LT219885;Lepidoptera;Plebejus argus;NA;0.181;Plebejus argus;0.805416086060811;
LT219886;Lepidoptera;Plebejus argus;NA;0.109;Plebejus argus;0.978419259470235;
LT219887;Lepidoptera;Plebejus argus;NA;0.042;Plebejus argus;0.948155414552817;
LT219888;Lepidoptera;Plebejus argus;NA;0.042;Plebejus argus;0.948155414552817;
LT219889;Lepidoptera;Plebejus argus;NA;0.119;Plebejus argus;0.948498951406236;
LT219890;Lepidoptera;Plebejus argus;NA;0.119;Plebejus argus;0.948498951406236;
LT219891;Lepidoptera;Plebejus argus;NA;0.119;Plebejus argus;0.948498951406236;
LT219892;Lepidoptera;Plebejus argus;NA;0.042;Plebejus argus;0.948155414552817;
LT219893;Lepidoptera;Plebejus argus;NA;0.119;Plebejus argus;0.948498951406236;
LT219894;Lepidoptera;Plebejus argus;NA;0.323;Plebejus argus;0.921546344013766;
LT219895;Lepidoptera;Plebejus argus;Plebejus argus;0.021;Plebejus argus;0.952241106289486;
LT219896;Lepidoptera;Plebejus argus;NA;0.323;Plebejus argus;0.921546344013766;
LT219897;Lepidoptera;Plebejus argus;NA;0.323;Plebejus argus;0.921546344013766;
LT219898;Lepidoptera;Plebejus argus;NA;0.323;Plebejus argus;0.921546344013766;
LT219899;Lepidoptera;Plebejus argus;NA;0.323;Plebejus argus;0.921546344013766;
LT219900;Lepidoptera;Plebejus argus;NA;0.323;Plebejus argus;0.921546344013766;
LT219901;Lepidoptera;Plebejus argus;NA;NA;Plebejus argus;0.920081658994444;
LT219902;Lepidoptera;Plebejus argus;NA;0.323;Plebejus argus;0.921546344013766;
LT219903;Lepidoptera;Plebejus argus;NA;0.323;Plebejus argus;0.921546344013766;
LT219904;Lepidoptera;Plebejus argus;NA;0.048;Plebejus argus;0.941128082574131;
LT219905;Lepidoptera;Plebejus argus;NA;NA;Plebejus argus;0.930049018367304;
LT219906;Lepidoptera;Plebejus argus;NA;0.323;Plebejus argus;0.921546344013766;
LT219907;Lepidoptera;Plebejus argus;NA;0.323;Plebejus argus;0.921546344013766;
LT219908;Lepidoptera;Plebejus argus;NA;0.323;Plebejus argus;0.921546344013766;
LT219909;Lepidoptera;Plebejus argus;NA;0.323;Plebejus argus;0.921546344013766;
LT219910;Lepidoptera;Plebejus argus;NA;0.467;Plebejus argus;0.773467457505203;
LT219911;Lepidoptera;Plebejus argus;NA;0.323;Plebejus argus;0.921546344013766;
LT219912;Lepidoptera;Plebejus argus;NA;0.323;Plebejus argus;0.921546344013766;
LT219913;Lepidoptera;Plebejus argus;NA;0.045;Plebejus argus;0.978409213421933;
LT219914;Lepidoptera;Plebejus argus;NA;0.133;Plebejus argus;0.979302527358729;
LT219915;Lepidoptera;Plebejus argus;NA;0.041;Plebejus argus;0.979288970293412;
LT219916;Lepidoptera;Plebejus argus;NA;0.323;Plebejus argus;0.921546344013766;
LT219917;Lepidoptera;Plebejus argus;NA;NA;Plebejus argus;0.92429871433038;
LT219918;Lepidoptera;Plebejus argus;NA;NA;Plebejus argus;0.950111284998235;
LT219919;Lepidoptera;Plebejus argus;NA;0.323;Plebejus argus;0.921546344013766;
LT219920;Lepidoptera;Plebejus argus;NA;0.323;Plebejus argus;0.921546344013766;
LT219921;Lepidoptera;Plebejus argus;NA;0.323;Plebejus argus;0.921546344013766;
LT219922;Lepidoptera;Plebejus argus;NA;0.041;Plebejus argus;0.979288970293412;
LT219923;Lepidoptera;Plebejus argus;NA;0.041;Plebejus argus;0.979288970293412;
LT219924;Lepidoptera;Plebejus argus;NA;0.041;Plebejus argus;0.979288970293412;
LT219925;Lepidoptera;Plebejus argus;NA;0.045;Plebejus argus;0.978409213421933;
LT219926;Lepidoptera;Plebejus argus;NA;0.041;Plebejus argus;0.979288970293412;
LT219927;Lepidoptera;Plebejus argus;NA;0.041;Plebejus argus;0.979288970293412;
LT219928;Lepidoptera;Plebejus argus;NA;0.041;Plebejus argus;0.979288970293412;
LT219929;Lepidoptera;Plebejus argus;NA;0.133;Plebejus argus;0.979302527358729;
LT219930;Lepidoptera;Plebejus argus;Plebejus argus;0.006;Plebejus argus;0.934029408528532;
LT219931;Lepidoptera;Plebejus argus;NA;0.119;Plebejus argus;0.948498951406236;
LT219932;Lepidoptera;Plebejus argus;Plebejus argus;0.006;Plebejus argus;0.934029408528532;
LT219933;Lepidoptera;Plebejus argus;NA;NA;Plebejus argus;0.930049018367304;
LT219934;Lepidoptera;Plebejus argus;NA;0.119;Plebejus argus;0.948498951406236;
LT219935;Lepidoptera;Plebejus argus;NA;0.001;Plebejus argus;0.964830489107857;
LT219936;Lepidoptera;Plebejus idas;NA;0.001;NA;0.998753943855449;
LT219937;Lepidoptera;Plebejus idas;NA;0.001;NA;0.998753943855449;
LT219938;Lepidoptera;Plebejus idas;NA;0.001;NA;0.998753943855449;
LT219939;Lepidoptera;Plebejus idas;NA;0.001;NA;0.998753943855449;
LT219940;Lepidoptera;Plebejus idas;NA;0.001;NA;0.998753943855449;
LT219941;Lepidoptera;Plebejus idas;NA;0.001;NA;0.998753943855449;
LT219942;Lepidoptera;Plebejus idas;NA;0.001;NA;0.998650809027638;
LT219943;Lepidoptera;Plebejus idas;NA;0.001;NA;0.998650809027638;
LT219944;Lepidoptera;Plebejus idas;NA;0.001;NA;0.997023072258307;
LT219945;Lepidoptera;Plebejus idas;NA;0.001;NA;0.988098477943813;
LT219946;Lepidoptera;Plebejus idas;NA;0.001;NA;0.998753943855449;
LT219947;Lepidoptera;Plebejus idas;NA;0.001;NA;0.998753943855449;
LT219948;Lepidoptera;Plebejus idas;NA;0.001;NA;0.998753943855449;
LT219949;Lepidoptera;Plebejus idas;NA;0.001;NA;0.998753943855449;
LT219950;Lepidoptera;Plebejus idas;NA;0.001;NA;0.998753943855449;
LT219951;Lepidoptera;Plebejus idas;NA;0.004;NA;0.998911061992308;
LT219952;Lepidoptera;Plebejus idas;NA;0.001;NA;0.998753943855449;
LT219953;Lepidoptera;Plebejus idas;NA;0.001;NA;0.998753943855449;
LT219954;Lepidoptera;Plebejus idas;NA;0.001;NA;0.998753943855449;
LT219955;Lepidoptera;Plebejus idas;NA;0.001;NA;0.998753943855449;
LT219956;Lepidoptera;Plebejus idas;NA;0.001;NA;0.997238735407381;
LT219957;Lepidoptera;Plebejus idas;NA;0.001;NA;0.998650809027638;
LT219958;Lepidoptera;Plebejus idas;NA;0.001;NA;0.998650809027638;
LT219959;Lepidoptera;Plebejus idas;NA;0.001;NA;0.998753943855449;
LT219960;Lepidoptera;Plebejus idas;NA;0.001;NA;0.998523518484146;
LT219961;Lepidoptera;Plebejus idas;NA;0.001;NA;0.998523518484146;
LT219962;Lepidoptera;Plebejus idas;NA;0.001;NA;0.998650809027638;

LT219963;Lepidoptera;Plebejus idas;NA;0.001;NA;0.998650809027638;
LT219964;Lepidoptera;Plebejus idas;NA;0.001;NA;0.998650809027638;
LT219965;Lepidoptera;Plebejus idas;NA;0.001;NA;0.998650809027638;
LT219966;Lepidoptera;Plebejus idas;NA;0.001;NA;0.998650809027638;
LT219967;Lepidoptera;Plebejus idas;NA;0.001;NA;0.998753943855449;
LT219968;Lepidoptera;Plebejus idas;NA;0.001;NA;0.998753943855449;
LT219969;Lepidoptera;Plebejus idas;NA;0.001;NA;0.998753943855449;
LT219970;Lepidoptera;Plebejus idas;NA;0.001;NA;0.998650809027638;
LT219971;Lepidoptera;Plebejus idas;NA;0.001;NA;0.998650809027638;
LT219972;Lepidoptera;Plebejus idas;NA;0.001;NA;0.998650809027638;
LT219973;Lepidoptera;Plebejus idas;NA;0.001;NA;0.998650809027638;
LT219974;Lepidoptera;Plebejus idas;NA;0.001;NA;0.998650809027638;
LT219975;Lepidoptera;Plebejus idas;NA;0.001;NA;0.998650809027638;
LT219976;Lepidoptera;Plebejus idas;NA;0.001;NA;0.998753943855449;
LT219977;Lepidoptera;Plebejus idas;NA;0.001;NA;0.998650809027638;
LT219978;Lepidoptera;Plebejus idas;NA;0.001;NA;0.998650809027638;
LT219979;Lepidoptera;Plebejus idas;NA;0.001;NA;0.999109895233321;
LT219980;Lepidoptera;Plebejus idas;NA;0.001;NA;0.998650809027638;
LT219981;Lepidoptera;Plebejus idas;NA;0.001;NA;0.998401268195457;
LT219982;Lepidoptera;Plebejus idas;NA;0.001;NA;0.998753943855449;
LT219983;Lepidoptera;Plebejus idas;NA;0.001;NA;0.998753943855449;
LT219984;Lepidoptera;Plebejus idas;NA;0.001;NA;0.998650809027638;
LT219985;Lepidoptera;Plebejus idas;NA;0.001;NA;0.998650809027638;
LT219986;Lepidoptera;Plebejus idas;NA;0.001;NA;0.998650809027638;
LT219987;Lepidoptera;Plebejus idas;NA;0.001;NA;0.998650809027638;
LT219988;Lepidoptera;Plebejus idas;NA;0.001;NA;0.998650809027638;
LT219989;Lepidoptera;Plebejus idas;NA;0.001;NA;0.998650809027638;
LT219990;Lepidoptera;Plebejus idas;NA;0.001;NA;0.998650809027638;
LT219991;Lepidoptera;Plebejus idas;NA;0.001;NA;0.998650809027638;
LT219992;Lepidoptera;Plebejus idas;NA;0.001;NA;0.998650809027638;
LT219993;Lepidoptera;Plebejus idas;NA;0.001;NA;0.998650809027638;
LT219994;Lepidoptera;Plebejus idas;NA;0.001;NA;0.998753943855449;
LT219995;Lepidoptera;Plebejus idas;NA;0.001;NA;0.998753943855449;
LT219996;Lepidoptera;Plebejus idas;NA;0.001;NA;0.998753943855449;
LT219997;Lepidoptera;Plebejus idas;NA;0.001;NA;0.998753943855449;
LT219998;Lepidoptera;Plebejus idas;NA;0.001;NA;0.998753943855449;
LT219999;Lepidoptera;Plebejus idas;NA;0.001;NA;0.998753943855449;
LT220000;Lepidoptera;Plebejus idas;NA;0.001;NA;0.998753943855449;
LT220001;Lepidoptera;Plebejus idas;NA;0.001;NA;0.998753943855449;
LT220002;Lepidoptera;Plebejus idas;NA;0.001;NA;0.998753943855449;
LT220003;Lepidoptera;Plebejus idas;NA;0.001;NA;0.998753943855449;
LT220004;Lepidoptera;Plebejus idas;NA;0.001;NA;0.998753943855449;
LT220005;Lepidoptera;Plebejus idas;NA;0.001;NA;0.998753943855449;
LT220006;Lepidoptera;Plebejus idas;NA;0.001;NA;0.998753943855449;
LT220007;Lepidoptera;Plebejus idas;NA;0.001;NA;0.998753943855449;
LT220008;Lepidoptera;Plebejus idas;NA;0.001;NA;0.998753943855449;
LT220009;Lepidoptera;Plebejus idas;NA;0.001;NA;0.998753943855449;
LT220010;Lepidoptera;Plebejus idas;NA;0.001;NA;0.998753943855449;
LT220011;Lepidoptera;Plebejus idas;NA;0.001;NA;0.998753943855449;
LT220012;Lepidoptera;Plebejus idas;NA;0.001;NA;0.998753943855449;
LT220013;Lepidoptera;Plebejus idas;NA;0.002;NA;0.99834166956006;
LT220014;Lepidoptera;Plebejus idas;NA;0.001;NA;0.998753943855449;
LT220015;Lepidoptera;Plebejus idas;NA;0.001;NA;0.998753943855449;
LT220016;Lepidoptera;Plebejus idas;NA;0.004;NA;0.998911061992308;
LT220017;Lepidoptera;Plebejus idas;NA;0.001;NA;0.998753943855449;
LT628553;Lepidoptera;Polyommatus amandus;Polyommatus amandus;0.002;Polyommatus amandus;0.999970849880921;
LT628556;Lepidoptera;Polyommatus amandus;Polyommatus amandus;0.012;Polyommatus amandus;0.999875264763331;
LT628804;Lepidoptera;Aricia agestis;NA;0.306;Aricia agestis;0.993846455426407;
LT628805;Lepidoptera;Aricia agestis;NA;0.405;Aricia agestis;0.995485315574387;
LT628881;Lepidoptera;Polyommatus dorylas;Polyommatus dorylas;0.002;Polyommatus dorylas;0.963617055955984;
LT628915;Lepidoptera;Aricia agestis;NA;NA;Aricia agestis;0.996270377984369;
LT628916;Lepidoptera;Aricia agestis;NA;NA;Aricia agestis;0.996270377984369;
LT628930;Lepidoptera;Polyommatus amandus;Polyommatus amandus;1;Polyommatus amandus;0.999961064788254;
LT628956;Lepidoptera;Polyommatus amandus;Polyommatus amandus;0.003;Polyommatus amandus;0.999969356061692;
LT628980;Lepidoptera;Polyommatus amandus;Polyommatus amandus;0.003;Polyommatus amandus;0.999969356061692;
LT628985;Lepidoptera;Plebejus argyrognomon;Plebejus argyrognomon;1;Plebejus argyrognomon;0.938702955637604;
LT628986;Lepidoptera;Plebejus argyrognomon;NA;0.002;NA;0.996750582460086;
LT628987;Lepidoptera;Plebejus idas;NA;0.001;NA;0.988098477943813;
LT628993;Lepidoptera;Polyommatus daphnis;NA;0.046;Polyommatus daphnis;0.990287310655549;
LT628999;Lepidoptera;Plebejus argus;Plebejus argus;0.001;Plebejus argus;0.851074983745483;
LT629003;Lepidoptera;Aricia agestis;NA;0.091;Aricia agestis;0.998135413168303;

LT629005;Lepidoptera;Aricia agestis;NA;0.073;Aricia agestis;0.999504421887332;
LT629009;Lepidoptera;Polyommatus icarus;NA;0.004;Polyommatus icarus;0.999481683747521;
LT629009;Lepidoptera;Polyommatus icarus;NA;0.004;Polyommatus icarus;0.999481683747521;
LT629010;Lepidoptera;Polyommatus icarus;NA;0.004;Polyommatus icarus;0.999481683747521;
LT629010;Lepidoptera;Polyommatus icarus;NA;0.004;Polyommatus icarus;0.999481683747521;
LT629016;Lepidoptera;Aricia agestis;NA;NA;Aricia agestis;0.996270377984369;
LT629025;Lepidoptera;Aricia agestis;NA;0.073;Aricia agestis;0.999504421887332;
LT629028;Lepidoptera;Aricia agestis;NA;0.085;Aricia agestis;0.998833911707261;
LT629035;Lepidoptera;Polyommatus icarus;Polyommatus icarus;0.001;Polyommatus icarus;0.999851522482238;
LT629035;Lepidoptera;Polyommatus icarus;Polyommatus icarus;0.001;Polyommatus icarus;0.999851522482238;
LT629040;Lepidoptera;Plebejus argus;Plebejus argus;0.001;Plebejus argus;0.995411334945632;
LT795297;Lepidoptera;Boloria selene;NA;0.42;Boloria selene;0.999996162146235;
LT795298;Lepidoptera;Boloria selene;NA;0.42;Boloria selene;0.999996162146235;
LT795304;Lepidoptera;Boloria selene;NA;0.044;Boloria selene;0.999999873314501;
LT795313;Lepidoptera;Boloria selene;NA;0.166;Boloria selene;0.999999971989496;
LT795320;Lepidoptera;Boloria selene;Boloria selene;NA;Boloria selene;0.999982457459507;
LT795322;Lepidoptera;Boloria selene;NA;0.08;Boloria selene;0.999999988470876;
LT795330;Lepidoptera;Boloria selene;NA;0.091;Boloria selene;0.999999914141771;
LT795331;Lepidoptera;Boloria selene;NA;0.154;Boloria selene;0.999999949774748;
LT795338;Lepidoptera;Boloria selene;Boloria selene;0.001;Boloria selene;0.999999992311274;
LT986363;Lepidoptera;Pieris napi;Pieris napi;0.001;Pieris napi;0.953140558002703;
LT986364;Lepidoptera;Pieris napi;Pieris napi;NA;Pieris napi;0.992000774048581;
LT986365;Lepidoptera;Pieris napi;NA;NA;Pieris napi;0.96403631573689;
LT986366;Lepidoptera;Pieris napi;NA;NA;NA;0.999999999997521;
LT986367;Lepidoptera;Pieris napi;NA;0.001;Pieris napi;0.901941565059126;
LT986368;Lepidoptera;Pieris napi;NA;0.007;Pieris napi;0.970875043233175;
LT986369;Lepidoptera;Pieris napi;NA;NA;NA;0.999999999997521;
LT986370;Lepidoptera;Pieris napi;NA;0.001;Pieris napi;0.901941565059126;
LTOL073-06;Lepidoptera;Parnassius apollo;Parnassius apollo;1;Parnassius apollo;0.997639119991203;
MBMPA022-07;Lepidoptera;Melitaea britomartis;NA;0.126;Melitaea britomartis;0.984864067137034;
MBMPA040-07;Lepidoptera;Melitaea trivia;Melitaea trivia;1;Melitaea trivia;0.999999783021304;
MBMPA043-07;Lepidoptera;Melitaea trivia;Melitaea trivia;1;Melitaea trivia;0.999999999977064;
MBMPA045-07;Lepidoptera;Melitaea trivia;Melitaea trivia;1;Melitaea trivia;0.999999999977064;
MBMPA046-07;Lepidoptera;Melitaea trivia;Melitaea trivia;1;Melitaea trivia;0.999999999977064;
MBMPA047-07;Lepidoptera;Melitaea trivia;Melitaea trivia;1;Melitaea trivia;0.999999999977064;
MBMPA048-07;Lepidoptera;Melitaea trivia;Melitaea trivia;1;Melitaea trivia;0.999999999977064;
MBMPA050-07;Lepidoptera;Melitaea trivia;Melitaea trivia;1;Melitaea trivia;0.999999999977064;
MBMPA053-07;Lepidoptera;Melitaea cinxia;Melitaea cinxia;1;Melitaea cinxia;0.999999999998977;
MBMPA054-07;Lepidoptera;Melitaea cinxia;NA;0.279;Melitaea cinxia;0.99999999999946;
MBMPA056-07;Lepidoptera;Melitaea trivia;Melitaea trivia;1;Melitaea trivia;0.999999999877389;
MBMPA057-07;Lepidoptera;Melitaea trivia;Melitaea trivia;1;Melitaea trivia;0.999999999877389;
MBMPA058-07;Lepidoptera;Melitaea trivia;Melitaea trivia;1;Melitaea trivia;0.999999999958618;
MBMPA060-07;Lepidoptera;Melitaea trivia;Melitaea trivia;1;Melitaea trivia;0.999999999877389;
MBMPA061-07;Lepidoptera;Melitaea trivia;Melitaea trivia;1;Melitaea trivia;0.999999999960949;
MBMPA062-07;Lepidoptera;Melitaea trivia;Melitaea trivia;1;Melitaea trivia;0.999999999954696;
MBMPA063-07;Lepidoptera;Melitaea trivia;Melitaea trivia;1;Melitaea trivia;0.999999999877389;
MBMPA064-07;Lepidoptera;Melitaea trivia;Melitaea trivia;1;Melitaea trivia;0.999999988387401;
MBMPA065-07;Lepidoptera;Melitaea trivia;Melitaea trivia;1;Melitaea trivia;0.999999999877389;
MBMPA066-07;Lepidoptera;Melitaea trivia;Melitaea trivia;1;Melitaea trivia;0.999999999960949;
MBMPA069-07;Lepidoptera;Melitaea trivia;Melitaea trivia;1;Melitaea trivia;0.999999999877389;
MBMPA071-07;Lepidoptera;Melitaea trivia;Melitaea trivia;1;Melitaea trivia;0.999999999960949;
MBMPA072-07;Lepidoptera;Melitaea trivia;Melitaea trivia;1;Melitaea trivia;0.999999999954696;
MBMPA073-07;Lepidoptera;Melitaea cinxia;NA;0.332;Melitaea cinxia;0.999999999998522;
MBMPA074-07;Lepidoptera;Melitaea cinxia;NA;0.324;Melitaea cinxia;0.999999999995623;
MBMPA075-07;Lepidoptera;Melitaea cinxia;NA;0.324;Melitaea cinxia;0.999999999995623;
MBMPA077-07;Lepidoptera;Melitaea diamina;NA;0.305;Melitaea diamina;0.999999994065661;
MBMPA078-07;Lepidoptera;Euphydryas aurinia;NA;0.001;Euphydryas aurinia;0.995576749826865;
MBMPA086-07;Lepidoptera;Melitaea didyma;Melitaea didyma;0.997;Melitaea didyma;0.999414231996446;
MBMPA087-07;Lepidoptera;Melitaea didyma;NA;0.099;Melitaea didyma;0.993412069257683;
MBMPA088-07;Lepidoptera;Melitaea didyma;Melitaea didyma;0.012;Melitaea didyma;0.99169844491886;
MBMPA089-07;Lepidoptera;Melitaea didyma;Melitaea didyma;0.014;Melitaea didyma;0.999112787489864;
MBMPA090-07;Lepidoptera;Melitaea didyma;Melitaea didyma;0.014;Melitaea didyma;0.999112787489864;
MBMPA091-07;Lepidoptera;Melitaea didyma;Melitaea didyma;0.014;Melitaea didyma;0.999112787489864;
MBMPA092-07;Lepidoptera;Melitaea didyma;Melitaea didyma;0.003;Melitaea didyma;0.999170363401737;
MBMPA093-07;Lepidoptera;Melitaea didyma;Melitaea didyma;0.014;Melitaea didyma;0.999112787489864;
MBMPA094-07;Lepidoptera;Melitaea didyma;Melitaea didyma;0.006;Melitaea didyma;0.998866265822718;
MBMPA158-07;Lepidoptera;Melitaea diamina;NA;0.305;Melitaea diamina;0.999999994065661;
MBMPA159-07;Lepidoptera;Melitaea diamina;NA;0.305;Melitaea diamina;0.999999994065661;
MBMPA160-07;Lepidoptera;Melitaea diamina;NA;0.305;Melitaea diamina;0.999999994065661;
MBMPA171-07;Lepidoptera;Melitaea britomartis;NA;NA;Melitaea britomartis;0.985732804805908;
MBMPA209-09;Lepidoptera;Melitaea britomartis;Melitaea britomartis;1;Melitaea britomartis;0.999407726535082;
MBMPA210-09;Lepidoptera;Melitaea britomartis;Melitaea britomartis;1;Melitaea britomartis;0.999407726535082;

MBMPA211-09;Lepidoptera;Melitaea britomartis;Melitaea britomartis;1;Melitaea britomartis;0.999407726535082;
MBMPA212-09;Lepidoptera;Melitaea britomartis;Melitaea britomartis;1;Melitaea britomartis;0.999407726535082;
MBMPA213-09;Lepidoptera;Melitaea britomartis;Melitaea britomartis;1;Melitaea britomartis;0.999407726535082;
MBMPA214-09;Lepidoptera;Melitaea britomartis;Melitaea britomartis;1;Melitaea britomartis;0.999407726535082;
MBMPA215-09;Lepidoptera;Melitaea britomartis;NA;NA;Melitaea britomartis;0.981389105125501;
MBMPA216-09;Lepidoptera;Melitaea britomartis;Mellicta britomartis;1;Melitaea britomartis;0.921480340185081;
MBMPA217-09;Lepidoptera;Melitaea britomartis;Melitaea britomartis;1;Melitaea britomartis;0.999407726535082;
MBMPA270-09;Lepidoptera;Melitaea cinxia;NA;0.32;Melitaea cinxia;0.999999999998977;
MBMPA271-09;Lepidoptera;Melitaea cinxia;NA;0.302;Melitaea arduinna;0.999997657342691;
MBMPA272-09;Lepidoptera;Melitaea cinxia;NA;0.066;Melitaea cinxia;0.999999999998977;
MBMPA273-09;Lepidoptera;Melitaea cinxia;NA;0.112;Melitaea cinxia;0.999999999998352;
MF458657;Lepidoptera;Coenonympha oedippus;Coenonympha oedippus;1;Coenonympha oedippus;0.999999999999915;
MF458658;Lepidoptera;Coenonympha oedippus;Coenonympha oedippus;1;Coenonympha oedippus;0.999999999999915;
MF458706;Lepidoptera;Euphydryas aurinia;Euphydryas aurinia;0.021;Euphydryas aurinia;0.99523814349476;
MG521930;Lepidoptera;Boloria titania;Boloria titania;0.001;Boloria titania;0.99983358374111;
MG521935;Lepidoptera;Lycaena virgaureae;NA;0.186;Lycaena virgaureae;0.999491227342155;
MG521937;Lepidoptera;Cupido minimus;NA;0.003;Cupido minimus;0.999853615233319;
MG521946;Lepidoptera;Cyaniris semiargus;Cyaniris semiargus;NA;Cyaniris semiargus;0.999858415559969;
MG521962;Lepidoptera;Cupido minimus;NA;0.009;Cupido minimus;0.999791453712643;
MG521992;Lepidoptera;Cupido minimus;NA;0.009;Cupido minimus;0.999791453712643;
MG522001;Lepidoptera;Lycaena virgaureae;NA;0.076;Lycaena virgaureae;0.999570679451439;
MG522002;Lepidoptera;Boloria dia;Boloria dia;1;Boloria dia;0.999999999930935;
MG522003;Lepidoptera;Lycaena virgaureae;NA;0.186;Lycaena virgaureae;0.999491227342155;
MG522029;Lepidoptera;Cupido minimus;NA;0.005;Cupido minimus;0.999638915488394;
MG522062;Lepidoptera;Boloria titania;Boloria titania;1;Boloria titania;0.999891597397718;
MG522081;Lepidoptera;Cyaniris semiargus;NA;NA;Cyaniris semiargus;0.999051841766953;
MG522099;Lepidoptera;Brenthis ino;Brenthis ino;0.004;Brenthis ino;0.988611064611184;
MG522124;Lepidoptera;Cyaniris semiargus;NA;NA;Cyaniris semiargus;0.999051841766953;
MG522137;Lepidoptera;Pontia callidice;NA;0.065;Pontia callidice;0.997856462024924;
MG522142;Lepidoptera;Aricia eumedon;Eumedonia eumedon;0.002;Eumedonia eumedon;0.998285932369328;
MG522147;Lepidoptera;Aricia eumedon;Eumedonia eumedon;0.03;Eumedonia eumedon;0.973536015583616;
MG522150;Lepidoptera;Coenonympha glycerion;NA;0.153;Coenonympha glycerion;0.999998511579803;
MG522157;Lepidoptera;Cupido minimus;NA;0.001;Cupido minimus;0.982773593972912;
MG522171;Lepidoptera;Aricia eumedon;Eumedonia eumedon;0.002;Eumedonia eumedon;0.997595421160923;
MG522178;Lepidoptera;Cupido minimus;NA;0.009;Cupido minimus;0.999791453712643;
MG522192;Lepidoptera;Coenonympha glycerion;NA;0.295;Coenonympha glycerion;0.999998958157621;
MG522195;Lepidoptera;Lycaena virgaureae;NA;0.069;Lycaena virgaureae;0.999558016312736;
MG522204;Lepidoptera;Pontia callidice;Pontia callidice;1;Pontia callidice;0.999999999870283;
MG522224;Lepidoptera;Boloria dia;Boloria dia;1;Boloria dia;0.999999999948301;
MG522227;Lepidoptera;Coenonympha glycerion;NA;0.264;Coenonympha glycerion;0.999998370144304;
MG522242;Lepidoptera;Pontia callidice;Pontia callidice;1;Pontia callidice;0.99999999919703;
MG522253;Lepidoptera;Cupido minimus;NA;0.009;Cupido minimus;0.999791453712643;
MG522254;Lepidoptera;Boloria titania;Boloria titania;0.001;Boloria titania;0.99983358374111;
MG522260;Lepidoptera;Aricia eumedon;Eumedonia eumedon;0.006;Eumedonia eumedon;0.996647121119569;
MG522268;Lepidoptera;Cupido minimus;NA;0.006;Cupido minimus;0.999768371759258;
MG522277;Lepidoptera;Cupido minimus;NA;0.001;Cupido minimus;0.999828420231565;
MG522291;Lepidoptera;Cyaniris semiargus;NA;NA;Cyaniris semiargus;0.999051841766953;
MG522295;Lepidoptera;Cupido minimus;NA;0.009;Cupido minimus;0.999791453712643;
MG522299;Lepidoptera;Aricia eumedon;NA;0.202;Eumedonia eumedon;0.785331104416398;
MG522314;Lepidoptera;Coenonympha glycerion;NA;0.264;Coenonympha glycerion;0.999998370144304;
MG522336;Lepidoptera;Boloria dia;Boloria dia;1;Boloria dia;0.999999999980503;
MG522342;Lepidoptera;Aricia eumedon;NA;0.497;Eumedonia eumedon;0.726749102481048;
MG522351;Lepidoptera;Cupido minimus;NA;0.004;Cupido minimus;0.99987402211542;
MG522352;Lepidoptera;Lycaena virgaureae;NA;0.186;Lycaena virgaureae;0.999491227342155;
MG522365;Lepidoptera;Cupido minimus;NA;0.003;Cupido minimus;0.99967591695379;
MG522374;Lepidoptera;Boloria dia;Boloria dia;1;Boloria dia;0.999999999980503;
MG522376;Lepidoptera;Boloria titania;Boloria titania;0.999;Boloria titania;0.999431261609546;
MG522386;Lepidoptera;Coenonympha glycerion;NA;0.295;Coenonympha glycerion;0.999998958157621;
MG522392;Lepidoptera;Boloria titania;NA;0.075;Boloria chariclea;0.875902760689287;
MG522398;Lepidoptera;Pontia callidice;Pontia callidice;1;Pontia callidice;0.999999997508183;
MG522399;Lepidoptera;Lycaena virgaureae;NA;0.073;Lycaena virgaureae;0.9995563158025;
MG522431;Lepidoptera;Lycaena virgaureae;NA;0.115;Lycaena virgaureae;0.999471885087677;
MG522453;Lepidoptera;Brenthis ino;Brenthis ino;0.001;Brenthis ino;0.989101365668547;
MG522456;Lepidoptera;Cupido minimus;NA;0.002;Cupido minimus;0.999824438611123;
MG522461;Lepidoptera;Cupido minimus;NA;0.006;Cupido minimus;0.999768371759258;
MG522492;Lepidoptera;Lycaena virgaureae;NA;0.076;Lycaena virgaureae;0.999570679451439;
MG522502;Lepidoptera;Boloria titania;NA;0.075;Boloria chariclea;0.875902760689287;
MG522518;Lepidoptera;Boloria dia;Boloria dia;1;Boloria dia;0.999999999980503;

MG522538;Lepidoptera;Cupido minimus;NA;0.006;Cupido minimus;0.999768371759258;
MG522545;Lepidoptera;Aricia eumedon;NA;0.497;Eumedonia eumedon;0.726749102481048;
MG522551;Lepidoptera;Cupido minimus;NA;0.009;Cupido minimus;0.999791453712643;
MG522564;Lepidoptera;Brenthis ino;NA;0.031;Brenthis ino;0.796814350318661;
MG522583;Lepidoptera;Lycaena virgaureae;NA;0.186;Lycaena virgaureae;0.999491227342155;
MG522584;Lepidoptera;Lycaena virgaureae;NA;0.186;Lycaena virgaureae;0.999491227342155;
MG522589;Lepidoptera;Lycaena virgaureae;NA;0.186;Lycaena virgaureae;0.999491227342155;
MG522603;Lepidoptera;Cupido minimus;NA;0.006;Cupido minimus;0.999768371759258;
MG522604;Lepidoptera;Boloria titania;NA;0.075;Boloria chariclea;0.875902760689287;
MG522609;Lepidoptera;Brenthis ino;Brenthis ino;0.001;Brenthis ino;0.994711912827522;
MG522621;Lepidoptera;Brenthis ino;Brenthis ino;0.007;Brenthis ino;0.977157381961834;
MG522629;Lepidoptera;Brenthis ino;Brenthis ino;0.001;Brenthis ino;0.998434780405276;
MG522639;Lepidoptera;Lycaena virgaureae;NA;0.217;Lycaena virgaureae;0.999511105136248;
MG522654;Lepidoptera;Aricia eumedon;Eumedonia eumedon;0.003;Eumedonia eumedon;0.997225950391515;
MG522679;Lepidoptera;Brenthis ino;Brenthis ino;0.007;Brenthis ino;0.977157381961834;
MG522697;Lepidoptera;Boloria titania;Boloria titania;0.001;Boloria titania;0.99983358374111;
MG522700;Lepidoptera;Aricia eumedon;Eumedonia eumedon;0.002;Eumedonia eumedon;0.997595421160923;
MG522702;Lepidoptera;Boloria dia;Boloria dia;1;Boloria dia;0.999999999980503;
MG522707;Lepidoptera;Brenthis ino;Brenthis ino;0.001;Brenthis ino;0.994711912827522;
MG522711;Lepidoptera;Lycaena virgaureae;NA;0.186;Lycaena virgaureae;0.999491227342155;
MG522721;Lepidoptera;Aricia eumedon;Eumedonia eumedon;0.003;Eumedonia eumedon;0.999165302110657;
MG522726;Lepidoptera;Brenthis ino;Brenthis ino;0.001;Brenthis ino;0.994711912827522;
MG522727;Lepidoptera;Coenonympha glycerion;NA;0.295;Coenonympha glycerion;0.999998958157621;
MG522744;Lepidoptera;Boloria dia;Boloria dia;1;Boloria dia;0.999999999980503;
MG522770;Lepidoptera;Lycaena virgaureae;NA;0.073;Lycaena virgaureae;0.9995563158025;
MG522785;Lepidoptera;Pontia callidice;Pontia callidice;1;Pontia callidice;0.999999997508183;
MG522806;Lepidoptera;Brenthis ino;Brenthis ino;0.001;Brenthis ino;0.994711912827522;
MG522815;Lepidoptera;Boloria dia;Boloria dia;1;Boloria dia;0.999999999980503;
MG522825;Lepidoptera;Lycaena virgaureae;NA;0.186;Lycaena virgaureae;0.999491227342155;
MG675184;Lepidoptera;Euphydryas aurinia;Euphydryas aurinia;0.023;Euphydryas aurinia;0.99260940098197;
MG675185;Lepidoptera;Euphydryas aurinia;Euphydryas aurinia;0.023;Euphydryas aurinia;0.99260940098197;
MG675186;Lepidoptera;Euphydryas aurinia;Euphydryas aurinia;0.023;Euphydryas aurinia;0.99260940098197;
MG675187;Lepidoptera;Euphydryas aurinia;NA;0.002;Euphydryas aurinia;0.962966658044978;
MG675189;Lepidoptera;Euphydryas aurinia;NA;0.002;Euphydryas aurinia;0.962966658044978;
MG675207;Lepidoptera;Euphydryas aurinia;NA;0.002;Euphydryas aurinia;0.962966658044978;
MG675208;Lepidoptera;Euphydryas aurinia;NA;0.002;Euphydryas aurinia;0.962966658044978;
MG675209;Lepidoptera;Euphydryas aurinia;NA;0.002;Euphydryas aurinia;0.962966658044978;
MG675210;Lepidoptera;Euphydryas aurinia;NA;0.002;Euphydryas aurinia;0.962966658044978;
MG675211;Lepidoptera;Euphydryas aurinia;NA;0.002;Euphydryas aurinia;0.976250031560894;
MG675212;Lepidoptera;Euphydryas aurinia;NA;0.002;Euphydryas aurinia;0.962966658044978;
MG675213;Lepidoptera;Euphydryas aurinia;NA;0.002;Euphydryas aurinia;0.962966658044978;
MG675214;Lepidoptera;Euphydryas aurinia;NA;0.138;Euphydryas aurinia;0.984163691779491;
MG675215;Lepidoptera;Euphydryas aurinia;NA;0.002;Euphydryas aurinia;0.976250031560894;
MG675216;Lepidoptera;Euphydryas aurinia;NA;0.002;Euphydryas aurinia;0.976250031560894;
MG675217;Lepidoptera;Euphydryas aurinia;NA;0.002;Euphydryas aurinia;0.976250031560894;
MG675218;Lepidoptera;Euphydryas aurinia;NA;0.002;Euphydryas aurinia;0.976250031560894;
MH089587;Lepidoptera;Pararge aegeria;Pararge aegeria;1;Pararge aegeria;0.999999999963165;
MH089588;Lepidoptera;Pararge aegeria;Pararge aegeria;1;Pararge aegeria;0.999999999963165;
MH089589;Lepidoptera;Pararge aegeria;Pararge aegeria;1;Pararge aegeria;0.999999999963165;
MH089590;Lepidoptera;Pararge aegeria;Pararge aegeria;1;Pararge aegeria;0.999999999963165;
MH089591;Lepidoptera;Pararge aegeria;Pararge aegeria;1;Pararge aegeria;0.999999999963165;
MH089592;Lepidoptera;Pararge aegeria;Pararge aegeria;1;Pararge aegeria;0.999999999963165;
MH089593;Lepidoptera;Pararge aegeria;Pararge aegeria;1;Pararge aegeria;0.999999999975955;
MH089594;Lepidoptera;Pararge aegeria;Pararge aegeria;1;Pararge aegeria;0.999999998847642;
MH089595;Lepidoptera;Pararge aegeria;Pararge aegeria;1;Pararge aegeria;0.999999999963165;
MH089596;Lepidoptera;Pararge aegeria;Pararge aegeria;1;Pararge aegeria;0.999999999963165;
MH089597;Lepidoptera;Pararge aegeria;Pararge aegeria;1;Pararge aegeria;0.999999999836319;
MH089598;Lepidoptera;Pararge aegeria;Pararge aegeria;1;Pararge aegeria;0.999999999365514;
MH089599;Lepidoptera;Pararge aegeria;Pararge aegeria;1;Pararge aegeria;0.999999970113123;
MH089600;Lepidoptera;Pararge aegeria;Pararge aegeria;1;Pararge aegeria;0.999999999963165;
MH089601;Lepidoptera;Pararge aegeria;Pararge aegeria;1;Pararge aegeria;0.999999999963165;
MH089602;Lepidoptera;Pararge aegeria;Pararge aegeria;1;Pararge aegeria;0.999999999932498;
MH089604;Lepidoptera;Pararge aegeria;Pararge aegeria;1;Pararge aegeria;0.999999999963165;
MH089605;Lepidoptera;Pararge aegeria;Pararge aegeria;1;Pararge aegeria;0.999999999963165;
MH089606;Lepidoptera;Pararge aegeria;Pararge aegeria;1;Pararge aegeria;0.999999999963165;
MH089608;Lepidoptera;Pararge aegeria;Pararge aegeria;1;Pararge aegeria;0.99999999997371;
MH089609;Lepidoptera;Pararge aegeria;Pararge aegeria;1;Pararge aegeria;0.999999999963165;
MH089610;Lepidoptera;Pararge aegeria;Pararge aegeria;1;Pararge aegeria;0.999999999963165;
MH089612;Lepidoptera;Pararge aegeria;Pararge aegeria;1;Pararge aegeria;0.999999999963165;
MH089613;Lepidoptera;Pararge aegeria;Pararge aegeria;1;Pararge aegeria;0.999999999963165;
MH089614;Lepidoptera;Pararge aegeria;Pararge aegeria;1;Pararge aegeria;0.999999999342521;
MH089615;Lepidoptera;Pararge aegeria;Pararge aegeria;1;Pararge aegeria;0.999999999963165;
MH089616;Lepidoptera;Pararge aegeria;Pararge aegeria;1;Pararge aegeria;0.999999999963165;
MH089617;Lepidoptera;Pararge aegeria;Pararge aegeria;1;Pararge aegeria;0.999999999762906;
MH089618;Lepidoptera;Pararge aegeria;Pararge aegeria;1;Pararge aegeria;0.999999999963165;

MH089619;Lepidoptera;Pararge aegeria;Pararge aegeria;1;Pararge aegeria;0.999999997556188;
MH089620;Lepidoptera;Pararge aegeria;Pararge aegeria;1;Pararge aegeria;0.999999999963165;
MH089621;Lepidoptera;Pararge aegeria;Pararge aegeria;1;Pararge aegeria;0.999999999605791;
MH089622;Lepidoptera;Pararge aegeria;Pararge aegeria;1;Pararge aegeria;0.999999999963165;
MH089623;Lepidoptera;Pararge aegeria;Pararge aegeria;1;Pararge aegeria;0.999999999963165;
MH089625;Lepidoptera;Pararge aegeria;Pararge aegeria;1;Pararge aegeria;0.999999999963165;
MH089626;Lepidoptera;Pararge aegeria;Pararge aegeria;1;Pararge aegeria;0.999999999963165;
MH089627;Lepidoptera;Pararge aegeria;Pararge aegeria;1;Pararge aegeria;0.999999999805141;
MH089628;Lepidoptera;Pararge aegeria;Pararge aegeria;1;Pararge aegeria;0.999999999932498;
MH089629;Lepidoptera;Pararge aegeria;Pararge aegeria;1;Pararge aegeria;0.999999999921556;
MH089630;Lepidoptera;Pararge aegeria;Pararge aegeria;1;Pararge aegeria;0.999999999530445;
MH089633;Lepidoptera;Pararge aegeria;Pararge aegeria;1;Pararge aegeria;0.999999999963165;
MH089634;Lepidoptera;Pararge aegeria;Pararge aegeria;1;Pararge aegeria;0.999999999963165;
MH089636;Lepidoptera;Pararge aegeria;Pararge aegeria;1;Pararge aegeria;0.999999999963165;
MH089637;Lepidoptera;Pararge aegeria;Pararge aegeria;1;Pararge aegeria;0.999999999963165;
MH089638;Lepidoptera;Pararge aegeria;Pararge aegeria;1;Pararge aegeria;0.999999999804942;
MH089639;Lepidoptera;Pararge aegeria;Pararge aegeria;1;Pararge aegeria;0.999999999963165;
MH089640;Lepidoptera;Pararge aegeria;Pararge aegeria;1;Pararge aegeria;0.999999999963165;
MH089642;Lepidoptera;Pararge aegeria;Pararge aegeria;1;Pararge aegeria;0.999999999762906;
MH089643;Lepidoptera;Pararge aegeria;Pararge aegeria;1;Pararge aegeria;0.999999999805141;
MH089644;Lepidoptera;Pararge aegeria;Pararge aegeria;1;Pararge aegeria;0.999999999791413;
MH089645;Lepidoptera;Pararge aegeria;Pararge aegeria;1;Pararge aegeria;0.999999999963165;
MH089646;Lepidoptera;Pararge aegeria;Pararge aegeria;1;Pararge aegeria;0.999999999963165;
MH089647;Lepidoptera;Pararge aegeria;Pararge aegeria;1;Pararge aegeria;0.999999999963165;
MH089648;Lepidoptera;Pararge aegeria;Pararge aegeria;1;Pararge aegeria;0.999999999963165;
MH089649;Lepidoptera;Pararge aegeria;Pararge aegeria;1;Pararge aegeria;0.999999999963165;
MH089650;Lepidoptera;Pararge aegeria;Pararge aegeria;1;Pararge aegeria;0.999999999963165;
MH089651;Lepidoptera;Pararge aegeria;Pararge aegeria;1;Pararge aegeria;0.999999999919339;
MH089652;Lepidoptera;Pararge aegeria;Pararge aegeria;1;Pararge aegeria;0.999999999963165;
MH089653;Lepidoptera;Pararge aegeria;Pararge aegeria;1;Pararge aegeria;0.999999999963165;
MH089654;Lepidoptera;Pararge aegeria;Pararge aegeria;1;Pararge aegeria;0.999999999365514;
MH089655;Lepidoptera;Pararge aegeria;Pararge aegeria;1;Pararge aegeria;0.999999999963165;
MH089656;Lepidoptera;Pararge aegeria;Pararge aegeria;1;Pararge aegeria;0.999999999963165;
MH089657;Lepidoptera;Pararge aegeria;Pararge aegeria;1;Pararge aegeria;0.999999999914024;
MH089658;Lepidoptera;Pararge aegeria;Pararge aegeria;1;Pararge aegeria;0.999999999805141;
MH089659;Lepidoptera;Pararge aegeria;Pararge aegeria;1;Pararge aegeria;0.999999999365514;
MH089660;Lepidoptera;Pararge aegeria;Pararge aegeria;1;Pararge aegeria;0.999999999963165;
MH089661;Lepidoptera;Pararge aegeria;Pararge aegeria;1;Pararge aegeria;0.999999999963165;
MH089663;Lepidoptera;Pararge aegeria;Pararge aegeria;1;Pararge aegeria;0.999999997743117;
MH089664;Lepidoptera;Pararge aegeria;Pararge aegeria;1;Pararge aegeria;0.999999999963165;
MH089665;Lepidoptera;Pararge aegeria;Pararge aegeria;1;Pararge aegeria;0.999999948278516;
MH089666;Lepidoptera;Pararge aegeria;Pararge aegeria;1;Pararge aegeria;0.999999999919339;
MH089667;Lepidoptera;Pararge aegeria;Pararge aegeria;1;Pararge aegeria;0.999999999963165;
MH089668;Lepidoptera;Pararge aegeria;Pararge aegeria;1;Pararge aegeria;0.999999999962398;
MH089669;Lepidoptera;Pararge aegeria;Pararge aegeria;1;Pararge aegeria;0.999999999963165;
MH089670;Lepidoptera;Pararge aegeria;Pararge aegeria;1;Pararge aegeria;0.999999999963165;
MH089671;Lepidoptera;Pararge aegeria;Pararge aegeria;1;Pararge aegeria;0.99999999985576;
MH089672;Lepidoptera;Pararge aegeria;Pararge aegeria;1;Pararge aegeria;0.999999999963165;
MH089674;Lepidoptera;Pararge aegeria;Pararge aegeria;1;Pararge aegeria;0.999999999963165;
MH089675;Lepidoptera;Pararge aegeria;Pararge aegeria;1;Pararge aegeria;0.999999999963165;
MH089676;Lepidoptera;Pararge aegeria;Pararge aegeria;1;Pararge aegeria;0.999999999963165;
MH089677;Lepidoptera;Pararge aegeria;Pararge aegeria;1;Pararge aegeria;0.999999999963165;
MH089678;Lepidoptera;Pararge aegeria;Pararge aegeria;1;Pararge aegeria;0.999999999805141;
MH089680;Lepidoptera;Pararge aegeria;Pararge aegeria;1;Pararge aegeria;0.999999999932498;
MH089681;Lepidoptera;Pararge aegeria;Pararge aegeria;1;Pararge aegeria;0.999999999963165;
MH089682;Lepidoptera;Pararge aegeria;Pararge aegeria;1;Pararge aegeria;0.999999999963165;
MH089683;Lepidoptera;Pararge aegeria;Pararge aegeria;1;Pararge aegeria;0.999999999963165;
MH089684;Lepidoptera;Pararge aegeria;Pararge aegeria;1;Pararge aegeria;0.999999999805141;
MH089686;Lepidoptera;Pararge aegeria;Pararge aegeria;1;Pararge aegeria;0.999999999963165;
MH089689;Lepidoptera;Pararge aegeria;Pararge aegeria;1;Pararge aegeria;0.999999999365514;
MH089690;Lepidoptera;Pararge aegeria;Pararge aegeria;1;Pararge aegeria;0.999999999951228;
MH089691;Lepidoptera;Pararge aegeria;Pararge aegeria;1;Pararge aegeria;0.999999999963165;
MH089693;Lepidoptera;Pararge aegeria;Pararge aegeria;1;Pararge aegeria;0.999999999963165;
MH089694;Lepidoptera;Pararge aegeria;Pararge aegeria;1;Pararge aegeria;0.999999994901117;
MH089695;Lepidoptera;Pararge aegeria;Pararge aegeria;1;Pararge aegeria;0.999999999963165;
MH089696;Lepidoptera;Pararge aegeria;Pararge aegeria;1;Pararge aegeria;0.999999999123304;
MH089697;Lepidoptera;Pararge aegeria;Pararge aegeria;1;Pararge aegeria;0.999999999963165;
MH089698;Lepidoptera;Pararge aegeria;Pararge aegeria;1;Pararge aegeria;0.999999999963165;
MH089699;Lepidoptera;Pararge aegeria;Pararge aegeria;1;Pararge aegeria;0.999999999963165;
MH089700;Lepidoptera;Pararge aegeria;Pararge aegeria;1;Pararge aegeria;0.999999999963165;
MH089701;Lepidoptera;Pararge aegeria;Pararge aegeria;1;Pararge aegeria;0.999999999963165;
MH089702;Lepidoptera;Pararge aegeria;Pararge aegeria;1;Pararge aegeria;0.999999999342521;
MH089703;Lepidoptera;Pararge aegeria;Pararge aegeria;1;Pararge aegeria;0.999999999929429;
MH089704;Lepidoptera;Pararge aegeria;Pararge aegeria;1;Pararge aegeria;0.999999999963165;
MH089706;Lepidoptera;Pararge aegeria;Pararge aegeria;1;Pararge aegeria;0.999999999919339;
MH089707;Lepidoptera;Pararge aegeria;Pararge aegeria;1;Pararge aegeria;0.999999999963165;
MH089708;Lepidoptera;Pararge aegeria;Pararge aegeria;1;Pararge aegeria;0.999999999963165;
MH089709;Lepidoptera;Pararge aegeria;Pararge aegeria;1;Pararge aegeria;0.999999999963165;

MH089710;Lepidoptera;Pararge aegeria;Pararge aegeria;1;Pararge aegeria;0.999999999963165;
MH089711;Lepidoptera;Pararge aegeria;Pararge aegeria;1;Pararge aegeria;0.999999999963165;
MH089714;Lepidoptera;Pararge aegeria;Pararge aegeria;1;Pararge aegeria;0.999999999963165;
MH089715;Lepidoptera;Pararge aegeria;Pararge aegeria;1;Pararge aegeria;0.999999999963165;
MH089716;Lepidoptera;Pararge aegeria;Pararge aegeria;1;Pararge aegeria;0.999999999963165;
MH089717;Lepidoptera;Pararge aegeria;Pararge aegeria;1;Pararge aegeria;0.999999999963165;
MH089718;Lepidoptera;Pararge aegeria;Pararge aegeria;1;Pararge aegeria;0.999999999963165;
MH089720;Lepidoptera;Pararge aegeria;Pararge aegeria;1;Pararge aegeria;0.999999999963165;
MH089721;Lepidoptera;Pararge aegeria;Pararge aegeria;1;Pararge aegeria;0.999999999365514;
MH089722;Lepidoptera;Pararge aegeria;Pararge aegeria;1;Pararge aegeria;0.999999999963165;
MH089723;Lepidoptera;Pararge aegeria;Pararge aegeria;1;Pararge aegeria;0.999999999310262;
MH089726;Lepidoptera;Pararge aegeria;Pararge aegeria;1;Pararge aegeria;0.999999999963165;
MH089727;Lepidoptera;Pararge aegeria;Pararge aegeria;1;Pararge aegeria;0.999999999963165;
MH089728;Lepidoptera;Pararge aegeria;Pararge aegeria;1;Pararge aegeria;0.999999999963165;
MH089729;Lepidoptera;Pararge aegeria;Pararge aegeria;1;Pararge aegeria;0.999999999365514;
MH089731;Lepidoptera;Pararge aegeria;Pararge aegeria;1;Pararge aegeria;0.999999999963165;
MH089732;Lepidoptera;Pararge aegeria;Pararge aegeria;1;Pararge aegeria;0.999999999963165;
MH089733;Lepidoptera;Pararge aegeria;Pararge aegeria;1;Pararge aegeria;0.999999999197257;
MH089734;Lepidoptera;Pararge aegeria;Pararge aegeria;1;Pararge aegeria;0.999999999919339;
MH089736;Lepidoptera;Pararge aegeria;Pararge aegeria;1;Pararge aegeria;0.999999999963165;
MH089737;Lepidoptera;Pararge aegeria;Pararge aegeria;1;Pararge aegeria;0.999999999963165;
MH089738;Lepidoptera;Pararge aegeria;Pararge aegeria;1;Pararge aegeria;0.999999999310262;
MH089739;Lepidoptera;Pararge aegeria;Pararge aegeria;1;Pararge aegeria;0.999999999963165;
MH089740;Lepidoptera;Pararge aegeria;Pararge aegeria;1;Pararge aegeria;0.999999999169063;
MH089741;Lepidoptera;Pararge aegeria;Pararge aegeria;1;Pararge aegeria;0.999999999365514;
MH089742;Lepidoptera;Pararge aegeria;Pararge aegeria;1;Pararge aegeria;0.999999999963165;
MH089743;Lepidoptera;Pararge aegeria;Pararge aegeria;1;Pararge aegeria;0.999999999919339;
MH089745;Lepidoptera;Pararge aegeria;Pararge aegeria;1;Pararge aegeria;0.999999999805141;
MH089746;Lepidoptera;Pararge aegeria;Pararge aegeria;1;Pararge aegeria;0.999999999963165;
MH089747;Lepidoptera;Pararge aegeria;Pararge aegeria;1;Pararge aegeria;0.999999999963165;
MH089748;Lepidoptera;Pararge aegeria;Pararge aegeria;1;Pararge aegeria;0.999999999978456;
MH089749;Lepidoptera;Pararge aegeria;Pararge aegeria;1;Pararge aegeria;0.999999999963165;
MH089750;Lepidoptera;Pararge aegeria;Pararge aegeria;1;Pararge aegeria;0.999999999963165;
MH089751;Lepidoptera;Pararge aegeria;Pararge aegeria;1;Pararge aegeria;0.999999999963165;
MH089752;Lepidoptera;Pararge aegeria;Pararge aegeria;1;Pararge aegeria;0.999999999951228;
MH089753;Lepidoptera;Pararge aegeria;Pararge aegeria;1;Pararge aegeria;0.999999999972374;
MH089754;Lepidoptera;Pararge aegeria;Pararge aegeria;1;Pararge aegeria;0.999999999963165;
MH089755;Lepidoptera;Pararge aegeria;Pararge aegeria;1;Pararge aegeria;0.999999999963165;
MH089756;Lepidoptera;Pararge aegeria;Pararge aegeria;1;Pararge aegeria;0.999999999805141;
MH089757;Lepidoptera;Pararge aegeria;Pararge aegeria;1;Pararge aegeria;0.999999999836319;
MH089758;Lepidoptera;Pararge aegeria;Pararge aegeria;1;Pararge aegeria;0.99999993568966;
MH089759;Lepidoptera;Pararge aegeria;Pararge aegeria;1;Pararge aegeria;0.999999999963165;
MH089760;Lepidoptera;Pararge aegeria;Pararge aegeria;1;Pararge aegeria;0.999999998455337;
MH089761;Lepidoptera;Pararge aegeria;Pararge aegeria;1;Pararge aegeria;0.999999999963165;
MH089762;Lepidoptera;Pararge aegeria;Pararge aegeria;1;Pararge aegeria;0.999999999963165;
MH089763;Lepidoptera;Pararge aegeria;Pararge aegeria;1;Pararge aegeria;0.999999999975955;
MH089764;Lepidoptera;Pararge aegeria;Pararge aegeria;1;Pararge aegeria;0.999999999963165;
MH089765;Lepidoptera;Pararge aegeria;Pararge aegeria;1;Pararge aegeria;0.999999999910472;
MH089766;Lepidoptera;Pararge aegeria;Pararge aegeria;1;Pararge aegeria;0.999999999963165;
MH089767;Lepidoptera;Pararge aegeria;Pararge aegeria;1;Pararge aegeria;0.999999999963165;
MH089768;Lepidoptera;Pararge aegeria;Pararge aegeria;1;Pararge aegeria;0.999999999963165;
MH089769;Lepidoptera;Pararge aegeria;Pararge aegeria;1;Pararge aegeria;0.999999999963165;
MH089770;Lepidoptera;Pararge aegeria;Pararge aegeria;1;Pararge aegeria;0.999999999963165;
MH089771;Lepidoptera;Pararge aegeria;Pararge aegeria;1;Pararge aegeria;0.999999999903309;
MH089772;Lepidoptera;Pararge aegeria;Pararge aegeria;1;Pararge aegeria;0.999999999963165;
MH089774;Lepidoptera;Pararge aegeria;Pararge aegeria;1;Pararge aegeria;0.999999999963165;
MH089775;Lepidoptera;Pararge aegeria;Pararge aegeria;1;Pararge aegeria;0.999999999963165;
MH089776;Lepidoptera;Pararge aegeria;Pararge aegeria;1;Pararge aegeria;0.999999999963165;
MH089777;Lepidoptera;Pararge aegeria;Pararge aegeria;1;Pararge aegeria;0.999999999963165;
MH089778;Lepidoptera;Pararge aegeria;Pararge aegeria;1;Pararge aegeria;0.999999999963165;
MH089779;Lepidoptera;Pararge aegeria;Pararge aegeria;1;Pararge aegeria;0.999999999805141;
MH089780;Lepidoptera;Pararge aegeria;Pararge aegeria;1;Pararge aegeria;0.999999999963165;
MH089781;Lepidoptera;Pararge aegeria;Pararge aegeria;1;Pararge aegeria;0.999999999963165;
MH089782;Lepidoptera;Pararge aegeria;Pararge aegeria;1;Pararge aegeria;0.999999999963165;
MH089783;Lepidoptera;Pararge aegeria;Pararge aegeria;1;Pararge aegeria;0.999999999963165;
MH089784;Lepidoptera;Pararge aegeria;Pararge aegeria;1;Pararge aegeria;0.999999999963165;
MH089785;Lepidoptera;Pararge aegeria;Pararge aegeria;1;Pararge aegeria;0.999999999963165;
MH089787;Lepidoptera;Pararge aegeria;Pararge aegeria;1;Pararge aegeria;0.999999999963165;
MH089788;Lepidoptera;Pararge aegeria;Pararge aegeria;1;Pararge aegeria;0.999999999963165;
MH089789;Lepidoptera;Pararge aegeria;Pararge aegeria;1;Pararge aegeria;0.999999999963165;
MH089790;Lepidoptera;Pararge aegeria;Pararge aegeria;1;Pararge aegeria;0.999999999963165;
MH089791;Lepidoptera;Pararge aegeria;Pararge aegeria;1;Pararge aegeria;0.999999999955861;
MH089792;Lepidoptera;Pararge aegeria;Pararge aegeria;1;Pararge aegeria;0.999999999963165;
MH089793;Lepidoptera;Pararge aegeria;Pararge aegeria;1;Pararge aegeria;0.999999999963165;
MH089794;Lepidoptera;Pararge aegeria;Pararge aegeria;1;Pararge aegeria;0.999999999963165;
MH089795;Lepidoptera;Pararge aegeria;Pararge aegeria;1;Pararge aegeria;0.999999999963165;
MH089796;Lepidoptera;Pararge aegeria;Pararge aegeria;1;Pararge aegeria;0.999999999963165;
MH089797;Lepidoptera;Pararge aegeria;Pararge aegeria;1;Pararge aegeria;0.999999999456179;

MH089798;Lepidoptera;Pararge aegeria;Pararge aegeria;1;Pararge aegeria;0.999999999963165;
MH089799;Lepidoptera;Pararge aegeria;Pararge aegeria;1;Pararge aegeria;0.999999999963165;
MH089800;Lepidoptera;Pararge aegeria;Pararge aegeria;1;Pararge aegeria;0.999999999963165;
MH089801;Lepidoptera;Pararge aegeria;Pararge aegeria;1;Pararge aegeria;0.999999999963165;
MH089802;Lepidoptera;Pararge aegeria;Pararge aegeria;1;Pararge aegeria;0.999999999963165;
MH089803;Lepidoptera;Pararge aegeria;Pararge aegeria;1;Pararge aegeria;0.999999999963165;
MH089804;Lepidoptera;Pararge aegeria;Pararge aegeria;1;Pararge aegeria;0.999999999967883;
MH089805;Lepidoptera;Pararge aegeria;Pararge aegeria;1;Pararge aegeria;0.999999999963165;
MH089806;Lepidoptera;Pararge aegeria;Pararge aegeria;1;Pararge aegeria;0.999999999963165;
MH089807;Lepidoptera;Pararge aegeria;Pararge aegeria;1;Pararge aegeria;0.999999999869857;
MH089808;Lepidoptera;Pararge aegeria;Pararge aegeria;1;Pararge aegeria;0.999999999963165;
MH089809;Lepidoptera;Pararge aegeria;Pararge aegeria;1;Pararge aegeria;0.999999999963165;
MH089810;Lepidoptera;Pararge aegeria;Pararge aegeria;1;Pararge aegeria;0.999999999975955;
MH089811;Lepidoptera;Pararge aegeria;Pararge aegeria;1;Pararge aegeria;0.999999999963165;
MH089812;Lepidoptera;Pararge aegeria;Pararge aegeria;1;Pararge aegeria;0.999999999963165;
MH089813;Lepidoptera;Pararge aegeria;Pararge aegeria;1;Pararge aegeria;0.999999999963165;
MH089814;Lepidoptera;Pararge aegeria;Pararge aegeria;1;Pararge aegeria;0.999999999963165;
MH089815;Lepidoptera;Pararge aegeria;Pararge aegeria;1;Pararge aegeria;0.999999999963165;
MH089816;Lepidoptera;Pararge aegeria;Pararge aegeria;1;Pararge aegeria;0.999999999963165;
MH089817;Lepidoptera;Pararge aegeria;Pararge aegeria;1;Pararge aegeria;0.999999965384661;
MH089819;Lepidoptera;Pararge aegeria;Pararge aegeria;1;Pararge aegeria;0.999999999963165;
MH089820;Lepidoptera;Pararge aegeria;Pararge aegeria;1;Pararge aegeria;0.999999999169063;
MH089821;Lepidoptera;Pararge aegeria;Pararge aegeria;1;Pararge aegeria;0.999999999963165;
MH089822;Lepidoptera;Pararge aegeria;Pararge aegeria;1;Pararge aegeria;0.999999999963165;
MH089823;Lepidoptera;Pararge aegeria;Pararge aegeria;1;Pararge aegeria;0.999999999963165;
MH089824;Lepidoptera;Pararge aegeria;Pararge aegeria;1;Pararge aegeria;0.999999999963165;
MH089825;Lepidoptera;Pararge aegeria;Pararge aegeria;1;Pararge aegeria;0.999999999963165;
MH089826;Lepidoptera;Pararge aegeria;Pararge aegeria;1;Pararge aegeria;0.999999999963165;
MH089827;Lepidoptera;Pararge aegeria;Pararge aegeria;1;Pararge aegeria;0.999999999963165;
MH089828;Lepidoptera;Pararge aegeria;Pararge aegeria;1;Pararge aegeria;0.999999999805141;
MH089830;Lepidoptera;Pararge aegeria;Pararge aegeria;1;Pararge aegeria;0.999999999975955;
MH089831;Lepidoptera;Pararge aegeria;Pararge aegeria;1;Pararge aegeria;0.999999999955435;
MH089832;Lepidoptera;Pararge aegeria;Pararge aegeria;1;Pararge aegeria;0.999999999963165;
MH089833;Lepidoptera;Pararge aegeria;Pararge aegeria;1;Pararge aegeria;0.999999999963165;
MH089834;Lepidoptera;Pararge aegeria;Pararge aegeria;1;Pararge aegeria;0.999999999169063;
MH089835;Lepidoptera;Pararge aegeria;Pararge aegeria;1;Pararge aegeria;0.999999999970726;
MH089836;Lepidoptera;Pararge aegeria;Pararge aegeria;1;Pararge aegeria;0.999999997556188;
MH089837;Lepidoptera;Pararge aegeria;Pararge aegeria;1;Pararge aegeria;0.999999999963165;
MH089838;Lepidoptera;Pararge aegeria;Pararge aegeria;1;Pararge aegeria;0.999999999963165;
MH089839;Lepidoptera;Pararge aegeria;Pararge aegeria;1;Pararge aegeria;0.999999999963165;
MH308299;Lepidoptera;Melanargia galathea;Melanargia galathea;NA;Melanargia galathea;0.991491340615494;
MH308300;Lepidoptera;Melanargia galathea;Melanargia galathea;NA;Melanargia galathea;0.959429932872284;
MH308302;Lepidoptera;Melanargia galathea;Melanargia galathea;NA;Melanargia galathea;0.981855578470499;
MH308326;Lepidoptera;Melanargia galathea;Melanargia galathea;0.004;Melanargia galathea;0.965820796066182;
MH308327;Lepidoptera;Melanargia galathea;Melanargia galathea;NA;Melanargia galathea;0.959429932872284;
MH308330;Lepidoptera;Melanargia galathea;Melanargia galathea;NA;Melanargia galathea;0.959429932872284;
MH308332;Lepidoptera;Melanargia galathea;Melanargia galathea;NA;Melanargia galathea;0.902844057936915;
MH308333;Lepidoptera;Melanargia galathea;Melanargia galathea;NA;Melanargia galathea;0.935799586887365;
MH308334;Lepidoptera;Melanargia galathea;Melanargia galathea;0.001;Melanargia galathea;0.963298866527903;
MH308361;Lepidoptera;Melanargia galathea;Melanargia galathea;NA;Melanargia galathea;0.959429932872284;
MH308362;Lepidoptera;Melanargia galathea;Melanargia galathea;NA;Melanargia galathea;0.959429932872284;
MH407225;Lepidoptera;Scolitantides orion;Scolitantides orion;NA;Scolitantides orion;0.99999996904694;
MH418490;Lepidoptera;Glaucopsyche alexis;Glaucopsyche alexis;0.001;Glaucopsyche alexis;0.94042380088325;
MH418492;Lepidoptera;Lasiommata megera;Lasiommata megera;1;Lasiommata megera;0.999379144055441;
MH418494;Lepidoptera;Hipparchia statilinus;Hipparchia statilinus;1;Hipparchia statilinus;0.999999999999943;
MH418496;Lepidoptera;Lasiommata paramegaera;Lasiommata paramegaera;0.001;Lasiommata paramegaera;0.974943507761532;
MH418497;Lepidoptera;Aricia agestis;NA;0.253;Aricia agestis;0.996350017387504;
MH418501;Lepidoptera;Lasiommata megera;Lasiommata megera;1;Lasiommata megera;0.999379144055441;
MH418502;Lepidoptera;Pieris napi;NA;0.028;Pieris napi;0.888205720664699;
MH418503;Lepidoptera;Melitaea didyma;Melitaea didyma;1;Melitaea didyma;0.999945824175549;
MH418505;Lepidoptera;Lasiommata megera;Lasiommata megera;1;Lasiommata megera;0.999379144055441;
MH418506;Lepidoptera;Coenonympha pamphilus;Coenonympha pamphilus;1;Coenonympha pamphilus;0.999999999999744;

MH418507;Lepidoptera;Callophrys rubi;NA;0.001;Callophrys rubi;0.998885705938309;
MH418509;Lepidoptera;Lycaena phlaeas;Lycaena phlaeas;1;Lycaena phlaeas;0.999999996363186;
MH418510;Lepidoptera;Aglais ichnusa;Aglais ichnusa;0.003;NA;0.999999990022183;
MH418511;Lepidoptera;Maniola cecilia;Pyronia cecilia;NA;Pyronia cecilia;1;
MH418512;Lepidoptera;Callophrys rubi;NA;0.001;Callophrys rubi;0.998885705938309;
MH418513;Lepidoptera;Argynnis paphia;Argynnis paphia;1;Argynnis paphia;0.999999992693603;
MH418516;Lepidoptera;Gegenes pumilio;Gegenes pumilio;1;Gegenes pumilio;0.999986726679266;
MH418517;Lepidoptera;Celastrina argiolus;Celastrina argiolus;1;Celastrina argiolus;0.999527385150531;
MH418521;Lepidoptera;Hipparchia neomiris;Hipparchia neomiris;1;Hipparchia neomiris;0.999999999998693;
MH418522;Lepidoptera;Charaxes jasius;Charaxes jasius;NA;Charaxes jasius;0.990397821479112;
MH418523;Lepidoptera;Maniola cecilia;Pyronia cecilia;NA;Pyronia cecilia;1;
MH418524;Lepidoptera;Lasiommata megera;Lasiommata megera;1;Lasiommata megera;0.999379144055441;
MH418525;Lepidoptera;Colias crocea;NA;0.059;NA;0.999999999999738;
MH418526;Lepidoptera;Lasiommata megera;Lasiommata megera;1;Lasiommata megera;0.999379144055441;
MH418527;Lepidoptera;Thymelicus acteon;Thymelicus acteon;1;Thymelicus acteon;0.999999954806129;
MH418528;Lepidoptera;Charaxes jasius;Charaxes jasius;NA;Charaxes jasius;0.990397821479112;
MH418529;Lepidoptera;Pieris napi;NA;0.011;Pieris napi;0.801985394724814;
MH418530;Lepidoptera;Melitaea cinxia;Melitaea cinxia;1;Melitaea cinxia;0.999999999998749;
MH418532;Lepidoptera;Colias crocea;NA;0.059;NA;0.999999999999738;
MH418533;Lepidoptera;Lampides boeticus;Lampides boeticus;1;Lampides boeticus;0.999999999550823;
MH418534;Lepidoptera;Hipparchia neomiris;Hipparchia neomiris;1;Hipparchia neomiris;0.999999999999204;
MH418535;Lepidoptera;Colias crocea;NA;0.059;NA;0.999999999999738;
MH418537;Lepidoptera;Thymelicus acteon;Thymelicus acteon;1;Thymelicus acteon;0.99999999752248;
MH418538;Lepidoptera;Papilio machaon;Papilio machaon;NA;Papilio machaon;0.99889956649962;
MH418539;Lepidoptera;Lycaena phlaeas;Lycaena phlaeas;1;Lycaena phlaeas;0.999999996363186;
MH418540;Lepidoptera;Anthocharis cardamines;Anthocharis cardamines;1;Anthocharis cardamines;0.999999988534341;
MH418541;Lepidoptera;Lampides boeticus;Lampides boeticus;1;Lampides boeticus;0.999999999545906;
MH418543;Lepidoptera;Colias crocea;NA;0.059;NA;0.999999999999738;
MH418544;Lepidoptera;Colias crocea;NA;0.059;NA;0.999999999999738;
MH418545;Lepidoptera;Lampides boeticus;Lampides boeticus;1;Lampides boeticus;0.999999999545906;
MH418546;Lepidoptera;Pieris napi;NA;0.013;Pieris napi;0.764655619178569;
MH418547;Lepidoptera;Gonepteryx cleopatra;Gonepteryx cleopatra;0.998;Gonepteryx cleopatra;0.999118636582266;
MH418549;Lepidoptera;Coenonympha pamphilus;Coenonympha pamphilus;1;Coenonympha pamphilus;0.999999999998835;
MH418551;Lepidoptera;Coenonympha pamphilus;Coenonympha pamphilus;1;Coenonympha pamphilus;0.999999999999744;
MH418554;Lepidoptera;Vanessa atalanta;Vanessa atalanta;1;Vanessa atalanta;0.999999999757762;
MH418556;Lepidoptera;Melitaea nevadensis;NA;0.009;Melitaea celadussa;0.785259955389478;
MH418557;Lepidoptera;Maniola cecilia;Pyronia cecilia;NA;Pyronia cecilia;0.999999999999972;
MH418558;Lepidoptera;Carcharodus alceae;Carcharodus alceae;0.001;Carcharodus alceae;0.999999994870137;
MH418559;Lepidoptera;Coenonympha corinna;Coenonympha corinna;1;Coenonympha corinna;0.999999999567109;
MH418561;Lepidoptera;Lampides boeticus;Lampides boeticus;1;Lampides boeticus;0.999999999545906;
MH418562;Lepidoptera;Hipparchia aristaeus;Hipparchia aristaeus;1;Hipparchia aristaeus;0.9999998864102;
MH418563;Lepidoptera;Lasiommata megera;Lasiommata megera;1;Lasiommata megera;0.999379144055441;
MH418567;Lepidoptera;Argynnis paphia;Argynnis paphia;1;Argynnis paphia;0.999999991389927;
MH418568;Lepidoptera;Colias crocea;NA;0.059;NA;0.999999999999738;
MH418570;Lepidoptera;Carcharodus alceae;Carcharodus alceae;1;Carcharodus alceae;0.999999990863188;
MH418571;Lepidoptera;Coenonympha pamphilus;Coenonympha pamphilus;1;Coenonympha pamphilus;0.999999999983061;
MH418573;Lepidoptera;Melitaea cinxia;Melitaea cinxia;1;Melitaea cinxia;0.999999999999631;
MH418578;Lepidoptera;Gonepteryx rhamni;NA;0.474;Gonepteryx rhamni;0.982076396127858;
MH418581;Lepidoptera;Maniola cecilia;Pyronia cecilia;NA;Pyronia cecilia;1;
MH418583;Lepidoptera;Lycaena phlaeas;Lycaena phlaeas;1;Lycaena phlaeas;0.999999996363186;
MH418586;Lepidoptera;Lasiommata megera;Lasiommata megera;1;Lasiommata megera;0.999379144055441;
MH418588;Lepidoptera;Melitaea nevadensis;NA;0.014;Melitaea celadussa;0.797886056890592;
MH418589;Lepidoptera;Hipparchia neomiris;Hipparchia neomiris;1;Hipparchia neomiris;0.999999999999375;
MH418590;Lepidoptera;Maniola cecilia;Pyronia cecilia;1;Pyronia cecilia;1;
MH418592;Lepidoptera;Vanessa atalanta;Vanessa atalanta;1;Vanessa atalanta;0.999999999549829;
MH418593;Lepidoptera;Coenonympha corinna;Coenonympha corinna;1;Coenonympha corinna;0.999999999995197;
MH418594;Lepidoptera;Lasiommata megera;Lasiommata megera;1;Lasiommata megera;0.999379144055441;
MH418595;Lepidoptera;Pyrgus armoricanus;Pyrgus armoricanus;1;Pyrgus armoricanus;0.999997353848817;
MH418597;Lepidoptera;Lasiommata megera;Lasiommata megera;1;Lasiommata megera;0.999379144055441;
MH418598;Lepidoptera;Favonius quercus;NA;NA;Favonius quercus;0.73046069582187;
MH418599;Lepidoptera;Papilio machaon;Papilio machaon;0.002;Papilio machaon;0.999372607272923;
MH418600;Lepidoptera;Vanessa atalanta;Vanessa atalanta;1;Vanessa atalanta;0.999999999805453;
MH418601;Lepidoptera;Vanessa atalanta;Vanessa atalanta;1;Vanessa atalanta;0.999999999757762;

MH418602;Lepidoptera;Pyrgus armoricanus;Pyrgus armoricanus;1;Pyrgus armoricanus;0.999997353848817;
MH418603;Lepidoptera;Carcharodus alceae;Carcharodus alceae;1;Carcharodus alceae;0.999999998465938;
MH418604;Lepidoptera;Lasiommata paramegaera;Lasiommata paramegaera;0.001;Lasiommata paramegaera;0.974943507761532;
MH418605;Lepidoptera;Coenonympha corinna;Coenonympha corinna;1;Coenonympha corinna;0.999999999567109;
MH418606;Lepidoptera;Lasiommata paramegaera;Lasiommata paramegaera;0.001;Lasiommata paramegaera;0.996916524417848;
MH418607;Lepidoptera;Gegenes pumilio;Gegenes pumilio;1;Gegenes pumilio;0.999986726679266;
MH418608;Lepidoptera;Favonius quercus;NA;0.002;Favonius quercus;0.732659082125295;
MH418609;Lepidoptera;Issoria lathonia;Issoria lathonia;1;Issoria lathonia;0.999999999989257;
MH418611;Lepidoptera;Coenonympha corinna;Coenonympha corinna;1;Coenonympha corinna;0.999999999481616;
MH418613;Lepidoptera;Glaucopsyche alexis;Glaucopsyche alexis;0.001;Glaucopsyche alexis;0.94042380088325;
MH418614;Lepidoptera;Vanessa atalanta;Vanessa atalanta;1;Vanessa atalanta;0.999999999757762;
MH418615;Lepidoptera;Papilio machaon;Papilio machaon;0.003;Papilio machaon;0.999482752749035;
MH418617;Lepidoptera;Lasiommata megera;Lasiommata megera;1;Lasiommata megera;0.998531205767617;
MH418618;Lepidoptera;Lycaena phlaeas;Lycaena phlaeas;1;Lycaena phlaeas;0.999999996363186;
MH418620;Lepidoptera;Coenonympha corinna;Coenonympha corinna;1;Coenonympha corinna;0.999999999567109;
MH418621;Lepidoptera;Celastrina argiolus;Celastrina argiolus;1;Celastrina argiolus;0.999527385150531;
MH418622;Lepidoptera;Satyrium ilicis;Satyrium ilicis;1;Satyrium ilicis;0.999999987901305;
MH418625;Lepidoptera;Papilio machaon;Papilio machaon;NA;Papilio machaon;0.99889956649962;
MH418628;Lepidoptera;Coenonympha corinna;Coenonympha corinna;1;Coenonympha corinna;0.999999881210819;
MH418629;Lepidoptera;Lycaena phlaeas;Lycaena phlaeas;1;Lycaena phlaeas;0.999999998167567;
MH418630;Lepidoptera;Carcharodus alceae;Carcharodus alceae;0.999;Carcharodus alceae;0.999999996339511;
MH418631;Lepidoptera;Lasiommata megera;Lasiommata megera;1;Lasiommata megera;0.999379144055441;
MH418632;Lepidoptera;Celastrina argiolus;Celastrina argiolus;1;Celastrina argiolus;0.999527385150531;
MH418634;Lepidoptera;Aglais urticae;NA;0.002;Aglais urticae;0.987497593219019;
MH418637;Lepidoptera;Celastrina argiolus;Celastrina argiolus;1;Celastrina argiolus;0.999037794018796;
MH418638;Lepidoptera;Gonepteryx cleopatra;Gonepteryx cleopatra;0.998;Gonepteryx cleopatra;0.999118636582266;
MH418639;Lepidoptera;Carcharodus alceae;Carcharodus alceae;1;Carcharodus alceae;0.999999927744201;
MH418640;Lepidoptera;Papilio machaon;Papilio machaon;0.001;Papilio machaon;0.999336308413213;
MH418641;Lepidoptera;Vanessa atalanta;Vanessa atalanta;1;Vanessa atalanta;0.999999999757762;
MH418642;Lepidoptera;Papilio machaon;Papilio machaon;NA;Papilio machaon;0.999582609409348;
MH418644;Lepidoptera;Lasiommata paramegaera;Lasiommata paramegaera;0.999;Lasiommata paramegaera;0.995283446287531;
MH418647;Lepidoptera;Lasiommata paramegaera;Lasiommata paramegaera;0.999;Lasiommata paramegaera;0.995283446287531;
MH418650;Lepidoptera;Maniola cecilia;Pyronia cecilia;1;Pyronia cecilia;0.999999999999972;
MH418651;Lepidoptera;Thymelicus acteon;Thymelicus acteon;1;Thymelicus acteon;0.99999999752248;
MH418652;Lepidoptera;Satyrium ilicis;Satyrium ilicis;1;Satyrium ilicis;0.999999987901305;
MH418654;Lepidoptera;Lycaena phlaeas;Lycaena phlaeas;1;Lycaena phlaeas;0.999999996363186;
MH418656;Lepidoptera;Gonepteryx cleopatra;Gonepteryx cleopatra;0.998;Gonepteryx cleopatra;0.999118636582266;
MH418657;Lepidoptera;Lasiommata megera;Lasiommata megera;0.999;Lasiommata megera;0.999701831224245;
MH418658;Lepidoptera;Argynnis paphia;Argynnis paphia;1;Argynnis paphia;0.999999991389927;
MH418659;Lepidoptera;Papilio machaon;Papilio machaon;NA;Papilio machaon;0.998335940984695;
MH418660;Lepidoptera;Vanessa atalanta;Vanessa atalanta;1;Vanessa atalanta;0.999999999757762;
MH418661;Lepidoptera;Lampides boeticus;Lampides boeticus;1;Lampides boeticus;0.999999999545906;
MH418662;Lepidoptera;Gonepteryx cleopatra;Gonepteryx cleopatra;0.998;Gonepteryx cleopatra;0.999118636582266;
MH418664;Lepidoptera;Callophrys rubi;NA;0.019;Callophrys rubi;0.998201459637982;
MH418665;Lepidoptera;Maniola cecilia;Pyronia cecilia;NA;Pyronia cecilia;1;
MH418666;Lepidoptera;Papilio machaon;Papilio machaon;NA;Papilio machaon;0.998335940984695;
MH418667;Lepidoptera;Colias crocea;NA;0.059;NA;0.999999999999738;
MH418669;Lepidoptera;Thymelicus acteon;Thymelicus acteon;1;Thymelicus acteon;0.99999999752248;
MH418670;Lepidoptera;Celastrina argiolus;Celastrina argiolus;1;Celastrina argiolus;0.998121368997996;
MH418671;Lepidoptera;Maniola tithonus;Pyronia tithonus;1;Pyronia tithonus;1;
MH418673;Lepidoptera;Coenonympha pamphilus;Coenonympha pamphilus;1;Coenonympha pamphilus;0.999999999999744;
MH418674;Lepidoptera;Issoria lathonia;Issoria lathonia;1;Issoria lathonia;0.999999999989257;
MH418675;Lepidoptera;Charaxes jasius;Charaxes jasius;NA;Charaxes jasius;0.979358487720088;
MH418678;Lepidoptera;Coenonympha corinna;Coenonympha corinna;1;Coenonympha corinna;0.999999999567109;
MH418679;Lepidoptera;Pieris napi;NA;0.028;Pieris napi;0.888205720664699;

MH418680;Lepidoptera;Vanessa atalanta;Vanessa atalanta;1;Vanessa atalanta;0.999999999757762;
MH418682;Lepidoptera;Maniola jurtina;NA;0.038;NA;0.999999999999524;
MH418683;Lepidoptera;Coenonympha pamphilus;Coenonympha pamphilus;1;Coenonympha pamphilus;0.999999999999744;
MH418684;Lepidoptera;Celastrina argiolus;Celastrina argiolus;1;Celastrina argiolus;0.999527385150531;
MH418685;Lepidoptera;Hipparchia aristaeus;Hipparchia aristaeus;1;Hipparchia aristaeus;0.999999993824275;
MH418687;Lepidoptera;Lampides boeticus;Lampides boeticus;1;Lampides boeticus;0.999999999545906;
MH418688;Lepidoptera;Lampides boeticus;Lampides boeticus;1;Lampides boeticus;0.99999999876789;
MH418689;Lepidoptera;Issoria lathonia;Issoria lathonia;1;Issoria lathonia;0.999999999989257;
MH418690;Lepidoptera;Vanessa atalanta;Vanessa atalanta;1;Vanessa atalanta;0.999999999757762;
MH418691;Lepidoptera;Carcharodus alceae;Carcharodus alceae;0.001;Carcharodus alceae;0.999999994870137;
MH418692;Lepidoptera;Lampides boeticus;Lampides boeticus;1;Lampides boeticus;0.999999999545906;
MH418693;Lepidoptera;Colias crocea;NA;0.059;NA;0.999999999999738;
MH418694;Lepidoptera;Colias crocea;NA;0.059;NA;0.999999999999738;
MH418695;Lepidoptera;Pieris napi;NA;0.009;Pieris napi;0.857602568978328;
MH418696;Lepidoptera;Lycaena phlaeas;Lycaena phlaeas;1;Lycaena phlaeas;0.999999992669558;
MH418697;Lepidoptera;Lasiommata megera;Lasiommata megera;1;Lasiommata megera;0.999379144055441;
MH418698;Lepidoptera;Gonepteryx cleopatra;Gonepteryx cleopatra;0.998;Gonepteryx cleopatra;0.999118636582266;
MH418699;Lepidoptera;Lasiommata megera;Lasiommata megera;1;Lasiommata megera;0.999379144055441;
MH418700;Lepidoptera;Papilio machaon;Papilio machaon;NA;Papilio machaon;0.997246443353099;
MH418701;Lepidoptera;Lasiommata megera;Lasiommata megera;1;Lasiommata megera;0.999379144055441;
MH418704;Lepidoptera;Colias crocea;NA;0.059;NA;0.999999999999738;
MH418705;Lepidoptera;Celastrina argiolus;Celastrina argiolus;1;Celastrina argiolus;0.999037794018796;
MH418706;Lepidoptera;Lasiommata megera;Lasiommata megera;1;Lasiommata megera;0.998531205767617;
MH418708;Lepidoptera;Lycaena phlaeas;Lycaena phlaeas;1;Lycaena phlaeas;0.999999996363186;
MH418709;Lepidoptera;Colias crocea;NA;0.059;NA;0.999999999999738;
MH418712;Lepidoptera;Lasiommata paramegaera;Lasiommata paramegaera;0.999;Lasiommata paramegaera;0.995283446287531;
MH418716;Lepidoptera;Aglais ichnusa;Aglais ichnusa;0.003;NA;0.999999990022183;
MH418719;Lepidoptera;Colias crocea;NA;0.059;NA;0.999999999999738;
MH418722;Lepidoptera;Papilio machaon;Papilio machaon;0.001;Papilio machaon;0.999463129150422;
MH418723;Lepidoptera;Colias crocea;NA;0.059;NA;0.999999999999738;
MH418724;Lepidoptera;Lycaena phlaeas;Lycaena phlaeas;1;Lycaena phlaeas;0.999999996363186;
MH418725;Lepidoptera;Lasiommata megera;Lasiommata megera;1;Lasiommata megera;0.999379144055441;
MH418726;Lepidoptera;Lycaena phlaeas;Lycaena phlaeas;1;Lycaena phlaeas;0.999999996363186;
MH418727;Lepidoptera;Carcharodus alceae;Carcharodus alceae;0.999;Carcharodus alceae;0.999999996339511;
MH418729;Lepidoptera;Vanessa atalanta;Vanessa atalanta;1;Vanessa atalanta;0.99999999976086;
MH418730;Lepidoptera;Vanessa atalanta;Vanessa atalanta;1;Vanessa atalanta;0.999999999757762;
MH418733;Lepidoptera;Lampides boeticus;Lampides boeticus;1;Lampides boeticus;0.999999999545906;
MH418737;Lepidoptera;Gonepteryx cleopatra;Gonepteryx cleopatra;0.998;Gonepteryx cleopatra;0.999118636582266;
MH418739;Lepidoptera;Lasiommata megera;Lasiommata megera;1;Lasiommata megera;0.998531205767617;
MH418740;Lepidoptera;Maniola cecilia;Pyronia cecilia;1;Pyronia cecilia;1;
MH418742;Lepidoptera;Celastrina argiolus;Celastrina argiolus;1;Celastrina argiolus;0.999037794018796;
MH418743;Lepidoptera;Aricia agestis;Aricia agestis;0.001;Aricia agestis;0.972120797608056;
MH418745;Lepidoptera;Lycaena phlaeas;Lycaena phlaeas;1;Lycaena phlaeas;0.999999998167567;
MH418746;Lepidoptera;Coenonympha corinna;Coenonympha corinna;1;Coenonympha corinna;0.999999999567109;
MH418747;Lepidoptera;Lasiommata paramegaera;Lasiommata paramegaera;0.999;Lasiommata paramegaera;0.995283446287531;
MH418751;Lepidoptera;Pieris rapae;NA;NA;Pieris rapae;0.999962030706249;
MH418752;Lepidoptera;Pieris rapae;Pieris rapae;NA;Pieris rapae;0.999992265968936;
MH418753;Lepidoptera;Lampides boeticus;Lampides boeticus;1;Lampides boeticus;0.999999999545906;
MH418754;Lepidoptera;Colias crocea;NA;0.065;NA;0.999999999999745;
MH418757;Lepidoptera;Argynnis paphia;Argynnis paphia;1;Argynnis paphia;0.999999991389927;
MH418758;Lepidoptera;Maniola cecilia;Pyronia cecilia;1;Pyronia cecilia;1;
MH418759;Lepidoptera;Lasiommata megera;Lasiommata megera;0.998;Lasiommata megera;0.998568158791128;
MH418760;Lepidoptera;Gonepteryx cleopatra;Gonepteryx cleopatra;0.001;Gonepteryx cleopatra;0.999134475039697;
MH418761;Lepidoptera;Lampides boeticus;Lampides boeticus;1;Lampides boeticus;0.999999999545906;
MH418762;Lepidoptera;Celastrina argiolus;Celastrina argiolus;1;Celastrina argiolus;0.999527385150531;
MH418763;Lepidoptera;Charaxes jasius;Charaxes jasius;NA;Charaxes jasius;0.990397821479112;
MH418767;Lepidoptera;Lasiommata megera;Lasiommata megera;1;Lasiommata megera;0.999379144055441;
MH418768;Lepidoptera;Lycaena phlaeas;Lycaena phlaeas;1;Lycaena phlaeas;0.999999998505558;
MH418770;Lepidoptera;Lampides boeticus;Lampides boeticus;1;Lampides boeticus;0.999999999411585;
MH418771;Lepidoptera;Maniola cecilia;Pyronia cecilia;1;Pyronia cecilia;1;
MH418776;Lepidoptera;Glaucopsyche alexis;NA;NA;Glaucopsyche alexis;0.979381169624069;
MH418779;Lepidoptera;Coenonympha corinna;Coenonympha corinna;1;Coenonympha corinna;0.999999999567109;

MH418780;Lepidoptera;Favonius quercus;NA;0.002;Favonius quercus;0.732659082125295;
MH418782;Lepidoptera;Coenonympha corinna;Coenonympha corinna;1;Coenonympha corinna;0.999999853807008;
MH418783;Lepidoptera;Satyrium ilicis;Satyrium ilicis;1;Satyrium ilicis;0.999999987901305;
MH418785;Lepidoptera;Papilio machaon;Papilio machaon;0.003;Papilio machaon;0.999659108582311;
MH418786;Lepidoptera;Celastrina argiolus;Celastrina argiolus;1;Celastrina argiolus;0.999037794018796;
MH418787;Lepidoptera;Argynnis paphia;Argynnis paphia;1;Argynnis paphia;0.999999991389927;
MH418789;Lepidoptera;Lycaena phlaeas;Lycaena phlaeas;1;Lycaena phlaeas;0.999999996363186;
MH418792;Lepidoptera;Lasiommata megera;Lasiommata megera;1;Lasiommata megera;0.999379144055441;
MH418793;Lepidoptera;Anthocharis cardamines;Anthocharis cardamines;1;Anthocharis cardamines;0.99999997953563;
MH418794;Lepidoptera;Lycaena phlaeas;Lycaena phlaeas;1;Lycaena phlaeas;0.999999996363186;
MH418795;Lepidoptera;Lasiommata megera;Lasiommata megera;1;Lasiommata megera;0.999379144055441;
MH418797;Lepidoptera;Lasiommata megera;Lasiommata megera;1;Lasiommata megera;0.999379144055441;
MH418798;Lepidoptera;Celastrina argiolus;Celastrina argiolus;1;Celastrina argiolus;0.999527385150531;
MH418799;Lepidoptera;Lasiommata megera;Lasiommata megera;1;Lasiommata megera;0.999939556832216;
MH418800;Lepidoptera;Colias crocea;NA;0.059;NA;0.999999999999738;
MH418803;Lepidoptera;Glaucopsyche alexis;Glaucopsyche alexis;0.001;Glaucopsyche alexis;0.94042380088325;
MH418804;Lepidoptera;Hipparchia statilinus;Hipparchia statilinus;1;Hipparchia statilinus;0.999999999999943;
MH418805;Lepidoptera;Lampides boeticus;Lampides boeticus;1;Lampides boeticus;0.999999999545906;
MH418806;Lepidoptera;Lycaena phlaeas;Lycaena phlaeas;1;Lycaena phlaeas;0.999999996363186;
MH418808;Lepidoptera;Coenonympha pamphilus;Coenonympha pamphilus;1;Coenonympha pamphilus;0.999999999999744;
MH418809;Lepidoptera;Celastrina argiolus;Celastrina argiolus;1;Celastrina argiolus;0.999527385150531;
MH418810;Lepidoptera;Pieris rapae;Pieris rapae;NA;Pieris rapae;0.999994286682757;
MH418811;Lepidoptera;Glaucopsyche alexis;Glaucopsyche alexis;0.001;Glaucopsyche alexis;0.94042380088325;
MH418813;Lepidoptera;Lasiommata megera;Lasiommata megera;1;Lasiommata megera;0.999379144055441;
MH418816;Lepidoptera;Pieris napi;NA;0.011;Pieris napi;0.801985394724814;
MH418818;Lepidoptera;Hipparchia statilinus;Hipparchia statilinus;1;Hipparchia statilinus;0.999999999999943;
MH418819;Lepidoptera;Gegenes pumilio;Gegenes pumilio;1;Gegenes pumilio;0.999986726679266;
MH418820;Lepidoptera;Celastrina argiolus;Celastrina argiolus;1;Celastrina argiolus;0.999527385150531;
MH418823;Lepidoptera;Favonius quercus;NA;NA;Favonius quercus;0.73046069582187;
MH418824;Lepidoptera;Pieris napi;NA;NA;Pieris napi;0.874112864145033;
MH418826;Lepidoptera;Pieris rapae;NA;NA;Pieris rapae;0.999970257792891;
MH418828;Lepidoptera;Pieris rapae;NA;NA;Pieris rapae;0.999970925323953;
MH418829;Lepidoptera;Coenonympha pamphilus;Coenonympha pamphilus;1;Coenonympha pamphilus;0.999999999983061;
MH418830;Lepidoptera;Satyrium ilicis;Satyrium ilicis;1;Satyrium ilicis;0.999999987901305;
MH418832;Lepidoptera;Thymelicus acteon;Thymelicus acteon;1;Thymelicus acteon;0.99999999752248;
MH418833;Lepidoptera;Pieris rapae;NA;NA;Pieris rapae;0.999992588119495;
MH418834;Lepidoptera;Lycaena phlaeas;Lycaena phlaeas;1;Lycaena phlaeas;0.999999996363186;
MH418836;Lepidoptera;Pieris napi;NA;0.011;Pieris napi;0.801985394724814;
MH418839;Lepidoptera;Maniola jurtina;NA;0.001;Maniola jurtina;0.939220105860875;
MH418840;Lepidoptera;Lasiommata megera;Lasiommata megera;1;Lasiommata megera;0.999379144055441;
MH418841;Lepidoptera;Lasiommata paramegaera;Lasiommata paramegaera;0.999;Lasiommata paramegaera;0.995283446287531;
MH418842;Lepidoptera;Pieris rapae;Pieris rapae;1;Pieris rapae;0.999996064425815;
MH418843;Lepidoptera;Issoria lathonia;Issoria lathonia;1;Issoria lathonia;0.999999999989257;
MH418844;Lepidoptera;Lasiommata megera;Lasiommata megera;1;Lasiommata megera;0.999379144055441;
MH418846;Lepidoptera;Lampides boeticus;Lampides boeticus;1;Lampides boeticus;0.999999999545906;
MH418850;Lepidoptera;Lampides boeticus;Lampides boeticus;1;Lampides boeticus;0.999999999545906;
MH418852;Lepidoptera;Charaxes jasius;Charaxes jasius;NA;Charaxes jasius;0.990397821479112;
MH418853;Lepidoptera;Lasiommata megera;Lasiommata megera;1;Lasiommata megera;0.998531205767617;
MH418854;Lepidoptera;Pieris rapae;NA;0.039;Pieris rapae;0.999989302159245;
MH418856;Lepidoptera;Celastrina argiolus;Celastrina argiolus;1;Celastrina argiolus;0.999527385150531;
MH418858;Lepidoptera;Vanessa atalanta;Vanessa atalanta;1;Vanessa atalanta;0.999999999470958;
MH418859;Lepidoptera;Colias crocea;NA;0.059;NA;0.999999999999738;
MH418860;Lepidoptera;Pieris rapae;NA;NA;Pieris rapae;0.999957794752662;
MH418861;Lepidoptera;Pieris rapae;NA;0.206;Pieris rapae;0.999929773639513;
MH418862;Lepidoptera;Satyrium ilicis;Satyrium ilicis;1;Satyrium ilicis;0.999999987901305;
MH418863;Lepidoptera;Vanessa atalanta;Vanessa atalanta;1;Vanessa atalanta;0.999999999757762;
MH418864;Lepidoptera;Carcharodus alceae;Carcharodus alceae;0.999;Carcharodus alceae;0.999999996339511;
MH418865;Lepidoptera;Maniola cecilia;Pyronia cecilia;NA;Pyronia cecilia;1;
MH418866;Lepidoptera;Lasiommata megera;Lasiommata megera;1;Lasiommata megera;0.999928528239676;
MH418868;Lepidoptera;Lycaena phlaeas;Lycaena phlaeas;1;Lycaena phlaeas;0.999999996363186;
MH418871;Lepidoptera;Aricia cramera;Aricia cramera;1;Aricia cramera;0.999993731037033;
MH418872;Lepidoptera;Maniola cecilia;Pyronia cecilia;1;Pyronia cecilia;1;
MH418874;Lepidoptera;Pieris rapae;Pieris rapae;NA;Pieris rapae;0.999988086141146;

MH418875;Lepidoptera;Celastrina argiolus;Celastrina argiolus;1;Celastrina argiolus;0.999527385150531;
MH418877;Lepidoptera;Lasiommata paramegaera;Lasiommata paramegaera;0.999;Lasiommata paramegaera;0.995283446287531;
MH418878;Lepidoptera;Callophrys rubi;NA;0.001;Callophrys rubi;0.998885705938309;
MH418879;Lepidoptera;Gonepteryx cleopatra;Gonepteryx cleopatra;0.998;Gonepteryx cleopatra;0.999118636582266;
MH418880;Lepidoptera;Maniola tithonus;Pyronia tithonus;1;Pyronia tithonus;1;
MH418882;Lepidoptera;Lasiommata megera;Lasiommata megera;1;Lasiommata megera;0.999928528239676;
MH418883;Lepidoptera;Colias crocea;NA;NA;NA;0.999999999999491;
MH418884;Lepidoptera;Gonepteryx cleopatra;Gonepteryx cleopatra;0.998;Gonepteryx cleopatra;0.999118636582266;
MH418887;Lepidoptera;Carcharodus alceae;Carcharodus alceae;1;Carcharodus alceae;0.999999998465938;
MH418889;Lepidoptera;Pieris rapae;NA;NA;Pieris rapae;0.999992588119495;
MH418890;Lepidoptera;Hipparchia neomiris;Hipparchia neomiris;1;Hipparchia neomiris;0.999999999999602;
MH418891;Lepidoptera;Celastrina argiolus;Celastrina argiolus;1;Celastrina argiolus;0.999527385150531;
MH418892;Lepidoptera;Charaxes jasius;Charaxes jasius;NA;Charaxes jasius;0.990397821479112;
MH418893;Lepidoptera;Vanessa atalanta;Vanessa atalanta;1;Vanessa atalanta;0.999999999757762;
MH418894;Lepidoptera;Glaucopsyche alexis;Glaucopsyche alexis;0.001;Glaucopsyche alexis;0.970555559513917;
MH418895;Lepidoptera;Gonepteryx cleopatra;Gonepteryx cleopatra;1;Gonepteryx cleopatra;0.998021026047298;
MH418897;Lepidoptera;Maniola cecilia;Pyronia cecilia;1;Pyronia cecilia;1;
MH418898;Lepidoptera;Lycaena phlaeas;Lycaena phlaeas;1;Lycaena phlaeas;0.999999998167567;
MH418899;Lepidoptera;Maniola jurtina;NA;0.001;Maniola jurtina;0.939220105860875;
MH418900;Lepidoptera;Celastrina argiolus;Celastrina argiolus;1;Celastrina argiolus;0.999631157817044;
MH418901;Lepidoptera;Melitaea nevadensis;NA;0.869;Melitaea celadussa;0.928192792494775;
MH418902;Lepidoptera;Lycaena phlaeas;Lycaena phlaeas;1;Lycaena phlaeas;0.999999996363186;
MH418903;Lepidoptera;Satyrium ilicis;Satyrium ilicis;1;Satyrium ilicis;0.999999987901305;
MH418904;Lepidoptera;Maniola cecilia;Pyronia cecilia;NA;Pyronia cecilia;1;
MH418906;Lepidoptera;Maniola cecilia;Pyronia cecilia;NA;Pyronia cecilia;1;
MH418907;Lepidoptera;Lycaena phlaeas;Lycaena phlaeas;1;Lycaena phlaeas;0.999999998167567;
MH418908;Lepidoptera;Pieris rapae;NA;NA;Pieris rapae;0.999992588119495;
MH418909;Lepidoptera;Lasiommata paramegaera;Lasiommata paramegaera;0.999;Lasiommata paramegaera;0.995283446287531;
MH418912;Lepidoptera;Gegenes pumilio;Gegenes pumilio;1;Gegenes pumilio;0.999989874205988;
MH418913;Lepidoptera;Lycaena phlaeas;Lycaena phlaeas;1;Lycaena phlaeas;0.999999998167567;
MH418914;Lepidoptera;Pieris rapae;NA;0.175;Pieris rapae;0.99996637543374;
MH418915;Lepidoptera;Charaxes jasius;Charaxes jasius;NA;Charaxes jasius;0.990397821479112;
MH418917;Lepidoptera;Pieris rapae;NA;NA;Pieris rapae;0.999957794752662;
MH418919;Lepidoptera;Hipparchia aristaeus;Hipparchia aristaeus;1;Hipparchia aristaeus;0.999999988161392;
MH418923;Lepidoptera;Pieris napi;NA;0.011;Pieris napi;0.801985394724814;
MH418924;Lepidoptera;Lycaena phlaeas;Lycaena phlaeas;1;Lycaena phlaeas;0.999999992669558;
MH418925;Lepidoptera;Coenonympha corinna;Coenonympha corinna;1;Coenonympha corinna;0.999999853807008;
MH418931;Lepidoptera;Lampides boeticus;Lampides boeticus;1;Lampides boeticus;0.999999999469992;
MH418933;Lepidoptera;Lycaena phlaeas;Lycaena phlaeas;1;Lycaena phlaeas;0.999999998167567;
MH418934;Lepidoptera;Maniola tithonus;Pyronia tithonus;1;Pyronia tithonus;0.999999999999972;
MH418936;Lepidoptera;Pieris rapae;NA;NA;Pieris rapae;0.999992588119495;
MH418938;Lepidoptera;Carcharodus alceae;Carcharodus alceae;1;Carcharodus alceae;0.999999998465938;
MH418939;Lepidoptera;Gegenes pumilio;Gegenes pumilio;1;Gegenes pumilio;0.999986726679266;
MH418940;Lepidoptera;Issoria lathonia;Issoria lathonia;1;Issoria lathonia;0.999999999989257;
MH418941;Lepidoptera;Vanessa atalanta;Vanessa atalanta;1;Vanessa atalanta;0.999999999757762;
MH418942;Lepidoptera;Coenonympha pamphilus;Coenonympha pamphilus;1;Coenonympha pamphilus;0.999999999983061;
MH418944;Lepidoptera;Lampides boeticus;Lampides boeticus;1;Lampides boeticus;0.999999999545906;
MH418945;Lepidoptera;Charaxes jasius;Charaxes jasius;NA;Charaxes jasius;0.990397821479112;
MH418947;Lepidoptera;Callophrys rubi;NA;0.019;Callophrys rubi;0.998201459637982;
MH418948;Lepidoptera;Favonius quercus;NA;NA;Favonius quercus;0.73046069582187;
MH418949;Lepidoptera;Colias crocea;NA;0.059;NA;0.999999999999738;
MH418950;Lepidoptera;Hipparchia aristaeus;Hipparchia aristaeus;1;Hipparchia aristaeus;0.999999996564412;
MH418952;Lepidoptera;Glaucopsyche alexis;Glaucopsyche alexis;0.001;Glaucopsyche alexis;0.94042380088325;
MH418953;Lepidoptera;Celastrina argiolus;Celastrina argiolus;1;Celastrina argiolus;0.999037794018796;
MH418954;Lepidoptera;Charaxes jasius;Charaxes jasius;NA;Charaxes jasius;0.990397821479112;
MH418955;Lepidoptera;Coenonympha pamphilus;Coenonympha pamphilus;1;Coenonympha pamphilus;0.999999999997954;
MH418957;Lepidoptera;Gonepteryx rhamni;Gonepteryx rhamni;0.008;Gonepteryx rhamni;0.999485025383278;

MH418963;Lepidoptera;Coenonympha corinna;Coenonympha corinna;1;Coenonympha corinna;0.999999999567109;
MH418964;Lepidoptera;Lasiommata megera;Lasiommata megera;1;Lasiommata megera;0.998531205767617;
MH418965;Lepidoptera;Pieris rapae;NA;NA;Pieris rapae;0.999984333973504;
MH418968;Lepidoptera;Lasiommata megera;Lasiommata megera;1;Lasiommata megera;0.998531205767617;
MH418970;Lepidoptera;Pieris napi;NA;0.011;Pieris napi;0.801985394724814;
MH418971;Lepidoptera;Papilio machaon;Papilio machaon;NA;Papilio machaon;0.998335940984695;
MH418972;Lepidoptera;Callophrys rubi;NA;0.001;Callophrys rubi;0.998885705938309;
MH418974;Lepidoptera;Aglais ichnusa;Aglais ichnusa;0.998;NA;0.999999996206876;
MH418979;Lepidoptera;Aricia agestis;Aricia agestis;1;Aricia agestis;0.979315827401608;
MH418980;Lepidoptera;Maniola cecilia;Pyronia cecilia;1;Pyronia cecilia;0.999999999999972;
MH418981;Lepidoptera;Coenonympha pamphilus;Coenonympha pamphilus;1;Coenonympha pamphilus;0.999999999999744;
MH418982;Lepidoptera;Aglais urticae;Aglais urticae;0.001;Aglais urticae;0.990690600684087;
MH418984;Lepidoptera;Coenonympha corinna;Coenonympha corinna;1;Coenonympha corinna;0.999999920013298;
MH418985;Lepidoptera;Pieris napi;NA;0.011;Pieris napi;0.801985394724814;
MH418986;Lepidoptera;Aricia agestis;NA;0.03;Aricia agestis;0.999295990875121;
MH418987;Lepidoptera;Lycaena phlaeas;Lycaena phlaeas;1;Lycaena phlaeas;0.999999996363186;
MH418989;Lepidoptera;Lasiommata megera;Lasiommata megera;1;Lasiommata megera;0.999379144055441;
MH418990;Lepidoptera;Colias crocea;NA;0.059;NA;0.999999999999738;
MH418992;Lepidoptera;Maniola cecilia;Pyronia cecilia;1;Pyronia cecilia;0.999999999999972;
MH418995;Lepidoptera;Hipparchia statilinus;Hipparchia statilinus;1;Hipparchia statilinus;0.999999999999915;
MH418997;Lepidoptera;Melitaea nevadensis;Melitaea celadussa;1;Melitaea celadussa;0.998328055835183;
MH418998;Lepidoptera;Maniola cecilia;Pyronia cecilia;1;Pyronia cecilia;1;
MH419000;Lepidoptera;Maniola cecilia;Pyronia cecilia;NA;Pyronia cecilia;1;
MH419001;Lepidoptera;Aglais ichnusa;Aglais ichnusa;0.998;NA;0.999999996206876;
MH419002;Lepidoptera;Lasiommata megera;Lasiommata megera;1;Lasiommata megera;0.999379144055441;
MH419004;Lepidoptera;Lampides boeticus;Lampides boeticus;1;Lampides boeticus;0.999999999545906;
MH419005;Lepidoptera;Vanessa atalanta;Vanessa atalanta;1;Vanessa atalanta;0.999999999757762;
MH419006;Lepidoptera;Maniola cecilia;Pyronia cecilia;1;Pyronia cecilia;0.999999999999972;
MH419007;Lepidoptera;Melitaea nevadensis;NA;0.001;Melitaea celadussa;0.727568537860761;
MH419008;Lepidoptera;Maniola cecilia;Pyronia cecilia;NA;Pyronia cecilia;1;
MH419011;Lepidoptera;Papilio machaon;Papilio machaon;0.003;Papilio machaon;0.999482752749035;
MH419012;Lepidoptera;Coenonympha pamphilus;Coenonympha pamphilus;1;Coenonympha pamphilus;0.999999999995282;
MH419013;Lepidoptera;Colias crocea;NA;0.059;NA;0.999999999999738;
MH419015;Lepidoptera;Lasiommata paramegaera;Lasiommata paramegaera;0.999;Lasiommata paramegaera;0.995283446287531;
MH419017;Lepidoptera;Callophrys rubi;NA;0.001;Callophrys rubi;0.998885705938309;
MH419019;Lepidoptera;Papilio machaon;Papilio machaon;0.003;Papilio machaon;0.999482752749035;
MH419020;Lepidoptera;Colias crocea;NA;0.059;NA;0.999999999999738;
MH419021;Lepidoptera;Favonius quercus;NA;0.002;Favonius quercus;0.732659082125295;
MH419023;Lepidoptera;Melitaea didyma;Melitaea didyma;1;Melitaea didyma;0.999946102605688;
MH419024;Lepidoptera;Lampides boeticus;Lampides boeticus;1;Lampides boeticus;0.999999999545906;
MH419025;Lepidoptera;Lampides boeticus;Lampides boeticus;1;Lampides boeticus;0.999999999545906;
MH419027;Lepidoptera;Gonepteryx cleopatra;Gonepteryx cleopatra;0.998;Gonepteryx cleopatra;0.999118636582266;
MH419028;Lepidoptera;Issoria lathonia;Issoria lathonia;1;Issoria lathonia;0.999999999989257;
MH419032;Lepidoptera;Issoria lathonia;Issoria lathonia;1;Issoria lathonia;0.999999999989257;
MH419033;Lepidoptera;Colias crocea;NA;0.059;NA;0.999999999999738;
MH419035;Lepidoptera;Melitaea didyma;Melitaea didyma;1;Melitaea didyma;0.999755830950219;
MH419037;Lepidoptera;Issoria lathonia;Issoria lathonia;1;Issoria lathonia;0.999999999987864;
MH419039;Lepidoptera;Hipparchia neomiris;Hipparchia neomiris;1;Hipparchia neomiris;0.999999999999034;
MH419041;Lepidoptera;Papilio machaon;Papilio machaon;0.003;Papilio machaon;0.999482752749035;
MH419043;Lepidoptera;Lycaena phlaeas;Lycaena phlaeas;1;Lycaena phlaeas;0.999999997915609;
MH419044;Lepidoptera;Pyrgus armoricanus;Pyrgus armoricanus;1;Pyrgus armoricanus;0.999997353848817;
MH419045;Lepidoptera;Colias crocea;NA;0.059;NA;0.999999999999738;
MH419047;Lepidoptera;Maniola cecilia;Pyronia cecilia;NA;Pyronia cecilia;1;
MH419048;Lepidoptera;Thymelicus acteon;Thymelicus acteon;1;Thymelicus acteon;0.99999999752248;
MH419049;Lepidoptera;Aricia agestis;NA;NA;Aricia agestis;0.996930365249266;
MH419050;Lepidoptera;Pieris napi;NA;0.084;Pieris napi;0.885343420349066;
MH419051;Lepidoptera;Coenonympha pamphilus;Coenonympha pamphilus;1;Coenonympha pamphilus;0.999999999999744;
MH419052;Lepidoptera;Celastrina argiolus;Celastrina argiolus;1;Celastrina argiolus;0.999527385150531;
MH419053;Lepidoptera;Hipparchia neomiris;Hipparchia neomiris;1;Hipparchia neomiris;0.999999999999801;
MH419054;Lepidoptera;Colias crocea;NA;0.059;NA;0.999999999999738;
MH419057;Lepidoptera;Lampides boeticus;Lampides boeticus;1;Lampides boeticus;0.999999999545906;
MH419058;Lepidoptera;Papilio machaon;Papilio machaon;0.001;Papilio machaon;0.999463129150422;
MH419060;Lepidoptera;Lycaena phlaeas;Lycaena phlaeas;1;Lycaena phlaeas;0.999999996363186;
MH419062;Lepidoptera;Lasiommata megera;Lasiommata megera;1;Lasiommata megera;0.999806890129977;
MH419065;Lepidoptera;Lycaena phlaeas;Lycaena phlaeas;1;Lycaena phlaeas;0.999999995520994;

MH419066;Lepidoptera;Maniola tithonus;Pyronia tithonus;1;Pyronia tithonus;1;
MH419070;Lepidoptera;Carcharodus alceae;Carcharodus alceae;1;Carcharodus alceae;0.999999998465938;
MH419071;Lepidoptera;Callophrys rubi;NA;0.001;Callophrys rubi;0.998885705938309;
MH419072;Lepidoptera;Callophrys rubi;NA;0.001;Callophrys rubi;0.998885705938309;
MH419079;Lepidoptera;Gonepteryx rhamni;NA;0.474;Gonepteryx rhamni;0.982076396127858;
MH419081;Lepidoptera;Callophrys rubi;NA;0.001;Callophrys rubi;0.998885705938309;
MH419083;Lepidoptera;Gonepteryx cleopatra;Gonepteryx cleopatra;0.998;Gonepteryx cleopatra;0.999118636582266;
MH419086;Lepidoptera;Gegenes pumilio;Gegenes pumilio;0.999;Gegenes pumilio;0.999991412663101;
MH419087;Lepidoptera;Aricia agestis;NA;NA;Aricia agestis;0.993980512246517;
MH419088;Lepidoptera;Lasiommata megera;Lasiommata megera;1;Lasiommata megera;0.999379144055441;
MH419091;Lepidoptera;Colias crocea;NA;NA;NA;0.999999999998525;
MH419096;Lepidoptera;Lampides boeticus;Lampides boeticus;1;Lampides boeticus;0.999999999545906;
MH419097;Lepidoptera;Carcharodus alceae;Carcharodus alceae;1;Carcharodus alceae;0.999999990863188;
MH419098;Lepidoptera;Celastrina argiolus;Celastrina argiolus;1;Celastrina argiolus;0.999037794018796;
MH419100;Lepidoptera;Thymelicus acteon;Thymelicus acteon;1;Thymelicus acteon;0.99999999752248;
MH419102;Lepidoptera;Carcharodus alceae;Carcharodus alceae;1;Carcharodus alceae;0.999999998465938;
MH419103;Lepidoptera;Lasiommata megera;Lasiommata megera;1;Lasiommata megera;0.999379144055441;
MH419104;Lepidoptera;Maniola cecilia;Pyronia cecilia;NA;Pyronia cecilia;1;
MH419105;Lepidoptera;Colias crocea;NA;0.059;NA;0.999999999999738;
MH419107;Lepidoptera;Callophrys rubi;NA;0.001;Callophrys rubi;0.998885705938309;
MH419108;Lepidoptera;Vanessa atalanta;Vanessa atalanta;1;Vanessa atalanta;0.999999999757762;
MH419109;Lepidoptera;Maniola jurtina;NA;0.169;NA;0.999999999999053;
MH419111;Lepidoptera;Anthocharis cardamines;Anthocharis cardamines;1;Anthocharis cardamines;0.999999940675226;
MH419113;Lepidoptera;Lampides boeticus;Lampides boeticus;1;Lampides boeticus;0.999999999545906;
MH419115;Lepidoptera;Lasiommata paramegaera;Lasiommata paramegaera;0.999;Lasiommata paramegaera;0.995283446287531;
MH419117;Lepidoptera;Lampides boeticus;Lampides boeticus;1;Lampides boeticus;0.999999999620769;
MH419119;Lepidoptera;Lampides boeticus;Lampides boeticus;1;Lampides boeticus;0.999999999545906;
MH419120;Lepidoptera;Anthocharis cardamines;Anthocharis cardamines;1;Anthocharis cardamines;0.999999987224612;
MH419121;Lepidoptera;Melitaea nevadensis;Melitaea celadussa;1;Melitaea celadussa;0.99883216908598;
MH419122;Lepidoptera;Lasiommata megera;Lasiommata megera;0.999;Lasiommata megera;0.999423486493351;
MH419123;Lepidoptera;Celastrina argiolus;Celastrina argiolus;1;Celastrina argiolus;0.999527385150531;
MH419124;Lepidoptera;Pieris napi;NA;0.066;Pieris napi;0.832895481558215;
MH419125;Lepidoptera;Lasiommata megera;Lasiommata megera;1;Lasiommata megera;0.999379144055441;
MH419126;Lepidoptera;Hipparchia statilinus;Hipparchia statilinus;1;Hipparchia statilinus;0.999999999999943;
MH419127;Lepidoptera;Carcharodus alceae;Carcharodus alceae;1;Carcharodus alceae;0.999999990863188;
MH419128;Lepidoptera;Hipparchia statilinus;Hipparchia statilinus;1;Hipparchia statilinus;0.999999999999915;
MH419129;Lepidoptera;Callophrys rubi;NA;0.019;Callophrys rubi;0.998201459637982;
MH419130;Lepidoptera;Pieris napi;NA;0.011;Pieris napi;0.801985394724814;
MH419133;Lepidoptera;Anthocharis cardamines;Anthocharis cardamines;1;Anthocharis cardamines;0.999999987200425;
MH419140;Lepidoptera;Carcharodus alceae;Carcharodus alceae;0.003;Carcharodus alceae;0.999999959808407;
MH419141;Lepidoptera;Coenonympha pamphilus;Coenonympha pamphilus;1;Coenonympha pamphilus;0.999999999999829;
MH419142;Lepidoptera;Celastrina argiolus;Celastrina argiolus;1;Celastrina argiolus;0.999527385150531;
MH419143;Lepidoptera;Lasiommata paramegaera;Lasiommata paramegaera;0.999;Lasiommata paramegaera;0.995283446287531;
MH419145;Lepidoptera;Carcharodus alceae;Carcharodus alceae;0.999;Carcharodus alceae;0.999999996339511;
MH419146;Lepidoptera;Carcharodus alceae;Carcharodus alceae;1;Carcharodus alceae;0.999999998465938;
MH419148;Lepidoptera;Carcharodus alceae;Carcharodus alceae;1;Carcharodus alceae;0.999999994142229;
MH419149;Lepidoptera;Callophrys rubi;NA;0.011;Callophrys rubi;0.997712979651619;
MH419150;Lepidoptera;Maniola cecilia;Pyronia cecilia;1;Pyronia cecilia;1;
MH419152;Lepidoptera;Lasiommata paramegaera;Lasiommata paramegaera;0.999;Lasiommata paramegaera;0.995283446287531;
MH419153;Lepidoptera;Favonius quercus;NA;0.086;Favonius quercus;0.870751085723328;
MH419154;Lepidoptera;Coenonympha corinna;Coenonympha corinna;1;Coenonympha corinna;0.999999999995879;
MH419155;Lepidoptera;Melitaea nevadensis;Melitaea celadussa;0.964;Melitaea celadussa;0.989024329521702;
MH419156;Lepidoptera;Callophrys rubi;NA;0.001;Callophrys rubi;0.998885705938309;

MH419157;Lepidoptera;Maniola cecilia;Pyronia cecilia;1;Pyronia cecilia;1;
MH419158;Lepidoptera;Argynnis paphia;Argynnis paphia;1;Argynnis paphia;0.999999991389927;
MH419159;Lepidoptera;Charaxes jasius;Charaxes jasius;NA;Charaxes jasius;0.990397821479112;
MH419162;Lepidoptera;Lasiommata megera;Lasiommata megera;1;Lasiommata megera;0.999806890129977;
MH419163;Lepidoptera;Coenonympha corinna;Coenonympha corinna;1;Coenonympha corinna;0.999999853807008;
MH419164;Lepidoptera;Celastrina argiolus;Celastrina argiolus;1;Celastrina argiolus;0.999631157817044;
MH419166;Lepidoptera;Coenonympha pamphilus;Coenonympha pamphilus;1;Coenonympha pamphilus;0.999999999997527;
MH419167;Lepidoptera;Vanessa atalanta;Vanessa atalanta;1;Vanessa atalanta;0.999999999647542;
MH419168;Lepidoptera;Lasiommata megera;Lasiommata megera;1;Lasiommata megera;0.999379144055441;
MH419169;Lepidoptera;Lasiommata paramegaera;Lasiommata paramegaera;0.999;Lasiommata paramegaera;0.995283446287531;
MH419171;Lepidoptera;Colias crocea;NA;0.059;NA;0.999999999999738;
MH419174;Lepidoptera;Lasiommata megera;Lasiommata megera;1;Lasiommata megera;0.999379144055441;
MH419179;Lepidoptera;Coenonympha corinna;Coenonympha corinna;1;Coenonympha corinna;0.999999556334798;
MH419180;Lepidoptera;Colias crocea;NA;0.059;NA;0.999999999999738;
MH419182;Lepidoptera;Callophrys rubi;Callophrys rubi;0.006;Callophrys rubi;0.998441800271511;
MH419183;Lepidoptera;Pyrgus armoricanus;Pyrgus armoricanus;1;Pyrgus armoricanus;0.999997353848817;
MH419184;Lepidoptera;Argynnis paphia;Argynnis paphia;1;Argynnis paphia;0.999999991389927;
MH419189;Lepidoptera;Coenonympha corinna;Coenonympha corinna;1;Coenonympha corinna;0.999999999995879;
MH419190;Lepidoptera;Hipparchia neomiris;Hipparchia neomiris;1;Hipparchia neomiris;0.999999999999801;
MH419191;Lepidoptera;Gegenes pumilio;Gegenes pumilio;1;Gegenes pumilio;0.999986726679266;
MH419192;Lepidoptera;Coenonympha pamphilus;Coenonympha pamphilus;1;Coenonympha pamphilus;0.999999999999744;
MH419193;Lepidoptera;Lampides boeticus;Lampides boeticus;1;Lampides boeticus;0.999999999658513;
MH419194;Lepidoptera;Gegenes pumilio;Gegenes pumilio;0.006;Gegenes pumilio;0.999976928027983;
MH419195;Lepidoptera;Anthocharis cardamines;Anthocharis cardamines;1;Anthocharis cardamines;0.999999986709838;
MH419196;Lepidoptera;Colias crocea;NA;0.059;NA;0.999999999999738;
MH419197;Lepidoptera;Coenonympha corinna;Coenonympha corinna;1;Coenonympha corinna;0.999999999960579;
MH419198;Lepidoptera;Gonepteryx cleopatra;Gonepteryx cleopatra;0.998;Gonepteryx cleopatra;0.999118636582266;
MH419200;Lepidoptera;Pieris napi;NA;0.017;Pieris napi;0.773618468477091;
MH419201;Lepidoptera;Lasiommata megera;Lasiommata megera;1;Lasiommata megera;0.999379144055441;
MH419204;Lepidoptera;Gonepteryx rhamni;NA;0.194;Gonepteryx rhamni;0.99583141915324;
MH419209;Lepidoptera;Maniola tithonus;Pyronia tithonus;1;Pyronia tithonus;0.999999999999972;
MH419211;Lepidoptera;Pieris napi;NA;NA;Pieris napi;0.883162684455157;
MH419213;Lepidoptera;Anthocharis cardamines;Anthocharis cardamines;1;Anthocharis cardamines;0.999999943434575;
MH419214;Lepidoptera;Argynnis paphia;Argynnis paphia;1;Argynnis paphia;0.999999991389927;
MH419215;Lepidoptera;Maniola tithonus;Pyronia tithonus;1;Pyronia tithonus;1;
MH419216;Lepidoptera;Celastrina argiolus;Celastrina argiolus;1;Celastrina argiolus;0.999527385150531;
MH419217;Lepidoptera;Lasiommata paramegaera;Lasiommata paramegaera;0.999;Lasiommata paramegaera;0.995283446287531;
MH419218;Lepidoptera;Gegenes pumilio;NA;0.92;Gegenes pumilio;0.999975451721427;
MH419220;Lepidoptera;Gegenes pumilio;Gegenes pumilio;1;Gegenes pumilio;0.999986726679266;
MH419222;Lepidoptera;Lycaena phlaeas;Lycaena phlaeas;1;Lycaena phlaeas;0.999999996363186;
MH419223;Lepidoptera;Vanessa cardui;Vanessa cardui;1;Vanessa cardui;0.999999370067598;
MH419225;Lepidoptera;Satyrium ilicis;Satyrium ilicis;1;Satyrium ilicis;0.999999987901305;
MH419226;Lepidoptera;Maniola cecilia;Pyronia cecilia;1;Pyronia cecilia;0.999999999999972;
MH419227;Lepidoptera;Maniola tithonus;Pyronia tithonus;1;Pyronia tithonus;1;
MH419228;Lepidoptera;Lampides boeticus;Lampides boeticus;1;Lampides boeticus;0.999999999545906;
MH419230;Lepidoptera;Celastrina argiolus;Celastrina argiolus;1;Celastrina argiolus;0.999527385150531;
MH419231;Lepidoptera;Coenonympha pamphilus;Coenonympha pamphilus;1;Coenonympha pamphilus;0.999999999999744;
MH419235;Lepidoptera;Maniola jurtina;NA;0.038;NA;0.999999999999524;
MH419236;Lepidoptera;Colias crocea;NA;0.059;NA;0.999999999999738;
MH419237;Lepidoptera;Maniola cecilia;Pyronia cecilia;NA;Pyronia cecilia;1;
MH419241;Lepidoptera;Colias crocea;NA;0.059;NA;0.999999999999738;
MH419242;Lepidoptera;Celastrina argiolus;Celastrina argiolus;1;Celastrina argiolus;0.999527385150531;
MH419243;Lepidoptera;Gegenes pumilio;Gegenes pumilio;1;Gegenes pumilio;0.999986726679266;
MH419244;Lepidoptera;Celastrina argiolus;Celastrina argiolus;1;Celastrina argiolus;0.999527385150531;
MH419245;Lepidoptera;Pieris napi;NA;0.028;Pieris napi;0.888205720664699;
MH419246;Lepidoptera;Lycaena phlaeas;Lycaena phlaeas;1;Lycaena phlaeas;0.999999996363186;
MH419247;Lepidoptera;Coenonympha pamphilus;Coenonympha pamphilus;1;Coenonympha pamphilus;0.999999999999744;
MH419249;Lepidoptera;Papilio machaon;Papilio machaon;0.003;Papilio machaon;0.997288237409893;

MH419250;Lepidoptera;Colias crocea;NA;0.059;NA;0.999999999999738;
MH419252;Lepidoptera;Anthocharis cardamines;Anthocharis cardamines;1;Anthocharis cardamines;0.99999997953563;
MH419254;Lepidoptera;Celastrina argiolus;Celastrina argiolus;1;Celastrina argiolus;0.999527385150531;
MH419255;Lepidoptera;Callophrys rubi;NA;0.019;Callophrys rubi;0.998201459637982;
MH419256;Lepidoptera;Maniola cecilia;Pyronia cecilia;NA;Pyronia cecilia;1;
MH419258;Lepidoptera;Lycaena phlaeas;Lycaena phlaeas;1;Lycaena phlaeas;0.999999998167567;
MH419259;Lepidoptera;Maniola jurtina;NA;0.038;NA;0.999999999999524;
MH419262;Lepidoptera;Pieris napi;NA;NA;Pieris napi;0.88800510213663;
MH419263;Lepidoptera;Colias crocea;NA;0.059;NA;0.999999999999738;
MH419266;Lepidoptera;Papilio machaon;Papilio machaon;0.001;Papilio machaon;0.999463129150422;
MH419267;Lepidoptera;Pieris napi;Pieris napi;0.004;Pieris napi;0.920488351592687;
MH419269;Lepidoptera;Lasiommata megera;Lasiommata megera;1;Lasiommata megera;0.999379144055441;
MH419270;Lepidoptera;Pyrgus armoricanus;Pyrgus armoricanus;1;Pyrgus armoricanus;0.999997353848817;
MH419272;Lepidoptera;Thymelicus acteon;Thymelicus acteon;1;Thymelicus acteon;0.99999999752248;
MH419273;Lepidoptera;Lasiommata megera;Lasiommata megera;1;Lasiommata megera;0.999379144055441;
MH419274;Lepidoptera;Lasiommata megera;Lasiommata megera;1;Lasiommata megera;0.999379144055441;
MH419276;Lepidoptera;Glaucopsyche alexis;NA;NA;Glaucopsyche alexis;0.899237597871183;
MH419277;Lepidoptera;Coenonympha pamphilus;Coenonympha pamphilus;1;Coenonympha pamphilus;0.999999999999744;
MH419278;Lepidoptera;Vanessa atalanta;Vanessa atalanta;1;Vanessa atalanta;0.999999998973152;
MH419279;Lepidoptera;Thymelicus acteon;Thymelicus acteon;1;Thymelicus acteon;0.999999995239193;
MH419280;Lepidoptera;Lasiommata megera;Lasiommata megera;1;Lasiommata megera;0.999379144055441;
MH419281;Lepidoptera;Maniola jurtina;NA;0.001;Maniola jurtina;0.939220105860875;
MH419282;Lepidoptera;Pieris napi;NA;0.011;Pieris napi;0.801985394724814;
MH419283;Lepidoptera;Pyrgus armoricanus;Pyrgus armoricanus;1;Pyrgus armoricanus;0.999997353848817;
MH419284;Lepidoptera;Lycaena phlaeas;Lycaena phlaeas;1;Lycaena phlaeas;0.999999996363186;
MH419286;Lepidoptera;Carcharodus alceae;Carcharodus alceae;0.001;Carcharodus alceae;0.999999994870137;
MH419287;Lepidoptera;Coenonympha corinna;Coenonympha corinna;1;Coenonympha corinna;0.99999999942446;
MH419288;Lepidoptera;Carcharodus alceae;Carcharodus alceae;0.999;Carcharodus alceae;0.999999998008462;
MH419289;Lepidoptera;Glaucopsyche alexis;Glaucopsyche alexis;0.001;Glaucopsyche alexis;0.94042380088325;
MH419292;Lepidoptera;Issoria lathonia;Issoria lathonia;1;Issoria lathonia;0.999999999989257;
MH419294;Lepidoptera;Colias crocea;NA;0.059;NA;0.999999999999738;
MH419295;Lepidoptera;Maniola cecilia;Pyronia cecilia;1;Pyronia cecilia;1;
MH419296;Lepidoptera;Plebejus idas;NA;0.001;NA;0.994620093546684;
MH419300;Lepidoptera;Issoria lathonia;Issoria lathonia;1;Issoria lathonia;0.999999999989257;
MH419301;Lepidoptera;Maniola cecilia;Pyronia cecilia;NA;Pyronia cecilia;1;
MH419302;Lepidoptera;Colias crocea;NA;0.059;NA;0.999999999999738;
MH419303;Lepidoptera;Charaxes jasius;Charaxes jasius;0.001;Charaxes jasius;0.992442307611551;
MH419304;Lepidoptera;Colias crocea;NA;0.059;NA;0.999999999999738;
MH419305;Lepidoptera;Coenonympha pamphilus;Coenonympha pamphilus;1;Coenonympha pamphilus;0.999999999999744;
MH419306;Lepidoptera;Colias crocea;NA;0.059;NA;0.999999999999738;
MH419309;Lepidoptera;Gegenes pumilio;Gegenes pumilio;1;Gegenes pumilio;0.999986726679266;
MH419311;Lepidoptera;Charaxes jasius;Charaxes jasius;NA;Charaxes jasius;0.990397821479112;
MH419312;Lepidoptera;Vanessa cardui;Vanessa cardui;1;Vanessa cardui;0.999999459983318;
MH419314;Lepidoptera;Papilio machaon;Papilio machaon;NA;Papilio machaon;0.996538843275905;
MH419317;Lepidoptera;Lasiommata paramegaera;Lasiommata paramegaera;0.999;Lasiommata paramegaera;0.995283446287531;
MH419318;Lepidoptera;Carcharodus alceae;Carcharodus alceae;1;Carcharodus alceae;0.999999998465938;
MH419319;Lepidoptera;Maniola cecilia;Pyronia cecilia;1;Pyronia cecilia;0.999999999999972;
MH419321;Lepidoptera;Thymelicus acteon;Thymelicus acteon;1;Thymelicus acteon;0.999999996091617;
MH419323;Lepidoptera;Lycaena phlaeas;Lycaena phlaeas;1;Lycaena phlaeas;0.999999998167567;
MH419324;Lepidoptera;Celastrina argiolus;Celastrina argiolus;1;Celastrina argiolus;0.999527385150531;
MH419325;Lepidoptera;Aricia agestis;NA;0.067;Aricia agestis;0.999509743227786;
MH419327;Lepidoptera;Pyrgus armoricanus;Pyrgus armoricanus;1;Pyrgus armoricanus;0.999997353848817;
MH419330;Lepidoptera;Celastrina argiolus;Celastrina argiolus;1;Celastrina argiolus;0.999037794018796;
MH419336;Lepidoptera;Maniola cecilia;Pyronia cecilia;NA;Pyronia cecilia;1;
MH419337;Lepidoptera;Lycaena phlaeas;Lycaena phlaeas;1;Lycaena phlaeas;0.999999996363186;
MH419338;Lepidoptera;Lycaena phlaeas;Lycaena phlaeas;1;Lycaena phlaeas;0.999999996363186;
MH419339;Lepidoptera;Hipparchia neomiris;Hipparchia neomiris;1;Hipparchia neomiris;0.999999999999915;
MH419343;Lepidoptera;Anthocharis cardamines;Anthocharis cardamines;1;Anthocharis cardamines;0.999999984567239;
MH419344;Lepidoptera;Vanessa atalanta;Vanessa atalanta;1;Vanessa atalanta;0.999999999383476;
MH419345;Lepidoptera;Lampides boeticus;Lampides boeticus;1;Lampides boeticus;0.999999999281187;

MH419346;Lepidoptera;Celastrina argiolus;Celastrina argiolus;1;Celastrina argiolus;0.999037794018796;
MH419347;Lepidoptera;Coenonympha pamphilus;Coenonympha pamphilus;1;Coenonympha pamphilus;0.999999999999744;
MH419348;Lepidoptera;Maniola cecilia;Pyronia cecilia;NA;Pyronia cecilia;1;
MH419350;Lepidoptera;Colias crocea;NA;0.059;NA;0.999999999999738;
MH419353;Lepidoptera;Celastrina argiolus;Celastrina argiolus;1;Celastrina argiolus;0.999527385150531;
MH419354;Lepidoptera;Lasiommata megera;Lasiommata megera;1;Lasiommata megera;0.999379144055441;
MH419355;Lepidoptera;Pyrgus armoricanus;Pyrgus armoricanus;1;Pyrgus armoricanus;0.999997353848817;
MH419357;Lepidoptera;Lycaena phlaeas;Lycaena phlaeas;1;Lycaena phlaeas;0.999999996363186;
MH419362;Lepidoptera;Vanessa cardui;Vanessa cardui;1;Vanessa cardui;0.999999803450484;
MH419369;Lepidoptera;Glaucopsyche alexis;Glaucopsyche alexis;0.001;Glaucopsyche alexis;0.938298388869861;
MH419370;Lepidoptera;Lasiommata megera;Lasiommata megera;1;Lasiommata megera;0.999379144055441;
MH419372;Lepidoptera;Gonepteryx rhamni;NA;0.474;Gonepteryx rhamni;0.982076396127858;
MH419373;Lepidoptera;Lampides boeticus;Lampides boeticus;1;Lampides boeticus;0.999999998557229;
MH419375;Lepidoptera;Vanessa cardui;Vanessa cardui;1;Vanessa cardui;0.999998479972181;
MH419377;Lepidoptera;Lasiommata megera;Lasiommata megera;1;Lasiommata megera;0.999379144055441;
MH419381;Lepidoptera;Maniola cecilia;Pyronia cecilia;1;Pyronia cecilia;1;
MH419382;Lepidoptera;Colias crocea;NA;0.059;NA;0.999999999999738;
MH419384;Lepidoptera;Gonepteryx cleopatra;Gonepteryx cleopatra;0.998;Gonepteryx cleopatra;0.999118636582266;
MH419385;Lepidoptera;Lampides boeticus;Lampides boeticus;1;Lampides boeticus;0.999999998728612;
MH419386;Lepidoptera;Lasiommata megera;Lasiommata megera;1;Lasiommata megera;0.999379144055441;
MH419387;Lepidoptera;Coenonympha pamphilus;Coenonympha pamphilus;1;Coenonympha pamphilus;0.999999999999744;
MH419388;Lepidoptera;Maniola tithonus;Pyronia tithonus;1;Pyronia tithonus;1;
MH419389;Lepidoptera;Lycaena phlaeas;Lycaena phlaeas;1;Lycaena phlaeas;0.999999996363186;
MH419392;Lepidoptera;Melitaea didyma;Melitaea didyma;1;Melitaea didyma;0.999945816868155;
MH419394;Lepidoptera;Gonepteryx cleopatra;Gonepteryx cleopatra;0.998;Gonepteryx cleopatra;0.999118636582266;
MH419396;Lepidoptera;Gegenes pumilio;Gegenes pumilio;1;Gegenes pumilio;0.999986726679266;
MH419397;Lepidoptera;Lampides boeticus;Lampides boeticus;1;Lampides boeticus;0.999999999561368;
MH419400;Lepidoptera;Pieris napi;Pieris napi;NA;Pieris napi;0.939506750684135;
MH419401;Lepidoptera;Carcharodus alceae;Carcharodus alceae;0.001;Carcharodus alceae;0.999999997849443;
MH419402;Lepidoptera;Gonepteryx cleopatra;Gonepteryx cleopatra;0.998;Gonepteryx cleopatra;0.999118636582266;
MH419404;Lepidoptera;Coenonympha pamphilus;Coenonympha pamphilus;1;Coenonympha pamphilus;0.999999999981668;
MH419405;Lepidoptera;Maniola cecilia;Pyronia cecilia;NA;Pyronia cecilia;1;
MH419407;Lepidoptera;Gonepteryx cleopatra;Gonepteryx cleopatra;0.998;Gonepteryx cleopatra;0.999118636582266;
MH419408;Lepidoptera;Favonius quercus;NA;0.002;Favonius quercus;0.732659082125295;
MH419412;Lepidoptera;Gonepteryx cleopatra;Gonepteryx cleopatra;0.999;Gonepteryx cleopatra;0.99900166502855;
MH419413;Lepidoptera;Coenonympha pamphilus;Coenonympha pamphilus;1;Coenonympha pamphilus;0.999999999999744;
MH419414;Lepidoptera;Callophrys rubi;NA;0.019;Callophrys rubi;0.998201459637982;
MH419417;Lepidoptera;Aglais ichnusa;Aglais ichnusa;0.998;NA;0.999999996206876;
MH419419;Lepidoptera;Coenonympha pamphilus;Coenonympha pamphilus;1;Coenonympha pamphilus;0.999999999999432;
MH419420;Lepidoptera;Lampides boeticus;Lampides boeticus;1;Lampides boeticus;0.999999999690317;
MH419421;Lepidoptera;Coenonympha pamphilus;Coenonympha pamphilus;1;Coenonympha pamphilus;0.999999999999744;
MH419422;Lepidoptera;Satyrium ilicis;Satyrium ilicis;1;Satyrium ilicis;0.999999987901305;
MH419425;Lepidoptera;Papilio machaon;NA;0.004;Papilio machaon;0.786457751316236;
MH419426;Lepidoptera;Aricia agestis;NA;0.095;Aricia agestis;0.998129958002497;
MH419428;Lepidoptera;Coenonympha pamphilus;Coenonympha pamphilus;1;Coenonympha pamphilus;0.999999999999829;
MH419429;Lepidoptera;Maniola cecilia;Pyronia cecilia;NA;Pyronia cecilia;1;
MH419432;Lepidoptera;Coenonympha pamphilus;Coenonympha pamphilus;1;Coenonympha pamphilus;0.999999999995282;
MH419433;Lepidoptera;Aricia agestis;NA;0.067;Aricia agestis;0.999509743227786;
MH419434;Lepidoptera;Lasiommata paramegaera;Lasiommata paramegaera;0.999;Lasiommata paramegaera;0.995283446287531;
MH419435;Lepidoptera;Pieris napi;NA;0.011;Pieris napi;0.801985394724814;
MH419438;Lepidoptera;Gonepteryx cleopatra;Gonepteryx cleopatra;0.998;Gonepteryx cleopatra;0.999118636582266;
MH419439;Lepidoptera;Lampides boeticus;Lampides boeticus;NA;Lampides boeticus;0.9999999990861;
MH419440;Lepidoptera;Argynnis paphia;Argynnis paphia;1;Argynnis paphia;0.999999991389927;
MH419441;Lepidoptera;Carcharodus alceae;Carcharodus alceae;1;Carcharodus alceae;0.999999996405506;
MH419443;Lepidoptera;Melitaea didyma;Melitaea didyma;1;Melitaea didyma;0.999945816868155;
MH419444;Lepidoptera;Celastrina argiolus;Celastrina argiolus;1;Celastrina argiolus;0.999652809829397;

MH419445;Lepidoptera;Pyrgus armoricanus;Pyrgus armoricanus;1;Pyrgus armoricanus;0.999997353848817;
MH419446;Lepidoptera;Anthocharis cardamines;Anthocharis cardamines;1;Anthocharis cardamines;0.999999907874159;
MH419448;Lepidoptera;Lasiommata megera;Lasiommata megera;1;Lasiommata megera;0.999379144055441;
MH419451;Lepidoptera;Carcharodus alceae;Carcharodus alceae;1;Carcharodus alceae;0.999999998465938;
MH419454;Lepidoptera;Celastrina argiolus;Celastrina argiolus;1;Celastrina argiolus;0.999631157817044;
MH419458;Lepidoptera;Papilio machaon;Papilio machaon;0.001;Papilio machaon;0.998357199376497;
MH419459;Lepidoptera;Maniola tithonus;Pyronia tithonus;1;Pyronia tithonus;1;
MH419461;Lepidoptera;Papilio machaon;Papilio machaon;0.003;Papilio machaon;0.999482752749035;
MH419462;Lepidoptera;Vanessa atalanta;Vanessa atalanta;1;Vanessa atalanta;0.999999999489717;
MH419463;Lepidoptera;Argynnis paphia;Argynnis paphia;1;Argynnis paphia;0.999999991389927;
MH419464;Lepidoptera;Aricia cramera;Aricia cramera;1;Aricia cramera;0.999993731037033;
MH419466;Lepidoptera;Maniola cecilia;Pyronia cecilia;NA;Pyronia cecilia;1;
MH419467;Lepidoptera;Charaxes jasius;Charaxes jasius;NA;Charaxes jasius;0.990397821479112;
MH419470;Lepidoptera;Aricia agestis;Aricia agestis;1;Aricia agestis;0.988437755993415;
MH419471;Lepidoptera;Lasiommata megera;Lasiommata megera;1;Lasiommata megera;0.999379144055441;
MH419472;Lepidoptera;Coenonympha pamphilus;Coenonympha pamphilus;1;Coenonympha pamphilus;0.999999999999744;
MH419473;Lepidoptera;Favonius quercus;NA;0.002;Favonius quercus;0.732659082125295;
MH419476;Lepidoptera;Coenonympha pamphilus;Coenonympha pamphilus;1;Coenonympha pamphilus;0.999999999999403;
MH419477;Lepidoptera;Coenonympha pamphilus;Coenonympha pamphilus;1;Coenonympha pamphilus;0.999999999999744;
MH419478;Lepidoptera;Charaxes jasius;Charaxes jasius;NA;Charaxes jasius;0.979798503826812;
MH419480;Lepidoptera;Aglais ichnusa;Aglais ichnusa;0.998;NA;0.999999996206876;
MH419481;Lepidoptera;Lampides boeticus;Lampides boeticus;1;Lampides boeticus;0.999999999545906;
MH419482;Lepidoptera;Lasiommata paramegaera;Lasiommata paramegaera;0.999;Lasiommata paramegaera;0.995283446287531;
MH419484;Lepidoptera;Lampides boeticus;Lampides boeticus;1;Lampides boeticus;0.999999998557229;
MH419485;Lepidoptera;Lasiommata paramegaera;Lasiommata paramegaera;0.999;Lasiommata paramegaera;0.995283446287531;
MH419486;Lepidoptera;Coenonympha pamphilus;Coenonympha pamphilus;1;Coenonympha pamphilus;0.999999999999744;
MH419487;Lepidoptera;Argynnis paphia;Argynnis paphia;1;Argynnis paphia;0.999999991389927;
MH419488;Lepidoptera;Colias crocea;NA;0.059;NA;0.999999999999738;
MH419489;Lepidoptera;Maniola tithonus;Pyronia tithonus;1;Pyronia tithonus;1;
MH419490;Lepidoptera;Gonepteryx cleopatra;Gonepteryx cleopatra;0.998;Gonepteryx cleopatra;0.999118636582266;
MH419492;Lepidoptera;Carcharodus alceae;Carcharodus alceae;1;Carcharodus alceae;0.999999998465938;
MH419493;Lepidoptera;Coenonympha pamphilus;Coenonympha pamphilus;1;Coenonympha pamphilus;0.999999999999744;
MH419494;Lepidoptera;Melitaea didyma;Melitaea didyma;1;Melitaea didyma;0.999945816868155;
MH419495;Lepidoptera;Lasiommata megera;Lasiommata megera;1;Lasiommata megera;0.999379144055441;
MH419496;Lepidoptera;Carcharodus alceae;Carcharodus alceae;1;Carcharodus alceae;0.999999990863188;
MH419499;Lepidoptera;Gegenes pumilio;Gegenes pumilio;1;Gegenes pumilio;0.999986726679266;
MH419500;Lepidoptera;Charaxes jasius;Charaxes jasius;0.001;Charaxes jasius;0.990234369397197;
MH419501;Lepidoptera;Coenonympha pamphilus;Coenonympha pamphilus;1;Coenonympha pamphilus;0.999999999983061;
MH419504;Lepidoptera;Maniola tithonus;Pyronia tithonus;1;Pyronia tithonus;1;
MH419508;Lepidoptera;Maniola cecilia;Pyronia cecilia;1;Pyronia cecilia;1;
MH419510;Lepidoptera;Carcharodus alceae;Carcharodus alceae;0.999;Carcharodus alceae;0.999999996339511;
MH419511;Lepidoptera;Colias crocea;NA;0.059;NA;0.999999999999738;
MH419512;Lepidoptera;Aglais ichnusa;Aglais ichnusa;0.003;NA;0.999999990022183;
MH419513;Lepidoptera;Coenonympha pamphilus;Coenonympha pamphilus;1;Coenonympha pamphilus;0.999999999999488;
MH419515;Lepidoptera;Melitaea nevadensis;NA;0.014;Melitaea celadussa;0.797886056890592;
MH419516;Lepidoptera;Gonepteryx cleopatra;Gonepteryx cleopatra;0.998;Gonepteryx cleopatra;0.999118636582266;
MH419518;Lepidoptera;Pyrgus armoricanus;Pyrgus armoricanus;1;Pyrgus armoricanus;0.999997353848817;
MH419519;Lepidoptera;Maniola tithonus;Pyronia tithonus;1;Pyronia tithonus;1;
MH419522;Lepidoptera;Coenonympha pamphilus;Coenonympha pamphilus;1;Coenonympha pamphilus;0.999999999999744;
MH419524;Lepidoptera;Gonepteryx cleopatra;Gonepteryx cleopatra;0.998;Gonepteryx cleopatra;0.999118636582266;
MH419527;Lepidoptera;Celastrina argiolus;Celastrina argiolus;1;Celastrina argiolus;0.999660808952023;
MH419528;Lepidoptera;Colias crocea;NA;0.059;NA;0.999999999999738;
MH419531;Lepidoptera;Hipparchia statilinus;Hipparchia statilinus;1;Hipparchia statilinus;0.999999999999943;
MH419532;Lepidoptera;Callophrys rubi;NA;0.003;Callophrys rubi;0.996753822785804;
MH419533;Lepidoptera;Callophrys rubi;NA;0.019;Callophrys rubi;0.998201459637982;

MH419534;Lepidoptera;Papilio machaon;Papilio machaon;NA;Papilio machaon;0.99931256565874;
MH419535;Lepidoptera;Melitaea didyma;Melitaea didyma;1;Melitaea didyma;0.999945824175549;
MH419538;Lepidoptera;Coenonympha corinna;Coenonympha corinna;1;Coenonympha corinna;0.999999999567109;
MH419542;Lepidoptera;Carcharodus alceae;Carcharodus alceae;0.006;Carcharodus alceae;0.999999965990839;
MH419543;Lepidoptera;Lasiommata paramegaera;Lasiommata paramegaera;0.999;Lasiommata paramegaera;0.995283446287531;
MH419545;Lepidoptera;Hipparchia neomiris;Hipparchia neomiris;1;Hipparchia neomiris;0.999999999999801;
MH419546;Lepidoptera;Vanessa cardui;Vanessa cardui;1;Vanessa cardui;0.999999160963125;
MH419547;Lepidoptera;Callophrys rubi;NA;0.001;Callophrys rubi;0.998885705938309;
MH419548;Lepidoptera;Papilio machaon;Papilio machaon;0.003;Papilio machaon;0.999482752749035;
MH419549;Lepidoptera;Vanessa atalanta;Vanessa atalanta;1;Vanessa atalanta;0.999999999757762;
MH419550;Lepidoptera;Coenonympha corinna;Coenonympha corinna;1;Coenonympha corinna;0.999999999995879;
MH419552;Lepidoptera;Callophrys rubi;NA;0.003;Callophrys rubi;0.996753822785804;
MH419553;Lepidoptera;Lasiommata paramegaera;Lasiommata paramegaera;0.999;Lasiommata paramegaera;0.995283446287531;
MH419555;Lepidoptera;Lycaena phlaeas;Lycaena phlaeas;1;Lycaena phlaeas;0.999999997206857;
MH419556;Lepidoptera;Carcharodus alceae;Carcharodus alceae;0.999;Carcharodus alceae;0.999999998008462;
MH419557;Lepidoptera;Lampides boeticus;Lampides boeticus;1;Lampides boeticus;0.999999998355293;
MH419561;Lepidoptera;Colias crocea;NA;0.059;NA;0.999999999999738;
MH419563;Lepidoptera;Pieris napi;NA;0.011;Pieris napi;0.801985394724814;
MH419565;Lepidoptera;Melitaea cinxia;Melitaea cinxia;1;Melitaea cinxia;0.999999999999687;
MH419567;Lepidoptera;Maniola jurtina;NA;0.146;NA;0.999999999999341;
MH419568;Lepidoptera;Celastrina argiolus;Celastrina argiolus;1;Celastrina argiolus;0.999037794018796;
MH419569;Lepidoptera;Lampides boeticus;Lampides boeticus;1;Lampides boeticus;0.999999999545906;
MH419570;Lepidoptera;Vanessa atalanta;Vanessa atalanta;1;Vanessa atalanta;0.999999999757762;
MH419575;Lepidoptera;Lampides boeticus;Lampides boeticus;1;Lampides boeticus;0.999999999545906;
MH419576;Lepidoptera;Lycaena phlaeas;Lycaena phlaeas;1;Lycaena phlaeas;0.999999996363186;
MH419577;Lepidoptera;Lampides boeticus;Lampides boeticus;1;Lampides boeticus;0.999999999411585;
MH419578;Lepidoptera;Gonepteryx cleopatra;Gonepteryx cleopatra;0.998;Gonepteryx cleopatra;0.999118636582266;
MH419579;Lepidoptera;Argynnis paphia;Argynnis paphia;1;Argynnis paphia;0.999999991389927;
MH419582;Lepidoptera;Coenonympha corinna;Coenonympha corinna;1;Coenonympha corinna;0.999999999567109;
MH419583;Lepidoptera;Melitaea didyma;Melitaea didyma;1;Melitaea didyma;0.999945824175549;
MH419584;Lepidoptera;Colias crocea;NA;0.059;NA;0.999999999999738;
MH419585;Lepidoptera;Charaxes jasius;Charaxes jasius;NA;Charaxes jasius;0.990397821479112;
MH419587;Lepidoptera;Lasiommata megera;Lasiommata megera;1;Lasiommata megera;0.999379144055441;
MH419588;Lepidoptera;Vanessa cardui;Vanessa cardui;1;Vanessa cardui;0.999999459983318;
MH419589;Lepidoptera;Callophrys rubi;NA;0.019;Callophrys rubi;0.998201459637982;
MH419590;Lepidoptera;Coenonympha corinna;Coenonympha corinna;1;Coenonympha corinna;0.999999556334798;
MH419591;Lepidoptera;Maniola tithonus;Pyronia tithonus;1;Pyronia tithonus;1;
MH419593;Lepidoptera;Favonius quercus;NA;0.002;Favonius quercus;0.732659082125295;
MH419594;Lepidoptera;Vanessa cardui;Vanessa cardui;1;Vanessa cardui;0.999997579965112;
MH419595;Lepidoptera;Coenonympha pamphilus;Coenonympha pamphilus;1;Coenonympha pamphilus;0.999999999964302;
MH419596;Lepidoptera;Argynnis paphia;Argynnis paphia;1;Argynnis paphia;0.999999991389927;
MH419597;Lepidoptera;Gonepteryx cleopatra;Gonepteryx cleopatra;0.998;Gonepteryx cleopatra;0.999118636582266;
MH419599;Lepidoptera;Lasiommata megera;Lasiommata megera;1;Lasiommata megera;0.999379144055441;
MH419601;Lepidoptera;Gonepteryx cleopatra;Gonepteryx cleopatra;0.998;Gonepteryx cleopatra;0.999118636582266;
MH419603;Lepidoptera;Coenonympha pamphilus;Coenonympha pamphilus;1;Coenonympha pamphilus;0.999999999999602;
MH419605;Lepidoptera;Celastrina argiolus;Celastrina argiolus;1;Celastrina argiolus;0.999527385150531;
MH419607;Lepidoptera;Lycaena phlaeas;Lycaena phlaeas;1;Lycaena phlaeas;0.999999996363186;
MH419608;Lepidoptera;Vanessa atalanta;Vanessa atalanta;1;Vanessa atalanta;0.999999999757762;
MH419609;Lepidoptera;Celastrina argiolus;Celastrina argiolus;1;Celastrina argiolus;0.999631157817044;
MH419612;Lepidoptera;Lasiommata paramegaera;Lasiommata paramegaera;0.999;Lasiommata paramegaera;0.995283446287531;
MH419613;Lepidoptera;Maniola jurtina;NA;0.038;NA;0.999999999999524;
MH419614;Lepidoptera;Papilio machaon;Papilio machaon;NA;Papilio machaon;0.998489747694706;
MH419616;Lepidoptera;Gonepteryx rhamni;Gonepteryx rhamni;0.008;Gonepteryx rhamni;0.999527533936108;
MH419617;Lepidoptera;Lycaena phlaeas;Lycaena phlaeas;1;Lycaena phlaeas;0.999999996363186;
MH419619;Lepidoptera;Celastrina argiolus;Celastrina argiolus;1;Celastrina argiolus;0.999358002620181;
MH419620;Lepidoptera;Lasiommata megera;Lasiommata megera;1;Lasiommata megera;0.999379144055441;
MH419621;Lepidoptera;Celastrina argiolus;Celastrina argiolus;1;Celastrina argiolus;0.999527385150531;

MH419622;Lepidoptera;Vanessa cardui;Vanessa cardui;1;Vanessa cardui;0.999999803450484;
MH419625;Lepidoptera;Lycaena phlaeas;Lycaena phlaeas;1;Lycaena phlaeas;0.999999996363186;
MH419626;Lepidoptera;Coenonympha pamphilus;Coenonympha pamphilus;1;Coenonympha pamphilus;0.999999999999744;
MH419627;Lepidoptera;Aglais ichnusa;NA;NA;Aglais ichnusa;0.712643726050504;
MH419628;Lepidoptera;Gonepteryx rhamni;NA;0.183;Gonepteryx rhamni;0.991353407168274;
MH419629;Lepidoptera;Lasiommata megera;Lasiommata megera;1;Lasiommata megera;0.999379144055441;
MH419634;Lepidoptera;Argynnis paphia;Argynnis paphia;1;Argynnis paphia;0.999999991641772;
MH419635;Lepidoptera;Glaucopsyche alexis;Glaucopsyche alexis;0.001;Glaucopsyche alexis;0.94042380088325;
MH419636;Lepidoptera;Glaucopsyche alexis;Glaucopsyche alexis;0.001;Glaucopsyche alexis;0.94042380088325;
MH419638;Lepidoptera;Gegenes pumilio;Gegenes pumilio;1;Gegenes pumilio;0.999986726679266;
MH419641;Lepidoptera;Vanessa atalanta;Vanessa atalanta;1;Vanessa atalanta;0.999999999757762;
MH419642;Lepidoptera;Maniola cecilia;Pyronia cecilia;NA;Pyronia cecilia;1;
MH419644;Lepidoptera;Coenonympha corinna;Coenonympha corinna;1;Coenonympha corinna;0.999999929826745;
MH419646;Lepidoptera;Vanessa cardui;Vanessa cardui;1;Vanessa cardui;0.999999850530696;
MH419651;Lepidoptera;Colias crocea;NA;0.059;NA;0.999999999999738;
MH419653;Lepidoptera;Lycaena phlaeas;Lycaena phlaeas;1;Lycaena phlaeas;0.999999997206857;
MH419656;Lepidoptera;Favonius quercus;NA;NA;Favonius quercus;0.73046069582187;
MH419657;Lepidoptera;Celastrina argiolus;Celastrina argiolus;1;Celastrina argiolus;0.999527385150531;
MH419658;Lepidoptera;Lampides boeticus;Lampides boeticus;1;Lampides boeticus;0.999999999617842;
MH419659;Lepidoptera;Maniola jurtina;NA;0.038;NA;0.999999999999524;
MH419660;Lepidoptera;Lasiommata paramegaera;Lasiommata paramegaera;1;Lasiommata paramegaera;0.996585757479472;
MH419662;Lepidoptera;Vanessa cardui;Vanessa cardui;1;Vanessa cardui;0.999999459983318;
MH419663;Lepidoptera;Lasiommata megera;Lasiommata megera;1;Lasiommata megera;0.999379144055441;
MH419664;Lepidoptera;Gonepteryx cleopatra;Gonepteryx cleopatra;0.998;Gonepteryx cleopatra;0.999118636582266;
MH419665;Lepidoptera;Papilio machaon;Papilio machaon;NA;Papilio machaon;0.998489747694706;
MH419666;Lepidoptera;Argynnis paphia;Argynnis paphia;1;Argynnis paphia;0.999999991389927;
MH419667;Lepidoptera;Maniola cecilia;Pyronia cecilia;NA;Pyronia cecilia;1;
MH419668;Lepidoptera;Gegenes pumilio;Gegenes pumilio;1;Gegenes pumilio;0.999986726679266;
MH419669;Lepidoptera;Coenonympha pamphilus;Coenonympha pamphilus;1;Coenonympha pamphilus;0.999999999999744;
MH419670;Lepidoptera;Papilio machaon;Papilio machaon;0.003;Papilio machaon;0.999482752749035;
MH419671;Lepidoptera;Thymelicus acteon;Thymelicus acteon;1;Thymelicus acteon;0.99999999752248;
MH419673;Lepidoptera;Melitaea didyma;Melitaea didyma;1;Melitaea didyma;0.999945824175549;
MH419674;Lepidoptera;Celastrina argiolus;Celastrina argiolus;1;Celastrina argiolus;0.999527385150531;
MH419675;Lepidoptera;Maniola cecilia;Pyronia cecilia;NA;Pyronia cecilia;1;
MH419677;Lepidoptera;Callophrys rubi;NA;0.001;Callophrys rubi;0.99870518510598;
MH419678;Lepidoptera;Lampides boeticus;Lampides boeticus;1;Lampides boeticus;0.999999999545906;
MH419679;Lepidoptera;Coenonympha pamphilus;Coenonympha pamphilus;1;Coenonympha pamphilus;0.999999999999744;
MH419680;Lepidoptera;Lasiommata megera;Lasiommata megera;0.001;Lasiommata megera;0.999408473107137;
MH419682;Lepidoptera;Celastrina argiolus;Celastrina argiolus;1;Celastrina argiolus;0.999037794018796;
MH419683;Lepidoptera;Papilio machaon;Papilio machaon;NA;Papilio machaon;0.998259418780604;
MH419684;Lepidoptera;Lasiommata paramegaera;Lasiommata paramegaera;0.999;Lasiommata paramegaera;0.995283446287531;
MH419685;Lepidoptera;Lasiommata paramegaera;Lasiommata paramegaera;0.999;Lasiommata paramegaera;0.995283446287531;
MH419687;Lepidoptera;Charaxes jasius;Charaxes jasius;NA;Charaxes jasius;0.990397821479112;
MH419688;Lepidoptera;Maniola cecilia;Pyronia cecilia;1;Pyronia cecilia;1;
MH419692;Lepidoptera;Thymelicus acteon;Thymelicus acteon;1;Thymelicus acteon;0.99999999752248;
MH419693;Lepidoptera;Callophrys rubi;NA;0.001;Callophrys rubi;0.998885705938309;
MH419695;Lepidoptera;Lasiommata megera;Lasiommata megera;1;Lasiommata megera;0.999379144055441;
MH419697;Lepidoptera;Aglais urticae;NA;0.115;Aglais urticae;0.987270759857581;
MH419698;Lepidoptera;Vanessa atalanta;Vanessa atalanta;1;Vanessa atalanta;0.999999999757762;
MH419699;Lepidoptera;Lycaena phlaeas;Lycaena phlaeas;1;Lycaena phlaeas;0.999999998390052;
MH419700;Lepidoptera;Callophrys rubi;NA;0.002;Callophrys rubi;0.998790227949348;
MH419702;Lepidoptera;Vanessa cardui;Vanessa cardui;1;Vanessa cardui;0.999999850530696;
MH419703;Lepidoptera;Maniola cecilia;Pyronia cecilia;1;Pyronia cecilia;1;
MH419704;Lepidoptera;Coenonympha pamphilus;Coenonympha pamphilus;1;Coenonympha pamphilus;0.999999999999744;
MH419705;Lepidoptera;Lasiommata megera;Lasiommata megera;1;Lasiommata megera;0.999379144055441;
MH419706;Lepidoptera;Aricia agestis;NA;0.253;Aricia agestis;0.996350017387504;
MH419707;Lepidoptera;Coenonympha pamphilus;Coenonympha pamphilus;1;Coenonympha pamphilus;0.999999999999744;
MH419708;Lepidoptera;Carcharodus alceae;Carcharodus alceae;0.001;Carcharodus alceae;0.999999994889151;
MH419710;Lepidoptera;Vanessa cardui;Vanessa cardui;1;Vanessa cardui;0.999999803450484;
MH419712;Lepidoptera;Callophrys rubi;NA;0.003;Callophrys rubi;0.997091529062725;
MH419714;Lepidoptera;Maniola cecilia;Pyronia cecilia;1;Pyronia cecilia;1;

MH419715;Lepidoptera;Hipparchia statilinus;Hipparchia statilinus;1;Hipparchia statilinus;0.999999999999915;
MH419716;Lepidoptera;Carcharodus alceae;Carcharodus alceae;1;Carcharodus alceae;0.999999998465938;
MH419718;Lepidoptera;Papilio machaon;Papilio machaon;0.003;Papilio machaon;0.999039382718766;
MH419720;Lepidoptera;Lasiommata paramegaera;Lasiommata paramegaera;0.999;Lasiommata paramegaera;0.979568320704936;
MH419723;Lepidoptera;Celastrina argiolus;Celastrina argiolus;1;Celastrina argiolus;0.999037794018796;
MH419724;Lepidoptera;Lasiommata paramegaera;Lasiommata paramegaera;0.999;Lasiommata paramegaera;0.995283446287531;
MH419725;Lepidoptera;Gegenes pumilio;Gegenes pumilio;1;Gegenes pumilio;0.999986726679266;
MH419726;Lepidoptera;Celastrina argiolus;Celastrina argiolus;1;Celastrina argiolus;0.999037794018796;
MH419730;Lepidoptera;Callophrys rubi;NA;0.001;Callophrys rubi;0.998885705938309;
MH419732;Lepidoptera;Hipparchia statilinus;Hipparchia statilinus;1;Hipparchia statilinus;0.999999999999915;
MH419733;Lepidoptera;Maniola cecilia;Pyronia cecilia;1;Pyronia cecilia;1;
MH419734;Lepidoptera;Lasiommata paramegaera;Lasiommata paramegaera;0.999;Lasiommata paramegaera;0.995283446287531;
MH419735;Lepidoptera;Coenonympha corinna;Coenonympha corinna;1;Coenonympha corinna;0.999999999619604;
MH419736;Lepidoptera;Vanessa cardui;Vanessa cardui;1;Vanessa cardui;0.999999850530696;
MH419737;Lepidoptera;Hipparchia aristaeus;Hipparchia aristaeus;1;Hipparchia aristaeus;0.999999998656648;
MH419738;Lepidoptera;Lampides boeticus;Lampides boeticus;1;Lampides boeticus;0.99999999876789;
MH419740;Lepidoptera;Gonepteryx cleopatra;Gonepteryx cleopatra;1;Gonepteryx cleopatra;0.999087339195645;
MH419741;Lepidoptera;Maniola tithonus;Pyronia tithonus;1;Pyronia tithonus;1;
MH419743;Lepidoptera;Thymelicus acteon;Thymelicus acteon;1;Thymelicus acteon;0.99999999752248;
MH419745;Lepidoptera;Gonepteryx cleopatra;Gonepteryx cleopatra;0.998;Gonepteryx cleopatra;0.999118636582266;
MH419749;Lepidoptera;Hipparchia aristaeus;Hipparchia aristaeus;1;Hipparchia aristaeus;0.999999993824275;
MH419750;Lepidoptera;Coenonympha corinna;Coenonympha corinna;1;Coenonympha corinna;0.999999999567109;
MH419752;Lepidoptera;Lycaena phlaeas;Lycaena phlaeas;1;Lycaena phlaeas;0.999999996363186;
MH419753;Lepidoptera;Lycaena phlaeas;Lycaena phlaeas;1;Lycaena phlaeas;0.999999996363186;
MH419754;Lepidoptera;Colias crocea;NA;0.059;NA;0.999999999999738;
MH419755;Lepidoptera;Vanessa atalanta;Vanessa atalanta;1;Vanessa atalanta;0.999999999757762;
MH419757;Lepidoptera;Vanessa cardui;Vanessa cardui;1;Vanessa cardui;0.999999459983318;
MH419758;Lepidoptera;Lampides boeticus;Lampides boeticus;1;Lampides boeticus;0.999999999545906;
MH419759;Lepidoptera;Gonepteryx cleopatra;Gonepteryx cleopatra;0.998;Gonepteryx cleopatra;0.999118636582266;
MH419760;Lepidoptera;Vanessa cardui;Vanessa cardui;1;Vanessa cardui;0.999999803450484;
MH419761;Lepidoptera;Gegenes pumilio;Gegenes pumilio;1;Gegenes pumilio;0.999986726679266;
MH419764;Lepidoptera;Lycaena phlaeas;Lycaena phlaeas;1;Lycaena phlaeas;0.999999996363186;
MH419768;Lepidoptera;Lampides boeticus;Lampides boeticus;1;Lampides boeticus;0.999999999545906;
MH419772;Lepidoptera;Lycaena phlaeas;Lycaena phlaeas;1;Lycaena phlaeas;0.999999996363186;
MH419775;Lepidoptera;Anthocharis cardamines;Anthocharis cardamines;1;Anthocharis cardamines;0.999999981710346;
MH419776;Lepidoptera;Celastrina argiolus;Celastrina argiolus;1;Celastrina argiolus;0.999527385150531;
MH419779;Lepidoptera;Gonepteryx cleopatra;Gonepteryx cleopatra;1;Gonepteryx cleopatra;0.999110851209713;
MH419780;Lepidoptera;Colias crocea;NA;0.059;NA;0.999999999999738;
MH419783;Lepidoptera;Carcharodus alceae;Carcharodus alceae;1;Carcharodus alceae;0.999999998465938;
MH419784;Lepidoptera;Maniola cecilia;Pyronia cecilia;NA;Pyronia cecilia;0.999999999999972;
MH419785;Lepidoptera;Celastrina argiolus;Celastrina argiolus;1;Celastrina argiolus;0.999527385150531;
MH419787;Lepidoptera;Vanessa cardui;Vanessa cardui;1;Vanessa cardui;0.999999459983318;
MH419789;Lepidoptera;Lasiommata megera;Lasiommata megera;1;Lasiommata megera;0.998531205767617;
MH419790;Lepidoptera;Pieris napi;NA;0.011;Pieris napi;0.801985394724814;
MH419793;Lepidoptera;Colias crocea;NA;0.059;NA;0.999999999999738;
MH419795;Lepidoptera;Lasiommata megera;Lasiommata megera;1;Lasiommata megera;0.999379144055441;
MH419796;Lepidoptera;Carcharodus alceae;Carcharodus alceae;1;Carcharodus alceae;0.999999990863188;
MH419797;Lepidoptera;Celastrina argiolus;Celastrina argiolus;1;Celastrina argiolus;0.999631157817044;
MH419798;Lepidoptera;Lampides boeticus;Lampides boeticus;1;Lampides boeticus;0.999999999545906;
MH419799;Lepidoptera;Vanessa cardui;Vanessa cardui;1;Vanessa cardui;0.999999561362909;
MH419800;Lepidoptera;Iphiclides podalirius;NA;0.302;Iphiclides podalirius;0.931082412606632;
MH419801;Lepidoptera;Vanessa atalanta;Vanessa atalanta;1;Vanessa atalanta;0.999999996750603;
MH419802;Lepidoptera;Gonepteryx cleopatra;Gonepteryx cleopatra;0.998;Gonepteryx cleopatra;0.999118636582266;
MH419804;Lepidoptera;Pieris napi;NA;NA;Pieris napi;0.726560864685254;

MH419805;Lepidoptera;Gonepteryx cleopatra;Gonepteryx cleopatra;0.998;Gonepteryx cleopatra;0.999118636582266;
MH419806;Lepidoptera;Melitaea nevadensis;NA;0.001;Melitaea celadussa;0.727568537860761;
MH419807;Lepidoptera;Vanessa atalanta;Vanessa atalanta;1;Vanessa atalanta;0.999999999757762;
MH419808;Lepidoptera;Colias crocea;NA;0.059;NA;0.999999999999738;
MH419809;Lepidoptera;Vanessa atalanta;Vanessa atalanta;1;Vanessa atalanta;0.999999999757762;
MH419810;Lepidoptera;Vanessa cardui;Vanessa cardui;1;Vanessa cardui;0.999999459983318;
MH419811;Lepidoptera;Colias crocea;NA;NA;NA;0.999999999999675;
MH419812;Lepidoptera;Callophrys rubi;NA;0.001;Callophrys rubi;0.998885705938309;
MH419814;Lepidoptera;Vanessa cardui;Vanessa cardui;1;Vanessa cardui;0.999999459983318;
MH419816;Lepidoptera;Lasiommata paramegaera;Lasiommata paramegaera;0.999;Lasiommata paramegaera;0.995283446287531;
MH419817;Lepidoptera;Lycaena phlaeas;Lycaena phlaeas;1;Lycaena phlaeas;0.999999996363186;
MH419819;Lepidoptera;Maniola cecilia;Pyronia cecilia;NA;Pyronia cecilia;1;
MH419820;Lepidoptera;Colias crocea;NA;0.065;NA;0.999999999999745;
MH419821;Lepidoptera;Iphiclides podalirius;NA;0.302;Iphiclides podalirius;0.931082412606632;
MH419822;Lepidoptera;Anthocharis cardamines;Anthocharis cardamines;1;Anthocharis cardamines;0.99999997953563;
MH419824;Lepidoptera;Lampides boeticus;Lampides boeticus;1;Lampides boeticus;0.999999999375433;
MH419826;Lepidoptera;Lycaena phlaeas;Lycaena phlaeas;1;Lycaena phlaeas;0.999999996623245;
MH419827;Lepidoptera;Coenonympha corinna;Coenonympha corinna;1;Coenonympha corinna;0.999999999567109;
MH419828;Lepidoptera;Pieris rapae;NA;NA;Pieris rapae;0.999992588119495;
MH419829;Lepidoptera;Melitaea didyma;Melitaea didyma;1;Melitaea didyma;0.999947466878989;
MH419831;Lepidoptera;Callophrys rubi;NA;0.019;Callophrys rubi;0.998201459637982;
MH419832;Lepidoptera;Vanessa atalanta;Vanessa atalanta;1;Vanessa atalanta;0.999999999383476;
MH419833;Lepidoptera;Gonepteryx cleopatra;Gonepteryx cleopatra;0.998;Gonepteryx cleopatra;0.999118636582266;
MH419834;Lepidoptera;Lampides boeticus;Lampides boeticus;1;Lampides boeticus;0.999999999688214;
MH419835;Lepidoptera;Gegenes pumilio;Gegenes pumilio;1;Gegenes pumilio;0.999986726679266;
MH419836;Lepidoptera;Lycaena phlaeas;Lycaena phlaeas;1;Lycaena phlaeas;0.999999996363186;
MH419837;Lepidoptera;Callophrys rubi;NA;0.019;Callophrys rubi;0.998201459637982;
MH419839;Lepidoptera;Pieris rapae;NA;NA;Pieris rapae;0.999984333973504;
MH419840;Lepidoptera;Lasiommata megera;Lasiommata megera;1;Lasiommata megera;0.999379144055441;
MH419842;Lepidoptera;Lampides boeticus;Lampides boeticus;1;Lampides boeticus;0.999999999545906;
MH419843;Lepidoptera;Gonepteryx cleopatra;Gonepteryx cleopatra;0.998;Gonepteryx cleopatra;0.999118636582266;
MH419845;Lepidoptera;Colias crocea;NA;0.059;NA;0.999999999999738;
MH419846;Lepidoptera;Melitaea didyma;Melitaea didyma;1;Melitaea didyma;0.99994753434867;
MH419847;Lepidoptera;Carcharodus alceae;Carcharodus alceae;0.002;Carcharodus alceae;0.999999991169204;
MH419849;Lepidoptera;Vanessa cardui;Vanessa cardui;1;Vanessa cardui;0.999999841763282;
MH419850;Lepidoptera;Celastrina argiolus;Celastrina argiolus;1;Celastrina argiolus;0.999527385150531;
MH419851;Lepidoptera;Lasiommata megera;Lasiommata megera;1;Lasiommata megera;0.999379144055441;
MH419853;Lepidoptera;Pieris rapae;NA;NA;Pieris rapae;0.999839508485527;
MH419855;Lepidoptera;Gonepteryx cleopatra;Gonepteryx cleopatra;0.998;Gonepteryx cleopatra;0.999118636582266;
MH419856;Lepidoptera;Carcharodus alceae;Carcharodus alceae;1;Carcharodus alceae;0.999999998465938;
MH419857;Lepidoptera;Plebejus idas;NA;0.002;NA;0.999434923024389;
MH419858;Lepidoptera;Hipparchia aristaeus;Hipparchia aristaeus;1;Hipparchia aristaeus;0.999999993824275;
MH419859;Lepidoptera;Vanessa atalanta;Vanessa atalanta;1;Vanessa atalanta;0.999999999757762;
MH419860;Lepidoptera;Papilio machaon;Papilio machaon;NA;Papilio machaon;0.998335940984695;
MH419861;Lepidoptera;Celastrina argiolus;Celastrina argiolus;1;Celastrina argiolus;0.999527385150531;
MH419862;Lepidoptera;Coenonympha pamphilus;Coenonympha pamphilus;1;Coenonympha pamphilus;0.999999999999659;
MH419863;Lepidoptera;Satyrium ilicis;Satyrium ilicis;1;Satyrium ilicis;0.999999987901305;
MH419865;Lepidoptera;Pieris rapae;NA;NA;Pieris rapae;0.999992588119495;
MH419866;Lepidoptera;Lasiommata paramegaera;Lasiommata paramegaera;0.999;Lasiommata paramegaera;0.995283446287531;
MH419867;Lepidoptera;Lycaena phlaeas;Lycaena phlaeas;1;Lycaena phlaeas;0.999999998167567;
MH419870;Lepidoptera;Colias crocea;NA;0.059;NA;0.999999999999738;
MH419871;Lepidoptera;Pieris rapae;Pieris rapae;0.002;Pieris rapae;0.999981648403229;
MH419872;Lepidoptera;Coenonympha pamphilus;Coenonympha pamphilus;1;Coenonympha pamphilus;0.999999999999744;
MH419873;Lepidoptera;Pieris rapae;NA;0.072;Pieris rapae;0.999944530512174;
MH419874;Lepidoptera;Lycaena phlaeas;Lycaena phlaeas;1;Lycaena phlaeas;0.999999998508258;
MH419875;Lepidoptera;Celastrina argiolus;Celastrina argiolus;1;Celastrina argiolus;0.999527385150531;
MH419876;Lepidoptera;Vanessa cardui;Vanessa cardui;1;Vanessa cardui;0.999999459983318;
MH419879;Lepidoptera;Lycaena phlaeas;Lycaena phlaeas;1;Lycaena phlaeas;0.999999998167567;
MH419880;Lepidoptera;Carcharodus alceae;Carcharodus alceae;0.001;Carcharodus alceae;0.999999994870137;
MH419881;Lepidoptera;Thymelicus acteon;Thymelicus acteon;1;Thymelicus acteon;0.99999999752248;

MH419882;Lepidoptera;Gonepteryx cleopatra;Gonepteryx cleopatra;1;Gonepteryx cleopatra;0.999072420135293;
MH419884;Lepidoptera;Carcharodus alceae;Carcharodus alceae;0.999;Carcharodus alceae;0.999999996339511;
MH419885;Lepidoptera;Favonius quercus;NA;NA;Favonius quercus;0.73046069582187;
MH419886;Lepidoptera;Thymelicus acteon;Thymelicus acteon;1;Thymelicus acteon;0.99999999752248;
MH419889;Lepidoptera;Melitaea nevadensis;NA;0.001;NA;0.999999999896095;
MH419891;Lepidoptera;Maniola jurtina;NA;0.062;Maniola jurtina;0.865337727363939;
MH419892;Lepidoptera;Gonepteryx rhamni;Gonepteryx rhamni;0.012;Gonepteryx rhamni;0.999526173034719;
MH419893;Lepidoptera;Gonepteryx cleopatra;Gonepteryx cleopatra;0.998;Gonepteryx cleopatra;0.999118636582266;
MH419894;Lepidoptera;Melitaea didyma;Melitaea didyma;1;Melitaea didyma;0.999945824175549;
MH419895;Lepidoptera;Iphiclides podalirius;NA;0.302;Iphiclides podalirius;0.931082412606632;
MH419896;Lepidoptera;Celastrina argiolus;Celastrina argiolus;1;Celastrina argiolus;0.999037794018796;
MH419898;Lepidoptera;Maniola jurtina;NA;0.038;NA;0.999999999999524;
MH419899;Lepidoptera;Coenonympha pamphilus;Coenonympha pamphilus;1;Coenonympha pamphilus;0.999999999999488;
MH419901;Lepidoptera;Polyommatus icarus;Polyommatus icarus;0.001;Polyommatus icarus;0.999847102144222;
MH419901;Lepidoptera;Polyommatus icarus;Polyommatus icarus;0.001;Polyommatus icarus;0.999847102144222;
MH419902;Lepidoptera;Satyrium ilicis;Satyrium ilicis;1;Satyrium ilicis;0.999999987901305;
MH419903;Lepidoptera;Pieris napi;NA;0.011;Pieris napi;0.801985394724814;
MH419904;Lepidoptera;Lampides boeticus;Lampides boeticus;1;Lampides boeticus;0.999999999545906;
MH419906;Lepidoptera;Colias crocea;NA;0.059;NA;0.999999999999738;
MH419908;Lepidoptera;Melitaea nevadensis;NA;0.007;Melitaea celadussa;0.857896833450963;
MH419911;Lepidoptera;Coenonympha pamphilus;Coenonympha pamphilus;1;Coenonympha pamphilus;0.999999999999744;
MH419913;Lepidoptera;Thymelicus acteon;Thymelicus acteon;1;Thymelicus acteon;0.999999995239193;
MH419914;Lepidoptera;Lampides boeticus;Lampides boeticus;1;Lampides boeticus;0.999999999545906;
MH419915;Lepidoptera;Lycaena phlaeas;Lycaena phlaeas;1;Lycaena phlaeas;0.999999996363186;
MH419916;Lepidoptera;Maniola cecilia;Pyronia cecilia;1;Pyronia cecilia;0.999999999999972;
MH419917;Lepidoptera;Colias crocea;NA;0.059;NA;0.999999999999738;
MH419919;Lepidoptera;Maniola cecilia;Pyronia cecilia;NA;Pyronia cecilia;1;
MH419920;Lepidoptera;Lasiommata megera;Lasiommata megera;1;Lasiommata megera;0.999806890129977;
MH419922;Lepidoptera;Polyommatus icarus;Polyommatus icarus;0.001;Polyommatus icarus;0.9997596187835;
MH419922;Lepidoptera;Polyommatus icarus;Polyommatus icarus;0.001;Polyommatus icarus;0.9997596187835;
MH419923;Lepidoptera;Pieris rapae;NA;0.175;Pieris rapae;0.99996637543374;
MH419924;Lepidoptera;Gegenes pumilio;Gegenes pumilio;0.999;Gegenes pumilio;0.999991412663101;
MH419925;Lepidoptera;Coenonympha corinna;Coenonympha corinna;1;Coenonympha corinna;0.999999999567109;
MH419926;Lepidoptera;Pieris rapae;NA;NA;Pieris rapae;0.999992588119495;
MH419927;Lepidoptera;Lasiommata paramegaera;Lasiommata paramegaera;0.999;Lasiommata paramegaera;0.995283446287531;
MH419928;Lepidoptera;Lycaena phlaeas;Lycaena phlaeas;1;Lycaena phlaeas;0.999999997688235;
MH419929;Lepidoptera;Hipparchia statilinus;Hipparchia statilinus;1;Hipparchia statilinus;0.999999999999858;
MH419931;Lepidoptera;Thymelicus acteon;Thymelicus acteon;1;Thymelicus acteon;0.99999999752248;
MH419933;Lepidoptera;Vanessa cardui;Vanessa cardui;1;Vanessa cardui;0.999999565055428;
MH419934;Lepidoptera;Coenonympha corinna;Coenonympha corinna;1;Coenonympha corinna;0.999999999481616;
MH419936;Lepidoptera;Vanessa atalanta;Vanessa atalanta;1;Vanessa atalanta;0.999999999297358;
MH419937;Lepidoptera;Lasiommata megera;Lasiommata megera;1;Lasiommata megera;0.999379144055441;
MH419939;Lepidoptera;Melitaea nevadensis;Melitaea celadussa;1;Melitaea celadussa;0.99883216908598;
MH419941;Lepidoptera;Celastrina argiolus;Celastrina argiolus;1;Celastrina argiolus;0.999037794018796;
MH419942;Lepidoptera;Pieris rapae;NA;0.175;Pieris rapae;0.99996637543374;
MH419944;Lepidoptera;Anthocharis cardamines;Anthocharis cardamines;1;Anthocharis cardamines;0.999999917840544;
MH419945;Lepidoptera;Coenonympha corinna;Coenonympha corinna;1;Coenonympha corinna;0.999999556334798;
MH419946;Lepidoptera;Maniola jurtina;NA;0.001;Maniola jurtina;0.939220105860875;
MH419949;Lepidoptera;Aricia agestis;Aricia agestis;1;Aricia agestis;0.979315827401608;
MH419950;Lepidoptera;Lampides boeticus;Lampides boeticus;1;Lampides boeticus;0.999999999690317;
MH419953;Lepidoptera;Pieris rapae;NA;0.175;Pieris rapae;0.99996637543374;
MH419954;Lepidoptera;Iphiclides podalirius;NA;0.313;Iphiclides podalirius;0.889198863100098;
MH419956;Lepidoptera;Aricia agestis;NA;0.253;Aricia agestis;0.996350017387504;
MH419958;Lepidoptera;Pieris rapae;Pieris rapae;0.01;Pieris rapae;0.999983538512174;
MH419962;Lepidoptera;Pieris rapae;NA;NA;Pieris rapae;0.999992588119495;
MH419964;Lepidoptera;Maniola cecilia;Pyronia cecilia;NA;Pyronia cecilia;1;
MH419965;Lepidoptera;Lampides boeticus;Lampides boeticus;1;Lampides boeticus;0.999999999545906;
MH419966;Lepidoptera;Lycaena phlaeas;Lycaena phlaeas;1;Lycaena phlaeas;0.999999992669558;
MH419971;Lepidoptera;Maniola cecilia;Pyronia cecilia;1;Pyronia cecilia;1;

MH419973;Lepidoptera;Plebejus argyrognomon;NA;0.001;NA;0.994620093546684;
MH419974;Lepidoptera;Lasiommata paramegaera;Lasiommata paramegaera;0.001;Lasiommata paramegaera;0.974943507761532;
MH419976;Lepidoptera;Iphiclides podalirius;NA;0.302;Iphiclides podalirius;0.931082412606632;
MH419977;Lepidoptera;Gegenes pumilio;Gegenes pumilio;1;Gegenes pumilio;0.999986726679266;
MH419978;Lepidoptera;Coenonympha pamphilus;Coenonympha pamphilus;1;Coenonympha pamphilus;0.999999999999744;
MH419979;Lepidoptera;Melitaea cinxia;Melitaea cinxia;1;Melitaea cinxia;0.999999999999631;
MH419980;Lepidoptera;Maniola jurtina;NA;0.018;Maniola jurtina;0.907655467713929;
MH419982;Lepidoptera;Lasiommata megera;Lasiommata megera;1;Lasiommata megera;0.999379144055441;
MH419983;Lepidoptera;Coenonympha pamphilus;Coenonympha pamphilus;1;Coenonympha pamphilus;0.999999999999744;
MH419984;Lepidoptera;Lycaena phlaeas;Lycaena phlaeas;1;Lycaena phlaeas;0.999999996363186;
MH419987;Lepidoptera;Callophrys rubi;NA;0.019;Callophrys rubi;0.998201459637982;
MH419989;Lepidoptera;Vanessa atalanta;Vanessa atalanta;1;Vanessa atalanta;0.999999999757762;
MH419991;Lepidoptera;Lasiommata megera;Lasiommata megera;1;Lasiommata megera;0.999379144055441;
MH419992;Lepidoptera;Gonepteryx cleopatra;Gonepteryx cleopatra;0.998;Gonepteryx cleopatra;0.999118636582266;
MH419993;Lepidoptera;Lasiommata megera;Lasiommata megera;1;Lasiommata megera;0.998777126513664;
MH419995;Lepidoptera;Vanessa atalanta;Vanessa atalanta;1;Vanessa atalanta;0.999999999757762;
MH419996;Lepidoptera;Lasiommata megera;Lasiommata megera;1;Lasiommata megera;0.999379144055441;
MH419997;Lepidoptera;Hipparchia statilinus;Hipparchia statilinus;1;Hipparchia statilinus;0.999999999999943;
MH419998;Lepidoptera;Thymelicus acteon;Thymelicus acteon;1;Thymelicus acteon;0.999999995239193;
MH420000;Lepidoptera;Glaucopsyche alexis;Glaucopsyche alexis;0.001;Glaucopsyche alexis;0.94042380088325;
MH420001;Lepidoptera;Coenonympha lyllus;Coenonympha pamphilus;1;Coenonympha pamphilus;0.999999999983061;
MH420002;Lepidoptera;Melitaea nevadensis;NA;0.007;Melitaea celadussa;0.856486104428833;
MH420003;Lepidoptera;Lampides boeticus;Lampides boeticus;1;Lampides boeticus;0.999999998137667;
MH420004;Lepidoptera;Argynnis paphia;Argynnis paphia;1;Argynnis paphia;0.999999991389927;
MH420007;Lepidoptera;Gonepteryx cleopatra;Gonepteryx cleopatra;0.998;Gonepteryx cleopatra;0.999118636582266;
MH420008;Lepidoptera;Maniola jurtina;NA;0.001;Maniola jurtina;0.939220105860875;
MH420009;Lepidoptera;Pieris rapae;NA;NA;Pieris rapae;0.999992588119495;
MH420010;Lepidoptera;Maniola tithonus;Pyronia tithonus;1;Pyronia tithonus;1;
MH420011;Lepidoptera;Lasiommata megera;Lasiommata megera;1;Lasiommata megera;0.999379144055441;
MH420012;Lepidoptera;Hipparchia aristaeus;Hipparchia aristaeus;1;Hipparchia aristaeus;0.999999996732214;
MH420014;Lepidoptera;Carcharodus alceae;Carcharodus alceae;1;Carcharodus alceae;0.999999998465938;
MH420015;Lepidoptera;Lycaena phlaeas;Lycaena phlaeas;1;Lycaena phlaeas;0.999999996363186;
MH420016;Lepidoptera;Hipparchia aristaeus;Hipparchia aristaeus;1;Hipparchia aristaeus;0.999999993824275;
MH420017;Lepidoptera;Coenonympha pamphilus;Coenonympha pamphilus;1;Coenonympha pamphilus;0.999999999999744;
MH420019;Lepidoptera;Vanessa atalanta;Vanessa atalanta;1;Vanessa atalanta;0.999999999757762;
MH420020;Lepidoptera;Gonepteryx rhamni;Gonepteryx rhamni;0.018;Gonepteryx rhamni;0.997635142289456;
MH420021;Lepidoptera;Thymelicus acteon;Thymelicus acteon;1;Thymelicus acteon;0.99999999752248;
MH420022;Lepidoptera;Anthocharis cardamines;Anthocharis cardamines;1;Anthocharis cardamines;0.99999997953563;
MH420023;Lepidoptera;Lampides boeticus;Lampides boeticus;1;Lampides boeticus;0.999999999545906;
MH420024;Lepidoptera;Lampides boeticus;Lampides boeticus;1;Lampides boeticus;0.99999997693169;
MH420026;Lepidoptera;Gonepteryx cleopatra;Gonepteryx cleopatra;0.998;Gonepteryx cleopatra;0.999118636582266;
MH420029;Lepidoptera;Charaxes jasius;Charaxes jasius;NA;Charaxes jasius;0.990397821479112;
MH420030;Lepidoptera;Argynnis paphia;Argynnis paphia;1;Argynnis paphia;0.999999991389927;
MH420032;Lepidoptera;Argynnis paphia;Argynnis paphia;1;Argynnis paphia;0.999999991389927;
MH420033;Lepidoptera;Lasiommata megera;Lasiommata megera;1;Lasiommata megera;0.999379144055441;
MH420034;Lepidoptera;Melitaea didyma;Melitaea didyma;1;Melitaea didyma;0.999887337851026;
MH420035;Lepidoptera;Celastrina argiolus;Celastrina argiolus;1;Celastrina argiolus;0.999037794018796;
MH420036;Lepidoptera;Gegenes pumilio;Gegenes pumilio;1;Gegenes pumilio;0.999986726679266;
MH420037;Lepidoptera;Vanessa cardui;Vanessa cardui;1;Vanessa cardui;0.999999459983318;
MH420041;Lepidoptera;Thymelicus acteon;Thymelicus acteon;1;Thymelicus acteon;0.999999995239193;
MH420043;Lepidoptera;Carcharodus alceae;Carcharodus alceae;1;Carcharodus alceae;0.999999998465938;
MH420044;Lepidoptera;Melitaea cinxia;Melitaea cinxia;1;Melitaea cinxia;0.999999999999829;
MH420048;Lepidoptera;Pyrgus armoricanus;Pyrgus armoricanus;1;Pyrgus armoricanus;0.999997353848817;
MH420049;Lepidoptera;Lasiommata paramegaera;Lasiommata paramegaera;0.999;Lasiommata paramegaera;0.995283446287531;
MH420050;Lepidoptera;Argynnis paphia;Argynnis paphia;1;Argynnis paphia;0.999999991389927;
MH420051;Lepidoptera;Lasiommata megera;Lasiommata megera;0.999;Lasiommata megera;0.999423486493351;
MH420052;Lepidoptera;Papilio machaon;Papilio machaon;0.003;Papilio machaon;0.999482752749035;

MH420053;Lepidoptera;Pyrgus armoricanus;Pyrgus armoricanus;1;Pyrgus armoricanus;0.999997353848817;
MH420054;Lepidoptera;Gonepteryx cleopatra;Gonepteryx cleopatra;0.998;Gonepteryx cleopatra;0.999118636582266;
MH420055;Lepidoptera;Lasiommata megera;Lasiommata megera;1;Lasiommata megera;0.999379144055441;
MH420056;Lepidoptera;Gegenes pumilio;Gegenes pumilio;1;Gegenes pumilio;0.999993089597811;
MH420057;Lepidoptera;Maniola tithonus;Pyronia tithonus;1;Pyronia tithonus;1;
MH420058;Lepidoptera;Pyrgus armoricanus;Pyrgus armoricanus;1;Pyrgus armoricanus;0.999997595351167;
MH420060;Lepidoptera;Colias crocea;NA;0.059;NA;0.999999999999738;
MH420061;Lepidoptera;Lasiommata megera;Lasiommata megera;0.998;Lasiommata megera;0.998568158791128;
MH420062;Lepidoptera;Pieris rapae;NA;NA;Pieris rapae;0.999992588119495;
MH420063;Lepidoptera;Papilio machaon;Papilio machaon;NA;Papilio machaon;0.998335940984695;
MH420065;Lepidoptera;Carcharodus alceae;Carcharodus alceae;1;Carcharodus alceae;0.999999990863188;
MH420066;Lepidoptera;Papilio machaon;Papilio machaon;0.003;Papilio machaon;0.999482752749035;
MH420067;Lepidoptera;Celastrina argiolus;Celastrina argiolus;1;Celastrina argiolus;0.999527385150531;
MH420069;Lepidoptera;Lycaena phlaeas;Lycaena phlaeas;1;Lycaena phlaeas;0.999999998424926;
MH420071;Lepidoptera;Lasiommata paramegaera;Lasiommata paramegaera;0.999;Lasiommata paramegaera;0.995283446287531;
MH420072;Lepidoptera;Lasiommata paramegaera;Lasiommata paramegaera;0.999;Lasiommata paramegaera;0.995283446287531;
MH420073;Lepidoptera;Lycaena phlaeas;Lycaena phlaeas;1;Lycaena phlaeas;0.999999998167567;
MH420074;Lepidoptera;Celastrina argiolus;Celastrina argiolus;1;Celastrina argiolus;0.999527385150531;
MH420075;Lepidoptera;Hipparchia statilinus;Hipparchia statilinus;1;Hipparchia statilinus;0.999999999999801;
MH420076;Lepidoptera;Lasiommata megera;Lasiommata megera;1;Lasiommata megera;0.999379144055441;
MH420077;Lepidoptera;Carcharodus alceae;Carcharodus alceae;0.999;Carcharodus alceae;0.999999996339511;
MH420078;Lepidoptera;Hipparchia neomiris;Hipparchia neomiris;1;Hipparchia neomiris;0.999999999999744;
MH420079;Lepidoptera;Anthocharis cardamines;Anthocharis cardamines;1;Anthocharis cardamines;0.99999997953563;
MH420080;Lepidoptera;Coenonympha corinna;Coenonympha corinna;1;Coenonympha corinna;0.999999999270614;
MH420083;Lepidoptera;Celastrina argiolus;Celastrina argiolus;1;Celastrina argiolus;0.999527385150531;
MH420084;Lepidoptera;Vanessa cardui;Vanessa cardui;1;Vanessa cardui;0.999999288711561;
MH420085;Lepidoptera;Lampides boeticus;Lampides boeticus;1;Lampides boeticus;0.999999999545906;
MH420087;Lepidoptera;Celastrina argiolus;Celastrina argiolus;1;Celastrina argiolus;0.999527385150531;
MH420091;Lepidoptera;Anthocharis cardamines;Anthocharis cardamines;1;Anthocharis cardamines;0.999999988534341;
MH420092;Lepidoptera;Anthocharis cardamines;Anthocharis cardamines;1;Anthocharis cardamines;0.999999988534341;
MH420093;Lepidoptera;Melitaea didyma;Melitaea didyma;1;Melitaea didyma;0.99994753434867;
MH420095;Lepidoptera;Pieris rapae;NA;NA;Pieris rapae;0.999992588119495;
MH420096;Lepidoptera;Papilio machaon;Papilio machaon;NA;Papilio machaon;0.99889956649962;
MH420097;Lepidoptera;Vanessa cardui;Vanessa cardui;1;Vanessa cardui;0.999999565055428;
MH420099;Lepidoptera;Thymelicus acteon;Thymelicus acteon;1;Thymelicus acteon;0.99999999752248;
MH420102;Lepidoptera;Glaucopsyche alexis;NA;NA;Glaucopsyche alexis;0.89375645259355;
MH420104;Lepidoptera;Thymelicus acteon;Thymelicus acteon;1;Thymelicus acteon;0.99999999752248;
MH420105;Lepidoptera;Hipparchia aristaeus;Hipparchia aristaeus;1;Hipparchia aristaeus;0.999999993824275;
MH420107;Lepidoptera;Colias crocea;NA;0.059;NA;0.999999999999738;
MH420111;Lepidoptera;Lampides boeticus;Lampides boeticus;1;Lampides boeticus;0.999999999545906;
MH420113;Lepidoptera;Lampides boeticus;Lampides boeticus;1;Lampides boeticus;0.999999999545906;
MH420114;Lepidoptera;Pieris rapae;Pieris rapae;0.02;Pieris rapae;0.999990692934826;
MH420116;Lepidoptera;Vanessa atalanta;Vanessa atalanta;1;Vanessa atalanta;0.999999999757762;
MH420117;Lepidoptera;Charaxes jasius;Charaxes jasius;NA;Charaxes jasius;0.990397821479112;
MH420121;Lepidoptera;Pieris rapae;NA;NA;Pieris rapae;0.999957794752662;
MH420123;Lepidoptera;Vanessa cardui;Vanessa cardui;1;Vanessa cardui;0.999999459983318;
MH420124;Lepidoptera;Colias crocea;NA;0.059;NA;0.999999999999738;
MH420125;Lepidoptera;Aricia agestis;Aricia agestis;0.001;Aricia agestis;0.972120797608056;
MH420126;Lepidoptera;Colias crocea;NA;0.059;NA;0.999999999999738;
MH420130;Lepidoptera;Colias crocea;NA;0.059;NA;0.999999999999738;
MH420131;Lepidoptera;Carcharodus alceae;Carcharodus alceae;1;Carcharodus alceae;0.999999927744201;
MH420133;Lepidoptera;Vanessa cardui;Vanessa cardui;1;Vanessa cardui;0.999999459983318;
MH420135;Lepidoptera;Pieris rapae;NA;NA;Pieris rapae;0.999970925323953;
MH420136;Lepidoptera;Gonepteryx rhamni;Gonepteryx rhamni;0.008;Gonepteryx rhamni;0.999527533936108;
MH420138;Lepidoptera;Maniola cecilia;Pyronia cecilia;1;Pyronia cecilia;1;
MH420139;Lepidoptera;Hipparchia aristaeus;Hipparchia aristaeus;1;Hipparchia aristaeus;0.999999993824275;

MH420140;Lepidoptera;Maniola cecilia;Pyronia cecilia;NA;Pyronia cecilia;0.999999999999972;
MH420141;Lepidoptera;Maniola cecilia;Pyronia cecilia;NA;Pyronia cecilia;1;
MH420142;Lepidoptera;Colias crocea;NA;NA;Colias crocea;0.742267258907009;
MH420143;Lepidoptera;Celastrina argiolus;Celastrina argiolus;0.999;Celastrina argiolus;0.997181880679251;
MH420144;Lepidoptera;Callophrys rubi;NA;0.001;Callophrys rubi;0.998885705938309;
MH420145;Lepidoptera;Maniola cecilia;Pyronia cecilia;1;Pyronia cecilia;1;
MH420146;Lepidoptera;Vanessa atalanta;Vanessa atalanta;1;Vanessa atalanta;0.999999999757762;
MH420147;Lepidoptera;Iphiclides podalirius;NA;0.302;Iphiclides podalirius;0.931082412606632;
MH420148;Lepidoptera;Polyommatus icarus;Polyommatus icarus;0.001;Polyommatus icarus;0.999851522482238;
MH420148;Lepidoptera;Polyommatus icarus;Polyommatus icarus;0.001;Polyommatus icarus;0.999851522482238;
MH420149;Lepidoptera;Glaucopsyche alexis;Glaucopsyche alexis;0.001;Glaucopsyche alexis;0.94042380088325;
MH420151;Lepidoptera;Papilio machaon;Papilio machaon;NA;Papilio machaon;0.99889956649962;
MH420152;Lepidoptera;Coenonympha pamphilus;Coenonympha pamphilus;1;Coenonympha pamphilus;0.999999999999744;
MH420153;Lepidoptera;Vanessa atalanta;Vanessa atalanta;1;Vanessa atalanta;0.999999999757762;
MH420156;Lepidoptera;Coenonympha pamphilus;Coenonympha pamphilus;1;Coenonympha pamphilus;0.999999999999744;
MH420158;Lepidoptera;Lycaena phlaeas;Lycaena phlaeas;1;Lycaena phlaeas;0.999999992669558;
MH420159;Lepidoptera;Papilio machaon;Papilio machaon;0.003;Papilio machaon;0.999039382718766;
MH420161;Lepidoptera;Aricia agestis;NA;NA;Aricia agestis;0.996930365249266;
MH420162;Lepidoptera;Lasiommata megera;Lasiommata megera;1;Lasiommata megera;0.999379144055441;
MH420163;Lepidoptera;Lasiommata megera;Lasiommata megera;1;Lasiommata megera;0.999379144055441;
MH420164;Lepidoptera;Carcharodus alceae;Carcharodus alceae;0.001;Carcharodus alceae;0.999999994870137;
MH420165;Lepidoptera;Vanessa atalanta;Vanessa atalanta;1;Vanessa atalanta;0.999999999757762;
MH420166;Lepidoptera;Carcharodus alceae;Carcharodus alceae;0.001;Carcharodus alceae;0.999999994870137;
MH420167;Lepidoptera;Gegenes pumilio;Gegenes pumilio;1;Gegenes pumilio;0.999986726679266;
MH420168;Lepidoptera;Lasiommata megera;Lasiommata megera;1;Lasiommata megera;0.999379144055441;
MH420169;Lepidoptera;Pieris rapae;NA;0.206;Pieris rapae;0.999929773639513;
MH420170;Lepidoptera;Celastrina argiolus;Celastrina argiolus;1;Celastrina argiolus;0.999527385150531;
MH420171;Lepidoptera;Coenonympha pamphilus;Coenonympha pamphilus;1;Coenonympha pamphilus;0.999999999999744;
MH420172;Lepidoptera;Vanessa atalanta;Vanessa atalanta;1;Vanessa atalanta;0.999999999757762;
MH420173;Lepidoptera;Coenonympha pamphilus;Coenonympha pamphilus;1;Coenonympha pamphilus;0.999999999999403;
MH420174;Lepidoptera;Pieris rapae;NA;NA;Pieris rapae;0.999992588119495;
MH420175;Lepidoptera;Coenonympha corinna;Coenonympha corinna;1;Coenonympha corinna;0.999999556334798;
MH420176;Lepidoptera;Lampides boeticus;Lampides boeticus;1;Lampides boeticus;0.999999999545906;
MH420180;Lepidoptera;Carcharodus alceae;Carcharodus alceae;0.999;Carcharodus alceae;0.999999996339511;
MH420181;Lepidoptera;Anthocharis cardamines;Anthocharis cardamines;1;Anthocharis cardamines;0.99999997953563;
MH420182;Lepidoptera;Vanessa atalanta;Vanessa atalanta;1;Vanessa atalanta;0.999999999757762;
MH420183;Lepidoptera;Argynnis paphia;Argynnis paphia;1;Argynnis paphia;0.999999991389927;
MH420185;Lepidoptera;Colias crocea;NA;NA;NA;0.999999999998548;
MH420189;Lepidoptera;Lasiommata megera;Lasiommata megera;1;Lasiommata megera;0.999379144055441;
MH420190;Lepidoptera;Lasiommata megera;Lasiommata megera;1;Lasiommata megera;0.999379144055441;
MH420191;Lepidoptera;Maniola cecilia;Pyronia cecilia;1;Pyronia cecilia;1;
MH420193;Lepidoptera;Vanessa atalanta;Vanessa atalanta;1;Vanessa atalanta;0.999999999757762;
MH420194;Lepidoptera;Lasiommata paramegaera;Lasiommata paramegaera;0.999;Lasiommata paramegaera;0.995283446287531;
MH420195;Lepidoptera;Vanessa cardui;Vanessa cardui;1;Vanessa cardui;0.999999803450484;
MH420196;Lepidoptera;Vanessa cardui;Vanessa cardui;1;Vanessa cardui;0.999999459983318;
MH420199;Lepidoptera;Gonepteryx cleopatra;Gonepteryx cleopatra;0.998;Gonepteryx cleopatra;0.999118636582266;
MH420200;Lepidoptera;Maniola cecilia;Pyronia cecilia;1;Pyronia cecilia;1;
MH420201;Lepidoptera;Maniola cecilia;Pyronia cecilia;1;Pyronia cecilia;1;
MH420203;Lepidoptera;Gonepteryx rhamni;NA;0.474;Gonepteryx rhamni;0.982076396127858;
MH420204;Lepidoptera;Lasiommata megera;Lasiommata megera;1;Lasiommata megera;0.999313301949205;
MH420205;Lepidoptera;Pieris rapae;NA;NA;Pieris rapae;0.999993359183004;
MH420207;Lepidoptera;Gonepteryx rhamni;NA;0.474;Gonepteryx rhamni;0.982076396127858;
MH420209;Lepidoptera;Celastrina argiolus;Celastrina argiolus;1;Celastrina argiolus;0.999037794018796;
MH420210;Lepidoptera;Pieris rapae;NA;NA;Pieris rapae;0.999992588119495;
MH420211;Lepidoptera;Coenonympha pamphilus;Coenonympha pamphilus;1;Coenonympha pamphilus;0.999999999999744;
MH420212;Lepidoptera;Maniola cecilia;Pyronia cecilia;NA;Pyronia cecilia;0.999999999999972;
MH420214;Lepidoptera;Lasiommata paramegaera;Lasiommata paramegaera;0.999;Lasiommata paramegaera;0.995283446287531;
MH420217;Lepidoptera;Vanessa atalanta;Vanessa atalanta;1;Vanessa atalanta;0.999999999757762;
MH420219;Lepidoptera;Colias crocea;NA;0.059;NA;0.999999999999738;

MH420220;Lepidoptera;Colias crocea;NA;0.059;NA;0.999999999999738;
MH420221;Lepidoptera;Coenonympha pamphilus;Coenonympha pamphilus;1;Coenonympha
pamphilus;0.999999999999744;
MH420223;Lepidoptera;Lycaena phlaeas;Lycaena phlaeas;1;Lycaena phlaeas;0.999999992669558;
MH420224;Lepidoptera;Lasiommata megera;Lasiommata megera;1;Lasiommata megera;0.999806890129977;
MH420225;Lepidoptera;Colias crocea;NA;0.059;NA;0.999999999999738;
MH420226;Lepidoptera;Coenonympha corinna;Coenonympha corinna;1;Coenonympha
corinna;0.999999556334798;
MH420229;Lepidoptera;Papilio machaon;Papilio machaon;0.001;Papilio machaon;0.999463129150422;
MH420231;Lepidoptera;Pieris rapae;NA;NA;Pieris rapae;0.999992588119495;
MH420232;Lepidoptera;Lycaena phlaeas;Lycaena phlaeas;1;Lycaena phlaeas;0.999999998167567;
MH420233;Lepidoptera;Lampides boeticus;Lampides boeticus;1;Lampides boeticus;0.99999999876789;
MH420234;Lepidoptera;Gonepteryx cleopatra;Gonepteryx cleopatra;0.998;Gonepteryx
cleopatra;0.999118636582266;
MH420236;Lepidoptera;Callophrys rubi;NA;0.001;Callophrys rubi;0.998885705938309;
MH420240;Lepidoptera;Vanessa cardui;Vanessa cardui;1;Vanessa cardui;0.999999850530696;
MH420241;Lepidoptera;Charaxes jasius;Charaxes jasius;NA;Charaxes jasius;0.990397821479112;
MH420242;Lepidoptera;Polyommatus icarus;Polyommatus icarus;0.001;Polyommatus
icarus;0.999851522482238;
MH420242;Lepidoptera;Polyommatus icarus;Polyommatus icarus;0.001;Polyommatus
icarus;0.999851522482238;
MH420243;Lepidoptera;Papilio machaon;Papilio machaon;0.003;Papilio machaon;0.999482752749035;
MH420246;Lepidoptera;Maniola cecilia;Pyronia cecilia;NA;Pyronia cecilia;0.999999999999972;
MH420248;Lepidoptera;Maniola jurtina;NA;0.038;NA;0.999999999999524;
MH420250;Lepidoptera;Lampides boeticus;Lampides boeticus;1;Lampides boeticus;0.999999999545906;
MH420251;Lepidoptera;Coenonympha corinna;Coenonympha corinna;1;Coenonympha
corinna;0.999999999567109;
MH420252;Lepidoptera;Gonepteryx cleopatra;Gonepteryx cleopatra;0.998;Gonepteryx
cleopatra;0.999118636582266;
MH420253;Lepidoptera;Maniola cecilia;Pyronia cecilia;NA;Pyronia cecilia;1;
MH420254;Lepidoptera;Vanessa cardui;Vanessa cardui;1;Vanessa cardui;0.999999459983318;
MH420255;Lepidoptera;Gonepteryx rhamni;NA;0.474;Gonepteryx rhamni;0.982076396127858;
MH420256;Lepidoptera;Vanessa atalanta;Vanessa atalanta;1;Vanessa atalanta;0.999999999647542;
MH420258;Lepidoptera;Aglais urticae;NA;0.002;Aglais urticae;0.987497593219019;
MH420259;Lepidoptera;Maniola cecilia;Pyronia cecilia;1;Pyronia cecilia;1;
MH420260;Lepidoptera;Anthocharis cardamines;Anthocharis cardamines;1;Anthocharis
cardamines;0.99999997953563;
MH420261;Lepidoptera;Pieris napi;NA;0.011;Pieris napi;0.801985394724814;
MH420262;Lepidoptera;Maniola cecilia;Pyronia cecilia;NA;Pyronia cecilia;1;
MH420263;Lepidoptera;Colias crocea;NA;0.059;NA;0.999999999999738;
MH420266;Lepidoptera;Pieris rapae;NA;0.087;Pieris rapae;0.999955370233307;
MH420267;Lepidoptera;Vanessa cardui;Vanessa cardui;1;Vanessa cardui;0.999991578978624;
MH420268;Lepidoptera;Maniola cecilia;Pyronia cecilia;1;Pyronia cecilia;1;
MH420269;Lepidoptera;Lasiommata megera;Lasiommata megera;1;Lasiommata megera;0.999379144055441;
MH420271;Lepidoptera;Vanessa cardui;Vanessa cardui;1;Vanessa cardui;0.999999850530696;
MH420273;Lepidoptera;Lasiommata megera;Lasiommata megera;1;Lasiommata megera;0.999379144055441;
MH420274;Lepidoptera;Pieris rapae;NA;NA;Pieris rapae;0.999992588119495;
MH420275;Lepidoptera;Celastrina argiolus;Celastrina argiolus;1;Celastrina
argiolus;0.999527385150531;
MH420276;Lepidoptera;Pyrgus armoricanus;Pyrgus armoricanus;1;Pyrgus
armoricanus;0.999997353848817;
MH420277;Lepidoptera;Coenonympha pamphilus;Coenonympha pamphilus;1;Coenonympha
pamphilus;0.999999999999744;
MH420280;Lepidoptera;Gonepteryx rhamni;NA;0.174;Gonepteryx rhamni;0.988654195211968;
MH420282;Lepidoptera;Celastrina argiolus;Celastrina argiolus;1;Celastrina
argiolus;0.999527385150531;
MH420284;Lepidoptera;Lampides boeticus;Lampides boeticus;1;Lampides boeticus;0.999999999545906;
MH420285;Lepidoptera;Vanessa cardui;Vanessa cardui;1;Vanessa cardui;0.999999850530696;
MH420286;Lepidoptera;Pieris rapae;NA;NA;Pieris rapae;0.999992588119495;
MH420287;Lepidoptera;Coenonympha corinna;Coenonympha corinna;1;Coenonympha
corinna;0.999999556334798;
MH420288;Lepidoptera;Pieris napi;NA;0.011;Pieris napi;0.801985394724814;
MH420289;Lepidoptera;Hipparchia aristaeus;Hipparchia aristaeus;1;Hipparchia
aristaeus;0.999999993824275;
MH420290;Lepidoptera;Lycaena phlaeas;Lycaena phlaeas;1;Lycaena phlaeas;0.999999996363186;
MH420292;Lepidoptera;Pyrgus armoricanus;Pyrgus armoricanus;1;Pyrgus
armoricanus;0.999997353848817;
MH420293;Lepidoptera;Carcharodus alceae;Carcharodus alceae;1;Carcharodus
alceae;0.999999998465938;
MH420294;Lepidoptera;Lasiommata megera;Lasiommata megera;1;Lasiommata megera;0.999379144055441;
MH420295;Lepidoptera;Vanessa atalanta;Vanessa atalanta;1;Vanessa atalanta;0.999999999757762;
MH420296;Lepidoptera;Carcharodus alceae;Carcharodus alceae;1;Carcharodus
alceae;0.999999998465938;
MH420297;Lepidoptera;Charaxes jasius;Charaxes jasius;NA;Charaxes jasius;0.990397821479112;
MH420298;Lepidoptera;Vanessa atalanta;Vanessa atalanta;1;Vanessa atalanta;0.999999999757762;
MH420300;Lepidoptera;Colias crocea;NA;0.059;NA;0.999999999999738;
MH420301;Lepidoptera;Celastrina argiolus;Celastrina argiolus;0.999;Celastrina
argiolus;0.999536508848955;

MH420307;Lepidoptera;Lampides boeticus;Lampides boeticus;1;Lampides boeticus;0.999999999620769;
MH420308;Lepidoptera;Lasiommata paramegaera;Lasiommata paramegaera;0.999;Lasiommata paramegaera;0.995283446287531;
MH420309;Lepidoptera;Coenonympha corinna;Coenonympha corinna;1;Coenonympha corinna;0.999999866312132;
MH420310;Lepidoptera;Carcharodus alceae;Carcharodus alceae;0.999;Carcharodus alceae;0.999999998008462;
MH420311;Lepidoptera;Papilio machaon;Papilio machaon;0.001;Papilio machaon;0.999463129150422;
MH420312;Lepidoptera;Celastrina argiolus;Celastrina argiolus;1;Celastrina argiolus;0.999527385150531;
MH420313;Lepidoptera;Pieris napi;NA;0.028;Pieris napi;0.888205720664699;
MH420314;Lepidoptera;Pieris rapae;NA;NA;Pieris rapae;0.999970925323953;
MH420315;Lepidoptera;Lycaena phlaeas;Lycaena phlaeas;1;Lycaena phlaeas;0.999999998167567;
MH420316;Lepidoptera;Lasiommata megera;Lasiommata megera;1;Lasiommata megera;0.999379144055441;
MH420320;Lepidoptera;Aricia agestis;Aricia agestis;0.001;Aricia agestis;0.972120797608056;
MH420321;Lepidoptera;Celastrina argiolus;Celastrina argiolus;1;Celastrina argiolus;0.999437501405169;
MH420324;Lepidoptera;Celastrina argiolus;Celastrina argiolus;1;Celastrina argiolus;0.999527385150531;
MH420325;Lepidoptera;Aricia agestis;NA;0.067;Aricia agestis;0.999509743227786;
MH420326;Lepidoptera;Lycaena phlaeas;Lycaena phlaeas;1;Lycaena phlaeas;0.999999998167567;
MH420327;Lepidoptera;Vanessa cardui;Vanessa cardui;1;Vanessa cardui;0.999999459983318;
MH420328;Lepidoptera;Carcharodus alceae;Carcharodus alceae;0.999;Carcharodus alceae;0.999999998008462;
MH420329;Lepidoptera;Hipparchia neomiris;Hipparchia neomiris;1;Hipparchia neomiris;0.999999999999801;
MH420330;Lepidoptera;Pieris rapae;Pieris rapae;NA;Pieris rapae;0.999994302747155;
MH420332;Lepidoptera;Colias crocea;NA;0.059;NA;0.999999999999738;
MH420333;Lepidoptera;Callophrys rubi;NA;0.001;Callophrys rubi;0.998885705938309;
MH420334;Lepidoptera;Satyrium ilicis;Satyrium ilicis;1;Satyrium ilicis;0.999999984618455;
MH420335;Lepidoptera;Lasiommata megera;Lasiommata megera;1;Lasiommata megera;0.999379144055441;
MH420336;Lepidoptera;Lampides boeticus;Lampides boeticus;1;Lampides boeticus;0.999999999545906;
MH420337;Lepidoptera;Celastrina argiolus;Celastrina argiolus;1;Celastrina argiolus;0.999037794018796;
MH420339;Lepidoptera;Gonepteryx rhamni;NA;0.474;Gonepteryx rhamni;0.982076396127858;
MH420340;Lepidoptera;Colias crocea;NA;0.059;NA;0.999999999999738;
MH420341;Lepidoptera;Anthocharis cardamines;Anthocharis cardamines;1;Anthocharis cardamines;0.99999997953563;
MH420342;Lepidoptera;Lycaena phlaeas;Lycaena phlaeas;1;Lycaena phlaeas;0.999999994647311;
MH420343;Lepidoptera;Coenonympha pamphilus;Coenonympha pamphilus;1;Coenonympha pamphilus;0.999999999999744;
MH420344;Lepidoptera;Coenonympha corinna;Coenonympha corinna;1;Coenonympha corinna;0.999999999567109;
MH420345;Lepidoptera;Lycaena phlaeas;Lycaena phlaeas;1;Lycaena phlaeas;0.999999998167567;
MH420347;Lepidoptera;Celastrina argiolus;Celastrina argiolus;1;Celastrina argiolus;0.999037794018796;
MH420348;Lepidoptera;Maniola cecilia;Pyronia cecilia;NA;Pyronia cecilia;1;
MH420350;Lepidoptera;Pieris rapae;Pieris rapae;1;Pieris rapae;0.999994248088769;
MH420351;Lepidoptera;Carcharodus alceae;Carcharodus alceae;0.999;Carcharodus alceae;0.999999998008462;
MH420352;Lepidoptera;Pieris rapae;NA;NA;Pieris rapae;0.999992588119495;
MH420353;Lepidoptera;Maniola jurtina;NA;0.001;Maniola jurtina;0.939220105860875;
MH420354;Lepidoptera;Gegenes pumilio;Gegenes pumilio;1;Gegenes pumilio;0.999986726679266;
MH420356;Lepidoptera;Coenonympha pamphilus;Coenonympha pamphilus;1;Coenonympha pamphilus;0.999999999999488;
MH420357;Lepidoptera;Aricia agestis;NA;NA;Aricia agestis;0.999677837947498;
MH420358;Lepidoptera;Gonepteryx cleopatra;Gonepteryx cleopatra;0.001;Gonepteryx cleopatra;0.999115549984292;
MH420360;Lepidoptera;Aglais urticae;Aglais urticae;0.001;Aglais urticae;0.990690600684087;
MH420361;Lepidoptera;Argynnis paphia;Argynnis paphia;1;Argynnis paphia;0.999999991389927;
MH420362;Lepidoptera;Maniola cecilia;Pyronia cecilia;1;Pyronia cecilia;1;
MH420365;Lepidoptera;Anthocharis cardamines;Anthocharis cardamines;1;Anthocharis cardamines;0.99999997953563;
MH420366;Lepidoptera;Lycaena phlaeas;Lycaena phlaeas;1;Lycaena phlaeas;0.999999996363186;
MH420367;Lepidoptera;Coenonympha pamphilus;Coenonympha pamphilus;1;Coenonympha pamphilus;0.999999999999744;
MH420368;Lepidoptera;Pieris napi;NA;0.011;Pieris napi;0.801985394724814;
MH420369;Lepidoptera;Celastrina argiolus;Celastrina argiolus;1;Celastrina argiolus;0.999527385150531;
MH420370;Lepidoptera;Issoria lathonia;Issoria lathonia;1;Issoria lathonia;0.999999999989257;
MH420371;Lepidoptera;Callophrys rubi;NA;0.001;Callophrys rubi;0.998885705938309;
MH420373;Lepidoptera;Gonepteryx rhamni;Gonepteryx rhamni;1;Gonepteryx rhamni;0.999673119534565;
MH420376;Lepidoptera;Maniola tithonus;Pyronia tithonus;1;Pyronia tithonus;1;
MH420378;Lepidoptera;Colias crocea;NA;0.059;NA;0.999999999999738;
MH420379;Lepidoptera;Carcharodus alceae;Carcharodus alceae;1;Carcharodus alceae;0.999999995267814;
MH420380;Lepidoptera;Maniola cecilia;Pyronia cecilia;1;Pyronia cecilia;1;
MH420381;Lepidoptera;Maniola jurtina;NA;0.195;Maniola jurtina;0.942918068360979;

MH420382;Lepidoptera;Maniola cecilia;Pyronia cecilia;1;Pyronia cecilia;1;
MH420388;Lepidoptera;Lasiommata megera;Lasiommata megera;1;Lasiommata megera;0.999379144055441;
MH420391;Lepidoptera;Pieris rapae;NA;0.175;Pieris rapae;0.99996637543374;
MH420392;Lepidoptera;Lampides boeticus;Lampides boeticus;1;Lampides boeticus;0.999999999545906;
MH420393;Lepidoptera;Maniola cecilia;Pyronia cecilia;1;Pyronia cecilia;1;
MH420394;Lepidoptera;Pyrgus armoricanus;Pyrgus armoricanus;1;Pyrgus armoricanus;0.999997353848817;
MH420398;Lepidoptera;Glaucopsyche alexis;NA;NA;Glaucopsyche alexis;0.97938116962406 9;
MH420399;Lepidoptera;Vanessa cardui;Vanessa cardui;1;Vanessa cardui;0.999999459983318;
MH420400;Lepidoptera;Celastrina argiolus;Celastrina argiolus;1;Celastrina argiolus;0.999037794018796;
MH420403;Lepidoptera;Thymelicus acteon;Thymelicus acteon;1;Thymelicus acteon;0.999999995239193;
MH420405;Lepidoptera;Coenonympha pamphilus;Coenonympha pamphilus;1;Coenonympha pamphilus;0.999999999999744;
MH420406;Lepidoptera;Lasiommata megera;Lasiommata megera;1;Lasiommata megera;0.998531205767617;
MH420407;Lepidoptera;Polyommatus icarus;Polyommatus icarus;0.001;Polyommatus icarus;0.999851522482238;
MH420407;Lepidoptera;Polyommatus icarus;Polyommatus icarus;0.001;Polyommatus icarus;0.999851522482238;
MH420409;Lepidoptera;Lasiommata megera;Lasiommata megera;1;Lasiommata megera;0.999928528239676;
MH420411;Lepidoptera;Coenonympha pamphilus;Coenonympha pamphilus;1;Coenonympha pamphilus;0.999999999983061;
MH420412;Lepidoptera;Vanessa cardui;Vanessa cardui;1;Vanessa cardui;0.999999459983318;
MH420413;Lepidoptera;Charaxes jasius;Charaxes jasius;NA;Charaxes jasius;0.990397821479112;
MH420415;Lepidoptera;Colias crocea;NA;0.059;NA;0.999999999999738;
MH420416;Lepidoptera;Lasiommata megera;Lasiommata megera;1;Lasiommata megera;0.999379144055441;
MH420417;Lepidoptera;Vanessa cardui;Vanessa cardui;1;Vanessa cardui;0.999999459983318;
MH420418;Lepidoptera;Vanessa atalanta;Vanessa atalanta;1;Vanessa atalanta;0.999999999130267;
MH670083;Lepidoptera;Erebia manto;NA;0.061;Erebia bubastis;0.999952281744477;
MH670084;Lepidoptera;Erebia manto;NA;0.031;Erebia bubastis;0.99994721927445;
MH670085;Lepidoptera;Erebia manto;NA;0.061;Erebia bubastis;0.999962951682882;
MH670086;Lepidoptera;Erebia manto;NA;0.073;Erebia bubastis;0.999956603717016;
MH670087;Lepidoptera;Erebia manto;NA;0.174;Erebia bubastis;0.999970399901456;
MH670088;Lepidoptera;Erebia manto;NA;0.174;Erebia bubastis;0.999970399901456;
MH670089;Lepidoptera;Erebia manto;Erebia manto;NA;Erebia bubastis;0.999886022205242;
MH670090;Lepidoptera;Erebia manto;Erebia manto;0.999;Erebia bubastis;0.999660353922442;
MH670091;Lepidoptera;Erebia manto;NA;0.187;Erebia bubastis;0.999973083726829;
MH670092;Lepidoptera;Erebia manto;NA;0.067;Erebia bubastis;0.99997153066746;
MH670097;Lepidoptera;Erebia manto;NA;0.009;Erebia bubastis;0.999981998688491;
MH670098;Lepidoptera;Erebia manto;NA;0.187;Erebia bubastis;0.999973083726829;
MH670099;Lepidoptera;Erebia manto;NA;0.009;Erebia bubastis;0.999982898283498;
MH670100;Lepidoptera;Erebia eriphyle;Erebia eriphyle;1;NA;0.795999353777463;
MH670101;Lepidoptera;Erebia manto;NA;0.009;Erebia bubastis;0.999982898283498;
MH670102;Lepidoptera;Erebia manto;NA;0.009;Erebia bubastis;0.999982898283498;
MH670103;Lepidoptera;Erebia manto;NA;0.187;Erebia bubastis;0.999973083726829;
MH670106;Lepidoptera;Erebia manto;NA;0.187;Erebia bubastis;0.999972982836091;
MH670108;Lepidoptera;Erebia manto;NA;0.181;Erebia bubastis;0.999943713439002;
MH670109;Lepidoptera;Erebia manto;NA;0.187;Erebia bubastis;0.999973083726829;
MH670110;Lepidoptera;Erebia manto;NA;0.067;Erebia bubastis;0.999969987709769;
MH670116;Lepidoptera;Erebia christi;Erebia christi;1;Erebia christi;0.997897965375107;
MH670121;Lepidoptera;Erebia manto;Erebia manto;NA;Erebia bubastis;0.999886022205242;
MH670122;Lepidoptera;Erebia manto;NA;0.187;Erebia bubastis;0.999973083726829;
MH670123;Lepidoptera;Erebia manto;NA;0.181;Erebia bubastis;0.999943713439002;
MH670124;Lepidoptera;Erebia manto;NA;0.067;Erebia bubastis;0.999969987709769;
MH670126;Lepidoptera;Erebia manto;Erebia manto;NA;Erebia bubastis;0.999886022205242;
MH670127;Lepidoptera;Erebia manto;Erebia manto;NA;Erebia bubastis;0.999866474678959;
MH670128;Lepidoptera;Erebia manto;NA;0.009;Erebia bubastis;0.999982898283498;
MH670129;Lepidoptera;Erebia manto;NA;0.009;Erebia bubastis;0.999981998688491;
MH670130;Lepidoptera;Erebia manto;NA;0.174;Erebia bubastis;0.999970399901456;
MH670131;Lepidoptera;Erebia manto;NA;0.031;Erebia bubastis;0.99994721927445;
MH670132;Lepidoptera;Erebia manto;NA;0.119;Erebia bubastis;0.980474940339695;
MH670133;Lepidoptera;Erebia manto;NA;0.067;Erebia bubastis;0.99997153066746;
MH670134;Lepidoptera;Erebia manto;NA;0.174;Erebia bubastis;0.999970399901456;
MH670135;Lepidoptera;Erebia manto;NA;0.174;Erebia bubastis;0.999970399901456;
MH670136;Lepidoptera;Erebia manto;NA;0.174;Erebia bubastis;0.999970399901456;
MH670138;Lepidoptera;Erebia manto;NA;0.187;Erebia bubastis;0.999973083726829;
MH670139;Lepidoptera;Erebia manto;NA;0.067;Erebia bubastis;0.999969987709769;
MH670140;Lepidoptera;Erebia manto;NA;0.067;Erebia bubastis;0.999969987709769;
MH670141;Lepidoptera;Erebia manto;NA;0.031;Erebia bubastis;0.99994721927445;
MH670142;Lepidoptera;Erebia manto;NA;0.187;Erebia bubastis;0.999972982836091;
MH670143;Lepidoptera;Erebia manto;NA;0.031;Erebia bubastis;0.99994721927445;
MH670144;Lepidoptera;Erebia christi;Erebia christi;1;Erebia christi;0.997897965375107;
MH670145;Lepidoptera;Erebia manto;NA;0.009;Erebia bubastis;0.999981998688491;
MH670146;Lepidoptera;Erebia manto;NA;NA;Erebia manto;0.998390110509447;
MH670147;Lepidoptera;Erebia manto;NA;0.187;Erebia bubastis;0.999973083726829;
MH670148;Lepidoptera;Erebia eriphyle;Erebia eriphyle;1;NA;0.795999353777463;
MH670149;Lepidoptera;Erebia manto;NA;0.174;Erebia bubastis;0.999970399901456;
MH670150;Lepidoptera;Erebia manto;NA;0.187;Erebia bubastis;0.999973083726829;

MH670151;Lepidoptera;Erebia manto;NA;NA;Erebia manto;0.994206031771192;
MH670152;Lepidoptera;Erebia manto;NA;0.187;Erebia bubastis;0.999972982836091;
MH670153;Lepidoptera;Erebia manto;NA;0.181;Erebia bubastis;0.999943713439002;
MH670154;Lepidoptera;Erebia manto;NA;0.009;Erebia bubastis;0.999981998688491;
MH670155;Lepidoptera;Erebia manto;NA;0.067;Erebia bubastis;0.999969987709769;
MH670156;Lepidoptera;Erebia manto;NA;0.187;Erebia bubastis;0.999973083726829;
MH670157;Lepidoptera;Erebia manto;NA;0.187;Erebia bubastis;0.999973083726829;
MH670158;Lepidoptera;Erebia manto;NA;0.174;Erebia bubastis;0.999970399901456;
MH670159;Lepidoptera;Erebia manto;Erebia manto;NA;Erebia bubastis;0.999886022205242;
MH670160;Lepidoptera;Erebia manto;NA;NA;Erebia manto;0.994206031771192;
MH670161;Lepidoptera;Erebia manto;NA;0.073;Erebia bubastis;0.999956603717016;
MH670162;Lepidoptera;Erebia manto;Erebia manto;NA;Erebia bubastis;0.999886022205242;
MH670163;Lepidoptera;Erebia manto;NA;0.073;Erebia bubastis;0.999956603717016;
MH670164;Lepidoptera;Erebia manto;NA;0.187;Erebia bubastis;0.999972982836091;
MH670165;Lepidoptera;Erebia eriphyle;Erebia eriphyle;1;NA;0.795999353777463;
MH670166;Lepidoptera;Erebia manto;NA;0.181;Erebia bubastis;0.999943713439002;
MH670167;Lepidoptera;Erebia manto;NA;NA;Erebia bubastis;0.999967994349337;
MH670168;Lepidoptera;Erebia manto;Erebia manto;NA;Erebia bubastis;0.999886022205242;
MH670169;Lepidoptera;Erebia manto;NA;0.187;Erebia bubastis;0.999973083726829;
MH670170;Lepidoptera;Erebia manto;NA;0.187;Erebia bubastis;0.999973083726829;
MH670171;Lepidoptera;Erebia manto;NA;0.009;Erebia bubastis;0.999981998688491;
MH670172;Lepidoptera;Erebia manto;NA;0.174;Erebia bubastis;0.999969474482721;
MH670173;Lepidoptera;Erebia manto;Erebia manto;NA;Erebia bubastis;0.999886022205242;
MH670174;Lepidoptera;Erebia manto;NA;0.009;Erebia bubastis;0.999982898283498;
MH670175;Lepidoptera;Erebia manto;NA;0.073;Erebia bubastis;0.999956603717016;
MH670176;Lepidoptera;Erebia manto;NA;0.009;Erebia bubastis;0.999981998688491;
MH670177;Lepidoptera;Erebia manto;NA;0.187;Erebia bubastis;0.999973083726829;
MH670178;Lepidoptera;Erebia manto;NA;0.174;Erebia bubastis;0.999969474482721;
MH670179;Lepidoptera;Erebia manto;NA;0.174;Erebia bubastis;0.99996840517015;
MH670180;Lepidoptera;Erebia manto;NA;0.187;Erebia bubastis;0.999973083726829;
MH670181;Lepidoptera;Erebia manto;NA;0.174;Erebia bubastis;0.999970399901456;
MH670182;Lepidoptera;Erebia manto;NA;0.054;Erebia bubastis;0.999957130162365;
MH670183;Lepidoptera;Erebia manto;NA;0.009;Erebia bubastis;0.999982898283498;
MH670184;Lepidoptera;Erebia manto;NA;0.009;Erebia bubastis;0.999982898283498;
MH670185;Lepidoptera;Erebia manto;NA;0.067;Erebia bubastis;0.999971289249363;
MH670186;Lepidoptera;Erebia manto;NA;NA;Erebia manto;0.998390110509447;
MH670187;Lepidoptera;Erebia manto;NA;NA;Erebia manto;0.998390110509447;
MH670188;Lepidoptera;Erebia manto;NA;0.174;Erebia bubastis;0.999968521048825;
MH670189;Lepidoptera;Erebia manto;NA;0.174;Erebia bubastis;0.999970399901456;
MH670190;Lepidoptera;Erebia manto;NA;NA;Erebia manto;0.994206031771192;
MH670191;Lepidoptera;Erebia manto;NA;0.187;Erebia bubastis;0.999972982836091;
MH670192;Lepidoptera;Erebia manto;NA;0.174;Erebia bubastis;0.999968521048825;
MH670193;Lepidoptera;Erebia manto;NA;0.187;Erebia bubastis;0.999972982836091;
MH670194;Lepidoptera;Erebia manto;NA;0.116;Erebia manto;0.987009895446114;
MH670195;Lepidoptera;Erebia manto;NA;0.073;Erebia bubastis;0.999956603717016;
MH670196;Lepidoptera;Erebia manto;NA;0.174;Erebia bubastis;0.999970399901456;
MH670197;Lepidoptera;Erebia manto;Erebia manto;NA;Erebia bubastis;0.999886022205242;
MH670198;Lepidoptera;Erebia manto;NA;NA;Erebia manto;0.994206031771192;
MH670199;Lepidoptera;Erebia manto;Erebia manto;NA;Erebia bubastis;0.999886022205242;
MH670200;Lepidoptera;Erebia manto;NA;0.187;Erebia bubastis;0.999973076462209;
MH670201;Lepidoptera;Erebia manto;NA;0.181;Erebia bubastis;0.999943713439002;
MH670202;Lepidoptera;Erebia manto;Erebia manto;0.001;Erebia bubastis;0.999825906151859;
MH670203;Lepidoptera;Erebia manto;NA;0.187;Erebia bubastis;0.999972982836091;
MH670204;Lepidoptera;Erebia manto;NA;0.009;Erebia bubastis;0.999981998688491;
MH670205;Lepidoptera;Erebia manto;NA;0.067;Erebia bubastis;0.999969987709769;
MH670206;Lepidoptera;Erebia manto;NA;0.174;Erebia bubastis;0.99996840517015;
MH670207;Lepidoptera;Erebia manto;NA;0.009;Erebia bubastis;0.999982898283498;
MH670208;Lepidoptera;Erebia manto;NA;0.009;Erebia bubastis;0.999981998688491;
MH670209;Lepidoptera;Erebia manto;NA;0.067;Erebia bubastis;0.999969987709769;
MH670210;Lepidoptera;Erebia manto;NA;0.116;Erebia manto;0.987009895446114;
MH670211;Lepidoptera;Erebia manto;NA;0.161;Erebia bubastis;0.986242916492014;
MH670212;Lepidoptera;Erebia manto;NA;0.187;Erebia bubastis;0.999973083726829;
MH670213;Lepidoptera;Erebia manto;NA;0.187;Erebia bubastis;0.999973083726829;
MH670214;Lepidoptera;Erebia manto;NA;0.067;Erebia bubastis;0.999969987709769;
MH670215;Lepidoptera;Erebia manto;Erebia manto;NA;Erebia bubastis;0.999769088786808;
MH670216;Lepidoptera;Erebia manto;NA;0.187;Erebia bubastis;0.999973083726829;
MH670217;Lepidoptera;Erebia manto;NA;0.054;Erebia bubastis;0.999954422787626;
MH670218;Lepidoptera;Erebia manto;NA;0.187;Erebia bubastis;0.999973083726829;
MH670219;Lepidoptera;Erebia manto;NA;NA;NA;0.869827053877523;
MH670220;Lepidoptera;Erebia manto;NA;0.009;Erebia bubastis;0.999981998688491;
MH670221;Lepidoptera;Erebia manto;NA;0.187;Erebia bubastis;0.999972982836091;
MH670222;Lepidoptera;Erebia eriphyle;Erebia eriphyle;1;NA;0.795999353777463;
MH670223;Lepidoptera;Erebia manto;NA;0.073;Erebia bubastis;0.999956603717016;
MH670224;Lepidoptera;Erebia manto;NA;0.174;Erebia bubastis;0.999971763691585;
MH670225;Lepidoptera;Erebia manto;NA;0.174;Erebia bubastis;0.999970399901456;
MH670226;Lepidoptera;Erebia manto;NA;0.187;Erebia bubastis;0.999972982836091;
MH670227;Lepidoptera;Erebia manto;NA;NA;Erebia bubastis;0.999928470140733;
MH670228;Lepidoptera;Erebia manto;NA;0.174;Erebia bubastis;0.999970541605215;

MH670229;Lepidoptera;Erebia manto;NA;0.009;Erebia bubastis;0.999982898283498;
MH670230;Lepidoptera;Erebia manto;NA;0.161;Erebia bubastis;0.986242916492014;
MH670231;Lepidoptera;Erebia eriphyle;Erebia eriphyle;1;NA;0.795999353777463;
MH670232;Lepidoptera;Erebia manto;NA;NA;Erebia manto;0.998390110509447;
MH670233;Lepidoptera;Erebia manto;Erebia manto;NA;Erebia bubastis;0.999886022205242;
MH670234;Lepidoptera;Erebia manto;NA;0.071;Erebia bubastis;0.999958106329886;
MH670235;Lepidoptera;Erebia manto;NA;0.174;Erebia bubastis;0.999970399901456;
MH670236;Lepidoptera;Erebia manto;NA;0.174;Erebia bubastis;0.99996840517015;
MIMAB007-21;Lepidoptera;Colias alfacariensis;Colias alfacariensis;0.001;Colias alfacariensis;0.999906024479126;
MIMAB008-21;Lepidoptera;Cupido argiades;NA;0.485;NA;0.999999999956215;
MIMAB009-21;Lepidoptera;Cupido argiades;NA;0.485;NA;0.999999999956215;
MIMAB010-21;Lepidoptera;Pieris napi;NA;0.023;Pieris napi;0.906511131816347;
MIMAB011-21;Lepidoptera;Cupido minimus;NA;0.001;Cupido minimus;0.999561411197707;
MIMAB015-21;Lepidoptera;Cupido alcetas;NA;0.206;Cupido alcetas;0.779913020015273;
MIMAB016-21;Lepidoptera;Cupido minimus;NA;0.001;Cupido minimus;0.999561411197707;
MIMAB018-21;Lepidoptera;Plebejus argus;NA;NA;Plebejus argus;0.925616774870762;
MIMAB019-21;Lepidoptera;Polyommatus thersites;NA;0.084;Polyommatus thersites;0.999999717339063;
MIMAB020-21;Lepidoptera;Plebejus argus;NA;0.124;Plebejus argus;0.964152976292701;
MIMAB024-21;Lepidoptera;Plebejus argus;NA;0.107;Plebejus argus;0.959600391999422;
MIMAB025-21;Lepidoptera;Plebejus argus;NA;0.001;Plebejus argus;0.915815988501866;
MIMAB026-21;Lepidoptera;Cupido minimus;NA;0.001;Cupido minimus;0.999561411197707;
MIMAB027-21;Lepidoptera;Cyaniris semiargus;NA;NA;Cyaniris semiargus;0.998328437758208;
MIMAB028-21;Lepidoptera;Colias alfacariensis;Colias alfacariensis;1;Colias alfacariensis;0.999982848414142;
MIMAB029-21;Lepidoptera;Cupido minimus;NA;0.001;Cupido minimus;0.999561411197707;
MIMAB030-21;Lepidoptera;Cupido minimus;NA;0.001;Cupido minimus;0.999561411197707;
MIMAB031-21;Lepidoptera;Polyommatus dorylas;NA;0.003;Polyommatus dorylas;0.983348973322966;
MIMAB032-21;Lepidoptera;Polyommatus dorylas;Polyommatus dorylas;0.007;Polyommatus dorylas;0.985357223577053;
MIMAB034-21;Lepidoptera;Plebejus argus;NA;0.312;Plebejus argus;0.959981154917144;
MIMAB035-21;Lepidoptera;Polyommatus thersites;Polyommatus thersites;0.001;Polyommatus thersites;0.999999810731499;
MIMAB036-21;Lepidoptera;Plebejus idas;NA;0.004;NA;0.98802695424595;
MIMAB037-21;Lepidoptera;Polyommatus escheri;Polyommatus escheri;1;Polyommatus escheri;0.998452839845543;
MIMAB038-21;Lepidoptera;Polyommatus escheri;Polyommatus escheri;0.998;Polyommatus escheri;0.994962659025331;
MIMAB040-21;Lepidoptera;Aricia artaxerxes;NA;0.001;Aricia artaxerxes;0.999371219094886;
MIMAB041-21;Lepidoptera;Plebejus idas;NA;0.004;NA;0.98802695424595;
MIMAB044-21;Lepidoptera;Erebia epiphron;NA;0.026;Erebia epiphron;0.987135038251135;
MIMAB045-21;Lepidoptera;Erebia medusa;NA;0.009;Erebia medusa;0.977889176387507;
MIMAB047-21;Lepidoptera;Erebia oeme;NA;0.127;Erebia oeme;0.99999999980264;
MIMAB048-21;Lepidoptera;Melitaea britomartis;Mellicta britomartis;NA;Melitaea britomartis;0.895364772813994;
MIMAB049-21;Lepidoptera;Aricia artaxerxes;NA;0.001;Aricia artaxerxes;0.999371219094886;
MIMAB050-21;Lepidoptera;Melitaea didyma;Melitaea didyma;1;Melitaea didyma;0.983780737467472;
MIMAB058-21;Lepidoptera;Coenonympha pamphilus;Coenonympha pamphilus;1;Coenonympha pamphilus;0.999999999997755;
MIMAB059-21;Lepidoptera;Hipparchia semele;NA;NA;NA;0.999999999999977;
MK155178;Lepidoptera;Erebia epiphron;NA;NA;Erebia epiphron;0.994401488812176;
MK155179;Lepidoptera;Erebia epiphron;NA;NA;Erebia epiphron;0.994401488812176;
MK155180;Lepidoptera;Erebia epiphron;Erebia epiphron;0.01;Erebia epiphron;0.995390903570694;
MK155181;Lepidoptera;Erebia epiphron;Erebia epiphron;1;Erebia epiphron;0.99544005977253;
MK155183;Lepidoptera;Erebia epiphron;Erebia epiphron;NA;Erebia epiphron;0.997944792650423;
MK155184;Lepidoptera;Erebia epiphron;Erebia epiphron;NA;Erebia epiphron;0.995580956478864;
MK155185;Lepidoptera;Erebia epiphron;Erebia epiphron;1;Erebia epiphron;0.99544005977253;
MK155187;Lepidoptera;Erebia epiphron;Erebia epiphron;1;Erebia epiphron;0.99544005977253;
MK155188;Lepidoptera;Erebia epiphron;Erebia epiphron;1;Erebia epiphron;0.99544005977253;
MK155190;Lepidoptera;Erebia epiphron;Erebia epiphron;NA;Erebia epiphron;0.997944792650423;
MK155191;Lepidoptera;Erebia epiphron;Erebia epiphron;1;Erebia epiphron;0.99544005977253;
MK155192;Lepidoptera;Erebia epiphron;NA;NA;Erebia epiphron;0.997605378796729;
MK155193;Lepidoptera;Erebia epiphron;Erebia epiphron;NA;Erebia epiphron;0.995231643306052;
MK155194;Lepidoptera;Erebia epiphron;NA;NA;Erebia epiphron;0.995769974843751;
MK155195;Lepidoptera;Erebia epiphron;Erebia epiphron;0.002;Erebia epiphron;0.998011506298672;
MK155196;Lepidoptera;Erebia epiphron;Erebia epiphron;NA;Erebia epiphron;0.998243429957028;
MK155197;Lepidoptera;Erebia epiphron;Erebia epiphron;NA;Erebia epiphron;0.998704677292374;
MK155198;Lepidoptera;Erebia epiphron;NA;NA;Erebia epiphron;0.950006471153786;
MK155199;Lepidoptera;Erebia epiphron;Erebia epiphron;NA;Erebia epiphron;0.998704677292374;
MK155202;Lepidoptera;Erebia epiphron;NA;NA;Erebia epiphron;0.991535930833796;
MK155204;Lepidoptera;Erebia epiphron;Erebia epiphron;NA;Erebia epiphron;0.995228972998118;
MK155205;Lepidoptera;Erebia epiphron;NA;NA;Erebia epiphron;0.994401488812176;
MK155208;Lepidoptera;Erebia epiphron;NA;NA;Erebia epiphron;0.997605378796729;
MK155210;Lepidoptera;Erebia epiphron;Erebia epiphron;1;Erebia epiphron;0.99544005977253;
MK155211;Lepidoptera;Erebia epiphron;NA;NA;Erebia epiphron;0.994401488812176;
MK155212;Lepidoptera;Erebia epiphron;NA;NA;Erebia epiphron;0.994401488812176;
MK155213;Lepidoptera;Erebia epiphron;NA;NA;Erebia epiphron;0.994401488812176;
MK155214;Lepidoptera;Erebia epiphron;Erebia epiphron;NA;Erebia epiphron;0.995228972998118;

MK155215;Lepidoptera;Erebia epiphron;NA;NA;Erebia epiphron;0.994401488812176;
MK155216;Lepidoptera;Erebia epiphron;Erebia epiphron;0.996;Erebia epiphron;0.998796590633588;
MK155217;Lepidoptera;Erebia epiphron;Erebia epiphron;1;Erebia epiphron;0.99544005977253;
MK186080;Lepidoptera;Aglais urticae;NA;NA;Aglais urticae;0.974538026641371;
MK186081;Lepidoptera;Aglais urticae;Aglais urticae;NA;Aglais urticae;0.99231717704338;
MK186082;Lepidoptera;Aglais urticae;Aglais urticae;0.001;Aglais urticae;0.985008525192552;
MK186083;Lepidoptera;Aglais urticae;NA;NA;Aglais urticae;0.978490324720571;
MK186084;Lepidoptera;Agriades glandon;NA;NA;Agriades glandon;0.98218506866083;
MK186085;Lepidoptera;Agriades glandon;NA;0.005;Agriades glandon;0.968622708022137;
MK186086;Lepidoptera;Agriades glandon;NA;0.002;Agriades glandon;0.986680741313409;
MK186087;Lepidoptera;Agriades glandon;NA;0.038;Agriades glandon;0.987540286070852;
MK186088;Lepidoptera;Agriades optilete;Agriades optilete;1;Agriades optilete;0.999999974686574;
MK186089;Lepidoptera;Agriades optilete;Agriades optilete;1;Agriades optilete;0.999999950306575;
MK186090;Lepidoptera;Agriades optilete;Agriades optilete;1;Agriades optilete;0.999999950306575;
MK186091;Lepidoptera;Agriades optilete;Agriades optilete;1;Agriades optilete;0.999999745052439;
MK186092;Lepidoptera;Agriades orbitulus;NA;0.007;Agriades orbitulus;0.998942443899012;
MK186093;Lepidoptera;Agriades orbitulus;NA;0.006;Agriades orbitulus;0.999040289385503;
MK186094;Lepidoptera;Agriades orbitulus;Agriades orbitulus;0.003;Agriades orbitulus;0.999098836056467;
MK186095;Lepidoptera;Agriades orbitulus;Agriades orbitulus;NA;Agriades orbitulus;0.999377047866573;
MK186096;Lepidoptera;Anthocharis cardamines;Anthocharis cardamines;1;Anthocharis cardamines;0.999999966992391;
MK186097;Lepidoptera;Anthocharis cardamines;Anthocharis cardamines;1;Anthocharis cardamines;0.999999985613698;
MK186098;Lepidoptera;Anthocharis cardamines;Anthocharis cardamines;1;Anthocharis cardamines;0.999999286738698;
MK186099;Lepidoptera;Anthocharis cardamines;Anthocharis cardamines;1;Anthocharis cardamines;0.999999925419277;
MK186100;Lepidoptera;Apatura ilia;NA;NA;Apatura ilia;0.953937859846525;
MK186101;Lepidoptera;Apatura ilia;NA;0.15;Apatura ilia;0.914163962669637;
MK186102;Lepidoptera;Apatura ilia;NA;NA;Apatura ilia;0.95526521600974;
MK186103;Lepidoptera;Apatura iris;Apatura iris;1;Apatura iris;0.999999999999574;
MK186104;Lepidoptera;Apatura iris;Apatura iris;1;Apatura iris;0.999999999999972;
MK186105;Lepidoptera;Aphantopus hyperantus;Aphantopus hyperantus;0.005;Aphantopus hyperantus;0.999999378983625;
MK186106;Lepidoptera;Aphantopus hyperantus;Aphantopus hyperantus;0.004;Aphantopus hyperantus;0.999996737240601;
MK186107;Lepidoptera;Aphantopus hyperantus;Aphantopus hyperantus;0.005;Aphantopus hyperantus;0.999999378983625;
MK186108;Lepidoptera;Aphantopus hyperantus;Aphantopus hyperantus;1;Aphantopus hyperantus;0.999997342368381;
MK186109;Lepidoptera;Aphantopus hyperantus;Aphantopus hyperantus;0.01;Aphantopus hyperantus;0.999445556923408;
MK186110;Lepidoptera;Aporia crataegi;NA;NA;Aporia crataegi;0.999994725800359;
MK186111;Lepidoptera;Aporia crataegi;NA;NA;Aporia crataegi;0.999973074492637;
MK186112;Lepidoptera;Aporia crataegi;NA;0.001;Aporia crataegi;0.999986855609891;
MK186113;Lepidoptera;Aporia crataegi;NA;0.021;Aporia crataegi;0.999991009226137;
MK186114;Lepidoptera;Aporia crataegi;NA;0.003;Aporia crataegi;0.999980213730065;
MK186115;Lepidoptera;Aporia crataegi;NA;0.057;Aporia crataegi;0.999959095839193;
MK186116;Lepidoptera;Aporia crataegi;NA;NA;Aporia crataegi;0.999994725800359;
MK186117;Lepidoptera;Araschnia levana;Araschnia levana;1;Araschnia levana;0.999999414185426;
MK186118;Lepidoptera;Araschnia levana;Araschnia levana;1;Araschnia levana;0.999999711999648;
MK186119;Lepidoptera;Araschnia levana;Araschnia levana;1;Araschnia levana;0.999999711999648;
MK186122;Lepidoptera;Argynnis paphia;Argynnis paphia;1;Argynnis paphia;0.999999991055574;
MK186123;Lepidoptera;Aricia agestis;NA;0.187;Aricia agestis;0.996420870963052;
MK186124;Lepidoptera;Aricia agestis;NA;0.202;Aricia agestis;0.993185236523708;
MK186125;Lepidoptera;Aricia agestis;NA;0.208;Aricia agestis;0.997002229916662;
MK186126;Lepidoptera;Aricia agestis;NA;0.208;Aricia agestis;0.997002229916662;
MK186127;Lepidoptera;Aricia agestis;NA;0.208;Aricia agestis;0.997002229916662;
MK186128;Lepidoptera;Aricia agestis;NA;0.208;Aricia agestis;0.997002229916662;
MK186129;Lepidoptera;Aricia agestis;NA;0.121;Aricia agestis;0.991407746092031;
MK186130;Lepidoptera;Aricia artaxerxes;Aricia artaxerxes;0.041;Aricia artaxerxes;0.99874233829851;
MK186131;Lepidoptera;Aricia artaxerxes;NA;0.397;Aricia artaxerxes;0.999459966837446;
MK186132;Lepidoptera;Aricia artaxerxes;NA;0.317;Aricia artaxerxes;0.999237960024884;
MK186133;Lepidoptera;Aricia artaxerxes;Plebejus argus;1;Aricia artaxerxes;0.999753293634469;
MK186134;Lepidoptera;Aricia artaxerxes;NA;0.5;Aricia artaxerxes;0.999632282267446;
MK186135;Lepidoptera;Aricia artaxerxes;Aricia artaxerxes;0.041;Aricia artaxerxes;0.99874233829851;
MK186136;Lepidoptera;Aricia artaxerxes;NA;0.002;Aricia artaxerxes;0.998931573225529;
MK186137;Lepidoptera;Aricia artaxerxes;NA;0.384;Aricia artaxerxes;0.999542610958834;
MK186138;Lepidoptera;Aricia artaxerxes;Plebejus argus;1;Aricia artaxerxes;0.999753293634469;
MK186139;Lepidoptera;Aricia artaxerxes;NA;0.397;Aricia artaxerxes;0.999459966837446;
MK186140;Lepidoptera;Aricia artaxerxes;Plebejus argus;1;Aricia artaxerxes;0.999753293634469;
MK186141;Lepidoptera;Aricia artaxerxes;Plebejus argus;1;Aricia artaxerxes;0.999682677463404;
MK186146;Lepidoptera;Boloria dia;Boloria dia;1;Boloria dia;0.999999999977035;
MK186147;Lepidoptera;Boloria dia;Boloria dia;1;Boloria dia;0.999999999972431;

MK186148;Lepidoptera;Boloria dia;Boloria dia;1;Boloria dia;0.9999999999865;
MK186149;Lepidoptera;Boloria dia;Boloria dia;1;Boloria dia;0.999999999977746;
MK186150;Lepidoptera;Boloria dia;Boloria dia;1;Boloria dia;0.999999999943299;
MK186151;Lepidoptera;Boloria euphrosyne;Boloria euphrosyne;0.004;Boloria euphrosyne;0.999985940608651;
MK186152;Lepidoptera;Boloria euphrosyne;Boloria euphrosyne;1;Boloria euphrosyne;0.999974952837756;
MK186153;Lepidoptera;Boloria euphrosyne;Boloria euphrosyne;0.001;Boloria euphrosyne;0.999955433068631;
MK186154;Lepidoptera;Boloria euphrosyne;Boloria euphrosyne;0.999;Boloria euphrosyne;0.999983438110448;
MK186155;Lepidoptera;Boloria euphrosyne;Boloria euphrosyne;0.005;Boloria euphrosyne;0.99998578072882;
MK186156;Lepidoptera;Boloria euphrosyne;Boloria euphrosyne;NA;Boloria euphrosyne;0.999987404178288;
MK186157;Lepidoptera;Boloria euphrosyne;Boloria euphrosyne;0.005;Boloria euphrosyne;0.99998578072882;
MK186161;Lepidoptera;Boloria pales;Boloria pales;0.001;Boloria pales;0.998878070533085;
MK186167;Lepidoptera;Boloria thore;Boloria thore;1;Boloria thore;0.999999998965706;
MK186168;Lepidoptera;Boloria thore;Boloria thore;1;Boloria thore;0.9999999984596;
MK186169;Lepidoptera;Boloria thore;Boloria thore;1;Boloria thore;0.99999999936594;
MK186170;Lepidoptera;Boloria thore;Boloria thore;1;Boloria thore;0.9999999984596;
MK186171;Lepidoptera;Boloria titania;Boloria titania;0.999;Boloria titania;0.999841641491547;
MK186172;Lepidoptera;Boloria titania;Boloria titania;0.999;Boloria titania;0.999841641491547;
MK186173;Lepidoptera;Boloria titania;Boloria titania;0.999;Boloria titania;0.999862683205575;
MK186174;Lepidoptera;Boloria titania;Boloria titania;0.998;Boloria titania;0.999884662749941;
MK186175;Lepidoptera;Brenthis daphne;Brenthis daphne;1;Brenthis daphne;0.998484609842471;
MK186176;Lepidoptera;Brenthis daphne;Brenthis daphne;0.003;Brenthis daphne;0.996304540060505;
MK186177;Lepidoptera;Brenthis daphne;Brenthis daphne;1;Brenthis daphne;0.998355654244706;
MK186178;Lepidoptera;Brenthis daphne;Brenthis daphne;1;Brenthis daphne;0.9972710834386;
MK186179;Lepidoptera;Brenthis ino;Brenthis ino;0.013;Brenthis ino;0.963059327700735;
MK186180;Lepidoptera;Brenthis ino;Brenthis ino;1;Brenthis ino;0.998333833872941;
MK186181;Lepidoptera;Brenthis ino;Brenthis ino;1;Brenthis ino;0.995925740941182;
MK186182;Lepidoptera;Brenthis ino;Brenthis ino;0.001;Brenthis ino;0.997846586011011;
MK186183;Lepidoptera;Brenthis ino;Brenthis ino;0.005;Brenthis ino;0.977015191874501;
MK186184;Lepidoptera;Brenthis ino;Brenthis ino;0.001;Brenthis ino;0.998453563477944;
MK186185;Lepidoptera;Brintesia circe;Brintesia circe;1;Brintesia circe;0.999999800536918;
MK186186;Lepidoptera;Brintesia circe;Brintesia circe;1;Brintesia circe;0.999999787064462;
MK186187;Lepidoptera;Brintesia circe;Brintesia circe;1;Brintesia circe;0.999999789669851;
MK186190;Lepidoptera;Callophrys rubi;NA;0.008;Callophrys rubi;0.972346549947987;
MK186191;Lepidoptera;Callophrys rubi;NA;0.041;Callophrys rubi;0.998365812030464;
MK186192;Lepidoptera;Callophrys rubi;NA;0.006;Callophrys rubi;0.997005454794404;
MK186193;Lepidoptera;Callophrys rubi;NA;0.001;Callophrys rubi;0.995967322192437;
MK186194;Lepidoptera;Carcharodus alceae;Carcharodus alceae;0.998;Carcharodus alceae;0.999999989147739;
MK186195;Lepidoptera;Carcharodus alceae;Carcharodus alceae;0.001;Carcharodus alceae;0.999999997919389;
MK186196;Lepidoptera;Carcharodus alceae;Carcharodus alceae;1;Carcharodus alceae;0.999999998403723;
MK186199;Lepidoptera;Carcharodus lavatherae;NA;0.055;Carcharodus lavatherae;0.997031739414004;
MK186200;Lepidoptera;Carcharodus lavatherae;NA;0.185;Carcharodus lavatherae;0.996619545330546;
MK186201;Lepidoptera;Carterocephalus palaemon;NA;0.047;Carterocephalus palaemon;0.723411819709686;
MK186202;Lepidoptera;Carterocephalus palaemon;Carterocephalus palaemon;0.017;Carterocephalus palaemon;0.868047837100633;
MK186203;Lepidoptera;Carterocephalus palaemon;NA;0.038;Carterocephalus palaemon;0.750224854704037;
MK186204;Lepidoptera;Celastrina argiolus;Celastrina argiolus;1;Celastrina argiolus;0.999508885935436;
MK186205;Lepidoptera;Celastrina argiolus;Celastrina argiolus;1;Celastrina argiolus;0.999508885935436;
MK186206;Lepidoptera;Celastrina argiolus;Celastrina argiolus;1;Celastrina argiolus;0.999508885935436;
MK186207;Lepidoptera;Chazara briseis;Chazara briseis;0.007;Chazara briseis;0.999938841544398;
MK186208;Lepidoptera;Coenonympha arcania;NA;0.077;Coenonympha arcania;0.986212060422033;
MK186209;Lepidoptera;Coenonympha arcania;Coenonympha arcania;0.012;Coenonympha arcania;0.99564845864458;
MK186210;Lepidoptera;Coenonympha arcania;NA;0.156;Coenonympha arcania;0.94930496508595;
MK186211;Lepidoptera;Coenonympha arcania;NA;0.066;Coenonympha arcania;0.981477171365832;
MK186212;Lepidoptera;Coenonympha arcania;NA;0.187;Coenonympha arcania;0.966116481342013;
MK186213;Lepidoptera;Coenonympha darwiniana;NA;0.01;Coenonympha darwiniana;0.740760258919092;
MK186214;Lepidoptera;Coenonympha darwiniana;NA;0.002;NA;0.999999999999989;
MK186215;Lepidoptera;Coenonympha darwiniana;NA;0.001;NA;0.999999999999997;
MK186216;Lepidoptera;Coenonympha darwiniana;NA;0.001;NA;0.999999999999997;
MK186217;Lepidoptera;Coenonympha darwiniana;NA;0.211;NA;0.999999999999988;
MK186218;Lepidoptera;Coenonympha darwiniana;NA;0.001;NA;0.999999999999997;
MK186219;Lepidoptera;Coenonympha darwiniana;NA;0.001;NA;0.999999999999997;
MK186220;Lepidoptera;Coenonympha darwiniana;NA;NA;NA;0.999999999998872;

MK186221;Lepidoptera;Coenonympha darwiniana;NA;0.001;NA;0.999999999999997;
MK186222;Lepidoptera;Coenonympha darwiniana;NA;0.028;NA;0.999999999999925;
MK186223;Lepidoptera;Coenonympha gardetta;NA;0.004;Coenonympha gardetta;0.733118270257489;
MK186224;Lepidoptera;Coenonympha gardetta;NA;0.002;NA;0.999999999999969;
MK186225;Lepidoptera;Coenonympha gardetta;NA;0.001;NA;0.999999999999919;
MK186226;Lepidoptera;Coenonympha gardetta;NA;0.002;NA;0.99999999999937;
MK186227;Lepidoptera;Coenonympha gardetta;Coenonympha gardetta;0.016;NA;0.999999999999819;
MK186228;Lepidoptera;Coenonympha gardetta;NA;0.007;NA;0.999999999999982;
MK186229;Lepidoptera;Coenonympha glycerion;NA;0.243;Coenonympha glycerion;0.999998186312577;
MK186230;Lepidoptera;Coenonympha pamphilus;Coenonympha pamphilus;1;Coenonympha pamphilus;0.999999999999687;
MK186231;Lepidoptera;Coenonympha pamphilus;Coenonympha pamphilus;1;Coenonympha pamphilus;0.99999999999784;
MK186232;Lepidoptera;Coenonympha pamphilus;Coenonympha pamphilus;1;Coenonympha pamphilus;0.999999999998892;
MK186233;Lepidoptera;Coenonympha pamphilus;Coenonympha pamphilus;1;Coenonympha pamphilus;0.999999999987693;
MK186234;Lepidoptera;Coenonympha pamphilus;Coenonympha pamphilus;1;Coenonympha pamphilus;0.999999999988404;
MK186241;Lepidoptera;Colias alfacariensis;Colias alfacariensis;1;Colias alfacariensis;0.999917135509572;
MK186242;Lepidoptera;Colias alfacariensis;Colias alfacariensis;1;Colias alfacariensis;0.999917135509572;
MK186243;Lepidoptera;Colias alfacariensis;Colias alfacariensis;1;Colias alfacariensis;0.999807197845331;
MK186244;Lepidoptera;Colias alfacariensis;Colias alfacariensis;1;Colias alfacariensis;0.999810175580931;
MK186245;Lepidoptera;Colias alfacariensis;Colias alfacariensis;1;Colias alfacariensis;0.999917135509572;
MK186246;Lepidoptera;Colias croceus;NA;NA;NA;0.999999999999422;
MK186247;Lepidoptera;Colias croceus;NA;NA;NA;0.999999999999755;
MK186248;Lepidoptera;Colias croceus;NA;0.047;NA;0.999999999998457;
MK186249;Lepidoptera;Colias croceus;NA;NA;NA;0.999999999999755;
MK186250;Lepidoptera;Colias croceus;NA;NA;NA;0.999999999999755;
MK186251;Lepidoptera;Colias croceus;NA;NA;NA;0.999999999999755;
MK186252;Lepidoptera;Colias croceus;NA;NA;NA;0.999999999999755;
MK186253;Lepidoptera;Colias hyale;Colias hyale;0.012;Colias hyale;0.999995743525396;
MK186254;Lepidoptera;Colias hyale;Colias hyale;0.013;Colias hyale;0.999998205688247;
MK186255;Lepidoptera;Colias hyale;Colias hyale;0.009;Colias hyale;0.999996135088304;
MK186256;Lepidoptera;Colias hyale;Colias hyale;0.011;Colias hyale;0.999998744338079;
MK186257;Lepidoptera;Colias hyale;Colias hyale;0.02;Colias hyale;0.999998277106761;
MK186258;Lepidoptera;Colias hyale;Colias hyale;0.013;Colias hyale;0.999998205688247;
MK186259;Lepidoptera;Colias palaeno;NA;0.001;NA;0.999999999983139;
MK186260;Lepidoptera;Colias palaeno;NA;0.001;NA;0.999999999983139;
MK186261;Lepidoptera;Colias phicomone;NA;0.001;Colias phicomone;0.986499654897221;
MK186262;Lepidoptera;Colias phicomone;NA;0.001;Colias phicomone;0.986499654897221;
MK186263;Lepidoptera;Colias phicomone;NA;0.001;Colias phicomone;0.986499654897221;
MK186264;Lepidoptera;Colias phicomone;NA;NA;Colias phicomone;0.985642572028338;
MK186265;Lepidoptera;Cupido alcetas;NA;0.081;Cupido alcetas;0.896139503165777;
MK186266;Lepidoptera;Cupido alcetas;NA;0.202;Cupido alcetas;0.833431942737901;
MK186267;Lepidoptera;Cupido argiades;NA;0.486;NA;0.999999999981462;
MK186268;Lepidoptera;Cupido argiades;NA;NA;NA;0.99999999978974;
MK186269;Lepidoptera;Cupido minimus;NA;0.003;Cupido minimus;0.999692883885763;
MK186270;Lepidoptera;Cupido minimus;NA;0.006;Cupido minimus;0.999784309190108;
MK186271;Lepidoptera;Cupido minimus;NA;0.006;Cupido minimus;0.999784309190108;
MK186272;Lepidoptera;Cupido minimus;NA;0.004;Cupido minimus;0.999836565963549;
MK186273;Lepidoptera;Cupido minimus;NA;0.006;Cupido minimus;0.999784309190108;
MK186274;Lepidoptera;Cupido osiris;Cupido osiris;1;Cupido osiris;0.999999358185626;
MK186296;Lepidoptera;Erebia christi;Erebia christi;1;Erebia christi;0.999999999952564;
MK186297;Lepidoptera;Erebia epiphron;Erebia epiphron;NA;Erebia epiphron;0.996531582765566;
MK186298;Lepidoptera;Erebia epiphron;Erebia epiphron;NA;Erebia epiphron;0.998174897162984;
MK186299;Lepidoptera;Erebia epiphron;NA;NA;Erebia epiphron;0.992450795577526;
MK186300;Lepidoptera;Erebia epiphron;Erebia epiphron;NA;Erebia epiphron;0.986636093736547;
MK186301;Lepidoptera;Erebia eriphyle;Erebia eriphyle;1;Erebia eriphyle;0.999999987544129;
MK186302;Lepidoptera;Erebia eriphyle;Erebia eriphyle;1;Erebia eriphyle;0.999999987544129;
MK186303;Lepidoptera;Erebia eriphyle;Erebia eriphyle;1;Erebia eriphyle;0.999999990333691;
MK186304;Lepidoptera;Erebia eriphyle;Erebia eriphyle;1;Erebia eriphyle;0.999999987544129;
MK186305;Lepidoptera;Erebia eriphyle;Erebia eriphyle;1;Erebia eriphyle;0.999999920194486;
MK186306;Lepidoptera;Erebia eriphyle;Erebia eriphyle;1;Erebia eriphyle;0.999999990333691;
MK186307;Lepidoptera;Erebia euryale;NA;NA;Erebia euryale;0.964425776567244;
MK186308;Lepidoptera;Erebia euryale;NA;NA;Erebia euryale;0.954958114812306;
MK186309;Lepidoptera;Erebia euryale;NA;NA;Erebia euryale;0.838371298441091;
MK186310;Lepidoptera;Erebia euryale;NA;NA;Erebia euryale;0.906848875243797;
MK186311;Lepidoptera;Erebia euryale;NA;NA;Erebia euryale;0.940627137105066;
MK186323;Lepidoptera;Erebia manto;Erebia manto;0.999;Erebia manto;0.999992810437002;
MK186324;Lepidoptera;Erebia manto;NA;0.126;Erebia manto;0.993934190700102;
MK186325;Lepidoptera;Erebia manto;Erebia manto;1;Erebia manto;0.999979589980411;
MK186326;Lepidoptera;Erebia manto;NA;0.183;Erebia manto;0.996820734645775;

MK186327;Lepidoptera;Erebia medusa;NA;0.011;Erebia medusa;0.987418637428601;
MK186328;Lepidoptera;Erebia medusa;NA;0.022;Erebia medusa;0.983487707416184;
MK186329;Lepidoptera;Erebia medusa;NA;0.002;Erebia medusa;0.98750185515635;
MK186330;Lepidoptera;Erebia medusa;NA;0.012;Erebia medusa;0.993036538842292;
MK186331;Lepidoptera;Erebia melampus;Erebia melampus;0.005;Erebia melampus;0.966311213048524;
MK186332;Lepidoptera;Erebia melampus;Erebia melampus;NA;Erebia melampus;0.963807699896503;
MK186333;Lepidoptera;Erebia melampus;Erebia melampus;0.007;Erebia melampus;0.966244394936425;
MK186334;Lepidoptera;Erebia meolans;Erebia meolans;0.012;Erebia meolans;0.998938281528579;
MK186335;Lepidoptera;Erebia meolans;NA;NA;Erebia meolans;0.999592058097425;
MK186336;Lepidoptera;Erebia meolans;Erebia meolans;NA;Erebia meolans;0.999801286702286;
MK186337;Lepidoptera;Erebia meolans;NA;NA;Erebia meolans;0.999592058097425;
MK186338;Lepidoptera;Erebia meolans;NA;NA;Erebia meolans;0.998679248695771;
MK186339;Lepidoptera;Erebia mnestra;Erebia mnestra;0.999;Erebia mnestra;0.999932163532723;
MK186340;Lepidoptera;Erebia mnestra;Erebia mnestra;0.999;Erebia mnestra;0.999932163532723;
MK186341;Lepidoptera;Erebia mnestra;Erebia mnestra;0.999;Erebia mnestra;0.999932163532723;
MK186342;Lepidoptera;Erebia mnestra;Erebia mnestra;0.999;Erebia mnestra;0.999871283181923;
MK186343;Lepidoptera;Erebia montana;Erebia montana;1;Erebia montana;0.999184327703051;
MK186344;Lepidoptera;Erebia montana;Erebia montana;1;Erebia montana;0.999184327703051;
MK186345;Lepidoptera;Erebia montana;Erebia montana;0.999;Erebia montana;0.997920550965226;
MK186346;Lepidoptera;Erebia montana;Erebia montana;1;Erebia montana;0.997858359689857;
MK186347;Lepidoptera;Erebia nivalis;NA;NA;NA;0.999999999999936;
MK186348;Lepidoptera;Erebia oeme;NA;0.27;Erebia oeme;0.999999999731443;
MK186349;Lepidoptera;Erebia oeme;NA;0.147;Erebia oeme;0.999999999929599;
MK186350;Lepidoptera;Erebia oeme;NA;0.179;Erebia oeme;0.999999999780613;
MK186351;Lepidoptera;Erebia pandrose;Erebia pandrose;0.019;Erebia pandrose;0.979041351081006;
MK186352;Lepidoptera;Erebia pandrose;Erebia pandrose;0.024;Erebia pandrose;0.990073867864573;
MK186353;Lepidoptera;Erebia pandrose;Erebia pandrose;0.007;Erebia pandrose;0.986598859542406;
MK186354;Lepidoptera;Erebia pandrose;Erebia pandrose;0.007;Erebia pandrose;0.986598859542406;
MK186355;Lepidoptera;Erebia pandrose;Erebia pandrose;0.024;Erebia pandrose;0.975070318718481;
MK186356;Lepidoptera;Erebia pharte;Erebia pharte;1;Erebia pharte;0.999999995722192;
MK186357;Lepidoptera;Erebia pharte;Erebia pharte;1;Erebia pharte;0.999999989915807;
MK186358;Lepidoptera;Erebia pharte;Erebia pharte;1;Erebia pharte;0.999999995722192;
MK186359;Lepidoptera;Erebia pluto;Erebia pluto;1;Erebia pluto;0.999999924879208;
MK186360;Lepidoptera;Erebia pluto;Erebia pluto;1;Erebia pluto;0.999999769636188;
MK186361;Lepidoptera;Erebia pronoe;Erebia pronoe;1;Erebia pronoe;0.999681223232352;
MK186362;Lepidoptera;Erebia pronoe;Erebia pronoe;0.999;Erebia pronoe;0.999752694597812;
MK186363;Lepidoptera;Erebia pronoe;Erebia pronoe;1;Erebia pronoe;0.999455067091311;
MK186364;Lepidoptera;Erebia pronoe;NA;NA;Erebia pronoe;0.741393290698191;
MK186365;Lepidoptera;Erebia pronoe;Erebia pronoe;NA;Erebia pronoe;0.910578235158016;
MK186366;Lepidoptera;Erebia styx;NA;0.062;Erebia styx;0.902168179723329;
MK186369;Lepidoptera;Erebia triaria;NA;0.31;NA;0.999999999999988;
MK186370;Lepidoptera;Erebia triaria;NA;0.315;NA;100.000.000.000.001;
MK186371;Lepidoptera;Erebia tyndarus;NA;NA;NA;0.999999999999899;
MK186372;Lepidoptera;Erebia tyndarus;NA;0.002;Erebia tyndarus;0.841879935623522;
MK186373;Lepidoptera;Erebia tyndarus;NA;NA;NA;0.999999999999746;
MK186374;Lepidoptera;Erebia tyndarus;NA;NA;NA;0.999999999999746;
MK186375;Lepidoptera;Erebia tyndarus;NA;NA;NA;0.999999999999899;
MK186376;Lepidoptera;Erebia tyndarus;NA;NA;NA;0.999999999999937;
MK186377;Lepidoptera;Erebia tyndarus;NA;0.001;NA;0.99999999999996;
MK186384;Lepidoptera;Aricia eumedon;Eumedonia eumedon;0.004;Eumedonia eumedon;0.997113153365715;
MK186385;Lepidoptera;Aricia eumedon;Eumedonia eumedon;1;Eumedonia eumedon;0.995200162930918;
MK186386;Lepidoptera;Aricia eumedon;Eumedonia eumedon;0.013;Eumedonia eumedon;0.996092387827183;
MK186387;Lepidoptera;Euphydryas aurinia;NA;0.055;Euphydryas aurinia;0.995357604297629;
MK186388;Lepidoptera;Euphydryas aurinia;NA;0.17;Euphydryas aurinia;0.925361068752259;
MK186389;Lepidoptera;Euphydryas aurinia;NA;0.303;Euphydryas aurinia;0.992531196347835;
MK186391;Lepidoptera;Euphydryas aurinia;NA;0.303;Euphydryas aurinia;0.992531196347835;
MK186392;Lepidoptera;Euphydryas aurinia;NA;0.312;Euphydryas aurinia;0.977908833370082;
MK186393;Lepidoptera;Euphydryas aurinia;NA;0.303;Euphydryas aurinia;0.992531196347835;
MK186394;Lepidoptera;Euphydryas cynthia;Euphydryas cynthia;1;Euphydryas
cynthia;0.99999999887271;
MK186395;Lepidoptera;Euphydryas cynthia;Euphydryas cynthia;1;Euphydryas
cynthia;0.999999999868578;
MK186396;Lepidoptera;Euphydryas cynthia;Euphydryas cynthia;1;Euphydryas
cynthia;0.999999999751026;
MK186397;Lepidoptera;Euphydryas cynthia;Euphydryas cynthia;1;Euphydryas
cynthia;0.999999999591722;
MK186398;Lepidoptera;Euphydryas intermedia;NA;0.309;NA;0.999999999996329;
MK186399;Lepidoptera;Euphydryas intermedia;NA;0.309;NA;0.999999999996329;
MK186400;Lepidoptera;Euphydryas intermedia;NA;0.309;NA;0.999999999996329;
MK186401;Lepidoptera;Fabriciana adippe;NA;0.01;Fabriciana adippe;0.825420270264828;
MK186402;Lepidoptera;Fabriciana adippe;NA;0.009;Fabriciana adippe;0.811434168177779;
MK186403;Lepidoptera;Fabriciana adippe;NA;NA;Fabriciana adippe;0.785717058301081;
MK186404;Lepidoptera;Fabriciana adippe;NA;NA;Fabriciana adippe;0.846733684548914;
MK186405;Lepidoptera;Fabriciana adippe;NA;NA;NA;0.999999999986551;
MK186406;Lepidoptera;Fabriciana adippe;NA;0.009;Fabriciana adippe;0.811434168177779;
MK186407;Lepidoptera;Fabriciana niobe;NA;0.003;Fabriciana niobe;0.99205397997248;
MK186408;Lepidoptera;Fabriciana niobe;NA;0.003;Fabriciana niobe;0.990304513022067;
MK186409;Lepidoptera;Fabriciana niobe;NA;0.003;Fabriciana niobe;0.99205397997248;

MK186410;Lepidoptera;Fabriciana niobe;NA;0.064;Fabriciana niobe;0.989112200722109;
MK186411;Lepidoptera;Fabriciana niobe;NA;0.001;Fabriciana niobe;0.984432102210124;
MK186412;Lepidoptera;Fabriciana niobe;NA;0.001;Fabriciana niobe;0.987973355744135;
MK186413;Lepidoptera;Favonius quercus;NA;NA;NA;0.999999999997669;
MK186414;Lepidoptera;Favonius quercus;NA;0.477;NA;0.999999999999351;
MK186415;Lepidoptera;Favonius quercus;NA;0.321;NA;0.999999999703664;
MK186416;Lepidoptera;Glaucopsyche alexis;Glaucopsyche alexis;0.001;Glaucopsyche alexis;0.963537137293225;
MK186417;Lepidoptera;Glaucopsyche alexis;NA;0.001;Glaucopsyche alexis;0.914259738757796;
MK186418;Lepidoptera;Gonepteryx rhamni;NA;0.499;Gonepteryx rhamni;0.985333803440189;
MK186419;Lepidoptera;Gonepteryx rhamni;NA;0.467;Gonepteryx rhamni;0.977925311300942;
MK186420;Lepidoptera;Gonepteryx rhamni;NA;0.467;Gonepteryx rhamni;0.977925311300942;
MK186421;Lepidoptera;Gonepteryx rhamni;Gonepteryx rhamni;0.001;Gonepteryx rhamni;0.99981840321782;
MK186422;Lepidoptera;Gonepteryx rhamni;Gonepteryx rhamni;0.006;Gonepteryx rhamni;0.999515928486512;
MK186423;Lepidoptera;Gonepteryx rhamni;NA;0.467;Gonepteryx rhamni;0.977925311300942;
MK186424;Lepidoptera;Hamearis lucina;NA;NA;Hamearis lucina;0.999999999987693;
MK186425;Lepidoptera;Hamearis lucina;NA;NA;Hamearis lucina;0.999999999987693;
MK186426;Lepidoptera;Hamearis lucina;NA;NA;Hamearis lucina;0.999999999987693;
MK186427;Lepidoptera;Hamearis lucina;Hamearis lucina;NA;Hamearis lucina;0.999999999987438;
MK186435;Lepidoptera;Hipparchia fagi;NA;NA;Hipparchia fagi;0.940167961922483;
MK186436;Lepidoptera;Hipparchia fagi;NA;NA;Hipparchia fagi;0.92225238936553;
MK186437;Lepidoptera;Hipparchia fagi;NA;NA;Hipparchia fagi;0.940167961922483;
MK186441;Lepidoptera;Hipparchia semele;NA;0.001;Hipparchia semele;0.745166355974807;
MK186442;Lepidoptera;Hipparchia semele;NA;NA;Hipparchia semele;0.777428011165802;
MK186443;Lepidoptera;Hipparchia statilinus;Hipparchia statilinus;1;Hipparchia statilinus;0.999999999999176;
MK186446;Lepidoptera;Iolana iolas;Iolana iolas;1;Iolana iolas;0.999999205380678;
MK186448;Lepidoptera;Iphiclides podalirius;NA;NA;Iphiclides podalirius;0.928526609839125;
MK186461;Lepidoptera;Lampides boeticus;Lampides boeticus;1;Lampides boeticus;0.999999999199133;
MK186462;Lepidoptera;Lasiommata maera;Lasiommata maera;1;Lasiommata maera;0.999996837286971;
MK186463;Lepidoptera;Lasiommata maera;Lasiommata maera;1;Lasiommata maera;0.999997390650514;
MK186464;Lepidoptera;Lasiommata maera;Lasiommata maera;0.001;Lasiommata maera;0.999998440187903;
MK186465;Lepidoptera;Lasiommata maera;Lasiommata maera;1;Lasiommata maera;0.999998364044702;
MK186466;Lepidoptera;Lasiommata maera;Lasiommata maera;1;Lasiommata maera;0.999996837286971;
MK186467;Lepidoptera;Lasiommata megera;Lasiommata megera;1;Lasiommata megera;0.99994124434038;
MK186468;Lepidoptera;Lasiommata megera;Lasiommata megera;1;Lasiommata megera;0.99988037861634;
MK186469;Lepidoptera;Lasiommata megera;Lasiommata megera;1;Lasiommata megera;0.999944012465835;
MK186470;Lepidoptera;Lasiommata megera;Lasiommata megera;1;Lasiommata megera;0.999818946947674;
MK186471;Lepidoptera;Lasiommata megera;Lasiommata megera;1;Lasiommata megera;0.999944012465835;
MK186485;Lepidoptera;Limenitis camilla;NA;0.056;Limenitis camilla;0.999922140605573;
MK186486;Lepidoptera;Limenitis camilla;Limenitis camilla;1;Limenitis camilla;0.999968161744423;
MK186487;Lepidoptera;Limenitis camilla;NA;0.031;Limenitis camilla;0.999976358578457;
MK186488;Lepidoptera;Limenitis camilla;NA;0.213;Limenitis camilla;0.999907155361037;
MK186489;Lepidoptera;Limenitis camilla;NA;NA;Limenitis camilla;0.999903286812386;
MK186498;Lepidoptera;Lycaena dispar;Lycaena dispar;1;Lycaena dispar;0.999999997276802;
MK186499;Lepidoptera;Helleia helle;Lycaena helle;1;Lycaena helle;0.999999985376519;
MK186500;Lepidoptera;Helleia helle;Lycaena helle;1;Lycaena helle;0.999999993429498;
MK186502;Lepidoptera;Lycaena hippothoe;Lycaena hippothoe;NA;Lycaena hippothoe;0.978273581027692;
MK186504;Lepidoptera;Lycaena hippothoe;NA;NA;Lycaena hippothoe;0.933880050432329;
MK186505;Lepidoptera;Lycaena hippothoe;NA;NA;Lycaena hippothoe;0.794808795821834;
MK186506;Lepidoptera;Lycaena hippothoe;NA;NA;Lycaena hippothoe;0.874227413846883;
MK186507;Lepidoptera;Lycaena hippothoe;NA;NA;Lycaena hippothoe;0.869388816094577;
MK186508;Lepidoptera;Lycaena hippothoe;NA;NA;Lycaena hippothoe;0.933880050432329;
MK186510;Lepidoptera;Lycaena phlaeas;Lycaena phlaeas;1;Lycaena phlaeas;0.999999974578884;
MK186511;Lepidoptera;Lycaena phlaeas;Lycaena phlaeas;1;Lycaena phlaeas;0.999999963570418;
MK186512;Lepidoptera;Lycaena phlaeas;Lycaena phlaeas;1;Lycaena phlaeas;0.999999990854633;
MK186513;Lepidoptera;Lycaena tityrus;Lycaena tityrus;1;Lycaena tityrus;0.99999565859004;
MK186514;Lepidoptera;Lycaena tityrus;Lycaena tityrus;1;Lycaena tityrus;0.999995507615411;
MK186515;Lepidoptera;Lycaena tityrus;Lycaena tityrus;1;Lycaena tityrus;0.999995507615411;
MK186516;Lepidoptera;Lycaena tityrus;Lycaena tityrus;1;Lycaena tityrus;0.999995507615411;
MK186517;Lepidoptera;Lycaena tityrus;Lycaena tityrus;1;Lycaena tityrus;0.999997596583785;
MK186518;Lepidoptera;Lycaena tityrus;Lycaena tityrus;1;Lycaena tityrus;0.999998846372518;
MK186519;Lepidoptera;Lycaena tityrus;Lycaena tityrus;1;Lycaena tityrus;0.999996515686308;
MK186520;Lepidoptera;Lycaena tityrus;Lycaena tityrus;1;Lycaena tityrus;0.999998846372518;
MK186521;Lepidoptera;Lycaena tityrus;Lycaena tityrus;1;Lycaena tityrus;0.999996150345644;
MK186522;Lepidoptera;Lycaena tityrus;Lycaena tityrus;1;Lycaena tityrus;0.999991069734676;
MK186523;Lepidoptera;Lycaena virgaureae;NA;0.226;Lycaena virgaureae;0.999331665618954;
MK186524;Lepidoptera;Lycaena virgaureae;NA;0.234;Lycaena virgaureae;0.999568266359117;
MK186527;Lepidoptera;Lysandra coridon;NA;NA;NA;0.997258082133847;
MK186528;Lepidoptera;Lysandra coridon;Lysandra coridon;NA;Lysandra coridon;0.927795013241946;
MK186529;Lepidoptera;Lysandra coridon;NA;NA;NA;0.997802687059559;
MK186530;Lepidoptera;Lysandra coridon;NA;NA;NA;0.997802687059559;
MK186531;Lepidoptera;Maniola jurtina;NA;0.005;NA;0.999999999999655;
MK186532;Lepidoptera;Maniola jurtina;NA;0.005;NA;0.999999999999655;
MK186533;Lepidoptera;Maniola jurtina;NA;0.007;NA;0.9999999999991;
MK186534;Lepidoptera;Maniola jurtina;NA;0.005;NA;0.999999999998714;

MK186535;Lepidoptera;Maniola jurtina;NA;0.005;NA;0.999999999999655;
MK186548;Lepidoptera;Melitaea cinxia;Melitaea cinxia;1;Melitaea cinxia;0.999999999999545;
MK186549;Lepidoptera;Melitaea cinxia;Melitaea cinxia;1;Melitaea cinxia;0.999999999999403;
MK186550;Lepidoptera;Melitaea cinxia;Melitaea cinxia;1;Melitaea cinxia;0.999999999999318;
MK186551;Lepidoptera;Melitaea deione;Melitaea deione;1;Melitaea deione;0.999999729994905;
MK186552;Lepidoptera;Melitaea deione;Melitaea deione;1;Melitaea deione;0.999999743683593;
MK186553;Lepidoptera;Melitaea diamina;NA;0.146;Melitaea diamina;0.999999735688767;
MK186554;Lepidoptera;Melitaea diamina;NA;0.177;Melitaea diamina;0.999999983670563;
MK186555;Lepidoptera;Melitaea diamina;NA;0.055;Melitaea diamina;0.999999987179621;
MK186556;Lepidoptera;Melitaea diamina;NA;0.128;Melitaea diamina;0.999999974054277;
MK186557;Lepidoptera;Melitaea diamina;NA;0.157;Melitaea diamina;0.999999996161478;
MK186558;Lepidoptera;Melitaea diamina;NA;0.157;Melitaea diamina;0.999999996161478;
MK186559;Lepidoptera;Melitaea didyma;Melitaea didyma;NA;Melitaea didyma;0.971462863566472;
MK186563;Lepidoptera;Melitaea nevadensis;NA;0.016;Melitaea nevadensis;0.895792250169311;
MK186565;Lepidoptera;Melitaea nevadensis;NA;0.001;NA;0.999999999857824;
MK186566;Lepidoptera;Melitaea nevadensis;NA;0.001;Melitaea nevadensis;0.749786150836048;
MK186567;Lepidoptera;Melitaea nevadensis;NA;NA;Melitaea nevadensis;0.772110120397072;
MK186568;Lepidoptera;Melitaea nevadensis;NA;0.001;NA;0.999999999912652;
MK186569;Lepidoptera;Melitaea nevadensis;NA;0.01;Melitaea nevadensis;0.707519738957255;
MK186570;Lepidoptera;Melitaea nevadensis;NA;0.001;NA;0.999999999953142;
MK186571;Lepidoptera;Melitaea parthenoides;Melitaea parthenoides;1;Melitaea parthenoides;0.999999979372405;
MK186572;Lepidoptera;Melitaea parthenoides;Melitaea parthenoides;1;Melitaea parthenoides;0.999999994653734;
MK186573;Lepidoptera;Melitaea parthenoides;Melitaea parthenoides;1;Melitaea parthenoides;0.999999997226809;
MK186578;Lepidoptera;Melitaea varia;Melitaea varia;1;Melitaea varia;0.999993530099357;
MK186579;Lepidoptera;Melitaea varia;Melitaea varia;1;Melitaea varia;0.999989030408412;
MK186580;Lepidoptera;Melitaea varia;Melitaea varia;1;Melitaea varia;0.999997643578913;
MK186581;Lepidoptera;Minois dryas;NA;0.025;Minois dryas;0.868196387308076;
MK186582;Lepidoptera;Minois dryas;NA;0.022;Minois dryas;0.871610991593088;
MK186583;Lepidoptera;Minois dryas;NA;0.022;Minois dryas;0.871610991593088;
MK186588;Lepidoptera;Nymphalis antiopa;NA;0.229;Nymphalis antiopa;0.999999949546323;
MK186597;Lepidoptera;Ochlodes sylvanus;NA;0.335;Ochlodes sylvanus;0.995423959299584;
MK186598;Lepidoptera;Ochlodes sylvanus;NA;0.304;Ochlodes sylvanus;0.995676248293362;
MK186599;Lepidoptera;Ochlodes sylvanus;NA;NA;Ochlodes sylvanus;0.995530013971128;
MK186600;Lepidoptera;Ochlodes sylvanus;NA;0.304;Ochlodes sylvanus;0.995676248293362;
MK186601;Lepidoptera;Ochlodes sylvanus;NA;0.304;Ochlodes sylvanus;0.995676248293362;
MK186602;Lepidoptera;Ochlodes sylvanus;NA;0.335;Ochlodes sylvanus;0.995423959299584;
MK186603;Lepidoptera;Ochlodes sylvanus;NA;0.304;Ochlodes sylvanus;0.995676248293362;
MK186604;Lepidoptera;Ochlodes sylvanus;NA;0.304;Ochlodes sylvanus;0.995676248293362;
MK186605;Lepidoptera;Ochlodes sylvanus;NA;0.32;Ochlodes sylvanus;0.995541032441178;
MK186607;Lepidoptera;Oeneis glacialis;Oeneis glacialis;NA;Oeneis glacialis;0.987143946313372;
MK186608;Lepidoptera;Papilio machaon;Papilio machaon;NA;Papilio machaon;0.997096642066626;
MK186611;Lepidoptera;Papilio machaon;Papilio machaon;0.003;Papilio machaon;0.993805994878478;
MK186612;Lepidoptera;Pararge aegeria;Pararge aegeria;1;Pararge aegeria;0.99999999981921;
MK186613;Lepidoptera;Pararge aegeria;Pararge aegeria;1;Pararge aegeria;0.999999999842714;
MK186614;Lepidoptera;Pararge aegeria;Pararge aegeria;1;Pararge aegeria;0.999999999634298;
MK186615;Lepidoptera;Pararge aegeria;Pararge aegeria;1;Pararge aegeria;0.999999999403428;
MK186616;Lepidoptera;Pararge aegeria;Pararge aegeria;1;Pararge aegeria;0.999999999919766;
MK186617;Lepidoptera;Pararge aegeria;Pararge aegeria;1;Pararge aegeria;0.999999999919766;
MK186620;Lepidoptera;Parnassius mnemosyne;Parnassius mnemosyne;1;Parnassius mnemosyne;0.999999182879371;
MK186621;Lepidoptera;Parnassius mnemosyne;Parnassius mnemosyne;1;Parnassius mnemosyne;0.999997237058446;
MK186622;Lepidoptera;Parnassius mnemosyne;Parnassius mnemosyne;1;Parnassius mnemosyne;0.999999468357372;
MK186623;Lepidoptera;Parnassius mnemosyne;Parnassius mnemosyne;1;Parnassius mnemosyne;0.999999182879371;
MK186629;Lepidoptera;Phengaris arion;NA;NA;Phengaris arion;0.848593181149039;
MK186630;Lepidoptera;Phengaris arion;NA;0.001;Phengaris arion;0.925858124607094;
MK186631;Lepidoptera;Phengaris arion;NA;0.04;Phengaris arion;0.934893699328482;
MK186632;Lepidoptera;Phengaris arion;NA;0.022;Phengaris arion;0.925772921223381;
MK186633;Lepidoptera;Phengaris arion;NA;0.003;Phengaris arion;0.813559255239592;
MK186634;Lepidoptera;Phengaris arion;NA;0.031;Phengaris arion;0.929945252366353;
MK186655;Lepidoptera;Pieris napi;NA;0.08;Pieris napi;0.823311022092086;
MK186656;Lepidoptera;Pieris napi;NA;0.008;Pieris napi;0.790825746989002;
MK186657;Lepidoptera;Pieris napi;NA;0.008;Pieris napi;0.790825746989002;
MK186660;Lepidoptera;Pieris napi;NA;NA;Pieris napi;0.881345985930259;
MK186661;Lepidoptera;Pieris napi;NA;0.012;Pieris napi;0.830982203598986;
MK186667;Lepidoptera;Plebejus argus;NA;0.04;Plebejus argus;0.988041859046217;
MK186668;Lepidoptera;Plebejus argus;Plebejus argus;0.006;Plebejus argus;0.995231441851159;
MK186669;Lepidoptera;Plebejus argus;NA;0.324;Plebejus argus;0.97124815046492;
MK186670;Lepidoptera;Plebejus argus;Plebejus argus;0.018;Plebejus argus;0.983942157133061;
MK186671;Lepidoptera;Plebejus argyrognomon;NA;0.001;NA;0.999931300450496;
MK186672;Lepidoptera;Plebejus argyrognomon;NA;0.002;Plebejus argyrognomon;0.712868781924731;
MK186673;Lepidoptera;Plebejus argyrognomon;NA;0.004;NA;0.999003523877672;
MK186674;Lepidoptera;Plebejus idas;NA;0.001;NA;0.999417276617746;

MK186675;Lepidoptera;Plebejus idas;NA;0.001;NA;0.999540941919522;
MK186676;Lepidoptera;Plebejus idas;NA;0.001;NA;0.999540941919522;
MK186677;Lepidoptera;Plebejus idas;NA;0.004;NA;0.998898352664053;
MK186678;Lepidoptera;Plebejus idas;NA;0.001;NA;0.999155883818852;
MK186679;Lepidoptera;Plebejus idas;NA;0.001;NA;0.999540941919522;
MK186680;Lepidoptera;Nymphalis c-album;NA;0.218;NA;0.999999260642547;
MK186681;Lepidoptera;Nymphalis c-album;NA;0.077;NA;0.999999576214005;
MK186682;Lepidoptera;Nymphalis c-album;NA;0.074;Polygonia c-album;0.729363089498517;
MK186683;Lepidoptera;Nymphalis c-album;NA;0.218;NA;0.999999260642547;
MK186684;Lepidoptera;Nymphalis c-album;NA;0.218;NA;0.999999260642547;
MK186685;Lepidoptera;Polyommatus amandus;Polyommatus amandus;0.003;Polyommatus amandus;0.999987004283814;
MK186686;Lepidoptera;Polyommatus amandus;Polyommatus amandus;0.004;Polyommatus amandus;0.999964874388038;
MK186687;Lepidoptera;Polyommatus amandus;Polyommatus amandus;0.004;Polyommatus amandus;0.999979888960249;
MK186688;Lepidoptera;Polyommatus damon;Polyommatus damon;1;Polyommatus damon;0.99999990253517;
MK186689;Lepidoptera;Polyommatus damon;Polyommatus damon;1;Polyommatus damon;0.999999948683269;
MK186690;Lepidoptera;Polyommatus damon;Polyommatus damon;1;Polyommatus damon;0.999999976959259;
MK186691;Lepidoptera;Polyommatus daphnis;Polyommatus daphnis;1;Polyommatus daphnis;0.99987322383589;
MK186692;Lepidoptera;Polyommatus daphnis;Polyommatus daphnis;1;Polyommatus daphnis;0.999883549769609;
MK186693;Lepidoptera;Polyommatus dorylas;NA;0.001;Polyommatus dorylas;0.847406218697546;
MK186694;Lepidoptera;Polyommatus dorylas;Polyommatus dorylas;0.009;Polyommatus dorylas;0.991722814510017;
MK186695;Lepidoptera;Polyommatus dorylas;Polyommatus dorylas;1;Polyommatus dorylas;0.997621787619456;
MK186696;Lepidoptera;Polyommatus eros;NA;0.001;Polyommatus eros;0.866010605610589;
MK186697;Lepidoptera;Polyommatus eros;NA;0.001;Polyommatus eros;0.944687707185616;
MK186698;Lepidoptera;Polyommatus eros;NA;0.001;Polyommatus eros;0.944687707185616;
MK186699;Lepidoptera;Polyommatus escheri;Polyommatus escheri;1;Polyommatus escheri;0.99999993340788;
MK186700;Lepidoptera;Polyommatus escheri;Polyommatus escheri;1;Polyommatus escheri;0.999999809757345;
MK186701;Lepidoptera;Polyommatus icarus;NA;0.109;Polyommatus icarus;0.974236533175821;
MK186701;Lepidoptera;Polyommatus icarus;NA;0.109;Polyommatus icarus;0.974236533175821;
MK186702;Lepidoptera;Polyommatus icarus;NA;0.001;Polyommatus icarus;0.991536624780024;
MK186702;Lepidoptera;Polyommatus icarus;NA;0.001;Polyommatus icarus;0.991536624780024;
MK186703;Lepidoptera;Polyommatus icarus;NA;0.001;Polyommatus icarus;0.991536624780024;
MK186703;Lepidoptera;Polyommatus icarus;NA;0.001;Polyommatus icarus;0.991536624780024;
MK186704;Lepidoptera;Polyommatus icarus;NA;0.002;Polyommatus icarus;0.998263177861678;
MK186704;Lepidoptera;Polyommatus icarus;NA;0.002;Polyommatus icarus;0.998263177861678;
MK186705;Lepidoptera;Polyommatus icarus;NA;0.002;Polyommatus icarus;0.979420462678099;
MK186705;Lepidoptera;Polyommatus icarus;NA;0.002;Polyommatus icarus;0.979420462678099;
MK186706;Lepidoptera;Polyommatus icarus;Polyommatus icarus;0.002;Polyommatus icarus;0.974025664619468;
MK186706;Lepidoptera;Polyommatus icarus;Polyommatus icarus;0.002;Polyommatus icarus;0.974025664619468;
MK186712;Lepidoptera;Pontia callidice;Pontia callidice;1;Pontia callidice;0.99999999972826;
MK186713;Lepidoptera;Pontia callidice;Pontia callidice;1;Pontia callidice;0.999999976267276;
MK186720;Lepidoptera;Pyrgus alveus;NA;0.002;NA;0.999999999997773;
MK186721;Lepidoptera;Pyrgus alveus;NA;0.002;NA;0.999999999997773;
MK186722;Lepidoptera;Pyrgus alveus;NA;0.01;NA;0.999999999997905;
MK186723;Lepidoptera;Pyrgus alveus;NA;NA;NA;0.9999999999956;
MK186724;Lepidoptera;Pyrgus andromedae;Pyrgus andromedae;0.001;Pyrgus andromedae;0.998616429582581;
MK186725;Lepidoptera;Pyrgus andromedae;Pyrgus andromedae;1;Pyrgus andromedae;0.999453288342031;
MK186726;Lepidoptera;Pyrgus andromedae;Pyrgus andromedae;0.001;Pyrgus andromedae;0.998616429582581;
MK186727;Lepidoptera;Pyrgus andromedae;Pyrgus andromedae;0.001;Pyrgus andromedae;0.998616429582581;
MK186728;Lepidoptera;Pyrgus armoricanus;Pyrgus armoricanus;1;Pyrgus armoricanus;0.999997212344957;
MK186729;Lepidoptera;Pyrgus armoricanus;Pyrgus armoricanus;1;Pyrgus armoricanus;0.999995923887249;
MK186730;Lepidoptera;Pyrgus armoricanus;Pyrgus armoricanus;1;Pyrgus armoricanus;0.999997571812165;
MK186731;Lepidoptera;Pyrgus armoricanus;Pyrgus armoricanus;1;Pyrgus armoricanus;0.999996398214456;
MK186732;Lepidoptera;Pyrgus armoricanus;Pyrgus armoricanus;1;Pyrgus armoricanus;0.999988520165187;
MK186733;Lepidoptera;Pyrgus armoricanus;Pyrgus armoricanus;1;Pyrgus armoricanus;0.999997498890424;
MK186734;Lepidoptera;Pyrgus armoricanus;Pyrgus armoricanus;1;Pyrgus armoricanus;0.999997212344957;
MK186735;Lepidoptera;Pyrgus cacaliae;Pyrgus cacaliae;0.001;Pyrgus cacaliae;0.998315214300087;
MK186736;Lepidoptera;Pyrgus cacaliae;Pyrgus cacaliae;0.001;Pyrgus cacaliae;0.998315214300087;

MK186737;Lepidoptera;Pyrgus cacaliae;Pyrgus cacaliae;0.001;Pyrgus cacaliae;0.998490079014641;
MK186738;Lepidoptera;Pyrgus cacaliae;Pyrgus cacaliae;0.999;Pyrgus cacaliae;0.994248709401488;
MK186739;Lepidoptera;Pyrgus carlinae;NA;0.061;Pyrgus carlinae;0.853193970604539;
MK186740;Lepidoptera;Pyrgus carlinae;NA;0.096;Pyrgus carlinae;0.7992289744845;
MK186741;Lepidoptera;Pyrgus carthami;Pyrgus carthami;1;Pyrgus carthami;0.999995673756424;
MK186742;Lepidoptera;Pyrgus carthami;Pyrgus carthami;1;Pyrgus carthami;0.999997648181059;
MK186743;Lepidoptera;Pyrgus carthami;Pyrgus carthami;1;Pyrgus carthami;0.999995673756424;
MK186744;Lepidoptera;Pyrgus malvae;Pyrgus malvae;NA;Pyrgus malvae;0.985537009228567;
MK186745;Lepidoptera;Pyrgus malvae;NA;0.331;Pyrgus malvoides;0.843314900290182;
MK186746;Lepidoptera;Pyrgus malvae;NA;0.33;Pyrgus malvoides;0.751261270197844;
MK186747;Lepidoptera;Pyrgus malvoides;NA;0.144;Pyrgus malvoides;0.922277091908561;
MK186748;Lepidoptera;Pyrgus malvoides;NA;0.144;Pyrgus malvoides;0.922277091908561;
MK186749;Lepidoptera;Pyrgus malvoides;NA;0.042;Pyrgus malvoides;0.872793748652385;
MK186750;Lepidoptera;Pyrgus malvoides;NA;0.097;Pyrgus malvoides;0.917770993608559;
MK186751;Lepidoptera;Pyrgus malvoides;NA;0.144;Pyrgus malvoides;0.922277091908561;
MK186752;Lepidoptera;Pyrgus onopordi;Pyrgus onopordi;1;Pyrgus onopordi;0.999989234814272;
MK186753;Lepidoptera;Pyrgus serratulae;Pyrgus serratulae;1;Pyrgus serratulae;0.999999629128025;
MK186754;Lepidoptera;Pyrgus serratulae;Pyrgus serratulae;1;Pyrgus serratulae;0.999999960093448;
MK186755;Lepidoptera;Pyrgus serratulae;Pyrgus serratulae;1;Pyrgus serratulae;0.99999991317404;
MK186756;Lepidoptera;Pyrgus serratulae;Pyrgus serratulae;1;Pyrgus serratulae;0.999999921710614;
MK186757;Lepidoptera;Pyrgus warrenensis;NA;0.001;Pyrgus warrenensis;0.794601956296812;
MK186758;Lepidoptera;Pyrgus warrenensis;NA;NA;NA;0.999999999996119;
MK186759;Lepidoptera;Pyrgus warrenensis;NA;0.002;NA;0.999999999997773;
MK186760;Lepidoptera;Pyrgus warrenensis;NA;NA;NA;0.999999999996255;
MK186761;Lepidoptera;Maniola tithonus;Pyronia tithonus;NA;Pyronia tithonus;1;
MK186762;Lepidoptera;Maniola tithonus;Pyronia tithonus;NA;Pyronia tithonus;0.999999999998636;
MK186764;Lepidoptera;Satyrium acaciae;Satyrium acaciae;1;Satyrium acaciae;0.999999920113882;
MK186765;Lepidoptera;Satyrium ilicis;Satyrium ilicis;1;Satyrium ilicis;0.999999934882398;
MK186766;Lepidoptera;Satyrium ilicis;Satyrium ilicis;1;Satyrium ilicis;0.999999994055429;
MK186767;Lepidoptera;Satyrium pruni;NA;0.085;Satyrium pruni;0.989461621719496;
MK186768;Lepidoptera;Satyrium pruni;NA;0.493;Satyrium pruni;0.984126704218791;
MK186769;Lepidoptera;Satyrium pruni;NA;0.481;Satyrium pruni;0.983627344641564;
MK186770;Lepidoptera;Satyrium spini;Satyrium spini;1;Satyrium spini;0.999999902782098;
MK186771;Lepidoptera;Satyrium spini;Satyrium spini;1;Satyrium spini;0.99999976789894;
MK186772;Lepidoptera;Satyrium spini;Satyrium spini;1;Satyrium spini;0.999999925519435;
MK186773;Lepidoptera;Satyrium w-album;NA;0.057;Satyrium w-album;0.988131882252173;
MK186774;Lepidoptera;Satyrium w-album;NA;0.082;Satyrium w-album;0.98891256429909;
MK186775;Lepidoptera;Satyrium w-album;NA;0.232;Satyrium w-album;0.91465176087597;
MK186776;Lepidoptera;Satyrium w-album;NA;0.082;Satyrium w-album;0.98891256429909;
MK186777;Lepidoptera;Satyrium w-album;NA;0.16;NA;0.999549535946431;
MK186778;Lepidoptera;Satyrus ferula;Satyrus ferula;0.001;Satyrus ferula;0.989556430852749;
MK186779;Lepidoptera;Satyrus ferula;Satyrus ferula;0.001;Satyrus ferula;0.99242731441577;
MK186780;Lepidoptera;Scolitantides orion;Scolitantides orion;1;Scolitantides
orion;0.999999989494057;
MK186781;Lepidoptera;Argynnis aglaja;NA;0.016;Speyeria aglaja;0.817491043916765;
MK186782;Lepidoptera;Argynnis aglaja;NA;0.03;NA;0.764666601197647;
MK186783;Lepidoptera;Argynnis aglaja;NA;0.016;Speyeria aglaja;0.817491043916765;
MK186784;Lepidoptera;Argynnis aglaja;NA;0.002;NA;0.755559854096972;
MK186785;Lepidoptera;Argynnis aglaja;NA;0.016;Speyeria aglaja;0.817491043916765;
MK186786;Lepidoptera;Argynnis aglaja;NA;0.016;Speyeria aglaja;0.817491043916765;
MK186787;Lepidoptera;Argynnis aglaja;NA;NA;Speyeria aglaja;0.782380408332011;
MK186792;Lepidoptera;Thecla betulae;Thecla betulae;1;Thecla betulae;0.999999999973227;
MK186793;Lepidoptera;Thecla betulae;Thecla betulae;1;Thecla betulae;0.999999999972317;
MK186794;Lepidoptera;Thecla betulae;Thecla betulae;1;Thecla betulae;0.999999999798774;
MK186795;Lepidoptera;Thymelicus acteon;Thymelicus acteon;1;Thymelicus acteon;0.999999995291489;
MK186796;Lepidoptera;Thymelicus acteon;Thymelicus acteon;1;Thymelicus acteon;0.99999999104989;
MK186797;Lepidoptera;Thymelicus lineola;Thymelicus lineola;1;Thymelicus
lineola;0.999993424589821;
MK186798;Lepidoptera;Thymelicus lineola;Thymelicus lineola;1;Thymelicus
lineola;0.999975403660303;
MK186799;Lepidoptera;Thymelicus lineola;Thymelicus lineola;1;Thymelicus
lineola;0.999993288936091;
MK186800;Lepidoptera;Thymelicus lineola;Thymelicus lineola;1;Thymelicus
lineola;0.999994236469757;
MK186801;Lepidoptera;Thymelicus lineola;Thymelicus lineola;1;Thymelicus
lineola;0.99997267117451;
MK186802;Lepidoptera;Thymelicus lineola;Thymelicus lineola;1;Thymelicus
lineola;0.999985481636036;
MK186803;Lepidoptera;Thymelicus lineola;Thymelicus lineola;1;Thymelicus
lineola;0.999974214484897;
MK186804;Lepidoptera;Thymelicus sylvestris;NA;0.335;Thymelicus sylvestris;0.999940092520524;
MK186805;Lepidoptera;Thymelicus sylvestris;NA;0.335;Thymelicus sylvestris;0.999940092520524;
MK186806;Lepidoptera;Thymelicus sylvestris;Thymelicus sylvestris;1;Thymelicus
sylvestris;0.999999982378;
MK186807;Lepidoptera;Thymelicus sylvestris;NA;0.304;Thymelicus sylvestris;0.999880347008416;
MK186808;Lepidoptera;Thymelicus sylvestris;NA;0.335;Thymelicus sylvestris;0.999940092520524;
MK186809;Lepidoptera;Thymelicus sylvestris;Thymelicus sylvestris;1;Thymelicus
sylvestris;0.999997781726409;

MK186810;Lepidoptera;Thymelicus sylvestris;Thymelicus sylvestris;1;Thymelicus sylvestris;0.999999985093609;
MK186811;Lepidoptera;Vanessa atalanta;Vanessa atalanta;1;Vanessa atalanta;0.999999999688242;
MK186812;Lepidoptera;Vanessa atalanta;Vanessa atalanta;1;Vanessa atalanta;0.999999999653568;
MK186813;Lepidoptera;Vanessa atalanta;Vanessa atalanta;1;Vanessa atalanta;0.999999999750116;
MK186814;Lepidoptera;Vanessa atalanta;Vanessa atalanta;1;Vanessa atalanta;0.999999997508098;
MK252946;Lepidoptera;Boloria selene;NA;0.115;Boloria selene;0.999999952525941;
MK252947;Lepidoptera;Boloria selene;NA;0.115;Boloria selene;0.999999952525941;
MK252948;Lepidoptera;Boloria selene;NA;0.06;Boloria selene;0.999999789050855;
MK252949;Lepidoptera;Boloria selene;NA;0.053;Boloria selene;0.999999995281456;
MK252950;Lepidoptera;Boloria selene;NA;0.115;Boloria selene;0.999999952525941;
MK252951;Lepidoptera;Boloria selene;NA;0.335;Boloria selene;0.999999975285192;
MK252953;Lepidoptera;Boloria selene;NA;0.103;Boloria selene;0.999999940815089;
MK252954;Lepidoptera;Boloria selene;NA;0.335;Boloria selene;0.999999975285192;
MK252955;Lepidoptera;Boloria selene;NA;0.14;Boloria selene;0.999999985987302;
MK252956;Lepidoptera;Boloria selene;NA;0.14;Boloria selene;0.999999985987302;
MK252957;Lepidoptera;Boloria selene;NA;0.335;Boloria selene;0.999999975285192;
MK252958;Lepidoptera;Boloria selene;NA;0.166;Boloria selene;0.999999961382259;
MK343429;Lepidoptera;Satyrium pruni;NA;0.347;Satyrium pruni;0.991870503237268;
MK455752;Lepidoptera;Agriades orbitulus;NA;0.013;Agriades orbitulus;0.998361127658296;
MK455753;Lepidoptera;Agriades orbitulus;NA;0.002;Agriades orbitulus;0.991706133880976;
MK587253;Lepidoptera;Iphiclides podalirius;NA;0.302;Iphiclides podalirius;0.931082412606632;
MK587254;Lepidoptera;Iphiclides podalirius;NA;0.121;Iphiclides podalirius;0.948523477961333;
MK587255;Lepidoptera;Iphiclides podalirius;NA;0.302;Iphiclides podalirius;0.931082412606632;
MK587256;Lepidoptera;Iphiclides podalirius;NA;0.302;Iphiclides podalirius;0.931082412606632;
MK587260;Lepidoptera;Iphiclides podalirius;NA;0.302;Iphiclides podalirius;0.931082412606632;
MK587261;Lepidoptera;Iphiclides podalirius;NA;0.114;Iphiclides podalirius;0.964718668048293;
MK587263;Lepidoptera;Iphiclides podalirius;NA;NA;Iphiclides podalirius;0.943913710269127;
MK587265;Lepidoptera;Iphiclides podalirius;NA;0.136;Iphiclides podalirius;0.967608413237976;
MK587266;Lepidoptera;Iphiclides podalirius;NA;0.302;Iphiclides podalirius;0.931082412606632;
MK587267;Lepidoptera;Iphiclides podalirius;NA;0.302;Iphiclides podalirius;0.931082412606632;
MK587268;Lepidoptera;Iphiclides podalirius;NA;0.302;Iphiclides podalirius;0.931082412606632;
MK587269;Lepidoptera;Iphiclides podalirius;NA;0.302;Iphiclides podalirius;0.931082412606632;
MK587270;Lepidoptera;Iphiclides podalirius;NA;0.302;Iphiclides podalirius;0.931082412606632;
MK587271;Lepidoptera;Iphiclides podalirius;NA;0.302;Iphiclides podalirius;0.931082412606632;
MK587272;Lepidoptera;Iphiclides podalirius;NA;0.302;Iphiclides podalirius;0.931082412606632;
MK812923;Lepidoptera;Thymelicus sylvestris;NA;0.132;Thymelicus sylvestris;0.999929534827865;
MK812924;Lepidoptera;Thymelicus sylvestris;Thymelicus sylvestris;1;Thymelicus sylvestris;0.999999981568351;
MK812925;Lepidoptera;Thymelicus sylvestris;NA;0.131;Thymelicus sylvestris;0.999961574491035;
MK812926;Lepidoptera;Thymelicus sylvestris;NA;0.324;Thymelicus sylvestris;0.999940778442454;
MK812927;Lepidoptera;Thymelicus sylvestris;Thymelicus sylvestris;1;Thymelicus sylvestris;0.999999981568351;
MK812928;Lepidoptera;Thymelicus sylvestris;NA;0.131;Thymelicus sylvestris;0.999961574491035;
MK812929;Lepidoptera;Thymelicus sylvestris;NA;0.324;Thymelicus sylvestris;0.999940778442454;
MK812931;Lepidoptera;Thymelicus sylvestris;NA;0.324;Thymelicus sylvestris;0.999940778442454;
MK812932;Lepidoptera;Thymelicus sylvestris;Thymelicus sylvestris;1;Thymelicus sylvestris;0.999993577919115;
MK812933;Lepidoptera;Thymelicus sylvestris;NA;0.131;Thymelicus sylvestris;0.999961574491035;
MK812934;Lepidoptera;Thymelicus lineola;Thymelicus lineola;1;Thymelicus lineola;0.999997156464765;
MK812935;Lepidoptera;Thymelicus sylvestris;NA;0.324;Thymelicus sylvestris;0.999940778442454;
MK812936;Lepidoptera;Thymelicus sylvestris;NA;0.324;Thymelicus sylvestris;0.999940778442454;
MK812937;Lepidoptera;Thymelicus sylvestris;Thymelicus sylvestris;1;Thymelicus sylvestris;0.999999985935205;
MK812938;Lepidoptera;Thymelicus sylvestris;NA;0.324;Thymelicus sylvestris;0.999940778442454;
MK812939;Lepidoptera;Thymelicus sylvestris;NA;0.14;Thymelicus sylvestris;0.999963443881537;
MK812940;Lepidoptera;Thymelicus sylvestris;Thymelicus sylvestris;1;Thymelicus sylvestris;0.99999876153052;
MK812941;Lepidoptera;Thymelicus sylvestris;Thymelicus sylvestris;1;Thymelicus sylvestris;0.999999981568351;
MK812942;Lepidoptera;Thymelicus sylvestris;NA;0.324;Thymelicus sylvestris;0.999940778442454;
MK812943;Lepidoptera;Thymelicus sylvestris;Thymelicus sylvestris;1;Thymelicus sylvestris;0.999999985935205;
MK812944;Lepidoptera;Thymelicus sylvestris;Thymelicus sylvestris;1;Thymelicus sylvestris;0.99999876153052;
MK812945;Lepidoptera;Thymelicus sylvestris;Thymelicus sylvestris;1;Thymelicus sylvestris;0.999999985935205;
MK812946;Lepidoptera;Thymelicus sylvestris;Thymelicus sylvestris;1;Thymelicus sylvestris;0.999999985935205;
MK812948;Lepidoptera;Thymelicus sylvestris;NA;0.324;Thymelicus sylvestris;0.999940778442454;
MK812949;Lepidoptera;Thymelicus sylvestris;Thymelicus sylvestris;1;Thymelicus sylvestris;0.999999979197469;
MK812950;Lepidoptera;Thymelicus sylvestris;NA;0.324;Thymelicus sylvestris;0.999940778442454;
MK812951;Lepidoptera;Thymelicus sylvestris;NA;0.324;Thymelicus sylvestris;0.999940778442454;
MK812952;Lepidoptera;Thymelicus acteon;Thymelicus acteon;1;Thymelicus acteon;0.99999999752248;
MK812953;Lepidoptera;Thymelicus sylvestris;Thymelicus sylvestris;1;Thymelicus sylvestris;0.999999990634535;

MK812954;Lepidoptera;Thymelicus sylvestris;NA;0.131;Thymelicus sylvestris;0.999961574491035;
MK812955;Lepidoptera;Thymelicus sylvestris;NA;0.004;Thymelicus sylvestris;0.998308003514278;
MK812956;Lepidoptera;Thymelicus sylvestris;NA;0.004;Thymelicus sylvestris;0.998308003514278;
MK812958;Lepidoptera;Thymelicus sylvestris;Thymelicus sylvestris;1;Thymelicus sylvestris;0.999999981568351;
MK812959;Lepidoptera;Thymelicus sylvestris;Thymelicus sylvestris;1;Thymelicus sylvestris;0.999999981568351;
MK812960;Lepidoptera;Thymelicus sylvestris;NA;0.324;Thymelicus sylvestris;0.999940778442454;
MK812961;Lepidoptera;Thymelicus lineola;Thymelicus lineola;1;Thymelicus lineola;0.999971535093703;
MK812962;Lepidoptera;Thymelicus sylvestris;NA;0.004;Thymelicus sylvestris;0.998308003514278;
MK812963;Lepidoptera;Thymelicus sylvestris;Thymelicus sylvestris;1;Thymelicus sylvestris;0.999999981568351;
MK812964;Lepidoptera;Thymelicus sylvestris;Thymelicus sylvestris;1;Thymelicus sylvestris;0.999998807745442;
MK812965;Lepidoptera;Thymelicus sylvestris;NA;0.324;Thymelicus sylvestris;0.999940778442454;
MK812966;Lepidoptera;Thymelicus sylvestris;Thymelicus sylvestris;1;Thymelicus sylvestris;0.999999985935205;
MK812967;Lepidoptera;Thymelicus sylvestris;NA;0.324;Thymelicus sylvestris;0.999940778442454;
MK924101;Lepidoptera;Aporia crataegi;NA;NA;Aporia crataegi;0.999996781126733;
MK924102;Lepidoptera;Aporia crataegi;NA;NA;Aporia crataegi;0.999996781126733;
MK924103;Lepidoptera;Aporia crataegi;NA;NA;Aporia crataegi;0.999996781126733;
MK924104;Lepidoptera;Aporia crataegi;NA;NA;Aporia crataegi;0.999996781126733;
MK924105;Lepidoptera;Aporia crataegi;NA;NA;Aporia crataegi;0.999997351946131;
MK924106;Lepidoptera;Aporia crataegi;NA;NA;Aporia crataegi;0.999996781126733;
MK924107;Lepidoptera;Aporia crataegi;NA;NA;Aporia crataegi;0.999996781126733;
MK924108;Lepidoptera;Aporia crataegi;NA;NA;Aporia crataegi;0.999998459680561;
MK924109;Lepidoptera;Aporia crataegi;NA;NA;Aporia crataegi;0.999996781126733;
MK924110;Lepidoptera;Aporia crataegi;NA;NA;Aporia crataegi;0.999996781126733;
MK924111;Lepidoptera;Aporia crataegi;NA;NA;Aporia crataegi;0.999996781126733;
MK924112;Lepidoptera;Aporia crataegi;NA;NA;Aporia crataegi;0.999998459680561;
MK924113;Lepidoptera;Aporia crataegi;NA;NA;Aporia crataegi;0.999996781126733;
MK924114;Lepidoptera;Aporia crataegi;NA;NA;Aporia crataegi;0.999996781126733;
MK924115;Lepidoptera;Aporia crataegi;NA;NA;Aporia crataegi;0.999997351946131;
MK924116;Lepidoptera;Aporia crataegi;NA;NA;Aporia crataegi;0.999996781126733;
MK924117;Lepidoptera;Aporia crataegi;NA;NA;Aporia crataegi;0.999996781126733;
MK924118;Lepidoptera;Aporia crataegi;NA;NA;Aporia crataegi;0.999996781126733;
MK924119;Lepidoptera;Aporia crataegi;NA;NA;Aporia crataegi;0.999996781126733;
MK924120;Lepidoptera;Aporia crataegi;NA;NA;Aporia crataegi;0.999996781126733;
MN107384;Lepidoptera;Aricia agestis;Aricia agestis;0.001;Aricia agestis;0.93433387200336;
MN107385;Lepidoptera;Aricia agestis;Aricia agestis;0.001;Aricia agestis;0.983169453027443;
MN107386;Lepidoptera;Aricia agestis;NA;NA;Aricia agestis;0.995003792644976;
MN107387;Lepidoptera;Aricia agestis;Aricia agestis;0.02;Aricia agestis;0.997360197036774;
MN107388;Lepidoptera;Aricia agestis;NA;0.026;Aricia agestis;0.998730844602412;
MN107389;Lepidoptera;Aricia agestis;NA;NA;Aricia agestis;0.997354154853625;
MN107390;Lepidoptera;Aricia agestis;NA;0.001;Aricia agestis;0.944618390939925;
MN107391;Lepidoptera;Aricia agestis;NA;0.001;Aricia agestis;0.987521320091523;
MN107392;Lepidoptera;Aricia agestis;NA;NA;Aricia agestis;0.997764569470653;
MN107393;Lepidoptera;Aricia agestis;NA;0.231;Aricia agestis;0.987554269931849;
MN107394;Lepidoptera;Aricia agestis;NA;0.231;Aricia agestis;0.987554269931849;
MN107395;Lepidoptera;Aricia agestis;NA;0.231;Aricia agestis;0.987554269931849;
MN107396;Lepidoptera;Aricia agestis;NA;0.001;Aricia agestis;0.908942032144794;
MN107397;Lepidoptera;Aricia agestis;NA;NA;Aricia agestis;0.995795194058403;
MN107398;Lepidoptera;Aricia artaxerxes;Plebejus argus;1;Aricia artaxerxes;0.99967524337741;
MN107399;Lepidoptera;Aricia artaxerxes;Plebejus argus;1;Aricia artaxerxes;0.999598132990806;
MN107400;Lepidoptera;Aricia artaxerxes;Plebejus argus;1;Aricia artaxerxes;0.999619916208509;
MN107401;Lepidoptera;Aricia artaxerxes;Plebejus argus;1;Aricia artaxerxes;0.999598132990806;
MN107402;Lepidoptera;Aricia artaxerxes;Plebejus argus;1;Aricia artaxerxes;0.999639973715367;
MN107403;Lepidoptera;Aricia artaxerxes;Plebejus argus;0.001;Aricia artaxerxes;0.999718461525682;
MN107404;Lepidoptera;Aricia artaxerxes;Plebejus argus;1;Aricia artaxerxes;0.99976599124702;
MN107405;Lepidoptera;Aricia artaxerxes;Plebejus argus;1;Aricia artaxerxes;0.99976599124702;
MN107406;Lepidoptera;Aricia artaxerxes;Plebejus argus;1;Aricia artaxerxes;0.99976599124702;
MN107407;Lepidoptera;Aricia artaxerxes;Plebejus argus;1;Aricia artaxerxes;0.99976599124702;
MN107408;Lepidoptera;Aricia artaxerxes;Plebejus argus;1;Aricia artaxerxes;0.99976599124702;
MN107409;Lepidoptera;Aricia artaxerxes;Plebejus argus;1;Aricia artaxerxes;0.99976599124702;
MN138462;Lepidoptera;Thymelicus sylvestris;NA;0.324;Thymelicus sylvestris;0.999940778442454;
MN138463;Lepidoptera;Pyrgus carthami;Pyrgus carthami;1;Pyrgus carthami;0.999996750848985;
MN138464;Lepidoptera;Polyommatus icarus;Polyommatus icarus;0.002;Polyommatus icarus;0.999574015662565;
MN138465;Lepidoptera;Polyommatus icarus;Polyommatus icarus;0.002;Polyommatus icarus;0.999716017140251;
MN138466;Lepidoptera;Aricia agestis;NA;0.067;Aricia agestis;0.999509743227786;
MN138468;Lepidoptera;Pieris napi;NA;0.011;Pieris napi;0.801985394724814;
MN138469;Lepidoptera;Hamearis lucina;NA;NA;Hamearis lucina;0.999999999984141;
MN138470;Lepidoptera;Issoria lathonia;Issoria lathonia;1;Issoria lathonia;0.999999999989257;
MN138471;Lepidoptera;Gegenes pumilio;Gegenes pumilio;1;Gegenes pumilio;0.999986726679266;
MN138472;Lepidoptera;Pieris rapae;NA;NA;Pieris rapae;0.999985659117927;
MN138473;Lepidoptera;Plebejus idas;NA;0.001;NA;0.998518811781132;

MN138474;Lepidoptera;Vanessa cardui;Vanessa cardui;1;Vanessa cardui;0.999999459983318;
MN138476;Lepidoptera;Aricia cramera;Aricia cramera;1;Aricia cramera;0.999993731037033;
MN138477;Lepidoptera;Pyrgus onopordi;Pyrgus onopordi;1;Pyrgus onopordi;0.999997111229755;
MN138478;Lepidoptera;Boloria selene;NA;0.104;Boloria selene;0.999999788844655;
MN138480;Lepidoptera;Cupido minimus;NA;0.001;Cupido minimus;0.999420080317202;
MN138481;Lepidoptera;Satyrium spini;Satyrium spini;1;Satyrium spini;0.999999835426407;
MN138482;Lepidoptera;Erynnis tages;Erynnis tages;1;Erynnis tages;0.999999281987018;
MN138483;Lepidoptera;Aricia cramera;Aricia cramera;1;Aricia cramera;0.999993731037033;
MN138485;Lepidoptera;Cupido minimus;NA;0.006;Cupido minimus;0.999768371759258;
MN138487;Lepidoptera;Erynnis tages;Erynnis tages;1;Erynnis tages;0.999999281987018;
MN138488;Lepidoptera;Hipparchia hermione;Hipparchia hermione;1;Hipparchia hermione;0.999892638915927;
MN138489;Lepidoptera;Celastrina argiolus;Celastrina argiolus;1;Celastrina argiolus;0.999527385150531;
MN138490;Lepidoptera;Cupido minimus;NA;0.004;Cupido minimus;0.99987402211542;
MN138492;Lepidoptera;Melitaea varia;Melitaea varia;1;Melitaea varia;0.999996867427246;
MN138494;Lepidoptera;Vanessa cardui;Vanessa cardui;1;Vanessa cardui;0.999999459983318;
MN138495;Lepidoptera;Gonepteryx rhamni;Gonepteryx rhamni;1;Gonepteryx rhamni;0.999850170258883;
MN138496;Lepidoptera;Fabriciana adippe;NA;NA;Fabriciana adippe;0.900255826442102;
MN138497;Lepidoptera;Hipparchia fidia;Hipparchia fidia;1;Hipparchia fidia;1;
MN138500;Lepidoptera;Lysandra coridon;NA;NA;NA;0.993087957572662;
MN138501;Lepidoptera;Anthocharis cardamines;Anthocharis cardamines;1;Anthocharis cardamines;0.999999984875757;
MN138502;Lepidoptera;Aricia eumedon;Eumedonia eumedon;0.004;Eumedonia eumedon;0.995707742228806;
MN138503;Lepidoptera;Pieris rapae;NA;NA;Pieris rapae;0.999992588119495;
MN138504;Lepidoptera;Parnassius apollo;Parnassius apollo;1;Parnassius apollo;0.992704703916692;
MN138507;Lepidoptera;Polyommatus damon;Polyommatus damon;1;Polyommatus damon;0.999999867736202;
MN138508;Lepidoptera;Erebia stiria;NA;NA;Erebia styx;0.890739244956497;
MN138509;Lepidoptera;Polyommatus eros;NA;0.001;Polyommatus eros;0.974879223675754;
MN138511;Lepidoptera;Coenonympha gardetta;NA;0.001;Coenonympha gardetta;0.741030700939901;
MN138513;Lepidoptera;Limenitis camilla;Limenitis camilla;0.009;Limenitis camilla;0.9999828626808;
MN138514;Lepidoptera;Erebia cassioides;NA;NA;NA;0.999999999999977;
MN138515;Lepidoptera;Cupido minimus;NA;0.001;Cupido minimus;0.999803920701362;
MN138516;Lepidoptera;Thymelicus lineola;Thymelicus lineola;1;Thymelicus lineola;0.999995927238524;
MN138518;Lepidoptera;Nymphalis c-album;NA;0.215;NA;0.999999192918557;
MN138519;Lepidoptera;Aporia crataegi;Aporia crataegi;0.001;Aporia crataegi;0.999999184111451;
MN138520;Lepidoptera;Ochlodes sylvanus;NA;0.316;Ochlodes sylvanus;0.996318754838625;
MN138521;Lepidoptera;Coenonympha pamphilus;Coenonympha pamphilus;1;Coenonympha pamphilus;0.999999999999744;
MN138523;Lepidoptera;Pieris rapae;NA;NA;Pieris rapae;0.999984333973504;
MN138524;Lepidoptera;Nymphalis c-album;NA;0.215;NA;0.999999192918557;
MN138527;Lepidoptera;Erebia euryale;NA;NA;Erebia euryale;0.919518760623792;
MN138529;Lepidoptera;Thymelicus lineola;Thymelicus lineola;1;Thymelicus lineola;0.999975349725498;
MN138530;Lepidoptera;Satyrus ferula;Satyrus ferula;0.001;Satyrus ferula;0.992107254386621;
MN138531;Lepidoptera;Maniola jurtina;NA;0.001;Maniola jurtina;0.939220105860875;
MN138532;Lepidoptera;Aricia cramera;Aricia cramera;1;Aricia cramera;0.999993731037033;
MN138534;Lepidoptera;Maniola jurtina;NA;0.001;Maniola jurtina;0.939220105860875;
MN138535;Lepidoptera;Pieris rapae;Pieris rapae;1;Pieris rapae;0.999994083514588;
MN138536;Lepidoptera;Polyommatus daphnis;Polyommatus daphnis;1;Polyommatus daphnis;0.99989331516623;
MN138537;Lepidoptera;Aporia crataegi;NA;NA;Aporia crataegi;0.999998459680561;
MN138538;Lepidoptera;Issoria lathonia;Issoria lathonia;1;Issoria lathonia;0.999999999989257;
MN138540;Lepidoptera;Erebia montana;Erebia montana;1;Erebia montana;0.999686952031256;
MN138543;Lepidoptera;Colias crocea;NA;0.059;NA;0.999999999999738;
MN138544;Lepidoptera;Polyommatus dorylas;Polyommatus dorylas;0.008;Polyommatus dorylas;0.991281865899703;
MN138545;Lepidoptera;Aricia eumedon;Eumedonia eumedon;0.001;Eumedonia eumedon;0.998061393042216;
MN138546;Lepidoptera;Melitaea didyma;Melitaea didyma;0.999;Melitaea didyma;0.975495284820171;
MN138547;Lepidoptera;Maniola cecilia;Pyronia cecilia;NA;Pyronia cecilia;1;
MN138548;Lepidoptera;Coenonympha pamphilus;Coenonympha pamphilus;1;Coenonympha pamphilus;0.999999999999886;
MN138549;Lepidoptera;Arethusana arethusa;NA;0.062;Arethusana arethusa;0.999962489703143;
MN138551;Lepidoptera;Colias phicomone;NA;0.001;Colias phicomone;0.985598879697681;
MN138553;Lepidoptera;Pieris rapae;NA;NA;Pieris rapae;0.999992588119495;
MN138554;Lepidoptera;Pieris rapae;NA;NA;Pieris rapae;0.999992588119495;
MN138555;Lepidoptera;Polyommatus icarus;NA;0.166;Polyommatus icarus;0.749935576218847;
MN138555;Lepidoptera;Polyommatus icarus;NA;0.166;Polyommatus icarus;0.749935576218847;
MN138557;Lepidoptera;Lampides boeticus;Lampides boeticus;1;Lampides boeticus;0.999999998781163;
MN138558;Lepidoptera;Celastrina argiolus;Celastrina argiolus;1;Celastrina argiolus;0.999527385150531;
MN138559;Lepidoptera;Pieris rapae;NA;0.175;Pieris rapae;0.99996637543374;
MN138560;Lepidoptera;Coenonympha oedippus;Coenonympha oedippus;1;Coenonympha oedippus;0.999999999999801;
MN138561;Lepidoptera;Vanessa atalanta;Vanessa atalanta;1;Vanessa atalanta;0.999999999757762;
MN138562;Lepidoptera;Favonius quercus;NA;NA;Favonius quercus;0.73046069582187;

MN138563;Lepidoptera;Agriades optilete;Agriades optilete;1;Agriades optilete;0.9999999864568;
MN138564;Lepidoptera;Lycaena virgaureae;NA;0.179;Lycaena virgaureae;0.999538199124597;
MN138565;Lepidoptera;Polyommatus amandus;Polyommatus amandus;0.002;Polyommatus amandus;0.99998879592523;
MN138567;Lepidoptera;Pieris rapae;NA;NA;Pieris rapae;0.999984333973504;
MN138568;Lepidoptera;Thymelicus lineola;Thymelicus lineola;1;Thymelicus lineola;0.999999301001071;
MN138569;Lepidoptera;Melanargia galathea;Melanargia galathea;NA;Melanargia galathea;0.987293011345651;
MN138570;Lepidoptera;Melitaea didyma;NA;NA;Melitaea didyma;0.96852168594018;
MN138571;Lepidoptera;Thymelicus sylvestris;Thymelicus sylvestris;1;Thymelicus sylvestris;0.999999981568351;
MN138572;Lepidoptera;Melitaea cinxia;Melitaea cinxia;1;Melitaea cinxia;0.999999999999631;
MN138573;Lepidoptera;Pieris rapae;NA;0.039;Pieris rapae;0.999989302159245;
MN138574;Lepidoptera;Nymphalis c-album;NA;0.215;NA;0.999999192918557;
MN138575;Lepidoptera;Lycaena virgaureae;NA;0.186;Lycaena virgaureae;0.999491227342155;
MN138576;Lepidoptera;Melitaea didyma;Melitaea didyma;1;Melitaea didyma;0.999953644765793;
MN138577;Lepidoptera;Melitaea varia;Melitaea varia;1;Melitaea varia;0.99999954770546;
MN138578;Lepidoptera;Anthocharis cardamines;Anthocharis cardamines;1;Anthocharis cardamines;0.999999990347561;
MN138579;Lepidoptera;Aricia eumedon;Eumedonia eumedon;0.001;Eumedonia eumedon;0.997362661323557;
MN138581;Lepidoptera;Anthocharis euphenoides;NA;0.172;Anthocharis euphenoides;0.943741664916385;
MN138582;Lepidoptera;Erebia meolans;Erebia meolans;1;Erebia meolans;0.999231432693854;
MN138583;Lepidoptera;Lycaena tityrus;Lycaena tityrus;1;Lycaena tityrus;0.999998928238034;
MN138585;Lepidoptera;Gonepteryx cleopatra;Gonepteryx cleopatra;0.998;Gonepteryx cleopatra;0.999118636582266;
MN138586;Lepidoptera;Lycaena virgaureae;NA;0.178;Lycaena virgaureae;0.999268521681078;
MN138587;Lepidoptera;Celastrina argiolus;Celastrina argiolus;1;Celastrina argiolus;0.999527385150531;
MN138588;Lepidoptera;Pyrgus malvae;Pyrgus malvae;NA;Pyrgus malvae;0.980121140374433;
MN138589;Lepidoptera;Lycaena phlaeas;Lycaena phlaeas;1;Lycaena phlaeas;0.999999996363186;
MN138590;Lepidoptera;Fabriciana niobe;NA;0.005;Fabriciana niobe;0.992125835358755;
MN138591;Lepidoptera;Maniola cecilia;Pyronia cecilia;1;Pyronia cecilia;1;
MN138593;Lepidoptera;Pieris rapae;NA;0.075;Pieris rapae;0.999920903481213;
MN138594;Lepidoptera;Polyommatus daphnis;Polyommatus daphnis;1;Polyommatus daphnis;0.999921258190997;
MN138595;Lepidoptera;Pieris napi;NA;0.011;Pieris napi;0.801985394724814;
MN138596;Lepidoptera;Thymelicus lineola;Thymelicus lineola;1;Thymelicus lineola;0.999995927238524;
MN138597;Lepidoptera;Cupido osiris;Cupido osiris;1;Cupido osiris;0.999999409588131;
MN138598;Lepidoptera;Thymelicus sylvestris;NA;0.324;Thymelicus sylvestris;0.999940778442454;
MN138599;Lepidoptera;Melitaea varia;Melitaea varia;1;Melitaea varia;0.999996867427246;
MN138601;Lepidoptera;Gegenes nostrodamus;Gegenes nostrodamus;1;Gegenes nostrodamus;0.999999996883645;
MN138602;Lepidoptera;Coenonympha corinna;Coenonympha corinna;1;Coenonympha corinna;0.999999999984936;
MN138603;Lepidoptera;Fabriciana niobe;NA;0.005;Fabriciana niobe;0.992125835358755;
MN138604;Lepidoptera;Thymelicus acteon;Thymelicus acteon;1;Thymelicus acteon;0.99999999752248;
MN138606;Lepidoptera;Coenonympha oedippus;Coenonympha oedippus;1;Coenonympha oedippus;0.999999999999432;
MN138607;Lepidoptera;Plebejus argus;NA;0.133;Plebejus argus;0.983021740279961;
MN138608;Lepidoptera;Cupido argiades;NA;0.499;NA;0.999999999984682;
MN138609;Lepidoptera;Cupido minimus;NA;0.009;Cupido minimus;0.999791453712643;
MN138610;Lepidoptera;Pyrgus malvoides;NA;0.176;Pyrgus malvoides;0.940490890379026;
MN138611;Lepidoptera;Erebia medusa;NA;0.009;Erebia medusa;0.986275612856827;
MN138613;Lepidoptera;Lycaena tityrus;Lycaena tityrus;1;Lycaena tityrus;0.999998928238034;
MN138614;Lepidoptera;Cupido alcetas;NA;0.218;Cupido alcetas;0.833501983138177;
MN138616;Lepidoptera;Plebejus idas;NA;0.007;NA;0.999546324331549;
MN138617;Lepidoptera;Vanessa atalanta;Vanessa atalanta;1;Vanessa atalanta;0.999999999757762;
MN138618;Lepidoptera;Lycaena alciphron;Lycaena alciphron;1;Lycaena alciphron;0.999999925232376;
MN138621;Lepidoptera;Lycaena tityrus;Lycaena tityrus;1;Lycaena tityrus;0.999998928238034;
MN138623;Lepidoptera;Erebia pharte;Erebia pharte;1;Erebia pharte;0.99999999630495;
MN138625;Lepidoptera;Boloria euphrosyne;Boloria euphrosyne;0.003;Boloria euphrosyne;0.999970094877776;
MN138626;Lepidoptera;Pieris napi;NA;0.004;Pieris napi;0.875055491166831;
MN138627;Lepidoptera;Thymelicus acteon;Thymelicus acteon;1;Thymelicus acteon;0.99999999752248;
MN138628;Lepidoptera;Erebia euryale;NA;NA;Erebia euryale;0.915139912216032;
MN138629;Lepidoptera;Melitaea varia;Melitaea varia;1;Melitaea varia;0.999996867427246;
MN138630;Lepidoptera;Maniola jurtina;NA;0.012;Maniola jurtina;0.852326677366872;
MN138631;Lepidoptera;Aricia nicias;Aricia nicias;1;Aricia nicias;0.999956923176291;
MN138632;Lepidoptera;Erebia euryale;NA;NA;Erebia euryale;0.919518760623792;
MN138633;Lepidoptera;Boloria graeca;Boloria graeca;1;Boloria graeca;0.999999804714795;
MN138635;Lepidoptera;Anthocharis cardamines;Anthocharis cardamines;1;Anthocharis cardamines;0.99999997953563;
MN138636;Lepidoptera;Celastrina argiolus;Celastrina argiolus;1;Celastrina argiolus;0.999527385150531;
MN138637;Lepidoptera;Vanessa cardui;Vanessa cardui;1;Vanessa cardui;0.999999803450484;
MN138638;Lepidoptera;Melitaea cinxia;Melitaea cinxia;1;Melitaea cinxia;0.999999999999403;

MN138641;Lepidoptera;Cupido minimus;NA;0.003;Cupido minimus;0.999853615233319;
MN138642;Lepidoptera;Melitaea didyma;Melitaea didyma;NA;Melitaea didyma;0.988520743221049;
MN138645;Lepidoptera;Maniola jurtina;NA;0.001;Maniola jurtina;0.939220105860875;
MN138646;Lepidoptera;Lampides boeticus;Lampides boeticus;1;Lampides boeticus;0.999999997713644;
MN138647;Lepidoptera;Boloria euphrosyne;Boloria euphrosyne;1;Boloria euphrosyne;0.999997433270633;
MN138648;Lepidoptera;Thymelicus lineola;Thymelicus lineola;1;Thymelicus lineola;0.999996187945915;
MN138649;Lepidoptera;Polyommatus icarus;Polyommatus icarus;0.001;Polyommatus icarus;0.999851522482238;
MN138649;Lepidoptera;Polyommatus icarus;Polyommatus icarus;0.001;Polyommatus icarus;0.999851522482238;
MN138650;Lepidoptera;Aricia cramera;Aricia cramera;1;Aricia cramera;0.999993731037033;
MN138651;Lepidoptera;Coenonympha pamphilus;Coenonympha pamphilus;1;Coenonympha pamphilus;0.999999999999744;
MN138652;Lepidoptera;Thymelicus acteon;Thymelicus acteon;1;Thymelicus acteon;0.99999999752248;
MN138653;Lepidoptera;Thymelicus acteon;Thymelicus acteon;1;Thymelicus acteon;0.99999999752248;
MN138654;Lepidoptera;Papilio machaon;Papilio machaon;0.003;Papilio machaon;0.999482752749035;
MN138655;Lepidoptera;Boloria titania;Boloria titania;0.999;Boloria titania;0.999431261609546;
MN138656;Lepidoptera;Aricia agestis;NA;0.09;Aricia agestis;0.999208347982274;
MN138657;Lepidoptera;Vanessa cardui;Vanessa cardui;1;Vanessa cardui;0.999999803450484;
MN138658;Lepidoptera;Lycaena phlaeas;Lycaena phlaeas;1;Lycaena phlaeas;0.999999994740023;
MN138659;Lepidoptera;Maniola jurtina;NA;0.012;Maniola jurtina;0.91755716175482;
MN138661;Lepidoptera;Pieris napi;NA;0.011;Pieris napi;0.801985394724814;
MN138663;Lepidoptera;Hipparchia hermione;NA;NA;NA;0.999999999999991;
MN138664;Lepidoptera;Colias crocea;NA;0.059;NA;0.999999999999738;
MN138665;Lepidoptera;Vanessa cardui;Vanessa cardui;1;Vanessa cardui;0.999999459983318;
MN138666;Lepidoptera;Pieris napi;NA;0.011;Pieris napi;0.801985394724814;
MN138668;Lepidoptera;Coenonympha pamphilus;Coenonympha pamphilus;1;Coenonympha pamphilus;0.999999999999488;
MN138669;Lepidoptera;Cupido argiades;NA;0.499;NA;0.999999999984682;
MN138672;Lepidoptera;Thymelicus sylvestris;Thymelicus sylvestris;1;Thymelicus sylvestris;0.999999981568351;
MN138673;Lepidoptera;Lampides boeticus;Lampides boeticus;1;Lampides boeticus;0.999999999545906;
MN138674;Lepidoptera;Lycaena alciphron;Lycaena alciphron;1;Lycaena alciphron;0.999999718239206;
MN138679;Lepidoptera;Hipparchia fagi;NA;NA;Hipparchia fagi;0.958291584360022;
MN138680;Lepidoptera;Erynnis tages;Erynnis tages;1;Erynnis tages;0.999999281987018;
MN138681;Lepidoptera;Nymphalis c-album;NA;0.215;NA;0.999999192918557;
MN138682;Lepidoptera;Fabriciana adippe;Fabriciana adippe;0.007;Fabriciana adippe;0.876018745965171;
MN138683;Lepidoptera;Gegenes nostrodamus;Gegenes nostrodamus;1;Gegenes nostrodamus;0.999999996883645;
MN138685;Lepidoptera;Pieris napi;NA;0.011;Pieris napi;0.801985394724814;
MN138686;Lepidoptera;Limenitis camilla;NA;0.005;Limenitis camilla;0.999962352277235;
MN138687;Lepidoptera;Argynnis aglaja;NA;0.009;NA;0.999999999998612;
MN138690;Lepidoptera;Polyommatus icarus;NA;0.13;Polyommatus icarus;0.979762695748748;
MN138690;Lepidoptera;Polyommatus icarus;NA;0.13;Polyommatus icarus;0.979762695748748;
MN138691;Lepidoptera;Aricia agestis;NA;0.099;Aricia agestis;0.994857168775148;
MN138693;Lepidoptera;Plebejus argus;NA;NA;Plebejus argus;0.969283117748927;
MN138694;Lepidoptera;Vanessa atalanta;Vanessa atalanta;1;Vanessa atalanta;0.999999999757762;
MN138695;Lepidoptera;Melitaea didyma;Melitaea didyma;NA;Melitaea didyma;0.986420113105139;
MN138697;Lepidoptera;Melitaea didyma;Melitaea didyma;1;Melitaea didyma;0.999412077660356;
MN138698;Lepidoptera;Boloria euphrosyne;Boloria euphrosyne;1;Boloria euphrosyne;0.999999050867014;
MN138699;Lepidoptera;Satyrium ilicis;Satyrium ilicis;1;Satyrium ilicis;0.999999987901305;
MN138700;Lepidoptera;Anthocharis cardamines;Anthocharis cardamines;1;Anthocharis cardamines;0.999999891507378;
MN138701;Lepidoptera;Aricia agestis;NA;0.253;Aricia agestis;0.996350017387504;
MN138703;Lepidoptera;Brintesia circe;Brintesia circe;1;Brintesia circe;0.99999974034464;
MN138704;Lepidoptera;Thymelicus acteon;Thymelicus acteon;1;Thymelicus acteon;0.999999997365563;
MN138705;Lepidoptera;Argynnis aglaja;NA;0.002;Speyeria aglaja;0.87239079736947;
MN138706;Lepidoptera;Vanessa cardui;Vanessa cardui;1;Vanessa cardui;0.999999459983318;
MN138707;Lepidoptera;Aporia crataegi;NA;0.009;Aporia crataegi;0.999996613335036;
MN138708;Lepidoptera;Euphydryas aurinia;NA;0.137;Euphydryas aurinia;0.991077119919041;
MN138709;Lepidoptera;Lasiommata megera;Lasiommata megera;1;Lasiommata megera;0.999928528239676;
MN138710;Lepidoptera;Hipparchia leighebi;Hipparchia leighebi;1;Hipparchia leighebi;0.921532082781005;
MN138711;Lepidoptera;Colias phicomone;NA;0.001;Colias phicomone;0.985598879697681;
MN138712;Lepidoptera;Maniola jurtina;NA;0.001;Maniola jurtina;0.939220105860875;
MN138713;Lepidoptera;Aricia agestis;NA;0.077;Aricia agestis;0.998556400834668;
MN138714;Lepidoptera;Maniola jurtina;NA;0.001;Maniola jurtina;0.939220105860875;
MN138715;Lepidoptera;Maniola cecilia;Pyronia cecilia;1;Pyronia cecilia;1;
MN138716;Lepidoptera;Lycaena alciphron;Lycaena alciphron;1;Lycaena alciphron;0.999999928764825;
MN138717;Lepidoptera;Melitaea varia;Melitaea varia;1;Melitaea varia;0.999998281358841;
MN138718;Lepidoptera;Pieris rapae;NA;NA;Pieris rapae;0.999992588119495;
MN138719;Lepidoptera;Thymelicus sylvestris;NA;0.305;Thymelicus sylvestris;0.99992851189478;
MN138720;Lepidoptera;Maniola jurtina;NA;0.038;NA;0.999999999999524;
MN138722;Lepidoptera;Polyommatus icarus;NA;0.002;Polyommatus icarus;0.998502314961965;

MN138722;Lepidoptera;Polyommatus icarus;NA;0.002;Polyommatus icarus;0.998502314961965;
MN138724;Lepidoptera;Argynnis aglaja;NA;NA;Speyeria aglaja;0.872991147917853;
MN138725;Lepidoptera;Polyommatus icarus;NA;0.001;Polyommatus icarus;0.981285528541973;
MN138725;Lepidoptera;Polyommatus icarus;NA;0.001;Polyommatus icarus;0.981285528541973;
MN138726;Lepidoptera;Aricia agestis;NA;0.069;Aricia agestis;0.995918114091591;
MN138727;Lepidoptera;Satyrium spini;Satyrium spini;1;Satyrium spini;0.999999835426407;
MN138728;Lepidoptera;Lycaena phlaeas;Lycaena phlaeas;1;Lycaena phlaeas;0.999999996363186;
MN138729;Lepidoptera;Thymelicus acteon;Thymelicus acteon;1;Thymelicus acteon;0.99999999752248;
MN138730;Lepidoptera;Fabriciana niobe;NA;0.001;Fabriciana niobe;0.977553955749008;
MN138731;Lepidoptera;Polyommatus icarus;NA;0.166;Polyommatus icarus;0.749935576218847;
MN138731;Lepidoptera;Polyommatus icarus;NA;0.166;Polyommatus icarus;0.749935576218847;
MN138732;Lepidoptera;Erebia medusa;NA;0.009;Erebia medusa;0.986275612856827;
MN138733;Lepidoptera;Lysandra hispana;NA;NA;NA;0.999868794849817;
MN138734;Lepidoptera;Colias crocea;NA;0.059;NA;0.999999999999738;
MN138735;Lepidoptera;Pieris napi;NA;0.011;Pieris napi;0.801985394724814;
MN138736;Lepidoptera;Melitaea didyma;Melitaea didyma;1;Melitaea didyma;0.99958019737737;
MN138738;Lepidoptera;Plebejus argus;NA;0.043;Plebejus argus;0.975534930033561;
MN138739;Lepidoptera;Aphantopus hyperantus;Aphantopus hyperantus;0.003;Aphantopus hyperantus;0.999999455871523;
MN138740;Lepidoptera;Erebia pronoe;Erebia pronoe;1;Erebia pronoe;0.999250891771858;
MN138741;Lepidoptera;Polyommatus dolus;Polyommatus dolus;1;Polyommatus dolus;0.978004991113075;
MN138742;Lepidoptera;Nymphalis c-album;NA;0.215;NA;0.999999192918557;
MN138744;Lepidoptera;Fabriciana niobe;Fabriciana niobe;0.001;Fabriciana niobe;0.93186202957215;
MN138745;Lepidoptera;Celastrina argiolus;Celastrina argiolus;1;Celastrina argiolus;0.999527385150531;
MN138746;Lepidoptera;Cupido argiades;NA;0.499;NA;0.999999999984682;
MN138747;Lepidoptera;Favonius quercus;NA;NA;Favonius quercus;0.87732961169742;
MN138748;Lepidoptera;Lasiommata megera;Lasiommata megera;1;Lasiommata megera;0.999379144055441;
MN138749;Lepidoptera;Maniola jurtina;Maniola jurtina;0.021;Maniola jurtina;0.802110870400691;
MN138750;Lepidoptera;Pyrgus sidae;Pyrgus sidae;1;Pyrgus sidae;0.999994277634217;
MN138751;Lepidoptera;Polyommatus amandus;Polyommatus amandus;0.003;Polyommatus amandus;0.999977069858459;
MN138752;Lepidoptera;Thymelicus lineola;Thymelicus lineola;1;Thymelicus lineola;0.999969483849691;
MN138753;Lepidoptera;Pieris rapae;NA;NA;Pieris rapae;0.999992588119495;
MN138754;Lepidoptera;Cercyonis lupina;Hyponephele lupina;1;Hyponephele lupina;0.999999917600863;
MN138755;Lepidoptera;Erynnis tages;Erynnis tages;1;Erynnis tages;0.999999281987018;
MN138756;Lepidoptera;Plebejus argus;NA;0.122;Plebejus argus;0.956948912376078;
MN138757;Lepidoptera;Parnassius apollo;Parnassius apollo;1;Parnassius apollo;0.992704703916692;
MN138758;Lepidoptera;Euphydryas cynthia;Euphydryas cynthia;1;Euphydryas cynthia;0.999998812102883;
MN138759;Lepidoptera;Nymphalis c-album;NA;0.215;NA;0.999999192918557;
MN138760;Lepidoptera;Ochlodes sylvanus;NA;0.316;Ochlodes sylvanus;0.996318754838625;
MN138761;Lepidoptera;Pyrgus onopordi;Pyrgus onopordi;1;Pyrgus onopordi;0.999997111229755;
MN138762;Lepidoptera;Thymelicus sylvestris;Thymelicus sylvestris;1;Thymelicus sylvestris;0.999999985935205;
MN138765;Lepidoptera;Lasiommata megera;Lasiommata megera;1;Lasiommata megera;0.999379144055441;
MN138766;Lepidoptera;Lasiommata maera;Lasiommata maera;1;Lasiommata maera;0.999996552555305;
MN138767;Lepidoptera;Melitaea cinxia;Melitaea cinxia;1;Melitaea cinxia;0.999999999999716;
MN138768;Lepidoptera;Cupido alcetas;NA;0.068;Cupido alcetas;0.88988907876118;
MN138769;Lepidoptera;Cercyonis lupina;Hyponephele lupina;1;Hyponephele lupina;0.999999806147021;
MN138770;Lepidoptera;Melitaea didyma;Melitaea didyma;0.002;Melitaea didyma;0.993980744397441;
MN138771;Lepidoptera;Maniola jurtina;NA;0.038;NA;0.999999999999524;
MN138773;Lepidoptera;Polyommatus icarus;NA;0.004;Polyommatus icarus;0.999481683747521;
MN138773;Lepidoptera;Polyommatus icarus;NA;0.004;Polyommatus icarus;0.999481683747521;
MN138774;Lepidoptera;Nymphalis c-album;NA;0.215;NA;0.999999192918557;
MN138776;Lepidoptera;Maniola tithonus;Pyronia tithonus;1;Pyronia tithonus;1;
MN138778;Lepidoptera;Colias alfacariensis;Colias alfacariensis;0.001;Colias alfacariensis;0.999857790810452;
MN138779;Lepidoptera;Anthocharis damone;Anthocharis damone;1;Anthocharis damone;0.99999999829717;
MN138780;Lepidoptera;Aricia cramera;Aricia cramera;1;Aricia cramera;0.999993731037033;
MN138781;Lepidoptera;Maniola tithonus;Pyronia tithonus;1;Pyronia tithonus;1;
MN138782;Lepidoptera;Anthocharis damone;Anthocharis damone;1;Anthocharis damone;0.999999974090599;
MN138784;Lepidoptera;Lasiommata megera;Lasiommata megera;1;Lasiommata megera;0.999928528239676;
MN138785;Lepidoptera;Lasiommata maera;Lasiommata maera;1;Lasiommata maera;0.999996552555305;
MN138786;Lepidoptera;Maniola jurtina;NA;0.063;Maniola jurtina;0.770025363931686;
MN138788;Lepidoptera;Pieris rapae;NA;NA;Pieris rapae;0.999992588119495;
MN138789;Lepidoptera;Lasiommata megera;Lasiommata megera;1;Lasiommata megera;0.999928528239676;
MN138790;Lepidoptera;Glaucopsyche alexis;Glaucopsyche alexis;0.001;Glaucopsyche alexis;0.94042380088325;
MN138791;Lepidoptera;Vanessa atalanta;Vanessa atalanta;1;Vanessa atalanta;0.999999999757762;
MN138793;Lepidoptera;Maniola jurtina;NA;0.001;Maniola jurtina;0.939220105860875;
MN138794;Lepidoptera;Gonepteryx rhamni;Gonepteryx rhamni;1;Gonepteryx rhamni;0.999673119534565;
MN138796;Lepidoptera;Hipparchia semele;Hipparchia semele;0.001;Hipparchia semele;0.924825538448229;
MN138798;Lepidoptera;Thymelicus sylvestris;NA;0.324;Thymelicus sylvestris;0.999940778442454;

MN138801;Lepidoptera;Euphydryas aurinia;NA;0.31;Euphydryas aurinia;0.993387708761386;
MN138803;Lepidoptera;Melitaea cinxia;Melitaea cinxia;1;Melitaea cinxia;0.999999999999631;
MN138805;Lepidoptera;Melanargia galathea;Melanargia galathea;NA;Melanargia galathea;0.981855578470499;
MN138809;Lepidoptera;Lysandra coridon;NA;NA;NA;0.998070184737648;
MN138810;Lepidoptera;Lasiommata megera;Lasiommata megera;1;Lasiommata megera;0.999928528239676;
MN138811;Lepidoptera;Favonius quercus;NA;0.002;Favonius quercus;0.732659082125295;
MN138812;Lepidoptera;Plebejus idas;NA;0.007;NA;0.999546324331549;
MN138813;Lepidoptera;Polyommatus daphnis;Polyommatus daphnis;1;Polyommatus daphnis;0.99977716269156;
MN138814;Lepidoptera;Plebejus argyrognomon;NA;0.001;NA;0.994620093546684;
MN138815;Lepidoptera;Maniola tithonus;Pyronia tithonus;1;Pyronia tithonus;1;
MN138816;Lepidoptera;Boloria graeca;Boloria graeca;1;Boloria graeca;0.999999954422493;
MN138818;Lepidoptera;Boloria euphrosyne;Boloria euphrosyne;1;Boloria euphrosyne;0.999998247490937;
MN138820;Lepidoptera;Erebia epiphron;NA;NA;Erebia epiphron;0.98984833968802;
MN138821;Lepidoptera;Maniola jurtina;NA;NA;Maniola jurtina;0.973823960006955;
MN138823;Lepidoptera;Euphydryas aurinia;NA;0.31;Euphydryas aurinia;0.993387708761386;
MN138824;Lepidoptera;Polyommatus dolus;Polyommatus dolus;0.003;Polyommatus dolus;0.946056341712732;
MN138827;Lepidoptera;Glaucopsyche alexis;Glaucopsyche alexis;0.001;Glaucopsyche alexis;0.94042380088325;
MN138829;Lepidoptera;Callophrys avis;Callophrys avis;1;Callophrys avis;0.992335035373355;
MN138830;Lepidoptera;Aricia agestis;Aricia agestis;0.004;Aricia agestis;0.999546222180046;
MN138831;Lepidoptera;Lasiommata maera;Lasiommata maera;1;Lasiommata maera;0.999988599344549;
MN138833;Lepidoptera;Callophrys rubi;NA;0.019;Callophrys rubi;0.998201459637982;
MN138834;Lepidoptera;Colias phicomone;NA;0.001;Colias phicomone;0.985598879697681;
MN138837;Lepidoptera;Lycaena virgaureae;NA;0.249;Lycaena virgaureae;0.999388659324442;
MN138840;Lepidoptera;Coenonympha pamphilus;Coenonympha pamphilus;1;Coenonympha pamphilus;0.999999999999744;
MN138841;Lepidoptera;Maniola jurtina;NA;0.667;NA;0.999999999940105;
MN138842;Lepidoptera;Carcharodus alceae;Carcharodus alceae;1;Carcharodus alceae;0.999999998465938;
MN138843;Lepidoptera;Vanessa cardui;Vanessa cardui;1;Vanessa cardui;0.999999803450484;
MN138844;Lepidoptera;Satyrium acaciae;Satyrium acaciae;1;Satyrium acaciae;0.999999951077798;
MN138845;Lepidoptera;Lycaena hippothoe;Lycaena hippothoe;0.001;Lycaena hippothoe;0.9980672811561;
MN138846;Lepidoptera;Thymelicus lineola;Thymelicus lineola;1;Thymelicus lineola;0.999995927238524;
MN138847;Lepidoptera;Lycaena dispar;Lycaena dispar;1;Lycaena dispar;0.999999990743959;
MN138848;Lepidoptera;Favonius quercus;NA;0.176;Favonius quercus;0.828947799604254;
MN138849;Lepidoptera;Aporia crataegi;NA;NA;Aporia crataegi;0.999997860566808;
MN138850;Lepidoptera;Cercyonis lupina;Hyponephele lupina;1;Hyponephele lupina;0.999999968814081;
MN138851;Lepidoptera;Melitaea varia;Melitaea varia;1;Melitaea varia;0.999998691569804;
MN138852;Lepidoptera;Maniola cecilia;Pyronia cecilia;NA;Pyronia cecilia;1;
MN138853;Lepidoptera;Colias phicomone;NA;0.001;Colias phicomone;0.985598879697681;
MN138854;Lepidoptera;Maniola cecilia;Pyronia cecilia;NA;Pyronia cecilia;1;
MN138855;Lepidoptera;Anthocharis cardamines;Anthocharis cardamines;1;Anthocharis cardamines;0.999999984875757;
MN138857;Lepidoptera;Maniola cecilia;Pyronia cecilia;1;Pyronia cecilia;1;
MN138858;Lepidoptera;Lasiommata megera;Lasiommata megera;1;Lasiommata megera;0.999934558983719;
MN138859;Lepidoptera;Gonepteryx cleopatra;Gonepteryx cleopatra;0.998;Gonepteryx cleopatra;0.999118636582266;
MN138860;Lepidoptera;Aricia cramera;Aricia cramera;1;Aricia cramera;0.999993731037033;
MN138863;Lepidoptera;Issoria lathonia;Issoria lathonia;1;Issoria lathonia;0.999999999989257;
MN138864;Lepidoptera;Maniola tithonus;Pyronia tithonus;1;Pyronia tithonus;1;
MN138866;Lepidoptera;Fabriciana niobe;NA;0.001;Fabriciana niobe;0.989214650993052;
MN138867;Lepidoptera;Apatura ilia;NA;0.165;Apatura ilia;0.956796812031481;
MN138868;Lepidoptera;Maniola jurtina;NA;0.001;Maniola jurtina;0.939220105860875;
MN138869;Lepidoptera;Gegenes nostrodamus;Gegenes nostrodamus;1;Gegenes nostrodamus;0.999999996883645;
MN138871;Lepidoptera;Issoria lathonia;Issoria lathonia;1;Issoria lathonia;0.999999999989257;
MN138872;Lepidoptera;Chazara briseis;Chazara briseis;0.001;Chazara briseis;0.999987492373468;
MN138874;Lepidoptera;Lasiommata megera;Lasiommata megera;1;Lasiommata megera;0.999928528239676;
MN138877;Lepidoptera;Brintesia circe;Brintesia circe;1;Brintesia circe;0.99999951254154;
MN138878;Lepidoptera;Brenthis hecate;NA;0.104;Brenthis hecate;0.999656452740552;
MN138879;Lepidoptera;Erebia cassioides;NA;NA;NA;0.999999999999901;
MN138880;Lepidoptera;Coenonympha corinna;Coenonympha corinna;1;Coenonympha corinna;0.999999999974449;
MN138881;Lepidoptera;Pararge aegeria;Pararge aegeria;1;Pararge aegeria;0.999999999963165;
MN138885;Lepidoptera;Boloria euphrosyne;Boloria euphrosyne;1;Boloria euphrosyne;0.999999700428037;
MN138887;Lepidoptera;Nymphalis c-album;NA;0.215;NA;0.999999192918557;
MN138888;Lepidoptera;Coenonympha glycerion;NA;0.221;Coenonympha glycerion;0.999998704466507;
MN138889;Lepidoptera;Nymphalis c-album;NA;0.215;NA;0.999999192918557;
MN138890;Lepidoptera;Satyrus ferula;Satyrus ferula;0.001;Satyrus ferula;0.997072695563869;
MN138893;Lepidoptera;Lycaena alciphron;Lycaena alciphron;1;Lycaena alciphron;0.999999899077386;
MN138894;Lepidoptera;Colias crocea;NA;0.059;NA;0.999999999999738;

MN138896;Lepidoptera;Vanessa atalanta;Vanessa atalanta;1;Vanessa atalanta;0.999999999757762;
MN138897;Lepidoptera;Hipparchia semele;NA;0.002;Hipparchia semele;0.784603069619426;
MN138898;Lepidoptera;Agriades orbitulus;NA;0.031;Agriades orbitulus;0.998409903321215;
MN138900;Lepidoptera;Brintesia circe;Brintesia circe;1;Brintesia circe;0.999999898349165;
MN138901;Lepidoptera;Pieris napi;NA;0.028;Pieris napi;0.888205720664699;
MN138902;Lepidoptera;Polyommatus icarus;NA;0.002;Polyommatus icarus;0.999149936742386;
MN138902;Lepidoptera;Polyommatus icarus;NA;0.002;Polyommatus icarus;0.999149936742386;
MN138903;Lepidoptera;Ochlodes sylvanus;NA;0.119;Ochlodes sylvanus;0.997373902608318;
MN138904;Lepidoptera;Callophrys rubi;NA;0.001;Callophrys rubi;0.99870518510598;
MN138905;Lepidoptera;Aphantopus hyperantus;Aphantopus hyperantus;0.006;Aphantopus hyperantus;0.999998615504572;
MN138906;Lepidoptera;Cupido minimus;NA;0.009;Cupido minimus;0.999791453712643;
MN138908;Lepidoptera;Pyrgus armoricanus;Pyrgus armoricanus;1;Pyrgus armoricanus;0.999997353848817;
MN138909;Lepidoptera;Aricia artaxerxes;Plebejus argus;1;Aricia artaxerxes;0.999743187199922;
MN138910;Lepidoptera;Fabriciana adippe;Fabriciana adippe;NA;Fabriciana adippe;0.861972738590056;
MN138911;Lepidoptera;Maniola jurtina;Maniola jurtina;0.009;Maniola jurtina;0.977790299613342;
MN138912;Lepidoptera;Lampides boeticus;Lampides boeticus;1;Lampides boeticus;0.999999999545906;
MN138914;Lepidoptera;Maniola cecilia;Pyronia cecilia;NA;Pyronia cecilia;1;
MN138916;Lepidoptera;Maniola jurtina;NA;NA;Maniola jurtina;0.979727053537369;
MN138917;Lepidoptera;Anthocharis cardamines;Anthocharis cardamines;1;Anthocharis cardamines;0.999999940675226;
MN138918;Lepidoptera;Maniola jurtina;Maniola jurtina;NA;Maniola jurtina;0.827771985565139;
MN138919;Lepidoptera;Lycaena tityrus;Lycaena tityrus;1;Lycaena tityrus;0.999997027007808;
MN138921;Lepidoptera;Maniola jurtina;NA;0.146;NA;0.999999999999341;
MN138922;Lepidoptera;Polyommatus escheri;Polyommatus escheri;1;Polyommatus escheri;0.99999995510217;
MN138923;Lepidoptera;Lycaena tityrus;Lycaena tityrus;1;Lycaena tityrus;0.999995729087172;
MN138927;Lepidoptera;Lasiommata maera;Lasiommata maera;1;Lasiommata maera;0.999996552555305;
MN138930;Lepidoptera;Vanessa atalanta;Vanessa atalanta;1;Vanessa atalanta;0.999999999757762;
MN138932;Lepidoptera;Euphydryas aurinia;NA;0.118;Euphydryas aurinia;0.995366309208913;
MN138933;Lepidoptera;Lasiommata megera;Lasiommata megera;1;Lasiommata megera;0.999941247118496;
MN138934;Lepidoptera;Lycaena tityrus;Lycaena tityrus;1;Lycaena tityrus;0.999998928238034;
MN138935;Lepidoptera;Lycaena alciphron;Lycaena alciphron;1;Lycaena alciphron;0.999999925232376;
MN138936;Lepidoptera;Apatura ilia;NA;NA;Apatura ilia;0.971867715725452;
MN138937;Lepidoptera;Callophrys rubi;NA;0.019;Callophrys rubi;0.998201459637982;
MN138938;Lepidoptera;Erebia pandrose;Erebia pandrose;0.013;Erebia pandrose;0.974388092470716;
MN138939;Lepidoptera;Lasiommata megera;Lasiommata megera;1;Lasiommata megera;0.999879158841;
MN138940;Lepidoptera;Pieris napi;NA;0.011;Pieris napi;0.801985394724814;
MN138941;Lepidoptera;Lasiommata megera;Lasiommata megera;1;Lasiommata megera;0.999928528239676;
MN138942;Lepidoptera;Aricia agestis;NA;0.067;Aricia agestis;0.999509743227786;
MN138944;Lepidoptera;Plebejus argyrognomon;NA;0.001;NA;0.994620093546684;
MN138945;Lepidoptera;Vanessa cardui;Vanessa cardui;1;Vanessa cardui;0.999999803450484;
MN138946;Lepidoptera;Maniola jurtina;NA;0.005;Maniola jurtina;0.944899890539899;
MN138947;Lepidoptera;Nymphalis c-album;NA;0.215;NA;0.999999192918557;
MN138950;Lepidoptera;Aricia cramera;Aricia cramera;1;Aricia cramera;0.999993731037033;
MN138951;Lepidoptera;Gonepteryx rhamni;NA;0.474;Gonepteryx rhamni;0.982076396127858;
MN138952;Lepidoptera;Melitaea deione;Melitaea deione;1;Melitaea deione;0.999993024796593;
MN138955;Lepidoptera;Satyrus actaea;Satyrus actaea;0.002;Satyrus actaea;0.996459824853088;
MN138956;Lepidoptera;Issoria lathonia;Issoria lathonia;1;Issoria lathonia;0.999999999989257;
MN138958;Lepidoptera;Erebia euryale;NA;NA;Erebia euryale;0.919518760623792;
MN138959;Lepidoptera;Nymphalis c-album;NA;0.028;Polygonia c-album;0.766222230265535;
MN138960;Lepidoptera;Colias hyale;Colias hyale;0.015;Colias hyale;0.999997884296128;
MN138964;Lepidoptera;Maniola jurtina;NA;NA;Maniola jurtina;0.950689021412593;
MN138965;Lepidoptera;Aricia agestis;NA;0.253;Aricia agestis;0.996350017387504;
MN138966;Lepidoptera;Colias alfacariensis;Colias alfacariensis;0.001;Colias alfacariensis;0.999926026317526;
MN138967;Lepidoptera;Lycaena hippothoe;NA;NA;Lycaena hippothoe;0.96171722036274;
MN138968;Lepidoptera;Melitaea parthenoides;Melitaea parthenoides;1;Melitaea parthenoides;0.999999995982648;
MN138970;Lepidoptera;Colias crocea;NA;0.059;NA;0.999999999999738;
MN138971;Lepidoptera;Issoria lathonia;Issoria lathonia;1;Issoria lathonia;0.999999999989257;
MN138972;Lepidoptera;Pieris napi;NA;NA;NA;100.000.000.000.001;
MN138973;Lepidoptera;Pyrgus carthami;Pyrgus carthami;1;Pyrgus carthami;0.999998997209607;
MN138974;Lepidoptera;Nymphalis c-album;NA;0.215;NA;0.999999192918557;
MN138976;Lepidoptera;Erebia pronoe;Erebia pronoe;NA;Erebia pronoe;0.790922387957511;
MN138977;Lepidoptera;Pyrgus armoricanus;Pyrgus armoricanus;1;Pyrgus armoricanus;0.999997353848817;
MN138978;Lepidoptera;Lasiommata maera;Lasiommata maera;1;Lasiommata maera;0.999995366023818;
MN138979;Lepidoptera;Maniola jurtina;NA;NA;NA;0.99999999985062;
MN138980;Lepidoptera;Thymelicus sylvestris;NA;0.324;Thymelicus sylvestris;0.999940778442454;
MN138982;Lepidoptera;Erebia medusa;NA;0.01;Erebia medusa;0.979621232162315;
MN138983;Lepidoptera;Thymelicus sylvestris;Thymelicus sylvestris;1;Thymelicus sylvestris;0.999999985935205;
MN138984;Lepidoptera;Satyrium spini;Satyrium spini;1;Satyrium spini;0.999999835426407;
MN138985;Lepidoptera;Cercyonis lupina;Hyponephele lupina;1;Hyponephele lupina;0.999999971627091;
MN138986;Lepidoptera;Pyrgus malvae;Pyrgus malvae;0.999;Pyrgus malvae;0.993531783407179;
MN138987;Lepidoptera;Euphydryas aurinia;NA;0.31;Euphydryas aurinia;0.993387708761386;

MN138988;Lepidoptera;Euphydryas aurinia;NA;0.001;Euphydryas aurinia;0.995576749826865;
MN138990;Lepidoptera;Parnassius apollo;Parnassius apollo;1;Parnassius apollo;0.992704703916692;
MN138992;Lepidoptera;Polyommatus dolus;Polyommatus dolus;0.997;Polyommatus dolus;0.955195871823379;
MN138993;Lepidoptera;Colias crocea;NA;0.059;NA;0.999999999999738;
MN138997;Lepidoptera;Fabriciana adippe;NA;0.001;Fabriciana adippe;0.957131879909897;
MN138998;Lepidoptera;Erynnis tages;Erynnis tages;1;Erynnis tages;0.999999281987018;
MN139000;Lepidoptera;Gonepteryx rhamni;NA;0.474;Gonepteryx rhamni;0.982076396127858;
MN139002;Lepidoptera;Minois dryas;NA;0.025;Minois dryas;0.850746026247397;
MN139003;Lepidoptera;Fabriciana niobe;NA;0.005;Fabriciana niobe;0.992125835358755;
MN139004;Lepidoptera;Anthocharis euphenoides;NA;0.326;Anthocharis euphenoides;0.917872480229179;
MN139006;Lepidoptera;Maniola jurtina;NA;0.001;Maniola jurtina;0.939220105860875;
MN139007;Lepidoptera;Nymphalis c-album;NA;0.215;NA;0.999999192918557;
MN139008;Lepidoptera;Erebia cassioides;NA;0.001;NA;0.999999999999959;
MN139010;Lepidoptera;Thymelicus acteon;Thymelicus acteon;1;Thymelicus acteon;0.999999997365563;
MN139011;Lepidoptera;Aglais urticae;NA;0.05;Aglais urticae;0.990090905940875;
MN139012;Lepidoptera;Satyrium spini;Satyrium spini;1;Satyrium spini;0.999999835426407;
MN139013;Lepidoptera;Lycaena phlaeas;Lycaena phlaeas;1;Lycaena phlaeas;0.999999996363186;
MN139014;Lepidoptera;Satyrium pruni;NA;0.076;Satyrium pruni;0.990322364021655;
MN139015;Lepidoptera;Lasiommata paramegaera;Lasiommata paramegaera;0.999;Lasiommata paramegaera;0.995283446287531;
MN139016;Lepidoptera;Melitaea deione;Melitaea deione;1;Melitaea deione;0.999996202527362;
MN139021;Lepidoptera;Lasiommata megera;Lasiommata megera;1;Lasiommata megera;0.999379144055441;
MN139022;Lepidoptera;Pieris napi;NA;NA;Pieris napi;0.863911025205274;
MN139023;Lepidoptera;Aricia agestis;NA;0.253;Aricia agestis;0.996350017387504;
MN139024;Lepidoptera;Cupido minimus;NA;0.006;Cupido minimus;0.999768371759258;
MN139025;Lepidoptera;Polyommatus icarus;Polyommatus icarus;0.001;Polyommatus icarus;0.999815390691822;
MN139025;Lepidoptera;Polyommatus icarus;Polyommatus icarus;0.001;Polyommatus icarus;0.999815390691822;
MN139026;Lepidoptera;Plebejus argyrognomon;NA;0.001;NA;0.998518811781132;
MN139029;Lepidoptera;Cupido alcetas;NA;0.068;Cupido alcetas;0.88988907876118;
MN139030;Lepidoptera;Hipparchia hermione;NA;NA;NA;0.999999999999991;
MN139034;Lepidoptera;Satyrium acaciae;Satyrium acaciae;1;Satyrium acaciae;0.999999374890702;
MN139036;Lepidoptera;Coenonympha arcania;Coenonympha arcania;0.033;Coenonympha arcania;0.993266644798764;
MN139037;Lepidoptera;Brenthis hecate;Brenthis hecate;0.006;Brenthis hecate;0.99995137380518;
MN139038;Lepidoptera;Lycaena hippothoe;Lycaena hippothoe;NA;Lycaena hippothoe;0.989480999146802;
MN139039;Lepidoptera;Erebia oeme;NA;0.114;Erebia oeme;0.999999999830635;
MN139040;Lepidoptera;Pyrgus cacaliae;Pyrgus cacaliae;0.001;Pyrgus cacaliae;0.997779968725098;
MN139041;Lepidoptera;Polyommatus damon;Polyommatus damon;1;Polyommatus damon;0.999999882210126;
MN139043;Lepidoptera;Satyrium spini;Satyrium spini;1;Satyrium spini;0.999999874306646;
MN139044;Lepidoptera;Coenonympha arcania;NA;0.004;NA;0.999999999999983;
MN139045;Lepidoptera;Euphydryas cynthia;Euphydryas cynthia;1;Euphydryas cynthia;0.999999999705039;
MN139048;Lepidoptera;Aricia agestis;NA;0.253;Aricia agestis;0.996350017387504;
MN139050;Lepidoptera;Polyommatus damon;Polyommatus damon;1;Polyommatus damon;0.999999882210126;
MN139051;Lepidoptera;Hipparchia leighebi;Hipparchia leighebi;0.001;Hipparchia leighebi;0.886425158869477;
MN139052;Lepidoptera;Thymelicus lineola;Thymelicus lineola;1;Thymelicus lineola;0.999971535093703;
MN139053;Lepidoptera;Lampides boeticus;Lampides boeticus;1;Lampides boeticus;0.99999999876789;
MN139055;Lepidoptera;Anthocharis cardamines;Anthocharis cardamines;1;Anthocharis cardamines;0.99999997953563;
MN139059;Lepidoptera;Melitaea cinxia;Melitaea cinxia;1;Melitaea cinxia;0.999999999999659;
MN139060;Lepidoptera;Pyrgus malvoides;NA;0.176;Pyrgus malvoides;0.940490890379026;
MN139061;Lepidoptera;Celastrina argiolus;Celastrina argiolus;NA;Celastrina argiolus;0.998817020643142;
MN139062;Lepidoptera;Aglais urticae;NA;NA;Aglais urticae;0.985780134057395;
MN139063;Lepidoptera;Hipparchia hermione;NA;NA;NA;0.99999999999999;
MN139065;Lepidoptera;Lasiommata megera;Lasiommata megera;1;Lasiommata megera;0.999928528239676;
MN139068;Lepidoptera;Argynnis aglaja;NA;NA;Speyeria aglaja;0.872991147917853;
MN139069;Lepidoptera;Gonepteryx cleopatra;Gonepteryx cleopatra;0.998;Gonepteryx cleopatra;0.999118636582266;
MN139070;Lepidoptera;Polyommatus icarus;NA;0.166;Polyommatus icarus;0.749935576218847;
MN139070;Lepidoptera;Polyommatus icarus;NA;0.166;Polyommatus icarus;0.749935576218847;
MN139071;Lepidoptera;Polyommatus icarus;Polyommatus icarus;0.001;Polyommatus icarus;0.999851522482238;
MN139071;Lepidoptera;Polyommatus icarus;Polyommatus icarus;0.001;Polyommatus icarus;0.999851522482238;
MN139072;Lepidoptera;Satyrium ilicis;Satyrium ilicis;1;Satyrium ilicis;0.999999987901305;
MN139073;Lepidoptera;Celastrina argiolus;Celastrina argiolus;1;Celastrina argiolus;0.999527385150531;
MN139078;Lepidoptera;Aglais urticae;Aglais urticae;0.001;Aglais urticae;0.994396335177485;
MN139079;Lepidoptera;Lycaena hippothoe;Lycaena hippothoe;0.001;Lycaena hippothoe;0.9980672811561;
MN139080;Lepidoptera;Erebia neoridas;Erebia neoridas;NA;Erebia neoridas;0.998631936578793;
MN139081;Lepidoptera;Pyrgus alveus;NA;NA;Pyrgus alveus;0.920439481638217;

MN139083;Lepidoptera;Glaucopsyche alexis;Glaucopsyche alexis;0.001;Glaucopsyche alexis;0.94042380088325;
MN139084;Lepidoptera;Maniola jurtina;NA;0.001;Maniola jurtina;0.939220105860875;
MN139085;Lepidoptera;Pyrgus carthami;Pyrgus carthami;1;Pyrgus carthami;0.999998997209607;
MN139086;Lepidoptera;Celastrina argiolus;Celastrina argiolus;1;Celastrina argiolus;0.999708846309046;
MN139087;Lepidoptera;Lycaena tityrus;Lycaena tityrus;1;Lycaena tityrus;0.999998928238034;
MN139088;Lepidoptera;Cupido alcetas;NA;0.218;Cupido alcetas;0.833501983138177;
MN139089;Lepidoptera;Erebia montana;Erebia montana;1;Erebia montana;0.999627744460498;
MN139091;Lepidoptera;Erebia melampus;Erebia melampus;NA;Erebia melampus;0.970136285597645;
MN139092;Lepidoptera;Hipparchia fidia;Hipparchia fidia;1;Hipparchia fidia;1;
MN139093;Lepidoptera;Erebia neoridas;NA;NA;Erebia neoridas;0.99731844101466;
MN139094;Lepidoptera;Maniola jurtina;NA;NA;Maniola jurtina;0.962368106146462;
MN139095;Lepidoptera;Vanessa cardui;Vanessa cardui;1;Vanessa cardui;0.999997579965112;
MN139096;Lepidoptera;Araschnia levana;Araschnia levana;1;Araschnia levana;0.999999772187832;
MN139100;Lepidoptera;Thymelicus acteon;Thymelicus acteon;1;Thymelicus acteon;0.99999999752248;
MN139101;Lepidoptera;Hipparchia sbordonii;NA;NA;Hipparchia semele;0.748345401572611;
MN139102;Lepidoptera;Colias palaeno;NA;0.002;NA;0.999999999983681;
MN139104;Lepidoptera;Erebia meolans;Erebia meolans;NA;Erebia meolans;0.999855133075834;
MN139106;Lepidoptera;Lasiommata maera;Lasiommata maera;1;Lasiommata maera;0.999996380940229;
MN139107;Lepidoptera;Maniola jurtina;NA;0.012;Maniola jurtina;0.91755716175482;
MN139108;Lepidoptera;Aricia cramera;Aricia cramera;1;Aricia cramera;0.999995777202847;
MN139111;Lepidoptera;Erebia stiria;NA;NA;Erebia styx;0.890739244956497;
MN139112;Lepidoptera;Thymelicus acteon;Thymelicus acteon;1;Thymelicus acteon;0.999999996630038;
MN139113;Lepidoptera;Polyommatus icarus;Polyommatus icarus;0.001;Polyommatus icarus;0.999851522482238;
MN139113;Lepidoptera;Polyommatus icarus;Polyommatus icarus;0.001;Polyommatus icarus;0.999851522482238;
MN139114;Lepidoptera;Polyommatus icarus;NA;0.002;Polyommatus icarus;0.998502314961965;
MN139114;Lepidoptera;Polyommatus icarus;NA;0.002;Polyommatus icarus;0.998502314961965;
MN139116;Lepidoptera;Plebejus argus;NA;0.095;Plebejus argus;0.975968053247715;
MN139117;Lepidoptera;Aricia eumedon;Eumedonia eumedon;0.002;Eumedonia eumedon;0.99911741929599;
MN139118;Lepidoptera;Pyrgus armoricanus;Pyrgus armoricanus;1;Pyrgus armoricanus;0.999914079595002;
MN139119;Lepidoptera;Maniola jurtina;NA;0.038;NA;0.999999999999524;
MN139120;Lepidoptera;Fabriciana adippe;Fabriciana adippe;NA;Fabriciana adippe;0.845993302533839;
MN139121;Lepidoptera;Maniola jurtina;NA;0.001;Maniola jurtina;0.970021298748324;
MN139122;Lepidoptera;Erynnis tages;Erynnis tages;1;Erynnis tages;0.999999281987018;
MN139124;Lepidoptera;Colias alfacariensis;Colias alfacariensis;0.001;Colias alfacariensis;0.999926026317526;
MN139126;Lepidoptera;Lycaena phlaeas;Lycaena phlaeas;1;Lycaena phlaeas;0.999999996618726;
MN139127;Lepidoptera;Lampides boeticus;Lampides boeticus;1;Lampides boeticus;0.999999999545906;
MN139128;Lepidoptera;Pyrgus onopordi;Pyrgus onopordi;1;Pyrgus onopordi;0.999997111229755;
MN139129;Lepidoptera;Thymelicus sylvestris;NA;0.333;Thymelicus sylvestris;0.999926375650344;
MN139130;Lepidoptera;Lycaena hippothoe;NA;NA;Lycaena hippothoe;0.96171722036274;
MN139132;Lepidoptera;Aricia agestis;NA;0.067;Aricia agestis;0.999509743227786;
MN139134;Lepidoptera;Lasiommata maera;Lasiommata maera;1;Lasiommata maera;0.999996200814449;
MN139136;Lepidoptera;Colias crocea;NA;0.059;NA;0.999999999999738;
MN139137;Lepidoptera;Anthocharis euphenoides;NA;0.131;Anthocharis euphenoides;0.934740202775498;
MN139138;Lepidoptera;Thymelicus acteon;Thymelicus acteon;1;Thymelicus acteon;0.99999999752248;
MN139139;Lepidoptera;Pyrgus serratulae;Pyrgus serratulae;1;Pyrgus serratulae;0.999999848602397;
MN139140;Lepidoptera;Satyrium spini;Satyrium spini;1;Satyrium spini;0.999999721054916;
MN139142;Lepidoptera;Colias crocea;NA;0.059;NA;0.999999999999738;
MN139143;Lepidoptera;Colias crocea;NA;0.059;NA;0.999999999999738;
MN139152;Lepidoptera;Erebia melampus;Erebia melampus;0.011;Erebia melampus;0.975499903768874;
MN139154;Lepidoptera;Maniola jurtina;NA;NA;Maniola jurtina;0.957544379788978;
MN139156;Lepidoptera;Fabriciana adippe;NA;NA;Fabriciana adippe;0.922280384106682;
MN139158;Lepidoptera;Thymelicus sylvestris;Thymelicus sylvestris;1;Thymelicus sylvestris;0.99999876153052;
MN139159;Lepidoptera;Aricia cramera;Aricia cramera;1;Aricia cramera;0.999990014848976;
MN139160;Lepidoptera;Cupido minimus;NA;0.006;Cupido minimus;0.999768371759258;
MN139161;Lepidoptera;Pyrgus sidae;Pyrgus sidae;1;Pyrgus sidae;0.999994277634217;
MN139163;Lepidoptera;Plebejus argus;Plebejus argus;0.016;Plebejus argus;0.962605109836459;
MN139164;Lepidoptera;Lycaena hippothoe;Lycaena hippothoe;0.002;Lycaena hippothoe;0.994699106153046;
MN139165;Lepidoptera;Carcharodus alceae;Carcharodus alceae;0.999;Carcharodus alceae;0.999999998008462;
MN139167;Lepidoptera;Ochlodes sylvanus;NA;0.119;Ochlodes sylvanus;0.997373902608318;
MN139170;Lepidoptera;Thymelicus sylvestris;NA;0.324;Thymelicus sylvestris;0.999940778442454;
MN139171;Lepidoptera;Plebejus argus;Plebejus argus;0.014;Plebejus argus;0.985827082916227;
MN139172;Lepidoptera;Ochlodes sylvanus;NA;0.316;Ochlodes sylvanus;0.996318754838625;
MN139173;Lepidoptera;Carcharodus alceae;Carcharodus alceae;0.999;Carcharodus alceae;0.999999998008462;
MN139174;Lepidoptera;Polyommatus icarus;NA;0.003;Polyommatus icarus;0.986125651677522;
MN139174;Lepidoptera;Polyommatus icarus;NA;0.003;Polyommatus icarus;0.986125651677522;
MN139175;Lepidoptera;Vanessa cardui;Vanessa cardui;1;Vanessa cardui;0.999999803450484;
MN139176;Lepidoptera;Melitaea didyma;NA;0.077;Melitaea didyma;0.978856953763553;

MN139178;Lepidoptera;Glaucopsyche alexis;Glaucopsyche alexis;0.001;Glaucopsyche alexis;0.94042380088325;
MN139180;Lepidoptera;Lasiommata megera;Lasiommata megera;1;Lasiommata megera;0.999928528239676;
MN139183;Lepidoptera;Erebia melampus;Erebia melampus;0.003;Erebia melampus;0.981722308229927;
MN139184;Lepidoptera;Maniola tithonus;Pyronia tithonus;1;Pyronia tithonus;1;
MN139186;Lepidoptera;Plebejus argyrognomon;NA;0.004;NA;0.998514827971857;
MN139188;Lepidoptera;Gonepteryx cleopatra;Gonepteryx cleopatra;0.998;Gonepteryx cleopatra;0.999118636582266;
MN139190;Lepidoptera;Colias alfacariensis;Colias alfacariensis;1;Colias alfacariensis;0.999986994131001;
MN139191;Lepidoptera;Aricia cramera;Aricia cramera;1;Aricia cramera;0.999990463719651;
MN139193;Lepidoptera;Polyommatus icarus;NA;0.166;Polyommatus icarus;0.749935576218847;
MN139193;Lepidoptera;Polyommatus icarus;NA;0.166;Polyommatus icarus;0.749935576218847;
MN139194;Lepidoptera;Issoria lathonia;Issoria lathonia;1;Issoria lathonia;0.999999999989257;
MN139195;Lepidoptera;Polyommatus icarus;Polyommatus icarus;0.002;Polyommatus icarus;0.999716017140251;
MN139195;Lepidoptera;Polyommatus icarus;Polyommatus icarus;0.002;Polyommatus icarus;0.999716017140251;
MN139196;Lepidoptera;Parnassius apollo;Parnassius apollo;1;Parnassius apollo;0.994691248300955;
MN139197;Lepidoptera;Lycaena thersamon;Lycaena thersamon;0.001;Lycaena thersamon;0.952360353045614;
MN139199;Lepidoptera;Lycaena tityrus;Lycaena tityrus;1;Lycaena tityrus;0.999998928238034;
MN139202;Lepidoptera;Issoria lathonia;Issoria lathonia;1;Issoria lathonia;0.999999999989257;
MN139204;Lepidoptera;Ochlodes sylvanus;NA;0.119;Ochlodes sylvanus;0.997373902608318;
MN139205;Lepidoptera;Hipparchia fagi;Hipparchia fagi;NA;Hipparchia fagi;0.987048294127975;
MN139206;Lepidoptera;Melitaea diamina;NA;0.149;Melitaea diamina;0.999999996592464;
MN139207;Lepidoptera;Colias crocea;NA;0.059;NA;0.999999999999738;
MN139208;Lepidoptera;Fabriciana niobe;NA;0.005;Fabriciana niobe;0.992125835358755;
MN139210;Lepidoptera;Ochlodes sylvanus;NA;0.316;Ochlodes sylvanus;0.996318754838625;
MN139212;Lepidoptera;Anthocharis damone;Anthocharis damone;1;Anthocharis damone;0.999999991170057;
MN139213;Lepidoptera;Erebia euryale;NA;NA;Erebia euryale;0.919518760623792;
MN139215;Lepidoptera;Chazara briseis;Chazara briseis;1;Chazara briseis;0.999992037130752;
MN139216;Lepidoptera;Nymphalis c-album;NA;0.215;NA;0.999999192918557;
MN139218;Lepidoptera;Nymphalis egea;Polygonia egea;1;Polygonia egea;0.999999878357791;
MN139219;Lepidoptera;Melitaea diamina;NA;0.001;Melitaea diamina;0.999999979350975;
MN139221;Lepidoptera;Thymelicus lineola;Thymelicus lineola;1;Thymelicus lineola;0.999976816785769;
MN139222;Lepidoptera;Nymphalis io;NA;0.491;Aglais io;0.930153237246992;
MN139223;Lepidoptera;Aporia crataegi;NA;NA;Aporia crataegi;0.999998459680561;
MN139225;Lepidoptera;Nymphalis egea;Polygonia egea;1;Polygonia egea;0.999999758078475;
MN139226;Lepidoptera;Lasiommata maera;Lasiommata maera;1;Lasiommata maera;0.99999510792253;
MN139227;Lepidoptera;Boloria euphrosyne;Boloria euphrosyne;0.008;Boloria euphrosyne;0.999964281413411;
MN139229;Lepidoptera;Lasiommata megera;Lasiommata megera;1;Lasiommata megera;0.999379144055441;
MN139230;Lepidoptera;Erebia eriphyle;Erebia eriphyle;1;Erebia eriphyle;0.999999983979336;
MN139231;Lepidoptera;Lysandra coridon;NA;NA;Lysandra coridon;0.811340269227995;
MN139232;Lepidoptera;Brenthis daphne;Brenthis daphne;0.011;Brenthis daphne;0.946409829233859;
MN139233;Lepidoptera;Ochlodes sylvanus;NA;0.316;Ochlodes sylvanus;0.996318754838625;
MN139234;Lepidoptera;Carcharodus alceae;Carcharodus alceae;0.999;Carcharodus alceae;0.999999996339511;
MN139235;Lepidoptera;Lasiommata megera;Lasiommata megera;1;Lasiommata megera;0.999928528239676;
MN139236;Lepidoptera;Lasiommata megera;Lasiommata megera;1;Lasiommata megera;0.999806890129977;
MN139237;Lepidoptera;Pyrgus carthami;Pyrgus carthami;1;Pyrgus carthami;0.99998821600502;
MN139238;Lepidoptera;Lasiommata paramegaera;Lasiommata paramegaera;0.999;Lasiommata paramegaera;0.995283446287531;
MN139239;Lepidoptera;Ochlodes sylvanus;NA;0.316;Ochlodes sylvanus;0.996318754838625;
MN139240;Lepidoptera;Aporia crataegi;NA;NA;Aporia crataegi;0.999998518523558;
MN139241;Lepidoptera;Fabriciana elisa;Fabriciana elisa;0.001;Fabriciana elisa;0.991687097294013;
MN139242;Lepidoptera;Coenonympha pamphilus;Coenonympha pamphilus;1;Coenonympha pamphilus;0.999999999999744;
MN139244;Lepidoptera;Polyommatus icarus;Polyommatus icarus;0.001;Polyommatus icarus;0.999851522482238;
MN139244;Lepidoptera;Polyommatus icarus;Polyommatus icarus;0.001;Polyommatus icarus;0.999851522482238;
MN139245;Lepidoptera;Pyrgus andromedae;Pyrgus andromedae;0.001;Pyrgus andromedae;0.998828669843251;
MN139246;Lepidoptera;Plebejus argus;NA;0.124;Plebejus argus;0.982974540879239;
MN139247;Lepidoptera;Pyrgus malvoides;NA;0.111;Pyrgus malvoides;0.968399374774442;
MN139249;Lepidoptera;Ochlodes sylvanus;NA;0.316;Ochlodes sylvanus;0.996318754838625;
MN139250;Lepidoptera;Coenonympha arcania;NA;0.105;NA;0.999999999999971;
MN139251;Lepidoptera;Hipparchia fagi;Hipparchia fagi;NA;Hipparchia fagi;0.986566456150817;
MN139256;Lepidoptera;Brenthis hecate;NA;0.056;Brenthis hecate;0.9998033982787;
MN139257;Lepidoptera;Erebia ottomana;Erebia ottomana;1;Erebia ottomana;0.999999991465188;
MN139258;Lepidoptera;Polyommatus icarus;NA;0.001;Polyommatus icarus;0.981285528541973;
MN139258;Lepidoptera;Polyommatus icarus;NA;0.001;Polyommatus icarus;0.981285528541973;
MN139259;Lepidoptera;Anthocharis euphenoides;Anthocharis euphenoides;0.009;Anthocharis euphenoides;0.985905235113516;

MN139260;Lepidoptera;Ochlodes sylvanus;NA;0.117;Ochlodes sylvanus;0.997347010117109;
MN139261;Lepidoptera;Plebejus idas;Plebejus idas;0.001;NA;0.999436143300552;
MN139262;Lepidoptera;Melitaea cinxia;Melitaea cinxia;1;Melitaea cinxia;0.999999999998835;
MN139263;Lepidoptera;Thymelicus acteon;Thymelicus acteon;1;Thymelicus acteon;0.99999999752248;
MN139264;Lepidoptera;Polyommatus icarus;NA;0.007;Polyommatus icarus;0.999081737461227;
MN139264;Lepidoptera;Polyommatus icarus;NA;0.007;Polyommatus icarus;0.999081737461227;
MN139265;Lepidoptera;Coenonympha arcania;NA;0.172;Coenonympha arcania;0.963036309473038;
MN139266;Lepidoptera;Pyrgus malvoides;NA;0.176;Pyrgus malvoides;0.940490890379026;
MN139268;Lepidoptera;Brintesia circe;Brintesia circe;NA;Brintesia circe;0.99999894309325;
MN139269;Lepidoptera;Coenonympha arcania;NA;0.316;NA;0.999999999999985;
MN139270;Lepidoptera;Favonius quercus;NA;NA;Favonius quercus;0.749488008187033;
MN139271;Lepidoptera;Erebia pronoe;Erebia pronoe;1;Erebia pronoe;0.999250891771858;
MN139272;Lepidoptera;Cupido minimus;NA;0.006;Cupido minimus;0.999753607968986;
MN139273;Lepidoptera;Maniola tithonus;Pyronia tithonus;1;Pyronia tithonus;1;
MN139276;Lepidoptera;Cupido alcetas;NA;0.218;Cupido alcetas;0.833501983138177;
MN139277;Lepidoptera;Lycaena phlaeas;Lycaena phlaeas;1;Lycaena phlaeas;0.999999996363186;
MN139278;Lepidoptera;Lycaena hippothoe;Lycaena hippothoe;NA;Lycaena hippothoe;0.929815071946357;
MN139279;Lepidoptera;Aricia agestis;NA;NA;Aricia agestis;0.993980512246517;
MN139280;Lepidoptera;Satyrium ilicis;Satyrium ilicis;1;Satyrium ilicis;0.999999987901305;
MN139283;Lepidoptera;Coenonympha pamphilus;Coenonympha pamphilus;1;Coenonympha
pamphilus;0.999999999999744;
MN139285;Lepidoptera;Melitaea didyma;Melitaea didyma;1;Melitaea didyma;0.999429129005203;
MN139286;Lepidoptera;Thymelicus sylvestris;NA;0.324;Thymelicus sylvestris;0.999940778442454;
MN139287;Lepidoptera;Erebia oeme;NA;0.114;Erebia oeme;0.999999999830635;
MN139288;Lepidoptera;Melitaea diamina;NA;0.149;Melitaea diamina;0.999999996592464;
MN139289;Lepidoptera;Brenthis daphne;Brenthis daphne;0.006;Brenthis daphne;0.984559944066479;
MN139291;Lepidoptera;Pyrgus malvae;Pyrgus malvae;NA;Pyrgus malvae;0.980121140374433;
MN139296;Lepidoptera;Melitaea cinxia;Melitaea cinxia;1;Melitaea cinxia;0.999999999998835;
MN139297;Lepidoptera;Hipparchia hermione;NA;NA;NA;0.999999999999991;
MN139298;Lepidoptera;Papilio machaon;Papilio machaon;NA;Papilio machaon;0.998335940984695;
MN139299;Lepidoptera;Maniola jurtina;NA;0.108;Maniola jurtina;0.870434006715787;
MN139300;Lepidoptera;Plebejus argus;NA;0.118;Plebejus argus;0.977297982426809;
MN139301;Lepidoptera;Satyrium pruni;NA;0.076;Satyrium pruni;0.990322364021655;
MN139302;Lepidoptera;Anthocharis euphenoides;Anthocharis euphenoides;0.006;Anthocharis
euphenoides;0.984813140103259;
MN139303;Lepidoptera;Colias crocea;NA;0.059;NA;0.999999999999738;
MN139304;Lepidoptera;Gegenes nostrodamus;Gegenes nostrodamus;1;Gegenes
nostrodamus;0.999999996883645;
MN139305;Lepidoptera;Thymelicus acteon;Thymelicus acteon;1;Thymelicus acteon;0.99999999752248;
MN139307;Lepidoptera;Brenthis ino;Brenthis ino;0.003;Brenthis ino;0.98906191156141;
MN139308;Lepidoptera;Maniola tithonus;Pyronia tithonus;NA;Pyronia tithonus;1;
MN139310;Lepidoptera;Plebejus idas;NA;0.007;NA;0.999546324331549;
MN139311;Lepidoptera;Boloria euphrosyne;Boloria euphrosyne;1;Boloria
euphrosyne;0.999998365644188;
MN139312;Lepidoptera;Pyrgus armoricanus;Pyrgus armoricanus;1;Pyrgus
armoricanus;0.999997353848817;
MN139313;Lepidoptera;Thymelicus sylvestris;Thymelicus sylvestris;1;Thymelicus
sylvestris;0.999999985935205;
MN139314;Lepidoptera;Lycaena dispar;Lycaena dispar;1;Lycaena dispar;0.999999994053013;
MN139315;Lepidoptera;Pyrgus malvoides;NA;0.016;Pyrgus malvoides;0.878050137245322;
MN139316;Lepidoptera;Charaxes jasius;Charaxes jasius;NA;Charaxes jasius;0.990397821479112;
MN139317;Lepidoptera;Maniola jurtina;NA;0.012;Maniola jurtina;0.91755716175482;
MN139319;Lepidoptera;Aricia agestis;NA;0.067;Aricia agestis;0.999509743227786;
MN139322;Lepidoptera;Erebia euryale;NA;NA;Erebia euryale;0.919518760623792;
MN139323;Lepidoptera;Plebejus argyrognomon;NA;0.001;NA;0.994620093546684;
MN139324;Lepidoptera;Plebejus argus;Plebejus argus;0.019;Plebejus argus;0.974059310897195;
MN139325;Lepidoptera;Melanargia galathea;Melanargia galathea;NA;Melanargia
galathea;0.959429932872284;
MN139326;Lepidoptera;Aglais urticae;NA;0.001;Aglais urticae;0.983623896759349;
MN139328;Lepidoptera;Vanessa cardui;Vanessa cardui;1;Vanessa cardui;0.999999459983318;
MN139329;Lepidoptera;Cercyonis lupina;Hyponephele lupina;1;Hyponephele lupina;0.999999940565518;
MN139332;Lepidoptera;Lampides boeticus;Lampides boeticus;1;Lampides boeticus;0.999999999545906;
MN139333;Lepidoptera;Aporia crataegi;NA;NA;Aporia crataegi;0.999998518523558;
MN139334;Lepidoptera;Maniola jurtina;NA;0.003;Maniola jurtina;0.934943936861184;
MN139335;Lepidoptera;Maniola jurtina;NA;0.038;NA;0.999999999999524;
MN139336;Lepidoptera;Aglais urticae;Aglais urticae;0.999;Aglais urticae;0.994120587202935;
MN139337;Lepidoptera;Pyrgus carthami;Pyrgus carthami;1;Pyrgus carthami;0.999995592447668;
MN139339;Lepidoptera;Coenonympha oedippus;Coenonympha oedippus;1;Coenonympha
oedippus;0.999999999999432;
MN139341;Lepidoptera;Polyommatus dolus;Polyommatus dolus;0.001;Polyommatus
dolus;0.945738812333627;
MN139344;Lepidoptera;Boloria euphrosyne;Boloria euphrosyne;1;Boloria
euphrosyne;0.999999754214146;
MN139345;Lepidoptera;Lasiommata megera;Lasiommata megera;1;Lasiommata megera;0.999379144055441;
MN139346;Lepidoptera;Maniola jurtina;NA;0.012;Maniola jurtina;0.91755716175482;
MN139347;Lepidoptera;Plebejus idas;NA;0.007;NA;0.999546324331549;
MN139348;Lepidoptera;Plebejus argyrognomon;NA;0.001;NA;0.998518811781132;
MN139349;Lepidoptera;Erynnis tages;Erynnis tages;1;Erynnis tages;0.999999281987018;

MN139351;Lepidoptera;Pyrgus andromedae;Pyrgus andromedae;0.001;Pyrgus andromedae;0.997448239493065;
MN139353;Lepidoptera;Cupido argiades;NA;0.173;Cupido argiades;0.80102807497701;
MN139354;Lepidoptera;Aglais urticae;Aglais urticae;0.003;Aglais urticae;0.987698041564968;
MN139355;Lepidoptera;Euphydryas cynthia;Euphydryas cynthia;1;Euphydryas cynthia;0.999999999705039;
MN139356;Lepidoptera;Anthocharis cardamines;Anthocharis cardamines;1;Anthocharis cardamines;0.999999986709838;
MN139357;Lepidoptera;Carcharodus alceae;Carcharodus alceae;1;Carcharodus alceae;0.999999998465938;
MN139358;Lepidoptera;Hipparchia hermione;Hipparchia hermione;NA;Hipparchia hermione;0.74525153929477;
MN139359;Lepidoptera;Agriades orbitulus;NA;0.01;Agriades orbitulus;0.998991302227708;
MN139362;Lepidoptera;Celastrina argiolus;Celastrina argiolus;1;Celastrina argiolus;0.999037794018796;
MN139363;Lepidoptera;Maniola jurtina;NA;0.038;NA;0.999999999999524;
MN139364;Lepidoptera;Plebejus argyrognomon;NA;0.001;NA;0.994620093546684;
MN139366;Lepidoptera;Nymphalis egea;Polygonia egea;1;Polygonia egea;0.999999758078475;
MN139369;Lepidoptera;Issoria lathonia;Issoria lathonia;1;Issoria lathonia;0.999999999989257;
MN139370;Lepidoptera;Ochlodes sylvanus;NA;0.316;Ochlodes sylvanus;0.996318754838625;
MN139371;Lepidoptera;Thymelicus lineola;Thymelicus lineola;1;Thymelicus lineola;0.999995927238524;
MN139374;Lepidoptera;Hipparchia semele;NA;0.001;Hipparchia semele;0.868056708468869;
MN139375;Lepidoptera;Colias hyale;Colias hyale;0.015;Colias hyale;0.999997884296128;
MN139376;Lepidoptera;Favonius quercus;NA;NA;Favonius quercus;0.791633840981009;
MN139377;Lepidoptera;Melitaea cinxia;Melitaea cinxia;1;Melitaea cinxia;0.999999999999801;
MN139378;Lepidoptera;Nymphalis c-album;NA;0.215;NA;0.999999192918557;
MN139379;Lepidoptera;Celastrina argiolus;Celastrina argiolus;1;Celastrina argiolus;0.999527385150531;
MN139380;Lepidoptera;Arethusana arethusa;NA;0.064;Arethusana arethusa;0.999932923751678;
MN139382;Lepidoptera;Thymelicus lineola;Thymelicus lineola;1;Thymelicus lineola;0.999995384857434;
MN139383;Lepidoptera;Boloria euphrosyne;Boloria euphrosyne;1;Boloria euphrosyne;0.999999450156461;
MN139384;Lepidoptera;Pyrgus malvae;Pyrgus malvae;0.999;Pyrgus malvae;0.993531783407179;
MN139387;Lepidoptera;Lycaena virgaureae;NA;0.089;Lycaena virgaureae;0.999665201081283;
MN139389;Lepidoptera;Melitaea cinxia;Melitaea cinxia;1;Melitaea cinxia;0.999999999999829;
MN139390;Lepidoptera;Ochlodes sylvanus;NA;0.316;Ochlodes sylvanus;0.996318754838625;
MN139392;Lepidoptera;Melitaea cinxia;Melitaea cinxia;1;Melitaea cinxia;0.999999999998721;
MN139393;Lepidoptera;Pyrgus andromedae;Pyrgus andromedae;0.001;Pyrgus andromedae;0.998828669843251;
MN139396;Lepidoptera;Hipparchia semele;NA;NA;Hipparchia semele;0.86892676007213;
MN139397;Lepidoptera;Cupido minimus;NA;0.019;Cupido minimus;0.999739809346213;
MN139398;Lepidoptera;Gonepteryx cleopatra;Gonepteryx cleopatra;0.998;Gonepteryx cleopatra;0.999118636582266;
MN139399;Lepidoptera;Carcharodus alceae;Carcharodus alceae;0.999;Carcharodus alceae;0.999999996339511;
MN139401;Lepidoptera;Melitaea trivia;Melitaea trivia;1;Melitaea trivia;0.999999999960437;
MN139402;Lepidoptera;Fabriciana elisa;Fabriciana elisa;0.001;Fabriciana elisa;0.994839902224823;
MN139403;Lepidoptera;Aglais urticae;NA;0.002;Aglais urticae;0.987497593219019;
MN139404;Lepidoptera;Glaucopsyche alexis;Glaucopsyche alexis;0.001;Glaucopsyche alexis;0.94042380088325;
MN139405;Lepidoptera;Coenonympha pamphilus;Coenonympha pamphilus;1;Coenonympha pamphilus;0.999999999999744;
MN139406;Lepidoptera;Nymphalis c-album;NA;0.215;NA;0.999999192918557;
MN139407;Lepidoptera;Satyrium w-album;NA;0.056;Satyrium w-album;0.989378230996316;
MN139408;Lepidoptera;Melitaea cinxia;Melitaea cinxia;1;Melitaea cinxia;0.999999999999801;
MN139409;Lepidoptera;Fabriciana adippe;NA;NA;Fabriciana adippe;0.900255826442102;
MN139411;Lepidoptera;Polyommatus celina;Polyommatus celina;0.007;Polyommatus celina;0.999999999839019;
MN139413;Lepidoptera;Aricia agestis;NA;0.253;Aricia agestis;0.996350017387504;
MN139414;Lepidoptera;Coenonympha arcania;NA;0.047;Coenonympha arcania;0.819452108223873;
MN139415;Lepidoptera;Fabriciana adippe;Fabriciana adippe;0.003;Fabriciana adippe;0.994298341064395;
MN139416;Lepidoptera;Maniola jurtina;NA;NA;NA;0.99999999985062;
MN139419;Lepidoptera;Pieris napi;NA;0.011;Pieris napi;0.801985394724814;
MN139420;Lepidoptera;Apatura ilia;NA;0.165;Apatura ilia;0.956796812031481;
MN139421;Lepidoptera;Maniola jurtina;NA;0.001;Maniola jurtina;0.939220105860875;
MN139422;Lepidoptera;Coenonympha pamphilus;Coenonympha pamphilus;1;Coenonympha pamphilus;0.999999999999744;
MN139423;Lepidoptera;Hamearis lucina;NA;NA;Hamearis lucina;0.999999999962824;
MN139424;Lepidoptera;Cupido argiades;NA;0.499;NA;0.999999999984682;
MN139425;Lepidoptera;Erebia euryale;NA;NA;Erebia euryale;0.948790417707171;
MN139426;Lepidoptera;Anthocharis cardamines;Anthocharis cardamines;1;Anthocharis cardamines;0.999999984875757;
MN139427;Lepidoptera;Coenonympha pamphilus;Coenonympha pamphilus;1;Coenonympha pamphilus;0.999999999999744;
MN139428;Lepidoptera;Ochlodes sylvanus;NA;0.119;Ochlodes sylvanus;0.997373902608318;

MN139429;Lepidoptera;Maniola jurtina;NA;NA;NA;0.99999999985062;
MN139431;Lepidoptera;Fabriciana adippe;NA;NA;Fabriciana adippe;0.900255826442102;
MN139432;Lepidoptera;Thymelicus lineola;Thymelicus lineola;1;Thymelicus lineola;0.999971535093703;
MN139433;Lepidoptera;Hipparchia semele;NA;NA;Hipparchia semele;0.748345401572611;
MN139434;Lepidoptera;Callophrys rubi;NA;0.002;Callophrys rubi;0.998841790162773;
MN139435;Lepidoptera;Coenonympha oedippus;Coenonympha oedippus;1;Coenonympha oedippus;0.999999999976467;
MN139437;Lepidoptera;Thymelicus acteon;Thymelicus acteon;1;Thymelicus acteon;0.99999999752248;
MN139438;Lepidoptera;Ochlodes sylvanus;NA;0.316;Ochlodes sylvanus;0.996318754838625;
MN139440;Lepidoptera;Lycaena hippothoe;NA;NA;Lycaena hippothoe;0.928973453460131;
MN139441;Lepidoptera;Agriades optilete;Agriades optilete;1;Agriades optilete;0.99999995660511;
MN139442;Lepidoptera;Coenonympha gardetta;NA;0.12;NA;0.999999999999993;
MN139444;Lepidoptera;Erebia pharte;Erebia pharte;1;Erebia pharte;0.99999999630495;
MN139445;Lepidoptera;Plebejus argyrognomon;NA;0.004;NA;0.997919811835919;
MN139446;Lepidoptera;Cupido minimus;NA;0.009;Cupido minimus;0.999791453712643;
MN139447;Lepidoptera;Pyrgus malvoides;NA;0.176;Pyrgus malvoides;0.940490890379026;
MN139452;Lepidoptera;Cyaniris semiargus;NA;NA;Cyaniris semiargus;0.999051841766953;
MN139453;Lepidoptera;Polyommatus icarus;NA;0.004;Polyommatus icarus;0.963846856760863;
MN139453;Lepidoptera;Polyommatus icarus;NA;0.004;Polyommatus icarus;0.963846856760863;
MN139454;Lepidoptera;Vanessa cardui;Vanessa cardui;1;Vanessa cardui;0.999999459983318;
MN139455;Lepidoptera;Coenonympha arcania;NA;0.172;Coenonympha arcania;0.963036309473038;
MN139456;Lepidoptera;Lycaena phlaeas;Lycaena phlaeas;1;Lycaena phlaeas;0.999999996363186;
MN139457;Lepidoptera;Pyrgus malvoides;NA;0.052;Pyrgus malvoides;0.943208768333892;
MN139458;Lepidoptera;Argynnis aglaja;NA;0.007;Speyeria aglaja;0.806162363046318;
MN139459;Lepidoptera;Aricia cramera;Aricia cramera;1;Aricia cramera;0.999993731037033;
MN139460;Lepidoptera;Coenonympha pamphilus;Coenonympha pamphilus;1;Coenonympha pamphilus;0.999999999999744;
MN139462;Lepidoptera;Plebejus idas;Plebejus idas;0.001;NA;0.999655983096466;
MN139463;Lepidoptera;Iolana iolas;Iolana iolas;1;Iolana iolas;0.999999046251105;
MN139464;Lepidoptera;Pyrgus carlinae;NA;NA;Pyrgus carlinae;0.854199018204849;
MN139465;Lepidoptera;Gonepteryx cleopatra;Gonepteryx cleopatra;0.998;Gonepteryx cleopatra;0.999118636582266;
MN139466;Lepidoptera;Lysandra coridon;NA;NA;NA;0.997904753762663;
MN139467;Lepidoptera;Thymelicus lineola;Thymelicus lineola;1;Thymelicus lineola;0.999995927238524;
MN139469;Lepidoptera;Erebia tyndarus;NA;NA;NA;0.999999999999923;
MN139471;Lepidoptera;Aporia crataegi;NA;NA;Aporia crataegi;0.999998459680561;
MN139472;Lepidoptera;Apatura ilia;NA;0.165;Apatura ilia;0.956796812031481;
MN139473;Lepidoptera;Coenonympha gardetta;NA;0.001;Coenonympha gardetta;0.731095812176462;
MN139474;Lepidoptera;Euphydryas aurinia;Euphydryas aurinia;0.017;Euphydryas aurinia;0.997183742560994;
MN139478;Lepidoptera;Thymelicus sylvestris;Thymelicus sylvestris;1;Thymelicus sylvestris;0.999999985935205;
MN139479;Lepidoptera;Cupido argiades;NA;0.499;NA;0.999999999984682;
MN139480;Lepidoptera;Aricia agestis;NA;0.253;Aricia agestis;0.996350017387504;
MN139481;Lepidoptera;Gonepteryx cleopatra;Gonepteryx cleopatra;0.998;Gonepteryx cleopatra;0.999118636582266;
MN139483;Lepidoptera;Polyommatus amandus;Polyommatus amandus;0.002;Polyommatus amandus;0.99998879592523;
MN139484;Lepidoptera;Issoria lathonia;Issoria lathonia;1;Issoria lathonia;0.999999999994344;
MN139486;Lepidoptera;Cyaniris semiargus;Cyaniris semiargus;NA;Cyaniris semiargus;0.999815796556651;
MN139488;Lepidoptera;Brenthis daphne;Brenthis daphne;0.001;Brenthis daphne;0.998360260866441;
MN139489;Lepidoptera;Aricia cramera;Aricia cramera;1;Aricia cramera;0.999994899228873;
MN139490;Lepidoptera;Favonius quercus;NA;NA;Favonius quercus;0.73046069582187;
MN139491;Lepidoptera;Melitaea didyma;Melitaea didyma;1;Melitaea didyma;0.999937162819573;
MN139494;Lepidoptera;Chazara briseis;Chazara briseis;1;Chazara briseis;0.999980393060755;
MN139496;Lepidoptera;Coenonympha oedippus;Coenonympha oedippus;1;Coenonympha oedippus;0.99999999999946;
MN139498;Lepidoptera;Lycaena tityrus;Lycaena tityrus;1;Lycaena tityrus;0.999998928238034;
MN139499;Lepidoptera;Plebejus idas;NA;0.007;NA;0.999546324331549;
MN139500;Lepidoptera;Aricia agestis;NA;0.067;Aricia agestis;0.999509743227786;
MN139501;Lepidoptera;Lycaena hippothoe;NA;NA;Lycaena hippothoe;0.96171722036274;
MN139502;Lepidoptera;Issoria lathonia;Issoria lathonia;1;Issoria lathonia;0.999999999989257;
MN139504;Lepidoptera;Polyommatus icarus;NA;0.007;Polyommatus icarus;0.999081737461227;
MN139504;Lepidoptera;Polyommatus icarus;NA;0.007;Polyommatus icarus;0.999081737461227;
MN139505;Lepidoptera;Maniola nurag;Maniola nurag;1;Maniola nurag;0.94592398344326;
MN139506;Lepidoptera;Aricia cramera;Aricia cramera;1;Aricia cramera;0.999993731037033;
MN139507;Lepidoptera;Argynnis aglaja;NA;NA;NA;0.999999999998051;
MN139508;Lepidoptera;Gonepteryx rhamni;NA;0.474;Gonepteryx rhamni;0.982076396127858;
MN139511;Lepidoptera;Apatura iris;Apatura iris;1;Apatura iris;0.999999999999972;
MN139512;Lepidoptera;Aricia agestis;NA;0.216;Aricia agestis;0.998446013113235;
MN139514;Lepidoptera;Maniola tithonus;Pyronia tithonus;1;Pyronia tithonus;1;
MN139515;Lepidoptera;Cupido minimus;NA;0.003;Cupido minimus;0.999878204579428;
MN139516;Lepidoptera;Parnassius apollo;Parnassius apollo;1;Parnassius apollo;0.992704703916692;
MN139518;Lepidoptera;Lycaena dispar;Lycaena dispar;1;Lycaena dispar;0.999999990743959;

MN139519;Lepidoptera;Polyommatus dorylas;Polyommatus dorylas;0.008;Polyommatus dorylas;0.991281865899703;
MN139520;Lepidoptera;Satyrium pruni;NA;0.076;Satyrium pruni;0.990322364021655;
MN139521;Lepidoptera;Lasiommata megera;Lasiommata megera;1;Lasiommata megera;0.999928528239676;
MN139522;Lepidoptera;Euphydryas aurinia;NA;0.31;Euphydryas aurinia;0.993387708761386;
MN139523;Lepidoptera;Celastrina argiolus;Celastrina argiolus;1;Celastrina argiolus;0.999527385150531;
MN139524;Lepidoptera;Chazara briseis;Chazara briseis;0.001;Chazara briseis;0.999987492373468;
MN139525;Lepidoptera;Hipparchia blachieri;NA;0.004;Hipparchia semele;0.783011492754646;
MN139526;Lepidoptera;Boloria titania;Boloria titania;1;Boloria titania;0.999958713201422;
MN139527;Lepidoptera;Lycaena tityrus;Lycaena tityrus;1;Lycaena tityrus;0.999998928238034;
MN139529;Lepidoptera;Maniola tithonus;Pyronia tithonus;1;Pyronia tithonus;1;
MN139530;Lepidoptera;Thymelicus lineola;Thymelicus lineola;1;Thymelicus lineola;0.999995927238524;
MN139533;Lepidoptera;Aricia agestis;NA;0.037;Aricia agestis;0.998710695905853;
MN139536;Lepidoptera;Lasiommata megera;Lasiommata megera;1;Lasiommata megera;0.999379144055441;
MN139537;Lepidoptera;Pyrgus malvae;Pyrgus malvae;NA;Pyrgus malvae;0.985600926823364;
MN139538;Lepidoptera;Glaucopsyche alexis;Glaucopsyche alexis;0.001;Glaucopsyche alexis;0.94042380088325;
MN139539;Lepidoptera;Plebejus argus;NA;0.036;Plebejus argus;0.988160277018267;
MN139540;Lepidoptera;Thecla betulae;Thecla betulae;1;Thecla betulae;0.999999999972886;
MN139541;Lepidoptera;Polyommatus amandus;Polyommatus amandus;0.006;Polyommatus amandus;0.999981460994076;
MN139544;Lepidoptera;Erebia melampus;Erebia melampus;0.001;Erebia melampus;0.991704985968506;
MN139545;Lepidoptera;Boloria euphrosyne;Boloria euphrosyne;1;Boloria euphrosyne;0.999999349564162;
MN139548;Lepidoptera;Polyommatus dorylas;Polyommatus dorylas;0.008;Polyommatus dorylas;0.991281865899703;
MN139550;Lepidoptera;Boloria euphrosyne;Boloria euphrosyne;0.001;Boloria euphrosyne;0.999988342496913;
MN139551;Lepidoptera;Polyommatus dolus;Polyommatus dolus;0.997;Polyommatus dolus;0.955195871823379;
MN139552;Lepidoptera;Satyrium w-album;NA;0.056;Satyrium w-album;0.989378230996316;
MN139553;Lepidoptera;Colias alfacariensis;Colias alfacariensis;0.001;Colias alfacariensis;0.999926026317526;
MN139554;Lepidoptera;Carcharodus alceae;Carcharodus alceae;1;Carcharodus alceae;0.999999998465938;
MN139555;Lepidoptera;Plebejus idas;NA;0.007;NA;0.999546324331549;
MN139557;Lepidoptera;Aglais urticae;NA;0.002;Aglais urticae;0.987497593219019;
MN139558;Lepidoptera;Brenthis ino;Brenthis ino;0.002;Brenthis ino;0.99842931627954;
MN139559;Lepidoptera;Maniola jurtina;NA;0.001;Maniola jurtina;0.939220105860875;
MN139560;Lepidoptera;Pyrgus warrenensis;NA;NA;Pyrgus warrenensis;0.765902945204032;
MN139562;Lepidoptera;Hipparchia fagi;NA;NA;Hipparchia fagi;0.958291584360022;
MN139563;Lepidoptera;Lasiommata megera;Lasiommata megera;1;Lasiommata megera;0.999379144055441;
MN139565;Lepidoptera;Euphydryas aurinia;NA;0.16;Euphydryas aurinia;0.991721078690876;
MN139566;Lepidoptera;Coenonympha pamphilus;Coenonympha pamphilus;1;Coenonympha pamphilus;0.999999999999545;
MN139567;Lepidoptera;Papilio machaon;Papilio machaon;0.003;Papilio machaon;0.997288237409893;
MN139570;Lepidoptera;Brintesia circe;Brintesia circe;1;Brintesia circe;0.99999951254154;
MN139571;Lepidoptera;Boloria selene;NA;0.275;Boloria selene;0.999999702697396;
MN139572;Lepidoptera;Brintesia circe;Brintesia circe;1;Brintesia circe;0.999999924576204;
MN139573;Lepidoptera;Coenonympha glycerion;NA;0.264;Coenonympha glycerion;0.999998370144304;
MN139574;Lepidoptera;Thymelicus sylvestris;NA;0.324;Thymelicus sylvestris;0.999940778442454;
MN139575;Lepidoptera;Papilio machaon;Papilio machaon;0.002;Papilio machaon;0.99964655301485;
MN139576;Lepidoptera;Pyrgus alveus;NA;NA;NA;0.999999999995496;
MN139578;Lepidoptera;Hipparchia fagi;Hipparchia fagi;NA;Hipparchia fagi;0.993333095914904;
MN139579;Lepidoptera;Apatura ilia;NA;0.165;Apatura ilia;0.956796812031481;
MN139580;Lepidoptera;Polyommatus dolus;Polyommatus dolus;0.004;Polyommatus dolus;0.876833853168453;
MN139582;Lepidoptera;Erebia oeme;NA;0.114;Erebia oeme;0.999999999830635;
MN139583;Lepidoptera;Anthocharis cardamines;Anthocharis cardamines;1;Anthocharis cardamines;0.999999805933546;
MN139584;Lepidoptera;Melitaea didyma;Melitaea didyma;1;Melitaea didyma;0.999870115406424;
MN139585;Lepidoptera;Brenthis daphne;Brenthis daphne;0.006;Brenthis daphne;0.984559944066479;
MN139586;Lepidoptera;Thymelicus lineola;Thymelicus lineola;1;Thymelicus lineola;0.999997716763366;
MN139589;Lepidoptera;Pieris napi;NA;0.011;Pieris napi;0.887274591866313;
MN139590;Lepidoptera;Brenthis ino;Brenthis ino;1;Brenthis ino;0.999575084556096;
MN139591;Lepidoptera;Aporia crataegi;NA;0.003;Aporia crataegi;0.999999100143817;
MN139592;Lepidoptera;Anthocharis cardamines;Anthocharis cardamines;1;Anthocharis cardamines;0.99999995961909;
MN139593;Lepidoptera;Colias hyale;Colias hyale;0.015;Colias hyale;0.999997884296128;
MN139594;Lepidoptera;Maniola jurtina;NA;0.001;Maniola jurtina;0.939220105860875;
MN139595;Lepidoptera;Glaucopsyche alexis;Glaucopsyche alexis;0.001;Glaucopsyche alexis;0.963789545207295;
MN139597;Lepidoptera;Pieris napi;NA;0.011;Pieris napi;0.801985394724814;
MN139598;Lepidoptera;Colias hyale;Colias hyale;0.015;Colias hyale;0.999997884296128;
MN139599;Lepidoptera;Maniola jurtina;NA;NA;NA;0.99999999991;

MN139600;Lepidoptera;Minois dryas;NA;0.025;Minois dryas;0.850746026247397;
MN139601;Lepidoptera;Polyommatus icarus;NA;0.166;Polyommatus icarus;0.749935576218847;
MN139601;Lepidoptera;Polyommatus icarus;NA;0.166;Polyommatus icarus;0.749935576218847;
MN139602;Lepidoptera;Vanessa atalanta;Vanessa atalanta;1;Vanessa atalanta;0.999999998973152;
MN139604;Lepidoptera;Gegenes nostrodamus;Gegenes nostrodamus;1;Gegenes nostrodamus;0.999999996883645;
MN139607;Lepidoptera;Argynnis aglaja;NA;NA;NA;0.999999999998051;
MN139608;Lepidoptera;Colias hyale;Colias hyale;0.015;Colias hyale;0.999997884296128;
MN139609;Lepidoptera;Coenonympha arcania;NA;0.037;Coenonympha gardetta;0.702268810129899;
MN139610;Lepidoptera;Callophrys rubi;NA;0.001;Callophrys rubi;0.99870518510598;
MN139611;Lepidoptera;Erebia euryale;NA;NA;Erebia euryale;0.90919204573005;
MN139612;Lepidoptera;Coenonympha pamphilus;Coenonympha pamphilus;1;Coenonympha pamphilus;0.999999999999545;
MN139613;Lepidoptera;Brintesia circe;Brintesia circe;NA;Brintesia circe;0.99999894309325;
MN139614;Lepidoptera;Pyrgus carthami;Pyrgus carthami;1;Pyrgus carthami;0.999998997209607;
MN139615;Lepidoptera;Anthocharis cardamines;Anthocharis cardamines;1;Anthocharis cardamines;0.999999982121466;
MN139616;Lepidoptera;Brenthis daphne;Brenthis daphne;1;Brenthis daphne;0.998584732066774;
MN139618;Lepidoptera;Maniola jurtina;NA;0.012;Maniola jurtina;0.91755716175482;
MN139619;Lepidoptera;Polyommatus icarus;NA;0.13;Polyommatus icarus;0.979762695748748;
MN139619;Lepidoptera;Polyommatus icarus;NA;0.13;Polyommatus icarus;0.979762695748748;
MN139620;Lepidoptera;Melitaea diamina;Melitaea diamina;0.002;Melitaea diamina;0.999999985187884;
MN139622;Lepidoptera;Maniola cecilia;Pyronia cecilia;1;Pyronia cecilia;1;
MN139624;Lepidoptera;Ochlodes sylvanus;NA;0.316;Ochlodes sylvanus;0.996318754838625;
MN139625;Lepidoptera;Pyrgus malvoides;NA;0.168;Pyrgus malvoides;0.939641056696634;
MN139626;Lepidoptera;Pyrgus armoricanus;Pyrgus armoricanus;1;Pyrgus armoricanus;0.999997353848817;
MN139627;Lepidoptera;Callophrys rubi;NA;0.019;Callophrys rubi;0.998201459637982;
MN139628;Lepidoptera;Polyommatus celina;NA;0.072;Polyommatus celina;0.999999999953559;
MN139629;Lepidoptera;Pieris napi;NA;0.011;Pieris napi;0.801985394724814;
MN139630;Lepidoptera;Melitaea varia;Melitaea varia;1;Melitaea varia;0.999998939515614;
MN139631;Lepidoptera;Lysandra coridon;Lysandra coridon;NA;NA;0.99554973499116;
MN139632;Lepidoptera;Vanessa cardui;Vanessa cardui;1;Vanessa cardui;0.999999459983318;
MN139633;Lepidoptera;Cupido alcetas;NA;0.218;Cupido alcetas;0.833501983138177;
MN139635;Lepidoptera;Fabriciana niobe;NA;0.009;Fabriciana niobe;0.989552124216525;
MN139636;Lepidoptera;Satyrium esculi;Satyrium esculi;1;Satyrium esculi;0.999999957367948;
MN139637;Lepidoptera;Gegenes nostrodamus;Gegenes nostrodamus;1;Gegenes nostrodamus;0.999999996883645;
MN139639;Lepidoptera;Lasiommata maera;Lasiommata maera;1;Lasiommata maera;0.99999677384159;
MN139640;Lepidoptera;Thymelicus lineola;Thymelicus lineola;1;Thymelicus lineola;0.999995927238524;
MN139641;Lepidoptera;Erynnis tages;Erynnis tages;1;Erynnis tages;0.999999281987018;
MN139642;Lepidoptera;Hipparchia semele;NA;NA;Hipparchia semele;0.860254543558736;
MN139643;Lepidoptera;Polyommatus dolus;Polyommatus dolus;0.997;Polyommatus dolus;0.955195871823379;
MN139644;Lepidoptera;Melitaea diamina;NA;0.149;Melitaea diamina;0.999999996592464;
MN139646;Lepidoptera;Erebia medusa;NA;0.01;Erebia medusa;0.979621232162315;
MN139648;Lepidoptera;Fabriciana adippe;Fabriciana adippe;0.007;Fabriciana adippe;0.876018745965171;
MN139650;Lepidoptera;Melanargia galathea;Melanargia galathea;NA;Melanargia galathea;0.959429932872284;
MN139652;Lepidoptera;Euphydryas aurinia;NA;0.001;Euphydryas aurinia;0.984636740722975;
MN139653;Lepidoptera;Aporia crataegi;NA;NA;Aporia crataegi;0.999996781126733;
MN139654;Lepidoptera;Erebia scipio;Erebia scipio;1;Erebia scipio;0.999999995588723;
MN139655;Lepidoptera;Brenthis daphne;Brenthis daphne;0.001;Brenthis daphne;0.997781825264304;
MN139659;Lepidoptera;Ochlodes sylvanus;NA;0.316;Ochlodes sylvanus;0.996318754838625;
MN139660;Lepidoptera;Coenonympha corinna;Coenonympha corinna;1;Coenonympha corinna;0.999999999974449;
MN139661;Lepidoptera;Maniola jurtina;NA;0.012;Maniola jurtina;0.91755716175482;
MN139662;Lepidoptera;Coenonympha dorus;Coenonympha dorus;1;Coenonympha dorus;0.99999999983973;
MN139663;Lepidoptera;Erynnis tages;Erynnis tages;1;Erynnis tages;0.999999253822119;
MN139664;Lepidoptera;Gonepteryx rhamni;NA;0.192;Gonepteryx rhamni;0.991002175992451;
MN139667;Lepidoptera;Pyrgus cacaliae;Pyrgus cacaliae;0.999;Pyrgus cacaliae;0.99258001670677;
MN139668;Lepidoptera;Erebia pharte;Erebia pharte;1;Erebia pharte;0.99999999630495;
MN139669;Lepidoptera;Callophrys rubi;NA;0.001;Callophrys rubi;0.99870518510598;
MN139670;Lepidoptera;Hipparchia neapolitana;NA;0.001;Hipparchia semele;0.802446092830354;
MN139671;Lepidoptera;Lampides boeticus;Lampides boeticus;1;Lampides boeticus;0.999999999545906;
MN139674;Lepidoptera;Thymelicus lineola;Thymelicus lineola;1;Thymelicus lineola;0.999971535093703;
MN139675;Lepidoptera;Thymelicus acteon;Thymelicus acteon;1;Thymelicus acteon;0.99999999752248;
MN139676;Lepidoptera;Pyrgus armoricanus;Pyrgus armoricanus;1;Pyrgus armoricanus;0.999997353848817;
MN139678;Lepidoptera;Lycaena alciphron;Lycaena alciphron;1;Lycaena alciphron;0.999999934247401;
MN139679;Lepidoptera;Aricia agestis;NA;0.067;Aricia agestis;0.999509743227786;
MN139680;Lepidoptera;Melitaea cinxia;NA;0.344;Melitaea cinxia;0.999999999998636;
MN139681;Lepidoptera;Coenonympha pamphilus;Coenonympha pamphilus;1;Coenonympha pamphilus;0.999999999999744;
MN139682;Lepidoptera;Erebia epiphron;NA;NA;Erebia epiphron;0.991535930833796;

MN139683;Lepidoptera;Hipparchia hermione;NA;NA;NA;0.999999999999991;
MN139686;Lepidoptera;Polyommatus dorylas;NA;0.002;Polyommatus dorylas;0.992103989657325;
MN139687;Lepidoptera;Erebia euryale;NA;NA;Erebia euryale;0.920428872281508;
MN139688;Lepidoptera;Ochlodes sylvanus;NA;0.316;Ochlodes sylvanus;0.996318754838625;
MN139689;Lepidoptera;Glaucopsyche alexis;NA;NA;Glaucopsyche alexis;0.991019528246159;
MN139691;Lepidoptera;Erebia euryale;NA;NA;Erebia euryale;0.919518760623792;
MN139692;Lepidoptera;Cyaniris semiargus;Cyaniris semiargus;NA;Cyaniris semiargus;0.999795734354061;
MN139693;Lepidoptera;Callophrys rubi;NA;0.009;Callophrys rubi;0.996560650629412;
MN139697;Lepidoptera;Favonius quercus;NA;0.021;Favonius quercus;0.742525423285576;
MN139698;Lepidoptera;Satyrium spini;Satyrium spini;1;Satyrium spini;0.999999721054916;
MN139699;Lepidoptera;Maniola jurtina;NA;NA;NA;0.99999999985062;
MN139700;Lepidoptera;Lasiommata megera;Lasiommata megera;1;Lasiommata megera;0.999928528239676;
MN139703;Lepidoptera;Cyaniris semiargus;Cyaniris semiargus;NA;Cyaniris semiargus;0.999795734354061;
MN139704;Lepidoptera;Brintesia circe;Brintesia circe;1;Brintesia circe;0.999997995192124;
MN139705;Lepidoptera;Lycaena tityrus;Lycaena tityrus;1;Lycaena tityrus;0.999998928238034;
MN139706;Lepidoptera;Lasiommata maera;Lasiommata maera;1;Lasiommata maera;0.99999510792253;
MN139707;Lepidoptera;Satyrus actaea;Satyrus actaea;0.01;Satyrus actaea;0.94366382029787;
MN139708;Lepidoptera;Brenthis daphne;Brenthis daphne;0.006;Brenthis daphne;0.984559944066479;
MN139709;Lepidoptera;Anthocharis cardamines;Anthocharis cardamines;1;Anthocharis cardamines;0.999999940675226;
MN139711;Lepidoptera;Iolana iolas;Iolana iolas;1;Iolana iolas;0.999999106389198;
MN139712;Lepidoptera;Melitaea parthenoides;Melitaea parthenoides;1;Melitaea parthenoides;0.999999968152765;
MN139713;Lepidoptera;Lampides boeticus;Lampides boeticus;1;Lampides boeticus;0.999999999545906;
MN139715;Lepidoptera;Coenonympha arcania;NA;0.002;NA;0.999999999999934;
MN139716;Lepidoptera;Polyommatus icarus;Polyommatus icarus;0.001;Polyommatus icarus;0.999851081176144;
MN139716;Lepidoptera;Polyommatus icarus;Polyommatus icarus;0.001;Polyommatus icarus;0.999851081176144;
MN139717;Lepidoptera;Nymphalis antiopa;NA;0.23;Nymphalis antiopa;0.999999944198096;
MN139718;Lepidoptera;Cyaniris semiargus;Cyaniris semiargus;NA;Cyaniris semiargus;0.99969577444782;
MN139719;Lepidoptera;Fabriciana niobe;NA;0.004;Fabriciana niobe;0.989536562020899;
MN139720;Lepidoptera;Pieris napi;NA;0.028;Pieris napi;0.888205720664699;
MN139721;Lepidoptera;Brintesia circe;Brintesia circe;1;Brintesia circe;0.99999951254154;
MN139722;Lepidoptera;Plebejus idas;NA;0.007;NA;0.999546324331549;
MN139723;Lepidoptera;Thymelicus sylvestris;NA;0.324;Thymelicus sylvestris;0.999940778442454;
MN139724;Lepidoptera;Polyommatus icarus;NA;0.048;Polyommatus icarus;0.976828298825372;
MN139724;Lepidoptera;Polyommatus icarus;NA;0.048;Polyommatus icarus;0.976828298825372;
MN139725;Lepidoptera;Fabriciana niobe;NA;0.005;Fabriciana niobe;0.992125835358755;
MN139726;Lepidoptera;Argynnis aglaja;NA;NA;NA;0.999999999998051;
MN139728;Lepidoptera;Lasiommata maera;Lasiommata maera;1;Lasiommata maera;0.999997945450226;
MN139731;Lepidoptera;Maniola jurtina;NA;0.063;Maniola jurtina;0.770025363931686;
MN139732;Lepidoptera;Apatura ilia;NA;0.165;Apatura ilia;0.956796812031481;
MN139734;Lepidoptera;Satyrium pruni;NA;0.076;Satyrium pruni;0.990322364021655;
MN139735;Lepidoptera;Pieris napi;NA;0.028;Pieris napi;0.888205720664699;
MN139736;Lepidoptera;Pyrgus onopordi;Pyrgus onopordi;1;Pyrgus onopordi;0.999997111229755;
MN139738;Lepidoptera;Maniola jurtina;NA;NA;Maniola jurtina;0.950689021412593;
MN139740;Lepidoptera;Ochlodes sylvanus;NA;0.316;Ochlodes sylvanus;0.996318754838625;
MN139743;Lepidoptera;Cupido argiades;NA;0.499;NA;0.999999999984682;
MN139745;Lepidoptera;Colias alfacariensis;Colias alfacariensis;0.001;Colias alfacariensis;0.999926026317526;
MN139749;Lepidoptera;Aricia agestis;NA;0.253;Aricia agestis;0.996350017387504;
MN139750;Lepidoptera;Brenthis daphne;Brenthis daphne;0.999;Brenthis daphne;0.99795709566592;
MN139751;Lepidoptera;Thymelicus acteon;Thymelicus acteon;1;Thymelicus acteon;0.99999999752248;
MN139752;Lepidoptera;Coenonympha glycerion;NA;0.28;Coenonympha glycerion;0.999998036263913;
MN139755;Lepidoptera;Thecla betulae;Thecla betulae;1;Thecla betulae;0.999999999972886;
MN139756;Lepidoptera;Maniola jurtina;NA;0.171;Maniola jurtina;0.963697912386445;
MN139757;Lepidoptera;Lysandra coridon;NA;NA;NA;0.998017994491822;
MN139759;Lepidoptera;Erebia tyndarus;NA;NA;NA;0.999999999999783;
MN139760;Lepidoptera;Lasiommata maera;Lasiommata maera;1;Lasiommata maera;0.99999776398485;
MN139761;Lepidoptera;Aporia crataegi;NA;0.004;Aporia crataegi;0.999984184707536;
MN139763;Lepidoptera;Polyommatus celina;Polyommatus celina;0.002;Polyommatus celina;0.999999997980922;
MN139764;Lepidoptera;Ochlodes sylvanus;NA;0.316;Ochlodes sylvanus;0.996318754838625;
MN139765;Lepidoptera;Pieris napi;NA;0.028;Pieris napi;0.888205720664699;
MN139766;Lepidoptera;Gonepteryx cleopatra;Gonepteryx cleopatra;0.998;Gonepteryx cleopatra;0.999118636582266;
MN139767;Lepidoptera;Boloria euphrosyne;Boloria euphrosyne;1;Boloria euphrosyne;0.999994787623591;
MN139769;Lepidoptera;Aricia cramera;Aricia cramera;1;Aricia cramera;0.999993731037033;
MN139770;Lepidoptera;Polyommatus damon;Polyommatus damon;1;Polyommatus damon;0.999997386150488;
MN139771;Lepidoptera;Coenonympha oedippus;Coenonympha oedippus;1;Coenonympha oedippus;0.999999999999488;
MN139773;Lepidoptera;Maniola jurtina;NA;0.012;Maniola jurtina;0.91755716175482;
MN139775;Lepidoptera;Vanessa cardui;Vanessa cardui;1;Vanessa cardui;0.999996792914969;

MN139776;Lepidoptera;Hipparchia hermione;NA;NA;NA;0.999999999999991;
MN139777;Lepidoptera;Satyrium acaciae;Satyrium acaciae;1;Satyrium acaciae;0.999999951077798;
MN139778;Lepidoptera;Agriades orbitulus;Agriades orbitulus;NA;Agriades orbitulus;0.999216256740517;
MN139779;Lepidoptera;Callophrys rubi;NA;0.019;Callophrys rubi;0.998201459637982;
MN139780;Lepidoptera;Nymphalis c-album;NA;0.215;NA;0.999999192918557;
MN139781;Lepidoptera;Coenonympha gardetta;Coenonympha gardetta;0.001;Coenonympha gardetta;0.891527308362633;
MN139782;Lepidoptera;Plebejus argus;NA;0.328;Plebejus argus;0.969789734199889;
MN139785;Lepidoptera;Vanessa cardui;Vanessa cardui;1;Vanessa cardui;0.999997579965112;
MN139786;Lepidoptera;Erebia melampus;Erebia melampus;NA;Erebia melampus;0.950990028966024;
MN139789;Lepidoptera;Satyrium ilicis;Satyrium ilicis;1;Satyrium ilicis;0.999999987901305;
MN139790;Lepidoptera;Lasiommata megera;Lasiommata megera;1;Lasiommata megera;0.999379144055441;
MN139792;Lepidoptera;Maniola tithonus;Pyronia tithonus;1;Pyronia tithonus;1;
MN139793;Lepidoptera;Aricia cramera;Aricia cramera;1;Aricia cramera;0.999969749613305;
MN139794;Lepidoptera;Fabriciana adippe;Fabriciana adippe;0.002;Fabriciana adippe;0.994515464870225;
MN139795;Lepidoptera;Lycaena phlaeas;Lycaena phlaeas;1;Lycaena phlaeas;0.999999996363186;
MN139796;Lepidoptera;Glaucopsyche alexis;Glaucopsyche alexis;0.001;Glaucopsyche alexis;0.94042380088325;
MN139798;Lepidoptera;Thymelicus sylvestris;Thymelicus sylvestris;1;Thymelicus sylvestris;0.999999985935205;
MN139801;Lepidoptera;Euphydryas aurinia;NA;0.106;Euphydryas aurinia;0.993939072040577;
MN139802;Lepidoptera;Plebejus argus;NA;0.043;Plebejus argus;0.956187234366662;
MN139803;Lepidoptera;Erebia neoridas;Erebia neoridas;NA;Erebia neoridas;0.998631936578793;
MN139804;Lepidoptera;Cupido alcetas;NA;0.218;Cupido alcetas;0.833501983138177;
MN139805;Lepidoptera;Hipparchia leighebi;Hipparchia leighebi;0.002;Hipparchia leighebi;0.856866627382455;
MN139807;Lepidoptera;Aricia cramera;Aricia cramera;1;Aricia cramera;0.999993731037033;
MN139811;Lepidoptera;Maniola jurtina;NA;0.063;Maniola jurtina;0.770025363931686;
MN139813;Lepidoptera;Melitaea cinxia;Melitaea cinxia;1;Melitaea cinxia;0.999999999999403;
MN139815;Lepidoptera;Melitaea didyma;Melitaea didyma;1;Melitaea didyma;0.999870090903784;
MN139817;Lepidoptera;Argynnis aglaja;NA;NA;Speyeria aglaja;0.872991147917853;
MN139819;Lepidoptera;Hipparchia semele;NA;NA;Hipparchia semele;0.748345401572611;
MN139823;Lepidoptera;Lycaena phlaeas;Lycaena phlaeas;1;Lycaena phlaeas;0.999999996363186;
MN139824;Lepidoptera;Maniola cecilia;Pyronia cecilia;NA;Pyronia cecilia;1;
MN139825;Lepidoptera;Nymphalis c-album;NA;0.215;NA;0.999999192918557;
MN139826;Lepidoptera;Callophrys rubi;NA;0.019;Callophrys rubi;0.998201459637982;
MN139828;Lepidoptera;Lasiommata megera;Lasiommata megera;1;Lasiommata megera;0.999379144055441;
MN139829;Lepidoptera;Melitaea cinxia;Melitaea cinxia;1;Melitaea cinxia;0.999999999999801;
MN139830;Lepidoptera;Pyrgus malvoides;NA;0.155;Pyrgus malvoides;0.943212125204022;
MN139831;Lepidoptera;Lycaena phlaeas;Lycaena phlaeas;1;Lycaena phlaeas;0.999999996363186;
MN139832;Lepidoptera;Aricia cramera;Aricia cramera;1;Aricia cramera;0.999993731037033;
MN139833;Lepidoptera;Aglais urticae;Aglais urticae;0.001;Aglais urticae;0.994396335177485;
MN139834;Lepidoptera;Apatura ilia;NA;0.039;Apatura ilia;0.977274423026401;
MN139835;Lepidoptera;Melitaea parthenoides;Melitaea parthenoides;1;Melitaea parthenoides;0.99999998877297;
MN139836;Lepidoptera;Colias alfacariensis;Colias alfacariensis;1;Colias alfacariensis;0.999986994131001;
MN139838;Lepidoptera;Maniola jurtina;NA;0.004;Maniola jurtina;0.945262064633298;
MN139839;Lepidoptera;Hipparchia leighebi;Hipparchia leighebi;0.001;Hipparchia leighebi;0.858290018061167;
MN139840;Lepidoptera;Melitaea didyma;Melitaea didyma;1;Melitaea didyma;0.999945816868155;
MN139841;Lepidoptera;Melitaea britomartis;Mellicta britomartis;NA;Melitaea britomartis;0.935497948168019;
MN139842;Lepidoptera;Ochlodes sylvanus;NA;0.316;Ochlodes sylvanus;0.996318754838625;
MN139843;Lepidoptera;Fabriciana niobe;Fabriciana niobe;0.001;Fabriciana niobe;0.93186202957215;
MN139844;Lepidoptera;Hipparchia fagi;Hipparchia fagi;NA;Hipparchia fagi;0.985731526452115;
MN139848;Lepidoptera;Thymelicus acteon;Thymelicus acteon;1;Thymelicus acteon;0.99999999752248;
MN139851;Lepidoptera;Polyommatus damon;Polyommatus damon;1;Polyommatus damon;0.999999916924512;
MN139854;Lepidoptera;Pyrgus malvoides;NA;0.076;Pyrgus malvoides;0.887854315566122;
MN139855;Lepidoptera;Erebia medusa;NA;0.009;Erebia medusa;0.986275612856827;
MN139856;Lepidoptera;Erebia euryale;NA;NA;Erebia euryale;0.985600540820829;
MN139857;Lepidoptera;Aporia crataegi;NA;0.009;Aporia crataegi;0.999996613335036;
MN139858;Lepidoptera;Colias crocea;NA;NA;NA;0.99999999999969;
MN139859;Lepidoptera;Pyrgus carthami;Pyrgus carthami;1;Pyrgus carthami;0.999995592447668;
MN139860;Lepidoptera;Coenonympha pamphilus;Coenonympha pamphilus;1;Coenonympha pamphilus;0.999999999999432;
MN139862;Lepidoptera;Pieris napi;NA;0.028;Pieris napi;0.888205720664699;
MN139863;Lepidoptera;Coenonympha corinna;Coenonympha corinna;1;Coenonympha corinna;0.999999999974449;
MN139864;Lepidoptera;Pieris napi;NA;0.028;Pieris napi;0.888205720664699;
MN139865;Lepidoptera;Nymphalis c-album;NA;0.215;NA;0.999999192918557;
MN139867;Lepidoptera;Aricia eumedon;Eumedonia eumedon;0.001;Eumedonia eumedon;0.997789624617489;
MN139871;Lepidoptera;Boloria titania;Boloria titania;0.001;Boloria titania;0.99983358374111;
MN139873;Lepidoptera;Maniola jurtina;NA;0.012;Maniola jurtina;0.91755716175482;
MN139874;Lepidoptera;Maniola tithonus;Pyronia tithonus;1;Pyronia tithonus;1;
MN139875;Lepidoptera;Colias crocea;NA;0.059;NA;0.999999999999738;

MN139876;Lepidoptera;Hipparchia hermione;Hipparchia hermione;NA;Hipparchia hermione;0.726013733187223;
MN139877;Lepidoptera;Cupido alcetas;NA;0.218;Cupido alcetas;0.833501983138177;
MN139878;Lepidoptera;Papilio machaon;Papilio machaon;0.001;Papilio machaon;0.999463129150422;
MN139880;Lepidoptera;Maniola jurtina;NA;0.001;Maniola jurtina;0.939220105860875;
MN139881;Lepidoptera;Agriades optilete;Agriades optilete;1;Agriades optilete;0.999999993460563;
MN139882;Lepidoptera;Glaucopsyche alexis;Glaucopsyche alexis;0.001;Glaucopsyche alexis;0.94042380088325;
MN139883;Lepidoptera;Pieris napi;NA;0.011;Pieris napi;0.801985394724814;
MN139884;Lepidoptera;Ochlodes sylvanus;NA;0.316;Ochlodes sylvanus;0.996318754838625;
MN139886;Lepidoptera;Argynnis aglaja;NA;NA;Speyeria aglaja;0.872991147917853;
MN139887;Lepidoptera;Coenonympha pamphilus;Coenonympha pamphilus;1;Coenonympha pamphilus;0.999999999990962;
MN139889;Lepidoptera;Carterocephalus palaemon;NA;0.052;Carterocephalus palaemon;0.728445051795325;
MN139890;Lepidoptera;Erebia pluto;Erebia pluto;1;Erebia pluto;0.999999696335028;
MN139891;Lepidoptera;Melitaea cinxia;Melitaea cinxia;1;Melitaea cinxia;0.999999999999801;
MN139893;Lepidoptera;Thymelicus acteon;Thymelicus acteon;1;Thymelicus acteon;0.999999998015312;
MN139894;Lepidoptera;Pyrgus armoricanus;Pyrgus armoricanus;1;Pyrgus armoricanus;0.999996875500834;
MN139895;Lepidoptera;Pyrgus malvoides;NA;0.066;Pyrgus malvoides;0.960778066111538;
MN139896;Lepidoptera;Argynnis aglaja;NA;NA;Speyeria aglaja;0.872991147917853;
MN139897;Lepidoptera;Lasiommata maera;Lasiommata maera;1;Lasiommata maera;0.99999510792253;
MN139899;Lepidoptera;Brintesia circe;Brintesia circe;NA;Brintesia circe;0.999999813082031;
MN139900;Lepidoptera;Hipparchia aristaeus;Hipparchia aristaeus;1;Hipparchia aristaeus;0.999999993824275;
MN139901;Lepidoptera;Brenthis daphne;Brenthis daphne;0.001;Brenthis daphne;0.997781825264304;
MN139902;Lepidoptera;Argynnis aglaja;NA;NA;Speyeria aglaja;0.901634087394819;
MN139903;Lepidoptera;Aporia crataegi;NA;NA;Aporia crataegi;0.999998459680561;
MN139904;Lepidoptera;Melitaea trivia;Melitaea trivia;1;Melitaea trivia;0.99999999990402;
MN139905;Lepidoptera;Boloria titania;Boloria titania;0.001;Boloria titania;0.999477346009661;
MN139906;Lepidoptera;Aricia cramera;Aricia cramera;1;Aricia cramera;0.999993731037033;
MN139910;Lepidoptera;Polyommatus icarus;NA;0.003;Polyommatus icarus;0.986125651677522;
MN139910;Lepidoptera;Polyommatus icarus;NA;0.003;Polyommatus icarus;0.986125651677522;
MN139912;Lepidoptera;Lasiommata maera;Lasiommata maera;1;Lasiommata maera;0.999996552555305;
MN139913;Lepidoptera;Favonius quercus;Favonius quercus;NA;Favonius quercus;0.856713594860622;
MN139914;Lepidoptera;Glaucopsyche alexis;Glaucopsyche alexis;0.001;Glaucopsyche alexis;0.94042380088325;
MN139915;Lepidoptera;Maniola jurtina;NA;0.012;Maniola jurtina;0.91755716175482;
MN139916;Lepidoptera;Melitaea cinxia;Melitaea cinxia;1;Melitaea cinxia;0.999999999999773;
MN139917;Lepidoptera;Polyommatus damon;Polyommatus damon;1;Polyommatus damon;0.999999897832231;
MN139919;Lepidoptera;Hipparchia semele;NA;NA;Hipparchia semele;0.748345401572611;
MN139920;Lepidoptera;Erynnis tages;Erynnis tages;1;Erynnis tages;0.999955880826156;
MN139921;Lepidoptera;Polyommatus icarus;Polyommatus icarus;0.001;Polyommatus icarus;0.999851522482238;
MN139921;Lepidoptera;Polyommatus icarus;Polyommatus icarus;0.001;Polyommatus icarus;0.999851522482238;
MN139922;Lepidoptera;Pyrgus carthami;Pyrgus carthami;1;Pyrgus carthami;0.99999551310754;
MN139924;Lepidoptera;Melitaea didyma;Melitaea didyma;1;Melitaea didyma;0.995729075061249;
MN139926;Lepidoptera;Aricia agestis;NA;0.069;Aricia agestis;0.995918114091591;
MN139927;Lepidoptera;Brintesia circe;Brintesia circe;1;Brintesia circe;0.99999951254154;
MN139929;Lepidoptera;Lampides boeticus;Lampides boeticus;1;Lampides boeticus;0.999999999545906;
MN139931;Lepidoptera;Colias alfacariensis;Colias alfacariensis;0.001;Colias alfacariensis;0.999926026317526;
MN139932;Lepidoptera;Maniola jurtina;NA;0.001;Maniola jurtina;0.939220105860875;
MN139933;Lepidoptera;Hipparchia fagi;NA;NA;Hipparchia fagi;0.958291584360022;
MN139934;Lepidoptera;Minois dryas;NA;0.025;Minois dryas;0.850746026247397;
MN139935;Lepidoptera;Lampides boeticus;Lampides boeticus;1;Lampides boeticus;0.99999999935136;
MN139936;Lepidoptera;Vanessa atalanta;Vanessa atalanta;1;Vanessa atalanta;0.999999999383476;
MN139938;Lepidoptera;Thymelicus lineola;Thymelicus lineola;1;Thymelicus lineola;0.999971535093703;
MN139939;Lepidoptera;Brenthis ino;Brenthis ino;0.001;Brenthis ino;0.994711912827522;
MN139940;Lepidoptera;Lycaena tityrus;Lycaena tityrus;1;Lycaena tityrus;0.999997855580031;
MN139942;Lepidoptera;Hipparchia hermione;Hipparchia hermione;1;Hipparchia hermione;0.999956033895189;
MN139944;Lepidoptera;Lysandra coridon;NA;NA;NA;0.999076788188261;
MN139946;Lepidoptera;Coenonympha pamphilus;Coenonympha pamphilus;1;Coenonympha pamphilus;0.999999999999744;
MN139947;Lepidoptera;Coenonympha arcania;NA;0.172;Coenonympha arcania;0.963036309473038;
MN139948;Lepidoptera;Melitaea didyma;Melitaea didyma;1;Melitaea didyma;0.999870115406424;
MN139949;Lepidoptera;Celastrina argiolus;Celastrina argiolus;1;Celastrina argiolus;0.999527385150531;
MN139950;Lepidoptera;Thymelicus acteon;Thymelicus acteon;1;Thymelicus acteon;0.99999999752248;
MN139954;Lepidoptera;Aricia artaxerxes;Plebejus argus;1;Aricia artaxerxes;0.999743187199922;
MN139955;Lepidoptera;Pyrgus alveus;NA;0.003;NA;0.999999999997732;
MN139956;Lepidoptera;Euphydryas intermedia;Euphydryas intermedia;0.017;Euphydryas intermedia;0.770732210581036;

MN139957;Lepidoptera;Anthocharis cardamines;Anthocharis cardamines;1;Anthocharis cardamines;0.999999933384063;
MN139958;Lepidoptera;Lasiommata megera;Lasiommata megera;0.001;Lasiommata megera;0.999458259886141;
MN139962;Lepidoptera;Colias alfacariensis;Colias alfacariensis;0.001;Colias alfacariensis;0.999926026317526;
MN139963;Lepidoptera;Brintesia circe;Brintesia circe;1;Brintesia circe;0.99999951254154;
MN139965;Lepidoptera;Polyommatus dorylas;Polyommatus dorylas;0.008;Polyommatus dorylas;0.991281865899703;
MN139966;Lepidoptera;Colias crocea;NA;0.059;NA;0.999999999999738;
MN139967;Lepidoptera;Melitaea didyma;Melitaea didyma;1;Melitaea didyma;0.997852099274181;
MN139969;Lepidoptera;Melitaea cinxia;NA;0.055;Melitaea cinxia;0.99999999999838;
MN139974;Lepidoptera;Pyrgus alveus;NA;0.003;NA;0.999999999997732;
MN139975;Lepidoptera;Erebia medusa;NA;0.04;Erebia medusa;0.984042649596477;
MN139976;Lepidoptera;Boloria thore;Boloria thore;1;Boloria thore;0.99999987376814;
MN139977;Lepidoptera;Erebia pronoe;Erebia pronoe;NA;Erebia pronoe;0.790922387957511;
MN139978;Lepidoptera;Polyommatus icarus;NA;0.13;Polyommatus icarus;0.979762695748748;
MN139978;Lepidoptera;Polyommatus icarus;NA;0.13;Polyommatus icarus;0.979762695748748;
MN139979;Lepidoptera;Polyommatus icarus;Polyommatus icarus;0.001;Polyommatus icarus;0.999821128709045;
MN139979;Lepidoptera;Polyommatus icarus;Polyommatus icarus;0.001;Polyommatus icarus;0.999821128709045;
MN139982;Lepidoptera;Melitaea cinxia;Melitaea cinxia;1;Melitaea cinxia;0.999999999999829;
MN139983;Lepidoptera;Lasiommata maera;Lasiommata maera;1;Lasiommata maera;0.99999510792253;
MN139984;Lepidoptera;Lasiommata maera;Lasiommata maera;1;Lasiommata maera;0.99999510792253;
MN139985;Lepidoptera;Pyrgus sidae;Pyrgus sidae;1;Pyrgus sidae;0.999973016525821;
MN139986;Lepidoptera;Coenonympha oedippus;Coenonympha oedippus;1;Coenonympha oedippus;0.999999999999972;
MN139987;Lepidoptera;Polyommatus icarus;Polyommatus icarus;0.001;Polyommatus icarus;0.999851522482238;
MN139987;Lepidoptera;Polyommatus icarus;Polyommatus icarus;0.001;Polyommatus icarus;0.999851522482238;
MN139988;Lepidoptera;Euphydryas aurinia;NA;0.31;Euphydryas aurinia;0.993387708761386;
MN139989;Lepidoptera;Brenthis daphne;NA;0.102;Brenthis daphne;0.717134521611551;
MN139991;Lepidoptera;Brenthis daphne;Brenthis daphne;0.003;Brenthis daphne;0.984519516505019;
MN139992;Lepidoptera;Maniola cecilia;Pyronia cecilia;1;Pyronia cecilia;1;
MN139993;Lepidoptera;Brintesia circe;Brintesia circe;1;Brintesia circe;0.99999951254154;
MN139997;Lepidoptera;Fabriciana elisa;Fabriciana elisa;0.001;Fabriciana elisa;0.994839902224823;
MN139998;Lepidoptera;Glaucopsyche melanops;Glaucopsyche melanops;1;Glaucopsyche melanops;0.999952924254607;
MN139999;Lepidoptera;Colias crocea;NA;0.059;NA;0.999999999999738;
MN140000;Lepidoptera;Nymphalis egea;Polygonia egea;1;Polygonia egea;0.999999758078475;
MN140001;Lepidoptera;Maniola jurtina;NA;0.001;Maniola jurtina;0.939220105860875;
MN140002;Lepidoptera;Pyrgus alveus;NA;NA;NA;0.999999999994797;
MN140003;Lepidoptera;Coenonympha pamphilus;Coenonympha pamphilus;1;Coenonympha pamphilus;0.999999999999744;
MN140005;Lepidoptera;Pieris napi;NA;0.028;Pieris napi;0.888205720664699;
MN140006;Lepidoptera;Thymelicus sylvestris;NA;0.324;Thymelicus sylvestris;0.999940778442454;
MN140007;Lepidoptera;Coenonympha arcania;NA;0.002;NA;0.999999999999972;
MN140008;Lepidoptera;Celastrina argiolus;Celastrina argiolus;1;Celastrina argiolus;0.999527385150531;
MN140009;Lepidoptera;Lycaena virgaureae;NA;0.186;Lycaena virgaureae;0.999491227342155;
MN140012;Lepidoptera;Euphydryas aurinia;NA;0.106;Euphydryas aurinia;0.993939072040577;
MN140013;Lepidoptera;Polyommatus daphnis;NA;0.062;Polyommatus daphnis;0.995724017767077;
MN140015;Lepidoptera;Plebejus argus;NA;0.043;Plebejus argus;0.956187234366662;
MN140016;Lepidoptera;Carcharodus alceae;Carcharodus alceae;0.999;Carcharodus alceae;0.999999996339511;
MN140017;Lepidoptera;Erebia cassioides;NA;NA;NA;0.999999999999977;
MN140018;Lepidoptera;Erebia eriphyle;Erebia eriphyle;1;Erebia eriphyle;0.999999983979336;
MN140019;Lepidoptera;Euphydryas cynthia;Euphydryas cynthia;1;Euphydryas cynthia;0.999999999402945;
MN140021;Lepidoptera;Apatura iris;Apatura iris;1;Apatura iris;0.999999999999972;
MN140022;Lepidoptera;Ochlodes sylvanus;NA;0.316;Ochlodes sylvanus;0.996318754838625;
MN140024;Lepidoptera;Hipparchia fagi;NA;NA;Hipparchia fagi;0.90747740458264;
MN140025;Lepidoptera;Pyrgus malvoides;NA;0.176;Pyrgus malvoides;0.940490890379026;
MN140027;Lepidoptera;Aricia eumedon;Eumedonia eumedon;0.001;Eumedonia eumedon;0.99836395629257;
MN140028;Lepidoptera;Coenonympha arcania;NA;0.172;Coenonympha arcania;0.963036309473038;
MN140029;Lepidoptera;Lysandra coridon;NA;NA;Lysandra coridon;0.749217519592352;
MN140030;Lepidoptera;Fabriciana adippe;NA;NA;Fabriciana adippe;0.922280384106682;
MN140031;Lepidoptera;Aricia cramera;Aricia cramera;1;Aricia cramera;0.999993596642023;
MN140034;Lepidoptera;Aricia agestis;NA;0.067;Aricia agestis;0.999509743227786;
MN140035;Lepidoptera;Pyrgus armoricanus;Pyrgus armoricanus;1;Pyrgus armoricanus;0.999997534125241;
MN140037;Lepidoptera;Nymphalis antiopa;NA;0.083;Nymphalis antiopa;0.999999719556979;
MN140038;Lepidoptera;Satyrus ferula;Satyrus ferula;0.001;Satyrus ferula;0.992107254386621;
MN140039;Lepidoptera;Erebia pronoe;Erebia pronoe;1;Erebia pronoe;0.999250891771858;
MN140041;Lepidoptera;Thymelicus sylvestris;NA;0.324;Thymelicus sylvestris;0.999940778442454;
MN140042;Lepidoptera;Cupido minimus;NA;0.009;Cupido minimus;0.999791453712643;

MN140043;Lepidoptera;Maniola cecilia;Pyronia cecilia;NA;Pyronia cecilia;1;
MN140044;Lepidoptera;Argynnis aglaja;NA;NA;NA;0.999999999998051;
MN140045;Lepidoptera;Argynnis aglaja;NA;0.005;Speyeria aglaja;0.896438572419641;
MN140046;Lepidoptera;Carcharodus alceae;Carcharodus alceae;1;Carcharodus alceae;0.999999998465938;
MN140047;Lepidoptera;Colias alfacariensis;Colias alfacariensis;0.001;Colias alfacariensis;0.999926026317526;
MN140048;Lepidoptera;Pyrgus malvoides;NA;0.176;Pyrgus malvoides;0.940490890379026;
MN140049;Lepidoptera;Hipparchia semele;NA;0.003;Hipparchia semele;0.875557777102739;
MN140050;Lepidoptera;Lasiommata maera;Lasiommata maera;1;Lasiommata maera;0.999988599344549;
MN140051;Lepidoptera;Argynnis aglaja;NA;0.005;Speyeria aglaja;0.896438572419641;
MN140054;Lepidoptera;Pyrgus sidae;Pyrgus sidae;1;Pyrgus sidae;0.999994277634217;
MN140055;Lepidoptera;Melanargia galathea;Melanargia galathea;NA;Melanargia galathea;0.987293011345651;
MN140056;Lepidoptera;Gonepteryx rhamni;NA;0.474;Gonepteryx rhamni;0.982076396127858;
MN140057;Lepidoptera;Polyommatus daphnis;NA;0.062;Polyommatus daphnis;0.995724017767077;
MN140059;Lepidoptera;Coenonympha pamphilus;Coenonympha pamphilus;1;Coenonympha pamphilus;0.999999999997613;
MN140060;Lepidoptera;Arethusana arethusa;Arethusana arethusa;0.019;Arethusana arethusa;0.999964353716342;
MN140062;Lepidoptera;Lasiommata maera;Lasiommata maera;1;Lasiommata maera;0.999996200814449;
MN140064;Lepidoptera;Colias crocea;NA;NA;NA;0.999999999997968;
MN140066;Lepidoptera;Brintesia circe;Brintesia circe;1;Brintesia circe;0.999999855299773;
MN140070;Lepidoptera;Thymelicus lineola;Thymelicus lineola;1;Thymelicus lineola;0.999996248507716;
MN140071;Lepidoptera;Thymelicus sylvestris;Thymelicus sylvestris;1;Thymelicus sylvestris;0.999999985935205;
MN140072;Lepidoptera;Celastrina argiolus;Celastrina argiolus;1;Celastrina argiolus;0.999527385150531;
MN140074;Lepidoptera;Hipparchia semele;NA;NA;Hipparchia semele;0.748345401572611;
MN140075;Lepidoptera;Limenitis camilla;Limenitis camilla;0.002;Limenitis camilla;0.999986372375421;
MN140076;Lepidoptera;Pyrgus malvoides;NA;0.2;Pyrgus malvoides;0.91582520613586;
MN140077;Lepidoptera;Maniola jurtina;NA;0.001;Maniola jurtina;0.939220105860875;
MN140081;Lepidoptera;Vanessa atalanta;Vanessa atalanta;1;Vanessa atalanta;0.999999999757762;
MN140082;Lepidoptera;Satyrium ilicis;Satyrium ilicis;1;Satyrium ilicis;0.999999987901305;
MN140083;Lepidoptera;Anthocharis cardamines;Anthocharis cardamines;1;Anthocharis cardamines;0.999999917840544;
MN140085;Lepidoptera;Gonepteryx cleopatra;Gonepteryx cleopatra;0.998;Gonepteryx cleopatra;0.999118636582266;
MN140086;Lepidoptera;Carterocephalus palaemon;NA;0.052;Carterocephalus palaemon;0.728445051795325;
MN140090;Lepidoptera;Anthocharis cardamines;Anthocharis cardamines;1;Anthocharis cardamines;0.999999979642354;
MN140091;Lepidoptera;Maniola jurtina;NA;0.001;Maniola jurtina;0.939220105860875;
MN140093;Lepidoptera;Maniola cecilia;Pyronia cecilia;1;Pyronia cecilia;1;
MN140094;Lepidoptera;Maniola jurtina;NA;0.001;Maniola jurtina;0.939220105860875;
MN140095;Lepidoptera;Melitaea trivia;Melitaea trivia;1;Melitaea trivia;0.999999999960437;
MN140096;Lepidoptera;Melitaea varia;Melitaea varia;1;Melitaea varia;0.999999455569287;
MN140097;Lepidoptera;Argynnis aglaja;NA;NA;Speyeria aglaja;0.872991147917853;
MN140098;Lepidoptera;Colias alfacariensis;Colias alfacariensis;0.001;Colias alfacariensis;0.999926026317526;
MN140099;Lepidoptera;Nymphalis c-album;NA;0.215;NA;0.999999192918557;
MN140100;Lepidoptera;Maniola cecilia;Pyronia cecilia;NA;Pyronia cecilia;1;
MN140102;Lepidoptera;Favonius quercus;NA;NA;Favonius quercus;0.73046069582187;
MN140104;Lepidoptera;Lycaena phlaeas;Lycaena phlaeas;1;Lycaena phlaeas;0.999999996363186;
MN140106;Lepidoptera;Maniola jurtina;NA;0.012;Maniola jurtina;0.91755716175482;
MN140109;Lepidoptera;Maniola jurtina;NA;0.012;Maniola jurtina;0.91755716175482;
MN140111;Lepidoptera;Pyrgus malvoides;NA;0.2;Pyrgus malvoides;0.91582520613586;
MN140113;Lepidoptera;Fabriciana adippe;Fabriciana adippe;0.001;Fabriciana adippe;0.999538788643547;
MN140115;Lepidoptera;Lycaena phlaeas;Lycaena phlaeas;1;Lycaena phlaeas;0.999999996363186;
MN140117;Lepidoptera;Aricia cramera;Aricia cramera;1;Aricia cramera;0.999993731037033;
MN140118;Lepidoptera;Melitaea cinxia;Melitaea cinxia;1;Melitaea cinxia;0.999999999999801;
MN140119;Lepidoptera;Lysandra coridon;NA;NA;NA;0.997949615104622;
MN140121;Lepidoptera;Erynnis tages;Erynnis tages;1;Erynnis tages;0.999999281987018;
MN140122;Lepidoptera;Chazara briseis;Chazara briseis;1;Chazara briseis;0.999980393060755;
MN140125;Lepidoptera;Celastrina argiolus;Celastrina argiolus;1;Celastrina argiolus;0.999037794018796;
MN140126;Lepidoptera;Gonepteryx cleopatra;Gonepteryx cleopatra;0.998;Gonepteryx cleopatra;0.999118636582266;
MN140127;Lepidoptera;Melitaea diamina;NA;0.149;Melitaea diamina;0.999999996592464;
MN140128;Lepidoptera;Pyrgus armoricanus;Pyrgus armoricanus;1;Pyrgus armoricanus;0.999914079595002;
MN140129;Lepidoptera;Lasiommata maera;Lasiommata maera;1;Lasiommata maera;0.999988599344549;
MN140130;Lepidoptera;Anthocharis damone;Anthocharis damone;1;Anthocharis damone;0.999999933275918;

MN140131;Lepidoptera;Euphydryas intermedia;Euphydryas intermedia;0.017;Euphydryas intermedia;0.770732210581036;
MN140132;Lepidoptera;Pyrgus armoricanus;Pyrgus armoricanus;1;Pyrgus armoricanus;0.999997975465877;
MN140133;Lepidoptera;Euphydryas intermedia;NA;NA;NA;0.999999999996973;
MN140135;Lepidoptera;Melitaea didyma;Melitaea didyma;1;Melitaea didyma;0.999945816868155;
MN140136;Lepidoptera;Fabriciana niobe;NA;0.005;Fabriciana niobe;0.992125835358755;
MN140137;Lepidoptera;Boloria euphrosyne;Boloria euphrosyne;1;Boloria euphrosyne;0.999997439411014;
MN140140;Lepidoptera;Maniola jurtina;NA;0.012;Maniola jurtina;0.91755716175482;
MN140141;Lepidoptera;Colias alfacariensis;Colias alfacariensis;0.001;Colias alfacariensis;0.999926026317526;
MN140142;Lepidoptera;Pyrgus carthami;Pyrgus carthami;1;Pyrgus carthami;0.999995592447668;
MN140144;Lepidoptera;Cupido alcetas;NA;0.218;Cupido alcetas;0.833501983138177;
MN140145;Lepidoptera;Carcharodus alceae;Carcharodus alceae;0.001;Carcharodus alceae;0.999999995290494;
MN140146;Lepidoptera;Gegenes nostrodamus;Gegenes nostrodamus;1;Gegenes nostrodamus;0.999999995055305;
MN140147;Lepidoptera;Chazara briseis;Chazara briseis;1;Chazara briseis;0.999980393060755;
MN140148;Lepidoptera;Vanessa atalanta;Vanessa atalanta;1;Vanessa atalanta;0.999999999776037;
MN140150;Lepidoptera;Maniola jurtina;NA;NA;Maniola jurtina;0.973823960006955;
MN140151;Lepidoptera;Pyrgus malvoides;NA;0.053;Pyrgus malvoides;0.959164206737781;
MN140152;Lepidoptera;Thymelicus acteon;Thymelicus acteon;1;Thymelicus acteon;0.999999997929734;
MN140153;Lepidoptera;Boloria euphrosyne;Boloria euphrosyne;1;Boloria euphrosyne;0.999999886706838;
MN140154;Lepidoptera;Polyommatus icarus;NA;0.069;Polyommatus icarus;0.700731738155863;
MN140154;Lepidoptera;Polyommatus icarus;NA;0.069;Polyommatus icarus;0.700731738155863;
MN140156;Lepidoptera;Gonepteryx cleopatra;Gonepteryx cleopatra;0.998;Gonepteryx cleopatra;0.999118636582266;
MN140157;Lepidoptera;Aricia cramera;Aricia cramera;1;Aricia cramera;0.999990014848976;
MN140158;Lepidoptera;Boloria euphrosyne;Boloria euphrosyne;1;Boloria euphrosyne;0.999998838188297;
MN140159;Lepidoptera;Lycaena virgaureae;NA;0.186;Lycaena virgaureae;0.999491227342155;
MN140161;Lepidoptera;Lycaena tityrus;Lycaena tityrus;1;Lycaena tityrus;0.999998928238034;
MN140163;Lepidoptera;Nymphalis c-album;NA;0.215;NA;0.999999192918557;
MN140164;Lepidoptera;Maniola jurtina;NA;0.171;Maniola jurtina;0.963697912386445;
MN140165;Lepidoptera;Boloria titania;Boloria titania;0.001;Boloria titania;0.99983358374111;
MN140166;Lepidoptera;Brenthis daphne;NA;0.102;Brenthis daphne;0.717134521611551;
MN140167;Lepidoptera;Cupido minimus;NA;0.009;Cupido minimus;0.999791453712643;
MN140168;Lepidoptera;Pyrgus alveus;NA;0.007;NA;0.999999999997191;
MN140171;Lepidoptera;Thecla betulae;Thecla betulae;1;Thecla betulae;0.999999999972886;
MN140174;Lepidoptera;Coenonympha gardetta;NA;0.096;NA;0.999999999999996;
MN140175;Lepidoptera;Maniola jurtina;NA;0.012;Maniola jurtina;0.91755716175482;
MN140176;Lepidoptera;Colias hyale;Colias hyale;0.015;Colias hyale;0.999997884296128;
MN140177;Lepidoptera;Lycaena phlaeas;Lycaena phlaeas;1;Lycaena phlaeas;0.999999996363186;
MN140182;Lepidoptera;Coenonympha oedippus;Coenonympha oedippus;1;Coenonympha oedippus;0.999999999999972;
MN140184;Lepidoptera;Thymelicus sylvestris;NA;0.324;Thymelicus sylvestris;0.999940778442454;
MN140185;Lepidoptera;Parnassius apollo;Parnassius apollo;1;Parnassius apollo;0.994616929802738;
MN140186;Lepidoptera;Boloria graeca;Boloria graeca;1;Boloria graeca;0.999999954920356;
MN140187;Lepidoptera;Plebejus argus;NA;0.043;Plebejus argus;0.956256547935817;
MN140188;Lepidoptera;Erebia melampus;Erebia melampus;NA;Erebia melampus;0.947570619386055;
MN140190;Lepidoptera;Lysandra coridon;NA;NA;NA;0.992315981017308;
MN140191;Lepidoptera;Melitaea didyma;NA;NA;Melitaea didyma;0.975336875545609;
MN140192;Lepidoptera;Apatura ilia;NA;0.165;Apatura ilia;0.956796812031481;
MN140193;Lepidoptera;Aphantopus hyperantus;Aphantopus hyperantus;0.003;Aphantopus hyperantus;0.999999455871523;
MN140194;Lepidoptera;Colias alfacariensis;Colias alfacariensis;0.001;Colias alfacariensis;0.999926026317526;
MN140196;Lepidoptera;Thymelicus sylvestris;NA;0.324;Thymelicus sylvestris;0.999940778442454;
MN140197;Lepidoptera;Carcharodus alceae;Carcharodus alceae;1;Carcharodus alceae;0.999999994809997;
MN140198;Lepidoptera;Brenthis daphne;Brenthis daphne;0.001;Brenthis daphne;0.996209297077896;
MN140200;Lepidoptera;Erebia pharte;Erebia pharte;1;Erebia pharte;0.99999999630495;
MN140201;Lepidoptera;Glaucopsyche alexis;Glaucopsyche alexis;0.001;Glaucopsyche alexis;0.94042380088325;
MN140202;Lepidoptera;Vanessa cardui;Vanessa cardui;1;Vanessa cardui;0.999999459983318;
MN140203;Lepidoptera;Euphydryas maturna;NA;0.126;Euphydryas maturna;0.885705216462044;
MN140204;Lepidoptera;Anthocharis euphenoides;NA;0.172;Anthocharis euphenoides;0.943741664916385;
MN140205;Lepidoptera;Thymelicus sylvestris;Thymelicus sylvestris;1;Thymelicus sylvestris;0.999999985935205;
MN140207;Lepidoptera;Callophrys rubi;NA;0.001;Callophrys rubi;0.99870518510598;
MN140209;Lepidoptera;Maniola jurtina;NA;0.003;Maniola jurtina;0.934943936861184;
MN140210;Lepidoptera;Gonepteryx rhamni;NA;0.189;Gonepteryx rhamni;0.989643004532712;
MN140211;Lepidoptera;Maniola jurtina;NA;0.153;Maniola jurtina;0.908984189778786;
MN140212;Lepidoptera;Erebia pronoe;Erebia pronoe;1;Erebia pronoe;0.999718339357544;
MN140213;Lepidoptera;Lycaena virgaureae;NA;0.186;Lycaena virgaureae;0.999491227342155;
MN140214;Lepidoptera;Melitaea didyma;Melitaea didyma;1;Melitaea didyma;0.999937162819573;

MN140215;Lepidoptera;Maniola jurtina;NA;0.001;Maniola jurtina;0.939220105860875;
MN140216;Lepidoptera;Argynnis aglaja;NA;NA;Speyeria aglaja;0.872991147917853;
MN140217;Lepidoptera;Pieris napi;NA;0.011;Pieris napi;0.801985394724814;
MN140219;Lepidoptera;Melitaea diamina;NA;0.305;Melitaea diamina;0.999999994065661;
MN140220;Lepidoptera;Lycaena phlaeas;Lycaena phlaeas;1;Lycaena phlaeas;0.999999996363186;
MN140221;Lepidoptera;Pyrgus onopordi;Pyrgus onopordi;1;Pyrgus onopordi;0.999997111229755;
MN140226;Lepidoptera;Thymelicus acteon;Thymelicus acteon;1;Thymelicus acteon;0.99999999752248;
MN140227;Lepidoptera;Melitaea didyma;Melitaea didyma;1;Melitaea didyma;0.99009216179415;
MN140229;Lepidoptera;Aricia cramera;Aricia cramera;1;Aricia cramera;0.999993731037033;
MN140231;Lepidoptera;Pyrgus malvae;Pyrgus malvae;NA;Pyrgus malvae;0.980121140374433;
MN140233;Lepidoptera;Aricia eumedon;Eumedonia eumedon;0.001;Eumedonia eumedon;0.998400646011029;
MN140235;Lepidoptera;Thymelicus acteon;Thymelicus acteon;1;Thymelicus acteon;0.99999999752248;
MN140236;Lepidoptera;Colias crocea;NA;0.059;NA;0.999999999999738;
MN140238;Lepidoptera;Maniola jurtina;NA;0.012;Maniola jurtina;0.91755716175482;
MN140239;Lepidoptera;Gonepteryx cleopatra;Gonepteryx cleopatra;0.998;Gonepteryx
cleopatra;0.999118636582266;
MN140240;Lepidoptera;Pyrgus armoricanus;Pyrgus armoricanus;1;Pyrgus
armoricanus;0.999998381055323;
MN140241;Lepidoptera;Pieris napi;NA;0.028;Pieris napi;0.888205720664699;
MN140244;Lepidoptera;Gonepteryx rhamni;NA;0.202;Gonepteryx rhamni;0.990544908845376;
MN140245;Lepidoptera;Maniola jurtina;NA;0.012;Maniola jurtina;0.91755716175482;
MN140246;Lepidoptera;Lycaena hippothoe;Lycaena hippothoe;NA;Lycaena hippothoe;0.995296610589267;
MN140247;Lepidoptera;Lasiommata paramegaera;Lasiommata paramegaera;0.999;Lasiommata
paramegaera;0.995283446287531;
MN140248;Lepidoptera;Pieris napi;NA;0.011;Pieris napi;0.801985394724814;
MN140250;Lepidoptera;Boloria euphrosyne;Boloria euphrosyne;1;Boloria
euphrosyne;0.999999888858929;
MN140251;Lepidoptera;Erebia eriphyle;Erebia eriphyle;1;Erebia eriphyle;0.999999983716066;
MN140252;Lepidoptera;Melitaea cinxia;Melitaea cinxia;1;Melitaea cinxia;0.999999999999289;
MN140253;Lepidoptera;Argynnis aglaja;NA;NA;Speyeria aglaja;0.872991147917853;
MN140254;Lepidoptera;Brenthis daphne;Brenthis daphne;0.001;Brenthis daphne;0.997781825264304;
MN140256;Lepidoptera;Polyommatus damon;Polyommatus damon;1;Polyommatus damon;0.999999921524452;
MN140257;Lepidoptera;Apatura ilia;Apatura ilia;NA;Apatura ilia;0.988792300608896;
MN140258;Lepidoptera;Coenonympha pamphilus;Coenonympha pamphilus;1;Coenonympha
pamphilus;0.999999999999744;
MN140259;Lepidoptera;Aricia cramera;Aricia cramera;1;Aricia cramera;0.999993731037033;
MN140262;Lepidoptera;Pyrgus carlinae;NA;NA;Pyrgus carlinae;0.854199018204849;
MN140263;Lepidoptera;Brenthis daphne;NA;0.186;Brenthis daphne;0.703003804838281;
MN140264;Lepidoptera;Plebejus argus;Plebejus argus;0.016;Plebejus argus;0.962605109836459;
MN140265;Lepidoptera;Aricia cramera;Aricia cramera;1;Aricia cramera;0.999993731037033;
MN140266;Lepidoptera;Brenthis daphne;Brenthis daphne;0.999;Brenthis daphne;0.999440682835247;
MN140267;Lepidoptera;Maniola cecilia;Pyronia cecilia;1;Pyronia cecilia;1;
MN140268;Lepidoptera;Lasiommata maera;Lasiommata maera;0.001;Lasiommata maera;0.999981462505912;
MN140269;Lepidoptera;Colias crocea;NA;0.059;NA;0.999999999999738;
MN140270;Lepidoptera;Melanargia galathea;Melanargia galathea;0.001;Melanargia
galathea;0.965001574126447;
MN140273;Lepidoptera;Pieris napi;NA;0.011;Pieris napi;0.801985394724814;
MN140274;Lepidoptera;Polyommatus icarus;Polyommatus icarus;0.001;Polyommatus
icarus;0.999242418163017;
MN140274;Lepidoptera;Polyommatus icarus;Polyommatus icarus;0.001;Polyommatus
icarus;0.999242418163017;
MN140275;Lepidoptera;Aricia eumedon;Eumedonia eumedon;0.002;Eumedonia eumedon;0.997793024117892;
MN140276;Lepidoptera;Gonepteryx rhamni;NA;0.474;Gonepteryx rhamni;0.982076396127858;
MN140278;Lepidoptera;Melitaea didyma;Melitaea didyma;0.021;Melitaea didyma;0.9742722334097;
MN140279;Lepidoptera;Maniola jurtina;NA;0.012;Maniola jurtina;0.91755716175482;
MN140280;Lepidoptera;Euphydryas cynthia;Euphydryas cynthia;1;Euphydryas
cynthia;0.999999243242402;
MN140281;Lepidoptera;Anthocharis damone;Anthocharis damone;1;Anthocharis
damone;0.999999996610995;
MN140282;Lepidoptera;Coenonympha pamphilus;Coenonympha pamphilus;1;Coenonympha
pamphilus;0.999999999999744;
MN140283;Lepidoptera;Vanessa atalanta;Vanessa atalanta;1;Vanessa atalanta;0.999999999757762;
MN140284;Lepidoptera;Carterocephalus palaemon;NA;0.052;Carterocephalus
palaemon;0.728445051795325;
MN140286;Lepidoptera;Apatura ilia;NA;0.165;Apatura ilia;0.956796812031481;
MN140289;Lepidoptera;Aricia eumedon;Eumedonia eumedon;0.002;Eumedonia eumedon;0.997793024117892;
MN140290;Lepidoptera;Maniola jurtina;NA;0.157;Maniola jurtina;0.72935823378201;
MN140292;Lepidoptera;Agriades orbitulus;NA;0.01;Agriades orbitulus;0.998991302227708;
MN140293;Lepidoptera;Colias alfacariensis;Colias alfacariensis;1;Colias
alfacariensis;0.999939067457475;
MN140294;Lepidoptera;Colias crocea;NA;0.059;NA;0.999999999999738;
MN140295;Lepidoptera;Coenonympha gardetta;NA;0.32;Coenonympha arcania;0.715629089660113;
MN140296;Lepidoptera;Maniola jurtina;NA;0.175;Maniola jurtina;0.950174303242181;
MN140297;Lepidoptera;Polyommatus icarus;NA;0.003;Polyommatus icarus;0.998191904408636;
MN140297;Lepidoptera;Polyommatus icarus;NA;0.003;Polyommatus icarus;0.998191904408636;
MN140298;Lepidoptera;Maniola jurtina;NA;0.003;Maniola jurtina;0.930461068589493;
MN140299;Lepidoptera;Erebia pharte;Erebia pharte;1;Erebia pharte;0.99999999630495;
MN140300;Lepidoptera;Aricia eumedon;Eumedonia eumedon;1;Eumedonia eumedon;0.99887319009208;

MN140301;Lepidoptera;Lysandra hispana;NA;NA;NA;0.991360562381433;
MN140304;Lepidoptera;Argynnis paphia;Argynnis paphia;1;Argynnis paphia;0.999999990911675;
MN140305;Lepidoptera;Aricia cramera;Aricia cramera;1;Aricia cramera;0.999993731037033;
MN140306;Lepidoptera;Aricia artaxerxes;NA;0.001;Aricia artaxerxes;0.999429422213984;
MN140307;Lepidoptera;Coenonympha pamphilus;Coenonympha pamphilus;1;Coenonympha pamphilus;0.999999999999744;
MN140308;Lepidoptera;Lycaena tityrus;Lycaena tityrus;1;Lycaena tityrus;0.999998928238034;
MN140309;Lepidoptera;Callophrys rubi;NA;0.003;Callophrys rubi;0.997328639918919;
MN140310;Lepidoptera;Aricia cramera;Aricia cramera;1;Aricia cramera;0.999993731037033;
MN140311;Lepidoptera;Arethusana arethusa;NA;0.062;Arethusana arethusa;0.999962489703143;
MN140312;Lepidoptera;Thymelicus lineola;Thymelicus lineola;1;Thymelicus lineola;0.999995927238524;
MN140314;Lepidoptera;Cupido argiades;NA;0.499;NA;0.999999999984682;
MN140315;Lepidoptera;Aricia agestis;NA;0.113;Aricia agestis;0.99685250063917;
MN140317;Lepidoptera;Erebia meolans;Erebia meolans;NA;Erebia meolans;0.99565582584285;
MN140319;Lepidoptera;Vanessa atalanta;Vanessa atalanta;1;Vanessa atalanta;0.999999999757762;
MN140320;Lepidoptera;Coenonympha pamphilus;Coenonympha pamphilus;1;Coenonympha pamphilus;0.999999999999886;
MN140321;Lepidoptera;Lasiommata maera;Lasiommata maera;1;Lasiommata maera;0.999988599344549;
MN140322;Lepidoptera;Erebia medusa;NA;0.002;Erebia medusa;0.991052367627986;
MN140324;Lepidoptera;Vanessa cardui;Vanessa cardui;1;Vanessa cardui;0.999999459983318;
MN140325;Lepidoptera;Aglais urticae;NA;0.002;Aglais urticae;0.987497593219019;
MN140326;Lepidoptera;Fabriciana niobe;Fabriciana niobe;0.001;Fabriciana niobe;0.93186202957215;
MN140327;Lepidoptera;Lycaena dispar;Lycaena dispar;1;Lycaena dispar;0.999999990743959;
MN140328;Lepidoptera;Polyommatus dorylas;Polyommatus dorylas;0.005;Polyommatus dorylas;0.987518562410738;
MN140329;Lepidoptera;Aphantopus hyperantus;Aphantopus hyperantus;1;Aphantopus hyperantus;0.999999677131849;
MN140330;Lepidoptera;Celastrina argiolus;Celastrina argiolus;1;Celastrina argiolus;0.999527385150531;
MN140335;Lepidoptera;Celastrina argiolus;Celastrina argiolus;1;Celastrina argiolus;0.999527385150531;
MN140336;Lepidoptera;Colias crocea;NA;0.059;NA;0.999999999999738;
MN140337;Lepidoptera;Cupido alcetas;NA;0.218;Cupido alcetas;0.833501983138177;
MN140338;Lepidoptera;Glaucopsyche alexis;Glaucopsyche alexis;NA;Glaucopsyche alexis;0.963341895524591;
MN140339;Lepidoptera;Polyommatus dorylas;Polyommatus dorylas;0.008;Polyommatus dorylas;0.98570396564071;
MN140341;Lepidoptera;Maniola jurtina;NA;0.001;Maniola jurtina;0.939220105860875;
MN140342;Lepidoptera;Aporia crataegi;NA;NA;Aporia crataegi;0.999998459680561;
MN140343;Lepidoptera;Chazara briseis;Chazara briseis;0.001;Chazara briseis;0.999987492373468;
MN140344;Lepidoptera;Polyommatus celina;Polyommatus celina;1;Polyommatus celina;0.999999997901256;
MN140345;Lepidoptera;Colias crocea;NA;0.059;NA;0.999999999999738;
MN140346;Lepidoptera;Maniola jurtina;NA;0.147;Maniola jurtina;0.955259384450175;
MN140347;Lepidoptera;Thymelicus sylvestris;Thymelicus sylvestris;1;Thymelicus sylvestris;0.999999985935205;
MN140351;Lepidoptera;Celastrina argiolus;Celastrina argiolus;1;Celastrina argiolus;0.999527385150531;
MN140353;Lepidoptera;Aricia cramera;Aricia cramera;1;Aricia cramera;0.999993731037033;
MN140354;Lepidoptera;Cupido osiris;Cupido osiris;1;Cupido osiris;0.999999834740279;
MN140355;Lepidoptera;Favonius quercus;NA;NA;Favonius quercus;0.73046069582187;
MN140356;Lepidoptera;Polyommatus dolus;Polyommatus dolus;0.004;Polyommatus dolus;0.876833853168453;
MN140357;Lepidoptera;Maniola jurtina;NA;0.001;Maniola jurtina;0.939220105860875;
MN140358;Lepidoptera;Lampides boeticus;Lampides boeticus;1;Lampides boeticus;0.999999999545906;
MN140361;Lepidoptera;Melitaea cinxia;Melitaea cinxia;1;Melitaea cinxia;0.999999999999318;
MN140363;Lepidoptera;Glaucopsyche melanops;Glaucopsyche melanops;1;Glaucopsyche melanops;0.999952924254607;
MN140364;Lepidoptera;Pyrgus malvoides;NA;0.176;Pyrgus malvoides;0.940490890379026;
MN140365;Lepidoptera;Maniola jurtina;NA;0.012;Maniola jurtina;0.91755716175482;
MN140368;Lepidoptera;Erebia medusa;NA;0.009;Erebia medusa;0.986275612856827;
MN140372;Lepidoptera;Lysandra coridon;NA;NA;NA;0.998070184737648;
MN140373;Lepidoptera;Anthocharis cardamines;Anthocharis cardamines;1;Anthocharis cardamines;0.999999986506026;
MN140375;Lepidoptera;Erebia meolans;Erebia meolans;NA;Erebia meolans;0.999835953426128;
MN140376;Lepidoptera;Pyrgus armoricanus;Pyrgus armoricanus;1;Pyrgus armoricanus;0.999998858647755;
MN140377;Lepidoptera;Aphantopus hyperantus;Aphantopus hyperantus;0.006;Aphantopus hyperantus;0.999998615504572;
MN140378;Lepidoptera;Hipparchia hermione;Hipparchia hermione;1;Hipparchia hermione;0.999791304970896;
MN140381;Lepidoptera;Polyommatus amandus;Polyommatus amandus;1;Polyommatus amandus;0.999993368166194;
MN140383;Lepidoptera;Aricia agestis;NA;0.067;Aricia agestis;0.999509743227786;
MN140384;Lepidoptera;Colias alfacariensis;Colias alfacariensis;1;Colias alfacariensis;0.999986994131001;
MN140385;Lepidoptera;Maniola jurtina;NA;0.012;Maniola jurtina;0.91755716175482;

MN140386;Lepidoptera;Coenonympha pamphilus;Coenonympha pamphilus;1;Coenonympha pamphilus;0.999999999999744;
MN140387;Lepidoptera;Lycaena tityrus;Lycaena tityrus;1;Lycaena tityrus;0.999997855580031;
MN140388;Lepidoptera;Maniola jurtina;NA;0.001;Maniola jurtina;0.939220105860875;
MN140389;Lepidoptera;Fabriciana niobe;Fabriciana niobe;0.001;Fabriciana niobe;0.93186202957215;
MN140392;Lepidoptera;Erynnis tages;Erynnis tages;1;Erynnis tages;0.999999281987018;
MN140393;Lepidoptera;Coenonympha pamphilus;Coenonympha pamphilus;1;Coenonympha pamphilus;0.999999999999744;
MN140394;Lepidoptera;Satyrium acaciae;Satyrium acaciae;1;Satyrium acaciae;0.999999374890702;
MN140396;Lepidoptera;Nymphalis c-album;NA;0.215;NA;0.999999192918557;
MN140397;Lepidoptera;Melitaea trivia;Melitaea trivia;1;Melitaea trivia;0.999999999970555;
MN140399;Lepidoptera;Hipparchia fagi;NA;NA;Hipparchia fagi;0.960942485014654;
MN140400;Lepidoptera;Maniola jurtina;NA;NA;Maniola jurtina;0.951668238126128;
MN140401;Lepidoptera;Colias alfacariensis;Colias alfacariensis;0.001;Colias alfacariensis;0.999926026317526;
MN140402;Lepidoptera;Pyrgus serratulae;Pyrgus serratulae;1;Pyrgus serratulae;0.99999989567508;
MN140405;Lepidoptera;Satyrium esculi;Satyrium esculi;1;Satyrium esculi;0.999999957367948;
MN140407;Lepidoptera;Cupido argiades;NA;0.499;NA;0.999999999984682;
MN140408;Lepidoptera;Carcharodus alceae;Carcharodus alceae;1;Carcharodus alceae;0.999999998465938;
MN140409;Lepidoptera;Colias alfacariensis;Colias alfacariensis;0.001;Colias alfacariensis;0.999926245067755;
MN140412;Lepidoptera;Hipparchia fagi;Hipparchia fagi;NA;Hipparchia fagi;0.981068649813775;
MN140413;Lepidoptera;Satyrium w-album;NA;0.056;Satyrium w-album;0.989378230996316;
MN140416;Lepidoptera;Aporia crataegi;NA;NA;Aporia crataegi;0.999996781126733;
MN140417;Lepidoptera;Ochlodes sylvanus;NA;0.316;Ochlodes sylvanus;0.996318754838625;
MN140418;Lepidoptera;Aphantopus hyperantus;Aphantopus hyperantus;0.006;Aphantopus hyperantus;0.999998615504572;
MN140419;Lepidoptera;Lycaena phlaeas;Lycaena phlaeas;1;Lycaena phlaeas;0.99999999308983;
MN140420;Lepidoptera;Glaucopsyche alexis;NA;NA;Glaucopsyche alexis;0.991019528246159;
MN140421;Lepidoptera;Carterocephalus palaemon;NA;0.052;Carterocephalus palaemon;0.728445051795325;
MN140422;Lepidoptera;Anthocharis damone;Anthocharis damone;1;Anthocharis damone;0.999999991170057;
MN140424;Lepidoptera;Boloria euphrosyne;Boloria euphrosyne;0.001;Boloria euphrosyne;0.999985640170951;
MN140426;Lepidoptera;Parnassius apollo;Parnassius apollo;1;Parnassius apollo;0.992704703916692;
MN140427;Lepidoptera;Lysandra coridon;NA;NA;NA;0.998070184737648;
MN140429;Lepidoptera;Polyommatus damon;Polyommatus damon;1;Polyommatus damon;0.999999878117855;
MN140431;Lepidoptera;Hipparchia hermione;NA;NA;NA;0.999999999999991;
MN140432;Lepidoptera;Aglais urticae;NA;0.002;Aglais urticae;0.987497593219019;
MN140433;Lepidoptera;Lysandra coridon;Lysandra coridon;NA;Lysandra coridon;0.931719578492241;
MN140435;Lepidoptera;Pyrgus serratulae;Pyrgus serratulae;1;Pyrgus serratulae;0.99999989567508;
MN140439;Lepidoptera;Melitaea cinxia;Melitaea cinxia;1;Melitaea cinxia;0.999999999999204;
MN140440;Lepidoptera;Brenthis daphne;Brenthis daphne;0.998;Brenthis daphne;0.998207407614684;
MN140444;Lepidoptera;Vanessa cardui;Vanessa cardui;1;Vanessa cardui;0.999999803450484;
MN140445;Lepidoptera;Maniola jurtina;NA;NA;Maniola jurtina;0.950689021412593;
MN140448;Lepidoptera;Hipparchia fagi;Hipparchia fagi;NA;Hipparchia fagi;0.986566456150817;
MN140449;Lepidoptera;Glaucopsyche alexis;Glaucopsyche alexis;0.001;Glaucopsyche alexis;0.94042380088325;
MN140450;Lepidoptera;Euphydryas aurinia;NA;0.31;Euphydryas aurinia;0.993387708761386;
MN140451;Lepidoptera;Polyommatus icarus;NA;0.001;NA;0.999994270558956;
MN140451;Lepidoptera;Polyommatus icarus;NA;0.001;NA;0.999994270558956;
MN140453;Lepidoptera;Coenonympha dorus;Coenonympha dorus;1;Coenonympha dorus;0.99999999988259;
MN140454;Lepidoptera;Pieris napi;NA;0.028;Pieris napi;0.888205720664699;
MN140455;Lepidoptera;Lampides boeticus;Lampides boeticus;1;Lampides boeticus;0.999999999545906;
MN140459;Lepidoptera;Pieris napi;NA;NA;Pieris napi;0.896177367075973;
MN140460;Lepidoptera;Arethusana arethusa;Arethusana arethusa;0.008;Arethusana arethusa;0.999972993005957;
MN140462;Lepidoptera;Boloria selene;NA;0.179;Boloria selene;0.999999092153374;
MN140463;Lepidoptera;Maniola jurtina;NA;0.008;Maniola jurtina;0.881964271783318;
MN140465;Lepidoptera;Celastrina argiolus;Celastrina argiolus;1;Celastrina argiolus;0.999185496292995;
MN140466;Lepidoptera;Callophrys rubi;NA;0.003;Callophrys rubi;0.997328639918919;
MN140467;Lepidoptera;Melitaea didyma;Melitaea didyma;1;Melitaea didyma;0.997360676186264;
MN140468;Lepidoptera;Maniola jurtina;NA;NA;Maniola jurtina;0.951668238126128;
MN140469;Lepidoptera;Lampides boeticus;Lampides boeticus;1;Lampides boeticus;0.999999999545906;
MN140470;Lepidoptera;Maniola jurtina;NA;0.001;Maniola jurtina;0.939220105860875;
MN140471;Lepidoptera;Thymelicus lineola;Thymelicus lineola;1;Thymelicus lineola;0.999995927238524;
MN140474;Lepidoptera;Aricia agestis;NA;0.253;Aricia agestis;0.996350017387504;
MN140475;Lepidoptera;Aricia cramera;Aricia cramera;1;Aricia cramera;0.999993731037033;
MN140477;Lepidoptera;Aricia cramera;Aricia cramera;1;Aricia cramera;0.999992229282534;
MN140478;Lepidoptera;Hipparchia semele;NA;0.001;Hipparchia semele;0.824269221281445;
MN140479;Lepidoptera;Pyrgus foulquieri;NA;NA;Pyrgus alveus;0.827119616974978;
MN140480;Lepidoptera;Aglais urticae;NA;0.002;Aglais urticae;0.987497593219019;
MN140481;Lepidoptera;Erebia pharte;Erebia pharte;1;Erebia pharte;0.99999999630495;

MN140482;Lepidoptera;Polyommatus daphnis;Polyommatus daphnis;1;Polyommatus daphnis;0.999946618182486;
MN140483;Lepidoptera;Argynnis paphia;Argynnis paphia;1;Argynnis paphia;0.999999993686771;
MN140484;Lepidoptera;Carcharodus alceae;Carcharodus alceae;0.999;Carcharodus alceae;0.999999998008462;
MN140485;Lepidoptera;Aricia cramera;Aricia cramera;1;Aricia cramera;0.999993731037033;
MN140487;Lepidoptera;Pieris napi;NA;0.028;Pieris napi;0.888205720664699;
MN140488;Lepidoptera;Lycaena alciphron;Lycaena alciphron;1;Lycaena alciphron;0.999999903149648;
MN140489;Lepidoptera;Boloria dia;Boloria dia;1;Boloria dia;0.999999999986613;
MN140491;Lepidoptera;Coenonympha oedippus;Coenonympha oedippus;1;Coenonympha oedippus;0.999999999999943;
MN140492;Lepidoptera;Vanessa cardui;Vanessa cardui;1;Vanessa cardui;0.999999459983318;
MN140494;Lepidoptera;Hipparchia aristaeus;Hipparchia aristaeus;1;Hipparchia aristaeus;0.99999998958458;
MN140496;Lepidoptera;Brenthis daphne;Brenthis daphne;0.006;Brenthis daphne;0.984559944066479;
MN140498;Lepidoptera;Arethusana arethusa;Arethusana arethusa;0.033;Arethusana arethusa;0.999959591411034;
MN140500;Lepidoptera;Anthocharis cardamines;Anthocharis cardamines;1;Anthocharis cardamines;0.999999934410001;
MN140501;Lepidoptera;Ochlodes sylvanus;NA;0.316;Ochlodes sylvanus;0.996318754838625;
MN140502;Lepidoptera;Argynnis aglaja;NA;NA;Speyeria aglaja;0.872991147917853;
MN140503;Lepidoptera;Erebia cassioides;NA;NA;NA;0.999999999999875;
MN140505;Lepidoptera;Maniola jurtina;NA;0.008;Maniola jurtina;0.881964271783318;
MN140506;Lepidoptera;Vanessa cardui;Vanessa cardui;1;Vanessa cardui;0.999999459983318;
MN140508;Lepidoptera;Aricia artaxerxes;NA;0.139;Aricia artaxerxes;0.999594219786776;
MN140509;Lepidoptera;Aphantopus hyperantus;Aphantopus hyperantus;0.006;Aphantopus hyperantus;0.999998615504572;
MN140512;Lepidoptera;Satyrus actaea;Satyrus actaea;0.01;Satyrus actaea;0.94366382029787;
MN140514;Lepidoptera;Aricia agestis;NA;0.081;Aricia agestis;0.998857812762894;
MN140515;Lepidoptera;Ochlodes sylvanus;NA;0.316;Ochlodes sylvanus;0.996318754838625;
MN140516;Lepidoptera;Lycaena tityrus;Lycaena tityrus;1;Lycaena tityrus;0.999998928238034;
MN140519;Lepidoptera;Polyommatus celina;Polyommatus celina;1;Polyommatus celina;0.999999999666926;
MN140521;Lepidoptera;Lycaena virgaureae;NA;0.463;Lycaena virgaureae;0.998978647124085;
MN140522;Lepidoptera;Pieris napi;NA;0.001;Pieris napi;0.869484797213931;
MN140523;Lepidoptera;Polyommatus dorylas;Polyommatus dorylas;0.025;Polyommatus dorylas;0.973229109265438;
MN140524;Lepidoptera;Hipparchia semele;NA;0.001;Hipparchia semele;0.887463969237647;
MN140526;Lepidoptera;Pieris napi;NA;0.028;Pieris napi;0.888205720664699;
MN140528;Lepidoptera;Erebia styx;NA;NA;Erebia styx;0.946491813354197;
MN140529;Lepidoptera;Parnassius apollo;Parnassius apollo;1;Parnassius apollo;0.996164492145035;
MN140531;Lepidoptera;Aglais urticae;Aglais urticae;0.001;Aglais urticae;0.994396335177485;
MN140534;Lepidoptera;Plebejus argus;Plebejus argus;0.016;Plebejus argus;0.962605109836459;
MN140536;Lepidoptera;Colias alfacariensis;Colias alfacariensis;0.001;Colias alfacariensis;0.999926026317526;
MN140537;Lepidoptera;Aglais urticae;NA;0.002;Aglais urticae;0.987497593219019;
MN140539;Lepidoptera;Arethusana arethusa;NA;0.062;Arethusana arethusa;0.999962489703143;
MN140541;Lepidoptera;Lycaena virgaureae;NA;0.184;Lycaena virgaureae;0.999653824584039;
MN140543;Lepidoptera;Colias crocea;NA;0.059;NA;0.999999999999738;
MN140544;Lepidoptera;Cupido minimus;NA;0.006;Cupido minimus;0.999768371759258;
MN140545;Lepidoptera;Aporia crataegi;NA;NA;Aporia crataegi;0.999997860566808;
MN140546;Lepidoptera;Gonepteryx cleopatra;Gonepteryx cleopatra;0.998;Gonepteryx cleopatra;0.999118636582266;
MN140548;Lepidoptera;Aricia cramera;Aricia cramera;1;Aricia cramera;0.999993731037033;
MN140549;Lepidoptera;Colias crocea;NA;0.059;NA;0.999999999999738;
MN140550;Lepidoptera;Coenonympha pamphilus;Coenonympha pamphilus;1;Coenonympha pamphilus;0.999999999999744;
MN140551;Lepidoptera;Cupido alcetas;NA;0.218;Cupido alcetas;0.833501983138177;
MN140552;Lepidoptera;Papilio machaon;Papilio machaon;0.003;Papilio machaon;0.998739556051929;
MN140553;Lepidoptera;Colias crocea;NA;0.059;NA;0.999999999999738;
MN140554;Lepidoptera;Maniola jurtina;NA;NA;Maniola jurtina;0.962368106146462;
MN140555;Lepidoptera;Thymelicus sylvestris;Thymelicus sylvestris;1;Thymelicus sylvestris;0.999999981568351;
MN140556;Lepidoptera;Cupido osiris;Cupido osiris;1;Cupido osiris;0.999999791352473;
MN140558;Lepidoptera;Pyrgus malvoides;NA;0.167;Pyrgus malvoides;0.940320745704742;
MN140559;Lepidoptera;Colias palaeno;Colias palaeno;0.001;NA;0.999999999992202;
MN140560;Lepidoptera;Plebejus argus;Plebejus argus;0.013;Plebejus argus;0.99029909759821;
MN140561;Lepidoptera;Pieris napi;NA;0.028;Pieris napi;0.888205720664699;
MN140562;Lepidoptera;Maniola cecilia;Pyronia cecilia;1;Pyronia cecilia;1;
MN140563;Lepidoptera;Vanessa cardui;Vanessa cardui;1;Vanessa cardui;0.999991578978624;
MN140566;Lepidoptera;Plebejus idas;NA;0.002;NA;0.99959062880967;
MN140567;Lepidoptera;Cupido osiris;Cupido osiris;1;Cupido osiris;0.999995435613969;
MN140568;Lepidoptera;Pieris napi;NA;0.011;Pieris napi;0.801985394724814;
MN140569;Lepidoptera;Argynnis aglaja;NA;NA;Speyeria aglaja;0.872991147917853;
MN140570;Lepidoptera;Aglais urticae;NA;0.002;Aglais urticae;0.987497593219019;
MN140571;Lepidoptera;Aglais urticae;NA;0.002;Aglais urticae;0.987497593219019;
MN140574;Lepidoptera;Pieris napi;NA;0.003;Pieris napi;0.841488404403525;
MN140576;Lepidoptera;Plebejus argus;NA;0.048;Plebejus argus;0.985619685577692;

MN140577;Lepidoptera;Colias hyale;Colias hyale;0.015;Colias hyale;0.999997884296128;
MN140578;Lepidoptera;Lysandra coridon;NA;NA;Lysandra coridon;0.72097638065991;
MN140579;Lepidoptera;Minois dryas;NA;0.025;Minois dryas;0.850746026247397;
MN140580;Lepidoptera;Pyrgus serratulae;Pyrgus serratulae;1;Pyrgus serratulae;0.999999807446234;
MN140581;Lepidoptera;Hipparchia semele;NA;0.001;Hipparchia semele;0.882394980075983;
MN140584;Lepidoptera;Argynnis aglaja;NA;NA;Speyeria aglaja;0.872991147917853;
MN140585;Lepidoptera;Gonepteryx rhamni;NA;0.474;Gonepteryx rhamni;0.982076396127858;
MN140587;Lepidoptera;Polyommatus dorylas;NA;0.004;Polyommatus dorylas;0.973574185045329;
MN140588;Lepidoptera;Argynnis paphia;Argynnis paphia;1;Argynnis paphia;0.999999991389927;
MN140589;Lepidoptera;Hipparchia fagi;Hipparchia fagi;NA;Hipparchia fagi;0.986566456150817;
MN140590;Lepidoptera;Melitaea didyma;Melitaea didyma;1;Melitaea didyma;0.999429129005203;
MN140591;Lepidoptera;Aricia agestis;NA;0.253;Aricia agestis;0.996350017387504;
MN140592;Lepidoptera;Pieris napi;NA;0.028;Pieris napi;0.888205720664699;
MN140593;Lepidoptera;Aporia crataegi;NA;NA;Aporia crataegi;0.999996781126733;
MN140594;Lepidoptera;Pieris napi;NA;0.011;Pieris napi;0.801985394724814;
MN140595;Lepidoptera;Aricia cramera;Aricia cramera;1;Aricia cramera;0.999995130925017;
MN140596;Lepidoptera;Fabriciana niobe;Fabriciana niobe;0.001;Fabriciana niobe;0.93186202957215;
MN140597;Lepidoptera;Melitaea didyma;Melitaea didyma;1;Melitaea didyma;0.9999226971447;
MN140598;Lepidoptera;Erebia pluto;Erebia pluto;1;Erebia pluto;0.999999977588772;
MN140599;Lepidoptera;Polyommatus icarus;NA;0.013;Polyommatus icarus;0.809997132735827;
MN140599;Lepidoptera;Polyommatus icarus;NA;0.013;Polyommatus icarus;0.809997132735827;
MN140600;Lepidoptera;Satyrus ferula;Satyrus ferula;0.001;Satyrus ferula;0.992107254386621;
MN140603;Lepidoptera;Lysandra coridon;NA;NA;NA;0.998017994491822;
MN140604;Lepidoptera;Maniola jurtina;NA;0.07;Maniola jurtina;0.968492444409788;
MN140605;Lepidoptera;Maniola cecilia;Pyronia cecilia;NA;Pyronia cecilia;0.999999999999972;
MN140607;Lepidoptera;Polyommatus amandus;Polyommatus amandus;0.002;Polyommatus amandus;0.999980796628209;
MN140609;Lepidoptera;Pieris napi;NA;0.028;Pieris napi;0.888205720664699;
MN140611;Lepidoptera;Argynnis aglaja;NA;NA;Speyeria aglaja;0.872991147917853;
MN140612;Lepidoptera;Carcharodus lavatherae;NA;0.221;Carcharodus lavatherae;0.997497103905344;
MN140613;Lepidoptera;Cyaniris semiargus;Cyaniris semiargus;NA;Cyaniris semiargus;0.999813148264808;
MN140614;Lepidoptera;Plebejus idas;NA;0.007;NA;0.999546324331549;
MN140615;Lepidoptera;Brintesia circe;Brintesia circe;1;Brintesia circe;0.999999586895285;
MN140616;Lepidoptera;Gegenes pumilio;Gegenes pumilio;1;Gegenes pumilio;0.999986726679266;
MN140618;Lepidoptera;Aporia crataegi;NA;NA;Aporia crataegi;0.999996781126733;
MN140620;Lepidoptera;Pyrgus malvoides;NA;0.052;Pyrgus malvoides;0.943208768333892;
MN140621;Lepidoptera;Vanessa atalanta;Vanessa atalanta;1;Vanessa atalanta;0.999999999757762;
MN140622;Lepidoptera;Thymelicus sylvestris;NA;0.321;Thymelicus sylvestris;0.999953344764614;
MN140623;Lepidoptera;Plebejus argus;NA;0.328;Plebejus argus;0.969789734199889;
MN140627;Lepidoptera;Lycaena phlaeas;Lycaena phlaeas;1;Lycaena phlaeas;0.999999996363186;
MN140628;Lepidoptera;Vanessa cardui;Vanessa cardui;1;Vanessa cardui;0.999997579965112;
MN140630;Lepidoptera;Lampides boeticus;Lampides boeticus;1;Lampides boeticus;0.999999999545906;
MN140631;Lepidoptera;Thymelicus lineola;Thymelicus lineola;1;Thymelicus lineola;0.999971535093703;
MN140632;Lepidoptera;Coenonympha pamphilus;Coenonympha pamphilus;1;Coenonympha pamphilus;0.999999999999744;
MN140633;Lepidoptera;Polyommatus icarus;Polyommatus icarus;0.001;Polyommatus icarus;0.999851522482238;
MN140633;Lepidoptera;Polyommatus icarus;Polyommatus icarus;0.001;Polyommatus icarus;0.999851522482238;
MN140634;Lepidoptera;Nymphalis c-album;NA;0.215;NA;0.999999192918557;
MN140635;Lepidoptera;Pyrgus serratulae;Pyrgus serratulae;1;Pyrgus serratulae;0.999999877361184;
MN140637;Lepidoptera;Colias alfacariensis;Colias alfacariensis;0.001;Colias alfacariensis;0.999857790810452;
MN140638;Lepidoptera;Polyommatus icarus;Polyommatus icarus;0.002;Polyommatus icarus;0.999653930279044;
MN140638;Lepidoptera;Polyommatus icarus;Polyommatus icarus;0.002;Polyommatus icarus;0.999653930279044;
MN140640;Lepidoptera;Vanessa cardui;Vanessa cardui;1;Vanessa cardui;0.999999459983318;
MN140641;Lepidoptera;Coenonympha arcania;NA;0.172;Coenonympha arcania;0.963036309473038;
MN140642;Lepidoptera;Melitaea trivia;Melitaea trivia;1;Melitaea trivia;0.999999999977604;
MN140643;Lepidoptera;Erynnis tages;Erynnis tages;1;Erynnis tages;0.999999281987018;
MN140644;Lepidoptera;Plebejus argus;Plebejus argus;0.013;Plebejus argus;0.99029909759821;
MN140645;Lepidoptera;Gonepteryx cleopatra;Gonepteryx cleopatra;0.998;Gonepteryx cleopatra;0.999118636582266;
MN140647;Lepidoptera;Pyrgus cacaliae;Pyrgus cacaliae;0.001;Pyrgus cacaliae;0.997779968725098;
MN140649;Lepidoptera;Pieris napi;NA;0.011;Pieris napi;0.801985394724814;
MN140652;Lepidoptera;Maniola cecilia;Pyronia cecilia;NA;Pyronia cecilia;1;
MN140653;Lepidoptera;Erebia nivalis;NA;NA;NA;0.999999999999947;
MN140654;Lepidoptera;Ochlodes sylvanus;NA;0.119;Ochlodes sylvanus;0.997373902608318;
MN140656;Lepidoptera;Maniola nurag;Maniola nurag;1;Maniola nurag;0.94592398344326;
MN140657;Lepidoptera;Vanessa atalanta;Vanessa atalanta;1;Vanessa atalanta;0.999999999501853;
MN140661;Lepidoptera;Polyommatus damon;Polyommatus damon;1;Polyommatus damon;0.999999897832231;
MN140664;Lepidoptera;Lasiommata maera;Lasiommata maera;1;Lasiommata maera;0.99999683404287;
MN140665;Lepidoptera;Lycaena virgaureae;NA;0.186;Lycaena virgaureae;0.999491227342155;
MN140666;Lepidoptera;Fabriciana niobe;NA;0.005;Fabriciana niobe;0.992125835358755;
MN140667;Lepidoptera;Ochlodes sylvanus;NA;0.121;Ochlodes sylvanus;0.997242099793337;

MN140668;Lepidoptera;Thymelicus sylvestris;Thymelicus sylvestris;1;Thymelicus sylvestris;0.999999985214032;
MN140669;Lepidoptera;Pieris napi;NA;0.011;Pieris napi;0.801985394724814;
MN140670;Lepidoptera;Cyaniris semiargus;NA;NA;Cyaniris semiargus;0.999051841766953;
MN140672;Lepidoptera;Boloria euphrosyne;Boloria euphrosyne;1;Boloria euphrosyne;0.999998621448425;
MN140675;Lepidoptera;Cyaniris semiargus;Cyaniris semiargus;NA;Cyaniris semiargus;0.999804935442027;
MN140677;Lepidoptera;Callophrys rubi;NA;0.001;Callophrys rubi;0.99870518510598;
MN140678;Lepidoptera;Glaucopsyche alexis;Glaucopsyche alexis;0.001;Glaucopsyche alexis;0.94042380088325;
MN140681;Lepidoptera;Polyommatus icarus;Polyommatus icarus;0.007;Polyommatus icarus;0.999507484826389;
MN140681;Lepidoptera;Polyommatus icarus;Polyommatus icarus;0.007;Polyommatus icarus;0.999507484826389;
MN140683;Lepidoptera;Thymelicus sylvestris;Thymelicus sylvestris;1;Thymelicus sylvestris;0.999999984752464;
MN140684;Lepidoptera;Aporia crataegi;NA;0.003;Aporia crataegi;0.999998656079776;
MN140685;Lepidoptera;Lycaena virgaureae;NA;0.186;Lycaena virgaureae;0.999491227342155;
MN140687;Lepidoptera;Erynnis tages;Erynnis tages;1;Erynnis tages;0.999999281987018;
MN140688;Lepidoptera;Polyommatus daphnis;Polyommatus daphnis;1;Polyommatus daphnis;0.999626942892628;
MN140689;Lepidoptera;Araschnia levana;Araschnia levana;1;Araschnia levana;0.999999772187832;
MN140691;Lepidoptera;Erebia pronoe;Erebia pronoe;1;Erebia pronoe;0.999250891771858;
MN140692;Lepidoptera;Glaucopsyche melanops;Glaucopsyche melanops;1;Glaucopsyche melanops;0.999952924254607;
MN140693;Lepidoptera;Cupido minimus;NA;0.001;Cupido minimus;0.999803920701362;
MN140694;Lepidoptera;Lycaena tityrus;Lycaena tityrus;1;Lycaena tityrus;0.999995729087172;
MN140696;Lepidoptera;Vanessa cardui;Vanessa cardui;1;Vanessa cardui;0.999991578978624;
MN140700;Lepidoptera;Maniola jurtina;Maniola jurtina;NA;Maniola jurtina;0.987213863396895;
MN140701;Lepidoptera;Pieris napi;NA;0.011;Pieris napi;0.801985394724814;
MN140702;Lepidoptera;Cyaniris semiargus;NA;NA;Cyaniris semiargus;0.999051841766953;
MN140703;Lepidoptera;Lycaena phlaeas;Lycaena phlaeas;1;Lycaena phlaeas;0.999999993939099;
MN140704;Lepidoptera;Vanessa atalanta;Vanessa atalanta;1;Vanessa atalanta;0.999999999757762;
MN140705;Lepidoptera;Erebia tyndarus;NA;NA;NA;0.999999999999721;
MN140706;Lepidoptera;Erebia meolans;Erebia meolans;NA;Erebia meolans;0.999835953426128;
MN140707;Lepidoptera;Fabriciana niobe;NA;0.005;Fabriciana niobe;0.992125835358755;
MN140708;Lepidoptera;Agriades glandon;NA;0.002;Agriades glandon;0.987189585708506;
MN140709;Lepidoptera;Aricia cramera;Aricia cramera;1;Aricia cramera;0.999993731037033;
MN140710;Lepidoptera;Maniola jurtina;NA;0.012;Maniola jurtina;0.91755716175482;
MN140711;Lepidoptera;Polyommatus eros;NA;0.005;Polyommatus eros;0.95275169203039;
MN140712;Lepidoptera;Coenonympha pamphilus;Coenonympha pamphilus;1;Coenonympha pamphilus;0.999999999999744;
MN140713;Lepidoptera;Fabriciana niobe;Fabriciana niobe;0.001;Fabriciana niobe;0.93186202957215;
MN140714;Lepidoptera;Arethusana arethusa;NA;0.153;Arethusana arethusa;0.999652039046744;
MN140715;Lepidoptera;Cupido argiades;NA;0.499;NA;0.999999999984682;
MN140717;Lepidoptera;Lycaena phlaeas;Lycaena phlaeas;1;Lycaena phlaeas;0.99999999340352;
MN140718;Lepidoptera;Celastrina argiolus;Celastrina argiolus;1;Celastrina argiolus;0.999527385150531;
MN140719;Lepidoptera;Ochlodes sylvanus;NA;0.316;Ochlodes sylvanus;0.996318754838625;
MN140720;Lepidoptera;Pyrgus malvoides;NA;0.076;Pyrgus malvoides;0.887854315566122;
MN140721;Lepidoptera;Erebia melampus;Erebia melampus;0.011;Erebia melampus;0.975499903768874;
MN140722;Lepidoptera;Lasiommata maera;Lasiommata maera;1;Lasiommata maera;0.999996552555305;
MN140723;Lepidoptera;Aricia artaxerxes;NA;0.394;Aricia artaxerxes;0.999502890976379;
MN140725;Lepidoptera;Coenonympha glycerion;NA;0.213;Coenonympha glycerion;0.999998402638365;
MN140727;Lepidoptera;Celastrina argiolus;Celastrina argiolus;1;Celastrina argiolus;0.999527385150531;
MN140729;Lepidoptera;Aporia crataegi;NA;NA;Aporia crataegi;0.999989613886149;
MN140731;Lepidoptera;Carcharodus alceae;Carcharodus alceae;0.996;Carcharodus alceae;0.999999976440904;
MN140732;Lepidoptera;Erebia euryale;NA;NA;Erebia euryale;0.945887149341484;
MN140733;Lepidoptera;Cupido minimus;NA;0.009;Cupido minimus;0.999791453712643;
MN140735;Lepidoptera;Pyrgus armoricanus;Pyrgus armoricanus;1;Pyrgus armoricanus;0.999997353848817;
MN140736;Lepidoptera;Boloria selene;NA;0.107;Boloria selene;0.999999824620305;
MN140737;Lepidoptera;Pyrgus onopordi;Pyrgus onopordi;1;Pyrgus onopordi;0.999997111229755;
MN140738;Lepidoptera;Euphydryas aurinia;NA;0.31;Euphydryas aurinia;0.993387708761386;
MN140739;Lepidoptera;Brintesia circe;Brintesia circe;1;Brintesia circe;0.99999969821626;
MN140741;Lepidoptera;Celastrina argiolus;Celastrina argiolus;1;Celastrina argiolus;0.999527385150531;
MN140742;Lepidoptera;Polyommatus dorylas;Polyommatus dorylas;0.008;Polyommatus dorylas;0.991281865899703;
MN140743;Lepidoptera;Minois dryas;NA;0.025;Minois dryas;0.850746026247397;
MN140745;Lepidoptera;Colias alfacariensis;Colias alfacariensis;0.001;Colias alfacariensis;0.999926026317526;
MN140750;Lepidoptera;Thymelicus acteon;Thymelicus acteon;1;Thymelicus acteon;0.99999999752248;
MN140752;Lepidoptera;Euphydryas intermedia;NA;NA;NA;0.999999999996973;
MN140753;Lepidoptera;Ochlodes sylvanus;NA;0.316;Ochlodes sylvanus;0.996318754838625;

MN140754;Lepidoptera;Lycaena alciphron;Lycaena alciphron;1;Lycaena alciphron;0.999999813633071;
MN140755;Lepidoptera;Gonepteryx rhamni;NA;0.202;Gonepteryx rhamni;0.990544908845376;
MN140757;Lepidoptera;Maniola jurtina;NA;0.171;Maniola jurtina;0.963697912386445;
MN140758;Lepidoptera;Pyrgus serratulae;Pyrgus serratulae;1;Pyrgus serratulae;0.99999989567508;
MN140759;Lepidoptera;Ochlodes sylvanus;NA;0.316;Ochlodes sylvanus;0.996318754838625;
MN140761;Lepidoptera;Pyrgus andromedae;Pyrgus andromedae;0.001;Pyrgus andromedae;0.985699239604726;
MN140763;Lepidoptera;Polyommatus icarus;NA;0.002;Polyommatus icarus;0.9989029998049;
MN140763;Lepidoptera;Polyommatus icarus;NA;0.002;Polyommatus icarus;0.9989029998049;
MN140764;Lepidoptera;Ochlodes sylvanus;NA;0.316;Ochlodes sylvanus;0.996318754838625;
MN140765;Lepidoptera;Lasiommata maera;Lasiommata maera;1;Lasiommata maera;0.999999176235688;
MN140766;Lepidoptera;Callophrys rubi;NA;0.019;Callophrys rubi;0.998201459637982;
MN140767;Lepidoptera;Lycaena dispar;Lycaena dispar;1;Lycaena dispar;0.999999990743959;
MN140768;Lepidoptera;Colias hyale;Colias hyale;0.015;Colias hyale;0.999997884296128;
MN140770;Lepidoptera;Melitaea didyma;Melitaea didyma;1;Melitaea didyma;0.999803304909779;
MN140771;Lepidoptera;Favonius quercus;NA;NA;Favonius quercus;0.73046069582187;
MN140772;Lepidoptera;Argynnis paphia;Argynnis paphia;1;Argynnis paphia;0.999999991389927;
MN140774;Lepidoptera;Vanessa atalanta;Vanessa atalanta;1;Vanessa atalanta;0.999999999297358;
MN140775;Lepidoptera;Boloria euphrosyne;Boloria euphrosyne;1;Boloria euphrosyne;0.999999820119927;
MN140777;Lepidoptera;Carcharodus alceae;Carcharodus alceae;1;Carcharodus alceae;0.999999998465938;
MN140778;Lepidoptera;Pyrgus armoricanus;Pyrgus armoricanus;1;Pyrgus armoricanus;0.999989012351076;
MN140779;Lepidoptera;Erebia oeme;NA;0.161;Erebia oeme;0.999999999457856;
MN140780;Lepidoptera;Thymelicus lineola;Thymelicus lineola;1;Thymelicus lineola;0.999993782911474;
MN140781;Lepidoptera;Polyommatus daphnis;Polyommatus daphnis;1;Polyommatus daphnis;0.99977716269156;
MN140782;Lepidoptera;Gegenes pumilio;Gegenes pumilio;1;Gegenes pumilio;0.999986726679266;
MN140783;Lepidoptera;Coenonympha pamphilus;Coenonympha pamphilus;1;Coenonympha pamphilus;0.999999999999574;
MN140784;Lepidoptera;Pyrgus malvoides;NA;0.176;Pyrgus malvoides;0.940490890379026;
MN140788;Lepidoptera;Maniola jurtina;NA;0.012;Maniola jurtina;0.91755716175482;
MN140789;Lepidoptera;Colias alfacariensis;Colias alfacariensis;1;Colias alfacariensis;0.999986994131001;
MN140790;Lepidoptera;Thymelicus acteon;Thymelicus acteon;1;Thymelicus acteon;0.999999998964029;
MN140791;Lepidoptera;Limenitis camilla;NA;0.025;Limenitis camilla;0.999994294272475;
MN140794;Lepidoptera;Pieris napi;NA;0.011;Pieris napi;0.801985394724814;
MN140795;Lepidoptera;Pieris napi;NA;0.011;Pieris napi;0.801985394724814;
MN140796;Lepidoptera;Coenonympha pamphilus;Coenonympha pamphilus;1;Coenonympha pamphilus;0.999999999999744;
MN140798;Lepidoptera;Polyommatus icarus;NA;0.001;Polyommatus icarus;0.981285528541973;
MN140798;Lepidoptera;Polyommatus icarus;NA;0.001;Polyommatus icarus;0.981285528541973;
MN140800;Lepidoptera;Coenonympha pamphilus;Coenonympha pamphilus;1;Coenonympha pamphilus;0.999999999999744;
MN140801;Lepidoptera;Erebia montana;Erebia montana;1;Erebia montana;0.999047244700624;
MN140802;Lepidoptera;Nymphalis egea;Polygonia egea;1;Polygonia egea;0.999999878357791;
MN140803;Lepidoptera;Melitaea deione;Melitaea deione;1;Melitaea deione;0.999999545882719;
MN140804;Lepidoptera;Brenthis daphne;NA;0.102;Brenthis daphne;0.717134521611551;
MN140805;Lepidoptera;Aricia eumedon;Eumedonia eumedon;0.001;Eumedonia eumedon;0.998109622286715;
MN140806;Lepidoptera;Aporia crataegi;NA;NA;Aporia crataegi;0.999996781126733;
MN140807;Lepidoptera;Aricia cramera;Aricia cramera;1;Aricia cramera;0.999995452012538;
MN140808;Lepidoptera;Parnassius apollo;Parnassius apollo;1;Parnassius apollo;0.995259714099824;
MN140809;Lepidoptera;Cyaniris semiargus;NA;NA;Cyaniris semiargus;0.999051841766953;
MN140811;Lepidoptera;Araschnia levana;Araschnia levana;1;Araschnia levana;0.999999772187832;
MN140813;Lepidoptera;Polyommatus eros;NA;0.004;Polyommatus eros;0.972411810082555;
MN140814;Lepidoptera;Polyommatus icarus;NA;0.004;Polyommatus icarus;0.999481683747521;
MN140814;Lepidoptera;Polyommatus icarus;NA;0.004;Polyommatus icarus;0.999481683747521;
MN140815;Lepidoptera;Anthocharis cardamines;Anthocharis cardamines;1;Anthocharis cardamines;0.999999990347561;
MN140817;Lepidoptera;Nymphalis c-album;NA;0.215;NA;0.999999192918557;
MN140819;Lepidoptera;Aglais urticae;Aglais urticae;0.001;Aglais urticae;0.994396335177485;
MN140820;Lepidoptera;Polyommatus daphnis;Polyommatus daphnis;1;Polyommatus daphnis;0.999874472754722;
MN140821;Lepidoptera;Pieris napi;NA;0.011;Pieris napi;0.801985394724814;
MN140822;Lepidoptera;Maniola jurtina;NA;0.001;Maniola jurtina;0.939220105860875;
MN140823;Lepidoptera;Colias alfacariensis;Colias alfacariensis;0.001;Colias alfacariensis;0.999926026317526;
MN140824;Lepidoptera;Lycaena phlaeas;Lycaena phlaeas;1;Lycaena phlaeas;0.999999996363186;
MN140825;Lepidoptera;Aricia cramera;Aricia cramera;1;Aricia cramera;0.999994067325963;
MN140826;Lepidoptera;Colias crocea;NA;0.059;NA;0.999999999999738;
MN140827;Lepidoptera;Pyrgus malvoides;NA;0.176;Pyrgus malvoides;0.940490890379026;
MN140828;Lepidoptera;Pieris napi;NA;0.011;Pieris napi;0.801985394724814;
MN140829;Lepidoptera;Pyrgus malvoides;Pyrgus malvoides;0.009;Pyrgus malvoides;0.912953074572214;
MN140830;Lepidoptera;Polyommatus amandus;Polyommatus amandus;0.006;Polyommatus amandus;0.999981460994076;
MN140831;Lepidoptera;Aglais urticae;NA;0.002;Aglais urticae;0.987497593219019;

MN140832;Lepidoptera;Hipparchia hermione;NA;NA;NA;0.999999999999991;
MN140833;Lepidoptera;Maniola jurtina;NA;NA;Maniola jurtina;0.977038297978319;
MN140834;Lepidoptera;Agriades orbitulus;Agriades orbitulus;NA;Agriades orbitulus;0.999216256740517;
MN140836;Lepidoptera;Plebejus idas;NA;0.007;NA;0.999546324331549;
MN140837;Lepidoptera;Pieris napi;NA;0.011;Pieris napi;0.801985394724814;
MN140838;Lepidoptera;Favonius quercus;NA;0.176;Favonius quercus;0.828947799604254;
MN140840;Lepidoptera;Aricia agestis;NA;0.069;Aricia agestis;0.995918114091591;
MN140841;Lepidoptera;Chazara briseis;Chazara briseis;1;Chazara briseis;0.999975731693442;
MN140843;Lepidoptera;Lysandra coridon;Lysandra coridon;0.001;Lysandra coridon;0.928646244815605;
MN140844;Lepidoptera;Plebejus idas;NA;0.007;NA;0.999546324331549;
MN140845;Lepidoptera;Colias crocea;NA;0.059;NA;0.999999999999738;
MN140846;Lepidoptera;Melitaea didyma;Melitaea didyma;1;Melitaea didyma;0.999376065333983;
MN140847;Lepidoptera;Coenonympha gardetta;NA;0.316;NA;0.999999999999985;
MN140848;Lepidoptera;Pieris napi;NA;0.011;Pieris napi;0.801985394724814;
MN140849;Lepidoptera;Chazara briseis;Chazara briseis;1;Chazara briseis;0.999975731693442;
MN140850;Lepidoptera;Celastrina argiolus;Celastrina argiolus;1;Celastrina argiolus;0.998625501352879;
MN140851;Lepidoptera;Polyommatus icarus;NA;0.001;Polyommatus icarus;0.981285528541973;
MN140851;Lepidoptera;Polyommatus icarus;NA;0.001;Polyommatus icarus;0.981285528541973;
MN140852;Lepidoptera;Glaucopsyche alexis;Glaucopsyche alexis;0.001;Glaucopsyche alexis;0.94042380088325;
MN140854;Lepidoptera;Coenonympha pamphilus;Coenonympha pamphilus;1;Coenonympha pamphilus;0.999999999999289;
MN140855;Lepidoptera;Maniola jurtina;NA;0.001;Maniola jurtina;0.939220105860875;
MN140857;Lepidoptera;Cupido minimus;NA;0.009;Cupido minimus;0.999791453712643;
MN140859;Lepidoptera;Minois dryas;NA;0.025;Minois dryas;0.850746026247397;
MN140860;Lepidoptera;Erynnis tages;Erynnis tages;1;Erynnis tages;0.999996973757536;
MN140861;Lepidoptera;Boloria thore;Boloria thore;1;Boloria thore;0.999999998359726;
MN140862;Lepidoptera;Lysandra coridon;NA;NA;NA;0.998017994491822;
MN140864;Lepidoptera;Pyrgus malvoides;NA;0.052;Pyrgus malvoides;0.943208768333892;
MN140865;Lepidoptera;Plebejus argus;NA;0.328;Plebejus argus;0.969789734199889;
MN140866;Lepidoptera;Fabriciana adippe;NA;NA;Fabriciana adippe;0.788180479135482;
MN140867;Lepidoptera;Nymphalis c-album;NA;0.215;NA;0.999999192918557;
MN140868;Lepidoptera;Maniola jurtina;NA;0.038;NA;0.999999999999524;
MN140870;Lepidoptera;Maniola jurtina;NA;0.171;Maniola jurtina;0.963697912386445;
MN140871;Lepidoptera;Maniola jurtina;NA;0.171;Maniola jurtina;0.963697912386445;
MN140873;Lepidoptera;Vanessa atalanta;Vanessa atalanta;1;Vanessa atalanta;0.999999999757762;
MN140874;Lepidoptera;Maniola jurtina;NA;0.153;Maniola jurtina;0.908984189778786;
MN140877;Lepidoptera;Vanessa atalanta;Vanessa atalanta;1;Vanessa atalanta;0.999999995828034;
MN140881;Lepidoptera;Colias crocea;NA;0.059;NA;0.999999999999738;
MN140882;Lepidoptera;Brintesia circe;Brintesia circe;NA;Brintesia circe;0.999999813082031;
MN140883;Lepidoptera;Polyommatus humedasae;Polyommatus humedasae;1;Polyommatus humedasae;0.998764240535817;
MN140885;Lepidoptera;Maniola jurtina;NA;NA;Maniola jurtina;0.951668238126128;
MN140888;Lepidoptera;Nymphalis io;NA;0.491;Aglais io;0.930153237246992;
MN140889;Lepidoptera;Lycaena virgaureae;NA;0.079;Lycaena virgaureae;0.99957744599144;
MN140890;Lepidoptera;Vanessa cardui;Vanessa cardui;1;Vanessa cardui;0.999999459983318;
MN140891;Lepidoptera;Maniola jurtina;NA;0.153;Maniola jurtina;0.908984189778786;
MN140892;Lepidoptera;Lycaena phlaeas;Lycaena phlaeas;1;Lycaena phlaeas;0.999999998467302;
MN140893;Lepidoptera;Ochlodes sylvanus;NA;0.316;Ochlodes sylvanus;0.996318754838625;
MN140895;Lepidoptera;Nymphalis antiopa;NA;0.23;Nymphalis antiopa;0.999999944198096;
MN140896;Lepidoptera;Carcharodus alceae;Carcharodus alceae;0.001;Carcharodus alceae;0.999999970193983;
MN140897;Lepidoptera;Polyommatus icarus;NA;0.025;Polyommatus icarus;0.997831523019962;
MN140897;Lepidoptera;Polyommatus icarus;NA;0.025;Polyommatus icarus;0.997831523019962;
MN140898;Lepidoptera;Hipparchia leighebi;Hipparchia leighebi;1;Hipparchia leighebi;0.921532082781005;
[truncated: 795,641 more chars]
