# Supplementary material for: Filling knowledge gaps in insect conservation by leveraging genetic data from public archives
Source: Database (Oxford). 2024 Jan 29;2024:baae002. doi: 10.1093/database/baae002 (PMC10878047; doi:10.1093/database/baae002)
Supplement: baae002_Supp [file baae002_supp.zip › suppl_data/supplementary_figures...docx]

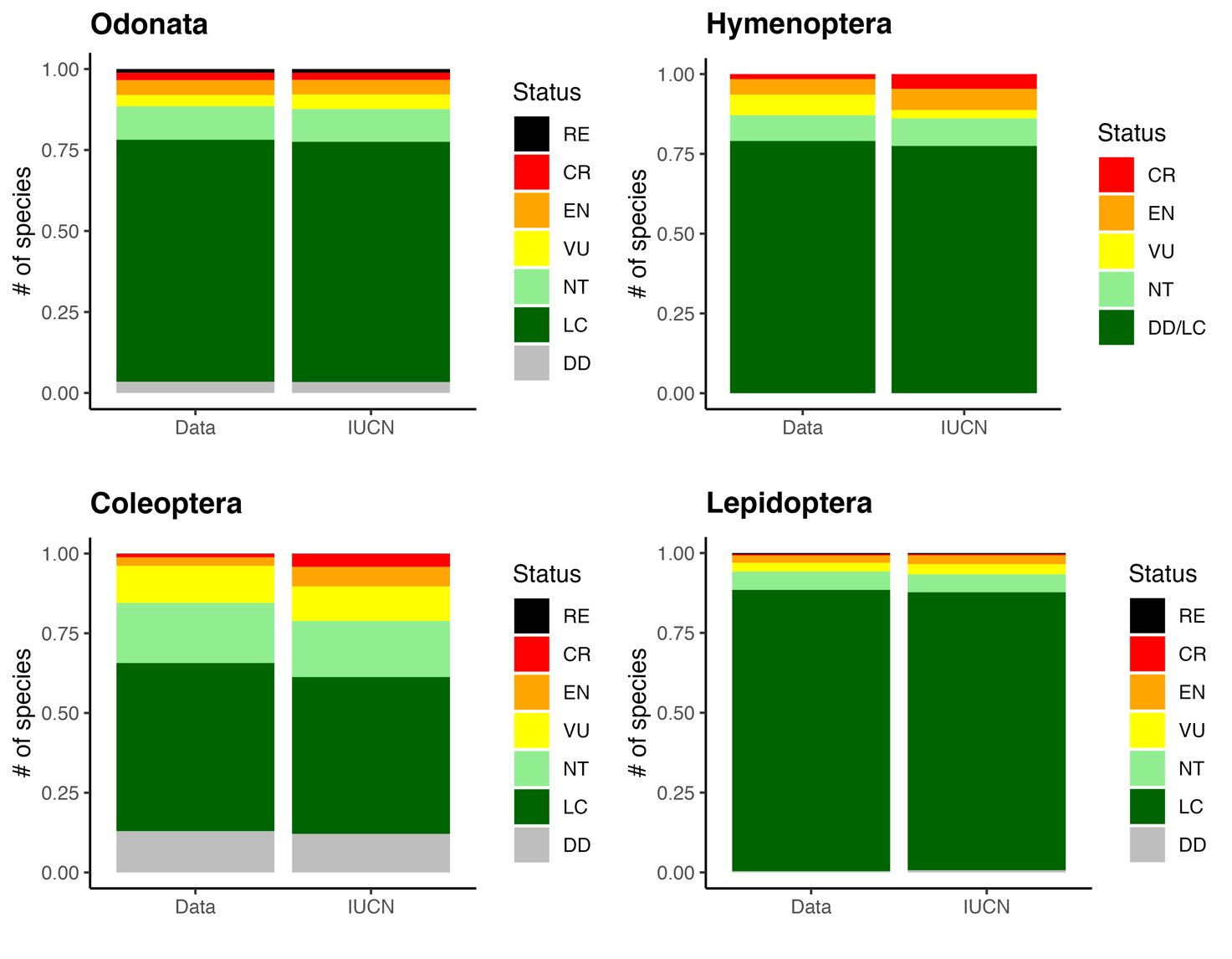


**Figure S1**. Stacked Bar plot showing IUCN status of data retrieved compared with IUCN species lists for the examined taxa in the final database.


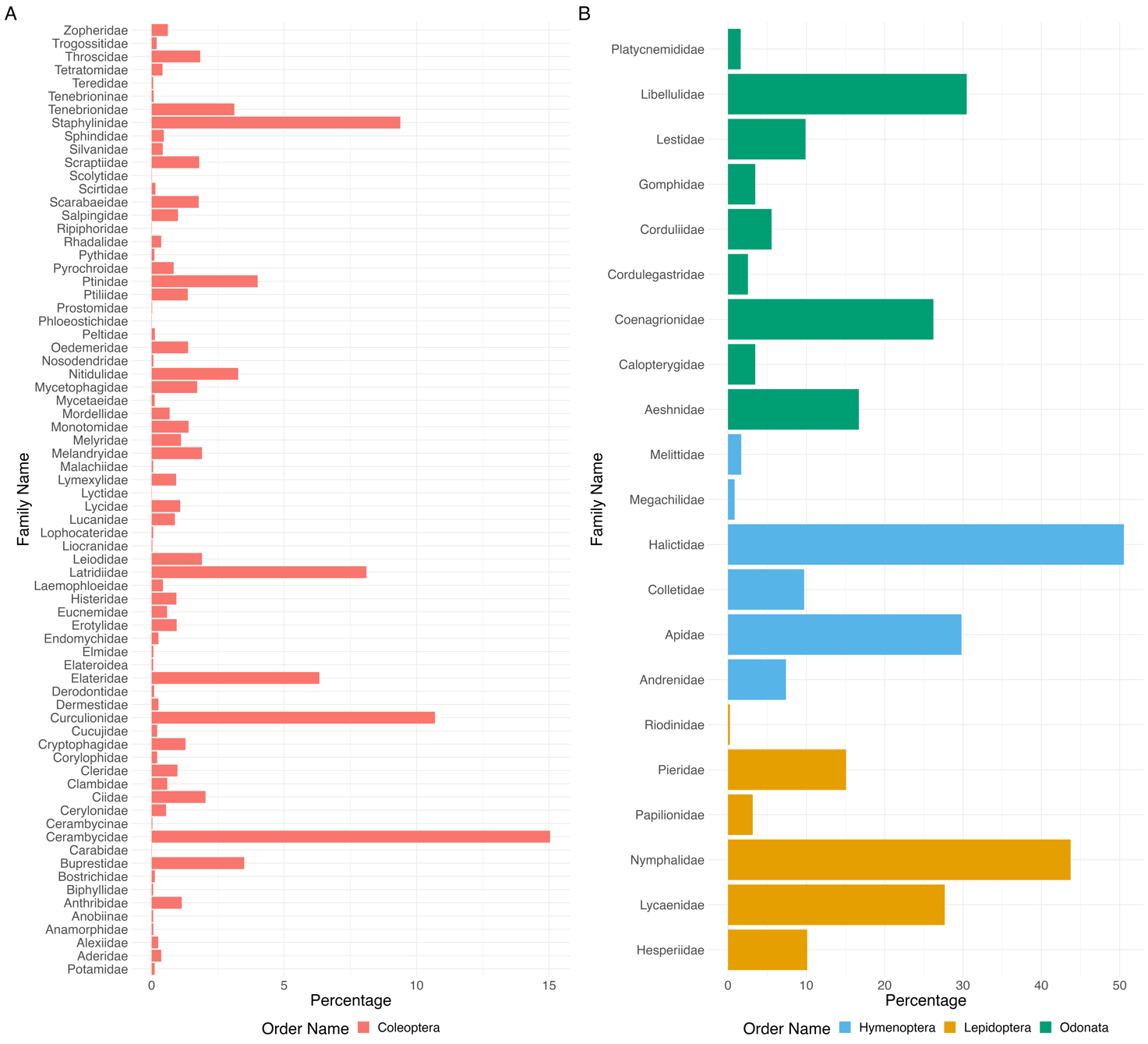


**Figure S2**. Bar plot showing the percentage of sequences for each family for Coleoptera (A) and the other examined taxa (B) in the final database.


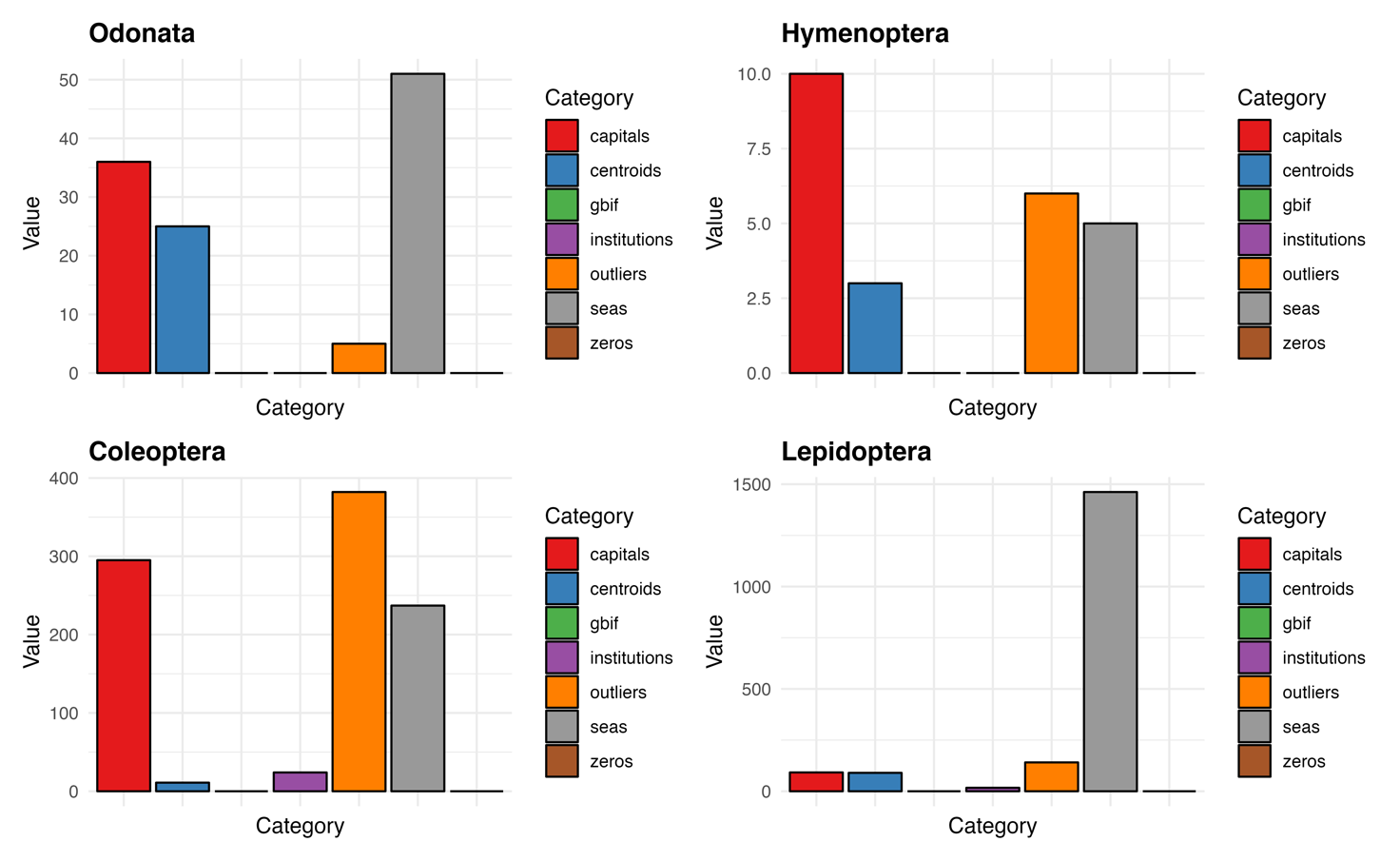


**Figure S3**. Plot showing the different error categories in the records of coordinates after using CoordinateCleaner.

**
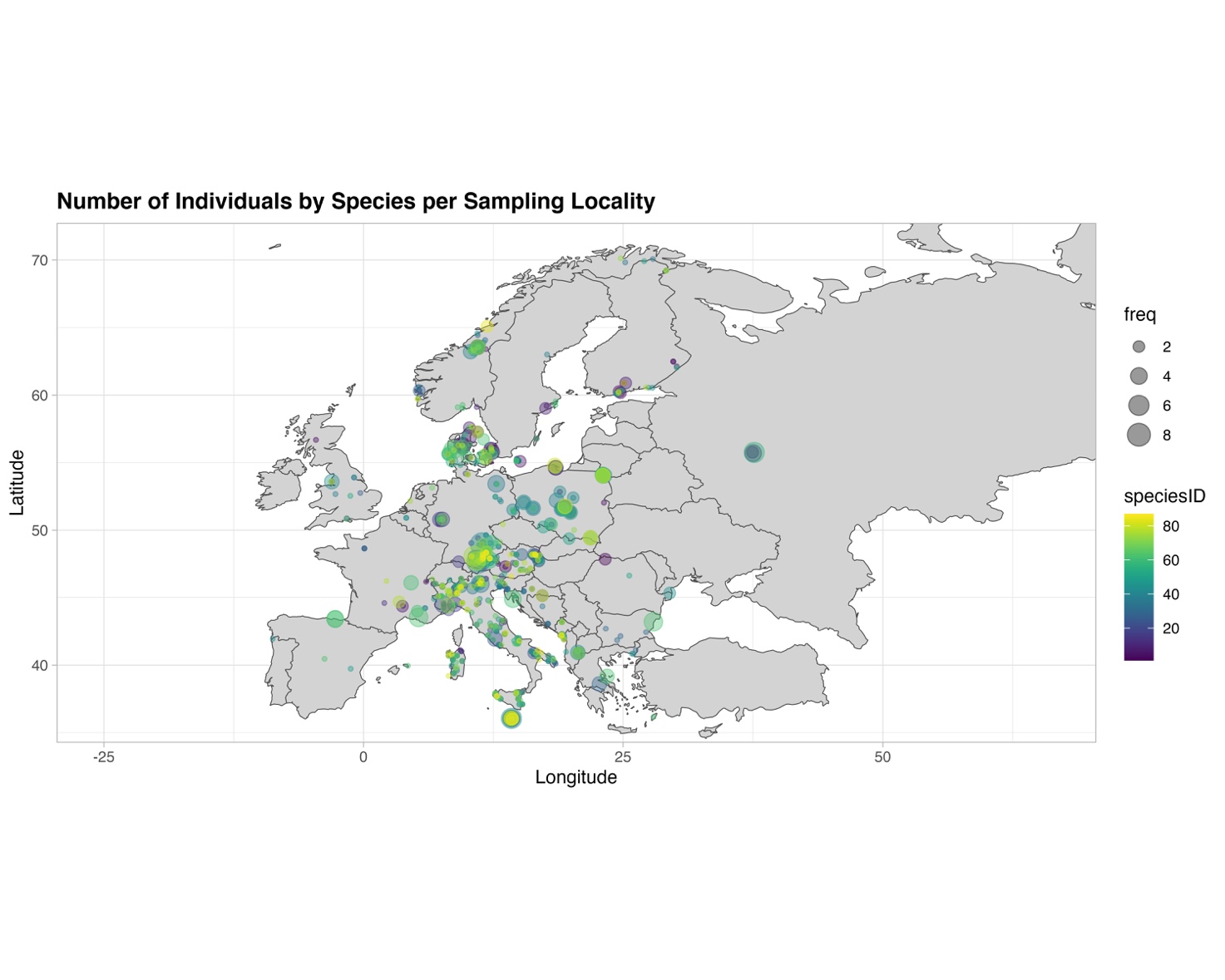
**

**Figure S4.** Graph showing the number of individuals per species per sampling locality in Odonata in the final database.


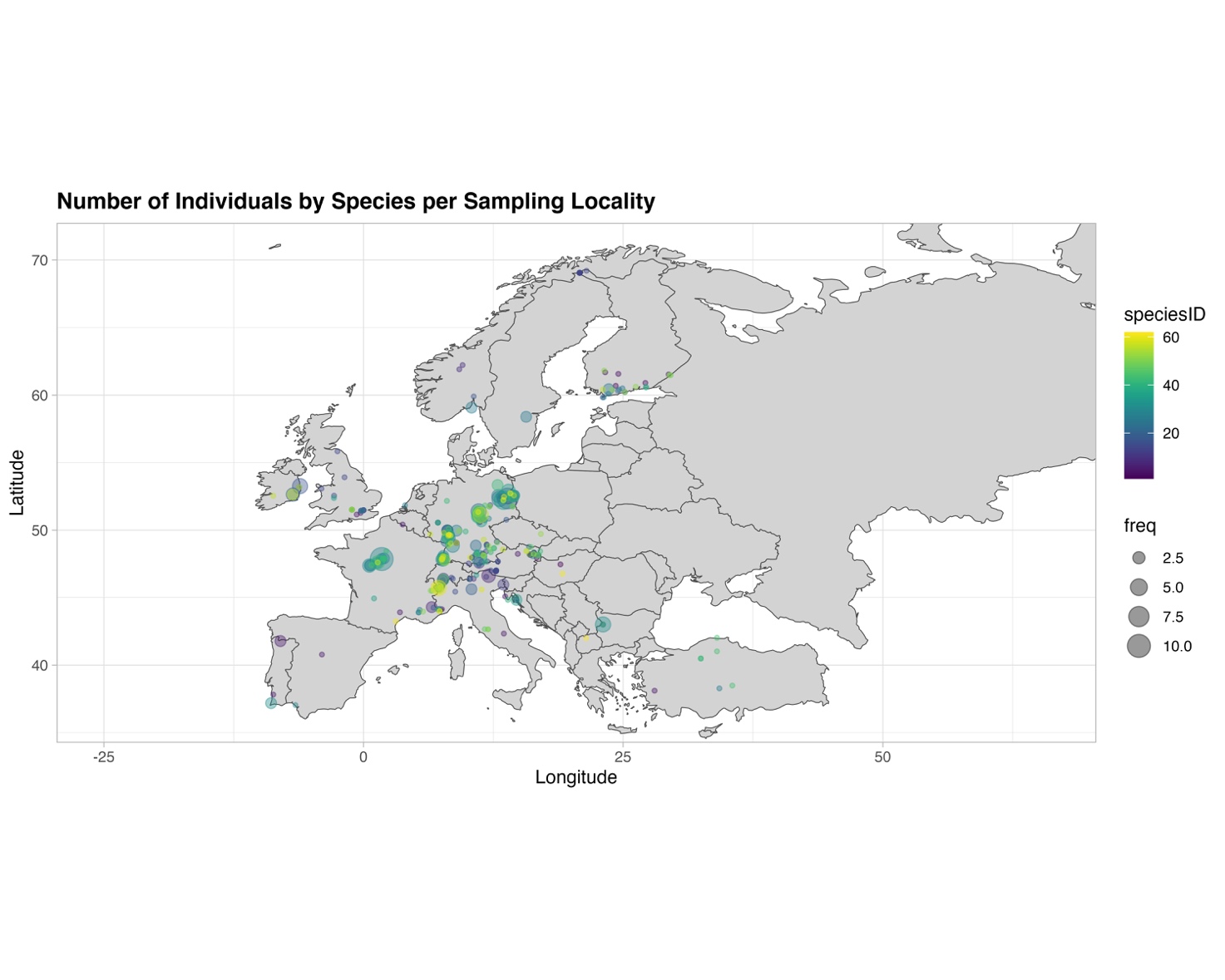
**Figure S5.** Graph showing the number of individuals per species per sampling locality in Hymenoptera in the final database.


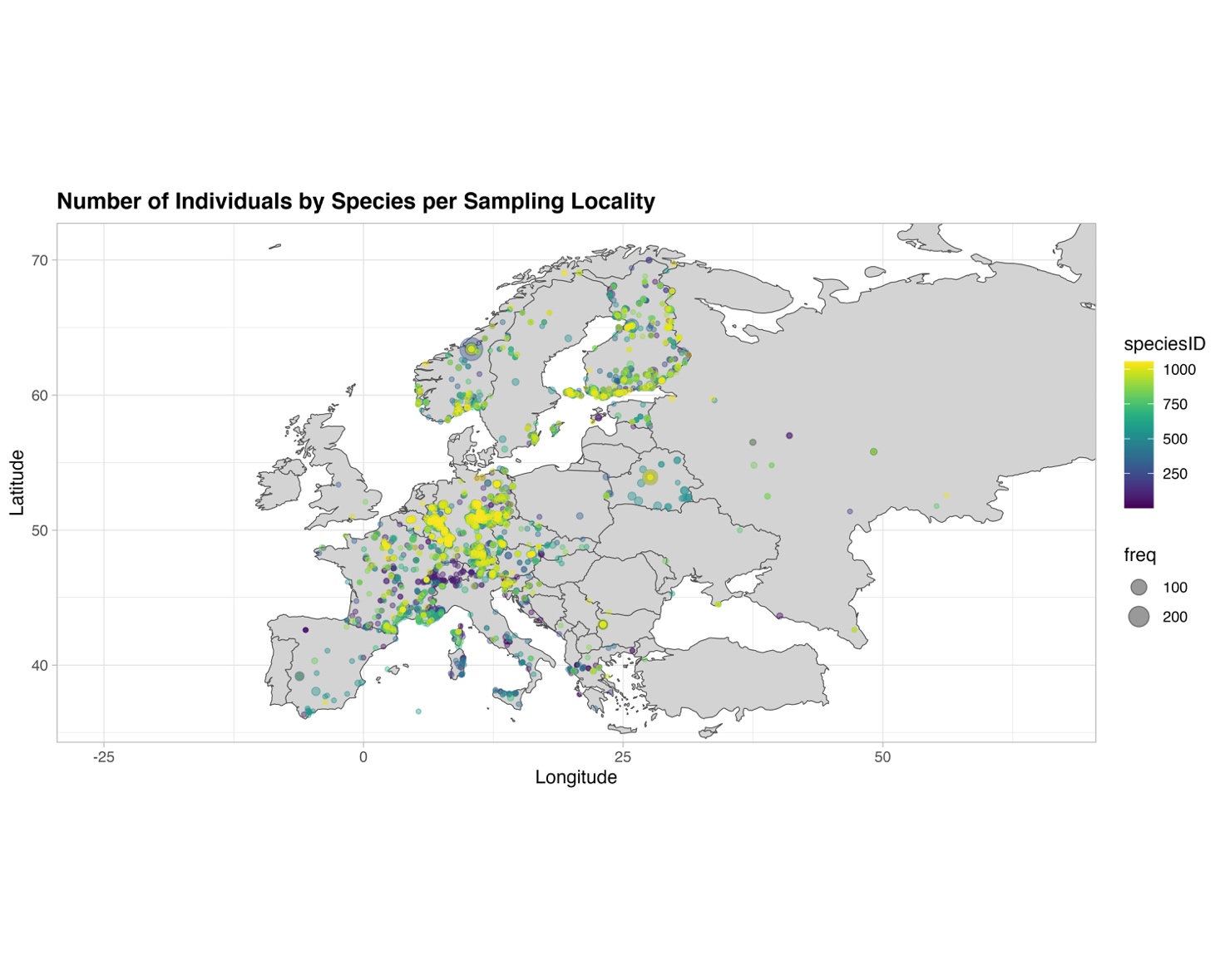


**Figure S6**. Graph showing the number of individuals per species per sampling locality in Coleoptera in the final database.


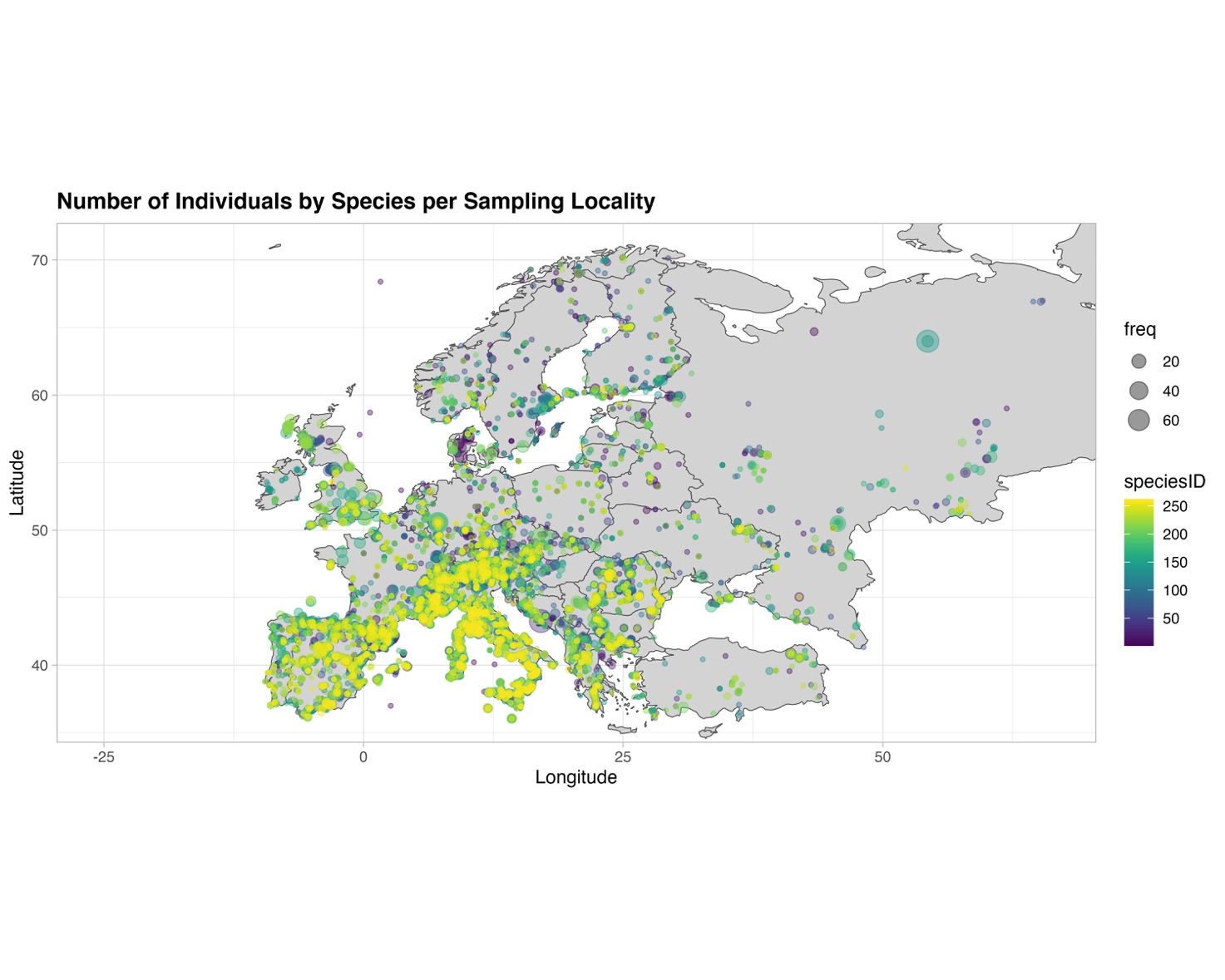


**Figure S7.** Graph showing the number of individuals per species per sampling locality in Lepidoptera in the final database.
