# Supplementary material for: Filling knowledge gaps in insect conservation by leveraging genetic data from public archives
Source: Database (Oxford). 2024 Jan 29;2024:baae002. doi: 10.1093/database/baae002 (PMC10878047; doi:10.1093/database/baae002)
Supplement: baae002_Supp [file baae002_supp.zip › suppl_data/Table_S1_Odonata.docx]

**Table S1***. Table showing Odonata species included in the IUCN Italian Red Lists, the conservation status, whether the species is present in our dataset, and how many individuals.*

| **Species name** | **IUCN status** | **Is the species in our dataset?** | **Number of individuals in the dataset** |
| --- | --- | --- | --- |
| *Aeshna affinis* | LC | YES | 9 |
| *Aeshna caerulea* | NT | YES | 9 |
| *Aeshna cyanea* | LC | YES | 30 |
| *Aeshna grandis* | VU | YES | 15 |
| *Aeshna isosceles* | LC | NO | 0 |
| *Aeshna juncea* | LC | YES | 39 |
| *Aeshna mixta* | LC | YES | 20 |
| *Aeshna subarctica* | VU | YES | 28 |
| *Anax ephippiger* | LC | YES | 4 |
| *Anax imperator* | LC | YES | 27 |
| *Anax parthenope* | LC | YES | 10 |
| *Boyeria irene* | LC | YES | 4 |
| *Brachythemis impartita* | LC | YES | 3 |
| *Brachytron pratense* | LC | YES | 13 |
| *Calopteryx haemorrhoidalis* | LC | YES | 9 |
| *Calopteryx splendens* | LC | YES | 20 |
| *Calopteryx virgo* | LC | YES | 11 |
| *Calopteryx xanthostoma* | LC | YES | 2 |
| *Ceriagrion tenellum* | LC | YES | 8 |
| *Chalcolestes parvidens* | LC | YES | 8 |
| *Chalcolestes viridis* | LC | YES | 20 |
| *Coenagrion caerulescens* | LC | YES | 8 |
| *Coenagrion hastulatum* | LC | YES | 14 |
| *Coenagrion mercuriale* | NT | YES | 13 |
| *Coenagrion ornatum* | DD | YES | 1 |
| *Coenagrion puella* | LC | YES | 3 |
| *Coenagrion pulchellum* | NT | YES | 10 |
| *Coenagrion scitulum* | LC | YES | 10 |
| *Cordulegaster bidentata* | LC | YES | 9 |
| *Cordulegaster boltoni* | LC | NO | 0 |
| *Cordulegaster heros* | NT | YES | 11 |
| *Cordulegaster trinacriae* | NT | YES | 2 |
| *Cordulia aenea* | NT | YES | 21 |
| *Crocothemis erythraea* | LC | YES | 18 |
| *Enallagma cyathigerum* | LC | YES | 52 |
| *Epitheca bimaculata* | RE | YES | 1 |
| *Erythromma lindenii* | LC | YES | 41 |
| *Erythromma najas* | EN | YES | 21 |
| *Erythromma viridulum* | LC | YES | 16 |
| *Gomphus flavipes* | LC | YES | 1 |
| *Gomphus vulgatissimus* | LC | YES | 20 |
| *Ischnura elegans* | LC | YES | 68 |
| *Ischnura fountainei* | VU | NO | 0 |
| *Ischnura genei* | LC | YES | 21 |
| *Ischnura pumilio* | LC | YES | 11 |
| *Lestes barbarus* | LC | YES | 11 |
| *Lestes dryas* | EN | YES | 11 |
| *Lestes macrostigma* | LC | YES | 5 |
| *Lestes sponsa* | LC | YES | 15 |
| *Lestes virens* | LC | YES | 22 |
| *Leucorrhinia dubia* | LC | YES | 24 |
| *Leucorrhinia pectoralis* | EN | YES | 7 |
| *Libellula depressa* | LC | YES | 15 |
| *Libellula fulva* | LC | YES | 13 |
| *Libellula quadrimaculata* | LC | YES | 36 |
| *Lindenia tetraphylla* | NT | YES | 7 |
| *Nehalennia speciosa* | CR | YES | 18 |
| *Onychogomphus uncatus* | LC | YES | 4 |
| *Ophiogomphus cecilia* | LC | YES | 13 |
| *Orthetrum albistylum* | LC | YES | 4 |
| *Orthetrum brunneum* | LC | YES | 16 |
| *Orthetrum cancellatum* | LC | YES | 58 |
| *Orthetrum coerulescens* | LC | YES | 67 |
| *Orthetrum nitidinerve* | DD | YES | 2 |
| *Orthetrum trinacria* | LC | YES | 4 |
| *Oxygastra curtisii* | NT | YES | 3 |
| *Paragomphus genei* | LC | YES | 3 |
| *Platycnemis pennipes* | LC | YES | 21 |
| *Pyrrhosoma nymphula* | LC | YES | 24 |
| *Selysiothemis nigra* | LC | YES | 3 |
| *Somatochlora alpestris* | LC | YES | 6 |
| *Somatochlora arctica* | NT | YES | 8 |
| *Somatochlora flavomaculata* | LC | YES | 14 |
| *Somatochlora meridionalis* | LC | YES | 10 |
| *Somatochlora metallica* | LC | YES | 9 |
| *Sympecma fusca* | LC | YES | 24 |
| *Sympecma paedisca* | CR | YES | 12 |
| *Sympetrum danae* | LC | YES | 31 |
| *Sympetrum depressiusculum* | EN | YES | 3 |
| *Sympetrum flaveolum* | VU | YES | 6 |
| *Sympetrum fonscolombii* | LC | YES | 20 |
| *Sympetrum meridionale* | LC | YES | 10 |
| *Sympetrum pedemontanum* | LC | YES | 5 |
| *Sympetrum sanguineum* | LC | YES | 16 |
| *Sympetrum striolatum* | LC | YES | 15 |
| *Sympetrum vulgatum* | LC | YES | 11 |
| *Trithemis annulata* | LC | YES | 6 |
| *Zygonyx torridus* | DD | YES | 1 |
