# Supplementary material for: Filling knowledge gaps in insect conservation by leveraging genetic data from public archives
Source: Database (Oxford). 2024 Jan 29;2024:baae002. doi: 10.1093/database/baae002 (PMC10878047; doi:10.1093/database/baae002)
Supplement: baae002_Supp [file baae002_supp.zip › suppl_data/Table_S2_Hymoneptera.docx]

**Table S2***. Table showing the Hymenoptera species included in the IUCN Italian Red Lists, the conservation status, whether the species is present in our dataset, and how many individuals.*

| **Species name** | **IUCN status** | **Is the species in our dataset?** | **Number of individuals in the dataset** |
| --- | --- | --- | --- |
| *Amegilla fasciata* | DD/LC | NO | 0 |
| *Ammobatoides abdominalis* | NT | NO | 0 |
| *Andrena aberrans* | DD/LC | YES | 2 |
| *Andrena argentata* | DD/LC | YES | 6 |
| *Andrena binominata* | DD/LC | NO | 0 |
| *Andrena boyerella* | DD/LC | NO | 0 |
| *Andrena curvana* | DD/LC | NO | 0 |
| *Andrena diomedia* | DD/LC | NO | 0 |
| *Andrena fulvida* | DD/LC | YES | 7 |
| *Andrena hattorfiana* | DD/LC | YES | 23 |
| *Andrena incisa* | DD/LC | NO | 0 |
| *Andrena iohannescaroli* | DD/LC | NO | 0 |
| *Andrena nobilis* | DD/LC | NO | 0 |
| *Andrena ovatula* | DD/LC | YES | 24 |
| *Andrena palumba* | EN | NO | 0 |
| *Andrena panurgina* | DD/LC | NO | 0 |
| *Andrena rhyssonota* | DD/LC | NO | 0 |
| *Andrena siciliana* | CR (PE) | NO | 0 |
| *Andrena suerinensis* | DD/LC | YES | 4 |
| *Anthidium montanum* | DD/LC | YES | 4 |
| *Anthophora calcarata* | DD/LC | NO | 0 |
| *Anthophora femorata* | DD/LC | NO | 0 |
| *Anthophora ferruginea* | DD/LC | NO | 0 |
| *Anthophora fulvitarsis* | DD/LC | NO | 0 |
| *Anthophora larvata* | DD/LC | NO | 0 |
| *Anthophora nigrovittata* | DD/LC | YES | 2 |
| *Anthophora pruinosa* | DD/LC | NO | 0 |
| *Anthophora sichelii* | DD/LC | NO | 0 |
| *Anthophora uniciliata* | DD/LC | NO | 0 |
| *Bombus alpinus* | EN | YES | 6 |
| *Bombus brodmannicus* | EN | NO | 0 |
| *Bombus confusus* | CR (PE) | YES | 2 |
| *Bombus gerstaeckeri* | DD/LC | YES | 5 |
| *Bombus haematurus* | DD/LC | YES | 2 |
| *Bombus inexspectatus* | DD/LC | NO | 0 |
| *Bombus konradini* | EN | NO | 0 |
| *Bombus mendax* | DD/LC | YES | 6 |
| *Bombus monticola* | DD/LC | YES | 20 |
| *Bombus mucidus* | DD/LC | YES | 5 |
| *Bombus muscorum* | VU | YES | 3 |
| *Bombus perezi* | DD/LC | NO | 0 |
| *Bombus xanthopus* | DD/LC | NO | 0 |
| *Camptopoeum friesei* | DD/LC | NO | 0 |
| *Chelostoma stefanii* | DD/LC | NO | 0 |
| *Colletes acutiformis* | DD/LC | NO | 0 |
| *Colletes albomaculatus* | DD/LC | NO | 0 |
| *Colletes collaris* | EN | NO | 0 |
| *Colletes floralis* | DD/LC | YES | 11 |
| *Colletes fodiens* | DD/LC | YES | 18 |
| *Colletes foveolaris* | DD/LC | NO | 0 |
| *Colletes graeffei* | DD/LC | YES | 2 |
| *Colletes succinctus* | DD/LC | YES | 10 |
| *Colletes tuberculatus* | NT | NO | 0 |
| *Colletes wolfi* | EN | NO | 0 |
| *Dasypoda argentata* | DD/LC | YES | 1 |
| *Dasypoda braccata* | CR | NO | 0 |
| *Dasypoda suripes* | EN | YES | 1 |
| *Dufourea dentiventris* | DD/LC | YES | 9 |
| *Dufourea inermis* | DD/LC | YES | 1 |
| *Dufourea minuta* | DD/LC | YES | 7 |
| *Epeolus compar* | DD/LC | NO | 0 |
| *Epeolus cruciger* | DD/LC | YES | 22 |
| *Epeolus fasciatus* | DD/LC | NO | 0 |
| *Epeolus siculus* | DD/LC | NO | 0 |
| *Epeolus tarsalis* | DD/LC | YES | 1 |
| *Eucera albofasciata* | DD/LC | NO | 0 |
| *Habropoda ezonata* | DD/LC | NO | 0 |
| *Halictus carinthiacus* | EN | NO | 0 |
| *Halictus leucaheneus* | DD/LC | YES | 12 |
| *Halictus mediterranellus* | DD/LC | NO | 0 |
| *Halictus pseudotetrazonius* | DD/LC | NO | 0 |
| *Halictus quadricinctus* | DD/LC | YES | 55 |
| *Heriades punctulifera* | DD/LC | NO | 0 |
| *Hoplitis occidentalis* | DD/LC | NO | 0 |
| *Hoplitis saxialis* | DD/LC | NO | 0 |
| *Hylaeus adriaticus* | DD/LC | NO | 0 |
| *Hylaeus crassanus* | DD/LC | NO | 0 |
| *Hylaeus pfankuchi* | DD/LC | YES | 5 |
| *Lasioglossum algirum* | DD/LC | NO | 0 |
| *Lasioglossum angusticeps* | DD/LC | YES | 7 |
| *Lasioglossum brevicorne* | DD/LC | YES | 2 |
| *Lasioglossum clypeare* | VU | YES | 2 |
| *Lasioglossum convexiusculum* | DD/LC | NO | 0 |
| *Lasioglossum costulatum* | DD/LC | YES | 17 |
| *Lasioglossum duckei* | DD/LC | NO | 0 |
| *Lasioglossum intermedium* | NT | YES | 4 |
| *Lasioglossum laevigatum* | DD/LC | YES | 10 |
| *Lasioglossum littorale* | NT | NO | 0 |
| *Lasioglossum majus* | DD/LC | YES | 5 |
| *Lasioglossum marginellum* | NT | YES | 2 |
| *Lasioglossum minutulum* | NT | YES | 5 |
| *Lasioglossum podolicum* | NT | NO | 0 |
| *Lasioglossum prasinum* | NT | NO | 0 |
| *Lasioglossum pygmaeum* | DD/LC | YES | 10 |
| *Lasioglossum quadrinotatulum* | VU | YES | 7 |
| *Lasioglossum quadrisignatum* | DD/LC | NO | 0 |
| *Lasioglossum setulellum* | NT | NO | 0 |
| *Lasioglossum sexnotatum* | DD/LC | YES | 18 |
| *Lasioglossum soror* | EN | NO | 0 |
| *Lasioglossum subfasciatum* | EN | YES | 4 |
| *Lasioglossum xanthopus* | DD/LC | YES | 13 |
| *Macropis frivaldszkyi* | CR (PE) | NO | 0 |
| *Megachile anatolica* | DD/LC | NO | 0 |
| *Megachile burdigalensis* | DD/LC | NO | 0 |
| *Megachile diabolica* | CR (PE) | NO | 0 |
| *Megachile pugillatoria* | DD/LC | NO | 0 |
| *Melecta festiva* | DD/LC | NO | 0 |
| *Melitta tomentosa* | DD/LC | NO | 0 |
| *Melitta tricincta* | DD/LC | YES | 6 |
| *Melitturga clavicornis* | NT | YES | 4 |
| *Nomada alpigena* | DD/LC | NO | 0 |
| *Nomada argentata* | NT | YES | 4 |
| *Nomada armata* | DD/LC | YES | 7 |
| *Nomada braunsiana* | DD/LC | NO | 0 |
| *Nomada duplex* | DD/LC | NO | 0 |
| *Nomada gribodoi* | DD/LC | NO | 0 |
| *Nomada incisa* | DD/LC | NO | 0 |
| *Nomada insignipes* | DD/LC | NO | 0 |
| *Nomada italica* | CR | NO | 0 |
| *Nomada mutica* | DD/LC | YES | 2 |
| *Nomada panurgina* | DD/LC | NO | 0 |
| *Nomada pectoralis* | DD/LC | NO | 0 |
| *Nomada rhenana* | DD/LC | YES | 2 |
| *Nomada roberjeotiana* | VU | YES | 13 |
| *Nomada rufoabdominalis* | DD/LC | NO | 0 |
| *Nomada siciliensis* | CR (PE) | NO | 0 |
| *Nomada sicula* | DD/LC | NO | 0 |
| *Nomada symphyti* | DD/LC | YES | 1 |
| *Nomada unispinosa* | DD/LC | NO | 0 |
| *Nomada villosa* | DD/LC | YES | 2 |
| *Osmia picena* | DD/LC | NO | 0 |
| *Osmia subcornuta* | DD/LC | NO | 0 |
| *Osmia teunisseni* | DD/LC | NO | 0 |
| *Panurgus corsicus* | DD/LC | NO | 0 |
| *Pseudoanthidium eximium* | NT | NO | 0 |
| *Rhodanthidium acuminatum* | DD/LC | NO | 0 |
| *Rophites quinquespinosus* | DD/LC | NO | 0 |
| *Sphecodes cristatus* | DD/LC | YES | 1 |
| *Sphecodes croaticus* | DD/LC | YES | 7 |
| *Sphecodes hyalinatus* | DD/LC | YES | 16 |
| *Sphecodes majalis* | DD/LC | YES | 7 |
| *Sphecodes pinguiculus* | DD/LC | NO | 0 |
| *Sphecodes rubicundus* | DD/LC | YES | 2 |
| *Sphecodes schenckii* | DD/LC | YES | 8 |
| *Sphecodes spinulosus* | DD/LC | YES | 3 |
| *Stenoheriades maroccana* | DD/LC | NO | 0 |
| *Systropha curvicornis* | DD/LC | YES | 5 |
| *Tarsalia ancyliformis* | DD/LC | NO | 0 |
| *Trachusa interrupta* | DD/LC | NO | 0 |
| *Triepeolus tristis* | DD/LC | YES | 1 |
