# Supplementary material for: Filling knowledge gaps in insect conservation by leveraging genetic data from public archives
Source: Database (Oxford). 2024 Jan 29;2024:baae002. doi: 10.1093/database/baae002 (PMC10878047; doi:10.1093/database/baae002)
Supplement: baae002_Supp [file baae002_supp.zip › suppl_data/Table_S3_Lepidoptera.docx]

**Table S3***. Table showing the Lepidoptera species included in the IUCN Italian Red Lists, the conservation status, whether the species is present in our dataset, and how many individuals.*

| **Species name** | **IUCN status** | **Is the species in our dataset?** | **Number of individuals in the dataset** |
| --- | --- | --- | --- |
| *Aglais ichnusa* | LC | YES | 4 |
| *Aglais urticae* | LC | YES | 186 |
| *Agriades glandon* | LC | YES | 41 |
| *Albulina optilete* | LC | YES | 148 |
| *Albulina orbitulus* | LC | YES | 26 |
| *Anthocharis cardamines* | LC | YES | 160 |
| *Anthocharis damone* | LC | YES | 23 |
| *Anthocharis euphenoides* | NT | YES | 35 |
| *Apatura ilia* | LC | YES | 56 |
| *Apatura iris* | LC | YES | 46 |
| *Aphantopus hyperantus* | LC | YES | 129 |
| *Aporia crataegi* | LC | YES | 138 |
| *Araschnia levana* | EN | YES | 65 |
| *Arethusana arethusa* | LC | YES | 71 |
| *Argynnis adippe* | LC | YES | 167 |
| *Argynnis aglaja* | LC | YES | 111 |
| *Argynnis elisa* | NT | YES | 10 |
| *Argynnis niobe* | LC | YES | 122 |
| *Argynnis pandora* | LC | YES | 59 |
| *Argynnis paphia* | LC | YES | 144 |
| *Aricia agestis* | LC | YES | 420 |
| *Aricia allous* | LC | YES | 214 |
| *Aricia cramera* | LC | YES | 195 |
| *Aricia nicias* | LC | YES | 31 |
| *Azanus ubaldus* | DD | NO | 0 |
| *Boloria dia* | LC | YES | 78 |
| *Boloria euphrosyne* | LC | YES | 139 |
| *Boloria graeca* | NT | YES | 12 |
| *Boloria napaea* | LC | YES | 52 |
| *Boloria pales* | LC | YES | 135 |
| *Boloria selene* | LC | YES | 108 |
| *Boloria thore* | LC | YES | 46 |
| *Boloria titania* | LC | YES | 60 |
| *Brenthis daphne* | LC | YES | 98 |
| *Brenthis hecate* | LC | YES | 35 |
| *Brenthis ino* | LC | YES | 93 |
| *Callophrys avis* | VU | YES | 10 |
| *Callophrys rubi* | LC | YES | 243 |
| *Carcharodus alceae* | LC | YES | 178 |
| *Carcharodus baeticus* | NT | YES | 25 |
| *Carcharodus flocciferus* | LC | YES | 65 |
| *Carcharodus lavatherae* | LC | YES | 38 |
| *Carterocephalus palaemon* | LC | YES | 69 |
| *Celastrina argiolus* | LC | YES | 231 |
| *Charaxes jasius* | LC | YES | 48 |
| *Chazara briseis* | LC | YES | 97 |
| *Coenonympha arcania* | LC | YES | 70 |
| *Coenonympha corinna* | LC | YES | 54 |
| *Coenonympha darwiniana* | LC | YES | 3 |
| *Coenonympha dorus* | LC | YES | 36 |
| *Coenonympha elbana* | LC | NO | 0 |
| *Coenonympha gardetta* | LC | YES | 32 |
| *Coenonympha glycerion* | LC | YES | 76 |
| *Coenonympha lyllus* | LC | YES | 2 |
| *Coenonympha oedippus* | LC | YES | 29 |
| *Coenonympha pamphilus* | LC | YES | 288 |
| *Coenonympha rhodopensis* | LC | YES | 26 |
| *Colias alfacariensis* | LC | YES | 158 |
| *Colias crocea* | LC | YES | 35 |
| *Colias hyale* | LC | YES | 62 |
| *Colias palaeno* | LC | YES | 10 |
| *Colias phicomone* | LC | YES | 52 |
| *Cupido alcetas* | LC | YES | 67 |
| *Cupido argiades* | LC | YES | 20 |
| *Cupido minimus* | LC | YES | 187 |
| *Cupido osiris* | LC | YES | 62 |
| *Cyaniris semiargus* | LC | YES | 150 |
| *Danaus chrysippus* | LC | YES | 15 |
| *Erebia aethiopellus* | LC | YES | 9 |
| *Erebia aethiops* | LC | YES | 87 |
| *Erebia albergana* | LC | YES | 23 |
| *Erebia calcaria* | NT | YES | 5 |
| *Erebia cassioides* | LC | YES | 20 |
| *Erebia christi* | EN | YES | 9 |
| *Erebia dromus* | LC | NO | 0 |
| *Erebia epiphron* | LC | YES | 265 |
| *Erebia eriphyle* | LC | YES | 24 |
| *Erebia euryale* | LC | YES | 187 |
| *Erebia flavofasciata* | VU | YES | 13 |
| *Erebia gorge* | LC | YES | 71 |
| *Erebia ligea* | LC | YES | 97 |
| *Erebia manto* | LC | YES | 222 |
| *Erebia medusa* | LC | YES | 100 |
| *Erebia melampus* | LC | YES | 45 |
| *Erebia meolans* | LC | YES | 66 |
| *Erebia mnestra* | LC | YES | 27 |
| *Erebia montana* | LC | YES | 40 |
| *Erebia neoridas* | LC | YES | 43 |
| *Erebia nivalis* | NT | NO | 0 |
| *Erebia oeme* | LC | YES | 67 |
| *Erebia ottomana* | NT | YES | 36 |
| *Erebia pandrose* | LC | YES | 69 |
| *Erebia pharte* | LC | YES | 37 |
| *Erebia pluto* | LC | YES | 42 |
| *Erebia pronoe* | LC | YES | 86 |
| *Erebia scipio* | LC | YES | 10 |
| *Erebia stiria* | LC | YES | 11 |
| *Erebia styx* | LC | YES | 27 |
| *Erebia triaria* | LC | YES | 4 |
| *Erebia tyndarus* | LC | YES | 8 |
| *Erynnis tages* | LC | YES | 118 |
| *Euchloe ausonia* | LC | YES | 61 |
| *Euchloe crameri* | LC | YES | 44 |
| *Euchloe insularis* | LC | YES | 12 |
| *Euchloe simplonia* | LC | YES | 12 |
| *Euchloe tagis* | NT | YES | 3 |
| *Eumedonia eumedon* | LC | YES | 158 |
| *Euphydryas aurinia* | VU | YES | 216 |
| *Euphydryas cynthia* | LC | YES | 31 |
| *Euphydryas glaciegenita* | LC | NO | 0 |
| *Euphydryas intermedia* | LC | YES | 5 |
| *Euphydryas maturna* | CR | YES | 28 |
| *Euphydryas provincialis* | LC | YES | 3 |
| *Favonius quercus* | LC | YES | 95 |
| *Gegenes nostrodamus* | LC | YES | 35 |
| *Gegenes pumilio* | LC | YES | 54 |
| *Glaucopsyche alexis* | LC | YES | 204 |
| *Glaucopsyche melanops* | LC | YES | 29 |
| *Gonepteryx cleopatra* | LC | YES | 118 |
| *Gonepteryx rhamni* | LC | YES | 168 |
| *Hamearis lucina* | LC | YES | 53 |
| *Hesperia comma* | LC | YES | 109 |
| *Heteropterus morpheus* | LC | YES | 43 |
| *Hipparchia aristaeus* | LC | YES | 23 |
| *Hipparchia blachieri* | LC | YES | 1 |
| *Hipparchia fagi* | LC | YES | 76 |
| *Hipparchia fidia* | NT | YES | 29 |
| *Hipparchia hermione* | LC | YES | 45 |
| *Hipparchia leighebi* | LC | YES | 13 |
| *Hipparchia neapolitana* | LC | YES | 6 |
| *Hipparchia neomiris* | LC | YES | 13 |
| *Hipparchia sbordonii* | EN | YES | 3 |
| *Hipparchia semele* | LC | YES | 124 |
| *Hipparchia statilinus* | LC | YES | 88 |
| *Hyponephele lupina* | LC | YES | 44 |
| *Hyponephele lycaon* | LC | YES | 84 |
| *Inachis io* | LC | NO | 0 |
| *Iolana iolas* | NT | YES | 30 |
| *Iphiclides podalirius* | LC | YES | 143 |
| *Issoria lathonia* | LC | YES | 144 |
| *Kanetisa circe* | LC | YES | 107 |
| *Lampides boeticus* | LC | YES | 166 |
| *Lasiommata achine* | NT | YES | 42 |
| *Lasiommata maera* | LC | YES | 183 |
| *Lasiommata megera* | LC | YES | 378 |
| *Lasiommata paramegaera* | LC | YES | 53 |
| *Lasiommata petropolitana* | LC | YES | 59 |
| *Leptidea juvernica* | LC | YES | 203 |
| *Leptidea reali* | LC | YES | 114 |
| *Leptidea sinapis* | LC | YES | 592 |
| *Leptotes pirithous* | LC | YES | 116 |
| *Libythea celtis* | LC | YES | 61 |
| *Limenitis camilla* | LC | YES | 64 |
| *Limenitis populi* | LC | YES | 22 |
| *Limenitis reducta* | LC | YES | 96 |
| *Lycaeides abetonicus* | LC | NO | 0 |
| *Lycaeides argyrognomon* | LC | YES | 1 |
| *Lycaeides corsicus* | LC | NO | 0 |
| *Lycaeides idas* | LC | YES | 19 |
| *Lycaena alciphron* | LC | YES | 92 |
| *Lycaena dispar* | LC | YES | 43 |
| *Lycaena eurydame* | LC | NO | 0 |
| *Lycaena helle* | RE | YES | 39 |
| *Lycaena hippothoe* | LC | YES | 99 |
| *Lycaena italica* | LC | NO | 0 |
| *Lycaena phlaeas* | LC | YES | 233 |
| *Lycaena subalpina* | LC | NO | 0 |
| *Lycaena thersamon* | LC | YES | 23 |
| *Lycaena tityrus* | LC | YES | 193 |
| *Lycaena virgaureae* | LC | YES | 99 |
| *Maculinea alcon* | VU | YES | 78 |
| *Maculinea arion* | LC | YES | 89 |
| *Maculinea rebeli* | LC | NO | 0 |
| *Maculinea teleius* | VU | YES | 30 |
| *Maniola jurtina* | LC | YES | 598 |
| *Maniola nurag* | LC | YES | 24 |
| *Melanargia arge* | LC | YES | 16 |
| *Melanargia galathea* | LC | YES | 195 |
| *Melanargia occitanica* | LC | YES | 23 |
| *Melanargia pherusa* | LC | YES | 3 |
| *Melanargia russiae* | LC | YES | 63 |
| *Melitaea aetherie* | VU | YES | 13 |
| *Melitaea asteria* | LC | YES | 11 |
| *Melitaea aurelia* | LC | YES | 102 |
| *Melitaea britomartis* | EN | YES | 49 |
| *Melitaea cinxia* | LC | YES | 131 |
| *Melitaea deione* | LC | YES | 36 |
| *Melitaea diamina* | LC | YES | 94 |
| *Melitaea didyma* | LC | YES | 250 |
| *Melitaea nevadensis* | LC | YES | 98 |
| *Melitaea ornata* | LC | YES | 34 |
| *Melitaea parthenoides* | LC | YES | 53 |
| *Melitaea phoebe* | LC | YES | 100 |
| *Melitaea trivia* | LC | YES | 107 |
| *Melitaea varia* | LC | YES | 38 |
| *Minois dryas* | LC | YES | 66 |
| *Neptis rivularis* | LC | YES | 23 |
| *Neptis sappho* | LC | YES | 19 |
| *Nymphalis antiopa* | LC | YES | 49 |
| *Nymphalis polychloros* | LC | YES | 85 |
| *Ochlodes sylvanus* | LC | YES | 181 |
| *Oeneis glacialis* | LC | YES | 26 |
| *Papilio alexanor* | EN | YES | 17 |
| *Papilio hospiton* | LC | YES | 12 |
| *Papilio machaon* | LC | YES | 191 |
| *Pararge aegeria* | LC | YES | 324 |
| *Parnassius apollo* | LC | YES | 95 |
| *Parnassius mnemosyne* | LC | YES | 120 |
| *Parnassius sacerdos* | LC | NO | 0 |
| *Pieris brassicae* | LC | YES | 233 |
| *Pieris bryoniae* | LC | YES | 19 |
| *Pieris callidice* | LC | YES | 31 |
| *Pieris daplidice* | LC | YES | 80 |
| *Pieris edusa* | LC | YES | 141 |
| *Pieris ergane* | LC | YES | 34 |
| *Pieris mannii* | LC | YES | 131 |
| *Pieris napi* | LC | YES | 318 |
| *Pieris rapae* | LC | YES | 565 |
| *Plebejus argus* | LC | YES | 308 |
| *Plebejus trappi* | VU | NO | 0 |
| *Polygonia c-album* | LC | YES | 20 |
| *Polygonia egea* | LC | YES | 24 |
| *Polyommatus bellargus* | LC | YES | 82 |
| *Polyommatus celinus* | LC | NO | 0 |
| *Polyommatus coridon* | LC | YES | 54 |
| *Polyommatus damon* | LC | YES | 79 |
| *Polyommatus daphnis* | LC | YES | 85 |
| *Polyommatus dolus* | NT | YES | 19 |
| *Polyommatus dorylas* | LC | YES | 102 |
| *Polyommatus eros* | LC | YES | 68 |
| *Polyommatus escheri* | LC | YES | 66 |
| *Polyommatus exuberans* | EN | NO | 0 |
| *Polyommatus galloi* | VU | NO | 0 |
| *Polyommatus gennargenti* | EN | NO | 0 |
| *Polyommatus hispanus* | LC | YES | 4 |
| *Polyommatus humedasae* | EN | YES | 8 |
| *Polyommatus icarius* | LC | NO | 0 |
| *Polyommatus icarus* | LC | YES | 762 |
| *Polyommatus ripartii* | NT | YES | 136 |
| *Polyommatus thersites* | LC | YES | 94 |
| *Polyommatus virgilius* | LC | NO | 0 |
| *Pseudophilotes barbagiae* | DD | YES | 13 |
| *Pseudophilotes baton* | LC | YES | 16 |
| *Pseudophilotes vicrama* | LC | YES | 5 |
| *Pyrgus accretus* | LC | NO | 0 |
| *Pyrgus alveus* | LC | YES | 125 |
| *Pyrgus andromedae* | LC | YES | 60 |
| *Pyrgus armoricanus* | LC | YES | 181 |
| *Pyrgus cacaliae* | LC | YES | 65 |
| *Pyrgus carlinae* | LC | YES | 21 |
| *Pyrgus carthami* | LC | YES | 65 |
| *Pyrgus centralitaliae* | LC | NO | 0 |
| *Pyrgus foulquieri* | LC | YES | 28 |
| *Pyrgus malvae* | LC | YES | 73 |
| *Pyrgus malvoides* | LC | YES | 124 |
| *Pyrgus onopordi* | LC | YES | 47 |
| *Pyrgus picenus* | LC | NO | 0 |
| *Pyrgus serratulae* | LC | YES | 83 |
| *Pyrgus sidae* | LC | YES | 65 |
| *Pyrgus warrenensis* | LC | YES | 6 |
| *Pyronia cecilia* | LC | YES | 157 |
| *Pyronia tithonus* | LC | YES | 110 |
| *Satyrium acaciae* | LC | YES | 54 |
| *Satyrium esculi* | LC | YES | 41 |
| *Satyrium ilicis* | LC | YES | 86 |
| *Satyrium pruni* | NT | YES | 45 |
| *Satyrium spini* | LC | YES | 95 |
| *Satyrium w-album* | LC | YES | 51 |
| *Satyrus actaea* | NT | YES | 27 |
| *Satyrus ferula* | LC | YES | 72 |
| *Scolitantides orion* | LC | YES | 42 |
| *Sloperia proto* | LC | YES | 68 |
| *Spialia orbifera* | LC | NO | 0 |
| *Spialia sertorius* | LC | YES | 202 |
| *Spialia therapne* | NT | YES | 13 |
| *Thecla betulae* | LC | YES | 63 |
| *Thymelicus acteon* | LC | YES | 157 |
| *Thymelicus lineola* | LC | YES | 196 |
| *Thymelicus sylvestris* | LC | YES | 241 |
| *Vanessa atalanta* | LC | YES | 168 |
| *Vanessa cardui* | LC | YES | 198 |
| *Zerynthia cassandra* | LC | YES | 70 |
| *Zerynthia polyxena* | LC | YES | 36 |
| *Zizeeria karsandra* | VU | YES | 1 |
