# Supplementary material for: Filling knowledge gaps in insect conservation by leveraging genetic data from public archives
Source: Database (Oxford). 2024 Jan 29;2024:baae002. doi: 10.1093/database/baae002 (PMC10878047; doi:10.1093/database/baae002)
Supplement: baae002_Supp [file baae002_supp.zip › suppl_data/Table_S4_Coleoptera.docx]

**Table S4***. Table showing the species of Coleoptera included in the IUCN Italian Red Lists, the conservation status, whether the species is present in our dataset, and how many individuals.*

| **Species name** | **IUCN status** | **Is the species in our dataset?** | **Number of individuals in the dataset** |
| --- | --- | --- | --- |
| *Abdera affinis* | NT | YES | 8 |
| *Abdera bifasciata* | NT | YES | 1 |
| *Abdera biflexuosa* | NT | NO | 0 |
| *Abdera flexuosa* | NT | YES | 9 |
| *Abdera quadrifasciata* | NT | YES | 5 |
| *Abraeus globosus* | LC | NO | 0 |
| *Abraeus granulum* | LC | YES | 3 |
| *Abraeus parvulus* | CR | YES | 1 |
| *Abraeus perpusillus* | LC | YES | 8 |
| *Acalles aubei* | LC | YES | 16 |
| *Acalles camelus* | LC | NO | 0 |
| *Acalles commutatus* | VU | YES | 4 |
| *Acalles dieckmanni* | NT | NO | 0 |
| *Acalles dubius* | VU | NO | 0 |
| *Acalles echinatus* | LC | NO | 0 |
| *Acalles humerosus* | VU | YES | 3 |
| *Acalles kippenbergi* | VU | NO | 0 |
| *Acalles lemur* | LC | NO | 0 |
| *Acalles longus* | NT | YES | 1 |
| *Acalles micros* | LC | NO | 0 |
| *Acalles papei* | LC | NO | 0 |
| *Acalles parvulus* | LC | NO | 0 |
| *Acalles pulchellus* | VU | NO | 0 |
| *Acalles sardiniaensis* | NT | NO | 0 |
| *Acalles setulipennis* | NT | NO | 0 |
| *Acalles temperei* | NT | NO | 0 |
| *Acalles tibialis* | NT | NO | 0 |
| *Acallocrates denticollis* | LC | YES | 7 |
| *Acallocrates minutesquamosus* | LC | NO | 0 |
| *Acallorneuma doderoi* | VU | YES | 1 |
| *Acallorneuma ingoi* | NT | YES | 4 |
| *Acallorneuma mainardii* | NT | YES | 1 |
| *Acallorneuma montisalbi* | VU | YES | 3 |
| *Acallorneuma reitteri* | NT | YES | 3 |
| *Acallorneuma sardeanense* | VU | NO | 0 |
| *Acanthocinus aedilis* | LC | YES | 13 |
| *Acanthocinus henschi* | EN | NO | 0 |
| *Acanthocinus reticulatus* | LC | YES | 1 |
| *Acanthocinus xanthoneurus* | NT | NO | 0 |
| *Accanthopus velikensis* | LC | YES | 1 |
| *Aclemmysa solarii* | NT | NO | 0 |
| *Acmaeodera bipunctata* | LC | NO | 0 |
| *Acmaeodera crinita* | EN | NO | 0 |
| *Acmaeodera cylindrica* | LC | NO | 0 |
| *Acmaeodera degener* | NT | NO | 0 |
| *Acmaeodera pilosellae* | LC | YES | 1 |
| *Acmaeodera prunneri* | LC | NO | 0 |
| *Acmaeodera quadrifasciata* | LC | NO | 0 |
| *Acmaeodera revelierei* | CR | NO | 0 |
| *Acmaeodera tassii* | VU | NO | 0 |
| *Acmaeoderella adspersula* | LC | NO | 0 |
| *Acmaeoderella flavofasciata* | LC | NO | 0 |
| *Acmaeops marginatus* | NT | YES | 4 |
| *Acmaeops pratensis* | LC | YES | 4 |
| *Acmaeops septentrionis* | NT | YES | 8 |
| *Acritus homoeopathicus* | NT | YES | 3 |
| *Acrulia inflata* | NT | YES | 8 |
| *Actidium aterrimum* | DD | NO | 0 |
| *Actidium boudieri* | DD | YES | 2 |
| *Actidium coarctatum* | DD | NO | 0 |
| *Actidium kraatzi* | DD | NO | 0 |
| *Actidium reitteri* | DD | NO | 0 |
| *Aderus populneus* | LC | YES | 22 |
| *Adistemia watsoni* | LC | YES | 2 |
| *Aegomorphus clavipes* | LC | YES | 1 |
| *Aegosoma scabricornis* | LC | NO | 0 |
| *Aeletes atomarius* | LC | YES | 4 |
| *Aesalus scarabaeoides* | EN | YES | 7 |
| *Aethiessa squamosa* | NT | NO | 0 |
| *Agathidium aglyptoides* | DD | NO | 0 |
| *Agathidium arcticum* | NT | YES | 2 |
| *Agathidium atrum* | LC | YES | 19 |
| *Agathidium badium* | LC | YES | 10 |
| *Agathidium banaticum* | DD | NO | 0 |
| *Agathidium bartolii* | NT | NO | 0 |
| *Agathidium bohemicum* | LC | NO | 0 |
| *Agathidium brisouti* | DD | NO | 0 |
| *Agathidium confusum* | LC | YES | 1 |
| *Agathidium convexum* | DD | NO | 0 |
| *Agathidium dentatum* | LC | NO | 0 |
| *Agathidium discoideum* | NT | NO | 0 |
| *Agathidium haemorrhoum* | LC | YES | 2 |
| *Agathidium italicum* | LC | NO | 0 |
| *Agathidium laevigatulum* | LC | NO | 0 |
| *Agathidium laevigatum* | LC | YES | 14 |
| *Agathidium mandibulare* | LC | NO | 0 |
| *Agathidium marginatum* | LC | YES | 4 |
| *Agathidium minimum* | NT | NO | 0 |
| *Agathidium montemurroi* | VU | NO | 0 |
| *Agathidium nigriceps* | NT | NO | 0 |
| *Agathidium nigrinum* | LC | YES | 6 |
| *Agathidium nigripenne* | LC | YES | 12 |
| *Agathidium nudum* | DD | NO | 0 |
| *Agathidium obenbergeri* | LC | NO | 0 |
| *Agathidium paganettianum* | LC | NO | 0 |
| *Agathidium pisanum* | LC | YES | 2 |
| *Agathidium plagiatum* | LC | NO | 0 |
| *Agathidium pseudopallidum* | NT | NO | 0 |
| *Agathidium rotundatum* | LC | YES | 12 |
| *Agathidium seminulum* | LC | YES | 16 |
| *Agathidium varians* | LC | YES | 13 |
| *Aglenus brunneus* | LC | NO | 0 |
| *Agnathus decoratus* | EN | NO | 0 |
| *Agrilus albomarginatus* | LC | NO | 0 |
| *Agrilus angustulus* | LC | YES | 12 |
| *Agrilus antiquus* | LC | NO | 0 |
| *Agrilus ater* | LC | YES | 3 |
| *Agrilus auricollis* | LC | NO | 0 |
| *Agrilus betuleti* | EN | YES | 3 |
| *Agrilus biguttatus* | LC | YES | 11 |
| *Agrilus convexicollis* | LC | YES | 8 |
| *Agrilus croaticus* | LC | NO | 0 |
| *Agrilus curtulus* | EN | YES | 1 |
| *Agrilus cyanescens* | LC | YES | 7 |
| *Agrilus cytisi* | VU | NO | 0 |
| *Agrilus derasofasciatus* | LC | YES | 5 |
| *Agrilus elegans* | LC | NO | 0 |
| *Agrilus graecus* | DD | YES | 1 |
| *Agrilus graminis* | LC | YES | 13 |
| *Agrilus grandiceps* | EN | NO | 0 |
| *Agrilus guerini* | LC | NO | 0 |
| *Agrilus hastulifer* | LC | YES | 1 |
| *Agrilus laticornis* | LC | YES | 8 |
| *Agrilus lineola* | LC | NO | 0 |
| *Agrilus litura* | EN | NO | 0 |
| *Agrilus marozzinii* | LC | NO | 0 |
| *Agrilus meloni* | CR | NO | 0 |
| *Agrilus obscuricollis* | LC | YES | 3 |
| *Agrilus olivicolor* | LC | YES | 2 |
| *Agrilus pratensis* | LC | YES | 3 |
| *Agrilus pseudocyaneus* | VU | NO | 0 |
| *Agrilus relegatus* | LC | NO | 0 |
| *Agrilus roscidus* | LC | YES | 4 |
| *Agrilus salicis* | CR | YES | 1 |
| *Agrilus sinuatus* | LC | NO | 0 |
| *Agrilus subauratus* | CR | YES | 1 |
| *Agrilus sulcicollis* | EN | YES | 18 |
| *Agrilus suvorovi* | LC | YES | 1 |
| *Agrilus viridis* | EN | YES | 53 |
| *Airaphilus nasutus* | NT | NO | 0 |
| *Allandrus undulatus* | LC | YES | 2 |
| *Allardius oculatus* | VU | NO | 0 |
| *Allardius sardiniensis* | NT | NO | 0 |
| *Allecula aterrima* | VU | NO | 0 |
| *Allecula morio* | LC | YES | 29 |
| *Allecula rhenana* | VU | YES | 1 |
| *Allecula suberina* | EN | YES | 1 |
| *Allonyx quadrimaculatus* | DD | NO | 0 |
| *Alosterna tabacicolor* | LC | YES | 51 |
| *Amauronyx maerkeli* | NT | NO | 0 |
| *Amaurorhinus bewickianus* | LC | NO | 0 |
| *Amaurorhinus caoduroi* | LC | NO | 0 |
| *Amaurorhinus cesaraccioi* | DD | NO | 0 |
| *Amaurorhinus lostiae* | DD | NO | 0 |
| *Amaurorhinus mediterraneus* | DD | NO | 0 |
| *Amaurorhinus sardous* | DD | NO | 0 |
| *Amorphocephala coronata* | LC | NO | 0 |
| *Ampedus auripes* | EN | YES | 2 |
| *Ampedus balteatus* | NT | YES | 50 |
| *Ampedus brunnicornis* | EN | YES | 7 |
| *Ampedus callegarii* | CR | NO | 0 |
| *Ampedus cardinalis* | LC | YES | 1 |
| *Ampedus cinnaberinus* | LC | YES | 7 |
| *Ampedus coenobita* | NT | NO | 0 |
| *Ampedus elegantulus* | VU | YES | 3 |
| *Ampedus erythrogonus* | NT | YES | 12 |
| *Ampedus forticornis* | VU | NO | 0 |
| *Ampedus glycereus* | LC | YES | 33 |
| *Ampedus magistrettii* | EN | NO | 0 |
| *Ampedus melanurus* | VU | YES | 2 |
| *Ampedus melonii* | VU | NO | 0 |
| *Ampedus nemoralis* | VU | YES | 12 |
| *Ampedus nigerrimus* | LC | YES | 14 |
| *Ampedus nigrinus* | NT | YES | 16 |
| *Ampedus nigroflavus* | EN | YES | 9 |
| *Ampedus pomonae* | NT | YES | 20 |
| *Ampedus pomorum* | LC | YES | 76 |
| *Ampedus praeustus* | NT | YES | 2 |
| *Ampedus quadrisignatus* | CR | NO | 0 |
| *Ampedus quercicola* | LC | YES | 39 |
| *Ampedus robustus* | EN | NO | 0 |
| *Ampedus rufipennis* | VU | YES | 17 |
| *Ampedus sanguineus* | LC | YES | 38 |
| *Ampedus sanguinolentus* | LC | YES | 25 |
| *Ampedus scrofa* | LC | YES | 19 |
| *Ampedus sinuatus* | VU | YES | 7 |
| *Ampedus triangulum* | EN | NO | 0 |
| *Ampedus tristis* | EN | YES | 5 |
| *Amphicerus bimaculatus* | LC | NO | 0 |
| *Amphicyllis globiformis* | LC | YES | 3 |
| *Amphicyllis globus* | LC | YES | 14 |
| *Amphotis marginata* | LC | YES | 16 |
| *Amphotis orientalis* | LC | NO | 0 |
| *Anaesthetis testacea* | LC | YES | 10 |
| *Anaglyptus gibbosus* | LC | YES | 1 |
| *Anaglyptus mysticus* | LC | YES | 16 |
| *Anaglyptus zappii* | VU | NO | 0 |
| *Anaspis costai* | VU | YES | 14 |
| *Anaspis flava* | LC | YES | 33 |
| *Anaspis frontalis* | LC | YES | 59 |
| *Anaspis lurida* | LC | YES | 9 |
| *Anaspis pulicaria* | LC | YES | 24 |
| *Anaspis ruficollis* | EN | YES | 13 |
| *Anaspis rufilabris* | EN | YES | 36 |
| *Anastrangalia dubia* | LC | YES | 15 |
| *Anastrangalia reyi* | LC | YES | 2 |
| *Anastrangalia sanguinolenta* | LC | YES | 39 |
| *Anelastes barbarus* | DD | NO | 0 |
| *Anidorus lateralis* | VU | NO | 0 |
| *Anidorus nigrinus* | LC | YES | 16 |
| *Anidorus sanguinolentus* | LC | NO | 0 |
| *Anisandrus dispar* | LC | YES | 25 |
| *Anisarthron barbipes* | NT | NO | 0 |
| *Anisorus quercus* | NT | YES | 2 |
| *Anisotoma axillaris* | VU | YES | 5 |
| *Anisotoma castanea* | LC | YES | 12 |
| *Anisotoma glabra* | VU | YES | 6 |
| *Anisotoma humeralis* | LC | YES | 22 |
| *Anisotoma orbicularis* | LC | YES | 12 |
| *Anisoxya fuscula* | NT | YES | 9 |
| *Anobium hederae* | LC | YES | 1 |
| *Anobium inexpectatum* | NT | NO | 0 |
| *Anobium punctatum* | LC | YES | 21 |
| *Anogcodes ferrugineus* | DD | NO | 0 |
| *Anogcodes fulvicollis* | LC | YES | 1 |
| *Anogcodes rufiventris* | LC | YES | 13 |
| *Anogcodes seladonius* | LC | YES | 5 |
| *Anogcodes ustulatus* | LC | YES | 3 |
| *Anomognathus cuspidatus* | LC | YES | 10 |
| *Anomognathus tricuspis* | DD | NO | 0 |
| *Anoplodera rufipes* | LC | YES | 4 |
| *Anoplodera sexguttata* | LC | YES | 16 |
| *Anthaxia aprutiana* | LC | NO | 0 |
| *Anthaxia candens* | LC | YES | 8 |
| *Anthaxia chevrieri* | LC | NO | 0 |
| *Anthaxia cichorii* | LC | NO | 0 |
| *Anthaxia confusa* | LC | YES | 1 |
| *Anthaxia corsica* | CR | NO | 0 |
| *Anthaxia dimidiata* | LC | NO | 0 |
| *Anthaxia flaviae* | LC | NO | 0 |
| *Anthaxia fulgurans* | LC | YES | 6 |
| *Anthaxia giorgioi* | CR | NO | 0 |
| *Anthaxia godeti* | LC | YES | 5 |
| *Anthaxia hackeri* | LC | NO | 0 |
| *Anthaxia helvetica* | LC | YES | 23 |
| *Anthaxia hungarica* | LC | YES | 1 |
| *Anthaxia istriana* | LC | YES | 7 |
| *Anthaxia kochi* | VU | NO | 0 |
| *Anthaxia liae* | LC | NO | 0 |
| *Anthaxia lucens* | LC | NO | 0 |
| *Anthaxia manca* | LC | YES | 2 |
| *Anthaxia mendizabali* | LC | YES | 4 |
| *Anthaxia midas* | EN | NO | 0 |
| *Anthaxia millefolii* | LC | YES | 1 |
| *Anthaxia morio* | LC | YES | 7 |
| *Anthaxia nereis* | EN | NO | 0 |
| *Anthaxia nigritula* | LC | NO | 0 |
| *Anthaxia nigrojubata* | EN | NO | 0 |
| *Anthaxia nitidula* | LC | YES | 28 |
| *Anthaxia passerinii* | VU | NO | 0 |
| *Anthaxia podolica* | LC | YES | 5 |
| *Anthaxia praeclara* | VU | NO | 0 |
| *Anthaxia quadripunctata* | LC | YES | 32 |
| *Anthaxia rugicollis* | VU | NO | 0 |
| *Anthaxia salicis* | LC | YES | 1 |
| *Anthaxia scutellaris* | LC | NO | 0 |
| *Anthaxia semicuprea* | LC | YES | 2 |
| *Anthaxia senicula* | LC | NO | 0 |
| *Anthaxia sepulchralis* | LC | YES | 1 |
| *Anthaxia spinolae* | LC | YES | 1 |
| *Anthaxia suzannae* | LC | NO | 0 |
| *Anthaxia thalassophila* | LC | NO | 0 |
| *Anthaxia umbellatarum* | LC | YES | 2 |
| *Anthribus fasciatus* | LC | YES | 1 |
| *Anthribus nebulosus* | LC | YES | 22 |
| *Anthribus scapularis* | DD | NO | 0 |
| *Apate monachus* | NT | NO | 0 |
| *Aphanommata filum* | NT | NO | 0 |
| *Aplocnemus acutangulus* | LC | NO | 0 |
| *Aplocnemus alpestris* | LC | YES | 2 |
| *Aplocnemus angelinii* | LC | NO | 0 |
| *Aplocnemus corcyricus* | LC | NO | 0 |
| *Aplocnemus crenicollis* | LC | NO | 0 |
| *Aplocnemus cribricollis* | LC | NO | 0 |
| *Aplocnemus difficilis* | LC | NO | 0 |
| *Aplocnemus duplicatus* | LC | NO | 0 |
| *Aplocnemus etruscus* | NT | NO | 0 |
| *Aplocnemus impressus* | LC | YES | 4 |
| *Aplocnemus integer* | LC | NO | 0 |
| *Aplocnemus januaventi* | LC | NO | 0 |
| *Aplocnemus jejunus* | LC | NO | 0 |
| *Aplocnemus koziorowiczi* | LC | NO | 0 |
| *Aplocnemus marginatus* | LC | NO | 0 |
| *Aplocnemus nigricornis* | LC | YES | 10 |
| *Aplocnemus panalpinus* | LC | NO | 0 |
| *Aplocnemus pectinatus* | LC | NO | 0 |
| *Aplocnemus quercicola* | VU | NO | 0 |
| *Aplocnemus rufomarginatus* | LC | NO | 0 |
| *Aplocnemus tarsalis* | LC | YES | 5 |
| *Aplocnemus trinacriensis* | LC | NO | 0 |
| *Aplocnemus virens* | LC | YES | 16 |
| *Araecerodes grenieri* | LC | NO | 0 |
| *Arhopalus ferus* | LC | YES | 1 |
| *Arhopalus rusticus* | LC | YES | 28 |
| *Arhopalus syriacus* | LC | NO | 0 |
| *Aromia moschata* | LC | YES | 17 |
| *Arthrolips nana* | DD | NO | 0 |
| *Arthrolips obscura* | DD | NO | 0 |
| *Asemum striatum* | XY | NO | 0 |
| *Asemum tenuicorne* | NT | NO | 0 |
| *Aspidiphorus lareyiniei* | NT | NO | 0 |
| *Aspidiphorus orbiculatus* | LC | YES | 25 |
| *Atheta liturata* | LC | YES | 2 |
| *Atheta pallidicornis* | LC | YES | 16 |
| *Atheta picipes* | LC | YES | 16 |
| *Atholus debeauxi* | CR | NO | 0 |
| *Atomaria linearis* | DD | YES | 18 |
| *Atomaria nigrirostris* | LC | YES | 17 |
| *Atomaria umbrina* | LC | YES | 2 |
| *Atomaria vespertina* | DD | YES | 3 |
| *Atrecus affinis* | LC | YES | 20 |
| *Atrecus ardeanus* | EN | NO | 0 |
| *Atrecus longiceps* | LC | YES | 4 |
| *Atrecus pilicornis* | VU | YES | 7 |
| *Attalus analis* | LC | YES | 3 |
| *Aulacochilus violaceus* | VU | NO | 0 |
| *Aulonium ruficorne* | LC | NO | 0 |
| *Aulonium trisulcum* | NT | YES | 7 |
| *Aulonothroscus brevicollis* | DD | YES | 34 |
| *Axinopalpis gracilis* | NT | YES | 3 |
| *Bacanius consobrinus* | CR | NO | 0 |
| *Bacanius solarii* | CR | NO | 0 |
| *Baeocera nobilis* | VU | NO | 0 |
| *Baeocera schirmeri* | VU | NO | 0 |
| *Batrisodes adnexus* | LC | NO | 0 |
| *Batrisodes buqueti* | NT | NO | 0 |
| *Batrisodes delaportei* | NT | NO | 0 |
| *Batrisodes hubenthali* | VU | NO | 0 |
| *Batrisodes oculatus* | LC | YES | 1 |
| *Batrisodes venustus* | LC | NO | 0 |
| *Batrisus formicarius* | VU | YES | 5 |
| *Berginus tamarisci* | LC | YES | 6 |
| *Bibloporus bicolor* | NT | YES | 21 |
| *Bibloporus mayeti* | NT | NO | 0 |
| *Bibloporus minutus* | NT | YES | 4 |
| *Bibloporus ultimus* | NT | YES | 2 |
| *Biphyllus frater* | LC | NO | 0 |
| *Biphyllus lunatus* | LC | YES | 2 |
| *Bitoma crenata* | LC | YES | 23 |
| *Bolitochara humeralis* | NT | NO | 0 |
| *Bolitochara lucida* | CR | YES | 22 |
| *Bolitochara mulsanti* | LC | YES | 13 |
| *Bolitochara obliqua* | LC | YES | 25 |
| *Bolitochara tecta* | LC | NO | 0 |
| *Bolitochara varia* | NT | NO | 0 |
| *Bolitophagus interruptus* | EN | NO | 0 |
| *Bolitophagus reticulatus* | VU | YES | 33 |
| *Bostrichus capucinus* | LC | YES | 3 |
| *Bothrideres bipunctatus* | NT | NO | 0 |
| *Brachygonus campadellii* | CR | YES | 1 |
| *Brachygonus megerlei* | VU | YES | 9 |
| *Brachygonus ruficeps* | EN | YES | 5 |
| *Brachypteroma ottomanum* | LC | NO | 0 |
| *Brachytemnus porcatus* | LC | NO | 0 |
| *Bryaxis curtisi* | LC | NO | 0 |
| *Bryaxis puncticollis* | LC | NO | 0 |
| *Buprestis aetnensis* | CR | NO | 0 |
| *Buprestis cupressi* | LC | NO | 0 |
| *Buprestis douei* | CR | NO | 0 |
| *Buprestis haemorrhoidalis* | LC | YES | 4 |
| *Buprestis humeralis* | EN | NO | 0 |
| *Buprestis novemmaculata* | LC | YES | 3 |
| *Buprestis octoguttata* | LC | YES | 2 |
| *Buprestis splendens* | EN | NO | 0 |
| *Bythinus burrelli* | LC | NO | 0 |
| *Cacotemnus rufipes* | NT | YES | 4 |
| *Cacotemnus thomsoni* | EN | NO | 0 |
| *Caenocara affine* | LC | NO | 0 |
| *Caenocara bovistae* | VU | YES | 3 |
| *Caenocara subglobosum* | LC | NO | 0 |
| *Caenoscelis angelinii* | CR | NO | 0 |
| *Caenoscelis ferruginea* | DD | NO | 0 |
| *Caenoscelis subdeplanata* | DD | YES | 1 |
| *Calambus bipustulatus* | VU | YES | 8 |
| *Calicnemis latreillii* | VU | YES | 8 |
| *Calicnemis sardiniensis* | EN | YES | 3 |
| *Calitys scabra* | VU | YES | 3 |
| *Callidium aeneum* | LC | YES | 8 |
| *Callidium coriaceum* | NT | YES | 12 |
| *Callidium violaceum* | LC | YES | 23 |
| *Callimus abdominalis* | LC | NO | 0 |
| *Callimus angulatus* | LC | NO | 0 |
| *Calopus serraticornis* | DD | YES | 4 |
| *Calyptomerus alpestris* | DD | YES | 6 |
| *Calyptomerus dubius* | LC | YES | 6 |
| *Camptorhinus simplex* | LC | NO | 0 |
| *Camptorhinus statua* | LC | NO | 0 |
| *Capnodis cariosa* | LC | NO | 0 |
| *Capnodis miliaris* | VU | NO | 0 |
| *Capnodis tenebrionis* | LC | NO | 0 |
| *Cardiophorus aetnensis* | DD | NO | 0 |
| *Cardiophorus albofasciatus* | CR | NO | 0 |
| *Cardiophorus anticus* | NT | YES | 2 |
| *Cardiophorus gramineus* | NT | YES | 5 |
| *Carphoborus minimus* | VU | NO | 0 |
| *Carphoborus perrisi* | LC | NO | 0 |
| *Carphoborus pini* | LC | NO | 0 |
| *Carpophilus bipustulatus* | DD | NO | 0 |
| *Carpophilus sexpustulatus* | NT | YES | 5 |
| *Cartodere constricta* | LC | YES | 9 |
| *Cartodere nodifer* | LC | YES | 54 |
| *Caryoscapha limbata* | VU | NO | 0 |
| *Cerambyx cerdo* | LC | YES | 8 |
| *Cerambyx miles* | LC | YES | 2 |
| *Cerambyx nodulosus* | LC | NO | 0 |
| *Cerambyx scopolii* | LC | YES | 22 |
| *Cerambyx welensii* | LC | YES | 16 |
| *Cercomorphus bicolor* | DD | NO | 0 |
| *Cercomorphus duvalii* | DD | NO | 0 |
| *Cerophytum elateroides* | DD | NO | 0 |
| *Ceruchus chrysomelinus* | EN | YES | 4 |
| *Cerylon deplanatum* | NT | YES | 5 |
| *Cerylon fagi* | LC | YES | 13 |
| *Cerylon ferrugineum* | LC | YES | 8 |
| *Cerylon histeroides* | LC | YES | 19 |
| *Cerylon impressum* | NT | YES | 3 |
| *Cetonia aurata* | LC | YES | 26 |
| *Cetonia carthami* | VU | NO | 0 |
| *Chaetoptelius vestitus* | LC | NO | 0 |
| *Chalcophora detrita* | EN | NO | 0 |
| *Chalcophora intermedia* | EN | NO | 0 |
| *Chalcophora mariana* | LC | YES | 5 |
| *Chalcophora massiliensis* | LC | NO | 0 |
| *Chalcophorella fabricii* | VU | NO | 0 |
| *Chlorophorus figuratus* | LC | YES | 3 |
| *Chlorophorus glabromaculatus* | LC | YES | 1 |
| *Chlorophorus glaucus* | EN | NO | 0 |
| *Chlorophorus sartor* | LC | YES | 6 |
| *Choerorhinus squalidus* | LC | NO | 0 |
| *Choragus aureolineatus* | DD | NO | 0 |
| *Choragus sheppardi* | LC | YES | 3 |
| *Chrysanthia geniculata* | DD | YES | 19 |
| *Chrysanthia viridissima* | LC | YES | 49 |
| *Chrysobothris affinis* | LC | YES | 10 |
| *Chrysobothris chrysostigma* | LC | YES | 7 |
| *Chrysobothris dorsata* | LC | NO | 0 |
| *Chrysobothris igniventris* | EN | YES | 1 |
| *Chrysobothris solieri* | LC | YES | 1 |
| *Cis alni* | LC | NO | 0 |
| *Cis bidentatus* | LC | YES | 6 |
| *Cis boleti* | LC | YES | 25 |
| *Cis castaneus* | LC | YES | 7 |
| *Cis coluber* | LC | NO | 0 |
| *Cis comptus* | LC | YES | 13 |
| *Cis dentatus* | LC | YES | 10 |
| *Cis festivus* | LC | YES | 8 |
| *Cis fissicollis* | LC | NO | 0 |
| *Cis fissicornis* | LC | NO | 0 |
| *Cis glabratus* | LC | YES | 14 |
| *Cis hispidus* | LC | YES | 13 |
| *Cis jacquemarti* | LC | NO | 0 |
| *Cis laminatus* | LC | NO | 0 |
| *Cis lineatocribratus* | LC | YES | 6 |
| *Cis lucasi* | VU | NO | 0 |
| *Cis micans* | LC | YES | 9 |
| *Cis multidentatus* | VU | NO | 0 |
| *Cis nitidus* | LC | YES | 22 |
| *Cis oblongus* | LC | NO | 0 |
| *Cis perrisi* | VU | NO | 0 |
| *Cis puctulatus* | LC | NO | 0 |
| *Cis punctifer* | LC | NO | 0 |
| *Cis pygmaeus* | VU | YES | 5 |
| *Cis quadridens* | LC | NO | 0 |
| *Cis quadridentulus* | LC | NO | 0 |
| *Cis rugulosus* | LC | NO | 0 |
| *Cis sericeus* | LC | NO | 0 |
| *Cis setiger* | LC | YES | 5 |
| *Cis striatulus* | LC | NO | 0 |
| *Cis vestitus* | LC | YES | 6 |
| *Cisurgus ragusae* | LC | NO | 0 |
| *Clambus armadillo* | DD | YES | 18 |
| *Clambus caucasus* | DD | NO | 0 |
| *Clambus dux* | LC | NO | 0 |
| *Clambus evae* | DD | NO | 0 |
| *Clambus hayekae* | DD | NO | 0 |
| *Clambus minutus* | LC | YES | 6 |
| *Clambus nigrellus* | DD | YES | 6 |
| *Clambus nigriclavis* | DD | NO | 0 |
| *Clambus pallidulus* | LC | YES | 2 |
| *Clambus pilosellus* | DD | NO | 0 |
| *Clambus pubescens* | LC | YES | 5 |
| *Clambus punctulum* | LC | YES | 13 |
| *Clemmus troglodytes* | CR | NO | 0 |
| *Clerus mutillarius* | NT | YES | 7 |
| *Clinidium canaliculatum* | VU | NO | 0 |
| *Clypastrea brunnea* | DD | NO | 0 |
| *Clypastrea lata* | DD | NO | 0 |
| *Clypastrea pusilla* | DD | NO | 0 |
| *Clypastrea reitteri* | DD | NO | 0 |
| *Clytus rhamni* | LC | YES | 3 |
| *Clytus triangulimacula* | VU | NO | 0 |
| *Colobicus hirtus* | NT | YES | 2 |
| *Colposis mutilatus* | NT | YES | 2 |
| *Colydium elongatum* | LC | YES | 18 |
| *Colydium filiforme* | NT | NO | 0 |
| *Combocerus glaber* | EN | NO | 0 |
| *Conopalpus brevicollis* | NT | YES | 12 |
| *Conopalpus testaceus* | NT | YES | 17 |
| *Coraebus fasciatus* | LC | YES | 1 |
| *Coraebus undatus* | NT | NO | 0 |
| *Cornumutila lineata* | CR | NO | 0 |
| *Corticaria abietorum* | DD | YES | 13 |
| *Corticaria bella* | DD | NO | 0 |
| *Corticaria ciliata* | DD | NO | 0 |
| *Corticaria corsica* | DD | NO | 0 |
| *Corticaria crenicollis* | LC | YES | 2 |
| *Corticaria crenulata* | LC | YES | 2 |
| *Corticaria cucujiformis* | DD | NO | 0 |
| *Corticaria elongata* | LC | YES | 13 |
| *Corticaria ferruginea* | LC | NO | 0 |
| *Corticaria foveola* | LC | YES | 4 |
| *Corticaria fulva* | LC | YES | 5 |
| *Corticaria illaesa* | LC | NO | 0 |
| *Corticaria impressa* | LC | YES | 14 |
| *Corticaria lapponica* | DD | YES | 4 |
| *Corticaria lateritia* | DD | NO | 0 |
| *Corticaria linearis* | DD | NO | 0 |
| *Corticaria longicornis* | LC | NO | 0 |
| *Corticaria pineti* | LC | NO | 0 |
| *Corticaria pubescens* | LC | NO | 0 |
| *Corticaria saginata* | LC | YES | 1 |
| *Corticaria serrata* | LC | YES | 9 |
| *Corticaria solarii* | DD | NO | 0 |
| *Corticaria umbilicata* | DD | YES | 2 |
| *Corticaria weisei* | LC | NO | 0 |
| *Corticarina fulvipes* | LC | NO | 0 |
| *Corticarina lambiana* | DD | NO | 0 |
| *Corticarina similata* | LC | YES | 65 |
| *Corticarina truncatella* | LC | YES | 12 |
| *Corticeus bicolor* | LC | YES | 7 |
| *Corticeus bicoloroides* | CR | NO | 0 |
| *Corticeus fasciatus* | LC | NO | 0 |
| *Corticeus linearis* | LC | YES | 3 |
| *Corticeus pini* | LC | YES | 2 |
| *Corticeus suberis* | DD | NO | 0 |
| *Corticeus unicolor* | LC | YES | 21 |
| *Corticeus versipellis* | DD | NO | 0 |
| *Corticus celtis* | LC | NO | 0 |
| *Cortinicara gibbosa* | LC | YES | 521 |
| *Cortodera aspromontana* | NT | NO | 0 |
| *Cortodera femorata* | NT | YES | 10 |
| *Cortodera humeralis* | LC | YES | 7 |
| *Corylophus sublaevipennis* | DD | NO | 0 |
| *Cossonus cylindricus* | LC | NO | 0 |
| *Cossonus linearis* | LC | YES | 6 |
| *Cossonus parallelepipedus* | LC | NO | 0 |
| *Cotaster cuneipennis* | NT | NO | 0 |
| *Cotaster uncipes* | LC | NO | 0 |
| *Coxelus pictus* | LC | YES | 13 |
| *Crowsoniella relicta* | DD | NO | 0 |
| *Cryphalus asperatus* | LC | YES | 3 |
| *Cryphalus intermedius* | LC | NO | 0 |
| *Cryphalus numidicus* | LC | NO | 0 |
| *Cryphalus piceae* | LC | YES | 2 |
| *Cryphalus saltuarius* | LC | YES | 4 |
| *Cryptarcha strigata* | LC | YES | 4 |
| *Cryptarcha undata* | NT | YES | 4 |
| *Cryptolestes abietis* | NT | NO | 0 |
| *Cryptolestes alternans* | NT | YES | 1 |
| *Cryptolestes clematidis* | LC | NO | 0 |
| *Cryptolestes corticinus* | VU | YES | 4 |
| *Cryptolestes duplicatus* | NT | YES | 13 |
| *Cryptolestes ferrugineus* | LC | YES | 5 |
| *Cryptolestes fractipennis* | LC | NO | 0 |
| *Cryptolestes hypobori* | LC | NO | 0 |
| *Cryptolestes juniperi* | LC | NO | 0 |
| *Cryptolestes perrisi* | NT | NO | 0 |
| *Cryptolestes spartii* | LC | YES | 2 |
| *Cryptolestes weisei* | CR | NO | 0 |
| *Cryptophagus acutangulus* | LC | YES | 2 |
| *Cryptophagus badius* | LC | YES | 2 |
| *Cryptophagus brisouti* | DD | NO | 0 |
| *Cryptophagus cellaris* | DD | NO | 0 |
| *Cryptophagus croaticus* | DD | NO | 0 |
| *Cryptophagus cylindrellus* | DD | NO | 0 |
| *Cryptophagus dentatus* | LC | YES | 5 |
| *Cryptophagus denticulatus* | LC | NO | 0 |
| *Cryptophagus dorsalis* | DD | YES | 4 |
| *Cryptophagus durus* | DD | NO | 0 |
| *Cryptophagus falcozi* | DD | NO | 0 |
| *Cryptophagus fasciatus* | LC | NO | 0 |
| *Cryptophagus fuscicornis* | DD | NO | 0 |
| *Cryptophagus intermedius* | DD | NO | 0 |
| *Cryptophagus labilis* | VU | YES | 1 |
| *Cryptophagus lapponicus* | DD | YES | 2 |
| *Cryptophagus laticollis* | LC | YES | 1 |
| *Cryptophagus montanus* | DD | NO | 0 |
| *Cryptophagus nitidulus* | DD | NO | 0 |
| *Cryptophagus pallidus* | LC | YES | 7 |
| *Cryptophagus parallelus* | DD | YES | 1 |
| *Cryptophagus pilosus* | LC | YES | 4 |
| *Cryptophagus populi* | DD | YES | 3 |
| *Cryptophagus punctipennis* | DD | NO | 0 |
| *Cryptophagus quercinus* | NT | YES | 2 |
| *Cryptophagus reflexicollis* | DD | NO | 0 |
| *Cryptophagus reflexus* | LC | NO | 0 |
| *Cryptophagus ruficornis* | LC | NO | 0 |
| *Cryptophagus scanicus* | LC | YES | 9 |
| *Cryptophagus schmidtii* | DD | YES | 5 |
| *Cryptophagus schroetteri* | DD | NO | 0 |
| *Cryptophagus scutellatus* | LC | YES | 1 |
| *Cryptophagus setulosus* | DD | YES | 5 |
| *Cryptophagus skalitzkyi* | DD | NO | 0 |
| *Cryptophagus sporadum* | DD | NO | 0 |
| *Cryptophagus subdepressus* | DD | NO | 0 |
| *Cryptophagus subfumatus* | LC | NO | 0 |
| *Cryptophagus thomsoni* | LC | NO | 0 |
| *Cryptophagus uncinatus* | LC | NO | 0 |
| *Cryptophilus integer* | LC | YES | 10 |
| *Cryptorhynchus lapathi* | LC | YES | 13 |
| *Crypturgus cinereus* | LC | YES | 6 |
| *Crypturgus cribrellus* | LC | NO | 0 |
| *Crypturgus hispidulus* | VU | YES | 11 |
| *Crypturgus mediterraneus* | LC | NO | 0 |
| *Crypturgus numidicus* | LC | NO | 0 |
| *Crypturgus pusillus* | LC | YES | 3 |
| *Cteniopus neapolitanus* | NT | NO | 0 |
| *Cteniopus sulphureus* | LC | YES | 20 |
| *Cteniopus sulphuripes* | NT | NO | 0 |
| *Ctesias serra* | LC | YES | 9 |
| *Cucujus cinnaberinus* | VU | YES | 3 |
| *Cucujus haematodes* | EN | YES | 9 |
| *Cucujus tulliae* | EN | NO | 0 |
| *Curelius exiguus* | LC | NO | 0 |
| *Curimus erinaceus* | DD | NO | 0 |
| *Curimus lariensis* | DD | NO | 0 |
| *Curimus petraeus* | DD | NO | 0 |
| *Cychramus luteus* | LC | YES | 38 |
| *Cychramus variegatus* | NT | YES | 10 |
| *Cyclobacanius medvidovici* | CR | NO | 0 |
| *Cyclobacanius soliman* | EN | NO | 0 |
| *Cyllodes ater* | CR | YES | 3 |
| *Cyphaea curtula* | EN | NO | 0 |
| *Cyrtosus abeillei* | LC | NO | 0 |
| *Dacne bipustulata* | LC | YES | 19 |
| *Dacne notata* | LC | NO | 0 |
| *Dacne pontica* | NT | NO | 0 |
| *Dacne rufifrons* | NT | YES | 5 |
| *Dadobia immersa* | LC | YES | 2 |
| *Danosoma fasciatum* | NT | YES | 6 |
| *Dasycerus sulcatus* | LC | YES | 1 |
| *Dasytes aeneiventris* | LC | NO | 0 |
| *Dasytes aeratus* | LC | YES | 18 |
| *Dasytes caeruleus* | LC | YES | 1 |
| *Dasytes croceipes* | LC | NO | 0 |
| *Dasytes doderoi* | CR | NO | 0 |
| *Dasytes iteratus* | LC | NO | 0 |
| *Dasytes nigroaeneus* | LC | NO | 0 |
| *Dasytes nigrocyaneus* | LC | YES | 2 |
| *Dasytes pauperculus* | LC | NO | 0 |
| *Dasytes plumbeus* | LC | YES | 48 |
| *Dasytes subalpinus* | LC | YES | 2 |
| *Dasytes thoracicus* | LC | NO | 0 |
| *Dasytes virens* | LC | YES | 19 |
| *Deilus fugax* | LC | NO | 0 |
| *Dendroctonus micans* | LC | NO | 0 |
| *Dendrophagus crenatus* | VU | YES | 4 |
| *Dendrophilus punctatus* | LC | YES | 12 |
| *Dendrophilus pygmaeus* | EN | YES | 11 |
| *Denops albofasciatus* | NT | YES | 3 |
| *Denticollis linearis* | CR | YES | 55 |
| *Denticollis rubens* | NT | YES | 12 |
| *Dermestoides sanguinicollis* | EN | YES | 4 |
| *Derodontus macularis* | RE | YES | 3 |
| *Derodontus raffrayi* | VU | NO | 0 |
| *Deroplia genei* | NT | YES | 1 |
| *Deroplia troberti* | NT | NO | 0 |
| *Dexiogyia corticina* | LC | YES | 6 |
| *Diacanthous undulatus* | VU | YES | 6 |
| *Diaclina fagi* | DD | YES | 8 |
| *Diaclina testudinea* | EN | NO | 0 |
| *Dialycera distincticornis* | LC | NO | 0 |
| *Diaperis boleti* | LC | YES | 24 |
| *Dicerca aenea* | LC | YES | 1 |
| *Dicerca alni* | NT | NO | 0 |
| *Dicerca berolinensis* | NT | YES | 1 |
| *Dicerca moesta* | CR | YES | 3 |
| *Dichromacalles rolletii* | NT | YES | 4 |
| *Dictyoptera aurora* | LC | YES | 37 |
| *Dienerella anatolica* | LC | NO | 0 |
| *Dienerella argus* | LC | NO | 0 |
| *Dienerella beloni* | DD | NO | 0 |
| *Dienerella clathrata* | LC | YES | 8 |
| *Dienerella corsica* | DD | NO | 0 |
| *Dienerella costulata* | LC | NO | 0 |
| *Dienerella elegans* | LC | NO | 0 |
| *Dienerella elongata* | LC | YES | 14 |
| *Dienerella filiformis* | LC | NO | 0 |
| *Dienerella filum* | DD | NO | 0 |
| *Dienerella parilis* | LC | NO | 0 |
| *Dienerella pilifera* | LC | NO | 0 |
| *Dienerella polyhymnia* | LC | NO | 0 |
| *Dienerella ruficollis* | LC | YES | 3 |
| *Dienerella separanda* | LC | NO | 0 |
| *Dienerella siciliana* | DD | NO | 0 |
| *Dienerella vincenti* | DD | YES | 2 |
| *Dima elateroides* | EN | YES | 2 |
| *Dinaraea aequata* | LC | YES | 21 |
| *Dinaraea angustula* | LC | YES | 2 |
| *Dinaraea arcana* | LC | YES | 3 |
| *Dinaraea linearis* | NT | YES | 4 |
| *Dinoptera collaris* | LC | YES | 36 |
| *Diodesma denticincta* | NT | NO | 0 |
| *Diodesma subterranea* | LC | NO | 0 |
| *Diphyllocis opaculus* | LC | NO | 0 |
| *Diplocoelus fagi* | LC | YES | 4 |
| *Dircaea australis* | DD | YES | 1 |
| *Dircaea quadriguttata* | NT | NO | 0 |
| *Dissoleucas niveirostris* | LC | YES | 20 |
| *Dolotarsus lividus* | NT | YES | 2 |
| *Dorcatoma chrysomelina* | LC | YES | 15 |
| *Dorcatoma dresdensis* | LC | YES | 13 |
| *Dorcatoma flavicornis* | LC | NO | 0 |
| *Dorcatoma lanuginosa* | EN | YES | 3 |
| *Dorcatoma punctulata* | VU | YES | 3 |
| *Dorcatoma setosella* | LC | YES | 3 |
| *Dorcatoma substriata* | LC | YES | 5 |
| *Dorcus musimon* | VU | NO | 0 |
| *Dorcus parallelipipedus* | LC | YES | 22 |
| *Drapetes mordelloides* | LC | YES | 3 |
| *Dromaeolus barnabita* | VU | YES | 1 |
| *Dropephylla ammanni* | NT | NO | 0 |
| *Dropephylla brevicornis* | NT | NO | 0 |
| *Dropephylla devillei* | NT | NO | 0 |
| *Dropephylla gracilicornis* | VU | YES | 1 |
| *Dropephylla ioptera* | LC | YES | 13 |
| *Dropephylla koltzei* | DD | YES | 2 |
| *Dropephylla linearis* | VU | YES | 5 |
| *Dropephylla perforata* | VU | NO | 0 |
| *Dropephylla vilis* | NT | NO | 0 |
| *Drymochares truquii* | VU | NO | 0 |
| *Dryocoetes alni* | LC | YES | 1 |
| *Dryocoetes autographus* | LC | YES | 31 |
| *Dryocoetes hectographus* | LC | YES | 11 |
| *Dryocoetes italus* | DD | NO | 0 |
| *Dryocoetes villosus* | LC | YES | 16 |
| *Dryophilus anobioides* | LC | YES | 1 |
| *Dryophilus densipilis* | LC | NO | 0 |
| *Dryophilus forticornis* | VU | NO | 0 |
| *Dryophilus longicollis* | LC | NO | 0 |
| *Dryophilus luigionii* | VU | NO | 0 |
| *Dryophilus pusillus* | LC | YES | 13 |
| *Dryophilus siculus* | NT | NO | 0 |
| *Dryophthorus corticalis* | NT | YES | 12 |
| *Ebaeus appendiculatus* | LC | NO | 0 |
| *Ebaeus battoni* | LC | NO | 0 |
| *Ebaeus coerulescens* | LC | NO | 0 |
| *Ebaeus collaris* | LC | NO | 0 |
| *Ebaeus flavicornis* | LC | YES | 3 |
| *Ebaeus gibbus* | LC | NO | 0 |
| *Ebaeus humilis* | LC | NO | 0 |
| *Ebaeus ruffoi* | LC | NO | 0 |
| *Ebaeus thoracicus* | LC | YES | 13 |
| *Echinodera bellieri* | NT | YES | 11 |
| *Echinodera brisouti* | LC | YES | 4 |
| *Echinodera capiomonti* | LC | YES | 1 |
| *Echinodera hypocrita* | LC | YES | 29 |
| *Echinodera ibleiensis* | NT | YES | 1 |
| *Echinodera kostenbaderi* | NT | NO | 0 |
| *Echinodera nebrodiensis* | NT | YES | 2 |
| *Echinodera peragalloi* | LC | YES | 12 |
| *Echinodera settefratelliensis* | NT | YES | 4 |
| *Echinodera siciliensis* | NT | YES | 5 |
| *Echinodera tyrrhenica* | NT | NO | 0 |
| *Echinodera variegata* | NT | YES | 2 |
| *Echinomorphus ravouxi* | NT | NO | 0 |
| *Ectamenogonus montandoni* | EN | NO | 0 |
| *Elater ferrugineus* | VU | YES | 4 |
| *Elateroides dermestoides* | NT | YES | 85 |
| *Eledona agricola* | NT | YES | 14 |
| *Eledonoprius armatus* | CR | YES | 1 |
| *Eledonoprius serrifrons* | CR | NO | 0 |
| *Endecatomus reticulatus* | NT | NO | 0 |
| *Endomychus coccineus* | LC | YES | 16 |
| *Endophloeus marcovichianus* | NT | YES | 1 |
| *Enedreytes hilaris* | LC | NO | 0 |
| *Enedreytes sepicola* | LC | YES | 5 |
| *Enicmus atriceps* | DD | NO | 0 |
| *Enicmus brevicornis* | LC | YES | 4 |
| *Enicmus fungicola* | LC | YES | 1 |
| *Enicmus histrio* | LC | YES | 1 |
| *Enicmus rugosus* | LC | YES | 9 |
| *Enicmus testaceus* | LC | YES | 2 |
| *Enicmus transversus* | LC | YES | 7 |
| *Enneadesmus trispinosus* | NT | NO | 0 |
| *Ennearthron cornutum* | LC | YES | 14 |
| *Ennearthron filum* | NT | NO | 0 |
| *Ennearthron fronticorne* | LC | NO | 0 |
| *Ennearthron pruinosulum* | VU | NO | 0 |
| *Enoplium doderoi* | EN | NO | 0 |
| *Enoplium serraticorne* | NT | NO | 0 |
| *Epierus comptus* | LC | NO | 0 |
| *Epierus italicus* | LC | NO | 0 |
| *Epiphanis cornutus* | VU | YES | 4 |
| *Episernus angulicollis* | VU | NO | 0 |
| *Episernus gentilis* | LC | NO | 0 |
| *Episernus granulatus* | VU | YES | 2 |
| *Episernus striatellus* | VU | NO | 0 |
| *Epuraea angustula* | VU | YES | 7 |
| *Epuraea argus* | DD | NO | 0 |
| *Epuraea biguttata* | LC | YES | 8 |
| *Epuraea binotata* | VU | YES | 19 |
| *Epuraea boreella* | LC | YES | 5 |
| *Epuraea deubeli* | EN | YES | 5 |
| *Epuraea distincta* | NT | YES | 6 |
| *Epuraea fageticola* | VU | NO | 0 |
| *Epuraea guttata* | LC | YES | 1 |
| *Epuraea laeviuscula* | EN | YES | 1 |
| *Epuraea limbata* | VU | NO | 0 |
| *Epuraea longiclavis* | NT | NO | 0 |
| *Epuraea longula* | LC | NO | 0 |
| *Epuraea marseuli* | LC | YES | 17 |
| *Epuraea melanocephala* | LC | YES | 20 |
| *Epuraea muehli* | NT | YES | 8 |
| *Epuraea neglecta* | VU | YES | 8 |
| *Epuraea oblonga* | VU | YES | 4 |
| *Epuraea pallescens* | LC | YES | 12 |
| *Epuraea placida* | VU | YES | 1 |
| *Epuraea pygmaea* | LC | YES | 9 |
| *Epuraea rufomarginata* | VU | YES | 8 |
| *Epuraea silacea* | VU | YES | 4 |
| *Epuraea terminalis* | LC | YES | 9 |
| *Epuraea thoracica* | VU | YES | 6 |
| *Epuraea unicolor* | LC | YES | 20 |
| *Epuraea variegata* | LC | YES | 14 |
| *Ergates faber* | LC | NO | 0 |
| *Ernobius abietinus* | LC | YES | 5 |
| *Ernobius abietis* | LC | YES | 11 |
| *Ernobius angelinii* | EN | NO | 0 |
| *Ernobius angusticollis* | VU | YES | 11 |
| *Ernobius freudei* | EN | NO | 0 |
| *Ernobius fulvus* | EN | YES | 1 |
| *Ernobius gigas* | EN | NO | 0 |
| *Ernobius juniperi* | VU | NO | 0 |
| *Ernobius kiesenwetteri* | LC | NO | 0 |
| *Ernobius laticollis* | NT | NO | 0 |
| *Ernobius longicornis* | LC | YES | 5 |
| *Ernobius mollis* | LC | YES | 9 |
| *Ernobius mulsanti* | VU | NO | 0 |
| *Ernobius nigrinus* | LC | YES | 9 |
| *Ernobius parens* | LC | NO | 0 |
| *Ernobius pini* | LC | YES | 6 |
| *Ernobius pruinosus* | EN | NO | 0 |
| *Ernobius rufus* | EN | NO | 0 |
| *Ernoporicus fagi* | LC | NO | 0 |
| *Ernoporus tiliae* | LC | YES | 13 |
| *Esarcus abeillei* | NT | NO | 0 |
| *Esarcus baudii* | VU | NO | 0 |
| *Esarcus fiorii* | VU | NO | 0 |
| *Etorofus pubescens* | NT | YES | 5 |
| *Eubrachium pusillum* | LC | NO | 0 |
| *Eucnemis capucina* | NT | YES | 3 |
| *Euplectus bonvouloiri* | LC | NO | 0 |
| *Euplectus brunneus* | NT | YES | 2 |
| *Euplectus corsicus* | LC | NO | 0 |
| *Euplectus decipiens* | VU | NO | 0 |
| *Euplectus doderoi* | VU | NO | 0 |
| *Euplectus duponti* | CR | NO | 0 |
| *Euplectus frater* | EN | NO | 0 |
| *Euplectus infirmus* | EN | YES | 6 |
| *Euplectus karsteni* | LC | YES | 16 |
| *Euplectus kirbyi* | LC | YES | 2 |
| *Euplectus linderi* | NT | NO | 0 |
| *Euplectus mutator* | NT | YES | 3 |
| *Euplectus nanus* | VU | YES | 3 |
| *Euplectus piceus* | NT | YES | 7 |
| *Euplectus punctatus* | NT | YES | 7 |
| *Euplectus sparsus* | NT | NO | 0 |
| *Euplectus theryi* | NT | NO | 0 |
| *Euplectus tholini* | VU | NO | 0 |
| *Euplectus validus* | VU | NO | 0 |
| *Euplectus verticalis* | NT | NO | 0 |
| *Euryptilium gillmeisteri* | DD | NO | 0 |
| *Euryptilium saxonicum* | DD | YES | 3 |
| *Eurythyrea austriaca* | VU | NO | 0 |
| *Eurythyrea micans* | LC | NO | 0 |
| *Eurythyrea quercus* | CR | NO | 0 |
| *Euryusa castanoptera* | NT | YES | 11 |
| *Euryusa optabilis* | LC | YES | 13 |
| *Euryusa pipitzi* | CR | YES | 3 |
| *Euryusa sinuata* | NT | YES | 1 |
| *Eusphyrus vasconicus* | DD | NO | 0 |
| *Eustrophus dermestoides* | NT | NO | 0 |
| *Evodinus clathratus* | NT | YES | 10 |
| *Exocentrus adspersus* | LC | YES | 28 |
| *Exocentrus lusitanus* | NT | YES | 13 |
| *Exocentrus punctipennis* | LC | YES | 11 |
| *Falsogastrallus unistriatus* | EN | NO | 0 |
| *Farsus dubius* | LC | NO | 0 |
| *Gabrius splendidulus* | LC | YES | 21 |
| *Gasterocercus depressirostris* | NT | NO | 0 |
| *Gastrallus corsicus* | LC | NO | 0 |
| *Gastrallus immarginatus* | LC | YES | 3 |
| *Gastrallus kocheri* | VU | NO | 0 |
| *Gastrallus laevigatus* | LC | YES | 10 |
| *Gastrallus mauritanicus* | VU | NO | 0 |
| *Gaurotes virginea* | LC | YES | 32 |
| *Gerandryus aetnensis* | EN | NO | 0 |
| *Glaphyra kiesenwetteri* | VU | NO | 0 |
| *Glaphyra marmottani* | VU | NO | 0 |
| *Glaphyra umbellatarum* | LC | NO | 0 |
| *Glischrochilus hortensis* | LC | YES | 16 |
| *Glischrochilus quadriguttatus* | VU | YES | 17 |
| *Glischrochilus quadripunctatus* | NT | YES | 13 |
| *Globicornis bifasciata* | NT | YES | 1 |
| *Globicornis corticalis* | NT | NO | 0 |
| *Globicornis emarginata* | LC | NO | 0 |
| *Globicornis fasciata* | LC | NO | 0 |
| *Globicornis luckowi* | NT | NO | 0 |
| *Globicornis nigripes* | LC | YES | 2 |
| *Globicornis picta* | LC | NO | 0 |
| *Globicornis sulcata* | NT | NO | 0 |
| *Globicornis tristis* | LC | NO | 0 |
| *Globicornis variegata* | LC | NO | 0 |
| *Gnathoncus rotundatus* | LC | YES | 1 |
| *Gnathotrichus materiarius* | VU | YES | 5 |
| *Gnorimus decempunctatus* | EN | NO | 0 |
| *Gnorimus nobilis* | NT | YES | 5 |
| *Gnorimus variabilis* | VU | YES | 9 |
| *Gracilia minuta* | LC | YES | 1 |
| *Grammoptera abdominalis* | NT | YES | 6 |
| *Grammoptera ruficornis* | LC | YES | 36 |
| *Grammoptera ustulata* | NT | YES | 9 |
| *Grammoptera viridipennis* | EN | NO | 0 |
| *Grynobius planus* | LC | NO | 0 |
| *Grynocharis oblonga* | NT | YES | 6 |
| *Hadreule elongatula* | VU | NO | 0 |
| *Hadrobregmus denticollis* | LC | YES | 10 |
| *Hadrobregmus pertinax* | LC | YES | 13 |
| *Halacritus punctum* | LC | NO | 0 |
| *Hallomenus axillaris* | NT | YES | 9 |
| *Hallomenus binotatus* | NT | YES | 10 |
| *Hapalaraea pygmaea* | VU | YES | 3 |
| *Haterumelater fulvago* | EN | NO | 0 |
| *Hedobia pubescens* | LC | NO | 0 |
| *Helops coeruleus* | LC | YES | 6 |
| *Helops rossii* | LC | NO | 0 |
| *Hemicoelus canaliculatus* | LC | YES | 10 |
| *Hemicoelus costatus* | LC | YES | 20 |
| *Hemicoelus fulvicornis* | LC | YES | 1 |
| *Hemicoelus rufipennis* | LC | NO | 0 |
| *Henoticus serratus* | LC | NO | 0 |
| *Herophila tristis* | LC | YES | 2 |
| *Hesperophanes sericeus* | LC | YES | 1 |
| *Hesperus rufipennis* | NT | YES | 10 |
| *Hexarthrum capitulum* | DD | NO | 0 |
| *Hexarthrum exiguum* | DD | NO | 0 |
| *Hololepta plana* | LC | YES | 9 |
| *Homalota plana* | LC | YES | 9 |
| *Homophthalmus rugicollis* | LC | YES | 6 |
| *Hylaia dalmatina* | DD | NO | 0 |
| *Hylaia rubricollis* | DD | NO | 0 |
| *Hylastes angustatus* | LC | YES | 2 |
| *Hylastes ater* | LC | YES | 4 |
| *Hylastes attenuatus* | LC | YES | 6 |
| *Hylastes batnensis* | DD | YES | 2 |
| *Hylastes brunneus* | VU | YES | 18 |
| *Hylastes cunicularius* | LC | YES | 27 |
| *Hylastes gergeri* | DD | NO | 0 |
| *Hylastes linearis* | LC | NO | 0 |
| *Hylastes opacus* | VU | YES | 17 |
| *Hylastinus fankhauseri* | LC | YES | 2 |
| *Hylastinus obscurus* | LC | YES | 3 |
| *Hylesinus crenatus* | LC | YES | 11 |
| *Hylesinus toranio* | LC | YES | 3 |
| *Hylesinus varius* | LC | YES | 7 |
| *Hylis cariniceps* | NT | YES | 1 |
| *Hylis foveicollis* | NT | YES | 2 |
| *Hylis olexai* | NT | YES | 3 |
| *Hylis procerulus* | DD | NO | 0 |
| *Hylis simonae* | NT | NO | 0 |
| *Hylobius abietis* | LC | YES | 35 |
| *Hylobius excavatus* | LC | NO | 0 |
| *Hylobius pinastri* | LC | YES | 1 |
| *Hylobius transversovittatus* | LC | YES | 1 |
| *Hylotrupes bajulus* | LC | YES | 13 |
| *Hylurgops glabratus* | LC | YES | 2 |
| *Hylurgops palliatus* | LC | YES | 34 |
| *Hylurgus ligniperda* | LC | NO | 0 |
| *Hylurgus micklitzi* | LC | NO | 0 |
| *Hymenalia rufipes* | LC | YES | 13 |
| *Hymenophorus doublieri* | NT | YES | 1 |
| *Hypebaeus flavicollis* | LC | NO | 0 |
| *Hypebaeus flavipes* | LC | YES | 7 |
| *Hyperisus declive* | EN | NO | 0 |
| *Hyperisus plumbeum* | LC | NO | 0 |
| *Hypnogyra angularis* | LC | YES | 15 |
| *Hypoborus ficus* | LC | YES | 1 |
| *Hypoganus inunctus* | EN | YES | 12 |
| *Hypulus bifasciatus* | NT | NO | 0 |
| *Hypulus quercinus* | NT | YES | 6 |
| *Icosium tomentosum* | LC | NO | 0 |
| *Iphthiminus italicus* | VU | YES | 1 |
| *Ipidia binotata* | VU | YES | 4 |
| *Ipidia sexguttata* | DD | NO | 0 |
| *Ips acuminatus* | LC | YES | 44 |
| *Ips amitinus* | LC | NO | 0 |
| *Ips cembrae* | LC | NO | 0 |
| *Ips sexdentatus* | LC | YES | 67 |
| *Ips typographus* | LC | YES | 38 |
| *Ischnodes sanguinicollis* | VU | YES | 4 |
| *Ischnoglossa elegantula* | NT | NO | 0 |
| *Ischnoglossa prolixa* | NT | YES | 5 |
| *Ischnomera caerulea* | LC | YES | 2 |
| *Ischnomera cinerascens* | LC | YES | 4 |
| *Ischnomera cyanea* | LC | YES | 13 |
| *Ischnomera sanguinicollis* | LC | YES | 4 |
| *Ischnomera xanthoderes* | DD | YES | 1 |
| *Isidus moreli* | VU | NO | 0 |
| *Isorhipis melasoides* | LC | YES | 3 |
| *Isotomus barbarae* | NT | NO | 0 |
| *Isotomus speciosus* | CR | NO | 0 |
| *Italohelops subchalybaeus* | NT | NO | 0 |
| *Judolia sexmaculata* | NT | YES | 8 |
| *Kisanthobia ariasi* | VU | NO | 0 |
| *Kissophagus hederae* | LC | YES | 1 |
| *Kissophagus novaki* | LC | YES | 1 |
| *Korynetes caeruleus* | NT | YES | 8 |
| *Korynetes pusillus* | NT | NO | 0 |
| *Kyklioacalles barbarus* | NT | NO | 0 |
| *Kyklioacalles characivorus* | LC | NO | 0 |
| *Kyklioacalles fausti* | LC | YES | 3 |
| *Kyklioacalles navieresi* | LC | YES | 8 |
| *Kyklioacalles provincialis* | VU | YES | 4 |
| *Kyklioacalles punctaticollis* | LC | YES | 20 |
| *Kyklioacalles roboris* | LC | YES | 13 |
| *Kyklioacalles saccoi* | NT | YES | 1 |
| *Kyklioacalles solarii* | LC | NO | 0 |
| *Kyklioacalles teter* | NT | YES | 2 |
| *Lacon lepidopterus* | EN | NO | 0 |
| *Lacon punctatus* | LC | YES | 1 |
| *Lacon querceus* | EN | YES | 2 |
| *Laemophloeus kraussi* | NT | NO | 0 |
| *Laemophloeus monilis* | LC | YES | 2 |
| *Laemophloeus nigricollis* | NT | NO | 0 |
| *Lamia textor* | NT | YES | 5 |
| *Lamprodila decipiens* | LC | NO | 0 |
| *Lamprodila festiva* | LC | NO | 0 |
| *Lamprodila mirifica* | LC | YES | 2 |
| *Lamprodila rutilans* | LC | NO | 0 |
| *Lamprodila solieri* | CR | NO | 0 |
| *Langelandia anophtalma* | LC | NO | 0 |
| *Langelandia antennaria* | CR | NO | 0 |
| *Langelandia ausonica* | CR | NO | 0 |
| *Langelandia exigua* | CR | NO | 0 |
| *Langelandia hummleri* | CR | NO | 0 |
| *Langelandia leonhardi* | EN | NO | 0 |
| *Langelandia montalbica* | CR | NO | 0 |
| *Langelandia nitidicollis* |  | NO | 0 |
| *Langelandia vienensis* |  | NO | 0 |
| *Laricobius erichsoni* | DD | YES | 7 |
| *Lathropus sepicola* | NT | YES | 1 |
| *Latipalpis plana* | LC | YES | 1 |
| *Latridius amplus* | DD | NO | 0 |
| *Latridius anthracinus* | LC | YES | 1 |
| *Latridius assimilis* | DD | NO | 0 |
| *Latridius brevicollis* | DD | NO | 0 |
| *Latridius consimilis* | LC | YES | 4 |
| *Latridius hirtus* | LC | YES | 1 |
| *Latridius minutus* | LC | YES | 9 |
| *Latridius pseudominutus* | DD | YES | 2 |
| *Leiestes seminiger* | VU | NO | 0 |
| *Leioderes kollari* | NT | NO | 0 |
| *Leiopus femoratus* | NT | YES | 3 |
| *Leiopus nebulosus* | LC | YES | 13 |
| *Leiopus settei* | CR | NO | 0 |
| *Leptoplectus spinolae* | VU | YES | 3 |
| *Leptura aethiops* | CR | YES | 1 |
| *Leptura aurulenta* | LC | YES | 2 |
| *Lepturobosca virens* | NT | YES | 9 |
| *Leptusa fuliginosa* | VU | NO | 0 |
| *Leptusa fumida* | LC | YES | 14 |
| *Leptusa major* | VU | NO | 0 |
| *Leptusa pulchella* | LC | YES | 20 |
| *Leptusa ruficollis* | LC | YES | 25 |
| *Lichenophanes numida* | EN | NO | 0 |
| *Lichenophanes varius* | EN | YES | 4 |
| *Lioderina linearis* | CR | NO | 0 |
| *Liodopria serricornis* | VU | YES | 12 |
| *Liparthrum genistae* | LC | NO | 0 |
| *Liparthrum mori* | LC | NO | 0 |
| *Lissodema cursor* | NT | YES | 12 |
| *Lissodema denticolle* | LC | YES | 1 |
| *Lissodema lituratum* | LC | YES | 1 |
| *Litargus coloratus* | NT | NO | 0 |
| *Litargus connexus* | LC | YES | 30 |
| *Lopherus rubens* | NT | YES | 4 |
| *Loricaster testaceus* | LC | NO | 0 |
| *Lucanus cervus* | LC | YES | 11 |
| *Lucanus tetraodon* | LC | YES | 1 |
| *Lycoperdina bovistae* | LC | NO | 0 |
| *Lycoperdina maritima* | VU | NO | 0 |
| *Lycoperdina succinta* | NT | NO | 0 |
| *Lycoperdina validicornis* | VU | NO | 0 |
| *Lyctus brunneus* | LC | YES | 4 |
| *Lyctus linearis* | LC | NO | 0 |
| *Lyctus pubescens* | LC | NO | 0 |
| *Lygistopterus anorachilus* | NT | NO | 0 |
| *Lygistopterus sanguineus* | LC | YES | 37 |
| *Lymantor coryli* | VU | YES | 3 |
| *Lymexylon navale* | NT | YES | 12 |
| *Lyphia tetraphylla* | EN | NO | 0 |
| *Macronychus quadrituberculatus* | EN | YES | 7 |
| *Malachius calabrus* | LC | NO | 0 |
| *Malachius italicus* | LC | NO | 0 |
| *Margarinotus merdarius* | LC | YES | 1 |
| *Margarinotus ruficornis* | VU | YES | 1 |
| *Margarinotus striola* | NT | YES | 7 |
| *Marolia variegata* | NT | YES | 1 |
| *Medon rufiventris* | NT | NO | 0 |
| *Megapenthes lugens* | VU | YES | 2 |
| *Megathous ficuzzensis* | CR | NO | 0 |
| *Megathous nigerrimus* | EN | NO | 0 |
| *Megathous valtopinensis* | EN | NO | 0 |
| *Megatoma ruficornis* | NT | NO | 0 |
| *Megatoma undata* | LC | YES | 6 |
| *Melandrya barbata* | NT | YES | 2 |
| *Melandrya caraboides* | NT | YES | 17 |
| *Melandrya dubia* | NT | YES | 2 |
| *Melanophila acuminata* | LC | YES | 4 |
| *Melanophila cuspidata* | LC | NO | 0 |
| *Melanophthalma curticollis* | LC | YES | 10 |
| *Melanophthalma distinguenda* | LC | NO | 0 |
| *Melanophthalma fuscipennis* | LC | NO | 0 |
| *Melanophthalma maura* | LC | YES | 3 |
| *Melanophthalma sericea* | LC | NO | 0 |
| *Melanophthalma suturalis* | LC | NO | 0 |
| *Melanophthalma taurica* | LC | NO | 0 |
| *Melanotus castanipes* | LC | YES | 21 |
| *Melanotus villosus* | LC | YES | 42 |
| *Melasis buprestoides* | LC | YES | 23 |
| *Meliboeus fulgidicollis* | LC | NO | 0 |
| *Meliceria sulciventris* | VU | NO | 0 |
| *Melicius cylindrus* | LC | NO | 0 |
| *Melicius gracilis* | LC | NO | 0 |
| *Menephilus cylindricus* | NT | NO | 0 |
| *Menesia bipunctata* | VU | YES | 4 |
| *Merohister ariasi* | EN | NO | 0 |
| *Mesites aquitanus* | DD | NO | 0 |
| *Mesites cunipes* | LC | NO | 0 |
| *Mesites pallidipennis* | LC | YES | 2 |
| *Mesocoelopus collaris* | LC | YES | 1 |
| *Mesocoelopus niger* | LC | YES | 13 |
| *Mesosa curculionoides* | LC | YES | 3 |
| *Mesosa nebulosa* | LC | YES | 13 |
| *Mesothes ferrugineus* | LC | NO | 0 |
| *Metholcus phoenicius* | LC | NO | 0 |
| *Metophthalmus niveicollis* | LC | NO | 0 |
| *Metophthalmus ragusae* | LC | NO | 0 |
| *Metophthalmus siculus* | DD | NO | 0 |
| *Metophthalmus solarii* | DD | NO | 0 |
| *Micrambe abietis* | DD | YES | 15 |
| *Micrambe pilosula* | DD | NO | 0 |
| *Micrambe umbripennis* | DD | NO | 0 |
| *Micrapate xyloperthoides* | LC | NO | 0 |
| *Micridium angulicolle* | DD | NO | 0 |
| *Microbregma emarginatum* | LC | YES | 6 |
| *Microrhagus emyi* | VU | NO | 0 |
| *Microrhagus hummleri* | CR | NO | 0 |
| *Microrhagus lepidus* | NT | YES | 6 |
| *Microrhagus pygmaeus* | NT | YES | 11 |
| *Migneauxia crassiuscula* | LC | NO | 0 |
| *Migneauxia inflata* | LC | NO | 0 |
| *Minthea rugicollis* | DD | NO | 0 |
| *Mizodorcatoma dommeri* | LC | NO | 0 |
| *Molorchus minor* | LC | YES | 28 |
| *Monochamus galloprovincialis* | LC | YES | 6 |
| *Monochamus saltuarius* | VU | YES | 1 |
| *Monochamus sartor* | LC | YES | 8 |
| *Monochamus sutor* | LC | YES | 9 |
| *Monotoma angusticollis* | LC | YES | 5 |
| *Monotoma bicolor* | LC | YES | 3 |
| *Monotoma brevicollis* | LC | YES | 1 |
| *Monotoma conicicollis* | DD | YES | 1 |
| *Monotoma diecki* | LC | NO | 0 |
| *Monotoma gotzi* | DD | NO | 0 |
| *Monotoma longicollis* | LC | YES | 15 |
| *Monotoma picipes* | LC | YES | 16 |
| *Monotoma punctaticollis* | LC | NO | 0 |
| *Monotoma quadricollis* | DD | YES | 2 |
| *Monotoma quadrifoveolata* | LC | NO | 0 |
| *Monotoma spinicollis* | LC | YES | 2 |
| *Monotoma testacea* | DD | YES | 2 |
| *Mordellistena humeralis* | EN | YES | 7 |
| *Mordellistena variegata* | VU | YES | 7 |
| *Mordellochroa abdominalis* | LC | YES | 33 |
| *Mordellochroa milleri* | CR | YES | 9 |
| *Morimus asper* | LC | YES | 2 |
| *Morimus funereus* | VU | NO | 0 |
| *Murmidius ovalis* | VU | NO | 0 |
| *Mycetaea subterranea* | LC | YES | 12 |
| *Mycetina cruciata* | LC | YES | 11 |
| *Mycetochara axillaris* | NT | YES | 8 |
| *Mycetochara flavipennis* | EN | NO | 0 |
| *Mycetochara flavipes* | NT | YES | 4 |
| *Mycetochara humeralis* | NT | YES | 6 |
| *Mycetochara linearis* | LC | YES | 8 |
| *Mycetochara pygmaea* | NT | NO | 0 |
| *Mycetochara quadrimaculata* | LC | YES | 2 |
| *Mycetochara straussii* | CR | NO | 0 |
| *Mycetochara thoracica* | NT | YES | 1 |
| *Mycetoma suturale* | DD | YES | 6 |
| *Mycetophagus atomarius* | LC | YES | 26 |
| *Mycetophagus decempunctatus* | NT | YES | 5 |
| *Mycetophagus fulvicollis* | NT | YES | 2 |
| *Mycetophagus multipunctatus* | NT | YES | 9 |
| *Mycetophagus piceus* | NT | YES | 18 |
| *Mycetophagus populi* | NT | YES | 7 |
| *Mycetophagus quadriguttatus* | LC | YES | 15 |
| *Mycetophagus quadripustulatus* | LC | YES | 25 |
| *Mycetophagus salicis* | NT | YES | 2 |
| *Mychothenus minutus* | NT | NO | 0 |
| *Nacerdes carniolica* | LC | YES | 5 |
| *Nacerdes gracilis* | LC | YES | 2 |
| *Nacerdes melanura* | LC | YES | 2 |
| *Nalassus alpigradus* | DD | NO | 0 |
| *Nalassus dermestoides* | LC | NO | 0 |
| *Nalassus dryadophilus* | LC | YES | 2 |
| *Nalassus genei* | LC | NO | 0 |
| *Nalassus pastai* | CR | NO | 0 |
| *Nalassus picinus* | NT | NO | 0 |
| *Nalassus planipennis* | LC | NO | 0 |
| *Nalassus plebejus* | CR | NO | 0 |
| *Nathrius brevipennis* | LC | YES | 1 |
| *Neatus noctivagus* | VU | NO | 0 |
| *Neatus picipes* | VU | YES | 2 |
| *Necydalis major* | VU | YES | 5 |
| *Necydalis ulmi* | NT | YES | 3 |
| *Nematodes filum* | VU | NO | 0 |
| *Nemozoma elongatum* | LC | YES | 13 |
| *Neohexarthrum bonnairei* | LC | NO | 0 |
| *Neomida haemorrhoidalis* | EN | YES | 5 |
| *Neopiciella sicula* | CR | NO | 0 |
| *Neumatora depressa* | CR | NO | 0 |
| *Nicobium castaneum* | LC | YES | 2 |
| *Niphona picticornis* | LC | YES | 1 |
| *Nosodendron fasciculare* | LC | YES | 7 |
| *Nosodomodes tuberculatus* |  | NO | 0 |
| *Nossidium flachi* | DD | NO | 0 |
| *Nossidium pilosellum* | DD | YES | 12 |
| *Nothorhina muricata* | NT | NO | 0 |
| *Notolaemus castaneus* | NT | NO | 0 |
| *Notolaemus unifasciatus* | NT | YES | 5 |
| *Noxius curtirostris* | LC | YES | 2 |
| *Nudobius collaris* | NT | NO | 0 |
| *Nudobius lentus* | LC | YES | 16 |
| *Oberea linearis* | LC | YES | 8 |
| *Oberea oculata* | LC | YES | 12 |
| *Obrium brunneum* | LC | YES | 12 |
| *Obrium cantharinum* | NT | NO | 0 |
| *Ochina ferruginea* | EN | NO | 0 |
| *Ochina hirsuta* | LC | NO | 0 |
| *Ochina latreillii* | NT | NO | 0 |
| *Ochina ptinoides* | LC | YES | 11 |
| *Octotemnus glabriculus* | LC | YES | 14 |
| *Octotemnus mandibularis* | VU | NO | 0 |
| *Odocnemis clypeatus* | NT | NO | 0 |
| *Odocnemis exaratus* | LC | NO | 0 |
| *Odocnemis ruffoi* | CR | NO | 0 |
| *Odontosphindus grandis* | VU | YES | 1 |
| *Oedemera femoralis* | LC | YES | 14 |
| *Ogmoderes angusticollis* | NT | NO | 0 |
| *Oligomerus brunneus* | LC | YES | 18 |
| *Oligomerus disruptus* | EN | NO | 0 |
| *Oligomerus ptilinoides* | LC | YES | 46 |
| *Omoglymmius germari* | VU | NO | 0 |
| *Onyxacalles croaticus* | NT | NO | 0 |
| *Onyxacalles henoni* | NT | NO | 0 |
| *Onyxacalles luigionii* | LC | NO | 0 |
| *Onyxacalles pyrenaeus* | LC | YES | 13 |
| *Opanthribus tessellatus* | LC | NO | 0 |
| *Opetiopalpus bicolor* | NT | NO | 0 |
| *Opetiopalpus scutellaris* | NT | NO | 0 |
| *Opilo domesticus* | LC | NO | 0 |
| *Opilo mollis* | LC | YES | 19 |
| *Opilo orocastaneus* | EN | YES | 1 |
| *Opilo pallidus* | NT | YES | 2 |
| *Opilo taeniatus* | VU | NO | 0 |
| *Oplosia cinerea* | NT | YES | 5 |
| *Orchesia blandula* | VU | NO | 0 |
| *Orchesia fasciata* | VU | YES | 5 |
| *Orchesia grandicollis* | VU | NO | 0 |
| *Orchesia maculata* | VU | NO | 0 |
| *Orchesia micans* | LC | YES | 13 |
| *Orchesia minor* | NT | YES | 18 |
| *Orchesia undulata* | LC | YES | 22 |
| *Orphilus niger* | LC | NO | 0 |
| *Orthocerus clavicornis* |  | NO | 0 |
| *Orthocerus crassicornis* |  | NO | 0 |
| *Orthoperus aequalis* | DD | NO | 0 |
| *Orthoperus atomus* | DD | YES | 13 |
| *Orthoperus corticalis* | DD | YES | 2 |
| *Orthoperus punctatus* | DD | YES | 4 |
| *Orthoperus rogeri* | DD | YES | 3 |
| *Orthotomicus erosus* | LC | YES | 1 |
| *Orthotomicus laricis* | LC | YES | 13 |
| *Orthotomicus longicollis* | VU | NO | 0 |
| *Orthotomicus mannsfeldi* | LC | NO | 0 |
| *Orthotomicus proximus* | LC | YES | 3 |
| *Orthotomicus suturalis* | LC | YES | 8 |
| *Oryctes nasicornis* | LC | YES | 10 |
| *Osmoderma cristinae* | EN | YES | 3 |
| *Osmoderma eremita* | VU | YES | 17 |
| *Osmoderma italicum* | EN | YES | 1 |
| *Osphya aeneipennis* | NT | YES | 6 |
| *Osphya bipunctata* | LC | YES | 11 |
| *Ostoma ferruginea* | NT | YES | 6 |
| *Oxylaemus cylindricus* | NT | YES | 3 |
| *Oxylaemus variolosus* | NT | NO | 0 |
| *Oxymirus cursor* | LC | YES | 9 |
| *Oxypleurus nodieri* | NT | YES | 1 |
| *Pachyta lamed* | NT | YES | 5 |
| *Pachyta quadrimaculata* | LC | YES | 21 |
| *Pachytodes cerambyciformis* | LC | YES | 23 |
| *Pachytodes erraticus* | LC | YES | 1 |
| *Palorus depressus* | LC | YES | 13 |
| *Paracorymbia fulva* | LC | YES | 5 |
| *Paracorymbia hybrida* | LC | NO | 0 |
| *Paracorymbia maculicornis* | LC | YES | 24 |
| *Paracorymbia simplonica* | VU | NO | 0 |
| *Paramecosoma melanocephalum* | LC | YES | 11 |
| *Paranopleta inhabilis* | DD | NO | 0 |
| *Parmena balteus* | LC | YES | 1 |
| *Parmena pubescens* | LC | NO | 0 |
| *Parmena subpubescens* | NT | NO | 0 |
| *Parmena unifasciata* | LC | NO | 0 |
| *Paromalus filum* | VU | NO | 0 |
| *Paromalus flavicornis* | LC | YES | 8 |
| *Paromalus parallelepipedus* | LC | YES | 3 |
| *Pediacus depressus* | NT | YES | 7 |
| *Pediacus dermestoides* | NT | YES | 1 |
| *Pediacus fuscus* | RE | YES | 2 |
| *Pedostrangalia revestita* | NT | YES | 4 |
| *Pedostrangalia verticalis* | VU | NO | 0 |
| *Pelecotoma fennica* | DD | YES | 1 |
| *Pelorinus ebeninus* | LC | NO | 0 |
| *Peltis grossa* | NT | YES | 7 |
| *Penichroa fasciata* | LC | YES | 1 |
| *Pentaphyllus chrysomeloides* | EN | NO | 0 |
| *Pentaphyllus testaceus* | EN | YES | 14 |
| *Perotis lugubris* | LC | YES | 1 |
| *Perotis unicolor* | CR | NO | 0 |
| *Phaenops cyanea* | LC | YES | 7 |
| *Phaenops formaneki* | VU | YES | 1 |
| *Phaenops knoteki* | VU | NO | 0 |
| *Phaenotherion fasciculatum* | LC | NO | 0 |
| *Phaeochrotes cinctus* | DD | NO | 0 |
| *Philothermus evanescens* | NT | YES | 9 |
| *Philothermus semistriatus* | LC | NO | 0 |
| *Phloeocharis subtilissima* | LC | YES | 23 |
| *Phloeonomus minimus* | VU | YES | 5 |
| *Phloeonomus punctipennis* | LC | YES | 19 |
| *Phloeonomus pusillus* | LC | YES | 8 |
| *Phloeophagus lignarius* | LC | YES | 1 |
| *Phloeopora concolor* | DD | YES | 1 |
| *Phloeopora corticalis* | LC | YES | 8 |
| *Phloeopora scribae* | LC | NO | 0 |
| *Phloeopora teres* | LC | YES | 9 |
| *Phloeopora testacea* | LC | YES | 4 |
| *Phloeosinus aubei* | LC | YES | 1 |
| *Phloeosinus thujae* | LC | YES | 1 |
| *Phloeostiba lapponica* | NT | YES | 3 |
| *Phloeostiba plana* | LC | YES | 16 |
| *Phloeostichus denticollis* | LC | YES | 1 |
| *Phloeotribus cristatus* | LC | NO | 0 |
| *Phloeotribus pubifrons* | LC | NO | 0 |
| *Phloeotribus rhododactylus* | LC | YES | 5 |
| *Phloeotribus scarabaeoides* | LC | YES | 1 |
| *Phloeotribus spinulosus* | LC | YES | 7 |
| *Phloiophilus edvardsii* | DD | NO | 0 |
| *Phloiotrya granicollis* | CR | NO | 0 |
| *Phloiotrya rufipes* | NT | YES | 6 |
| *Phloiotrya tenuis* | NT | YES | 4 |
| *Phyllocerus elateroides* | DD | NO | 0 |
| *Phyllocerus flavipennis* | VU | NO | 0 |
| *Phyllocerus ullmanni* | CR | NO | 0 |
| *Phyllodrepa melanocephala* | NT | YES | 5 |
| *Phyllodrepa nigra* | VU | YES | 11 |
| *Phyllodrepa salicis* | VU | NO | 0 |
| *Phyllodrepoidea crenata* | NT | YES | 5 |
| *Phymatodes testaceus* | LC | YES | 29 |
| *Phytobaenus amabilis* | NT | NO | 0 |
| *Pidonia lurida* | NT | YES | 17 |
| *Pissodes castaneus* | LC | NO | 0 |
| *Pissodes harcyniae* | DD | YES | 4 |
| *Pissodes piceae* | LC | NO | 0 |
| *Pissodes pini* | LC | YES | 14 |
| *Pissodes piniphilus* | DD | YES | 2 |
| *Pissodes scabricollis* | DD | NO | 0 |
| *Pissodes validirostris* | LC | YES | 10 |
| *Pityogenes bidentatus* | LC | YES | 16 |
| *Pityogenes bistridentatus* | LC | YES | 2 |
| *Pityogenes calcaratus* | LC | YES | 2 |
| *Pityogenes chalcographus* | LC | YES | 23 |
| *Pityogenes conjunctus* | LC | NO | 0 |
| *Pityogenes quadridens* | LC | YES | 2 |
| *Pityogenes trepanatus* | LC | YES | 2 |
| *Pityokteines curvidens* | LC | NO | 0 |
| *Pityokteines spinidens* | LC | NO | 0 |
| *Pityokteines vorontzovi* | LC | NO | 0 |
| *Pityophagus ferrugineus* | LC | YES | 13 |
| *Pityophagus laevior* | VU | YES | 1 |
| *Pityophagus quercus* | EN | YES | 2 |
| *Pityophthorus buyssoni* | LC | NO | 0 |
| *Pityophthorus carniolicus* | LC | NO | 0 |
| *Pityophthorus exsculptus* | VU | NO | 0 |
| *Pityophthorus glabratus* | LC | YES | 1 |
| *Pityophthorus henscheli* | LC | NO | 0 |
| *Pityophthorus knoteki* | LC | NO | 0 |
| *Pityophthorus lichtensteinii* | LC | YES | 6 |
| *Pityophthorus pityographus* | LC | YES | 8 |
| *Pityophthorus pubescens* | LC | YES | 4 |
| *Placonotus testaceus* | LC | YES | 12 |
| *Placusa adscita* | NT | NO | 0 |
| *Placusa atrata* | LC | YES | 4 |
| *Placusa complanata* | LC | YES | 1 |
| *Placusa depressa* | LC | YES | 4 |
| *Placusa pumilio* | LC | YES | 6 |
| *Placusa tachyporoides* | LC | YES | 15 |
| *Plagionotus arcuatus* | LC | YES | 13 |
| *Plagionotus detritus* | NT | YES | 4 |
| *Platycerus caprea* | LC | YES | 15 |
| *Platycerus caraboides* | LC | YES | 18 |
| *Platycis minutus* | LC | YES | 11 |
| *Platydema europaea* | CR | YES | 1 |
| *Platydema violacea* | NT | YES | 11 |
| *Platylister algiricus* | EN | NO | 0 |
| *Platylomalus complanatus* | LC | NO | 0 |
| *Platypus cylindrus* | LC | YES | 5 |
| *Platyrhinus resinosus* | LC | YES | 9 |
| *Platysoma angustatum* | VU | NO | 0 |
| *Platysoma compressum* | LC | YES | 2 |
| *Platysoma elongatum* | LC | NO | 0 |
| *Platysoma filiforme* | NT | NO | 0 |
| *Platysoma lineare* | VU | NO | 0 |
| *Platystomos albinus* | LC | YES | 36 |
| *Plectophloeus binaghii* | NT | NO | 0 |
| *Plectophloeus erichsoni* | VU | YES | 1 |
| *Plectophloeus fischeri* | LC | YES | 8 |
| *Plectophloeus nitidus* | LC | YES | 11 |
| *Plectophloeus nubigena* | VU | YES | 14 |
| *Plegaderus caesus* | CR | YES | 8 |
| *Plegaderus discisus* | VU | NO | 0 |
| *Plegaderus dissectus* | LC | YES | 13 |
| *Plegaderus otti* | VU | NO | 0 |
| *Plegaderus sanatus* | CR | NO | 0 |
| *Plegaderus saucius* | VU | YES | 2 |
| *Plegaderus vulneratus* | VU | NO | 0 |
| *Podeonius acuticornis* | VU | YES | 2 |
| *Poecilium alni* | LC | YES | 16 |
| *Poecilium fasciatum* | LC | NO | 0 |
| *Poecilium glabratum* | NT | NO | 0 |
| *Poecilium lividum* | LC | YES | 1 |
| *Poecilium pusillum* | NT | YES | 2 |
| *Poecilium rufipes* | NT | NO | 0 |
| *Poecilonota variolosa* | LC | YES | 3 |
| *Pogonocherus decoratus* | NT | YES | 10 |
| *Pogonocherus eugeniae* | NT | NO | 0 |
| *Pogonocherus fasciculatus* | LC | YES | 12 |
| *Pogonocherus hispidulus* | LC | YES | 7 |
| *Pogonocherus hispidus* | LC | YES | 21 |
| *Pogonocherus marcoi* | CR | NO | 0 |
| *Pogonocherus neuhausi* | NT | NO | 0 |
| *Pogonocherus ovatoides* | CR | NO | 0 |
| *Pogonocherus ovatus* | NT | YES | 1 |
| *Pogonocherus perroudi* | LC | YES | 2 |
| *Polygraphus grandiclava* | LC | YES | 6 |
| *Polygraphus poligraphus* | LC | YES | 13 |
| *Potamophilus acuminatus* | EN | YES | 12 |
| *Priartobium leonhardi* | EN | NO | 0 |
| *Priartobium serrifunis* | EN | NO | 0 |
| *Prinobius myardi* | NT | YES | 19 |
| *Priobium carpini* | NT | YES | 15 |
| *Prionocyphon serricornis* | NT | YES | 15 |
| *Prionus coriarius* | NT | YES | 12 |
| *Prionychus ater* | NT | NO | 0 |
| *Prionychus fairmairii* | NT | NO | 0 |
| *Prionychus lugens* | VU | NO | 0 |
| *Prionychus melanarius* | NT | YES | 2 |
| *Probaticus anthrax* | DD | NO | 0 |
| *Probaticus ebeninus* | DD | NO | 0 |
| *Probaticus gibbithorax* | DD | NO | 0 |
| *Probaticus sphaericollis* | DD | NO | 0 |
| *Probaticus tomentosus* | NT | NO | 0 |
| *Procraerus tibialis* | EN | YES | 13 |
| *Pronocera angusta* | EN | YES | 1 |
| *Prostomis mandibularis* | LC | YES | 2 |
| *Protaetia affinis* | LC | YES | 1 |
| *Protaetia angustata* | DD | NO | 0 |
| *Protaetia cuprea* | LC | YES | 33 |
| *Protaetia cuprea lc sx* | LC | NO | 0 |
| *Protaetia fieberi* | VU | YES | 4 |
| *Protaetia lugubris* | VU | YES | 4 |
| *Protaetia mirifica* | CR | YES | 1 |
| *Protaetia oblonga* | NT | YES | 1 |
| *Protaetia opaca* | LC | YES | 1 |
| *Protaetia sardea* | VU | NO | 0 |
| *Protaetia speciosissima* | LC | YES | 3 |
| *Protaetia squamosa* | VU | NO | 0 |
| *Pselactus caoduroi* | DD | NO | 0 |
| *Pselactus spadix* | LC | YES | 2 |
| *Pseudepierus italicus* | LC | NO | 0 |
| *Pseudeuparius centromaculatus* | LC | NO | 0 |
| *Pseudocistela ceramboides* | NT | YES | 22 |
| *Pseudodryophilus paradoxus* | EN | NO | 0 |
| *Pseudosphegesthes cinerea* | NT | NO | 0 |
| *Pseudothamnurgus mediterraneus* | LC | NO | 0 |
| *Pseudotriphyllus suturalis* | NT | NO | 0 |
| *Pseudovadonia livida* | LC | YES | 32 |
| *Psoa dubia* | LC | NO | 0 |
| *Psoa viennensis* | VU | NO | 0 |
| *Pteleobius kraatzi* | LC | YES | 1 |
| *Pteleobius vittatus* | LC | YES | 1 |
| *Ptenidium brenskei* | DD | YES | 2 |
| *Ptenidium formicetorum* | DD | YES | 6 |
| *Ptenidium fuscicorne* | DD | YES | 9 |
| *Ptenidium insulare* | DD | NO | 0 |
| *Ptenidium intermedium* | DD | YES | 7 |
| *Ptenidium laevigatum* | DD | YES | 2 |
| *Ptenidium longicorne* | DD | YES | 1 |
| *Ptenidium nitidum* | DD | YES | 11 |
| *Ptenidium ponteleccianum* | NT | NO | 0 |
| *Ptenidium punctatum* | DD | YES | 4 |
| *Ptenidium pusillum* | DD | YES | 11 |
| *Ptenidium reitteri* | DD | NO | 0 |
| *Ptenidium turgidum* | DD | YES | 1 |
| *Pteryngium crenatum* | LC | YES | 13 |
| *Pteryx ganglbaueri* | NT | NO | 0 |
| *Pteryx suturalis* | DD | YES | 23 |
| *Ptilinus fuscus* | LC | YES | 6 |
| *Ptilinus pectinicornis* | LC | YES | 20 |
| *Ptiliola brevicollis* | DD | NO | 0 |
| *Ptiliola kunzei* | DD | YES | 3 |
| *Ptiliolum caledonicum* | DD | YES | 7 |
| *Ptiliolum fuscum* | DD | NO | 0 |
| *Ptiliolum hopffgarteni* | DD | NO | 0 |
| *Ptiliolum marginatum* | DD | YES | 3 |
| *Ptiliolum oedipus* | DD | NO | 0 |
| *Ptiliolum sahlbergi* | DD | YES | 6 |
| *Ptiliolum schwarzi* | DD | NO | 0 |
| *Ptiliolum spencei* | DD | NO | 0 |
| *Ptilium affine* | DD | NO | 0 |
| *Ptilium caesum* | DD | NO | 0 |
| *Ptilium exaratum* | DD | YES | 8 |
| *Ptilium latum* | DD | NO | 0 |
| *Ptilium modestum* | DD | YES | 2 |
| *Ptilium myrmecophilum* | DD | NO | 0 |
| *Ptilium tenue* | DD | NO | 0 |
| *Ptilium vexans* | DD | NO | 0 |
| *Ptinella aptera* | DD | YES | 5 |
| *Ptinella britannica* | DD | NO | 0 |
| *Ptinella denticollis* | DD | NO | 0 |
| *Ptinella limbata* | DD | NO | 0 |
| *Ptinella mekula* | DD | NO | 0 |
| *Ptinomorphus angustatus* | VU | NO | 0 |
| *Ptinomorphus imperialis* | LC | YES | 25 |
| *Ptinomorphus regalis* | LC | YES | 6 |
| *Ptinus lichenum* | LC | YES | 5 |
| *Ptosima undecimmaculata* | LC | YES | 8 |
| *Purpuricenus apiceniger* | CR | NO | 0 |
| *Purpuricenus budensis* | VU | NO | 0 |
| *Purpuricenus globulicollis* | NT | NO | 0 |
| *Purpuricenus kaehleri* | LC | YES | 5 |
| *Pycnomerus italicus* |  | NO | 0 |
| *Pycnomerus terebrans* |  | NO | 0 |
| *Pyrochroa coccinea* | LC | YES | 36 |
| *Pyrochroa serraticornis* | LC | YES | 18 |
| *Pyropterus nigroruber* | LC | YES | 24 |
| *Pyrrhidium sanguineum* | LC | YES | 13 |
| *Pytho depressus* | DD | YES | 11 |
| *Quedius abietum* | VU | NO | 0 |
| *Quedius aetolicus* | VU | NO | 0 |
| *Quedius andreinii* | VU | NO | 0 |
| *Quedius brevicornis* | EN | YES | 14 |
| *Quedius cruentus* | LC | YES | 17 |
| *Quedius maurus* | LC | YES | 11 |
| *Quedius microps* | VU | YES | 5 |
| *Quedius plagiatus* | LC | YES | 7 |
| *Quedius scitus* | NT | YES | 18 |
| *Quedius truncicola* | VU | YES | 12 |
| *Quedius xanthopus* | LC | YES | 18 |
| *Rabdocerus foveolatus* | LC | NO | 0 |
| *Rabdocerus gabrieli* | NT | NO | 0 |
| *Raiboscelis azureus* | DD | NO | 0 |
| *Reitterelater bouyoni* | CR | YES | 1 |
| *Reitterelater dubius* | CR | YES | 1 |
| *Revelieria genei* | LC | NO | 0 |
| *Rhacopus sahlbergi* | DD | YES | 4 |
| *Rhagium bifasciatum* | LC | YES | 16 |
| *Rhagium inquisitor* | LC | YES | 26 |
| *Rhagium mordax* | LC | YES | 37 |
| *Rhagium sycophanta* | NT | YES | 12 |
| *Rhamnusium bicolor* | NT | YES | 4 |
| *Rhamnusium graecum* | CR | NO | 0 |
| *Rhaphitropis marchicus* | LC | YES | 11 |
| *Rhaphitropis oxyacanthae* | LC | YES | 1 |
| *Rhizophagus aeneus* | DD | YES | 2 |
| *Rhizophagus bipustulatus* | LC | YES | 11 |
| *Rhizophagus brancsiki* | DD | YES | 1 |
| *Rhizophagus cribratus* | DD | YES | 11 |
| *Rhizophagus depressus* | DD | YES | 16 |
| *Rhizophagus dispar* | LC | YES | 14 |
| *Rhizophagus ferrugineus* | LC | YES | 5 |
| *Rhizophagus grandis* | DD | YES | 5 |
| *Rhizophagus nitidulus* | NT | YES | 9 |
| *Rhizophagus oblongicollis* | DD | NO | 0 |
| *Rhizophagus parallelocollis* | DD | YES | 1 |
| *Rhizophagus parvulus* | DD | YES | 6 |
| *Rhizophagus perforatus* | DD | YES | 13 |
| *Rhizophagus picipes* | LC | YES | 5 |
| *Rhizophagus puncticollis* | DD | NO | 0 |
| *Rhizophagus unicolor* | LC | NO | 0 |
| *Rhopalocerus rondanii* |  | NO | 0 |
| *Rhopalodontus baudueri* | EN | NO | 0 |
| *Rhopalodontus novorossicus* | EN | NO | 0 |
| *Rhopalodontus perforatus* | LC | YES | 9 |
| *Rhopalodontus populi* | LC | NO | 0 |
| *Rhyncolus ater* | LC | YES | 10 |
| *Rhyncolus elongatus* | LC | YES | 2 |
| *Rhyncolus punctatulus* | LC | NO | 0 |
| *Rhyncolus reflexus* | LC | NO | 0 |
| *Rhyncolus sculpturatus* | LC | YES | 1 |
| *Rhyncolus strangulatus* | NT | NO | 0 |
| *Rhysodes sulcatus* | EN | YES | 1 |
| *Ropalopus clavipes* | LC | NO | 0 |
| *Ropalopus femoratus* | NT | NO | 0 |
| *Ropalopus insubricus* | VU | NO | 0 |
| *Ropalopus siculus* | EN | NO | 0 |
| *Ropalopus ungaricus* | NT | NO | 0 |
| *Ropalopus varini* | NT | NO | 0 |
| *Rosalia alpina* | NT | YES | 3 |
| *Rugilus mixtus* | CR | YES | 2 |
| *Rushia parreyssi* | NT | YES | 1 |
| *Rusticoclytus pantherinus* | EN | NO | 0 |
| *Rusticoclytus rusticus* | LC | NO | 0 |
| *Rutpela maculata* | LC | YES | 53 |
| *Salpingus aeneus* | LC | YES | 1 |
| *Salpingus planirostris* | LC | YES | 32 |
| *Salpingus ruficollis* | NT | YES | 20 |
| *Salpingus tapirus* | NT | YES | 1 |
| *Saperda carcharias* | NT | YES | 3 |
| *Saperda octopunctata* | NT | YES | 2 |
| *Saperda perforata* | EN | YES | 10 |
| *Saperda punctata* | LC | YES | 1 |
| *Saperda scalaris* | LC | YES | 27 |
| *Saperda similis* | NT | NO | 0 |
| *Saphanus piceus* | NT | NO | 0 |
| *Sardulus spelaeus* | CR | NO | 0 |
| *Scaphidema metallica* | LC | NO | 0 |
| *Scaphidium quadrimaculatum* | NT | YES | 35 |
| *Scaphisoma agaricinum* | LC | YES | 22 |
| *Scaphisoma assimile* | LC | YES | 2 |
| *Scaphisoma balcanicum* | LC | YES | 3 |
| *Scaphisoma boreale* | NT | NO | 0 |
| *Scaphisoma inopinatum* | NT | NO | 0 |
| *Scaphisoma italicum* | LC | NO | 0 |
| *Scaphisoma loebli* | NT | NO | 0 |
| *Scaphisoma obenbergeri* | NT | NO | 0 |
| *Scaphisoma palumboi* | NT | NO | 0 |
| *Scaphisoma subalpinum* | LC | YES | 6 |
| *Scaphium immaculatum* | NT | NO | 0 |
| *Schizotus pectinicornis* | LC | YES | 33 |
| *Schurmannia sicula* | CR | NO | 0 |
| *Scobicia chevrieri* | LC | YES | 1 |
| *Scobicia pustulata* | LC | YES | 1 |
| *Scolytus amygdali* | LC | NO | 0 |
| *Scolytus carpini* | VU | YES | 1 |
| *Scolytus ensifer* | VU | YES | 1 |
| *Scolytus intricatus* | LC | YES | 3 |
| *Scolytus kirschii* | LC | NO | 0 |
| *Scolytus koenigi* | LC | NO | 0 |
| *Scolytus mali* | LC | YES | 3 |
| *Scolytus multistriatus* | LC | YES | 4 |
| *Scolytus pygmaeus* | LC | YES | 3 |
| *Scolytus ratzeburgii* | DD | YES | 10 |
| *Scolytus rugulosus* | LC | YES | 35 |
| *Scolytus scolytus* | VU | NO | 0 |
| *Scolytus sulcifrons* | LC | NO | 0 |
| *Scolytus triarmatus* | EN | NO | 0 |
| *Scotoplectus capellae* | EN | NO | 0 |
| *Scraptia ophthalmica* | VU | NO | 0 |
| *Scydmaenus hellwigi* | LC | NO | 0 |
| *Scydmaenus perrisi* | VU | YES | 1 |
| *Scydmaenus rufus* | VU | YES | 7 |
| *Selatosomus cruciatus* | VU | YES | 4 |
| *Semanotus laurasii* | CR | NO | 0 |
| *Semanotus russicus* | NT | NO | 0 |
| *Semanotus undatus* | VU | YES | 3 |
| *Sepedophilus aestivus* | NT | NO | 0 |
| *Sepedophilus binotatus* | NT | NO | 0 |
| *Sepedophilus bipunctatus* | NT | YES | 13 |
| *Sepedophilus bipustulatus* | NT | NO | 0 |
| *Sepedophilus constans* | NT | NO | 0 |
| *Sepedophilus immaculatus* | LC | YES | 10 |
| *Sepedophilus lusitanicus* | NT | NO | 0 |
| *Sepedophilus marshami* | LC | YES | 20 |
| *Sepedophilus testaceus* | LC | YES | 51 |
| *Serropalpus barbatus* | NT | YES | 11 |
| *Setariola sericea* | LC | NO | 0 |
| *Siagonium humerale* | NT | NO | 0 |
| *Siagonium quadricorne* | NT | YES | 11 |
| *Silusa rubiginosa* | VU | YES | 5 |
| *Silusa rubra* | VU | NO | 0 |
| *Silvanoprus fagi* | NT | YES | 15 |
| *Silvanus bidentatus* | LC | YES | 16 |
| *Silvanus unidentatus* | LC | YES | 8 |
| *Sinodendron cylindricum* | LC | YES | 14 |
| *Sinoxylon perforans* | LC | YES | 1 |
| *Sinoxylon sexdentatum* | LC | NO | 0 |
| *Soronia grisea* | LC | YES | 12 |
| *Soronia oblonga* | LC | NO | 0 |
| *Soronia punctatissima* | EN | YES | 8 |
| *Sparedrus orsinii* | LC | NO | 0 |
| *Sparedrus testaceus* | LC | YES | 3 |
| *Sphaeriestes Aeratus* | NT | NO | 0 |
| *Sphaeriestes Bimaculatus* | VU | NO | 0 |
| *Sphaeriestes Castaneus* | NT | NO | 0 |
| *Sphaeriestes Stockmanni* | NT | NO | 0 |
| *Sphaeriestes reyi* | NT | NO | 0 |
| *Sphaerosoma apuanum* | CR | NO | 0 |
| *Sphaerosoma aspromontanum* | DD | NO | 0 |
| *Sphaerosoma fiorii* | NT | NO | 0 |
| *Sphaerosoma globosum* | LC | NO | 0 |
| *Sphaerosoma laevicolle* | DD | NO | 0 |
| *Sphaerosoma latitarse* | DD | NO | 0 |
| *Sphaerosoma maritimum* | VU | NO | 0 |
| *Sphaerosoma paganettii* | CR | NO | 0 |
| *Sphaerosoma piliferum* | LC | YES | 6 |
| *Sphaerosoma pilosum* | LC | YES | 20 |
| *Sphaerosoma punctatum* | LC | NO | 0 |
| *Sphaerosoma reitteri* | LC | NO | 0 |
| *Sphaerosoma seidlitzi* | LC | NO | 0 |
| *Sphaerosoma solarii* | LC | NO | 0 |
| *Sphaerosoma sparsum* | LC | NO | 0 |
| *Sphaerosoma vallombrosae* | LC | NO | 0 |
| *Sphindus dubius* | NT | YES | 22 |
| *Sphinginus coarctatus* | LC | NO | 0 |
| *Sphinginus constrictus* | LC | NO | 0 |
| *Sphinginus lobatus* | LC | YES | 3 |
| *Spondylis buprestoides* | NT | YES | 17 |
| *Stagetus andalusiacus* | LC | NO | 0 |
| *Stagetus byrrhoides* | LC | YES | 1 |
| *Stagetus elongatus* | LC | NO | 0 |
| *Stagetus italicus* | LC | NO | 0 |
| *Stagetus pilula* | LC | NO | 0 |
| *Stagetus sardous* | EN | NO | 0 |
| *Stenagostus rhombeus* | VU | YES | 29 |
| *Stenagostus rufus* | VU | YES | 1 |
| *Stenagostus sardiniensis* | EN | NO | 0 |
| *Stenhomalus bicolor* | NT | NO | 0 |
| *Stenocorus meridianus* | NT | YES | 15 |
| *Stenohelops carlofortinus* | DD | NO | 0 |
| *Stenomax aeneus* | LC | YES | 6 |
| *Stenomax foudrasi* | DD | YES | 1 |
| *Stenomax piceus* | NT | NO | 0 |
| *Stenopterus ater* | LC | YES | 2 |
| *Stenopterus flavicornis* | NT | NO | 0 |
| *Stenopterus rufus* | LC | YES | 13 |
| *Stenoscelis submuricata* | LC | NO | 0 |
| *Stenostola dubia* | NT | YES | 17 |
| *Stenostola ferrea* | NT | YES | 2 |
| *Stenostoma cossyrense* | NT | NO | 0 |
| *Stenostoma rostratum* | NT | NO | 0 |
| *Stenurella bifasciata* | LC | YES | 20 |
| *Stenurella melanura* | LC | YES | 72 |
| *Stenurella nigra* | LC | YES | 32 |
| *Stenurella sennii* | DD | NO | 0 |
| *Stenurella septempunctata* | VU | YES | 3 |
| *Stephanopachys linearis* | EN | YES | 2 |
| *Stephanopachys quadricollis* | VU | NO | 0 |
| *Stephanopachys substriatus* | EN | NO | 0 |
| *Stephostethus alternans* | LC | YES | 5 |
| *Stephostethus angusticollis* | LC | YES | 5 |
| *Stephostethus lardarius* | DD | YES | 17 |
| *Stephostethus pandellei* | LC | YES | 3 |
| *Stephostethus productus* | DD | NO | 0 |
| *Stephostethus rugicollis* | DD | YES | 1 |
| *Stephostethus sinuaticollis* | DD | NO | 0 |
| *Stereocorynes truncorum* | LC | YES | 1 |
| *Sternodea baudii* | LC | NO | 0 |
| *Stictoleptura cordigera* | LC | YES | 3 |
| *Stictoleptura erythroptera* | CR | NO | 0 |
| *Stictoleptura oblongomaculata* | EN | NO | 0 |
| *Stictoleptura rubra* | LC | YES | 42 |
| *Stictoleptura rufa* | NT | NO | 0 |
| *Stictoleptura scutellata* | NT | YES | 9 |
| *Strangalia attenuata* | NT | YES | 3 |
| *Stromatium unicolor* | LC | NO | 0 |
| *Styphloderes exsculptus* | VU | NO | 0 |
| *Sulcacis affinis* | LC | YES | 13 |
| *Sulcacis bicornis* | LC | NO | 0 |
| *Sulcacis bidentulus* | LC | YES | 2 |
| *Sulcacis fronticornis* | LC | YES | 9 |
| *Symbiotes armatus* | EN | NO | 0 |
| *Symbiotes gibberosus* | LC | YES | 7 |
| *Symbiotes latus* | LC | NO | 0 |
| *Synchita fallax* |  | NO | 0 |
| *Synchita humeralis* |  | NO | 0 |
| *Synchita mediolanensis* |  | NO | 0 |
| *Synchita separanda* |  | NO | 0 |
| *Synchita undata* |  | NO | 0 |
| *Synchita variegata* |  | NO | 0 |
| *Taphrorychus bicolor* | LC | YES | 8 |
| *Taphrorychus minor* | LC | NO | 0 |
| *Taphrorychus villifrons* | LC | YES | 1 |
| *Tarphius gibbulus* |  | NO | 0 |
| *Teloclerus compressicornis* | VU | NO | 0 |
| *Temnochila coerulea* | LC | YES | 2 |
| *Tenebrio obscurus* | LC | YES | 3 |
| *Tenebrio opacus* | CR | NO | 0 |
| *Tenebrio punctipennis* | DD | NO | 0 |
| *Tenebroides fuscus* | DD | NO | 0 |
| *Tenebroides maroccanus* | DD | NO | 0 |
| *Tenebroides mauritanicus* | LC | YES | 2 |
| *Teredus cylindricus* | LC | YES | 3 |
| *Teredus opacus* | VU | NO | 0 |
| *Teretrius fabricii* | VU | YES | 1 |
| *Teretrius parasita* | VU | NO | 0 |
| *Teretrius picipes* | LC | NO | 0 |
| *Tetratoma ancora* | NT | YES | 3 |
| *Tetratoma desmarestii* | EN | YES | 2 |
| *Tetratoma fungorum* | LC | YES | 13 |
| *Tetratoma tedaldi* | VU | NO | 0 |
| *Tetropium castaneum* | LC | YES | 14 |
| *Tetropium fuscum* | NT | YES | 7 |
| *Tetropium gabrieli* | NT | YES | 1 |
| *Tetrops praeustus* | LC | YES | 26 |
| *Tetrops starkii* | NT | YES | 14 |
| *Thamiaraea cinnamomea* | LC | YES | 3 |
| *Thamiaraea hospita* | LC | YES | 3 |
| *Thamnurgus characiae* | DD | NO | 0 |
| *Thamnurgus delphinii* | LC | NO | 0 |
| *Thamnurgus euphorbiae* | LC | YES | 1 |
| *Thamnurgus kaltenbachii* | LC | NO | 0 |
| *Thamnurgus sardus* | DD | NO | 0 |
| *Thanasimus femoralis* | NT | YES | 9 |
| *Thanasimus formicarius* | LC | YES | 28 |
| *Thes bergrothi* | DD | YES | 5 |
| *Thoracophorus corticinus* | EN | YES | 2 |
| *Tilloidea unifasciata* | NT | YES | 2 |
| *Tillus elongatus* | NT | YES | 23 |
| *Tillus espinosai* | EN | NO | 0 |
| *Tillus pallidipennis* | DD | NO | 0 |
| *Tomicus destruens* | LC | NO | 0 |
| *Tomicus minor* | LC | YES | 1 |
| *Tomicus piniperda* | LC | YES | 23 |
| *Tomoxia bucephala* | LC | YES | 15 |
| *Trachodes heydeni* | DD | NO | 0 |
| *Trachodes hispidus* | LC | YES | 18 |
| *Trachypteris picta* | LC | NO | 0 |
| *Tragosoma depsarium* | CR | YES | 2 |
| *Treptoplatypus oxyurus* | LC | NO | 0 |
| *Trichius fasciatus* | LC | YES | 28 |
| *Trichius gallicus* | LC | NO | 0 |
| *Trichius sexualis* | VU | YES | 10 |
| *Trichoceble floralis* | VU | NO | 0 |
| *Trichoceble memnonia* | LC | YES | 1 |
| *Trichoferus fasciculatus* | LC | YES | 1 |
| *Trichoferus griseus* | LC | NO | 0 |
| *Trichoferus holosericeus* | LC | YES | 1 |
| *Trichoferus pallidus* | EN | YES | 2 |
| *Trichonyx sulcicollis* | NT | YES | 2 |
| *Trigonorhinus areolatus* | DD | NO | 0 |
| *Trigonurus mellyi* | EN | NO | 0 |
| *Trimium aemonae* | LC | NO | 0 |
| *Trimium amplipenne* | NT | NO | 0 |
| *Trimium besucheti* | VU | NO | 0 |
| *Trimium brevicorne* | LC | YES | 18 |
| *Trimium diecki* | CR | NO | 0 |
| *Trimium minimum* | NT | NO | 0 |
| *Trimium paganettii* | VU | NO | 0 |
| *Trimium zoufali* | LC | NO | 0 |
| *Trinodes hirtus* | LC | YES | 5 |
| *Triotemnus ulianai* | DD | NO | 0 |
| *Triphyllus bicolor* | LC | YES | 24 |
| *Triplax aenea* | LC | YES | 5 |
| *Triplax andreinii* | DD | NO | 0 |
| *Triplax collaris* | DD | YES | 3 |
| *Triplax elongata* | NT | NO | 0 |
| *Triplax lacordairii* | NT | NO | 0 |
| *Triplax lepida* | NT | YES | 5 |
| *Triplax marseuli* | NT | NO | 0 |
| *Triplax melanocephala* | NT | NO | 0 |
| *Triplax nigritarsis* | EN | NO | 0 |
| *Triplax rufipes* | LC | YES | 8 |
| *Triplax russica* | LC | YES | 12 |
| *Triplax scutellaris* | EN | YES | 1 |
| *Triplax tergestana* | EN | NO | 0 |
| *Tritoma bipustulata* | LC | YES | 31 |
| *Tritoma subbasalis* | CR | NO | 0 |
| *Trixagus algiricus* | DD | NO | 0 |
| *Trixagus asiaticus* | DD | NO | 0 |
| *Trixagus atticus* | DD | YES | 5 |
| *Trixagus carinifrons* | DD | YES | 131 |
| *Trixagus dermestoides* | LC | NO | 0 |
| *Trixagus duvalii* | DD | NO | 0 |
| *Trixagus elateroides* | LC | NO | 0 |
| *Trixagus gracilis* | LC | NO | 0 |
| *Trixagus leseigneuri* | DD | YES | 14 |
| *Trixagus minutus* | DD | NO | 0 |
| *Trixagus myebohmi* | NT | NO | 0 |
| *Trixagus obtusus* | LC | YES | 8 |
| *Trixagus rougeti* | DD | NO | 0 |
| *Troglops albicans* | LC | YES | 6 |
| *Troglops cephalotes* | CR | NO | 0 |
| *Troglops italicus* | LC | NO | 0 |
| *Troglops silo* | LC | NO | 0 |
| *Trogoxylon impressum* | LC | NO | 0 |
| *Tropideres albirostris* | LC | YES | 3 |
| *Tropideres dorsalis* | DD | YES | 4 |
| *Trox perrisi* | DD | NO | 0 |
| *Trypodendron domesticum* | LC | YES | 23 |
| *Trypodendron lineatum* | LC | YES | 34 |
| *Trypodendron signatum* | LC | YES | 18 |
| *Trypophloeus alni* | DD | YES | 3 |
| *Trypophloeus binodulus* | LC | YES | 2 |
| *Typhaea angusta* | DD | NO | 0 |
| *Typhaea stercorea* | LC | YES | 11 |
| *Typhaeola maculata* | LC | NO | 0 |
| *Tyrus mucronatus* | NT | YES | 13 |
| *Uleiota planatus* | LC | YES | 1 |
| *Uloma culinaris* | LC | YES | 22 |
| *Uloma rufa* | EN | YES | 16 |
| *Ulorhinus bilineatus* | LC | NO | 0 |
| *Valgus hemipterus* | LC | YES | 18 |
| *Vincenzellus ruficollis* | LC | YES | 27 |
| *Wanachia triguttata* | DD | NO | 0 |
| *Xenoscelis costipennis* | LC | NO | 0 |
| *Xestobium rufovillosum* | LC | YES | 17 |
| *Xestobium subincanum* | EN | NO | 0 |
| *Xyleborinus saxesenii* | LC | YES | 24 |
| *Xyleborus cryptographus* | DD | YES | 3 |
| *Xyleborus dryographus* | LC | YES | 5 |
| *Xyleborus eurygraphus* | LC | NO | 0 |
| *Xyleborus monographus* | LC | YES | 20 |
| *Xyleborus pfeili* | VU | NO | 0 |
| *Xylechinus pilosus* | LC | YES | 3 |
| *Xyletinus ater* | LC | YES | 10 |
| *Xyletinus balcanicus* | VU | NO | 0 |
| *Xyletinus laticollis* | VU | YES | 1 |
| *Xyletinus longitarsis* | LC | YES | 2 |
| *Xyletinus pectinatus* | VU | YES | 2 |
| *Xyletinus pectiniferus* | NT | NO | 0 |
| *Xyletinus ruficollis* | VU | NO | 0 |
| *Xylita laevigata* | NT | YES | 5 |
| *Xylocleptes bispinus* | LC | YES | 19 |
| *Xylocleptes biuncus* | LC | NO | 0 |
| *Xylolaemus fasciculosus* |  | NO | 0 |
| *Xylomedes coronata* | DD | NO | 0 |
| *Xylopertha praeusta* | LC | NO | 0 |
| *Xylopertha retusa* | VU | NO | 0 |
| *Xyloperthella picea* | LC | NO | 0 |
| *Xylophilus corticalis* | NT | NO | 0 |
| *Xylophilus testaceus* | EN | NO | 0 |
| *Xylosteus spinolae* | EN | NO | 0 |
| *Xylostiba bosnica* | VU | YES | 17 |
| *Xylostiba monilicornis* | NT | YES | 3 |
| *Xylotrechus antilope* | LC | YES | 7 |
| *Xylotrechus arvicola* | LC | YES | 2 |
| *Zeteotomus brevicornis* | EN | NO | 0 |
| *Zilora obscura* | VU | YES | 5 |
