# Supplementary material for: Filling knowledge gaps in insect conservation by leveraging genetic data from public archives
Source: Database (Oxford). 2024 Jan 29;2024:baae002. doi: 10.1093/database/baae002 (PMC10878047; doi:10.1093/database/baae002)
Supplement: baae002_Supp [file baae002_supp.zip › suppl_data/Table_S5_Column_Description.docx]

**Table S5**. *Table providing a description of each field in the database records*

| **Column name** | **Description** |
| --- | --- |
| barcode_id | unique identifier for each record |
| phylum_name | phylum name |
| class_name | class name |
| order_name | order name |
| family_name | family name |
| genus_name | genus name |
| species name | species_name |
| country | country |
| Latitude | latitude in Decimal degrees (DD) |
| Longitude | longitude in Decimal degrees (DD) |
| elev | elevation of specimen finding |
| depth | depth of specimen finding |
| sourceDatabase | database from which the sample was retrieved |
| bin_uri | BOLD barcode index numbers (BINs) are operational units of species based on patterns of COI |
| processid | unique identifier of a BOLD sequence record |
| genbank_accession | unique identifier of a Genbank sequence record |
| gene | gene extracted |
| nucleotides | barcoding sequence |
| SeqLength | length of barcoding sequence |
| statusIUCN | IUCN conservation status |
